# GRWD1-WDR5-MLL2 Epigenetic Complex Mediates H3K4me3 Mark and Is Essential for Kaposi’s Sarcoma-Associated Herpesvirus-Induced Cellular Transformation

**DOI:** 10.1128/mbio.03431-21

**Published:** 2021-12-21

**Authors:** Shan Wei, Songjian Lu, Lifan Liang, Xian Wang, Wan Li, Tingting Li, Luping Chen, Enguo Ju, Xinquan Zhang, Zhao Lai, Yufei Huang, Xinghua Lu, Shou-Jiang Gao

**Affiliations:** a Cancer Virology Program, UPMC Hillman Cancer Center, and Department of Microbiology and Molecular Genetics, University of Pittsburgh, Pittsburgh, Pennsylvania, USA; b Department of Biomedical Informatics, University of Pittsburgh, Pittsburgh, Pennsylvania, USA; c Greehey Children’s Cancer Research Institute and Department of Molecular Medicine, University of Texas Health Science Center at San Antonio, San Antonio, Texas, USA; d Department of Medicine, University of Pittsburgh, Pittsburgh, Pennsylvania, USA; University of Calgary

**Keywords:** glutamate-rich WD repeat containing 1, GRWD1, WD repeat domain 5, WDR5, myeloid/lymphoid or mixed-lineage leukemia 2, MLL2, epigenetic regulators, H3 lysine 4 trimethylation, H3K4me3, Kaposi’s sarcoma-associated herpesvirus, KSHV, Kaposi’s sarcoma, KS

## Abstract

Infection by Kaposi’s sarcoma-associated herpesvirus (KSHV) is causally associated with numerous cancers. The mechanism of KSHV-induced oncogenesis remains unclear. By performing a CRISPR-Cas9 screening in a model of KSHV-induced cellular transformation of primary cells, we identified epigenetic regulators that were essential for KSHV-induced cellular transformation. Examination of TCGA data sets of the top 9 genes, including glutamate-rich WD repeat containing 1 (GRWD1), a WD40 family protein upregulated by KSHV, that had positive effects on cell proliferation and survival of KSHV-transformed cells (KMM) but not the matched primary cells (MM), uncovered the predictive values of their expressions for patient survival in numerous types of cancer. We revealed global epigenetic remodeling including H3K4me3 epigenetic active mark in KMM cells compared to MM cells. Knockdown of GRWD1 inhibited cell proliferation, cellular transformation, and tumor formation and caused downregulation of global H3K4me3 mark in KMM cells. GRWD1 interacted with WD repeat domain 5 (WDR5), the core protein of H3K4 methyltransferase complex, and several H3K4me3 methyltransferases, including myeloid leukemia 2 (MLL2). Knockdown of WDR5 and MLL2 phenocopied GRWD1 knockdown, caused global reduction of H3K4me3 mark, and altered the expression of similar sets of genes. Transcriptome sequencing (RNA-seq) and chromatin immunoprecipitation sequencing (ChIP-seq) analyses further identified common and distinct cellular genes and pathways that were regulated by GRWD1, WDR5, and MLL2. These results indicate that KSHV hijacks the GRWD1-WDR5-MLL2 epigenetic complex to regulate H3K4me3 methylation of specific genes, which is essential for KSHV-induced cellular transformation. Our work has identified an epigenetic complex as a novel therapeutic target for KSHV-induced cancers.

## INTRODUCTION

Epigenetics is the study of heritable changes that regulate gene expression without affecting the sequence of DNA (1). The most common epigenetic processes that regulate the dynamic structure of chromatin are DNA methylation and posttranslational modifications of histones (1, 2). Histone lysine methylation has been recognized as a key mark among histone modifications (2, 3). The methylation of lysine side chains of the histone tails leads to activation or repression of transcription, depending on the location and methylation status (mono-, di-, or tri-) (2, 3). Over 700 proteins have been reported to participate in epigenetic remodeling (4); however, the functions of many of these proteins remain unclear, and additional novel epigenetic factors remain to be discovered. Epigenetic dysregulation is involved in many human diseases, including cancers, cardiovascular diseases, and Alzheimer’s disease, etc. (2, 5). Understanding the mechanism of epigenetic regulation and the functions of epigenetic factors is essential for identifying novel therapies.

Kaposi’s sarcoma-associated herpesvirus (KSHV) is a human tumor virus causally linked to Kaposi’s sarcoma (KS) and primary effusion lymphomas (PEL) (6). As KSHV is a member of the herpesvirus family, its life cycle has both latent and lytic replication phases. The majority of KS tumor cells are latently infected by KSHV expressing only a few viral latent products, including LANA, vCyclin, vFLIP, and 12 viral precursor microRNAs (miRNAs) (7, 8). These proteins and miRNAs repress KSHV lytic replication, mediate the replication of viral episome, and promote the survival of cells (7, 8). Hence, KSHV latent products are required to maintain KSHV latency and are directly responsible for the development of KSHV-induced tumors (9). Despite extensive studies, the mechanism of KSHV-induced oncogenesis remains unclear in part due to the lack of an experimental model of KSHV-induced cellular transformation of primary cells. We have succeeded in transforming primary rat embryonic metanephric mesenchymal precursor (MM) cells with KSHV (10). Compared to untransformed primary cells, KSHV-transformed MM (KMM) cells are immortalized, have a higher proliferation rate, show no contact inhibition, and gain the ability to form tumors *in vivo* (10). This unique system has been used to identify viral and cellular genes mediating KSHV-induced tumorigenesis (11–20). For this purpose, a genome-wide CRISPR-Cas9 screening was performed with matched MM and KMM cells, which led to the identification of a group of genes that were essential for the survival of KMM but not MM cells (21).

Numerous KSHV latent products regulate epigenetic remodeling (22, 23). However, whether epigenetic remodeling is critical for KSHV-induced cellular transformation and the underlying mechanisms remain unknown. To investigate the epigenetic factors that are essential for KSHV-transformed cells, we have combined the results of the CRISPR-Cas9 screening (21) with those of the EpiFactors database (4) and the TCGA survival data (24), and identified a set of epigenetic regulators that are potentially important for the survival of KSHV-transformed cells. Among them, glutamate-rich WD repeat containing 1 (GRWD1) is a highly conserved protein belonging to the WD40 family functionally involved in ribosome biogenesis (25, 26). GRWD1 has two domains, an N-terminal glutamate-rich acidic domain and a C-terminal WD40 repeat domain (26). GRWD1 is a histone-binding protein interacting with both H2A/H2B and H3-H4 through its N-terminal domain. It regulates chromatin dynamics and the loading of MCM2 helicase (26) and is predicted to regulate H3K4 methylation (27). Furthermore, overexpression of GRWD1 represses the function of tumor suppressor p53 and, together with constitutively active oncogene KRAS and human papillomavirus (HPV) oncogene E7, transforms a telomerase-immortalized cell line, HFF2/T (28), suggesting that GRWD1 is a potential oncogene. Indeed, our results show that GRWD1 dysregulation is associated with poor prognosis of patients in several types of cancer (24), including brain lower-grade glioma (LGG), mesothelioma (MESO), and skin cutaneous melanoma (SKCM). Taken together, GRWD1 is a strong candidate epigenetic regulator that might be involved in KSHV-induced tumorigenesis.

In this study, we have hypothesized that GRWD1 might function as an essential epigenetic factor mediating KSHV-induced cellular transformation by regulating the expression of specific cellular genes. We have found that GRWD1 recruits an H3K4 methyltransferase complex to the promoters of growth-promoting genes to increase their expression and enhance KSHV-induced cell proliferation and cellular transformation. We have demonstrated that GRWD1 forms a complex with the core protein of H3K4 methyltransferase complex, WD repeat domain 5 (WDR5), and the H3K4 methyltransferase myeloid/lymphoid or mixed-lineage leukemia 2 (MLL2), also known as MLL4 or histone-lysine N-methyltransferase 2B (KMT2B). By chromatin immunoprecipitation sequencing (ChIP-seq) and transcriptome sequencing (RNA-seq) study, we have determined that GRWD1 functions as an essential regulator of histone H3 lysine 4 (H3K4) trimethylation (H3K4me3) through the GRWD1-WDR5-MLL2 complex.

## RESULTS

### Alterations of global epigenetic modifications in KSHV-transformed cells.

**FIG 1:**
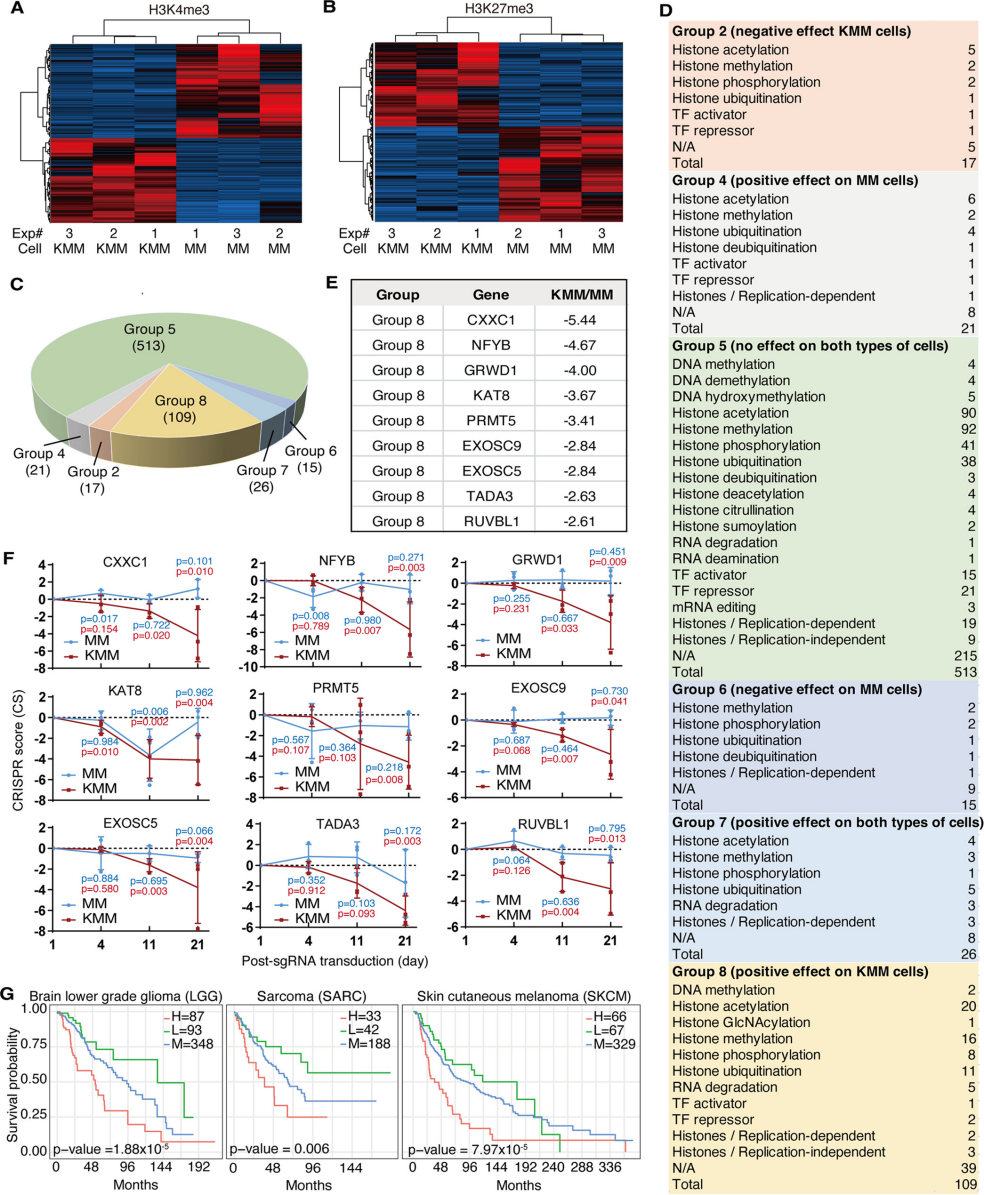
Epigenetic factors that are essential for KSHV-transformed cells identified by CRISPR-Cas9 screening. (A and B) Heatmaps of H3K4me3 (A) and H3K27me3 (B) peaks of MM and KMM cells. (C) Distribution of epigenetic factors in different groups identified by CRISPR-Cas9 screening of MM and KMM cells (21). (D) Functional classification of epigenetic factors by CRISPR-Cas9 screening of MM and KMM cells (21). TF, transcription factor; N/A, not available. (E) Top 9 epigenetic factors with the largest differences of CRISPR score between MM and KMM cells in group 8 identified by CRISPR-Cas9 screening. CRISPR score is defined as the average [log_2_(final sgRNA abundance/initial sgRNA abundance)] of 3 single guide RNAs (sgRNAs) (21). (F) CRISPR scores at days 4, 11, and 21 of the top 9 epigenetic factors with the largest differences of CRISPR score between MM and KMM cells. *P* values are from comparisons between day 4, 11, or 21 and day 1 for MM (blue) and KMM (red) cells, respectively. (G) Survival analysis of GRWD1 expression in brain lower-grade glioma (LGG), sarcoma (SARC), and skin cutaneous melanoma (SKCM). H, L, and M, high, low, and medium, respectively.

To determine alterations of the epigenetic landscape of KSHV-transformed cells, we performed ChIP-seq to identify the common active mark H3K4me3 and H3 lysine 27 trimethylation (H3K27me3) repressive mark in MM and KMM cells. Compared to MM cells, there were significant changes of H3K4me3 and H3K27me3 marks in KMM cells (Fig. 1A and B), which were consistent with alterations of gene expression in these cells (11). We have previously performed a CRISPR-Cas9 screening with MM and KMM cells and identified genes that promote or suppress cell proliferation and survival (21). By combining the EpiFactors database (4) and the epigenetic factors newly described between 2015 and 2021 (26, 29–33), we identified 701 epigenetics-related genes that had differential effects on cell proliferation and survival between MM and KMM cells following their knockout (Fig. 1C). Of the 6 groups of genes identified, group 2 (17 genes) had negative and group 8 (109 genes) had positive effects on cell proliferation and survival of KMM but not MM cells, respectively (21) (Fig. 1D). The top 9 genes in group 8 with the most differences in CRISPR scores between KMM and MM cells at day 21 following knockout were CXXC1, NFYB, GRWD1, KAT8, PRMT5, EXOSC9, EXOSC5, TADA3, and RUVBL1 (Fig. 1E and F). These genes are likely essential for the proliferation of KMM but not MM cells. To determine the likely involvement of these genes in other types of cancer, we examined their prognostic values using the TCGA database (Fig. 1G; see also Fig. S1 and Table S1 in the supplemental material). Patients with a higher expression level of CXXC1 had a worse prognosis for liver hepatocellular carcinoma (LIHC) but a better prognosis for bladder urothelial carcinoma (BLCA) and uterine corpus endometrial carcinoma (UCEC). Patients with a higher expression level of NFYB had a worse prognosis for kidney renal clear cell carcinoma (KIRC) and kidney renal papillary cell carcinoma (KIRP) but a better prognosis for BLCA, cervical squamous cell carcinoma, and endocervical adenocarcinoma (CESC), brain lower-grade glioma (LGG), LIHC, sarcoma (SARC), and skin cutaneous melanoma (SKCM). Patients with a higher expression level of GRWD1 had a worse prognosis for LGG, lung adenocarcinoma (LUAD), breast cancer (BC), SARC, and SKCM. Patients with a higher expression level of KAT8 had a better prognosis for BC. Patients with a higher expression level of PRMT5 had a worse prognosis for BLCA, head and neck squamous cell carcinoma (HNSC), LIHC, and SARC but a better prognosis for glioblastoma multiforme (GBM) and KIRC. Patients with a higher expression level of EXOSC9 had a worse prognosis for BC. Patients with a higher expression level of EXOSC5 had a worse prognosis for KIRC, KIRP, BC, and prostate adenocarcinoma (PRAD). Patients with a higher expression level of TADA3 had a worse prognosis for KIRC but a better prognosis for BLCA. Patients with a higher expression level of RUVBL1 had a worse prognosis for LGG, LIHC, BC, and SARC. Since a higher expression level of GRWD1 is associated with a worse prognosis of 5 types of cancer, especially SARC, which is similar to KS and the KMM tumor, and its epigenetic role in cancer is largely unknown, we chose to further investigate GRWD1’s role in KSHV-induced cellular transformation.

10.1128/mbio.03431-21.1 FIG S1 The survival plots for the top 9 epigenetic factors including CXXC1, NFYB, GRWD1, KAT8, PRMT5, EXOSC9, EXOSC5, TADA3, and RUVBL1 with the largest differences of CRISPR scores between MM and KMM cells identified in CRISPR-Cas9 screening in different types of cancer from the TCGA data set including bladder urothelial carcinoma (BLCA), kidney renal clear cell carcinoma (KIRC), kidney renal papillary cell carcinoma (KIRP), liver hepatocellular carcinoma (LIHC), breast cancer (MetaBric), sarcoma (SARC), uterine corpus endometrial carcinoma (UCEC), glioblastoma multiforme (GBM), lung squamous cell carcinoma (LUSC), prostate adenocarcinoma (PRAD), brain lower-grade glioma (LGG), lung adenocarcinoma (LUAD), skin cutaneous melanoma (SKCM), cervical squamous cell carcinoma and endocervical adenocarcinoma (CESC), head-neck squamous cell carcinoma (HNSC), and ovarian serous cystadenocarcinoma (OV). Patients were grouped into high (H), medium (M), and low (L) based on the expression of the target gene. Download FIG S1, PDF file, 0.3 MB. Copyright © 2021 Wei et al. 2021 Wei et al. https://creativecommons.org/licenses/by/4.0/ This content is distributed under the terms of the Creative Commons Attribution 4.0 International license.

10.1128/mbio.03431-21.6 TABLE S1 Summary of survival analysis of top 9 epigenetic genes in group 8 identified by CRISPR-Cas9 screening of MM and KMM cells (21). Download Table S1, PDF file, 0.04 MB. Copyright © 2021 Wei et al. 2021 Wei et al. https://creativecommons.org/licenses/by/4.0/ This content is distributed under the terms of the Creative Commons Attribution 4.0 International license.

### GRWD1 is essential for cell proliferation and cellular transformation of KSHV-transformed cells.

**FIG 2:**
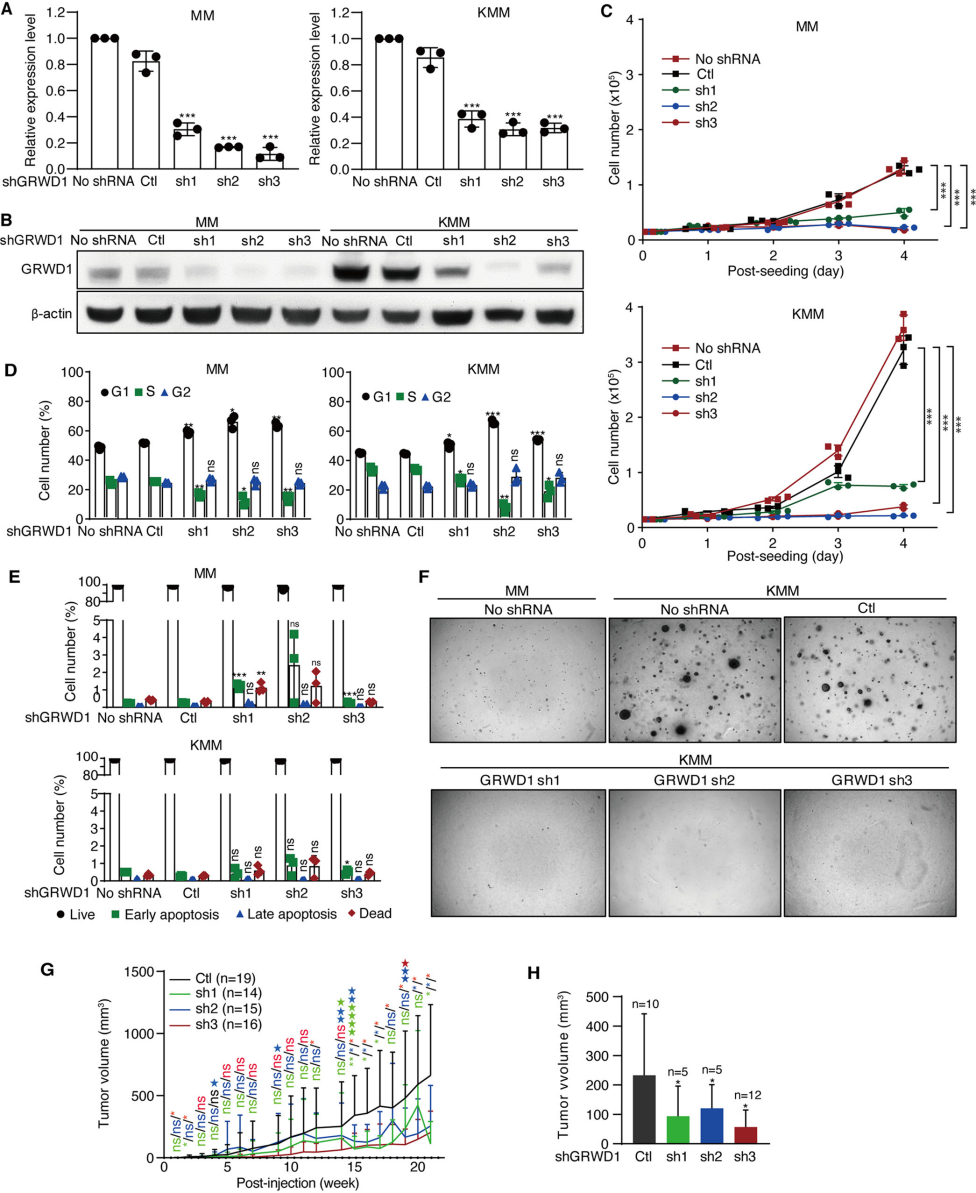
GRWD1 is essential for KSHV-induced cell proliferation and cellular transformation. (A and B) Knockdown efficiencies of GRWD1 shRNAs examined by RT-qPCR (A) and Western blotting (B). (C to E) The effects of GRWD1 knockdown on cell proliferation (C), cell cycle progression (D), and apoptosis (E) of MM and KMM cells. (F) GRWD1 knockdown reduced the efficiency of colony formation on soft agar of KMM cells. (G) GRWD1 knockdown inhibited the progression of KMM tumors in nude mice. Mice from different groups terminated before the final day of the experiment were labeled with the matched color stars. (H) At the endpoint, week 21, GRWD1 knockdown reduced the volume of KMM tumors in nude mice. *P* values are from comparisons between each of the shRNA-treated groups (sh1, sh2, and sh3) and the scrambled control (Ctl).

To confirm the essential role of GRWD1 in the proliferation of KSHV-transformed cells, we performed lentivirus-mediated short hairpin RNA (shRNA) knockdown of GRWD1. At day 3 posttransduction, GRWD1 RNA and protein levels were reduced by >70% and >60% in MM and KMM cells, respectively (Fig. 2A and B). Interestingly, the protein level of GRWD1 was higher in untransduced KMM than MM cells (Fig. 2B). As expected, KMM cells proliferated at a much higher rate than MM cells did (10). Compared to untransduced cells or cells transduced with scrambled shRNA, both MM and KMM cells transduced with the shRNAs had significantly reduced proliferation rates with a more profound effect observed in KMM than MM cells (Fig. 2C). GRWD1 knockdown induced cell cycle arrest by increasing G_1_ phase and reducing S phase cell numbers of both MM and KMM cells, respectively (Fig. 2D), but had minimal effect on the numbers of apoptotic cells for both types of cells (Fig. 2E). Importantly, GRWD1 knockdown completely abolished colony formation of KMM cells in soft agar (Fig. 2F). As expected, no colony was observed with MM cells with or without GRWD1 knockdown.

We further examined the effect of GRWD1 knockdown on tumor formation of KMM cells in nude mice. GRWD1 knockdown significantly reduced the numbers of tumors as well as the progression of tumors (Fig. 2G and H; see also Fig. S2). At the endpoint of week 21, the average volume of tumors induced by KMM cells transduced with scrambled shRNA was 233.03 mm^3^ while the values for those transduced with the three shRNAs were 93.28 mm^3^, 120.27 mm^3^, and 56.76 mm^3^, respectively, excluding the excessive large tumors terminated in advance (Fig. 2H).

10.1128/mbio.03431-21.2 FIG S2 The tumor volumes of individual tumors from each group showing that GRWD1 knockdown inhibited the progression of KMM tumors. Thicker lines are the averages of the tumor volumes. Mice with tumors exceeding the 1.5-cm^3^ volume limit were terminated before the final day of the experiment (Fig. 2 provides details). Download FIG S2, PDF file, 0.7 MB. Copyright © 2021 Wei et al. 2021 Wei et al. https://creativecommons.org/licenses/by/4.0/ This content is distributed under the terms of the Creative Commons Attribution 4.0 International license.

Taken together, these results indicate that GRWD1 is essential for cell cycle progression, cell proliferation, and cellular transformation of KSHV-transformed cells and that GRWD1 is also required for the proliferation of primary cells.

### GRWD1 maintains H3K4me3 mark at specific loci of KSHV-transformed cells.

**FIG 3:**
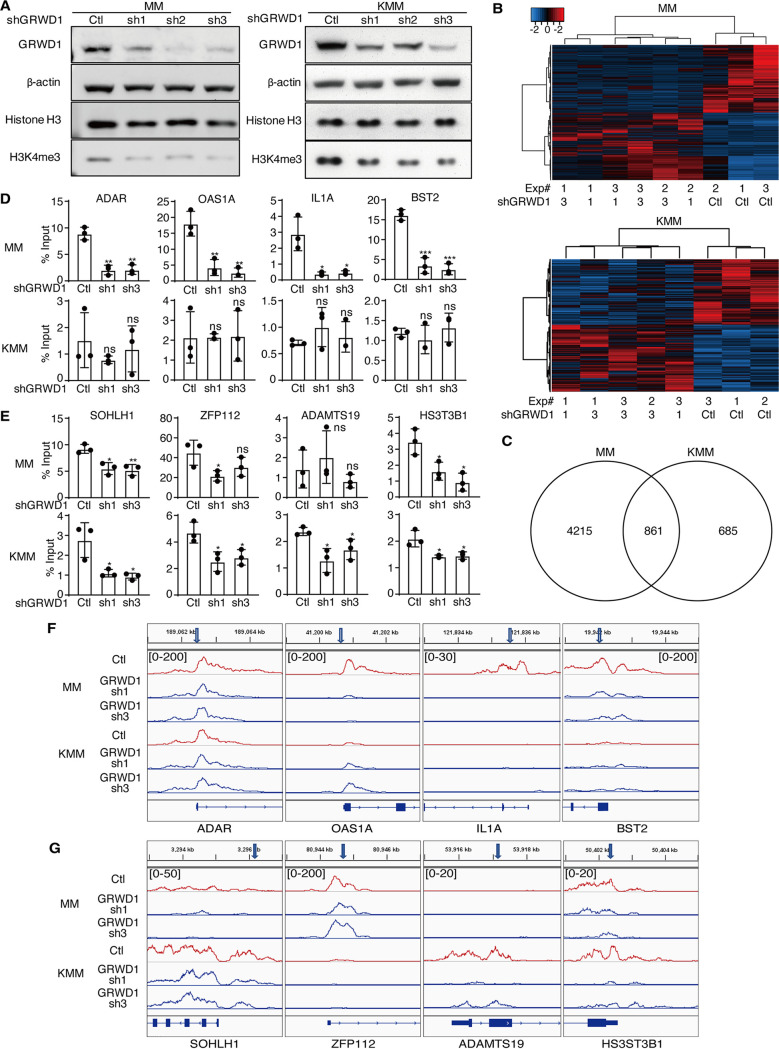
GRWD1 knockdown reduces levels of H3K4me3 marks. (A) Western blot assays showed that the signals of H3K4me3 marks were decreased after GRWD1 knockdown in MM and KMM cells. (B) Heatmaps showing alterations of H3K4me3 marks after GRWD1 knockdown in MM and KMM cells. (C) Differential alterations of H3K4me3 marks between MM and KMM cells after GRWD1 knockdown. (D and E) ChIP-qPCR validation of reduction of H3K4me3 peaks at specific gene loci in MM (D) and KMM (E) cells. The same H3K4me3 peaks were also examined in other cells for comparison. *P* values are from comparisons between each of the shRNA-treated groups (sh1 and sh3) and the scrambled control (Ctl). ns, not significant. (F and G) Tracks of H3K4me3 peaks in the promoters of the four candidate GRWD1 targets in MM (F) and KMM (G) cells. Primer positions were labeled with arrows.

GRWD1 regulates chromatin architecture (26, 34) and is potentially involved in histone methylation (27). Thus, we examined GRWD1’s role in epigenetic remodeling in KSHV-transformed cells. Transient GRWD1 knockdown was sufficient to reduce the overall H3K4me3 level but not those of H3K27me3 H3K4me2 and H3K4me in both MM and KMM cells (Fig. 3A; see also Fig. S3A). To identify the specific H3K4me3 loci that were regulated by GRWD1, we performed ChIP-seq in cells with stable GRWD1 knockdown. We achieved high efficiency of GRWD1 knockdown with shRNA2. Because of GRWD1’s essential role, we failed to generate stable knockdown KMM cells with this shRNA (Fig. 2B and C). However, we were able to generate GRWD1 stable knockdown cells with shRNA1 and shRNA3. GRWD1 knockdown differentially altered the H3K4me3 peaks in MM and KMM cells (Fig. 3B).

10.1128/mbio.03431-21.3 FIG S3 Changes of different histone marks and expression of representative genes following GRWD1 knockdown. (A) Western blots showing that H3K27me3, H3K4me2, and H3K4me epigenetic marks and total histone H3 remain unchanged after GRWD1 knockdown in MM and KMM cells. (B) The tracks of H3K4me3 and H3K27me3 marks at the promoter regions of the top target genes from ChIP-seq analysis in MM and KMM cells. (C) Top four genes with reduction of H3K4me3 peaks at specific gene loci in MM cells after GRWD1 knockdown. The expression levels were normalized to the scrambled control-treated MM cells. (D) Top four genes with reduction of H3K4me3 peaks at specific gene loci in KMM cells after GRWD1 knockdown. The expression levels were normalized to the scrambled control-treated KMM cells. *P* values are from comparisons between each of the shRNA-treated groups (sh1, sh2, and sh3) and the scrambled control (Ctl). Download FIG S3, PDF file, 0.9 MB. Copyright © 2021 Wei et al. 2021 Wei et al. https://creativecommons.org/licenses/by/4.0/ This content is distributed under the terms of the Creative Commons Attribution 4.0 International license.

To identify the GRWD1-regulated H3K4me3 peaks, we defined differential peaks between GRWD1 and scrambled shRNA knockdown cells with a *P* value of <0.05 and a fold change of >0.5. We identified 8,501 and 1,765 differential H3K4me3 peaks for MM and KMM cells, respectively (Table S2A and B). The altered H3K4me3 peaks were annotated to 5,076 and 1,546 genes in MM and KMM cells, respectively, of which 861 genes were shared in the two types of cells (Fig. 3C), indicating both common and distinct epigenetic regulations between MM and KMM cells. Among the top distinct representative genes regulated by GRWD1 in MM cells, we validated ADAR, OAS1A, IL1A, and BST2 by ChIP-qPCR, which had minimal changes in KMM cells following GRWD1 knockdown (Fig. 3D). Indeed, there were significant reductions of H3K4me3 peaks for all four genes in MM cells in ChIP-Seq analysis (Fig. 3F; see also Fig. S3B). In contrast, there was no change of H3K4me3 peak for the ADAR gene while there was only a minor or no peak for the OAS1A, IL1A, or BST2 gene in KMM cells (Fig. 3F). In agreement with these results, the expression levels of OAS1A, IL1A, and BST2 genes were significantly reduced in MM cells following GRWD1 knockdown (Fig. S3C). However, only one shRNA marginally reduced the expression of the ADAR gene in MM cells while the second one had no effect (Fig. S3C), suggesting that the expression of the ADAR gene might also be regulated by other epigenetic factors and marks. No significant expression level change was found for these four genes in KMM cells following GRWD1 knockdown (Fig. S3C). Interestingly, the expression levels of OAS1A, IL1A, and BST2 genes were 2.2-, 10-, and 5-fold lower in KMM cells than MM cells, respectively (Fig. S3C), suggesting that KSHV might downregulate the expression of these genes. Similarly, we validated SOHLH1, ZFP112, ADAMTS19, and HS3ST3B1 as the top representative genes regulated by GRWD1 in KMM cells by ChIP-qPCR (Fig. 3E). ZFP112 and ADAMTS19 genes had no significant change of H3K4me3 while reductions for SOHLH1 and HS3ST3B1 genes were observed in MM cells following GRWD1 knockdown (Fig. 3E). Consistent with these results, significant reductions of H3K4me3 peaks for ZFP112 and ADAMTS19 were observed in KMM cells in ChIP-Seq analysis, which were not observed in MM cells, while SOHLH1 and HS3ST3B1 peaks were reduced significantly in both cell types (Fig. 3G). In addition, SOHLH1 and HS3ST3B1 had both H3K4me3 and H3K27me3 marks at the promoters in MM cells, indicating the bivalent nature of these promoters (Fig. S3B). In agreement with these results, the expression levels of these four genes were significantly reduced in KMM cells following GRWD1 knockdown (Fig. S3D). However, no significant change was observed in MM cells following GRWD1 knockdown (Fig. S3D), suggesting they were regulated by another mechanism. In addition, the expression levels of SOHLH1 and HS3ST3B1 were 9.4- and 3.2-fold higher, respectively, in KMM than MM cells, while ZFP112 gene expression was 6-fold lower in KMM than MM cells (Fig. S3D), suggesting KSHV regulation of these genes. Together, these results indicate that GRWD1 is required for sustaining specific H3K4me3 peaks in both MM and KMM cells, and the epigenetic landscapes were distinct between the primary and transformed cells.

10.1128/mbio.03431-21.7 TABLE S2 Altered H3K4me3 peaks and associated genes after GRWD1 knockdown in MM (A) and KMM (B) cells. Download Table S2, PDF file, 1.5 MB. Copyright © 2021 Wei et al. 2021 Wei et al. https://creativecommons.org/licenses/by/4.0/ This content is distributed under the terms of the Creative Commons Attribution 4.0 International license.

### GRWD1 interacts with WDR5, the core protein of H3K4 methyltransferase complex.

**FIG 4:**
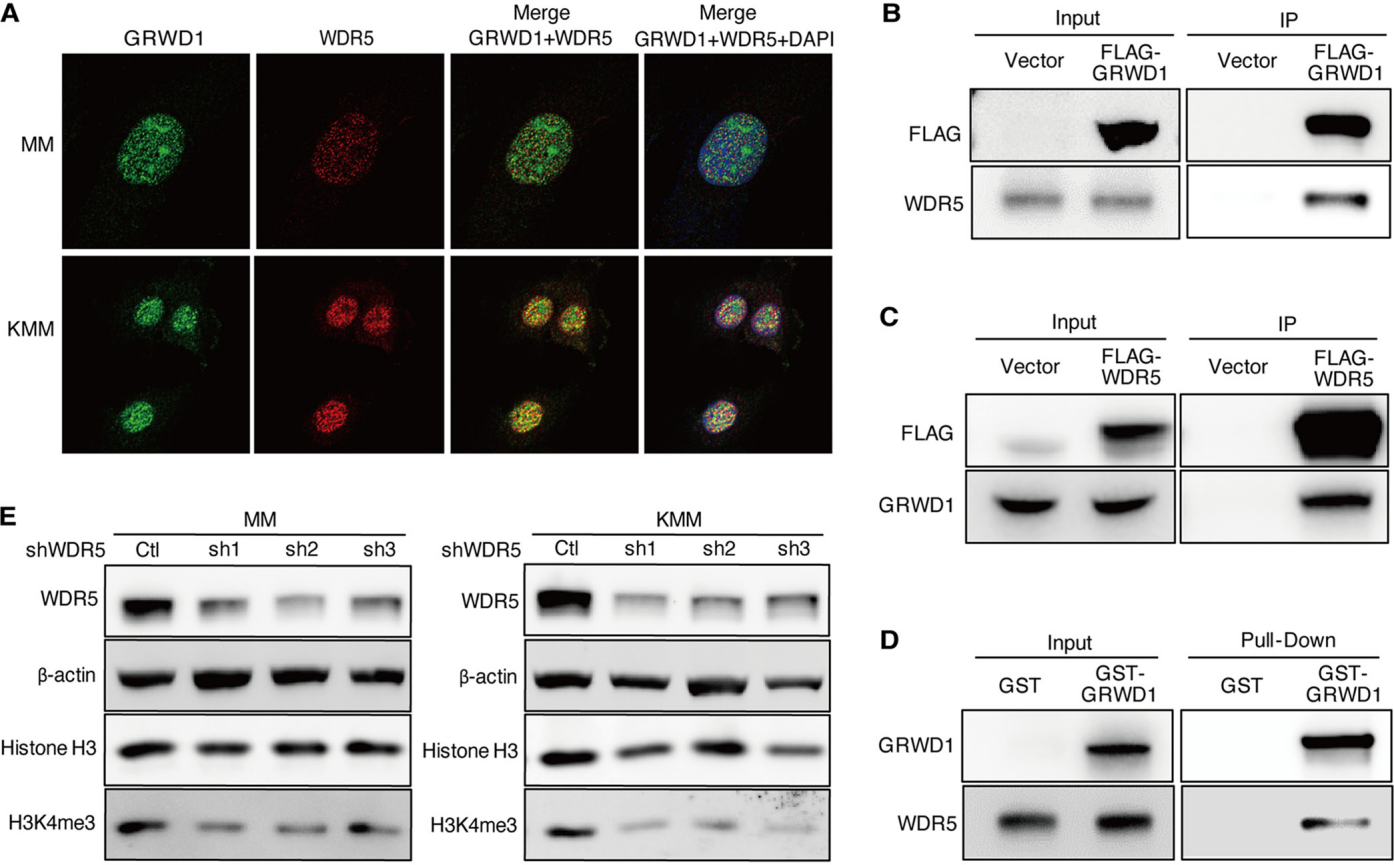
GRWD1 directly interacts with WDR5. (A) Confocal images showing the colocalization of GRWD1 and WDR5 in MM and KMM cells. (B) FLAG-GRWD1 immunoprecipitated endogenous WDR5 in 293T cells. (C) FLAG-WDR5 immunoprecipitated endogenous GRWD1 in 293T cells. (D) Purified recombinant GST-GRWD1 but not GST physically pulled down purified WDR5 *in vitro*. (E) WDR5 knockdown reduced the levels of total H3K4me3 in MM and KMM cells.

To investigate the mechanism of GRWD1 regulation of H3K4me3 modification, we searched the protein interaction databases IntAct (35) and BioGRID (36, 37). Among the potential binding partners of GRWD1, WDR5 mediates the assembly of MLL and SET1 histone methyltransferase complexes to regulate histone H3 methylation at lysine 4 (H3K4) (38, 39). WDR5 knockdown reduced the total H3K4 methylation level (27, 40). Hence, WDR5 might mediate GRWD1 regulation of histone H3K4 trimethylation. Indeed, confocal microscopy examination revealed that GRWD1 colocalized with WDR5 in both MM and KMM cells (Fig. 4A). In coimmunoprecipitation (co-IP), FLAG-GRWD1 specifically pulled down endogenous WDR5 (Fig. 4B) while FLAG-WDR5 specifically pulled down GRWD1 (Fig. 4C). Furthermore, *in vitro* pulldown assay revealed the direct interaction between GRWD1 and WDR5 proteins (Fig. 4D). Similar to GRWD1 knockdown, WDR5 knockdown also reduced the global H3K4me3 level in MM and KMM cells (Fig. 4E). These results indicate that GRWD1 and WDR5 physically interact with each other, and both regulate the H3K4me3 modifications.

### WDR5 knockdown phenocopies GRWD1 knockdown.

**FIG 5:**
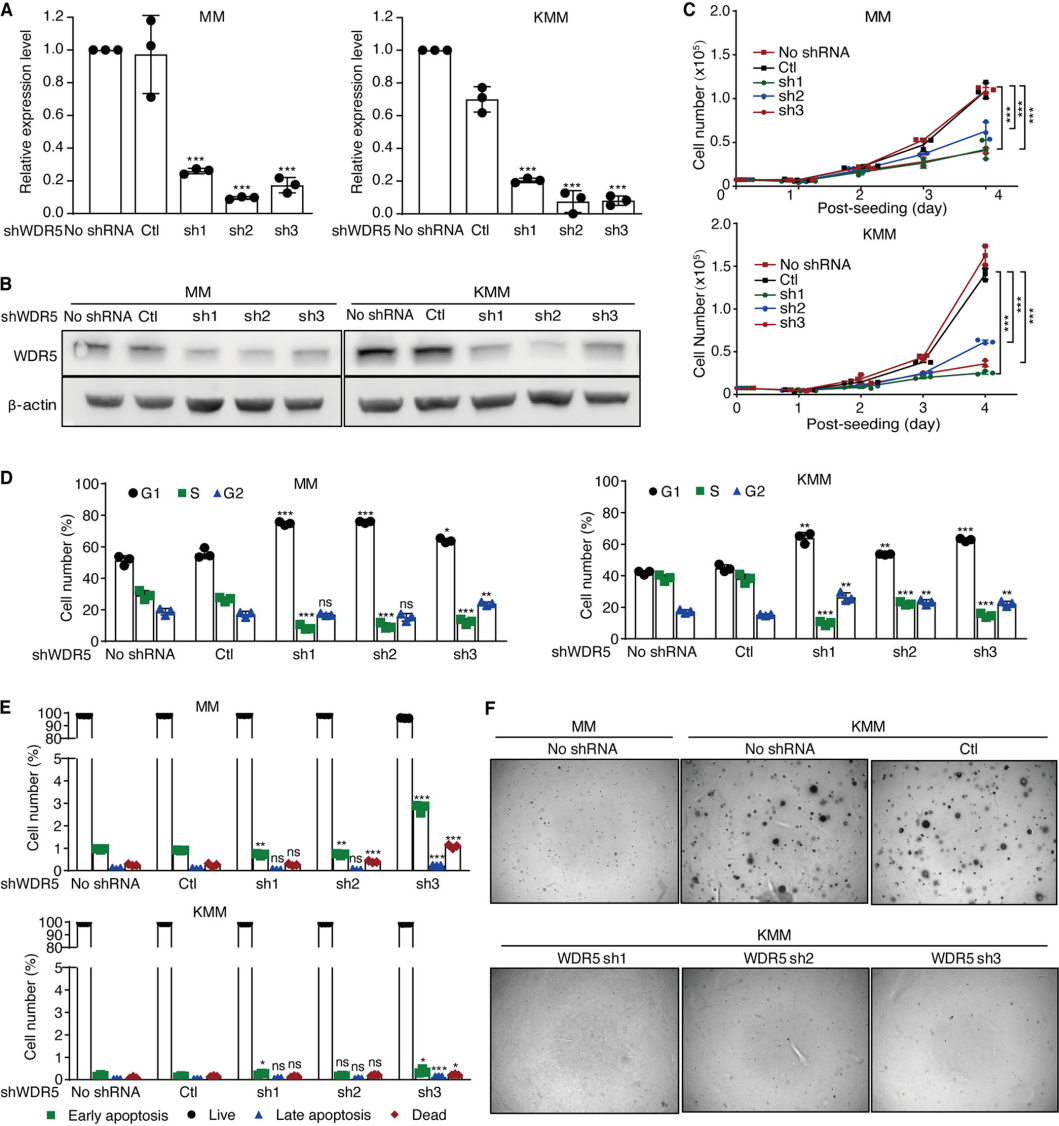
WDR5 knockdown phenocopies GRWD1 knockdown. (A and B) Knockdown efficiencies of WDR5 shRNAs examined by RT-qPCR (A) and Western blotting (B). (C to E) The effects of WDR5 knockdown on cell proliferation (C), cell cycle progression (D), and apoptosis (E) in MM and KMM cells. (F) WDR5 knockdown reduced the efficiency of colony formation on soft agar of KMM cells. *P* values are from comparisons between each of the shRNA-treated groups (sh1, sh2, and sh3) and the scrambled control (Ctl).

Since GRWD1 and WDR5 are in the same complex, the loss of WDR5 should have an effect on cells similar to the loss of GRWD1. We performed shRNA-mediated WDR5 knockdown in MM and KMM cells (Fig. 5A and B). Indeed, WDR5 knockdown inhibited the proliferation of both MM and KMM cells with a more profound effect observed in KMM than MM cells (Fig. 5C). WDR5 knockdown also induced cell cycle arrest but had minimal effect on apoptosis in both MM and KMM cells (Fig. 5D and E). Furthermore, WDR5 knockdown abolished colony formation of KMM cells in a soft agar assay (Fig. 5F). Together, these results show that knockdown of WDR5 has an effect on MM and KMM cells similar to that of GRWD1 knockdown, suggesting that the two proteins might regulate similar sets of genes.

### GRWD1 interacts with MLL2, and MLL2 knockdown phenocopies GRWD1 knockdown.

**FIG 6:**
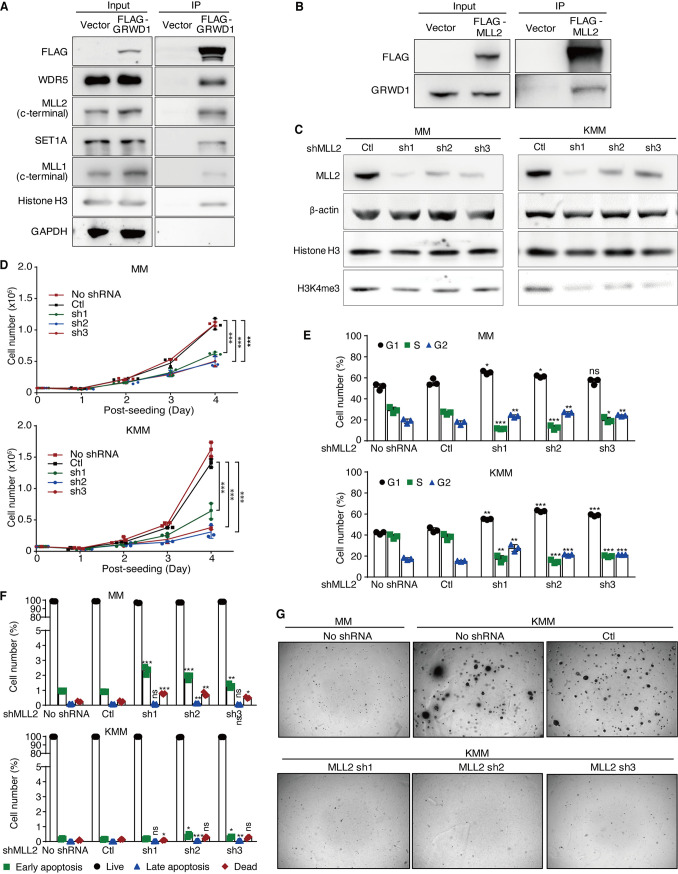
GRWD1 interacts with multiple methyltransferases, and MLL2 knockdown shares the same phenotype of GRWD1 knockdown. (A) FLAG-GRWD1 immunoprecipitated endogenous methyltransferases MLL1, MLL2, and SET1A in 293T cells. GAPDH, glyceraldehyde-3-phosphate dehydrogenase. (B) FLAG-MLL2 immunoprecipitated endogenous GRWD1 in 293T cells. (C) MLL2 knockdown reduced the level of H3K4me3 in MM and KMM cells. (D to F) The effects of MLL2 knockdown on cell proliferation (C), cell cycle progression (D), and apoptosis (F) in MM and KMM cells. *P* values are from comparisons between each of the shRNA-treated groups (sh1, sh2, and sh3) and the scrambled control (Ctl). (G) MLL2 knockdown reduced the efficiency of colony formation on soft agar of KMM cells.

Since WDR5 was required for the assembly of MLLs and SET1 histone methyltransferase complexes, we examined the interaction of GRWD1 with this complex. GRWD1 immunoprecipitated MLL2, SET1A, and MLL1 with MLL2 having the strongest interaction (Fig. 6A). Co-IP further showed the interaction between GRWD1 and MLL2 (Fig. 6B). Therefore, MLL2 is likely one of the major methyltransferases in the GRWD1 complexes.

To confirm if MLL2 is important to maintain the global level of H3K4me3 in our model, we examined the H3K4me3 mark following MLL2 knockdown. Similar to GRWD1 and WDR5, MLL2 knockdown reduced the level of H3K4me3 in both MM and KMM cells (Fig. 6C). Consistent with these results, MLL2 knockdown reduced cell proliferation of both MM and KMM cells with a stronger effect observed in KMM than MM cells (Fig. 6D). Similarly, MLL2 knockdown induced cell cycle arrest but had a minimal effect on apoptosis in both MM and KMM cells (Fig. 6E and F). MLL2 knockdown also abolished colony formation of KMM cells in soft agar (Fig. 6G). Hence, MLL2 knockdown shared the same phenotype as that of GRWD1 or WDR5 knockdown.

### GRWD1, WDR5, and MLL2 share the same complex to regulate specific sets of genes in primary and KSHV-transformed cells.

**FIG 7:**
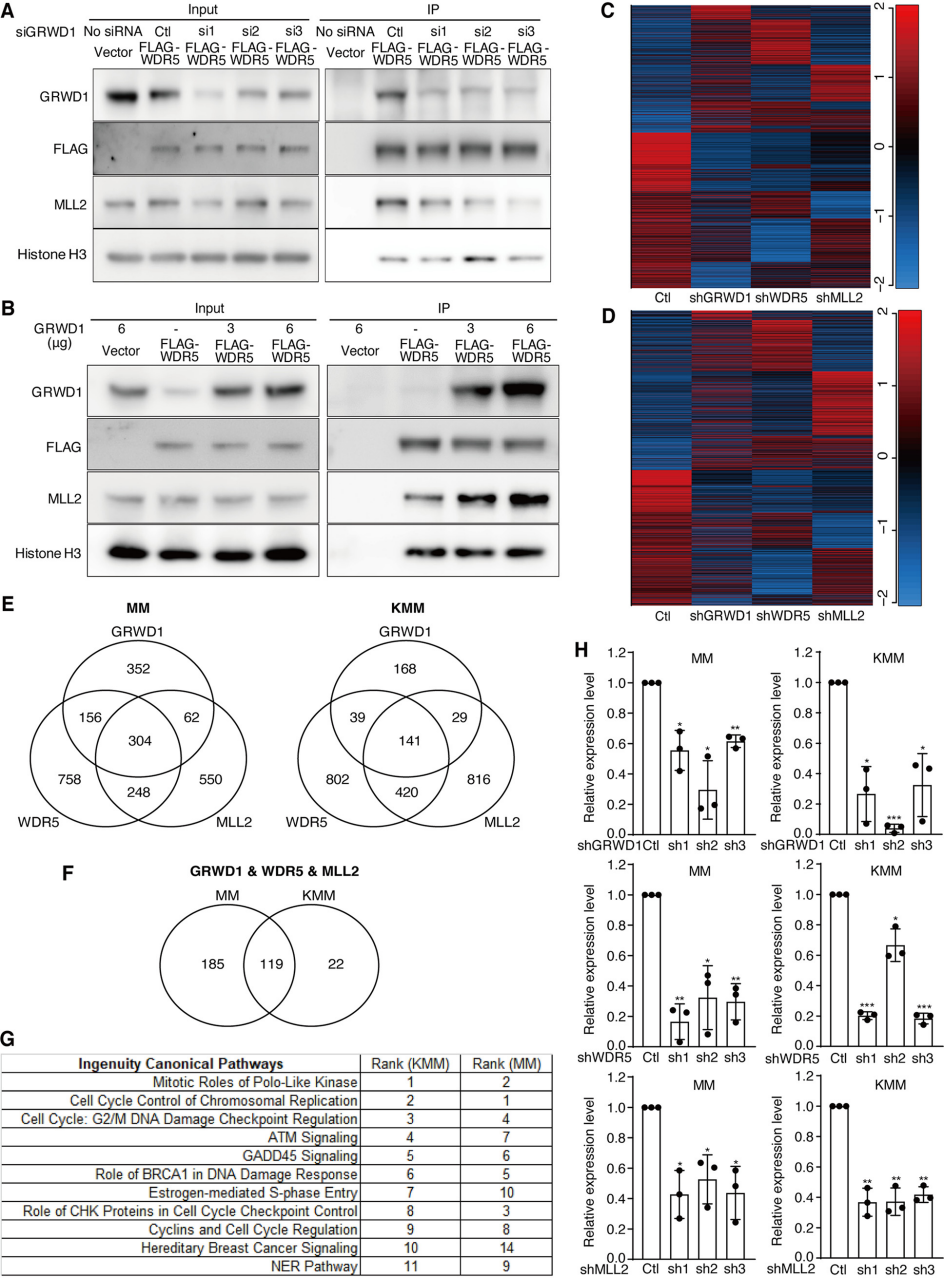
GRWD1 is important for maintaining the GRWD1-WDR5-MLL2 complex, but the three proteins also regulate distinct sets of genes. (A) GRWD1 knockdown reduced the efficiency of WDR5 immunoprecipitation of MLL2 but not histone H3. (B) GRWD1 overexpression increased the efficiency of WDR5 immunoprecipitation of MLL2 but not histone H3. (C and D) Heatmap of differential gene expression after GRWD1, WDR5, or MLL2 knockdown in MM (C) and KMM (D) cells. (E) Common and unique gene sets altered following GRWD1, WDR5, or MLL2 knockdown. (F) Shared and distinct common genes altered following GRWD1, WDR5, or MLL2 knockdown between MM and KMM cells. (G) The rank of the top enriched pathways of common genes altered following GRWD1, WDR5, or MLL2 knockdown in MM and KMM cells. NER, nucleotide excision repair. (H) RT-qPCR validation of CDK1 expression in MM and KMM cells after GRWD1, WDR5, or MLL2 knockdown. *P* values are from comparisons between each of the shRNA-treated groups (sh1, sh2, and sh3) and the scrambled control (Ctl).

Because GRWD1, WDR5, and MLL2 interacted with one another and regulated H3K4me3 mark and cell proliferation, we examined the role of GRWD1 in the complex. In co-IP, GRWD1 knockdown reduced the amount of MLL2 protein pulled down by WDR5 (Fig. 7A). Conversely, overexpression of GRWD1 increased the amount of MLL2 protein pulled down by WDR5 in a dose-dependent manner (Fig. 7B). However, the ability of WDR5 to bind to histone H3 was not affected under both conditions (Fig. 7A and B). These results indicate that GRWD1 protein is essential for maintaining the interaction between WDR5 and MLL2 and hence the stability of the GRWD1-WDR5-MLL2 complex.

We performed RNA-seq after shRNA-mediated knockdown of GRWD1, WDR5, or MLL2 and identified genes that were regulated by the GRWD1-WDR5-MLL2 complex using *P* < 0.05 and >1.3-fold change as filters. The heatmaps indeed showed that GRWD1, WDR5, and MLL2 coregulated subsets of genes in MM and KMM cells, respectively (Fig. 7C to E; see also Table S3A and B). However, GRWD1, WDR5, and MLL2 alone or in combination also regulated distinct sets of genes, respectively (Fig. 7C to E; see also Table S4A to F), suggesting that these proteins might form different complexes with or independent of one another. As WDR5 coexists in all MLL2 methyltransferase complexes (38, 39), we observed more common genes shared between WDR5 and MLL2 than between GRWD1 and WDR5 or MLL2 in both MM and KMM cells (Fig. 7E). Of the 304 and 141 genes that were coregulated by GRWD1, WDR5, and MLL2 in MM and KMM cells, respectively, 119 genes are shared between the two types of cells (Fig. 7F; see also Table S3A and B). We identified different subsets of genes that were regulated by GRWD1, WDR5, or MLL2 alone, or coregulated by one another shared by MM and KMM cells (Fig. S4; see also Table S3A and B and Table S4A to F), which supported the common and distinct phenotypes of the two types of cells observed following knockdown of each of these genes.

10.1128/mbio.03431-21.4 FIG S4 Common and distinct genes altered in MM and KMM cells following GRWD1, WDR5, or MLL2 knockdown. Download FIG S4, PDF file, 0.1 MB. Copyright © 2021 Wei et al. 2021 Wei et al. https://creativecommons.org/licenses/by/4.0/ This content is distributed under the terms of the Creative Commons Attribution 4.0 International license.

10.1128/mbio.03431-21.8 TABLE S3 Common and distinct genes altered in MM (A) and KMM (B) cells following knockdown of GRWD1, WDR5, or MLL2. Download Table S3, PDF file, 0.5 MB. Copyright © 2021 Wei et al. 2021 Wei et al. https://creativecommons.org/licenses/by/4.0/ This content is distributed under the terms of the Creative Commons Attribution 4.0 International license.

10.1128/mbio.03431-21.9 TABLE S4 Top altered genes following knockdown of GRWD1 (A and D), WDR5 (B and E), and MLL2 (C and F) in MM (A, B, and C) and KMM (D, E, and F) cells. Download Table S4, PDF file, 0.9 MB. Copyright © 2021 Wei et al. 2021 Wei et al. https://creativecommons.org/licenses/by/4.0/ This content is distributed under the terms of the Creative Commons Attribution 4.0 International license.

We performed Ingenuity Pathway Analysis (IPA) to identify the pathways of the coregulated genes of GRWD1, WDR5, and MLL2 in MM and KMM cells (Table S5A and B). Among the top 11 enriched pathways identified in MM cells, 10 were also enriched in KMM cells, many of which were involved in cell cycle progression, cytokinesis, and DNA repair (Fig. 7G), explaining the common phenotypes observed in these two types of cells. However, we also observed numerous enriched pathways in one cell type but not the other, for example, cell cycle regulation by BTG family proteins and Myc-mediated apoptosis signaling. We confirmed the changes of four cell cycle-related genes, CDK1, CDK2, CDT1, and PCNA, as the common downregulated genes following GRWD1, WDR1, or MLL2 knockdown in both MM and KMM cells (Fig. 7H; see also Fig. S5). Together, these results indicate that the GRWD1-WDR5-MLL2 complex mediates cell proliferation by regulating the expression of key cell cycle genes.

10.1128/mbio.03431-21.5 FIG S5 RT-qPCR validation of CDK2, CDT1, and PCNA expression after GRWD1, WDR5, or MLL2 knockdown in MM and KMM cells. *P* values are from comparisons between each of the shRNA-treated groups (sh1, sh2, and sh3) and the scrambled control (Ctl). Download FIG S5, PDF file, 0.6 MB. Copyright © 2021 Wei et al. 2021 Wei et al. https://creativecommons.org/licenses/by/4.0/ This content is distributed under the terms of the Creative Commons Attribution 4.0 International license.

10.1128/mbio.03431-21.10 TABLE S5 Top enriched pathways of common genes after GRWD1, WDR5, or MLL2 knockdown in MM (A) and KMM (B) cells. Download Table S5, PDF file, 0.3 MB. Copyright © 2021 Wei et al. 2021 Wei et al. https://creativecommons.org/licenses/by/4.0/ This content is distributed under the terms of the Creative Commons Attribution 4.0 International license.

## DISCUSSION

It has been well studied that the KSHV episome is subjected to epigenetic modifications, including DNA methylation and histone methylation and acetylation (22, 41–45). KSHV utilizes the epigenetic machinery of the host cell to control its life cycle (41–44). On the other hand, multiple KSHV viral genes can also act as epigenetic regulators to manipulate the expression of cellular genes (44–47). For example, KSHV LANA has been reported to interact with both DNA and histone methyltransferase complexes and is associated with promoters of multiple cellular genes (22, 48). In this study, we have shown alterations of H3K4me3 and H3K27me3 marks in the predominantly latent KSHV-transformed KMM cells, providing direct evidence that KSHV latent products might alter the global histone methylation pattern of the host genome. Indeed, we have previously shown that KSHV latent products vFLIP and LANA enhance KSHV-induced angiogenesis by upregulating an epigenetic regulator, enhancer of zeste homolog 2 (EZH2), through the NF-κB pathway (23). These results suggest that KSHV might induce cellular transformation by reprograming the cellular epigenome.

In a CRISPR-Cas9 screening, we have previously identified cellular epigenetic regulators that are essential for the survival of KSHV-transformed cells. We speculated that these epigenetic factors might mediate KSHV reprogramming of the cellular epigenome. Among the top genes, GRWD1 is an oncogene (28) and has been speculated on as a potential epigenetic regulator (26). Interestingly, GRWD1 is upregulated in KSHV-transformed cells (Fig. 2B), and high expression of GRWD1 predicts poor patient survival in numerous types of cancer (Fig. 1G). Similarly, we have identified 8 other epigenetic regulators that have been reported to play important roles in cancer (28, 49–54), and their expression levels predict the survival of various types of cancer patients (see Fig. S1 in the supplemental material). For example, NFYB was reported to induce the high expression of E2F1 in colorectal cancer and mediate oxaliplatin resistance (49). Thus, the KSHV-induced cellular transformation system is useful for identifying essential genes not only for KSHV-induced cancers but also for other types of cancer.

Although GRWD1 has been identified as a histone-binding protein (26), its role in epigenetic modification remains unclear. A previous study showed that it was pulled down by CUL4–DDB1 ubiquitin ligase together with several methyltransferase core proteins, suggesting its potential role in histone methylation (27). In this study, we have shown that knockdown of GRWD1 leads to a global reduction of H3K4me3 marks by Western blotting and ChIP-seq, confirming an essential role of GRWD1 in maintaining cellular H3K4me3 marks. We have identified the GRWD1-regulated H3K4me3 peaks in the promoters of specific genes in both KSHV-transformed cells and primary cells (Fig. 3B to G; see also Table S2A and B).

To understand the mechanism of GRWD1 mediating epigenetic modification, we have confirmed the direct interaction of GRWD1 with the histone H3 lysine 4 (H3K4) methyltransferase core protein WDR5. Since WDR5 is a core protein of human MLL and SET1 H3K4 methyltransferase complexes (38, 39), GRWD1 can potentially regulate H3K4me3 peaks by interacting with these complexes. Indeed, we have identified MLL2 as the major GRWD1-interacting H3K4 methyltransferase. Using both knockdown and overexpression approaches, we have found GRWD1 is directly involved in the interaction of WDR5 and MLL2, possibly by serving as a bridging factor to connect these two proteins in the GRWD1-WDR5-MLL2 complex and affecting the recruitment of MLL2 to WDR5. Although the absence of MLL2 did not show global bulk downregulation of H3K4 methylation in mouse embryonic stem cells or fibroblasts (55, 56), other studies revealed the reduction of H3K4me3 marks by ChIP-seq and H3K4me3 levels by Western blotting after MLL2 knockdown (57, 58), which are consistent with our observations. In agreement with the report that MLL2 is essential for maintaining the H3K4me3 level on bivalent promoters of genes with low expression levels (56, 58, 59), we have also identified a set of GRWD1 targets located at bivalent promoters of genes by ChIP-seq, including SOHLH1 and HS3ST3B1 shared by MM and KMM cells (Fig. 3E and G; see also Fig. S3B). However, many H3K4me3 peaks affected by GRWD1 knockdown, such as those of the ADAR gene, were not at bivalent promoters (Fig. 3D to G; see also Fig. S3B), suggesting potential GRWD1 interactions with other methyltransferases in addition to MLL2. Indeed, we have found genes that are coregulated by GRWD1 and WDR5 but not MLL2, which could be downstream targets of other methyltransferases (Fig. 7E). Similarly, we have identified genes that are coregulated by GRWD1 and MLL2 but not WDR5 and genes that are coregulated by WDR5 and MLL2 but not GRWD1 (Fig. 7E). Furthermore, we have identified genes that are regulated by GRWD1, WDR5, or MLL2 alone. These results indicate that these proteins might also independently form complexes with other proteins without involving one another.

Among the common pathways that are enriched following knockdown of GRWD1, WDR5, or MLL2, most of them are involved in cell cycle progression (Fig. 7G), suggesting the important role of the GRWD1-WDR5-MLL2 complex in this pathway. Consistent with these results, knockdown of any of the three proteins caused cell cycle arrest (Fig. 2D, Fig. 5D, and Fig. 6E). Hence, the GRWD1-WDR5-MLL2 complex might mediate KSHV reprogramming of the epigenome and contribute to cell cycle progression and cellular transformation. Among the KSHV products that can alter epigenetic modifications, LANA is associated with human H3K4 methyltransferase complexes (22) and can directly bind to viral and cellular genomes. It is possible that LANA might interact with the GRWD1-WDR5-MLL2 complex to regulate specific epigenetic loci on the genome. Further investigation of KSHV hijacking of the host machinery to alter the specific epigenetic marks on both viral and cellular genomes could provide insights into the mechanism of KSHV-induced oncogenesis.

Taken together, we have identified an epigenetic complex that mediates KSHV-induced cellular transformation and cell cycle progression by reprogramming the cellular epigenome and gene expression. This complex represents a potential novel therapeutic target for KSHV-induced cancers, which could be extended to other types of cancers.

## MATERIALS AND METHODS

### Cell culture.

MM cells and 293T cells were cultured in Dulbecco modified Eagle medium (DMEM) (25-500; Genesee) with 10% fetal bovine serum (F2442; Sigma-Aldrich). KMM cells were cultured under the same condition as MM cells except with 250 μg/mL of hygromycin. MM and KMM cells with stable GRWD1 knockdown by lentivirus infection were cultured in their respective media with 1 μg/mL and 5 μg/mL of puromycin, respectively. Cells were cultured in medium without selection for 1 week before any experiments.

### Plasmids, shRNAs, and siRNAs.

The shRNA plasmid was constructed by inserting the shRNA oligonucleotides (Integrated DNA Technologies) into the pLKO.1 lentiviral vector. The shRNA oligonucleotide sequences were as follows: GRWD1 shRNA1 (F, 5′-CCGGGGAGCTGGTAATGGATGAAGACTCGAGTCTTCATCCATTACCAGCTCCTTTTTG-3′; R, 5′-AATTCAAAAAGGAGCTGGTAATGGATGAAGACTCGAGTCTTCATCCATTACCAGCTCC-3′), GRWD1 shRNA2 (F, 5′-CCGGGGATGGTGGTTCCTGGAATGTCTCGAGACATTCCAGGAACCACCATCCTTTTTG-3′; R, 5′-AATTCAAAAAGGATGGTGGTTCCTGGAATGTCTCGAGACATTCCAGGAACCACCATCC-3′), GRWD1 shRNA3 (F, 5′-CCGGGCAGTTGCTGTTCGTGCATCACTCGAGTGATGCACGAACAGCAACTGCTTTTTG-3′; R, 5′-AATTCAAAAAGCAGTTGCTGTTCGTGCATCACTCGAGTGATGCACGAACAGCAACTGC-3′), WDR5 shRNA1 (F, 5′-CCGGGCTCATTGATGACGACAATCCCTCGAGGGATTGTCGTCATCAATGAGCTTTTTG-3′; R, 5′-AATTCAAAAAGCTCATTGATGACGACAATCCCTCGAGGGATTGTCGTCATCAATGAGC-3′), WDR5 shRNA2 (F, 5′-CCGGGGGAAGTTCCTGGTCTGTTCTCTCGAGAGAACAGACCAGGAACTTCCCTTTTTG-3′; R, 5′-AATTCAAAAAGGGAAGTTCCTGGTCTGTTCTCTCGAGAGAACAGACCAGGAACTTCCC-3′), WDR5 shRNA3 (F, 5′-CCGGGCAGCTTGCGAGGTCAATACTCTCGAGAGTATTGACCTCGCAAGCTGCTTTTTG-3′; R, 5′-AATTCAAAAAGCAGCTTGCGAGGTCAATACTCTCGAGAGTATTGACCTCGCAAGCTGC-3′), MLL2 shRNA1 (F, 5′-CCGGGCGGCTGTGACAATCCCTAAACTCGAGTTTAGGGATTGTCACAGCCGCTTTTTG-3′; R, 5′-AATTCAAAAAGCGGCTGTGACAATCCCTAAACTCGAGTTTAGGGATTGTCACAGCCGC-3′), MLL2 shRNA2 (F, 5′-CCGGGCAGAATGAGTGGACACATGTCTCGAGACATGTGTCCACTCATTCTGCTTTTTG-3′; R, 5′-AATTCAAAAAGCAGAATGAGTGGACACATGTCTCGAGACATGTGTCCACTCATTCTGC-3′), and MLL2 shRNA3 (F, 5′-CCGGGGTCTGAAGATGAATCCATGGCTCGAGCCATGGATTCATCTTCAGACCTTTTTG-3′; R, 5′-AATTCAAAAAGGTCTGAAGATGAATCCATGGCTCGAGCCATGGATTCATCTTCAGACC-3′). The small interfering RNAs (siRNAs) against GRWD1 were obtained from Sigma-Aldrich (SASI_Rn01_00060441, SASI_Rn01_00060442, SASI_Rn01_00060443).

The overexpression plasmid was constructed by cloning the coding sequence from cDNA of 293T cells with an N-terminal FLAG tag into pCDH vector using the following primers: GRWD1 (F, 5′-TGCTTATCTAGACGGCCACCATGGATTACAAGGATGACGACGATAAGGCGGCGCGCAAGG-3′; R, 5′-TGCTTAGAATTCTCAGACGCTGATGGTGCG-3′) and WDR5 (F, 5′-TGCTTATCTAGACGGCCACCATGGATTACAAGGATGACGACGATAAGGCGACGGAGGAGAAGAAGC-3′; R, 5′-TAAGCAGAATTCTTAGCAGTCACTCTTCCACAGTTTAATTG-3′).

The FLAG tag MLL2 C-terminal plasmid pcDNA3 MLL2 653 was a gift from Matthew Meyerson (Addgene plasmid no. 11017; http://n2t.net/addgene:11017; RRID: Addgene_11017).

### Lentiviral infection.

Supernatants of 293T cells transfected with p8.74 and pMDG packaging vectors as well as the shRNA vectors were collected and filtered at 48 or 72 h after the transfection. Viral transduction was performed by spinning infection at 1,500 rpm for 1 h with Polybrene at 10 μg/mL. The knockdown efficiency was examined at 48 or 72 h posttransduction.

### Soft agar assay.

The soft agar assay was performed as previously described (10). Briefly, 2 × 10^4^ cells suspended in 1 mL of 0.3% top agar (A5431; Sigma-Aldrich) were plated onto 0.5% base agar in one well of 6-well plates and covered by cultured medium. After 3 weeks, the plates were photographed with a microscope under a 2× lens objective, and colonies with diameters of >50 μm were counted.

### Cell cycle and apoptosis assays.

Cell cycle analysis was performed by propidium iodide (PI) staining, and flow cytometry was carried out with a FACSCanto II flow cytometry system (BD Biosciences). The fixable viability dye eFluor 660 kit (650864; eBioscience) and a phycoerythrin (PE)-Cy7 annexin V apoptosis detection set (88810374; eBioscience) were used to detect apoptotic cells following the instructions of the manufacturer. The data were analyzed with the FlowJo software (BD Biosciences).

### GST pulldown assay.

Purified recombinant glutathione *S*-transferase (GST), GST-GRWD1, and WDR5 proteins were purchased from Abcam (ab81793, ab164438, and ab98079). Recombinant WDR5 protein was incubated with GST-GRWD1 or GST protein and then bound with glutathione Sepharose 4B (GE17-0756-01; Sigma-Aldrich). The beads were washed five times, and the pulldown proteins were eluted and analyzed by Western blotting.

### Immunofluorescence assay, co-IP, and Western blotting.

For immunofluorescence assay, cells were fixed with 4% paraformaldehyde (P6148; Sigma-Aldrich) for 12 min at room temperature, permeabilized with 100% methanol at −20°C for 12 min, and blocked with 1% bovine serum albumin (BSA) and 0.1% Tween 20 in phosphate-buffered saline (PBS) for 1 h. The cells were stained with primary antibodies for 1 h followed by 1 h of incubation with Alexa Fluor-labeled secondary antibodies (Invitrogen, Themo Fisher Scientific) at room temperature. Primary and secondary antibodies were diluted in 3% BSA in PBS. 4′,6-Diamidino-2-phenylindole (DAPI) (D9542; Sigma-Aldrich) was used for the nuclear counterstaining.

Co-IP experiments were performed with the anti-FLAG M2 affinity gel (A2220; Sigma-Aldrich) according to the manufacturer’s instructions. Cell lysates were treated with Benzonase Nnuclease (E1014; Sigma-Aldrich) for 1 h with rotation at 4°C. Before adding the affinity gel, the lysates were precleared with mouse IgG-agarose (A0919; Sigma-Aldrich) with rotation at 4°C for 4 h.

For Western blotting, protein samples were resolved by SDS-PAGE and transferred to nitrocellulose membranes (10600004; GE Healthcare). Protein signals were detected with chemiluminescent substrate (34096; Thermo Scientific) after the incubation with primary and secondary antibodies.

Mouse monoclonal antibodies against GRWD1 (sc-514125; Santa Cruz), histone H3 (sc-517576; Santa Cruz), and β-actin (sc-376421; Santa Cruz) and rabbit monoclonal antibodies against WDR5 (catalog no.13105; Cell Signaling Technology), MLL2 C-terminal sequence (catalog no. 38058; Cell Signaling Technology), H3K4me3 (catalog no. 9751; Cell Signaling Technology), FLAG tag (catalog no. 14793; Cell Signaling Technology), and GST tag (catalog no. 2625; Cell Signaling Technology) were used for the experiments.

### Real-time quantitative reverse transcription-PCR (RT-qPCR) and RNA-seq.

Cells were collected and lysed in TRI reagent (T9424; Sigma-Aldrich), and the total RNA was isolated following the manufacturer’s instructions. Reverse transcription was performed with the isolated total RNA with the Maxima H Minus first-strand cDNA synthesis kit (K1652; Thermo Scientific). The SsoAdvanced universal SYBR green supermix kit (172-5272; Bio-Rad) was used for the quantitative PCR. The primers used for these experiments were as follows (for Rattus norvegicus): GRWD1 (F, 5′-GTGAGGGCTTTGCTCTTGAC-3′; R, 5′-CACTGCAGATCCTCCACAGA-3′), WDR5 (F, 5′-GGTGCACCTCCTCTCTGAAG-3′; R, 5′-TGTGCACTGGGCAATACAAT-3′), and MLL2 (F, 5′-TGCTCAGTGGAGACAACAGG-3′; R, 5′-ACCAAATGGCACAGTTGACA-3′).

The isolated mRNA was used to prepare the RNA-seq library using the Illumina TruSeq stranded mRNA-seq sample preparation guide (Illumina, San Diego, CA, USA) and subjected to sequencing using a 50-bp single-read sequencing module with a HiSeq 3000 sequencing system from Illumina.

### ChIP-seq and ChIP-qPCR.

ChIP experiments were conducted using the SimpleChIP enzymatic chromatin IP kit (magnetic beads) (catalog no. 9003; Cell Signaling Technology) according to the manufacturer’s instructions. The ChIP DNA was used for the ChIP-seq library preparation by using the Swift Biosciences Accel-NGS 2S Plus DNA library kit (Swift Biosciences, Ann Arbor, MI, USA) and subjected to sequencing using a 50-bp single-read sequencing module with the HiSeq 3000 sequencing system from Illumina.

ChIP DNA was analyzed by quantitative PCR with the SsoAdvanced universal SYBR green supermix kit and the following primers (for Rattus norvegicus promoters): ADAR (F, 5′-GTAGCCTTCAGGAGAGTCGG-3′; R, 5′-GGACCAGAGCAGGTAACAACA-3′), OAS1A (F, 5′-TCGACTGGATTGATGGACCC-3′; R, 5′-AATGCGATTCGAAGGACCAGT-3′), IL1A (F, 5′-TGCTGATAGACTCGCTCACG-3′; R, 5′-GAGAACTTAGGGAGCAGCTGAA-3′), BST2 (F, 5′-TCAAGTTCCTTGATGCGGGC-3′; R, 5′-TAACAGCCAGCCCATGTTTCT-3′), SOHLH1 (F, 5′-GGGCACTACTGCCTCAGTTT-3′; R, 5′-AGCTAGGATCCATGCTGTGG-3′), ZFP112 (F, 5′-CAGTCACCTGGATGGAGGAT-3′; R, 5′-TTTGAGCCTTGCAGGAAACT-3′), ADAMTS19 (F, 5′-CCCTTTGCAGAGCGTGTACT-3′; R, 5′-ACCAGAGGAGCAGTCCAGTC-3′), and HS3T3B1 (F, 5′-AGAAGCTCGAGATGGGACTG-3′; R, 5′-TGATCACAGCTCCGAATGAG-3′).

### Bioinformatic analysis.

We performed patients’ survival analysis using the Kaplan-Meier survival analysis for selected epigenetic genes based on their gene expression data to partition tumors. For expression values of each gene in a particular tumor type, we used μ − δ and μ + δ to partition tumors into three groups, where μ and δ are the mean and standard deviation (SD) of the expression values of the gene in tumors, respectively.

For the RNA-seq data processing, we first used Partek flow to process raw RNA-seq data and matched sequenced RNA segments into genes to obtain expression values of genes. Then, the gene expression data were normalized with dChip (60). For each gene *g*, we obtained a set of expression values for all control samples and a set of expression values for test samples of each shRNA knockdown of GRWD1, WDR5, or MLL2. The gene *g* was determined as differentially expressed if (i) fold change of mean values for control samples and test samples was at least 1.3 and (ii) *t* test *P* value for control samples and test samples was at most 0.05.

For ChIP-seq analysis, quality control, read mapping, and signal track visualization were performed by deploying the ChIP-seq pipeline developed in the ENCODE project (61). Specifically, we used Bowtie2 (62) to align the single-end read fragments to the Rattus norvegicus genome (Rn6). Bowtie2 was configured with default parameters. Signal tracks of fold change and *P* values were generated from MACS2 by comparing each immunoprecipitation profile with its corresponding background/input profile.

Within each peak set generated by MACS2, the peak regions were restricted to 150 bp downstream and upstream to the peak summit. Hence, the width of peak regions was fixed at 301 bp. To generate the consensus peak set across samples, the peak summits were recentered to the optimal enrichment across samples within the same condition. We used DiffBind (63) to quantify read counts in consensus peak sets. Read counts were then normalized with TMM (64) and minus the full library size of input. We visualized the similarity among samples with heatmap and multidimensional scaling (MDS). Samples were removed before differential analysis if they were not consistent with other repeats (i.e., the H3K4me3 data of the second replicate of GRWD1 knockdown in KMM cells and the H3K27me3 data of the first replicate of control cells).

With the read counts of the remaining samples, we used EdgeR (64) to perform differential analysis on GRWD1 knockdown versus vehicle control within each cell type and each histone position. Peak regions with *P* values of <0.05 and absolute log fold change of >0.5 were selected as differential binding sites. Heatmaps were also plotted to verify the effect size. We then used ChIPseeker (65) to annotate the binding sites with their closest genes.

### Tumor growth in mice.

The growth of subcutaneous tumors was performed as previously described (10). Cells at 5 × 10^6^ were injected into both flanks of the nude mice. Tumor volumes were measured twice a week using 0.2 cm^3^ as a threshold. The experiment was terminated at week 21 following inoculation. Mice were terminated when the tumor volume reached 1.5 cm^3^. Tumor analysis was performed as previously described (10).

### Statistical analysis.

Data are shown as mean ± SD (standard deviations) where appropriate. The two-tailed Student *t* test or one-way analysis of variance (ANOVA) was used to compare data between the experimental groups. Statistical significance was considered at *P* values less than 0.05, 0.01, or 0.001, represented by *, **, or ***, respectively.

### Data availability.

All the RNA-seq and ChIP-seq results are available at GenBank, Project ID PRJNA781746.
