# Supplementary figures and images for: GRWD1-WDR5-MLL2 Epigenetic Complex Mediates H3K4me3 Mark and Is Essential for Kaposi’s Sarcoma-Associated Herpesvirus-Induced Cellular Transformation
Source: mBio. 2021 Dec 21;12(6):e03431-21. doi: 10.1128/mbio.03431-21 (PMC8689518; doi:10.1128/mbio.03431-21)

Supplemental Figure S1

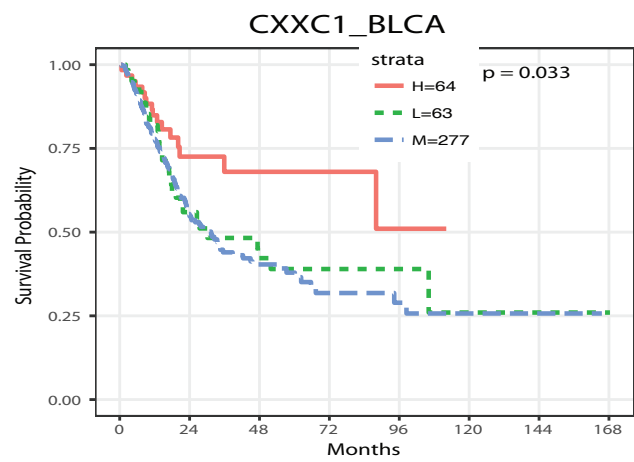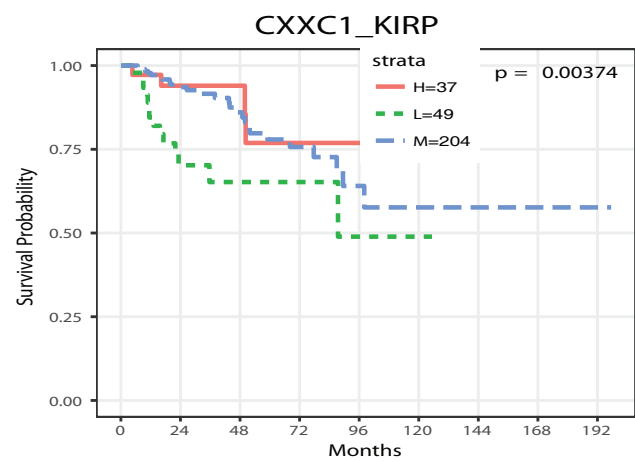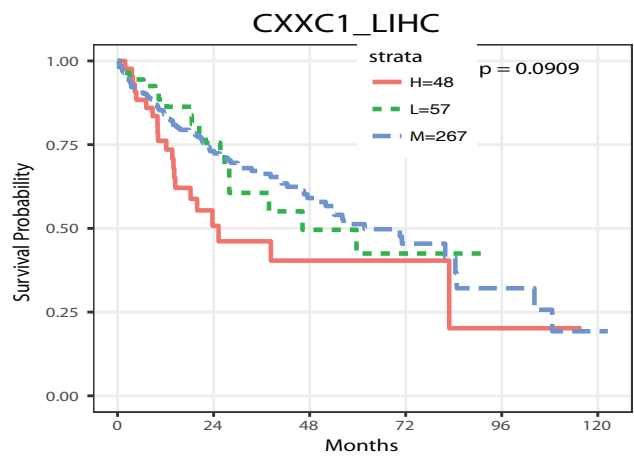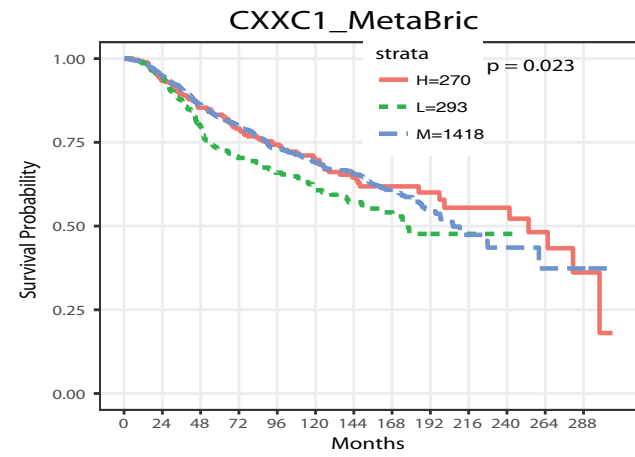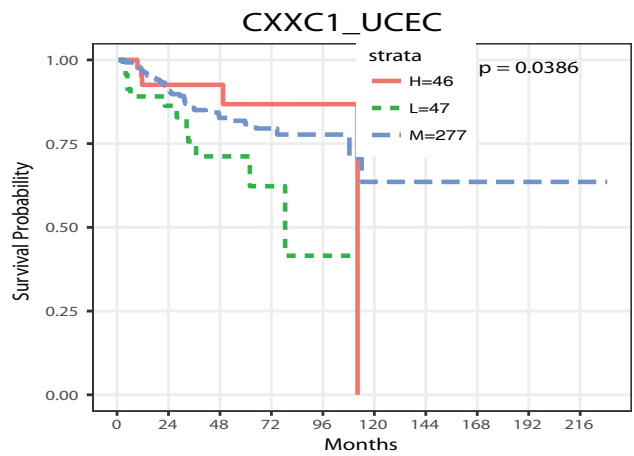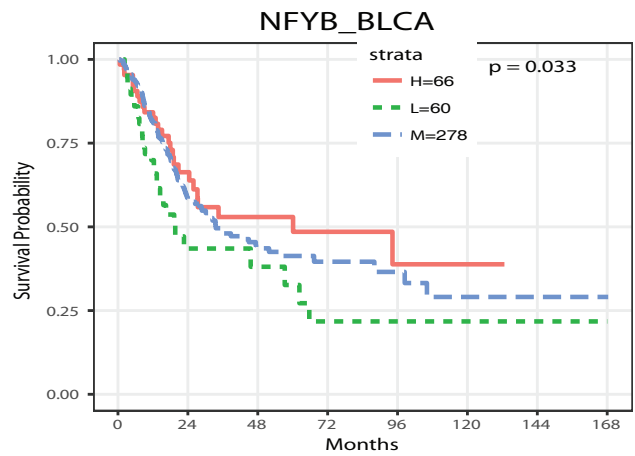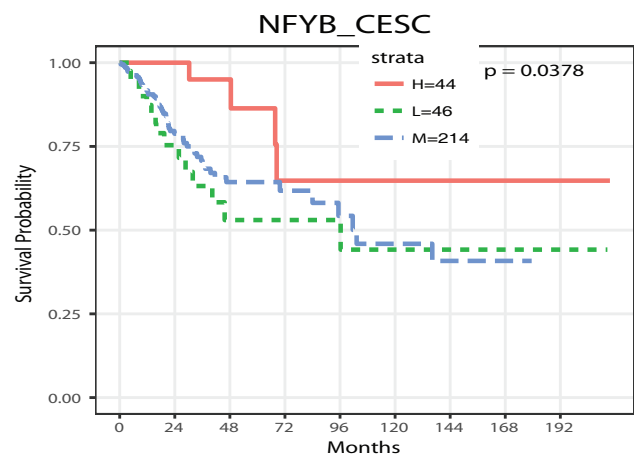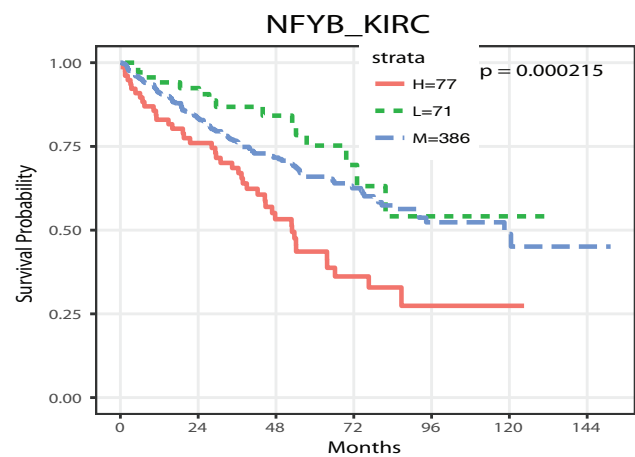

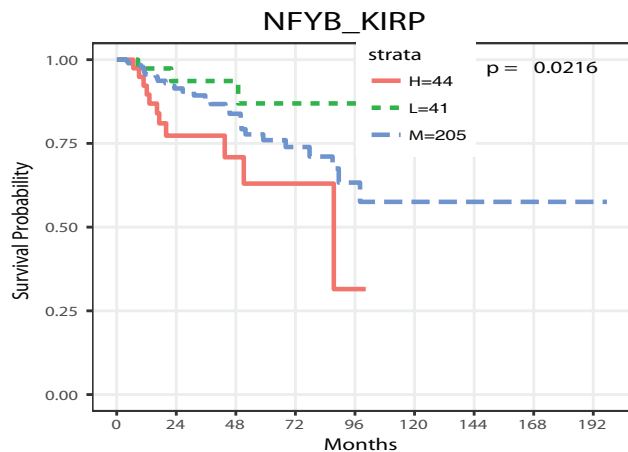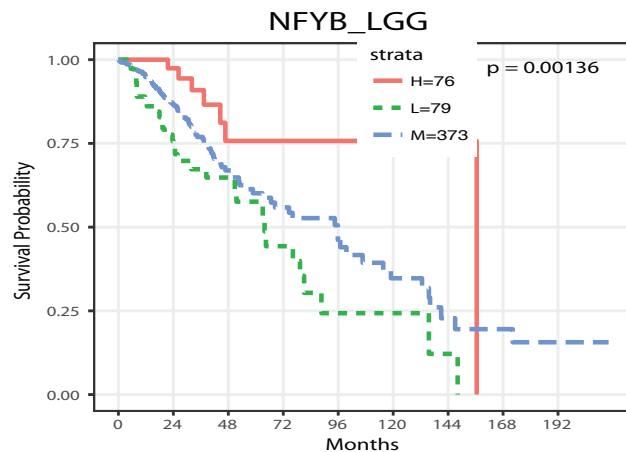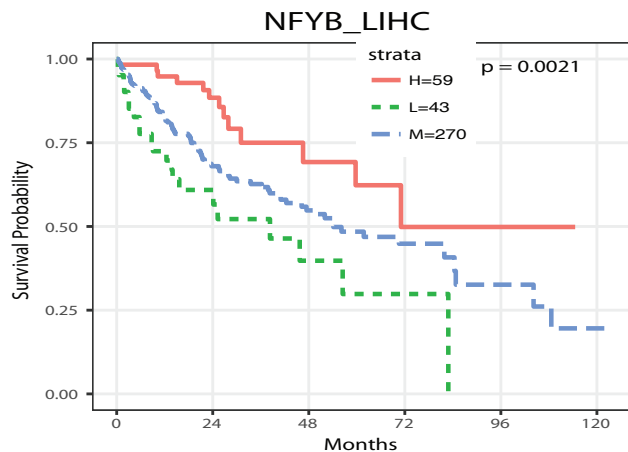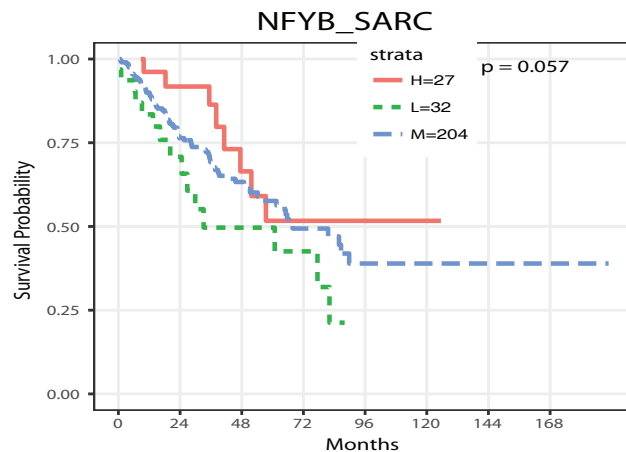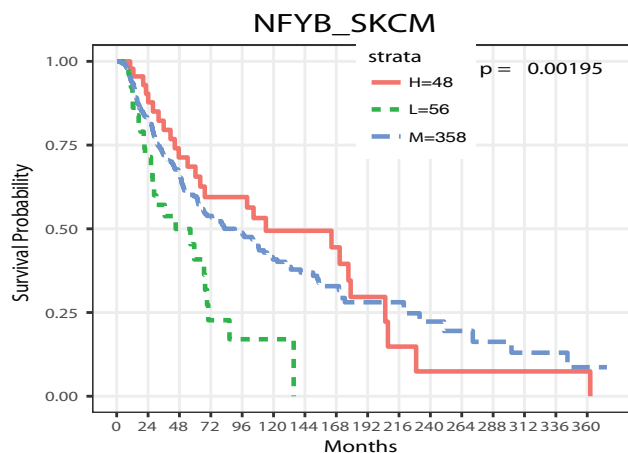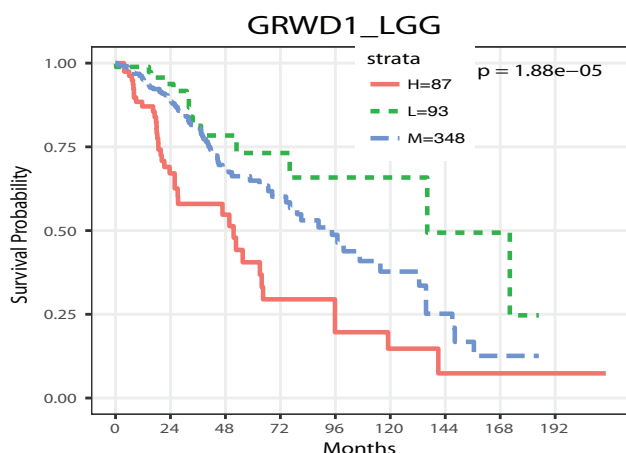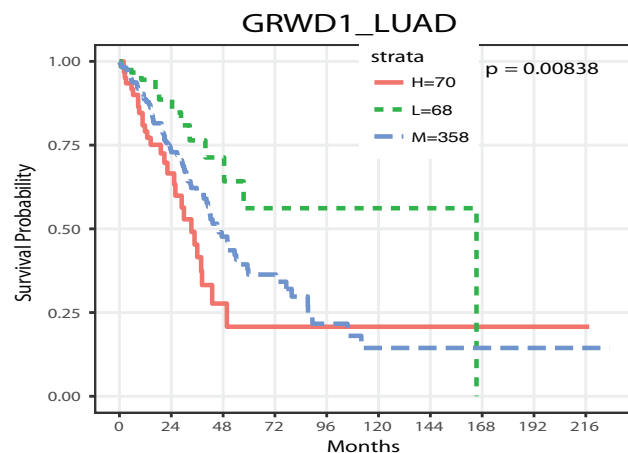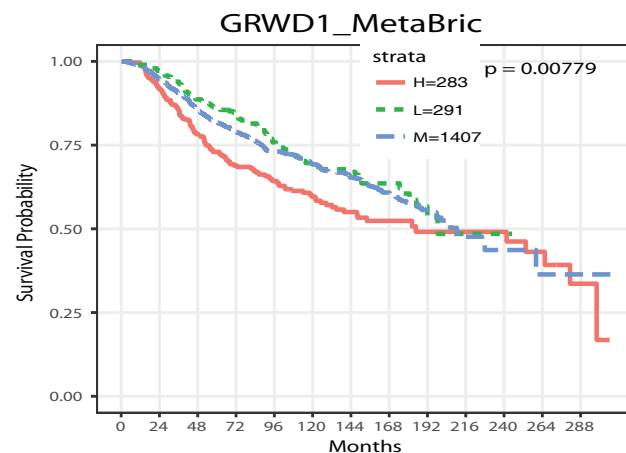

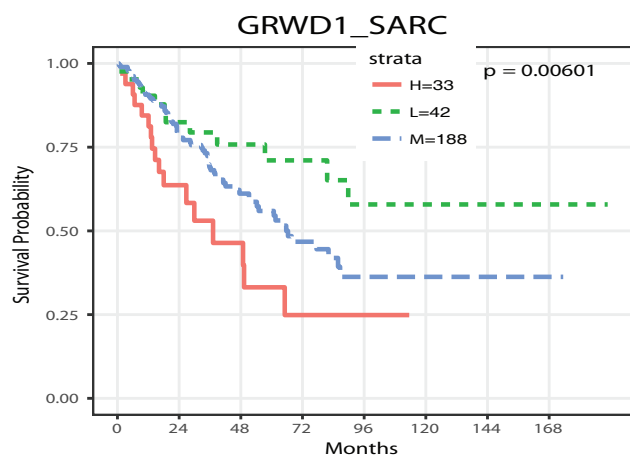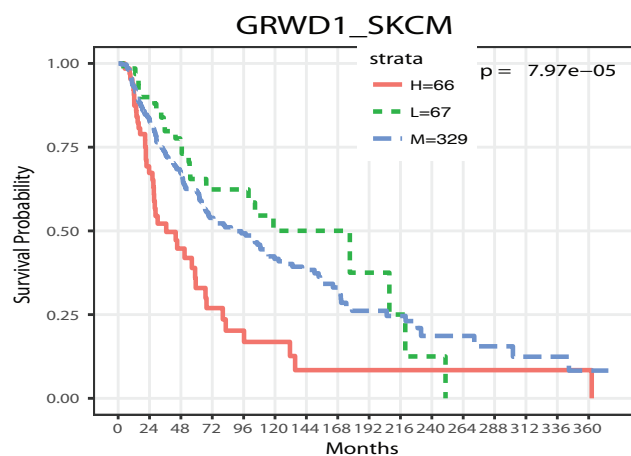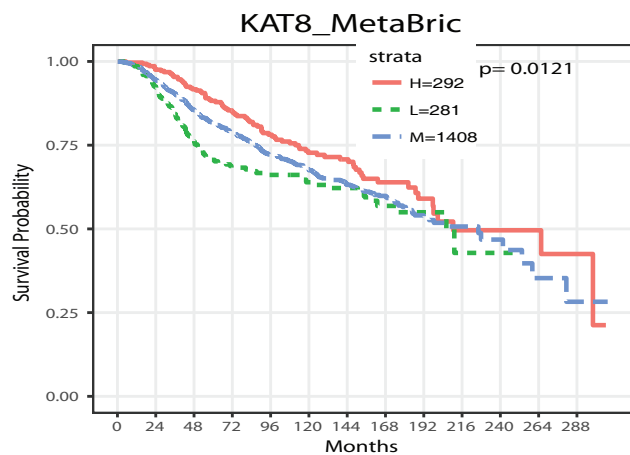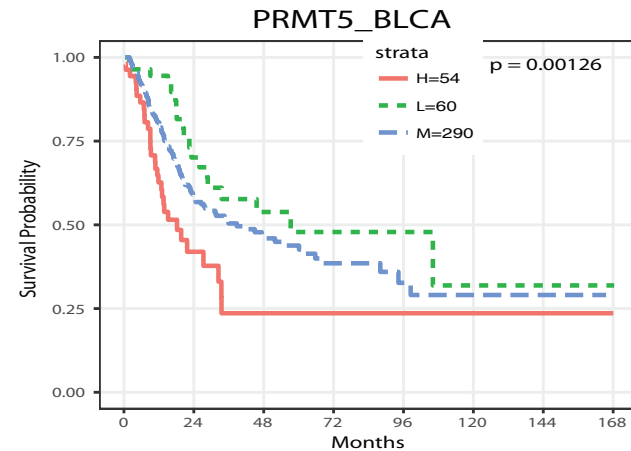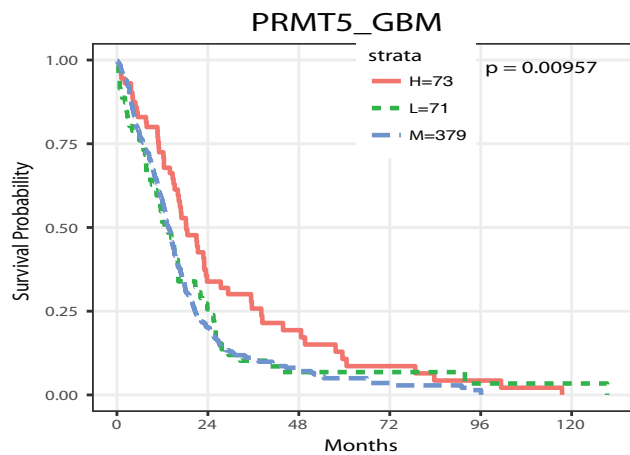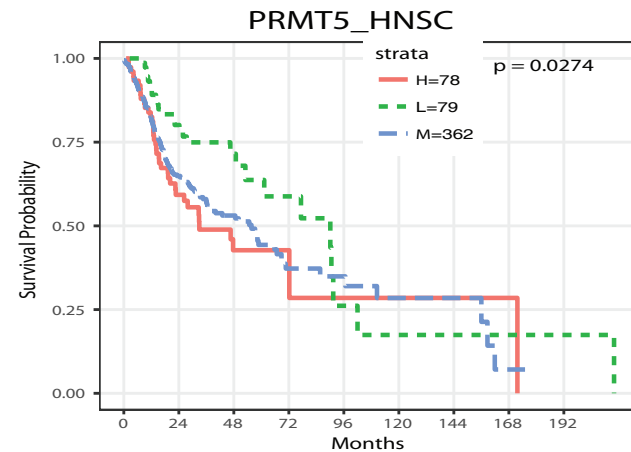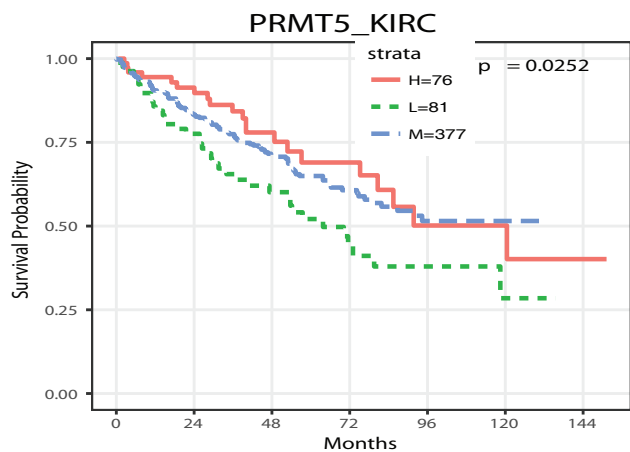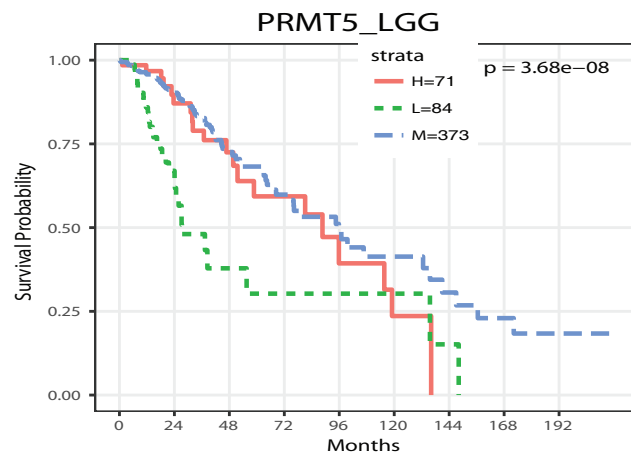

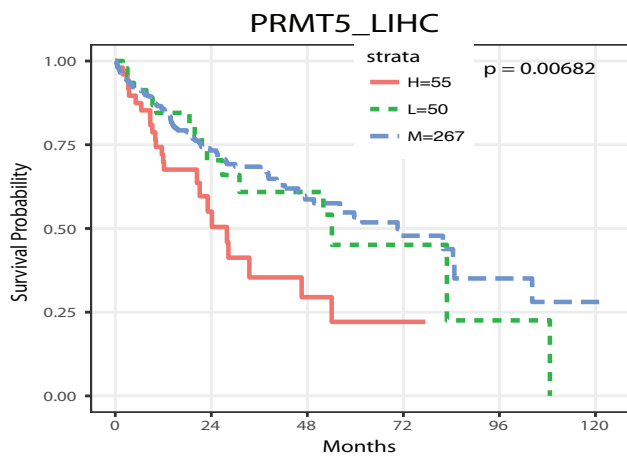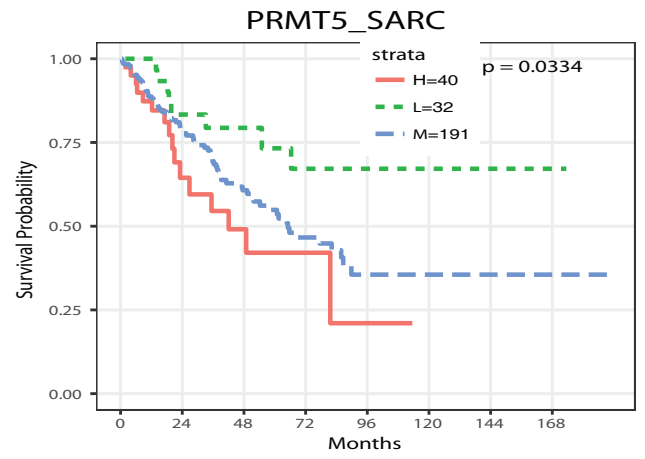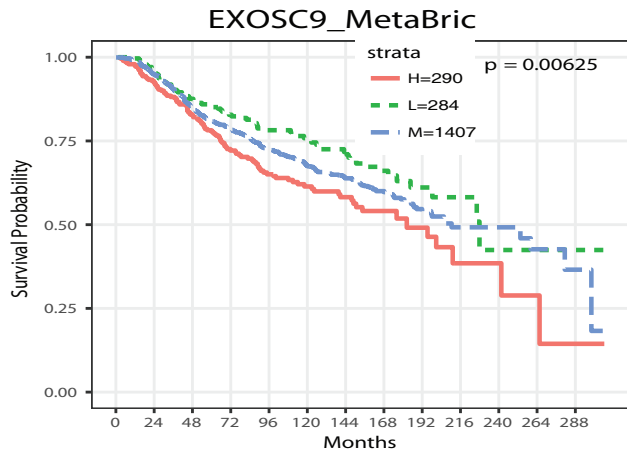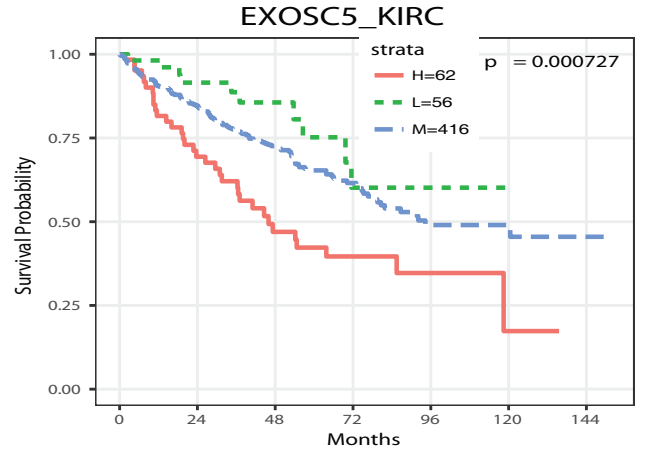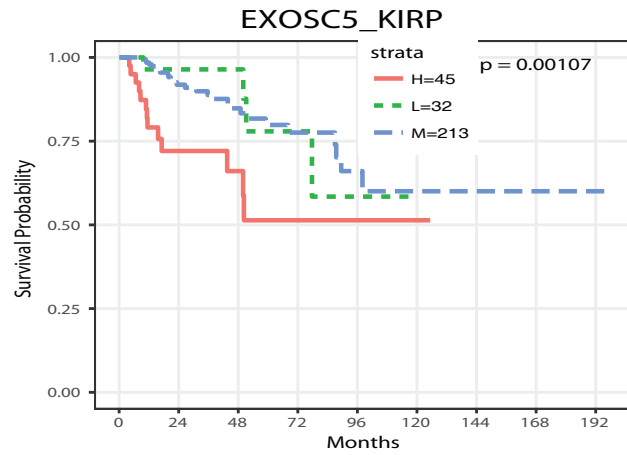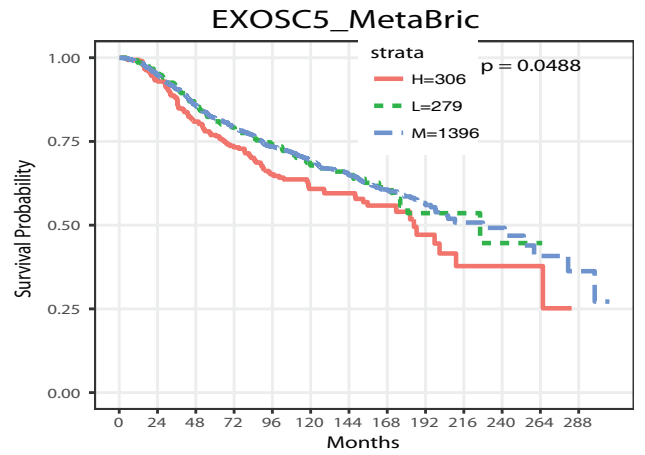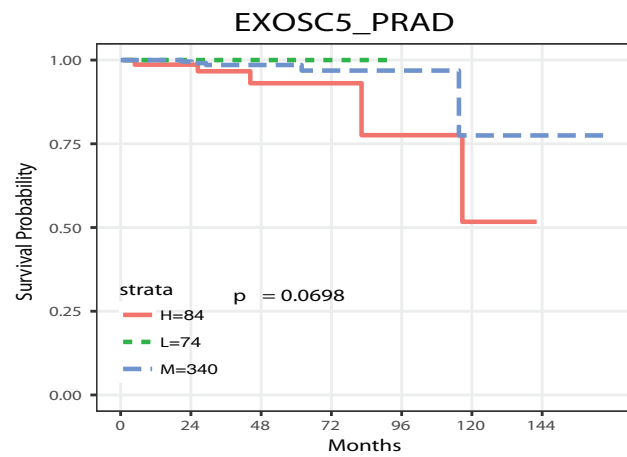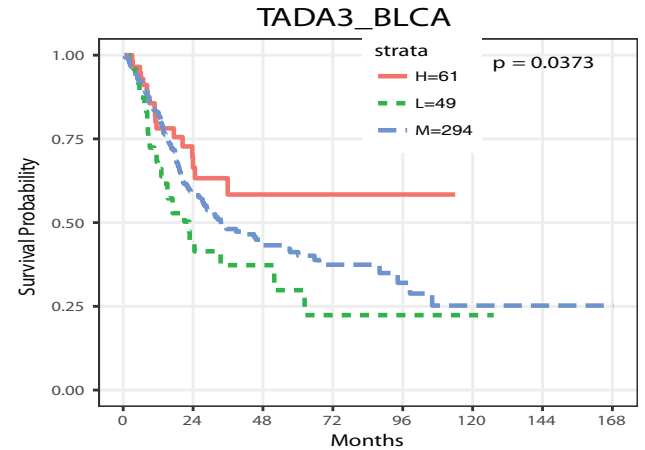

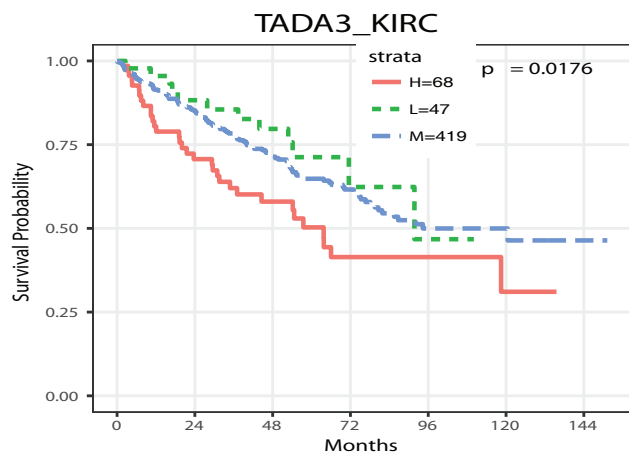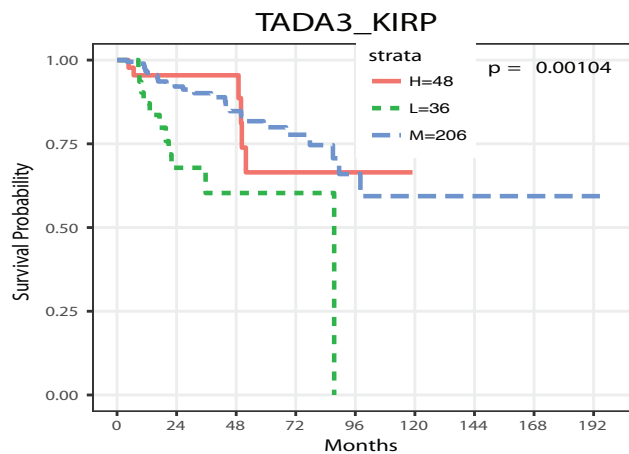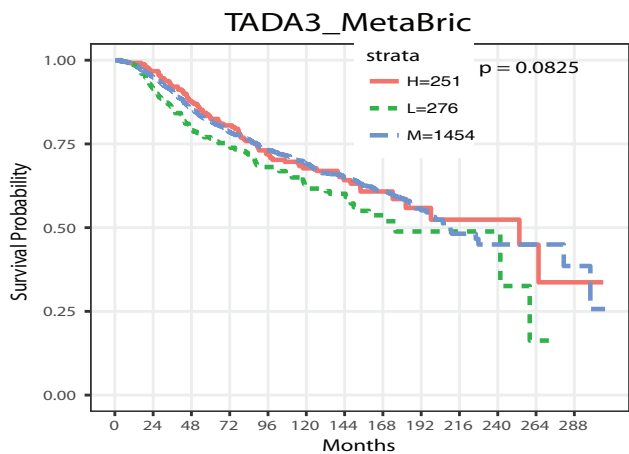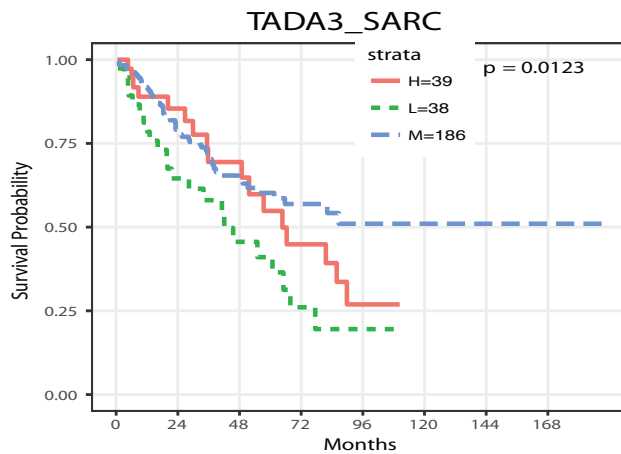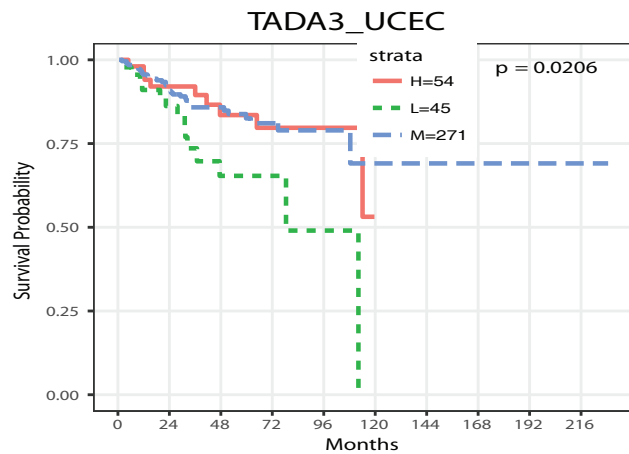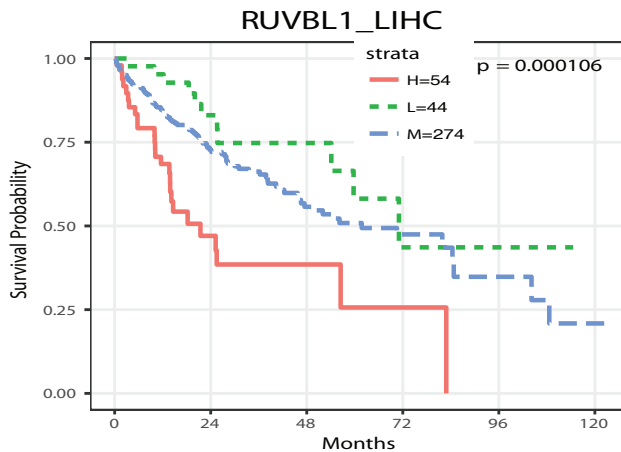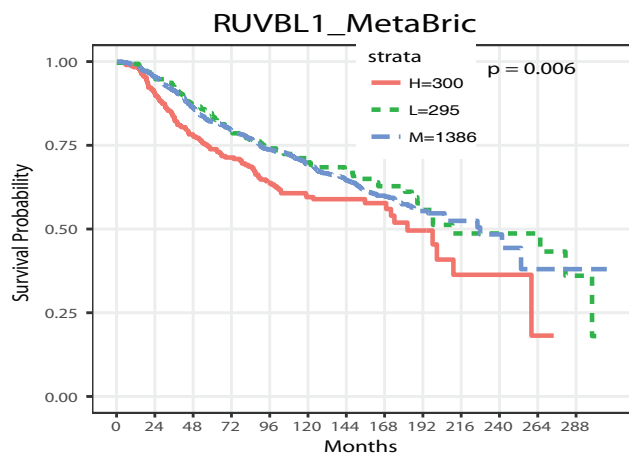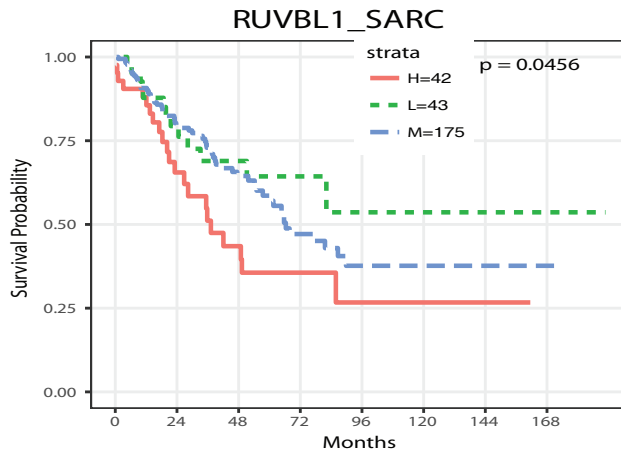

# RUVBL1\_LGG

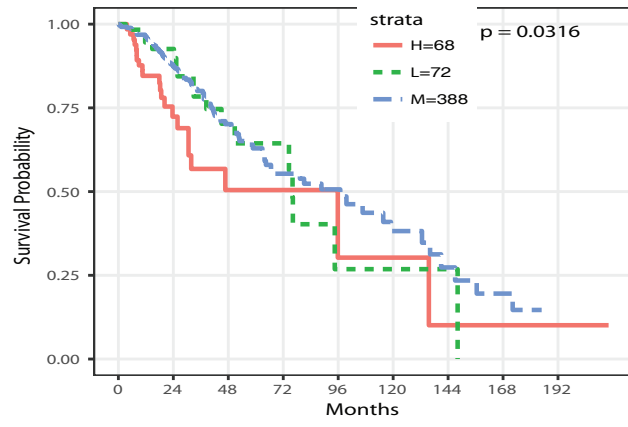

Supplement: FIG S1 [file mbio.03431-21-sf001.pdf]

Supplemental Figure S2

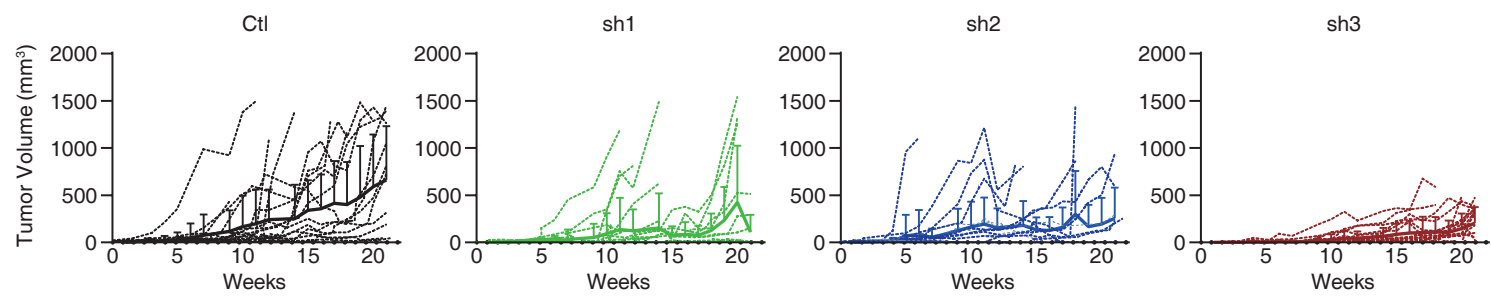

Supplement: FIG S2 [file mbio.03431-21-sf002.pdf]

Supplemental Figure S3

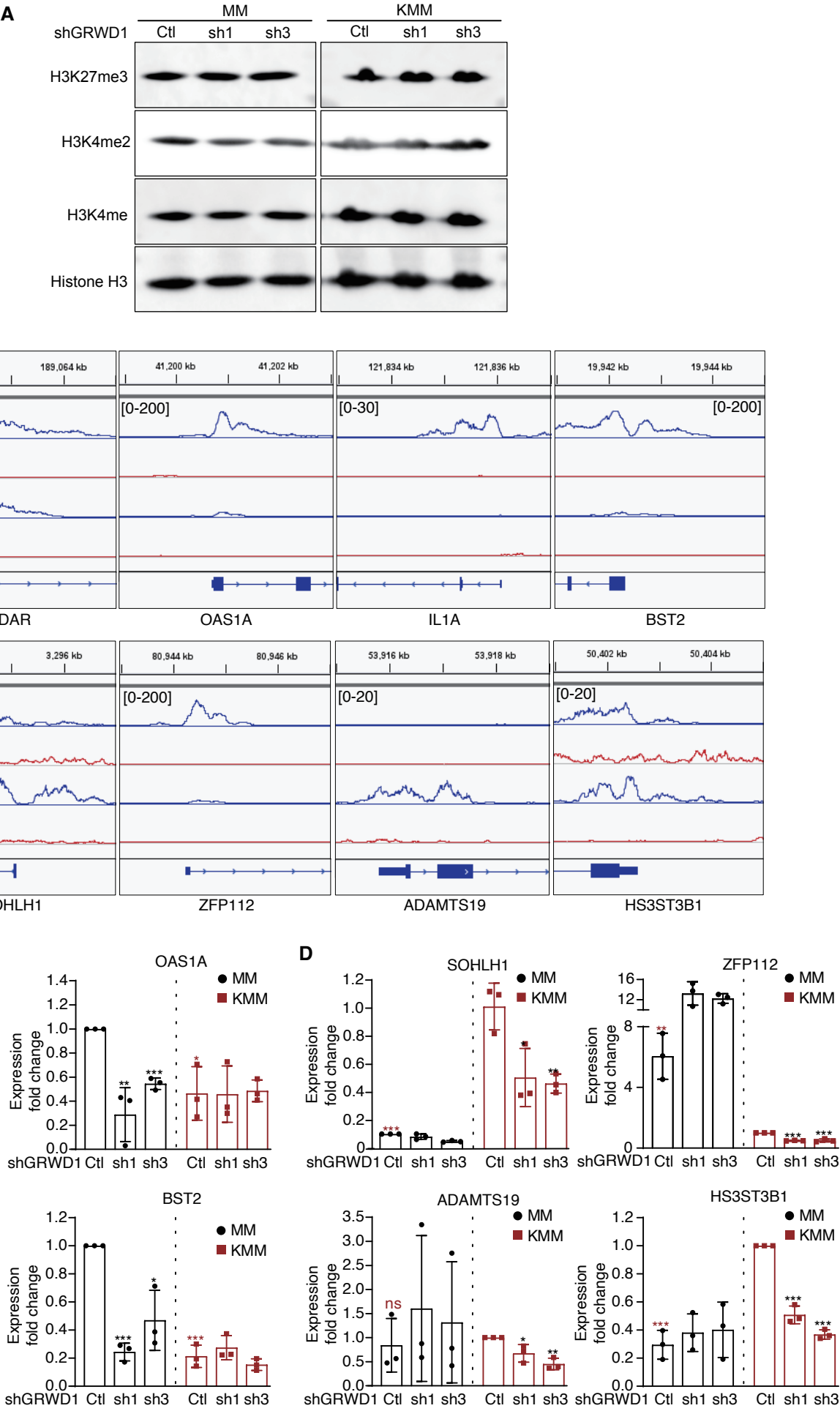

Supplement: FIG S3 [file mbio.03431-21-sf003.pdf]

Supplemental Figure S4

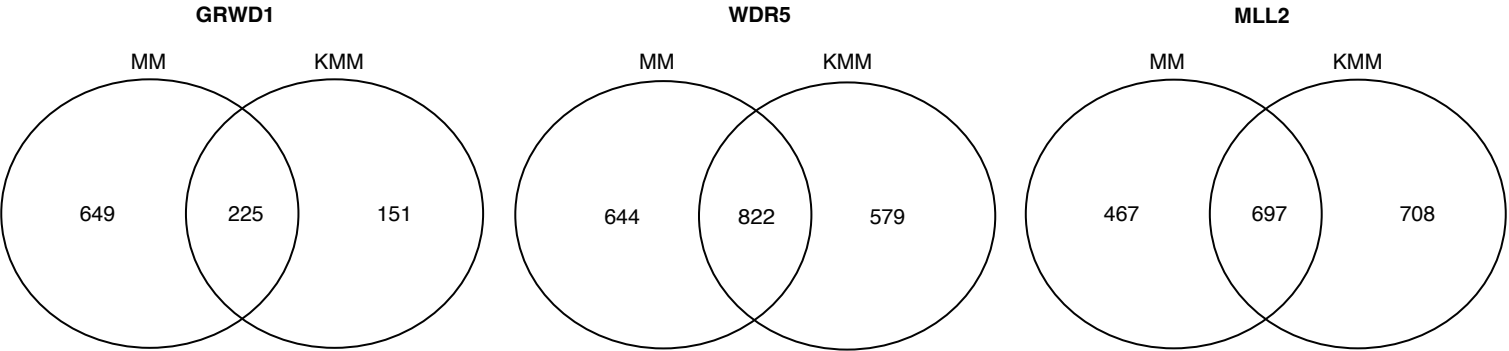

Supplement: FIG S4 [file mbio.03431-21-sf004.pdf]

Supplemental Figure S5

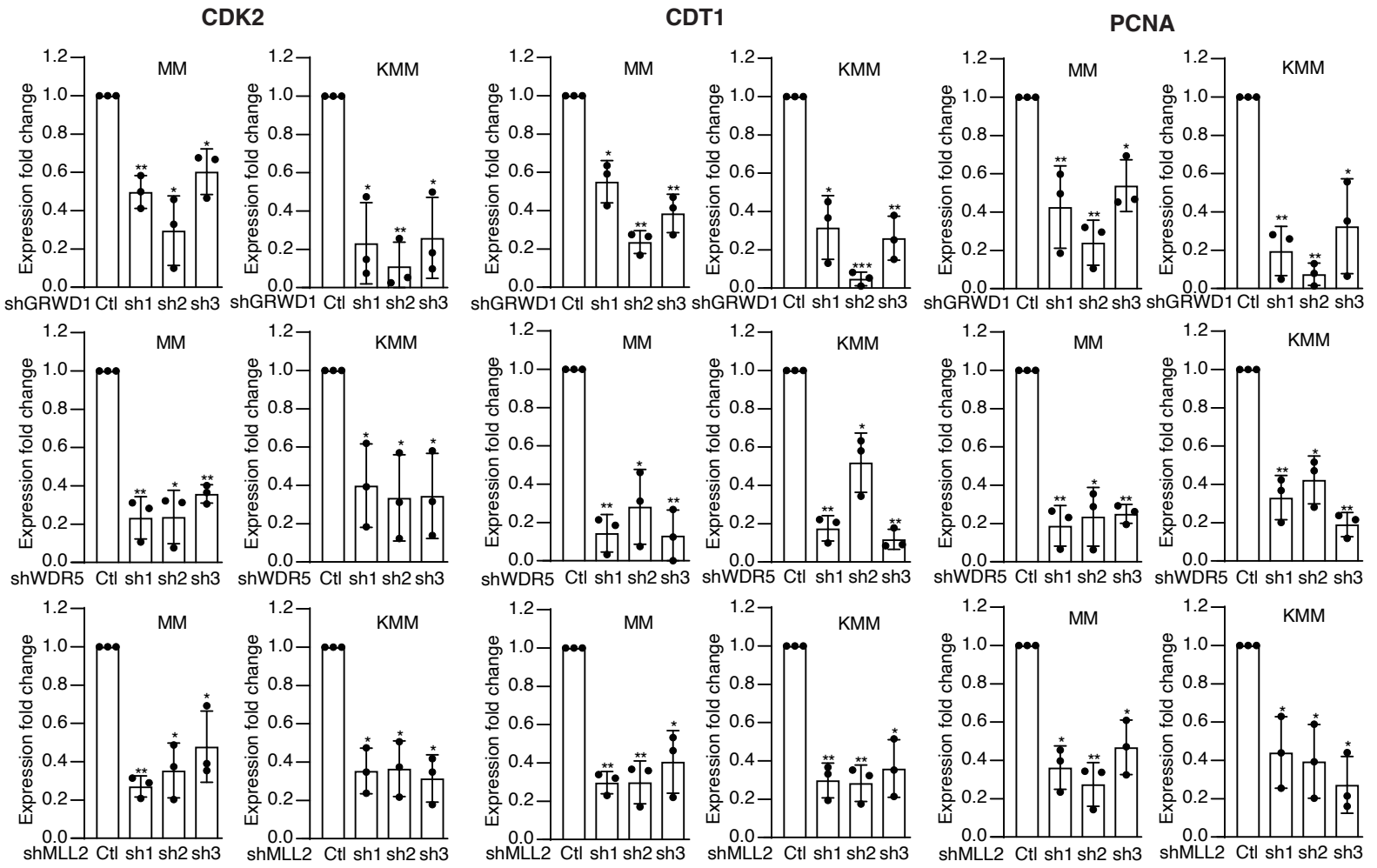

Supplement: FIG S5 [file mbio.03431-21-sf005.pdf]
