# Supplementary material for: GRWD1-WDR5-MLL2 Epigenetic Complex Mediates H3K4me3 Mark and Is Essential for Kaposi’s Sarcoma-Associated Herpesvirus-Induced Cellular Transformation
Source: mBio. 2021 Dec 21;12(6):e03431-21. doi: 10.1128/mbio.03431-21 (PMC8689518; doi:10.1128/mbio.03431-21)
Supplement: TABLE S1 [file mbio.03431-21-st001.pdf]

TABLE S1 Summary of survival analysis of top 9 epigenetic genes

Gene	Disease Type	ID	P-Value
CXXC1	Bladder Urothelial Carcinoma	BLCA	0.033
	Kidney Renal Clear Cell Carcinoma	KIRC	0.021
	Kidney Renal Papillary Cell Carcinoma	KIRP	0.004
	Liver Hepatocellular Carcinoma	LIHC	0.091
	Breast Cancer	MetaBric	0.010
	Sarcoma	SARC	0.001
	Uterine Corpus Endometrial Carcinoma	UCEC	0.039
EXOSC5	Glioblastoma Multiforme	GBM	0.094
	Kidney Renal Clear Cell Carcinoma	KIRC	0.001
	Kidney Renal Papillary Cell Carcinoma	KIRP	0.001
	Lung Squamous Cell Carcinoma	LUSC	0.043
	Breast Cancer	MetaBric	0.049
	Prostate Adenocarcinoma	PRAD	0.070
EXOSC9	Breast Cancer	MetaBric	0.006
GRWD1	Glioblastoma Multiforme	GBM	0.086
	Brain Lower Grade Glioma	LGG	0.000
	Lung Adenocarcinoma	LUAD	0.008
	Breast Cancer	MetaBric	0.008
	Sarcoma	SARC	0.006
	Skin Cutaneous Melanoma	SKCM	0.000
KAT8	Breast Cancer	MetaBric	0.012
NFYB	Bladder Urothelial Carcinoma	BLCA	0.033
	Cervical Squamous Cell Carcinoma and Endocervical Adenocarcinom	CESC	0.038
	Kidney Renal Clear Cell Carcinoma	KIRC	0.000
	Kidney Renal Papillary Cell Carcinoma	KIRP	0.022
	Brain Lower Grade Glioma	LGG	0.001
	Liver Hepatocellular Carcinoma	LIHC	0.002
	Sarcoma	SARC	0.057
	Skin Cutaneous Melanoma	SKCM	0.002
PRMT5	Bladder Urothelial Carcinoma	BLCA	0.001
	Glioblastoma Multiforme	GBM	0.010
	Head-Neck Squamous Cell Carcinoma	HNSC	0.027
	Kidney Renal Clear Cell Carcinoma	KIRC	0.025
	Brain Lower Grade Glioma	LGG	0.000
	Liver Hepatocellular Carcinoma	LIHC	0.007
	Breast Cancer	MetaBric	0.000
	Ovarian Serous Cystadenocarcinoma	OV	0.067
	Sarcoma	SARC	0.033
RUVBL1	Kidney Renal Papillary Cell Carcinoma	KIRP	0.039
	Brain Lower Grade Glioma	LGG	0.032
	Liver Hepatocellular Carcinoma	LIHC	0.000
	Breast Cancer	MetaBric	0.006
	Sarcoma	SARC	0.046
TADA3	Bladder Urothelial Carcinoma	BLCA	0.037
	Kidney Renal Clear Cell Carcinoma	KIRC	0.018
	Kidney Renal Papillary Cell Carcinoma	KIRP	0.001
	Breast Cancer	MetaBric	0.083
	Sarcoma	SARC	0.012
	Uterine Corpus Endometrial Carcinoma	UCEC	0.021
