# Supplementary material for: GRWD1-WDR5-MLL2 Epigenetic Complex Mediates H3K4me3 Mark and Is Essential for Kaposi’s Sarcoma-Associated Herpesvirus-Induced Cellular Transformation
Source: mBio. 2021 Dec 21;12(6):e03431-21. doi: 10.1128/mbio.03431-21 (PMC8689518; doi:10.1128/mbio.03431-21)
Supplement: TABLE S2 [file mbio.03431-21-st002.pdf]

TABLE S2A Altered H3K4me3 peaks and associated genes after GRWD1 knockdown in MM cells

Symbol	GeneID	Chromosome	Start	End	Distance To TSS	Fold	P-Value	FDR	Annotation
RGD1560028	500157	chr4	96451582	96451882	198948	-3.82	3.13E-13	1.72E-08	Distal Intergenic
Ly6e	362934	chr7	116356271	116356571	102	-3.59	8.95E-12	2.47E-07	Promoter (<=1kb)
Oasl2	304549	chr12	47482534	47482834	113	-2.35	1.39E-10	1.64E-06	Promoter (<=1kb)
Exd2	362759	chr6	103994137	103994437	-23452	-3.71	1.45E-10	1.64E-06	Distal Intergenic
Ccl7	287561	chr10	69423492	69423792	409	-2.71	1.68E-10	1.64E-06	Promoter (<=1kb)
LOC500846	500846	chr7	69537094	69537394	-432144	-3.64	1.78E-10	1.64E-06	Distal Intergenic
Plxnd1	312652	chr4	147888302	147888602	5390	-3.54	4.02E-10	3.17E-06	Intron (NM_001107881/312652, intron 1 of 36)
Ccl2	24770	chr10	69412804	69413104	739	-3.12	6.32E-10	4.35E-06	Promoter (<=1kb)
Mx2	286918	chr11	38035930	38036230	417	-3.22	7.09E-10	4.35E-06	Promoter (<=1kb)
Prok2	192206	chr4	132436400	132436700	-265247	-3.23	9.77E-10	5.39E-06	Distal Intergenic
Ccl20	29538	chr9	88918836	88919136	410	-3.89	2.22E-09	1.02E-05	Promoter (<=1kb)
Mir3565	100526634	chr14	66820668	66820968	-102115	-3.59	2.22E-09	1.02E-05	Distal Intergenic
Hopx	171160	chr14	33375044	33375344	20237	-3.06	2.48E-09	1.05E-05	Distal Intergenic
Rsad2	65190	chr6	45668452	45668752	331	-2.83	3.37E-09	1.33E-05	Promoter (<=1kb)
Selenop	29360	chr2	52827849	52828149	-277763	-3	4.61E-09	1.69E-05	Distal Intergenic
Pla2g2a	29692	chr5	157284217	157284517	1499	-4.04	5.21E-09	1.80E-05	Promoter (1-2kb)
Chchd6	297436	chr4	121380015	121380315	184897	-3.39	6.03E-09	1.95E-05	Intron (NM_001106608/297436, intron 5 of 7)
Hepacam2	296846	chr4	28323968	28324268	113396	-1.96	8.70E-09	2.66E-05	Distal Intergenic
Rtp4	360733	chr11	80643483	80643783	7019	-2.38	1.04E-08	3.01E-05	Exon (NM_001108321/360733, exon 3 of 4)
Tead1	361630	chr1	177384615	177384915	-110867	-4.1	1.31E-08	3.62E-05	Intron (NM_020656/57341, intron 5 of 12)
Cbr4	359725	chr16	31313678	31313978	658529	-3.09	2.62E-08	6.88E-05	Distal Intergenic
Ext1	299907	chr7	92762164	92762464	118928	-3.23	3.55E-08	8.90E-05	Intron (NM_001130540/299907, intron 1 of 10)
Map3k8	116596	chr17	56180812	56181112	71409	-3.77	6.96E-08	1.67E-04	Distal Intergenic
Mir222	100314059	chrX	3633574	3633874	-50045	-3.13	1.12E-07	2.57E-04	Distal Intergenic
Serpine2	29366	chr9	85796577	85796877	-170483	-2.55	1.24E-07	2.61E-04	Distal Intergenic
C1rl	408246	chr4	157103740	157104040	-4150	-3.26	1.26E-07	2.61E-04	Distal Intergenic
Cx3cl1	89808	chr19	10659387	10659687	-5626	-2.56	1.28E-07	2.61E-04	Distal Intergenic
LOC500846	500846	chr7	69538206	69538506	-433256	-2.85	1.60E-07	3.15E-04	Distal Intergenic
Mrpl35	297334	chr4	99744160	99744460	2100	-3.17	2.13E-07	3.92E-04	Promoter (2-3kb)
Por	29441	chr12	24034185	24034485	12329	-3.34	2.13E-07	3.92E-04	Intron (NM_031576/29441, intron 1 of 15)
Plcd4	140693	chr9	81831299	81831599	14427	-2.6	2.49E-07	4.43E-04	Exon (NM_080688/140693, exon 7 of 17)
Psma8	364814	chr18	6169123	6169423	54366	-3.45	2.66E-07	4.44E-04	Intron (NM_001108884/364814, intron 4 of 6)
Socs3	89829	chr10	107042651	107042951	-66611	-3.94	2.66E-07	4.44E-04	Distal Intergenic
Smad7	81516	chr18	71487891	71488191	92061	-3	3.44E-07	5.42E-04	Intron (NM_001108891/364900, intron 3 of 4)
Txndc5	100362805	chr17	27017590	27017890	91762	-3.34	3.44E-07	5.42E-04	Intron (NM_013107/25644, intron 1 of 6)
Nos2	24599	chr10	66190317	66190617	2027	-3.39	3.62E-07	5.54E-04	Promoter (2-3kb)
Cx3cl1	89808	chr19	10653488	10653788	0	-2.8	4.20E-07	6.25E-04	Promoter (<=1kb)
Pik3r1	25513	chr2	31811324	31811624	4761	-2.98	4.66E-07	6.77E-04	Intron (NM_013005/25513, intron 1 of 14)
Sinhcaf	686611	chr4	183103079	183103379	314288	-3.3	5.17E-07	7.15E-04	Distal Intergenic
Tmsb10	50665	chr4	100862116	100862416	20859	-2.99	5.19E-07	7.15E-04	Distal Intergenic
Prkar1b	25521	chr12	17669252	17669552	54687	-2.77	6.21E-07	8.35E-04	Intron (NM_001033679/25521, intron 6 of 10)
Dcaf5	314273	chr6	103793820	103794120	-188564	-2.22	6.51E-07	8.54E-04	Distal Intergenic
Uspl1	288447	chr12	6979766	6980066	-22852	-3.19	7.32E-07	9.39E-04	Distal Intergenic
Ppp4r1	140943	chr9	113522042	113522342	6751	-2.91	7.80E-07	9.77E-04	Intron (NM_080907/140943, intron 3 of 20)
C2cd2	304055	chr11	38375339	38375639	44393	3.42	8.19E-07	9.91E-04	Intron (NM_199391/304055, intron 11 of 13)
Usp18	312688	chr4	153812489	153812789	6409	-2.94	8.27E-07	9.91E-04	Exon (NM_001014058/312688, exon 2 of 11)
Sag	25539	chr9	95033138	95033438	106072	-2.02	8.84E-07	1.02E-03	Distal Intergenic
Uqcc2	361805	chr20	5732164	5732464	-8262	-2.75	8.89E-07	1.02E-03	Distal Intergenic
Cish	83681	chr8	116061401	116061701	6854	-3.09	9.27E-07	1.04E-03	Distal Intergenic
Llgl1	54265	chr10	46933883	46934183	-6826	3.81	1.13E-06	1.24E-03	Distal Intergenic
Kcna6	64358	chr4	159277027	159277327	9866	-2.94	1.18E-06	1.28E-03	Intron (NM_023954/64358, intron 1 of 1)
Slc41a2	362861	chr7	26685549	26685849	163235	-2.54	1.22E-06	1.29E-03	Intron (NM_001108079/314694, intron 2 of 2)
Mir3084a	104795674	chr19	43441976	43442276	38873	-1.82	1.27E-06	1.30E-03	Distal Intergenic
Fhod3	100360334	chr18	16650767	16651067	0	2.03	1.30E-06	1.30E-03	Promoter (<=1kb)
Fam136a	297415	chr4	118127396	118127696	-32451	2.94	1.30E-06	1.30E-03	Distal Intergenic
Rasgrp2	361714	chr1	221772303	221772603	-651	3.43	1.33E-06	1.31E-03	Promoter (<=1kb)
Hmga2	84017	chr7	65412996	65413296	-137588	-2.39	1.46E-06	1.41E-03	Distal Intergenic
Gns	299825	chr7	65462926	65463226	-153172	-2.91	1.50E-06	1.42E-03	Distal Intergenic
Ttc1	287208	chr10	29336357	29336657	31208	-2.7	1.61E-06	1.51E-03	Distal Intergenic
Mgp	25333	chr4	170859309	170859609	496	-2.03	1.73E-06	1.59E-03	Promoter (<=1kb)
Hoxc4	24459	chr7	144704038	144704338	56451	-3.17	1.78E-06	1.61E-03	Distal Intergenic
Mirlet7i	100313993	chr7	66896727	66897027	93996	-2.29	1.82E-06	1.62E-03	Intron (NM_001271079/314897, intron 6 of 9)
Ripk4	304053	chr11	38246617	38246917	27317	-2.73	1.90E-06	1.66E-03	Distal Intergenic
Trim8	688785	chr1	266236197	266236497	-19300	-2.6	2.05E-06	1.77E-03	Intron (NM_001024899/361769, intron 10 of 11)
Rgr	306307	chr16	14169617	14169917	131034	-2.63	2.16E-06	1.81E-03	Distal Intergenic
Bdkrb2	25245	chr6	129323139	129323439	-76029	-2.95	2.16E-06	1.81E-03	Distal Intergenic
Bid	64625	chr4	153477183	153477483	-11936	-2.27	2.42E-06	1.99E-03	Distal Intergenic
Lcn2	170496	chr3	11416808	11417108	426	-3.15	2.70E-06	2.19E-03	Promoter (<=1kb)
Stx8	59074	chr10	54575348	54575648	62363	-3	2.79E-06	2.21E-03	Intron (NM_031656/59074, intron 6 of 7)
Traf3	362788	chr6	135604503	135604803	-5940	-2.6	2.81E-06	2.21E-03	Distal Intergenic
Rnf43	303412	chr10	74959018	74959318	0	-2.86	3.01E-06	2.34E-03	Promoter (<=1kb)
Gnaq	81666	chr1	233576049	233576349	193271	-2.52	3.24E-06	2.47E-03	Intron (NM_031036/81666, intron 3 of 6)
Fam3c	312159	chr4	49573618	49573918	-133726	-3.01	3.27E-06	2.47E-03	Distal Intergenic
Pabpc1	171350	chr7	75383577	75383877	37997	-3.03	3.42E-06	2.55E-03	Distal Intergenic
Ahsp	293522	chr1	199736751	199737051	16713	-2.68	3.48E-06	2.55E-03	Distal Intergenic
Gbp4	310917	chr2	248215553	248215853	-33617	-2.64	3.51E-06	2.55E-03	Distal Intergenic
Rbm34	307956	chr19	59891101	59891401	3536	-2.75	3.75E-06	2.66E-03	Intron (NM_001014015/307956, intron 3 of 10)
Fgd6	500824	chr7	34990519	34990819	38641	-2.69	3.77E-06	2.66E-03	Intron (NM_001137645/500824, intron 2 of 20)
Csf1	78965	chr2	210535031	210535331	15215	-2.52	3.93E-06	2.74E-03	Exon (NM_023981/78965, exon 4 of 9)
LOC310926	310926	chr1	11910966	11911266	3274	4.1	4.13E-06	2.85E-03	Intron (NM_001025002/310926, intron 6 of 6)
Arhgap45	314618	chr7	12733624	12733924	7325	-2.8	4.36E-06	2.94E-03	Exon (NM_001108067/314618, exon 11 of 23)
Scap	301024	chr8	118551750	118552050	-18453	2.18	4.37E-06	2.94E-03	Distal Intergenic
Wfdc21	360228	chr10	71047432	71047732	106	-2.61	4.92E-06	3.26E-03	Promoter (<=1kb)
Abhd2	293050	chr1	141022040	141022340	23800	-1.96	4.96E-06	3.26E-03	Intron (NM_001106275/293050, intron 1 of 10)
Uspl1	288447	chr12	6978580	6978880	-21666	-2.87	5.35E-06	3.44E-03	Distal Intergenic
Ccnd1	58919	chr1	218092425	218092725	7547	-2.57	5.41E-06	3.44E-03	3' UTR
Tlr4	29260	chr5	82587530	82587830	106	3	5.44E-06	3.44E-03	Promoter (<=1kb)
Scube3	294297	chr20	7718392	7718692	110	-2.82	5.52E-06	3.44E-03	Promoter (<=1kb)
Il1a	24493	chr3	121835671	121835971	151	-3.18	5.56E-06	3.44E-03	Promoter (<=1kb)
Lgalsl	360983	chr14	105141811	105142111	-86390	-2.77	5.67E-06	3.47E-03	Distal Intergenic
Cflar	117279	chr9	65549700	65550000	15072	-2.39	6.03E-06	3.65E-03	Intron (NM_057138/117279, intron 2 of 5)
Pfdn1	361310	chr18	29293755	29294055	-3308	-2.93	6.31E-06	3.78E-03	Distal Intergenic

Gprc5a	312790	chr4	168842310	168842610	9373	-2.84	6.45E-06	3.79E-03	Intron (NM_001079890/312790, intron 1 of 3)
Gfod1	306842	chr17	23941851	23942151	-18162	-2.7	6.47E-06	3.79E-03	Distal Intergenic
Nipal2	362899	chr7	73449511	73449811	451	1.34	6.60E-06	3.83E-03	Promoter (<=1kb)
RGD1560028	500157	chr4	96449378	96449678	196744	-2.72	7.39E-06	4.20E-03	Distal Intergenic
Neurl3	316326	chr9	42838919	42839219	618	-2.78	7.43E-06	4.20E-03	Promoter (<=1kb)
Rsad2	65190	chr6	45671560	45671860	-2477	-2.53	7.46E-06	4.20E-03	Promoter (2-3kb)
Pdlim2	290354	chr15	51852341	51852641	2684	2.41	7.81E-06	4.29E-03	Promoter (2-3kb)
Fendrr	104845258	chr19	52910679	52910979	99530	1.97	7.84E-06	4.29E-03	Distal Intergenic
Ripk4	304053	chr11	38237186	38237486	36748	-2.53	7.86E-06	4.29E-03	Distal Intergenic
Jun	24516	chr5	114064868	114065168	-50591	-2.96	8.00E-06	4.31E-03	Distal Intergenic
Psmd7	307821	chr19	40266831	40267131	-620113	-2.83	8.07E-06	4.31E-03	Distal Intergenic
Pdgfa	25266	chr12	17744218	17744518	10077	-2.99	8.14E-06	4.31E-03	Intron (NM_012801/25266, intron 5 of 7)
Pou2f2	117058	chr1	81992871	81993171	10520	-3.1	8.20E-06	4.31E-03	Exon (NM_001271204/117058, exon 2 of 14)
C3	24232	chr9	9722494	9722794	1357	-3.27	8.50E-06	4.42E-03	Promoter (1-2kb)
Dao	114027	chr12	48342570	48342870	30777	-2.52	9.25E-06	4.77E-03	Exon (NM_134404/171442, exon 12 of 16)
Mink1	303259	chr10	57175891	57176191	-9156	-2.81	1.03E-05	5.26E-03	Exon (NM_033299/25097, exon 32 of 40)
Ngf	310738	chr2	204840680	204840980	-45222	-3.03	1.07E-05	5.38E-03	Distal Intergenic
Elmo1	361251	chr17	46794828	46795128	93660	1.94	1.07E-05	5.38E-03	5' UTR
Tsga10	252923	chr9	44301572	44301872	154695	-2.93	1.14E-05	5.67E-03	Distal Intergenic
Crisp2	360445	chr9	23528203	23528503	24967	-2.97	1.22E-05	5.98E-03	Distal Intergenic
Dusp7	300980	chr8	115090319	115090619	21224	-2.63	1.26E-05	6.17E-03	Distal Intergenic
Crlf1	290655	chr16	20685952	20686252	65	2.02	1.38E-05	6.66E-03	Promoter (<=1kb)
Gbp5	362050	chr2	248178867	248179167	426	-2.67	1.40E-05	6.71E-03	Promoter (<=1kb)
Egln3	54702	chr6	75236068	75236368	-160273	-2.69	1.43E-05	6.80E-03	Distal Intergenic
Irak4	300177	chr7	135536104	135536404	-267319	2.63	1.47E-05	6.91E-03	Distal Intergenic
Met	24553	chr4	44854276	44854576	78943	3.05	1.48E-05	6.91E-03	Distal Intergenic
Bzw2	171439	chr6	55635014	55635314	12336	-2.49	1.52E-05	7.04E-03	Intron (NM_134402/171439, intron 1 of 11)
Hipk2	362342	chr4	66595559	66595859	29053	-2.57	1.55E-05	7.10E-03	Intron (NM_001108622/362342, intron 1 of 14)
Atp2a2	29693	chr12	39572527	39572827	18624	-2.77	1.57E-05	7.10E-03	Intron (NM_001110823/29693, intron 5 of 19)
Rtp3	316018	chr8	119264296	119264596	561	-2.6	1.58E-05	7.10E-03	Promoter (<=1kb)
Sos1	313845	chr6	3248812	3249112	-65835	-2.86	1.58E-05	7.10E-03	Distal Intergenic
Avpr1a	25107	chr7	67344737	67345037	253	1.1	1.61E-05	7.17E-03	Promoter (<=1kb)
Pde2a	81743	chr1	166494843	166495143	-39461	-2.78	1.63E-05	7.17E-03	Distal Intergenic
Fgf5	60662	chr14	13202996	13203296	-207412	-2.6	1.64E-05	7.17E-03	Distal Intergenic
Fst	24373	chr2	46744080	46744380	-199628	-2.32	1.71E-05	7.37E-03	Distal Intergenic
Ifit3	309526	chr1	252906980	252907280	746	-2.44	1.71E-05	7.37E-03	Promoter (<=1kb)
Sgsm3	362963	chr7	122190829	122191129	-12403	3.37	1.92E-05	8.21E-03	Intron (NM_001130503/315150, intron 12 of 12)
Plekhb2	301337	chr9	41361317	41361617	12870	-2.65	1.95E-05	8.21E-03	Intron (NM_001106899/301337, intron 1 of 7)
Pon1	84024	chr4	30193585	30193885	82412	-2.16	1.95E-05	8.21E-03	Intron (NM_053473/84685, intron 6 of 14)
Cd180	294706	chr2	33214379	33214679	394057	-3.07	2.00E-05	8.32E-03	Distal Intergenic
Oasl	304545	chr12	47455781	47456081	79	-1.37	2.01E-05	8.32E-03	Promoter (<=1kb)
Cxcl13	498335	chr14	15221543	15221843	36342	-2.68	2.10E-05	8.64E-03	Distal Intergenic
Scube3	294297	chr20	7717434	7717734	-548	-2.35	2.16E-05	8.84E-03	Promoter (<=1kb)
Bcl2l1	24888	chr3	148293470	148293770	18515	-2.69	2.20E-05	8.92E-03	Intron (NM_001033671/24888, intron 1 of 1)
Dao	114027	chr12	48340381	48340681	32966	-2.66	2.28E-05	8.99E-03	Intron (NM_134404/171442, intron 11 of 15)
Gpd1	60666	chr7	141370638	141370938	98	3.48	2.31E-05	8.99E-03	Promoter (<=1kb)
Nod2	291912	chr19	19377567	19377867	-778	-2.94	2.32E-05	8.99E-03	Promoter (<=1kb)
Abcc9	25560	chr4	176928454	176928754	0	1.59	2.32E-05	8.99E-03	Promoter (<=1kb)
Cyth4	500906	chr7	119847055	119847355	26515	-2.12	2.33E-05	8.99E-03	Distal Intergenic
Hnrnpm	116655	chr7	18549670	18549970	4278	-2.53	2.33E-05	8.99E-03	Intron (NM_001109911/116655, intron 1 of 15)
Ccl5	81780	chr10	70742903	70743203	1100	-3.41	2.35E-05	8.99E-03	Promoter (1-2kb)
Slc44a5	365962	chr2	260300746	260301046	-143181	-2.6	2.35E-05	8.99E-03	Intron (NM_019123/29758, intron 2 of 10)
Smug1	315344	chr7	144777131	144777431	1222	-3.05	2.37E-05	9.01E-03	Promoter (1-2kb)
Gpc1	58920	chr9	99942852	99943152	-55123	2.45	2.41E-05	9.09E-03	Intron (NM_001009825/301618, intron 5 of 9)
Pou2f2	117058	chr1	81991663	81991963	11728	-2.73	2.45E-05	9.12E-03	Intron (NM_001271204/117058, intron 4 of 13)
Hepacam2	296846	chr4	28316376	28316676	120988	-2.95	2.47E-05	9.12E-03	Distal Intergenic
N6amt1	288309	chr11	27030860	27031160	26525	2.62	2.48E-05	9.12E-03	Exon (NM_001024235/288308, exon 26 of 30)
MGC116197	367620	chr9	8934726	8935026	208174	-3.17	2.48E-05	9.12E-03	Distal Intergenic
Tnfaip2	299339	chr6	135911169	135911469	20238	-2.04	2.52E-05	9.22E-03	Distal Intergenic
Fgf5	60662	chr14	13202051	13202351	-206467	-2.95	2.59E-05	9.39E-03	Distal Intergenic
Fermt2	289992	chr15	19826664	19826964	49425	-2.49	2.63E-05	9.49E-03	Intron (NM_001011915/289992, intron 4 of 14)
Irf2bp1	308404	chr1	79919697	79919997	8181	1.06	2.69E-05	9.62E-03	Downstream (1-2kb)
Wdfy3	305164	chr14	9141479	9141779	-27630	2.97	2.72E-05	9.68E-03	Distal Intergenic
Zfp217	311764	chr3	166996856	166997156	-2916	-2.53	2.83E-05	1.00E-02	Promoter (2-3kb)
Psip1	313323	chr5	102099227	102099527	-511129	-2.56	2.86E-05	1.01E-02	Distal Intergenic
Hmga2	84017	chr7	65268403	65268703	6705	-2.53	3.10E-05	1.08E-02	Intron (NM_032070/84017, intron 2 of 4)
Manba	310864	chr2	240677600	240677900	9387	-2.42	3.27E-05	1.13E-02	Intron (NM_001031655/310864, intron 1 of 16)
Ntng2	311836	chr3	7759722	7760022	35736	-2.68	3.30E-05	1.13E-02	Intron (NM_001107825/311836, intron 2 of 7)
Slfn4	114247	chr10	70412882	70413182	1144	-2.56	3.31E-05	1.13E-02	Promoter (1-2kb)
Ciita	85483	chr10	5251506	5251806	3	-2.23	3.31E-05	1.13E-02	Promoter (<=1kb)
Hexb	294673	chr2	28027870	28028170	-24610	-2.28	3.34E-05	1.13E-02	Intron (NM_001003401/294674, intron 1 of 3)
Rapgef2	310533	chr2	178023677	178023977	-73160	-2.45	3.36E-05	1.13E-02	Distal Intergenic
Bmp2	29373	chr3	126341810	126342110	5625	-2.67	3.48E-05	1.16E-02	Intron (NM_017178/29373, intron 3 of 3)
Gtf2h2	294693	chr2	30477985	30478285	44427	-2.85	3.49E-05	1.16E-02	Distal Intergenic
Prdx5	113898	chr1	222155273	222155573	11874	-2.48	3.54E-05	1.17E-02	Distal Intergenic
Kcnd1	116695	chrX	15521974	15522274	-1262	2.14	3.59E-05	1.18E-02	Promoter (1-2kb)
Hrh3	85268	chr3	175478938	175479238	157	3.73	3.62E-05	1.18E-02	Promoter (<=1kb)
Ccdc175	500668	chr6	95010363	95010663	-77595	1.7	3.63E-05	1.18E-02	Intron (NM_053865/116644, intron 25 of 30)
Tex43	498870	chr18	51689123	51689423	9355	-2.72	3.65E-05	1.18E-02	Distal Intergenic
Rap1b	171337	chr7	60874334	60874634	-13344	-2.63	3.71E-05	1.19E-02	Distal Intergenic
Rab7a	29448	chr4	119931101	119931401	27772	-2.48	3.80E-05	1.21E-02	Intron (NM_023950/29448, intron 1 of 5)
Asip	24152	chr3	150482616	150482916	-9094	-2.76	3.85E-05	1.22E-02	Distal Intergenic
Dyrk3	304775	chr13	47896069	47896369	20508	-2.31	3.86E-05	1.22E-02	Distal Intergenic
Gcgr	24953	chr10	109696979	109697279	-10683	-2.21	3.90E-05	1.22E-02	Distal Intergenic
Furin	54281	chr1	142362918	142363218	-165736	-2.74	3.95E-05	1.23E-02	Distal Intergenic
Mcf2l	117020	chr16	82004630	82004930	-59503	-2.81	4.16E-05	1.28E-02	Intron (NM_001107324/306600, intron 25 of 28)
Mrps31	290850	chr16	74506459	74506759	-9906	2.09	4.17E-05	1.28E-02	Distal Intergenic
Nfib	29227	chr5	100174531	100174831	472467	-2.64	4.27E-05	1.31E-02	Distal Intergenic
Fars2	306879	chr17	29247621	29247921	190747	-2.39	4.32E-05	1.32E-02	Intron (NM_001013139/306879, intron 5 of 8)
Thumpd3	500288	chr4	144977316	144977616	-8252	2.6	4.41E-05	1.32E-02	Distal Intergenic
Rtp3	316018	chr8	119262078	119262378	2779	-2.7	4.41E-05	1.32E-02	Promoter (2-3kb)
C1s	192262	chr4	157155047	157155347	249	-1.22	4.41E-05	1.32E-02	Promoter (<=1kb)
Ccnd2	64033	chr4	160013763	160014063	-316556	-3.01	4.49E-05	1.33E-02	Distal Intergenic
Mmp23	94339	chr5	173079395	173079695	1745	3.04	4.50E-05	1.33E-02	Promoter (1-2kb)

Rdh8	690953	chr8	21847778	21848078	12675	1.44	4.52E-05	1.33E-02	Distal Intergenic
Gfra4	66023	chr3	123594520	123594820	-13002	-2.72	4.57E-05	1.34E-02	Distal Intergenic
Chst11	314694	chr7	26770928	26771228	88488	-2.41	4.65E-05	1.35E-02	Intron (NM_001108079/314694, intron 1 of 2)
Cd180	294706	chr2	33228490	33228790	408168	-2.45	4.66E-05	1.35E-02	Distal Intergenic
Ndc1	362557	chr5	127077863	127078163	101223	-2.16	4.71E-05	1.35E-02	Distal Intergenic
Cdk1	54237	chr20	20584599	20584899	8222	-2.51	4.72E-05	1.35E-02	Intron (NM_019296/54237, intron 4 of 7)
Cxadr	89843	chr11	16713870	16714170	-112229	2.88	4.74E-05	1.35E-02	Distal Intergenic
Dtwd2	361326	chr18	43533383	43533683	411590	-2.43	4.78E-05	1.36E-02	Distal Intergenic
Chchd6	297436	chr4	121264308	121264608	300604	-2.51	4.87E-05	1.37E-02	Distal Intergenic
Abcg3	498327	chr14	5701772	5702072	-269912	-1.45	4.89E-05	1.37E-02	Intron (NM_001037205/360997, intron 10 of 15)
Eif2s2	296302	chr3	150423527	150423827	-11348	-2.76	4.89E-05	1.37E-02	Distal Intergenic
Tanc1	311055	chr3	45732148	45732448	13567	-2.6	4.91E-05	1.37E-02	Intron (NM_001002854/311055, intron 1 of 25)
LOC687707	687707	chr10	57215722	57216022	-23971	-2.68	4.94E-05	1.37E-02	Intron (NM_001271136/303259, intron 1 of 32)
Atp2c1	170699	chr8	114064188	114064488	-24223	-2.36	4.97E-05	1.37E-02	Distal Intergenic
Rbpj	679028	chr14	59675146	59675446	60004	-2.67	5.07E-05	1.39E-02	Intron (NM_001106631/679028, intron 4 of 11)
LOC682259	682259	chr1	129273390	129273690	340713	-2.74	5.09E-05	1.39E-02	Distal Intergenic
Bcl2	24224	chr13	26645431	26645731	123643	1.98	5.19E-05	1.41E-02	Intron (NM_016993/24224, intron 1 of 1)
Ston1	360202	chr6	12357668	12357968	-4845	1.52	5.22E-05	1.41E-02	Distal Intergenic
Oas2	363938	chr12	41341412	41341712	0	-3.13	5.24E-05	1.41E-02	Promoter (<=1kb)
Kcnq5	259273	chr9	27932608	27932908	208206	-1.89	5.28E-05	1.41E-02	Intron (NM_001134643/259273, intron 1 of 14)
Tcf7l1	312451	chr4	100574585	100574885	85257	2.45	5.81E-05	1.55E-02	Intron (NM_001107865/312451, intron 3 of 11)
Ifi27l2b	299269	chr6	127359913	127360213	-22122	-2.22	5.86E-05	1.55E-02	Distal Intergenic
Erlin2	290823	chr16	69211488	69211788	-16466	2.17	5.96E-05	1.57E-02	Distal Intergenic
Lhfpl2	294643	chr2	23610617	23610917	-159791	-2.18	5.98E-05	1.57E-02	Distal Intergenic
Rcbtb1	361050	chr15	39687900	39688200	65328	-2.34	6.00E-05	1.57E-02	Distal Intergenic
Serpine2	29366	chr9	85620473	85620773	5321	-2.01	6.05E-05	1.57E-02	Intron (NM_019197/29366, intron 1 of 8)
Bfsp2	501046	chr8	112168742	112169042	-202853	-2.46	6.23E-05	1.61E-02	Distal Intergenic
Neurl1	309459	chr1	267007696	267007996	54557	1.48	6.27E-05	1.61E-02	Intron (NM_001107605/309459, intron 1 of 3)
Cyth3	116693	chr12	12933367	12933667	-25595	-2.77	6.28E-05	1.61E-02	Distal Intergenic
Adora2b	29316	chr10	48580426	48580726	10860	-2.64	6.48E-05	1.65E-02	Intron (NM_017161/29316, intron 1 of 1)
Paqr9	315904	chr8	103186131	103186431	-151041	-2.32	6.52E-05	1.65E-02	Distal Intergenic
Atp2a2	29693	chr12	39527174	39527474	-26429	-2.58	6.58E-05	1.65E-02	Distal Intergenic
Cxcl12	24772	chr4	149451614	149451914	190570	-2.12	6.59E-05	1.65E-02	Distal Intergenic
Il17ra	312679	chr4	153017365	153017665	21500	1.89	6.59E-05	1.65E-02	3' UTR
Osbp2	305475	chr14	84002266	84002566	104431	1.88	6.73E-05	1.67E-02	Intron (NM_001107232/305475, intron 2 of 14)
Aqp5	25241	chr7	141250626	141250926	1576	3.38	6.76E-05	1.67E-02	Promoter (1-2kb)
Aldh6a1	81708	chr6	108171025	108171325	-3840	-2.69	6.77E-05	1.67E-02	Distal Intergenic
Rpp38	291317	chr17	78928676	78928976	13072	-2.9	6.91E-05	1.69E-02	Intron (NM_207590/291318, intron 10 of 13)
Ntng2	311836	chr3	7786301	7786601	9157	-2.74	6.91E-05	1.69E-02	Intron (NM_001107825/311836, intron 1 of 7)
Tssc4	361682	chr1	216260789	216261089	5878	-2.37	7.02E-05	1.71E-02	Exon (NM_001191896/365391, exon 19 of 24)
Nova1	298992	chr6	67085023	67085323	67	1.65	7.07E-05	1.72E-02	Promoter (<=1kb)
Adipor2	312670	chr4	151443217	151443517	-14398	-2.01	7.13E-05	1.72E-02	Distal Intergenic
Kctd3	305055	chr13	107400918	107401218	70625	1.91	7.35E-05	1.77E-02	Intron (NM_001302219/289369, intron 63 of 70)
Eif2ak2	54287	chr6	1458909	1459209	6984	-1.65	7.38E-05	1.77E-02	Intron (NM_019335/54287, intron 1 of 15)
Tnip1	363599	chr10	40314430	40314730	-11358	-2.07	7.45E-05	1.78E-02	Distal Intergenic
Sh3bp4	64634	chr9	96413589	96413889	202839	3.03	7.50E-05	1.78E-02	Distal Intergenic
Pelp1	360552	chr10	56997639	56997939	7172	-2.44	7.61E-05	1.79E-02	Intron (NM_001024270/360552, intron 3 of 16)
Cacna1a	25398	chr19	25644978	25645278	118227	-2.68	7.62E-05	1.79E-02	Intron (NM_012918/25398, intron 7 of 46)
Cd180	294706	chr2	32776861	32777161	-43161	-2.13	7.72E-05	1.81E-02	Distal Intergenic
RGD1564149	499419	chr20	12940120	12940420	-1229	3.02	7.76E-05	1.81E-02	Promoter (1-2kb)
Ptger3	24929	chr2	263900525	263900825	5320	-2.2	7.78E-05	1.81E-02	Intron (NM_012704/24929, intron 1 of 2)
Depp1	500300	chr4	148782994	148783294	515	-1.41	7.81E-05	1.81E-02	Promoter (<=1kb)
Cab39	301574	chr9	92801859	92802159	-32198	2.3	8.02E-05	1.85E-02	Distal Intergenic
Prpf18	171552	chr17	77694586	77694886	92672	-2.5	8.10E-05	1.86E-02	Intron (NM_001191821/307128, intron 11 of 21)
Rps3a	29288	chr2	185283863	185284163	160683	-2.4	8.15E-05	1.86E-02	Distal Intergenic
Cx3cl1	89808	chr19	10644295	10644595	9166	-2.76	8.15E-05	1.86E-02	3' UTR
Kcnh6	116745	chr10	94214999	94215299	7663	-2.16	8.23E-05	1.87E-02	Intron (NM_053937/116745, intron 4 of 12)
Nlk	497961	chr10	65950256	65950556	12231	-2.28	8.36E-05	1.88E-02	Intron (NM_001191924/497961, intron 1 of 10)
Sdk2	360652	chr10	102577443	102577743	-154359	1.8	8.38E-05	1.88E-02	Distal Intergenic
Cpt1a	25757	chr1	218578661	218578961	9151	-2.46	8.39E-05	1.88E-02	Intron (NM_031559/25757, intron 1 of 18)
Ampd3	25095	chr1	175591370	175591670	5273	-3.02	8.52E-05	1.90E-02	Intron (NM_031544/25095, intron 1 of 14)
Gsap	311984	chr4	10516094	10516394	1438	-2.31	8.58E-05	1.91E-02	Promoter (1-2kb)
Ybx1	500538	chr5	138330737	138331037	5444	-2.44	8.65E-05	1.92E-02	Intron (NM_031563/500538, intron 2 of 7)
Nedd4	25489	chr8	79347210	79347510	23802	-2.63	8.86E-05	1.95E-02	Exon (NM_012986/25489, exon 6 of 29)
Chfr	288734	chr12	52588096	52588396	7680	2.71	8.97E-05	1.96E-02	Intron (NM_001009258/288734, intron 3 of 17)
Slc35e3	362883	chr7	60772457	60772757	5379	-2.75	8.97E-05	1.96E-02	Exon (NM_001134687/362883, exon 3 of 5)
Guca2b	64055	chr5	138844276	138844576	-146635	-2.62	9.12E-05	1.99E-02	Distal Intergenic
Elmod1	315670	chr8	58542623	58542923	0	1.68	9.16E-05	1.99E-02	Promoter (<=1kb)
Slc29a1	63997	chr9	17760626	17760926	-23542	-2.42	9.35E-05	2.02E-02	Distal Intergenic
Gpr158	291352	chr17	88158269	88158569	-56562	-1.94	9.38E-05	2.02E-02	Distal Intergenic
Lama4	309816	chr20	44061094	44061394	379	1.75	9.48E-05	2.03E-02	Promoter (<=1kb)
Jun	24516	chr5	114018545	114018845	-4268	-2.42	9.58E-05	2.04E-02	Distal Intergenic
Adal	311352	chr3	113141170	113141470	39080	-2.31	9.63E-05	2.04E-02	Distal Intergenic
Rn5-8s	100861534	chr1	11909902	11910202	-3308	3.62	9.71E-05	2.04E-02	Intron (NM_001025002/310926, intron 6 of 6)
Chfr	288734	chr12	52588855	52589155	6921	2.96	9.74E-05	2.04E-02	Intron (NM_001009258/288734, intron 3 of 17)
Atp11a	306600	chr16	82077910	82078210	22012	-2.33	9.81E-05	2.04E-02	Intron (NM_001107324/306600, intron 1 of 28)
Lum	81682	chr7	38825234	38825534	5176	3.27	9.82E-05	2.04E-02	Intron (NM_031050/81682, intron 2 of 2)
Dlx2	296499	chr3	58280879	58281179	-98908	1.21	9.85E-05	2.04E-02	Distal Intergenic
Slc16a7	29735	chr7	68489529	68489829	59934	-1.32	9.87E-05	2.04E-02	Intron (NM_017302/29735, intron 2 of 4)
Oas1i	304507	chr12	41136643	41136943	-18546	-1.95	9.91E-05	2.04E-02	Intron (NM_133518/171039, intron 13 of 21)
Nfib	29227	chr5	100278150	100278450	368848	-2.17	9.96E-05	2.04E-02	Distal Intergenic
Fcrl6	305103	chr13	90995837	90996137	-3422	-2.33	9.96E-05	2.04E-02	Distal Intergenic
Stat5a	24918	chr10	88764494	88764794	0	1.4	9.99E-05	2.04E-02	Promoter (<=1kb)
Extl3	56819	chr15	48441942	48442242	3350	-2.17	9.99E-05	2.04E-02	Intron (NM_020097/56819, intron 1 of 4)
Tdo2	64206	chr2	180842051	180842351	72568	-2.67	1.03E-04	2.09E-02	Distal Intergenic
Olfm2	313783	chr8	21755910	21756210	-12598	-1.94	1.04E-04	2.10E-02	Distal Intergenic
Lrch1	502020	chr15	56914900	56915200	55165	-2.49	1.06E-04	2.13E-02	Intron (NM_001134727/502020, intron 1 of 18)
Stk36	301516	chr9	81918843	81919143	38666	-2.7	1.06E-04	2.14E-02	Distal Intergenic
G2e3	299002	chr6	72036694	72036994	-55200	-2.34	1.09E-04	2.18E-02	Distal Intergenic
Csf1	78965	chr2	210565180	210565480	-14634	-2.65	1.10E-04	2.19E-02	Distal Intergenic
Smtnl2	679629	chr10	58972714	58973014	6	2.89	1.12E-04	2.22E-02	Promoter (<=1kb)
Asap1	314961	chr7	104891576	104891876	59214	-1.87	1.12E-04	2.22E-02	Intron (NM_001044245/314961, intron 1 of 27)
Vps37b	288659	chr12	37998321	37998621	13531	-2.11	1.12E-04	2.22E-02	Intron (NM_001105928/288659, intron 1 of 3)
Zfp131	310375	chr2	52808658	52808958	-277573	-1.34	1.13E-04	2.22E-02	Distal Intergenic

Lta	25008	chr20	4847227	4847527	-5144	-2.78	1.13E-04	2.22E-02	Intron (NM_001008837/414270, intron 6 of 7)
Alpl	25586	chr5	156141271	156141571	0	1.39	1.14E-04	2.22E-02	Promoter (<=1kb)
Rpn2	64701	chr3	153426712	153427012	28280	2.1	1.14E-04	2.22E-02	Intron (NM_031698/64701, intron 9 of 16)
Aebp2	297705	chr4	174891540	174891840	80581	2.75	1.16E-04	2.24E-02	Distal Intergenic
Tagln	25123	chr8	50228123	50228423	0	1.45	1.17E-04	2.26E-02	Promoter (<=1kb)
Fign	295649	chr3	50111855	50112155	8237	-2.48	1.17E-04	2.26E-02	Intron (NM_001106484/295649, intron 2 of 2)
Tnfaip1	287543	chr10	65799807	65800107	5586	-1.9	1.18E-04	2.27E-02	Intron (NM_182950/287543, intron 1 of 6)
Serpine2	29366	chr9	85799141	85799441	-173047	-2.44	1.19E-04	2.28E-02	Distal Intergenic
Otp	294640	chr2	24523222	24523522	-22871	2.21	1.19E-04	2.28E-02	Distal Intergenic
Cbr4	359725	chr16	31760799	31761099	211408	-2.32	1.23E-04	2.33E-02	Distal Intergenic
Acod1	306127	chr15	93613512	93613812	544	-2.7	1.23E-04	2.33E-02	Promoter (<=1kb)
Cyb5b	80773	chr19	39360876	39361176	3062	-2.55	1.24E-04	2.33E-02	Intron (NM_030586/80773, intron 1 of 4)
Ccdc92	100036765	chr12	37193559	37193859	-17457	-2.52	1.25E-04	2.34E-02	Distal Intergenic
Rptor	287871	chr10	108836229	108836529	85609	-2.57	1.26E-04	2.34E-02	Intron (NM_001134499/287871, intron 3 of 33)
Bcat1	29592	chr4	179266870	179267170	39925	-2.57	1.26E-04	2.34E-02	Exon (NM_017253/29592, exon 9 of 11)
Men1	29417	chr1	221692778	221693078	-11415	2.56	1.26E-04	2.34E-02	3' UTR
Ahsp	293522	chr1	199746236	199746536	26198	-2.36	1.28E-04	2.37E-02	Distal Intergenic
Mitf	25094	chr4	130227216	130227516	54360	-2.6	1.29E-04	2.38E-02	Intron (NM_001191089/25094, intron 1 of 9)
Snx4	360725	chr11	70739102	70739402	14218	-2.49	1.29E-04	2.38E-02	Intron (NM_001127550/360725, intron 1 of 13)
Ror2	306782	chr17	12042977	12043277	89425	1.55	1.30E-04	2.39E-02	Intron (NM_001107339/306782, intron 1 of 8)
Dele1	307480	chr18	31355373	31355673	-41314	-2.77	1.31E-04	2.39E-02	Distal Intergenic
Cacna1i	56827	chr7	121635138	121635438	113351	-2.05	1.31E-04	2.39E-02	Distal Intergenic
Tmem179	314472	chr6	137063873	137064173	20566	1.64	1.32E-04	2.39E-02	Distal Intergenic
R3hdm2	362894	chr7	70727061	70727361	30011	-2.3	1.33E-04	2.41E-02	Intron (NM_001130557/362894, intron 1 of 22)
Pax3	114502	chr9	84107493	84107793	-6321	-2.62	1.34E-04	2.42E-02	Distal Intergenic
Sh2b2	114203	chr12	23253395	23253695	-219575	-2.22	1.34E-04	2.42E-02	Distal Intergenic
Tnfsf8	683163	chr5	79633935	79634235	56680	-2.44	1.35E-04	2.43E-02	Distal Intergenic
Bcor	317346	chrX	11574417	11574717	-74272	1.77	1.36E-04	2.43E-02	Distal Intergenic
Ptk2b	50646	chr15	42923029	42923329	24327	-2.12	1.36E-04	2.43E-02	Intron (NM_017318/50646, intron 1 of 30)
Pja2	192256	chr9	111990946	111991246	57601	2.45	1.37E-04	2.43E-02	Distal Intergenic
Atp6v0a4	296981	chr4	65840929	65841229	-22408	-2.09	1.40E-04	2.48E-02	Distal Intergenic
Aknad1	691416	chr2	211542905	211543205	-3355	2.49	1.41E-04	2.49E-02	Distal Intergenic
Spg7	353231	chr19	55835425	55835725	-44836	-2.32	1.42E-04	2.49E-02	Distal Intergenic
Pdgfc	79429	chr2	180006522	180006822	54295	-2.58	1.42E-04	2.49E-02	Intron (NM_031317/79429, intron 1 of 5)
Ptger2	81752	chr15	19383357	19383657	45027	-2.41	1.45E-04	2.53E-02	Distal Intergenic
Cald1	25687	chr4	62287520	62287820	66509	2.59	1.45E-04	2.53E-02	Intron (NM_013146/25687, intron 11 of 11)
Selenop	29360	chr2	52914080	52914380	-191532	-2.5	1.47E-04	2.55E-02	Distal Intergenic
Shc3	114858	chr17	13664568	13664868	-5652	-2.47	1.47E-04	2.55E-02	Distal Intergenic
Cgnl1	315795	chr8	78202052	78202352	74202	2.4	1.48E-04	2.55E-02	Intron (NM_001108164/315795, intron 8 of 20)
Chst15	286974	chr1	204315699	204315999	-40157	-2.69	1.49E-04	2.56E-02	Distal Intergenic
RT1-N1	24748	chr20	3146849	3147149	0	-1.35	1.53E-04	2.62E-02	Promoter (<=1kb)
Tnfsf12	360548	chr10	56309447	56309747	-9370	-1.97	1.55E-04	2.63E-02	Distal Intergenic
Trib2	313974	chr6	40934509	40934809	104628	-1.75	1.55E-04	2.63E-02	Distal Intergenic
Rcbtb1	361050	chr15	39683207	39683507	60635	-2.32	1.55E-04	2.63E-02	Distal Intergenic
Ttc39c	686179	chr18	3864564	3864864	-93568	-3.02	1.55E-04	2.63E-02	Distal Intergenic
Kcnh2	117018	chr4	7377572	7377872	21971	2.09	1.56E-04	2.63E-02	Intron (NM_053949/117018, intron 5 of 14)
Tbc1d7	361227	chr17	23582690	23582990	209363	-2.12	1.56E-04	2.63E-02	Intron (NM_214457/306844, intron 5 of 13)
Rbks	362706	chr6	26131722	26132022	80108	-2.16	1.57E-04	2.63E-02	Distal Intergenic
Eif4a3	688288	chr10	108530647	108530947	-105452	-1.85	1.61E-04	2.70E-02	Distal Intergenic
Htt	29424	chr14	81184178	81184478	70159	-1.99	1.62E-04	2.70E-02	Intron (NM_024357/29424, intron 26 of 66)
Atp5po	192241	chr11	32043899	32044199	43719	2.85	1.62E-04	2.71E-02	Intron (NM_001136096/29491, intron 28 of 38)
Tead1	361630	chr1	177513872	177514172	18090	-2.07	1.64E-04	2.72E-02	Intron (NM_001198589/361630, intron 1 of 12)
Ifi47	246208	chr10	34278051	34278351	58	-1.55	1.65E-04	2.74E-02	Promoter (<=1kb)
Frs2	314850	chr7	60195752	60196052	14430	-2.33	1.67E-04	2.76E-02	Intron (NM_001108097/314850, intron 1 of 9)
Slc25a19	303676	chr10	104216002	104216302	-36479	-2.36	1.68E-04	2.76E-02	Intron (NM_030846/81504, intron 2 of 5)
C1rl	408246	chr4	157112965	157113265	4775	-2.6	1.68E-04	2.76E-02	Intron (NM_001002804/408246, intron 1 of 4)
Acod1	306127	chr15	93614506	93614806	1538	-2.71	1.69E-04	2.76E-02	Promoter (1-2kb)
Veph1	361954	chr2	158364537	158364837	-208387	-2.29	1.69E-04	2.76E-02	Distal Intergenic
Slc7a5	50719	chr19	54714765	54715065	7498	-1.95	1.70E-04	2.76E-02	Intron (NM_017353/50719, intron 1 of 9)
Fer	301737	chr9	111730345	111730645	80714	-2.07	1.71E-04	2.78E-02	Intron (NM_001106928/301737, intron 7 of 13)
Nbn	85482	chr5	29620717	29621017	-1330	-2.53	1.73E-04	2.80E-02	Promoter (1-2kb)
Tlr2	310553	chr2	182845551	182845851	210	-2.71	1.77E-04	2.85E-02	Promoter (<=1kb)
Serpina3n	24795	chr6	128076475	128076775	3131	-2.59	1.77E-04	2.85E-02	Intron (NM_031531/24795, intron 2 of 4)
Maoa	29253	chrX	6621338	6621638	-616	1.49	1.78E-04	2.85E-02	Promoter (<=1kb)
Optn	246294	chr17	77178283	77178583	1578	2	1.81E-04	2.89E-02	Promoter (1-2kb)
Oasl2	304549	chr12	47486516	47486816	-3569	-2.61	1.82E-04	2.90E-02	Distal Intergenic
Klf10	81813	chr7	77154142	77154442	7654	2.7	1.84E-04	2.92E-02	Downstream (1-2kb)
Mirlet7f2	100313992	chrX	21446283	21446583	-136689	-2.04	1.85E-04	2.93E-02	Distal Intergenic
Hlx	364069	chr13	102648466	102648766	-5090	-1.92	1.87E-04	2.95E-02	Distal Intergenic
Tnfrsf26	361685	chr1	216867857	216868157	-39276	-1.37	1.88E-04	2.96E-02	Intron (NM_001015024/361686, intron 21 of 21)
Nkain4	296469	chr3	176464720	176465020	142	2.83	1.89E-04	2.96E-02	Promoter (<=1kb)
Oasl	304545	chr12	47463653	47463953	-7493	-2.45	1.89E-04	2.96E-02	Distal Intergenic
Sulf2	311642	chr3	162854372	162854672	18159	-2.62	1.90E-04	2.96E-02	Intron (NM_001034927/311642, intron 1 of 20)
Tnip1	363599	chr10	40288650	40288950	14122	-2.44	1.91E-04	2.97E-02	Intron (NM_001108826/363599, intron 2 of 18)
Pla2g2a	29692	chr5	157281742	157282042	-676	-2.48	1.93E-04	2.99E-02	Promoter (<=1kb)
Oas1i	304507	chr12	41137463	41137763	-17726	-2.1	1.94E-04	3.00E-02	Intron (NM_133518/171039, intron 13 of 21)
Myh9l1	25745	chr7	118846106	118846406	-53599	-2.28	1.95E-04	3.01E-02	Distal Intergenic
Ifitm2	114709	chr1	213747459	213747759	3640	-2.18	1.96E-04	3.02E-02	Downstream (2-3kb)
Slc30a4	64469	chr3	114822341	114822641	5513	-2.3	2.00E-04	3.07E-02	Intron (NM_172066/64469, intron 1 of 6)
Tspan3	300733	chr8	60940885	60941185	-110437	2.81	2.01E-04	3.08E-02	Intron (NM_001108149/315686, intron 3 of 6)
Arhgap22	306279	chr16	9500804	9501104	-62114	-2.63	2.07E-04	3.14E-02	Distal Intergenic
Lpar3	66025	chr2	252117563	252117863	3190	-2.63	2.07E-04	3.14E-02	Intron (NM_023969/66025, intron 1 of 1)
Kcnt1	60444	chr3	3316162	3316462	-9308	-2.63	2.07E-04	3.14E-02	Distal Intergenic
Lmod1	304816	chr13	52169275	52169575	21707	1.03	2.09E-04	3.16E-02	Intron (NM_001107179/304816, intron 1 of 3)
Cyb561	303601	chr10	94149758	94150058	-2202	-2.06	2.10E-04	3.16E-02	Promoter (2-3kb)
Tsen2	312649	chr4	147448253	147448553	-6965	2.34	2.13E-04	3.21E-02	Distal Intergenic
Oas1a	192281	chr12	41200782	41201082	53	-2.07	2.14E-04	3.21E-02	Promoter (<=1kb)
Dcaf5	314273	chr6	103797569	103797869	-192313	-2.54	2.15E-04	3.22E-02	Distal Intergenic
Fam180a	362336	chr4	62859540	62859840	521	3.07	2.17E-04	3.24E-02	Promoter (<=1kb)
Mrpl45	287656	chr10	85381269	85381569	123393	1.6	2.18E-04	3.25E-02	Distal Intergenic
LOC362901	362901	chr7	75576812	75577112	-7034	-2.06	2.19E-04	3.25E-02	Intron (NM_013011/25578, intron 5 of 5)
Fstl1	79210	chr11	65884742	65885042	-39367	-2.16	2.20E-04	3.25E-02	Distal Intergenic
Fam49b	299909	chr7	104766115	104766415	-124320	-2.26	2.20E-04	3.25E-02	Intron (NM_001044245/314961, intron 3 of 27)
Ptprz1	25613	chr4	49888760	49889060	-52244	-2.35	2.20E-04	3.25E-02	Distal Intergenic

Majin	499306	chr1	221537595	221537895	-20198	-2.29	2.22E-04	3.27E-02	Distal Intergenic
Il23a	155140	chr7	2713373	2713673	-650	-2.64	2.24E-04	3.28E-02	Promoter (<=1kb)
Fam133b	362320	chr4	27767143	27767443	-11961	-2.18	2.28E-04	3.32E-02	Distal Intergenic
Arih1	300756	chr8	64254062	64254362	14193	-2.16	2.29E-04	3.32E-02	Intron (NM_001013108/300756, intron 1 of 13)
Jun	24516	chr5	113989009	113989309	24968	-2.46	2.29E-04	3.32E-02	Distal Intergenic
Pdhx	311254	chr3	92865132	92865432	68293	-2.53	2.30E-04	3.32E-02	Distal Intergenic
Ccdc82	300359	chr8	11982644	11982944	94124	-2.23	2.30E-04	3.32E-02	Distal Intergenic
Mtnr1a	114211	chr16	50315734	50316034	42775	2.24	2.31E-04	3.32E-02	Distal Intergenic
Diaph1	307483	chr18	31054904	31055204	16167	-1.97	2.32E-04	3.32E-02	Intron (NM_001107393/307483, intron 1 of 29)
Pelp1	360552	chr10	56994150	56994450	10661	-2.69	2.32E-04	3.32E-02	Intron (NM_001024270/360552, intron 5 of 16)
Gsdme	353316	chr4	80116704	80117004	-117026	-2.69	2.32E-04	3.32E-02	Distal Intergenic
Tubg1	252921	chr10	89048245	89048545	17380	2.28	2.33E-04	3.33E-02	Distal Intergenic
Siglec5	292843	chr1	98720511	98720811	-149785	-2.47	2.34E-04	3.33E-02	Distal Intergenic
Pik3c2g	116720	chr4	173734608	173734908	2360	-2.03	2.35E-04	3.34E-02	Promoter (2-3kb)
Klhl40	316088	chr8	130364604	130364904	-51451	-2.07	2.36E-04	3.35E-02	Distal Intergenic
Tns3	360980	chr14	88896348	88896648	-49477	-2.26	2.37E-04	3.35E-02	Distal Intergenic
Rab32	365042	chr1	4655551	4655851	-2331	-2.64	2.38E-04	3.35E-02	Promoter (2-3kb)
Atp5mg	300677	chr8	49179801	49180101	47172	-2.44	2.38E-04	3.35E-02	Distal Intergenic
Tp73	362675	chr5	171409838	171410138	5216	-2.63	2.41E-04	3.36E-02	Intron (NM_001108696/362675, intron 1 of 13)
Kdm4d	689582	chr8	12942458	12942758	50893	-2.58	2.41E-04	3.36E-02	Distal Intergenic
Sh3gl2	116743	chr5	104156561	104156861	676794	2.56	2.41E-04	3.36E-02	Distal Intergenic
Il1rn	60582	chr3	1453320	1453620	-9588	-1.79	2.42E-04	3.36E-02	Distal Intergenic
Smad2	29357	chr18	72375912	72376212	-174007	-2.65	2.44E-04	3.39E-02	Intron (NM_001127375/679155, intron 3 of 5)
Galnt7	29750	chr16	35928036	35928336	-6723	-2.38	2.47E-04	3.42E-02	Distal Intergenic
Ppa2	310856	chr2	238613151	238613451	84079	1.96	2.47E-04	3.42E-02	Distal Intergenic
Ncor2	360801	chr12	36951851	36952151	79852	2.8	2.49E-04	3.43E-02	Intron (NM_001108334/360801, intron 7 of 46)
Mir532	100314265	chrX	15988027	15988327	-121543	1.67	2.50E-04	3.44E-02	Distal Intergenic
Pdgfa	25266	chr12	17717618	17717918	-16223	-2.23	2.51E-04	3.44E-02	Distal Intergenic
Scx	680712	chr7	117526982	117527282	7907	2.86	2.52E-04	3.45E-02	Intron (NM_001024250/300050, intron 3 of 15)
Ciapin1	307649	chr19	10605433	10605733	8419	-2.3	2.55E-04	3.47E-02	Intron (NM_001007689/307649, intron 5 of 8)
Cx3cl1	89808	chr19	10658566	10658866	-4805	-2.12	2.58E-04	3.51E-02	Distal Intergenic
Tomm20	266601	chr19	59753037	59753337	120074	-2.15	2.59E-04	3.51E-02	Distal Intergenic
Epdr1	291180	chr17	47403106	47403406	5548	-2.3	2.61E-04	3.54E-02	Intron (NM_001007625/291180, intron 1 of 2)
Etf1	307503	chr18	27716026	27716326	9368	-2.49	2.64E-04	3.54E-02	Intron (NM_001008344/307503, intron 2 of 10)
Acp1	24161	chr4	99107711	99108011	13133	-2.49	2.64E-04	3.54E-02	Intron (NM_001106595/297333, intron 1 of 9)
Rspo1	313589	chr5	142986676	142986976	150	1.84	2.64E-04	3.54E-02	Promoter (<=1kb)
Sh3bp4	64634	chr9	96057734	96058034	-152716	-1.46	2.65E-04	3.54E-02	Distal Intergenic
Oaz2	501454	chr8	71220429	71220729	4418	-1.97	2.65E-04	3.54E-02	Intron (NM_001109899/501454, intron 1 of 4)
Fat1	83720	chr16	50975727	50976027	-474011	2.49	2.65E-04	3.54E-02	Distal Intergenic
Hmgcr	25675	chr2	27510349	27510649	-9695	-2.22	2.67E-04	3.54E-02	Distal Intergenic
Ppp1r8	313030	chr5	151022708	151023008	6219	-2.06	2.67E-04	3.54E-02	Intron (NM_001107911/313030, intron 2 of 6)
Sgcg	305941	chr15	41604698	41604998	-9423	1.68	2.69E-04	3.56E-02	Distal Intergenic
Mir30a	100314011	chr9	29550611	29550911	7976	-2.41	2.70E-04	3.56E-02	Distal Intergenic
Vps26c	360703	chr11	34802494	34802794	-10501	2.55	2.70E-04	3.56E-02	Distal Intergenic
Bmper	300455	chr8	24523401	24523701	153485	2.55	2.71E-04	3.56E-02	Intron (NM_001135799/300455, intron 11 of 14)
Fam102b	365903	chr2	211814607	211814907	16930	-1.97	2.71E-04	3.56E-02	Intron (NM_001163568/365903, intron 1 of 10)
Adss	289276	chr13	95938129	95938429	5332	-2.08	2.74E-04	3.59E-02	Intron (NM_001105975/289276, intron 1 of 12)
Ltc4s	114097	chr10	35739186	35739486	133	2.73	2.75E-04	3.59E-02	Promoter (<=1kb)
Daam1	314212	chr6	94624414	94624714	-11508	-2.14	2.77E-04	3.59E-02	Distal Intergenic
Mkln1	83536	chr4	58603665	58603965	-89419	1.88	2.77E-04	3.59E-02	Distal Intergenic
Itgb1	24511	chr19	61722580	61722880	45038	-2.23	2.77E-04	3.59E-02	Intron (NM_017022/24511, intron 15 of 15)
Atf7	315333	chr7	144236169	144236469	-12740	1.63	2.78E-04	3.59E-02	Distal Intergenic
Tbc1d2b	315880	chr8	97639427	97639727	7345	-2.46	2.78E-04	3.59E-02	Intron (NM_001108175/315880, intron 2 of 13)
Smug1	315344	chr7	144778954	144779254	-301	-2.5	2.81E-04	3.62E-02	Promoter (<=1kb)
Paqr8	316275	chr9	26993498	26993798	-40224	-2.22	2.83E-04	3.64E-02	Distal Intergenic
Gnpda1	683570	chr18	31490075	31490375	-921	2.73	2.87E-04	3.67E-02	Promoter (<=1kb)
Stn1	294025	chr1	267273005	267273305	42283	-1.86	2.87E-04	3.67E-02	Distal Intergenic
Otulin	100362554	chr2	80305468	80305768	-12282	-1.62	2.88E-04	3.67E-02	Distal Intergenic
Mir21	100314000	chr10	73918621	73918921	-16320	-1.35	2.89E-04	3.68E-02	Intron (NM_138839/192129, intron 10 of 11)
Mad2l1	297176	chr4	97590901	97591201	59818	-1.95	2.90E-04	3.68E-02	Distal Intergenic
Apol9a	503164	chr7	118930699	118930999	2779	-2.11	2.91E-04	3.68E-02	Promoter (2-3kb)
Selenop	29360	chr2	52925079	52925379	-180533	-2.13	2.91E-04	3.68E-02	Distal Intergenic
Slc25a37	306000	chr15	51168692	51168992	-308	-1.02	2.92E-04	3.68E-02	Promoter (<=1kb)
Arrdc3	309945	chr2	9022997	9023297	289830	-2.48	2.96E-04	3.71E-02	Distal Intergenic
Dip2c	307067	chr17	63351363	63351663	-75353	-2.36	2.96E-04	3.71E-02	Distal Intergenic
Zbtb10	80338	chr2	94529900	94530200	200776	2.85	2.96E-04	3.71E-02	Distal Intergenic
Ankh	114506	chr2	80138844	80139144	7278	-1.95	2.97E-04	3.71E-02	Intron (NM_053714/114506, intron 1 of 12)
Fcgr2b	289211	chr13	89363156	89363456	-19264	-2.55	3.01E-04	3.75E-02	Distal Intergenic
Sh2b3	58838	chr12	40287418	40287718	25428	2.78	3.01E-04	3.75E-02	Distal Intergenic
Slc22a23	64559	chr17	31237920	31238220	1905	-2.59	3.03E-04	3.75E-02	Promoter (1-2kb)
Gda	83585	chr1	239106117	239106417	-48454	-2.23	3.03E-04	3.75E-02	Distal Intergenic
Acot7	26759	chr5	169379851	169380151	15545	-2.37	3.05E-04	3.78E-02	Intron (NM_013214/26759, intron 1 of 8)
Cpz	83575	chr14	80402779	80403079	0	2.77	3.08E-04	3.79E-02	Promoter (<=1kb)
Tmem212	499586	chr2	113607364	113607664	9102	-1.84	3.09E-04	3.79E-02	Intron (NM_001164439/499586, intron 1 of 3)
Parp9	303905	chr11	67746989	67747289	9508	-2.64	3.09E-04	3.79E-02	Intron (NM_001103351/303905, intron 4 of 10)
Cpd	25306	chr10	63330204	63330504	4440	-2.31	3.10E-04	3.79E-02	Intron (NM_012836/25306, intron 1 of 20)
Tmcc3	314751	chr7	35368158	35368458	-103644	2.58	3.11E-04	3.79E-02	Distal Intergenic
Gab2	84477	chr1	162054014	162054314	-28728	1.14	3.11E-04	3.79E-02	Distal Intergenic
Tspan18	311210	chr3	82189256	82189556	47108	2.94	3.13E-04	3.81E-02	Intron (NM_001107750/311210, intron 2 of 9)
Gsdme	353316	chr4	80109812	80110112	-110134	-2.09	3.14E-04	3.81E-02	Distal Intergenic
Yars1	313047	chr5	147379174	147379474	3824	-2.23	3.15E-04	3.81E-02	Intron (NM_001025696/313047, intron 1 of 12)
P2ry2	29597	chr1	166042686	166042986	2434	-2.41	3.15E-04	3.81E-02	Promoter (2-3kb)
Myo1c	65261	chr10	63792599	63792899	-10410	2.75	3.16E-04	3.81E-02	Intron (NM_001013859/287533, intron 8 of 20)
Ccdc152	499536	chr2	53127176	53127476	10797	-2.52	3.18E-04	3.83E-02	Intron (NM_001191959/499536, intron 4 of 7)
Rasal3	314596	chr7	14470843	14471143	-51893	-2.48	3.19E-04	3.83E-02	Distal Intergenic
Tcp11	309641	chr20	7679901	7680201	-25125	-2.48	3.20E-04	3.83E-02	Distal Intergenic
Apoa1	25081	chr8	50462594	50462894	-62197	-2.46	3.24E-04	3.88E-02	Intron (NM_001271216/684112, intron 4 of 24)
Smad2	29357	chr18	72565030	72565330	14576	-2.3	3.28E-04	3.91E-02	Intron (NM_001277450/29357, intron 1 of 10)
Ttc39c	686179	chr18	3840066	3840366	-118066	-2.48	3.29E-04	3.92E-02	Distal Intergenic
Pmfbp1	171414	chr19	42146309	42146609	34355	-2.38	3.30E-04	3.92E-02	Intron (NM_134393/171414, intron 9 of 19)
Shh	29499	chr4	1032345	1032645	-304654	-1.87	3.32E-04	3.93E-02	Distal Intergenic
Arf6	79121	chr6	91778161	91778461	81052	-2.46	3.33E-04	3.93E-02	Distal Intergenic
Myct1	292264	chr1	41870828	41871128	-250444	-2.14	3.34E-04	3.94E-02	Distal Intergenic
Kcna6	64358	chr4	159287440	159287740	-247	-2.38	3.40E-04	4.01E-02	Promoter (<=1kb)

Cldn11	84588	chr2	115876070	115876370	-39224	-2.43	3.44E-04	4.03E-02	Distal Intergenic
Prkar1b	25521	chr12	17617346	17617646	2781	-2.38	3.44E-04	4.03E-02	Promoter (2-3kb)
Mgat3	29582	chr7	121394738	121395038	-13791	1.29	3.44E-04	4.03E-02	Distal Intergenic
Fam20c	304334	chr12	18022166	18022466	-49433	-2.03	3.46E-04	4.03E-02	Distal Intergenic
Prepl	298771	chr6	8323898	8324198	21999	-2.31	3.47E-04	4.03E-02	Intron (NM_001010951/298771, intron 9 of 13)
Cdh15	361432	chr19	55742168	55742468	72507	-1.63	3.47E-04	4.03E-02	Distal Intergenic
Zbtb10	80338	chr2	94784615	94784915	-53639	-2.21	3.47E-04	4.03E-02	Distal Intergenic
Klf9	117560	chr1	240919225	240919525	10742	-1.85	3.49E-04	4.04E-02	Intron (NM_057211/117560, intron 1 of 1)
Tcea3	298559	chr5	154559699	154559999	-38766	-2.73	3.49E-04	4.04E-02	Distal Intergenic
Slit2	360272	chr14	68347324	68347624	-1176963	2.4	3.54E-04	4.08E-02	Distal Intergenic
Mettl21c	301378	chr9	50761818	50762118	0	-2.09	3.55E-04	4.08E-02	Promoter (<=1kb)
Vmac	363327	chr9	10362663	10362963	23588	2.62	3.56E-04	4.08E-02	Distal Intergenic
Mgat4a	367252	chr9	44250054	44250354	-123135	-2.27	3.56E-04	4.08E-02	Distal Intergenic
Kl	83504	chr12	869051	869351	-73623	-2.1	3.57E-04	4.08E-02	Distal Intergenic
Lamc1	117036	chr13	70757979	70758279	25236	-2.15	3.61E-04	4.12E-02	Intron (NM_053966/117036, intron 1 of 27)
Zfp746	312303	chr4	77742625	77742925	4145	-2.39	3.64E-04	4.14E-02	Exon (NM_001100852/312303, exon 4 of 7)
Acsl3	114024	chr9	84571102	84571402	1502	-2.14	3.65E-04	4.14E-02	Promoter (1-2kb)
Gadd45b	299626	chr7	11635873	11636173	12139	-2.07	3.70E-04	4.19E-02	Distal Intergenic
Akt1	24185	chr6	137230871	137231171	5030	-2.6	3.70E-04	4.19E-02	Intron (NM_033230/24185, intron 1 of 12)
Cyld	312937	chr19	19290522	19290822	32995	2.7	3.71E-04	4.19E-02	Intron (NM_001017380/312937, intron 9 of 19)
Scrt1	366951	chr7	117587425	117587725	-322	3.23	3.75E-04	4.23E-02	Promoter (<=1kb)
Sema3f	315996	chr8	116469514	116469814	101	1.46	3.76E-04	4.23E-02	Promoter (<=1kb)
Ifi44	310969	chr2	256948905	256949205	-33342	-2.71	3.77E-04	4.23E-02	Distal Intergenic
Kcnk1	59324	chr19	58937169	58937469	113333	2.41	3.78E-04	4.23E-02	Distal Intergenic
Fzd5	317674	chr9	71537355	71537655	-91814	2.75	3.80E-04	4.25E-02	Distal Intergenic
Abhd2	293050	chr1	140980137	140980437	-17803	-2.42	3.82E-04	4.26E-02	Distal Intergenic
Cd180	294706	chr2	33221808	33222108	401486	-2.42	3.82E-04	4.26E-02	Distal Intergenic
RGD1562146	500612	chr6	9669244	9669544	90624	2.4	3.85E-04	4.28E-02	Intron (NM_017171/29340, intron 1 of 13)
Arf6	79121	chr6	91846485	91846785	149376	1.3	3.86E-04	4.28E-02	Distal Intergenic
Fam53b	309060	chr1	204739237	204739537	66201	-1.76	3.88E-04	4.30E-02	Intron (NM_001107556/309060, intron 4 of 4)
Stx8	59074	chr10	54604105	54604405	91120	-2.61	3.90E-04	4.31E-02	Intron (NM_031656/59074, intron 6 of 7)
Ngdn	305887	chr15	33714297	33714597	36700	2.38	3.93E-04	4.32E-02	Distal Intergenic
Tmem232	501199	chr9	113265360	113265660	-26967	2.68	3.93E-04	4.32E-02	Distal Intergenic
Sec24b	295461	chr2	230503769	230504069	-146935	-2.14	3.95E-04	4.34E-02	Intron (NM_001047887/311013, intron 1 of 1)
Exd2	362759	chr6	103997664	103997964	-19925	-2.13	3.96E-04	4.34E-02	Distal Intergenic
Mfsd11	360667	chr10	105802157	105802457	5477	-2.45	3.97E-04	4.34E-02	Exon (NM_001108308/360667, exon 5 of 13)
Ptgs1	24693	chr3	15571604	15571904	10881	-2.52	4.00E-04	4.36E-02	Intron (NM_017043/24693, intron 7 of 10)
Hdac11	297453	chr4	122769973	122770273	-10822	-2.72	4.01E-04	4.36E-02	Distal Intergenic
LOC499843	499843	chr3	90060493	90060793	690262	2.43	4.01E-04	4.36E-02	Distal Intergenic
Fgd2	309653	chr20	7004605	7004905	31207	-2.61	4.02E-04	4.36E-02	Intron (NM_022666/24417, intron 1 of 8)
Bzw2	171439	chr6	55633232	55633532	14118	-2.44	4.05E-04	4.37E-02	Intron (NM_134402/171439, intron 1 of 11)
Ero1a	171562	chr15	19652692	19652992	2389	-1.72	4.05E-04	4.37E-02	Promoter (2-3kb)
Barhl2	65050	chr14	4251527	4251827	-110890	2.25	4.06E-04	4.37E-02	Distal Intergenic
Lsm3	297455	chr4	123190186	123190486	28100	2.89	4.07E-04	4.37E-02	Distal Intergenic
Nmu	63887	chr14	34384475	34384775	29972	-2.63	4.07E-04	4.37E-02	Distal Intergenic
Emp1	25314	chr4	169232527	169232827	70899	-2.23	4.08E-04	4.37E-02	Distal Intergenic
Mn1	498194	chr12	50875235	50875535	374695	3.14	4.08E-04	4.37E-02	Distal Intergenic
Smim20	501923	chr14	59994259	59994559	75311	-1.81	4.09E-04	4.37E-02	Distal Intergenic
Cd8a	24930	chr4	99290880	99291180	51765	-2.01	4.10E-04	4.37E-02	Distal Intergenic
Dock7	313388	chr5	117787677	117787977	-33106	-2.52	4.11E-04	4.37E-02	Distal Intergenic
Myof	309499	chr1	256705211	256705511	29216	-2.02	4.13E-04	4.39E-02	Intron (NM_001354115/309499, intron 3 of 52)
Polg	85472	chr1	141202002	141202302	-14169	1.07	4.20E-04	4.45E-02	Distal Intergenic
Ldhd	307858	chr19	43870684	43870984	-21747	-2.09	4.22E-04	4.45E-02	Distal Intergenic
Trh	25569	chr4	124328615	124328915	-215373	-2.39	4.22E-04	4.45E-02	Intron (NM_001107876/312563, intron 2 of 7)
Nlrp3	287362	chr10	45903880	45904180	10862	-2.27	4.23E-04	4.45E-02	Intron (NM_001191642/287362, intron 4 of 8)
Trib2	313974	chr6	41026169	41026469	12968	-2.16	4.23E-04	4.45E-02	Intron (NM_001108015/313974, intron 2 of 2)
Cdh1	83502	chr19	38914715	38915015	146248	-2.18	4.25E-04	4.46E-02	Distal Intergenic
Oxr1	117520	chr7	80042967	80043267	-308507	1.34	4.27E-04	4.47E-02	Distal Intergenic
Cap1	64185	chr5	140572495	140572795	12613	-2.14	4.28E-04	4.48E-02	Intron (NM_022383/64185, intron 1 of 12)
Gsr	116686	chr16	62233951	62234251	5736	-2.08	4.30E-04	4.48E-02	Intron (NM_053906/116686, intron 1 of 12)
Arhgap11a	296060	chr3	105293720	105294020	4088	-2.03	4.30E-04	4.48E-02	Intron (NM_001168524/296060, intron 2 of 11)
Lgals9	25476	chr10	64736996	64737296	0	-2.08	4.31E-04	4.48E-02	Promoter (<=1kb)
Lima1	300228	chr7	141489604	141489904	4881	-2.35	4.31E-04	4.48E-02	Intron (NM_001191615/300228, intron 1 of 9)
Srcin1	56029	chr10	85517863	85518163	-199	1.61	4.35E-04	4.50E-02	Promoter (<=1kb)
Mef2a	309957	chr1	128273936	128274236	67027	-2.25	4.35E-04	4.50E-02	Intron (NM_001014035/309957, intron 3 of 10)
Ercc1	292673	chr1	80259934	80260234	2961	-2.4	4.37E-04	4.51E-02	Promoter (2-3kb)
Fbxo36	363268	chr9	92476987	92477287	41115	-2.68	4.40E-04	4.52E-02	Exon (NM_001108804/363268, exon 2 of 4)
Zbtb43	311872	chr3	12489958	12490258	19047	-2.02	4.41E-04	4.52E-02	3' UTR
Gpbp1	294734	chr2	43057989	43058289	10388	-2.37	4.41E-04	4.52E-02	Intron (NM_001106410/294734, intron 1 of 11)
LOC691083	691083	chr3	95245285	95245585	13119	2.33	4.41E-04	4.52E-02	Intron (NM_001144862/691083, intron 1 of 2)
Scaf11	312030	chr7	137847664	137847964	8373	-2.35	4.45E-04	4.55E-02	Intron (NM_001271170/312030, intron 1 of 14)
Olfm1	93667	chr3	6701585	6701885	-71928	-2.27	4.50E-04	4.59E-02	Distal Intergenic
Msn	81521	chrX	65259391	65259691	32557	-2.04	4.52E-04	4.59E-02	Intron (NM_030863/81521, intron 1 of 12)
Plvap	56765	chr16	19928534	19928834	-9866	-2.26	4.52E-04	4.59E-02	Distal Intergenic
Cckbr	25706	chr1	170261886	170262186	-32	-2.28	4.52E-04	4.59E-02	Promoter (<=1kb)
Cd276	315716	chr8	63340728	63341028	9284	-2.01	4.56E-04	4.61E-02	Intron (NM_182824/315716, intron 1 of 7)
Ccl5	81780	chr10	70762548	70762848	-18245	-2.62	4.56E-04	4.61E-02	Distal Intergenic
Ndst3	295430	chr2	227930112	227930412	-40035	-2.24	4.57E-04	4.61E-02	Distal Intergenic
Qdpr	64192	chr14	70206886	70207186	42204	-2.54	4.58E-04	4.61E-02	Distal Intergenic
Tspan3	300733	chr8	60869846	60870146	-39398	-2.4	4.58E-04	4.61E-02	Exon (NM_001108149/315686, exon 7 of 7)
Six4	299138	chr6	95990625	95990925	7604	-2.25	4.63E-04	4.64E-02	Intron (NM_001106739/299138, intron 4 of 4)
Oas1a	192281	chr12	41204284	41204584	3555	-2.41	4.64E-04	4.64E-02	Intron (NM_138913/192281, intron 2 of 5)
Bcar1	25414	chr19	43935648	43935948	19835	-2.41	4.64E-04	4.64E-02	Exon (NM_012931/25414, exon 5 of 7)
Ifitm3	361673	chr1	213835792	213836092	-23971	-2.44	4.67E-04	4.66E-02	Distal Intergenic
Ccdc148	311051	chr3	45169633	45169933	40541	-2.02	4.70E-04	4.69E-02	Intron (NM_001107732/311051, intron 1 of 14)
Sh2b3	58838	chr12	40270946	40271246	8956	2.32	4.72E-04	4.70E-02	Distal Intergenic
Rbpj	679028	chr14	59711097	59711397	24053	-2.1	4.74E-04	4.70E-02	Intron (NM_001106631/679028, intron 2 of 11)
Abhd2	293050	chr1	141033832	141034132	35592	-2.38	4.74E-04	4.70E-02	Intron (NM_001106275/293050, intron 3 of 10)
Spg7	353231	chr19	55821816	55822116	-58445	-1.94	4.75E-04	4.70E-02	Distal Intergenic
RGD1304694	362974	chr7	125899876	125900176	-6708	-2.13	4.76E-04	4.70E-02	Distal Intergenic
Galnt3	366061	chr3	52210037	52210337	-10726	-2.07	4.78E-04	4.71E-02	Distal Intergenic
Srgap3	500287	chr4	144852226	144852526	16599	2.84	4.79E-04	4.72E-02	Intron (NM_001191975/500287, intron 1 of 21)
Btd	306262	chr16	7789073	7789373	28433	-2.43	4.83E-04	4.75E-02	Intron (NM_001012047/306262, intron 3 of 3)
Pfdn1	361310	chr18	29285273	29285573	4874	-2.42	4.84E-04	4.75E-02	Intron (NM_001108427/361310, intron 2 of 3)

Pdpr	307852	chr19	43177253	43177553	-34542	2.76	4.87E-04	4.77E-02	Distal Intergenic
Mir297	100314063	chr10	101776623	101776923	-80285	-2.33	4.89E-04	4.78E-02	Intron (NM_001013042/287796, intron 7 of 9)
Rasgrf1	192213	chr8	97242885	97243185	-34065	-2.32	4.89E-04	4.78E-02	Distal Intergenic
Hk2	25059	chr4	113598241	113598541	11356	-2.42	4.92E-04	4.79E-02	Intron (NM_012735/25059, intron 1 of 17)
Slc4a7	117955	chr15	11990553	11990853	-77637	1.5	4.93E-04	4.79E-02	Distal Intergenic
Mir99a	100314019	chr11	15835875	15836175	-215978	2.69	4.93E-04	4.79E-02	Distal Intergenic
Id3	25585	chr5	154521486	154521786	31883	-2.39	4.97E-04	4.82E-02	Distal Intergenic
Rptor	287871	chr10	108801012	108801312	50392	-2.26	5.08E-04	4.90E-02	Intron (NM_001134499/287871, intron 1 of 33)
Spin1	361217	chr16	1128417	1128717	-197890	-2.3	5.10E-04	4.90E-02	Distal Intergenic
Gpsm1	246254	chr3	3761764	3762064	-5330	2.68	5.10E-04	4.90E-02	Distal Intergenic
Wls	362065	chr2	266261400	266261700	-53336	-2.53	5.11E-04	4.90E-02	Distal Intergenic
Brf2	306542	chr16	69110204	69110504	20249	-2.4	5.11E-04	4.90E-02	Distal Intergenic
Mrps14	289143	chr13	77921968	77922268	-18186	2.69	5.11E-04	4.90E-02	Distal Intergenic
Foxd1	171299	chr2	28493721	28494021	33653	2.79	5.12E-04	4.90E-02	Distal Intergenic
Hand2	64637	chr16	36631995	36632295	-258446	-2.35	5.16E-04	4.92E-02	Distal Intergenic
Ddc	24311	chr14	92045345	92045645	-48571	2.13	5.16E-04	4.92E-02	Intron (NM_001109093/498416, intron 6 of 17)
Atp1a1	24211	chr2	203911699	203911999	120024	-2.21	5.17E-04	4.92E-02	Intron (NM_012830/497761, intron 1 of 6)
Lbx1	499362	chr1	264981689	264981989	-6557	-2.14	5.17E-04	4.92E-02	Distal Intergenic
Tubb6	307351	chr18	63109990	63110290	-20252	2.94	5.20E-04	4.93E-02	Distal Intergenic
Mtss2	307845	chr19	40942523	40942823	-16863	2.74	5.21E-04	4.93E-02	Intron (NM_177930/307842, intron 14 of 18)
Acvrl1	25237	chr7	142719431	142719731	-56521	2.9	5.23E-04	4.94E-02	Distal Intergenic
Dhrs9	170635	chr3	55690574	55690874	66903	-2.31	5.24E-04	4.94E-02	Exon (NM_030827/29216, exon 62 of 79)
Hapln3	308773	chr1	140832738	140833038	2113	-2.36	5.29E-04	4.99E-02	Promoter (2-3kb)
Pdlim4	24915	chr10	39405049	39405349	0	1.41	5.33E-04	5.01E-02	Promoter (<=1kb)
Rps3a	29288	chr2	185283064	185283364	161482	-2.13	5.35E-04	5.02E-02	Distal Intergenic
Guca2a	25656	chr5	138666060	138666360	-19274	2.61	5.38E-04	5.03E-02	Intron (NM_001107971/313554, intron 7 of 12)
Usp12	360763	chr12	10046663	10046963	8977	-2.38	5.38E-04	5.03E-02	Intron (NM_001166576/360763, intron 1 of 8)
Cfap97d1	498004	chr10	89925313	89925613	6940	-2.34	5.38E-04	5.03E-02	Distal Intergenic
Prkcd	170538	chr16	6672150	6672450	3296	-2.44	5.40E-04	5.04E-02	Intron (NM_133307/170538, intron 1 of 18)
Rpa1	287524	chr10	62146573	62146873	38001	-2.49	5.42E-04	5.05E-02	Exon (NM_001047843/287524, exon 15 of 17)
Pitrm1	307081	chr17	68333141	68333441	-144026	-2.36	5.44E-04	5.05E-02	Distal Intergenic
Nod2	291912	chr19	19361222	19361522	15267	-1.76	5.46E-04	5.06E-02	Intron (NM_001106172/291912, intron 3 of 9)
Ca9	313495	chr5	59013382	59013682	4449	-2.38	5.46E-04	5.06E-02	Exon (NM_001107956/313495, exon 7 of 11)
Epha4	316539	chr9	83545134	83545434	-291676	-2.3	5.47E-04	5.06E-02	Distal Intergenic
Tead1	361630	chr1	177515900	177516200	20118	-2.09	5.49E-04	5.06E-02	Intron (NM_001198589/361630, intron 1 of 12)
Fn1	25661	chr9	79122783	79123083	-153770	2.78	5.50E-04	5.06E-02	Distal Intergenic
Galnt10	170501	chr10	43096950	43097250	29634	-2.15	5.51E-04	5.06E-02	Intron (NM_130742/170501, intron 1 of 11)
Cdk6	114483	chr4	27888924	27889224	77174	-2.15	5.51E-04	5.06E-02	Intron (NM_001191861/114483, intron 2 of 6)
Tsku	308843	chr1	163311841	163312141	16450	-2.37	5.53E-04	5.08E-02	Distal Intergenic
Med27	296612	chr3	8072806	8073106	187984	1.78	5.58E-04	5.11E-02	Distal Intergenic
Ppp1r12a	116670	chr7	51499647	51499947	15274	-2.35	5.60E-04	5.12E-02	Intron (NM_053890/116670, intron 1 of 23)
Zpbp2	363676	chr10	86514767	86515067	0	0.91	5.62E-04	5.13E-02	Promoter (<=1kb)
Lpin1	313977	chr6	41787027	41787327	82719	-2.33	5.68E-04	5.16E-02	Distal Intergenic
RGD1563060	291608	chr18	38444518	38444818	-114738	-1.9	5.68E-04	5.16E-02	Distal Intergenic
Il1r1	25663	chr9	47001972	47002272	4174	-2.06	5.68E-04	5.16E-02	Intron (NM_013123/25663, intron 1 of 10)
Capns1	29156	chr1	88671528	88671828	-12731	2.61	5.70E-04	5.16E-02	Distal Intergenic
Tmod3	300838	chr8	82464994	82465294	26949	-2.38	5.70E-04	5.16E-02	Intron (NM_001011997/300838, intron 1 of 9)
Morc3	304074	chr11	34049539	34049839	-2271	-2.11	5.71E-04	5.16E-02	Promoter (2-3kb)
Sp7	300260	chr7	143966831	143967131	0	1.57	5.73E-04	5.16E-02	Promoter (<=1kb)
Snx1	84471	chr8	71771716	71772016	12535	-1.86	5.73E-04	5.16E-02	Intron (NM_053411/84471, intron 1 of 14)
Olr202	365337	chr1	170208125	170208425	-21663	-2.38	5.74E-04	5.16E-02	Distal Intergenic
Gfod1	306842	chr17	23889611	23889911	33778	-1.65	5.74E-04	5.16E-02	Intron (NM_001170334/306842, intron 1 of 1)
Rap2a	114560	chr15	106032281	106032581	180738	-1.63	5.76E-04	5.16E-02	Distal Intergenic
Bcat1	29592	chr4	179148804	179149104	157991	-2.5	5.79E-04	5.17E-02	Distal Intergenic
Tp53i11	311209	chr3	82147329	82147629	65432	2.31	5.79E-04	5.17E-02	Intron (NM_001107750/311210, intron 2 of 9)
Cdc42ep4	303653	chr10	102301663	102301963	-32391	-2.07	5.82E-04	5.19E-02	Downstream (1-2kb)
MGC105567	498873	chr18	55685802	55686102	189	-1.86	5.84E-04	5.20E-02	Promoter (<=1kb)
Lemd3	680066	chr7	62676584	62676884	368844	-1.94	5.88E-04	5.22E-02	Distal Intergenic
Tab2	308267	chr1	2163081	2163381	-89185	2.64	5.88E-04	5.22E-02	Distal Intergenic
Slc30a3	366568	chr6	26649172	26649472	6342	1.61	5.89E-04	5.22E-02	3' UTR
Kmt2e	311968	chr4	8243097	8243397	12181	-2.29	5.91E-04	5.23E-02	Intron (NM_001100851/311968, intron 1 of 26)
Arx	317268	chrX	62366627	62366927	2870	2.54	5.95E-04	5.25E-02	Promoter (2-3kb)
Fam8a1	291031	chr17	18401352	18401652	20310	-2.08	5.96E-04	5.25E-02	Intron (NM_001100470/25281, intron 16 of 21)
Zdhhc5	362156	chr3	72113225	72113525	155	-2.27	5.98E-04	5.27E-02	Promoter (<=1kb)
Adrb3	25645	chr16	69004245	69004545	377	1.06	6.02E-04	5.28E-02	Promoter (<=1kb)
Ifitm3	361673	chr1	213813241	213813541	-1420	-2.04	6.02E-04	5.28E-02	Promoter (1-2kb)
Sema3d	246262	chr4	19412593	19412893	626	0.84	6.06E-04	5.31E-02	Promoter (<=1kb)
Mapk1	116590	chr11	88254771	88255071	18223	-2.39	6.07E-04	5.31E-02	Intron (NM_053842/116590, intron 1 of 8)
Lgals3bp	245955	chr10	107420792	107421092	3577	-2.08	6.12E-04	5.34E-02	Exon (NM_139096/245955, exon 3 of 6)
Plvap	56765	chr16	19914155	19914455	4213	-1.88	6.12E-04	5.34E-02	Exon (NM_020086/56765, exon 3 of 6)
LOC499643	499643	chr2	178212698	178212998	95215	-2.5	6.16E-04	5.35E-02	Intron (NM_001024301/499643, intron 3 of 4)
Rex1bd	306348	chr16	20675354	20675654	2980	1.35	6.17E-04	5.35E-02	Promoter (2-3kb)
Perp	292949	chr1	14359081	14359381	134689	-1.95	6.17E-04	5.35E-02	Distal Intergenic
Bcl2l14	500348	chr4	167953002	167953302	-186706	-2.4	6.17E-04	5.35E-02	Intron (NM_001037353/312777, intron 3 of 7)
Tgif1	316742	chr9	119122306	119122606	68092	2.66	6.22E-04	5.38E-02	Intron (NM_022946/65040, intron 9 of 12)
RGD1304587	303180	chr10	45568422	45568722	8844	-1.71	6.24E-04	5.39E-02	Intron (NM_022518/64310, intron 1 of 4)
Pdhx	311254	chr3	92889107	92889407	44318	-2.21	6.34E-04	5.46E-02	Distal Intergenic
Bclaf1	293017	chr1	15806692	15806992	6839	-1.92	6.34E-04	5.46E-02	Intron (NM_001047852/293017, intron 2 of 12)
Aldh1l2	299699	chr7	26375824	26376124	0	1.2	6.43E-04	5.53E-02	Promoter (<=1kb)
Optn	246294	chr17	77165600	77165900	-10805	-1.92	6.45E-04	5.53E-02	Distal Intergenic
Crybb3	64349	chr12	49483935	49484235	-81757	2.95	6.45E-04	5.53E-02	Distal Intergenic
Rhox5	24631	chrX	123914989	123915289	1917	-2.01	6.46E-04	5.53E-02	Promoter (1-2kb)
Mir101-2	100313988	chr1	247312972	247313272	49249	-2.24	6.53E-04	5.58E-02	Distal Intergenic
Pdgfb	24628	chr7	121232917	121233217	0	1.67	6.55E-04	5.59E-02	Promoter (<=1kb)
Myc	24577	chr7	103027005	103027305	440692	-2.3	6.56E-04	5.59E-02	Distal Intergenic
Gpr3	266769	chr5	151352559	151352859	43648	-2.25	6.57E-04	5.59E-02	Intron (NM_001013167/313024, intron 1 of 13)
Pmvk	310645	chr2	188807618	188807918	23289	-1.84	6.58E-04	5.59E-02	Distal Intergenic
Adamts4	66015	chr13	89625831	89626131	2835	-2.32	6.61E-04	5.61E-02	Promoter (2-3kb)
Pmp22	24660	chr10	49546233	49546533	7645	-2.16	6.63E-04	5.61E-02	Intron (NM_017037/24660, intron 3 of 4)
Nrbf2	58839	chr20	22800181	22800481	71973	-1.55	6.69E-04	5.66E-02	Intron (NM_001191719/171120, intron 3 of 25)
Dennd2b	308944	chr1	174226574	174226874	62773	2.6	6.72E-04	5.68E-02	Intron (NM_001107547/308944, intron 2 of 19)
Tagln3	63837	chr11	57706058	57706358	200374	-1.83	6.74E-04	5.68E-02	Distal Intergenic
Adcy5	64532	chr11	69030869	69031169	-188549	-2.14	6.75E-04	5.68E-02	Intron (NM_001105874/288057, intron 27 of 31)
Papola	314417	chr6	129619658	129619958	10584	-1.96	6.77E-04	5.68E-02	Intron (NM_001108056/314417, intron 1 of 21)

Afg1l	502479	chr20	46965138	46965438	243374	-2.33	6.78E-04	5.68E-02	Distal Intergenic
Nubpl	299008	chr6	73053664	73053964	161906	-2.21	6.80E-04	5.70E-02	Intron (NM_001185025/299008, intron 6 of 10)
Upb1	116593	chr20	14161021	14161321	32403	-1.35	6.83E-04	5.71E-02	Distal Intergenic
Raly	296301	chr3	150218642	150218942	-104282	-2.59	6.87E-04	5.74E-02	Distal Intergenic
Cebpb	24253	chr3	164511655	164511955	87153	1.6	6.88E-04	5.74E-02	Distal Intergenic
Parva	57341	chr1	177320001	177320301	70699	-2.03	6.90E-04	5.74E-02	Intron (NM_020656/57341, intron 1 of 12)
Egfr	24329	chr14	99893094	99893394	-26091	-2.25	6.93E-04	5.76E-02	Distal Intergenic
Sh3pxd2a	309460	chr1	267155418	267155718	48268	-1.75	6.94E-04	5.76E-02	Intron (NM_001107606/309460, intron 2 of 13)
Zfp362	297879	chr5	146985656	146985956	-11724	1.65	6.95E-04	5.76E-02	Distal Intergenic
Capza2	493810	chr4	44920381	44920681	-15609	-2.27	6.97E-04	5.77E-02	Distal Intergenic
Aspg	246266	chr6	136631052	136631352	-50927	1.45	6.98E-04	5.77E-02	Distal Intergenic
Lamtor3	362045	chr2	243163991	243164291	3074	-2.08	7.00E-04	5.77E-02	Intron (NM_001008375/362045, intron 3 of 6)
Xrcc5	363247	chr9	79784396	79784696	125121	-2.19	7.09E-04	5.84E-02	Distal Intergenic
Tcp11	309641	chr20	7678284	7678584	-23508	-2.57	7.12E-04	5.84E-02	Distal Intergenic
Lamc1	117036	chr13	70774067	70774367	9148	-2.31	7.15E-04	5.84E-02	Intron (NM_053966/117036, intron 1 of 27)
Rassf4	362423	chr4	148800062	148800362	3626	-2.65	7.16E-04	5.84E-02	Intron (NM_001024275/362423, intron 1 of 10)
Tmem204	287129	chr10	14444623	14444923	-1613	1.29	7.16E-04	5.84E-02	Promoter (1-2kb)
Nt5c3a	312373	chr4	87266476	87266776	14359	-2.45	7.17E-04	5.84E-02	Intron (NM_001107862/312373, intron 1 of 9)
Fgf5	60662	chr14	13146820	13147120	-151236	-1.71	7.17E-04	5.84E-02	Distal Intergenic
Mepe	79110	chr14	6740313	6740613	52948	-2.31	7.19E-04	5.84E-02	Distal Intergenic
Castor2	304410	chr12	25618343	25618643	20163	2.26	7.19E-04	5.84E-02	Intron (NM_001100561/304410, intron 1 of 8)
Arsi	307404	chr18	56162032	56162332	-17757	-1.93	7.20E-04	5.84E-02	Distal Intergenic
Dusp6	116663	chr7	41667124	41667424	191961	-1.84	7.20E-04	5.84E-02	Distal Intergenic
Camk1g	171358	chr13	112144882	112145182	-45546	-2.27	7.23E-04	5.84E-02	Distal Intergenic
Apol3	315108	chr7	118686390	118686690	1373	-2.34	7.24E-04	5.84E-02	Promoter (1-2kb)
Sipa1l1	246212	chr6	105912325	105912625	-139587	-2.37	7.25E-04	5.84E-02	Distal Intergenic
Fahd2a	296131	chr3	120027864	120028164	-16490	-2.32	7.25E-04	5.84E-02	Intron (NM_032462/65199, intron 5 of 8)
Prdm2	313678	chr5	161808605	161808905	70228	-2.01	7.26E-04	5.84E-02	Intron (NM_001077648/313678, intron 6 of 9)
Dapk2	300799	chr8	71988937	71989237	74168	-2.21	7.27E-04	5.84E-02	Distal Intergenic
LOC691083	691083	chr3	95241336	95241636	9170	2.23	7.28E-04	5.84E-02	Intron (NM_001144862/691083, intron 1 of 2)
Tab2	308267	chr1	2164413	2164713	-90517	2.57	7.29E-04	5.84E-02	Distal Intergenic
Adamts15	300474	chr8	32000114	32000414	0	2.4	7.29E-04	5.84E-02	Promoter (<=1kb)
Stim2	117087	chr14	59365041	59365341	-41094	-2.17	7.32E-04	5.84E-02	Distal Intergenic
Uqcc2	361805	chr20	5754427	5754727	-30525	-2.27	7.34E-04	5.84E-02	Distal Intergenic
Lhfpl2	294643	chr2	23611652	23611952	-158756	-1.97	7.35E-04	5.84E-02	Distal Intergenic
Bcl2l11	64547	chr3	120887217	120887517	160311	-2.17	7.35E-04	5.84E-02	Distal Intergenic
Lrp12	314941	chr7	78954662	78954962	-23830	-2	7.36E-04	5.84E-02	Distal Intergenic
Cadm3	360882	chr13	91921733	91922033	-48780	-1.69	7.36E-04	5.84E-02	Distal Intergenic
LOC498592	498592	chr7	91181933	91182233	66359	-1.99	7.37E-04	5.84E-02	Distal Intergenic
Kiaa0895l	688736	chr19	37247043	37247343	-1826	2.51	7.37E-04	5.84E-02	Promoter (1-2kb)
Sulf2	311642	chr3	162779203	162779503	93328	-2.19	7.39E-04	5.85E-02	Distal Intergenic
Tlr12	362604	chr5	146784878	146785178	10185	-1.92	7.41E-04	5.85E-02	Distal Intergenic
Rgs17	308118	chr1	42686086	42686386	-98365	-2.33	7.44E-04	5.86E-02	Distal Intergenic
Hcn4	59266	chr8	63744004	63744304	144097	-2.33	7.44E-04	5.86E-02	Distal Intergenic
Kcnt1	60444	chr3	3313620	3313920	-11850	-1.96	7.50E-04	5.89E-02	Distal Intergenic
Slfn2	303380	chr10	70379909	70380209	9528	-2.25	7.54E-04	5.92E-02	Distal Intergenic
Aqp1	25240	chr4	85486451	85486751	-64752	-2.14	7.56E-04	5.93E-02	Distal Intergenic
Fgf2	54250	chr2	124092533	124092833	11461	-1.77	7.61E-04	5.96E-02	Intron (NM_019305/54250, intron 1 of 2)
Bcor	317346	chrX	12014691	12014991	365702	-2.22	7.63E-04	5.96E-02	Distal Intergenic
Efr3a	362923	chr7	106541338	106541638	5904	-2.47	7.63E-04	5.96E-02	Intron (NM_001130564/362923, intron 1 of 22)
Myo1e	25484	chr8	76688784	76689084	44069	-2.35	7.64E-04	5.96E-02	Intron (NM_173101/25484, intron 1 of 26)
Scarb2	117106	chr14	17081452	17081752	17279	-1.95	7.70E-04	5.99E-02	Intron (NM_054001/117106, intron 1 of 11)
Kif3b	296284	chr3	148940043	148940343	166784	1.16	7.72E-04	5.99E-02	Distal Intergenic
RGD1562310	498188	chr12	43781818	43782118	158680	2.66	7.72E-04	5.99E-02	Distal Intergenic
Hspa4	266759	chr10	38635692	38635992	6405	-2.29	7.73E-04	5.99E-02	Intron (NM_153629/266759, intron 1 of 18)
Ccl2	24770	chr10	69383074	69383374	-28691	-2.32	7.73E-04	5.99E-02	Distal Intergenic
Ppp2r5c	691318	chr6	134887085	134887385	42426	2.34	7.76E-04	5.99E-02	Intron (NM_001191112/691318, intron 2 of 13)
Pogk	304941	chr13	84628669	84628969	-9627	-2.25	7.76E-04	5.99E-02	Distal Intergenic
Smpd3	94338	chr19	38293506	38293806	27722	-2.17	7.78E-04	5.99E-02	Intron (NM_053605/94338, intron 1 of 8)
RGD1562310	498188	chr12	43751379	43751679	189119	-1.61	7.79E-04	6.00E-02	Distal Intergenic
Irf1	24508	chr10	39137693	39137993	28163	-2.36	7.88E-04	6.05E-02	Distal Intergenic
Il1a	24493	chr3	121829520	121829820	6302	-2.36	7.88E-04	6.05E-02	Exon (NM_017019/24493, exon 7 of 9)
Fbxl14	312675	chr4	151967380	151967680	-19213	-1.66	7.89E-04	6.05E-02	Distal Intergenic
Klrk1	24934	chr4	163402808	163403108	545	-2.13	7.94E-04	6.07E-02	Promoter (<=1kb)
Rem1	366232	chr3	148110929	148111229	2377	-2.39	7.96E-04	6.07E-02	Promoter (2-3kb)
Slc16a1	25027	chr2	207157805	207158105	49253	-2.21	7.96E-04	6.07E-02	Distal Intergenic
Pfkm	65152	chr7	139681199	139681499	-16649	-2.25	7.97E-04	6.07E-02	Distal Intergenic
Reep3	294375	chr20	22913489	22913789	0	1.6	8.01E-04	6.08E-02	Promoter (<=1kb)
Lmcd1	494021	chr4	144175973	144176273	-16716	-1.89	8.01E-04	6.08E-02	Distal Intergenic
Pstpip2	307248	chr18	74170430	74170730	-4181	-1.84	8.01E-04	6.08E-02	Distal Intergenic
Krt71	683613	chr7	143328213	143328513	25473	2.13	8.02E-04	6.08E-02	Intron (NM_183333/369017, intron 1 of 8)
LOC500846	500846	chr7	69539233	69539533	-434283	-2.52	8.08E-04	6.10E-02	Distal Intergenic
Htra3	360959	chr14	80256625	80256925	8485	2.71	8.09E-04	6.10E-02	Intron (NM_001271027/360959, intron 2 of 8)
Fbxw9	288921	chr19	26176552	26176852	2781	2.5	8.10E-04	6.10E-02	Promoter (2-3kb)
Cyp19a1	25147	chr8	58734751	58735051	37357	-2.05	8.10E-04	6.10E-02	Distal Intergenic
Magi1	500261	chr4	126386051	126386351	-86641	-2.13	8.12E-04	6.10E-02	Distal Intergenic
Gprc5b	293546	chr1	188699641	188699941	13339	-2.39	8.13E-04	6.10E-02	Intron (NM_001106304/293546, intron 2 of 3)
Sh3bp4	64634	chr9	96206441	96206741	-4009	-2.39	8.13E-04	6.10E-02	Distal Intergenic
Tnip1	363599	chr10	40289149	40289449	13623	-2.28	8.14E-04	6.10E-02	Intron (NM_001108826/363599, intron 2 of 18)
Pom121l2	502128	chr17	44674666	44674966	-79343	1	8.19E-04	6.13E-02	Distal Intergenic
Hadha	170670	chr6	27601266	27601566	11426	-2.06	8.20E-04	6.14E-02	Exon (NM_130826/170670, exon 3 of 20)
Vgf	29461	chr12	22673993	22674293	6337	-1.72	8.29E-04	6.18E-02	Intron (NM_001108331/360785, intron 4 of 4)
Tns1	301509	chr9	81313137	81313437	87550	0.8	8.30E-04	6.18E-02	Intron (NM_001191810/301509, intron 3 of 34)
Kcnn4	65206	chr1	81234339	81234639	3492	-1.99	8.30E-04	6.18E-02	Intron (NM_001270701/65206, intron 1 of 8)
Rasgrp3	313874	chr6	21129409	21129709	6171	2.64	8.31E-04	6.18E-02	Intron (NM_001108009/313874, intron 1 of 15)
Lca5	300866	chr8	91007952	91008252	-23728	-2.78	8.36E-04	6.21E-02	Distal Intergenic
Slc4a7	117955	chr15	12014573	12014873	-101657	-1.82	8.37E-04	6.21E-02	Distal Intergenic
Mir664-1	100314170	chr18	47528024	47528324	39996	1.44	8.43E-04	6.25E-02	Intron (NM_017061/24914, intron 2 of 8)
Lancl3	302540	chrX	14331178	14331478	-26746	1.24	8.49E-04	6.28E-02	Distal Intergenic
Phc2	313038	chr5	146889953	146890253	7197	-1.99	8.56E-04	6.30E-02	Exon (NM_001013169/313038, exon 5 of 14)
Adcy7	84420	chr19	19735783	19736083	-8702	-1.53	8.57E-04	6.30E-02	Distal Intergenic
Sec16b	89868	chr13	75158355	75158655	-16599	1.21	8.57E-04	6.30E-02	Distal Intergenic
Rbpj	679028	chr14	59716158	59716458	18992	-2.21	8.58E-04	6.30E-02	Intron (NM_001106631/679028, intron 2 of 11)
Slco4a1	171144	chr3	175825271	175825571	-13423	-2.58	8.58E-04	6.30E-02	Distal Intergenic

Egln3	54702	chr6	75069630	75069930	5865	-2.25	8.60E-04	6.30E-02	Intron (NM_019371/54702, intron 1 of 4)
Ube2f	363284	chr9	98355782	98356082	-38086	2.22	8.60E-04	6.30E-02	Intron (NM_031645/58965, intron 2 of 2)
Aptr	104968332	chr4	10761557	10761857	-12266	-1.8	8.63E-04	6.32E-02	Intron (NM_001135872/311987, intron 1 of 7)
Slc16a4	295356	chr2	210080420	210080720	35127	-1.92	8.66E-04	6.33E-02	Distal Intergenic
Apaf1	78963	chr7	31784173	31784473	0	1.08	8.68E-04	6.33E-02	Promoter (<=1kb)
Rpap3	300189	chr7	139196079	139196379	-1049	0.94	8.68E-04	6.33E-02	Promoter (1-2kb)
Lamb3	305078	chr13	112026955	112027255	-4402	-2.04	8.72E-04	6.35E-02	Distal Intergenic
Armc1	294948	chr2	103979295	103979595	2	0.94	8.74E-04	6.35E-02	Promoter (<=1kb)
Plekhb2	301337	chr9	41611220	41611520	262773	-1.77	8.75E-04	6.35E-02	Distal Intergenic
Npas2	316351	chr9	45937746	45938046	36005	-2.31	8.78E-04	6.36E-02	Intron (NM_001108214/316351, intron 1 of 20)
Rpgrip1l	307724	chr19	17163721	17164021	48309	2.76	8.78E-04	6.36E-02	Exon (NM_001107414/307724, exon 16 of 26)
Bcat1	29592	chr4	179263974	179264274	42821	-1.9	8.86E-04	6.40E-02	Intron (NM_017253/29592, intron 10 of 10)
Pik3ca	170911	chr2	118795575	118795875	-35475	-1.93	8.86E-04	6.40E-02	Distal Intergenic
Brap	687346	chr12	40351168	40351468	66296	-1.85	8.90E-04	6.42E-02	Distal Intergenic
Psma6	29673	chr6	76184253	76184553	-3533	-1.91	8.97E-04	6.44E-02	Distal Intergenic
Lrrc49	300763	chr8	65450637	65450937	136490	-2.5	8.97E-04	6.44E-02	Distal Intergenic
Ly6e	362934	chr7	116361118	116361418	4949	-1.92	8.97E-04	6.44E-02	Distal Intergenic
Mkln1	83536	chr4	58531214	58531514	-161870	-1.8	8.98E-04	6.44E-02	Distal Intergenic
Ddi2	313668	chr5	160277016	160277316	5494	-1.98	8.99E-04	6.44E-02	Intron (NM_001034149/313668, intron 1 of 9)
Runx2	367218	chr9	18660266	18660566	16696	-2.08	9.02E-04	6.46E-02	Intron (NM_001278483/367218, intron 4 of 8)
Uqcc2	361805	chr20	5753130	5753430	-29228	-2.59	9.03E-04	6.46E-02	Distal Intergenic
Mto1	300852	chr8	85799056	85799356	-8685	-2.28	9.07E-04	6.47E-02	Distal Intergenic
Cul1	362356	chr4	76687051	76687351	-524463	2.87	9.10E-04	6.47E-02	Distal Intergenic
RGD1562146	500612	chr6	9627517	9627817	132351	2.79	9.11E-04	6.47E-02	Intron (NM_017171/29340, intron 1 of 13)
Kcnc2	246153	chr7	55179525	55179825	199362	-2.11	9.12E-04	6.47E-02	Distal Intergenic
Arrdc3	309945	chr2	8812416	8812716	79249	0.94	9.12E-04	6.47E-02	Distal Intergenic
R3hdm1	304763	chr13	44847902	44848202	35335	-2.1	9.13E-04	6.47E-02	Intron (NM_001134867/304763, intron 1 of 26)
Pik3ca	170911	chr2	118812698	118812998	-18352	-2.15	9.14E-04	6.48E-02	Distal Intergenic
Neurl1b	303019	chr10	17122552	17122852	-47413	2.76	9.19E-04	6.50E-02	Distal Intergenic
Mlph	316620	chr9	98003343	98003643	-69395	0.78	9.21E-04	6.51E-02	Distal Intergenic
Tcf20	366964	chr7	123734077	123734377	33420	-2.2	9.23E-04	6.51E-02	Intron (NM_001130574/366964, intron 1 of 4)
LOC100911360	100911360	chr8	73863749	73864049	-328754	-2.03	9.24E-04	6.51E-02	Distal Intergenic
Ap4s1	366618	chr6	72439869	72440169	-21808	-2.23	9.25E-04	6.51E-02	Intron (NM_001029897/114520, intron 1 of 17)
Slc25a37	306000	chr15	51144015	51144315	24069	-2.27	9.31E-04	6.54E-02	Intron (NM_001013996/306000, intron 1 of 3)
Psrc1	691380	chr2	211189683	211189983	13057	1.76	9.32E-04	6.54E-02	Exon (NM_001191110/83465, exon 19 of 34)
Lyrm9	497962	chr10	66122369	66122669	22838	-2.16	9.32E-04	6.54E-02	Distal Intergenic
Vom2r77	690227	chr9	7905087	7905387	-89943	-1.77	9.35E-04	6.55E-02	Distal Intergenic
Ptpn2	117063	chr18	63452693	63452993	35034	-1.93	9.37E-04	6.55E-02	Intron (NM_053990/117063, intron 2 of 9)
Grhl2	299979	chr7	75945431	75945731	-113720	1.28	9.38E-04	6.55E-02	Distal Intergenic
Zfp709l1	690419	chr7	10364253	10364553	72164	1.87	9.40E-04	6.56E-02	Distal Intergenic
Yy1	24919	chr6	132715294	132715594	12713	-1.89	9.41E-04	6.56E-02	Intron (NM_173290/24919, intron 1 of 4)
Itgb1bp1	298914	chr6	43249228	43249528	113807	-2.13	9.44E-04	6.57E-02	Distal Intergenic
Ttc7b	362768	chr6	124509716	124510016	-75652	1.75	9.47E-04	6.58E-02	Distal Intergenic
Ndst3	295430	chr2	227931227	227931527	-41150	-2.17	9.47E-04	6.58E-02	Distal Intergenic
Flrt2	299236	chr6	119387211	119387511	-132203	-2.11	9.55E-04	6.62E-02	Distal Intergenic
Mdm1	314859	chr7	61143147	61143447	-30922	-2.01	9.56E-04	6.62E-02	Distal Intergenic
Mir31	100314232	chr5	107178225	107178525	-27990	1.12	9.58E-04	6.63E-02	Distal Intergenic
Fam214b	298201	chr5	58487830	58488130	-2930	2.56	9.62E-04	6.64E-02	Promoter (2-3kb)
Polr3b	362858	chr7	24855100	24855400	-3873	2.85	9.64E-04	6.64E-02	Distal Intergenic
LOC500846	500846	chr7	69499459	69499759	-394509	-2.33	9.64E-04	6.64E-02	Distal Intergenic
Adora2b	29316	chr10	48453430	48453730	-115836	-1.74	9.66E-04	6.65E-02	Distal Intergenic
Itgb5	257645	chr11	70309301	70309601	-137137	2.18	9.69E-04	6.65E-02	Distal Intergenic
Mycn	298894	chr6	38228311	38228611	0	1.04	9.69E-04	6.65E-02	Promoter (<=1kb)
Nupl2	499974	chr4	7480066	7480366	48740	-2.01	9.71E-04	6.66E-02	Distal Intergenic
Cpg1	299247	chr6	123262932	123263232	228628	2.17	9.74E-04	6.67E-02	Intron (NM_001108047/314374, intron 5 of 6)
Oat	64313	chr1	204443211	204443511	138559	-2.02	9.76E-04	6.67E-02	Distal Intergenic
Adm2	399475	chr7	130297134	130297434	239	1.93	9.80E-04	6.69E-02	Promoter (<=1kb)
Adamtsl3	308787	chr1	144317493	144317793	78535	2.65	9.81E-04	6.69E-02	Intron (NM_001107533/308787, intron 3 of 29)
Stk38l	691337	chr4	181003096	181003396	-23816	-1.57	9.87E-04	6.72E-02	Distal Intergenic
Septin9	83788	chr10	106274874	106275174	-31859	-2.27	9.92E-04	6.74E-02	Intron (NM_176856/83788, intron 3 of 7)
Thy1	24832	chr8	48328434	48328734	-53387	-2.27	9.92E-04	6.74E-02	Distal Intergenic
Atoh1	500156	chr4	95457636	95457936	-40067	2.75	9.93E-04	6.74E-02	Distal Intergenic
Nrbp2	680451	chr7	117177944	117178244	-26250	-2.21	9.94E-04	6.74E-02	Distal Intergenic
Notch1	25496	chr3	3947628	3947928	3087	-2	9.97E-04	6.75E-02	Intron (NM_001105721/25496, intron 2 of 33)
Mir365b	100314252	chr10	67180383	67180683	100587	2.78	1.00E-03	6.76E-02	Distal Intergenic
Vom2r80	502285	chr1	62134218	62134518	-277520	1.12	1.00E-03	6.76E-02	Distal Intergenic
Hmg20a	315689	chr8	61120515	61120815	40412	-2.13	1.00E-03	6.78E-02	Intron (NM_001108150/315689, intron 1 of 9)
Ppp1cc	24669	chr12	39850691	39850991	31039	-2.03	1.01E-03	6.80E-02	Distal Intergenic
Myo1d	25485	chr10	68096992	68097292	45572	-2.24	1.01E-03	6.82E-02	Intron (NM_012983/25485, intron 1 of 21)
Nab1	64824	chr9	53913024	53913324	6951	-2.27	1.02E-03	6.83E-02	Intron (NM_022856/64824, intron 1 of 7)
Emc1	362643	chr5	157824360	157824660	3452	-2.04	1.02E-03	6.85E-02	Intron (NM_001108690/362643, intron 4 of 22)
Arsj	311013	chr2	230029693	230029993	-133021	-2.29	1.02E-03	6.85E-02	Distal Intergenic
Cdk17	314743	chr7	34042847	34043147	41341	-2.25	1.02E-03	6.86E-02	Intron (NM_001108082/314743, intron 1 of 16)
Tacc2	309025	chr1	201170135	201170435	29082	-2.16	1.03E-03	6.87E-02	Intron (NM_001004415/309025, intron 3 of 21)
Dennd2b	308944	chr1	174253419	174253719	35928	-2.26	1.03E-03	6.87E-02	Intron (NM_001107547/308944, intron 1 of 19)
Eea1	314764	chr7	37144467	37144767	43053	-2.15	1.03E-03	6.89E-02	Intron (NM_001108086/314764, intron 12 of 27)
Tfap2a	306862	chr17	24661572	24661872	6670	-2.66	1.03E-03	6.89E-02	Exon (NM_001107345/306862, exon 4 of 10)
Mtus1	306487	chr16	54384182	54384482	51473	2.6	1.04E-03	6.91E-02	3' UTR
Cdc42ep1	315121	chr7	120056313	120056613	-10939	2.55	1.04E-03	6.91E-02	Distal Intergenic
Abhd2	293050	chr1	141030149	141030449	31909	-2.24	1.05E-03	6.98E-02	Intron (NM_001106275/293050, intron 3 of 10)
Rbpj	679028	chr14	59840228	59840528	-104778	-2	1.05E-03	6.98E-02	Distal Intergenic
Psmd8	292766	chr1	88126502	88126802	-2859	1.64	1.05E-03	6.98E-02	Promoter (2-3kb)
Klhl42	500367	chr4	181536361	181536661	55214	-2.08	1.05E-03	6.98E-02	Distal Intergenic
Mgst2	295037	chr2	140719434	140719734	11036	-1.79	1.06E-03	6.98E-02	Intron (NM_001106430/295037, intron 1 of 3)
Arhgap18	293947	chr1	19432840	19433140	-56539	-2.12	1.06E-03	6.99E-02	Distal Intergenic
Prrx2	113931	chr3	9701770	9702070	19899	1.35	1.07E-03	7.02E-02	Intron (NM_001105739/113931, intron 1 of 3)
Pla2g4a	24653	chr13	67089845	67090145	116543	-2.16	1.07E-03	7.04E-02	Intron (NM_133551/24653, intron 14 of 17)
Sag	25539	chr9	95030826	95031126	103760	-2.23	1.07E-03	7.05E-02	Distal Intergenic
Hexim1	498008	chr10	91199784	91200084	-790	0.73	1.07E-03	7.05E-02	Promoter (<=1kb)
Hif1a	29560	chr6	96700365	96700665	-110520	-2.23	1.07E-03	7.05E-02	Distal Intergenic
Hs3st6	684979	chr10	14122952	14123252	74	2.28	1.08E-03	7.06E-02	Promoter (<=1kb)
Fn1	25661	chr9	78951579	78951879	17134	-1.6	1.08E-03	7.07E-02	Intron (NM_019143/25661, intron 12 of 45)
Slc38a1	170567	chr7	137986262	137986562	53068	-2.16	1.08E-03	7.08E-02	Intron (NM_138832/170567, intron 7 of 15)
Bdh2	295458	chr2	240460598	240460898	-607	2.16	1.09E-03	7.14E-02	Promoter (<=1kb)

Aida	682999	chr13	101721223	101721523	23127	-2.2	1.09E-03	7.14E-02	Intron (NM_001127600/682999, intron 8 of 9)
Nr4a1	79240	chr7	142899426	142899726	-12583	1.02	1.10E-03	7.16E-02	Distal Intergenic
Mir674	100314115	chr3	108872462	108872762	8000	2.46	1.10E-03	7.16E-02	Distal Intergenic
Hook3	306548	chr16	70710005	70710305	-42	1.47	1.10E-03	7.16E-02	Promoter (<=1kb)
Tubgcp3	306599	chr16	82184414	82184714	12	0.95	1.10E-03	7.17E-02	Promoter (<=1kb)
Mir3084a	104795674	chr19	43438453	43438753	35350	-2.31	1.11E-03	7.19E-02	Distal Intergenic
Lbp	29469	chr3	154782651	154782951	-3281	-2.31	1.11E-03	7.19E-02	Distal Intergenic
Pou3f1	192110	chr5	142488717	142489017	-154582	-2.31	1.11E-03	7.19E-02	Distal Intergenic
Acss3	314800	chr7	49046103	49046403	204550	-2.01	1.12E-03	7.22E-02	Distal Intergenic
Zc3h12a	313587	chr5	143112664	143112964	7167	-1.85	1.12E-03	7.24E-02	Exon (NM_001077671/313587, exon 6 of 6)
Slc2a1	24778	chr5	138171814	138172114	17134	-2.14	1.12E-03	7.24E-02	Intron (NM_138827/24778, intron 2 of 9)
Synpo2	499702	chr2	227353058	227353358	58566	-1.82	1.12E-03	7.24E-02	Intron (NM_001191963/499702, intron 1 of 4)
Ier3	294235	chr20	3476649	3476949	-36647	-1.47	1.13E-03	7.24E-02	Distal Intergenic
Bmp6	25644	chr17	27162959	27163259	-50139	-2.52	1.13E-03	7.25E-02	Distal Intergenic
Niban1	63912	chr13	69035326	69035626	85661	-2.63	1.13E-03	7.25E-02	Intron (NM_022242/63912, intron 5 of 13)
Eif4b	300253	chr7	143694542	143694842	14886	-1.76	1.13E-03	7.25E-02	Intron (NM_001008324/300253, intron 9 of 14)
Slc6a7	117100	chr18	56298208	56298508	26671	2.06	1.14E-03	7.29E-02	Distal Intergenic
Chek2	114212	chr12	51693963	51694263	183361	0.91	1.14E-03	7.29E-02	Distal Intergenic
Pelp1	360552	chr10	56998464	56998764	6347	-2.29	1.14E-03	7.30E-02	Intron (NM_001024270/360552, intron 3 of 16)
Mical3	362427	chr4	153567507	153567807	64179	-1.8	1.15E-03	7.32E-02	Intron (NM_001191085/362427, intron 17 of 33)
Pnrc1	286988	chr5	48527959	48528259	-23448	-2.43	1.15E-03	7.32E-02	Distal Intergenic
LOC498154	498154	chr12	17285176	17285476	7541	-1.74	1.15E-03	7.32E-02	Intron (NM_001025033/498154, intron 2 of 4)
Mn1	498194	chr12	51002006	51002306	247924	1.78	1.15E-03	7.32E-02	Distal Intergenic
Hmga2	84017	chr7	65436854	65437154	-161446	-2.28	1.16E-03	7.34E-02	Distal Intergenic
LOC100909416	100909416	chr8	79382480	79382780	-36623	-1.75	1.16E-03	7.34E-02	Exon (NM_012986/25489, exon 12 of 29)
Krt23	287678	chr10	87394471	87394771	12863	-2.28	1.16E-03	7.34E-02	Exon (NM_001008753/287678, exon 6 of 8)
Tial1	361655	chr1	199870235	199870535	-4687	-2.3	1.16E-03	7.34E-02	Distal Intergenic
Otulinl	310190	chr2	80717944	80718244	-309272	-2	1.17E-03	7.41E-02	Distal Intergenic
Olr1768	293878	chrX	156154772	156155072	-54930	1.26	1.18E-03	7.43E-02	Distal Intergenic
Ihh	84399	chr9	82246038	82246338	-31598	-2.1	1.18E-03	7.44E-02	Intron (NM_001014217/363251, intron 5 of 7)
Adamts9	312566	chr4	124857848	124858148	102	0.94	1.19E-03	7.48E-02	Promoter (<=1kb)
Zmat3	64394	chr2	118785062	118785362	-38953	1.54	1.19E-03	7.51E-02	Distal Intergenic
Phlpp1	59265	chr13	26272519	26272819	100304	2.3	1.19E-03	7.51E-02	Intron (NM_021657/59265, intron 1 of 16)
Rsph6a	292684	chr1	79989110	79989410	29519	1.19	1.20E-03	7.51E-02	Distal Intergenic
Zap70	301348	chr9	43423115	43423415	91966	-1.85	1.20E-03	7.51E-02	Distal Intergenic
Elk3	362871	chr7	34158881	34159181	-37187	-2.33	1.20E-03	7.51E-02	Distal Intergenic
Slc26a10	366909	chr7	70464659	70464959	2494	-2.27	1.20E-03	7.51E-02	Promoter (2-3kb)
Rassf4	362423	chr4	148802980	148803280	708	-2.31	1.21E-03	7.51E-02	Promoter (<=1kb)
Ackr3	84348	chr9	97439973	97440273	84049	1.84	1.21E-03	7.51E-02	Distal Intergenic
Myzap	363091	chr8	78117207	78117507	-20904	2.53	1.21E-03	7.51E-02	Distal Intergenic
Gnb1	24400	chr5	172932207	172932507	-2483	-2.24	1.21E-03	7.51E-02	Promoter (2-3kb)
Ppwd1	294711	chr2	34561817	34562117	-248723	-2.21	1.21E-03	7.51E-02	Distal Intergenic
Brf1	299347	chr6	137833645	137833945	-25342	-2.21	1.21E-03	7.51E-02	Distal Intergenic
Rp1	681377	chr5	14983310	14983610	-60345	1.88	1.21E-03	7.53E-02	Distal Intergenic
Dao	114027	chr12	48341150	48341450	32197	-2.2	1.21E-03	7.53E-02	Intron (NM_134404/171442, intron 11 of 15)
Nck2	316369	chr9	50277194	50277494	29502	-1.78	1.22E-03	7.53E-02	Intron (NM_001108216/316369, intron 1 of 4)
Jmjd6	360665	chr10	105783800	105784100	3703	-1.89	1.22E-03	7.53E-02	Intron (NM_001012143/360665, intron 4 of 5)
Oplah	116684	chr7	117377602	117377902	-8443	-2.17	1.22E-03	7.53E-02	Distal Intergenic
Gmfg	113940	chr1	85323954	85324254	-6492	2.06	1.22E-03	7.54E-02	Distal Intergenic
Twist2	59327	chr9	98801597	98801897	-122237	-2.12	1.24E-03	7.61E-02	Distal Intergenic
Il1rn	60582	chr3	1462781	1463081	-127	-2.39	1.24E-03	7.61E-02	Promoter (<=1kb)
Cops8	363283	chr9	97609723	97610023	-162201	-1.81	1.24E-03	7.61E-02	Distal Intergenic
Lrrc74a	314328	chr6	110780808	110781108	99257	-2.12	1.24E-03	7.61E-02	Distal Intergenic
Pla2g4a	24653	chr13	67088834	67089134	117554	-2.09	1.24E-03	7.61E-02	Intron (NM_133551/24653, intron 14 of 17)
Gli2	304729	chr13	35080459	35080759	-194974	1.98	1.24E-03	7.61E-02	Distal Intergenic
Elk3	362871	chr7	34105551	34105851	15843	-1.86	1.24E-03	7.61E-02	Intron (NM_001108743/362871, intron 1 of 3)
Mir3569	100526585	chr1	89042061	89042361	-445	3.25	1.24E-03	7.61E-02	Promoter (<=1kb)
Mir3573	100526647	chrX	69035815	69036115	142843	2.6	1.25E-03	7.65E-02	Distal Intergenic
Agfg1	363266	chr9	88628585	88628885	21077	-1.95	1.26E-03	7.67E-02	Intron (NM_001135596/363266, intron 1 of 13)
Antxr1	362393	chr4	118993050	118993350	137852	-1.43	1.26E-03	7.67E-02	Intron (NM_001044249/362393, intron 16 of 17)
Bcor	317346	chrX	11640948	11641248	-7741	0.91	1.26E-03	7.67E-02	Distal Intergenic
P2ry6	117264	chr1	165982690	165982990	14761	-2.28	1.26E-03	7.67E-02	Intron (NM_057124/117264, intron 1 of 2)
Umps	288051	chr11	70048979	70049279	14798	2.42	1.27E-03	7.67E-02	Distal Intergenic
Mylip	306825	chr17	19778859	19779159	-75178	-2.34	1.27E-03	7.67E-02	Distal Intergenic
Map4k3	170920	chr6	3384105	3384405	60114	-2.34	1.27E-03	7.67E-02	Intron (NM_133407/170920, intron 1 of 32)
Slc39a14	306009	chr15	52002277	52002577	27239	-2.28	1.27E-03	7.67E-02	Intron (NM_001107275/306009, intron 2 of 9)
Fam160b2	306015	chr15	52297877	52298177	-8984	-2.06	1.27E-03	7.68E-02	Intron (NM_001108385/361069, intron 11 of 14)
Hebp2	308632	chr1	13673641	13673941	46029	-2.07	1.27E-03	7.68E-02	Distal Intergenic
Fads2	83512	chr1	226189249	226189549	-36681	-1.76	1.27E-03	7.68E-02	Distal Intergenic
Lcat	24530	chr19	37924839	37925139	-8040	2.64	1.27E-03	7.68E-02	Exon (NM_019229/29501, exon 9 of 24)
Lrp12	314941	chr7	78956389	78956689	-25557	-1.95	1.27E-03	7.68E-02	Distal Intergenic
Zfp689	286996	chr1	198832156	198832456	67908	1.87	1.28E-03	7.71E-02	Distal Intergenic
Pbp2	246145	chr4	169088183	169088483	4652	2.87	1.28E-03	7.71E-02	Distal Intergenic
Cadm4	365216	chr1	81353112	81353412	-11788	2.72	1.29E-03	7.73E-02	Distal Intergenic
Tnfrsf21	316256	chr9	20620556	20620856	195	2.75	1.29E-03	7.73E-02	Promoter (<=1kb)
Stk24	361092	chr15	106658049	106658349	63690	2.47	1.29E-03	7.73E-02	Intron (NM_001127494/361092, intron 2 of 10)
Epdr1	291180	chr17	47406263	47406563	8705	-2	1.29E-03	7.74E-02	Intron (NM_001007625/291180, intron 1 of 2)
Snrpe	100360682	chr13	50227535	50227835	-24872	-2.19	1.29E-03	7.74E-02	Intron (NM_001047902/360845, intron 11 of 16)
Cdc42ep2	309175	chr1	221298001	221298301	-16821	1.98	1.30E-03	7.77E-02	Intron (NM_053480/85242, intron 17 of 17)
Slc7a8	84551	chr15	33428693	33428993	0	-1.78	1.31E-03	7.81E-02	Promoter (<=1kb)
F3	25584	chr2	225310490	225310790	0	1.11	1.31E-03	7.83E-02	Promoter (<=1kb)
Il17rd	498576	chr16	2750955	2751255	80337	-1.69	1.32E-03	7.85E-02	Distal Intergenic
Mx1	24575	chr11	37914525	37914825	158	-2.95	1.32E-03	7.85E-02	Promoter (<=1kb)
Lif	60584	chr14	84524433	84524733	27963	-2.2	1.32E-03	7.85E-02	Distal Intergenic
Gan	307893	chr19	49699657	49699957	4078	-2.2	1.32E-03	7.85E-02	Intron (NM_001107434/307893, intron 1 of 10)
Malsu1	297082	chr4	78794846	78795146	59567	-2.04	1.32E-03	7.85E-02	Distal Intergenic
Lsp1	361680	chr1	215629119	215629419	334	-2.56	1.33E-03	7.85E-02	Promoter (<=1kb)
Sertad2	498423	chr14	104880869	104881169	59654	-2.12	1.33E-03	7.85E-02	Intron (NM_001024903/498423, intron 1 of 1)
Myl1	56781	chr9	73998156	73998456	-39676	-2.59	1.35E-03	7.97E-02	Distal Intergenic
Trim29	300656	chr8	47682208	47682508	7887	2.51	1.35E-03	7.97E-02	Intron (NM_001106815/300656, intron 1 of 8)
Srpk2	296753	chr4	8005684	8005984	-60753	1.21	1.35E-03	7.97E-02	Distal Intergenic
Atp6v0a4	296981	chr4	65771532	65771832	46689	-2.2	1.35E-03	7.97E-02	Intron (NM_001106591/296981, intron 6 of 19)
Inpp5a	365382	chr1	211778386	211778686	45916	2.51	1.36E-03	7.97E-02	Intron (NM_001108923/365382, intron 1 of 15)
Spata3	363270	chr9	93025146	93025446	-5268	-2.1	1.36E-03	7.98E-02	Distal Intergenic

Thbs1	445442	chr3	109741464	109741764	-120356	-2.1	1.36E-03	7.98E-02	Distal Intergenic
Dock6	367039	chr8	22881597	22881897	-6960	-1.72	1.38E-03	8.06E-02	Distal Intergenic
Zfp347	170902	chr7	9990392	9990692	268	-1.98	1.38E-03	8.06E-02	Promoter (<=1kb)
Flt3	140635	chr12	9326727	9327027	-33412	-2.05	1.38E-03	8.06E-02	Distal Intergenic
Dpep2	291984	chr19	37969439	37969739	798	1.91	1.38E-03	8.07E-02	Promoter (<=1kb)
Nfix	81524	chr19	25866581	25866881	47815	2.64	1.38E-03	8.07E-02	Intron (NM_030866/81524, intron 2 of 9)
Cetn3	170895	chr2	9768783	9769083	85102	-1.75	1.39E-03	8.07E-02	Distal Intergenic
Chchd6	297436	chr4	121504086	121504386	60826	-2.1	1.39E-03	8.07E-02	Intron (NM_001106608/297436, intron 4 of 7)
Lrch1	502020	chr15	56913148	56913448	56917	-1.9	1.39E-03	8.07E-02	Intron (NM_001134727/502020, intron 1 of 18)
Tubb4a	29213	chr9	9964195	9964495	3175	-2.19	1.39E-03	8.08E-02	Intron (NM_080882/29213, intron 3 of 3)
Tmem19	299800	chr7	58378691	58378991	-35554	-2.18	1.39E-03	8.08E-02	Distal Intergenic
Tspan18	311210	chr3	82201489	82201789	34875	1.86	1.39E-03	8.08E-02	Intron (NM_001107750/311210, intron 2 of 9)
Zbtb10	80338	chr2	94826609	94826909	-95633	-2.35	1.41E-03	8.20E-02	Distal Intergenic
Slc30a4	64469	chr3	114845475	114845775	-17321	-2.09	1.42E-03	8.22E-02	Distal Intergenic
Azin1	58961	chr7	77327301	77327601	44797	1.17	1.42E-03	8.24E-02	Distal Intergenic
Tmem98	303356	chr10	68173131	68173431	0	0.92	1.43E-03	8.25E-02	Promoter (<=1kb)
Cpvl	502774	chr4	84215798	84216098	-84768	2.68	1.43E-03	8.25E-02	Distal Intergenic
Limd1	316101	chr8	132577409	132577709	24191	-2.03	1.43E-03	8.26E-02	Intron (NM_001112737/316101, intron 2 of 7)
Gpr45	301372	chr9	49795970	49796270	-41598	2.27	1.43E-03	8.26E-02	Distal Intergenic
Hif1an	309434	chr1	264401821	264402121	92607	-2.53	1.43E-03	8.26E-02	Distal Intergenic
Apol3	315108	chr7	118685270	118685570	253	-1.66	1.44E-03	8.29E-02	Promoter (<=1kb)
Saraf	290796	chr16	61750134	61750434	3004	-1.94	1.45E-03	8.31E-02	Intron (NM_001004213/290796, intron 1 of 5)
Dusp6	116663	chr7	41417047	41417347	-57816	1.75	1.45E-03	8.31E-02	Distal Intergenic
Cnot1	291841	chr19	9702729	9703029	34543	2.31	1.45E-03	8.31E-02	Exon (NM_001134840/291841, exon 8 of 49)
Afdn	26955	chr1	53746857	53747157	94099	2.1	1.45E-03	8.31E-02	Intron (NM_013217/26955, intron 29 of 39)
Csrp1	29276	chr13	52413481	52413781	-140062	0.9	1.46E-03	8.33E-02	Distal Intergenic
Dnttip1	171437	chr3	161215283	161215583	3127	-1.91	1.46E-03	8.37E-02	Intron (NM_134400/171437, intron 3 of 12)
Slc35b1	287642	chr10	83197438	83197738	-3794	1.8	1.47E-03	8.37E-02	Exon (NM_001109039/497983, exon 8 of 8)
Neil3	290729	chr16	41084120	41084420	4676	-2.14	1.47E-03	8.39E-02	Intron (NM_001170346/290729, intron 1 of 10)
Dpy30	286897	chr6	22328491	22328791	26422	-2.02	1.48E-03	8.39E-02	Distal Intergenic
Kcnh6	116745	chr10	94211647	94211947	4311	-1.95	1.48E-03	8.39E-02	Intron (NM_053937/116745, intron 2 of 12)
Bmi1	307151	chr17	85364274	85364574	0	2.23	1.48E-03	8.39E-02	Promoter (<=1kb)
Tes	500040	chr4	44406643	44406943	84760	-2.08	1.48E-03	8.39E-02	Distal Intergenic
Cnksr3	308113	chr1	43856715	43857015	27252	-1.98	1.48E-03	8.39E-02	Intron (NM_001012061/308113, intron 1 of 12)
Pde4dip	64183	chr2	200016999	200017299	-13556	-2.12	1.48E-03	8.39E-02	Distal Intergenic
Serpine2	29366	chr9	85593377	85593677	32417	-2.08	1.49E-03	8.39E-02	Intron (NM_019197/29366, intron 1 of 8)
Tnfrsf1a	25625	chr4	157932466	157932766	67476	-2.22	1.49E-03	8.39E-02	Distal Intergenic
Lamc1	117036	chr13	70779510	70779810	3705	-1.27	1.49E-03	8.39E-02	Intron (NM_053966/117036, intron 1 of 27)
Nr3c2	25672	chr19	34495106	34495406	265597	-2.21	1.49E-03	8.39E-02	Intron (NM_013131/25672, intron 4 of 9)
Cnn3	54321	chr2	225020034	225020334	14965	-2.21	1.49E-03	8.39E-02	Intron (NM_019359/54321, intron 1 of 6)
Lrrc74a	314328	chr6	110785447	110785747	103896	-2.13	1.50E-03	8.42E-02	Distal Intergenic
Tmem170b	361230	chr17	22842363	22842663	21303	-2.19	1.50E-03	8.43E-02	Intron (NM_001008774/361230, intron 1 of 2)
Mrpl1	289491	chr14	14945352	14945652	62484	1.47	1.50E-03	8.43E-02	Distal Intergenic
Tnfrsf1b	156767	chr5	163174016	163174316	-6717	-2.36	1.50E-03	8.43E-02	Distal Intergenic
Kcmf1	684322	chr4	100811678	100811978	-28661	-2.05	1.51E-03	8.45E-02	Distal Intergenic
Ablim3	307395	chr18	57287009	57287309	-41310	1.2	1.51E-03	8.45E-02	Distal Intergenic
Tmcc3	314751	chr7	35475444	35475744	3342	2.53	1.52E-03	8.48E-02	Intron (NM_001108084/314751, intron 1 of 3)
Pxylp1	315939	chr8	104752772	104753072	37567	-1.72	1.52E-03	8.51E-02	Intron (NM_001007710/315939, intron 2 of 5)
Slc38a6	299139	chr6	96255651	96255951	83895	2	1.53E-03	8.55E-02	Distal Intergenic
Mmp24	83513	chr3	151310641	151310941	3324	2.65	1.55E-03	8.62E-02	Exon (NM_031757/83513, exon 7 of 21)
Cebpb	24253	chr3	164521029	164521329	96527	-2.28	1.55E-03	8.62E-02	Distal Intergenic
Cyth4	500906	chr7	119848416	119848716	27876	-2.21	1.55E-03	8.62E-02	Distal Intergenic
Slc29a3	353307	chr20	30323878	30324178	3165	2.4	1.55E-03	8.62E-02	Exon (NM_181639/353307, exon 2 of 6)
Cxcl10	245920	chr14	17211240	17211540	507	-1.84	1.55E-03	8.62E-02	Promoter (<=1kb)
Csnk2a2	307641	chr19	9982147	9982447	9610	-1.46	1.56E-03	8.64E-02	Intron (NM_001107409/307641, intron 2 of 11)
Ccdc80	64387	chr11	60668248	60668548	10701	-1.85	1.56E-03	8.67E-02	Intron (NM_022543/64387, intron 3 of 7)
B3gnt2	305571	chr14	107599974	107600274	16887	-1.7	1.56E-03	8.67E-02	Intron (NM_001107240/305571, intron 2 of 2)
Map4k4	301363	chr9	46724368	46724668	66446	-2.04	1.57E-03	8.67E-02	Intron (NM_001106904/301363, intron 2 of 28)
Ppp4r2	297486	chr4	133207000	133207300	-78252	2.43	1.57E-03	8.69E-02	Distal Intergenic
Usp21	688466	chr13	89657564	89657864	3286	2.32	1.57E-03	8.69E-02	Exon (NM_001127638/688466, exon 6 of 13)
Dtnbp1	641528	chr17	20196783	20197083	106647	-1.73	1.58E-03	8.69E-02	Distal Intergenic
Myo18a	360570	chr10	65026298	65026598	53937	2.09	1.58E-03	8.70E-02	Exon (NM_001172137/360570, exon 4 of 46)
Serpine1	24617	chr12	22651172	22651472	10068	-1.4	1.58E-03	8.70E-02	3' UTR
Mir328b	100526626	chr14	45364254	45364554	33108	1.95	1.58E-03	8.70E-02	Distal Intergenic
Atp11a	306600	chr16	82119392	82119692	-19170	-2.02	1.59E-03	8.72E-02	Distal Intergenic
Them6	300015	chr7	115953131	115953431	-2845	1.87	1.59E-03	8.73E-02	Promoter (2-3kb)
Rtn4rl1	303311	chr10	62035646	62035946	0	1.04	1.59E-03	8.74E-02	Promoter (<=1kb)
Rpl35al1	57809	chr5	164913822	164914122	-9179	2.25	1.59E-03	8.74E-02	Distal Intergenic
Spaca5	314431	chrX	894223	894523	21430	1.4	1.60E-03	8.76E-02	Distal Intergenic
Mir29b1	100314008	chr4	58401864	58402164	-57474	-1.97	1.60E-03	8.76E-02	Distal Intergenic
Rtbdn	304667	chr19	26074523	26074823	8396	-2.03	1.60E-03	8.76E-02	Downstream (<1kb)
LOC690120	690120	chr7	99897898	99898198	55886	-1.92	1.60E-03	8.76E-02	Intron (NM_001024876/299957, intron 6 of 6)
Tcf12	25720	chr8	78708390	78708690	-52534	2.32	1.61E-03	8.76E-02	Distal Intergenic
Ubac1	362087	chr3	3483842	3484142	-7627	-2.08	1.62E-03	8.80E-02	Distal Intergenic
Kpna6	362607	chr5	147883986	147884286	-17804	-2.09	1.62E-03	8.80E-02	Distal Intergenic
Klf5	84410	chr15	83732578	83732878	24843	-1.93	1.63E-03	8.85E-02	Distal Intergenic
Fas	246097	chr1	252651773	252652073	61988	-1.61	1.63E-03	8.85E-02	Distal Intergenic
Tuba4a	316531	chr9	82420646	82420946	-1358	2.61	1.63E-03	8.85E-02	Promoter (1-2kb)
Slc4a11	311423	chr3	123223535	123223835	12700	2.65	1.64E-03	8.86E-02	Downstream (<1kb)
Oat	64313	chr1	204469253	204469553	112517	-1.78	1.64E-03	8.88E-02	Distal Intergenic
Akap12	83425	chr1	40890413	40890713	10666	2.8	1.64E-03	8.88E-02	Intron (NM_057103/83425, intron 2 of 3)
Egln3	54702	chr6	75057416	75057716	18079	-2	1.64E-03	8.88E-02	Intron (NM_019371/54702, intron 1 of 4)
Chst11	314694	chr7	26820286	26820586	39130	-1.89	1.65E-03	8.93E-02	Intron (NM_001108079/314694, intron 1 of 2)
Dtwd2	361326	chr18	42794086	42794386	1150887	-1.96	1.66E-03	8.93E-02	Distal Intergenic
Strap	297699	chr4	171515815	171516115	-214514	2.21	1.66E-03	8.93E-02	Distal Intergenic
Aak1	500244	chr4	118694861	118695161	39133	1.79	1.67E-03	9.00E-02	Intron (NM_001173450/500244, intron 1 of 20)
Spred1	296072	chr3	108829380	108829680	34043	-1.85	1.67E-03	9.00E-02	Intron (NM_001047089/296072, intron 1 of 6)
Map3k3	303604	chr10	94307630	94307930	26927	2.48	1.68E-03	9.03E-02	Exon (NM_001107058/303604, exon 3 of 16)
Zfp507	292816	chr1	92146088	92146388	-26137	-2.11	1.68E-03	9.03E-02	Distal Intergenic
Fam107b	498796	chr17	78523036	78523336	38277	1.89	1.68E-03	9.03E-02	Intron (NM_001025034/498796, intron 1 of 3)
Mapk1	116590	chr11	88253357	88253657	19637	-1.87	1.69E-03	9.03E-02	Intron (NM_053842/116590, intron 1 of 8)
Nuak2	289419	chr13	49106109	49106409	13803	-2.19	1.69E-03	9.03E-02	Exon (NM_001007617/289419, exon 6 of 7)
Hnrnpa3	362152	chr3	62488137	62488437	6894	-1.57	1.70E-03	9.07E-02	3' UTR
Dazap1	362836	chr7	12311024	12311324	0	1.59	1.70E-03	9.08E-02	Promoter (<=1kb)

Zfp608	307296	chr18	49792695	49792995	144527	-1.62	1.70E-03	9.09E-02	Distal Intergenic
Gria1	50592	chr10	42623756	42624056	182033	-2.13	1.71E-03	9.10E-02	Intron (NM_031608/50592, intron 11 of 22)
Popdc3	641520	chr20	50394841	50395141	168	1.96	1.71E-03	9.10E-02	Promoter (<=1kb)
RGD1563302	361049	chr15	39460192	39460492	145511	-2.07	1.71E-03	9.11E-02	Distal Intergenic
Chchd6	297436	chr4	121375987	121376287	188925	-2.13	1.71E-03	9.11E-02	Intron (NM_001106608/297436, intron 6 of 7)
RGD1562310	498188	chr12	43690112	43690412	250386	1.56	1.72E-03	9.12E-02	Distal Intergenic
Zbtb49	305428	chr14	77333058	77333358	11018	-2.2	1.72E-03	9.13E-02	Intron (NM_001107223/305428, intron 3 of 6)
LOC689766	689766	chr13	95855624	95855924	-31784	-1.79	1.72E-03	9.13E-02	Distal Intergenic
Hmga2	84017	chr7	65382223	65382523	-106815	-2.01	1.73E-03	9.13E-02	Distal Intergenic
Phtf2	296762	chr4	11545312	11545612	722054	2.47	1.73E-03	9.13E-02	Intron (NM_053621/113970, intron 3 of 23)
RGD1309489	314915	chr7	83666065	83666365	-3816	0.99	1.73E-03	9.14E-02	Distal Intergenic
Srpra	315548	chr8	36419546	36419846	8863	-1.43	1.73E-03	9.15E-02	Intron (NM_001025283/315549, intron 5 of 6)
Slc16a1	25027	chr2	207113108	207113408	4556	-2.26	1.73E-03	9.15E-02	Intron (NM_012716/25027, intron 1 of 4)
Rnf222	363627	chr10	55463163	55463463	-14835	-2.1	1.74E-03	9.16E-02	Distal Intergenic
Kcnk1	59324	chr19	58967882	58968182	144046	1.43	1.75E-03	9.22E-02	Distal Intergenic
Smad2	29357	chr18	72741559	72741859	191105	1.22	1.76E-03	9.26E-02	Distal Intergenic
Hsdl2	313200	chr5	76862797	76863097	49866	-2.07	1.77E-03	9.26E-02	Distal Intergenic
Wnt7a	114850	chr4	123040585	123040885	0	2.65	1.77E-03	9.26E-02	Promoter (<=1kb)
Mir145	100314036	chr18	56938004	56938304	31690	-2.11	1.77E-03	9.26E-02	Distal Intergenic
Trim8	688785	chr1	266210766	266211066	-44731	-1.91	1.77E-03	9.26E-02	Intron (NM_001024899/361769, intron 8 of 11)
Tbx2	303398	chr10	73208682	73208982	-70017	1.68	1.77E-03	9.27E-02	Intron (NM_001173430/363662, intron 16 of 17)
Slc66a1	362642	chr5	157737598	157737898	19000	2.36	1.78E-03	9.28E-02	Exon (NM_001005903/298584, exon 7 of 9)
Rft1	290552	chr16	6695157	6695457	-16932	1.63	1.78E-03	9.28E-02	Distal Intergenic
Capn13	362701	chr6	24758584	24758884	-82166	-2.32	1.78E-03	9.28E-02	Distal Intergenic
S100a6	85247	chr2	190016838	190017138	9552	-1.74	1.78E-03	9.28E-02	Distal Intergenic
Tpra1	85494	chr4	120868479	120868779	24980	1.06	1.78E-03	9.28E-02	Distal Intergenic
Cilp	315761	chr8	70766688	70766988	5766	2.21	1.79E-03	9.28E-02	Intron (NM_001108161/315761, intron 4 of 9)
St3gal4	363040	chr8	36305218	36305518	9293	-2.09	1.79E-03	9.28E-02	Intron (NM_203337/363040, intron 1 of 10)
Mepe	79110	chr14	6738602	6738902	54659	-1.49	1.79E-03	9.28E-02	Distal Intergenic
Kbtbd11	306617	chr16	79715145	79715445	-14153	1.29	1.79E-03	9.28E-02	Distal Intergenic
Lemd3	680066	chr7	62973713	62974013	71715	-2.03	1.79E-03	9.28E-02	Distal Intergenic
Foxs1	311547	chr3	148417139	148417439	-9361	-1.83	1.79E-03	9.28E-02	Downstream (<1kb)
Atp13a1	290673	chr16	21417250	21417550	-37108	1.41	1.79E-03	9.28E-02	Distal Intergenic
Pdgfa	25266	chr12	17720854	17721154	-12987	-0.85	1.80E-03	9.28E-02	Distal Intergenic
Cdk6	114483	chr4	27879542	27879842	86556	-2.04	1.81E-03	9.31E-02	Intron (NM_001191861/114483, intron 2 of 6)
Fut8	432392	chr6	100348750	100349050	-48003	-2.04	1.81E-03	9.31E-02	Distal Intergenic
Cblc	292699	chr1	80717028	80717328	-882	-2.21	1.81E-03	9.33E-02	Promoter (<=1kb)
Npepps	50558	chr10	85216229	85216529	6332	-1.82	1.81E-03	9.34E-02	Intron (NM_080395/50558, intron 1 of 22)
Kcna6	64358	chr4	159308130	159308430	-20937	-2.12	1.82E-03	9.35E-02	Distal Intergenic
Kank4	313385	chr5	117412679	117412979	27206	2.3	1.82E-03	9.36E-02	Intron (NM_001107947/313385, intron 1 of 9)
Abhd2	293050	chr1	141024934	141025234	26694	-2.05	1.82E-03	9.37E-02	Intron (NM_001106275/293050, intron 2 of 10)
Sh3bgrl2	501026	chr8	91111191	91111491	41266	-1.59	1.83E-03	9.39E-02	Intron (NM_001137647/501026, intron 2 of 3)
Arhgap10	688429	chr19	34229584	34229884	89587	-1.31	1.83E-03	9.39E-02	Intron (NM_001109501/688429, intron 4 of 22)
Ptprg	171357	chr15	13362086	13362386	271453	-2.13	1.84E-03	9.39E-02	Intron (NM_134356/171357, intron 2 of 41)
Atp6ap2	302526	chrX	11165057	11165357	-203	1.33	1.84E-03	9.40E-02	Promoter (<=1kb)
Zfp131	310375	chr2	52708207	52708507	-177122	-1.89	1.84E-03	9.41E-02	Distal Intergenic
Asap1	314961	chr7	104826266	104826566	124524	-1.85	1.84E-03	9.41E-02	Intron (NM_001044245/314961, intron 1 of 27)
Rb1cc1	312927	chr5	13105089	13105389	-7748	-2.2	1.85E-03	9.42E-02	Distal Intergenic
Runx2	367218	chr9	18610328	18610628	-32942	-1.87	1.85E-03	9.43E-02	Intron (NM_001278483/367218, intron 2 of 8)
Zfp418	292548	chr1	69617728	69618028	2125	-1.79	1.85E-03	9.43E-02	Promoter (2-3kb)
Grem1	50566	chr3	104925419	104925719	289270	-1.83	1.86E-03	9.44E-02	Distal Intergenic
Fzd7	100360552	chr9	66314892	66315192	9161	-1.75	1.86E-03	9.44E-02	Distal Intergenic
Phlpp1	59265	chr13	26165448	26165748	-6467	2.47	1.86E-03	9.45E-02	Distal Intergenic
Ust	361450	chr1	2945328	2945628	-317853	-2.3	1.86E-03	9.45E-02	Distal Intergenic
Ubash3b	315579	chr8	45417251	45417551	-42117	-2.03	1.87E-03	9.45E-02	Distal Intergenic
Mir3591	100526608	chr18	60761011	60761311	-5631	2.75	1.87E-03	9.46E-02	Intron (NM_001025057/502176, intron 3 of 3)
Ndst3	295430	chr2	227953813	227954113	-63736	-2.04	1.88E-03	9.46E-02	Distal Intergenic
Klhl9	313348	chr5	107322570	107322870	-388	1.26	1.88E-03	9.46E-02	Promoter (<=1kb)
Lamc1	117036	chr13	70746898	70747198	36317	-1.97	1.88E-03	9.46E-02	Intron (NM_053966/117036, intron 1 of 27)
Specc1	303208	chr10	48302295	48302595	61965	-1.66	1.88E-03	9.46E-02	Intron (NM_001039017/303208, intron 1 of 7)
Gls	24398	chr9	54130454	54130754	-82013	-1.59	1.88E-03	9.46E-02	Distal Intergenic
Sgk1	29517	chr1	24399317	24399617	-205750	-1.97	1.88E-03	9.46E-02	Distal Intergenic
Sgce	432360	chr4	29761224	29761524	8378	-2.02	1.88E-03	9.46E-02	Intron (NM_001002023/432360, intron 3 of 9)
Mmd	303439	chr10	77755068	77755368	-220	1.25	1.89E-03	9.49E-02	Promoter (<=1kb)
MGC94199	362483	chr5	23460333	23460633	101527	-2.22	1.89E-03	9.49E-02	Distal Intergenic
Col1a2	84352	chr4	31734863	31735163	200638	-2.21	1.89E-03	9.49E-02	Distal Intergenic
Mkln1	83536	chr4	58593585	58593885	-99499	-2.04	1.89E-03	9.49E-02	Distal Intergenic
Vom2r27	286914	chr1	64723074	64723374	-23918	-2.07	1.90E-03	9.49E-02	Distal Intergenic
Sgsm3	362963	chr7	122191569	122191869	-11663	2.19	1.90E-03	9.49E-02	Exon (NM_001130503/315150, exon 13 of 13)
Porcn	317368	chrX	15035509	15035809	0	0.83	1.91E-03	9.54E-02	Promoter (<=1kb)
Prkacb	293508	chr2	254327969	254328269	-1636083	-2.01	1.91E-03	9.54E-02	Distal Intergenic
Depp1	500300	chr4	148775480	148775780	-6699	-2.01	1.91E-03	9.54E-02	Intron (NM_001024275/362423, intron 6 of 10)
Il12b	64546	chr10	30064431	30064731	25722	2.03	1.91E-03	9.54E-02	Distal Intergenic
Hils1	690026	chr10	82777368	82777668	1677	-1.68	1.92E-03	9.54E-02	Promoter (1-2kb)
Eef2k	25435	chr1	190811841	190812141	13136	2.1	1.92E-03	9.54E-02	Intron (NM_012947/25435, intron 1 of 16)
Gmds	291095	chr17	33521535	33521835	112813	-2.01	1.92E-03	9.54E-02	Intron (NM_001039606/291095, intron 4 of 10)
Adcy8	29241	chr7	105376701	105377001	215803	-2.14	1.92E-03	9.54E-02	Intron (NM_017142/29241, intron 13 of 17)
Tmed5	289883	chr14	2656866	2657166	43548	-1.88	1.92E-03	9.55E-02	Downstream (1-2kb)
Klrk1	24934	chr4	163403749	163404049	-96	-1.94	1.94E-03	9.60E-02	Promoter (<=1kb)
Slc47a1	360539	chr10	47682421	47682721	-15545	-2.02	1.94E-03	9.60E-02	Distal Intergenic
Sgo2	316425	chr9	64891410	64891710	-8818	-1.57	1.94E-03	9.60E-02	Intron (NM_001106914/301436, intron 3 of 8)
Fkbp3	299104	chr6	86810541	86810841	11274	2.21	1.94E-03	9.61E-02	Downstream (<1kb)
Ccnd1	58919	chr1	218189941	218190241	-89669	-1.76	1.95E-03	9.61E-02	Distal Intergenic
Hcn2	114244	chr7	12869558	12869858	229	1.45	1.95E-03	9.62E-02	Promoter (<=1kb)
Asic1	79123	chr7	141325639	141325939	-382	1.83	1.96E-03	9.64E-02	Promoter (<=1kb)
LOC691083	691083	chr3	95239851	95240151	7685	2.14	1.96E-03	9.67E-02	Intron (NM_001144862/691083, intron 1 of 2)
Mtdh	170910	chr7	72327701	72328001	-444439	2.72	1.97E-03	9.67E-02	Distal Intergenic
Il7	25647	chr2	96415791	96416091	-14677	-1.6	1.97E-03	9.68E-02	Distal Intergenic
Kank4	313385	chr5	117503567	117503867	-63382	1.97	1.97E-03	9.69E-02	Distal Intergenic
Map3k9	500690	chr6	105518557	105518857	0	1.45	1.97E-03	9.69E-02	Promoter (<=1kb)
Sf3b5	680891	chr1	7095461	7095761	-5954	-2.06	1.98E-03	9.69E-02	Distal Intergenic
Imp4	316317	chr9	37788239	37788539	-13142	-2.17	1.98E-03	9.69E-02	Distal Intergenic
Phf11	361051	chr15	38786628	38786928	-76643	-1.88	1.98E-03	9.69E-02	Distal Intergenic
Oma1	298282	chr5	122806824	122807124	-40521	-2.29	1.98E-03	9.70E-02	Distal Intergenic

Nampt	297508	chr6	52126596	52126896	4511	-1.97	1.99E-03	9.74E-02	Intron (NM_177928/297508, intron 1 of 10)
Etv6	312777	chr4	167759891	167760191	5207	-1.86	2.00E-03	9.75E-02	Intron (NM_001037353/312777, intron 1 of 7)
Nkain4	296469	chr3	176458969	176459269	5893	2.55	2.00E-03	9.77E-02	Intron (NM_001106550/296469, intron 1 of 4)
Abcd3	25270	chr2	225388799	225389099	21	1.04	2.01E-03	9.77E-02	Promoter (<=1kb)
C1rl	408246	chr4	157101050	157101350	-6840	-2.28	2.01E-03	9.77E-02	Distal Intergenic
Ttc12	300696	chr8	53870730	53871030	-54277	2.83	2.01E-03	9.78E-02	Intron (NM_031521/24586, intron 14 of 18)
Herpud2	300463	chr8	26186153	26186453	124897	2.49	2.01E-03	9.78E-02	Distal Intergenic
Slc25a37	306000	chr15	51170246	51170546	-1862	-2.21	2.01E-03	9.78E-02	Promoter (1-2kb)
Pabpc6	292295	chr1	51525237	51525537	-94338	-1.67	2.02E-03	9.80E-02	Distal Intergenic
Cubn	80848	chr17	80812732	80813032	-5551	1.92	2.02E-03	9.80E-02	Distal Intergenic
Hspb1	24471	chr12	23836477	23836777	4274	2.52	2.02E-03	9.80E-02	Downstream (2-3kb)
Dele1	307480	chr18	31356889	31357189	-39798	-2.05	2.03E-03	9.80E-02	Distal Intergenic
Wwtr1	295062	chr2	147668157	147668457	24576	-2.08	2.03E-03	9.81E-02	Intron (NM_001024869/295062, intron 2 of 6)
Wwtr1	295062	chr2	147666819	147667119	25914	-1.85	2.03E-03	9.82E-02	Intron (NM_001024869/295062, intron 2 of 6)
Mras	25482	chr8	107601848	107602148	54703	0.78	2.04E-03	9.82E-02	Distal Intergenic
Utp23	299900	chr7	91437942	91438242	53613	-2.29	2.04E-03	9.83E-02	Distal Intergenic
Nr2f6	245980	chr16	19780608	19780908	-3194	2.51	2.04E-03	9.83E-02	Downstream (<1kb)
Fgf5	60662	chr14	13204380	13204680	-208796	-1.85	2.05E-03	9.85E-02	Distal Intergenic
Nol3	85383	chr19	37238429	37238729	3428	-1.87	2.05E-03	9.86E-02	Exon (NM_001044292/688736, exon 6 of 7)
Tes	500040	chr4	44390561	44390861	68678	-2.27	2.05E-03	9.87E-02	Distal Intergenic
Ppp1r15a	171071	chr1	101508390	101508690	6284	-1.87	2.06E-03	9.89E-02	Intron (NM_001012168/361576, intron 5 of 9)
Atxn1	25049	chr17	19235476	19235776	74482	-2.14	2.06E-03	9.89E-02	Intron (NM_012726/25049, intron 1 of 6)
Ccdc97	292724	chr1	82506224	82506524	4803	-2.01	2.06E-03	9.89E-02	Intron (NM_001106235/292724, intron 4 of 4)
Igtp	303163	chr10	43628385	43628685	-2420	-2	2.07E-03	9.89E-02	Promoter (2-3kb)
Nup58	245922	chr15	40466553	40466853	78971	1.25	2.07E-03	9.89E-02	Distal Intergenic
Adnp	64622	chr3	164950303	164950603	13583	-2.16	2.07E-03	9.89E-02	Intron (NM_001347532/64622, intron 1 of 3)
Kif13a	308173	chr17	18222476	18222776	70494	1.56	2.07E-03	9.90E-02	Intron (NM_001107462/308173, intron 1 of 36)
Rhob	64373	chr6	33667787	33668087	23211	2.18	2.07E-03	9.90E-02	Distal Intergenic
Tnip1	363599	chr10	40287388	40287688	15384	-2.22	2.08E-03	9.91E-02	Intron (NM_001108826/363599, intron 2 of 18)
Frmd8	309172	chr1	221187325	221187625	46298	2.15	2.08E-03	9.91E-02	Distal Intergenic
Eed	293104	chr1	154202123	154202423	13917	-1.94	2.08E-03	9.91E-02	Intron (NM_001106278/293104, intron 7 of 11)
Ypel2	360590	chr10	74262195	74262495	36104	-1.91	2.09E-03	9.92E-02	Intron (NM_001108286/360590, intron 2 of 4)
Lyn	81515	chr5	16554512	16554812	28454	-1.95	2.09E-03	9.92E-02	Intron (NM_001111098/81515, intron 1 of 12)
Paqr9	315904	chr8	102986778	102987078	-350394	-1.73	2.09E-03	9.92E-02	Intron (NM_001271438/363115, intron 14 of 15)
Wasf2	313024	chr5	151284355	151284655	13510	2.49	2.09E-03	9.92E-02	Intron (NM_001013167/313024, intron 1 of 13)
Slc22a12	365398	chr1	221906380	221906680	11221	1.12	2.09E-03	9.92E-02	Exon (NM_053846/116595, exon 22 of 22)
Rdh13	361504	chr1	72956104	72956404	87	0.9	2.10E-03	9.92E-02	Promoter (<=1kb)
Spint1	311331	chr3	111063598	111063898	14283	-1.92	2.10E-03	9.93E-02	Distal Intergenic
LOC499843	499843	chr3	90061785	90062085	688970	1.45	2.11E-03	9.99E-02	Distal Intergenic
Fhl2	63839	chr9	49933280	49933580	16491	-1.96	2.12E-03	1.00E-01	Intron (NM_031677/63839, intron 2 of 4)
Bmp7	85272	chr3	170823293	170823593	132227	1.97	2.12E-03	1.00E-01	Distal Intergenic
Olfm2	313783	chr8	21761738	21762038	-18426	1.27	2.13E-03	1.00E-01	Distal Intergenic
Pls3	81748	chrX	118946884	118947184	-83235	-2.04	2.13E-03	1.00E-01	Distal Intergenic
Clip1	65201	chr12	38353565	38353865	8109	1.89	2.13E-03	1.00E-01	Intron (NM_031745/65201, intron 1 of 24)
Dynlrb2	361415	chr19	49048188	49048488	31269	0.82	2.13E-03	1.00E-01	Distal Intergenic
Nod1	500133	chr4	85168238	85168538	6393	-2.06	2.13E-03	1.00E-01	Intron (NM_001109236/500133, intron 1 of 11)
Hs6st1	316325	chr9	42454154	42454454	-165552	-2.06	2.14E-03	1.00E-01	Distal Intergenic
Ms4a2	25316	chr1	227943679	227943979	21333	2.06	2.14E-03	1.00E-01	Distal Intergenic
Rxra	25271	chr3	6256397	6256697	44581	-1.62	2.14E-03	1.00E-01	Intron (NM_012805/25271, intron 1 of 9)
Afg3l1	361436	chr19	56272080	56272380	0	0.8	2.14E-03	1.00E-01	Promoter (<=1kb)
Myc	24577	chr7	103494034	103494334	907721	-2.26	2.15E-03	1.01E-01	Distal Intergenic
Wdr78	313417	chr5	122616462	122616762	25399	1.5	2.15E-03	1.01E-01	Intron (NM_001024786/313417, intron 4 of 16)
Serpina3n	24795	chr6	128073880	128074180	536	-1.73	2.15E-03	1.01E-01	Promoter (<=1kb)
Peak1	315686	chr8	61013407	61013707	65819	-1.93	2.16E-03	1.01E-01	Intron (NM_001108149/315686, intron 2 of 6)
H1f1	291145	chr17	43614380	43614680	164	-1.91	2.16E-03	1.01E-01	Promoter (<=1kb)
Cebpb	24253	chr3	164486024	164486324	61522	-1.98	2.16E-03	1.01E-01	Distal Intergenic
Gclc	25283	chr8	85072605	85072905	13554	-2.05	2.16E-03	1.01E-01	Intron (NM_012815/25283, intron 1 of 15)
Cav1	25404	chr4	44591007	44591307	-5816	-1.35	2.17E-03	1.01E-01	Distal Intergenic
Agfg1	363266	chr9	88600182	88600482	-7026	-1.15	2.17E-03	1.01E-01	Distal Intergenic
Fgfr2	25022	chr1	200433769	200434069	262859	2.59	2.17E-03	1.01E-01	Distal Intergenic
Cnot9	301513	chr9	81778937	81779237	-4112	-1.75	2.17E-03	1.01E-01	Distal Intergenic
Glul	24957	chr13	71516937	71517237	185885	-2.24	2.18E-03	1.01E-01	Distal Intergenic
Serpine2	29366	chr9	85658228	85658528	-32134	-1.99	2.18E-03	1.01E-01	Distal Intergenic
Paqr9	315904	chr8	102985778	102986078	-351394	-1.27	2.18E-03	1.01E-01	Intron (NM_001271438/363115, intron 14 of 15)
Ppp4r1	140943	chr9	113526006	113526306	10715	-1.79	2.19E-03	1.01E-01	Intron (NM_080907/140943, intron 3 of 20)
Mfap5	362429	chr4	155313566	155313866	0	-1.72	2.19E-03	1.01E-01	Promoter (<=1kb)
Thap4	363291	chr9	100860939	100861239	26868	-1.97	2.19E-03	1.01E-01	Intron (NM_001005564/363291, intron 3 of 5)
Parp9	303905	chr11	67748400	67748700	8097	-2.53	2.19E-03	1.01E-01	Intron (NM_001103351/303905, intron 4 of 10)
Sema4b	293042	chr1	141975435	141975735	-10410	-1.6	2.19E-03	1.01E-01	Distal Intergenic
Kcnn4	65206	chr1	81225613	81225913	-4934	0.87	2.19E-03	1.01E-01	Distal Intergenic
Reck	313488	chr5	59348590	59348890	0	0.92	2.19E-03	1.01E-01	Promoter (<=1kb)
Rad51c	497976	chr10	74758087	74758387	-34140	2.22	2.20E-03	1.01E-01	Distal Intergenic
Grhl2	299979	chr7	76119309	76119609	59858	0.84	2.20E-03	1.01E-01	Intron (NM_001134527/299979, intron 7 of 15)
Extl3	56819	chr15	48469329	48469629	-23737	-2.04	2.20E-03	1.01E-01	Distal Intergenic
Smad2	29357	chr18	72555078	72555378	4624	-2.04	2.20E-03	1.01E-01	Intron (NM_001277450/29357, intron 1 of 10)
Fgf5	60662	chr14	13216240	13216540	-220656	-1.99	2.20E-03	1.01E-01	Distal Intergenic
Arx	317268	chrX	62365568	62365868	1811	2.39	2.20E-03	1.01E-01	Promoter (1-2kb)
Ormdl3	360618	chr10	86554083	86554383	95	-1.69	2.21E-03	1.01E-01	Promoter (<=1kb)
Mb	59108	chr7	118231488	118231788	-122624	-2.11	2.22E-03	1.01E-01	Intron (NM_001079895/362950, intron 1 of 12)
Tomm20	266601	chr19	59416529	59416829	456582	-2.03	2.22E-03	1.01E-01	Distal Intergenic
Cds2	114101	chr3	124900849	124901149	4192	-1.95	2.22E-03	1.01E-01	Intron (NM_053643/114101, intron 1 of 12)
Cdk17	314743	chr7	34025373	34025673	23867	-2.15	2.22E-03	1.01E-01	Intron (NM_001108082/314743, intron 1 of 16)
Commd7	296285	chr3	148944538	148944838	170371	0.93	2.23E-03	1.01E-01	Distal Intergenic
Ptk2	25614	chr7	114726843	114727143	-136635	2.49	2.23E-03	1.01E-01	Distal Intergenic
RGD1305350	313699	chr5	164771874	164772174	20434	1.76	2.23E-03	1.01E-01	Distal Intergenic
Specc1	303208	chr10	48391702	48392002	151372	-2.01	2.23E-03	1.01E-01	Intron (NM_001039017/303208, intron 3 of 7)
Rnf130	652955	chr10	35553456	35553756	15480	-2.33	2.24E-03	1.01E-01	Intron (NM_001037658/652955, intron 1 of 8)
Mtss2	307845	chr19	40923054	40923354	2306	1.37	2.24E-03	1.01E-01	Promoter (2-3kb)
Ttc22	298300	chr5	126237183	126237483	10811	1.06	2.24E-03	1.01E-01	Exon (NM_001106671/298300, exon 2 of 7)
Smad2	29357	chr18	72357525	72357825	-192394	2.23	2.24E-03	1.01E-01	Intron (NM_001127375/679155, intron 3 of 5)
Pou2af1	690528	chr8	55742126	55742426	138158	-1.81	2.24E-03	1.01E-01	Distal Intergenic
Dok1	312477	chr4	113834324	113834624	32050	-1.94	2.24E-03	1.01E-01	Distal Intergenic
Hspa4	266759	chr10	38634415	38634715	7682	-2.12	2.24E-03	1.01E-01	Intron (NM_153629/266759, intron 1 of 18)
Sptbn1	305614	chr14	114651299	114651599	41165	-2.12	2.24E-03	1.01E-01	Intron (NM_001013130/305614, intron 1 of 36)

Zfp36l2	298765	chr6	7344892	7345192	76264	-2.12	2.24E-03	1.01E-01	Distal Intergenic
Thap4	363291	chr9	100876084	100876384	11723	-2.12	2.24E-03	1.01E-01	Intron (NM_001005564/363291, intron 2 of 5)
P2ry2	29597	chr1	166040531	166040831	4589	-2.06	2.25E-03	1.01E-01	Intron (NM_017255/29597, intron 1 of 3)
Myo1b	117057	chr9	54650237	54650537	92035	2.13	2.26E-03	1.01E-01	Intron (NM_053986/117057, intron 5 of 30)
Fgf5	60662	chr14	13199022	13199322	-203438	-1.68	2.26E-03	1.01E-01	Distal Intergenic
Amz2	360650	chr10	97722994	97723294	12089	1.3	2.26E-03	1.02E-01	Distal Intergenic
Arhgef3	290541	chr16	2961176	2961476	2893	-2.14	2.26E-03	1.02E-01	Promoter (2-3kb)
Cldn15	304388	chr12	22748477	22748777	0	2.85	2.27E-03	1.02E-01	Promoter (<=1kb)
Kyat1	311844	chr3	8757703	8758003	8430	2.14	2.27E-03	1.02E-01	Exon (NM_001013164/311844, exon 3 of 13)
Spen	690911	chr5	160056101	160056401	14514	-2.13	2.27E-03	1.02E-01	Intron (NM_001271495/690911, intron 2 of 14)
Atxn7l3b	100192313	chr7	55604098	55604398	5	0.69	2.27E-03	1.02E-01	Promoter (<=1kb)
Dtwd2	361326	chr18	43532242	43532542	412731	-2.05	2.27E-03	1.02E-01	Distal Intergenic
Abcc4	170924	chr15	103892955	103893255	34337	-2.15	2.28E-03	1.02E-01	Intron (NM_133411/170924, intron 1 of 36)
Naa15	310399	chr2	140492043	140492343	20353	-2.15	2.28E-03	1.02E-01	Intron (NM_001107674/310399, intron 1 of 19)
Ppp2r5e	299147	chr6	98506420	98506720	60246	-2.15	2.28E-03	1.02E-01	Intron (NM_001106740/299147, intron 2 of 11)
Ttc12	300696	chr8	53830031	53830331	-13578	-2.15	2.28E-03	1.02E-01	Distal Intergenic
Zfp365	499425	chr20	22106393	22106693	46233	-2.01	2.29E-03	1.02E-01	Distal Intergenic
Irak4	300177	chr7	135460815	135461115	-342608	-2.36	2.29E-03	1.02E-01	Distal Intergenic
Ubr2	363188	chr9	16018466	16018766	15408	2.58	2.30E-03	1.02E-01	Intron (NM_001178071/363188, intron 3 of 45)
Gemin6	362688	chr6	2915665	2915965	8520	2.67	2.30E-03	1.02E-01	Distal Intergenic
Pde2a	81743	chr1	166523858	166524158	-10446	-2.4	2.30E-03	1.02E-01	Distal Intergenic
Pik3r1	25513	chr2	31807011	31807311	9074	-2.4	2.30E-03	1.02E-01	Intron (NM_013005/25513, intron 1 of 14)
Eea1	314764	chr7	37151371	37151671	49957	-2.02	2.30E-03	1.02E-01	Intron (NM_001108086/314764, intron 13 of 27)
Crnkl1	100910202	chr3	140751350	140751650	-609671	-1.84	2.32E-03	1.03E-01	Distal Intergenic
Prag1	306506	chr16	59647234	59647534	73712	-2.16	2.32E-03	1.03E-01	Distal Intergenic
Arhgef19	362648	chr5	159761068	159761368	5533	1.11	2.32E-03	1.03E-01	Exon (NM_001108692/362648, exon 3 of 18)
Zbtb1	314246	chr6	99412331	99412631	-1801	-1.79	2.32E-03	1.03E-01	Promoter (1-2kb)
Mier2	362841	chr7	13040082	13040382	301	1.25	2.33E-03	1.03E-01	Promoter (<=1kb)
Stoml1	300748	chr8	63022569	63022869	-14287	-2.22	2.33E-03	1.03E-01	Distal Intergenic
Cd47	29364	chr11	53452609	53452909	122266	-1.99	2.33E-03	1.03E-01	Distal Intergenic
Ddit4l2	140582	chr2	242882398	242882698	0	1.17	2.34E-03	1.03E-01	Promoter (<=1kb)
Slc22a23	64559	chr17	31175952	31176252	-59763	-2.1	2.34E-03	1.03E-01	Distal Intergenic
Ppm1a	24666	chr6	94421659	94421959	-11702	-1.83	2.34E-03	1.03E-01	Distal Intergenic
Trdmt1	291324	chr17	80855131	80855431	4154	-1.69	2.34E-03	1.03E-01	Intron (NM_001031643/291324, intron 1 of 10)
Tgif1	316742	chr9	119084670	119084970	105728	-2.03	2.35E-03	1.03E-01	Intron (NM_022946/65040, intron 7 of 12)
Arhgef1	60323	chr1	81779881	81780181	10669	-2	2.35E-03	1.03E-01	Exon (NM_021694/60323, exon 4 of 29)
Stx7	60466	chr1	22278508	22278808	2980	-2	2.35E-03	1.03E-01	Promoter (2-3kb)
Tasp1	311468	chr3	132981283	132981583	141172	-2.1	2.35E-03	1.03E-01	Intron (NM_001044243/311468, intron 9 of 12)
Tradd	246756	chr19	37209899	37210199	6373	0.99	2.36E-03	1.03E-01	Distal Intergenic
LOC304725	304725	chr13	12984436	12984736	2410881	2.14	2.36E-03	1.03E-01	Distal Intergenic
Wdr53	498097	chr11	71796251	71796551	-455	1.65	2.37E-03	1.03E-01	Promoter (<=1kb)
Chd2	308738	chr1	134963730	134964030	-92562	-2	2.37E-03	1.03E-01	Distal Intergenic
Armt1	292267	chr1	41031356	41031656	48494	1.85	2.37E-03	1.03E-01	Distal Intergenic
Gatad2b	310614	chr2	189655193	189655493	-209	1.1	2.38E-03	1.04E-01	Promoter (<=1kb)
Sertad4	360899	chr13	111519521	111519821	64948	1.68	2.39E-03	1.04E-01	Distal Intergenic
Myc	24577	chr7	103027635	103027935	441322	-1.8	2.39E-03	1.04E-01	Distal Intergenic
Satb2	501145	chr9	63634228	63634528	3149	-2.24	2.39E-03	1.04E-01	Intron (NM_001109306/501145, intron 2 of 10)
Nr1h3	58852	chr3	80013829	80014129	-1086	1.85	2.39E-03	1.04E-01	Promoter (1-2kb)
Nfkbia	25493	chr6	76249960	76250260	20197	-1.9	2.40E-03	1.04E-01	Distal Intergenic
Smoc1	314280	chr6	104855691	104855991	137014	2.72	2.40E-03	1.04E-01	Intron (NM_001002835/314280, intron 7 of 12)
Mir551b	100314268	chr2	117262853	117263153	395206	-1.93	2.41E-03	1.04E-01	Distal Intergenic
Bach2	313125	chr5	47435263	47435563	-110451	2.41	2.41E-03	1.04E-01	Distal Intergenic
Itga11	315744	chr8	67570570	67570870	3371	1	2.41E-03	1.04E-01	Intron (NM_001108156/315744, intron 1 of 33)
Foxq1	64826	chr17	34320242	34320542	-93301	1.81	2.41E-03	1.04E-01	Distal Intergenic
Osr1	298878	chr6	35201952	35202252	-116849	-1.99	2.42E-03	1.04E-01	Distal Intergenic
Smug1	315344	chr7	144789436	144789736	-10783	1.23	2.42E-03	1.04E-01	Distal Intergenic
Lrpap1	116565	chr14	80911156	80911456	0	0.88	2.43E-03	1.05E-01	Promoter (<=1kb)
Uqcc2	361805	chr20	5730271	5730571	-6369	-2.14	2.44E-03	1.05E-01	Distal Intergenic
Mbnl1	282635	chr2	150748742	150749042	-7216	-1.62	2.44E-03	1.05E-01	Distal Intergenic
Pex14	64460	chr5	165731292	165731592	186853	1.84	2.44E-03	1.05E-01	Distal Intergenic
Lnx1	360926	chr14	35968424	35968724	-78420	-2.17	2.44E-03	1.05E-01	Distal Intergenic
N4bp1	291921	chr19	21252208	21252508	33867	-2.17	2.44E-03	1.05E-01	Intron (NM_001305181/291921, intron 2 of 6)
Ube2l6	295704	chr3	72185998	72186298	-5235	-2.17	2.44E-03	1.05E-01	Distal Intergenic
Trim63	140939	chr5	152526265	152526565	-6797	-2.17	2.44E-03	1.05E-01	Distal Intergenic
Ctnna1	307505	chr18	27934229	27934529	10657	-1.88	2.45E-03	1.05E-01	Intron (NM_001007145/307505, intron 1 of 17)
Heph	117240	chrX	65559830	65560130	-2992	2.01	2.45E-03	1.05E-01	Promoter (2-3kb)
Haao	56823	chr6	7207526	7207826	-149212	-2.01	2.45E-03	1.05E-01	Distal Intergenic
RGD1359290	360649	chr10	95646309	95646609	11603	-2.12	2.46E-03	1.05E-01	Intron (NM_001047898/360649, intron 5 of 5)
Spaca4	691120	chr1	101731728	101732028	-23442	-1.85	2.46E-03	1.05E-01	Intron (NM_001039665/292915, intron 1 of 6)
Acox3	83522	chr14	80311832	80312132	43288	2.57	2.46E-03	1.05E-01	Downstream (2-3kb)
Hsph1	288444	chr12	6313052	6313352	28546	-2.1	2.46E-03	1.05E-01	Distal Intergenic
Sdk2	360652	chr10	102724180	102724480	-301096	1.54	2.46E-03	1.05E-01	Distal Intergenic
Pspc1	305910	chr15	37160152	37160452	-129932	-2.29	2.47E-03	1.05E-01	Distal Intergenic
Fgf5	60662	chr14	13346394	13346694	-350810	-1.99	2.47E-03	1.05E-01	Distal Intergenic
Pgm2	289632	chr14	45718570	45718870	145002	-1.92	2.47E-03	1.05E-01	Distal Intergenic
Mdm1	314859	chr7	61057731	61058031	-116338	-1.79	2.47E-03	1.05E-01	Distal Intergenic
Zswim3	311630	chr3	161267305	161267605	-4780	-1.93	2.48E-03	1.05E-01	Exon (NM_130756/170588, exon 3 of 6)
Fbxw17	361219	chr17	15094320	15094620	0	0.82	2.49E-03	1.06E-01	Promoter (<=1kb)
Ilf3	84472	chr8	22398912	22399212	-14107	-2.41	2.49E-03	1.06E-01	Distal Intergenic
Rnf139	315000	chr7	98770897	98771197	57	0.97	2.50E-03	1.06E-01	Promoter (<=1kb)
Retreg1	619558	chr2	78107735	78108035	4280	-2.2	2.51E-03	1.06E-01	Intron (NM_001034912/619558, intron 1 of 8)
Tprg1	360731	chr11	79115110	79115410	-253365	-1.45	2.51E-03	1.06E-01	Distal Intergenic
Lims1	499443	chr20	27940370	27940670	-13897	-1.66	2.52E-03	1.06E-01	Distal Intergenic
Miip	298643	chr5	164430125	164430425	125218	-1.84	2.52E-03	1.06E-01	Distal Intergenic
Ppp1r15b	304799	chr13	49904796	49905096	-28059	-2.13	2.52E-03	1.07E-01	Exon (NM_001105951/289021, exon 28 of 32)
Antxr1	362393	chr4	119130572	119130872	330	0.94	2.53E-03	1.07E-01	Promoter (<=1kb)
Pskh1	364993	chr19	37879856	37880156	6341	-1.55	2.53E-03	1.07E-01	Intron (NM_001108897/364993, intron 1 of 2)
Mrpl14	301250	chr9	17643589	17643889	49311	-1.66	2.54E-03	1.07E-01	Distal Intergenic
Kalrn	84009	chr11	69450239	69450539	-33754	-1.97	2.54E-03	1.07E-01	Distal Intergenic
Csgalnact2	297554	chr4	150184730	150185030	14	0.87	2.54E-03	1.07E-01	Promoter (<=1kb)
Bace1	29392	chr8	50057858	50058158	-81934	-1.7	2.54E-03	1.07E-01	Distal Intergenic
Mfsd12	362824	chr7	11219619	11219919	3730	-1.52	2.55E-03	1.07E-01	Exon (NM_001108730/362824, exon 4 of 10)
Cul1	362356	chr4	77102861	77103161	-108653	2.39	2.55E-03	1.07E-01	Distal Intergenic
Med27	296612	chr3	7882977	7883277	-1545	-1.92	2.55E-03	1.07E-01	Promoter (1-2kb)

Wdr89	314243	chr6	98687415	98687715	-81306	-1.86	2.56E-03	1.07E-01	Distal Intergenic
Paqr3	305203	chr14	14214063	14214363	106483	-1.85	2.57E-03	1.07E-01	Distal Intergenic
Col16a1	366474	chr5	148286914	148287214	29321	2.4	2.57E-03	1.07E-01	Intron (NM_001302967/366474, intron 42 of 69)
LOC365238	365238	chr1	94880162	94880462	-95711	1.01	2.57E-03	1.07E-01	Distal Intergenic
Dlx2	296499	chr3	58284300	58284600	-102329	1.86	2.58E-03	1.07E-01	Distal Intergenic
Cdc27	360643	chr10	92595171	92595471	6611	-2.06	2.58E-03	1.07E-01	Intron (NM_001024793/360643, intron 1 of 18)
Nedd4l	291553	chr18	60553207	60553507	160831	-1.69	2.58E-03	1.07E-01	Intron (NM_001008300/291553, intron 2 of 31)
Runx3	156726	chr5	153448249	153448549	-58540	1.61	2.58E-03	1.07E-01	Distal Intergenic
Sfxn4	361778	chr1	282220231	282220531	15360	2.02	2.58E-03	1.07E-01	Intron (NM_001108527/361778, intron 11 of 13)
Cavin4	313225	chr5	64413181	64413481	176	0.98	2.58E-03	1.07E-01	Promoter (<=1kb)
Nin	299117	chr6	92529098	92529398	-1387	2.76	2.58E-03	1.07E-01	Promoter (1-2kb)
Tacc2	309025	chr1	201222102	201222402	81049	-1.91	2.58E-03	1.07E-01	Intron (NM_001004415/309025, intron 4 of 21)
Elavl1	363854	chr12	2475443	2475743	13941	-2.07	2.58E-03	1.07E-01	Intron (NM_001108848/363854, intron 1 of 5)
Aff1	305152	chr14	7289551	7289851	35794	2.35	2.58E-03	1.07E-01	Intron (NM_001107206/305152, intron 3 of 19)
Cap1	64185	chr5	140573798	140574098	11310	-1.76	2.59E-03	1.08E-01	Intron (NM_022383/64185, intron 1 of 12)
Etv3	295297	chr2	186858471	186858771	-15464	-1.75	2.60E-03	1.08E-01	Distal Intergenic
Tcf4	84382	chr18	65223763	65224063	-61257	-2.12	2.60E-03	1.08E-01	Distal Intergenic
Sel1l2	311470	chr3	134128635	134128935	277625	-1.76	2.61E-03	1.08E-01	Distal Intergenic
Ucp3	25708	chr1	165445391	165445691	-37221	-1.88	2.61E-03	1.08E-01	Intron (NM_001191602/293148, intron 24 of 32)
Npr2	116564	chr5	59128328	59128628	-275	1.16	2.61E-03	1.08E-01	Promoter (<=1kb)
Shq1	297483	chr4	133187445	133187745	-60270	1.32	2.62E-03	1.08E-01	Distal Intergenic
Angpt2	89805	chr16	75978816	75979116	12336	2.46	2.63E-03	1.08E-01	Intron (NM_134454/89805, intron 1 of 8)
Anxa7	155423	chr15	4257747	4258047	3248	-1.68	2.63E-03	1.08E-01	Intron (NM_130416/155423, intron 1 of 12)
Armc3	100361506	chr17	85917761	85918061	3174	-1.17	2.63E-03	1.08E-01	Intron (NM_001287020/100361506, intron 2 of 18)
Tenm3	306451	chr16	44711801	44712101	-1711768	2.36	2.63E-03	1.08E-01	Distal Intergenic
Acat2	308100	chr1	47957651	47957951	-14448	-1.92	2.64E-03	1.08E-01	Intron (NM_001113543/499020, intron 5 of 5)
Adam34	685846	chr16	51330047	51330347	-420443	-2.13	2.64E-03	1.09E-01	Distal Intergenic
Ttc7b	362768	chr6	124517635	124517935	-83571	1.83	2.65E-03	1.09E-01	Distal Intergenic
Pawr	64513	chr7	51308751	51309051	44017	1.61	2.66E-03	1.09E-01	Intron (NM_033485/64513, intron 1 of 5)
RGD1562146	500612	chr6	9625034	9625334	134834	2.21	2.66E-03	1.09E-01	Intron (NM_017171/29340, intron 1 of 13)
Lbx1	499362	chr1	264976031	264976331	-899	2.09	2.67E-03	1.09E-01	Promoter (<=1kb)
Ccl5	81780	chr10	70761356	70761656	-17053	-1.95	2.67E-03	1.09E-01	Distal Intergenic
Trps1	299897	chr7	90206618	90206918	111303	-1.95	2.67E-03	1.09E-01	Intron (NM_001134837/299897, intron 5 of 6)
Ern1	498013	chr10	94670913	94671213	-18255	-2.11	2.68E-03	1.09E-01	Distal Intergenic
Ugcg	83626	chr5	76506948	76507248	120110	-2.11	2.68E-03	1.09E-01	Distal Intergenic
Hmg20a	315689	chr8	61085840	61086140	5737	-1.79	2.69E-03	1.10E-01	Intron (NM_001108150/315689, intron 1 of 9)
Bbox1	64564	chr3	101340813	101341113	124882	1.71	2.69E-03	1.10E-01	Distal Intergenic
Hace1	361866	chr20	51034447	51034747	381702	2.52	2.70E-03	1.10E-01	Distal Intergenic
Cttnbp2nl	310760	chr2	207530284	207530584	11058	-2.04	2.70E-03	1.10E-01	Intron (NM_001107712/310760, intron 1 of 5)
Mapk1	116590	chr11	88266234	88266534	6760	-1.67	2.71E-03	1.10E-01	Intron (NM_053842/116590, intron 1 of 8)
Slc35b4	296969	chr4	61613609	61613909	-40606	-2.16	2.71E-03	1.10E-01	Distal Intergenic
Bst2	378947	chr16	19939555	19939855	2498	-1.96	2.72E-03	1.10E-01	Promoter (2-3kb)
Myo10	310178	chr2	77902263	77902563	33831	-2.07	2.72E-03	1.10E-01	Intron (NM_001107657/310178, intron 1 of 39)
Oasl2	304549	chr12	47487444	47487744	-4497	-2.01	2.72E-03	1.10E-01	Distal Intergenic
LOC500350	500350	chr4	170426686	170426986	79276	-1.46	2.73E-03	1.10E-01	Distal Intergenic
Kif13a	308173	chr17	18151689	18151989	0	0.88	2.73E-03	1.10E-01	Promoter (<=1kb)
Dusp8	361679	chr1	215037755	215038055	5056	-1.59	2.74E-03	1.10E-01	Intron (NM_001108510/361679, intron 2 of 5)
Ext1	299907	chr7	92907842	92908142	-26450	2.16	2.74E-03	1.10E-01	Distal Intergenic
Arhgap35	306400	chr1	78555657	78555957	17417	-1.3	2.74E-03	1.10E-01	Intron (NM_001271132/306400, intron 1 of 6)
Fgfr2	25022	chr1	200620531	200620831	76097	-1.88	2.74E-03	1.10E-01	Intron (NM_001109892/25022, intron 9 of 17)
Ywhaq	25577	chr6	43547560	43547860	-53744	-1.67	2.74E-03	1.10E-01	Distal Intergenic
Aebp1	305494	chr14	86091054	86091354	-9978	2.44	2.74E-03	1.10E-01	Distal Intergenic
Max	60661	chr6	100083196	100083496	-71970	-2.09	2.74E-03	1.10E-01	Distal Intergenic
Oat	64313	chr1	204443748	204444048	138022	-1.98	2.74E-03	1.10E-01	Distal Intergenic
Nfix	81524	chr19	25908058	25908358	6338	1.47	2.75E-03	1.10E-01	Intron (NM_030866/81524, intron 1 of 9)
Pla2g4a	24653	chr13	67090938	67091238	115450	-2.19	2.75E-03	1.10E-01	Exon (NM_133551/24653, exon 14 of 18)
Herpud1	85430	chr19	10991725	10992025	65229	-2.19	2.75E-03	1.10E-01	Distal Intergenic
Fbxl20	64039	chr10	86088612	86088912	9682	1.6	2.75E-03	1.10E-01	Intron (NM_022272/64039, intron 1 of 9)
Msx3	114504	chr1	212516644	212516944	207	-1.61	2.76E-03	1.11E-01	Promoter (<=1kb)
Cert1	365652	chr2	27368877	27369177	3729	-1.96	2.76E-03	1.11E-01	Intron (NM_001108935/365652, intron 1 of 17)
Nav2	171563	chr1	104477836	104478136	-98453	-1.81	2.76E-03	1.11E-01	Distal Intergenic
Kmt2e	311968	chr4	8247065	8247365	8213	-1.79	2.76E-03	1.11E-01	Intron (NM_001100851/311968, intron 1 of 26)
Mir29b1	100314008	chr4	58480625	58480925	-136235	-1.74	2.77E-03	1.11E-01	Distal Intergenic
Trdmt1	291324	chr17	80856480	80856780	2805	-1.78	2.77E-03	1.11E-01	Promoter (2-3kb)
Elovl5	171400	chr8	85230667	85230967	-29021	-1.91	2.78E-03	1.11E-01	Distal Intergenic
Sohlh1	362085	chr3	3294665	3294965	136	-1.49	2.78E-03	1.11E-01	Promoter (<=1kb)
Akt1	24185	chr6	137230190	137230490	5711	-1.94	2.79E-03	1.11E-01	Intron (NM_033230/24185, intron 1 of 12)
Trmt44	305443	chr14	80380612	80380912	19090	2.25	2.79E-03	1.11E-01	Distal Intergenic
Apobec3	315137	chr7	121116874	121117174	7624	-1.95	2.79E-03	1.11E-01	Intron (NM_001033703/315137, intron 4 of 7)
Asap1	314961	chr7	104822708	104823008	128082	-1.51	2.80E-03	1.11E-01	Intron (NM_001044245/314961, intron 1 of 27)
RGD1562310	498188	chr12	43507276	43507576	433222	-1.25	2.80E-03	1.11E-01	Distal Intergenic
Ctf1	29201	chr1	199163132	199163432	46	0.9	2.80E-03	1.11E-01	Promoter (<=1kb)
Mir568	100314178	chr11	62171378	62171678	-175256	-1.31	2.80E-03	1.11E-01	Intron (NM_001105880/288105, intron 5 of 10)
Bend6	363212	chr9	38120967	38121267	-175986	2.26	2.81E-03	1.11E-01	Distal Intergenic
Smtn	289734	chr14	83799092	83799392	-22229	-1.51	2.81E-03	1.11E-01	Distal Intergenic
Tor1aip1	246314	chr13	73703854	73704154	514	0.94	2.81E-03	1.11E-01	Promoter (<=1kb)
Myc	24577	chr7	102749999	102750299	163686	-1.9	2.82E-03	1.11E-01	Distal Intergenic
Napa	140673	chr1	77944197	77944497	-49730	0.71	2.82E-03	1.11E-01	Distal Intergenic
Kat7	303470	chr10	83112660	83112960	15382	-2	2.82E-03	1.11E-01	Intron (NM_181081/303470, intron 5 of 12)
Ide	25700	chr1	256001676	256001976	11519	-2.13	2.82E-03	1.11E-01	Intron (NM_013159/25700, intron 1 of 24)
Ier2	494344	chr19	25700032	25700332	75338	-2.07	2.82E-03	1.11E-01	Exon (NM_012918/25398, exon 30 of 47)
Dld	298942	chr6	50624175	50624475	-5481	2.41	2.83E-03	1.12E-01	Distal Intergenic
Ube3d	315863	chr8	94098849	94099149	21309	-1.72	2.83E-03	1.12E-01	Intron (NM_001039610/315863, intron 1 of 10)
Chst10	140568	chr9	45529584	45529884	29256	1.49	2.84E-03	1.12E-01	3' UTR
Six1	114634	chr6	95954907	95955207	-20611	2.4	2.84E-03	1.12E-01	Distal Intergenic
LOC499643	499643	chr2	178211838	178212138	94355	-2.26	2.84E-03	1.12E-01	Intron (NM_001024301/499643, intron 3 of 4)
Snx27	260323	chr2	195785222	195785522	36086	-2.26	2.84E-03	1.12E-01	Intron (NM_001110151/260323, intron 2 of 11)
Stxbp2	81804	chr12	2180043	2180343	0	1.15	2.84E-03	1.12E-01	Promoter (<=1kb)
Pdgfa	25266	chr12	17718311	17718611	-15530	-1.85	2.85E-03	1.12E-01	Distal Intergenic
Cryab	25420	chr8	55178799	55179099	256	1.04	2.85E-03	1.12E-01	Promoter (<=1kb)
Prr3	361788	chr20	3303420	3303720	3173	2.6	2.85E-03	1.12E-01	Intron (NM_212544/361788, intron 2 of 3)
Grem1	50566	chr3	104900437	104900737	314252	0.73	2.86E-03	1.12E-01	Distal Intergenic
Tax1bp1	246244	chr4	82906500	82906800	129754	1.43	2.86E-03	1.12E-01	Distal Intergenic
RGD1562310	498188	chr12	43595119	43595419	345379	-2.13	2.86E-03	1.12E-01	Distal Intergenic

Eea1	314764	chr7	37337187	37337487	235773	-1.9	2.87E-03	1.12E-01	Distal Intergenic
Fzd1	58868	chr4	26471004	26471304	140	1.28	2.87E-03	1.12E-01	Promoter (<=1kb)
C1qtnf6	315114	chr7	119752206	119752506	160	0.82	2.87E-03	1.12E-01	Promoter (<=1kb)
Shisa3	498356	chr14	42005396	42005696	215529	-1.76	2.87E-03	1.12E-01	Distal Intergenic
Tmem30c	288175	chr11	45262089	45262389	-75129	2.19	2.87E-03	1.12E-01	Intron (NM_001013866/288176, intron 1 of 9)
Tbc1d2	313234	chr5	62369761	62370061	-109672	2.63	2.88E-03	1.12E-01	Intron (NM_031802/83633, intron 7 of 19)
Senp18	408222	chr4	160704925	160705225	98333	2.33	2.88E-03	1.12E-01	Distal Intergenic
Psmd8	292766	chr1	88123972	88124272	-329	-1.34	2.89E-03	1.12E-01	Promoter (<=1kb)
Pias1	300772	chr8	67865305	67865605	3410	-1.96	2.89E-03	1.12E-01	Intron (NM_001106829/300772, intron 1 of 13)
Sumo3	499417	chr20	11736647	11736947	103	-1.9	2.89E-03	1.12E-01	Promoter (<=1kb)
Pspc1	305910	chr15	37156717	37157017	-126497	1.14	2.90E-03	1.13E-01	Distal Intergenic
Angel2	305035	chr13	109504679	109504979	-718	-1.65	2.90E-03	1.13E-01	Promoter (<=1kb)
Nckap1	58823	chr3	67787840	67788140	16540	-1.75	2.91E-03	1.13E-01	Exon (NM_031618/58823, exon 2 of 30)
Dlg4	29495	chr10	56638289	56638589	10742	-2.06	2.91E-03	1.13E-01	Intron (NM_019621/29495, intron 2 of 19)
Svil	361256	chr17	55475517	55475817	-129238	1.64	2.92E-03	1.13E-01	Distal Intergenic
Fam43a	288031	chr11	73537234	73537534	141469	-1.49	2.93E-03	1.13E-01	Distal Intergenic
Trim13	364398	chr15	41925514	41925814	-1427	2.48	2.93E-03	1.13E-01	Promoter (1-2kb)
Canx	29144	chr10	35826691	35826991	6870	-1.83	2.94E-03	1.13E-01	Intron (NM_172008/29144, intron 1 of 14)
Mkln1	83536	chr4	58663102	58663402	-29982	-1.67	2.94E-03	1.14E-01	Distal Intergenic
Gpam	29653	chr1	275615071	275615371	291315	-1.63	2.94E-03	1.14E-01	Distal Intergenic
Nav2	171563	chr1	104692734	104693034	116145	1.93	2.95E-03	1.14E-01	Intron (NM_138529/171563, intron 1 of 37)
Smad1	25671	chr19	32211409	32211709	23134	-1.84	2.95E-03	1.14E-01	Intron (NM_013130/25671, intron 1 of 6)
Parn	360464	chr10	1432888	1433188	27935	-1.93	2.95E-03	1.14E-01	Intron (NM_001270415/360464, intron 12 of 24)
Mrpl42	299743	chr7	36597100	36597400	62	0.75	2.97E-03	1.14E-01	Promoter (<=1kb)
Kdm4a	313539	chr5	137044493	137044793	-30118	2.45	2.97E-03	1.14E-01	Intron (NM_019249/360406, intron 20 of 30)
Lhfpl2	294643	chr2	23743794	23744094	-26614	-1.9	2.98E-03	1.14E-01	Distal Intergenic
Rprd1a	291736	chr18	16492958	16493258	4655	-1.87	2.98E-03	1.14E-01	Intron (NM_001305179/291736, intron 1 of 6)
Slc38a6	299139	chr6	96206180	96206480	34424	-1.97	2.98E-03	1.14E-01	Intron (NM_001013099/299139, intron 5 of 15)
Lctl	315750	chr8	69031936	69032236	-41679	1.02	2.98E-03	1.14E-01	Distal Intergenic
Csrp1	29276	chr13	52483664	52483964	-69879	-1.89	2.98E-03	1.14E-01	Distal Intergenic
Ripk4	304053	chr11	38275124	38275424	-890	-1.93	2.99E-03	1.14E-01	Promoter (<=1kb)
Rpl35al1	57809	chr5	164935486	164935786	12185	-1.18	2.99E-03	1.14E-01	Distal Intergenic
Ropn1	288053	chr11	69375352	69375652	-19564	-2.01	2.99E-03	1.14E-01	Distal Intergenic
Tdp2	498749	chr17	42238193	42238493	2532	-2.01	2.99E-03	1.14E-01	Promoter (2-3kb)
Gdpd2	302421	chrX	70563878	70564178	369	2.39	3.01E-03	1.15E-01	Promoter (<=1kb)
Pdgfrb	24629	chr18	56358919	56359219	-5458	-1.54	3.01E-03	1.15E-01	Distal Intergenic
Ppp4r1	140943	chr9	113520231	113520531	4940	-1.94	3.01E-03	1.15E-01	Intron (NM_080907/140943, intron 3 of 20)
Ptprf	360406	chr5	137123908	137124208	-18915	1.76	3.01E-03	1.15E-01	Distal Intergenic
Kpna3	361055	chr15	41822210	41822510	-52098	-2.66	3.02E-03	1.15E-01	Distal Intergenic
Angptl2	171100	chr3	12263182	12263482	360	0.73	3.03E-03	1.15E-01	Promoter (<=1kb)
Arglu1	290912	chr16	86094185	86094485	-506392	1.39	3.03E-03	1.15E-01	Distal Intergenic
Plin5	501283	chr9	10963686	10963986	7630	1.81	3.04E-03	1.16E-01	Distal Intergenic
Ubald1	302941	chr10	11291231	11291531	-46775	-2	3.04E-03	1.16E-01	Distal Intergenic
Tnnt2	24837	chr13	52664365	52664665	-3304	1.63	3.05E-03	1.16E-01	Distal Intergenic
Galnt1	79214	chr18	16086916	16087216	-59231	-1.98	3.05E-03	1.16E-01	Distal Intergenic
Aldh3a1	25375	chr10	47490405	47490705	237	1.27	3.05E-03	1.16E-01	Promoter (<=1kb)
Parvb	362973	chr7	125189857	125190157	30	1.21	3.06E-03	1.16E-01	Promoter (<=1kb)
Slc12a9	171443	chr12	22417751	22418051	-16794	1.15	3.06E-03	1.16E-01	Distal Intergenic
Golga2	64528	chr3	11348155	11348455	30827	0.87	3.06E-03	1.16E-01	Intron (NM_080689/140694, intron 14 of 21)
Pcdh9	306091	chr15	77738423	77738723	-1648	1.84	3.06E-03	1.16E-01	Promoter (1-2kb)
Cmklr1	60669	chr12	48779177	48779477	-9784	0.95	3.07E-03	1.16E-01	Distal Intergenic
Stk11	314621	chr7	12450621	12450921	6592	-1.89	3.08E-03	1.16E-01	Intron (NM_001108069/314621, intron 1 of 9)
Eif2b5	192234	chr11	84104312	84104612	-14053	-1.95	3.08E-03	1.16E-01	Distal Intergenic
Shq1	297483	chr4	133179299	133179599	-52124	1.81	3.08E-03	1.16E-01	Distal Intergenic
Mfap1a	499878	chr3	113510379	113510679	-64944	2.2	3.08E-03	1.16E-01	Distal Intergenic
Ubl3	363869	chr12	7885221	7885521	19283	-1.91	3.08E-03	1.16E-01	Intron (NM_001015030/363869, intron 1 of 4)
Kifap3	289168	chr13	81962564	81962864	-109633	1.75	3.09E-03	1.16E-01	Distal Intergenic
Cdca4	500727	chr6	137413893	137414193	7090	-1.13	3.10E-03	1.16E-01	Intron (NM_001037214/500727, intron 1 of 1)
Gbp2	171164	chr2	248279336	248279636	2541	-2.31	3.10E-03	1.17E-01	Promoter (2-3kb)
Nfix	81524	chr19	25874257	25874557	40139	2.66	3.11E-03	1.17E-01	Intron (NM_030866/81524, intron 2 of 9)
Atg10	688555	chr2	20249015	20249315	-96131	-1.79	3.11E-03	1.17E-01	Distal Intergenic
Spast	362700	chr6	22267788	22268088	13798	-2.41	3.12E-03	1.17E-01	Intron (NM_001108702/362700, intron 2 of 15)
Atp6v0d1	291969	chr19	37503304	37503604	22158	2.95	3.12E-03	1.17E-01	Intron (NM_001011927/291969, intron 1 of 7)
Cwc15	300361	chr8	13041644	13041944	47489	2.07	3.12E-03	1.17E-01	Distal Intergenic
Tlr2	310553	chr2	182868587	182868887	-22526	-1.95	3.12E-03	1.17E-01	Distal Intergenic
Amotl2	65157	chr8	111224651	111224951	13840	1.2	3.12E-03	1.17E-01	3' UTR
Fzd8	364754	chr17	61696150	61696450	-565679	1.82	3.12E-03	1.17E-01	Distal Intergenic
Padi6	298595	chr5	159214196	159214496	42203	1.01	3.13E-03	1.17E-01	Distal Intergenic
Cmss1	288176	chr11	45104194	45104494	72497	-2	3.13E-03	1.17E-01	Intron (NM_001013866/288176, intron 1 of 9)
Elf4	302811	chrX	135284191	135284491	-33672	1.14	3.15E-03	1.17E-01	Distal Intergenic
Arhgap22	306279	chr16	9595002	9595302	31784	1.23	3.15E-03	1.17E-01	Intron (NM_001107297/306279, intron 2 of 5)
Myl1	56781	chr9	73945614	73945914	2669	-1.79	3.16E-03	1.17E-01	Promoter (2-3kb)
Aqp1	25240	chr4	85491132	85491432	-60071	-1.76	3.16E-03	1.17E-01	Distal Intergenic
Kctd7	688993	chr12	30065507	30065807	-32150	2	3.16E-03	1.17E-01	Intron (NM_001011903/288617, intron 3 of 5)
Pid1	501174	chr9	91779702	91780002	-96234	-1.75	3.16E-03	1.18E-01	Distal Intergenic
Kifc3	307644	chr19	10247558	10247858	-72746	-1.88	3.17E-03	1.18E-01	Distal Intergenic
Ankrd13c	685374	chr2	264764045	264764345	-21897	-1.31	3.17E-03	1.18E-01	Distal Intergenic
Fam49b	299909	chr7	104761744	104762044	-119949	-1.79	3.17E-03	1.18E-01	Intron (NM_001044245/314961, intron 3 of 27)
Zfp7	315101	chr7	118446988	118447288	-41210	-1.22	3.17E-03	1.18E-01	Distal Intergenic
Hes1	29577	chr11	74320506	74320806	-5257	2.09	3.19E-03	1.18E-01	Distal Intergenic
Ncoa4	619385	chr16	8326183	8326483	16944	-1.99	3.19E-03	1.18E-01	Intron (NM_019352/54312, intron 6 of 6)
Atg14	305831	chr15	24336411	24336711	38065	2.51	3.19E-03	1.18E-01	Distal Intergenic
Fstl3	114031	chr7	12810280	12810580	0	1.18	3.19E-03	1.18E-01	Promoter (<=1kb)
Brf2	306542	chr16	69111268	69111568	21313	-1.57	3.21E-03	1.18E-01	Distal Intergenic
Igfbp7	289560	chr14	33019611	33019911	9311	1.51	3.21E-03	1.18E-01	Intron (NM_001013048/289560, intron 1 of 4)
Raph1	363239	chr9	67306525	67306825	63383	1.83	3.21E-03	1.18E-01	Exon (NM_001108798/363239, exon 9 of 15)
Eef1e1	291057	chr17	26770857	26771157	-13860	-2.22	3.22E-03	1.19E-01	Distal Intergenic
Tmem37	245953	chr13	36110719	36111019	-9606	2.67	3.23E-03	1.19E-01	Distal Intergenic
Kalrn	84009	chr11	69528611	69528911	44318	-1.85	3.23E-03	1.19E-01	Intron (NM_032062/84009, intron 1 of 59)
Ptafr	58949	chr5	150746699	150746999	-7022	1.58	3.23E-03	1.19E-01	Distal Intergenic
Dmac1	298147	chr5	93365300	93365600	-1326154	1.35	3.24E-03	1.19E-01	Distal Intergenic
Shq1	297483	chr4	133195120	133195420	-67945	2.51	3.24E-03	1.19E-01	Distal Intergenic
PCOLCE2	684050	chr8	103445787	103446087	-13132	-1.39	3.24E-03	1.19E-01	Distal Intergenic
Pdgfa	25266	chr12	17792411	17792711	58270	-1.81	3.24E-03	1.19E-01	Distal Intergenic

Olr63	365323	chr1	167849570	167849870	-20582	1	3.25E-03	1.19E-01	Distal Intergenic
Ehd4	192204	chr3	111934505	111934805	7036	-1.95	3.25E-03	1.19E-01	Intron (NM_139324/192204, intron 1 of 5)
RGD1308706	291925	chr19	22705991	22706291	6183	-1.72	3.25E-03	1.19E-01	Exon (NM_001134421/291925, exon 2 of 4)
Srgap3	500287	chr4	144789330	144789630	79495	2.19	3.25E-03	1.19E-01	Intron (NM_001191975/500287, intron 1 of 21)
Mir222	100314059	chrX	3683083	3683383	-536	-2.06	3.26E-03	1.19E-01	Promoter (<=1kb)
Kpnb1	24917	chr10	85158914	85159214	-34273	2.35	3.26E-03	1.19E-01	Intron (NM_080395/50558, intron 16 of 22)
Slc20a2	29502	chr16	74403626	74403926	4104	-1.97	3.26E-03	1.19E-01	Intron (NM_017223/29502, intron 1 of 10)
Prkar1b	25521	chr12	17665623	17665923	51058	-1.19	3.27E-03	1.19E-01	Intron (NM_001033679/25521, intron 3 of 10)
Lppos	106455137	chr11	79988785	79989085	161057	2.07	3.27E-03	1.19E-01	Distal Intergenic
Iqsec2	685244	chrX	22206442	22206742	-5395	1.31	3.27E-03	1.19E-01	Distal Intergenic
Glyatl3	688536	chr9	23389844	23390144	10644	-1.88	3.27E-03	1.19E-01	Exon (NM_001145062/688536, exon 5 of 5)
Pdxk	83578	chr20	10937398	10937698	6747	2.15	3.27E-03	1.19E-01	Intron (NM_031769/83578, intron 1 of 10)
Ptprq	360417	chr7	50081723	50082023	-46791	-1.58	3.27E-03	1.19E-01	Distal Intergenic
Swap70	293410	chr1	174911080	174911380	48355	2.1	3.28E-03	1.19E-01	Intron (NM_001106288/293410, intron 7 of 11)
Rbp7	362662	chr5	166271806	166272106	10726	-1.82	3.29E-03	1.19E-01	Distal Intergenic
Ptbp3	83515	chr5	76735349	76735649	20491	-1.95	3.29E-03	1.19E-01	Intron (NM_031346/83515, intron 1 of 13)
Ndufv2	81728	chr9	113869229	113869529	30640	-1.74	3.29E-03	1.19E-01	Distal Intergenic
Socs3	89829	chr10	106992050	106992350	-16010	-1.99	3.30E-03	1.20E-01	Distal Intergenic
Luc7l	360503	chr10	15530801	15531101	-9667	-2.03	3.30E-03	1.20E-01	Distal Intergenic
Psma6	29673	chr6	76224251	76224551	36165	-1.96	3.31E-03	1.20E-01	Distal Intergenic
Fut8	432392	chr6	100310068	100310368	-86685	-1.82	3.31E-03	1.20E-01	Distal Intergenic
Bicd1	362466	chr4	183883754	183884054	-135033	-2.06	3.31E-03	1.20E-01	Distal Intergenic
Lyl1	304663	chr19	25845629	25845929	30422	2.1	3.31E-03	1.20E-01	Intron (NM_030866/81524, intron 2 of 9)
Dab2ip	192126	chr3	15013391	15013691	124128	-1.77	3.31E-03	1.20E-01	Intron (NM_138710/192126, intron 3 of 15)
Bbx	303970	chr11	52524132	52524432	-556593	-1.81	3.32E-03	1.20E-01	Distal Intergenic
Phf14	500030	chr4	38636944	38637244	381762	1.8	3.33E-03	1.20E-01	Distal Intergenic
Abcg3	498327	chr14	5913636	5913936	-58048	-1.38	3.33E-03	1.20E-01	Intron (NM_001037205/360997, intron 9 of 15)
Ubr1	499877	chr3	112902126	112902426	7612	-1.78	3.34E-03	1.20E-01	Intron (NM_001178072/499877, intron 1 of 47)
Rai14	294804	chr2	60652753	60653053	30398	-1.59	3.34E-03	1.20E-01	Intron (NM_001011947/294804, intron 2 of 16)
Lrch1	502020	chr15	56934196	56934496	35869	-1.29	3.34E-03	1.20E-01	Intron (NM_001134727/502020, intron 1 of 18)
Layn	500996	chr8	55450567	55450867	16712	-1.08	3.34E-03	1.20E-01	Exon (NM_001191997/500996, exon 8 of 8)
Traf3ip3	360900	chr13	111905577	111905877	11491	-1.65	3.34E-03	1.20E-01	Intron (NM_001014132/360900, intron 7 of 14)
Rxfp2	363866	chr12	6091518	6091818	-13107	1.16	3.34E-03	1.20E-01	Distal Intergenic
Epn2	60443	chr10	47842020	47842320	15030	-2.1	3.35E-03	1.20E-01	Intron (NM_001033914/60443, intron 1 of 9)
Atp2b1	29598	chr7	41119129	41119429	4432	-1.91	3.35E-03	1.20E-01	Intron (NM_053311/29598, intron 1 of 21)
Sugct	361253	chr17	50604489	50604789	613175	2.11	3.35E-03	1.20E-01	Intron (NM_001014146/361253, intron 10 of 12)
Cldn24	502083	chr16	47596694	47596994	-59218	1.88	3.36E-03	1.20E-01	Distal Intergenic
Extl3	56819	chr15	48435823	48436123	9469	-1.92	3.36E-03	1.20E-01	Intron (NM_020097/56819, intron 1 of 4)
Nob1	291996	chr19	38385508	38385808	-11658	1.23	3.36E-03	1.20E-01	Distal Intergenic
Prune1	310664	chr2	196451573	196451873	4673	-1.67	3.36E-03	1.20E-01	Intron (NM_001024302/499671, intron 3 of 3)
Mef2a	309957	chr1	128302393	128302693	38570	-1.89	3.37E-03	1.20E-01	Intron (NM_001014035/309957, intron 2 of 10)
Pgam1	24642	chr1	261081041	261081341	-76863	-1.81	3.37E-03	1.20E-01	Distal Intergenic
Tlr2	310553	chr2	182786746	182787046	59015	-1.89	3.37E-03	1.20E-01	Distal Intergenic
Rcbtb2	290363	chr15	55005083	55005383	-28650	2.37	3.37E-03	1.20E-01	Distal Intergenic
Thoc7	305714	chr15	12514422	12514722	-54513	2.76	3.37E-03	1.20E-01	Distal Intergenic
Ppp1r15b	304799	chr13	49906429	49906729	-26426	-1.65	3.37E-03	1.20E-01	Intron (NM_001105951/289021, intron 30 of 31)
Edem1	297504	chr4	140901931	140902231	15287	1.62	3.38E-03	1.20E-01	Intron (NM_001305279/297504, intron 4 of 11)
Large1	361368	chr19	12795184	12795484	147459	2.52	3.39E-03	1.20E-01	Intron (NM_001108439/361368, intron 2 of 12)
Ube2m	361509	chr1	65537757	65538057	117	1	3.39E-03	1.20E-01	Promoter (<=1kb)
Dcaf5	314273	chr6	103792796	103793096	-187540	-1.92	3.40E-03	1.21E-01	Distal Intergenic
Adar	81635	chr2	189066740	189067040	4297	-2.16	3.41E-03	1.21E-01	Exon (NM_031006/81635, exon 2 of 15)
LOC690784	690784	chr4	181360976	181361276	-12177	-1.94	3.41E-03	1.21E-01	Intron (NM_001107896/312855, intron 2 of 25)
St3gal2	64442	chr19	43281145	43281445	8918	-1.58	3.41E-03	1.21E-01	Intron (NM_031695/64442, intron 2 of 5)
Unc5c	362049	chr2	247045361	247045661	-152	1	3.42E-03	1.21E-01	Promoter (<=1kb)
Arid5a	316327	chr9	42875633	42875933	-4284	-1.95	3.42E-03	1.21E-01	Distal Intergenic
Mir378b	102465146	chr5	5513516	5513816	61883	-2	3.42E-03	1.21E-01	Distal Intergenic
Pdgfrl	290771	chr16	54420046	54420346	30080	1.91	3.43E-03	1.21E-01	Intron (NM_001011921/290771, intron 2 of 5)
Bag3	293524	chr1	199968215	199968515	26957	-2.05	3.43E-03	1.21E-01	Distal Intergenic
Daam1	314212	chr6	94635271	94635571	-651	-1.75	3.44E-03	1.21E-01	Promoter (<=1kb)
Slc39a14	306009	chr15	52023827	52024127	5689	-1.69	3.44E-03	1.21E-01	Intron (NM_001107275/306009, intron 1 of 9)
Rad23b	298012	chr5	72047600	72047900	-55804	2.33	3.45E-03	1.21E-01	Distal Intergenic
Mir30c2	100314012	chr9	29599729	29600029	37353	-1.74	3.45E-03	1.21E-01	Distal Intergenic
Gpr153	619550	chr5	169452591	169452891	98	1.11	3.45E-03	1.21E-01	Promoter (<=1kb)
Tpcn1	246215	chr12	41527501	41527801	19717	-1.92	3.46E-03	1.21E-01	Intron (NM_139332/246215, intron 2 of 29)
Barx1	364680	chr17	14831239	14831539	-3043	1.12	3.46E-03	1.21E-01	Distal Intergenic
Celf5	314647	chr7	11118083	11118383	22615	-2.29	3.46E-03	1.21E-01	3' UTR
Cdkn2aipnl	287278	chr10	37396097	37396397	-26250	-1.97	3.47E-03	1.21E-01	Distal Intergenic
Cgnl1	315795	chr8	78386880	78387180	-110326	2.43	3.47E-03	1.21E-01	Intron (NM_013176/25720, intron 9 of 20)
Slc11a2	25715	chr7	142056773	142057073	5797	-1.95	3.47E-03	1.21E-01	Intron (NM_013173/25715, intron 1 of 15)
Snx5	296199	chr3	138233453	138233753	164191	2.38	3.47E-03	1.21E-01	Distal Intergenic
Mtm1	288762	chr6	18819533	18819833	-1989	-1.86	3.48E-03	1.21E-01	Promoter (1-2kb)
Tmem231	361410	chr19	44160562	44160862	-1938	2.16	3.48E-03	1.21E-01	Promoter (1-2kb)
Thap3	362667	chr5	169164183	169164483	-3196	2.44	3.48E-03	1.21E-01	Exon (NM_001107995/313742, exon 3 of 4)
Bend6	363212	chr9	38297032	38297332	0	2.37	3.48E-03	1.21E-01	Promoter (<=1kb)
Mir34a	100314015	chr5	167077805	167078105	-14386	-1.62	3.48E-03	1.21E-01	Distal Intergenic
Lurap1l	362535	chr5	98500458	98500758	31411	-1.65	3.49E-03	1.21E-01	Intron (NM_001025022/362535, intron 1 of 1)
ST7	296911	chr4	44981130	44981430	-19625	-1.58	3.49E-03	1.21E-01	Distal Intergenic
Eif5b	308306	chr9	44658744	44659044	20	1.06	3.49E-03	1.21E-01	Promoter (<=1kb)
Lrrc8b	305135	chr14	5506322	5506622	-80832	-2.12	3.49E-03	1.21E-01	Distal Intergenic
Hoxd12	366082	chr3	61601871	61602171	4489	-1.29	3.49E-03	1.21E-01	Distal Intergenic
Galnt4	500826	chr7	41311918	41312218	6111	-2.03	3.49E-03	1.21E-01	Distal Intergenic
Raph1	363239	chr9	67362587	67362887	7321	-2.03	3.49E-03	1.21E-01	Intron (NM_001108798/363239, intron 1 of 13)
Slc19a3	316559	chr9	88872491	88872791	-44079	-2.03	3.49E-03	1.21E-01	Distal Intergenic
LOC498592	498592	chr7	91154074	91154374	94218	-1.82	3.50E-03	1.22E-01	Distal Intergenic
Meox2	29279	chr6	56645622	56645922	19854	2.25	3.50E-03	1.22E-01	Intron (NM_017149/29279, intron 1 of 2)
Igfbp7	289560	chr14	33017866	33018166	7566	2.35	3.50E-03	1.22E-01	Intron (NM_001013048/289560, intron 1 of 4)
Cdc42ep5	361505	chr1	73725380	73725680	6375	-1.82	3.51E-03	1.22E-01	Exon (NM_001037790/361506, exon 14 of 15)
Ppp2r5c	691318	chr6	134881212	134881512	36553	1.95	3.51E-03	1.22E-01	Intron (NM_001191112/691318, intron 1 of 13)
Dab2	79128	chr2	55610120	55610420	-136936	-1.62	3.51E-03	1.22E-01	Distal Intergenic
Cd44	25406	chr3	92759129	92759429	24229	-2.05	3.52E-03	1.22E-01	Intron (NM_012924/25406, intron 1 of 19)
Mdm2	314856	chr7	60704545	60704845	38483	-1.96	3.52E-03	1.22E-01	Intron (NM_001108098/314855, intron 7 of 8)
Acta2	81633	chr1	252563846	252564146	-13452	1.32	3.54E-03	1.22E-01	Distal Intergenic
Stat2	288774	chr7	2693588	2693888	2219	-1.69	3.54E-03	1.22E-01	Promoter (2-3kb)
Gsr	116686	chr16	62233266	62233566	6421	-1.73	3.55E-03	1.22E-01	Intron (NM_053906/116686, intron 1 of 12)

Mcl1	60430	chr2	197773030	197773330	-12882	-2.08	3.55E-03	1.22E-01	Distal Intergenic
Rab14	94197	chr3	14240132	14240432	145097	-2.08	3.55E-03	1.22E-01	Distal Intergenic
Lrif1	310775	chr2	209358661	209358961	-74142	0.78	3.56E-03	1.22E-01	Distal Intergenic
Ywhag	56010	chr12	23803675	23803975	14037	-1.62	3.56E-03	1.22E-01	Intron (NM_019376/56010, intron 1 of 2)
Cds1	81925	chr14	9425611	9425911	31053	-1.25	3.56E-03	1.22E-01	Intron (NM_031242/81925, intron 3 of 12)
Casp6	83584	chr2	235516160	235516460	174786	-1.23	3.57E-03	1.23E-01	Distal Intergenic
Dsel	297865	chr13	1948245	1948545	18	0.99	3.57E-03	1.23E-01	Promoter (<=1kb)
Atp11a	306600	chr16	82084456	82084756	15466	-1.63	3.58E-03	1.23E-01	Intron (NM_001107324/306600, intron 1 of 28)
Baiap2l1	304282	chr12	12220831	12221131	-5883	1.91	3.58E-03	1.23E-01	Distal Intergenic
Stk17b	170904	chr9	60413894	60414194	15887	-1.5	3.58E-03	1.23E-01	Intron (NM_133392/170904, intron 3 of 7)
Mir23b	100314002	chr17	700193	700493	-122729	-1.49	3.58E-03	1.23E-01	Intron (NM_001012346/290963, intron 6 of 14)
Ociad1	289590	chr14	37517466	37517766	-42048	-1.94	3.58E-03	1.23E-01	Distal Intergenic
Cul3	301555	chr9	86119897	86120197	-16739	-1.94	3.59E-03	1.23E-01	Distal Intergenic
Gpbp1	294734	chr2	43070418	43070718	-1741	1.05	3.59E-03	1.23E-01	Promoter (1-2kb)
Msn	81521	chrX	65258554	65258854	31720	-1.74	3.60E-03	1.23E-01	Intron (NM_030863/81521, intron 1 of 12)
Sdc1	25216	chr6	33875306	33875606	-9970	1.09	3.60E-03	1.23E-01	Distal Intergenic
Arhgap22	306279	chr16	9498302	9498602	-64616	-2	3.62E-03	1.23E-01	Distal Intergenic
LOC499796	499796	chr3	36231964	36232264	58962	2.32	3.62E-03	1.23E-01	Distal Intergenic
Nthl1	29541	chr10	14000124	14000424	3464	-1.58	3.62E-03	1.23E-01	Intron (NM_001105728/29541, intron 4 of 5)
Prdm4	170820	chr7	23948006	23948306	-16410	1.16	3.62E-03	1.23E-01	Distal Intergenic
Nt5c3b	360629	chr10	88353025	88353325	3213	-2.04	3.62E-03	1.23E-01	Intron (NM_001007723/360629, intron 6 of 10)
Acox3	83522	chr14	80316836	80317136	38284	1.87	3.63E-03	1.23E-01	Intron (NM_053339/83522, intron 19 of 19)
Gnb5	83579	chr8	82232150	82232450	-25399	-1.6	3.63E-03	1.24E-01	Distal Intergenic
Cul2	361258	chr17	57006524	57006824	-14035	1.24	3.63E-03	1.24E-01	Distal Intergenic
Acod1	306127	chr15	93584818	93585118	-27850	-1.84	3.63E-03	1.24E-01	Distal Intergenic
Grb14	58844	chr3	51006862	51007162	47285	-2.26	3.64E-03	1.24E-01	Intron (NM_031623/58844, intron 3 of 14)
Ambp	25377	chr5	78871514	78871814	114112	2.26	3.64E-03	1.24E-01	Distal Intergenic
Epc1	100362678	chr17	57360984	57361284	33691	-1.75	3.65E-03	1.24E-01	Intron (NM_001309462/100362678, intron 1 of 13)
Prpsap1	64390	chr10	105377466	105377766	74462	2.57	3.65E-03	1.24E-01	Distal Intergenic
Snx29	689142	chr10	4053956	4054256	195459	1.48	3.66E-03	1.24E-01	Intron (NM_001109526/689142, intron 14 of 20)
Smim3	286910	chr18	55754817	55755117	16613	-1.8	3.67E-03	1.24E-01	Intron (NM_173126/286910, intron 1 of 1)
Pnpla1	361812	chr20	6163506	6163806	61135	2.01	3.67E-03	1.24E-01	Distal Intergenic
Osgin1	171493	chr19	52063293	52063593	-6634	-1.96	3.67E-03	1.24E-01	Distal Intergenic
Sestd1	295678	chr3	64134399	64134699	-21067	1.29	3.68E-03	1.24E-01	Distal Intergenic
Mefv	58923	chr10	12042907	12043207	-3494	-1.71	3.69E-03	1.24E-01	Distal Intergenic
Acat1	25014	chr8	58186699	58186999	8885	-2.02	3.69E-03	1.25E-01	Intron (NM_017075/25014, intron 1 of 11)
Zbtb17	313666	chr5	160002399	160002699	8600	2.03	3.69E-03	1.25E-01	Intron (NM_001012105/313666, intron 2 of 15)
Atp1a1	24211	chr2	203913333	203913633	118390	-1.84	3.70E-03	1.25E-01	Intron (NM_012830/497761, intron 1 of 6)
Gfra4	66023	chr3	123596149	123596449	-14631	-1.57	3.70E-03	1.25E-01	Distal Intergenic
Herc3	362377	chr4	89059707	89060007	-18704	-1.81	3.71E-03	1.25E-01	Distal Intergenic
Palm2	103692368	chr5	74642132	74642432	-7333	1.98	3.71E-03	1.25E-01	Distal Intergenic
MGC94199	362483	chr5	23440554	23440854	81748	2.3	3.71E-03	1.25E-01	Distal Intergenic
LOC690120	690120	chr7	99823873	99824173	129911	-1.42	3.71E-03	1.25E-01	Intron (NM_001024876/299957, intron 4 of 6)
Cdh17	117048	chr5	25396891	25397191	5655	-1.9	3.72E-03	1.25E-01	Intron (NM_053977/117048, intron 1 of 17)
Ypel2	360590	chr10	74275237	74275537	23062	2.02	3.72E-03	1.25E-01	Intron (NM_001108286/360590, intron 2 of 4)
Chsy1	292999	chr1	127059156	127059456	48542	-2.15	3.72E-03	1.25E-01	Intron (NM_001106268/292999, intron 2 of 2)
Baiap2l1	304282	chr12	12235914	12236214	8900	-1.8	3.72E-03	1.25E-01	Intron (NM_001034140/304282, intron 1 of 13)
Mrm3	360569	chr10	64464576	64464876	66237	2.58	3.72E-03	1.25E-01	Intron (NM_001108285/360577, intron 1 of 7)
Ifi47	246208	chr10	34280847	34281147	2854	-2.05	3.72E-03	1.25E-01	Promoter (2-3kb)
Pitrm1	307081	chr17	68430809	68431109	-46358	2.58	3.73E-03	1.25E-01	Distal Intergenic
Zfp217	311764	chr3	166855025	166855325	138615	2.48	3.73E-03	1.25E-01	Distal Intergenic
Nfic	29228	chr7	11144188	11144488	-7550	-1.92	3.74E-03	1.25E-01	Distal Intergenic
Ier5l	499772	chr3	9051973	9052273	-14031	-2.05	3.74E-03	1.25E-01	Distal Intergenic
Ptpa	362102	chr3	8993322	8993622	11200	1.98	3.74E-03	1.25E-01	Intron (NM_001108577/362102, intron 3 of 9)
Chsy1	292999	chr1	127023848	127024148	13234	-2.07	3.74E-03	1.25E-01	Intron (NM_001106268/292999, intron 1 of 2)
Ddx52	85432	chr10	71209140	71209440	-44227	-1.86	3.74E-03	1.25E-01	Exon (NM_001308148/25640, exon 9 of 10)
Babam2	362704	chr6	25867618	25867918	183311	-1.88	3.74E-03	1.25E-01	Intron (NM_199270/362704, intron 6 of 11)
RT1-T24-3	414788	chr20	3234114	3234414	7723	-1.89	3.75E-03	1.25E-01	Intron (NR_002597/360231, intron 7 of 8)
Fn1	25661	chr9	79068969	79069269	-99956	-1.91	3.75E-03	1.25E-01	Distal Intergenic
Dhx58	303538	chr10	88596936	88597236	13659	-2	3.76E-03	1.25E-01	Downstream (2-3kb)
RGD1561161	294747	chr2	45155433	45155733	-26658	-2	3.76E-03	1.25E-01	Distal Intergenic
Tlr2	310553	chr2	182843263	182843563	2498	-2	3.76E-03	1.25E-01	Promoter (2-3kb)
Bcat1	29592	chr4	179193081	179193381	113714	-2.04	3.76E-03	1.25E-01	Distal Intergenic
Pdgfa	25266	chr12	17757660	17757960	23519	-1.88	3.77E-03	1.25E-01	Distal Intergenic
Mn1	498194	chr12	50873670	50873970	376260	1.81	3.78E-03	1.25E-01	Distal Intergenic
Ugcg	83626	chr5	76520280	76520580	133442	2.53	3.79E-03	1.26E-01	Distal Intergenic
Dnmt3a	444984	chr6	28205752	28206052	-29643	1.56	3.79E-03	1.26E-01	Distal Intergenic
Tuba1b	500929	chr7	140626295	140626595	-8574	2.17	3.79E-03	1.26E-01	Distal Intergenic
Aven	311299	chr3	104248324	104248624	206156	-2.1	3.80E-03	1.26E-01	Distal Intergenic
Acsl6	117243	chr10	39668808	39669108	1817	0.99	3.80E-03	1.26E-01	Promoter (1-2kb)
Atg16l1	363278	chr9	94895762	94896062	15709	-1.41	3.81E-03	1.26E-01	Intron (NM_001108809/363278, intron 6 of 17)
Mrpl14	301250	chr9	17632343	17632643	60557	-1.84	3.81E-03	1.26E-01	Distal Intergenic
Ptf1a	117034	chr17	86380435	86380735	180812	-1.19	3.81E-03	1.26E-01	Distal Intergenic
Zfp316	304293	chr12	13348280	13348580	-4679	1.58	3.81E-03	1.26E-01	Distal Intergenic
Gkn2	297419	chr4	119182851	119183151	11606	-1.88	3.81E-03	1.26E-01	Distal Intergenic
Tubb3	246118	chr19	56220808	56221108	49	-1.83	3.82E-03	1.26E-01	Promoter (<=1kb)
Map4k4	301363	chr9	46673917	46674217	15995	-1.69	3.82E-03	1.26E-01	Intron (NM_001106904/301363, intron 1 of 28)
Tpra1	85494	chr4	120866976	120867276	23477	1.02	3.82E-03	1.26E-01	Distal Intergenic
Fmo5	246248	chr2	199667869	199668169	-128701	-2.11	3.82E-03	1.26E-01	Distal Intergenic
Hey2	155430	chr1	29233925	29234225	42755	2.43	3.82E-03	1.26E-01	Distal Intergenic
Palmd	310811	chr2	220599582	220599882	-63578	-1.73	3.84E-03	1.26E-01	Distal Intergenic
Runx2	367218	chr9	18713746	18714046	70176	-1.85	3.85E-03	1.26E-01	Intron (NM_001278483/367218, intron 5 of 8)
Bnc2	298189	chr5	102682544	102682844	60573	-1.74	3.86E-03	1.27E-01	Intron (NM_001106666/298189, intron 2 of 6)
Nsmce2	299957	chr7	99741507	99741807	64172	-1.45	3.86E-03	1.27E-01	Intron (NM_001024876/299957, intron 3 of 6)
Sarnp	362819	chr7	3267976	3268276	21756	-1.87	3.87E-03	1.27E-01	Intron (NM_001033070/362819, intron 4 of 10)
Clcf1	365395	chr1	219458364	219458664	-10202	-1.91	3.87E-03	1.27E-01	Distal Intergenic
Ldhd	307858	chr19	43804931	43805231	43706	-2.1	3.88E-03	1.27E-01	Distal Intergenic
Tmem79	310626	chr2	187708324	187708624	-2024	0.99	3.88E-03	1.27E-01	Promoter (2-3kb)
Tbx2	303398	chr10	73285583	73285883	6584	-1.58	3.88E-03	1.27E-01	Intron (NM_001107033/303398, intron 3 of 6)
Ldb3	498587	chr16	10940888	10941188	230	0.83	3.89E-03	1.27E-01	Promoter (<=1kb)
Ppp4r1	140943	chr9	113515868	113516168	577	0.75	3.89E-03	1.27E-01	Promoter (<=1kb)
Atf6b	406169	chr20	4428661	4428961	14229	2.23	3.89E-03	1.27E-01	Intron (NM_031504/24233, intron 35 of 40)
Slc10a2	29500	chr16	90620083	90620383	294886	-2.1	3.89E-03	1.27E-01	Distal Intergenic
Cd200	24560	chr11	60371717	60372017	0	0.88	3.89E-03	1.27E-01	Promoter (<=1kb)

Insig2	288985	chr13	37175168	37175468	104398	0.92	3.89E-03	1.27E-01	Distal Intergenic
Fktn	362520	chr5	70416306	70416606	-105395	1.85	3.90E-03	1.27E-01	Intron (NM_053492/85254, intron 15 of 15)
Vopp1	362374	chr4	88430429	88430729	-10338	-2.15	3.91E-03	1.27E-01	Distal Intergenic
St3gal5	83505	chr4	99937705	99938005	147	1.16	3.92E-03	1.27E-01	Promoter (<=1kb)
Rgsl2h	364021	chr13	71277832	71278132	4285	-1.63	3.92E-03	1.27E-01	Distal Intergenic
Met	24553	chr4	44832409	44832709	57076	2.5	3.92E-03	1.27E-01	Intron (NM_031517/24553, intron 13 of 19)
Gnrh1	25194	chr15	44550928	44551228	109072	-2.02	3.92E-03	1.27E-01	Distal Intergenic
Cd8a	24930	chr4	99305687	99305987	66572	1.41	3.93E-03	1.28E-01	Distal Intergenic
Pard3	81918	chr19	60022665	60022965	4919	-1.86	3.94E-03	1.28E-01	Intron (NM_031235/81918, intron 1 of 24)
Nup62	65274	chr1	100823850	100824150	12095	-1.3	3.94E-03	1.28E-01	Intron (NM_023098/65274, intron 1 of 1)
Slc39a11	287796	chr10	102099800	102100100	-16320	-1.84	3.94E-03	1.28E-01	Distal Intergenic
C1ql2	288979	chr13	36710370	36710670	332183	1.07	3.96E-03	1.28E-01	Distal Intergenic
Mir3596a	100526642	chr8	45762526	45762826	-9159	-1.8	3.97E-03	1.28E-01	Intron (NR_126581/104845260, intron 3 of 3)
Scrn1	502776	chr4	84740257	84740557	144	0.77	3.97E-03	1.28E-01	Promoter (<=1kb)
Hmgn2	114637	chr5	152193381	152193681	5132	-1.59	3.97E-03	1.28E-01	Downstream (1-2kb)
Cd180	294706	chr2	32801886	32802186	-18136	-1.58	3.97E-03	1.28E-01	Distal Intergenic
Prom1	60357	chr14	71491916	71492216	-40847	1.91	3.98E-03	1.28E-01	Distal Intergenic
Brd4	362844	chr7	14278246	14278546	24509	1.87	3.98E-03	1.28E-01	Intron (NM_001100903/362844, intron 1 of 18)
Tprg1	360731	chr11	78823521	78823821	37924	-1.78	3.99E-03	1.29E-01	Intron (NM_001108320/360731, intron 2 of 5)
Bach2	313125	chr5	47466682	47466982	-79032	1.34	3.99E-03	1.29E-01	Distal Intergenic
Ptgis	25527	chr3	163985848	163986148	0	0.79	4.00E-03	1.29E-01	Promoter (<=1kb)
Phyhd1	296621	chr3	8836696	8836996	3956	-1.88	4.01E-03	1.29E-01	Intron (NM_001013081/296621, intron 2 of 10)
Ssc5d	308341	chr1	72461427	72461727	0	1.2	4.01E-03	1.29E-01	Promoter (<=1kb)
Usp7	360471	chr10	6974574	6974874	-415	1.1	4.04E-03	1.30E-01	Promoter (<=1kb)
Ldlrad4	679578	chr18	63832430	63832730	-11269	-1.97	4.04E-03	1.30E-01	Distal Intergenic
Mirlet7i	100313993	chr7	66827521	66827821	24790	-1.62	4.05E-03	1.30E-01	Distal Intergenic
Zfp689	286996	chr1	198930477	198930777	-30113	-1.55	4.06E-03	1.30E-01	Distal Intergenic
Mtmr7	306490	chr16	54813365	54813665	48040	1.84	4.07E-03	1.30E-01	Intron (NM_001107312/306490, intron 3 of 13)
Tmsb10	50665	chr4	100900702	100901002	-17427	-1.07	4.07E-03	1.30E-01	Distal Intergenic
Tchp	304547	chr12	47629807	47630107	22600	1.37	4.07E-03	1.30E-01	Intron (NM_001005553/304546, intron 19 of 19)
Igfbp7	289560	chr14	32896328	32896628	-113672	1.4	4.07E-03	1.30E-01	Distal Intergenic
Reep3	294375	chr20	23160254	23160554	246605	-1.96	4.08E-03	1.31E-01	Distal Intergenic
Cript	56725	chr6	10630686	10630986	36539	2.38	4.08E-03	1.31E-01	Distal Intergenic
Ezr	54319	chr1	47374942	47375242	-43530	1.1	4.09E-03	1.31E-01	Distal Intergenic
Efnb2	306636	chr16	86804708	86805008	173557	1.99	4.09E-03	1.31E-01	Distal Intergenic
Serpine1	24617	chr12	22607112	22607412	-33692	-0.99	4.10E-03	1.31E-01	Distal Intergenic
Phf7	364510	chr16	7326029	7326329	9901	2.14	4.10E-03	1.31E-01	Exon (NM_001012211/364510, exon 7 of 11)
Sgms1	353229	chr1	250871273	250871573	80113	2.46	4.11E-03	1.31E-01	Intron (NM_181386/353229, intron 2 of 9)
Cxcl12	24772	chr4	149560024	149560324	298980	-1.42	4.11E-03	1.31E-01	Distal Intergenic
Rrlt	679825	chr1	1173173	1173473	-56247	0.89	4.11E-03	1.31E-01	Intron (NM_001013063/292461, intron 5 of 6)
Gask1b	310540	chr2	178847686	178847986	168645	2.34	4.12E-03	1.31E-01	Distal Intergenic
Tmem196	500750	chr6	147877380	147877680	1143	1.83	4.12E-03	1.31E-01	Promoter (1-2kb)
Cbx7	362962	chr7	121172355	121172655	-18972	1.96	4.14E-03	1.31E-01	Distal Intergenic
Dimt1	294718	chr2	37892304	37892604	-227982	-2.3	4.14E-03	1.31E-01	Distal Intergenic
Bicd1	362466	chr4	183888198	183888498	-130589	-1.78	4.14E-03	1.32E-01	Distal Intergenic
Abraxas2	293570	chr1	204938079	204938379	76311	1.65	4.15E-03	1.32E-01	Distal Intergenic
Foxo1	84482	chr2	141830889	141831189	379655	2.41	4.15E-03	1.32E-01	Distal Intergenic
Clip4	298801	chr6	23198422	23198722	118240	2.59	4.15E-03	1.32E-01	Exon (NM_001169101/266802, exon 27 of 28)
Arntl	29657	chr1	178023815	178024115	-14948	-1.56	4.16E-03	1.32E-01	Distal Intergenic
Fndc3b	294925	chr2	113391410	113391710	-45859	-1.62	4.17E-03	1.32E-01	Distal Intergenic
Hs6st2	302489	chrX	138972002	138972302	382	-2.04	4.17E-03	1.32E-01	Promoter (<=1kb)
Rap2a	114560	chr15	105851471	105851771	0	1.06	4.17E-03	1.32E-01	Promoter (<=1kb)
Atp6v0e2	436582	chr4	78132687	78132987	-35130	1.15	4.17E-03	1.32E-01	Exon (NM_001007016/474348, exon 102 of 103)
Elavl2	286973	chr5	109624534	109624834	965	-2.28	4.18E-03	1.32E-01	Promoter (<=1kb)
Kif3b	296284	chr3	148817660	148817960	44401	2.4	4.19E-03	1.32E-01	Distal Intergenic
Mir875	100314134	chr7	74306730	74307030	116275	2.46	4.20E-03	1.32E-01	Intron (NM_001134886/315036, intron 19 of 61)
Rasl10a	364190	chr14	85311133	85311433	-400	2.47	4.21E-03	1.33E-01	Promoter (<=1kb)
Mir28	100314152	chr11	79477372	79477672	-96764	1.93	4.21E-03	1.33E-01	Intron (NM_001013864/288010, intron 5 of 11)
Slc41a3	641603	chr4	123487923	123488223	6519	-1.62	4.23E-03	1.33E-01	Exon (NM_001037492/641603, exon 3 of 10)
Flna	293860	chrX	156469330	156469630	5377	-1.92	4.23E-03	1.33E-01	Exon (NM_001134599/293860, exon 8 of 46)
St6galnac1	287920	chr10	105722655	105722955	745	-2.14	4.23E-03	1.33E-01	Promoter (<=1kb)
Cdk14	362316	chr4	26065228	26065528	239661	-1.2	4.24E-03	1.33E-01	Intron (NM_001108617/362316, intron 4 of 12)
Tmem263	362857	chr7	24744943	24745243	-77642	-2	4.25E-03	1.34E-01	3' UTR
Exoc2	171455	chr17	34486560	34486860	-178950	-1.62	4.26E-03	1.34E-01	Distal Intergenic
Rap1gap2	303298	chr10	61242494	61242794	-10163	-2.12	4.26E-03	1.34E-01	Distal Intergenic
Nnt	310378	chr2	52216648	52216948	65600	-1.84	4.27E-03	1.34E-01	Intron (NM_001013157/310378, intron 17 of 21)
Nkain4	296469	chr3	176460183	176460483	4679	2.44	4.27E-03	1.34E-01	Intron (NM_001106550/296469, intron 1 of 4)
Nucb2	59295	chr1	185121763	185122063	21144	1.33	4.27E-03	1.34E-01	Intron (NM_021663/59295, intron 4 of 13)
LOC688925	688925	chr10	70419517	70419817	5118	-1.58	4.28E-03	1.34E-01	Intron (NM_053687/114247, intron 2 of 5)
Stat6	362896	chr7	70937226	70937526	-8702	-1.69	4.28E-03	1.34E-01	Distal Intergenic
Pgd	100360180	chr5	166025809	166026109	-43482	-1.4	4.29E-03	1.34E-01	Intron (NM_057200/117548, intron 32 of 48)
Niban2	362115	chr3	11947484	11947784	25769	-1.59	4.29E-03	1.34E-01	Intron (NM_001109885/362115, intron 1 of 13)
Il13ra2	171060	chrX	118712529	118712829	-197813	-1.87	4.29E-03	1.34E-01	Distal Intergenic
Pgam2	24959	chr14	86046874	86047174	0	2.17	4.30E-03	1.34E-01	Promoter (<=1kb)
Septin9	83788	chr10	106542188	106542488	235155	1.88	4.30E-03	1.34E-01	Distal Intergenic
Fam166a	311797	chr3	2433942	2434242	-755	2.27	4.30E-03	1.34E-01	Promoter (<=1kb)
Sema5a	310207	chr2	85421375	85421675	44057	-2.05	4.31E-03	1.34E-01	Intron (NM_001107659/310207, intron 1 of 22)
Ube2n	116725	chr7	36621955	36622255	11808	2.12	4.32E-03	1.34E-01	Intron (NM_053928/116725, intron 1 of 3)
Slc48a1	300191	chr7	139322384	139322684	50686	2.36	4.32E-03	1.34E-01	Distal Intergenic
Stam	498798	chr17	81218364	81218664	8497	-1.9	4.33E-03	1.35E-01	Intron (NM_001109121/498798, intron 1 of 14)
Oat	64313	chr1	204448954	204449254	132816	-1.92	4.33E-03	1.35E-01	Distal Intergenic
Eea1	314764	chr7	37082354	37082654	-18760	-1.69	4.33E-03	1.35E-01	Distal Intergenic
Sgk1	29517	chr1	24565194	24565494	-371627	-1.74	4.33E-03	1.35E-01	Distal Intergenic
Map1a	25152	chr3	113261672	113261972	3984	2.31	4.34E-03	1.35E-01	Exon (NM_030995/25152, exon 4 of 6)
Tssc4	361682	chr1	216262061	216262361	7150	-1.79	4.35E-03	1.35E-01	Intron (NM_001191896/365391, intron 18 of 23)
Gnai2	81664	chr8	116376880	116377180	14127	2.34	4.35E-03	1.35E-01	Intron (NM_031035/81664, intron 1 of 8)
Sprtn	292101	chr19	57699757	57700057	49594	1.83	4.35E-03	1.35E-01	Distal Intergenic
Ttc39c	686179	chr18	3865874	3866174	-92258	-2.04	4.35E-03	1.35E-01	Distal Intergenic
Lhfpl6	499615	chr2	142276276	142276576	14040	-2.04	4.35E-03	1.35E-01	Intron (NM_001109183/499615, intron 2 of 3)
Atp6v0a4	296981	chr4	65839740	65840040	-21219	-2.31	4.36E-03	1.35E-01	Distal Intergenic
Smim3	286910	chr18	55751463	55751763	19967	-1.5	4.37E-03	1.35E-01	Intron (NM_173126/286910, intron 1 of 1)
Sspo	474348	chr4	78106239	78106539	25929	2.23	4.38E-03	1.35E-01	Exon (NM_001007016/474348, exon 50 of 103)
Tmem71	690657	chr7	107371148	107371448	21360	-1.99	4.38E-03	1.35E-01	Intron (NM_001271215/690657, intron 5 of 8)
Fbp2	114508	chr17	110677	110977	278941	0.74	4.38E-03	1.35E-01	Distal Intergenic

Mgat3	29582	chr7	121397721	121398021	-10808	2.11	4.39E-03	1.35E-01	Distal Intergenic
Pcbp2	363005	chr7	144082442	144082742	4536	-1.77	4.39E-03	1.35E-01	Intron (NM_001013223/363005, intron 5 of 14)
Ttc12	300696	chr8	53939955	53940255	-123502	2.33	4.39E-03	1.35E-01	Intron (NM_031521/24586, intron 1 of 18)
Vta1	292640	chr1	8794499	8794799	83337	-2.3	4.39E-03	1.35E-01	Distal Intergenic
Nlk	497961	chr10	65968503	65968803	-5716	-1.5	4.39E-03	1.35E-01	Distal Intergenic
Oat	64313	chr1	204470437	204470737	111333	-1.67	4.40E-03	1.36E-01	Distal Intergenic
Pdhx	311254	chr3	92902684	92902984	30741	-1.81	4.41E-03	1.36E-01	Distal Intergenic
Afdn	26955	chr1	53827577	53827877	13379	-2.13	4.42E-03	1.36E-01	Intron (NM_013217/26955, intron 2 of 39)
Gzmk	29165	chr2	45088067	45088367	-10913	-2.13	4.42E-03	1.36E-01	Distal Intergenic
LOC690120	690120	chr7	99940492	99940792	13292	-2.13	4.42E-03	1.36E-01	Distal Intergenic
Plet1	363060	chr8	54875707	54876007	-49659	-2.13	4.42E-03	1.36E-01	Distal Intergenic
Vil1	316521	chr9	81705621	81705921	15819	-2.13	4.42E-03	1.36E-01	Intron (NM_001108224/316521, intron 13 of 19)
Rbpj	679028	chr14	59714074	59714374	21076	-1.98	4.45E-03	1.36E-01	Intron (NM_001106631/679028, intron 2 of 11)
Cd247	25300	chr13	84023565	84023865	27485	-1.82	4.45E-03	1.36E-01	Intron (NM_001205304/25300, intron 1 of 6)
Gpr61	310780	chr2	210939220	210939520	4100	-1.71	4.45E-03	1.36E-01	Exon (NM_001107715/310780, exon 2 of 2)
Wwtr1	295062	chr2	147684524	147684824	8209	-1.46	4.47E-03	1.37E-01	Intron (NM_001024869/295062, intron 2 of 6)
Kpna3	361055	chr15	41766090	41766390	3722	-2.13	4.48E-03	1.37E-01	Intron (NM_001014792/361055, intron 1 of 16)
Cdc27	360643	chr10	92454211	92454511	147571	-1.8	4.48E-03	1.37E-01	Distal Intergenic
Lamc2	192362	chr13	70630381	70630681	-4129	-1.72	4.49E-03	1.37E-01	Distal Intergenic
Meox2	29279	chr6	56626144	56626444	376	0.82	4.50E-03	1.37E-01	Promoter (<=1kb)
RGD1562024	498699	chr17	8708704	8709004	-88967	1.72	4.50E-03	1.37E-01	Distal Intergenic
Slc6a6	29464	chr4	123728106	123728406	-14787	-1.43	4.50E-03	1.37E-01	Distal Intergenic
Uvrag	308846	chr1	163969904	163970204	131364	2.31	4.51E-03	1.37E-01	Intron (NM_001107536/308846, intron 7 of 13)
Arl4a	29308	chr6	59616551	59616851	333735	-1.71	4.51E-03	1.37E-01	Distal Intergenic
Hmox1	24451	chr19	14508037	14508337	-297	-1.41	4.52E-03	1.37E-01	Promoter (<=1kb)
Nek7	360850	chr13	55607925	55608225	-46199	-1.97	4.52E-03	1.37E-01	Distal Intergenic
Pik3ca	170911	chr2	118797230	118797530	-33820	-1.42	4.53E-03	1.37E-01	Distal Intergenic
Foxp1	297480	chr4	131622884	131623184	-24580	-1.83	4.53E-03	1.37E-01	Distal Intergenic
Pkig	266709	chr3	160066419	160066719	19123	-1.57	4.53E-03	1.37E-01	Intron (NM_153469/266709, intron 1 of 2)
Swap70	293410	chr1	174913190	174913490	50465	1.16	4.53E-03	1.37E-01	Intron (NM_001106288/293410, intron 7 of 11)
Gan	307893	chr19	49706413	49706713	10834	1.98	4.54E-03	1.38E-01	Intron (NM_001107434/307893, intron 1 of 10)
Pelp1	360552	chr10	56995195	56995495	9616	-1.74	4.54E-03	1.38E-01	Intron (NM_001024270/360552, intron 5 of 16)
Sestd1	295678	chr3	63952077	63952377	160955	-1.54	4.54E-03	1.38E-01	Distal Intergenic
Otp	294640	chr2	24539487	24539787	-6606	1.1	4.55E-03	1.38E-01	Distal Intergenic
Zfand3	361816	chr20	8670639	8670939	-25509	-1.64	4.55E-03	1.38E-01	Distal Intergenic
Fzd2	64512	chr10	90529773	90530073	-20074	0.85	4.55E-03	1.38E-01	Distal Intergenic
Tmem176b	171411	chr4	78457572	78457872	307	-1.59	4.55E-03	1.38E-01	Promoter (<=1kb)
Gas7	85246	chr10	54010982	54011282	-75561	1.41	4.56E-03	1.38E-01	Distal Intergenic
LOC499643	499643	chr2	178213287	178213587	95804	-1.62	4.56E-03	1.38E-01	Intron (NM_001024301/499643, intron 3 of 4)
Pus3	315554	chr8	36771898	36772198	4921	0.75	4.57E-03	1.38E-01	Distal Intergenic
Arhgap24	305156	chr14	8480428	8480728	-48352	1.79	4.57E-03	1.38E-01	Distal Intergenic
Hspa5	25617	chr3	13824378	13824678	-13626	1.37	4.57E-03	1.38E-01	Distal Intergenic
Myo7a	266714	chr1	163056081	163056381	15127	2.13	4.57E-03	1.38E-01	Intron (NM_153473/266714, intron 3 of 48)
Cdkn2b	25164	chr5	107939939	107940239	-82554	-1.74	4.57E-03	1.38E-01	Distal Intergenic
Fut8	432392	chr6	100312239	100312539	-84514	-1.58	4.58E-03	1.38E-01	Distal Intergenic
Zfp217	311764	chr3	166607009	166607309	386631	1.29	4.58E-03	1.38E-01	Distal Intergenic
Ttc17	311224	chr3	83299605	83299905	6685	-1.68	4.59E-03	1.38E-01	Intron (NM_001107752/311224, intron 1 of 24)
Exoc6b	500233	chr4	116653473	116653773	132651	-2.1	4.59E-03	1.38E-01	Intron (NM_001109246/500233, intron 6 of 22)
Map4k4	301363	chr9	46701828	46702128	43906	-1.8	4.59E-03	1.38E-01	Intron (NM_001106904/301363, intron 1 of 28)
Eva1b	362597	chr5	144138643	144138943	-21165	2.15	4.60E-03	1.38E-01	Exon (NM_183056/360230, exon 5 of 11)
Sgta	64667	chr7	11521293	11521593	-437	-1.14	4.60E-03	1.38E-01	Promoter (<=1kb)
Opn4	192223	chr16	11033936	11034236	-81387	-1.88	4.60E-03	1.38E-01	Distal Intergenic
Arhgef3	290541	chr16	2973017	2973317	14734	-1.79	4.60E-03	1.38E-01	Intron (NM_001106061/290541, intron 1 of 9)
Igf2bp1	303477	chr10	83799561	83799861	23135	1.71	4.60E-03	1.38E-01	Intron (NM_175594/303477, intron 2 of 14)
Kif3b	296284	chr3	148932713	148933013	159454	2.32	4.61E-03	1.38E-01	Distal Intergenic
LOC100302465	100302465	chr1	206399378	206399678	17465	-1.8	4.62E-03	1.38E-01	Intron (NM_001163214/100302465, intron 6 of 11)
Pi4kb	81747	chr2	196151392	196151692	11637	1.39	4.63E-03	1.38E-01	Intron (NM_031083/81747, intron 2 of 12)
Cebpb	24253	chr3	164483437	164483737	58935	-1.97	4.63E-03	1.38E-01	Distal Intergenic
Ptprj	29645	chr3	79359588	79359888	31068	2.86	4.64E-03	1.38E-01	Intron (NM_017269/29645, intron 1 of 23)
Smim38	246306	chr1	218251957	218252257	122362	-0.87	4.65E-03	1.38E-01	Distal Intergenic
Hsdl2	313200	chr5	76860596	76860896	47665	1.02	4.65E-03	1.38E-01	Distal Intergenic
Acin1	305884	chr15	33332700	33333000	256	0.79	4.65E-03	1.38E-01	Promoter (<=1kb)
Ddx24	373065	chr6	127312965	127313265	6097	-1.99	4.65E-03	1.38E-01	Exon (NM_199119/373065, exon 3 of 9)
RGD621098	207123	chr19	37169692	37169992	-9858	-1.57	4.65E-03	1.38E-01	3' UTR
Fez2	94269	chr6	998907	999207	4717	-1.66	4.66E-03	1.38E-01	Exon (NM_053600/94269, exon 2 of 9)
Cep63	300963	chr8	111103313	111103613	3562	-1.62	4.66E-03	1.38E-01	Exon (NR_045200/300963, exon 3 of 18)
Npdc1	296562	chr3	2625020	2625320	5	0.89	4.66E-03	1.38E-01	Promoter (<=1kb)
Vta1	292640	chr1	8747943	8748243	129893	-2.07	4.66E-03	1.38E-01	Distal Intergenic
Socs1	252971	chr10	5005380	5005680	48587	-2.07	4.66E-03	1.38E-01	Distal Intergenic
Mir26a	100314290	chr8	127600446	127600746	-113695	2.43	4.67E-03	1.38E-01	Intron (NM_001106865/301056, intron 2 of 7)
Jag1	29146	chr3	130065748	130066048	48733	1.2	4.67E-03	1.38E-01	Exon (NM_001271320/499895, exon 8 of 8)
Mir365b	100314252	chr10	67185894	67186194	106098	2.48	4.67E-03	1.38E-01	Distal Intergenic
Erc1	266806	chr4	152191484	152191784	188239	2.41	4.68E-03	1.38E-01	Intron (NM_170788/266806, intron 14 of 18)
Vac14	307842	chr19	40986080	40986380	42826	1.68	4.68E-03	1.38E-01	Intron (NM_177930/307842, intron 12 of 18)
Actrt2	298671	chr5	172103370	172103670	-24680	2.59	4.68E-03	1.38E-01	Distal Intergenic
Mllt3	114510	chr5	106342750	106343050	-140189	1.05	4.68E-03	1.38E-01	Distal Intergenic
Socs3	89829	chr10	106967726	106968026	8014	-1.94	4.68E-03	1.38E-01	Distal Intergenic
Ube2v2	287927	chr11	89846224	89846524	288951	-1.94	4.68E-03	1.38E-01	Distal Intergenic
Ift43	299209	chr6	109958278	109958578	18933	2.24	4.68E-03	1.38E-01	Intron (NM_001134525/299209, intron 2 of 8)
Sfxn1	364678	chr17	11050528	11050828	31196	-1.58	4.69E-03	1.39E-01	Intron (NM_001012213/364678, intron 8 of 10)
Rala	81757	chr17	49691182	49691482	-22808	-2.08	4.69E-03	1.39E-01	Distal Intergenic
Hs6st1	316325	chr9	42338973	42339273	-280733	1.89	4.69E-03	1.39E-01	Distal Intergenic
Tlk1	311118	chr3	57167973	57168273	-63943	2.05	4.70E-03	1.39E-01	Distal Intergenic
Slc7a1	25648	chr12	8037881	8038181	5106	-2.1	4.70E-03	1.39E-01	Intron (NM_013111/25648, intron 1 of 11)
Dclk3	316023	chr8	119831213	119831513	139646	1.63	4.71E-03	1.39E-01	Distal Intergenic
Zc3h18	292067	chr19	55197464	55197764	-8691	1.56	4.71E-03	1.39E-01	Distal Intergenic
Fgf2	54250	chr2	124096817	124097117	15745	-2.24	4.72E-03	1.39E-01	Intron (NM_019305/54250, intron 1 of 2)
Mapkapk5	498183	chr12	40510122	40510422	0	1.24	4.72E-03	1.39E-01	Promoter (<=1kb)
Tmcc2	305095	chr13	49169633	49169933	0	1	4.73E-03	1.39E-01	Promoter (<=1kb)
Tomm20	266601	chr19	59601529	59601829	271582	-2.04	4.73E-03	1.39E-01	Distal Intergenic
Tmem165	364137	chr14	34524450	34524750	3512	-1.72	4.74E-03	1.39E-01	Intron (NM_001024802/364137, intron 1 of 5)
Sertad2	498423	chr14	104817869	104818169	-3046	2.25	4.74E-03	1.39E-01	Distal Intergenic
Faap20	362678	chr5	172598611	172598911	-50039	-1.69	4.74E-03	1.39E-01	Distal Intergenic
Klhl29	298867	chr6	29286621	29286921	196539	-2.01	4.75E-03	1.39E-01	Intron (NM_001106713/298867, intron 3 of 13)

Apaf1	78963	chr7	31517146	31517446	266746	-1.73	4.75E-03	1.39E-01	Intron (NM_001271371/314721, intron 22 of 30)
Tm4sf1	295061	chr2	147386614	147386914	5148	-1.68	4.76E-03	1.40E-01	Intron (NM_001106434/295061, intron 3 of 4)
Ceacam9	116711	chr1	78663480	78663780	4045	-1.7	4.77E-03	1.40E-01	3' UTR
Nr2f1	81808	chr2	7123164	7123464	-1543270	1.79	4.80E-03	1.40E-01	Distal Intergenic
Rangap1	362965	chr7	122982700	122983000	-15522	-1.75	4.80E-03	1.40E-01	Distal Intergenic
Dnm1l	114114	chr11	88792602	88792902	-38066	-2.26	4.80E-03	1.40E-01	Intron (NM_139263/246174, intron 19 of 20)
Etv4	360635	chr10	89748588	89748888	-48305	-1.63	4.81E-03	1.40E-01	Distal Intergenic
Arhgap35	306400	chr1	78529161	78529461	43913	-1.83	4.81E-03	1.40E-01	Intron (NM_001271132/306400, intron 1 of 6)
Xpnpep1	170751	chr1	273755244	273755544	2703	-1.86	4.81E-03	1.40E-01	Promoter (2-3kb)
Tmem126a	293113	chr1	156286511	156286811	3383	-1.98	4.82E-03	1.40E-01	Intron (NM_022282/64053, intron 2 of 22)
Kif23	315740	chr8	66892809	66893109	70	0.76	4.82E-03	1.40E-01	Promoter (<=1kb)
Prpsap1	64390	chr10	105393222	105393522	58706	1.23	4.82E-03	1.40E-01	Distal Intergenic
RGD1562638	290818	chr16	67885913	67886213	-700022	2.38	4.84E-03	1.41E-01	Distal Intergenic
Rassf3	362886	chr7	63562226	63562526	15964	-1.9	4.84E-03	1.41E-01	Intron (NM_001108747/362886, intron 1 of 4)
Wt1	24883	chr3	95169766	95170066	36053	2.04	4.84E-03	1.41E-01	Intron (NM_031534/24883, intron 5 of 9)
Nkain4	296469	chr3	176457820	176458120	7042	2.07	4.85E-03	1.41E-01	Intron (NM_001106550/296469, intron 1 of 4)
Oasl2	304549	chr12	47474774	47475074	7873	-1.74	4.86E-03	1.41E-01	Intron (NM_001009682/304549, intron 4 of 5)
Plekhf1	308543	chr1	94576911	94577211	33633	-1.87	4.86E-03	1.41E-01	Distal Intergenic
Nuak1	299694	chr7	25019756	25020056	-19280	-1.07	4.87E-03	1.41E-01	Distal Intergenic
Pcmtd1	366300	chr5	12202368	12202668	-30359	0.78	4.87E-03	1.41E-01	Distal Intergenic
Upk3bl1	367994	chr12	23682162	23682462	-21153	2.37	4.87E-03	1.41E-01	Distal Intergenic
Met	24553	chr4	44852584	44852884	77251	2.39	4.87E-03	1.41E-01	Distal Intergenic
Neurod1	29458	chr3	66374033	66374333	43284	-1.88	4.87E-03	1.41E-01	Distal Intergenic
Ndufa5	25488	chr4	51625612	51625912	-26846	0.74	4.87E-03	1.41E-01	Distal Intergenic
Ankrd40	690586	chr10	82037275	82037575	4619	-1.69	4.88E-03	1.41E-01	Intron (NM_001134699/690586, intron 1 of 5)
Rb1cc1	312927	chr5	13103168	13103468	-5827	-1.72	4.88E-03	1.41E-01	Distal Intergenic
Rasgrf2	114513	chr2	21782070	21782370	-83133	-1.98	4.88E-03	1.41E-01	Distal Intergenic
Gne	114711	chr5	59535620	59535920	7181	1.66	4.89E-03	1.41E-01	Intron (NM_053765/114711, intron 2 of 11)
Hibadh	63938	chr4	82739174	82739474	-36745	2.39	4.90E-03	1.41E-01	Distal Intergenic
Golga7b	309378	chr1	261488311	261488611	-5900	-1.78	4.90E-03	1.41E-01	Distal Intergenic
Swi5	499779	chr3	11311918	11312218	4831	-1.89	4.90E-03	1.41E-01	Intron (NM_001246661/499779, intron 2 of 4)
Pear1	295293	chr2	187128944	187129244	4749	2.25	4.90E-03	1.41E-01	Intron (NM_001134959/295293, intron 1 of 24)
Klhl25	293023	chr1	137447338	137447638	-88266	2.34	4.90E-03	1.41E-01	Distal Intergenic
Fn1	25661	chr9	79065214	79065514	-96201	-2.04	4.91E-03	1.41E-01	Distal Intergenic
Plxnb2	315217	chr7	130154108	130154408	7105	-1.57	4.91E-03	1.42E-01	Intron (NM_001108106/315217, intron 1 of 37)
LOC100361645	100361645	chr3	46115046	46115346	-70014	2.04	4.92E-03	1.42E-01	Intron (NM_001108260/317627, intron 3 of 38)
Fendrr	104845258	chr19	53010156	53010456	53	1.02	4.92E-03	1.42E-01	Promoter (<=1kb)
Igfbp2	25662	chr9	80079267	80079567	-38462	2.68	4.92E-03	1.42E-01	Distal Intergenic
Matn3	313954	chr6	34064093	34064393	-7035	-1.78	4.93E-03	1.42E-01	Distal Intergenic
Nxn	360577	chr10	64492228	64492528	57617	2.3	4.93E-03	1.42E-01	Intron (NM_001108285/360577, intron 1 of 7)
Tmem163	360839	chr13	44166665	44166965	178770	1.81	4.93E-03	1.42E-01	3' UTR
Klhl29	298867	chr6	29590946	29591246	-107486	1.79	4.94E-03	1.42E-01	Distal Intergenic
Plrg1	60376	chr2	182272216	182272516	231878	2.31	4.94E-03	1.42E-01	Distal Intergenic
Pabpc6	292295	chr1	51523889	51524189	-95686	-1.87	4.95E-03	1.42E-01	Distal Intergenic
Dtx3l	498089	chr11	67762845	67763145	4834	-2.15	4.96E-03	1.42E-01	Exon (NM_001109053/498089, exon 3 of 5)
Lmcd1	494021	chr4	144194467	144194767	1478	-1.75	4.96E-03	1.42E-01	Promoter (1-2kb)
Hoxc4	24459	chr7	144697664	144697964	50077	-1.81	4.97E-03	1.42E-01	Distal Intergenic
Irak3	314870	chr7	64972754	64973054	9170	-1.05	4.97E-03	1.42E-01	Intron (NM_001108101/314870, intron 1 of 11)
RGD1564664	499839	chr3	82844452	82844752	11419	-1.67	5.00E-03	1.43E-01	Distal Intergenic
Traf3ip2	361857	chr20	44667912	44668212	-12237	1.96	5.00E-03	1.43E-01	Distal Intergenic
Ckb	24264	chr6	136138760	136139060	6778	2.01	5.01E-03	1.43E-01	Distal Intergenic
Stam2	311030	chr3	38436782	38437082	-159349	-1.77	5.01E-03	1.43E-01	Distal Intergenic
Map3k3	303604	chr10	94309368	94309668	28665	2.21	5.02E-03	1.43E-01	Intron (NM_001107058/303604, intron 3 of 15)
Ube2f	363284	chr9	98356799	98357099	-37069	1.7	5.02E-03	1.43E-01	Intron (NM_031645/58965, intron 2 of 2)
Mrps16	688912	chr15	4308724	4309024	-42268	2.12	5.03E-03	1.43E-01	Distal Intergenic
Fndc3b	294925	chr2	113217360	113217660	127891	-1.99	5.04E-03	1.44E-01	Intron (NM_001191704/294925, intron 5 of 24)
Csrp1	29276	chr13	52452677	52452977	-100866	-2.02	5.05E-03	1.44E-01	Distal Intergenic
Myc	24577	chr7	102668357	102668657	82044	-1.99	5.06E-03	1.44E-01	Distal Intergenic
Gli2	304729	chr13	34819439	34819739	65746	2.28	5.06E-03	1.44E-01	Distal Intergenic
Jun	24516	chr5	114117344	114117644	-103067	1.01	5.06E-03	1.44E-01	Distal Intergenic
Oasl2	304549	chr12	47483514	47483814	-567	-1.79	5.08E-03	1.44E-01	Promoter (<=1kb)
Ash1l	310638	chr2	188277361	188277661	23983	-1.45	5.08E-03	1.44E-01	Intron (NM_001107689/310638, intron 2 of 25)
Ptprn2	29714	chr6	144437238	144437538	52465	-2.06	5.09E-03	1.44E-01	Intron (NM_031600/29714, intron 1 of 22)
Dpy19l3	308519	chr1	92066909	92067209	1951	-2.06	5.10E-03	1.44E-01	Promoter (1-2kb)
Epha4	316539	chr9	83203206	83203506	49952	-1.96	5.10E-03	1.44E-01	Intron (NM_001162411/316539, intron 3 of 17)
Tom1l2	360537	chr10	46714974	46715274	5636	2.22	5.10E-03	1.44E-01	Intron (NM_001108277/360537, intron 1 of 11)
Clic5	94272	chr9	19362200	19362500	10173	-2.3	5.10E-03	1.44E-01	Intron (NM_053603/94272, intron 1 of 6)
Arhgap22	306279	chr16	9607681	9607981	44463	-1.88	5.11E-03	1.44E-01	Exon (NM_001107297/306279, exon 4 of 6)
Sox4	364712	chr17	37612957	37613257	-1765	-1.94	5.11E-03	1.44E-01	Promoter (1-2kb)
Oasl	304545	chr12	47448532	47448832	7328	-1.78	5.11E-03	1.44E-01	Intron (NM_001009681/304545, intron 3 of 5)
Spns2	100270678	chr10	59037685	59037985	11497	1.75	5.11E-03	1.44E-01	Intron (NM_001144991/100270678, intron 1 of 12)
Fip1l1	289582	chr14	36175305	36175605	32673	-2.21	5.12E-03	1.44E-01	Intron (NM_001008295/289582, intron 9 of 15)
Siva1	362791	chr6	137149968	137150268	-59853	-1.88	5.12E-03	1.44E-01	Distal Intergenic
Agfg1	363266	chr9	88634686	88634986	27178	-2.07	5.12E-03	1.44E-01	Intron (NM_001135596/363266, intron 2 of 13)
Atp1b3	25390	chr8	104203338	104203638	17704	-1.63	5.12E-03	1.44E-01	Intron (NM_012913/25390, intron 1 of 6)
Desi1	315160	chr7	123259366	123259666	0	0.88	5.13E-03	1.45E-01	Promoter (<=1kb)
Rrnad1	361976	chr2	187309017	187309317	-6326	-1.73	5.13E-03	1.45E-01	Intron (NM_001007741/361977, intron 2 of 3)
Csgalnact1	306375	chr16	22841027	22841327	-138341	-1.67	5.14E-03	1.45E-01	Distal Intergenic
Rab10	50993	chr6	27698817	27699117	22003	-1.35	5.14E-03	1.45E-01	Intron (NM_017359/50993, intron 1 of 5)
Anapc7	304490	chr12	39600994	39601294	40705	-1.88	5.15E-03	1.45E-01	Intron (NM_001110139/29693, intron 20 of 20)
Ncam1	24586	chr8	54359439	54359739	-224708	2.25	5.15E-03	1.45E-01	Distal Intergenic
Mef2a	309957	chr1	128353540	128353840	-12277	2.29	5.15E-03	1.45E-01	Distal Intergenic
H2aj	690795	chr4	170763463	170763763	-3137	0.8	5.15E-03	1.45E-01	Distal Intergenic
Prkcd	170538	chr16	6686912	6687212	-11166	2.14	5.16E-03	1.45E-01	Distal Intergenic
Ltbp4	292734	chr1	84145208	84145508	4576	0.83	5.17E-03	1.45E-01	Intron (NM_001170336/292734, intron 4 of 32)
Git2	304546	chr12	47589981	47590281	0	1.18	5.17E-03	1.45E-01	Promoter (<=1kb)
Wdr1	360950	chr14	76990087	76990387	73	1.12	5.17E-03	1.45E-01	Promoter (<=1kb)
Ldhd	307858	chr19	43766247	43766547	82390	-1.57	5.18E-03	1.45E-01	Distal Intergenic
C1s	192262	chr4	157157520	157157820	-1924	-1.43	5.18E-03	1.45E-01	Promoter (1-2kb)
Nxnl2	689232	chr17	14226462	14226762	-167820	-1.73	5.20E-03	1.45E-01	Distal Intergenic
Myof	309499	chr1	256685277	256685577	49150	-1.25	5.20E-03	1.45E-01	Intron (NM_001354115/309499, intron 5 of 52)
Myo10	310178	chr2	77934189	77934489	65757	-1.86	5.20E-03	1.45E-01	Intron (NM_001107657/310178, intron 2 of 39)
Trim2	361970	chr2	183297060	183297360	-73241	-1.8	5.20E-03	1.45E-01	Distal Intergenic
Csnk1g3	64823	chr18	48863096	48863396	9925	-1.82	5.21E-03	1.45E-01	Intron (NM_022855/64823, intron 2 of 11)

Ldhd	307858	chr19	43774646	43774946	73991	-1.76	5.21E-03	1.45E-01	Distal Intergenic
Zbtb10	80338	chr2	94529268	94529568	201408	2.06	5.22E-03	1.45E-01	Distal Intergenic
Megf8	114029	chr1	82182591	82182891	-2143	2.26	5.23E-03	1.45E-01	Promoter (2-3kb)
Kif3b	296284	chr3	148933315	148933615	160056	2.46	5.23E-03	1.45E-01	Distal Intergenic
Lrp1	299858	chr7	70936141	70936441	-9238	-1.55	5.23E-03	1.45E-01	Distal Intergenic
Saa4	365245	chr1	102801052	102801352	-20671	1.95	5.23E-03	1.45E-01	Distal Intergenic
Tgm2	56083	chr3	154654373	154654673	-27116	-2.15	5.23E-03	1.45E-01	Distal Intergenic
Wac	307029	chr17	61463703	61464003	-517073	2.24	5.24E-03	1.45E-01	Distal Intergenic
Man2a1	25478	chr9	112461948	112462248	168560	1.43	5.24E-03	1.45E-01	Distal Intergenic
Lif	60584	chr14	84521630	84521930	25160	-1.55	5.24E-03	1.45E-01	Distal Intergenic
Gclc	25283	chr8	85136666	85136966	77615	-1.72	5.24E-03	1.45E-01	Distal Intergenic
Phtf2	296762	chr4	10858350	10858650	35092	-1.5	5.24E-03	1.45E-01	Intron (NM_001106577/296762, intron 2 of 17)
Ttc39c	686179	chr18	3843921	3844221	-114211	-1.54	5.24E-03	1.45E-01	Distal Intergenic
LOC100910802	100910802	chr12	22791852	22792152	7210	-2.08	5.24E-03	1.45E-01	Intron (NR_102359/100910802, intron 3 of 3)
Rnf216	304294	chr12	13573473	13573773	65044	2.13	5.24E-03	1.45E-01	Intron (NM_001107122/304294, intron 13 of 17)
Pdlim5	64353	chr2	247907667	247907967	80495	-1.81	5.26E-03	1.46E-01	Intron (NM_053326/64353, intron 3 of 12)
Carmil1	306941	chr17	43054594	43054894	3633	-1.74	5.26E-03	1.46E-01	Intron (NM_001191692/306941, intron 1 of 37)
Dennd2b	308944	chr1	174259299	174259599	30048	2.22	5.26E-03	1.46E-01	Intron (NM_001107547/308944, intron 1 of 19)
Zmynd8	296374	chr3	162551560	162551860	27341	-1.6	5.26E-03	1.46E-01	Intron (NM_001100838/296374, intron 2 of 21)
LOC499584	499584	chr2	109384459	109384759	-921604	1.78	5.26E-03	1.46E-01	Distal Intergenic
Dip2c	307067	chr17	63336655	63336955	-90061	1.39	5.27E-03	1.46E-01	Distal Intergenic
Cln5	306128	chr15	93624087	93624387	-10428	-1.73	5.27E-03	1.46E-01	Distal Intergenic
Spats2l	316426	chr9	64749534	64749834	4483	0.74	5.27E-03	1.46E-01	Intron (NM_001014102/316426, intron 1 of 11)
Zfp579	308339	chr1	72399247	72399547	90	1.12	5.27E-03	1.46E-01	Promoter (<=1kb)
Thnsl1	498805	chr17	88119727	88120027	23897	-1.44	5.29E-03	1.46E-01	Distal Intergenic
Spin2a	317395	chrX	18238681	18238981	103415	2.77	5.30E-03	1.46E-01	Distal Intergenic
Unc119b	288702	chr12	47230685	47230985	-8678	1.87	5.32E-03	1.47E-01	Distal Intergenic
Nol10	313981	chr6	42656511	42656811	0	0.7	5.33E-03	1.47E-01	Promoter (<=1kb)
Oat	64313	chr1	204429889	204430189	151881	-1.62	5.33E-03	1.47E-01	Distal Intergenic
Prdx1	117254	chr5	135539846	135540146	3433	-1.69	5.33E-03	1.47E-01	Intron (NM_057114/117254, intron 2 of 5)
Mir28	100314152	chr11	79474041	79474341	-93433	-1.95	5.33E-03	1.47E-01	Intron (NM_001013864/288010, intron 5 of 11)
Adam17	57027	chr6	43448038	43448338	0	0.79	5.33E-03	1.47E-01	Promoter (<=1kb)
Fbxo43	315034	chr7	74899331	74899631	39032	-1.44	5.33E-03	1.47E-01	Distal Intergenic
Dtnbp1	641528	chr17	20179787	20180087	89651	-1.98	5.34E-03	1.47E-01	Intron (NM_001037664/641528, intron 7 of 8)
Ptf1a	117034	chr17	86415053	86415353	215430	-1.98	5.34E-03	1.47E-01	Distal Intergenic
Top1	64550	chr3	156663546	156663846	27858	-1.98	5.34E-03	1.47E-01	Intron (NM_022615/64550, intron 3 of 20)
Srrm1	313620	chr5	153710886	153711186	-2772	2.3	5.34E-03	1.47E-01	Promoter (2-3kb)
B4galnt3	500306	chr4	152845377	152845677	-10195	-1.79	5.35E-03	1.47E-01	Distal Intergenic
Faap20	362678	chr5	172610696	172610996	-37954	-1.58	5.36E-03	1.47E-01	Distal Intergenic
Slc11a1	316519	chr9	81662705	81663005	7009	-1.88	5.36E-03	1.47E-01	Exon (NM_001031658/316519, exon 8 of 14)
Usf1	83586	chr13	89811463	89811763	13663	2	5.36E-03	1.47E-01	Distal Intergenic
LOC301165	301165	chr9	4513635	4513935	-120422	-1.82	5.36E-03	1.47E-01	Intron (NM_001013177/316153, intron 4 of 8)
Mir297	100314063	chr10	101775283	101775583	-78945	-2.27	5.37E-03	1.47E-01	Intron (NM_001013042/287796, intron 7 of 9)
Depdc1	295538	chr2	264704640	264704940	0	0.97	5.37E-03	1.47E-01	Promoter (<=1kb)
RGD1304587	303180	chr10	45558756	45559056	-522	-2.02	5.38E-03	1.47E-01	Promoter (<=1kb)
Cyp24a1	25279	chr3	168215557	168215857	-103637	-1.76	5.38E-03	1.47E-01	Distal Intergenic
Gas8	361438	chr19	56355272	56355572	38828	-1.94	5.39E-03	1.47E-01	Distal Intergenic
Ndufa9	362440	chr4	159353167	159353467	46154	-2.14	5.39E-03	1.47E-01	Distal Intergenic
Thoc7	305714	chr15	12569277	12569577	42	1.19	5.39E-03	1.47E-01	Promoter (<=1kb)
Ppp3ca	24674	chr2	241923972	241924272	14140	-1.87	5.39E-03	1.47E-01	Intron (NM_017041/24674, intron 1 of 13)
Tomm20	266601	chr19	59380559	59380859	492552	-1.76	5.40E-03	1.47E-01	Distal Intergenic
N6amt1	288309	chr11	26905199	26905499	-98836	2.39	5.42E-03	1.47E-01	Distal Intergenic
Spen	690911	chr5	160067628	160067928	2987	-1.67	5.43E-03	1.48E-01	Promoter (2-3kb)
Eif6	305506	chr3	151346637	151346937	16178	2.4	5.45E-03	1.48E-01	Intron (NM_031757/83513, intron 12 of 15)
Dennd2b	308944	chr1	174232482	174232782	56865	1.94	5.45E-03	1.48E-01	Intron (NM_001107547/308944, intron 2 of 19)
Herc3	362377	chr4	89087495	89087795	8784	-1.55	5.45E-03	1.48E-01	Intron (NM_001108631/362377, intron 2 of 25)
Usp12	360763	chr12	10060991	10061291	23305	-1.79	5.45E-03	1.48E-01	Intron (NM_001166576/360763, intron 1 of 8)
Metrnl	316842	chr10	110992874	110993174	99613	2.49	5.46E-03	1.48E-01	Distal Intergenic
Reep3	294375	chr20	22984791	22985091	71142	-1.61	5.46E-03	1.48E-01	Intron (NM_001106386/294375, intron 5 of 7)
Rras2	365355	chr1	178987700	178988000	22257	-1.4	5.46E-03	1.48E-01	Intron (NM_001013434/365355, intron 1 of 5)
Taf1a	360893	chr13	102197275	102197575	426997	1.76	5.47E-03	1.48E-01	Distal Intergenic
Arhgap22	306279	chr16	9497074	9497374	-65844	-1.89	5.47E-03	1.48E-01	Distal Intergenic
G3bp1	171092	chr10	40853171	40853471	40313	-1.84	5.48E-03	1.48E-01	Downstream (2-3kb)
S1pr3	306792	chr17	13812260	13812560	55	0.7	5.49E-03	1.49E-01	Promoter (<=1kb)
Aptr	104968332	chr4	10759188	10759488	-9897	-1.6	5.50E-03	1.49E-01	Intron (NM_001135872/311987, intron 1 of 7)
Eif2s2	296302	chr3	150394973	150395273	16906	-2.37	5.50E-03	1.49E-01	Intron (NM_199380/296302, intron 7 of 8)
Sgsm1	288743	chr12	49413265	49413565	83831	1.74	5.50E-03	1.49E-01	Distal Intergenic
Rundc3b	688590	chr4	22445347	22445647	0	1.5	5.50E-03	1.49E-01	Promoter (<=1kb)
N6amt1	288309	chr11	26660951	26661251	-343084	-1.96	5.52E-03	1.49E-01	Distal Intergenic
Flrt2	299236	chr6	119526242	119526542	6528	-1.79	5.53E-03	1.49E-01	Intron (NM_001106750/299236, intron 1 of 1)
Adam17	57027	chr6	43437821	43438121	10159	-1.4	5.53E-03	1.49E-01	Intron (NM_020306/57027, intron 1 of 18)
Atg14	305831	chr15	24432089	24432389	-57313	2.03	5.53E-03	1.49E-01	Distal Intergenic
Vom1r90	266771	chr4	121678176	121678476	5785	2.29	5.54E-03	1.49E-01	Intron (NM_153729/266771, intron 2 of 2)
Ddx1	84474	chr6	38736663	38736963	-282901	-1.73	5.54E-03	1.49E-01	Distal Intergenic
Adpgk	315722	chr8	64049655	64049955	-38958	-1.9	5.55E-03	1.49E-01	Distal Intergenic
Cd180	294706	chr2	32739582	32739882	-80440	-1.95	5.55E-03	1.49E-01	Distal Intergenic
Fam209	296411	chr3	170483974	170484274	8143	2.12	5.56E-03	1.50E-01	Distal Intergenic
Mex3c	307271	chr18	69558346	69558646	8409	-1.77	5.58E-03	1.50E-01	Intron (NM_001107377/307271, intron 1 of 1)
Pak1	29431	chr1	162825678	162825978	57522	2.36	5.58E-03	1.50E-01	Intron (NM_017198/29431, intron 1 of 14)
Rab11fip1	498650	chr16	69062122	69062422	13383	-1.66	5.58E-03	1.50E-01	Intron (NM_001191555/498650, intron 1 of 5)
Sgk1	29517	chr1	24596155	24596455	-402588	-1.84	5.58E-03	1.50E-01	Distal Intergenic
Pabpc1	171350	chr7	75384711	75385011	36863	-2.02	5.59E-03	1.50E-01	Distal Intergenic
LOC100362981	100362981	chr8	33148516	33148816	-27514	1.34	5.59E-03	1.50E-01	Distal Intergenic
Map1a	25152	chr3	113257814	113258114	126	0.66	5.60E-03	1.50E-01	Promoter (<=1kb)
Timm22	79463	chr10	64567242	64567542	11178	1.97	5.61E-03	1.50E-01	3' UTR
Xbp1	289754	chr14	85655261	85655561	-98175	2.29	5.61E-03	1.50E-01	Distal Intergenic
Rab5a	64633	chr14	72606439	72606739	-282506	-1.87	5.62E-03	1.50E-01	Distal Intergenic
Oxnad1	306270	chr16	8176351	8176651	-30572	0.98	5.63E-03	1.50E-01	Distal Intergenic
Vps4b	360834	chr13	26863426	26863726	-12966	-2.01	5.63E-03	1.50E-01	Distal Intergenic
Scfd2	498353	chr14	36345782	36346082	129780	-2.01	5.63E-03	1.50E-01	Intron (NM_001017499/498353, intron 5 of 8)
Gcnt2	306860	chr17	21648940	21649240	28237	-2.01	5.63E-03	1.50E-01	Intron (NM_001001511/306860, intron 1 of 2)
Zbtb43	311872	chr3	12493721	12494021	15284	-2.01	5.63E-03	1.50E-01	Exon (NM_001012094/311872, exon 4 of 4)
Gns	299825	chr7	65463847	65464147	-152251	-2.01	5.63E-03	1.50E-01	Distal Intergenic
Abhd8	306338	chr16	19855241	19855541	17224	-1.3	5.64E-03	1.50E-01	Distal Intergenic

Ppm1d	287585	chr10	72985570	72985870	76020	-1.04	5.65E-03	1.51E-01	Intron (NM_001173430/363662, intron 13 of 17)
Igf2r	25151	chr1	48153843	48154143	-21952	-1.96	5.66E-03	1.51E-01	Distal Intergenic
Cmpk1	298410	chr5	133771534	133771834	14618	-1.35	5.66E-03	1.51E-01	Intron (NM_001025655/298410, intron 1 of 5)
Sh3bp5	117186	chr16	7516343	7516643	21558	2.22	5.67E-03	1.51E-01	Intron (NM_054011/117186, intron 2 of 8)
Emc8	361425	chr19	54220592	54220892	24918	2.52	5.68E-03	1.51E-01	Distal Intergenic
Adck1	366698	chr6	111642214	111642514	0	0.79	5.68E-03	1.51E-01	Promoter (<=1kb)
Suclg1	114597	chr4	101181318	101181618	0	0.74	5.69E-03	1.51E-01	Promoter (<=1kb)
Epop	691153	chr10	85574621	85574921	0	1.18	5.69E-03	1.51E-01	Promoter (<=1kb)
Sema3c	296787	chr4	14511221	14511521	-22996	-1.85	5.69E-03	1.51E-01	Distal Intergenic
Parp16	315760	chr8	70630776	70631076	-81704	1.3	5.70E-03	1.51E-01	Distal Intergenic
D2hgdh	301624	chr9	100956745	100957045	145	0.64	5.70E-03	1.51E-01	Promoter (<=1kb)
Susd3	306810	chr17	15802490	15802790	-11342	1.16	5.72E-03	1.52E-01	Distal Intergenic
Efs	290212	chr15	33587810	33588110	1184	1.78	5.73E-03	1.52E-01	Promoter (1-2kb)
RGD1562310	498188	chr12	43921096	43921396	19402	1.9	5.73E-03	1.52E-01	3' UTR
Specc1l	361828	chr20	14384395	14384695	9177	-1.54	5.74E-03	1.52E-01	Intron (NM_001039455/361828, intron 1 of 15)
Tfg	360709	chr11	46421617	46421917	241370	2.43	5.74E-03	1.52E-01	Distal Intergenic
Trim29	300656	chr8	47649491	47649791	-24530	1.62	5.75E-03	1.52E-01	Distal Intergenic
Btg1	29618	chr7	37822842	37823142	10011	-1.61	5.75E-03	1.52E-01	Distal Intergenic
Map3k7	313121	chr9	113986253	113986553	-35482	-1.85	5.75E-03	1.52E-01	Distal Intergenic
Gask1b	310540	chr2	178904076	178904376	225035	2.32	5.76E-03	1.52E-01	Distal Intergenic
Dmrt1	114498	chr1	243401292	243401592	-75901	1.91	5.76E-03	1.52E-01	Distal Intergenic
Slit2	360272	chr14	67234497	67234797	-64136	-1.63	5.76E-03	1.52E-01	Distal Intergenic
Nanp	311530	chr3	146773414	146773714	39274	2.03	5.77E-03	1.52E-01	Distal Intergenic
Fa2h	307855	chr19	43660195	43660495	-63407	-1.79	5.77E-03	1.52E-01	Distal Intergenic
Aplnr	83518	chr3	72517873	72518173	-22365	-1.71	5.77E-03	1.52E-01	Distal Intergenic
Osbpl8	314824	chr7	53801005	53801305	-77601	1.76	5.77E-03	1.52E-01	Distal Intergenic
Opn3	498289	chr13	93767207	93767507	-20213	1	5.78E-03	1.52E-01	Distal Intergenic
Pacrg	499021	chr1	50446437	50446737	376164	2.39	5.79E-03	1.52E-01	Intron (NM_001077677/499021, intron 4 of 4)
Iqgap1	361598	chr1	142600553	142600853	14820	-1.51	5.79E-03	1.52E-01	Intron (NM_001108489/361598, intron 2 of 37)
Phlpp1	59265	chr13	26294297	26294597	122082	-1.86	5.79E-03	1.52E-01	Intron (NM_021657/59265, intron 3 of 16)
Csde1	117180	chr2	205522896	205523196	-1998	-1.86	5.79E-03	1.52E-01	Promoter (1-2kb)
Upp1	289801	chr14	89325148	89325448	9459	-1.67	5.80E-03	1.52E-01	Intron (NM_001030025/289801, intron 2 of 7)
Stard3nl	291182	chr17	47666560	47666860	4007	-1.57	5.80E-03	1.52E-01	Intron (NM_001008298/291182, intron 1 of 8)
Eea1	314764	chr7	37338188	37338488	236774	-1.94	5.80E-03	1.52E-01	Distal Intergenic
LOC100362981	100362981	chr8	33169694	33169994	-48692	1.45	5.82E-03	1.53E-01	Distal Intergenic
Irak2	362418	chr4	145600711	145601011	6136	-1.43	5.83E-03	1.53E-01	Intron (NM_001025422/362418, intron 1 of 12)
Cnot6	287249	chr10	35185446	35185746	9380	1.52	5.83E-03	1.53E-01	Intron (NM_001013856/287249, intron 2 of 14)
Cttnbp2nl	310760	chr2	207541442	207541742	0	0.81	5.83E-03	1.53E-01	Promoter (<=1kb)
Zfp217	311764	chr3	167004356	167004656	-10416	1.26	5.83E-03	1.53E-01	Distal Intergenic
Per3	78962	chr5	168123131	168123431	-101	1.11	5.84E-03	1.53E-01	Promoter (<=1kb)
Sh3bp4	64634	chr9	96415721	96416021	204971	2.3	5.84E-03	1.53E-01	Distal Intergenic
Rfx3	361746	chr1	246469083	246469383	-358541	2.17	5.84E-03	1.53E-01	Distal Intergenic
Mark3	170577	chr6	136037880	136038180	-3879	2.24	5.84E-03	1.53E-01	Distal Intergenic
Enoph1	305177	chr14	11192423	11192723	5471	-1.69	5.84E-03	1.53E-01	Intron (NM_001009391/305177, intron 1 of 5)
Pitrm1	307081	chr17	68416050	68416350	-61117	1.8	5.86E-03	1.53E-01	Distal Intergenic
Zfp84	308482	chr1	87664360	87664660	17309	1.76	5.86E-03	1.53E-01	Distal Intergenic
Abhd2	293050	chr1	141009797	141010097	11557	-1.39	5.86E-03	1.53E-01	Intron (NM_001106275/293050, intron 1 of 10)
Rai14	294804	chr2	60644695	60644995	38456	-1.53	5.86E-03	1.53E-01	Intron (NM_001011947/294804, intron 2 of 16)
Sesn3	315427	chr8	12832613	12832913	9458	2.21	5.87E-03	1.53E-01	Intron (NM_001108125/315427, intron 1 of 9)
Rdh14	500629	chr6	36267702	36268002	160776	2.24	5.87E-03	1.53E-01	Distal Intergenic
Rabep1	54190	chr10	57462428	57462728	0	1.77	5.88E-03	1.53E-01	Promoter (<=1kb)
Hus1b	691382	chr17	35380251	35380551	-43467	-1.75	5.88E-03	1.53E-01	Distal Intergenic
Inhba	29200	chr17	51416980	51417280	495143	-2.25	5.89E-03	1.53E-01	Distal Intergenic
Wdr18	314617	chr7	12570863	12571163	-1743	2.26	5.89E-03	1.53E-01	Promoter (1-2kb)
Rn5-8s	100861534	chr1	11971606	11971906	1515	2.43	5.89E-03	1.53E-01	Promoter (1-2kb)
Rnf4	29274	chr14	81638668	81638968	40685	1.01	5.91E-03	1.53E-01	Distal Intergenic
Ap4s1	366618	chr6	72433257	72433557	-28420	-1.56	5.91E-03	1.53E-01	Intron (NM_001029897/114520, intron 1 of 17)
Sgcg	305941	chr15	41490282	41490582	104693	-1.87	5.91E-03	1.53E-01	Distal Intergenic
Mertk	65037	chr3	121275513	121275813	40283	1.16	5.92E-03	1.53E-01	Intron (NM_022943/65037, intron 4 of 18)
Ifrd1	29596	chr6	60440738	60441038	-289384	1.47	5.93E-03	1.53E-01	Distal Intergenic
Mir147	100314100	chr3	114791181	114791481	15838	-1.58	5.93E-03	1.53E-01	Distal Intergenic
Nxn	360577	chr10	64479614	64479914	70231	2.16	5.93E-03	1.53E-01	Intron (NM_001108285/360577, intron 1 of 7)
Zfp217	311764	chr3	166993448	166993748	192	-1.76	5.93E-03	1.53E-01	Promoter (<=1kb)
Zfp131	310375	chr2	52772591	52772891	-241506	-1.79	5.93E-03	1.53E-01	Distal Intergenic
Ercc1	292673	chr1	80243345	80243645	-13328	-1.55	5.94E-03	1.53E-01	Distal Intergenic
Stat1	25124	chr9	54340666	54340966	-12708	-2.06	5.94E-03	1.53E-01	3' UTR
Cul3	301555	chr9	86107227	86107527	-4069	-2.06	5.94E-03	1.53E-01	Distal Intergenic
RGD1562885	502412	chr20	3704337	3704637	-13173	0.67	5.94E-03	1.53E-01	Distal Intergenic
Dcaf7	303602	chr10	94229886	94230186	-1399	-1.76	5.94E-03	1.53E-01	Promoter (1-2kb)
Foxe3	171302	chr5	133238963	133239263	486393	1.45	5.95E-03	1.53E-01	Distal Intergenic
Scgb1c1	309100	chr1	213594817	213595117	-123	-1.92	5.95E-03	1.53E-01	Promoter (<=1kb)
LOC501038	501038	chr8	103399769	103400069	56964	-1.82	5.96E-03	1.54E-01	Distal Intergenic
Fem1a	316131	chr9	10788610	10788910	12230	-1.71	5.97E-03	1.54E-01	Distal Intergenic
Nod1	500133	chr4	85150927	85151227	23704	-1.49	5.97E-03	1.54E-01	Intron (NM_001109236/500133, intron 1 of 11)
LOC100302465	100302465	chr1	206299201	206299501	117642	-1.67	5.97E-03	1.54E-01	Distal Intergenic
Meis2	311311	chr3	107310761	107311061	449528	1.4	5.98E-03	1.54E-01	Distal Intergenic
Ier5l	499772	chr3	9019254	9019554	18388	-1.52	5.99E-03	1.54E-01	Distal Intergenic
Arhgap21	307178	chr17	87334938	87335238	514491	-1.66	5.99E-03	1.54E-01	Distal Intergenic
Fam122a	309420	chr1	242389983	242390283	-16219	-1.84	5.99E-03	1.54E-01	Intron (NM_001012743/309419, intron 1 of 14)
Dennd1a	311913	chr3	22356671	22356971	143794	2.23	5.99E-03	1.54E-01	Intron (NM_001191747/311913, intron 4 of 20)
St6gal1	25197	chr11	81023376	81023676	-41954	1.46	5.99E-03	1.54E-01	Distal Intergenic
Gaa	367562	chr10	108393442	108393742	-2247	-1.55	6.00E-03	1.54E-01	Promoter (2-3kb)
Myc	24577	chr7	102788160	102788460	201847	-1.75	6.00E-03	1.54E-01	Distal Intergenic
Tnip1	363599	chr10	40313105	40313405	-10033	-1.99	6.01E-03	1.54E-01	Distal Intergenic
Rhebl1	359959	chr7	140547075	140547375	9264	1.12	6.02E-03	1.54E-01	Distal Intergenic
Slc25a37	306000	chr15	51158625	51158925	9459	-1.49	6.02E-03	1.54E-01	Intron (NM_001013996/306000, intron 1 of 3)
Marchf7	311059	chr3	46300074	46300374	8160	-1.82	6.02E-03	1.54E-01	Intron (NM_001012087/311059, intron 1 of 9)
Cyld	312937	chr19	19261182	19261482	62335	2.34	6.03E-03	1.54E-01	Distal Intergenic
Vezf1	287615	chr10	75442476	75442776	76665	2.03	6.03E-03	1.54E-01	Intron (NM_001013971/303419, intron 1 of 10)
Col1a2	84352	chr4	29638813	29639113	108959	1.18	6.03E-03	1.54E-01	Distal Intergenic
Lsm2	684148	chr20	4969295	4969595	2101	-2.12	6.03E-03	1.54E-01	Promoter (2-3kb)
Rasgrp3	313874	chr6	21130931	21131231	4649	2.2	6.04E-03	1.54E-01	Intron (NM_001108009/313874, intron 1 of 15)
Chst3	84468	chr20	29763340	29763640	-24932	1.71	6.04E-03	1.54E-01	Distal Intergenic
Tmigd1	363654	chr10	63268000	63268300	6298	1.77	6.07E-03	1.55E-01	Intron (NM_001135029/363654, intron 3 of 5)

Ldhd	307858	chr19	43775879	43776179	72758	-1.71	6.08E-03	1.55E-01	Distal Intergenic
Fbxl22	363083	chr8	72201732	72202032	2698	0.75	6.09E-03	1.55E-01	Promoter (2-3kb)
Sag	25539	chr9	94999551	94999851	72485	-1.66	6.09E-03	1.55E-01	Distal Intergenic
Llph	299818	chr6	125866853	125867153	2831	-1.47	6.10E-03	1.55E-01	Promoter (2-3kb)
Phc2	313038	chr5	146907561	146907861	24805	0.92	6.11E-03	1.56E-01	Intron (NM_001013169/313038, intron 8 of 13)
Gnpda1	683570	chr18	31500693	31500993	-11539	-1.32	6.13E-03	1.56E-01	Distal Intergenic
Fst	24373	chr2	46723843	46724143	-179391	-1.6	6.13E-03	1.56E-01	Distal Intergenic
Cyp19a1	25147	chr8	58765496	58765796	6612	-1.48	6.13E-03	1.56E-01	Intron (NM_017085/25147, intron 2 of 9)
Nxnl2	689232	chr17	14287886	14288186	-229244	-1.82	6.13E-03	1.56E-01	Distal Intergenic
Foxp1	297480	chr4	131846269	131846569	-247965	-1.77	6.14E-03	1.56E-01	Distal Intergenic
Acod1	306127	chr15	93574015	93574315	-38653	-2.06	6.14E-03	1.56E-01	Distal Intergenic
Acad11	315973	chr8	112604789	112605089	10031	1.47	6.15E-03	1.56E-01	Intron (NM_001108181/315973, intron 2 of 19)
Atxn10	170821	chr7	126236973	126237273	8557	-1.56	6.15E-03	1.56E-01	Intron (NM_133313/170821, intron 1 of 11)
Ppp1r14a	114004	chr1	87230057	87230357	1476	-1.98	6.15E-03	1.56E-01	Promoter (1-2kb)
Ilvbl	362843	chr7	14053630	14053930	709	0.81	6.15E-03	1.56E-01	Promoter (<=1kb)
Gpr20	60667	chr7	114889521	114889821	55523	1.3	6.15E-03	1.56E-01	Distal Intergenic
Plpp4	309014	chr1	202821644	202821944	389278	1.76	6.15E-03	1.56E-01	Distal Intergenic
Irak4	300177	chr7	135492353	135492653	-311070	1.03	6.16E-03	1.56E-01	Distal Intergenic
Phlda1	29380	chr7	54341716	54342016	94256	2.08	6.16E-03	1.56E-01	Distal Intergenic
Cdc42	64465	chr5	155712074	155712374	15926	-1.9	6.18E-03	1.56E-01	Intron (NM_171994/64465, intron 1 of 5)
Ryk	140585	chr8	111357483	111357783	31129	-1.9	6.18E-03	1.56E-01	Intron (NM_080402/140585, intron 2 of 14)
Sgms2	310849	chr2	236503559	236503859	-23057	-1.69	6.18E-03	1.56E-01	Distal Intergenic
Runx2	367218	chr9	18708487	18708787	64917	-1.62	6.19E-03	1.56E-01	Intron (NM_001278483/367218, intron 5 of 8)
LOC498592	498592	chr7	91160853	91161153	87439	-1.76	6.20E-03	1.56E-01	Distal Intergenic
Prex2	312912	chr5	8027980	8028280	-153071	2.06	6.20E-03	1.56E-01	Distal Intergenic
Arhgap11a	296060	chr3	105345971	105346271	-47863	-1.29	6.20E-03	1.56E-01	Distal Intergenic
Bhmt	81508	chr2	23136842	23137142	119016	-1.43	6.20E-03	1.56E-01	Distal Intergenic
Osr2	315039	chr7	74047838	74048138	18	0.74	6.21E-03	1.56E-01	Promoter (<=1kb)
Slc41a3	641603	chr4	123352888	123353188	141554	0.63	6.22E-03	1.56E-01	Distal Intergenic
Rras2	365355	chr1	179059948	179060248	-49691	-2.02	6.22E-03	1.56E-01	Distal Intergenic
Cd48	245962	chr13	90096393	90096693	-20150	-2.04	6.22E-03	1.56E-01	Distal Intergenic
Smad4	50554	chr18	69661771	69662071	-4398	-2.04	6.22E-03	1.56E-01	Distal Intergenic
Btg1	29618	chr7	37483796	37484096	-328735	-2.04	6.22E-03	1.56E-01	Distal Intergenic
Nsrp1	303346	chr10	63070366	63070666	-27449	-1.47	6.23E-03	1.56E-01	Distal Intergenic
Cbr4	359725	chr16	31687112	31687412	285095	0.7	6.25E-03	1.57E-01	Distal Intergenic
Prkacb	293508	chr2	252757931	252758231	-66045	-1.7	6.25E-03	1.57E-01	Distal Intergenic
Ankmy2	314046	chr6	55653845	55654145	5824	-1.65	6.25E-03	1.57E-01	Intron (NM_001108019/314046, intron 1 of 8)
Slc6a6	29464	chr4	123693832	123694132	19187	-1.79	6.27E-03	1.57E-01	Intron (NM_017206/29464, intron 2 of 14)
Abhd2	293050	chr1	141047246	141047546	49006	-1.88	6.27E-03	1.57E-01	Intron (NM_001106275/293050, intron 3 of 10)
Foxp1	297480	chr4	131391116	131391416	206888	-1.64	6.27E-03	1.57E-01	Intron (NM_001034131/297480, intron 9 of 15)
Nek2	114482	chr13	110507478	110507778	-3768	1.2	6.28E-03	1.57E-01	Distal Intergenic
Plpp4	309014	chr1	202825401	202825701	393035	2.42	6.28E-03	1.57E-01	Distal Intergenic
Trpt1	293704	chr1	222260345	222260645	9350	-1.57	6.29E-03	1.57E-01	Intron (NM_001127543/309186, intron 6 of 14)
Etaa1	498420	chr14	102438979	102439279	-145302	2.18	6.29E-03	1.57E-01	Distal Intergenic
Rxra	25271	chr3	6311459	6311759	99643	1.07	6.29E-03	1.57E-01	Distal Intergenic
Scaf11	312030	chr7	137914259	137914559	-57922	0.83	6.30E-03	1.57E-01	Distal Intergenic
Cript	56725	chr6	10590490	10590790	-3357	2.5	6.31E-03	1.58E-01	Distal Intergenic
Hspa5	25617	chr3	13763143	13763443	-74861	1.21	6.33E-03	1.58E-01	Distal Intergenic
Gcgr	24953	chr10	109692845	109693145	-14817	-1.8	6.34E-03	1.58E-01	Distal Intergenic
Fto	291905	chr19	17044703	17045003	70095	1.51	6.35E-03	1.58E-01	Intron (NM_001039713/291905, intron 1 of 8)
Slc35b1	287642	chr10	83183830	83184130	-17402	1.97	6.35E-03	1.58E-01	Intron (NM_001109039/497983, intron 2 of 7)
Bend6	363212	chr9	38114217	38114517	-182736	-1.7	6.35E-03	1.58E-01	Distal Intergenic
Pim1	24649	chr20	8167461	8167761	2038	-1.34	6.36E-03	1.58E-01	Promoter (2-3kb)
RGD1311517	313775	chr5	173529552	173529852	-12506	2.28	6.37E-03	1.58E-01	Distal Intergenic
Dnm2	25751	chr8	22468381	22468681	9441	1.83	6.37E-03	1.58E-01	Intron (NM_013199/25751, intron 1 of 20)
Maml3	310405	chr2	141101879	141102179	174707	-1.47	6.39E-03	1.59E-01	Intron (NM_001107675/310405, intron 1 of 5)
Cpz	83575	chr14	80384296	80384596	-18386	1.92	6.39E-03	1.59E-01	Distal Intergenic
Tmem263	362857	chr7	24667818	24668118	-517	0.8	6.39E-03	1.59E-01	Promoter (<=1kb)
Nipsnap1	360971	chr14	85113605	85113905	27	0.69	6.39E-03	1.59E-01	Promoter (<=1kb)
Gak	81659	chr14	2143839	2144139	43733	-1.84	6.40E-03	1.59E-01	Intron (NM_031030/81659, intron 18 of 27)
Nkd1	364952	chr19	19463955	19464255	44832	1.8	6.41E-03	1.59E-01	Intron (NM_001271381/364952, intron 4 of 10)
Smap2	298500	chr5	139944153	139944453	-12787	-1.74	6.42E-03	1.59E-01	Distal Intergenic
Fcgr3a	304966	chr13	89365312	89365612	-20407	-2.16	6.42E-03	1.59E-01	Distal Intergenic
Atp23	299828	chr7	70198416	70198716	0	0.78	6.42E-03	1.59E-01	Promoter (<=1kb)
Elf2	361944	chr2	140330600	140330900	4012	-1.7	6.42E-03	1.59E-01	Intron (NM_001012181/361944, intron 1 of 6)
Rexo1	314630	chr7	12018842	12019142	-3126	1.62	6.42E-03	1.59E-01	Distal Intergenic
Mir760	100314118	chr2	225872937	225873237	196	1.37	6.42E-03	1.59E-01	Promoter (<=1kb)
Plec	64204	chr7	117230260	117230560	24205	2.64	6.43E-03	1.59E-01	3' UTR
Lpcat1	361467	chr1	32415377	32415677	14240	-1.37	6.44E-03	1.59E-01	Intron (NM_001100735/361467, intron 1 of 13)
Nufip1	364430	chr15	58238454	58238754	67291	1.9	6.46E-03	1.59E-01	Distal Intergenic
Col5a2	85250	chr9	52203688	52203988	34747	-1.91	6.46E-03	1.59E-01	Intron (NM_053488/85250, intron 1 of 55)
Pitrm1	307081	chr17	68431924	68432224	-45243	1.79	6.46E-03	1.59E-01	Distal Intergenic
Slc10a4	305309	chr14	37769777	37770077	0	1.71	6.46E-03	1.59E-01	Promoter (<=1kb)
Fto	291905	chr19	16950807	16951107	163991	-1.46	6.47E-03	1.60E-01	Intron (NM_001039713/291905, intron 7 of 8)
Tprn	499749	chr3	2483247	2483547	3015	1.1	6.48E-03	1.60E-01	Intron (NM_001024309/499749, intron 1 of 3)
Jag1	29146	chr3	130012322	130012622	102159	2.42	6.49E-03	1.60E-01	Intron (NM_001271320/499895, intron 3 of 7)
Ccdc174	297458	chr4	123748705	123749005	-11738	2.13	6.52E-03	1.60E-01	Distal Intergenic
Thbs2	292406	chr1	56683062	56683362	209	0.79	6.52E-03	1.60E-01	Promoter (<=1kb)
Atp2b1	29598	chr7	41152962	41153262	38265	-1.75	6.52E-03	1.60E-01	Intron (NM_053311/29598, intron 1 of 21)
Asb13	361268	chr17	70186377	70186677	47426	-1.32	6.53E-03	1.61E-01	Distal Intergenic
Sertad4	360899	chr13	111584694	111584994	0	0.87	6.54E-03	1.61E-01	Promoter (<=1kb)
Egln2	308457	chr1	83977251	83977551	-15	1.2	6.54E-03	1.61E-01	Promoter (<=1kb)
Eif2b3	171145	chr5	135875467	135875767	-7211	-1.8	6.55E-03	1.61E-01	Distal Intergenic
Ehf	295965	chr3	93103164	93103464	112952	2.18	6.56E-03	1.61E-01	Distal Intergenic
Npepo	290963	chr17	608661	608961	50477	-1.28	6.56E-03	1.61E-01	Intron (NM_001012346/290963, intron 3 of 14)
Pvr	25066	chr1	80825467	80825767	9945	-1.58	6.57E-03	1.61E-01	Intron (NM_017076/25066, intron 4 of 7)
Scg5	25719	chr3	105258513	105258813	20586	-0.97	6.57E-03	1.61E-01	Intron (NM_013175/25719, intron 2 of 5)
Snapc5	691501	chr8	69165995	69166295	38287	-2.18	6.57E-03	1.61E-01	Intron (NM_031643/170851, intron 1 of 10)
Pif1	367645	chr8	71083579	71083879	-41535	-1.71	6.58E-03	1.61E-01	Distal Intergenic
Plcb4	25031	chr3	128786341	128786641	185011	-1.78	6.58E-03	1.61E-01	Intron (NM_024353/25031, intron 3 of 38)
P4ha1	64475	chr20	28929656	28929956	9040	-1.39	6.58E-03	1.61E-01	Intron (NM_172062/64475, intron 1 of 14)
Ddx25	58856	chr8	36738578	36738878	21842	-1.31	6.59E-03	1.61E-01	Distal Intergenic
Otulin	100362554	chr2	80258346	80258646	34540	-1.73	6.60E-03	1.62E-01	Distal Intergenic
Lyst	85419	chr17	90509213	90509513	12578	1.88	6.61E-03	1.62E-01	Exon (NM_053518/85419, exon 2 of 53)

Pif1	367645	chr8	71076262	71076562	-48852	2.07	6.61E-03	1.62E-01	Distal Intergenic
Cenpf	257649	chr13	108169953	108170253	8356	-1.76	6.62E-03	1.62E-01	Intron (NM_001100827/257649, intron 6 of 19)
Tacc2	309025	chr1	201235290	201235590	94237	-1.32	6.62E-03	1.62E-01	Intron (NM_001004415/309025, intron 5 of 21)
Psmb11	290206	chr15	33297409	33297709	29996	-2	6.63E-03	1.62E-01	Intron (NM_001170468/305884, intron 9 of 18)
Pou6f1	116545	chr7	142224582	142224882	-13844	1.02	6.63E-03	1.62E-01	Distal Intergenic
Mirlet7d	100313972	chr17	16419771	16420071	0	1.85	6.63E-03	1.62E-01	Promoter (<=1kb)
Pgrmc1	291948	chrX	123254371	123254671	48502	0.96	6.63E-03	1.62E-01	Distal Intergenic
Carmil1	306941	chr17	43128567	43128867	77606	-1.61	6.64E-03	1.62E-01	Intron (NM_001191692/306941, intron 2 of 37)
Arl8b	500282	chr4	140847034	140847334	9289	-1.2	6.64E-03	1.62E-01	Intron (NM_001024332/500282, intron 1 of 6)
RGD1560028	500157	chr4	96500864	96501164	248230	-1.48	6.64E-03	1.62E-01	Distal Intergenic
Mgll	29254	chr4	120682879	120683179	11390	-2.17	6.64E-03	1.62E-01	Intron (NM_138502/29254, intron 2 of 7)
Cyb561	303601	chr10	94052056	94052356	95200	-1.92	6.65E-03	1.62E-01	Intron (NM_001191653/303599, intron 11 of 25)
Setdb1	689883	chr2	196526662	196526962	165	0.85	6.66E-03	1.62E-01	Promoter (<=1kb)
Wdr18	314617	chr7	12559172	12559472	9648	2.09	6.66E-03	1.62E-01	Intron (NM_001108067/314618, intron 22 of 22)
Slc41a3	641603	chr4	123380848	123381148	113594	1.2	6.67E-03	1.62E-01	Distal Intergenic
Prrc2a	294250	chr20	5142442	5142742	10657	1.78	6.67E-03	1.62E-01	Intron (NM_212462/294250, intron 23 of 29)
Bud31	89819	chr12	11237101	11237401	2965	2.35	6.67E-03	1.62E-01	Promoter (2-3kb)
Mtbp	500870	chr7	95254638	95254938	-54990	2.2	6.67E-03	1.62E-01	Intron (NM_001130548/314981, intron 44 of 47)
Cxadr	89843	chr11	16733850	16734150	-92249	1.95	6.67E-03	1.62E-01	Distal Intergenic
Yaf2	690262	chr7	134560642	134560942	0	1.71	6.68E-03	1.62E-01	Promoter (<=1kb)
Runx2	367218	chr9	18783887	18784187	140317	-1.16	6.68E-03	1.62E-01	Distal Intergenic
Ppp4r3a	314388	chr6	125075600	125075900	7092	-1.58	6.70E-03	1.62E-01	Intron (NM_001108050/314388, intron 1 of 19)
Ccdc42	303234	chr10	55184039	55184339	14727	1.78	6.71E-03	1.62E-01	Distal Intergenic
Mthfd2	680308	chr4	115000558	115000858	15107	2.06	6.71E-03	1.62E-01	Intron (NM_212512/297386, intron 26 of 26)
Apoc3	24207	chr8	50531598	50531898	-100	2.28	6.71E-03	1.62E-01	Promoter (<=1kb)
Klhl28	299103	chr6	86720039	86720339	-6669	-1.91	6.72E-03	1.62E-01	Distal Intergenic
Ros1	25346	chr20	33359167	33359467	-35800	0.9	6.72E-03	1.62E-01	Distal Intergenic
Kif13b	305967	chr15	48043857	48044157	12783	-1.83	6.72E-03	1.62E-01	Intron (NM_213626/305967, intron 1 of 37)
Tmem160	292654	chr1	78416270	78416570	-1149	2.21	6.73E-03	1.62E-01	Promoter (1-2kb)
Slc45a1	246258	chr5	167614804	167615104	81227	0.88	6.73E-03	1.62E-01	Intron (NM_053885/116665, intron 11 of 22)
Serp2	498546	chr15	58709771	58710071	1801	-1.86	6.73E-03	1.62E-01	Promoter (1-2kb)
Cmklr1	60669	chr12	48773449	48773749	-15512	0.92	6.73E-03	1.62E-01	Distal Intergenic
Rnd3	295588	chr3	36642263	36642563	18164	2.28	6.74E-03	1.62E-01	Downstream (<1kb)
Eps8l3	295361	chr2	210615684	210615984	-52419	-1.34	6.74E-03	1.62E-01	Distal Intergenic
Zfp709	266773	chr16	19557694	19557994	85277	2.04	6.74E-03	1.62E-01	Distal Intergenic
Trim16	303214	chr10	49236065	49236365	4104	-1.77	6.75E-03	1.62E-01	Intron (NM_001135033/303214, intron 1 of 5)
Tsn	60381	chr13	34251392	34251692	0	0.74	6.76E-03	1.63E-01	Promoter (<=1kb)
Ptp4a2	85237	chr5	148035043	148035343	-15783	2.2	6.78E-03	1.63E-01	Distal Intergenic
Canx	29144	chr10	35825957	35826257	7604	-1.77	6.78E-03	1.63E-01	Intron (NM_172008/29144, intron 1 of 14)
Cpa6	312913	chr5	8365388	8365688	-93972	-2.14	6.79E-03	1.63E-01	Distal Intergenic
Akap5	171026	chr6	99340488	99340788	-15721	-1.57	6.79E-03	1.63E-01	Intron (NM_022508/64300, intron 23 of 27)
Gmds	291095	chr17	33496491	33496791	87769	1.88	6.80E-03	1.63E-01	Intron (NM_001039606/291095, intron 1 of 10)
Aicda	399679	chr4	155369507	155369807	9598	-1.77	6.80E-03	1.63E-01	3' UTR
Echdc3	684538	chr17	75981128	75981428	-20876	-1.82	6.80E-03	1.63E-01	Distal Intergenic
Anpep	81641	chr1	141612651	141612951	-32831	2.3	6.81E-03	1.63E-01	Downstream (<1kb)
Trpc7	282822	chr17	8182339	8182639	47088	2.25	6.81E-03	1.63E-01	Intron (NM_001191691/282822, intron 3 of 11)
Stard13	498130	chr12	1195298	1195598	0	0.66	6.82E-03	1.63E-01	Promoter (<=1kb)
Sf3b1	84486	chr9	61634058	61634358	152	0.6	6.82E-03	1.63E-01	Promoter (<=1kb)
Meis2	311311	chr3	107773232	107773532	-12643	2.26	6.82E-03	1.63E-01	Distal Intergenic
Fndc3b	294925	chr2	113217921	113218221	127330	-1.76	6.83E-03	1.63E-01	Intron (NM_001191704/294925, intron 5 of 24)
Mir875	100314134	chr7	74317929	74318229	105076	2.45	6.83E-03	1.63E-01	Intron (NM_001134886/315036, intron 19 of 61)
Rhno1	297627	chr4	161671539	161671839	13155	-1.78	6.83E-03	1.63E-01	Distal Intergenic
Prkar2b	24679	chr6	51352468	51352768	3615	-1.76	6.84E-03	1.63E-01	Intron (NM_001030020/24679, intron 1 of 10)
Agfg1	363266	chr9	88577954	88578254	-29254	-1.7	6.84E-03	1.63E-01	Distal Intergenic
Stard10	293150	chr1	166441148	166441448	8039	1.6	6.84E-03	1.63E-01	Intron (NM_001013069/293150, intron 2 of 6)
Zfp395	305972	chr15	48750667	48750967	-38353	-1.57	6.84E-03	1.63E-01	Distal Intergenic
Pfdn1	361310	chr18	29243832	29244132	46315	-1.51	6.85E-03	1.63E-01	Intron (NM_001108427/361310, intron 3 of 3)
Agpat3	294324	chr20	11060667	11060967	-53197	1.89	6.86E-03	1.63E-01	Distal Intergenic
Kalrn	84009	chr11	69435492	69435792	-48501	-1.68	6.87E-03	1.63E-01	Distal Intergenic
Pcdha3	116780	chr18	29967914	29968214	1669	-1.75	6.87E-03	1.63E-01	Promoter (1-2kb)
Tnfrsf26	361685	chr1	216864120	216864420	-35539	-1.02	6.87E-03	1.63E-01	Downstream (1-2kb)
Ms4a1	309217	chr1	227358392	227358692	77851	-1.68	6.88E-03	1.64E-01	Distal Intergenic
Hsd17b4	79244	chr18	44780786	44781086	-29376	-1.7	6.89E-03	1.64E-01	Distal Intergenic
Elf1	85424	chr15	61837905	61838205	11199	-1.97	6.89E-03	1.64E-01	Intron (NM_053520/85424, intron 1 of 7)
Serpinf1	287526	chr10	62253519	62253819	326	0.78	6.89E-03	1.64E-01	Promoter (<=1kb)
Zc3hc1	296957	chr4	57666125	57666425	40188	-1.52	6.89E-03	1.64E-01	Distal Intergenic
Ddr2	685781	chr13	88372062	88372362	-10380	-1.75	6.90E-03	1.64E-01	Distal Intergenic
Pak4	292756	chr1	85159041	85159341	-3121	-1.61	6.92E-03	1.64E-01	Distal Intergenic
Hmga2	84017	chr7	65442812	65443112	-167404	-1.82	6.92E-03	1.64E-01	Distal Intergenic
Serpine1	24617	chr12	22646381	22646681	5277	-1.95	6.92E-03	1.64E-01	Exon (NM_012620/24617, exon 5 of 9)
Chpt1	362866	chr7	29070467	29070767	161	1.06	6.93E-03	1.64E-01	Promoter (<=1kb)
Hand2	64637	chr16	36697770	36698070	-324221	-1.93	6.93E-03	1.64E-01	Distal Intergenic
Rps21	81775	chr3	175644476	175644776	15300	1.01	6.93E-03	1.64E-01	Distal Intergenic
Ajuba	85265	chr15	33209258	33209558	8898	1.56	6.95E-03	1.64E-01	3' UTR
Clec2d2	362445	chr4	162438005	162438305	906	-1.89	6.95E-03	1.64E-01	Promoter (<=1kb)
Wnt4	84426	chr5	155648201	155648501	-737	1.44	6.95E-03	1.64E-01	Promoter (<=1kb)
Fech	361338	chr18	59968620	59968920	6272	-1.44	6.95E-03	1.64E-01	Intron (NM_001108434/361338, intron 2 of 10)
Coq10b	301416	chr9	61661245	61661545	5282	-1.72	6.95E-03	1.64E-01	Intron (NM_001009671/301416, intron 1 of 4)
Ddx59	289402	chr13	53328490	53328790	28618	-1.54	6.96E-03	1.64E-01	Distal Intergenic
Slc14a2	54302	chr18	74894380	74894680	46709	1.26	6.96E-03	1.64E-01	Intron (NM_019347/54302, intron 2 of 21)
Mtfr1	311403	chr2	104009486	104009786	-11169	-1.91	6.96E-03	1.64E-01	Distal Intergenic
Ppwd1	294711	chr2	34522094	34522394	-209000	2.12	6.96E-03	1.64E-01	Distal Intergenic
LOC688925	688925	chr10	70420376	70420676	4259	-1.6	6.97E-03	1.64E-01	5' UTR
Slc2a10	366251	chr3	162182171	162182471	15	1.24	6.97E-03	1.64E-01	Promoter (<=1kb)
Armc3	100361506	chr17	85916875	85917175	2288	-1.41	6.97E-03	1.64E-01	Promoter (2-3kb)
Lsm11	501688	chr10	30937851	30938151	103475	-1.88	6.97E-03	1.64E-01	Intron (NM_001002022/360515, intron 1 of 9)
Csnk1g3	64823	chr18	48827965	48828265	-24906	1.04	6.97E-03	1.64E-01	Distal Intergenic
PCOLCE2	684050	chr8	103404902	103405202	-54017	2.33	6.97E-03	1.64E-01	Distal Intergenic
Nr2c2	50659	chr4	123978495	123978795	-26492	1.83	6.97E-03	1.64E-01	Distal Intergenic
Wwtr1	295062	chr2	147653430	147653730	39303	-1.7	6.97E-03	1.64E-01	Intron (NM_001024869/295062, intron 2 of 6)
Clstn1	313717	chr5	166518041	166518341	-14894	-1.74	6.98E-03	1.64E-01	Distal Intergenic
Map4k4	301363	chr9	46709619	46709919	51697	-1.51	6.98E-03	1.64E-01	Intron (NM_001106904/301363, intron 1 of 28)
Bbx	303970	chr11	52838455	52838755	-242270	2.3	6.99E-03	1.64E-01	Distal Intergenic
Peli3	309157	chr1	220198542	220198842	5190	2.01	6.99E-03	1.64E-01	Intron (NM_001127542/309157, intron 3 of 6)

Alas2	25748	chrX	23246683	23246983	-59327	2.73	6.99E-03	1.64E-01	Distal Intergenic
Mir193b	104797223	chr14	30926856	30927156	-595725	-2.02	7.01E-03	1.64E-01	Distal Intergenic
Fbxl14	312675	chr4	151986947	151987247	54	1.2	7.02E-03	1.65E-01	Promoter (<=1kb)
Mir875	100314134	chr7	74420718	74421018	2287	-1.47	7.02E-03	1.65E-01	Promoter (2-3kb)
Dkk3	171548	chr1	177030209	177030509	-47164	-1.69	7.03E-03	1.65E-01	Distal Intergenic
LOC100911498	100911498	chrX	74336309	74336609	5543	-1.83	7.04E-03	1.65E-01	Intron (NM_001047974/680227, intron 9 of 9)
Sbno2	314619	chr7	12484538	12484838	12714	-1.8	7.04E-03	1.65E-01	Intron (NM_001108068/314619, intron 1 of 32)
Samd12	362910	chr7	93233414	93233714	53043	-1.78	7.06E-03	1.65E-01	Intron (NM_001130562/362910, intron 2 of 3)
Mob1a	297387	chr4	115025033	115025333	106	0.81	7.07E-03	1.65E-01	Promoter (<=1kb)
Jade1	310352	chr2	128462828	128463128	1604	1.25	7.07E-03	1.65E-01	Promoter (1-2kb)
Pdp1	54705	chr5	25594009	25594309	-9731	2.1	7.07E-03	1.65E-01	Distal Intergenic
Tpm1	24851	chr8	72828369	72828669	7490	2.1	7.08E-03	1.65E-01	Exon (NM_001034073/24851, exon 2 of 8)
Pigx	288041	chr11	71938400	71938700	12	0.77	7.09E-03	1.65E-01	Promoter (<=1kb)
Bhlhe23	499952	chr3	176274925	176275225	6986	-1.59	7.09E-03	1.65E-01	Distal Intergenic
Lamb2	25473	chr8	117266924	117267224	-1150	0.89	7.09E-03	1.65E-01	Promoter (1-2kb)
RGD1311703	293160	chr1	185563323	185563623	5567	-1.66	7.11E-03	1.65E-01	Exon (NM_001013898/293160, exon 2 of 5)
Srebf2	300095	chr7	123394707	123395007	13625	-1.78	7.11E-03	1.65E-01	Intron (NM_001033694/300095, intron 1 of 19)
Plk2	83722	chr2	41916366	41916666	5223	-1.87	7.11E-03	1.65E-01	3' UTR
Anks1a	309639	chr20	7520472	7520772	35922	-1.87	7.11E-03	1.65E-01	Intron (NM_001107613/309639, intron 1 of 23)
Fbxo7	366854	chr7	23843216	23843516	0	0.77	7.11E-03	1.65E-01	Promoter (<=1kb)
Zmpste24	313564	chr5	140073188	140073488	-57647	-1.71	7.12E-03	1.65E-01	Distal Intergenic
Rbpj	679028	chr14	59715324	59715624	19826	-1.81	7.12E-03	1.65E-01	Intron (NM_001106631/679028, intron 2 of 11)
Maml3	310405	chr2	141101132	141101432	175454	-1.44	7.12E-03	1.65E-01	Intron (NM_001107675/310405, intron 1 of 5)
C1ql1	363686	chr10	91049178	91049478	-2001	1.81	7.12E-03	1.65E-01	Promoter (2-3kb)
Ahcyl1	362013	chr2	210451365	210451665	22096	-1.66	7.14E-03	1.66E-01	Exon (NM_001108561/362013, exon 3 of 16)
Gucy2g	245708	chr1	276227033	276227333	1241	1.98	7.14E-03	1.66E-01	Promoter (1-2kb)
Adamts7	315879	chr8	97591014	97591314	55237	-1.97	7.15E-03	1.66E-01	Intron (NM_001108175/315880, intron 10 of 13)
Txndc2	316777	chr9	113384626	113384926	-26068	-1.78	7.15E-03	1.66E-01	Intron (NM_145094/246324, intron 6 of 6)
Plvap	56765	chr16	19927392	19927692	-8724	-1.41	7.15E-03	1.66E-01	Distal Intergenic
Fkbp3	299104	chr6	86860548	86860848	-38433	-1.77	7.16E-03	1.66E-01	Distal Intergenic
Ccdc92	100036765	chr12	37199976	37200276	-11040	-1.77	7.16E-03	1.66E-01	Distal Intergenic
Bcl3	680611	chr1	80734747	80735047	9946	-1.57	7.18E-03	1.66E-01	Exon (NM_001109422/680611, exon 3 of 9)
Sh3bgrl2	501026	chr8	91084281	91084581	14356	-2.02	7.18E-03	1.66E-01	Intron (NM_001137647/501026, intron 1 of 3)
Smad2	29357	chr18	72412324	72412624	-137595	-1.73	7.19E-03	1.66E-01	Intron (NM_001127375/679155, intron 3 of 5)
RGD1562024	498699	chr17	8611956	8612256	7481	1.96	7.19E-03	1.66E-01	Downstream (<1kb)
Smim20	501923	chr14	59966180	59966480	103390	-1.83	7.20E-03	1.66E-01	Distal Intergenic
Sox4	364712	chr17	37498234	37498534	-116488	-1.79	7.22E-03	1.67E-01	Distal Intergenic
Atg7	312647	chr4	146602467	146602767	4051	-1.7	7.22E-03	1.67E-01	Intron (NM_001012097/312647, intron 3 of 17)
RSA-14-44	297173	chr4	96703119	96703419	-128461	-1.88	7.22E-03	1.67E-01	Distal Intergenic
Hoxb13	303480	chr10	84037717	84038017	5762	1.15	7.23E-03	1.67E-01	Distal Intergenic
Fosl2	25446	chr6	25734724	25735024	-117729	1.98	7.23E-03	1.67E-01	Intron (NM_199270/362704, intron 10 of 11)
Prickle2	312563	chr4	124358846	124359146	224899	0.89	7.23E-03	1.67E-01	Intron (NM_001107876/312563, intron 1 of 7)
Cox17	89786	chr11	64985597	64985897	-17160	-1.67	7.24E-03	1.67E-01	Distal Intergenic
Naa20	362228	chr3	140101582	140101882	-4844	-1.87	7.24E-03	1.67E-01	Distal Intergenic
Kif3c	85248	chr6	27847284	27847584	78341	-1.87	7.24E-03	1.67E-01	Distal Intergenic
Casc4	362204	chr3	113716301	113716601	-102048	-1.97	7.24E-03	1.67E-01	Distal Intergenic
Fcho2	309129	chr2	29028741	29029041	230	1.39	7.24E-03	1.67E-01	Promoter (<=1kb)
Pip5k1a	365865	chr2	196261221	196261521	9305	-1.43	7.25E-03	1.67E-01	Intron (NM_001042621/365865, intron 1 of 14)
Dhx8	287727	chr10	89614137	89614437	-31758	2.34	7.26E-03	1.67E-01	Distal Intergenic
Kcnn3	54263	chr2	188844835	188845135	725	-1.84	7.27E-03	1.67E-01	Promoter (<=1kb)
Pacsin1	29704	chr20	7246909	7247209	5661	-1.69	7.28E-03	1.67E-01	Intron (NM_017294/29704, intron 1 of 9)
Tmem51	500578	chr5	160624610	160624910	-4276	-1.84	7.28E-03	1.67E-01	Distal Intergenic
Dpy30	286897	chr6	22320714	22321014	18645	0.92	7.29E-03	1.67E-01	Distal Intergenic
Ltbp2	59106	chr6	108559552	108559852	36594	-1.91	7.29E-03	1.67E-01	Intron (NM_021586/59106, intron 3 of 34)
Map4k4	301363	chr9	46623631	46623931	-33991	-1.81	7.30E-03	1.67E-01	Distal Intergenic
Atg10	688555	chr2	20088275	20088575	64309	0.78	7.30E-03	1.67E-01	Intron (NM_001109505/688555, intron 2 of 7)
Ppm1d	287585	chr10	72973851	72974151	64301	-1.88	7.31E-03	1.68E-01	Intron (NM_001173430/363662, intron 13 of 17)
Snn	29140	chr10	4684046	4684346	-39476	2.12	7.32E-03	1.68E-01	Distal Intergenic
Samd12	362910	chr7	93234785	93235085	51672	1.67	7.33E-03	1.68E-01	Intron (NM_001130562/362910, intron 2 of 3)
Selenot	365802	chr2	148769785	148770085	4385	-1.53	7.33E-03	1.68E-01	Intron (NM_001014253/365802, intron 1 of 5)
Map4k4	301363	chr9	46687758	46688058	29836	-1.41	7.33E-03	1.68E-01	Intron (NM_001106904/301363, intron 1 of 28)
Ddo	685325	chr20	48812643	48812943	-53747	1.95	7.33E-03	1.68E-01	Distal Intergenic
Cat	24248	chr3	93398594	93398894	13164	1.96	7.33E-03	1.68E-01	Intron (NM_012520/24248, intron 4 of 12)
Opn4	192223	chr16	11029369	11029669	-76820	0.97	7.36E-03	1.68E-01	Distal Intergenic
Akr7a2	171445	chr5	157759312	157759612	0	0.63	7.36E-03	1.68E-01	Promoter (<=1kb)
St3gal4	363040	chr8	36304225	36304525	10286	-1.67	7.37E-03	1.68E-01	Intron (NM_203337/363040, intron 1 of 10)
Cilp	315761	chr8	70762740	70763040	1818	1.47	7.38E-03	1.68E-01	Promoter (1-2kb)
Igsf11	303926	chr11	64469290	64469590	-48042	-1.7	7.38E-03	1.68E-01	Distal Intergenic
Snx13	362731	chr6	54679653	54679953	191578	-1.85	7.38E-03	1.68E-01	Distal Intergenic
Crabp2	29563	chr2	187314911	187315211	-7205	-1.36	7.40E-03	1.68E-01	Distal Intergenic
Reep3	294375	chr20	23163889	23164189	250240	-1.87	7.40E-03	1.68E-01	Distal Intergenic
Atp13a4	288026	chr11	74830482	74830782	-3475	-1.64	7.40E-03	1.68E-01	Distal Intergenic
RGD1561870	502353	chr1	163949289	163949589	-134925	-1.76	7.41E-03	1.68E-01	Intron (NM_001107536/308846, intron 9 of 13)
Akap2	298024	chr5	74948912	74949212	6821	-1.69	7.41E-03	1.68E-01	Intron (NM_001011974/298024, intron 1 of 3)
Usp6nl	291309	chr17	75842617	75842917	43606	-1.33	7.41E-03	1.69E-01	Intron (NM_001106120/291309, intron 2 of 14)
Gtf2e2	306516	chr16	62077151	62077451	86684	-1.25	7.42E-03	1.69E-01	Intron (NM_001271244/498642, intron 5 of 6)
Evi2a	685433	chr10	66873423	66873723	225	-1.16	7.43E-03	1.69E-01	Promoter (<=1kb)
Sh3bp1	300067	chr7	120125604	120125904	0	0.76	7.43E-03	1.69E-01	Promoter (<=1kb)
Chd2	308738	chr1	134799314	134799614	71554	-1.19	7.43E-03	1.69E-01	Exon (NM_001107523/308738, exon 26 of 39)
Sinhcaf	686611	chr4	183423241	183423541	2898	2.26	7.44E-03	1.69E-01	Promoter (2-3kb)
Atp6v1g3	289407	chr13	55335647	55335947	61448	-1.77	7.44E-03	1.69E-01	Distal Intergenic
Mir222	100314059	chrX	3674174	3674474	-9445	-1.22	7.45E-03	1.69E-01	Distal Intergenic
Rasl12	315762	chr8	70916146	70916446	193	0.8	7.45E-03	1.69E-01	Promoter (<=1kb)
Gab1	361388	chr19	30839631	30839931	45341	-1.8	7.45E-03	1.69E-01	Intron (NM_001108444/361388, intron 1 of 9)
Disp1	289338	chr13	101566777	101567077	30493	1.42	7.46E-03	1.69E-01	Intron (NM_001105983/289338, intron 2 of 9)
Acap2	619382	chr11	73115771	73116071	36789	-1.84	7.47E-03	1.69E-01	Intron (NM_001034006/619382, intron 1 of 22)
Rgs3	54293	chr5	78595310	78595610	111417	-1.31	7.47E-03	1.69E-01	Distal Intergenic
Kpnb1	24917	chr10	85109483	85109783	14858	1.97	7.47E-03	1.69E-01	Exon (NM_017063/24917, exon 11 of 23)
Mtmr11	689613	chr2	198303111	198303411	0	0.69	7.47E-03	1.69E-01	Promoter (<=1kb)
Rraga	117044	chr5	104650505	104650805	-290262	1.97	7.48E-03	1.69E-01	Distal Intergenic
Cpa6	312913	chr5	8215475	8215775	-243885	-2	7.48E-03	1.69E-01	Distal Intergenic
Maml3	310405	chr2	141092609	141092909	183977	2.09	7.48E-03	1.69E-01	Intron (NM_001107675/310405, intron 1 of 5)
Fbxo28	305105	chr13	100817245	100817545	-76517	0.93	7.50E-03	1.69E-01	Distal Intergenic
Lnc215	104845260	chr8	45706809	45707109	15613	-1.59	7.50E-03	1.69E-01	Intron (NR_126581/104845260, intron 2 of 3)

Ssbp3	84354	chr5	126536122	126536422	24772	2.25	7.50E-03	1.69E-01	Intron (NM_053358/84354, intron 4 of 16)
Mkrn2	297525	chr4	147546588	147546888	32468	-1.9	7.52E-03	1.70E-01	Exon (NM_012639/24703, exon 5 of 17)
Alpl	25586	chr5	156094467	156094767	46746	2.1	7.52E-03	1.70E-01	Exon (NM_013059/25586, exon 7 of 12)
Fblim1	362650	chr5	160155485	160155785	412	1.4	7.53E-03	1.70E-01	Promoter (<=1kb)
Aco2	79250	chr7	123116054	123116354	13561	1.79	7.54E-03	1.70E-01	Intron (NM_024398/79250, intron 1 of 17)
Utrn	25600	chr1	6932610	6932910	37130	1.16	7.54E-03	1.70E-01	Intron (NM_013070/25600, intron 2 of 74)
Arl6ip6	499798	chr3	38745965	38746265	3893	-1.61	7.55E-03	1.70E-01	Intron (NM_001024310/499798, intron 2 of 3)
Vta1	292640	chr1	8739752	8740052	138084	-1.94	7.55E-03	1.70E-01	Distal Intergenic
Pwwp2b	361671	chr1	211593124	211593424	11047	-1.33	7.56E-03	1.70E-01	Intron (NM_001108507/361671, intron 2 of 2)
Tomm20	266601	chr19	59431655	59431955	441456	2.12	7.56E-03	1.70E-01	Distal Intergenic
Kcnn3	54263	chr2	188846407	188846707	2297	-1.73	7.57E-03	1.70E-01	Promoter (2-3kb)
Enah	360891	chr13	100327003	100327303	78036	-1.71	7.57E-03	1.70E-01	Intron (NM_001012150/360891, intron 3 of 12)
Rps13	161477	chr1	185304036	185304336	-24566	-1.42	7.57E-03	1.70E-01	Exon (NM_001108500/361632, exon 20 of 34)
Ube2e3	295686	chr3	65815457	65815757	381	1.49	7.57E-03	1.70E-01	Promoter (<=1kb)
Ppp1r11	294207	chr20	2093039	2093339	-1592	2.32	7.58E-03	1.70E-01	Promoter (1-2kb)
Foxc2	171356	chr19	53099525	53099825	55146	1.61	7.58E-03	1.70E-01	Distal Intergenic
Fam117b	363236	chr9	66716470	66716770	-199	1.44	7.58E-03	1.70E-01	Promoter (<=1kb)
Zfp786	100158223	chr4	77512685	77512985	-2481	-1.82	7.59E-03	1.70E-01	Promoter (2-3kb)
Ccnl1	114121	chr2	157763216	157763516	-3378	0.91	7.59E-03	1.70E-01	Distal Intergenic
Atp2b1	29598	chr7	41085821	41086121	-28576	-1.92	7.60E-03	1.70E-01	Distal Intergenic
Ldha	24533	chr1	102906191	102906491	5903	-2.09	7.60E-03	1.70E-01	Exon (NM_017025/24533, exon 6 of 8)
Pdgfa	25266	chr12	17751250	17751550	17109	-2	7.60E-03	1.70E-01	Intron (NM_012801/25266, intron 6 of 7)
Trps1	299897	chr7	90084916	90085216	233005	1.77	7.62E-03	1.70E-01	Distal Intergenic
Csrp2	29317	chr7	53630499	53630799	0	0.75	7.62E-03	1.70E-01	Promoter (<=1kb)
Tfap2a	306862	chr17	25125778	25126078	470876	-1.89	7.63E-03	1.70E-01	Distal Intergenic
Pax8	81819	chr3	1684540	1684840	-99615	-1.19	7.63E-03	1.70E-01	Distal Intergenic
Gpr12	80840	chr12	10367332	10367632	111913	1	7.63E-03	1.70E-01	Distal Intergenic
Traf1	687813	chr3	14019997	14020297	-793	-1.65	7.64E-03	1.70E-01	Promoter (<=1kb)
Kank4	313385	chr5	117440156	117440456	0	1.31	7.66E-03	1.71E-01	Promoter (<=1kb)
Chd2	308738	chr1	134854342	134854642	16526	2.04	7.66E-03	1.71E-01	Intron (NM_001107523/308738, intron 2 of 38)
Ctsh	25425	chr8	97434343	97434643	-4426	-1.75	7.66E-03	1.71E-01	Distal Intergenic
Tnfsf12	360548	chr10	56299654	56299954	123	0.82	7.67E-03	1.71E-01	Promoter (<=1kb)
Tnfaip6	84397	chr3	37601789	37602089	56546	-1.83	7.67E-03	1.71E-01	Distal Intergenic
Spryd7	290303	chr15	41883398	41883698	6986	-1.67	7.69E-03	1.71E-01	Intron (NM_001009635/290303, intron 2 of 4)
Plch1	310463	chr2	154397461	154397761	-53790	2.04	7.70E-03	1.71E-01	Distal Intergenic
Rad9b	363924	chr12	39729168	39729468	29987	-1.51	7.70E-03	1.71E-01	3' UTR
Cdh13	192248	chr19	50849470	50849770	677	-1.94	7.70E-03	1.71E-01	Promoter (<=1kb)
Evi2b	100910940	chr10	66864035	66864335	-5437	-1.77	7.71E-03	1.71E-01	Intron (NM_012609/24592, intron 39 of 60)
Mitf	25094	chr4	130309396	130309696	136540	-1.62	7.71E-03	1.71E-01	Intron (NM_001191089/25094, intron 1 of 9)
Cacul1	365493	chr1	281866245	281866545	7966	-1.65	7.71E-03	1.71E-01	Intron (NM_001014248/365493, intron 1 of 7)
Csad	60356	chr7	143810241	143810541	-16271	1.73	7.72E-03	1.71E-01	Distal Intergenic
Uros	309070	chr1	205777059	205777359	0	0.77	7.73E-03	1.71E-01	Promoter (<=1kb)
Vamp1	25624	chr4	157723376	157723676	-3265	-1.85	7.73E-03	1.71E-01	Distal Intergenic
Cab39	301574	chr9	92791294	92791594	-42763	1.5	7.75E-03	1.72E-01	Distal Intergenic
Spen	690911	chr5	160109484	160109784	-38569	1.76	7.75E-03	1.72E-01	Distal Intergenic
Frs3	316213	chr9	15306288	15306588	-1671	1.35	7.76E-03	1.72E-01	Promoter (1-2kb)
Nek7	360850	chr13	55603875	55604175	-42149	-2.01	7.76E-03	1.72E-01	Distal Intergenic
Tyms	29261	chr9	121915144	121915444	16120	1.93	7.77E-03	1.72E-01	Distal Intergenic
Mrps23	360594	chr10	75522220	75522520	-6489	2.21	7.78E-03	1.72E-01	Distal Intergenic
Zfat	362925	chr7	109232616	109232916	-27247	2.08	7.79E-03	1.72E-01	Distal Intergenic
Kdm4d	689582	chr8	12912650	12912950	80701	1.24	7.79E-03	1.72E-01	Distal Intergenic
Slc26a7	297910	chr5	28130338	28130638	495	1.66	7.80E-03	1.72E-01	Promoter (<=1kb)
Aff1	305152	chr14	7269588	7269888	55757	-1.64	7.80E-03	1.72E-01	Intron (NM_001107206/305152, intron 3 of 19)
RGD1563941	500993	chr8	52829002	52829302	0	0.69	7.80E-03	1.72E-01	Promoter (<=1kb)
Kcna6	64358	chr4	159313610	159313910	-26417	-1.28	7.81E-03	1.72E-01	Distal Intergenic
Col1a2	84352	chr4	31650326	31650626	116101	0.85	7.81E-03	1.72E-01	Distal Intergenic
Fosl2	25446	chr6	25716310	25716610	-99315	-1.66	7.82E-03	1.72E-01	Intron (NM_199270/362704, intron 10 of 11)
Dimt1	294718	chr2	38103845	38104145	-16441	-1.77	7.84E-03	1.73E-01	Distal Intergenic
Irak3	314870	chr7	64981898	64982198	26	0.73	7.84E-03	1.73E-01	Promoter (<=1kb)
Nupl2	499974	chr4	7485693	7485993	43113	-1.1	7.85E-03	1.73E-01	Distal Intergenic
Thop1	64517	chr7	11513811	11514111	-322	-1.03	7.86E-03	1.73E-01	Promoter (<=1kb)
Cachd1	298267	chr5	119656607	119656907	59881	-1.78	7.86E-03	1.73E-01	Intron (NM_001191758/298267, intron 1 of 25)
Zfp608	307296	chr18	50374600	50374900	-437078	-1.35	7.86E-03	1.73E-01	Distal Intergenic
Rgcc	117183	chr15	61589792	61590092	-25097	2.91	7.86E-03	1.73E-01	Distal Intergenic
Yy1	24919	chr6	132702087	132702387	-194	1.27	7.87E-03	1.73E-01	Promoter (<=1kb)
Slc29a3	353307	chr20	30326995	30327295	48	0.63	7.87E-03	1.73E-01	Promoter (<=1kb)
Pde7a	81744	chr2	104134028	104134328	0	1.48	7.89E-03	1.73E-01	Promoter (<=1kb)
Col6a2	361821	chr20	12658629	12658929	-114591	0.76	7.90E-03	1.73E-01	Distal Intergenic
Epb41l5	304733	chr13	35675487	35675787	-2038	2.44	7.91E-03	1.74E-01	Promoter (2-3kb)
Cenpf	257649	chr13	108165872	108166172	12437	-1.57	7.92E-03	1.74E-01	Intron (NM_001100827/257649, intron 7 of 19)
Nlrp3	287362	chr10	45901051	45901351	8033	-1.86	7.92E-03	1.74E-01	Intron (NM_001191642/287362, intron 3 of 8)
Ppp1r3e	691447	chr15	33536436	33536736	410	1.52	7.92E-03	1.74E-01	Promoter (<=1kb)
Smim15	100359861	chr2	39145105	39145405	-169309	1.24	7.92E-03	1.74E-01	Distal Intergenic
Vav1	25156	chr9	9674788	9675088	79	1.79	7.93E-03	1.74E-01	Promoter (<=1kb)
Aldh3a2	65183	chr10	47572114	47572414	-26077	-2.09	7.93E-03	1.74E-01	Distal Intergenic
Cxcr3	84475	chrX	71788246	71788546	-171249	2.34	7.97E-03	1.74E-01	Distal Intergenic
Zfp438	307024	chr17	55151678	55151978	143413	1.71	7.98E-03	1.74E-01	Distal Intergenic
Lrrc8a	311846	chr3	8807550	8807850	4698	-1.67	7.98E-03	1.74E-01	Intron (NM_001024782/311846, intron 2 of 3)
Map3k1	116667	chr2	43635725	43636025	-242521	-1.85	7.98E-03	1.74E-01	Distal Intergenic
Cenpf	257649	chr13	108167066	108167366	11243	-1.46	7.99E-03	1.75E-01	Intron (NM_001100827/257649, intron 7 of 19)
Sspn	500364	chr4	180266486	180266786	-24603	-1.65	8.00E-03	1.75E-01	Distal Intergenic
Tuba1a	64158	chr7	140663080	140663380	-22127	-1.8	8.00E-03	1.75E-01	Distal Intergenic
Bicd1	362466	chr4	184129159	184129459	110072	-1.85	8.01E-03	1.75E-01	Intron (NM_001108653/362466, intron 7 of 8)
Thoc3	290519	chr17	10677043	10677343	100	0.68	8.01E-03	1.75E-01	Promoter (<=1kb)
Igfbp5	25285	chr9	80166903	80167203	-90	2.13	8.02E-03	1.75E-01	Promoter (<=1kb)
Arid1a	297867	chr5	151981491	151981791	-33772	0.83	8.03E-03	1.75E-01	Distal Intergenic
Prorsd1	289864	chr14	113800662	113800962	139368	2.17	8.04E-03	1.75E-01	Distal Intergenic
Rybp	312603	chr4	132734792	132735092	5731	-1.74	8.05E-03	1.75E-01	Intron (NM_001107879/312603, intron 1 of 3)
Kank2	100361376	chr8	22826437	22826737	-5214	1.46	8.05E-03	1.75E-01	Intron (NM_001108997/367039, intron 41 of 47)
Dclk1	83825	chr2	144799169	144799469	-61976	-1.82	8.06E-03	1.75E-01	Distal Intergenic
Mtx1	295241	chr2	188527809	188528109	18	0.84	8.06E-03	1.75E-01	Promoter (<=1kb)
Extl3	56819	chr15	48449142	48449442	-3550	-1.35	8.07E-03	1.75E-01	Distal Intergenic
Omp	24612	chr1	163090642	163090942	6767	1.92	8.07E-03	1.75E-01	Intron (NM_134461/171495, intron 3 of 12)
LOC498122	498122	chr11	86305128	86305428	-1675	2.34	8.07E-03	1.75E-01	Promoter (1-2kb)

Lrrc32	293135	chr1	163499326	163499626	53758	-1.82	8.07E-03	1.75E-01	Distal Intergenic
Pik3r1	25513	chr2	31804502	31804802	11583	-1.71	8.08E-03	1.75E-01	Intron (NM_013005/25513, intron 1 of 14)
Nfib	29227	chr5	100130329	100130629	516669	2.28	8.08E-03	1.75E-01	Distal Intergenic
Col11a1	25654	chr2	216772088	216772388	-91040	1.26	8.08E-03	1.75E-01	Distal Intergenic
Serpinc1	304917	chr13	78819125	78819425	13018	1.98	8.08E-03	1.75E-01	Intron (NM_001012027/304917, intron 6 of 6)
Znrf2	362367	chr4	85009460	85009760	110	1.44	8.09E-03	1.75E-01	Promoter (<=1kb)
Zfpm1	691504	chr19	55106087	55106387	11502	1.26	8.09E-03	1.75E-01	Intron (NM_001242627/691504, intron 2 of 10)
Tob2	315159	chr7	123067642	123067942	20298	1.16	8.10E-03	1.75E-01	Distal Intergenic
Zfp474	307310	chr18	47574273	47574573	-2993	2.08	8.10E-03	1.75E-01	Promoter (2-3kb)
Npvf	60570	chr4	80550000	80550300	-154523	-1.52	8.11E-03	1.75E-01	Distal Intergenic
Chic2	83835	chr14	35860617	35860917	176960	-1.86	8.11E-03	1.75E-01	Distal Intergenic
Casp8	64044	chr9	65576799	65577099	-37043	-1.55	8.13E-03	1.76E-01	Intron (NM_001033864/117279, intron 9 of 10)
Mtx1	295241	chr2	188522629	188522929	5198	2.3	8.13E-03	1.76E-01	3' UTR
Vps35	25479	chr19	27468947	27469247	4010	-1.49	8.14E-03	1.76E-01	Intron (NM_001105718/25479, intron 1 of 16)
Sumf1	362409	chr4	140190775	140191075	29407	1.75	8.16E-03	1.76E-01	Intron (NM_001108639/362409, intron 3 of 8)
Rfx3	361746	chr1	246064912	246065212	45330	-1.49	8.16E-03	1.76E-01	Intron (NM_001012172/361746, intron 1 of 15)
Zmynd11	291259	chr17	63904969	63905269	8575	-1.74	8.16E-03	1.76E-01	Intron (NM_203369/291259, intron 1 of 13)
Bcat1	29592	chr4	179325682	179325982	-18587	-1.87	8.18E-03	1.76E-01	Distal Intergenic
Ghitm	290596	chr16	14375915	14376215	6400	-1.55	8.18E-03	1.76E-01	Intron (NM_001005908/290596, intron 5 of 8)
Adamts15	300474	chr8	32022367	32022667	-21989	-1.98	8.18E-03	1.76E-01	Distal Intergenic
Rrm2b	299976	chr7	76882678	76882978	-101861	-1.67	8.19E-03	1.76E-01	Distal Intergenic
Exosc2	366017	chr3	10180859	10181159	-18862	-1.72	8.20E-03	1.77E-01	Distal Intergenic
Osbpl9	298369	chr5	128880814	128881114	-41379	1.66	8.20E-03	1.77E-01	Distal Intergenic
B2m	24223	chr3	114080071	114080371	-6916	-1.6	8.21E-03	1.77E-01	Distal Intergenic
Mafb	54264	chr3	156231073	156231373	109540	-1.73	8.21E-03	1.77E-01	Distal Intergenic
Loxl1	315714	chr8	63067947	63068247	23877	-1.84	8.22E-03	1.77E-01	3' UTR
Grsf1	305256	chr14	21068228	21068528	28238	2.23	8.22E-03	1.77E-01	Downstream (2-3kb)
LOC688459	688459	chr9	23409079	23409379	-11275	-1.6	8.22E-03	1.77E-01	Distal Intergenic
Aptr	104968332	chr4	10754677	10754977	-5386	-1.33	8.22E-03	1.77E-01	Intron (NM_001135872/311987, intron 1 of 7)
RGD1562310	498188	chr12	43581879	43582179	358619	-1.71	8.23E-03	1.77E-01	Distal Intergenic
Kras	24525	chr4	179564392	179564692	-51921	1.81	8.23E-03	1.77E-01	Distal Intergenic
Tnnt2	24837	chr13	52688053	52688353	20084	-1.79	8.24E-03	1.77E-01	Distal Intergenic
MGC95208	304176	chr11	1798290	1798590	20352	-1.78	8.25E-03	1.77E-01	Distal Intergenic
Serpine2	29366	chr9	85778889	85779189	-152795	-1.42	8.25E-03	1.77E-01	Distal Intergenic
Clcc1	170927	chr2	211450528	211450828	44	0.7	8.26E-03	1.77E-01	Promoter (<=1kb)
Nrf1	312195	chr4	57272767	57273067	-63598	0.78	8.26E-03	1.77E-01	Distal Intergenic
Gdf6	252834	chr5	22769824	22770124	-286221	-1.87	8.27E-03	1.77E-01	Distal Intergenic
Brf2	306542	chr16	69132438	69132738	42483	1.01	8.27E-03	1.77E-01	Distal Intergenic
Gtf2ird1	246770	chr12	25264227	25264527	35	0.99	8.27E-03	1.77E-01	Promoter (<=1kb)
Wnt4	84426	chr5	155682686	155682986	33448	-1.17	8.27E-03	1.77E-01	Distal Intergenic
Hspb1	24471	chr12	23851916	23852216	-10865	0.78	8.27E-03	1.77E-01	Distal Intergenic
Pkig	266709	chr3	160088947	160089247	41651	-1.88	8.27E-03	1.77E-01	Intron (NM_153469/266709, intron 1 of 2)
Adck2	312258	chr4	67338946	67339246	-20285	-1.88	8.27E-03	1.77E-01	Distal Intergenic
Elovl4	315851	chr8	91260138	91260438	78187	-1.88	8.27E-03	1.77E-01	Distal Intergenic
Col5a2	85250	chr9	52221064	52221364	17371	-1.88	8.27E-03	1.77E-01	Intron (NM_053488/85250, intron 1 of 55)
Bckdhb	29711	chr8	91838138	91838438	373909	2.18	8.29E-03	1.77E-01	Distal Intergenic
Txn2	79462	chr7	119144646	119144946	13227	2.17	8.29E-03	1.77E-01	3' UTR
Vamp3	29528	chr5	168329211	168329511	-192583	1.47	8.30E-03	1.77E-01	Intron (NM_001195559/362665, intron 8 of 28)
Enpp1	85496	chr1	21756818	21757118	8582	2.43	8.31E-03	1.77E-01	Intron (NM_053535/85496, intron 1 of 24)
Loxl4	309380	chr1	261872968	261873268	-19471	-1.71	8.33E-03	1.77E-01	Distal Intergenic
Dld	298942	chr6	50535200	50535500	83194	-1.48	8.35E-03	1.78E-01	Distal Intergenic
Ptprg	171357	chr15	13620185	13620485	13354	-1.54	8.35E-03	1.78E-01	Intron (NM_134356/171357, intron 1 of 41)
Erbb2	24337	chr10	86370294	86370594	2698	-1.46	8.35E-03	1.78E-01	Promoter (2-3kb)
Egr2	114090	chr20	22458860	22459160	-107	0.9	8.35E-03	1.78E-01	Promoter (<=1kb)
Ctif	364900	chr18	71869151	71869451	-167728	-0.97	8.37E-03	1.78E-01	Distal Intergenic
Shc1	85385	chr2	188752634	188752934	4275	-1.59	8.37E-03	1.78E-01	Intron (NM_053517/85385, intron 8 of 12)
Idnk	498695	chr17	6895616	6895916	-3158	1.29	8.37E-03	1.78E-01	Distal Intergenic
Acpp	56780	chr8	112953469	112953769	-69407	-1.82	8.38E-03	1.78E-01	Distal Intergenic
Snx27	260323	chr2	195799881	195800181	21427	1.86	8.38E-03	1.78E-01	Intron (NM_001110151/260323, intron 1 of 11)
Slc6a6	29464	chr4	123685900	123686200	27119	-1.56	8.38E-03	1.78E-01	Intron (NM_017206/29464, intron 2 of 14)
Epc1	100362678	chr17	57373774	57374074	20901	-1.65	8.40E-03	1.78E-01	Intron (NM_001309462/100362678, intron 1 of 13)
Tead1	361630	chr1	177575640	177575940	79858	-1.71	8.40E-03	1.78E-01	Intron (NM_001198589/361630, intron 2 of 12)
Hspa4	266759	chr10	38618372	38618672	23725	-1.75	8.40E-03	1.78E-01	Intron (NM_153629/266759, intron 7 of 18)
Mir222	100314059	chrX	3630593	3630893	-53026	-1.53	8.42E-03	1.78E-01	Distal Intergenic
Ackr3	84348	chr9	97508459	97508759	152535	2.15	8.43E-03	1.78E-01	Distal Intergenic
Igfbp4	360622	chr10	86950637	86950937	82	0.79	8.44E-03	1.78E-01	Promoter (<=1kb)
Arf6	79121	chr6	91737669	91737969	40560	-1.91	8.44E-03	1.78E-01	Distal Intergenic
Exd2	362759	chr6	103876304	103876604	-141285	-1.91	8.44E-03	1.78E-01	Distal Intergenic
Ets1	24356	chr8	33846545	33846845	30159	-1.91	8.44E-03	1.78E-01	Intron (NM_012555/24356, intron 1 of 7)
Myzap	363091	chr8	78072001	78072301	24002	-1.91	8.44E-03	1.78E-01	Intron (NM_001014211/363091, intron 2 of 12)
Col11a1	25654	chr2	216863975	216864275	547	0.86	8.47E-03	1.79E-01	Promoter (<=1kb)
Chmp4bl1	679886	chr4	66978070	66978370	-81569	1.04	8.47E-03	1.79E-01	Distal Intergenic
Ccnd1	58919	chr1	218189059	218189359	-88787	-1.81	8.47E-03	1.79E-01	Distal Intergenic
Oaf	315594	chr8	47513242	47513542	16147	-1.7	8.47E-03	1.79E-01	Intron (NM_001014090/315594, intron 3 of 3)
Smu1	117541	chr5	57060996	57061296	17	0.78	8.48E-03	1.79E-01	Promoter (<=1kb)
Il10rb	304091	chr11	31566465	31566765	5935	-1.74	8.48E-03	1.79E-01	Intron (NM_001107111/304091, intron 2 of 6)
Nkd1	364952	chr19	19467089	19467389	41698	1.67	8.49E-03	1.79E-01	Intron (NM_001271381/364952, intron 4 of 10)
Marcks	25603	chr20	42494483	42494783	-471357	1.97	8.49E-03	1.79E-01	Distal Intergenic
Trim37	360592	chr10	74442806	74443106	6634	-1.76	8.49E-03	1.79E-01	Intron (NM_001108288/360592, intron 3 of 25)
Mef2a	309957	chr1	128326198	128326498	14765	-1.85	8.49E-03	1.79E-01	Intron (NM_001014035/309957, intron 1 of 10)
Fbxw11	303024	chr10	17580598	17580898	38224	-1.85	8.49E-03	1.79E-01	Intron (NM_001106993/303024, intron 2 of 11)
Rbpj	679028	chr14	59676741	59677041	58409	-1.85	8.49E-03	1.79E-01	Intron (NM_001106631/679028, intron 4 of 11)
Mecom	294924	chr2	117534314	117534614	458990	-1.85	8.49E-03	1.79E-01	Intron (NM_001106423/294924, intron 2 of 16)
Eloa	25562	chr5	154365690	154365990	-2366	-1.85	8.49E-03	1.79E-01	Promoter (2-3kb)
Pphln1	366975	chr7	134639365	134639665	36243	-1.85	8.49E-03	1.79E-01	Intron (NM_001108992/366975, intron 4 of 9)
Antxr1	362393	chr4	119045717	119046017	85185	1.3	8.51E-03	1.79E-01	Intron (NM_001044249/362393, intron 10 of 17)
Specc1l	361828	chr20	14340436	14340736	53136	2.15	8.51E-03	1.79E-01	Intron (NM_001039455/361828, intron 9 of 15)
Atoh8	500200	chr4	100039322	100039622	59895	-1.21	8.51E-03	1.79E-01	Distal Intergenic
Bid	64625	chr4	153472185	153472485	-6938	-1.74	8.52E-03	1.79E-01	Distal Intergenic
Ppp1r1b	360616	chr10	86294057	86294357	-9370	1.09	8.52E-03	1.79E-01	Distal Intergenic
Ppm1a	24666	chr6	94471848	94472148	38187	-1.38	8.53E-03	1.79E-01	Intron (NM_017038/24666, intron 1 of 5)
Trabd	300142	chr7	130058270	130058570	18	1.05	8.53E-03	1.79E-01	Promoter (<=1kb)
Ripk3	246240	chr15	34484377	34484677	-4636	-1.8	8.54E-03	1.79E-01	Distal Intergenic
Col4a1	290905	chr16	83507756	83508056	-14106	1.04	8.54E-03	1.79E-01	Distal Intergenic

Lrrc28	361588	chr1	128571705	128572005	32165	-1.68	8.55E-03	1.79E-01	Intron (NM_001108486/361588, intron 5 of 9)
Atxn1	25049	chr17	19265569	19265869	104575	-1.56	8.56E-03	1.79E-01	Intron (NM_012726/25049, intron 2 of 6)
Camk2d	24246	chr2	230924577	230924877	23451	2.11	8.57E-03	1.79E-01	Intron (NM_012519/24246, intron 2 of 20)
Mir6332	102466626	chr5	167543326	167543626	140358	1.84	8.58E-03	1.79E-01	Intron (NM_053885/116665, intron 7 of 22)
Ezh1	303547	chr10	89136134	89136434	-5795	-1.55	8.58E-03	1.79E-01	Distal Intergenic
Ifnar1	288264	chr11	31645791	31646091	5384	-2.09	8.58E-03	1.79E-01	Intron (NM_001105893/288264, intron 1 of 10)
Pabpc1	171350	chr7	75379760	75380060	41814	-2.09	8.58E-03	1.79E-01	Distal Intergenic
Ldhd	307858	chr19	43810414	43810714	38223	-1.92	8.59E-03	1.79E-01	Distal Intergenic
Odf3l1	315695	chr8	61516028	61516328	2964	-1.92	8.59E-03	1.79E-01	Promoter (2-3kb)
Syncrip	363113	chr8	96119832	96120132	12606	-1.61	8.60E-03	1.79E-01	Intron (NM_001047916/363113, intron 4 of 8)
Gfpt1	297417	chr4	118910864	118911164	58745	2.07	8.60E-03	1.79E-01	Distal Intergenic
P2ry2	29597	chr1	166082982	166083282	-37562	-1.81	8.60E-03	1.79E-01	Distal Intergenic
Abca1	313210	chr5	69980192	69980492	-27726	-1.78	8.61E-03	1.79E-01	Distal Intergenic
Elob	81807	chr10	13168211	13168511	0	1.34	8.61E-03	1.79E-01	Promoter (<=1kb)
Socs3	89829	chr10	106950030	106950330	25710	-1.33	8.61E-03	1.79E-01	Distal Intergenic
Parva	57341	chr1	177226408	177226708	-22594	-1.43	8.62E-03	1.79E-01	Intron (NM_182669/293180, intron 8 of 9)
Glrx	64045	chr2	2609952	2610252	3920	-1.44	8.62E-03	1.79E-01	Intron (NM_022278/64045, intron 1 of 2)
Kcnab1	29737	chr2	155718815	155719115	456	-1.81	8.63E-03	1.79E-01	Promoter (<=1kb)
Nenf	289380	chr13	109951631	109951931	45161	1.63	8.63E-03	1.79E-01	Distal Intergenic
Olig3	293012	chr1	14602183	14602483	-195283	-1.86	8.63E-03	1.79E-01	Distal Intergenic
Rybp	312603	chr4	132742004	132742304	-1181	0.93	8.64E-03	1.80E-01	Promoter (1-2kb)
Sipa1l3	292771	chr1	87372678	87372978	-74075	-1.64	8.65E-03	1.80E-01	Distal Intergenic
Fhl4	314678	chr7	26144003	26144303	-2738	0.79	8.65E-03	1.80E-01	Promoter (2-3kb)
Ror2	306782	chr17	12006152	12006452	52600	1.49	8.66E-03	1.80E-01	Intron (NM_001107339/306782, intron 1 of 8)
Thy1	24832	chr8	48271504	48271804	-110317	-1.51	8.67E-03	1.80E-01	Distal Intergenic
Pipox	303272	chr10	64953798	64954098	1488	2.64	8.67E-03	1.80E-01	Promoter (1-2kb)
Gli2	304729	chr13	34807402	34807702	77783	-1.72	8.68E-03	1.80E-01	Distal Intergenic
Slc6a6	29464	chr4	123712622	123712922	397	0.78	8.68E-03	1.80E-01	Promoter (<=1kb)
Psip1	313323	chr5	102086540	102086840	-498442	-1.78	8.69E-03	1.80E-01	Distal Intergenic
Cldn20	680178	chr1	44252751	44253051	-193714	2.04	8.70E-03	1.80E-01	Distal Intergenic
Ier3	294235	chr20	3487332	3487632	-47330	-0.98	8.71E-03	1.80E-01	Distal Intergenic
Nampt	297508	chr6	52128666	52128966	6581	-1.84	8.72E-03	1.80E-01	Intron (NM_177928/297508, intron 1 of 10)
Bnc2	298189	chr5	102342773	102343073	400344	-1.86	8.73E-03	1.81E-01	Distal Intergenic
Psen1	29192	chr6	107176643	107176943	-4109	-1.8	8.74E-03	1.81E-01	Distal Intergenic
Capza2	493810	chr4	44936381	44936681	91	0.92	8.74E-03	1.81E-01	Promoter (<=1kb)
Psmb6	29666	chr10	57130732	57131032	-346	-1.03	8.75E-03	1.81E-01	Promoter (<=1kb)
Zfp516	291406	chr18	79764536	79764836	-48923	2.01	8.75E-03	1.81E-01	Distal Intergenic
Dusp7	300980	chr8	115031159	115031459	-37636	2.16	8.75E-03	1.81E-01	Exon (NM_001109296/501048, exon 9 of 16)
Ptgs1	24693	chr3	15581184	15581484	20461	-1.91	8.75E-03	1.81E-01	3' UTR
Kcng3	171011	chr6	6936089	6936389	-93331	-2.1	8.76E-03	1.81E-01	Distal Intergenic
Tmem265	102554586	chr1	199009387	199009687	1750	2.12	8.76E-03	1.81E-01	Promoter (1-2kb)
Rnf11l1	100364162	chr5	129112710	129113010	122	1.28	8.76E-03	1.81E-01	Promoter (<=1kb)
Zc3h12c	315658	chr8	56696540	56696840	128	1.16	8.76E-03	1.81E-01	Promoter (<=1kb)
Nfx1	313166	chr5	57306357	57306657	15201	-1.64	8.77E-03	1.81E-01	Exon (NM_001024784/313166, exon 5 of 24)
Cep120	307302	chr18	48715237	48715537	4935	-1.54	8.77E-03	1.81E-01	Intron (NM_001191697/307302, intron 3 of 20)
Sgf29	293488	chr1	198067080	198067380	14	0.68	8.78E-03	1.81E-01	Promoter (<=1kb)
Vsig8	289236	chr13	90950656	90950956	7401	1.25	8.78E-03	1.81E-01	Exon (NM_001105972/289236, exon 7 of 7)
Tspan18	311210	chr3	82217691	82217991	18673	2.26	8.79E-03	1.81E-01	Intron (NM_001107750/311210, intron 1 of 9)
Fndc5	260327	chr5	147330481	147330781	7241	2.23	8.80E-03	1.81E-01	Distal Intergenic
Glg1	29476	chr19	43057207	43057507	73328	1.81	8.80E-03	1.81E-01	Intron (NM_017211/29476, intron 8 of 25)
C1qtnf7	305423	chr14	72038710	72039010	-13573	2.19	8.81E-03	1.81E-01	Distal Intergenic
Oasl	304545	chr12	47459331	47459631	-3171	-1.67	8.82E-03	1.81E-01	Distal Intergenic
Slc1a7	366432	chr5	127587650	127587950	16536	1.76	8.82E-03	1.81E-01	Intron (NM_001108973/366432, intron 2 of 10)
Pex14	64460	chr5	165741486	165741786	176659	1.87	8.85E-03	1.82E-01	Distal Intergenic
Lyz2	25211	chr7	60333957	60334257	7007	1.68	8.87E-03	1.82E-01	Downstream (1-2kb)
Rybp	312603	chr4	132767333	132767633	-26510	-1.46	8.88E-03	1.82E-01	Distal Intergenic
Nqo1	24314	chr19	38429557	38429857	7347	-1.7	8.88E-03	1.82E-01	Intron (NM_017000/24314, intron 1 of 5)
Itgb4	25724	chr10	104514920	104515220	-8780	-1.8	8.88E-03	1.82E-01	Distal Intergenic
Metrnl	316842	chr10	110908554	110908854	15293	1.84	8.88E-03	1.82E-01	Distal Intergenic
Akap2	298024	chr5	74811096	74811396	-130695	-1.68	8.89E-03	1.82E-01	Distal Intergenic
Tpx2	311546	chr3	148332349	148332649	4384	-1.7	8.89E-03	1.82E-01	Intron (NM_001107790/311546, intron 1 of 16)
Kif11	171304	chr1	256040710	256041010	4844	-1.55	8.90E-03	1.82E-01	Intron (NM_001169112/171304, intron 1 of 21)
Ddias	499204	chr1	157696386	157696686	3378	-1.89	8.90E-03	1.82E-01	Intron (NM_001126294/499204, intron 2 of 5)
Col16a1	366474	chr5	148257277	148257577	-16	0.81	8.90E-03	1.82E-01	Promoter (<=1kb)
Ier3	294235	chr20	3451313	3451613	-11311	-1.5	8.90E-03	1.82E-01	Distal Intergenic
Ssrp1	81785	chr3	72475291	72475591	27322	-1.51	8.91E-03	1.82E-01	Distal Intergenic
Cep83	366872	chr7	35898785	35899085	159144	1.33	8.91E-03	1.82E-01	Distal Intergenic
Nfil3	114519	chr17	12277616	12277916	16514	-1.38	8.91E-03	1.82E-01	Distal Intergenic
Elk3	362871	chr7	34110084	34110384	11310	1.22	8.91E-03	1.82E-01	Intron (NM_001108743/362871, intron 1 of 3)
Fus	317385	chr1	199418768	199419068	5934	-1.72	8.93E-03	1.82E-01	Intron (NM_001012137/317385, intron 6 of 14)
Bag5	366734	chr6	136278923	136279223	139	1.47	8.96E-03	1.83E-01	Promoter (<=1kb)
Rhoc	295342	chr2	207278726	207279026	6767	2.37	8.96E-03	1.83E-01	Exon (NM_001107711/310756, exon 19 of 21)
Cplx2	116657	chr17	10804749	10805049	-38496	-1.89	8.97E-03	1.83E-01	Distal Intergenic
Manea	140808	chr5	42036783	42037083	-1176149	1.8	8.99E-03	1.83E-01	Distal Intergenic
Mical1	294520	chr20	46200277	46200577	296	0.74	9.00E-03	1.83E-01	Promoter (<=1kb)
Mirlet7i	100313993	chr7	66898937	66899237	96206	-1.99	9.00E-03	1.83E-01	Intron (NM_001271079/314897, intron 6 of 9)
Inhbb	25196	chr13	35441549	35441849	373	1.35	9.01E-03	1.83E-01	Promoter (<=1kb)
Osbp2	305475	chr14	84093372	84093672	13325	1.32	9.01E-03	1.83E-01	Intron (NM_001107232/305475, intron 1 of 14)
Mogat1	363261	chr9	84477850	84478150	66878	-1.85	9.01E-03	1.83E-01	Distal Intergenic
Klf5	84410	chr15	83711141	83711441	3406	-1.71	9.01E-03	1.83E-01	Exon (NM_053394/84410, exon 2 of 4)
Gns	299825	chr7	63407119	63407419	-59703	2.24	9.02E-03	1.83E-01	Distal Intergenic
Nup50	25497	chr7	125837023	125837323	3466	-1.83	9.02E-03	1.83E-01	5' UTR
Ypel2	360590	chr10	74273821	74274121	24478	1.83	9.02E-03	1.83E-01	Intron (NM_001108286/360590, intron 2 of 4)
Ccl17	117518	chr19	10617508	10617808	2863	-1.94	9.02E-03	1.83E-01	Promoter (2-3kb)
Tnfrsf1b	156767	chr5	163180402	163180702	-13103	-1.94	9.02E-03	1.83E-01	Distal Intergenic
Ubap2	313169	chr5	57626978	57627278	4904	-1.72	9.03E-03	1.83E-01	Intron (NM_001107928/313169, intron 1 of 28)
Tmem17	360985	chr14	107329355	107329655	26547	-1.59	9.04E-03	1.83E-01	Distal Intergenic
Fgl1	246186	chr16	54203556	54203856	50470	2.34	9.04E-03	1.83E-01	Distal Intergenic
Ccnq	303321	chr10	66020317	66020617	65	0.83	9.05E-03	1.83E-01	Promoter (<=1kb)
Spryd4	288772	chr7	2625281	2625581	-1500	-1.9	9.08E-03	1.84E-01	Promoter (1-2kb)
Ubn2	312248	chr4	66165966	66166266	121	1.12	9.09E-03	1.84E-01	Promoter (<=1kb)
Ube2v2	287927	chr11	89816805	89817105	259532	2.18	9.09E-03	1.84E-01	Distal Intergenic
Rmdn2	313840	chr6	2141829	2142129	-74494	2.14	9.09E-03	1.84E-01	Distal Intergenic
Tsga10	252923	chr9	44299613	44299913	156654	-2	9.09E-03	1.84E-01	Distal Intergenic

Srsf9	288701	chr12	47042112	47042412	3610	2.27	9.10E-03	1.84E-01	Exon (NM_001009255/288701, exon 2 of 4)
Dip2c	307067	chr17	63516240	63516540	89224	-1.9	9.10E-03	1.84E-01	Intron (NM_001107360/307067, intron 1 of 37)
Myo9a	171296	chr8	64573293	64573593	45	0.75	9.11E-03	1.84E-01	Promoter (<=1kb)
Mad2l1bp	316237	chr9	17219259	17219559	2118	-1.23	9.11E-03	1.84E-01	Promoter (2-3kb)
Rap1b	171337	chr7	60879322	60879622	-18332	-1.42	9.11E-03	1.84E-01	Distal Intergenic
Aipl1	59110	chr10	58563197	58563497	45409	-2.06	9.11E-03	1.84E-01	Distal Intergenic
Srsf4	362612	chr5	150082360	150082660	49361	-1.62	9.12E-03	1.84E-01	Distal Intergenic
Lrrc2	301033	chr8	119208845	119209145	-19585	1.5	9.12E-03	1.84E-01	Distal Intergenic
Sec31a	93646	chr14	10861362	10861662	6625	-1.82	9.14E-03	1.84E-01	Intron (NM_033021/93646, intron 1 of 27)
Mtmr7	306490	chr16	54807736	54808036	42411	-1.82	9.14E-03	1.84E-01	Intron (NM_001107312/306490, intron 3 of 13)
Apol9a	503164	chr7	118928858	118929158	4620	-1.51	9.16E-03	1.85E-01	5' UTR
Rpn1	25596	chr4	120003953	120004253	6721	-1.86	9.16E-03	1.85E-01	Intron (NM_013067/25596, intron 3 of 9)
Trim39	309591	chr20	2501061	2501361	370	0.98	9.19E-03	1.85E-01	Promoter (<=1kb)
Clpx	300786	chr8	70795269	70795569	5887	-1.21	9.19E-03	1.85E-01	Intron (NM_001007803/300786, intron 1 of 13)
RGD1304884	307907	chr19	53709525	53709825	-13997	-1.54	9.19E-03	1.85E-01	Distal Intergenic
Gadd45g	291005	chr17	13139091	13139391	253828	-1.72	9.21E-03	1.85E-01	Distal Intergenic
Adam15	57025	chr2	188672084	188672384	0	1.04	9.22E-03	1.85E-01	Promoter (<=1kb)
Pxdc1	361238	chr17	30936482	30936782	-29160	-1.5	9.22E-03	1.85E-01	Distal Intergenic
G2e3	299002	chr6	72044606	72044906	-47288	-1.55	9.22E-03	1.85E-01	Distal Intergenic
Hdac4	363287	chr9	99298923	99299223	492	1.07	9.22E-03	1.85E-01	Promoter (<=1kb)
Zfp608	307296	chr18	49864046	49864346	73176	-1.42	9.22E-03	1.85E-01	Intron (NM_001107378/307296, intron 3 of 10)
Mapk11	689314	chr7	130128250	130128550	39	1.08	9.24E-03	1.85E-01	Promoter (<=1kb)
Utp15	310019	chr2	28314670	28314970	55291	-1.5	9.24E-03	1.85E-01	Distal Intergenic
Tlr2	310553	chr2	182867851	182868151	-21790	-1.51	9.24E-03	1.85E-01	Distal Intergenic
Fndc3a	306022	chr15	54566518	54566818	-38038	1.18	9.25E-03	1.85E-01	Distal Intergenic
Lta	25008	chr20	4848248	4848548	-4123	-1.73	9.25E-03	1.85E-01	Intron (NM_001008837/414270, intron 6 of 7)
Ppp1cb	25594	chr6	23569299	23569599	11453	-1.7	9.26E-03	1.85E-01	Intron (NM_013065/25594, intron 1 of 7)
Mir21	100314000	chr10	73934426	73934726	-32125	-1.82	9.26E-03	1.85E-01	Intron (NM_138839/192129, intron 7 of 11)
Chst11	314694	chr7	26706406	26706706	153010	-1.69	9.26E-03	1.85E-01	Intron (NM_001108079/314694, intron 2 of 2)
Abhd17a	299617	chr7	11992638	11992938	95	1.28	9.27E-03	1.85E-01	Promoter (<=1kb)
Mir222	100314059	chrX	3682286	3682586	-1333	-1.72	9.27E-03	1.85E-01	Promoter (1-2kb)
Ldlrad4	679578	chr18	63733533	63733833	-110166	1.1	9.28E-03	1.85E-01	Distal Intergenic
Cmas	312826	chr4	177049620	177049920	55491	-1.53	9.28E-03	1.85E-01	Distal Intergenic
Irx3	307721	chr19	16057912	16058212	-216922	-1.27	9.28E-03	1.85E-01	Distal Intergenic
Chd2	308738	chr1	134852985	134853285	17883	-1.38	9.28E-03	1.85E-01	Intron (NM_001107523/308738, intron 2 of 38)
Robo1	58946	chr11	9763309	9763609	553741	-2.08	9.29E-03	1.85E-01	Intron (NM_022188/58946, intron 2 of 29)
Galnt7	29750	chr16	35935836	35936136	777	0.8	9.30E-03	1.86E-01	Promoter (<=1kb)
Errfi1	313729	chr5	167958230	167958530	5502	-1.34	9.31E-03	1.86E-01	Intron (NM_001014071/313729, intron 1 of 3)
Tmx1	362751	chr6	93069839	93070139	205716	2.33	9.31E-03	1.86E-01	Distal Intergenic
Scgn	306942	chr17	43255687	43255987	-101901	-1.41	9.32E-03	1.86E-01	Intron (NM_001191692/306941, intron 21 of 37)
Bcl2l13	312682	chr4	153410162	153410462	24958	-1.42	9.32E-03	1.86E-01	Intron (NM_001107885/312682, intron 3 of 6)
Als2	363235	chr9	66030787	66031087	2784	-1.75	9.33E-03	1.86E-01	Promoter (2-3kb)
Cdk5r1	116671	chr10	67880938	67881238	18874	1.76	9.33E-03	1.86E-01	Intron (NM_001012010/303350, intron 6 of 6)
Cavin2	316384	chr9	55252988	55253288	2865	2.07	9.33E-03	1.86E-01	Promoter (2-3kb)
Ckap2	306575	chr16	74756998	74757298	4343	-1.76	9.34E-03	1.86E-01	Intron (NM_001169139/306575, intron 1 of 8)
Disc1	307940	chr19	58084309	58084609	264049	-1.58	9.35E-03	1.86E-01	Distal Intergenic
Fbln1	315191	chr7	126132435	126132735	35642	1.44	9.36E-03	1.86E-01	Intron (NM_001127547/315191, intron 13 of 16)
Rassf8	312846	chr4	180078709	180079009	15910	-1.69	9.36E-03	1.86E-01	Intron (NM_001191753/312846, intron 1 of 4)
Spg7	353231	chr19	55861679	55861979	-18582	1.23	9.37E-03	1.86E-01	Distal Intergenic
Mir30d	100314010	chr7	109315137	109315437	-28593	0.77	9.37E-03	1.86E-01	Distal Intergenic
Slc7a1	25648	chr12	7987840	7988140	-44635	-1.6	9.38E-03	1.86E-01	Distal Intergenic
Septin9	83788	chr10	106236986	106237286	-3915	-1.85	9.38E-03	1.86E-01	Intron (NM_176856/83788, intron 2 of 7)
C2cd6	501147	chr9	65862559	65862859	16662	-1.77	9.38E-03	1.86E-01	Intron (NM_001024363/501147, intron 3 of 12)
Kcnt1	60444	chr3	3351283	3351583	25513	-0.85	9.38E-03	1.86E-01	Exon (NM_021853/60444, exon 19 of 32)
Nr2f1	81808	chr2	5575675	5575975	3919	1.9	9.39E-03	1.86E-01	Intron (NM_031130/81808, intron 3 of 4)
Tmem53	313529	chr5	136112381	136112681	0	0.68	9.40E-03	1.86E-01	Promoter (<=1kb)
Csrp2	29317	chr7	53641930	53642230	11255	0.93	9.42E-03	1.87E-01	5' UTR
Cutc	361760	chr1	263511624	263511924	87	0.71	9.43E-03	1.87E-01	Promoter (<=1kb)
Ropn1l	685646	chr2	84529547	84529847	1345	2.02	9.45E-03	1.87E-01	Promoter (1-2kb)
Sipa1l1	246212	chr6	105834839	105835139	-217073	1.53	9.45E-03	1.87E-01	Distal Intergenic
Nedd4	25489	chr8	79360818	79361118	37410	-1.7	9.45E-03	1.87E-01	Intron (NM_012986/25489, intron 6 of 28)
Ankrd50	294988	chr2	125318606	125318906	2994	-1.45	9.45E-03	1.87E-01	Promoter (2-3kb)
Creb3l1	362165	chr3	81095428	81095728	-162145	1.05	9.46E-03	1.87E-01	Distal Intergenic
LOC257642	257642	chr14	46655474	46655774	-3648	2.7	9.46E-03	1.87E-01	Exon (NR_046239/24723, exon 2 of 2)
Ap1s1	360785	chr12	22659676	22659976	-5182	-1.62	9.46E-03	1.87E-01	Distal Intergenic
Slc22a14	316061	chr8	127869725	127870025	1167	0.94	9.46E-03	1.87E-01	Promoter (1-2kb)
RGD1359158	361740	chr1	239255773	239256073	9924	-1.24	9.48E-03	1.87E-01	Intron (NM_001007737/361740, intron 1 of 3)
Mrps10	363187	chr9	15654616	15654916	-3462	2.13	9.48E-03	1.87E-01	Downstream (<1kb)
P2rx5	113995	chr10	59718191	59718491	-7033	-1.33	9.48E-03	1.87E-01	Distal Intergenic
Chd2	308738	chr1	134857690	134857990	13178	-1.41	9.49E-03	1.87E-01	Intron (NM_001107523/308738, intron 2 of 38)
Lrrc28	361588	chr1	128570388	128570688	33482	-1.61	9.49E-03	1.87E-01	Intron (NM_001108486/361588, intron 5 of 9)
Atxn1	25049	chr17	19123986	19124286	-36708	-1.29	9.49E-03	1.87E-01	Distal Intergenic
Cox6b1	688869	chr1	89081620	89081920	1297	2.47	9.50E-03	1.87E-01	Promoter (1-2kb)
Mfrp	315597	chr8	48424828	48425128	-13131	2.03	9.50E-03	1.87E-01	Intron (NM_053774/115771, intron 2 of 12)
Smyd3	498295	chr13	97792420	97792720	15093	0.72	9.50E-03	1.87E-01	Intron (NM_001025762/498295, intron 1 of 9)
Itgb1	24511	chr19	61629420	61629720	-47822	-0.98	9.51E-03	1.87E-01	Distal Intergenic
Camk2d	24246	chr2	231331755	231332055	430629	1.56	9.52E-03	1.87E-01	Distal Intergenic
Arcn1	300674	chr8	49069221	49069521	6340	-1.73	9.53E-03	1.87E-01	Intron (NM_001007662/300674, intron 1 of 9)
Ptpn11	25622	chr12	40913599	40913899	18084	1.94	9.53E-03	1.87E-01	Intron (NM_001177593/25622, intron 1 of 15)
Elovl5	171400	chr8	85213840	85214140	-45848	0.65	9.53E-03	1.87E-01	Distal Intergenic
Adcy8	29241	chr7	105284626	105284926	307878	-1.76	9.54E-03	1.87E-01	Distal Intergenic
Lhx5	124451	chr12	41802197	41802497	-130760	1	9.54E-03	1.87E-01	Distal Intergenic
Npc2	286898	chr6	108484301	108484601	3729	-1.85	9.54E-03	1.87E-01	Intron (NM_173118/286898, intron 1 of 3)
Ttc23	308708	chr1	128663251	128663551	49113	2.06	9.55E-03	1.87E-01	Intron (NM_001025681/308708, intron 7 of 10)
Dlg1	25252	chr11	72280906	72281206	97777	-1.7	9.56E-03	1.87E-01	Intron (NM_012788/25252, intron 6 of 29)
B4galt6	65196	chr18	15498414	15498714	35501	-1.52	9.56E-03	1.87E-01	Intron (NM_001101000/679221, intron 3 of 6)
Utrn	25600	chr1	6914561	6914861	55179	1.76	9.57E-03	1.88E-01	Intron (NM_013070/25600, intron 2 of 74)
C1galt1	65044	chr4	33892531	33892831	-15468	1.6	9.57E-03	1.88E-01	Distal Intergenic
Ehd3	192249	chr6	25081606	25081906	5459	-1.41	9.58E-03	1.88E-01	Intron (NM_138890/192249, intron 1 of 5)
Chd2	308738	chr1	134820125	134820425	50743	-1.67	9.58E-03	1.88E-01	Intron (NM_001107523/308738, intron 16 of 38)
Niban2	362115	chr3	11939434	11939734	17719	-1.87	9.59E-03	1.88E-01	Intron (NM_001109885/362115, intron 1 of 13)
Stat3	25125	chr10	88841908	88842208	25	0.78	9.60E-03	1.88E-01	Promoter (<=1kb)
Nt5c3b	360629	chr10	88343082	88343382	13156	2	9.60E-03	1.88E-01	Intron (NM_001007723/360629, intron 10 of 10)
Gpx4	29328	chr7	12504239	12504539	14145	-1.05	9.60E-03	1.88E-01	Intron (NM_001108068/314619, intron 6 of 32)

Arhgap12	307016	chr17	54273172	54273472	-7379	-1.33	9.62E-03	1.88E-01	Distal Intergenic
Psmd1	83806	chr9	93084456	93084756	3838	-1.71	9.62E-03	1.88E-01	Intron (NM_031978/83806, intron 1 of 24)
Elovl5	171400	chr8	85250251	85250551	-9437	-1.46	9.62E-03	1.88E-01	Distal Intergenic
Plekhb2	301337	chr9	41883215	41883515	534768	-1.29	9.63E-03	1.88E-01	Distal Intergenic
Cebpb	24253	chr3	164523748	164524048	99246	-0.95	9.63E-03	1.88E-01	Distal Intergenic
Lpcat2	100359680	chr19	15538739	15539039	1665	-1.1	9.65E-03	1.88E-01	Promoter (1-2kb)
Arrdc3	309945	chr2	8738862	8739162	5695	-1.72	9.65E-03	1.88E-01	Intron (NM_001007797/309945, intron 3 of 7)
F7	260320	chr16	81879863	81880163	-44940	0.86	9.66E-03	1.88E-01	Exon (NM_053951/117020, exon 4 of 31)
Zfp52	361487	chr1	61763507	61763807	365013	-1.6	9.66E-03	1.88E-01	Distal Intergenic
Arsj	311013	chr2	230162529	230162829	-185	0.71	9.68E-03	1.89E-01	Promoter (<=1kb)
Zc3h6	311415	chr3	121510634	121510934	11439	-1.81	9.72E-03	1.89E-01	Intron (NM_001107772/311415, intron 1 of 11)
Zc3h13	305955	chr15	57340594	57340894	15	0.72	9.72E-03	1.89E-01	Promoter (<=1kb)
Actl7b	313183	chr5	73170150	73170450	323580	1.88	9.73E-03	1.89E-01	Distal Intergenic
E2f3	291105	chr17	36339283	36339583	5147	-1.62	9.73E-03	1.89E-01	Intron (NM_001137626/291105, intron 2 of 6)
Gprasp1	171407	chrX	106251069	106251369	-55426	0.8	9.73E-03	1.89E-01	Distal Intergenic
Lhfpl2	294643	chr2	23752693	23752993	-17715	0.89	9.73E-03	1.89E-01	Distal Intergenic
Myc	24577	chr7	103109267	103109567	522954	-1.67	9.74E-03	1.89E-01	Distal Intergenic
Speg	363256	chr9	82566509	82566809	-4536	-1.5	9.74E-03	1.89E-01	Distal Intergenic
Nabp1	363227	chr9	54946888	54947188	-103006	-1.8	9.75E-03	1.89E-01	Distal Intergenic
Misp	500797	chr7	12703946	12704246	3676	-1.75	9.75E-03	1.89E-01	Intron (NM_001108067/314618, intron 22 of 22)
Zfp347	170902	chr7	9992535	9992835	-1575	-1.57	9.76E-03	1.89E-01	Promoter (1-2kb)
LOC100910945	100910945	chr20	27380488	27380788	27	-2.08	9.76E-03	1.89E-01	Promoter (<=1kb)
Tmem11	303196	chr10	47110004	47110304	21538	-1.67	9.76E-03	1.89E-01	Intron (NM_001008507/287380, intron 6 of 6)
Egln3	54702	chr6	75063638	75063938	11857	-2.08	9.76E-03	1.89E-01	Intron (NM_019371/54702, intron 1 of 4)
Zfp637	362425	chr4	149691136	149691436	-186453	2.02	9.77E-03	1.89E-01	Distal Intergenic
Tmem192	361137	chr16	26739221	26739521	-96704	2.31	9.77E-03	1.89E-01	Distal Intergenic
Gpat2	296130	chr3	119908093	119908393	-86144	-1.9	9.77E-03	1.89E-01	Distal Intergenic
Spin1	361217	chr16	1107116	1107416	-176589	-1.71	9.77E-03	1.89E-01	Distal Intergenic
H1f0	24437	chr7	120235026	120235326	-25450	1.79	9.78E-03	1.89E-01	Distal Intergenic
Gprc5c	287805	chr10	103368830	103369130	-27025	2.37	9.78E-03	1.89E-01	Distal Intergenic
B4galnt1	64828	chr7	70452631	70452931	0	1.41	9.78E-03	1.89E-01	Promoter (<=1kb)
Hmgcll1	367112	chr8	82891555	82891855	-178639	1.88	9.78E-03	1.89E-01	Intron (NM_001108168/315824, intron 3 of 6)
Csf1	78965	chr2	210552148	210552448	-1602	-1.55	9.78E-03	1.89E-01	Promoter (1-2kb)
Mir6215	102465145	chr5	158071393	158071693	-10333	-1.98	9.79E-03	1.89E-01	Exon (NM_001134703/641315, exon 3 of 9)
Paqr8	316275	chr9	27033517	27033817	-205	2.14	9.80E-03	1.89E-01	Promoter (<=1kb)
Tmem37	245953	chr13	36086408	36086708	14405	2.25	9.80E-03	1.89E-01	Intron (NM_031115/81779, intron 13 of 14)
Cfdp1	292027	chr19	44044457	44044757	33563	-1.48	9.81E-03	1.89E-01	Intron (NM_199378/292027, intron 5 of 6)
Nedd1	299730	chr7	33584093	33584393	13	0.68	9.81E-03	1.89E-01	Promoter (<=1kb)
Plac8	360914	chr14	10695113	10695413	2314	-1.78	9.81E-03	1.89E-01	Promoter (2-3kb)
Cyth4	500906	chr7	119829855	119830155	9315	-1.43	9.81E-03	1.89E-01	Intron (NM_001130577/500906, intron 3 of 11)
Tubg1	252921	chr10	89031176	89031476	311	0.67	9.82E-03	1.89E-01	Promoter (<=1kb)
Dad1	192275	chr15	32855692	32855992	31941	-1.25	9.82E-03	1.89E-01	Distal Intergenic
Tspan18	311210	chr3	82200687	82200987	35677	1.88	9.83E-03	1.89E-01	Intron (NM_001107750/311210, intron 2 of 9)
Dhrs3	313689	chr5	162919833	162920133	110743	2.1	9.83E-03	1.89E-01	Intron (NM_001108006/313825, intron 66 of 69)
Ambp	25377	chr5	78806550	78806850	179076	2.59	9.84E-03	1.89E-01	Distal Intergenic
Exoc6	50556	chr1	256255362	256255662	29178	-1.61	9.85E-03	1.89E-01	Exon (NM_019277/50556, exon 2 of 22)
Sema5a	310207	chr2	85467645	85467945	90327	-1.49	9.87E-03	1.90E-01	Intron (NM_001107659/310207, intron 1 of 22)
Lmna	60374	chr2	187886226	187886526	-22674	-1.79	9.87E-03	1.90E-01	Distal Intergenic
Agap1	316611	chr9	96699569	96699869	-46571	1.27	9.88E-03	1.90E-01	Distal Intergenic
Ripk4	304053	chr11	38218971	38219271	54963	-1.5	9.88E-03	1.90E-01	Distal Intergenic
Clic6	304081	chr11	32619834	32620134	-35519	0.94	9.89E-03	1.90E-01	Distal Intergenic
Tinagl1	94174	chr5	148385114	148385414	6667	1.65	9.89E-03	1.90E-01	Exon (NM_053582/94174, exon 5 of 12)
Crip3	501100	chr9	16977660	16977960	-42946	1.02	9.90E-03	1.90E-01	Distal Intergenic
Cdk5r1	116671	chr10	67874657	67874957	12593	1.84	9.91E-03	1.90E-01	Intron (NM_001012010/303350, intron 6 of 6)
Magi3	245903	chr2	206675255	206675555	23550	2.17	9.92E-03	1.90E-01	Intron (NM_139084/245903, intron 1 of 20)
Bcorl1	302810	chrX	135168990	135169290	-18178	1.58	9.94E-03	1.90E-01	Distal Intergenic
Rraga	117044	chr5	104350287	104350587	-590480	-1.49	9.94E-03	1.90E-01	Distal Intergenic
Serpine2	29366	chr9	85727054	85727354	-100960	-1.14	9.95E-03	1.91E-01	Distal Intergenic
Cops8	363283	chr9	97682369	97682669	-89555	-1.5	9.95E-03	1.91E-01	Distal Intergenic
Zbtb43	311872	chr3	12524135	12524435	-14830	0.81	9.95E-03	1.91E-01	Distal Intergenic
Rnd1	362993	chr7	140364316	140364616	-8107	-1.66	9.97E-03	1.91E-01	Distal Intergenic
Riox1	314300	chr6	107398777	107399077	-29431	-1.71	9.98E-03	1.91E-01	Distal Intergenic
Lamc2	192362	chr13	70505224	70505524	120728	1.91	9.98E-03	1.91E-01	Intron (NM_001048042/289095, intron 1 of 10)
Fgf5	60662	chr14	13145734	13146034	-150150	-1.79	1.00E-02	1.91E-01	Distal Intergenic
Map3k8	116596	chr17	56185916	56186216	76513	-1.7	1.00E-02	1.91E-01	Distal Intergenic
Spg7	353231	chr19	55844157	55844457	-36104	-1.65	1.00E-02	1.91E-01	Distal Intergenic
Vrk1	362779	chr6	129829634	129829934	-5985	-1.72	1.00E-02	1.91E-01	Distal Intergenic
Syngr2	89815	chr10	106812823	106813123	80	1.05	1.00E-02	1.91E-01	Promoter (<=1kb)
Cfap300	654482	chr8	6233107	6233407	2560	2.2	1.00E-02	1.91E-01	Promoter (2-3kb)
Plaa	116645	chr5	113557803	113558103	20825	-1.57	1.00E-02	1.91E-01	Intron (NM_053866/116645, intron 10 of 13)
Slc27a1	94172	chr16	20009130	20009430	10018	-1.48	1.00E-02	1.91E-01	Exon (NM_053580/94172, exon 4 of 13)
Snw1	500695	chr6	111534404	111534704	-26622	1.07	1.00E-02	1.91E-01	Distal Intergenic
Tns3	360980	chr14	88835936	88836236	10635	-1.81	1.00E-02	1.91E-01	Intron (NM_001170459/360980, intron 1 of 28)
Extl3	56819	chr15	48368744	48369044	76548	-1.81	1.00E-02	1.91E-01	Distal Intergenic
Fosl2	25446	chr6	25712699	25712999	-95704	2.18	1.00E-02	1.91E-01	Intron (NM_199270/362704, intron 10 of 11)
Shroom4	317391	chrX	16929527	16929827	2	0.86	1.00E-02	1.91E-01	Promoter (<=1kb)
Clcn3	84360	chr16	32476154	32476454	27038	0.7	1.00E-02	1.91E-01	Intron (NM_053363/84360, intron 2 of 12)
Nf1	24592	chr10	66694448	66694748	4502	-1.66	1.01E-02	1.91E-01	Intron (NM_012609/24592, intron 5 of 60)
Epc1	100362678	chr17	57329672	57329972	65003	-1.78	1.01E-02	1.91E-01	Intron (NM_001309462/100362678, intron 2 of 13)
Hsd17b3	117182	chr17	1440369	1440669	170076	2.08	1.01E-02	1.91E-01	Distal Intergenic
Dpysl3	25418	chr18	37772519	37772819	3634	-1.2	1.01E-02	1.91E-01	Intron (NM_012934/25418, intron 1 of 13)
LOC100361645	100361645	chr3	46107548	46107848	-77512	-1.66	1.01E-02	1.91E-01	Intron (NM_001108260/317627, intron 3 of 38)
Tmem135	293098	chr1	152908525	152908825	187355	2.03	1.01E-02	1.91E-01	Intron (NM_001013896/293098, intron 7 of 14)
Prrx1	266813	chr13	81373292	81373592	-158470	-1.58	1.01E-02	1.91E-01	Distal Intergenic
Rc3h2	311909	chr3	21676326	21676626	53	0.92	1.01E-02	1.91E-01	Promoter (<=1kb)
Il9	116558	chr17	8580271	8580571	21444	-1.94	1.01E-02	1.91E-01	Distal Intergenic
Hsd17b12	84013	chr3	83000606	83000906	47383	-2.1	1.01E-02	1.91E-01	Intron (NM_032066/84013, intron 3 of 10)
Abcc12	291923	chr19	21543921	21544221	2179	1.86	1.01E-02	1.91E-01	Promoter (2-3kb)
Etv6	312777	chr4	167809295	167809595	54611	-1.96	1.01E-02	1.91E-01	Intron (NM_001037353/312777, intron 1 of 7)
Fst	24373	chr2	46676414	46676714	-131962	2.06	1.01E-02	1.91E-01	Distal Intergenic
Sntb2	689421	chr19	39126802	39127102	213	1.12	1.01E-02	1.91E-01	Promoter (<=1kb)
LOC691807	691807	chr2	227095515	227095815	28	0.6	1.01E-02	1.91E-01	Promoter (<=1kb)
Fgf5	60662	chr14	13187316	13187616	-191732	-1.69	1.01E-02	1.91E-01	Distal Intergenic
Ankrd50	294988	chr2	125064019	125064319	257581	1.79	1.01E-02	1.91E-01	Distal Intergenic

Crcp	114205	chr12	30149671	30149971	10886	-1.51	1.01E-02	1.91E-01	Intron (NM_053670/114205, intron 2 of 5)
Ipo4	290228	chr15	34310942	34311242	3103	-1.5	1.01E-02	1.91E-01	Exon (NM_001106038/290228, exon 11 of 30)
Ipo7	308939	chr1	174663262	174663562	7323	-1.7	1.01E-02	1.91E-01	Intron (NM_001107545/308939, intron 1 of 24)
Cfap97	306469	chr16	49318100	49318400	0	0.85	1.01E-02	1.91E-01	Promoter (<=1kb)
Ptpn13	498331	chr14	7850281	7850581	13083	0.73	1.01E-02	1.91E-01	Intron (NM_001100789/498331, intron 1 of 47)
Tsc22d2	499624	chr2	148401481	148401781	-206275	1.09	1.01E-02	1.91E-01	Distal Intergenic
Ido1	66029	chr16	72290686	72290986	74360	-1.53	1.01E-02	1.91E-01	Distal Intergenic
Arhgap5	299012	chr6	73349763	73350063	-8049	-1.9	1.02E-02	1.92E-01	Distal Intergenic
Rai1	303188	chr10	46497800	46498100	-13171	-1.13	1.02E-02	1.92E-01	Distal Intergenic
Rab2a	65158	chr5	21706502	21706802	73593	1.82	1.02E-02	1.92E-01	Distal Intergenic
Atp2b1	29598	chr7	41150939	41151239	36242	-1.59	1.02E-02	1.92E-01	Intron (NM_053311/29598, intron 1 of 21)
Il34	498951	chr19	40544880	40545180	-345364	-0.97	1.02E-02	1.92E-01	Distal Intergenic
Tcof1	291571	chr18	56139175	56139475	-23456	1.52	1.02E-02	1.92E-01	Distal Intergenic
Cdc42ep3	313838	chr6	2051865	2052165	-108893	-1.52	1.02E-02	1.92E-01	Distal Intergenic
Ip6k1	50560	chr8	116788113	116788413	-16038	-1.66	1.02E-02	1.92E-01	Distal Intergenic
Klk13	292848	chr1	99675948	99676248	27290	-1.57	1.02E-02	1.92E-01	Distal Intergenic
Cat	24248	chr3	93399964	93400264	11794	-1.48	1.02E-02	1.92E-01	Intron (NM_012520/24248, intron 3 of 12)
Osr1	298878	chr6	35089935	35090235	-228866	-1.61	1.02E-02	1.92E-01	Distal Intergenic
Emg1	312706	chr4	157207875	157208175	22472	-1.52	1.02E-02	1.92E-01	Intron (NM_001012189/362434, intron 1 of 12)
S100pbp	500551	chr5	147369063	147369363	5646	-1.58	1.02E-02	1.92E-01	Intron (NM_001134628/500551, intron 1 of 6)
Sox6	293165	chr1	185863116	185863416	189939	1.52	1.03E-02	1.93E-01	Intron (NM_001024751/293165, intron 2 of 15)
Ugcg	83626	chr5	76427202	76427502	40364	1.44	1.03E-02	1.93E-01	Distal Intergenic
Perp	292949	chr1	14386911	14387211	162519	-1.05	1.03E-02	1.93E-01	Distal Intergenic
Lefty1	498299	chr13	99207910	99208210	-6179	-1.55	1.03E-02	1.93E-01	Distal Intergenic
Prex1	311647	chr3	163230535	163230835	246987	2.23	1.03E-02	1.93E-01	Distal Intergenic
Polr2m	192147	chr8	77988747	77989047	3574	-1.94	1.03E-02	1.93E-01	Intron (NM_183402/192147, intron 2 of 3)
Pcnx1	314288	chr6	105633958	105634258	22502	2.14	1.03E-02	1.93E-01	Intron (NM_001170347/314288, intron 1 of 35)
Map4k3	170920	chr6	3444090	3444390	129	1	1.03E-02	1.93E-01	Promoter (<=1kb)
Vangl1	690366	chr2	204625374	204625674	120	1.12	1.03E-02	1.93E-01	Promoter (<=1kb)
Slc48a1	300191	chr7	139317909	139318209	46211	1.41	1.03E-02	1.93E-01	Distal Intergenic
Cenpt	307805	chr19	37815656	37815956	3768	2.4	1.03E-02	1.93E-01	Intron (NM_001024257/307805, intron 7 of 12)
Tcf4	84382	chr18	65295617	65295917	10297	-1.73	1.03E-02	1.93E-01	Intron (NM_053369/84382, intron 2 of 16)
Ccn4	65154	chr7	107686373	107686673	-8554	2.03	1.03E-02	1.93E-01	Distal Intergenic
Rcsd1	360872	chr13	83942885	83943185	-25884	-1.7	1.03E-02	1.93E-01	Distal Intergenic
Dmac1	298147	chr5	93536169	93536469	-1497023	0.91	1.03E-02	1.93E-01	Distal Intergenic
Ston1	360202	chr6	12363231	12363531	418	0.75	1.03E-02	1.93E-01	Promoter (<=1kb)
Fat1	83720	chr16	50488079	50488379	13337	-1.75	1.03E-02	1.93E-01	Intron (NM_031819/83720, intron 1 of 26)
Camk2d	24246	chr2	230992390	230992690	91264	-1.7	1.03E-02	1.93E-01	Intron (NM_012519/24246, intron 4 of 20)
Sema3b	363142	chr8	116363513	116363813	-2170	0.92	1.03E-02	1.93E-01	Promoter (2-3kb)
Gatad2a	290669	chr16	21178023	21178323	230	1.17	1.03E-02	1.93E-01	Promoter (<=1kb)
Ehmt2	361798	chr20	4576084	4576384	-150	2.02	1.03E-02	1.93E-01	Promoter (<=1kb)
Gpr132	314480	chr6	137658039	137658339	5794	-1.79	1.04E-02	1.93E-01	Intron (NM_001170595/314480, intron 2 of 2)
Eps8l3	295361	chr2	210651140	210651440	-16963	-1.83	1.04E-02	1.93E-01	Distal Intergenic
Mpdz	29365	chr5	99643887	99644187	-77570	1.83	1.04E-02	1.94E-01	Distal Intergenic
Cdk1	54237	chr20	20602868	20603168	26491	-1.53	1.04E-02	1.94E-01	Distal Intergenic
Raly	296301	chr3	150331223	150331523	7999	-1.73	1.04E-02	1.94E-01	Intron (NM_001011958/296301, intron 1 of 8)
Casp6	83584	chr2	235534618	235534918	193244	-1.93	1.04E-02	1.94E-01	Distal Intergenic
Nsmaf	353233	chr5	19546612	19546912	12332	-1.93	1.04E-02	1.94E-01	Intron (NM_181389/353233, intron 1 of 31)
Syncrip	363113	chr8	96123127	96123427	9311	-1.93	1.04E-02	1.94E-01	Intron (NM_001047916/363113, intron 4 of 8)
Slc1a4	305540	chr14	104709049	104709349	-96632	1.3	1.04E-02	1.94E-01	Distal Intergenic
Nfic	29228	chr7	11150545	11150845	-1193	-1.02	1.04E-02	1.94E-01	Promoter (1-2kb)
Rpusd3	362416	chr4	145370264	145370564	428	0.65	1.04E-02	1.94E-01	Promoter (<=1kb)
Usp32	303394	chr10	72435660	72435960	-18590	-1.68	1.04E-02	1.94E-01	Distal Intergenic
Pias2	83422	chr18	73357558	73357858	-20836	-1.58	1.04E-02	1.94E-01	Distal Intergenic
Ndfip1	291609	chr18	31558681	31558981	-15840	-1.84	1.04E-02	1.94E-01	Distal Intergenic
Itgb1bp1	298914	chr6	43202559	43202859	160476	-1	1.04E-02	1.94E-01	Distal Intergenic
Bcl2l13	312682	chr4	153409316	153409616	24112	-1.51	1.05E-02	1.94E-01	Exon (NM_001107885/312682, exon 3 of 7)
Atp6v1g1	298103	chr5	79352334	79352634	-15029	-1.77	1.05E-02	1.94E-01	Distal Intergenic
Psmc4	117262	chr1	84958749	84959049	27487	0.75	1.05E-02	1.94E-01	Distal Intergenic
Ern1	498013	chr10	94668040	94668340	-15382	-1.74	1.05E-02	1.94E-01	Distal Intergenic
Sdc2	25615	chr7	71586359	71586659	13628	-1.74	1.05E-02	1.94E-01	Intron (NM_013082/25615, intron 1 of 4)
Traf3ip2	361857	chr20	44688720	44689020	8271	1.54	1.05E-02	1.94E-01	Intron (NM_001044248/361857, intron 1 of 8)
Gpr84	688730	chr7	144932142	144932442	4361	0.9	1.05E-02	1.94E-01	Downstream (2-3kb)
Plekha3	295674	chr3	63543211	63543511	7045	-2.02	1.05E-02	1.94E-01	Intron (NM_001013077/295674, intron 2 of 7)
Me3	361602	chr1	153870469	153870769	8900	2.18	1.05E-02	1.94E-01	Intron (NM_001108491/361602, intron 2 of 14)
Skiv2l	294260	chr20	4526639	4526939	3179	2.39	1.05E-02	1.94E-01	Exon (NM_213559/294260, exon 10 of 28)
Ccl5	81780	chr10	70754132	70754432	-9829	-1.56	1.05E-02	1.94E-01	Distal Intergenic
Msln	60333	chr10	15128196	15128496	-2808	1.27	1.05E-02	1.94E-01	Promoter (2-3kb)
Ikzf2	301476	chr9	76682152	76682452	86354	1.63	1.05E-02	1.94E-01	Intron (NM_001106916/301476, intron 3 of 7)
R3hdm1	304763	chr13	44840754	44841054	28187	-1.76	1.05E-02	1.94E-01	Intron (NM_001134867/304763, intron 1 of 26)
Ccpg1	363098	chr8	79664762	79665062	4105	-1.76	1.05E-02	1.94E-01	Intron (NM_001108770/363098, intron 1 of 8)
RGD1561149	500552	chr5	147423100	147423400	-10395	1.23	1.05E-02	1.94E-01	Distal Intergenic
Capn13	362701	chr6	24559299	24559599	116819	1.89	1.05E-02	1.94E-01	Distal Intergenic
Cenpj	305909	chr15	36851153	36851453	-45545	-1.74	1.05E-02	1.94E-01	Distal Intergenic
Elf3	304815	chr13	52089009	52089309	-316	-1.64	1.05E-02	1.94E-01	Promoter (<=1kb)
Mical3	362427	chr4	153636198	153636498	-4212	2.4	1.05E-02	1.94E-01	Distal Intergenic
Lgals3	83781	chr15	24156940	24157240	3338	-1.58	1.05E-02	1.94E-01	Intron (NM_031832/83781, intron 1 of 5)
Arhgap11a	296060	chr3	105293048	105293348	4760	-1.64	1.05E-02	1.94E-01	Intron (NM_001168524/296060, intron 2 of 11)
Wdr6	301007	chr8	117380933	117381233	-16172	-1.98	1.05E-02	1.94E-01	Distal Intergenic
Mbnl1	282635	chr2	150801227	150801527	44969	-1.63	1.06E-02	1.94E-01	Intron (NM_001191566/282635, intron 1 of 7)
Igf1r	25718	chr1	129118716	129119016	193795	2.09	1.06E-02	1.94E-01	Intron (NM_052807/25718, intron 2 of 20)
Ncl	25135	chr9	93372510	93372810	4833	-1.73	1.06E-02	1.94E-01	Intron (NM_012749/25135, intron 7 of 13)
Zfp709l1	690419	chr7	10612455	10612755	-175738	-1.03	1.06E-02	1.95E-01	Distal Intergenic
Wdr19	305349	chr14	44743469	44743769	23351	1.83	1.06E-02	1.95E-01	Exon (NM_001191679/305349, exon 7 of 36)
Fibp	282837	chr1	220836569	220836869	-3270	0.71	1.06E-02	1.95E-01	Distal Intergenic
Nsun7	305339	chr14	43415162	43415462	168858	-1.66	1.06E-02	1.95E-01	Distal Intergenic
Ncoa6	116464	chr3	150989826	150990126	-29796	1.55	1.06E-02	1.95E-01	Distal Intergenic
Pard6b	362279	chr3	164822134	164822434	23	1.17	1.06E-02	1.95E-01	Promoter (<=1kb)
Dcaf6	289181	chr13	83661286	83661586	18744	-1.75	1.06E-02	1.95E-01	Intron (NM_001305159/289181, intron 1 of 18)
Gpx5	113919	chr17	45553783	45554083	45126	-1.53	1.06E-02	1.95E-01	Distal Intergenic
Carm1	363026	chr8	22577931	22578231	208	1.01	1.07E-02	1.95E-01	Promoter (<=1kb)
Edem3	289085	chr13	69068545	69068845	-66684	1.07	1.07E-02	1.95E-01	Intron (NM_022242/63912, intron 5 of 13)
Klf13	499171	chr1	124794817	124795117	8246	-1.65	1.07E-02	1.95E-01	Intron (NM_001109147/499171, intron 1 of 1)
Sipa1l1	246212	chr6	105903891	105904191	-148021	-1.67	1.07E-02	1.95E-01	Distal Intergenic

Gnb1	24400	chr5	172931667	172931967	-3023	-1.8	1.07E-02	1.95E-01	Distal Intergenic
Ppm1a	24666	chr6	94439108	94439408	5447	-1.75	1.07E-02	1.95E-01	Intron (NM_017038/24666, intron 1 of 5)
Btg1	29618	chr7	37889112	37889412	76281	-1.75	1.07E-02	1.95E-01	Distal Intergenic
Pkp2	287925	chr11	88931822	88932122	40091	-1.4	1.07E-02	1.95E-01	Intron (NM_001100499/287925, intron 4 of 10)
Hsf2	64441	chr20	38435588	38435888	-499932	-1.5	1.07E-02	1.95E-01	Distal Intergenic
Mir147	100314100	chr3	114798010	114798310	22667	-1.68	1.07E-02	1.95E-01	Distal Intergenic
Vcl	305679	chr15	3523163	3523463	21222	-1.8	1.07E-02	1.95E-01	Intron (NM_001107248/305679, intron 1 of 20)
Gng5	79218	chr2	252367696	252367996	7720	-1.69	1.07E-02	1.96E-01	3' UTR
Pid1	501174	chr9	92037371	92037671	-353903	-1.68	1.07E-02	1.96E-01	Distal Intergenic
Fam13a	362378	chr4	89761558	89761858	-480336	-1.35	1.07E-02	1.96E-01	Distal Intergenic
Mgll	29254	chr4	120696040	120696340	24551	1.76	1.07E-02	1.96E-01	Intron (NM_138502/29254, intron 2 of 7)
Ern1	498013	chr10	94666489	94666789	-13831	-1.45	1.07E-02	1.96E-01	Distal Intergenic
Riok2	308201	chr1	59126335	59126635	-3373	2.07	1.07E-02	1.96E-01	Distal Intergenic
Tgfb3	25717	chr6	109829992	109830292	105241	-1.72	1.08E-02	1.96E-01	Distal Intergenic
Brd4	362844	chr7	14296012	14296312	6743	-1.72	1.08E-02	1.96E-01	Intron (NM_001100903/362844, intron 1 of 18)
Ldhal6b	369018	chr1	45880931	45881231	428045	1.18	1.08E-02	1.96E-01	Distal Intergenic
Mfsd1	361957	chr2	164716398	164716698	-30979	2.04	1.08E-02	1.96E-01	Distal Intergenic
Ttc19	691506	chr10	48599224	48599524	0	1.5	1.08E-02	1.96E-01	Promoter (<=1kb)
Pou1f1	25517	chr11	3153330	3153630	507465	1.2	1.08E-02	1.96E-01	Distal Intergenic
Ccni	289500	chr14	16382058	16382358	40522	-1.89	1.08E-02	1.96E-01	Exon (NM_001107208/305227, exon 7 of 11)
Serpine2	29366	chr9	85598499	85598799	27295	-1.89	1.08E-02	1.96E-01	Intron (NM_019197/29366, intron 1 of 8)
Fam76b	367021	chr8	12541509	12541809	185742	-1.37	1.08E-02	1.97E-01	Distal Intergenic
Hand1	59112	chr10	43216451	43216751	36545	-1.6	1.08E-02	1.97E-01	Distal Intergenic
Mdfi	501097	chr9	15167416	15167716	1298	0.87	1.08E-02	1.97E-01	Promoter (1-2kb)
Slc22a17	305886	chr15	33575929	33576229	102	1.3	1.08E-02	1.97E-01	Promoter (<=1kb)
Gfra4	66023	chr3	123597417	123597717	-15899	-1.73	1.08E-02	1.97E-01	Distal Intergenic
Unc50	192356	chr9	44025002	44025302	0	0.71	1.08E-02	1.97E-01	Promoter (<=1kb)
Bcl10	83477	chr2	251810008	251810308	4616	-1.7	1.08E-02	1.97E-01	Intron (NM_031328/83477, intron 1 of 2)
Dusp7	300980	chr8	115093771	115094071	24676	-1.76	1.08E-02	1.97E-01	Distal Intergenic
Atp11a	306600	chr16	82095100	82095400	4822	-1.73	1.09E-02	1.97E-01	Intron (NM_001107324/306600, intron 1 of 28)
Errfi1	313729	chr5	167956365	167956665	3637	-1.73	1.09E-02	1.97E-01	Intron (NM_001014071/313729, intron 1 of 3)
Ss18	361295	chr18	6036704	6037004	46029	-1.17	1.09E-02	1.97E-01	Intron (NM_001100900/361295, intron 5 of 10)
Zfp64	311661	chr3	165790464	165790764	-48532	1.85	1.09E-02	1.97E-01	Distal Intergenic
Ube2n	116725	chr7	36610644	36610944	497	0.89	1.09E-02	1.97E-01	Promoter (<=1kb)
Cemip2	309400	chr1	239452656	239452956	39772	-1.58	1.09E-02	1.97E-01	Intron (NM_001107596/309400, intron 15 of 21)
Hepacam2	296846	chr4	28349118	28349418	88246	-1.08	1.09E-02	1.97E-01	Distal Intergenic
Wac	307029	chr17	60790942	60791242	155388	2.31	1.09E-02	1.97E-01	Distal Intergenic
Fas	246097	chr1	252589893	252590193	108	-0.89	1.09E-02	1.97E-01	Promoter (<=1kb)
Kif1c	113886	chr10	57322508	57322808	177	0.72	1.09E-02	1.97E-01	Promoter (<=1kb)
Spop	287643	chr10	83217747	83218047	-13362	-1.56	1.09E-02	1.97E-01	Distal Intergenic
Dock1	309081	chr1	206896686	206896986	-3631	2.05	1.09E-02	1.97E-01	Distal Intergenic
LOC497899	497899	chr10	36719458	36719758	-2857	-1.19	1.09E-02	1.97E-01	Promoter (2-3kb)
Osbpl8	314824	chr7	53878630	53878930	0	0.78	1.09E-02	1.97E-01	Promoter (<=1kb)
Edem3	289085	chr13	69043125	69043425	-92104	-1.53	1.09E-02	1.97E-01	Intron (NM_022242/63912, intron 5 of 13)
Sipa1l1	246212	chr6	106085897	106086197	33685	-1.6	1.09E-02	1.97E-01	Intron (NM_139330/246212, intron 5 of 20)
Tmem229b	503035	chr6	102157716	102158016	38122	-1.8	1.09E-02	1.97E-01	Intron (NM_001109359/503035, intron 2 of 2)
Adgrl2	171447	chr2	258928688	258928988	3135	-1.8	1.09E-02	1.97E-01	Intron (NM_001271361/365984, intron 2 of 4)
Zcchc24	361104	chr16	2016345	2016645	31647	2.02	1.09E-02	1.97E-01	Intron (NM_001108394/361104, intron 2 of 3)
Psmd13	365388	chr1	213636050	213636350	0	0.73	1.09E-02	1.97E-01	Promoter (<=1kb)
Chst12	304322	chr12	16246786	16247086	-45158	1.3	1.09E-02	1.97E-01	Distal Intergenic
Pid1	501174	chr9	91867881	91868181	-184413	-1.72	1.10E-02	1.97E-01	Distal Intergenic
Slc44a3	295417	chr2	225136812	225137112	-23953	1.33	1.10E-02	1.98E-01	Distal Intergenic
Lrp11	292462	chr1	1665046	1665346	-37350	1.55	1.10E-02	1.98E-01	Distal Intergenic
Nostrin	311111	chr3	55209330	55209630	-159652	-1.64	1.10E-02	1.98E-01	Distal Intergenic
Tbl1xr1	365755	chr2	106907694	106907994	-325060	-1.38	1.10E-02	1.98E-01	Distal Intergenic
Tfcp2l1	304741	chr13	34648514	34648814	37825	2.04	1.10E-02	1.98E-01	Intron (NM_001107170/304741, intron 7 of 15)
Ldhal6b	369018	chr1	45883177	45883477	425799	0.9	1.10E-02	1.98E-01	Distal Intergenic
Cxcl12	24772	chr4	149261289	149261589	245	0.73	1.10E-02	1.98E-01	Promoter (<=1kb)
Fgfr2	25022	chr1	200645523	200645823	51105	2.15	1.10E-02	1.98E-01	Intron (NM_001109892/25022, intron 6 of 17)
Rin2	311494	chr3	139953115	139953415	58784	-1.55	1.10E-02	1.98E-01	Intron (NM_001107786/311494, intron 2 of 12)
Smug1	315344	chr7	144778183	144778483	170	-1.59	1.10E-02	1.98E-01	Promoter (<=1kb)
Fgfbp3	499349	chr1	255564227	255564527	-7172	0.92	1.10E-02	1.98E-01	Distal Intergenic
Tnpo1	309126	chr2	29120768	29121068	36	1.18	1.11E-02	1.98E-01	Promoter (<=1kb)
Tubb4a	29213	chr9	9949651	9949951	-11069	0.95	1.11E-02	1.98E-01	Distal Intergenic
Wwtr1	295062	chr2	147674232	147674532	18501	-1.55	1.11E-02	1.98E-01	Intron (NM_001024869/295062, intron 2 of 6)
Adam9	290834	chr16	71831395	71831695	21018	-1.85	1.11E-02	1.98E-01	Exon (NM_001014772/290834, exon 7 of 23)
Rfc2	116468	chr12	25140657	25140957	2523	-1.81	1.11E-02	1.99E-01	Promoter (2-3kb)
Chmp6	287873	chr10	109018106	109018406	-55272	1.29	1.11E-02	1.99E-01	Exon (NM_001134499/287871, exon 19 of 34)
Scrib	362938	chr7	117128440	117128740	58	1.24	1.11E-02	1.99E-01	Promoter (<=1kb)
Cpn1	365466	chr1	263715196	263715496	47262	-1.53	1.11E-02	1.99E-01	Distal Intergenic
Mir3120	100526611	chr13	80184471	80184771	-53446	-1.26	1.11E-02	1.99E-01	Intron (NM_138538/171574, intron 12 of 20)
Enthd1	685900	chr7	121707734	121708034	75401	2.35	1.11E-02	1.99E-01	Intron (NM_001135913/685900, intron 5 of 6)
Smoc1	314280	chr6	104732842	104733142	14165	2.17	1.11E-02	1.99E-01	Intron (NM_001002835/314280, intron 1 of 12)
Kif13a	308173	chr17	18207106	18207406	55124	-1.48	1.11E-02	1.99E-01	Intron (NM_001107462/308173, intron 1 of 36)
Hic1	303310	chr10	61987560	61987860	22914	2.34	1.11E-02	1.99E-01	Intron (NM_001105808/287522, intron 15 of 18)
Vom2r1	678740	chr10	112598637	112598937	-1031963	0.77	1.11E-02	1.99E-01	Distal Intergenic
Creb3l1	362165	chr3	81077082	81077382	-143799	2.01	1.11E-02	1.99E-01	Distal Intergenic
Tnfrsf9	500590	chr5	168019374	168019674	9981	-1.13	1.11E-02	1.99E-01	Intron (NM_001025773/500590, intron 1 of 7)
Sp8	299499	chr6	146944187	146944487	159272	-1.57	1.12E-02	1.99E-01	Distal Intergenic
Foxk2	303753	chr10	110469256	110469556	0	1.02	1.12E-02	1.99E-01	Promoter (<=1kb)
Zfp266	367034	chr8	21566235	21566535	-23168	-1.41	1.12E-02	1.99E-01	Distal Intergenic
Atp11a	306600	chr16	82081003	82081303	18919	-1.83	1.12E-02	1.99E-01	Intron (NM_001107324/306600, intron 1 of 28)
Smad3	25631	chr8	68650403	68650703	27646	-1.82	1.12E-02	1.99E-01	Intron (NM_013095/25631, intron 1 of 8)
Lect2	361205	chr17	8470808	8471108	-18153	-1.48	1.12E-02	1.99E-01	Distal Intergenic
Stk17b	170904	chr9	60413070	60413370	16711	-1.48	1.12E-02	1.99E-01	Intron (NM_133392/170904, intron 3 of 7)
Smim14	364154	chr14	44433401	44433701	19765	-1.26	1.12E-02	1.99E-01	Intron (NM_001037792/364154, intron 1 of 4)
Ctsl	25697	chr17	1873474	1873774	5489	-1.67	1.12E-02	1.99E-01	Exon (NM_013156/25697, exon 8 of 8)
Snrpa1	100361269	chr1	126970070	126970370	8681	-1.95	1.12E-02	1.99E-01	Intron (NM_001271323/100361269, intron 5 of 8)
Pc	25104	chr1	219791523	219791823	32340	-1.38	1.12E-02	2.00E-01	Intron (NM_012744/25104, intron 1 of 19)
Hadha	170670	chr6	27598310	27598610	8470	-1.46	1.12E-02	2.00E-01	Intron (NM_130826/170670, intron 1 of 19)
Tmie	501061	chr8	119144966	119145266	11805	1.77	1.12E-02	2.00E-01	Intron (NM_001109299/501061, intron 4 of 5)
Slc25a44	365841	chr2	187781565	187781865	4847	-1.77	1.12E-02	2.00E-01	Intron (NM_001108947/365841, intron 2 of 3)
Sptbn1	305614	chr14	114688881	114689181	3583	-1.9	1.13E-02	2.00E-01	Intron (NM_001013130/305614, intron 1 of 36)
Cblb	171136	chr11	51192107	51192407	10296	-1.64	1.13E-02	2.00E-01	Intron (NM_133601/171136, intron 2 of 17)

Rab31	246324	chr9	113492077	113492377	12229	-1.15	1.13E-02	2.00E-01	Intron (NM_145094/246324, intron 1 of 6)
Cldn7	65132	chr10	56573132	56573432	-3010	-1.42	1.13E-02	2.00E-01	Distal Intergenic
Dclk1	83825	chr2	144846368	144846668	-14777	-1.44	1.13E-02	2.00E-01	Distal Intergenic
Klhl40	316088	chr8	130398825	130399125	-17230	1.22	1.13E-02	2.00E-01	Distal Intergenic
Mrvi1	308899	chr1	175766443	175766743	-4149	-1.22	1.13E-02	2.00E-01	Intron (NM_001105210/308899, intron 1 of 20)
Ldlrad4	679578	chr18	63785891	63786191	-57808	-1.46	1.13E-02	2.00E-01	Distal Intergenic
RGD1565355	499985	chr4	14303583	14303883	90020	2.01	1.13E-02	2.00E-01	Distal Intergenic
Cnot1	291841	chr19	9701969	9702269	33783	1.7	1.13E-02	2.00E-01	Intron (NM_001134840/291841, intron 7 of 48)
Rab36	690407	chr20	14546416	14546716	26803	1.84	1.13E-02	2.01E-01	Distal Intergenic
Rhog	308875	chr1	167340779	167341079	6584	-1.61	1.13E-02	2.01E-01	Intron (NM_001037195/308875, intron 1 of 1)
Padi6	298595	chr5	159135328	159135628	121071	1.86	1.13E-02	2.01E-01	Distal Intergenic
Eif2ak3	29702	chr4	98648714	98649014	169	0.98	1.13E-02	2.01E-01	Promoter (<=1kb)
Kcnf1	298908	chr6	42473185	42473485	253	0.7	1.14E-02	2.01E-01	Promoter (<=1kb)
Diaph1	307483	chr18	31056633	31056933	14438	-1.75	1.14E-02	2.01E-01	Intron (NM_001107393/307483, intron 1 of 29)
Cdh1	83502	chr19	38800868	38801168	32401	1.79	1.14E-02	2.01E-01	Intron (NM_031334/83502, intron 2 of 15)
Ptgs1	24693	chr3	15588310	15588610	27587	-1.38	1.14E-02	2.01E-01	Distal Intergenic
Stk32a	364858	chr18	37510108	37510408	-86897	2.11	1.14E-02	2.01E-01	Distal Intergenic
Tex29	498664	chr16	82901318	82901618	98209	2.12	1.14E-02	2.01E-01	Distal Intergenic
Hsd17b12	84013	chr3	83014948	83015248	33041	-1.74	1.14E-02	2.01E-01	Intron (NM_032066/84013, intron 1 of 10)
C1ql2	288979	chr13	36402317	36402617	24130	1.56	1.14E-02	2.01E-01	Distal Intergenic
Bicd2	306809	chr17	15694327	15694627	23408	-1.6	1.14E-02	2.02E-01	Intron (NM_001033674/306809, intron 1 of 6)
Efna5	116683	chr9	110138091	110138391	191296	-2.13	1.14E-02	2.02E-01	Intron (NM_053903/116683, intron 1 of 4)
Tuba1a	64158	chr7	140640525	140640825	128	0.82	1.14E-02	2.02E-01	Promoter (<=1kb)
Bnc2	298189	chr5	102806950	102807250	-63533	0.88	1.14E-02	2.02E-01	Distal Intergenic
Fam98a	313873	chr6	20253489	20253789	-797538	2.18	1.14E-02	2.02E-01	Distal Intergenic
Srgap3	500287	chr4	144830151	144830451	38674	1.77	1.14E-02	2.02E-01	Intron (NM_001191975/500287, intron 1 of 21)
Slc11a2	25715	chr7	142055328	142055628	7242	-1.61	1.14E-02	2.02E-01	Intron (NM_013173/25715, intron 1 of 15)
Rad18	362412	chr4	144686563	144686863	-65979	1.33	1.15E-02	2.02E-01	Intron (NM_001191975/500287, intron 9 of 21)
Runx1	50662	chr11	32836018	32836318	22507	-1.61	1.15E-02	2.02E-01	Intron (NM_017325/50662, intron 2 of 5)
Rem1	366232	chr3	148108525	148108825	0	0.9	1.15E-02	2.02E-01	Promoter (<=1kb)
Dynlrb1	170714	chr3	150765768	150766068	4464	-1.64	1.15E-02	2.02E-01	Intron (NM_131910/170714, intron 1 of 3)
Rpp14	361020	chr15	18644920	18645220	-8787	1.03	1.15E-02	2.02E-01	Downstream (2-3kb)
Slc38a4	170573	chr7	138387164	138387464	125488	0.74	1.15E-02	2.02E-01	Distal Intergenic
Olfml2a	296708	chr3	23223432	23223732	-8223	0.71	1.15E-02	2.02E-01	Distal Intergenic
Ptcd2	310025	chr2	29396772	29397072	201181	1.04	1.15E-02	2.02E-01	Distal Intergenic
Sik3	684112	chr8	50324718	50325018	14455	-1.71	1.15E-02	2.02E-01	Intron (NM_001271216/684112, intron 1 of 24)
Fus	317385	chr1	199417925	199418225	5091	-1.77	1.15E-02	2.02E-01	Intron (NM_001012137/317385, intron 6 of 14)
Nfix	81524	chr19	25898890	25899190	15506	0.71	1.15E-02	2.02E-01	Intron (NM_030866/81524, intron 1 of 9)
Bend6	363212	chr9	38126517	38126817	-170436	0.81	1.15E-02	2.02E-01	Distal Intergenic
Clic4	83718	chr5	153617392	153617692	7977	-1.16	1.15E-02	2.02E-01	Intron (NM_031818/83718, intron 1 of 5)
Erg28	299207	chr6	109688449	109688749	3469	-1.65	1.15E-02	2.02E-01	Intron (NM_001106749/299207, intron 3 of 5)
Mir32	100314013	chr5	73938964	73939264	-4640	2.11	1.15E-02	2.02E-01	Distal Intergenic
Lhfpl2	294643	chr2	23612622	23612922	-157786	-1.72	1.15E-02	2.02E-01	Distal Intergenic
Lysmd3	315923	chr2	9374965	9375265	-150948	-1.48	1.15E-02	2.02E-01	Distal Intergenic
Endog	362100	chr3	8739200	8739500	-2333	1.92	1.15E-02	2.02E-01	Promoter (2-3kb)
RGD1311345	361201	chr17	6679260	6679560	-3592	-1.63	1.15E-02	2.02E-01	Distal Intergenic
Thy1	24832	chr8	48326336	48326636	-55485	-1.54	1.16E-02	2.02E-01	Distal Intergenic
Usp20	311856	chr3	9822611	9822911	25	0.62	1.16E-02	2.02E-01	Promoter (<=1kb)
Gata6	29300	chr18	2715692	2715992	299871	-1.5	1.16E-02	2.02E-01	Distal Intergenic
Gpr88	64443	chr2	219177630	219177930	84971	-1.7	1.16E-02	2.02E-01	Distal Intergenic
Ptcd2	310025	chr2	29388049	29388349	209904	1.88	1.16E-02	2.02E-01	Distal Intergenic
P4ha2	360526	chr10	39440605	39440905	5378	-1.58	1.16E-02	2.02E-01	Intron (NM_001108275/360526, intron 2 of 15)
Matr3	29150	chr18	28366366	28366666	5083	-1.66	1.16E-02	2.02E-01	Intron (NM_019149/29150, intron 1 of 14)
Atp11a	306600	chr16	82118473	82118773	-18251	-1.41	1.16E-02	2.02E-01	Distal Intergenic
Anxa6	79125	chr10	40359593	40359893	15712	1.38	1.16E-02	2.02E-01	Intron (NM_024156/79125, intron 2 of 25)
Lactb	300803	chr8	72800260	72800560	-33953	-1.23	1.16E-02	2.02E-01	Distal Intergenic
Plekha5	246237	chr4	174608386	174608686	2924	-1.5	1.16E-02	2.03E-01	Promoter (2-3kb)
Armcx3	367902	chrX	105575991	105576291	2082	-1.83	1.16E-02	2.03E-01	Promoter (2-3kb)
Cnot1	291841	chr19	9701108	9701408	32922	2.14	1.16E-02	2.03E-01	Intron (NM_001134840/291841, intron 6 of 48)
Ldlrad4	679578	chr18	63781660	63781960	-62039	-1.81	1.16E-02	2.03E-01	Distal Intergenic
Dcaf15	304653	chr19	25240380	25240680	-20577	0.68	1.16E-02	2.03E-01	Distal Intergenic
Mycbp	100361133	chr5	141560268	141560568	76	1.31	1.16E-02	2.03E-01	Promoter (<=1kb)
Crispld1	316482	chr5	18133	18433	692600	-1.5	1.16E-02	2.03E-01	Distal Intergenic
Nxt1	296219	chr3	142993682	142993982	24	0.63	1.16E-02	2.03E-01	Promoter (<=1kb)
Col8a1	304021	chr11	44885773	44886073	7855	-1.61	1.17E-02	2.03E-01	Intron (NM_001107100/304021, intron 1 of 3)
Bcar1	25414	chr19	43945447	43945747	10036	-1.52	1.17E-02	2.03E-01	Exon (NM_012931/25414, exon 2 of 7)
Bc1	29294	chr1	218068880	218069180	-6179	1.7	1.17E-02	2.03E-01	Distal Intergenic
Gsta6	501110	chr9	27533112	27533412	-21936	-1.12	1.17E-02	2.03E-01	Distal Intergenic
Pik3r1	25513	chr2	31453628	31453928	362457	-1.56	1.17E-02	2.03E-01	Distal Intergenic
Nt5m	287368	chr10	46238528	46238828	21588	-1.64	1.17E-02	2.03E-01	Intron (NM_001105785/287368, intron 3 of 4)
Msc	312897	chr5	4366466	4366766	411239	0.82	1.17E-02	2.03E-01	Distal Intergenic
Tgm2	56083	chr3	154602475	154602775	24482	-1.5	1.17E-02	2.04E-01	Intron (NM_019386/56083, intron 10 of 12)
Smoc2	292401	chr1	56304475	56304775	62129	-1.6	1.17E-02	2.04E-01	Intron (NM_001106215/292401, intron 4 of 12)
Sptb	314251	chr6	99671860	99672160	110909	1.16	1.17E-02	2.04E-01	Downstream (2-3kb)
Ncald	553106	chr7	76377171	76377471	-82508	-1.99	1.17E-02	2.04E-01	Distal Intergenic
Hoxc4	24459	chr7	144708000	144708300	60413	-1.99	1.17E-02	2.04E-01	Distal Intergenic
Prdm1	309871	chr20	49557580	49557880	-71035	0.91	1.17E-02	2.04E-01	Distal Intergenic
Tbl1xr1	365755	chr2	106908354	106908654	-324400	-1.66	1.17E-02	2.04E-01	Distal Intergenic
Mcam	78967	chr8	48473035	48473335	211	0.69	1.18E-02	2.04E-01	Promoter (<=1kb)
Il1rn	60582	chr3	1460748	1461048	-2160	-1.61	1.18E-02	2.04E-01	Promoter (2-3kb)
Atg14	305831	chr15	24471080	24471380	-96304	-1.55	1.18E-02	2.04E-01	Distal Intergenic
Dpysl3	25418	chr18	37743473	37743773	32680	-1.54	1.18E-02	2.04E-01	Intron (NM_012934/25418, intron 3 of 13)
Aqp1	25240	chr4	85484632	85484932	-66571	-1.44	1.18E-02	2.04E-01	Distal Intergenic
Cpvl	502774	chr4	84193970	84194270	-62940	0.91	1.18E-02	2.04E-01	Distal Intergenic
Tnip1	363599	chr10	40290240	40290540	12532	-1.63	1.18E-02	2.04E-01	Intron (NM_001108826/363599, intron 2 of 18)
Scara5	305974	chr15	49118854	49119154	49674	2.29	1.18E-02	2.04E-01	Intron (NM_001135855/305974, intron 3 of 8)
Exd2	362759	chr6	103998908	103999208	-18681	-1.56	1.18E-02	2.04E-01	Distal Intergenic
Pik3r1	25513	chr2	32289458	32289758	-473073	-1.24	1.18E-02	2.04E-01	Distal Intergenic
Shoc2	309548	chr1	274663746	274664046	63	1.35	1.18E-02	2.04E-01	Promoter (<=1kb)
Cox6a2	25278	chr1	199624319	199624619	164	-1.37	1.18E-02	2.04E-01	Promoter (<=1kb)
Mmadhc	362134	chr3	35827275	35827575	-25868	-1.89	1.18E-02	2.04E-01	Distal Intergenic
Pik3cb	85243	chr8	107375354	107375654	-27781	2.3	1.18E-02	2.04E-01	Distal Intergenic
Zic4	315882	chr8	98852450	98852750	97346	2.17	1.18E-02	2.04E-01	Distal Intergenic
Topors	362501	chr5	56564988	56565288	1215	0.75	1.18E-02	2.04E-01	Promoter (1-2kb)

Ksr1	360573	chr10	66406802	66407102	75048	2.37	1.18E-02	2.04E-01	5' UTR
Lhfpl6	499615	chr2	142285202	142285502	22966	1.05	1.19E-02	2.04E-01	Intron (NM_001109183/499615, intron 2 of 3)
Mypop	499090	chr1	79902151	79902451	3600	-1.84	1.19E-02	2.04E-01	Intron (NM_001109139/499090, intron 2 of 3)
Rptor	287871	chr10	108728232	108728532	-22088	0.82	1.19E-02	2.04E-01	Distal Intergenic
Ptpn12	117255	chr4	10641705	10642005	10576	-1.61	1.19E-02	2.05E-01	Intron (NM_057115/117255, intron 1 of 17)
Acat2	308100	chr1	47967913	47968213	-4186	1.55	1.19E-02	2.05E-01	Distal Intergenic
Tcf7l1	312451	chr4	100717036	100717336	-56894	1.86	1.19E-02	2.05E-01	Distal Intergenic
Dpp8	315758	chr8	70592332	70592632	70235	0.87	1.19E-02	2.05E-01	Distal Intergenic
Lmo7	361084	chr15	86310630	86310930	67482	-1.83	1.19E-02	2.05E-01	Intron (NM_001001515/361084, intron 1 of 30)
Rab28	117049	chr14	73908816	73909116	19479	2.13	1.19E-02	2.05E-01	Intron (NM_053978/117049, intron 3 of 6)
Ube2f	363284	chr9	98413928	98414228	19760	2.24	1.19E-02	2.05E-01	Intron (NM_001008381/363284, intron 3 of 8)
Rab21	299799	chr7	58279299	58279599	7171	-1.65	1.19E-02	2.05E-01	Intron (NM_001004238/299799, intron 1 of 6)
Klhl9	313348	chr5	107323304	107323604	46	0.66	1.19E-02	2.05E-01	Promoter (<=1kb)
H3f3b	117056	chr10	104586763	104587063	-10876	-1.64	1.19E-02	2.05E-01	Distal Intergenic
LOC102550367	102550367	chr3	120910787	120911087	172149	-1.23	1.19E-02	2.05E-01	Distal Intergenic
Upp1	289801	chr14	89318806	89319106	3117	-1.61	1.19E-02	2.05E-01	Intron (NM_001030025/289801, intron 1 of 7)
Lpar1	116744	chr5	75587663	75587963	88621	1.51	1.19E-02	2.05E-01	Intron (NM_053936/116744, intron 3 of 3)
Miat	102552664	chr12	50566858	50567158	119607	1.51	1.19E-02	2.05E-01	Distal Intergenic
Exoc4	116654	chr4	60867072	60867372	317875	-1.64	1.20E-02	2.05E-01	Intron (NM_053875/116654, intron 14 of 22)
Edn1	24323	chr17	22143426	22143726	-102	0.6	1.20E-02	2.05E-01	Promoter (<=1kb)
Dnai1	500442	chr5	57947777	57948077	0	-1.34	1.20E-02	2.05E-01	Promoter (<=1kb)
C1d	289810	chr14	101042301	101042601	626656	1.51	1.20E-02	2.05E-01	Distal Intergenic
Isca1	290985	chr17	5230681	5230981	-50746	-1.78	1.20E-02	2.05E-01	Distal Intergenic
Twsg1	363294	chr9	113769139	113769439	67663	-1.89	1.20E-02	2.05E-01	Distal Intergenic
Prag1	306506	chr16	59646040	59646340	74906	-1.69	1.20E-02	2.05E-01	Distal Intergenic
Sparc	24791	chr10	40745075	40745375	18766	-1.21	1.20E-02	2.05E-01	Intron (NM_012656/24791, intron 8 of 9)
Casp3	25402	chr16	48829053	48829353	33851	-1.28	1.20E-02	2.05E-01	Distal Intergenic
Ldhal6b	369018	chr1	46290460	46290760	18516	-1.69	1.20E-02	2.05E-01	Distal Intergenic
Accsl	690470	chr3	82810173	82810473	-32359	-1.69	1.20E-02	2.05E-01	Distal Intergenic
Mycn	298894	chr6	38327416	38327716	-99037	1.86	1.20E-02	2.05E-01	Distal Intergenic
Kctd10	494521	chr12	48005517	48005817	13360	2.2	1.20E-02	2.05E-01	Exon (NM_001009973/494521, exon 5 of 7)
Pold3	293144	chr1	165113924	165114224	8161	-1.39	1.20E-02	2.05E-01	Intron (NM_001024750/293144, intron 2 of 11)
Zswim3	311630	chr3	161260171	161260471	-11914	2.14	1.20E-02	2.05E-01	Downstream (<1kb)
Palm2	103692368	chr5	74735858	74736158	86093	-1.46	1.20E-02	2.05E-01	Intron (NM_001305995/103692368, intron 3 of 5)
Arnt2	25243	chr1	146483282	146483582	72561	-1.57	1.20E-02	2.05E-01	Intron (NM_012781/25243, intron 5 of 18)
Nt5dc3	691922	chr7	27310118	27310418	152	1.11	1.20E-02	2.05E-01	Promoter (<=1kb)
RGD1306746	312511	chr4	118279057	118279357	35925	-1.52	1.20E-02	2.05E-01	Distal Intergenic
Cdk13	306998	chr17	49833675	49833975	481	1.47	1.21E-02	2.06E-01	Promoter (<=1kb)
Ccdc82	300359	chr8	12008616	12008916	120096	-1.61	1.21E-02	2.06E-01	Distal Intergenic
Alox5ap	29624	chr12	6832305	6832605	46507	1.17	1.21E-02	2.06E-01	Distal Intergenic
Nobox	502759	chr4	72715436	72715736	41151	-1.57	1.21E-02	2.06E-01	Distal Intergenic
Cst7	296257	chr3	145751976	145752276	-612816	0.81	1.21E-02	2.06E-01	Distal Intergenic
Pard3b	301455	chr9	68690845	68691145	276506	1.3	1.21E-02	2.06E-01	Intron (NM_001191808/301455, intron 2 of 22)
Tars3	308701	chr1	126391455	126391755	-131414	-1.59	1.21E-02	2.06E-01	Distal Intergenic
Ywhaq	25577	chr6	43477134	43477434	16382	-1.67	1.21E-02	2.06E-01	Intron (NM_013053/25577, intron 2 of 5)
Nrp2	81527	chr9	69534273	69534573	36839	-1.52	1.21E-02	2.06E-01	Intron (NM_030869/81527, intron 3 of 16)
Antxr1	362393	chr4	119051883	119052183	79019	1.61	1.21E-02	2.06E-01	Intron (NM_001044249/362393, intron 10 of 17)
Kif3b	296284	chr3	148829102	148829402	55843	1.27	1.21E-02	2.06E-01	Distal Intergenic
Larp6	315731	chr8	65637198	65637498	25628	1.95	1.21E-02	2.06E-01	Distal Intergenic
Galnt7	29750	chr16	35923381	35923681	-11378	-1.29	1.22E-02	2.06E-01	Distal Intergenic
Fads2	83512	chr1	226187502	226187802	-34934	-1.73	1.22E-02	2.06E-01	Distal Intergenic
Cryba1	25583	chr10	65187705	65188005	26553	-1.73	1.22E-02	2.06E-01	Distal Intergenic
Tiam1	304109	chr11	30117978	30118278	-56805	0.95	1.22E-02	2.06E-01	Distal Intergenic
Cd24	25145	chr20	48335580	48335880	40	1.02	1.22E-02	2.07E-01	Promoter (<=1kb)
Dusp6	116663	chr7	41658716	41659016	183553	-1.24	1.22E-02	2.07E-01	Distal Intergenic
Ppp2r2a	117104	chr15	43709697	43709997	23185	-1.67	1.22E-02	2.07E-01	Intron (NM_053999/117104, intron 2 of 9)
Nkx2-2	366214	chr3	141409778	141410078	1092	-1.58	1.22E-02	2.07E-01	Promoter (1-2kb)
Bnip3l	140923	chr15	43649485	43649785	17244	1.76	1.22E-02	2.07E-01	Intron (NM_080888/140923, intron 3 of 5)
Gsk3b	84027	chr11	65201520	65201820	7017	-1.53	1.22E-02	2.07E-01	Intron (NM_032080/84027, intron 1 of 10)
Kansl3	316328	chr9	42972679	42972979	-29527	1.65	1.22E-02	2.07E-01	Distal Intergenic
Atf2	81647	chr3	60796325	60796625	-374	0.62	1.22E-02	2.07E-01	Promoter (<=1kb)
Cmtm8	301045	chr8	122915759	122916059	-10846	2	1.22E-02	2.07E-01	Distal Intergenic
Steap1	297738	chr4	25434090	25434390	-1495	-1.29	1.22E-02	2.07E-01	Promoter (1-2kb)
MGC95208	304176	chr11	1818520	1818820	122	0.66	1.22E-02	2.07E-01	Promoter (<=1kb)
Pbx1	304947	chr13	86579537	86579837	91621	1.69	1.23E-02	2.07E-01	Intron (NM_001100681/304947, intron 2 of 7)
Gmeb2	83635	chr3	176791577	176791877	83	1.22	1.23E-02	2.07E-01	Promoter (<=1kb)
Plekha7	499249	chr1	185454288	185454588	26688	-1.72	1.23E-02	2.07E-01	Intron (NM_001144861/499249, intron 1 of 7)
Basp1	64160	chr2	77600984	77601284	31342	-1.92	1.23E-02	2.07E-01	Intron (NM_022300/64160, intron 1 of 3)
Fa2h	307855	chr19	43715883	43716183	-119095	2.03	1.23E-02	2.07E-01	Distal Intergenic
Krit1	362317	chr4	27467977	27468277	4873	-2.01	1.23E-02	2.07E-01	Intron (NM_001108618/362317, intron 3 of 16)
Tns1	301509	chr9	81401049	81401349	-62	0.83	1.23E-02	2.07E-01	Promoter (<=1kb)
Nsun7	305339	chr14	43502513	43502813	81507	-1.7	1.23E-02	2.07E-01	Distal Intergenic
Elk3	362871	chr7	34148986	34149286	-27292	0.74	1.23E-02	2.07E-01	Distal Intergenic
Rpl38	689284	chr10	103174745	103175045	-23379	1.38	1.23E-02	2.07E-01	Distal Intergenic
Dear	446170	chr2	184334270	184334570	90294	-1.39	1.23E-02	2.07E-01	Distal Intergenic
Riox1	314300	chr6	107394930	107395230	-33278	1.7	1.23E-02	2.07E-01	Distal Intergenic
Pigl	192263	chr10	48820660	48820960	46642	1.83	1.23E-02	2.07E-01	Intron (NM_138901/192263, intron 2 of 6)
Csrp1	29276	chr13	52570142	52570442	16299	2.13	1.23E-02	2.07E-01	Exon (NM_017148/29276, exon 4 of 6)
Adgrl4	64124	chr2	256569843	256570143	-39644	1.47	1.23E-02	2.07E-01	Distal Intergenic
Nkd1	364952	chr19	19489894	19490194	18893	1.35	1.23E-02	2.07E-01	Intron (NM_001271381/364952, intron 4 of 10)
Lage3	293863	chrX	156355580	156355880	204	0.93	1.23E-02	2.07E-01	Promoter (<=1kb)
Spink7	408237	chr18	57926872	57927172	-6742	-1.58	1.23E-02	2.07E-01	Distal Intergenic
Ints6	361057	chr15	45924575	45924875	2929	1.86	1.23E-02	2.07E-01	Promoter (2-3kb)
Tnfrsf25	500592	chr5	169303940	169304240	15069	2.46	1.23E-02	2.07E-01	Exon (NM_019622/56227, exon 8 of 14)
Lta4h	299732	chr7	34288468	34288768	103	0.82	1.23E-02	2.07E-01	Promoter (<=1kb)
Aftph	305544	chr14	104963708	104964008	11587	-1.42	1.23E-02	2.07E-01	Intron (NM_001305127/305544, intron 1 of 8)
Ehmt1	362078	chr3	2084602	2084902	38904	-1.66	1.24E-02	2.07E-01	Intron (NM_001108572/362078, intron 1 of 25)
Siva1	362791	chr6	137184801	137185101	-25020	1.4	1.24E-02	2.07E-01	Distal Intergenic
Faap20	362678	chr5	172607547	172607847	-41103	-1.37	1.24E-02	2.07E-01	Distal Intergenic
Gstm1	24423	chr2	210805465	210805765	3481	-1.64	1.24E-02	2.07E-01	Intron (NM_017014/24423, intron 7 of 7)
Alkbh5	303193	chr10	46906339	46906639	25	1.05	1.24E-02	2.07E-01	Promoter (<=1kb)
Echdc3	684538	chr17	76097271	76097571	94967	-1.55	1.24E-02	2.07E-01	Distal Intergenic
Pax8	81819	chr3	1580548	1580848	4077	-1.77	1.24E-02	2.07E-01	Intron (NM_031141/81819, intron 2 of 11)
Col4a1	290905	chr16	83521871	83522171	0	0.88	1.24E-02	2.07E-01	Promoter (<=1kb)

Arl8b	500282	chr4	140848601	140848901	10856	-1.48	1.24E-02	2.07E-01	Intron (NM_001024332/500282, intron 1 of 6)
Sec23a	58817	chr6	80106867	80107167	69	0.62	1.24E-02	2.07E-01	Promoter (<=1kb)
Cbr4	359725	chr16	31808304	31808604	163903	-1.5	1.24E-02	2.07E-01	Distal Intergenic
Bnc2	298189	chr5	102519375	102519675	223742	-1.76	1.24E-02	2.07E-01	Intron (NM_001106666/298189, intron 5 of 6)
Popdc3	641520	chr20	50387816	50388116	-6557	1.35	1.24E-02	2.07E-01	Distal Intergenic
Pigb	315807	chr8	79712843	79713143	2141	2.13	1.24E-02	2.07E-01	Promoter (2-3kb)
Barx1	364680	chr17	14822343	14822643	5553	1.96	1.24E-02	2.07E-01	Downstream (2-3kb)
Pdk4	89813	chr4	30556440	30556740	34	0.67	1.24E-02	2.07E-01	Promoter (<=1kb)
Scgb1a1	25575	chr1	225271195	225271495	11751	-1.72	1.24E-02	2.07E-01	Distal Intergenic
Pfdn1	361310	chr18	29186371	29186671	103776	-1.57	1.24E-02	2.07E-01	Distal Intergenic
Nmt2	291318	chr17	79258299	79258599	-295889	-2.02	1.24E-02	2.07E-01	Distal Intergenic
Dpp8	315758	chr8	70554495	70554795	32398	2.04	1.24E-02	2.07E-01	Exon (NM_001108159/315758, exon 15 of 21)
Bcl2l14	500348	chr4	168198410	168198710	58402	-1.44	1.24E-02	2.07E-01	Intron (NM_001107892/312781, intron 18 of 19)
Stat5b	25126	chr10	88754618	88754918	0	1.62	1.24E-02	2.07E-01	Promoter (<=1kb)
Alox5	25290	chr4	148383262	148383562	62711	-1.31	1.24E-02	2.07E-01	Intron (NM_001107882/312656, intron 4 of 7)
Upk1b	303924	chr11	64543757	64544057	21542	2.43	1.25E-02	2.07E-01	Intron (NM_001024253/303924, intron 5 of 7)
Ercc1	292673	chr1	80256566	80256866	-107	0.77	1.25E-02	2.07E-01	Promoter (<=1kb)
Rbm43	311020	chr3	37442744	37443044	2863	-1.97	1.25E-02	2.07E-01	Promoter (2-3kb)
C1s	192262	chr4	157148140	157148440	7156	-0.87	1.25E-02	2.07E-01	Intron (NM_138900/192262, intron 7 of 11)
Adamts2	287899	chr10	36173625	36173925	75574	1.7	1.25E-02	2.07E-01	Intron (NM_001137622/287899, intron 3 of 20)
Shq1	297483	chr4	133186548	133186848	-59373	0.87	1.25E-02	2.07E-01	Distal Intergenic
Fzd2	64512	chr10	90578047	90578347	27900	0.78	1.25E-02	2.07E-01	Distal Intergenic
Hmgb3	305373	chr14	51462157	51462457	264	1.76	1.25E-02	2.07E-01	Promoter (<=1kb)
P2ry2	29597	chr1	166091611	166091911	-46191	1.57	1.25E-02	2.07E-01	Distal Intergenic
Tomm20	266601	chr19	59428981	59429281	444130	-0.81	1.25E-02	2.07E-01	Distal Intergenic
Slc27a1	94172	chr16	20008625	20008925	9513	-1.45	1.25E-02	2.07E-01	Exon (NM_053580/94172, exon 3 of 13)
Foxa2	25099	chr3	142554968	142555268	171678	2.16	1.25E-02	2.07E-01	Distal Intergenic
Mirlet7i	100313993	chr7	66898117	66898417	95386	-1.69	1.25E-02	2.07E-01	Intron (NM_001271079/314897, intron 6 of 9)
Csnk1g3	64823	chr18	48861968	48862268	8797	-1.48	1.25E-02	2.07E-01	Intron (NM_022855/64823, intron 2 of 11)
Taok1	286993	chr10	62508432	62508732	-57910	-1.59	1.25E-02	2.07E-01	Distal Intergenic
Mustn1	290553	chr16	6962187	6962487	-250	0.92	1.25E-02	2.07E-01	Promoter (<=1kb)
Akap8l	299569	chr7	14384564	14384864	-14743	0.93	1.25E-02	2.08E-01	Intron (NM_001108064/314598, intron 3 of 8)
Tnk2	303882	chr11	71391433	71391733	-3441	1.5	1.25E-02	2.08E-01	Distal Intergenic
Marcksl1	81520	chr5	147707899	147708199	-5964	-1.65	1.25E-02	2.08E-01	Distal Intergenic
Adcy6	25289	chr7	140291045	140291345	-249	0.77	1.25E-02	2.08E-01	Promoter (<=1kb)
Mir143	100314035	chr18	56974597	56974897	-3220	1.1	1.25E-02	2.08E-01	Distal Intergenic
Ankrd42	293117	chr1	157479703	157480003	-18115	-1.41	1.25E-02	2.08E-01	Distal Intergenic
Nckap1	58823	chr3	67790854	67791154	13526	-1.67	1.25E-02	2.08E-01	Intron (NM_031618/58823, intron 1 of 29)
Zfp609	363412	chr8	71413721	71414021	-75975	1.51	1.26E-02	2.08E-01	Intron (NM_001134981/315769, intron 14 of 15)
Foxn3	314374	chr6	123655914	123656214	-78219	0.95	1.26E-02	2.08E-01	Distal Intergenic
Trub1	361775	chr1	278416935	278417235	105555	-1.14	1.26E-02	2.08E-01	Distal Intergenic
Cdkn2b	25164	chr5	108052705	108053005	-195320	-1.58	1.26E-02	2.08E-01	Distal Intergenic
Swsap1	363029	chr8	22966886	22967186	134	0.77	1.26E-02	2.08E-01	Promoter (<=1kb)
Mapk1ip1	499280	chr1	211077296	211077596	119272	-1.58	1.26E-02	2.08E-01	Distal Intergenic
Itpr2	81678	chr4	180775622	180775922	24166	-1.53	1.26E-02	2.08E-01	Intron (NM_031046/81678, intron 1 of 63)
Otud4	307774	chr19	32007949	32008249	-65769	0.66	1.26E-02	2.08E-01	Distal Intergenic
Crb2	366031	chr3	22010259	22010559	-27798	2.01	1.26E-02	2.08E-01	Distal Intergenic
Efnb2	306636	chr16	86830905	86831205	199754	1.63	1.26E-02	2.08E-01	Distal Intergenic
Ppox	289219	chr13	89653833	89654133	96	0.68	1.26E-02	2.08E-01	Promoter (<=1kb)
Csnk1g3	64823	chr18	48857840	48858140	4669	-1.29	1.26E-02	2.08E-01	Intron (NM_022855/64823, intron 1 of 11)
Mtmr3	305482	chr14	84809569	84809869	10546	-1.69	1.26E-02	2.08E-01	Intron (NM_001012038/305482, intron 1 of 18)
Purg	361162	chr16	62483080	62483380	0	1.39	1.26E-02	2.08E-01	Promoter (<=1kb)
Cdon	50938	chr8	36692413	36692713	35769	-1.53	1.26E-02	2.08E-01	Exon (NM_017358/50938, exon 16 of 19)
RGD1562310	498188	chr12	43470348	43470648	470150	-1.62	1.26E-02	2.08E-01	Distal Intergenic
RGD1304884	307907	chr19	53767576	53767876	43754	1.21	1.26E-02	2.08E-01	Distal Intergenic
Furin	54281	chr1	142197700	142198000	-518	1.24	1.26E-02	2.08E-01	Promoter (<=1kb)
Kdm1a	500569	chr5	154964929	154965229	0	1.68	1.26E-02	2.08E-01	Promoter (<=1kb)
Fbxo34	305830	chr15	24254371	24254671	-12652	0.9	1.26E-02	2.08E-01	Distal Intergenic
Chd2	308738	chr1	134949683	134949983	-78515	-1.59	1.27E-02	2.08E-01	Distal Intergenic
Vipr2	29555	chr6	143971106	143971406	33145	1.16	1.27E-02	2.08E-01	Intron (NM_017238/29555, intron 4 of 12)
Zg16b	363551	chr10	13328193	13328493	-4344	-2	1.27E-02	2.08E-01	Distal Intergenic
Mkln1	83536	chr4	58699417	58699717	6033	-1.59	1.27E-02	2.08E-01	Intron (NM_031359/83536, intron 1 of 24)
Rab31	246324	chr9	113452257	113452557	52049	-1.28	1.27E-02	2.08E-01	Exon (NM_145094/246324, exon 2 of 7)
Psme4	498433	chr14	115164492	115164792	-1373	1.54	1.27E-02	2.08E-01	Promoter (1-2kb)
Frzb	295691	chr3	67668320	67668620	152	1.01	1.27E-02	2.08E-01	Promoter (<=1kb)
Cdkn1a	114851	chr20	6332042	6332342	-16080	-1.82	1.27E-02	2.08E-01	Distal Intergenic
Pdx1	29535	chr12	9494517	9494817	6394	-1.64	1.27E-02	2.08E-01	Downstream (1-2kb)
Slc30a1	58976	chr13	110677814	110678114	4	0.99	1.27E-02	2.08E-01	Promoter (<=1kb)
Ccdc196	500678	chr6	101300745	101301045	11794	-1.54	1.27E-02	2.08E-01	Intron (NM_001134630/500678, intron 5 of 5)
Rassf3	362886	chr7	63578000	63578300	190	0.77	1.27E-02	2.08E-01	Promoter (<=1kb)
Gjb6	84403	chr15	37430806	37431106	-21138	1.15	1.27E-02	2.08E-01	Distal Intergenic
Brap	687346	chr12	40370007	40370307	47457	0.89	1.27E-02	2.08E-01	Distal Intergenic
Enpp3	54410	chr1	21604507	21604807	-8341	1.21	1.27E-02	2.09E-01	Distal Intergenic
Ppp3ca	24674	chr2	242054362	242054662	144530	-1.93	1.27E-02	2.09E-01	Intron (NM_017041/24674, intron 2 of 13)
Hdhd3	688746	chr5	78361350	78361650	0	0.67	1.28E-02	2.09E-01	Promoter (<=1kb)
Pard6b	362279	chr3	164791591	164791891	-30220	1.48	1.28E-02	2.09E-01	Distal Intergenic
Ssrp1	81785	chr3	72471199	72471499	23230	0.67	1.28E-02	2.09E-01	Distal Intergenic
Ppp6c	171121	chr3	23504487	23504787	6172	-1.4	1.28E-02	2.09E-01	Intron (NM_133589/171121, intron 1 of 6)
Cdk14	362316	chr4	25825660	25825960	93	0.92	1.28E-02	2.09E-01	Promoter (<=1kb)
Ahcyl2	312192	chr4	57057836	57058136	7595	1.91	1.28E-02	2.09E-01	Intron (NM_001173510/312192, intron 1 of 16)
Nat14	361500	chr1	72472638	72472938	-8146	0.97	1.28E-02	2.09E-01	Distal Intergenic
Mir21	100314000	chr10	73906436	73906736	-4135	-1.05	1.28E-02	2.09E-01	Intron (NM_138839/192129, intron 10 of 11)
Tspan8	171048	chr7	58877387	58877687	62582	0.87	1.28E-02	2.09E-01	Distal Intergenic
Dab2ip	192126	chr3	14922173	14922473	32910	-1.59	1.28E-02	2.09E-01	Intron (NM_138710/192126, intron 1 of 15)
Cdc42	64465	chr5	155713111	155713411	14889	-1.66	1.28E-02	2.09E-01	Intron (NM_171994/64465, intron 1 of 5)
Kcnmb4	66016	chr7	59515012	59515312	-253	1.25	1.28E-02	2.09E-01	Promoter (<=1kb)
Edem2	296304	chr3	151252661	151252961	5598	-1.53	1.28E-02	2.09E-01	Intron (NM_001004230/296304, intron 3 of 10)
Hexb	294673	chr2	28028542	28028842	-25282	-1.7	1.28E-02	2.09E-01	Intron (NM_001003401/294674, intron 1 of 3)
Col9a2	362584	chr5	139963651	139963951	649	-1.76	1.28E-02	2.09E-01	Promoter (<=1kb)
Hapln1	29331	chr2	18453267	18453567	98725	-1.67	1.28E-02	2.09E-01	Distal Intergenic
Pdcd6	308061	chr1	31576239	31576539	56	0.58	1.28E-02	2.09E-01	Promoter (<=1kb)
Naa40	361718	chr1	222491616	222491916	3466	-1.56	1.28E-02	2.09E-01	Intron (NM_001108518/361718, intron 1 of 7)
Atg7	312647	chr4	146636627	146636927	38211	1.56	1.28E-02	2.09E-01	Intron (NM_001012097/312647, intron 14 of 17)
Smarcad1	312398	chr4	95888554	95888854	3811	-1.68	1.28E-02	2.09E-01	Intron (NM_001107864/312398, intron 1 of 22)

Arih1	300756	chr8	64256074	64256374	12181	-1.36	1.28E-02	2.09E-01	Intron (NM_001013108/300756, intron 1 of 13)
Nr3c2	25672	chr19	34607496	34607796	153207	1.75	1.28E-02	2.09E-01	Intron (NM_013131/25672, intron 2 of 9)
Lama1	316758	chr9	115866997	115867297	-49610	0.91	1.29E-02	2.09E-01	Distal Intergenic
Il1rapl1	317553	chrX	58567572	58567872	-1801679	0.85	1.29E-02	2.09E-01	Distal Intergenic
Rara	24705	chr10	86879429	86879729	8899	2.27	1.29E-02	2.09E-01	Intron (NM_031528/24705, intron 3 of 7)
Decr2	64461	chr10	15426683	15426983	32914	-1.62	1.29E-02	2.09E-01	Distal Intergenic
Snx18	310097	chr2	45373835	45374135	106663	1.78	1.29E-02	2.09E-01	Distal Intergenic
Gnai2	81664	chr8	116390889	116391189	118	0.85	1.29E-02	2.09E-01	Promoter (<=1kb)
Grb2	81504	chr10	104272059	104272359	-8988	-1.43	1.29E-02	2.09E-01	Distal Intergenic
Psip1	313323	chr5	101656493	101656793	-68395	0.93	1.29E-02	2.09E-01	Distal Intergenic
Maff	366960	chr7	120587353	120587653	6610	-1.59	1.29E-02	2.09E-01	Intron (NM_001130573/366960, intron 1 of 2)
Ccdc91	312863	chr4	181898767	181899067	23906	-1.73	1.29E-02	2.10E-01	Intron (NM_001014061/312863, intron 1 of 12)
Actr3	81732	chr13	41726681	41726981	11641	2.17	1.29E-02	2.10E-01	Intron (NM_001177819/100362110, intron 2 of 8)
C1d	289810	chr14	101120774	101121074	705129	-1.68	1.29E-02	2.10E-01	Distal Intergenic
Cdcp1	301082	chr8	132293967	132294267	2394	-1.77	1.29E-02	2.10E-01	Promoter (2-3kb)
Ldlrad4	679578	chr18	63781240	63781540	-62459	-1.57	1.30E-02	2.10E-01	Distal Intergenic
Rai14	294804	chr2	60641825	60642125	41326	-1.53	1.30E-02	2.10E-01	Intron (NM_001011947/294804, intron 2 of 16)
Nxn	360577	chr10	64549682	64549982	163	1.14	1.30E-02	2.10E-01	Promoter (<=1kb)
Rbpj	679028	chr14	59778599	59778899	-43149	-0.84	1.30E-02	2.10E-01	Distal Intergenic
Naca	288770	chr7	2451789	2452089	-6161	-1.32	1.30E-02	2.10E-01	Distal Intergenic
LOC100911360	100911360	chr8	73689336	73689636	-154341	-1.45	1.30E-02	2.10E-01	Distal Intergenic
Pgr	25154	chr8	7135040	7135340	6384	-1.45	1.30E-02	2.10E-01	Intron (NM_022847/25154, intron 7 of 12)
Phf3	363210	chr9	37143388	37143688	327	1.63	1.30E-02	2.10E-01	Promoter (<=1kb)
Tmpo	25359	chr7	31871823	31872123	248	1.13	1.30E-02	2.10E-01	Promoter (<=1kb)
Ldhd	307858	chr19	43836292	43836592	12345	-1.34	1.30E-02	2.10E-01	Intron (NM_001008893/307858, intron 10 of 10)
Asah2	114104	chr1	250744531	250744831	-113802	-1.8	1.30E-02	2.10E-01	Intron (NM_181386/353229, intron 5 of 9)
Evi2a	685433	chr10	66871696	66871996	1952	-1.8	1.30E-02	2.10E-01	Promoter (1-2kb)
Svil	361256	chr17	55344824	55345124	1155	-1.8	1.30E-02	2.10E-01	Promoter (1-2kb)
Palmd	310811	chr2	220663132	220663432	-127128	-1.8	1.30E-02	2.10E-01	Distal Intergenic
Upb1	116593	chr20	14168551	14168851	24873	-1.8	1.30E-02	2.10E-01	Intron (NM_053845/116593, intron 8 of 8)
Lims1	499443	chr20	27933532	27933832	-20735	-1.8	1.30E-02	2.10E-01	Distal Intergenic
Hif1a	29560	chr6	96679267	96679567	-131618	-1.8	1.30E-02	2.10E-01	Distal Intergenic
Ncam1	24586	chr8	54337739	54338039	-203008	-1.8	1.30E-02	2.10E-01	Distal Intergenic
Abhd5	316122	chr8	130945000	130945300	-27922	-1.8	1.30E-02	2.10E-01	Distal Intergenic
Shc4	679845	chr3	117975072	117975372	14917	-1.92	1.30E-02	2.10E-01	Intron (NM_001191065/679845, intron 1 of 11)
Sec31a	93646	chr14	10860251	10860551	5514	-1.62	1.30E-02	2.10E-01	Intron (NM_033021/93646, intron 1 of 27)
Agr3	298959	chr6	55213635	55213935	-157803	-1.62	1.30E-02	2.10E-01	Distal Intergenic
Igf2bp2	303824	chr11	82495312	82495612	29241	-1.12	1.30E-02	2.10E-01	Intron (NM_001270598/303824, intron 2 of 15)
Banp	292064	chr19	54849990	54850290	83395	1.91	1.30E-02	2.10E-01	Distal Intergenic
Cav2	363425	chr4	44580949	44581249	7685	1.86	1.30E-02	2.10E-01	Distal Intergenic
Cnnm3	301345	chr9	43093334	43093634	196	1.32	1.30E-02	2.10E-01	Promoter (<=1kb)
Ambp	25377	chr5	78815549	78815849	170077	-1.58	1.30E-02	2.10E-01	Distal Intergenic
LOC499469	499469	chr20	46700560	46700860	-6502	-1.53	1.30E-02	2.10E-01	Intron (NM_001106396/294518, intron 1 of 7)
Id3	25585	chr5	154456808	154457108	-32495	-1.49	1.31E-02	2.10E-01	Distal Intergenic
Twist2	59327	chr9	98875210	98875510	-48624	-1.67	1.31E-02	2.10E-01	Distal Intergenic
Stk40	360230	chr5	144135390	144135690	21378	-1.38	1.31E-02	2.10E-01	Intron (NM_183056/360230, intron 2 of 10)
Ggnbp2	360584	chr10	72188020	72188320	0	0.69	1.31E-02	2.10E-01	Promoter (<=1kb)
Erbb3	29496	chr7	3015538	3015838	-5226	-1.49	1.31E-02	2.10E-01	Distal Intergenic
Smc4	295107	chr2	165601685	165601985	678	0.75	1.31E-02	2.10E-01	Promoter (<=1kb)
Rflna	689711	chr12	36976637	36976937	70691	2.34	1.31E-02	2.10E-01	Intron (NM_001108334/360801, intron 14 of 46)
Tmem255b	290877	chr16	81211581	81211881	6815	1.54	1.31E-02	2.10E-01	Downstream (1-2kb)
Sh3rf1	306417	chr16	32185558	32185858	39919	-1.77	1.31E-02	2.10E-01	Intron (NM_198764/306417, intron 1 of 10)
Smad2	29357	chr18	72590966	72591266	40512	-1.77	1.31E-02	2.10E-01	Intron (NM_001277450/29357, intron 2 of 10)
Osbpl9	298369	chr5	128825857	128826157	13278	-1.77	1.31E-02	2.10E-01	Intron (NM_001044234/298369, intron 1 of 23)
Adcy5	64532	chr11	68989776	68990076	-147456	0.85	1.31E-02	2.10E-01	Distal Intergenic
Klhl25	293023	chr1	137422592	137422892	-63520	-1.64	1.31E-02	2.10E-01	Distal Intergenic
Hat1	296501	chr3	57965663	57965963	-56432	-1.52	1.31E-02	2.10E-01	Distal Intergenic
Sgk1	29517	chr1	24338473	24338773	-144906	-1.62	1.31E-02	2.10E-01	Distal Intergenic
Rapgef3	59326	chr7	139260037	139260337	-5841	2	1.31E-02	2.10E-01	Distal Intergenic
Acpp	56780	chr8	112879952	112880252	3770	-1.39	1.31E-02	2.10E-01	Intron (NM_001134901/56780, intron 1 of 10)
Zbtb5	298084	chr5	60559153	60559453	-818	0.8	1.31E-02	2.10E-01	Promoter (<=1kb)
Bend6	363212	chr9	38089568	38089868	-207385	0.73	1.31E-02	2.10E-01	Distal Intergenic
Hoxb13	303480	chr10	84039173	84039473	7218	2.04	1.31E-02	2.10E-01	Distal Intergenic
Slc35a3	310808	chr2	219741613	219741913	0	1.17	1.32E-02	2.10E-01	Promoter (<=1kb)
Zfp36l1	29344	chr6	102991247	102991547	321527	2.06	1.32E-02	2.10E-01	Distal Intergenic
Slc14a2	54302	chr18	74662179	74662479	-120102	2	1.32E-02	2.10E-01	Intron (NM_019347/54302, intron 3 of 21)
Uts2b	378939	chr11	76838464	76838764	4703	1.66	1.32E-02	2.10E-01	Intron (NM_198133/378939, intron 2 of 4)
Spag1	315033	chr7	74999422	74999722	4817	1.34	1.32E-02	2.10E-01	Intron (NM_001012116/315033, intron 2 of 18)
Fcgbpl1	292746	chr1	85040217	85040517	-18024	-1.72	1.32E-02	2.10E-01	Intron (NM_001164657/100303643, intron 16 of 19)
Zfp36l2	298765	chr6	7348140	7348440	73016	-1.48	1.32E-02	2.10E-01	Distal Intergenic
Csnk1g3	64823	chr18	48842349	48842649	-10522	-1.28	1.32E-02	2.10E-01	Distal Intergenic
Kdm2b	304495	chr12	39021860	39022160	0	0.64	1.32E-02	2.10E-01	Promoter (<=1kb)
Kansl3	316328	chr9	42935035	42935335	7817	-1.72	1.32E-02	2.10E-01	Intron (NM_001034835/316328, intron 3 of 19)
Jak2	24514	chr1	247424913	247425213	26246	-1.49	1.32E-02	2.10E-01	Intron (NM_031514/24514, intron 4 of 24)
Brd4	362844	chr7	14302394	14302694	361	0.99	1.32E-02	2.10E-01	Promoter (<=1kb)
Gemin5	691231	chr10	43583078	43583378	15854	2.02	1.32E-02	2.10E-01	Exon (NM_001172089/691231, exon 11 of 28)
Zcchc7	298086	chr5	60248655	60248955	-1591	2.19	1.32E-02	2.10E-01	Promoter (1-2kb)
Kctd21	499209	chr1	162214904	162215204	-107105	0.91	1.32E-02	2.10E-01	Intron (NM_053417/84477, intron 2 of 9)
Xrcc4	309995	chr2	18954571	18954871	-27206	-1.63	1.32E-02	2.11E-01	Distal Intergenic
Dmrt2	309430	chr1	243704349	243704649	41526	2.35	1.32E-02	2.11E-01	Distal Intergenic
Purg	361162	chr16	62648919	62649219	-165624	-2.11	1.32E-02	2.11E-01	Distal Intergenic
Abcc12	291923	chr19	21682286	21682586	140544	-1.33	1.32E-02	2.11E-01	Distal Intergenic
Vopp1	362374	chr4	88437193	88437493	-3574	2.05	1.33E-02	2.11E-01	Distal Intergenic
Ipmk	171458	chr20	18485510	18485810	4018	-1.7	1.33E-02	2.11E-01	Intron (NM_134417/171458, intron 1 of 5)
Kif2c	171529	chr5	136066923	136067223	-13712	1.65	1.33E-02	2.11E-01	Distal Intergenic
Cchcr1	406196	chr20	3737830	3738130	1836	-1.73	1.33E-02	2.11E-01	Promoter (1-2kb)
Pitpnb	114561	chr12	51568379	51568679	-226692	1.11	1.33E-02	2.11E-01	Distal Intergenic
Ptgfr	25652	chr2	257084175	257084475	-46070	1.86	1.33E-02	2.11E-01	Distal Intergenic
Cd9	24936	chr4	157971330	157971630	38461	-1.52	1.33E-02	2.11E-01	Distal Intergenic
Ppp6r1	361502	chr1	72748626	72748926	-8955	-1.38	1.33E-02	2.11E-01	Intron (NM_139261/246146, intron 4 of 7)
Zc3h15	362154	chr3	71026205	71026505	5671	-1.66	1.33E-02	2.11E-01	Intron (NM_001010963/362154, intron 1 of 9)
Kctd9	364410	chr15	44411803	44412103	0	0.87	1.33E-02	2.11E-01	Promoter (<=1kb)
Kcnj5	29713	chr8	33331891	33332191	131219	-1.48	1.33E-02	2.11E-01	Distal Intergenic
Cbfa2t3	361431	chr19	55571336	55571636	-60876	0.72	1.33E-02	2.11E-01	Distal Intergenic

Zfp64	311661	chr3	165787089	165787389	-45157	1.28	1.33E-02	2.11E-01	Distal Intergenic
Avil	79253	chr7	70272491	70272791	-19774	0.75	1.34E-02	2.11E-01	Distal Intergenic
Zic5	361095	chr15	108844953	108845253	53450	1.51	1.34E-02	2.12E-01	Distal Intergenic
Srebf1	78968	chr10	46581344	46581644	1210	-1.63	1.34E-02	2.12E-01	Promoter (1-2kb)
Pip4p2	362490	chr5	28518167	28518467	122650	-1.21	1.34E-02	2.12E-01	Intron (NM_022302/64169, intron 10 of 12)
Maf	54267	chr19	47763814	47764114	432634	-1.41	1.34E-02	2.12E-01	Distal Intergenic
Ccdc50	288022	chr11	76429740	76430040	374415	0.82	1.34E-02	2.12E-01	Distal Intergenic
Inhba	29200	chr17	51385375	51385675	526748	-1.9	1.34E-02	2.12E-01	Distal Intergenic
Cd180	294706	chr2	33216866	33217166	396544	-0.96	1.34E-02	2.12E-01	Distal Intergenic
Olr1743	294197	chr20	1853056	1853356	-6905	-1.75	1.34E-02	2.12E-01	Distal Intergenic
Ppef2	305246	chr14	17340962	17341262	-7374	1.35	1.34E-02	2.12E-01	Distal Intergenic
Kremen1	114107	chr14	85479936	85480236	23425	1.54	1.34E-02	2.12E-01	Intron (NM_053649/114107, intron 2 of 8)
Kalrn	84009	chr11	69427842	69428142	-56151	0.71	1.34E-02	2.12E-01	Distal Intergenic
Tsga10	252923	chr9	44300338	44300638	155929	-1.67	1.34E-02	2.12E-01	Distal Intergenic
Gpr107	311857	chr3	10741009	10741309	-46355	0.72	1.34E-02	2.12E-01	Distal Intergenic
Tle4	25565	chr1	231531778	231532078	-992	1.07	1.34E-02	2.12E-01	Promoter (<=1kb)
Zfpm2	314930	chr7	79907201	79907501	435924	1.59	1.34E-02	2.12E-01	Intron (NM_001130501/314930, intron 4 of 6)
Zc3h8	311414	chr3	121488147	121488447	81	0.67	1.34E-02	2.12E-01	Promoter (<=1kb)
Cenpf	257649	chr13	108175359	108175659	2950	-1.71	1.34E-02	2.12E-01	Promoter (2-3kb)
Acat1	25014	chr8	58188952	58189252	6632	-1.55	1.35E-02	2.12E-01	Intron (NM_017075/25014, intron 1 of 11)
Bdp1	294687	chr2	30346004	30346304	-5796	0.6	1.35E-02	2.12E-01	Distal Intergenic
Txndc11	302899	chr10	4576276	4576576	-1893	1.92	1.35E-02	2.12E-01	Promoter (1-2kb)
P4ha2	360526	chr10	39438784	39439084	3557	-1.6	1.35E-02	2.12E-01	Intron (NM_001108275/360526, intron 2 of 15)
Kpna3	361055	chr15	41765027	41765327	4785	-1.83	1.35E-02	2.12E-01	Intron (NM_001014792/361055, intron 1 of 16)
Gng12	114120	chr4	97692415	97692715	34744	-1.83	1.35E-02	2.12E-01	Intron (NM_053661/114120, intron 2 of 3)
Rela	309165	chr1	220992741	220993041	0	0.88	1.35E-02	2.12E-01	Promoter (<=1kb)
Pnisr	297942	chr5	35998030	35998330	6962	-1.85	1.35E-02	2.12E-01	Intron (NM_001025274/297942, intron 2 of 11)
Rnf182	498726	chr17	24323740	24324040	81098	1.59	1.35E-02	2.12E-01	Distal Intergenic
Mki67	291234	chr1	208187808	208188108	-167354	0.97	1.35E-02	2.12E-01	Distal Intergenic
Ldhal6b	369018	chr1	45793041	45793341	515935	-1.56	1.35E-02	2.12E-01	Distal Intergenic
Aak1	500244	chr4	118708119	118708419	52391	1.32	1.35E-02	2.12E-01	Intron (NM_001173450/500244, intron 1 of 20)
Rcc1	682908	chr5	150578040	150578340	-52786	-1.87	1.35E-02	2.12E-01	Distal Intergenic
Mir107	100314022	chr1	253110257	253110557	17779	-1.62	1.35E-02	2.13E-01	Distal Intergenic
Ccnl1	114121	chr2	157722722	157723022	36816	2.01	1.36E-02	2.13E-01	Distal Intergenic
Smlr1	686032	chr1	20950898	20951198	94711	-1.64	1.36E-02	2.13E-01	Distal Intergenic
Cdk14	362316	chr4	25879015	25879315	53448	-1.67	1.36E-02	2.13E-01	Intron (NM_001108617/362316, intron 2 of 12)
Mlycd	85239	chr19	52047797	52048097	14847	1.06	1.36E-02	2.13E-01	Exon (NM_053477/85239, exon 5 of 5)
Cldn20	680178	chr1	44236502	44236802	-209963	1.93	1.36E-02	2.13E-01	Distal Intergenic
Ppp2r5c	691318	chr6	134900993	134901293	56334	1.17	1.36E-02	2.13E-01	Intron (NM_001191112/691318, intron 2 of 13)
Crlf3	54395	chr10	67412077	67412377	-10239	-1.53	1.36E-02	2.13E-01	Distal Intergenic
Pth1r	56813	chr8	118983246	118983546	24261	2.04	1.36E-02	2.13E-01	Distal Intergenic
Pparg	25664	chr4	147231464	147231764	-42343	2.07	1.36E-02	2.13E-01	Distal Intergenic
Ptprm	29616	chr9	115531176	115531476	23785	1.54	1.36E-02	2.13E-01	Intron (NM_001168632/29616, intron 1 of 32)
Pla2g5	29354	chr5	157270544	157270844	-1641	-1.84	1.36E-02	2.13E-01	Promoter (1-2kb)
Cr1l	54243	chr13	113868570	113868870	3227	-1.72	1.36E-02	2.13E-01	Exon (NM_001005265/54243, exon 2 of 12)
Trarg1	360576	chr10	63549734	63550034	-58021	1.97	1.36E-02	2.13E-01	Distal Intergenic
Hif1a	29560	chr6	96825458	96825758	14273	-1.57	1.36E-02	2.13E-01	Intron (NM_024359/29560, intron 1 of 15)
Aplp2	64312	chr8	32360394	32360694	-31573	1.03	1.36E-02	2.13E-01	Distal Intergenic
Hebp2	308632	chr1	13679512	13679812	40158	1.9	1.37E-02	2.13E-01	Distal Intergenic
Sema3b	363142	chr8	116375882	116376182	-14539	2.03	1.37E-02	2.13E-01	Exon (NM_031035/81664, exon 3 of 9)
Actb	81822	chr12	13728900	13729200	13057	0.62	1.37E-02	2.13E-01	Distal Intergenic
Sbk2	691411	chr1	72431265	72431565	5537	1.58	1.37E-02	2.13E-01	Intron (NM_001127539/691411, intron 3 of 3)
Fndc3b	294925	chr2	113323343	113323643	21908	-1.6	1.37E-02	2.14E-01	Intron (NM_001191704/294925, intron 1 of 24)
Fnbp1l	310839	chr2	226779044	226779344	0	0.9	1.37E-02	2.14E-01	Promoter (<=1kb)
Crebbp	54244	chr10	11590741	11591041	-4003	2.27	1.37E-02	2.14E-01	Distal Intergenic
Etv4	360635	chr10	89675839	89676139	24144	2.07	1.37E-02	2.14E-01	Intron (NM_001047844/287727, intron 16 of 21)
Hist2h2aa3	365877	chr2	198381603	198381903	-6863	1.7	1.37E-02	2.14E-01	Distal Intergenic
Pcyox1	246302	chr4	118197749	118198049	0	0.71	1.37E-02	2.14E-01	Promoter (<=1kb)
Tbc1d2b	315880	chr8	97633052	97633352	13720	-2.14	1.37E-02	2.14E-01	Intron (NM_001108175/315880, intron 2 of 13)
Ndufb7	361385	chr19	24710838	24711138	9771	2.2	1.37E-02	2.14E-01	Intron (NM_138549/191576, intron 1 of 12)
Tlr2	310553	chr2	182869226	182869526	-23165	-1.49	1.37E-02	2.14E-01	Distal Intergenic
Klf7	363243	chr9	70796420	70796720	-8507	-1.92	1.37E-02	2.14E-01	Distal Intergenic
Coq9	498909	chr19	10593887	10594187	2661	-1.57	1.37E-02	2.14E-01	Promoter (2-3kb)
LOC100910973	100910973	chr1	227113567	227113867	0	-1.5	1.37E-02	2.14E-01	Promoter (<=1kb)
Timp2	29543	chr10	107389031	107389331	-2959	1.97	1.37E-02	2.14E-01	Promoter (2-3kb)
Prpf40a	295607	chr3	38551538	38551838	189275	-1.46	1.38E-02	2.14E-01	Distal Intergenic
Itpkb	54260	chr13	98667889	98668189	52602	-1.93	1.38E-02	2.14E-01	Intron (NM_019312/54260, intron 2 of 7)
Lrrc8d	305131	chr14	5168353	5168653	-67176	2.09	1.38E-02	2.14E-01	Distal Intergenic
Lrrc49	300763	chr8	65484139	65484439	102988	1.82	1.38E-02	2.14E-01	Intron (NM_001134469/300763, intron 15 of 15)
Myom2	306616	chr16	78443530	78443830	1227889	-1.55	1.38E-02	2.14E-01	Intron (NM_001037327/364634, intron 10 of 67)
Dcun1d1	310324	chr2	122337844	122338144	182328	1.3	1.38E-02	2.14E-01	Distal Intergenic
Cdca2	305984	chr15	44366523	44366823	44181	1.4	1.38E-02	2.14E-01	Exon (NM_001107273/305984, exon 15 of 15)
Upp1	289801	chr14	89312944	89313244	-2445	-1.73	1.38E-02	2.14E-01	Promoter (2-3kb)
Kirrel1	310695	chr2	186470288	186470588	9690	-1.73	1.38E-02	2.14E-01	Intron (NM_207606/310695, intron 1 of 14)
Eea1	314764	chr7	37097672	37097972	-3442	-1.73	1.38E-02	2.14E-01	Distal Intergenic
Neurl3	316326	chr9	42841208	42841508	-1371	-1.73	1.38E-02	2.14E-01	Promoter (1-2kb)
Nrg1	112400	chr16	62969285	62969585	-403	-1.76	1.38E-02	2.14E-01	Promoter (<=1kb)
Mfsd4a	498228	chr13	48848610	48848910	87	2.35	1.38E-02	2.14E-01	Promoter (<=1kb)
Ddb2	100362121	chr3	80050915	80051215	1738	-1.74	1.38E-02	2.14E-01	Promoter (1-2kb)
Tle4	25565	chr1	231530290	231530590	196	1.19	1.38E-02	2.14E-01	Promoter (<=1kb)
Itgb5	257645	chr11	70161131	70161431	10733	-1.4	1.38E-02	2.14E-01	Intron (NM_147139/257645, intron 1 of 15)
Vapb	60431	chr3	171860727	171861027	28169	-1.34	1.38E-02	2.14E-01	Intron (NM_021847/60431, intron 2 of 5)
Adora2b	29316	chr10	48573450	48573750	3884	-1.7	1.38E-02	2.14E-01	Intron (NM_017161/29316, intron 1 of 1)
Ap4s1	366618	chr6	72462142	72462442	165	0.92	1.38E-02	2.14E-01	Promoter (<=1kb)
Ppwd1	294711	chr2	34549197	34549497	-236103	-1.67	1.38E-02	2.14E-01	Distal Intergenic
Htra1	65164	chr1	201507665	201507965	8598	-1.47	1.39E-02	2.14E-01	Intron (NM_031721/65164, intron 1 of 8)
Mocs1	301221	chr9	13363992	13364292	149668	-1.91	1.39E-02	2.14E-01	Distal Intergenic
Pigy	502782	chr4	88584321	88584621	79	0.67	1.39E-02	2.14E-01	Promoter (<=1kb)
Engase	303702	chr10	107514747	107515047	12111	1.03	1.39E-02	2.15E-01	3' UTR
Vps41	306991	chr17	48823873	48824173	-13240	-1.19	1.39E-02	2.15E-01	Distal Intergenic
Ext1	299907	chr7	92600067	92600367	281025	-1.51	1.39E-02	2.15E-01	Distal Intergenic
Zfp958	100302405	chr12	5421606	5421906	70348	1.03	1.39E-02	2.15E-01	Distal Intergenic
Ric8b	314681	chr7	25810457	25810757	-109110	1.38	1.39E-02	2.15E-01	Intron (NM_001271382/100910996, intron 2 of 5)
Foxn3	314374	chr6	123574858	123575158	2537	2.02	1.39E-02	2.15E-01	Promoter (2-3kb)

Mettl15	295985	chr3	100359281	100359581	4675	-1.73	1.39E-02	2.15E-01	Intron (NM_001024981/295985, intron 2 of 4)
Mir3075	102465157	chr16	1816945	1817245	-5560	1.25	1.40E-02	2.15E-01	Intron (NM_001108393/361103, intron 2 of 23)
Zfand2a	360772	chr12	17257666	17257966	5573	-1.42	1.40E-02	2.15E-01	Intron (NM_001008363/360772, intron 3 of 6)
Pja2	192256	chr9	112048372	112048672	175	1.18	1.40E-02	2.15E-01	Promoter (<=1kb)
Gpr20	60667	chr7	114866051	114866351	78993	1.59	1.40E-02	2.16E-01	Distal Intergenic
Ndufs8	293652	chr1	219144229	219144529	81	0.63	1.40E-02	2.16E-01	Promoter (<=1kb)
Spata25	499943	chr3	161294490	161294790	-365	1.93	1.40E-02	2.16E-01	Promoter (<=1kb)
Hk2	25059	chr4	113600673	113600973	8924	-1.19	1.40E-02	2.16E-01	Intron (NM_012735/25059, intron 1 of 17)
Rab2a	65158	chr5	21750296	21750596	117387	-1.38	1.40E-02	2.16E-01	Distal Intergenic
Mir488	100314120	chr13	76412518	76412818	213925	-1.48	1.40E-02	2.16E-01	Distal Intergenic
Sertad2	498423	chr14	104799574	104799874	-21341	-1.28	1.40E-02	2.16E-01	Distal Intergenic
Ndfip1	291609	chr18	31534000	31534300	-40521	-1.39	1.40E-02	2.16E-01	Distal Intergenic
Il1a	24493	chr3	121832383	121832683	3439	-1.64	1.40E-02	2.16E-01	Intron (NM_017019/24493, intron 3 of 6)
Atp6v0a2	116455	chr12	37374275	37374575	23658	-1.57	1.41E-02	2.16E-01	Intron (NM_053775/116455, intron 14 of 19)
Extl3	56819	chr15	48484833	48485133	-39241	-1.49	1.41E-02	2.16E-01	Distal Intergenic
Cxxc1	291440	chr18	70191216	70191516	-1293	-1.64	1.41E-02	2.16E-01	Promoter (1-2kb)
Ptpdc1	291022	chr17	16351713	16352013	18298	-1.46	1.41E-02	2.16E-01	Intron (NM_001106104/291022, intron 1 of 9)
Mir6332	102466626	chr5	167516650	167516950	113682	-1.39	1.41E-02	2.16E-01	Exon (NM_053885/116665, exon 5 of 23)
Echs1	140547	chr1	141792410	141792710	-29230	-1.17	1.41E-02	2.16E-01	Distal Intergenic
Btbd16	361658	chr1	201322331	201322631	-14829	2.27	1.41E-02	2.16E-01	Intron (NM_001004415/309025, intron 18 of 21)
Pcdh18	295027	chr2	138878217	138878517	-44352	-1.63	1.41E-02	2.16E-01	Distal Intergenic
Vps26a	361846	chr20	32111061	32111361	3949	-1.57	1.41E-02	2.16E-01	Intron (NM_001007740/361846, intron 1 of 8)
Dmrta2	313471	chr5	129758057	129758357	3762	2.19	1.41E-02	2.16E-01	Exon (NM_001107951/313471, exon 3 of 4)
Etv3	295297	chr2	186854863	186855163	-19072	-1.49	1.41E-02	2.16E-01	Distal Intergenic
Mbnl1	282635	chr2	150798198	150798498	41940	-1.59	1.41E-02	2.16E-01	Intron (NM_001191566/282635, intron 1 of 7)
Nagk	297393	chr4	115515981	115516281	41949	0.62	1.41E-02	2.16E-01	Distal Intergenic
Cers4	304208	chr12	4718635	4718935	-243906	0.75	1.41E-02	2.16E-01	Distal Intergenic
Ksr1	360573	chr10	66409501	66409801	72349	-1.47	1.41E-02	2.16E-01	Intron (NM_001108284/360573, intron 1 of 17)
Eps8l3	295361	chr2	210610609	210610909	-57494	-1.5	1.42E-02	2.17E-01	Distal Intergenic
Itgbl1	498564	chr15	110114091	110114391	0	2.14	1.42E-02	2.17E-01	Promoter (<=1kb)
Nme2	83782	chr10	81631392	81631692	22025	1.95	1.42E-02	2.17E-01	Distal Intergenic
LOC682259	682259	chr1	129469016	129469316	145087	1.22	1.42E-02	2.17E-01	Distal Intergenic
Spock1	306759	chr17	7479888	7480188	-195349	1.5	1.42E-02	2.17E-01	Distal Intergenic
Fam43a	288031	chr11	73682805	73683105	-3802	-1.69	1.42E-02	2.17E-01	Distal Intergenic
Cbr4	359725	chr16	31885563	31885863	86644	1.29	1.42E-02	2.17E-01	Distal Intergenic
PCOLCE2	684050	chr8	103446732	103447032	-12187	-1.71	1.42E-02	2.17E-01	Distal Intergenic
Tsku	308843	chr1	163356825	163357125	-28234	-2.02	1.42E-02	2.17E-01	Distal Intergenic
Copg2	301742	chr4	58194864	58195164	78	0.97	1.42E-02	2.17E-01	Promoter (<=1kb)
Capn13	362701	chr6	24456259	24456559	219859	1	1.42E-02	2.17E-01	Distal Intergenic
Ypel2	360590	chr10	74253146	74253446	45153	-1.55	1.42E-02	2.17E-01	Intron (NM_001108286/360590, intron 2 of 4)
Tax1bp1	246244	chr4	82908043	82908343	131297	1.62	1.42E-02	2.17E-01	Distal Intergenic
H1f4	201097	chr17	43734481	43734781	20	0.67	1.42E-02	2.17E-01	Promoter (<=1kb)
Uspl1	288447	chr12	6987974	6988274	-31060	-1.6	1.42E-02	2.17E-01	Distal Intergenic
Tpcn1	246215	chr12	41519315	41519615	11531	-1.55	1.42E-02	2.17E-01	Intron (NM_139332/246215, intron 2 of 29)
LOC682870	682870	chr1	37937694	37937994	-29942	0.62	1.42E-02	2.17E-01	Distal Intergenic
Prkag2	373545	chr4	6783618	6783918	30190	-1.15	1.42E-02	2.17E-01	Intron (NM_184051/373545, intron 3 of 11)
Coro1c	501841	chr12	48482190	48482490	-27252	1.08	1.42E-02	2.17E-01	Distal Intergenic
Pik3r1	25513	chr2	31795059	31795359	21026	-1.61	1.42E-02	2.17E-01	Intron (NM_013005/25513, intron 1 of 14)
Tmem87b	362212	chr3	121358087	121358387	81	0.62	1.42E-02	2.17E-01	Promoter (<=1kb)
Ptafr	58949	chr5	150764612	150764912	10591	2.25	1.43E-02	2.17E-01	Intron (NM_053321/58949, intron 1 of 1)
Cav2	363425	chr4	44573409	44573709	145	0.98	1.43E-02	2.17E-01	Promoter (<=1kb)
Clrn2	689515	chr14	70143208	70143508	17116	-1.55	1.43E-02	2.17E-01	Distal Intergenic
Map3k14	360640	chr10	91379141	91379441	-25540	-1.35	1.43E-02	2.17E-01	Distal Intergenic
Rala	81757	chr17	49710758	49711058	-3232	-1.66	1.43E-02	2.17E-01	Distal Intergenic
Phc2	313038	chr5	146888810	146889110	6054	-1.66	1.43E-02	2.17E-01	Intron (NM_001013169/313038, intron 4 of 13)
Igsf3	295325	chr2	203712927	203713227	-55502	1.58	1.43E-02	2.17E-01	Intron (NM_012830/497761, intron 2 of 6)
Ctxn1	29145	chr12	2528915	2529215	-4997	1.54	1.43E-02	2.17E-01	Intron (NM_017267/29635, intron 10 of 12)
Arhgap28	301709	chr9	116146108	116146408	76032	2.03	1.43E-02	2.17E-01	Intron (NM_001191815/301709, intron 1 of 17)
Enpep	64017	chr2	233714847	233715147	28719	-1.51	1.43E-02	2.17E-01	Intron (NM_022251/64017, intron 3 of 23)
Pak1	29431	chr1	162860351	162860651	92195	-1.72	1.43E-02	2.17E-01	Exon (NM_017198/29431, exon 7 of 15)
Zfp574	308434	chr1	81964469	81964769	12402	-1.34	1.43E-02	2.17E-01	Downstream (2-3kb)
Plekhd1	500685	chr6	104456807	104457107	116693	-1.62	1.43E-02	2.17E-01	Distal Intergenic
Capza2	493810	chr4	44945534	44945834	9244	-1.68	1.44E-02	2.17E-01	Intron (NM_001009180/493810, intron 1 of 9)
Csf1	78965	chr2	210598642	210598942	-48096	-1.64	1.44E-02	2.17E-01	Distal Intergenic
Whamm	293057	chr1	143433924	143434224	31938	1.36	1.44E-02	2.17E-01	Distal Intergenic
Zfp637	362425	chr4	149692513	149692813	-185076	1.59	1.44E-02	2.17E-01	Distal Intergenic
Bach2	313125	chr5	47425538	47425838	-120176	2.22	1.44E-02	2.17E-01	Distal Intergenic
Ryk	140585	chr8	111380853	111381153	54499	-1.32	1.44E-02	2.17E-01	Intron (NM_080402/140585, intron 9 of 14)
Lpcat2b	289439	chr14	3351358	3351658	7642	0.71	1.44E-02	2.18E-01	Distal Intergenic
Camsap1	296580	chr3	3418036	3418336	15691	-1.53	1.44E-02	2.18E-01	Intron (NM_001168549/296580, intron 1 of 17)
Dipk2a	315891	chr8	102093022	102093322	66506	-1.43	1.44E-02	2.18E-01	Distal Intergenic
Wnt5b	282582	chr4	151512888	151513188	3706	-1.57	1.44E-02	2.18E-01	Intron (NM_001100489/282582, intron 3 of 4)
Pla2g4a	24653	chr13	67186568	67186868	19820	-1.65	1.44E-02	2.18E-01	Intron (NM_133551/24653, intron 1 of 17)
Rfx3	361746	chr1	246406194	246406494	-295652	-1.5	1.44E-02	2.18E-01	Distal Intergenic
Lifr	81680	chr2	56432113	56432413	-4313	-1.37	1.44E-02	2.18E-01	Distal Intergenic
Stx6	60562	chr13	72810528	72810828	6310	-1.69	1.45E-02	2.18E-01	Intron (NM_031665/60562, intron 1 of 7)
Gtf3c5	362095	chr3	7174049	7174349	-9382	-1.36	1.45E-02	2.18E-01	Distal Intergenic
Nudt5	361274	chr17	76401525	76401825	8469	-1.36	1.45E-02	2.18E-01	Intron (NM_001007733/361274, intron 1 of 9)
Myoz2	295426	chr2	227262357	227262657	-54773	-1.76	1.45E-02	2.18E-01	Intron (NM_001191963/499702, intron 4 of 4)
Parl	287979	chr11	84528636	84528936	11268	2.44	1.45E-02	2.18E-01	Intron (NM_001035249/287979, intron 4 of 12)
Mob2	499288	chr1	215026151	215026451	-15238	-1.75	1.45E-02	2.18E-01	Distal Intergenic
Efna5	116683	chr9	110056671	110056971	272716	-2.02	1.45E-02	2.18E-01	Downstream (<1kb)
Sema3c	296787	chr4	14788437	14788737	-300212	1.87	1.45E-02	2.18E-01	Distal Intergenic
Gba3	289687	chr14	64686774	64687074	-209978	1.44	1.45E-02	2.18E-01	Distal Intergenic
Akr1c19	307096	chr17	69650963	69651263	-2907	1.98	1.45E-02	2.18E-01	Promoter (2-3kb)
Mef2a	309957	chr1	128294532	128294832	46431	-1.73	1.45E-02	2.18E-01	Intron (NM_001014035/309957, intron 2 of 10)
Cysltr2	170926	chr15	54757215	54757515	148688	1.74	1.45E-02	2.18E-01	Distal Intergenic
Rai14	294804	chr2	60593714	60594014	89437	-1.63	1.45E-02	2.18E-01	Intron (NM_001011947/294804, intron 3 of 16)
Slc9a8	311651	chr3	164161967	164162267	-12090	-1.63	1.45E-02	2.18E-01	Distal Intergenic
Irak4	300177	chr7	135803525	135803825	0	0.62	1.45E-02	2.18E-01	Promoter (<=1kb)
Bhlhe23	499952	chr3	176274107	176274407	7804	-1.38	1.45E-02	2.18E-01	Distal Intergenic
Zfp322a	680201	chr17	44028995	44029295	3728	-1.51	1.45E-02	2.18E-01	Intron (NM_001135084/680201, intron 2 of 3)
Tm6sf1	361600	chr1	143680050	143680350	22443	-1.48	1.45E-02	2.18E-01	Intron (NM_001108490/361600, intron 3 of 10)
Gabpb1l	364738	chr17	52851348	52851648	-87332	-1.49	1.45E-02	2.18E-01	Distal Intergenic

Tcerg1	307474	chr18	36829206	36829506	144	1.11	1.45E-02	2.18E-01	Promoter (<=1kb)
Cep57l1	294519	chr20	46599084	46599384	67396	2	1.46E-02	2.18E-01	Distal Intergenic
Cav1	25404	chr4	44674906	44675206	76349	-1.17	1.46E-02	2.18E-01	Distal Intergenic
Il6st	25205	chr2	44285012	44285312	-4763	-1.65	1.46E-02	2.18E-01	Distal Intergenic
Cdr2	308958	chr1	190914363	190914663	0	1.22	1.46E-02	2.18E-01	Promoter (<=1kb)
Fars2	306879	chr17	29248334	29248634	190034	-1.83	1.46E-02	2.18E-01	Intron (NM_001013139/306879, intron 5 of 8)
Chsy1	292999	chr1	127026020	127026320	15406	-1.86	1.46E-02	2.18E-01	Intron (NM_001106268/292999, intron 2 of 2)
Sema3c	296787	chr4	14459390	14459690	28535	-1.86	1.46E-02	2.18E-01	Intron (NM_001106578/296787, intron 1 of 16)
Ccr9	282832	chr8	132830565	132830865	2474	-1.86	1.46E-02	2.18E-01	Promoter (2-3kb)
Cers5	366984	chr7	141459359	141459659	-35016	1.69	1.46E-02	2.18E-01	Intron (NM_001191615/300228, intron 3 of 9)
Apex2	317628	chrX	23145589	23145889	-196	0.91	1.46E-02	2.18E-01	Promoter (<=1kb)
Mapkap1	296648	chr3	13596140	13596440	111160	1.16	1.46E-02	2.18E-01	Intron (NM_001011964/296648, intron 6 of 11)
Nf2	25744	chr14	85079098	85079398	9125	-1.56	1.46E-02	2.19E-01	Intron (NM_013193/25744, intron 1 of 16)
Emc2	362905	chr7	82338698	82338998	59	0.68	1.46E-02	2.19E-01	Promoter (<=1kb)
Top1	64550	chr3	156635699	156635999	11	0.87	1.46E-02	2.19E-01	Promoter (<=1kb)
Mrpl35	297334	chr4	99696554	99696854	49706	-1.51	1.46E-02	2.19E-01	Intron (NM_001108633/362384, intron 3 of 6)
Zfand4	286998	chr4	148233306	148233606	34317	-1.42	1.46E-02	2.19E-01	Exon (NM_173332/286998, exon 5 of 9)
Zc3h15	362154	chr3	71025445	71025745	4911	-1.5	1.46E-02	2.19E-01	Intron (NM_001010963/362154, intron 1 of 9)
Npas1	308387	chr1	78483025	78483325	-42287	-2.14	1.46E-02	2.19E-01	Intron (NM_001271132/306400, intron 3 of 6)
Tdg	114521	chr7	27187095	27187395	26281	-1.47	1.46E-02	2.19E-01	Distal Intergenic
Mir33	100314214	chr7	123417138	123417438	-14174	1.24	1.47E-02	2.19E-01	Intron (NM_001033694/300095, intron 10 of 19)
Pvr	25066	chr1	80828482	80828782	6930	-1.43	1.47E-02	2.19E-01	Exon (NM_017076/25066, exon 4 of 8)
Setd5	297514	chr4	145028870	145029170	11262	-1.49	1.47E-02	2.19E-01	Intron (NM_001106614/297514, intron 1 of 23)
Cdh17	117048	chr5	25371378	25371678	-19558	-1.23	1.47E-02	2.19E-01	Distal Intergenic
Taf1a	360893	chr13	101737598	101737898	-32380	-1.55	1.47E-02	2.19E-01	Distal Intergenic
Myo10	310178	chr2	77956986	77957286	88554	-1.39	1.47E-02	2.19E-01	Intron (NM_001107657/310178, intron 2 of 39)
Abtb1	297432	chr4	120734371	120734671	50136	-1.39	1.47E-02	2.19E-01	Intron (NM_138502/29254, intron 3 of 7)
Khdrbs3	64015	chr7	111824284	111824584	1792466	0.85	1.47E-02	2.19E-01	Distal Intergenic
Dnm2	25751	chr8	22496699	22496999	37759	-1.43	1.47E-02	2.19E-01	Intron (NM_013199/25751, intron 2 of 20)
Vldlr	25696	chr1	245164336	245164636	-73100	-1.66	1.47E-02	2.19E-01	Distal Intergenic
Llgl1	54265	chr10	46927626	46927926	-13083	2.07	1.47E-02	2.19E-01	3' UTR
Nudt14	299346	chr6	137741390	137741690	3453	-1.65	1.47E-02	2.19E-01	Exon (NM_001106760/299346, exon 4 of 5)
Adprm	287406	chr10	53590965	53591265	4494	-1.99	1.47E-02	2.19E-01	Intron (NM_001009246/287406, intron 2 of 4)
Yars1	313047	chr5	147390858	147391158	15508	-1.23	1.47E-02	2.19E-01	Intron (NM_001025696/313047, intron 4 of 12)
Wwtr1	295062	chr2	147619066	147619366	73667	-1.92	1.47E-02	2.19E-01	Intron (NM_001024869/295062, intron 3 of 6)
Slx4ip	499895	chr3	129989383	129989683	103557	1.96	1.47E-02	2.19E-01	Intron (NM_001271320/499895, intron 2 of 7)
Npas2	316351	chr9	45967983	45968283	66242	-1.21	1.47E-02	2.19E-01	Intron (NM_001108214/316351, intron 1 of 20)
Arnt2	25243	chr1	146555923	146556223	0	1.26	1.47E-02	2.19E-01	Promoter (<=1kb)
Camk2d	24246	chr2	230874511	230874811	-26315	-1.29	1.47E-02	2.19E-01	Distal Intergenic
Mir762	104797225	chr1	199158688	199158988	0	1.08	1.48E-02	2.19E-01	Promoter (<=1kb)
Dld	298942	chr6	50592878	50593178	25516	1.21	1.48E-02	2.19E-01	Distal Intergenic
Mical2	365352	chr1	177106969	177107269	13582	-1.68	1.48E-02	2.19E-01	Intron (NM_001139508/365352, intron 1 of 17)
Rcor2	305811	chr1	222525539	222525839	6278	2.08	1.48E-02	2.19E-01	Downstream (1-2kb)
Fndc1	308099	chr1	47605237	47605537	-20562	1.03	1.48E-02	2.19E-01	Distal Intergenic
Mirlet7a1	100314213	chr17	16395067	16395367	-22486	-1.22	1.48E-02	2.19E-01	Distal Intergenic
Slc14a2	54302	chr18	75452791	75453091	-511402	1.22	1.48E-02	2.19E-01	Distal Intergenic
Ccdc152	499536	chr2	53140201	53140501	-1928	1.65	1.48E-02	2.19E-01	Promoter (1-2kb)
Cd44	25406	chr3	92761255	92761555	22103	-1.66	1.48E-02	2.19E-01	Intron (NM_012924/25406, intron 1 of 19)
Snapin	295217	chr2	189887040	189887340	-6292	-1.38	1.48E-02	2.20E-01	Downstream (<1kb)
Rap2b	170923	chr2	151545882	151546182	-139069	-1.55	1.48E-02	2.20E-01	Distal Intergenic
Slc52a2	362942	chr7	117619693	117619993	14643	1.7	1.48E-02	2.20E-01	Distal Intergenic
Xylt2	64134	chr10	82399143	82399443	42	1.24	1.48E-02	2.20E-01	Promoter (<=1kb)
Hivep1	117140	chr17	22403075	22403375	0	1.02	1.48E-02	2.20E-01	Promoter (<=1kb)
Lmo7	361084	chr15	86311396	86311696	68248	-1.54	1.49E-02	2.20E-01	Intron (NM_001001515/361084, intron 1 of 30)
Smad4	50554	chr18	69671207	69671507	-13834	2.07	1.49E-02	2.20E-01	Distal Intergenic
Gskip	362778	chr6	129519252	129519552	-161	0.7	1.49E-02	2.20E-01	Promoter (<=1kb)
Msc	312897	chr5	4446164	4446464	490937	1.87	1.49E-02	2.20E-01	Distal Intergenic
Ctnnd1	311163	chr3	72025216	72025516	27474	-1.5	1.49E-02	2.20E-01	Intron (NM_001107740/311163, intron 2 of 17)
Shisa4	360848	chr13	52196908	52197208	0	0.79	1.49E-02	2.20E-01	Promoter (<=1kb)
Mreg	501162	chr9	79562413	79562713	-17389	-1.42	1.49E-02	2.20E-01	Distal Intergenic
Sort1	83576	chr2	211117499	211117799	39165	2.15	1.49E-02	2.20E-01	Intron (NM_031767/83576, intron 4 of 19)
Atad1	309532	chr1	251378792	251379092	46	0.63	1.49E-02	2.20E-01	Promoter (<=1kb)
Ccne2	362485	chr5	24434420	24434720	-152	0.94	1.49E-02	2.20E-01	Promoter (<=1kb)
Pitpnb	114561	chr12	51484080	51484380	-142393	2.37	1.49E-02	2.20E-01	Distal Intergenic
RT1-A2	24974	chr20	5346583	5346883	-28128	-1.35	1.49E-02	2.20E-01	Distal Intergenic
T2	681288	chr1	52962355	52962655	0	-1.33	1.49E-02	2.20E-01	Promoter (<=1kb)
LOC100910620	100910620	chr5	74517288	74517588	-74616	-1.54	1.49E-02	2.20E-01	Distal Intergenic
Nfia	25492	chr5	116416350	116416650	-5248	0.71	1.49E-02	2.20E-01	Distal Intergenic
Lrch1	502020	chr15	56888923	56889223	81142	-1.68	1.49E-02	2.20E-01	Intron (NM_001134727/502020, intron 1 of 18)
Dhx40	287595	chr10	74178484	74178784	-59452	1.56	1.49E-02	2.20E-01	Distal Intergenic
Fendrr	104845258	chr19	53012478	53012778	-1969	0.79	1.49E-02	2.20E-01	Promoter (1-2kb)
Lnx2	360761	chr12	9740891	9741191	12405	-1.59	1.50E-02	2.20E-01	Intron (NM_001108329/360761, intron 1 of 9)
Tppp	361466	chr1	31835010	31835310	55468	0.7	1.50E-02	2.20E-01	Distal Intergenic
Afap1	140935	chr14	79951334	79951634	36281	-1.46	1.50E-02	2.20E-01	Intron (NM_080900/140935, intron 9 of 16)
Txndc17	287474	chr10	58716911	58717211	-59581	2.08	1.50E-02	2.20E-01	Distal Intergenic
Bmp4	25296	chr15	20833606	20833906	-53804	2.04	1.50E-02	2.20E-01	Distal Intergenic
Hspa5	25617	chr3	13719427	13719727	-118577	0.85	1.50E-02	2.20E-01	Distal Intergenic
Col3a1	84032	chr9	52024044	52024344	749	0.73	1.50E-02	2.20E-01	Promoter (<=1kb)
Chd1	308215	chr1	57690439	57690739	-2089	0.69	1.50E-02	2.20E-01	Promoter (2-3kb)
Sh2d4a	306376	chr16	23383450	23383750	-226625	1.04	1.50E-02	2.20E-01	Distal Intergenic
Baz1b	368002	chr12	24531368	24531668	5183	-1.76	1.50E-02	2.20E-01	Intron (NM_001191916/368002, intron 1 of 18)
Slc41a3	641603	chr4	123430407	123430707	64035	0.85	1.50E-02	2.21E-01	Distal Intergenic
Phtf2	296762	chr4	10949845	10950145	126587	0.71	1.50E-02	2.21E-01	Distal Intergenic
Pou3f3	192109	chr9	49496963	49497263	18023	1.8	1.50E-02	2.21E-01	Distal Intergenic
Rfc3	288414	chr12	1360573	1360873	-99708	-1.27	1.50E-02	2.21E-01	Distal Intergenic
Lsm11	501688	chr10	30960135	30960435	81191	-1.47	1.50E-02	2.21E-01	Intron (NM_001002022/360515, intron 1 of 9)
Umps	288051	chr11	70050015	70050315	15834	1.57	1.50E-02	2.21E-01	Distal Intergenic
Cadm1	363058	chr8	51978799	51979099	119893	1.62	1.50E-02	2.21E-01	Intron (NM_001012201/363058, intron 1 of 11)
Bmi1	307151	chr17	85372401	85372701	7918	1.97	1.50E-02	2.21E-01	Distal Intergenic
Mlana	293890	chr1	247848410	247848710	-21046	1.61	1.50E-02	2.21E-01	Distal Intergenic
Il6r	24499	chr2	189198999	189199299	55253	-1.39	1.51E-02	2.21E-01	Distal Intergenic
Ackr3	84348	chr9	97516849	97517149	160925	1.5	1.51E-02	2.21E-01	Distal Intergenic
Ncam1	24586	chr8	53980849	53981149	153582	2.08	1.51E-02	2.21E-01	Intron (NM_031521/24586, intron 1 of 18)
Edn1	24323	chr17	22200599	22200899	-57275	1.64	1.51E-02	2.21E-01	Distal Intergenic

Rbpj	679028	chr14	59780337	59780637	-44887	-1.77	1.51E-02	2.21E-01	Distal Intergenic
Foxa3	25100	chr1	79922380	79922680	7539	1.78	1.51E-02	2.21E-01	Exon (NM_017077/25100, exon 2 of 2)
Setd5	297514	chr4	145031826	145032126	14218	-1.72	1.51E-02	2.22E-01	Intron (NM_001106614/297514, intron 1 of 23)
Ube2v1	296390	chr3	164366238	164366538	146	1.02	1.51E-02	2.22E-01	Promoter (<=1kb)
LOC100911367	100911367	chr18	31785482	31785782	-6721	-1.49	1.51E-02	2.22E-01	Intron (NM_012576/24413, intron 1 of 7)
Dusp1	114856	chr10	16944411	16944711	-25931	1.1	1.52E-02	2.22E-01	Distal Intergenic
Smad2	29357	chr18	72402130	72402430	-147789	1.32	1.52E-02	2.22E-01	Intron (NM_001127375/679155, intron 3 of 5)
Acsl1	25288	chr16	49047781	49048081	-65801	-1.61	1.52E-02	2.22E-01	Distal Intergenic
Mlnr	252859	chr19	55176379	55176679	4720	-1.26	1.52E-02	2.22E-01	Downstream (1-2kb)
Cyp4v3	266761	chr16	50111812	50112112	9	0.71	1.52E-02	2.22E-01	Promoter (<=1kb)
Fbxw11	303024	chr10	17563200	17563500	20826	-1.7	1.52E-02	2.22E-01	Intron (NM_001106993/303024, intron 1 of 11)
Mx2	286918	chr11	38051052	38051352	15539	-1.7	1.52E-02	2.22E-01	Exon (NM_134350/286918, exon 9 of 15)
Chd2	308738	chr1	134958615	134958915	-87447	-1	1.52E-02	2.22E-01	Distal Intergenic
Sestd1	295678	chr3	64178477	64178777	-65145	0.9	1.52E-02	2.22E-01	Distal Intergenic
Mef2b	498607	chr16	21010032	21010332	6831	2.09	1.52E-02	2.22E-01	Intron (NM_001017507/498607, intron 1 of 8)
Ptgir	292661	chr1	78838877	78839177	5428	-1.71	1.52E-02	2.22E-01	Distal Intergenic
Gan	307893	chr19	49690559	49690859	-4720	2.04	1.52E-02	2.22E-01	Distal Intergenic
Camta1	362665	chr5	169017858	169018158	-563	0.78	1.52E-02	2.22E-01	Promoter (<=1kb)
Pdcd10	494345	chr2	173998262	173998562	14114	-1.66	1.52E-02	2.22E-01	Intron (NM_001009542/494345, intron 1 of 7)
Hnrnpa1	29578	chr7	144856457	144856757	-8883	-1.66	1.52E-02	2.22E-01	Distal Intergenic
Shc3	114858	chr17	13668622	13668922	-1598	-1.8	1.52E-02	2.22E-01	Promoter (1-2kb)
Rab5a	64633	chr14	72888954	72889254	0	0.94	1.53E-02	2.22E-01	Promoter (<=1kb)
Rsad2	65190	chr6	45670557	45670857	-1474	-1.75	1.53E-02	2.22E-01	Promoter (1-2kb)
Ptprf	360406	chr5	137118626	137118926	-13633	1.07	1.53E-02	2.22E-01	Distal Intergenic
Afap1l2	292130	chr1	277761617	277761917	65352	1.84	1.53E-02	2.23E-01	Intron (NM_001305184/292130, intron 4 of 19)
Emp2	360468	chr10	5435524	5435824	2276	-1.43	1.53E-02	2.23E-01	Promoter (2-3kb)
Kcnk5	364241	chr15	4633518	4633818	78915	1.97	1.53E-02	2.23E-01	Distal Intergenic
Cyp26b1	312495	chr4	116483571	116483871	-204956	-1.56	1.53E-02	2.23E-01	Intron (NM_001109246/500233, intron 19 of 22)
Pdia6	286906	chr6	42557656	42557956	-10313	1.9	1.53E-02	2.23E-01	Distal Intergenic
Vangl1	690366	chr2	204606302	204606602	19192	-1.37	1.53E-02	2.23E-01	Intron (NM_001109584/690366, intron 3 of 7)
Clstn3	171393	chr4	157077763	157078063	0	-1.44	1.53E-02	2.23E-01	Promoter (<=1kb)
Ppp1cc	24669	chr12	39852450	39852750	29280	-1.58	1.53E-02	2.23E-01	Distal Intergenic
B3gnt2	305571	chr14	107604305	107604605	12556	-1.64	1.53E-02	2.23E-01	Intron (NM_001107240/305571, intron 2 of 2)
B4galt3	494342	chr13	89636510	89636810	-7070	-1.45	1.53E-02	2.23E-01	Distal Intergenic
Me2	307270	chr18	69786817	69787117	-2718	2.1	1.53E-02	2.23E-01	Promoter (2-3kb)
Fzd1	58868	chr4	26700980	26701280	230116	-1.2	1.54E-02	2.23E-01	Distal Intergenic
Pex7	308718	chr1	15374512	15374812	0	1.14	1.54E-02	2.23E-01	Promoter (<=1kb)
Bcl11a	305589	chr14	108825461	108825761	-1178	0.78	1.54E-02	2.23E-01	Promoter (1-2kb)
Nectin3	288124	chr11	57993164	57993464	84	1.12	1.54E-02	2.23E-01	Promoter (<=1kb)
Tm4sf19	288044	chr11	71614156	71614456	4652	-1.75	1.54E-02	2.23E-01	Intron (NM_001105873/288044, intron 1 of 4)
Tsfm	679068	chr7	70319091	70319391	0	0.54	1.54E-02	2.23E-01	Promoter (<=1kb)
Notch1	25496	chr3	3963781	3964081	-12766	1.21	1.54E-02	2.23E-01	Distal Intergenic
Hsd11b1	25116	chr13	111971868	111972168	435	-1.33	1.54E-02	2.23E-01	Promoter (<=1kb)
Ash1l	310638	chr2	188278784	188279084	25406	-1.74	1.54E-02	2.23E-01	Intron (NM_001107689/310638, intron 2 of 25)
Loxl3	312478	chr4	113875070	113875370	7672	-1.74	1.54E-02	2.23E-01	Intron (NM_001107866/312478, intron 4 of 13)
Ccdc82	300359	chr8	11984913	11985213	96393	-1.74	1.54E-02	2.23E-01	Distal Intergenic
Snrpc	361808	chr20	7403372	7403672	-5729	-1.6	1.54E-02	2.23E-01	Distal Intergenic
Slc24a3	85267	chr3	139333205	139333505	-437	0.8	1.55E-02	2.24E-01	Promoter (<=1kb)
Dgkb	54248	chr6	57517037	57517337	25	-1.35	1.55E-02	2.24E-01	Promoter (<=1kb)
Ppat	117544	chr14	33574449	33574749	-5792	-1.64	1.55E-02	2.24E-01	Intron (NM_080910/140946, intron 3 of 9)
Mapkap1	296648	chr3	13601347	13601647	116367	1.81	1.55E-02	2.24E-01	Intron (NM_001011964/296648, intron 6 of 11)
Vac14	307842	chr19	40987202	40987502	41704	1.77	1.55E-02	2.24E-01	Intron (NM_177930/307842, intron 12 of 18)
Nsmce2	299957	chr7	99740281	99740581	62946	-0.86	1.55E-02	2.24E-01	Intron (NM_001024876/299957, intron 3 of 6)
Robo3	315564	chr8	39931655	39931955	-24177	1.1	1.55E-02	2.24E-01	Distal Intergenic
Slc49a4	303902	chr11	68198667	68198967	0	0.86	1.55E-02	2.24E-01	Promoter (<=1kb)
Oasl2	304549	chr12	47477471	47477771	5176	-1.66	1.55E-02	2.24E-01	Intron (NM_001009682/304549, intron 3 of 5)
Socs5	500616	chr6	10711485	10711785	37114	-1.33	1.55E-02	2.24E-01	Distal Intergenic
Trpc1	89821	chr8	103554403	103554703	-22	1.07	1.55E-02	2.24E-01	Promoter (<=1kb)
Arhgap10	688429	chr19	34177466	34177766	37469	-1.32	1.55E-02	2.24E-01	Intron (NM_001109501/688429, intron 1 of 22)
Tasp1	311468	chr3	133143054	133143354	-20299	1.62	1.55E-02	2.24E-01	Distal Intergenic
Fzd1	58868	chr4	26175416	26175716	-295148	2.09	1.55E-02	2.24E-01	Intron (NM_001108617/362316, intron 7 of 12)
Lrrc49	300763	chr8	65453125	65453425	134002	-1.56	1.55E-02	2.24E-01	Distal Intergenic
Arsj	311013	chr2	230166565	230166865	3551	-1.56	1.55E-02	2.24E-01	Intron (NM_001047887/311013, intron 1 of 1)
Ptprg	171357	chr15	13327559	13327859	305980	-2	1.56E-02	2.24E-01	Intron (NM_134356/171357, intron 2 of 41)
Rras2	365355	chr1	179009805	179010105	152	1.32	1.56E-02	2.24E-01	Promoter (<=1kb)
Eipr1	362721	chr6	48017331	48017631	-4140	-1.2	1.56E-02	2.24E-01	Distal Intergenic
Pdgfra	25267	chr14	35572704	35573004	8027	-1.32	1.56E-02	2.24E-01	Intron (NM_012802/25267, intron 1 of 22)
Hivep2	29721	chr1	8133815	8134115	4461	-1.62	1.56E-02	2.24E-01	Intron (NM_024137/29721, intron 1 of 7)
Ewsr1	289752	chr4	24538859	24539159	-73028	-1.31	1.56E-02	2.24E-01	Distal Intergenic
Pdlim5	64353	chr2	247861204	247861504	126958	1.81	1.56E-02	2.24E-01	Intron (NM_053326/64353, intron 7 of 12)
Efcab9	688481	chr10	17327110	17327410	87841	0.97	1.56E-02	2.24E-01	Distal Intergenic
Cyp2s1	308445	chr1	82596230	82596530	13820	-1.68	1.56E-02	2.25E-01	3' UTR
Spaca6	688452	chr1	59718517	59718817	13519	-1.5	1.56E-02	2.25E-01	Downstream (1-2kb)
Fabp12	499570	chr2	93719829	93720129	-31103	1.31	1.56E-02	2.25E-01	Distal Intergenic
Nsd2	680537	chr14	82196644	82196944	-25164	1.31	1.56E-02	2.25E-01	Distal Intergenic
Crebbp	54244	chr10	11638642	11638942	43598	-1.42	1.56E-02	2.25E-01	Intron (NM_133381/54244, intron 2 of 30)
Mir423	100314264	chr10	63104837	63105137	6471	-1.63	1.57E-02	2.25E-01	Intron (NM_001037189/303346, intron 2 of 6)
Nup153	25281	chr17	18269745	18270045	-88667	-1.63	1.57E-02	2.25E-01	Exon (NM_001107462/308173, exon 9 of 37)
Dlg1	25252	chr11	72367014	72367314	11669	-1.35	1.57E-02	2.25E-01	Intron (NM_012788/25252, intron 4 of 29)
Hnf4a	25735	chr3	159899375	159899675	-2766	-1.59	1.57E-02	2.25E-01	Promoter (2-3kb)
Lsm14a	361554	chr1	90245774	90246074	169	0.79	1.57E-02	2.25E-01	Promoter (<=1kb)
Kdm2b	304495	chr12	39032379	39032679	10379	-1.66	1.57E-02	2.25E-01	Intron (NM_001100679/304495, intron 4 of 28)
Dnajc6	313409	chr5	120330164	120330464	-10271	0.97	1.57E-02	2.25E-01	Distal Intergenic
Carmil1	306941	chr17	43130015	43130315	79054	-1.43	1.57E-02	2.25E-01	Intron (NM_001191692/306941, intron 2 of 37)
Mapk1	116590	chr11	88254176	88254476	18818	-1.56	1.57E-02	2.25E-01	Intron (NM_053842/116590, intron 1 of 8)
Diras2	291006	chr17	12737863	12738163	-24589	-1.75	1.57E-02	2.25E-01	Distal Intergenic
Slc31a1	171135	chr5	78173737	78174037	-48467	1.77	1.57E-02	2.25E-01	Distal Intergenic
Pomk	306549	chr16	70876645	70876945	17365	1.32	1.57E-02	2.25E-01	Distal Intergenic
Echdc3	684538	chr17	75982625	75982925	-19379	-0.86	1.57E-02	2.25E-01	Distal Intergenic
Pax9	362741	chr6	77568465	77568765	-38940	-2.02	1.57E-02	2.25E-01	Distal Intergenic
Six1	114634	chr6	95955570	95955870	-21274	2.5	1.57E-02	2.25E-01	Distal Intergenic
Cdh11	84407	chr19	2443539	2443839	50480	-1.7	1.57E-02	2.25E-01	Intron (NM_053392/84407, intron 1 of 12)
LOC100911360	100911360	chr8	73282623	73282923	252072	1.88	1.57E-02	2.25E-01	Distal Intergenic
Gls	24398	chr9	54213084	54213384	317	0.87	1.57E-02	2.25E-01	Promoter (<=1kb)

Mymk	296601	chr3	5642427	5642727	-25202	-1.2	1.57E-02	2.25E-01	Distal Intergenic
Rtn4	83765	chr14	114134258	114134558	7292	-1.56	1.57E-02	2.25E-01	Intron (NM_031831/83765, intron 1 of 8)
Txndc17	287474	chr10	58707436	58707736	-69056	1.85	1.57E-02	2.25E-01	Distal Intergenic
Baz1a	314126	chr6	75885498	75885798	-11644	0.59	1.58E-02	2.25E-01	Distal Intergenic
Lbh	683626	chr6	24154572	24154872	-5139	2.03	1.58E-02	2.25E-01	Distal Intergenic
Adamts12	294809	chr2	61072585	61072885	33513	1.88	1.58E-02	2.25E-01	Intron (NM_001106420/294809, intron 2 of 23)
Gzmk	29165	chr2	45047782	45048082	29072	-1.95	1.58E-02	2.25E-01	Distal Intergenic
Slc4a7	117955	chr15	11793548	11793848	119068	1.77	1.58E-02	2.25E-01	Distal Intergenic
Cerk	300129	chr7	126975306	126975606	82450	1.55	1.58E-02	2.25E-01	Distal Intergenic
Actr3	81732	chr13	41725736	41726036	12586	-1.67	1.58E-02	2.25E-01	Intron (NM_001177819/100362110, intron 2 of 8)
Fbxl5	305424	chr14	71839303	71839603	-13127	-1.59	1.58E-02	2.25E-01	Distal Intergenic
Cblb	171136	chr11	51196255	51196555	6148	-1.57	1.58E-02	2.25E-01	Intron (NM_133601/171136, intron 2 of 17)
Snrpa1	100361269	chr1	126965270	126965570	3881	-1.48	1.58E-02	2.25E-01	Intron (NM_001271323/100361269, intron 3 of 8)
RGD1563049	298493	chr5	142132449	142132749	-19462	2.08	1.58E-02	2.25E-01	Distal Intergenic
Il10rb	304091	chr11	31560471	31560771	0	0.79	1.58E-02	2.25E-01	Promoter (<=1kb)
Fam49b	299909	chr7	104641440	104641740	55	1.09	1.58E-02	2.25E-01	Promoter (<=1kb)
Rbms1	362138	chr3	46929584	46929884	95244	-1.57	1.58E-02	2.25E-01	Intron (NM_001012184/362138, intron 1 of 13)
Msi1	259272	chr12	46939208	46939508	-18294	-1.42	1.58E-02	2.25E-01	Distal Intergenic
Metrnl	316842	chr10	110912870	110913170	19609	0.58	1.58E-02	2.25E-01	Distal Intergenic
G6pd	24377	chrX	156280500	156280800	5700	-1.68	1.58E-02	2.25E-01	Intron (NM_017006/24377, intron 2 of 12)
Ttc7b	362768	chr6	124518496	124518796	-84432	1.73	1.58E-02	2.25E-01	Distal Intergenic
Scn2b	25349	chr8	49424422	49424722	5419	-1.57	1.58E-02	2.25E-01	Intron (NM_012877/25349, intron 1 of 3)
Tra2a	500116	chr4	78840139	78840439	83742	-1.6	1.59E-02	2.25E-01	Distal Intergenic
Gask1b	310540	chr2	178697751	178698051	18710	1.32	1.59E-02	2.25E-01	Intron (NM_199105/310540, intron 2 of 4)
Entr1	306322	chr3	3833813	3834113	106	0.98	1.59E-02	2.26E-01	Promoter (<=1kb)
Rps27a	100912032	chr14	113964917	113965217	3167	-1.2	1.59E-02	2.26E-01	Intron (NM_001004254/305606, intron 14 of 14)
Ap3b1	309969	chr2	24024792	24025092	0	0.74	1.59E-02	2.26E-01	Promoter (<=1kb)
Mapk14	81649	chr20	5929838	5930138	-3165	-1.29	1.59E-02	2.26E-01	Distal Intergenic
Podxl	192181	chr4	58964866	58965166	-89113	-1.61	1.59E-02	2.26E-01	Distal Intergenic
Cotl1	361422	chr19	52499069	52499369	64	0.55	1.59E-02	2.26E-01	Promoter (<=1kb)
Il17b	116472	chr18	56997550	56997850	-13725	-1.34	1.59E-02	2.26E-01	Distal Intergenic
Faap20	362678	chr5	172586497	172586797	-62153	-1.64	1.59E-02	2.26E-01	Distal Intergenic
Rapgef3	59326	chr7	139262156	139262456	-7960	1.64	1.59E-02	2.26E-01	Distal Intergenic
Zfp472	314587	chr7	15271538	15271838	29389	0.78	1.60E-02	2.26E-01	Distal Intergenic
Sntb2	689421	chr19	39121666	39121966	-4623	1.17	1.60E-02	2.26E-01	Distal Intergenic
Atad2b	500625	chr6	29012021	29012321	13941	0.86	1.60E-02	2.26E-01	Intron (NM_001025151/500625, intron 2 of 18)
Acvr1b	29381	chr7	142817197	142817497	23	1.24	1.60E-02	2.26E-01	Promoter (<=1kb)
Ahsp	293522	chr1	199730646	199730946	10608	-1.4	1.60E-02	2.26E-01	Distal Intergenic
Klf10	81813	chr7	77211163	77211463	-49067	-1.47	1.60E-02	2.26E-01	Distal Intergenic
Ctnnbip1	503000	chr5	166464374	166464674	122	1.15	1.60E-02	2.26E-01	Promoter (<=1kb)
Atp5pd	641434	chr10	103972850	103973150	-337	1.06	1.60E-02	2.26E-01	Promoter (<=1kb)
Myo5a	25017	chr8	81983044	81983344	3321	0.77	1.60E-02	2.26E-01	Intron (NM_022178/25017, intron 4 of 43)
Lsr	64355	chr1	89429076	89429376	-30035	-1.14	1.60E-02	2.26E-01	Distal Intergenic
Eif4e3	297481	chr4	131905232	131905532	205560	1.45	1.60E-02	2.26E-01	Distal Intergenic
Golga4	501069	chr8	127176247	127176547	4611	1.78	1.60E-02	2.26E-01	Intron (NM_001106865/301056, intron 1 of 7)
Isca1	290985	chr17	5278620	5278920	-2807	1.9	1.60E-02	2.26E-01	Promoter (2-3kb)
Yod1	363982	chr13	47441067	47441367	0	0.76	1.60E-02	2.26E-01	Promoter (<=1kb)
Mir222	100314059	chrX	3632354	3632654	-51265	-1.62	1.60E-02	2.26E-01	Distal Intergenic
Dusp1	114856	chr10	16963283	16963583	-7059	1.86	1.60E-02	2.26E-01	Distal Intergenic
Sec24d	310843	chr2	227468291	227468591	12568	-1.42	1.60E-02	2.26E-01	Intron (NM_001309453/310843, intron 3 of 22)
Hsf2bp	499413	chr20	10779266	10779566	62804	-1.51	1.60E-02	2.26E-01	Intron (NM_001127683/499413, intron 7 of 8)
Ttyh3	304315	chr12	16084115	16084415	0	1.19	1.60E-02	2.26E-01	Promoter (<=1kb)
Nckap5l	315297	chr7	141098364	141098664	-4440	-1.42	1.60E-02	2.26E-01	Distal Intergenic
Ocrl	317576	chrX	134742217	134742517	0	0.91	1.60E-02	2.26E-01	Promoter (<=1kb)
Pde4d	24627	chr2	41441654	41441954	283672	1.14	1.61E-02	2.26E-01	Intron (NM_001113329/24627, intron 7 of 15)
Fat1	83720	chr16	50461383	50461683	40033	-1.45	1.61E-02	2.26E-01	Intron (NM_031819/83720, intron 2 of 26)
Plcd3	287745	chr10	91185965	91186265	0	0.63	1.61E-02	2.26E-01	Promoter (<=1kb)
Higd1a	140937	chr8	130491315	130491615	273	0.69	1.61E-02	2.26E-01	Promoter (<=1kb)
Map3k1	116667	chr2	43652580	43652880	-259376	-1.64	1.61E-02	2.26E-01	Distal Intergenic
Tgfb2	81809	chr13	104902966	104903266	237514	2.02	1.61E-02	2.26E-01	Distal Intergenic
Srp54a	116650	chr6	76000093	76000393	3450	-1.61	1.61E-02	2.26E-01	Intron (NM_053871/116650, intron 1 of 15)
Neurl1b	303019	chr10	17074836	17075136	3	1.14	1.61E-02	2.26E-01	Promoter (<=1kb)
Brsk1	499073	chr1	72726946	72727246	0	0.88	1.61E-02	2.27E-01	Promoter (<=1kb)
Pdgfc	79429	chr2	180005635	180005935	53408	-1.48	1.61E-02	2.27E-01	Intron (NM_031317/79429, intron 1 of 5)
Sh3bp1	300067	chr7	120119694	120119994	-5753	1.78	1.61E-02	2.27E-01	3' UTR
Iqgap1	361598	chr1	142639571	142639871	-23898	-1.3	1.61E-02	2.27E-01	Distal Intergenic
Extl3	56819	chr15	48480947	48481247	-35355	-1.18	1.61E-02	2.27E-01	Distal Intergenic
Ppdpf	296470	chr3	176697604	176697904	6275	1.72	1.61E-02	2.27E-01	Downstream (<1kb)
Lrrc8c	289443	chr14	5374933	5375233	3465	-1.65	1.62E-02	2.27E-01	Intron (NM_001037179/289443, intron 1 of 3)
Fhl3	313582	chr5	142685666	142685966	-3138	-1.64	1.62E-02	2.27E-01	Distal Intergenic
Asap1	314961	chr7	104843813	104844113	106977	-1.64	1.62E-02	2.27E-01	Intron (NM_001044245/314961, intron 1 of 27)
Rarg	685072	chr7	143862805	143863105	101	0.64	1.62E-02	2.27E-01	Promoter (<=1kb)
Chn1	84030	chr3	60672427	60672727	-116471	-1.28	1.62E-02	2.27E-01	Distal Intergenic
Epdr1	291180	chr17	47404034	47404334	6476	-1.61	1.62E-02	2.27E-01	Intron (NM_001007625/291180, intron 1 of 2)
Spop	287643	chr10	83218710	83219010	-12399	-1.02	1.62E-02	2.27E-01	Distal Intergenic
Mylk	288057	chr11	69097060	69097360	125798	-1.65	1.62E-02	2.27E-01	Intron (NM_001105874/288057, intron 16 of 31)
Cdk13	306998	chr17	49844472	49844772	11278	-1.49	1.62E-02	2.27E-01	Intron (NM_001271295/306998, intron 1 of 13)
Chrna1	79557	chr3	60407545	60407845	52879	0.75	1.62E-02	2.27E-01	Distal Intergenic
Dlc1	58834	chr16	59144401	59144701	-52666	-1.38	1.62E-02	2.27E-01	Distal Intergenic
Abhd5	316122	chr8	130981470	130981770	8248	-1.56	1.62E-02	2.27E-01	Intron (NM_212524/316122, intron 1 of 6)
Med4	306030	chr15	55430606	55430906	24063	-1.64	1.62E-02	2.27E-01	Intron (NM_001106049/290365, intron 1 of 3)
Mllt3	114510	chr5	106257858	106258158	-55297	-1.64	1.62E-02	2.27E-01	Distal Intergenic
Atxn1	25049	chr17	19274291	19274591	113297	1.4	1.62E-02	2.27E-01	Intron (NM_012726/25049, intron 2 of 6)
Socs6	307200	chr18	85940890	85941190	39643	1.44	1.62E-02	2.27E-01	Distal Intergenic
Dok6	498898	chr18	87628619	87628919	-750477	0.7	1.62E-02	2.27E-01	Distal Intergenic
Dusp6	116663	chr7	41784763	41785063	309600	1.3	1.62E-02	2.27E-01	Distal Intergenic
Robo3	315564	chr8	39932512	39932812	-25034	-1.65	1.62E-02	2.27E-01	Distal Intergenic
RGD1307461	300990	chr8	116119835	116120135	24984	0.72	1.62E-02	2.27E-01	Distal Intergenic
Csnk1e	58822	chr7	120699697	120699997	-27347	1.08	1.62E-02	2.27E-01	Distal Intergenic
Eif2s2	296302	chr3	150396689	150396989	15190	-1.88	1.62E-02	2.27E-01	Intron (NM_199380/296302, intron 6 of 8)
Fdxr	79122	chr10	103826138	103826438	0	0.77	1.62E-02	2.27E-01	Promoter (<=1kb)
Rap2c	302495	chrX	138237369	138237669	-212	1.03	1.63E-02	2.27E-01	Promoter (<=1kb)
Baz1b	368002	chr12	24519624	24519924	16927	-1.77	1.63E-02	2.27E-01	Intron (NM_001191916/368002, intron 3 of 18)
Ngf	310738	chr2	204891436	204891736	5234	-1.77	1.63E-02	2.27E-01	Intron (NM_001277055/310738, intron 1 of 2)

Plpp5	680466	chr16	71124810	71125110	206	0.71	1.63E-02	2.27E-01	Promoter (<=1kb)
Ipmk	171458	chr20	18473704	18474004	15824	-1.38	1.63E-02	2.27E-01	Intron (NM_134417/171458, intron 2 of 5)
Arf6	79121	chr6	91710620	91710920	13511	-1.67	1.63E-02	2.27E-01	Distal Intergenic
Jag1	29146	chr3	130108051	130108351	6430	-1.71	1.63E-02	2.27E-01	Intron (NM_019147/29146, intron 2 of 27)
Cdc14a	310806	chr2	219503298	219503598	-44953	1.5	1.63E-02	2.27E-01	Distal Intergenic
Plekha1	361659	chr1	201432444	201432744	2673	-1.4	1.63E-02	2.27E-01	Promoter (2-3kb)
Ptk2b	50646	chr15	42889220	42889520	58136	-1.08	1.63E-02	2.27E-01	Intron (NM_017318/50646, intron 1 of 30)
Wnt7a	114850	chr4	123070917	123071217	-30308	-1.33	1.63E-02	2.27E-01	Distal Intergenic
S100a11	445415	chr2	193675351	193675651	-191300	1.59	1.63E-02	2.27E-01	Distal Intergenic
Slirp	688717	chr6	111472888	111473188	-3580	-1.2	1.63E-02	2.27E-01	Intron (NM_001108718/362766, intron 1 of 5)
Kcns1	117023	chr3	160541632	160541932	19809	1.48	1.63E-02	2.27E-01	Intron (NM_001107800/311622, intron 10 of 10)
Vav2	296603	chr3	5975566	5975866	378	1.14	1.63E-02	2.27E-01	Promoter (<=1kb)
Tnrc6a	308971	chr1	193088308	193088608	11878	-1.53	1.63E-02	2.27E-01	Intron (NM_001107549/308971, intron 1 of 24)
Ube2v1	296390	chr3	164330945	164331245	35439	-1.53	1.63E-02	2.27E-01	Distal Intergenic
Pum1	362609	chr5	148800806	148801106	19539	-1.34	1.63E-02	2.27E-01	Intron (NM_001108684/362609, intron 2 of 21)
Papola	314417	chr6	129614732	129615032	5658	-1.28	1.63E-02	2.27E-01	Intron (NM_001108056/314417, intron 1 of 21)
Fchsd1	307482	chr18	31197118	31197418	-88103	-1.67	1.63E-02	2.27E-01	Distal Intergenic
Ttc7a	362696	chr6	10920877	10921177	8494	-1.75	1.63E-02	2.27E-01	Exon (NM_001100756/362696, exon 2 of 20)
Plod2	300901	chr8	100002918	100003218	25584	-1.44	1.63E-02	2.27E-01	Intron (NM_001142915/300901, intron 1 of 18)
Fam102b	365903	chr2	211831417	211831717	120	0.99	1.64E-02	2.27E-01	Promoter (<=1kb)
Hdac1l	84576	chr9	84927982	84928282	-33383	-1.89	1.64E-02	2.27E-01	Distal Intergenic
LOC500846	500846	chr7	69213312	69213612	-108362	1.22	1.64E-02	2.27E-01	Distal Intergenic
Ackr3	84348	chr9	97356283	97356583	359	0.77	1.64E-02	2.27E-01	Promoter (<=1kb)
Dpep2	291984	chr19	37970070	37970370	167	0.87	1.64E-02	2.27E-01	Promoter (<=1kb)
Ppp6r2	300146	chr7	130188551	130188851	0	1.35	1.64E-02	2.28E-01	Promoter (<=1kb)
Trappc3	362599	chr5	144294139	144294439	12419	1.14	1.64E-02	2.28E-01	Intron (NM_001008376/362599, intron 4 of 4)
Uck2	304944	chr13	85415989	85416289	27687	-1.36	1.64E-02	2.28E-01	Intron (NM_001102408/304944, intron 1 of 6)
Minpp1	29688	chr1	251045440	251045740	0	0.86	1.64E-02	2.28E-01	Promoter (<=1kb)
Rpl3l	287122	chr10	14099492	14099792	4723	-1.89	1.64E-02	2.28E-01	Exon (NM_001191589/287122, exon 3 of 10)
Slc25a10	170943	chr10	109683497	109683797	17815	-1.89	1.64E-02	2.28E-01	Distal Intergenic
Crispld1	316482	chr5	286305	286605	424428	-1.89	1.64E-02	2.28E-01	Distal Intergenic
Pid1	501174	chr9	91885340	91885640	-201872	-1.89	1.64E-02	2.28E-01	Distal Intergenic
Dele1	307480	chr18	31386750	31387050	-9937	1.17	1.65E-02	2.28E-01	Distal Intergenic
Arfgef1	312915	chr5	8676533	8676833	10333	-1.52	1.65E-02	2.28E-01	Intron (NM_001277056/312915, intron 1 of 38)
Wt1	24883	chr3	95113883	95114183	-19530	1.5	1.65E-02	2.28E-01	Distal Intergenic
Kdm4d	689582	chr8	12924460	12924760	68891	-1.49	1.65E-02	2.28E-01	Distal Intergenic
Inpp1	316376	chr9	53562755	53563055	131	-1.15	1.65E-02	2.28E-01	Promoter (<=1kb)
Cacna1c	24239	chr4	151176010	151176310	92849	1.02	1.65E-02	2.28E-01	Intron (NM_012517/24239, intron 1 of 46)
Nudcd1	362906	chr7	83343472	83343772	4658	-1.54	1.65E-02	2.28E-01	Intron (NM_001130561/362906, intron 1 of 9)
Ube2t	360847	chr13	51849195	51849495	58318	-1.55	1.65E-02	2.28E-01	Distal Intergenic
Rai1	303188	chr10	46511112	46511412	0	1.14	1.65E-02	2.28E-01	Promoter (<=1kb)
Adamts4	66015	chr13	89628010	89628310	5014	-1.56	1.65E-02	2.28E-01	Intron (NM_023959/66015, intron 4 of 8)
Tmem65	500874	chr7	98709100	98709400	213	1.11	1.65E-02	2.28E-01	Promoter (<=1kb)
Atf1	315305	chr7	141702173	141702473	-179788	1.19	1.65E-02	2.28E-01	Distal Intergenic
Mycn	298894	chr6	38225367	38225667	2712	1.61	1.65E-02	2.28E-01	Promoter (2-3kb)
Cdkn2b	25164	chr5	108051578	108051878	-194193	-1.73	1.65E-02	2.28E-01	Distal Intergenic
Trib2	313974	chr6	41023061	41023361	16076	-1.45	1.65E-02	2.28E-01	Intron (NM_001108015/313974, intron 2 of 2)
Pltp	296371	chr3	161321463	161321763	526	0.6	1.65E-02	2.28E-01	Promoter (<=1kb)
Foxe3	171302	chr5	133736148	133736448	-10492	-1.39	1.65E-02	2.28E-01	Distal Intergenic
Rfc4	288003	chr11	81367841	81368141	9249	-1.5	1.66E-02	2.28E-01	Intron (NM_001105869/288003, intron 4 of 10)
Cahm	106182183	chr1	50503841	50504141	323254	0.92	1.66E-02	2.28E-01	Distal Intergenic
Lama5	140433	chr3	175588383	175588683	12429	-1.46	1.66E-02	2.28E-01	Intron (NM_001191609/140433, intron 2 of 78)
Hnf1b	25640	chr10	71185284	71185584	25421	1.01	1.66E-02	2.28E-01	Intron (NM_001308148/25640, intron 5 of 9)
Lyn	81515	chr5	16555599	16555899	29541	-1.71	1.66E-02	2.28E-01	Intron (NM_001111098/81515, intron 1 of 12)
Smoc1	314280	chr6	104718450	104718750	0	0.93	1.66E-02	2.28E-01	Promoter (<=1kb)
Arf5	79117	chr4	55657452	55657752	-57992	-1.52	1.66E-02	2.28E-01	Distal Intergenic
Afdn	26955	chr1	53827020	53827320	13936	-1.85	1.66E-02	2.28E-01	Intron (NM_013217/26955, intron 2 of 39)
Vsir	690899	chr20	29905622	29905922	8028	-1.2	1.66E-02	2.28E-01	Intron (NM_001044300/690899, intron 1 of 6)
Fndc3b	294925	chr2	113402130	113402430	-56579	-1.5	1.66E-02	2.28E-01	Distal Intergenic
Exoc6	50556	chr1	256179810	256180110	-46074	-1.63	1.66E-02	2.28E-01	Distal Intergenic
Gsdmd	315084	chr7	116889599	116889899	54	0.69	1.66E-02	2.28E-01	Promoter (<=1kb)
Setd3	299295	chr6	132089518	132089818	254	1.32	1.66E-02	2.28E-01	Promoter (<=1kb)
Trim8	688785	chr1	266272979	266273279	17182	1.58	1.66E-02	2.28E-01	Distal Intergenic
Mir29b1	100314008	chr4	58498019	58498319	-153629	-1.44	1.66E-02	2.28E-01	Distal Intergenic
Prm1	24685	chr10	4880644	4880944	-65051	1.82	1.66E-02	2.28E-01	Distal Intergenic
RGD1359290	360649	chr10	95649284	95649584	14578	-1.41	1.66E-02	2.28E-01	Intron (NM_001047898/360649, intron 5 of 5)
Rgcc	117183	chr15	61553751	61554051	10644	2.04	1.66E-02	2.28E-01	Intron (NM_054008/117183, intron 3 of 4)
Tnn	304913	chr13	77895664	77895964	777	1.59	1.66E-02	2.28E-01	Promoter (<=1kb)
Sertad2	498423	chr14	104881627	104881927	60412	-1.43	1.67E-02	2.29E-01	Intron (NM_001024903/498423, intron 1 of 1)
Tmem165	364137	chr14	34527847	34528147	115	1.38	1.67E-02	2.29E-01	Promoter (<=1kb)
Frmd6	257646	chr6	93290344	93290644	8936	-1.7	1.67E-02	2.29E-01	Intron (NM_001271054/257646, intron 1 of 13)
Trim28	116698	chr1	65550489	65550789	254	1.11	1.67E-02	2.29E-01	Promoter (<=1kb)
Zfp365	499425	chr20	22060302	22060602	142	0.99	1.67E-02	2.29E-01	Promoter (<=1kb)
Arnt2	25243	chr1	146510575	146510875	45268	1.18	1.67E-02	2.29E-01	Intron (NM_012781/25243, intron 1 of 18)
Mef2a	309957	chr1	128320848	128321148	20115	-1.59	1.67E-02	2.29E-01	Intron (NM_001014035/309957, intron 1 of 10)
Dusp5	171109	chr1	274253349	274253649	8165	-1.38	1.67E-02	2.29E-01	Intron (NM_133578/171109, intron 2 of 3)
Nf1	24592	chr10	66690080	66690380	134	0.84	1.67E-02	2.29E-01	Promoter (<=1kb)
Fbxl17	316663	chr9	110876356	110876656	59975	-1.86	1.67E-02	2.29E-01	Intron (NM_001108235/316663, intron 4 of 8)
Rap1gds1	310909	chr2	244403157	244403457	-32174	1.18	1.67E-02	2.29E-01	Distal Intergenic
Fmo2	246245	chr13	80774393	80774693	571	1.64	1.68E-02	2.29E-01	Promoter (<=1kb)
Apaf1	78963	chr7	31517763	31518063	266129	-1.24	1.68E-02	2.29E-01	Intron (NM_001271371/314721, intron 22 of 30)
Ero1a	171562	chr15	19655117	19655417	0	0.99	1.68E-02	2.29E-01	Promoter (<=1kb)
Ndufa9	362440	chr4	159351722	159352022	47599	0.75	1.68E-02	2.29E-01	Distal Intergenic
Slc37a2	500973	chr8	39733976	39734276	0	0.68	1.68E-02	2.29E-01	Promoter (<=1kb)
Myl1	56781	chr9	73996553	73996853	-38073	-1.48	1.68E-02	2.29E-01	Distal Intergenic
Lrguk	296968	chr4	61233256	61233556	-186454	0.97	1.68E-02	2.29E-01	Intron (NM_053875/116654, intron 18 of 22)
Prdm4	170820	chr7	23960174	23960474	-4242	0.83	1.68E-02	2.29E-01	Distal Intergenic
Fn1	25661	chr9	79068146	79068446	-99133	-1.79	1.68E-02	2.29E-01	Distal Intergenic
Sik3	684112	chr8	50415331	50415631	105068	-1.51	1.68E-02	2.29E-01	Intron (NM_001271216/684112, intron 1 of 24)
Lrp6	312781	chr4	168294408	168294708	2665	1.64	1.68E-02	2.29E-01	Promoter (2-3kb)
Col2a1	25412	chr7	139557584	139557884	-73587	1.27	1.68E-02	2.29E-01	Distal Intergenic
Rufy1	360521	chr10	35936668	35936968	-9400	-1.31	1.68E-02	2.29E-01	Distal Intergenic
Runx2	367218	chr9	18711664	18711964	68094	-1.04	1.68E-02	2.30E-01	Intron (NM_001278483/367218, intron 5 of 8)
Vac14	307842	chr19	41000818	41001118	28088	2	1.68E-02	2.30E-01	Intron (NM_177930/307842, intron 10 of 18)

Krt71	683613	chr7	143317139	143317439	36547	1.98	1.69E-02	2.30E-01	Downstream (2-3kb)
Exoc6b	500233	chr4	116691700	116692000	94424	-0.94	1.69E-02	2.30E-01	Intron (NM_001109246/500233, intron 6 of 22)
Plaur	50692	chr1	81319086	81319386	-8785	-1.27	1.69E-02	2.30E-01	Distal Intergenic
Pak1	29431	chr1	162834920	162835220	66764	2.13	1.69E-02	2.30E-01	Intron (NM_017198/29431, intron 2 of 14)
Prrc2c	360865	chr13	80614412	80614712	213	1.02	1.69E-02	2.30E-01	Promoter (<=1kb)
Hmgn5	100910616	chrX	79909323	79909623	233279	0.7	1.69E-02	2.30E-01	Distal Intergenic
Zfp608	307296	chr18	49602414	49602714	334808	-1.46	1.69E-02	2.30E-01	Distal Intergenic
Mir148b	100313977	chr7	144919893	144920193	7855	1.31	1.69E-02	2.30E-01	Intron (NM_001108117/315345, intron 4 of 8)
Idh2	361596	chr1	141885348	141885648	8026	-1.49	1.69E-02	2.30E-01	Intron (NM_001014161/361596, intron 1 of 10)
Fn1	25661	chr9	79072229	79072529	-103216	-1.53	1.69E-02	2.30E-01	Distal Intergenic
Ccr9	282832	chr8	132817280	132817580	-10511	1.93	1.69E-02	2.30E-01	Distal Intergenic
Tgfbr2	81810	chr8	124392905	124393205	5659	-1.38	1.69E-02	2.30E-01	Intron (NM_031132/81810, intron 1 of 7)
Ganc	24382	chr3	112174173	112174473	266	0.72	1.69E-02	2.30E-01	Promoter (<=1kb)
Meis2	311311	chr3	107311670	107311970	448619	1.22	1.69E-02	2.30E-01	Distal Intergenic
Ppp4r3a	314388	chr6	125074106	125074406	8586	-1.23	1.69E-02	2.30E-01	Intron (NM_001108050/314388, intron 1 of 19)
Hif1a	29560	chr6	96676533	96676833	-134352	-1.67	1.69E-02	2.30E-01	3' UTR
Tmf1	114206	chr4	129544074	129544374	132	0.87	1.69E-02	2.30E-01	Promoter (<=1kb)
Zbtb21	304056	chr11	38456834	38457134	241	0.77	1.69E-02	2.30E-01	Promoter (<=1kb)
Fam133b	362320	chr4	27796258	27796558	-41076	-1.37	1.69E-02	2.30E-01	Intron (NM_001191861/114483, intron 4 of 6)
Oprd1	24613	chr5	150217430	150217730	105333	1.41	1.69E-02	2.30E-01	Distal Intergenic
Cldn15	304388	chr12	22750413	22750713	-1751	1.15	1.69E-02	2.30E-01	Promoter (1-2kb)
Hpse	64537	chr14	10549351	10549651	14993	-1.18	1.69E-02	2.30E-01	Intron (NM_022605/64537, intron 3 of 19)
Zc3h12a	313587	chr5	143119875	143120175	0	0.87	1.70E-02	2.30E-01	Promoter (<=1kb)
Gnb5	83579	chr8	82203302	82203602	-54247	1.3	1.70E-02	2.30E-01	Distal Intergenic
Hipk2	362342	chr4	66624993	66625293	-81	1.73	1.70E-02	2.30E-01	Promoter (<=1kb)
Hpcal1	50871	chr6	43072104	43072404	70156	-1.5	1.70E-02	2.30E-01	Intron (NM_017356/50871, intron 1 of 4)
Dact3	499088	chr1	78800839	78801139	85	0.96	1.70E-02	2.30E-01	Promoter (<=1kb)
Akr1c14	191574	chr17	69817849	69818149	8976	1.93	1.70E-02	2.30E-01	Intron (NM_138547/191574, intron 3 of 8)
Sinhcaf	686611	chr4	183504047	183504347	-77608	-1.44	1.70E-02	2.30E-01	Distal Intergenic
Kdm2b	304495	chr12	39050563	39050863	28563	-1.42	1.70E-02	2.30E-01	Intron (NM_001100679/304495, intron 4 of 28)
Ldlrad4	679578	chr18	63725492	63725792	-118207	-1.14	1.70E-02	2.30E-01	Distal Intergenic
Mir190	100314160	chr8	73095001	73095301	-64870	1.39	1.70E-02	2.30E-01	Distal Intergenic
Noct	310395	chr2	140251307	140251607	-35054	-1.46	1.70E-02	2.30E-01	Distal Intergenic
Ptcd3	500199	chr4	99822794	99823094	0	0.67	1.70E-02	2.30E-01	Promoter (<=1kb)
Rin3	314397	chr6	126104359	126104659	-66061	1.46	1.70E-02	2.30E-01	Intron (NM_001108051/314396, intron 3 of 13)
RGD1359290	360649	chr10	95521256	95521556	-113150	-1.26	1.70E-02	2.30E-01	Distal Intergenic
Ttc39c	686179	chr18	3993466	3993766	35034	-1.69	1.70E-02	2.30E-01	Intron (NM_001077231/686179, intron 1 of 13)
Bend5	362564	chr5	131434711	131435011	177584	2.09	1.70E-02	2.30E-01	Intron (NM_001350232/100911395, intron 9 of 12)
Tnfaip2	299339	chr6	135906794	135907094	15863	-1.65	1.70E-02	2.30E-01	Distal Intergenic
Hs6st3	364476	chr15	104606545	104606845	158066	-1.59	1.70E-02	2.30E-01	Intron (NM_001271404/364476, intron 1 of 1)
Cdk16	81741	chrX	1717957	1718257	380	1.01	1.70E-02	2.30E-01	Promoter (<=1kb)
Gpd1	60666	chr7	141378626	141378926	8086	0.69	1.70E-02	2.30E-01	Distal Intergenic
Fgf1	25317	chr18	32387532	32387832	51430	1.36	1.70E-02	2.30E-01	Intron (NM_012576/24413, intron 1 of 7)
Fam8a1	291031	chr17	18421583	18421883	79	1.17	1.71E-02	2.30E-01	Promoter (<=1kb)
Usp3	363084	chr8	72284209	72284509	362	1.08	1.71E-02	2.30E-01	Promoter (<=1kb)
Pla2g5	29354	chr5	157266455	157266755	2148	-1.51	1.71E-02	2.30E-01	Promoter (2-3kb)
C1ql4	300220	chr7	140773111	140773411	-2375	-1.72	1.71E-02	2.30E-01	Promoter (2-3kb)
Rps3a	29288	chr2	185439996	185440296	4550	-1.47	1.71E-02	2.30E-01	Downstream (<1kb)
Bend6	363212	chr9	38226183	38226483	-70770	0.7	1.71E-02	2.30E-01	Distal Intergenic
Fbxl14	312675	chr4	152056619	152056919	69726	1.85	1.71E-02	2.30E-01	Distal Intergenic
Rnase4	56759	chr15	28011810	28012110	-5930	1.48	1.71E-02	2.30E-01	Distal Intergenic
Il9r	24500	chr10	15715002	15715302	17775	1.31	1.71E-02	2.30E-01	Distal Intergenic
Mtss2	307845	chr19	40921576	40921876	3784	1.36	1.71E-02	2.30E-01	Exon (NM_001191558/307845, exon 6 of 18)
Rasl11b	305302	chr14	36523982	36524282	30292	1.7	1.71E-02	2.30E-01	Intron (NM_001017499/498353, intron 7 of 8)
Cyp4v3	266761	chr16	50080297	50080597	-31206	1.73	1.71E-02	2.30E-01	Distal Intergenic
Olr905	288797	chr1	10909183	10909483	-848747	1.19	1.71E-02	2.30E-01	Distal Intergenic
Setd2	316013	chr8	118809998	118810298	7520	-1.65	1.71E-02	2.30E-01	Intron (NM_001108189/316013, intron 1 of 20)
Arhgap27	303583	chr10	91450818	91451118	-2341	0.82	1.71E-02	2.30E-01	Promoter (2-3kb)
Rara	24705	chr10	86877861	86878161	7331	2.16	1.71E-02	2.30E-01	Exon (NM_031528/24705, exon 3 of 8)
Anks1b	314721	chr7	30738118	30738418	230983	-1.47	1.71E-02	2.30E-01	Intron (NM_001271371/314721, intron 13 of 30)
Wdr60	314523	chr6	144167570	144167870	-43974	-0.87	1.71E-02	2.30E-01	Distal Intergenic
Gnaq	81666	chr1	233598576	233598876	215798	1.44	1.71E-02	2.30E-01	Intron (NM_031036/81666, intron 5 of 6)
Hspa5	25617	chr3	13747514	13747814	-90490	1.22	1.72E-02	2.30E-01	Distal Intergenic
Ptpn9	266611	chr8	61655825	61656125	-3320	-1.11	1.72E-02	2.30E-01	Distal Intergenic
Serpinh1	29345	chr1	164307412	164307712	595	0.67	1.72E-02	2.30E-01	Promoter (<=1kb)
Itgb7	25713	chr7	143839188	143839488	-5805	-1.68	1.72E-02	2.30E-01	Downstream (1-2kb)
Rap1gap2	303298	chr10	61247941	61248241	-15610	-1.28	1.72E-02	2.30E-01	Distal Intergenic
Pde6h	114248	chr4	171059517	171059817	101321	-1.25	1.72E-02	2.30E-01	Distal Intergenic
Fam133b	362320	chr4	27754836	27755136	46	1.15	1.72E-02	2.31E-01	Promoter (<=1kb)
Fto	291905	chr19	17114796	17115096	2	0.72	1.72E-02	2.31E-01	Promoter (<=1kb)
Vdac3	83532	chr16	74292583	74292883	117	0.83	1.72E-02	2.31E-01	Promoter (<=1kb)
Ppa2	310856	chr2	238529228	238529528	156	1.22	1.72E-02	2.31E-01	Promoter (<=1kb)
Tnfrsf8	25069	chr5	163347588	163347888	-116018	-1.61	1.72E-02	2.31E-01	Distal Intergenic
Tm6sf1	361600	chr1	143660970	143661270	3363	-1.95	1.73E-02	2.31E-01	Intron (NM_001108490/361600, intron 1 of 10)
Myct1	292264	chr1	42121956	42122256	384	-1.77	1.73E-02	2.31E-01	Promoter (<=1kb)
Sema4b	293042	chr1	141976807	141977107	-9038	-1.68	1.73E-02	2.31E-01	Distal Intergenic
Rpia	362383	chr4	98575768	98576068	17596	-1.63	1.73E-02	2.31E-01	Exon (NM_001108632/362383, exon 7 of 9)
LOC100910558	100910558	chr5	100757793	100758093	107281	-1.42	1.73E-02	2.31E-01	Distal Intergenic
Runx2	367218	chr9	18675910	18676210	32340	-1.37	1.73E-02	2.31E-01	Intron (NM_001278483/367218, intron 5 of 8)
Runx3	156726	chr5	153474198	153474498	-32591	0.78	1.73E-02	2.31E-01	Distal Intergenic
Ubqln2	317396	chrX	18726017	18726317	415	0.83	1.73E-02	2.31E-01	Promoter (<=1kb)
Fn1	25661	chr9	78942579	78942879	26134	-1.36	1.73E-02	2.31E-01	Exon (NM_019143/25661, exon 18 of 46)
Bri3bp	498176	chr12	36589919	36590219	-2080	2.11	1.73E-02	2.31E-01	Promoter (2-3kb)
Il6st	25205	chr2	44285860	44286160	-3915	-1.55	1.73E-02	2.31E-01	Distal Intergenic
Agap1	316611	chr9	96617980	96618280	-128160	-1.36	1.73E-02	2.31E-01	Distal Intergenic
Abhd16b	311720	chr3	176963305	176963605	-22295	2.02	1.73E-02	2.31E-01	Distal Intergenic
Cryba1	25583	chr10	65181524	65181824	20372	-1.6	1.73E-02	2.31E-01	Distal Intergenic
Cxcl12	24772	chr4	149415484	149415784	154440	1.91	1.73E-02	2.31E-01	Distal Intergenic
Pogk	304941	chr13	84615292	84615592	3450	-1.56	1.73E-02	2.31E-01	Intron (NM_001107194/304941, intron 2 of 4)
Tcof1	291571	chr18	56131758	56132058	-16039	-1.68	1.74E-02	2.31E-01	Distal Intergenic
Kbtbd8	500262	chr4	127165047	127165347	154	1.14	1.74E-02	2.31E-01	Promoter (<=1kb)
Klf13	499171	chr1	124781063	124781363	22000	-1.61	1.74E-02	2.31E-01	Intron (NM_001109147/499171, intron 1 of 1)
Rasgrp1	29434	chr3	109168365	109168665	-123945	0.8	1.74E-02	2.31E-01	Distal Intergenic
Lingo1	315691	chr8	61324807	61325107	-34567	0.92	1.74E-02	2.31E-01	Distal Intergenic

Arrdc3	309945	chr2	8740812	8741112	7645	-1.75	1.74E-02	2.31E-01	Exon (NM_001007797/309945, exon 7 of 8)
Spg7	353231	chr19	55849291	55849591	-30970	-1.15	1.74E-02	2.31E-01	Distal Intergenic
Ndc1	362557	chr5	127043210	127043510	66570	1.27	1.74E-02	2.31E-01	Distal Intergenic
Ddr2	685781	chr13	88378980	88379280	-17298	-1.62	1.74E-02	2.31E-01	Distal Intergenic
Gadd45g	291005	chr17	13240073	13240373	152846	-1.05	1.74E-02	2.31E-01	Distal Intergenic
Tmem26	309724	chr20	21702013	21702313	-12358	-1.64	1.74E-02	2.31E-01	Intron (NM_001107624/309728, intron 1 of 11)
Mpnd	681944	chr9	11061347	11061647	0	0.71	1.74E-02	2.31E-01	Promoter (<=1kb)
Dapk2	300799	chr8	71940448	71940748	25679	-1.37	1.74E-02	2.31E-01	Intron (NM_001013109/300799, intron 7 of 7)
Clint1	360515	chr10	30833884	30834184	0	1.83	1.74E-02	2.31E-01	Promoter (<=1kb)
Art4	312806	chr4	170831284	170831584	9603	1.65	1.74E-02	2.31E-01	3' UTR
Ints7	289382	chr13	110201144	110201444	-56127	2.04	1.74E-02	2.31E-01	Distal Intergenic
Acad11	315973	chr8	112639147	112639447	44389	1.57	1.74E-02	2.31E-01	Intron (NM_001108181/315973, intron 11 of 19)
Nr1i3	65035	chr13	89581630	89581930	-4406	-1.33	1.74E-02	2.31E-01	Distal Intergenic
Ccdc148	311051	chr3	45245460	45245760	-34986	-1.69	1.74E-02	2.31E-01	Distal Intergenic
Akap2	298024	chr5	74813872	74814172	-127919	1.41	1.75E-02	2.31E-01	Distal Intergenic
Rnf144b	364681	chr17	17893989	17894289	53488	-1.3	1.75E-02	2.31E-01	Intron (NM_001108881/364681, intron 1 of 7)
Bicd1	362466	chr4	183903562	183903862	-115225	-1.38	1.75E-02	2.31E-01	Distal Intergenic
Tmem17	360985	chr14	107371882	107372182	69074	1.92	1.75E-02	2.31E-01	Distal Intergenic
St3gal4	363040	chr8	36299381	36299681	15130	-1.53	1.75E-02	2.31E-01	Intron (NM_203337/363040, intron 1 of 10)
Cyp46a1	362782	chr6	132283209	132283509	40881	1.48	1.75E-02	2.31E-01	Distal Intergenic
Ptk2	25614	chr7	114650705	114651005	-60497	-1.6	1.75E-02	2.31E-01	Distal Intergenic
Sgk1	29517	chr1	24362606	24362906	-169039	1.97	1.75E-02	2.31E-01	Distal Intergenic
Hsf2bp	499413	chr20	10785867	10786167	56203	2.06	1.75E-02	2.32E-01	Intron (NM_001127683/499413, intron 7 of 8)
Metrnl	316842	chr10	110893577	110893877	316	0.8	1.75E-02	2.32E-01	Promoter (<=1kb)
Met	24553	chr4	44747208	44747508	-27825	0.96	1.75E-02	2.32E-01	Distal Intergenic
Glcci1	296884	chr4	34362422	34362722	79797	-1.54	1.75E-02	2.32E-01	Intron (NM_001177442/296884, intron 2 of 10)
RGD1305110	305579	chr14	108376194	108376494	72	1.36	1.75E-02	2.32E-01	Promoter (<=1kb)
Abtb1	297432	chr4	120785423	120785723	-616	0.99	1.75E-02	2.32E-01	Promoter (<=1kb)
Mrgprd	293648	chr1	218505453	218505753	17629	1.98	1.75E-02	2.32E-01	Distal Intergenic
Slc38a6	299139	chr6	96190305	96190605	18549	1.05	1.75E-02	2.32E-01	Intron (NM_001013099/299139, intron 3 of 15)
Sdcbp	83841	chr5	19469692	19469992	-1672	1.98	1.75E-02	2.32E-01	Promoter (1-2kb)
Ccdc146	499980	chr4	10269463	10269763	-35	0.79	1.75E-02	2.32E-01	Promoter (<=1kb)
Nob1	291996	chr19	38396915	38397215	-251	-1.44	1.75E-02	2.32E-01	Promoter (<=1kb)
Ces5a	307660	chr19	11901538	11901838	-195691	-1.29	1.75E-02	2.32E-01	Distal Intergenic
Neurl1	309459	chr1	267060789	267061089	107650	1.06	1.76E-02	2.32E-01	Intron (NM_001107606/309460, intron 9 of 13)
LOC100302465	100302465	chr1	206231683	206231983	185160	-1.44	1.76E-02	2.32E-01	Distal Intergenic
Cdc14b	361195	chr17	1797461	1797761	0	1.11	1.76E-02	2.32E-01	Promoter (<=1kb)
RGD1309106	360864	chr13	80000687	80000987	-101208	1.9	1.76E-02	2.32E-01	Intron (NM_138538/171574, intron 16 of 20)
Trim52	290458	chr15	90299148	90299448	-65157	-1.9	1.76E-02	2.32E-01	Distal Intergenic
Abi1	79249	chr17	89950970	89951270	-482	1.18	1.76E-02	2.32E-01	Promoter (<=1kb)
Cbr4	359725	chr16	31703890	31704190	268317	2.07	1.76E-02	2.32E-01	Distal Intergenic
B4galt5	362275	chr3	164095309	164095609	-39748	0.88	1.76E-02	2.32E-01	Distal Intergenic
Sos2	85384	chr6	92007698	92007998	0	0.85	1.76E-02	2.32E-01	Promoter (<=1kb)
Mta1	64520	chr6	137911727	137912027	-12543	0.99	1.76E-02	2.32E-01	Distal Intergenic
Nfkb1	81736	chr2	240885221	240885521	4532	-1.34	1.77E-02	2.32E-01	Intron (NM_001276711/81736, intron 1 of 24)
Itgb8	362800	chr6	147249017	147249317	-76204	-1.59	1.77E-02	2.32E-01	Distal Intergenic
Ccdc184	500925	chr7	139777025	139777325	14411	0.71	1.77E-02	2.32E-01	Distal Intergenic
Dclk1	83825	chr2	144911816	144912116	50371	1.76	1.77E-02	2.33E-01	Intron (NM_053343/83825, intron 11 of 12)
Fbxl14	312675	chr4	151969322	151969622	-17271	-0.89	1.77E-02	2.33E-01	Distal Intergenic
Cbr4	359725	chr16	31772813	31773113	199394	-1.66	1.77E-02	2.33E-01	Distal Intergenic
Tnfsf4	89814	chr13	79380593	79380893	110620	-1.59	1.77E-02	2.33E-01	Distal Intergenic
Bfsp1	25394	chr3	137959180	137959480	10178	-1.46	1.77E-02	2.33E-01	Intron (NM_031555/25394, intron 2 of 7)
Lnc056	104845257	chr5	22006346	22006646	121746	-2.03	1.77E-02	2.33E-01	Distal Intergenic
Utrn	25600	chr1	6905555	6905855	64185	-1.2	1.77E-02	2.33E-01	Intron (NM_013070/25600, intron 2 of 74)
Tgfa	24827	chr4	117992705	117993005	30527	-1.36	1.77E-02	2.33E-01	Intron (NM_012671/24827, intron 1 of 5)
Ror2	306782	chr17	11953689	11953989	137	0.82	1.77E-02	2.33E-01	Promoter (<=1kb)
Jun	24516	chr5	114014818	114015118	-541	0.65	1.77E-02	2.33E-01	Promoter (<=1kb)
Smad6	367100	chr8	68930745	68931045	35063	-1.53	1.77E-02	2.33E-01	Intron (NM_001109002/367100, intron 3 of 3)
Erc1	266806	chr4	152385015	152385315	-4992	-1.5	1.77E-02	2.33E-01	Distal Intergenic
Mdh1b	316444	chr9	70356485	70356785	93385	-1.77	1.77E-02	2.33E-01	Distal Intergenic
Eif4e3	297481	chr4	131958507	131958807	152285	0.71	1.77E-02	2.33E-01	Distal Intergenic
Naa15	310399	chr2	140494608	140494908	22918	-1.32	1.77E-02	2.33E-01	Exon (NM_001107674/310399, exon 2 of 20)
Ccn5	29576	chr3	160208783	160209083	814	0.65	1.78E-02	2.33E-01	Promoter (<=1kb)
Odf1	24610	chr7	77039126	77039426	-27529	-1.46	1.78E-02	2.33E-01	Distal Intergenic
Golph3	78961	chr2	62373450	62373750	12696	-1.78	1.78E-02	2.33E-01	Intron (NM_023977/78961, intron 1 of 3)
Cebpg	25301	chr1	91289524	91289824	6843	-1.56	1.78E-02	2.33E-01	Downstream (<1kb)
Nfe2	366998	chr7	144879869	144880169	0	1.18	1.78E-02	2.33E-01	Promoter (<=1kb)
Samd4b	308473	chr1	85343547	85343847	5742	1.95	1.78E-02	2.33E-01	Intron (NM_001107498/308473, intron 1 of 11)
Slc13a4	503568	chr4	62830670	62830970	9387	-1.24	1.78E-02	2.33E-01	Intron (NM_001012621/503568, intron 3 of 16)
Paqr3	305203	chr14	14202779	14203079	95199	-1.52	1.78E-02	2.33E-01	Distal Intergenic
Hpcal1	50871	chr6	43070580	43070880	68632	-1.57	1.78E-02	2.33E-01	Intron (NM_017356/50871, intron 1 of 4)
LOC690120	690120	chr7	99872239	99872539	81545	-1.53	1.78E-02	2.33E-01	Intron (NM_001024876/299957, intron 4 of 6)
Mtss1	362918	chr7	98941812	98942112	18624	1.32	1.78E-02	2.33E-01	Intron (NM_001130563/362918, intron 2 of 15)
Spsb4	300950	chr8	104991101	104991401	-78142	-1.62	1.78E-02	2.33E-01	Distal Intergenic
Srpk2	296753	chr4	8089085	8089385	22348	-1.79	1.78E-02	2.33E-01	Intron (NM_001106575/296753, intron 1 of 14)
Trak1	316085	chr8	129981991	129982291	-27282	-1.52	1.78E-02	2.33E-01	Distal Intergenic
Mir3556b	100526572	chr1	38542980	38543280	-303978	0.68	1.78E-02	2.33E-01	Distal Intergenic
Igsf3	295325	chr2	203800702	203801002	31973	-1.08	1.79E-02	2.33E-01	Intron (NM_001106455/295325, intron 1 of 8)
C2cd2	304055	chr11	38382289	38382589	37443	1.64	1.79E-02	2.33E-01	Intron (NM_199391/304055, intron 6 of 13)
Runx2	367218	chr9	18819290	18819590	175720	0.84	1.79E-02	2.33E-01	Distal Intergenic
Lbr	89789	chr13	100484714	100485014	-34505	1.64	1.79E-02	2.33E-01	Distal Intergenic
Ttc17	311224	chr3	83298252	83298552	8038	-1.63	1.79E-02	2.33E-01	Intron (NM_001107752/311224, intron 1 of 24)
Fgf2	54250	chr2	124093844	124094144	12772	-1.6	1.79E-02	2.33E-01	Intron (NM_019305/54250, intron 1 of 2)
Mir6332	102466626	chr5	167419909	167420209	16941	0.82	1.79E-02	2.33E-01	Intron (NM_053885/116665, intron 1 of 22)
Cdc27	360643	chr10	92455783	92456083	145999	-1.52	1.79E-02	2.33E-01	Distal Intergenic
Lmod1	304816	chr13	52147393	52147693	0	0.75	1.79E-02	2.33E-01	Promoter (<=1kb)
Macf1	362587	chr5	141367117	141367417	-3593	1.05	1.79E-02	2.33E-01	Distal Intergenic
Rgs3	54293	chr5	78562845	78563145	78952	-1.36	1.79E-02	2.33E-01	Intron (NM_019340/54293, intron 12 of 15)
Atg7	312647	chr4	146582567	146582867	-15549	-1.23	1.79E-02	2.33E-01	Distal Intergenic
Marveld1	309375	chr1	261389879	261390179	75	0.67	1.79E-02	2.33E-01	Promoter (<=1kb)
Dysf	312492	chr4	115802739	115803039	89735	1.5	1.79E-02	2.33E-01	Intron (NM_001107869/312492, intron 22 of 52)
Ldhal6b	369018	chr1	46123122	46123422	185854	-1.4	1.79E-02	2.33E-01	Distal Intergenic
Uspl1	288447	chr12	6974895	6975195	-17981	-1.58	1.79E-02	2.33E-01	Distal Intergenic
Smurf2	303614	chr10	95072043	95072343	45764	-2.05	1.79E-02	2.33E-01	Intron (NM_001107061/303614, intron 2 of 23)

Sema6b	84609	chr9	10898354	10898654	-42959	-2.05	1.79E-02	2.33E-01	Distal Intergenic
LOC100912071	100912071	chr1	165226266	165226566	10886	-1.58	1.79E-02	2.33E-01	Intron (NR_131097/100912071, intron 1 of 2)
Eif4e2	363275	chr9	94320514	94320814	10038	-1.8	1.79E-02	2.33E-01	Intron (NM_001108808/363275, intron 4 of 6)
Pptc7	304488	chr12	39772421	39772721	-2941	1.49	1.80E-02	2.33E-01	Promoter (2-3kb)
Fam214a	300836	chr8	81887711	81888011	24092	1.97	1.80E-02	2.33E-01	Intron (NM_001106838/300836, intron 2 of 14)
Actr10	299121	chr6	93367874	93368174	-17283	0.73	1.80E-02	2.33E-01	Distal Intergenic
Ptpra	25167	chr3	122980864	122981164	4760	2.06	1.80E-02	2.33E-01	Intron (NM_012763/25167, intron 1 of 20)
Gpr146	498153	chr12	17363248	17363548	-4631	1.51	1.80E-02	2.33E-01	Intron (NM_001025033/498154, intron 2 of 4)
Tpcn1	246215	chr12	41507758	41508058	0	0.61	1.80E-02	2.33E-01	Promoter (<=1kb)
Smtnl1	311167	chr3	72206582	72206882	8328	-1.45	1.80E-02	2.33E-01	Downstream (<1kb)
Nubp1	287042	chr10	5343634	5343934	15	0.64	1.80E-02	2.33E-01	Promoter (<=1kb)
Sec63	309858	chr20	47494611	47494911	341	1.12	1.80E-02	2.33E-01	Promoter (<=1kb)
Gng11	64199	chr4	31319111	31319411	-68009	-1.61	1.80E-02	2.33E-01	Distal Intergenic
Dupd1	361003	chr15	2986146	2986446	219217	1.85	1.80E-02	2.33E-01	Distal Intergenic
Ift172	116475	chr6	26392752	26393052	2060	1.94	1.80E-02	2.33E-01	Promoter (2-3kb)
Nrp2	81527	chr9	69234469	69234769	-262665	-1.44	1.80E-02	2.33E-01	Intron (NM_001191808/301455, intron 19 of 22)
Hbegf	25433	chr18	29343482	29343782	-3297	1.91	1.80E-02	2.34E-01	Distal Intergenic
Rab1a	81754	chr14	104530650	104530950	55568	1.53	1.80E-02	2.34E-01	Distal Intergenic
Ahdc1	362617	chr5	151238469	151238769	27127	0.87	1.80E-02	2.34E-01	Intron (NM_001134956/362617, intron 1 of 5)
Lrrfip1	367314	chr9	98216866	98217166	26037	1.57	1.80E-02	2.34E-01	Intron (NM_001014269/367314, intron 2 of 7)
Rbm26	306137	chr15	89339025	89339325	0	1.04	1.80E-02	2.34E-01	Promoter (<=1kb)
Tmbim4	362884	chr7	64992276	64992576	4414	-1.41	1.81E-02	2.34E-01	Intron (NM_199116/362884, intron 1 of 6)
Fbxo11	301674	chr6	11649762	11650062	36620	-1.58	1.81E-02	2.34E-01	Distal Intergenic
Acad11	315973	chr8	112652410	112652710	57652	1.89	1.81E-02	2.34E-01	Intron (NM_001108181/315973, intron 13 of 19)
Dlc1	58834	chr16	59197520	59197820	153	0.65	1.81E-02	2.34E-01	Promoter (<=1kb)
Gclc	25283	chr8	85074975	85075275	15924	-1.63	1.81E-02	2.34E-01	Intron (NM_012815/25283, intron 1 of 15)
Sapcd1	406170	chr20	5025960	5026260	-5909	-1.15	1.81E-02	2.34E-01	Intron (NM_212536/294252, intron 12 of 24)
Cbr4	359725	chr16	31880068	31880368	92139	-1.75	1.81E-02	2.34E-01	Distal Intergenic
Fam241a	691931	chr2	232267578	232267878	-22259	1.26	1.81E-02	2.34E-01	Distal Intergenic
Tle1	362533	chr5	88629863	88630163	-372	0.77	1.81E-02	2.34E-01	Promoter (<=1kb)
RGD1562024	498699	chr17	8662376	8662676	-42639	2.26	1.81E-02	2.34E-01	Distal Intergenic
Nfkb1	81736	chr2	240879004	240879304	10749	-1.52	1.81E-02	2.34E-01	Intron (NM_001276711/81736, intron 1 of 24)
Arhgap21	307178	chr17	87851227	87851527	-1498	1.29	1.81E-02	2.34E-01	Promoter (1-2kb)
Tkt	64524	chr16	6603318	6603618	-6052	1.92	1.81E-02	2.34E-01	Distal Intergenic
Ldhal6b	369018	chr1	45921870	45922170	387106	-1.28	1.81E-02	2.34E-01	Distal Intergenic
Prpf18	171552	chr17	77708996	77709296	107082	-1.72	1.81E-02	2.34E-01	Intron (NM_001191821/307128, intron 11 of 21)
Ubac2	361094	chr15	108286643	108286943	190	0.75	1.81E-02	2.34E-01	Promoter (<=1kb)
Rala	81757	chr17	49701191	49701491	-12799	-1.48	1.81E-02	2.34E-01	Distal Intergenic
RGD1311188	315088	chr7	117026089	117026389	172	0.85	1.81E-02	2.34E-01	Promoter (<=1kb)
Ptprs	25529	chr9	10585414	10585714	54	1.04	1.81E-02	2.34E-01	Promoter (<=1kb)
Bad	64639	chr1	222197985	222198285	-249	0.6	1.82E-02	2.34E-01	Promoter (<=1kb)
Mogat2	681211	chr1	164180932	164181232	23990	1.23	1.82E-02	2.34E-01	Downstream (<1kb)
Lima1	300228	chr7	141469852	141470152	24633	0.77	1.82E-02	2.34E-01	Exon (NM_001191615/300228, exon 3 of 10)
Arhgap10	688429	chr19	34404970	34405270	264973	1.36	1.82E-02	2.34E-01	Distal Intergenic
Atp6v0e2	436582	chr4	78136305	78136605	-31512	0.64	1.82E-02	2.34E-01	Distal Intergenic
Adam4l1	500688	chr6	104868247	104868547	140969	2.06	1.82E-02	2.34E-01	Intron (NM_001002835/314280, intron 10 of 12)
Vsir	690899	chr20	29937930	29938230	40336	2.06	1.82E-02	2.34E-01	Intron (NM_053644/114102, intron 29 of 65)
Ncam1	24586	chr8	54339325	54339625	-204594	-1.45	1.82E-02	2.34E-01	Distal Intergenic
Smtn	289734	chr14	83799965	83800265	-23102	-1.61	1.82E-02	2.34E-01	Distal Intergenic
Tp53i11	311209	chr3	82145576	82145876	63679	1.95	1.82E-02	2.35E-01	Intron (NM_001107750/311210, intron 2 of 9)
Adpgk	315722	chr8	64093788	64094088	4875	-1.59	1.83E-02	2.35E-01	Intron (NM_001100723/315722, intron 1 of 6)
LOC100910620	100910620	chr5	74459469	74459769	-16797	-1.01	1.83E-02	2.35E-01	Distal Intergenic
LOC100363289	100363289	chr8	86935801	86936101	-269210	1.71	1.83E-02	2.35E-01	Distal Intergenic
Caprin1	362173	chr3	93727105	93727405	6877	-1.43	1.83E-02	2.35E-01	Intron (NM_001012185/362173, intron 2 of 18)
RGD1306746	312511	chr4	118271419	118271719	28287	2	1.83E-02	2.35E-01	3' UTR
Tbc1d2b	315880	chr8	97609776	97610076	36996	-1.23	1.83E-02	2.35E-01	Intron (NM_001108175/315880, intron 4 of 13)
Slf1	294601	chr2	4195411	4195711	108	0.64	1.83E-02	2.36E-01	Promoter (<=1kb)
Frem2	310418	chr2	142880546	142880846	4758	1.4	1.83E-02	2.36E-01	Exon (NM_001245978/310418, exon 3 of 26)
Epha2	366492	chr5	159825761	159826061	-19712	0.73	1.84E-02	2.36E-01	Distal Intergenic
Map3k5	365057	chr1	15423473	15423773	10870	-1.56	1.84E-02	2.36E-01	Intron (NM_001277694/365057, intron 1 of 34)
Fam20b	304885	chr13	74333017	74333317	-1803	0.84	1.84E-02	2.36E-01	Promoter (1-2kb)
Dnttip1	171437	chr3	161212387	161212687	231	0.75	1.84E-02	2.36E-01	Promoter (<=1kb)
Tsku	308843	chr1	163359463	163359763	-30872	-1.82	1.84E-02	2.36E-01	Distal Intergenic
Il17re	362417	chr4	145414027	145414327	69	0.66	1.84E-02	2.36E-01	Promoter (<=1kb)
Clic2	294141	chr20	297671	297971	2333	1.99	1.84E-02	2.36E-01	Promoter (2-3kb)
Tmem176b	171411	chr4	78433902	78434202	23977	-1.3	1.84E-02	2.36E-01	Distal Intergenic
Tctex1d1	362553	chr5	122301025	122301325	-205048	0.7	1.84E-02	2.36E-01	Distal Intergenic
Nfib	29227	chr5	100378909	100379209	268089	1.66	1.84E-02	2.36E-01	Distal Intergenic
Fkbp9	297123	chr4	87167554	87167854	40	0.69	1.84E-02	2.36E-01	Promoter (<=1kb)
Drd1	24316	chr17	11214951	11215251	114024	1.27	1.84E-02	2.36E-01	Distal Intergenic
Nceh1	294930	chr2	112868845	112869145	11	0.7	1.85E-02	2.36E-01	Promoter (<=1kb)
Catsper3	290989	chr17	8994506	8994806	-69139	0.86	1.85E-02	2.36E-01	Distal Intergenic
Srpra	315548	chr8	36394610	36394910	-15773	-1.48	1.85E-02	2.36E-01	Distal Intergenic
Gnai2	81664	chr8	116379505	116379805	11502	1.98	1.85E-02	2.36E-01	Intron (NM_031035/81664, intron 1 of 8)
Snrpa1	100361269	chr1	126969376	126969676	7987	-1.65	1.85E-02	2.36E-01	Exon (NM_001271323/100361269, exon 4 of 9)
Klhl21	313743	chr5	169181748	169182048	330	1.11	1.85E-02	2.37E-01	Promoter (<=1kb)
Micos10	362641	chr5	157566393	157566693	6490	-1.37	1.85E-02	2.37E-01	Intron (NM_001173556/362641, intron 1 of 3)
H6pd	298655	chr5	167030032	167030332	109	1.12	1.85E-02	2.37E-01	Promoter (<=1kb)
Chst3	84468	chr20	29768565	29768865	-30157	0.85	1.85E-02	2.37E-01	Distal Intergenic
Acad11	315973	chr8	112605712	112606012	10954	1.56	1.85E-02	2.37E-01	Exon (NM_001108181/315973, exon 3 of 20)
Magea11	302845	chrX	141848970	141849270	1878	0.85	1.85E-02	2.37E-01	Promoter (1-2kb)
Clcn5	25749	chrX	16170706	16171006	121	1.04	1.86E-02	2.37E-01	Promoter (<=1kb)
RGD1565959	301230	chr9	14854830	14855130	-28166	-1.42	1.86E-02	2.37E-01	Distal Intergenic
Slc2a13	171147	chr7	132757168	132757468	90	1.55	1.86E-02	2.37E-01	Promoter (<=1kb)
Limd2	360646	chr10	94352421	94352721	159	0.86	1.86E-02	2.37E-01	Promoter (<=1kb)
Ptpmt1	29390	chr3	79773163	79773463	-29426	-1.34	1.86E-02	2.37E-01	Distal Intergenic
Large1	361368	chr19	13108751	13109051	-165808	-1.48	1.86E-02	2.37E-01	Distal Intergenic
Bmp4	25296	chr15	20924191	20924491	-144389	1.92	1.86E-02	2.37E-01	Distal Intergenic
Phf5a	192246	chr7	123098470	123098770	3081	-1.32	1.86E-02	2.37E-01	Intron (NM_138888/192246, intron 3 of 3)
Cntnap5c	297846	chr13	7177421	7177721	864544	-1.61	1.86E-02	2.37E-01	Intron (NM_001047866/297846, intron 13 of 23)
Rasgrf1	192213	chr8	97342524	97342824	65198	1.44	1.86E-02	2.37E-01	Exon (NM_001170531/192213, exon 19 of 35)
Cerk	300129	chr7	126939953	126940253	117803	0.95	1.86E-02	2.37E-01	Distal Intergenic
Srsf5	29667	chr6	104617747	104618047	6721	-1.21	1.86E-02	2.37E-01	3' UTR
Cnot2	299805	chr7	59617045	59617345	-29660	0.77	1.87E-02	2.37E-01	Distal Intergenic

Pde8a	308776	chr1	143040140	143040440	3922	-1.48	1.87E-02	2.37E-01	Intron (NM_198767/308776, intron 1 of 21)
Dusp6	116663	chr7	41719548	41719848	244385	-1.48	1.87E-02	2.37E-01	Distal Intergenic
Sgsm1	288743	chr12	49357578	49357878	28144	0.71	1.87E-02	2.37E-01	Exon (NM_001105937/288743, exon 10 of 25)
Spred1	296072	chr3	108638445	108638745	-156592	0.72	1.87E-02	2.38E-01	Distal Intergenic
LOC499542	499542	chr2	61765805	61766105	-7888	-1.34	1.87E-02	2.38E-01	Distal Intergenic
Ccr5	117029	chr8	133240912	133241212	30428	-1.06	1.87E-02	2.38E-01	Distal Intergenic
Sertad2	498423	chr14	104773364	104773664	-47551	-1.31	1.87E-02	2.38E-01	Distal Intergenic
Pgm2	289632	chr14	45863490	45863790	82	0.51	1.87E-02	2.38E-01	Promoter (<=1kb)
Clpx	300786	chr8	70778242	70778542	-10840	-1.53	1.87E-02	2.38E-01	Distal Intergenic
Commd10	361323	chr18	41022517	41022817	0	0.61	1.87E-02	2.38E-01	Promoter (<=1kb)
Bola3	297388	chr4	115046690	115046990	0	0.65	1.87E-02	2.38E-01	Promoter (<=1kb)
Loxl3	312478	chr4	113876472	113876772	9074	-1.52	1.87E-02	2.38E-01	Intron (NM_001107866/312478, intron 4 of 13)
Tmem114	501675	chr10	7311374	7311674	31454	-1.45	1.87E-02	2.38E-01	Distal Intergenic
Serp2	498546	chr15	58929199	58929499	-217327	-1.53	1.87E-02	2.38E-01	Distal Intergenic
Hexim2	303580	chr10	91217086	91217386	7	0.74	1.87E-02	2.38E-01	Promoter (<=1kb)
Kpna2	85245	chr10	95225277	95225577	-13166	0.74	1.87E-02	2.38E-01	Distal Intergenic
Fer	301737	chr9	111559721	111560021	-89610	0.93	1.87E-02	2.38E-01	Distal Intergenic
Pim3	64534	chr7	129856223	129856523	0	0.82	1.88E-02	2.38E-01	Promoter (<=1kb)
R3hdm1	304763	chr13	44833420	44833720	20853	-1.45	1.88E-02	2.38E-01	Intron (NM_001134867/304763, intron 1 of 26)
Ppp1r12c	499076	chr1	72903253	72903553	90	1.36	1.88E-02	2.38E-01	Promoter (<=1kb)
Rpl27a	293418	chr1	174160600	174160900	27802	1.87	1.88E-02	2.38E-01	Intron (NM_001107547/308944, intron 4 of 19)
Eef1d	300033	chr7	116931857	116932157	4517	-1.45	1.88E-02	2.38E-01	Intron (NM_001013104/300033, intron 3 of 7)
Gna13	303634	chr10	97672534	97672834	25338	2.21	1.88E-02	2.38E-01	Intron (NM_001013119/303634, intron 2 of 3)
Ldlrap1	500564	chr5	152953944	152954244	33042	1.51	1.88E-02	2.38E-01	Distal Intergenic
Rev1	316344	chr9	44971588	44971888	-210539	1.81	1.88E-02	2.38E-01	Intron (NM_001191887/363220, intron 8 of 21)
Ube2l6	295704	chr3	72192053	72192353	520	0.68	1.88E-02	2.38E-01	Promoter (<=1kb)
Slc25a26	362403	chr4	126717402	126717702	195067	1.31	1.88E-02	2.38E-01	Distal Intergenic
Pdia4	116598	chr4	77446323	77446623	42912	0.53	1.88E-02	2.38E-01	Distal Intergenic
Nmi	311021	chr3	37475813	37476113	4871	-1.44	1.88E-02	2.38E-01	Intron (NM_001034148/311021, intron 1 of 6)
Ralbp1	84014	chr9	113587808	113588108	10370	-1.36	1.88E-02	2.38E-01	Exon (NM_032067/84014, exon 5 of 10)
Armc5	361653	chr1	199659568	199659868	3921	-1.53	1.88E-02	2.38E-01	Intron (NM_001009455/361653, intron 3 of 5)
Paxip1	311944	chr4	3957040	3957340	-2300	1.5	1.88E-02	2.38E-01	Promoter (2-3kb)
Vgll4	297523	chr4	146896500	146896800	-57103	-1.68	1.88E-02	2.38E-01	Distal Intergenic
Trub2	366012	chr3	8194230	8194530	154216	1.43	1.89E-02	2.38E-01	Distal Intergenic
Zfp36l1	29344	chr6	103108628	103108928	204146	-1.35	1.89E-02	2.38E-01	Distal Intergenic
Tead1	361630	chr1	177508088	177508388	12306	-1.55	1.89E-02	2.38E-01	Intron (NM_001198589/361630, intron 1 of 12)
Ei24	300514	chr8	39270853	39271153	0	1.09	1.89E-02	2.38E-01	Promoter (<=1kb)
St3gal5	83505	chr4	99916293	99916593	-20965	1.21	1.89E-02	2.38E-01	Distal Intergenic
Mapk8ip1	116457	chr3	81300362	81300662	3519	0.78	1.89E-02	2.38E-01	Exon (NM_053777/116457, exon 3 of 12)
Fn1	25661	chr9	79209953	79210253	-240940	-1.14	1.89E-02	2.38E-01	Distal Intergenic
Defb21	641636	chr3	148046940	148047240	-398	1.27	1.89E-02	2.38E-01	Promoter (<=1kb)
Gramd2b	307288	chr18	51122638	51122938	-369258	1.58	1.89E-02	2.38E-01	Distal Intergenic
Gpbp1	294734	chr2	43056392	43056692	11985	-1.49	1.89E-02	2.39E-01	Intron (NM_001106410/294734, intron 1 of 11)
Prnp	24686	chr3	124525386	124525686	9408	-1.36	1.89E-02	2.39E-01	Intron (NM_012631/24686, intron 2 of 2)
Atrn	83526	chr3	123409047	123409347	-25062	1.5	1.89E-02	2.39E-01	Distal Intergenic
Cops8	363283	chr9	97907616	97907916	135392	-1.46	1.89E-02	2.39E-01	Distal Intergenic
Myc	24577	chr7	102718692	102718992	132379	-1.37	1.89E-02	2.39E-01	Distal Intergenic
Lurap1l	362535	chr5	98517673	98517973	48626	1.83	1.90E-02	2.39E-01	Distal Intergenic
Zbtb10	80338	chr2	94547222	94547522	183454	-1.45	1.90E-02	2.39E-01	Distal Intergenic
Pparg	25664	chr4	147294978	147295278	19644	-1.19	1.90E-02	2.39E-01	Intron (NM_001145367/25664, intron 1 of 7)
Hic1	303310	chr10	61979207	61979507	31267	1.34	1.90E-02	2.39E-01	Intron (NM_001105808/287522, intron 13 of 18)
Pias1	300772	chr8	68023959	68024259	-154944	0.62	1.90E-02	2.39E-01	Distal Intergenic
Mesd	308796	chr1	146140232	146140532	102806	1.37	1.90E-02	2.39E-01	Distal Intergenic
Bcl2l14	500348	chr4	168188199	168188499	48191	-1.39	1.90E-02	2.39E-01	Distal Intergenic
Kitlg	60427	chr7	42327477	42327777	57693	2.24	1.90E-02	2.39E-01	Intron (NM_021843/60427, intron 3 of 9)
Fbxo11	301674	chr6	11737254	11737554	-50572	0.98	1.90E-02	2.39E-01	Distal Intergenic
Csf1	78965	chr2	210608783	210609083	-58237	-1.59	1.90E-02	2.39E-01	Distal Intergenic
Tulp4	499016	chr1	47024035	47024335	-7977	-1.3	1.90E-02	2.39E-01	Distal Intergenic
Chd2	308738	chr1	134919858	134920158	-48690	-0.71	1.90E-02	2.39E-01	Distal Intergenic
Ciao2a	300797	chr8	71801463	71801763	15127	1.72	1.90E-02	2.39E-01	Distal Intergenic
Mast3	688540	chr16	20392777	20393077	-7500	-1.86	1.91E-02	2.39E-01	Distal Intergenic
Mapk1	116590	chr11	88245213	88245513	27781	-1.56	1.91E-02	2.39E-01	Intron (NM_053842/116590, intron 1 of 8)
Akna	362530	chr5	79226143	79226443	-3456	-1.56	1.91E-02	2.39E-01	Distal Intergenic
RGD1566265	363487	chrX	78911248	78911548	53	0.78	1.91E-02	2.39E-01	Promoter (<=1kb)
Il1f10	362077	chr3	1417349	1417649	3678	-1.73	1.91E-02	2.39E-01	Distal Intergenic
Matn4	296358	chr3	160860070	160860370	-6420	-1.32	1.91E-02	2.39E-01	Exon (NM_001108604/362268, exon 8 of 12)
Fez2	94269	chr6	1031769	1032069	-27845	-1.32	1.91E-02	2.39E-01	Distal Intergenic
Sema4f	29745	chr4	113729790	113730090	34442	1.04	1.91E-02	2.39E-01	Distal Intergenic
Fn1	25661	chr9	79078975	79079275	-109962	-1.43	1.91E-02	2.39E-01	Distal Intergenic
Cerk	300129	chr7	127060231	127060531	-2175	2.15	1.91E-02	2.39E-01	Promoter (2-3kb)
Nop58	60373	chr9	66501275	66501575	5394	-1.59	1.91E-02	2.39E-01	Exon (NM_021754/60373, exon 3 of 15)
Klhl9	313348	chr5	107329823	107330123	6565	-1.38	1.91E-02	2.39E-01	Distal Intergenic
Sdc2	25615	chr7	71572868	71573168	137	0.51	1.91E-02	2.39E-01	Promoter (<=1kb)
Rad21	314949	chr7	91538199	91538499	174	1.02	1.91E-02	2.39E-01	Promoter (<=1kb)
Mef2a	309957	chr1	128214262	128214562	126701	-1.41	1.91E-02	2.39E-01	Intron (NM_001014035/309957, intron 9 of 10)
Myo1b	117057	chr9	54586149	54586449	27947	1.91	1.91E-02	2.39E-01	Intron (NM_053986/117057, intron 1 of 30)
Nostrin	311111	chr3	55163699	55163999	-205283	-1.4	1.91E-02	2.39E-01	Distal Intergenic
Olah	64669	chr17	78878765	78879065	0	-1.18	1.91E-02	2.39E-01	Promoter (<=1kb)
Enpp6	306460	chr16	48492656	48492956	-55414	1.68	1.92E-02	2.39E-01	Distal Intergenic
Gmds	291095	chr17	33235475	33235775	-172947	-1.47	1.92E-02	2.39E-01	Distal Intergenic
Gpbp1	294734	chr2	43069314	43069614	-637	1.08	1.92E-02	2.39E-01	Promoter (<=1kb)
Zfp532	307362	chr18	61259757	61260057	-1361	1.11	1.92E-02	2.39E-01	Promoter (1-2kb)
Gpsm1	246254	chr3	3767818	3768118	424	0.68	1.92E-02	2.39E-01	Promoter (<=1kb)
Ldlrap1	500564	chr5	152986949	152987249	37	0.9	1.92E-02	2.39E-01	Promoter (<=1kb)
Dok1	312477	chr4	113847079	113847379	19295	-1.41	1.92E-02	2.39E-01	Distal Intergenic
Itpkb	54260	chr13	98649146	98649446	33859	0.6	1.92E-02	2.39E-01	Intron (NM_019312/54260, intron 2 of 7)
Slc38a1	170567	chr7	138026191	138026491	13139	-1.6	1.92E-02	2.39E-01	Intron (NM_138832/170567, intron 1 of 15)
Hic1	303310	chr10	61891460	61891760	119014	1.71	1.92E-02	2.39E-01	Intron (NM_001105808/287522, intron 12 of 18)
Isca1	290985	chr17	4919791	4920091	-361636	-1.31	1.92E-02	2.39E-01	Distal Intergenic
Rnf139	315000	chr7	98776117	98776417	5277	-1.37	1.92E-02	2.39E-01	Intron (NM_001127545/315000, intron 1 of 1)
Ptpn14	305064	chr13	108757341	108757641	83841	0.86	1.92E-02	2.39E-01	Intron (NM_001107200/305064, intron 3 of 17)
Gnb1	24400	chr5	172926964	172927264	-7726	-1.48	1.92E-02	2.39E-01	Distal Intergenic
Mmp14	81707	chr15	33085377	33085677	10936	-1.31	1.92E-02	2.39E-01	Distal Intergenic
Mkks	311456	chr3	129878839	129879139	5671	-1.45	1.92E-02	2.39E-01	Intron (NM_001008353/311456, intron 1 of 5)

Tut4	313481	chr5	128064424	128064724	544	1.05	1.92E-02	2.39E-01	Promoter (<=1kb)
Swi5	499779	chr3	11313002	11313302	3747	-1.4	1.92E-02	2.39E-01	Intron (NM_001246661/499779, intron 2 of 4)
Pdgfa	25266	chr12	17752043	17752343	17902	-1.46	1.92E-02	2.39E-01	Intron (NM_012801/25266, intron 6 of 7)
Tcf7l1	312451	chr4	100659884	100660184	0	1.11	1.92E-02	2.39E-01	Promoter (<=1kb)
Lims1	499443	chr20	27896077	27896377	-58190	1.01	1.92E-02	2.39E-01	Distal Intergenic
Cdyl	361237	chr17	29808881	29809181	86205	1.18	1.92E-02	2.39E-01	Intron (NM_001014145/361237, intron 3 of 9)
Dlx3	287638	chr10	82918872	82919172	-18799	1.64	1.93E-02	2.40E-01	Distal Intergenic
Ppm1b	24667	chr6	8226830	8227130	6602	-1.6	1.93E-02	2.40E-01	Intron (NM_001270620/24667, intron 2 of 7)
Tulp4	499016	chr1	47049141	47049441	16829	-1.44	1.93E-02	2.40E-01	Intron (NM_001109137/499016, intron 1 of 13)
Mpzl1	360871	chr13	83846774	83847074	-4756	-1.74	1.93E-02	2.40E-01	Distal Intergenic
Lrch1	502020	chr15	56939926	56940226	30139	-1.58	1.93E-02	2.40E-01	Intron (NM_001134727/502020, intron 1 of 18)
Mitf	25094	chr4	130181460	130181760	8604	-1.42	1.93E-02	2.40E-01	Intron (NM_001191089/25094, intron 1 of 9)
Rnf144b	364681	chr17	17863583	17863883	83894	-1.46	1.93E-02	2.40E-01	Intron (NM_001108881/364681, intron 1 of 7)
Wwc2	498630	chr16	47369317	47369617	549	0.94	1.93E-02	2.40E-01	Promoter (<=1kb)
Dipk1a	360906	chr14	2819074	2819374	29376	1.55	1.93E-02	2.40E-01	Intron (NM_001170456/360906, intron 1 of 4)
Tnrc18	304302	chr12	13828359	13828659	20540	1.9	1.93E-02	2.40E-01	Intron (NM_001107123/304302, intron 2 of 34)
Ncor1	54299	chr10	48772586	48772886	4	1.03	1.93E-02	2.40E-01	Promoter (<=1kb)
Ccnd1	58919	chr1	218190872	218191172	-90600	-1.26	1.93E-02	2.40E-01	Distal Intergenic
Mir21	100314000	chr10	73932108	73932408	-29807	-1.57	1.93E-02	2.40E-01	Intron (NM_138839/192129, intron 7 of 11)
Grap	363616	chr10	47925454	47925754	-4879	-1.45	1.93E-02	2.40E-01	Distal Intergenic
Shq1	297483	chr4	133137620	133137920	-10445	0.91	1.93E-02	2.40E-01	Distal Intergenic
Znrf2	362367	chr4	85015410	85015710	6060	-1.36	1.93E-02	2.40E-01	Intron (NM_001108628/362367, intron 3 of 6)
Irak3	314870	chr7	64902037	64902337	79887	-1.06	1.93E-02	2.40E-01	Distal Intergenic
Pef1	297900	chr5	148324739	148325039	4129	-1.24	1.94E-02	2.40E-01	Intron (NM_001007651/297900, intron 1 of 4)
Rragc	298514	chr5	141572729	141573029	193	0.87	1.94E-02	2.40E-01	Promoter (<=1kb)
Rbms2	288771	chr7	2591320	2591620	-2634	-1.7	1.94E-02	2.40E-01	Promoter (2-3kb)
Ext1	299907	chr7	92880454	92880754	638	0.54	1.94E-02	2.40E-01	Promoter (<=1kb)
B4galt6	65196	chr18	15466522	15466822	3609	-1.51	1.94E-02	2.40E-01	Intron (NM_001101000/679221, intron 3 of 6)
Fndc3b	294925	chr2	113414454	113414754	-68903	0.79	1.94E-02	2.40E-01	Distal Intergenic
Mir193	100314244	chr10	67058370	67058670	-7226	0.67	1.94E-02	2.40E-01	Distal Intergenic
Tfpi	29436	chr3	71899787	71900087	-3368	-1.42	1.94E-02	2.40E-01	Distal Intergenic
Foxc1	364706	chr17	33734125	33734425	217059	1.98	1.94E-02	2.40E-01	Intron (NM_001039606/291095, intron 7 of 10)
Ncor2	360801	chr12	36950232	36950532	78233	1.56	1.94E-02	2.40E-01	Intron (NM_001108334/360801, intron 7 of 46)
Sox9	140586	chr10	101288760	101289060	232	0.71	1.94E-02	2.40E-01	Promoter (<=1kb)
Pla2g1b	29526	chr12	46882919	46883219	5848	-1.33	1.94E-02	2.40E-01	Exon (NM_031585/29526, exon 2 of 4)
Fn1	25661	chr9	79240695	79240995	-271682	-1.41	1.95E-02	2.40E-01	Distal Intergenic
Srsf10	362630	chr5	154146046	154146346	-59056	1.62	1.95E-02	2.40E-01	Distal Intergenic
Pld1	25096	chr2	113660414	113660714	-35440	-1.89	1.95E-02	2.41E-01	Distal Intergenic
Arhgef3	290541	chr16	3082096	3082396	123813	-1.71	1.95E-02	2.41E-01	Distal Intergenic
Uchl1	29545	chr14	43291728	43292028	-147786	1.74	1.95E-02	2.41E-01	Distal Intergenic
Fermt2	289992	chr15	19876139	19876439	0	1.89	1.95E-02	2.41E-01	Promoter (<=1kb)
Lnx1	360926	chr14	35960507	35960807	-86337	1.78	1.95E-02	2.41E-01	Distal Intergenic
Ccdc82	300359	chr8	11995867	11996167	107347	-1.5	1.95E-02	2.41E-01	Distal Intergenic
Czib	298384	chr5	127489547	127489847	129	0.76	1.95E-02	2.41E-01	Promoter (<=1kb)
Smad6	367100	chr8	68951401	68951701	14407	-1.36	1.95E-02	2.41E-01	Intron (NM_001109002/367100, intron 3 of 3)
Aplp2	64312	chr8	32347152	32347452	-18331	-1.13	1.95E-02	2.41E-01	Distal Intergenic
Ank3	361833	chr20	20318140	20318440	213093	1.76	1.95E-02	2.41E-01	Exon (NM_031805/361833, exon 9 of 44)
Zc3h15	362154	chr3	71044739	71045039	24205	-1.17	1.96E-02	2.41E-01	Distal Intergenic
Magi2	113970	chr4	12472129	12472429	0	0.73	1.96E-02	2.41E-01	Promoter (<=1kb)
Sparc	24791	chr10	40753751	40754051	10090	1.44	1.96E-02	2.41E-01	Intron (NM_012656/24791, intron 2 of 9)
Hnrnpa3	362152	chr3	62439872	62440172	-41071	-1.42	1.96E-02	2.41E-01	Distal Intergenic
Cars2	361184	chr16	83288666	83288966	2	0.7	1.96E-02	2.41E-01	Promoter (<=1kb)
Zap70	301348	chr9	43515306	43515606	184157	1.19	1.96E-02	2.41E-01	Distal Intergenic
Otulin	100362554	chr2	80281859	80282159	11027	-1.65	1.96E-02	2.41E-01	Exon (NM_001302889/100362554, exon 3 of 7)
Spin1	361217	chr16	1045061	1045361	-114534	1.35	1.96E-02	2.41E-01	Distal Intergenic
Efcab9	688481	chr10	17322760	17323060	92191	-1.41	1.96E-02	2.41E-01	Distal Intergenic
Tnnt2	24837	chr13	52663216	52663516	-4453	1.63	1.96E-02	2.41E-01	Distal Intergenic
Hmga2	84017	chr7	65267183	65267483	7925	-1.55	1.96E-02	2.41E-01	Intron (NM_032070/84017, intron 2 of 4)
Cbfa2t3	361431	chr19	55536091	55536391	-25631	1.61	1.96E-02	2.41E-01	Distal Intergenic
Tex26	498133	chr12	6590135	6590435	113269	-0.81	1.96E-02	2.41E-01	Distal Intergenic
Ccn4	65154	chr7	107695777	107696077	550	0.62	1.96E-02	2.41E-01	Promoter (<=1kb)
Fgd3	361223	chr17	15736952	15737252	-12711	-1.45	1.96E-02	2.41E-01	Distal Intergenic
Bnc2	298189	chr5	102801962	102802262	-58545	-1.51	1.96E-02	2.41E-01	Distal Intergenic
Nrf1	312195	chr4	57336844	57337144	179	1.76	1.96E-02	2.41E-01	Promoter (<=1kb)
Fgfrl1	360903	chr14	2008905	2009205	22964	2.15	1.96E-02	2.41E-01	Distal Intergenic
Htatip2	292935	chr1	105074107	105074407	-20435	1.19	1.96E-02	2.41E-01	Distal Intergenic
Prss42	301027	chr8	119058813	119059113	-5848	1.66	1.96E-02	2.41E-01	Distal Intergenic
Nudt2	297998	chr5	57845710	57846010	0	0.67	1.96E-02	2.41E-01	Promoter (<=1kb)
Pawr	64513	chr7	51277245	51277545	75523	-1.53	1.97E-02	2.42E-01	Intron (NM_033485/64513, intron 4 of 5)
F5	304929	chr13	82463642	82463942	-16055	-1.02	1.97E-02	2.42E-01	3' UTR
Csk	315707	chr8	62414418	62414718	-4080	-1.52	1.97E-02	2.42E-01	Distal Intergenic
Fbxo33	314157	chr6	80334317	80334617	0	1.19	1.97E-02	2.42E-01	Promoter (<=1kb)
Scel	361086	chr15	87389843	87390143	-314197	1.32	1.97E-02	2.42E-01	Distal Intergenic
Inpp5f	309008	chr1	200037895	200038195	146	0.98	1.97E-02	2.42E-01	Promoter (<=1kb)
Foxq1	64826	chr17	34153098	34153398	73543	-1.21	1.97E-02	2.42E-01	Distal Intergenic
Mab21l1	688394	chr2	145483663	145483963	308787	-1.2	1.97E-02	2.42E-01	Distal Intergenic
Paip2	361309	chr18	28396585	28396885	-1852	-1.27	1.97E-02	2.42E-01	Promoter (1-2kb)
Angpt1	89807	chr7	81593346	81593646	-1047	-1.65	1.97E-02	2.42E-01	Promoter (1-2kb)
Zadh2	291403	chr18	81172906	81173206	233031	1.97	1.97E-02	2.42E-01	Distal Intergenic
Fbxo42	362646	chr5	159677969	159678269	7307	1.95	1.97E-02	2.42E-01	Intron (NM_001108691/362646, intron 1 of 9)
Vav1	25156	chr9	9681388	9681688	-6221	-1.57	1.97E-02	2.42E-01	Distal Intergenic
Zfp592	293038	chr1	142820236	142820536	-14682	0.83	1.98E-02	2.42E-01	Distal Intergenic
Anxa3	25291	chr14	14322188	14322488	103949	-1.51	1.98E-02	2.42E-01	Distal Intergenic
Itgb5	257645	chr11	70171775	70172075	89	0.94	1.98E-02	2.42E-01	Promoter (<=1kb)
Tjp1	292994	chr1	126227196	126227496	-15757	1.16	1.98E-02	2.42E-01	Distal Intergenic
Dnajb6	362293	chr4	2720279	2720579	8894	-1.45	1.98E-02	2.42E-01	Intron (NM_001013209/362293, intron 1 of 9)
Chek2	114212	chr12	51644985	51645285	232339	-1.3	1.98E-02	2.42E-01	Distal Intergenic
Gfi1	24388	chr14	3077815	3078115	18822	2.04	1.98E-02	2.42E-01	Distal Intergenic
Gpr3	266769	chr5	151371108	151371408	25099	-1.16	1.98E-02	2.42E-01	Exon (NM_001013167/313024, exon 4 of 20)
Fadd	266610	chr1	217734460	217734760	13821	-1.64	1.98E-02	2.42E-01	Intron (NM_001177828/100364769, intron 11 of 11)
Camk2d	24246	chr2	230966461	230966761	65335	-1.17	1.98E-02	2.42E-01	Intron (NM_012519/24246, intron 3 of 20)
Usp24	313427	chr5	125357949	125358249	-538476	1.99	1.98E-02	2.42E-01	Distal Intergenic
Ier5l	499772	chr3	9094283	9094583	-56341	-1.55	1.98E-02	2.42E-01	Distal Intergenic
Evi5	100360066	chr14	2898239	2898539	5486	1.61	1.98E-02	2.42E-01	Intron (NM_001271410/100360066, intron 1 of 17)

Zfyve26	314265	chr6	102538163	102538463	-65237	-1.45	1.98E-02	2.42E-01	Distal Intergenic
Mchr1	83567	chr7	122388733	122389033	-67813	-1.04	1.99E-02	2.42E-01	Distal Intergenic
Il1r1	25663	chr9	47003148	47003448	5350	-1.5	1.99E-02	2.42E-01	Intron (NM_013123/25663, intron 1 of 10)
Baz1b	368002	chr12	24530215	24530515	6336	-1.2	1.99E-02	2.42E-01	Intron (NM_001191916/368002, intron 1 of 18)
Calml4	691455	chr8	67753446	67753746	167	0.81	1.99E-02	2.42E-01	Promoter (<=1kb)
Nipsnap3b	313211	chr5	69833232	69833532	0	0.63	1.99E-02	2.42E-01	Promoter (<=1kb)
Hexb	294673	chr2	28002983	28003283	0	0.74	1.99E-02	2.42E-01	Promoter (<=1kb)
Rbms1	362138	chr3	46934761	46935061	90067	1.33	1.99E-02	2.42E-01	Intron (NM_001012184/362138, intron 1 of 13)
Spin1	361217	chr16	1179660	1179960	-249133	-1.09	1.99E-02	2.42E-01	Distal Intergenic
Ccdc120	317377	chrX	15608586	15608886	-1344	1.2	1.99E-02	2.42E-01	Promoter (1-2kb)
Zfr	365703	chr2	62150585	62150885	334	0.99	1.99E-02	2.42E-01	Promoter (<=1kb)
Fxr1	361927	chr2	120562893	120563193	7160	-1.71	1.99E-02	2.42E-01	Intron (NM_001012179/361927, intron 1 of 13)
Gata3	85471	chr17	72420220	72420520	-10297	-1.14	1.99E-02	2.42E-01	Distal Intergenic
Krt71	683613	chr7	143326972	143327272	26714	1.85	1.99E-02	2.42E-01	Intron (NM_183333/369017, intron 1 of 8)
Tcp11l2	314683	chr7	24893544	24893844	-7769	0.72	1.99E-02	2.43E-01	Distal Intergenic
Shq1	297483	chr4	133193719	133194019	-66544	1.89	1.99E-02	2.43E-01	Distal Intergenic
Pgam2	24959	chr14	86039849	86040149	6967	-1.23	1.99E-02	2.43E-01	Intron (NM_001277211/83527, intron 5 of 12)
Nrp2	81527	chr9	69655034	69655334	157600	1.66	2.00E-02	2.43E-01	Distal Intergenic
Tspo	24230	chr7	124460363	124460663	5	0.66	2.00E-02	2.43E-01	Promoter (<=1kb)
Smarcd2	83833	chr10	94460354	94460654	78	1.07	2.00E-02	2.43E-01	Promoter (<=1kb)
Zp3	114639	chr12	23760295	23760595	-18105	0.6	2.00E-02	2.43E-01	Distal Intergenic
Cgref1	245918	chr6	26797170	26797470	44	0.83	2.00E-02	2.43E-01	Promoter (<=1kb)
Myh9	100911597	chr7	119084096	119084396	-12374	1.83	2.00E-02	2.43E-01	Distal Intergenic
Lrr1	685860	chr6	91465535	91465835	2227	-1.19	2.00E-02	2.43E-01	Promoter (2-3kb)
Cul1	362356	chr4	77006947	77007247	-204567	1.66	2.00E-02	2.43E-01	Distal Intergenic
Smarce1	303518	chr10	87132487	87132787	5172	-1.59	2.00E-02	2.43E-01	Intron (NM_001024993/303518, intron 3 of 10)
Mir6316	102466622	chr1	278198263	278198563	-100112	0.89	2.00E-02	2.43E-01	Distal Intergenic
S100a10	81778	chr2	193932473	193932773	39884	1.44	2.00E-02	2.43E-01	Distal Intergenic
Lrrc8c	289443	chr14	5374197	5374497	4201	-1.38	2.00E-02	2.43E-01	Intron (NM_001037179/289443, intron 1 of 3)
Dusp26	306527	chr16	65091278	65091578	-285228	0.89	2.00E-02	2.43E-01	Distal Intergenic
Lrrc20	499430	chr20	31096251	31096551	37	0.62	2.00E-02	2.43E-01	Promoter (<=1kb)
Tmem230	681315	chr3	124856302	124856602	22614	1.11	2.00E-02	2.43E-01	Distal Intergenic
Snx7	310815	chr2	221052496	221052796	46942	1.94	2.00E-02	2.43E-01	Intron (NM_001012083/310815, intron 7 of 8)
Syde1	362842	chr7	14037802	14038102	156	0.73	2.01E-02	2.43E-01	Promoter (<=1kb)
Nudt12	367323	chr9	105693036	105693336	21	0.61	2.01E-02	2.43E-01	Promoter (<=1kb)
Serpine2	29366	chr9	85601777	85602077	24017	-1.78	2.01E-02	2.43E-01	Intron (NM_019197/29366, intron 1 of 8)
Steap3	170824	chr13	36288026	36288326	-13880	-1.63	2.01E-02	2.43E-01	Distal Intergenic
Slc7a7	83509	chr15	33048163	33048463	3834	-1.54	2.01E-02	2.43E-01	Intron (NM_031341/83509, intron 2 of 9)
Malrd1	100361092	chr17	82313183	82313483	-117353	1	2.01E-02	2.43E-01	Distal Intergenic
Nrep	338475	chr18	26212500	26212800	-1055	1.1	2.01E-02	2.43E-01	Promoter (1-2kb)
Tcf4	84382	chr18	65168854	65169154	-116166	0.75	2.01E-02	2.43E-01	Distal Intergenic
Pip4k2b	89812	chr10	85683738	85684038	100	0.91	2.01E-02	2.43E-01	Promoter (<=1kb)
Tbk1	299827	chr7	63687713	63688013	0	0.79	2.01E-02	2.43E-01	Promoter (<=1kb)
Ccnb2	363088	chr1	21238294	21238594	-91448	-1.34	2.01E-02	2.43E-01	Intron (NM_001001801/361458, intron 6 of 6)
Jdp2	116674	chr6	109465863	109466163	-8277	1.26	2.01E-02	2.43E-01	Distal Intergenic
Ctla4	63835	chr9	67632677	67632977	-66538	-1.55	2.01E-02	2.43E-01	Distal Intergenic
Kcnrg	305947	chr15	41997320	41997620	59440	-1.47	2.01E-02	2.43E-01	Distal Intergenic
Syndig1	362235	chr3	144845522	144845822	-186355	-1.52	2.02E-02	2.43E-01	Distal Intergenic
Kpna5	294392	chr20	32509618	32509918	-3755	0.69	2.02E-02	2.43E-01	Distal Intergenic
B4galt6	65196	chr18	15414199	15414499	-48414	-1.15	2.02E-02	2.43E-01	Intron (NM_001101000/679221, intron 3 of 6)
Cry1	299691	chr7	24534553	24534853	0	1.12	2.02E-02	2.43E-01	Promoter (<=1kb)
Ddx17	315133	chr7	120767211	120767511	-2199	-1.22	2.02E-02	2.43E-01	Promoter (2-3kb)
Myo9b	25486	chr16	19695676	19695976	26265	1.47	2.02E-02	2.43E-01	Intron (NM_001271066/25486, intron 2 of 41)
Zeb2	311071	chr3	29924633	29924933	59268	-1.62	2.02E-02	2.43E-01	Intron (NM_001033701/311071, intron 1 of 8)
Slc39a14	306009	chr15	52003556	52003856	25960	-1.65	2.02E-02	2.43E-01	Intron (NM_001107275/306009, intron 2 of 9)
Rpl31	64298	chr9	46032813	46033113	-53846	-1.66	2.02E-02	2.43E-01	Intron (NM_001108214/316351, intron 7 of 20)
Itprid2	311146	chr3	66603349	66603649	9574	-1.38	2.02E-02	2.43E-01	Intron (NM_001107738/311146, intron 8 of 17)
Dtd2	366619	chr6	72642108	72642408	144427	-1.44	2.02E-02	2.43E-01	Distal Intergenic
Rraga	117044	chr5	104363500	104363800	-577267	1.2	2.02E-02	2.43E-01	Distal Intergenic
Dock8	499337	chr1	242959562	242959862	78	0.96	2.02E-02	2.43E-01	Promoter (<=1kb)
Serpine1	24617	chr12	22647166	22647466	6062	-1.43	2.02E-02	2.43E-01	Intron (NM_012620/24617, intron 5 of 8)
Sag	25539	chr9	94998260	94998560	71194	-1.41	2.02E-02	2.43E-01	Distal Intergenic
Cbx8	303731	chr10	108154495	108154795	-3984	1.87	2.02E-02	2.43E-01	Intron (NM_001134688/287867, intron 1 of 26)
Stk38l	691337	chr4	181027314	181027614	102	0.94	2.02E-02	2.43E-01	Promoter (<=1kb)
Hes1	29577	chr11	74308941	74309241	6008	0.81	2.02E-02	2.43E-01	Distal Intergenic
Amacr	25284	chr2	60949256	60949556	0	0.71	2.02E-02	2.43E-01	Promoter (<=1kb)
Hrh2	25461	chr17	10850708	10851008	79053	1.83	2.02E-02	2.43E-01	Distal Intergenic
Wdfy1	301549	chr9	85510044	85510344	18516	-1.57	2.02E-02	2.43E-01	Intron (NM_001008331/301549, intron 1 of 10)
Tnfrsf26	361685	chr1	216836147	216836447	-7566	-1.36	2.02E-02	2.43E-01	Distal Intergenic
Pth1r	56813	chr8	119007702	119008002	0	2.07	2.03E-02	2.43E-01	Promoter (<=1kb)
Dad1	192275	chr15	32852403	32852703	35230	-1.62	2.03E-02	2.43E-01	Distal Intergenic
Pum1	362609	chr5	148788160	148788460	6893	-0.93	2.03E-02	2.43E-01	Intron (NM_001108684/362609, intron 1 of 21)
Pex6	117265	chr9	16575327	16575627	5273	1.95	2.03E-02	2.43E-01	Intron (NM_057125/117265, intron 3 of 16)
Terf2ip	307861	chr19	44231482	44231782	165	0.69	2.03E-02	2.43E-01	Promoter (<=1kb)
Tmed5	289883	chr14	2613193	2613493	0	0.66	2.03E-02	2.43E-01	Promoter (<=1kb)
Il6r	24499	chr2	189197567	189197867	56685	-1.35	2.03E-02	2.43E-01	Distal Intergenic
Stx2	25130	chr12	31335395	31335695	0	0.82	2.03E-02	2.43E-01	Promoter (<=1kb)
Slc48a1	300191	chr7	139303521	139303821	31823	1.31	2.03E-02	2.43E-01	Distal Intergenic
Rgcc	117183	chr15	61590325	61590625	-25630	1.82	2.03E-02	2.43E-01	Distal Intergenic
Ptpre	114767	chr1	207947030	207947330	0	-1.56	2.03E-02	2.43E-01	Promoter (<=1kb)
Slfn2	303380	chr10	70378425	70378725	8044	-1.17	2.03E-02	2.43E-01	Distal Intergenic
Ash1l	310638	chr2	188253060	188253360	-18	0.86	2.03E-02	2.43E-01	Promoter (<=1kb)
Arglu1	290912	chr16	86092561	86092861	-508016	1.93	2.03E-02	2.43E-01	Distal Intergenic
Srcin1	56029	chr10	85474917	85475217	42447	1.37	2.03E-02	2.43E-01	Intron (NM_019378/56029, intron 10 of 17)
Rflna	689711	chr12	36991669	36991969	55659	0.66	2.03E-02	2.43E-01	Intron (NM_001108334/360801, intron 17 of 46)
Echs1	140547	chr1	141775222	141775522	-46418	1.94	2.03E-02	2.43E-01	Distal Intergenic
Virma	313061	chr5	25046672	25046972	3900	-1.59	2.03E-02	2.43E-01	Intron (NM_001107915/313061, intron 1 of 24)
Rbms1	362138	chr3	46879669	46879969	145159	-1.55	2.03E-02	2.43E-01	Intron (NM_001012184/362138, intron 2 of 13)
Alcam	79559	chr11	50777554	50777854	-3273	2.09	2.03E-02	2.43E-01	Distal Intergenic
Pdhx	311254	chr3	92864410	92864710	69015	-1.51	2.03E-02	2.43E-01	Distal Intergenic
Sfi1	305467	chr14	83337926	83338226	66707	0.63	2.04E-02	2.43E-01	Distal Intergenic
Prm1	24685	chr10	4892312	4892612	-53383	1.45	2.04E-02	2.43E-01	Distal Intergenic
Efna5	116683	chr9	110329997	110330297	-310	1.12	2.04E-02	2.43E-01	Promoter (<=1kb)
Rad51c	497976	chr10	74765756	74766056	-41809	1.5	2.04E-02	2.43E-01	Distal Intergenic

Plcb4	25031	chr3	128601153	128601453	0	1.51	2.04E-02	2.44E-01	Promoter (<=1kb)
Camkk2	83506	chr12	39253609	39253909	200	0.91	2.04E-02	2.44E-01	Promoter (<=1kb)
Sos1	313845	chr6	3182904	3183204	0	1.37	2.04E-02	2.44E-01	Promoter (<=1kb)
Me3	361602	chr1	153878083	153878383	16514	1.99	2.04E-02	2.44E-01	Intron (NM_001108491/361602, intron 2 of 14)
Vamp3	29528	chr5	168394906	168395206	-258278	1.77	2.04E-02	2.44E-01	Intron (NM_001195559/362665, intron 5 of 28)
Il17ra	312679	chr4	152995868	152996168	3	0.87	2.04E-02	2.44E-01	Promoter (<=1kb)
Mbp	24547	chr18	79509260	79509560	102879	2.16	2.04E-02	2.44E-01	Distal Intergenic
Itsn1	29491	chr11	31893479	31893779	0	1.62	2.04E-02	2.44E-01	Promoter (<=1kb)
Gja1	24392	chr20	38173263	38173563	296613	-1.79	2.05E-02	2.44E-01	Distal Intergenic
Ror1	362550	chr5	118893068	118893368	194	1.02	2.05E-02	2.44E-01	Promoter (<=1kb)
Cstf1	311670	chr3	170380581	170380881	0	0.54	2.05E-02	2.44E-01	Promoter (<=1kb)
Hecw2	316395	chr9	60587538	60587838	84408	-1.63	2.05E-02	2.44E-01	Intron (NM_001108218/316395, intron 3 of 27)
Smad5	59328	chr17	8371217	8371517	-48797	0.88	2.05E-02	2.44E-01	Distal Intergenic
Synpo	60324	chr18	55900318	55900618	-8608	1.6	2.05E-02	2.44E-01	Distal Intergenic
Spsb4	300950	chr8	104882826	104883126	29833	1.19	2.05E-02	2.44E-01	Intron (NM_001106849/300950, intron 2 of 2)
Rb1cc1	312927	chr5	13097024	13097324	17	0.96	2.05E-02	2.44E-01	Promoter (<=1kb)
Wipi1	303630	chr10	97896170	97896470	27	0.72	2.05E-02	2.44E-01	Promoter (<=1kb)
Dlx2	296499	chr3	58274361	58274661	-92390	-1.57	2.05E-02	2.44E-01	Distal Intergenic
Tmem19	299800	chr7	58360497	58360797	-17360	-1.57	2.05E-02	2.44E-01	Distal Intergenic
Stx8	59074	chr10	54620962	54621262	107977	-1.11	2.05E-02	2.44E-01	Intron (NM_031656/59074, intron 6 of 7)
Itga7	81008	chr7	3359430	3359730	4171	-1.2	2.05E-02	2.44E-01	Intron (NM_030842/81008, intron 1 of 24)
Mrln	100911740	chr20	19824619	19824919	-105971	0.71	2.05E-02	2.44E-01	Distal Intergenic
Cops8	363283	chr9	97678007	97678307	-93917	-1.68	2.05E-02	2.44E-01	Distal Intergenic
Agr3	298959	chr6	55330622	55330922	-40816	-1.53	2.06E-02	2.44E-01	Distal Intergenic
Ralgds	29622	chr3	7090314	7090614	-19306	1.3	2.06E-02	2.44E-01	Distal Intergenic
Ppp2r5c	691318	chr6	134882006	134882306	37347	1.6	2.06E-02	2.45E-01	Intron (NM_001191112/691318, intron 1 of 13)
Rab7a	29448	chr4	119930134	119930434	28739	-1.36	2.06E-02	2.45E-01	Intron (NM_023950/29448, intron 1 of 5)
Nsd3	290831	chr16	71190011	71190311	46807	-1.36	2.06E-02	2.45E-01	Intron (NM_001106090/290831, intron 4 of 22)
Bcl2	24224	chr13	26770848	26771148	-1474	1.28	2.06E-02	2.45E-01	Promoter (1-2kb)
Mob2	499288	chr1	215001164	215001464	9449	-1.53	2.06E-02	2.45E-01	Intron (NM_001109159/499288, intron 1 of 5)
Mpp1	652956	chr1	148442059	148442359	-7854	0.6	2.06E-02	2.45E-01	Distal Intergenic
Rgr	306307	chr16	14204025	14204325	96626	1.74	2.06E-02	2.45E-01	Distal Intergenic
Rab11fip2	308003	chr1	281101310	281101610	-147	1.3	2.06E-02	2.45E-01	Promoter (<=1kb)
Atxn2l	361649	chr1	197919031	197919331	149	0.9	2.06E-02	2.45E-01	Promoter (<=1kb)
Parva	57341	chr1	177287491	177287791	38189	-1.67	2.06E-02	2.45E-01	Intron (NM_020656/57341, intron 1 of 12)
Acot11	100363074	chr5	126442633	126442933	-46978	0.63	2.06E-02	2.45E-01	Distal Intergenic
Mir3596a	100526642	chr8	45773005	45773305	-19638	1.62	2.07E-02	2.45E-01	Intron (NR_126581/104845260, intron 3 of 3)
Oat	64313	chr1	204581644	204581944	126	0.65	2.07E-02	2.45E-01	Promoter (<=1kb)
Tpd52l1	689256	chr1	28379298	28379598	-75453	-1.71	2.07E-02	2.45E-01	Intron (NM_001106204/292188, intron 3 of 5)
Rbpj	679028	chr14	59818208	59818508	-82758	-1.71	2.07E-02	2.45E-01	Distal Intergenic
Ldhd	307858	chr19	43778656	43778956	69981	-1.71	2.07E-02	2.45E-01	Distal Intergenic
Nceh1	294930	chr2	112912219	112912519	43385	-1.71	2.07E-02	2.45E-01	Intron (NM_001127524/294930, intron 2 of 4)
Anks6	362515	chr5	62677287	62677587	6800	-1.43	2.07E-02	2.45E-01	Intron (NM_001015028/362515, intron 1 of 15)
Ralgds	29622	chr3	7110072	7110372	152	1.21	2.07E-02	2.45E-01	Promoter (<=1kb)
Nox4	85431	chr1	150796997	150797297	0	0.71	2.07E-02	2.45E-01	Promoter (<=1kb)
Snap23	64630	chr3	112290047	112290347	-43635	1.26	2.07E-02	2.45E-01	Distal Intergenic
Vgll4	297523	chr4	146821446	146821746	17651	1.9	2.07E-02	2.45E-01	Intron (NM_001015004/297523, intron 1 of 4)
Smtn	289734	chr14	83797225	83797525	-20362	-1.74	2.07E-02	2.45E-01	Distal Intergenic
Fndc3b	294925	chr2	113386685	113386985	-41134	-1.38	2.07E-02	2.45E-01	Distal Intergenic
RGD1311703	293160	chr1	185518724	185519024	50166	1.8	2.07E-02	2.45E-01	Distal Intergenic
Pdpk1	81745	chr10	13482711	13483011	32431	1.93	2.07E-02	2.45E-01	Exon (NM_031081/81745, exon 2 of 14)
Mnx1	682076	chr4	2506165	2506465	-124857	0.86	2.07E-02	2.45E-01	Distal Intergenic
Cited2	114490	chr1	12684444	12684744	-138619	-1.69	2.07E-02	2.45E-01	Distal Intergenic
Tmem63a	289318	chr13	99220540	99220840	30	0.79	2.07E-02	2.45E-01	Promoter (<=1kb)
Traf4	303285	chr10	65424373	65424673	129	0.83	2.07E-02	2.45E-01	Promoter (<=1kb)
Bbx	303970	chr11	52791841	52792141	-288884	1.04	2.07E-02	2.45E-01	Distal Intergenic
Pitx3	29609	chr1	265902143	265902443	-2199	-1.53	2.07E-02	2.45E-01	Promoter (2-3kb)
Nfu1	297416	chr4	118743229	118743529	-70755	-1.19	2.07E-02	2.45E-01	Intron (NM_001173450/500244, intron 4 of 20)
Pclaf	300795	chr8	71457207	71457507	-56774	1.87	2.08E-02	2.45E-01	Intron (NM_001134981/315769, intron 14 of 15)
Stx8	59074	chr10	54547931	54548231	34946	-1.61	2.08E-02	2.45E-01	Intron (NM_031656/59074, intron 4 of 7)
Fign	295649	chr3	50121262	50121562	-870	0.99	2.08E-02	2.45E-01	Promoter (<=1kb)
Plaat5	293711	chr1	222907497	222907797	338	-1.65	2.08E-02	2.45E-01	Promoter (<=1kb)
Kif27	246209	chr17	6701224	6701524	0	1.5	2.08E-02	2.45E-01	Promoter (<=1kb)
Elf2	361944	chr2	140396817	140397117	-9312	-1.34	2.08E-02	2.45E-01	Distal Intergenic
Lif	60584	chr14	84492955	84493255	-3215	-1.76	2.08E-02	2.45E-01	Distal Intergenic
Ttc7a	362696	chr6	10945477	10945777	33094	-0.91	2.08E-02	2.45E-01	Intron (NM_001100756/362696, intron 5 of 19)
Vdac3	83532	chr16	74299246	74299546	6780	-1.52	2.08E-02	2.45E-01	Intron (NM_031355/83532, intron 3 of 8)
Foxp1	297480	chr4	131398759	131399059	199245	1.63	2.08E-02	2.45E-01	Intron (NM_001034131/297480, intron 8 of 15)
LOC102547290	102547290	chr5	173212258	173212558	2348	1.74	2.08E-02	2.45E-01	Promoter (2-3kb)
Ppp4r2	297486	chr4	133285919	133286219	367	0.97	2.08E-02	2.45E-01	Promoter (<=1kb)
Zfp386	25165	chr6	141622778	141623078	-2278216	1.66	2.08E-02	2.45E-01	Distal Intergenic
Sfxn1	364678	chr17	11081647	11081947	77	1	2.08E-02	2.45E-01	Promoter (<=1kb)
Mir140	100314276	chr19	39603397	39603697	-5254	0.64	2.08E-02	2.45E-01	Intron (NM_001106184/291999, intron 7 of 19)
Adgrl4	64124	chr2	256455909	256456209	-153578	-1.11	2.09E-02	2.45E-01	Distal Intergenic
Cxcl1	81503	chr14	18754913	18755213	-9456	-1.54	2.09E-02	2.45E-01	Distal Intergenic
Zfyve28	305454	chr14	81725242	81725542	0	1.4	2.09E-02	2.45E-01	Promoter (<=1kb)
Fh	24368	chr13	93676995	93677295	76	0.63	2.09E-02	2.45E-01	Promoter (<=1kb)
Cdk6	114483	chr4	27934237	27934537	31861	-1.51	2.09E-02	2.46E-01	Intron (NM_001191861/114483, intron 1 of 6)
Anxa11	290527	chr16	3897799	3898099	13944	-1.42	2.09E-02	2.46E-01	Intron (NM_170787/259269, intron 10 of 16)
Rmdn2	313840	chr6	2089098	2089398	-127225	2.12	2.09E-02	2.46E-01	Distal Intergenic
Zmiz1	361103	chr16	1645620	1645920	-103271	1.82	2.09E-02	2.46E-01	Distal Intergenic
Dnajc24	362184	chr3	96065453	96065753	0	0.66	2.09E-02	2.46E-01	Promoter (<=1kb)
Dnase1l1	363522	chrX	156434218	156434518	4633	-1.53	2.09E-02	2.46E-01	Intron (NM_001014223/363522, intron 1 of 7)
Zfp706	500855	chr7	75744542	75744842	65011	1.51	2.09E-02	2.46E-01	Distal Intergenic
Cpq	58952	chr7	71709301	71709601	0	0.73	2.09E-02	2.46E-01	Promoter (<=1kb)
Ogdh	360975	chr14	86415151	86415451	175	0.72	2.09E-02	2.46E-01	Promoter (<=1kb)
Spns2	100270678	chr10	59061841	59062141	-12359	1.89	2.09E-02	2.46E-01	Intron (NM_001109035/497946, intron 8 of 10)
Palm2	103692368	chr5	74684860	74685160	35095	-1.41	2.09E-02	2.46E-01	Intron (NM_001305995/103692368, intron 1 of 5)
Wipf2	360620	chr10	86785257	86785557	-10230	-1.22	2.09E-02	2.46E-01	Distal Intergenic
Gtpbp10	312054	chr4	25635752	25636052	0	0.65	2.09E-02	2.46E-01	Promoter (<=1kb)
Krtap1-5	497995	chr10	87492435	87492735	8158	-1.34	2.09E-02	2.46E-01	Distal Intergenic
Pcp4l1	685448	chr13	89554892	89555192	10621	-1.22	2.10E-02	2.46E-01	Intron (NM_001126093/685448, intron 1 of 2)
Bcap29	298943	chr6	50923574	50923874	0	0.67	2.10E-02	2.46E-01	Promoter (<=1kb)
Rfc5	304528	chr12	44942434	44942734	10940	2.47	2.10E-02	2.46E-01	3' UTR

Kif2a	84391	chr2	38704120	38704420	-495355	-1.45	2.10E-02	2.46E-01	Distal Intergenic
Psma2	29669	chr17	53101911	53102211	113	0.6	2.10E-02	2.46E-01	Promoter (<=1kb)
Pde4d	24627	chr2	41422322	41422622	264340	-1.2	2.10E-02	2.46E-01	Intron (NM_001113329/24627, intron 7 of 15)
Ssbp1	54304	chr4	68634945	68635245	51	0.7	2.10E-02	2.46E-01	Promoter (<=1kb)
Oasl	304545	chr12	47462603	47462903	-6443	-0.89	2.10E-02	2.46E-01	Distal Intergenic
Eya3	313027	chr5	150842174	150842474	8355	-1.88	2.10E-02	2.46E-01	Intron (NM_001107910/313027, intron 1 of 17)
Thap6	305244	chr14	17616027	17616327	0	0.71	2.10E-02	2.46E-01	Promoter (<=1kb)
Enah	360891	chr13	100373936	100374236	31103	-1.44	2.10E-02	2.46E-01	Intron (NM_001012150/360891, intron 1 of 12)
Mir199a2	100314245	chr13	80071626	80071926	-53561	2.01	2.10E-02	2.46E-01	Intron (NM_138538/171574, intron 15 of 20)
Eps15	313474	chr5	128924078	128924378	269	1.05	2.10E-02	2.46E-01	Promoter (<=1kb)
Babam1	290631	chr16	19819351	19819651	26501	-1.43	2.10E-02	2.46E-01	Distal Intergenic
Lrp5	293649	chr1	218914907	218915207	4887	-1.3	2.11E-02	2.46E-01	Intron (NM_001106321/293649, intron 1 of 22)
Osr1	298878	chr6	35621330	35621630	302229	1.66	2.11E-02	2.46E-01	Distal Intergenic
Irf2	290749	chr16	48590920	48591220	101256	-1.32	2.11E-02	2.46E-01	Intron (NM_001047086/290749, intron 7 of 8)
Ednrb	50672	chr15	88207390	88207690	-171036	-1.65	2.11E-02	2.46E-01	Distal Intergenic
Slco3a1	140915	chr1	136073216	136073516	0	1.19	2.11E-02	2.46E-01	Promoter (<=1kb)
Ctsl	25697	chr17	1882340	1882640	-3077	-1.53	2.11E-02	2.46E-01	Distal Intergenic
Rab21	299799	chr7	58280193	58280493	6277	-1.37	2.11E-02	2.46E-01	Intron (NM_001004238/299799, intron 1 of 6)
Sidt2	315617	chr8	50245801	50246101	555	1.12	2.11E-02	2.46E-01	Promoter (<=1kb)
Zbtb43	311872	chr3	12470186	12470486	38819	0.76	2.11E-02	2.46E-01	Distal Intergenic
Ywhaz	25578	chr7	75596939	75597239	37	1.13	2.11E-02	2.46E-01	Promoter (<=1kb)
Gnaq	81666	chr1	233414771	233415071	31993	-1.09	2.11E-02	2.46E-01	Intron (NM_031036/81666, intron 1 of 6)
Morn4	293950	chr1	261316866	261317166	16217	-1.26	2.11E-02	2.46E-01	Intron (NM_001106355/293949, intron 6 of 6)
Elovl6	171402	chr2	234749055	234749355	373740	-1.54	2.11E-02	2.46E-01	Distal Intergenic
Slc1a5	292657	chr1	78719691	78719991	9005	-1.59	2.11E-02	2.46E-01	Intron (NM_175758/292657, intron 4 of 7)
Zap70	301348	chr9	43423664	43423964	92515	-1.59	2.11E-02	2.46E-01	Distal Intergenic
Armc8	315949	chr8	107818709	107819009	41	0.76	2.11E-02	2.46E-01	Promoter (<=1kb)
Bak1	116502	chr20	5608337	5608637	9623	0.89	2.11E-02	2.46E-01	Downstream (<1kb)
Rbm34	307956	chr19	59886614	59886914	8023	-1.21	2.11E-02	2.46E-01	Intron (NM_001014015/307956, intron 5 of 10)
Ociad1	289590	chr14	37524542	37524842	-49124	-1.48	2.11E-02	2.46E-01	Distal Intergenic
Cdc42ep3	313838	chr6	2045011	2045311	-102039	0.7	2.11E-02	2.46E-01	Distal Intergenic
Map7	293016	chr1	15647402	15647702	5249	-1.48	2.12E-02	2.46E-01	Intron (NM_001198638/293016, intron 1 of 15)
Sema6d	311384	chr3	116898971	116899271	-635	1.56	2.12E-02	2.46E-01	Promoter (<=1kb)
Anxa2	56611	chr8	75675948	75676248	-10886	-1.12	2.12E-02	2.46E-01	Distal Intergenic
Pdgfrl	290771	chr16	54446181	54446481	3945	1.03	2.12E-02	2.46E-01	Intron (NM_001011921/290771, intron 1 of 5)
Pdk1	116551	chr3	58751062	58751362	220192	-1.56	2.12E-02	2.46E-01	Distal Intergenic
Serpine2	29366	chr9	85617885	85618185	7909	-1.58	2.12E-02	2.46E-01	Intron (NM_019197/29366, intron 1 of 8)
Hdac9	687001	chr6	53965803	53966103	93016	-1.47	2.12E-02	2.46E-01	Intron (NM_001200045/687001, intron 2 of 25)
Herc3	362377	chr4	89085178	89085478	6467	-1.45	2.12E-02	2.46E-01	Intron (NM_001108631/362377, intron 2 of 25)
Slc6a7	117100	chr18	56300695	56300995	24184	2.11	2.12E-02	2.46E-01	Distal Intergenic
Slc24a1	56814	chr8	70413383	70413683	22651	-1.23	2.12E-02	2.46E-01	Exon (NM_020090/56814, exon 7 of 9)
Mcur1	291034	chr17	24182123	24182423	2	1.12	2.12E-02	2.46E-01	Promoter (<=1kb)
ST7	296911	chr4	45001181	45001481	126	1.37	2.12E-02	2.46E-01	Promoter (<=1kb)
St3gal4	363040	chr8	36306258	36306558	8253	-1.57	2.12E-02	2.46E-01	Intron (NM_203337/363040, intron 1 of 10)
Zfp458	499563	chr2	86946264	86946564	-24565	-1.64	2.12E-02	2.46E-01	Distal Intergenic
Rbms1	362138	chr3	46922088	46922388	102740	-1.64	2.12E-02	2.46E-01	Intron (NM_001012184/362138, intron 1 of 13)
Sox17	312936	chr5	14797022	14797322	-93086	-1.64	2.12E-02	2.46E-01	Distal Intergenic
Rap1b	171337	chr7	60871362	60871662	-10372	-1.64	2.12E-02	2.46E-01	Distal Intergenic
Hand1	59112	chr10	43210643	43210943	42353	1.39	2.12E-02	2.46E-01	Distal Intergenic
Jak2	24514	chr1	247398483	247398783	0	1.11	2.12E-02	2.46E-01	Promoter (<=1kb)
Yeats2	498112	chr11	84595032	84595332	-11490	1.07	2.12E-02	2.46E-01	Distal Intergenic
Ddx4	310090	chr2	44513044	44513344	-8898	0.68	2.12E-02	2.46E-01	Distal Intergenic
Sipa1l1	246212	chr6	105833094	105833394	-218818	1.72	2.12E-02	2.46E-01	Distal Intergenic
Gata6	29300	chr18	2702457	2702757	286636	1.47	2.12E-02	2.46E-01	Distal Intergenic
Wdr43	362703	chr6	23472743	23473043	126	1.1	2.12E-02	2.46E-01	Promoter (<=1kb)
Col26a1	685612	chr12	23071099	23071399	236233	1.14	2.12E-02	2.46E-01	Distal Intergenic
Pik3r1	25513	chr2	31981311	31981611	-164926	1.46	2.12E-02	2.46E-01	Distal Intergenic
Cop1	360860	chr13	76943415	76943715	-36896	1.11	2.12E-02	2.46E-01	Distal Intergenic
Arrdc3	309945	chr2	8800154	8800454	66987	-1.49	2.13E-02	2.46E-01	Distal Intergenic
Myc	24577	chr7	102721717	102722017	135404	-1.33	2.13E-02	2.46E-01	Distal Intergenic
Trmt112	293700	chr1	222172285	222172585	4358	-1.61	2.13E-02	2.46E-01	Intron (NM_001008511/293701, intron 2 of 6)
Ube3a	361585	chr1	116588131	116588431	316	0.91	2.13E-02	2.46E-01	Promoter (<=1kb)
Tmem65	500874	chr7	98698473	98698773	10840	1.98	2.13E-02	2.46E-01	Intron (NM_001100995/500874, intron 4 of 10)
Pde11a	140928	chr3	63167672	63167972	20056	-1.65	2.13E-02	2.46E-01	Intron (NM_080893/140928, intron 1 of 19)
Jak1	84598	chr5	120062276	120062576	21328	-1.6	2.13E-02	2.46E-01	Intron (NM_053466/84598, intron 1 of 24)
Atg10	688555	chr2	20409056	20409356	-256172	1.3	2.13E-02	2.46E-01	Distal Intergenic
Mir147	100314100	chr3	114775790	114776090	447	-1.51	2.13E-02	2.46E-01	Promoter (<=1kb)
Agtpbp1	290986	chr17	5864801	5865101	353416	1.35	2.13E-02	2.46E-01	Distal Intergenic
Itpr3	25679	chr20	5634996	5635296	-10801	-1.4	2.13E-02	2.46E-01	Distal Intergenic
Pdzk1ip1	81916	chr5	133902844	133903144	6946	-1.26	2.13E-02	2.46E-01	Distal Intergenic
Map7d1	681287	chr5	144258864	144259164	15817	-1.47	2.13E-02	2.46E-01	Exon (NM_001109438/681287, exon 5 of 18)
Lmo7	361084	chr15	86304471	86304771	61323	1.36	2.13E-02	2.46E-01	Intron (NM_001001515/361084, intron 1 of 30)
Nr4a2	54278	chr3	43128175	43128475	-10769	1.07	2.13E-02	2.46E-01	Distal Intergenic
Rbbp7	83712	chrX	33665148	33665448	373	1.18	2.13E-02	2.46E-01	Promoter (<=1kb)
Hgf	24446	chr4	15106747	15107047	398236	-1.19	2.13E-02	2.46E-01	Distal Intergenic
Lrrc51	293156	chr1	167030535	167030835	-13343	-1.62	2.13E-02	2.46E-01	Distal Intergenic
Fnip1	100362347	chr10	39786751	39787051	64	1.05	2.13E-02	2.46E-01	Promoter (<=1kb)
Gpr20	60667	chr7	114866932	114867232	78112	0.86	2.14E-02	2.46E-01	Distal Intergenic
Atf6b	406169	chr20	4417540	4417840	3108	1.86	2.14E-02	2.46E-01	Exon (NM_001002809/406169, exon 6 of 17)
Tnfaip8	307428	chr18	44708937	44709237	-27917	-1.12	2.14E-02	2.46E-01	Distal Intergenic
Tmem178a	362691	chr6	3615397	3615697	-41658	-1.58	2.14E-02	2.46E-01	Distal Intergenic
Hs3st3b1	303218	chr10	50395137	50395437	7147	-1.51	2.14E-02	2.46E-01	Intron (NM_001191646/303218, intron 1 of 1)
Stard5	502348	chr1	145814748	145815048	44613	-1.63	2.14E-02	2.46E-01	Intron (NM_001105749/116996, intron 6 of 18)
Ube2f	363284	chr9	98404181	98404481	10013	1.62	2.14E-02	2.46E-01	Intron (NM_001008381/363284, intron 2 of 8)
Ralbp1	84014	chr9	113588776	113589076	9402	-1.36	2.14E-02	2.46E-01	Exon (NM_032067/84014, exon 4 of 10)
Agps	84114	chr3	62599468	62599768	-48584	-1.63	2.14E-02	2.46E-01	Distal Intergenic
Ccdc93	304743	chr13	37400618	37400918	142	0.69	2.14E-02	2.46E-01	Promoter (<=1kb)
Tulp4	499016	chr1	47082898	47083198	50586	-1.51	2.14E-02	2.46E-01	Intron (NM_001109137/499016, intron 3 of 13)
Zfp458	499563	chr2	86602076	86602376	-368753	-1.58	2.14E-02	2.46E-01	Distal Intergenic
Nln	117041	chr2	34089135	34089435	96247	1.26	2.14E-02	2.46E-01	Intron (NM_053970/117041, intron 12 of 12)
Bbx	303970	chr11	52522916	52523216	-557809	-1.77	2.14E-02	2.46E-01	Distal Intergenic
Cdk14	362316	chr4	25932922	25933222	107355	-1.51	2.14E-02	2.46E-01	Intron (NM_001108617/362316, intron 2 of 12)
Tnc	116640	chr5	79852491	79852791	-2626	1.98	2.14E-02	2.46E-01	Promoter (2-3kb)
Pafah1b3	114113	chr1	82151247	82151547	14712	1.28	2.14E-02	2.46E-01	Distal Intergenic

Pik3ap1	294048	chr1	260744102	260744402	-105286	-1.39	2.14E-02	2.46E-01	Distal Intergenic
Pcdh19	317183	chrX	104493264	104493564	150	1.17	2.15E-02	2.46E-01	Promoter (<=1kb)
Fndc3a	306022	chr15	54582571	54582871	-54091	0.83	2.15E-02	2.46E-01	Distal Intergenic
Atp6ap1l	361875	chr2	19371237	19371537	437400	1.92	2.15E-02	2.47E-01	Distal Intergenic
Chst12	304322	chr12	16236705	16237005	-35077	1.05	2.15E-02	2.47E-01	Distal Intergenic
Olr1111	300205	chr7	140116718	140117018	9230	2	2.15E-02	2.47E-01	Distal Intergenic
Hnrnpa3	362152	chr3	62438916	62439216	-42027	-1.87	2.15E-02	2.47E-01	Distal Intergenic
Acsl3	114024	chr9	84561190	84561490	-8110	-1.58	2.15E-02	2.47E-01	Distal Intergenic
Dyrk2	314862	chr7	61871234	61871534	-72505	1.21	2.16E-02	2.47E-01	Distal Intergenic
Atf7	315333	chr7	144196926	144197226	26203	-1.48	2.16E-02	2.47E-01	Intron (NM_001108115/315333, intron 1 of 11)
Htra4	306564	chr16	71788286	71788586	320	1.34	2.16E-02	2.47E-01	Promoter (<=1kb)
Plekhj1	314634	chr7	11787159	11787459	2818	-1.23	2.16E-02	2.47E-01	Promoter (2-3kb)
St6galnac6	407765	chr3	11628501	11628801	-4790	0.79	2.16E-02	2.47E-01	Distal Intergenic
Emb	114511	chr2	49544683	49544983	-137780	1.65	2.16E-02	2.47E-01	Distal Intergenic
Slc43a3	311170	chr3	72344889	72345189	14858	-1.39	2.16E-02	2.47E-01	Intron (NM_001107743/311170, intron 10 of 12)
Fgl1	246186	chr16	54172735	54173035	19649	-1.52	2.16E-02	2.47E-01	Intron (NM_172010/246186, intron 3 of 8)
Ptch1	89830	chr17	1172309	1172609	-86424	1	2.16E-02	2.47E-01	Distal Intergenic
Sgcg	305941	chr15	41474634	41474934	120341	-0.86	2.16E-02	2.47E-01	Distal Intergenic
Mat2b	683630	chr10	25845351	25845651	0	0.61	2.16E-02	2.47E-01	Promoter (<=1kb)
Slc6a6	29464	chr4	123689713	123690013	23306	-1.33	2.16E-02	2.47E-01	Intron (NM_017206/29464, intron 2 of 14)
Lrrc14b	502225	chr1	31493291	31493591	-37552	-1.75	2.16E-02	2.47E-01	Distal Intergenic
Mef2a	309957	chr1	128261193	128261493	79770	-1.75	2.16E-02	2.47E-01	Intron (NM_001014035/309957, intron 4 of 10)
Bnip3	84480	chr1	211258679	211258979	6303	-1.75	2.16E-02	2.47E-01	Intron (NM_053420/84480, intron 1 of 5)
Cd47	29364	chr11	53453637	53453937	121238	-1.75	2.16E-02	2.47E-01	Distal Intergenic
Anapc4	305420	chr14	60500007	60500307	3496	-1.75	2.16E-02	2.47E-01	Intron (NM_001107220/305420, intron 2 of 27)
C1qtnf3	294806	chr2	60904633	60904933	-15324	-1.75	2.16E-02	2.47E-01	Distal Intergenic
LOC294154	294154	chr20	7385725	7386025	11368	-1.75	2.16E-02	2.47E-01	Intron (NM_001039607/294154, intron 1 of 4)
Ndfip2	361089	chr15	89400076	89400376	-7050	-1.64	2.17E-02	2.47E-01	Distal Intergenic
Ttc12	300696	chr8	53938468	53938768	-122015	1.4	2.17E-02	2.47E-01	Intron (NM_031521/24586, intron 1 of 18)
Arl8b	500282	chr4	140843871	140844171	6126	-1.43	2.17E-02	2.47E-01	Intron (NM_001024332/500282, intron 1 of 6)
Kifc3	307644	chr19	10305624	10305924	-14680	0.86	2.17E-02	2.47E-01	Distal Intergenic
Enpp4	301261	chr9	19448312	19448612	43	-1.25	2.17E-02	2.47E-01	Promoter (<=1kb)
Mir182	100314172	chr4	57455853	57456153	3681	-1.13	2.17E-02	2.47E-01	Distal Intergenic
LOC688459	688459	chr9	23403091	23403391	-17263	-1.28	2.17E-02	2.47E-01	Distal Intergenic
Arl6ip5	66028	chr4	129846332	129846632	271969	0.85	2.17E-02	2.47E-01	Distal Intergenic
Slc38a4	170573	chr7	138467838	138468138	44814	0.7	2.17E-02	2.47E-01	Intron (NM_130748/170573, intron 13 of 15)
Tomm70	304017	chr11	45507338	45507638	3460	-1.62	2.17E-02	2.48E-01	Intron (NM_212519/304017, intron 1 of 13)
Vcan	114122	chr2	18590864	18591164	-3524	-1.32	2.17E-02	2.48E-01	Distal Intergenic
Arl5a	117050	chr3	37938992	37939292	8142	-1.07	2.17E-02	2.48E-01	Exon (NM_053979/117050, exon 2 of 6)
Smim15	100359861	chr2	39121060	39121360	-193354	-1.41	2.18E-02	2.48E-01	Distal Intergenic
Ube4b	298652	chr5	166245858	166246158	12911	-1.53	2.18E-02	2.48E-01	Intron (NM_001271198/298652, intron 1 of 26)
Cflar	117279	chr9	65562537	65562837	27909	-1.24	2.18E-02	2.48E-01	Intron (NM_001033864/117279, intron 6 of 10)
Mccc1	294972	chr2	122641466	122641766	48774	1.5	2.18E-02	2.48E-01	Intron (NM_001009653/294972, intron 9 of 18)
Gas6	58935	chr16	81229669	81229969	13788	1.75	2.18E-02	2.48E-01	Intron (NM_057100/58935, intron 4 of 14)
Klhl25	293023	chr1	137260900	137261200	97872	-1.5	2.18E-02	2.48E-01	Intron (NM_001106271/293024, intron 17 of 38)
Napb	499903	chr3	143066455	143066755	-2551	1.86	2.18E-02	2.48E-01	Promoter (2-3kb)
Lrrfip1	367314	chr9	98190750	98191050	0	0.64	2.18E-02	2.48E-01	Promoter (<=1kb)
Sh2d3c	362111	chr3	11763641	11763941	7214	-1.58	2.18E-02	2.48E-01	Intron (NM_001108579/362111, intron 2 of 11)
Myo1d	25485	chr10	68125970	68126270	16594	-1.19	2.19E-02	2.48E-01	Intron (NM_012983/25485, intron 1 of 21)
Fbxl6	362941	chr7	117604590	117604890	64	0.66	2.19E-02	2.48E-01	Promoter (<=1kb)
Trim35	498538	chr15	42972073	42972373	11766	1.84	2.19E-02	2.48E-01	Intron (NM_001025142/498538, intron 3 of 5)
Trhr	25570	chr7	82829361	82829661	-284011	1.38	2.19E-02	2.49E-01	Distal Intergenic
Rhobtb3	309922	chr2	2679767	2680067	3238	1.96	2.19E-02	2.49E-01	Intron (NM_001107645/309922, intron 2 of 11)
Bzw2	171439	chr6	55636139	55636439	11211	-0.88	2.19E-02	2.49E-01	Intron (NM_134402/171439, intron 1 of 11)
Pxmp4	282634	chr3	150115132	150115432	-6239	0.65	2.19E-02	2.49E-01	Distal Intergenic
Irf5	296953	chr4	56805271	56805571	139	0.99	2.19E-02	2.49E-01	Promoter (<=1kb)
Frs2	314850	chr7	60210109	60210409	73	1.11	2.19E-02	2.49E-01	Promoter (<=1kb)
Zbtb24	365590	chr20	46136278	46136578	-31599	-1.71	2.19E-02	2.49E-01	Distal Intergenic
Pomt1	84430	chr3	11186709	11187009	-67017	-1.34	2.19E-02	2.49E-01	Distal Intergenic
Il6st	25205	chr2	44279201	44279501	-10574	1.02	2.19E-02	2.49E-01	Distal Intergenic
Nkiras1	305751	chr15	8163461	8163761	24831	1.62	2.20E-02	2.49E-01	Distal Intergenic
Prdm2	313678	chr5	161848314	161848614	30519	1.71	2.20E-02	2.49E-01	Intron (NM_001077648/313678, intron 2 of 9)
Islr	686539	chr8	62949054	62949354	-77	1.62	2.20E-02	2.49E-01	Promoter (<=1kb)
Prdm2	313678	chr5	161823130	161823430	55703	0.99	2.20E-02	2.49E-01	Exon (NM_001077648/313678, exon 6 of 10)
Jtb	29439	chr2	189591801	189592101	94	0.6	2.20E-02	2.49E-01	Promoter (<=1kb)
Tmem154	361972	chr2	183702689	183702989	28167	-1.51	2.20E-02	2.49E-01	Intron (NM_001108553/361972, intron 5 of 5)
Sipa1l1	246212	chr6	105953280	105953580	-98632	-1.32	2.20E-02	2.49E-01	Distal Intergenic
Phactr2	308291	chr1	7480453	7480753	36	1.14	2.20E-02	2.49E-01	Promoter (<=1kb)
Zfp563	314584	chr7	15413980	15414280	52	0.74	2.20E-02	2.49E-01	Promoter (<=1kb)
Mppe1	361344	chr18	62984907	62985207	-19369	1.32	2.20E-02	2.49E-01	Distal Intergenic
Ccdc63	304484	chr12	39913779	39914079	8659	1.53	2.20E-02	2.49E-01	Exon (NM_001025665/304484, exon 3 of 28)
Chsy1	292999	chr1	127060479	127060779	49865	-1.74	2.20E-02	2.49E-01	Intron (NM_001106268/292999, intron 2 of 2)
Mtpn	79215	chr4	63039077	63039377	45	0.6	2.20E-02	2.49E-01	Promoter (<=1kb)
Mtss1	362918	chr7	98902882	98903182	57554	-1.66	2.20E-02	2.49E-01	Intron (NM_001130563/362918, intron 4 of 15)
Odf1	24610	chr7	76954935	76955235	-111720	1.33	2.21E-02	2.49E-01	Distal Intergenic
Rhoq	85428	chr6	10533271	10533571	120	1.31	2.21E-02	2.49E-01	Promoter (<=1kb)
Idnk	498695	chr17	6909600	6909900	-17142	1	2.21E-02	2.49E-01	Distal Intergenic
Abcb10	361439	chr19	56772541	56772841	63	0.95	2.21E-02	2.49E-01	Promoter (<=1kb)
Arhgef12	367072	chr8	47393362	47393662	-320	1.19	2.21E-02	2.49E-01	Promoter (<=1kb)
Npepps	50558	chr10	85222419	85222719	142	1.25	2.21E-02	2.49E-01	Promoter (<=1kb)
Krr1	314830	chr7	54649294	54649594	-106449	-1.5	2.21E-02	2.49E-01	Distal Intergenic
Banp	292064	chr19	54766914	54767214	319	1.43	2.21E-02	2.49E-01	Promoter (<=1kb)
Mtfr1	311403	chr2	104006700	104007000	-13955	0.68	2.21E-02	2.49E-01	Distal Intergenic
Synpo	60324	chr18	55895891	55896191	-4181	-1.18	2.21E-02	2.49E-01	Distal Intergenic
Tgfb2	81809	chr13	105014986	105015286	125494	-1.64	2.21E-02	2.49E-01	Distal Intergenic
RGD1561870	502353	chr1	163957149	163957449	-142785	-1.51	2.21E-02	2.50E-01	Intron (NM_001107536/308846, intron 8 of 13)
Nfe2l2	83619	chr3	62554896	62555196	-29902	-1.36	2.21E-02	2.50E-01	Distal Intergenic
Triqk	500413	chr5	26493322	26493622	110	0.66	2.21E-02	2.50E-01	Promoter (<=1kb)
Ccnl1	114121	chr2	157854716	157855016	-94878	-1.36	2.22E-02	2.50E-01	Distal Intergenic
Isg20l2	361977	chr2	187302107	187302407	0	0.91	2.22E-02	2.50E-01	Promoter (<=1kb)
Fam181b	499205	chr1	157930480	157930780	9694	-1.56	2.22E-02	2.50E-01	Distal Intergenic
Zcchc24	361104	chr16	2019012	2019312	28980	1.71	2.22E-02	2.50E-01	Intron (NM_001108394/361104, intron 2 of 3)
Cd28	25660	chr9	67516264	67516564	-29844	-1.28	2.22E-02	2.50E-01	Distal Intergenic
Stn1	294025	chr1	267328445	267328745	-12857	-1.73	2.22E-02	2.50E-01	Distal Intergenic

Arhgap21	307178	chr17	87385058	87385358	464371	-1.09	2.22E-02	2.50E-01	Distal Intergenic
Fer	301737	chr9	111729297	111729597	79666	-1.63	2.22E-02	2.50E-01	Intron (NM_001106928/301737, intron 7 of 13)
Cald1	25687	chr4	62257880	62258180	36869	1.61	2.22E-02	2.50E-01	Exon (NM_013146/25687, exon 3 of 12)
Myo1d	25485	chr10	68104120	68104420	38444	-1.4	2.22E-02	2.50E-01	Intron (NM_012983/25485, intron 1 of 21)
Cib2	300719	chr8	59136078	59136378	3568	-1.63	2.22E-02	2.50E-01	Intron (NM_001015010/300719, intron 1 of 5)
Acox1	50681	chr10	104735690	104735990	12013	-0.85	2.22E-02	2.50E-01	Intron (NM_017340/50681, intron 3 of 13)
Ampd3	25095	chr1	175574591	175574891	-11206	-1.51	2.22E-02	2.50E-01	Distal Intergenic
Lamc2	192362	chr13	70575249	70575549	50703	-1.05	2.22E-02	2.50E-01	Exon (NM_001100640/192362, exon 19 of 24)
Zbtb39	299510	chr7	71036677	71036977	96	1.13	2.23E-02	2.50E-01	Promoter (<=1kb)
Soat1	81782	chr13	74051310	74051610	26149	-1.31	2.23E-02	2.50E-01	Intron (NM_031118/81782, intron 3 of 15)
Ankrd17	289521	chr14	19293506	19293806	-24759	1.16	2.23E-02	2.50E-01	Distal Intergenic
Prkd3	313834	chr6	1596085	1596385	18466	-1.38	2.23E-02	2.50E-01	Intron (NM_001024263/313834, intron 1 of 18)
Slc7a3	29485	chrX	70945840	70946140	31488	1.03	2.23E-02	2.50E-01	Distal Intergenic
Ift88	305918	chr15	37740531	37740831	49137	1.34	2.23E-02	2.50E-01	Intron (NM_001107266/305918, intron 15 of 25)
Yae1	306994	chr17	49627494	49627794	16946	-1.97	2.23E-02	2.50E-01	Distal Intergenic
Ccne1	25729	chr1	94500298	94500598	-5231	1.73	2.23E-02	2.50E-01	Distal Intergenic
Ppp3ca	24674	chr2	241913602	241913902	3770	-1.34	2.23E-02	2.50E-01	Intron (NM_017041/24674, intron 1 of 13)
Swap70	293410	chr1	174951912	174952212	89187	2.22	2.23E-02	2.50E-01	Intron (NM_001134970/691042, intron 38 of 39)
Gpr173	64021	chrX	22440111	22440411	-21238	0.81	2.23E-02	2.50E-01	Distal Intergenic
Tmcc3	314751	chr7	35472236	35472536	134	0.91	2.23E-02	2.50E-01	Promoter (<=1kb)
Tnrc18	304302	chr12	13832674	13832974	24855	-1.52	2.23E-02	2.51E-01	Intron (NM_001107123/304302, intron 2 of 34)
Mir6324	102466624	chr11	36416266	36416566	-32617	1.86	2.23E-02	2.51E-01	Distal Intergenic
Ndufv3	64539	chr20	10289365	10289665	23539	1.77	2.24E-02	2.51E-01	Distal Intergenic
Hpd	29531	chr12	38862015	38862315	33409	-1.31	2.24E-02	2.51E-01	Distal Intergenic
Pcdh18	295027	chr2	138826727	138827027	6838	-1.43	2.24E-02	2.51E-01	Intron (NM_001100524/295027, intron 3 of 3)
Sirt5	306840	chr17	24067860	24068160	81064	-1.27	2.24E-02	2.51E-01	Distal Intergenic
Rnf144b	364681	chr17	17928168	17928468	19309	1.24	2.24E-02	2.51E-01	Intron (NM_001108881/364681, intron 1 of 7)
Cep295nl	498028	chr10	107368285	107368585	8060	-1.32	2.24E-02	2.51E-01	Intron (NM_021989/29543, intron 1 of 4)
Nfx1	313166	chr5	57295059	57295359	3903	-1.42	2.25E-02	2.51E-01	Intron (NM_001024784/313166, intron 1 of 23)
Slc25a32	315023	chr7	78004644	78004944	31	0.67	2.25E-02	2.51E-01	Promoter (<=1kb)
Fa2h	307855	chr19	43570772	43571072	25716	-1.09	2.25E-02	2.52E-01	Intron (NM_001135583/307855, intron 1 of 6)
Chmp4c	361916	chr2	93623434	93623734	17763	1.33	2.25E-02	2.52E-01	Intron (NM_001017466/361916, intron 1 of 4)
Maf	54267	chr19	47882438	47882738	314010	1.5	2.25E-02	2.52E-01	Distal Intergenic
Vom2r47	365807	chr2	155281962	155282262	99577	1.47	2.25E-02	2.52E-01	Distal Intergenic
Ramp1	58965	chr9	98313158	98313458	-174	1.68	2.25E-02	2.52E-01	Promoter (<=1kb)
Tmem260	361030	chr15	25294407	25294707	0	1.21	2.25E-02	2.52E-01	Promoter (<=1kb)
Ube2v2	287927	chr11	89557307	89557607	34	0.74	2.25E-02	2.52E-01	Promoter (<=1kb)
Ccdc91	312863	chr4	181979373	181979673	104512	1.58	2.25E-02	2.52E-01	Intron (NM_001014061/312863, intron 7 of 12)
Nek7	360850	chr13	55602337	55602637	-40611	-1.62	2.25E-02	2.52E-01	Distal Intergenic
Egfr	24329	chr14	99944052	99944352	24567	-1.56	2.25E-02	2.52E-01	Intron (NM_031507/24329, intron 1 of 27)
Bicd2	306809	chr17	15717545	15717845	190	0.78	2.25E-02	2.52E-01	Promoter (<=1kb)
Col5a3	60379	chr8	21827652	21827952	3716	2.01	2.25E-02	2.52E-01	Intron (NM_021760/60379, intron 1 of 66)
Dnajb2	689593	chr9	82440668	82440968	4210	1.9	2.25E-02	2.52E-01	Exon (NM_001109541/689593, exon 5 of 9)
Inpp5j	171088	chr14	83741599	83741899	70	-1.48	2.25E-02	2.52E-01	Promoter (<=1kb)
Trip12	316575	chr9	92390141	92390441	44952	-1.53	2.25E-02	2.52E-01	Intron (NM_001031659/316575, intron 2 of 40)
Ifi27	170512	chr6	127327057	127327357	-602	-0.86	2.25E-02	2.52E-01	Promoter (<=1kb)
Snx11	303493	chr10	84642038	84642338	-2933	1.79	2.26E-02	2.52E-01	Promoter (2-3kb)
Tgfbr3	29610	chr14	3588397	3588697	81981	-1.77	2.26E-02	2.52E-01	Intron (NM_017256/29610, intron 3 of 16)
Rps6ka1	81771	chr5	152119971	152120271	2413	1.98	2.26E-02	2.52E-01	Promoter (2-3kb)
Nbl1	50594	chr5	157542781	157543081	-7080	1.98	2.26E-02	2.52E-01	Distal Intergenic
Flii	287375	chr10	46959646	46959946	9460	1.52	2.26E-02	2.52E-01	Exon (NM_001008279/287375, exon 18 of 30)
Zfp111	170849	chr1	81058105	81058405	-11171	0.99	2.26E-02	2.52E-01	Distal Intergenic
Dnaja2	84026	chr19	22575579	22575879	5580	-1.21	2.26E-02	2.52E-01	Intron (NM_032079/84026, intron 3 of 8)
Rtn4	83765	chr14	114064082	114064382	-62584	1.38	2.26E-02	2.52E-01	Distal Intergenic
Gpr176	117257	chr3	110109255	110109555	31201	1	2.26E-02	2.52E-01	Intron (NM_001270986/117257, intron 1 of 2)
Enpp3	54410	chr1	21614154	21614454	1006	1.46	2.26E-02	2.52E-01	Promoter (1-2kb)
Xpo6	293476	chr1	197415978	197416278	173999	1.86	2.26E-02	2.52E-01	Distal Intergenic
Tnfaip2	299339	chr6	135911884	135912184	20953	-1.34	2.26E-02	2.52E-01	Distal Intergenic
Asah2	114104	chr1	250737768	250738068	-107039	-1.62	2.26E-02	2.52E-01	Intron (NM_181386/353229, intron 5 of 9)
Cyb5b	80773	chr19	39410055	39410355	52241	-1.06	2.26E-02	2.52E-01	Distal Intergenic
Myom2	306616	chr16	79634398	79634698	37021	2.05	2.27E-02	2.52E-01	Intron (NM_001169141/306616, intron 10 of 36)
Setd5	297514	chr4	145047850	145048150	30242	-1.43	2.27E-02	2.52E-01	Intron (NM_001106614/297514, intron 1 of 23)
Xpot	314879	chr7	63728665	63728965	23	0.87	2.27E-02	2.52E-01	Promoter (<=1kb)
Cyp4v3	266761	chr16	50129249	50129549	17446	-1.64	2.27E-02	2.52E-01	Intron (NM_001135600/266761, intron 7 of 10)
Pmepa1	311676	chr3	171315823	171316123	-13325	-1.23	2.27E-02	2.52E-01	Distal Intergenic
Rhbdf1	303008	chr10	15672172	15672472	0	1.04	2.27E-02	2.52E-01	Promoter (<=1kb)
Eif5	108348073	chr6	136003228	136003528	170	1.13	2.27E-02	2.52E-01	Promoter (<=1kb)
Mrpl27	287635	chr10	82375631	82375931	59	0.73	2.27E-02	2.52E-01	Promoter (<=1kb)
Bckdha	25244	chr1	82451690	82451990	67	0.62	2.27E-02	2.52E-01	Promoter (<=1kb)
Psme1	29630	chr15	34259274	34259574	3203	-1.66	2.27E-02	2.52E-01	Exon (NM_001008296/290224, exon 3 of 5)
Derl1	362912	chr7	97759646	97759946	0	0.71	2.27E-02	2.52E-01	Promoter (<=1kb)
Rab3il1	171452	chr1	226066795	226067095	-10091	0.52	2.27E-02	2.52E-01	Intron (NM_031344/83512, intron 10 of 11)
Ttl	171572	chr3	121595486	121595786	-1032	1.78	2.27E-02	2.52E-01	Promoter (1-2kb)
Trak1	316085	chr8	129962659	129962959	-46614	-1.09	2.27E-02	2.52E-01	Distal Intergenic
Clec2g	362447	chr4	162955331	162955631	21136	-1.46	2.27E-02	2.52E-01	Distal Intergenic
Aspm	289054	chr13	56551138	56551438	5117	-1.61	2.27E-02	2.52E-01	Intron (NM_001105955/289054, intron 3 of 27)
Dhx15	289693	chr14	61142693	61142993	5947	-1.61	2.27E-02	2.52E-01	Intron (NM_001191597/289693, intron 2 of 13)
Fbxo8	306436	chr16	36986978	36987278	189755	-1.61	2.27E-02	2.52E-01	Distal Intergenic
Tnks	290794	chr16	60966505	60966805	41412	-1.61	2.27E-02	2.52E-01	Intron (NM_001106084/290794, intron 2 of 26)
Btn1a1	306956	chr17	43871397	43871697	-28988	-1.61	2.27E-02	2.52E-01	Distal Intergenic
Spred1	296072	chr3	108795161	108795461	0	1.09	2.28E-02	2.52E-01	Promoter (<=1kb)
Spry1	294981	chr2	124303994	124304294	-96395	-1.28	2.28E-02	2.52E-01	Intron (NM_001108549/361935, intron 14 of 14)
Afdn	26955	chr1	53904372	53904672	-63116	-1.56	2.28E-02	2.52E-01	Distal Intergenic
Slc28a3	140944	chr17	6448026	6448326	-10783	0.74	2.28E-02	2.52E-01	Distal Intergenic
Pcgf3	305624	chr14	2296720	2297020	135	0.91	2.28E-02	2.52E-01	Promoter (<=1kb)
Ssr3	81784	chr2	156020516	156020816	3037	-1.22	2.28E-02	2.52E-01	Intron (NM_031120/81784, intron 1 of 2)
Zfat	362925	chr7	109238446	109238746	-33077	1.75	2.28E-02	2.52E-01	Distal Intergenic
Kdm4c	298144	chr5	90800177	90800477	38	0.7	2.28E-02	2.52E-01	Promoter (<=1kb)
Lrp6	312781	chr4	168260099	168260399	36974	1.57	2.28E-02	2.52E-01	Exon (NM_001107892/312781, exon 6 of 20)
Krt8	25626	chr7	143597240	143597540	6205	1.78	2.28E-02	2.53E-01	Exon (NM_199370/25626, exon 8 of 9)
Chrdl2	308854	chr1	164977134	164977434	-31563	1.39	2.28E-02	2.53E-01	Distal Intergenic
Npr3	25339	chr2	61875758	61876058	73648	1.87	2.28E-02	2.53E-01	Distal Intergenic
Nid2	302248	chr15	4989917	4990217	-38024	-1.64	2.28E-02	2.53E-01	Distal Intergenic
Kctd4	691835	chr15	58021394	58021694	5156	-1.22	2.28E-02	2.53E-01	Intron (NM_031042/81674, intron 4 of 7)

Ccnt2	304758	chr13	44496663	44496963	20693	-1.55	2.29E-02	2.53E-01	Intron (NM_001107171/304758, intron 2 of 8)
Gpd1l	363159	chr8	122986753	122987053	183	0.6	2.29E-02	2.53E-01	Promoter (<=1kb)
Chp1	64152	chr3	111371663	111371963	17157	-1.36	2.29E-02	2.53E-01	Intron (NM_024139/64152, intron 3 of 6)
Kalrn	84009	chr11	69442677	69442977	-41316	-1.57	2.29E-02	2.53E-01	Distal Intergenic
Sft2d3	364835	chr18	24677742	24678042	15439	1.37	2.29E-02	2.53E-01	Intron (NM_001107398/307524, intron 17 of 21)
Rbm34	307956	chr19	59903619	59903919	-8682	-1.58	2.29E-02	2.53E-01	Distal Intergenic
Rbms2	288771	chr7	2573116	2573416	15270	-1.55	2.29E-02	2.53E-01	Intron (NM_001025403/288771, intron 1 of 11)
Stx11	292483	chr1	7046364	7046664	18206	-1.68	2.29E-02	2.53E-01	Intron (NM_001025638/292483, intron 1 of 1)
Dnajc22	362998	chr7	140781990	140782290	55	0.67	2.29E-02	2.53E-01	Promoter (<=1kb)
Pgrmc1	291948	chrX	123206025	123206325	156	0.85	2.29E-02	2.53E-01	Promoter (<=1kb)
Tspan5	362048	chr2	244119816	244120116	61630	0.87	2.29E-02	2.53E-01	Intron (NM_001004090/362048, intron 1 of 7)
Arap2	305367	chr14	48725306	48725606	-439	1.24	2.29E-02	2.53E-01	Promoter (<=1kb)
Ddx25	58856	chr8	36748184	36748484	12236	0.72	2.30E-02	2.53E-01	Exon (NM_031630/58856, exon 10 of 12)
Itgb1	24511	chr19	61695037	61695337	17495	-1.1	2.30E-02	2.53E-01	Intron (NM_017022/24511, intron 1 of 15)
Slc46a3	288454	chr12	8504541	8504841	-220695	1.05	2.30E-02	2.53E-01	Distal Intergenic
Bub1	296137	chr3	120399696	120399996	4914	1.78	2.30E-02	2.54E-01	Exon (NM_001106507/296137, exon 4 of 25)
Map4k4	301363	chr9	46684608	46684908	26686	-1.42	2.30E-02	2.54E-01	Intron (NM_001106904/301363, intron 1 of 28)
Rce1	309153	chr1	219867636	219867936	-3710	1.89	2.30E-02	2.54E-01	Distal Intergenic
Cxcl12	24772	chr4	149522822	149523122	261778	1.83	2.31E-02	2.54E-01	Distal Intergenic
Carf	301446	chr9	66878705	66879005	154	0.81	2.31E-02	2.54E-01	Promoter (<=1kb)
Fam120a	291019	chr17	16136844	16137144	30743	-1.19	2.31E-02	2.54E-01	Intron (NM_001191816/291019, intron 3 of 17)
Zdhhc3	301081	chr8	132191060	132191360	-1672	-1.24	2.31E-02	2.54E-01	Promoter (1-2kb)
Tp53inp1	297822	chr5	24409020	24409320	-1575	-1.32	2.31E-02	2.54E-01	Promoter (1-2kb)
Sox4	364712	chr17	37491216	37491516	-123506	-1.42	2.31E-02	2.54E-01	Distal Intergenic
Sdcbp2	311532	chr3	147064386	147064686	-8474	-1.43	2.31E-02	2.54E-01	Distal Intergenic
Eepd1	315500	chr8	26697616	26697916	45495	1.63	2.31E-02	2.54E-01	Intron (NM_001014088/315500, intron 1 of 6)
Tmem144	361968	chr2	178618514	178618814	-1795	1.89	2.31E-02	2.54E-01	Promoter (1-2kb)
Nr2c1	252924	chr7	35038456	35038756	-31051	-1.43	2.31E-02	2.54E-01	Intron (NM_001137645/500824, intron 8 of 20)
Zfpm2	314930	chr7	79809769	79810069	338492	-1.07	2.31E-02	2.54E-01	Intron (NM_001130501/314930, intron 4 of 6)
Abi1	79249	chr17	89987326	89987626	35574	-1.59	2.31E-02	2.54E-01	Intron (NM_024397/79249, intron 2 of 10)
Pla2g12a	362039	chr2	235311797	235312097	78	1.02	2.32E-02	2.54E-01	Promoter (<=1kb)
Tent5a	300870	chr8	93116687	93116987	-174462	1.88	2.32E-02	2.54E-01	Distal Intergenic
Rbm45	266631	chr3	63257723	63258023	37063	0.8	2.32E-02	2.54E-01	Distal Intergenic
Rab2a	65158	chr5	21584783	21585083	-47826	1.71	2.32E-02	2.54E-01	Distal Intergenic
Gmpr	117533	chr17	19576914	19577214	3715	-1.28	2.32E-02	2.54E-01	Intron (NM_057188/117533, intron 1 of 8)
Prr7	498704	chr17	9697028	9697328	-1736	1.46	2.32E-02	2.54E-01	Promoter (1-2kb)
Hmga2	84017	chr7	65211374	65211674	63734	-1.69	2.32E-02	2.54E-01	Intron (NM_032070/84017, intron 3 of 4)
Slc11a1	316519	chr9	81660978	81661278	5282	-1.72	2.32E-02	2.54E-01	Exon (NM_001031658/316519, exon 5 of 14)
Crispld2	171547	chr19	52615567	52615867	-48206	1.54	2.32E-02	2.54E-01	Distal Intergenic
Slc38a2	29642	chr7	138111621	138111921	-10798	0.6	2.32E-02	2.54E-01	Distal Intergenic
Pcnx1	314288	chr6	105822917	105823217	211461	1.3	2.32E-02	2.54E-01	Distal Intergenic
Atf1	315305	chr7	141766407	141766707	-115554	-0.73	2.32E-02	2.54E-01	Distal Intergenic
Atp11a	306600	chr16	82092900	82093200	7022	-1.39	2.32E-02	2.55E-01	Intron (NM_001107324/306600, intron 1 of 28)
Mirlet7a1	100314213	chr17	16409577	16409877	-7976	1.03	2.32E-02	2.55E-01	Distal Intergenic
Morn1	298676	chr5	172536806	172537106	47631	1.63	2.32E-02	2.55E-01	Intron (NM_001005544/298676, intron 12 of 13)
Zfp474	307310	chr18	47573300	47573600	-3966	1.93	2.33E-02	2.55E-01	Intron (NM_017061/24914, intron 1 of 8)
Zc3h15	362154	chr3	71024212	71024512	3678	-1.44	2.33E-02	2.55E-01	Intron (NM_001010963/362154, intron 1 of 9)
Snx19	315478	chr8	31495619	31495919	-1720	-1.63	2.33E-02	2.55E-01	Promoter (1-2kb)
Cldn20	680178	chr1	44362101	44362401	-84364	-0.96	2.33E-02	2.55E-01	Distal Intergenic
Uri1	308537	chr1	93691440	93691740	712471	-1.44	2.33E-02	2.55E-01	Distal Intergenic
Ppp3ca	24674	chr2	241923270	241923570	13438	-1.41	2.33E-02	2.55E-01	Intron (NM_017041/24674, intron 1 of 13)
Plin1	25629	chr1	141459458	141459758	11169	0.83	2.33E-02	2.55E-01	Exon (NM_001308145/25629, exon 9 of 9)
S1pr2	29415	chr8	21995041	21995341	465	0.57	2.33E-02	2.55E-01	Promoter (<=1kb)
Nelfb	311796	chr3	2430749	2431049	99	0.73	2.33E-02	2.55E-01	Promoter (<=1kb)
Src	83805	chr3	153548358	153548658	-32211	1.38	2.33E-02	2.55E-01	Distal Intergenic
Ptch1	89830	chr17	1085498	1085798	87	0.73	2.33E-02	2.55E-01	Promoter (<=1kb)
Ambp	25377	chr5	78861037	78861337	124589	-1.39	2.34E-02	2.55E-01	Distal Intergenic
Stk17b	170904	chr9	60402372	60402672	27409	-1.44	2.34E-02	2.55E-01	Intron (NM_133392/170904, intron 7 of 7)
Igf1r	25718	chr1	129143155	129143455	218234	1.7	2.34E-02	2.55E-01	Intron (NM_052807/25718, intron 3 of 20)
Srr	303306	chr10	61822969	61823269	-50676	-1.53	2.34E-02	2.55E-01	Exon (NM_001105808/287522, exon 9 of 19)
Irak2	362418	chr4	145614873	145615173	20298	-1.56	2.34E-02	2.55E-01	Intron (NM_001025422/362418, intron 2 of 12)
Uxt	299313	chrX	1275491	1275791	0	1.04	2.34E-02	2.55E-01	Promoter (<=1kb)
Zfp385a	685474	chr7	144949164	144949464	10282	1.4	2.34E-02	2.55E-01	Intron (NM_001135088/685474, intron 2 of 7)
Erp44	298066	chr5	64030563	64030863	0	0.62	2.34E-02	2.55E-01	Promoter (<=1kb)
Ssrp1	81785	chr3	72455803	72456103	7834	-1.55	2.34E-02	2.55E-01	Exon (NM_031121/81785, exon 13 of 16)
Coro6	245982	chr10	62683171	62683471	15070	-1.54	2.34E-02	2.55E-01	Distal Intergenic
B4galt6	65196	chr18	15412274	15412574	-50339	-1.41	2.34E-02	2.55E-01	Intron (NM_001101000/679221, intron 3 of 6)
Asns	25612	chr4	33760765	33761065	-49	0.65	2.34E-02	2.55E-01	Promoter (<=1kb)
Spatc1	315091	chr7	117323387	117323687	-2592	-1.48	2.34E-02	2.55E-01	Promoter (2-3kb)
Dlx2	296499	chr3	58273042	58273342	-91071	-1.23	2.34E-02	2.55E-01	Distal Intergenic
Bcar3	310838	chr2	226558143	226558443	-4607	1.17	2.34E-02	2.55E-01	Distal Intergenic
Dexi	497857	chr10	5199300	5199600	74	1.11	2.34E-02	2.55E-01	Promoter (<=1kb)
Vsnl1	24877	chr6	37145673	37145973	-23704	-1.03	2.34E-02	2.55E-01	Distal Intergenic
Hnrnpu	117280	chr13	96229333	96229633	8841	-1.66	2.34E-02	2.55E-01	Downstream (<1kb)
Ermp1	373544	chr1	247814952	247815252	6476	-1.34	2.34E-02	2.55E-01	Intron (NM_184050/373544, intron 2 of 14)
Ctsd	171293	chr1	215549521	215549821	3625	-1.34	2.34E-02	2.55E-01	Intron (NM_134334/171293, intron 2 of 8)
Mir21	100314000	chr10	73933404	73933704	-31103	-1.38	2.34E-02	2.55E-01	Intron (NM_138839/192129, intron 7 of 11)
Ccdc126	500117	chr4	78982019	78982319	0	0.64	2.35E-02	2.55E-01	Promoter (<=1kb)
Exoc2	171455	chr17	34640520	34640820	-24990	1.74	2.35E-02	2.55E-01	Distal Intergenic
Tgfb2	81809	chr13	105086551	105086851	53929	-1.43	2.35E-02	2.55E-01	Intron (NM_031131/81809, intron 2 of 7)
Cdk17	314743	chr7	34011179	34011479	9673	-1.13	2.35E-02	2.55E-01	Intron (NM_001108082/314743, intron 1 of 16)
Rbpj	679028	chr14	59879348	59879648	-143898	-0.85	2.35E-02	2.55E-01	Distal Intergenic
Ptprf	360406	chr5	137164649	137164949	-59656	1.93	2.35E-02	2.55E-01	Distal Intergenic
Fam20c	304334	chr12	17966730	17967030	5703	-1.53	2.35E-02	2.55E-01	Intron (NM_001012238/304334, intron 2 of 9)
Vamp3	29528	chr5	168441831	168442131	-305203	1.03	2.35E-02	2.55E-01	Intron (NM_001195559/362665, intron 5 of 28)
C1s	192262	chr4	157161284	157161584	-5688	-1.19	2.35E-02	2.55E-01	Distal Intergenic
Jph3	307916	chr19	54539518	54539818	-13601	-1.15	2.35E-02	2.55E-01	Distal Intergenic
Tcf3	171046	chr7	12144234	12144534	-2108	1.21	2.35E-02	2.55E-01	Promoter (2-3kb)
Defb36	641640	chr3	148091070	148091370	8280	1.27	2.35E-02	2.56E-01	Intron (NM_001037515/641640, intron 1 of 1)
Arhgap22	306279	chr16	9523656	9523956	-39262	-1.52	2.36E-02	2.56E-01	Distal Intergenic
Jam2	619374	chr11	24206344	24206644	-28666	1.15	2.36E-02	2.56E-01	Distal Intergenic
Eef1e1	291057	chr17	26479011	26479311	-305706	0.55	2.36E-02	2.56E-01	Distal Intergenic
Rab1a	81754	chr14	104493024	104493324	17942	-1.6	2.36E-02	2.56E-01	Intron (NM_031090/81754, intron 3 of 5)
Bmpr1a	81507	chr16	10831253	10831553	-28741	-1.6	2.36E-02	2.56E-01	Distal Intergenic

Hid1	287822	chr10	103911015	103911315	8330	0.79	2.36E-02	2.56E-01	Intron (NM_001304290/287822, intron 4 of 18)
Pcgf3	305624	chr14	2291920	2292220	4935	-1.35	2.36E-02	2.56E-01	Intron (NM_001107245/305624, intron 1 of 10)
Cadm3	360882	chr13	91925150	91925450	-52197	-1.53	2.36E-02	2.56E-01	Distal Intergenic
Eif4e2	363275	chr9	94319250	94319550	8774	-1.49	2.36E-02	2.56E-01	Intron (NM_001108808/363275, intron 4 of 6)
Nudt12	367323	chr9	106550978	106551278	-857621	-1.35	2.36E-02	2.56E-01	Distal Intergenic
Ddx6	500988	chr8	48911856	48912156	-13448	-1.41	2.36E-02	2.56E-01	Distal Intergenic
Bmper	300455	chr8	24315231	24315531	-54385	1.84	2.36E-02	2.56E-01	Distal Intergenic
LOC100361645	100361645	chr3	46196752	46197052	11392	0.77	2.36E-02	2.56E-01	5' UTR
Casq1	686019	chr13	90610616	90610916	-8251	1.51	2.36E-02	2.56E-01	Exon (NM_001271030/29132, exon 21 of 23)
Col5a2	85250	chr9	52202237	52202537	36198	-1.42	2.36E-02	2.56E-01	Intron (NM_053488/85250, intron 1 of 55)
Mir146a	100314241	chr10	28961881	28962181	389	-1.78	2.36E-02	2.56E-01	Promoter (<=1kb)
Zfp608	307296	chr18	49922485	49922785	14737	-1.78	2.36E-02	2.56E-01	Intron (NM_001107378/307296, intron 2 of 10)
Pappa1	313262	chr5	80395671	80395971	-524650	-1.78	2.36E-02	2.56E-01	Distal Intergenic
Igsf3	295325	chr2	203854413	203854713	85684	1.44	2.36E-02	2.56E-01	3' UTR
Tp53i11	311209	chr3	82150354	82150654	68457	2.04	2.36E-02	2.56E-01	Intron (NM_001107750/311210, intron 2 of 9)
Sec24d	310843	chr2	227464903	227465203	9180	1.88	2.37E-02	2.56E-01	Intron (NM_001309453/310843, intron 2 of 22)
Dmrt2	309430	chr1	244004253	244004553	341430	-1.41	2.37E-02	2.56E-01	Distal Intergenic
Map3k8	116596	chr17	56116392	56116692	6989	-1.31	2.37E-02	2.56E-01	Intron (NM_053847/116596, intron 2 of 7)
Gpc1	58920	chr9	99925642	99925942	-72333	1.73	2.37E-02	2.56E-01	Intron (NM_001009825/301618, intron 5 of 9)
Apobec3	315137	chr7	121121173	121121473	11923	-1.5	2.37E-02	2.56E-01	Intron (NM_001033703/315137, intron 4 of 7)
Nfkb1	81736	chr2	240921979	240922279	-31926	1.01	2.37E-02	2.56E-01	Distal Intergenic
Igsf21	298591	chr5	158887679	158887979	-151977	1.55	2.37E-02	2.56E-01	Distal Intergenic
Slc41a3	641603	chr4	123376171	123376471	118271	-1.47	2.37E-02	2.56E-01	Distal Intergenic
Csnk1g3	64823	chr18	49302908	49303208	449737	0.9	2.37E-02	2.56E-01	Distal Intergenic
Cyth3	116693	chr12	12934137	12934437	-26365	-1.53	2.37E-02	2.56E-01	Distal Intergenic
Septin11	305227	chr14	16405417	16405717	47173	-1.43	2.37E-02	2.56E-01	Exon (NM_001107208/305227, exon 2 of 11)
Slc39a10	363229	chr9	59986117	59986417	-35117	-1.07	2.37E-02	2.56E-01	Distal Intergenic
Selenoo	315216	chr7	130071301	130071601	179	0.76	2.37E-02	2.56E-01	Promoter (<=1kb)
Tbck	295446	chr2	237751685	237751985	31	0.73	2.37E-02	2.56E-01	Promoter (<=1kb)
Asb18	316614	chr9	97180573	97180873	-28741	1.37	2.37E-02	2.56E-01	Distal Intergenic
Phactr1	306844	chr17	23455409	23455709	209986	-1.04	2.37E-02	2.56E-01	Intron (NM_214457/306844, intron 3 of 13)
Pik3ap1	294048	chr1	260679135	260679435	-40319	1.51	2.37E-02	2.56E-01	Distal Intergenic
Pnrc1	286988	chr5	48504133	48504433	78	0.94	2.37E-02	2.56E-01	Promoter (<=1kb)
Slc2a12	308028	chr1	24047692	24047992	8381	-1.52	2.37E-02	2.56E-01	Intron (NM_001107451/308028, intron 1 of 4)
Cldn20	680178	chr1	44248966	44249266	-197499	1.73	2.37E-02	2.56E-01	Distal Intergenic
Jade2	303113	chr10	37277727	37278027	33598	1.66	2.38E-02	2.56E-01	Intron (NM_001106998/303113, intron 9 of 11)
Fis1	288584	chr12	22758145	22758445	6850	-1.6	2.38E-02	2.56E-01	Intron (NM_001105919/288584, intron 1 of 4)
Aak1	500244	chr4	118663006	118663306	7278	-1.18	2.38E-02	2.56E-01	Intron (NM_001173450/500244, intron 1 of 20)
Hmga1	117062	chr20	7124024	7124324	-11683	-1.46	2.38E-02	2.56E-01	Distal Intergenic
Bcar3	310838	chr2	226633190	226633490	70140	0.77	2.38E-02	2.56E-01	Intron (NM_001107722/310838, intron 3 of 11)
Cmss1	288176	chr11	45091584	45091884	59887	-1.37	2.38E-02	2.56E-01	Intron (NM_001013866/288176, intron 1 of 9)
Ctnnb1	84353	chr8	129601522	129601822	-16031	0.71	2.38E-02	2.56E-01	Distal Intergenic
Arhgef16	687105	chr5	171718550	171718850	-70049	2.14	2.38E-02	2.56E-01	Distal Intergenic
Tpx2	311546	chr3	148328037	148328337	72	0.65	2.38E-02	2.56E-01	Promoter (<=1kb)
Txnrd3	297437	chr4	121612353	121612653	21	0.89	2.38E-02	2.56E-01	Promoter (<=1kb)
Gsn	296654	chr3	14435278	14435578	-46810	-1.43	2.38E-02	2.56E-01	Distal Intergenic
Lhpp	361663	chr1	204640497	204640797	22645	1.8	2.38E-02	2.56E-01	Intron (NM_001009706/361663, intron 2 of 6)
RGD1562024	498699	chr17	8690088	8690388	-70351	1.88	2.38E-02	2.56E-01	Distal Intergenic
Sod2	24787	chr1	47928837	47929137	-7250	-1.34	2.38E-02	2.56E-01	Distal Intergenic
Tmem26	309724	chr20	21827559	21827859	-137904	-1.44	2.39E-02	2.56E-01	Intron (NM_001107624/309728, intron 4 of 11)
Pdp1	54705	chr5	25590831	25591131	-6553	1.05	2.39E-02	2.56E-01	Distal Intergenic
Golga4	501069	chr8	127177736	127178036	6100	1.42	2.39E-02	2.56E-01	Intron (NM_001106865/301056, intron 1 of 7)
Prnd	113910	chr3	124547292	124547592	1928	1.4	2.39E-02	2.56E-01	Promoter (1-2kb)
Mcfd2	246117	chr6	10892346	10892646	6537	-1.46	2.39E-02	2.56E-01	Intron (NM_139253/246117, intron 1 of 3)
Specc1	303208	chr10	48280807	48281107	40477	-1.4	2.39E-02	2.57E-01	Intron (NM_001039017/303208, intron 1 of 7)
Plk5	314627	chr7	12220785	12221085	245	1.56	2.39E-02	2.57E-01	Promoter (<=1kb)
Plaur	50692	chr1	81320021	81320321	-7850	-1.36	2.39E-02	2.57E-01	Distal Intergenic
Hunk	288275	chr11	30712280	30712580	162139	0.96	2.39E-02	2.57E-01	Distal Intergenic
Ext2	311215	chr3	82734178	82734478	55	1.34	2.39E-02	2.57E-01	Promoter (<=1kb)
Sdccag8	305002	chr13	94906404	94906704	18230	-1.42	2.39E-02	2.57E-01	Intron (NM_177929/305002, intron 5 of 17)
Ext1	299907	chr7	92675705	92676005	205387	-1.38	2.40E-02	2.57E-01	Intron (NM_001130540/299907, intron 1 of 10)
Map3k10	308463	chr1	84493681	84493981	-2202	-1.53	2.40E-02	2.57E-01	Promoter (2-3kb)
Adk	25368	chr15	3400245	3400545	35343	-1.53	2.40E-02	2.57E-01	Intron (NM_012895/25368, intron 1 of 10)
Dbx1	292934	chr1	104948309	104948609	25039	1.19	2.40E-02	2.57E-01	Distal Intergenic
Col1a2	84352	chr4	31756640	31756940	222415	0.68	2.40E-02	2.57E-01	Distal Intergenic
Dlx3	287638	chr10	82938525	82938825	554	1.09	2.40E-02	2.57E-01	Promoter (<=1kb)
Iqgap1	361598	chr1	142477269	142477569	138104	1.45	2.40E-02	2.57E-01	Distal Intergenic
Rnf126	314613	chr7	12820924	12821224	112	1.1	2.40E-02	2.57E-01	Promoter (<=1kb)
Marchf8	312656	chr4	148291986	148292286	5132	-1.8	2.40E-02	2.57E-01	Intron (NM_001107882/312656, intron 2 of 7)
H1f6	24438	chr17	43673453	43673753	2181	0.58	2.40E-02	2.57E-01	Promoter (2-3kb)
Sema3e	296789	chr4	17503483	17503783	90815	1.51	2.40E-02	2.57E-01	Intron (NM_001106579/296789, intron 1 of 16)
Senp17	408216	chr4	161029420	161029720	61290	1.97	2.40E-02	2.57E-01	Intron (NM_001002833/408216, intron 1 of 1)
Slco6d1	367321	chr9	104966712	104967012	-96478	-1.23	2.40E-02	2.57E-01	Distal Intergenic
Hspa5	25617	chr3	13716027	13716327	-121977	1.03	2.40E-02	2.57E-01	Distal Intergenic
Vps54	286932	chr14	106130186	106130486	-23091	1.27	2.40E-02	2.57E-01	Distal Intergenic
Ube2v1	296390	chr3	164371288	164371588	-4604	1.64	2.41E-02	2.57E-01	Distal Intergenic
Rock1	81762	chr18	1379012	1379312	10726	-1.61	2.41E-02	2.57E-01	Intron (NM_031098/81762, intron 1 of 34)
Ttc12	300696	chr8	53971268	53971568	-154815	-1.52	2.41E-02	2.57E-01	Intron (NM_031521/24586, intron 1 of 18)
Crppa	493574	chr6	56189701	56190001	308217	0.87	2.41E-02	2.57E-01	Distal Intergenic
Tcf4	84382	chr18	65490595	65490895	205275	-1.31	2.41E-02	2.57E-01	Intron (NM_053369/84382, intron 12 of 16)
Arntl	29657	chr1	178087925	178088225	48862	-1.59	2.41E-02	2.57E-01	Intron (NM_024362/29657, intron 3 of 20)
Pias1	300772	chr8	67814080	67814380	54635	-1.59	2.41E-02	2.57E-01	Intron (NM_001106829/300772, intron 2 of 13)
Mir29b1	100314008	chr4	58482065	58482365	-137675	-1.41	2.41E-02	2.57E-01	Distal Intergenic
Relt	361615	chr1	165898372	165898672	-6745	2.16	2.41E-02	2.57E-01	Distal Intergenic
Lrrc43	288751	chr12	38609744	38610044	-85029	-1.14	2.41E-02	2.57E-01	Distal Intergenic
Tbc1d2b	315880	chr8	97633742	97634042	13030	-1.36	2.41E-02	2.57E-01	Intron (NM_001108175/315880, intron 2 of 13)
Magi1	500261	chr4	126214362	126214662	84748	-0.81	2.41E-02	2.57E-01	Intron (NM_001030045/500261, intron 1 of 22)
Il1r2	117022	chr9	46905852	46906152	64828	-1.49	2.41E-02	2.57E-01	Distal Intergenic
Serp2	498546	chr15	58863953	58864253	-152081	1.88	2.41E-02	2.57E-01	Distal Intergenic
Upk1a	365227	chr1	89067133	89067433	915	1.85	2.41E-02	2.57E-01	Promoter (<=1kb)
Scarf2	287949	chr11	87722825	87723125	475	0.69	2.42E-02	2.58E-01	Promoter (<=1kb)
Nudt4	94267	chr7	36769621	36769921	-109480	-1.27	2.42E-02	2.58E-01	Distal Intergenic
Fgf5	60662	chr14	13188123	13188423	-192539	-1.18	2.42E-02	2.58E-01	Distal Intergenic
Fam160a1	365834	chr2	185022256	185022556	-28906	1.78	2.42E-02	2.58E-01	Distal Intergenic

Ccnt2	304758	chr13	44482174	44482474	6204	-1.37	2.42E-02	2.58E-01	Intron (NM_001107171/304758, intron 2 of 8)
Nr2c2	50659	chr4	123983118	123983418	-21869	-1.63	2.42E-02	2.58E-01	Distal Intergenic
Dusp23	360881	chr13	91019628	91019928	0	0.92	2.42E-02	2.58E-01	Promoter (<=1kb)
Bend7	361275	chr17	77510904	77511204	16684	-1.86	2.42E-02	2.58E-01	Intron (NM_001191834/361275, intron 1 of 7)
Ndufaf8	690871	chr10	109330048	109330348	51336	1.82	2.42E-02	2.58E-01	Distal Intergenic
Sema5a	310207	chr2	85437469	85437769	60151	-1.15	2.42E-02	2.58E-01	Intron (NM_001107659/310207, intron 1 of 22)
Smarcad1	312398	chr4	95916220	95916520	31477	-1.58	2.42E-02	2.58E-01	Intron (NM_001107864/312398, intron 8 of 22)
Slc39a10	363229	chr9	60201545	60201845	180011	-1.58	2.42E-02	2.58E-01	Distal Intergenic
Acrbp	500316	chr4	157605182	157605482	41192	2.17	2.42E-02	2.58E-01	Distal Intergenic
RGD1311899	288704	chr12	47438651	47438951	0	0.59	2.43E-02	2.58E-01	Promoter (<=1kb)
Ripk4	304053	chr11	38275936	38276236	-1702	-1.27	2.43E-02	2.58E-01	Promoter (1-2kb)
Cilp	315761	chr8	70745537	70745837	-15085	1.88	2.43E-02	2.58E-01	Distal Intergenic
Srek1	56763	chr2	33972803	33973103	-143882	1.89	2.43E-02	2.58E-01	Distal Intergenic
Tmod3	300838	chr8	82447693	82447993	44250	-1.31	2.43E-02	2.58E-01	Intron (NM_001011997/300838, intron 3 of 9)
Frmd4a	307128	chr17	77836447	77836747	81463	-1.54	2.43E-02	2.58E-01	Intron (NM_001191821/307128, intron 1 of 21)
Olr1286	405084	chr15	35765243	35765543	3855	1.57	2.43E-02	2.58E-01	Distal Intergenic
Fhl1	25177	chrX	159159376	159159676	1182	1.46	2.43E-02	2.58E-01	Promoter (1-2kb)
Spc25	295661	chr3	55457530	55457830	-5732	1.43	2.43E-02	2.58E-01	Distal Intergenic
Nsd2	680537	chr14	82169160	82169460	2020	-1.61	2.43E-02	2.58E-01	Promoter (2-3kb)
Ing3	312154	chr4	49059170	49059470	41859	-1.46	2.43E-02	2.58E-01	Distal Intergenic
Lrrc75b	100365744	chr20	14059417	14059717	8952	-1.13	2.43E-02	2.58E-01	Distal Intergenic
Ank1	306570	chr16	73848762	73849062	-21274	-1.31	2.43E-02	2.58E-01	Distal Intergenic
Fam241a	691931	chr2	232263962	232264262	-18643	-1.2	2.44E-02	2.58E-01	Distal Intergenic
Sec14l2	116486	chr14	84359858	84360158	-4330	1.04	2.44E-02	2.58E-01	Distal Intergenic
Diaph3	290396	chr15	70359096	70359396	40592	-1.34	2.44E-02	2.58E-01	Intron (NM_001305172/290396, intron 2 of 27)
Afap1	140935	chr14	79984668	79984968	2947	-1.5	2.44E-02	2.58E-01	Promoter (2-3kb)
Fam174a	301634	chr9	102862859	102863159	0	1.07	2.44E-02	2.58E-01	Promoter (<=1kb)
Limk2	29524	chr14	83662928	83663228	-21036	1.53	2.44E-02	2.58E-01	Intron (NM_001024271/360967, intron 2 of 6)
Adamts9	312566	chr4	124977664	124977964	-119414	1.7	2.44E-02	2.58E-01	Distal Intergenic
Gnaq	81666	chr1	233503652	233503952	120874	-1.39	2.44E-02	2.59E-01	Intron (NM_031036/81666, intron 2 of 6)
Ttc17	311224	chr3	83306256	83306556	34	0.72	2.44E-02	2.59E-01	Promoter (<=1kb)
Ubash3b	315579	chr8	45325770	45326070	49064	-1.31	2.44E-02	2.59E-01	Intron (NM_001191792/315579, intron 1 of 13)
Prr11	360591	chr10	74409020	74409320	4102	1.97	2.44E-02	2.59E-01	Intron (NM_001108287/360591, intron 2 of 9)
Abhd2	293050	chr1	141031582	141031882	33342	-1.55	2.45E-02	2.59E-01	Intron (NM_001106275/293050, intron 3 of 10)
Jak1	84598	chr5	120061705	120062005	21899	-1.56	2.45E-02	2.59E-01	Intron (NM_053466/84598, intron 1 of 24)
Kiss1r	78976	chr7	12608989	12609289	579	1.38	2.45E-02	2.59E-01	Promoter (<=1kb)
Capza2	493810	chr4	44940055	44940355	3765	-1.26	2.45E-02	2.59E-01	Intron (NM_001009180/493810, intron 1 of 9)
Nemp2	503257	chr9	53732616	53732916	0	0.67	2.45E-02	2.59E-01	Promoter (<=1kb)
Dnajb9	24908	chr6	64224868	64225168	-54746	0.89	2.45E-02	2.59E-01	Distal Intergenic
Slc48a1	300191	chr7	139271713	139272013	15	0.8	2.45E-02	2.59E-01	Promoter (<=1kb)
Ciart	365871	chr2	197991299	197991599	-101	0.83	2.45E-02	2.59E-01	Promoter (<=1kb)
Cp	24268	chr2	104679355	104679655	-64788	0.72	2.45E-02	2.59E-01	Distal Intergenic
Kdm3a	312440	chr4	99546816	99547116	-358	0.66	2.45E-02	2.59E-01	Promoter (<=1kb)
Naa25	360811	chr12	40680011	40680311	63	1.06	2.46E-02	2.59E-01	Promoter (<=1kb)
Idnk	498695	chr17	6924688	6924988	-32230	0.63	2.46E-02	2.59E-01	Distal Intergenic
Ube2h	296956	chr4	57624990	57625290	0	1.09	2.46E-02	2.59E-01	Promoter (<=1kb)
Runx2	367218	chr9	18659163	18659463	15593	-1.11	2.46E-02	2.60E-01	Intron (NM_001278483/367218, intron 4 of 8)
Pmp2	688790	chr2	93844913	93845213	17409	1.16	2.46E-02	2.60E-01	Distal Intergenic
Atn1	29515	chr9	20264261	20264561	0	2.04	2.46E-02	2.60E-01	Promoter (<=1kb)
Cdc42se2	691031	chr10	40068020	40068320	54595	-1.47	2.46E-02	2.60E-01	Intron (NM_001126089/691031, intron 3 of 5)
Nrg1	112400	chr16	63552098	63552398	-328030	-1.46	2.46E-02	2.60E-01	Intron (NM_001271130/112400, intron 1 of 12)
RGD1311251	315665	chr8	57978573	57978873	-4694	-1.36	2.46E-02	2.60E-01	Exon (NM_001025123/315664, exon 7 of 8)
Gpc1	58920	chr9	99998509	99998809	234	0.98	2.46E-02	2.60E-01	Promoter (<=1kb)
Frmd6	257646	chr6	93296367	93296667	14959	1.8	2.47E-02	2.60E-01	Intron (NM_001271054/257646, intron 1 of 13)
Csk	315707	chr8	62424348	62424648	-14010	1.09	2.47E-02	2.60E-01	Distal Intergenic
Impact	497198	chr18	4326125	4326425	253	0.64	2.47E-02	2.60E-01	Promoter (<=1kb)
Cxxc5	291670	chr18	28665121	28665421	11468	1.31	2.47E-02	2.60E-01	Distal Intergenic
Gata2	25159	chr4	120124774	120125074	-8639	1.57	2.47E-02	2.60E-01	Distal Intergenic
Zc3h15	362154	chr3	69872373	69872673	-1147861	-1.56	2.47E-02	2.60E-01	Distal Intergenic
Zfp111	170849	chr1	81046713	81047013	0	0.69	2.47E-02	2.60E-01	Promoter (<=1kb)
Nfya	29508	chr9	14551991	14552291	233	0.8	2.47E-02	2.60E-01	Promoter (<=1kb)
Fmr1	24948	chrX	154721793	154722093	221	0.95	2.47E-02	2.60E-01	Promoter (<=1kb)
Tenm3	306451	chr16	46057087	46057387	-366482	-1.53	2.47E-02	2.60E-01	Distal Intergenic
Slc35e1	498599	chr16	19042183	19042483	-9838	-1.62	2.47E-02	2.60E-01	Intron (NM_001107300/306328, intron 1 of 2)
Emb	114511	chr2	49457209	49457509	-225254	1.42	2.47E-02	2.60E-01	Distal Intergenic
Syt13	80977	chr3	81814243	81814543	0	0.91	2.47E-02	2.60E-01	Promoter (<=1kb)
Fbxl14	312675	chr4	151961125	151961425	-25468	-1.47	2.47E-02	2.60E-01	Distal Intergenic
B9d2	308443	chr1	82473748	82474048	-58	0.93	2.47E-02	2.60E-01	Promoter (<=1kb)
Caprin1	362173	chr3	93728333	93728633	5649	-1.22	2.48E-02	2.60E-01	Intron (NM_001012185/362173, intron 2 of 18)
Pax3	114502	chr9	83704647	83704947	396225	-1.51	2.48E-02	2.60E-01	Distal Intergenic
Prss22	302971	chr10	13384589	13384889	-9781	-1.28	2.48E-02	2.60E-01	Distal Intergenic
Per3	78962	chr5	168120673	168120973	2057	-1.46	2.48E-02	2.60E-01	Promoter (2-3kb)
Pcolce	29569	chr12	22154377	22154677	382	0.59	2.48E-02	2.60E-01	Promoter (<=1kb)
Vamp3	29528	chr5	168434704	168435004	-298076	0.99	2.48E-02	2.60E-01	Intron (NM_001195559/362665, intron 5 of 28)
Sipa1l1	246212	chr6	105886443	105886743	-165469	0.62	2.48E-02	2.60E-01	Distal Intergenic
Mtmr7	306490	chr16	54802005	54802305	36680	-1.6	2.48E-02	2.60E-01	Intron (NM_001107312/306490, intron 2 of 13)
Thap12	308845	chr1	163620905	163621205	-42202	1.3	2.48E-02	2.60E-01	Distal Intergenic
Alox15	81639	chr10	56983012	56983312	-20872	-1.42	2.48E-02	2.60E-01	Distal Intergenic
Pik3cb	85243	chr8	107380509	107380809	-32936	1.09	2.48E-02	2.60E-01	Distal Intergenic
Hgfac	58947	chr14	80998932	80999232	-25476	2.03	2.48E-02	2.60E-01	Intron (NM_019339/54292, intron 3 of 15)
Mtmr12	310155	chr2	62255069	62255369	18479	1.7	2.48E-02	2.60E-01	Intron (NM_001012077/310155, intron 1 of 14)
Pianp	312711	chr4	157511542	157511842	0	1.17	2.48E-02	2.60E-01	Promoter (<=1kb)
Glg1	29476	chr19	42984102	42984402	223	0.66	2.49E-02	2.60E-01	Promoter (<=1kb)
Mir3565	100526634	chr14	66771331	66771631	-151452	0.66	2.49E-02	2.60E-01	Distal Intergenic
Rybp	312603	chr4	132688120	132688420	52403	1.23	2.49E-02	2.60E-01	Downstream (1-2kb)
Cilp	315761	chr8	70765509	70765809	4587	0.84	2.49E-02	2.60E-01	Exon (NM_001108161/315761, exon 4 of 11)
Pten	50557	chr1	251421169	251421469	-345	1.06	2.49E-02	2.60E-01	Promoter (<=1kb)
Plekhg5	310999	chr5	169240384	169240684	-4094	0.82	2.49E-02	2.60E-01	Distal Intergenic
Uchl1	29545	chr14	43332988	43333288	-189046	1.76	2.49E-02	2.60E-01	Distal Intergenic
Fem1b	315745	chr8	67714495	67714795	-6182	-1.31	2.49E-02	2.60E-01	Distal Intergenic
Fnip2	310538	chr2	178287893	178288193	46707	-1.52	2.49E-02	2.60E-01	Intron (NM_001271167/310538, intron 1 of 16)
Fos	314322	chr6	109281888	109282188	-18245	0.69	2.49E-02	2.60E-01	Distal Intergenic
Itgb1bp1	298914	chr6	43253502	43253802	109533	1.31	2.49E-02	2.60E-01	Distal Intergenic
Arhgap30	498282	chr13	89765571	89765871	-8893	0.95	2.49E-02	2.60E-01	Exon (NM_001109076/498281, exon 2 of 9)

Gne	114711	chr5	59565992	59566292	-22891	-1.24	2.49E-02	2.60E-01	Distal Intergenic
Enah	360891	chr13	100375221	100375521	29818	-1.57	2.49E-02	2.60E-01	Intron (NM_001012150/360891, intron 1 of 12)
Gulp1	314543	chr9	51263618	51263918	0	0.63	2.49E-02	2.60E-01	Promoter (<=1kb)
Acaca	60581	chr10	71503364	71503664	-15728	1.03	2.49E-02	2.60E-01	Distal Intergenic
Col27a1	298101	chr5	79055494	79055794	0	1.13	2.49E-02	2.60E-01	Promoter (<=1kb)
Acad11	315973	chr8	112650993	112651293	56235	-1.37	2.49E-02	2.61E-01	Intron (NM_001108181/315973, intron 13 of 19)
Tmem170b	361230	chr17	22960960	22961260	-96994	0.99	2.49E-02	2.61E-01	Distal Intergenic
Jun	24516	chr5	114110241	114110541	-95964	0.7	2.50E-02	2.61E-01	Distal Intergenic
LOC499643	499643	chr2	178056853	178057153	-60330	0.8	2.50E-02	2.61E-01	Distal Intergenic
Rfk	499328	chr1	236465220	236465520	36213	1.86	2.50E-02	2.61E-01	Distal Intergenic
Pkp2	287925	chr11	88964236	88964536	7677	-1.45	2.50E-02	2.61E-01	Intron (NM_001100499/287925, intron 1 of 10)
Itgb5	257645	chr11	70269972	70270272	-97808	1.91	2.50E-02	2.61E-01	Distal Intergenic
Spryd3	315327	chr7	143731955	143732255	5982	1.96	2.50E-02	2.61E-01	Intron (NM_001191790/315327, intron 5 of 10)
Ndufv2	81728	chr9	113899762	113900062	107	0.66	2.50E-02	2.61E-01	Promoter (<=1kb)
Epha4	316539	chr9	83252976	83253276	182	0.6	2.50E-02	2.61E-01	Promoter (<=1kb)
Dusp7	300980	chr8	115029704	115030004	-39091	0.91	2.50E-02	2.61E-01	Intron (NM_001109296/501048, intron 8 of 12)
Morn1	298676	chr5	172552747	172553047	63572	1.32	2.50E-02	2.61E-01	Distal Intergenic
Rhobtb3	309922	chr2	2653897	2654197	29108	1.89	2.50E-02	2.61E-01	Intron (NM_001107645/309922, intron 6 of 11)
Cald1	25687	chr4	62183159	62183459	-37552	1.44	2.50E-02	2.61E-01	Distal Intergenic
Ppil2	360746	chr11	88151271	88151571	3894	2.06	2.50E-02	2.61E-01	Intron (NM_001017383/360746, intron 3 of 19)
Id4	291023	chr17	16746762	16747062	-51636	0.95	2.50E-02	2.61E-01	Distal Intergenic
Arhgap22	306279	chr16	9503428	9503728	-59490	-1.6	2.50E-02	2.61E-01	Distal Intergenic
Rnf130	652955	chr10	35510301	35510601	-27375	-1.32	2.50E-02	2.61E-01	Distal Intergenic
Pde8a	308776	chr1	143036178	143036478	0	0.95	2.50E-02	2.61E-01	Promoter (<=1kb)
Rap1gap2	303298	chr10	61275004	61275304	-42673	-1.68	2.50E-02	2.61E-01	Distal Intergenic
Bbs4	300754	chr8	64154028	64154328	68	0.83	2.50E-02	2.61E-01	Promoter (<=1kb)
Slk	54308	chr1	267371307	267371607	11500	1.84	2.50E-02	2.61E-01	Intron (NM_019349/54308, intron 1 of 17)
Siah1	140941	chr19	21372393	21372693	230	0.95	2.50E-02	2.61E-01	Promoter (<=1kb)
Clec2g	362447	chr4	162956382	162956682	22187	-1.25	2.51E-02	2.61E-01	Distal Intergenic
Plcxd2	363781	chr11	57207843	57208143	187	-1.68	2.51E-02	2.61E-01	Promoter (<=1kb)
Akt1	24185	chr6	137238704	137239004	-2503	0.99	2.51E-02	2.61E-01	Promoter (2-3kb)
Lamc2	192362	chr13	70570515	70570815	55437	-1.5	2.51E-02	2.61E-01	Intron (NM_001100640/192362, intron 23 of 23)
Fbln5	29158	chr6	125699002	125699302	24655	1.85	2.51E-02	2.61E-01	Intron (NM_019153/29158, intron 4 of 10)
Mir3120	100526611	chr13	80254594	80254894	-123569	-1.29	2.51E-02	2.61E-01	Intron (NM_138538/171574, intron 2 of 20)
Nfu1	297416	chr4	118814423	118814723	139	0.6	2.51E-02	2.61E-01	Promoter (<=1kb)
Atp2a2	29693	chr12	39561766	39562066	7863	-1.77	2.51E-02	2.61E-01	Intron (NM_001110823/29693, intron 3 of 19)
Wdr26	498301	chr13	99534819	99535119	-2860	0.62	2.51E-02	2.61E-01	Promoter (2-3kb)
Oser1	296346	chr3	159802648	159802948	0	0.73	2.51E-02	2.61E-01	Promoter (<=1kb)
Fam110b	500400	chr5	18835862	18836162	-64877	1.86	2.52E-02	2.62E-01	Distal Intergenic
Padi6	298595	chr5	159140469	159140769	115930	1.14	2.52E-02	2.62E-01	Distal Intergenic
Hivep1	117140	chr17	22356055	22356355	47007	-1.5	2.52E-02	2.62E-01	Intron (NM_001105751/117140, intron 2 of 9)
Klhdc10	312199	chr4	57716012	57716312	0	1.07	2.52E-02	2.62E-01	Promoter (<=1kb)
Ptpn5	29644	chr1	103235258	103235558	-23228	-1.14	2.52E-02	2.62E-01	Distal Intergenic
Tgfb2	81809	chr13	104735633	104735933	404847	-1.36	2.52E-02	2.62E-01	Distal Intergenic
Fgf5	60662	chr14	13055353	13055653	-59769	1.02	2.53E-02	2.62E-01	Distal Intergenic
Eif4e	117045	chr2	243820920	243821220	224	1.69	2.53E-02	2.62E-01	Promoter (<=1kb)
Dusp6	116663	chr7	41621135	41621435	145972	-1.46	2.53E-02	2.62E-01	Distal Intergenic
Cachd1	298267	chr5	119655954	119656254	59228	-1.44	2.53E-02	2.62E-01	Intron (NM_001191758/298267, intron 1 of 25)
Map4k4	301363	chr9	46692967	46693267	35045	-1.44	2.53E-02	2.62E-01	Intron (NM_001106904/301363, intron 1 of 28)
Wnt4	84426	chr5	155646878	155647178	-2060	1.78	2.53E-02	2.62E-01	Promoter (2-3kb)
Ehd4	192204	chr3	111903028	111903328	38513	-1.26	2.53E-02	2.62E-01	Intron (NM_139324/192204, intron 3 of 5)
Idi1	89784	chr17	58167028	58167328	-182992	1.41	2.53E-02	2.62E-01	Intron (NM_133302/117088, intron 4 of 9)
Tgfb2	81809	chr13	105005520	105005820	134960	1.93	2.53E-02	2.62E-01	Distal Intergenic
Rybp	312603	chr4	132740612	132740912	0	0.65	2.53E-02	2.62E-01	Promoter (<=1kb)
Mef2a	309957	chr1	128459222	128459522	-117959	2.09	2.53E-02	2.62E-01	Distal Intergenic
Actr8	361107	chr16	6048118	6048418	98	0.58	2.53E-02	2.62E-01	Promoter (<=1kb)
Dctn4	84428	chr18	55797383	55797683	180	0.68	2.53E-02	2.62E-01	Promoter (<=1kb)
Mttp	310900	chr2	243325066	243325366	82242	-0.77	2.53E-02	2.62E-01	Distal Intergenic
Rexo1	314630	chr7	12044662	12044962	22394	0.76	2.53E-02	2.62E-01	Distal Intergenic
Kbtbd2	312372	chr4	87110833	87111133	0	1.51	2.53E-02	2.62E-01	Promoter (<=1kb)
Tmbim6	24822	chr7	141032880	141033180	-20696	0.76	2.53E-02	2.62E-01	Distal Intergenic
Septin9	83788	chr10	106275512	106275812	-31221	-1.09	2.54E-02	2.62E-01	Intron (NM_176856/83788, intron 3 of 7)
Pex26	297570	chr4	153704892	153705192	-43151	1.99	2.54E-02	2.62E-01	Distal Intergenic
RGD735065	294311	chr20	6868127	6868427	-1446	-1.74	2.54E-02	2.62E-01	Promoter (1-2kb)
Dnmt3a	444984	chr6	28107214	28107514	-128181	-1.28	2.54E-02	2.62E-01	Intron (NM_001012191/362715, intron 9 of 18)
Ltc4s	114097	chr10	35768131	35768431	-28512	1.54	2.54E-02	2.62E-01	Exon (NM_001106997/303101, exon 5 of 5)
Vezf1	287615	chr10	75366071	75366371	260	1.2	2.54E-02	2.62E-01	Promoter (<=1kb)
Gstcd	310855	chr2	238068439	238068739	184548	0.78	2.54E-02	2.62E-01	Distal Intergenic
Ttc4	362556	chr5	126282001	126282301	29	0.71	2.54E-02	2.62E-01	Promoter (<=1kb)
Ankrd37	361149	chr16	49467027	49467327	4011	-1.69	2.54E-02	2.62E-01	Exon (NM_001014142/361151, exon 11 of 12)
Pgr	25154	chr8	7304172	7304472	175516	-1.46	2.54E-02	2.62E-01	Distal Intergenic
Kmt5c	308345	chr1	72673937	72674237	34	0.73	2.54E-02	2.62E-01	Promoter (<=1kb)
Rab2a	65158	chr5	21632878	21633178	0	1	2.54E-02	2.62E-01	Promoter (<=1kb)
Dnaaf4	363096	chr8	79638625	79638925	0	0.76	2.54E-02	2.62E-01	Promoter (<=1kb)
Kcmf1	684322	chr4	100761767	100762067	20950	-1.36	2.54E-02	2.62E-01	Intron (NM_001128192/684322, intron 5 of 5)
Hand1	59112	chr10	43368926	43369226	-115630	-1.5	2.55E-02	2.62E-01	Distal Intergenic
Ubtd1	309373	chr1	261229743	261230043	396	0.75	2.55E-02	2.62E-01	Promoter (<=1kb)
Traf1	687813	chr3	14169797	14170097	-150593	1.32	2.55E-02	2.62E-01	Distal Intergenic
Cabcoco1	361834	chr20	21152626	21152926	-163900	1.61	2.55E-02	2.62E-01	Distal Intergenic
Zfp362	297879	chr5	146988954	146989254	-15022	-1.46	2.55E-02	2.62E-01	Distal Intergenic
Rab10	50993	chr6	27687012	27687312	33808	-1.32	2.55E-02	2.62E-01	Intron (NM_017359/50993, intron 1 of 5)
Kcnb1	25736	chr3	163946310	163946610	-10700	1.69	2.55E-02	2.62E-01	Distal Intergenic
Selplg	363930	chr12	48551129	48551429	-26476	2.01	2.55E-02	2.62E-01	Exon (NM_001109327/501841, exon 9 of 10)
Parp16	315760	chr8	70720088	70720388	7308	2.01	2.55E-02	2.62E-01	Exon (NM_001014093/315760, exon 3 of 7)
Rps27a	100912032	chr14	113961493	113961793	6591	-1.52	2.55E-02	2.62E-01	Exon (NM_001004254/305606, exon 11 of 15)
Naa20	362228	chr3	140070079	140070379	-36347	-1.52	2.55E-02	2.62E-01	Intron (NM_001107786/311494, intron 9 of 12)
Hopx	171160	chr14	33371152	33371452	16345	-1.55	2.55E-02	2.62E-01	Distal Intergenic
Casp6	83584	chr2	230242864	230243164	56650	-1.23	2.55E-02	2.62E-01	Intron (NM_001047887/311013, intron 1 of 1)
RGD1562146	500612	chr6	9649065	9649365	110803	1.51	2.55E-02	2.62E-01	Intron (NM_017171/29340, intron 1 of 13)
Psip1	313323	chr5	102157353	102157653	-569255	-1.47	2.55E-02	2.63E-01	Distal Intergenic
Frmd4a	307128	chr17	77941788	77942088	-23578	-1.36	2.55E-02	2.63E-01	Distal Intergenic
Aftph	305544	chr14	104969794	104970094	5501	-1.32	2.56E-02	2.63E-01	Intron (NM_001305127/305544, intron 1 of 8)
Vgll4	297523	chr4	146779522	146779822	59575	1.51	2.56E-02	2.63E-01	Exon (NM_001015004/297523, exon 5 of 5)
Gnai1	25686	chr4	13405308	13405608	157	0.95	2.56E-02	2.63E-01	Promoter (<=1kb)

Klf13	499171	chr1	124790394	124790694	12669	-1.44	2.56E-02	2.63E-01	Intron (NM_001109147/499171, intron 1 of 1)
Iqsec2	685244	chrX	22267809	22268109	20620	1.69	2.56E-02	2.63E-01	Intron (NM_001277425/685244, intron 2 of 14)
Ascc2	498402	chr14	84902637	84902937	29130	1.83	2.56E-02	2.63E-01	Intron (NM_001109091/498402, intron 12 of 18)
Unc50	192356	chr9	44066007	44066307	40985	-1.01	2.56E-02	2.63E-01	Intron (NM_001012225/367252, intron 5 of 15)
Mirlet7g	104796557	chr8	114866841	114867141	-7316	0.96	2.56E-02	2.63E-01	Distal Intergenic
Abcd1	363516	chrX	157094848	157095148	363	0.69	2.56E-02	2.63E-01	Promoter (<=1kb)
Wdr44	246152	chrX	120860153	120860453	0	0.7	2.56E-02	2.63E-01	Promoter (<=1kb)
Slc12a4	29501	chr19	37930599	37930899	7958	-1.42	2.56E-02	2.63E-01	Intron (NM_019229/29501, intron 3 of 23)
Itpk1	500709	chr6	126521254	126521554	60480	-1.13	2.56E-02	2.63E-01	Intron (NM_001191985/500709, intron 2 of 9)
Mllt10	361285	chr17	84847436	84847736	0	1.1	2.56E-02	2.63E-01	Promoter (<=1kb)
Ncoa5	296372	chr3	161498214	161498514	437	0.79	2.56E-02	2.63E-01	Promoter (<=1kb)
Svop	171442	chr12	48323311	48323611	30965	1.44	2.56E-02	2.63E-01	Intron (NM_134404/171442, intron 6 of 15)
Sik3	684112	chr8	50321665	50321965	11402	1.85	2.57E-02	2.63E-01	Intron (NM_001271216/684112, intron 1 of 24)
Dusp6	116663	chr7	41408571	41408871	-66292	-1.45	2.57E-02	2.63E-01	Distal Intergenic
Usp33	310960	chr2	257562641	257562941	-5802	-1.51	2.57E-02	2.63E-01	Distal Intergenic
Acot11	100363074	chr5	126390523	126390823	4832	1.76	2.57E-02	2.63E-01	Intron (NM_001271383/100363074, intron 1 of 15)
Usp47	308896	chr1	176854948	176855248	295	0.69	2.57E-02	2.63E-01	Promoter (<=1kb)
Tnip1	363599	chr10	40276566	40276866	26206	-1.38	2.57E-02	2.63E-01	Intron (NM_001108826/363599, intron 6 of 18)
Ywhah	25576	chr14	83062009	83062309	115	1.15	2.57E-02	2.63E-01	Promoter (<=1kb)
Rab17	503269	chr9	98139503	98139803	-7620	0.81	2.57E-02	2.63E-01	Distal Intergenic
Rab1a	81754	chr14	104475212	104475512	130	0.77	2.57E-02	2.63E-01	Promoter (<=1kb)
Plekho1	310674	chr2	198119922	198120222	0	1.11	2.57E-02	2.63E-01	Promoter (<=1kb)
Krt42	450231	chr10	88190775	88191075	-17865	0.8	2.57E-02	2.63E-01	Distal Intergenic
Csf1	78965	chr2	210560752	210561052	-10206	-1.52	2.58E-02	2.63E-01	Distal Intergenic
Ubxn7	303878	chr11	71694537	71694837	194	0.65	2.58E-02	2.63E-01	Promoter (<=1kb)
Tsga10	252923	chr9	44306419	44306719	149848	-1.42	2.58E-02	2.63E-01	Distal Intergenic
Nfkb1	81736	chr2	240882449	240882749	7304	-1.27	2.58E-02	2.63E-01	Intron (NM_001276711/81736, intron 1 of 24)
Ccm2	305505	chr14	86813371	86813671	289	0.74	2.58E-02	2.63E-01	Promoter (<=1kb)
Sdccag8	305002	chr13	94892663	94892963	4489	-1.45	2.58E-02	2.63E-01	Intron (NM_177929/305002, intron 1 of 17)
Nr2f1	81808	chr2	5579738	5580038	0	1.27	2.58E-02	2.63E-01	Promoter (<=1kb)
Atp5f1a	65262	chr18	74156588	74156888	35	0.58	2.58E-02	2.63E-01	Promoter (<=1kb)
Hdgfl1	171074	chr17	40547300	40547600	376778	1.84	2.58E-02	2.63E-01	Distal Intergenic
Pgc	24864	chr9	15263427	15263727	11190	0.82	2.58E-02	2.63E-01	Downstream (2-3kb)
Chic2	83835	chr14	35683562	35683862	0	0.76	2.58E-02	2.63E-01	Promoter (<=1kb)
Adipor2	312670	chr4	151439655	151439955	-10836	-1.87	2.58E-02	2.63E-01	Distal Intergenic
Parn	360464	chr10	1375639	1375939	85184	-1.65	2.58E-02	2.63E-01	Intron (NM_001270415/360464, intron 21 of 24)
LOC689574	689574	chr4	62794324	62794624	13728	1.5	2.58E-02	2.63E-01	Distal Intergenic
Tenm3	306451	chr16	46624494	46624794	200625	-1.6	2.58E-02	2.63E-01	Intron (NM_001169133/306451, intron 2 of 28)
Pou1f1	25517	chr11	3161502	3161802	515637	-1.54	2.58E-02	2.63E-01	Distal Intergenic
Mllt3	114510	chr5	106193866	106194166	8395	-1.55	2.58E-02	2.63E-01	Intron (NM_053718/114510, intron 1 of 7)
Lpin1	313977	chr6	41483999	41484299	385747	-1.55	2.58E-02	2.63E-01	Distal Intergenic
Snx33	315696	chr8	61576094	61576394	18638	1.78	2.58E-02	2.63E-01	Distal Intergenic
Zfat	362925	chr7	108613593	108613893	591476	1.17	2.58E-02	2.63E-01	Distal Intergenic
C1qtnf7	305423	chr14	72378200	72378500	-353063	1.02	2.58E-02	2.63E-01	Distal Intergenic
Ypel5	298792	chr6	24064599	24064899	-4452	1.07	2.58E-02	2.63E-01	Distal Intergenic
Hif1a	29560	chr6	96784101	96784401	-26784	-1.78	2.59E-02	2.63E-01	Distal Intergenic
Ttc39a	298366	chr5	129042870	129043170	-451	1.55	2.59E-02	2.63E-01	Promoter (<=1kb)
Agpat5	306582	chr16	75855444	75855744	1	1.01	2.59E-02	2.63E-01	Promoter (<=1kb)
Tab2	308267	chr1	2073542	2073842	54	1.12	2.59E-02	2.63E-01	Promoter (<=1kb)
Creb1	81646	chr9	71105023	71105323	-124785	1.45	2.59E-02	2.63E-01	Distal Intergenic
Smad2	29357	chr18	72550543	72550843	89	1.54	2.59E-02	2.63E-01	Promoter (<=1kb)
Btg1	29618	chr7	38121586	38121886	308755	-1.24	2.59E-02	2.63E-01	Distal Intergenic
Gsdme	353316	chr4	79999717	80000017	-39	0.65	2.59E-02	2.63E-01	Promoter (<=1kb)
Zfp281	305083	chr13	53537400	53537700	6320	-1.46	2.59E-02	2.63E-01	Distal Intergenic
Dram1	679937	chr7	28963448	28963748	-30807	-1.37	2.59E-02	2.63E-01	Distal Intergenic
Uspl1	288447	chr12	6975985	6976285	-19071	-1.59	2.59E-02	2.63E-01	Distal Intergenic
Zmiz1	361103	chr16	1763745	1764045	14554	-1.52	2.59E-02	2.63E-01	Intron (NM_001108393/361103, intron 1 of 23)
Ddit4	140942	chr20	29478950	29479250	32132	-1.49	2.59E-02	2.63E-01	Distal Intergenic
Zzz3	310958	chr2	257604716	257605016	-28409	1.29	2.59E-02	2.63E-01	Distal Intergenic
Pin1	298696	chr8	21673406	21673706	4170	1.63	2.59E-02	2.63E-01	Intron (NM_001106701/298696, intron 2 of 3)
Sfxn5	261737	chr4	117154373	117154673	-42825	0.88	2.59E-02	2.63E-01	Distal Intergenic
Sohlh2	619575	chr2	144592215	144592515	460	-1.41	2.59E-02	2.63E-01	Promoter (<=1kb)
Tmem204	287129	chr10	14447458	14447758	-4448	1.69	2.59E-02	2.63E-01	Distal Intergenic
Cmtm4	498902	chr19	771507	771807	-12811	1.21	2.59E-02	2.63E-01	Distal Intergenic
Dab2ip	192126	chr3	14881584	14881884	-7379	-1.29	2.59E-02	2.63E-01	Distal Intergenic
Klf6	58954	chr17	67285875	67286175	618743	-1.35	2.60E-02	2.64E-01	Distal Intergenic
Fgfr2	25022	chr1	200448654	200448954	247974	1.81	2.60E-02	2.64E-01	Distal Intergenic
Dguok	297389	chr4	115142080	115142380	65681	0.64	2.60E-02	2.64E-01	Distal Intergenic
Tcf7l2	679869	chr1	276677701	276678001	-26844	-1.29	2.60E-02	2.64E-01	Distal Intergenic
Atp1b2	24214	chr10	56213685	56213985	-1828	1.42	2.60E-02	2.64E-01	Promoter (1-2kb)
Zfp458	499563	chr2	86462411	86462711	-508418	0.69	2.60E-02	2.64E-01	Distal Intergenic
Gpam	29653	chr1	275543869	275544169	362517	-1.53	2.60E-02	2.64E-01	Distal Intergenic
Mef2a	309957	chr1	128324538	128324838	16425	-1.49	2.60E-02	2.64E-01	Intron (NM_001014035/309957, intron 1 of 10)
Sf3b5	680891	chr1	7109513	7109813	7798	-1.44	2.60E-02	2.64E-01	Distal Intergenic
Apba1	83589	chr1	241594523	241594823	0	1.21	2.60E-02	2.64E-01	Promoter (<=1kb)
Nadk	100125370	chr5	172994288	172994588	7997	-1.66	2.60E-02	2.64E-01	Intron (NM_001109678/100125370, intron 1 of 11)
Aebp2	297705	chr4	174809978	174810278	-681	-1.68	2.60E-02	2.64E-01	Promoter (<=1kb)
Mir551b	100314268	chr2	117337352	117337652	469705	-1.52	2.61E-02	2.64E-01	Distal Intergenic
Plppr3	314614	chr7	12652164	12652464	-91	1.06	2.61E-02	2.64E-01	Promoter (<=1kb)
Cdc42ep1	315121	chr7	120063602	120063902	-3650	0.7	2.61E-02	2.64E-01	Distal Intergenic
Rhag	65207	chr9	23481264	23481564	11517	-1.13	2.61E-02	2.64E-01	Intron (NM_023022/65207, intron 1 of 9)
Nek7	360850	chr13	55597877	55598177	-36151	-1	2.61E-02	2.64E-01	Distal Intergenic
Myof	309499	chr1	256712001	256712301	22426	-1.1	2.61E-02	2.64E-01	Exon (NM_001354115/309499, exon 2 of 53)
Rad54l2	363135	chr8	115482270	115482570	8070	-1.79	2.61E-02	2.64E-01	Intron (NM_001134520/363135, intron 1 of 22)
Cox18	289522	chr14	19434846	19435146	93	0.73	2.61E-02	2.65E-01	Promoter (<=1kb)
Gcc2	309798	chr20	27832985	27833285	0	0.63	2.62E-02	2.65E-01	Promoter (<=1kb)
Asf1a	294408	chr20	34929746	34930046	35327	1.07	2.62E-02	2.65E-01	Distal Intergenic
Fbxw8	304522	chr12	44046157	44046457	0	1.29	2.62E-02	2.65E-01	Promoter (<=1kb)
Noc3l	361753	chr1	257498486	257498786	58	0.57	2.62E-02	2.65E-01	Promoter (<=1kb)
Acadl	25287	chr9	73815796	73816096	55758	1.29	2.62E-02	2.65E-01	Distal Intergenic
LOC103695172	103695172	chr3	59688625	59688925	-129008	0.99	2.62E-02	2.65E-01	Distal Intergenic
Tssk6	290670	chr16	21234231	21234531	40157	-1.02	2.62E-02	2.65E-01	Intron (NM_001013881/290669, intron 2 of 12)
Tnfaip8	307428	chr18	44468431	44468731	-268423	0.96	2.62E-02	2.65E-01	Distal Intergenic
Nrip1	304157	chr11	14741471	14741771	0	1.18	2.62E-02	2.65E-01	Promoter (<=1kb)

Prkd1	85421	chr6	71203878	71204178	145071	-1.36	2.62E-02	2.65E-01	Intron (NM_001276715/85421, intron 1 of 18)
Ssu72	298681	chr5	173139810	173140110	-12854	1.1	2.62E-02	2.65E-01	Distal Intergenic
Tcf7l2	679869	chr1	276648175	276648475	-56370	1.13	2.62E-02	2.65E-01	Distal Intergenic
Actr2	289820	chr14	104375394	104375694	0	1.48	2.62E-02	2.65E-01	Promoter (<=1kb)
Eif4g3	298573	chr5	156422025	156422325	25330	-1.35	2.62E-02	2.65E-01	Intron (NM_001106693/298573, intron 1 of 30)
Cnih2	361705	chr1	220478035	220478335	1797	1.57	2.62E-02	2.65E-01	Promoter (1-2kb)
Stxbp1	25558	chr3	11881479	11881779	3638	-1.5	2.62E-02	2.65E-01	Intron (NM_013038/25558, intron 1 of 18)
Bcl9l	300673	chr8	48805497	48805797	0	1.46	2.63E-02	2.65E-01	Promoter (<=1kb)
Htra3	360959	chr14	80254847	80255147	6707	-1.29	2.63E-02	2.65E-01	Intron (NM_001271027/360959, intron 1 of 8)
Fignl1	289777	chr14	91907664	91907964	-3231	-1.52	2.63E-02	2.65E-01	3' UTR
Sh3glb1	292156	chr2	250743601	250743901	295	1.53	2.63E-02	2.65E-01	Promoter (<=1kb)
Itga8	364786	chr17	79701801	79702101	-25302	-1.28	2.63E-02	2.65E-01	Distal Intergenic
Creb3l2	362339	chr4	64906948	64907248	74271	-1.39	2.63E-02	2.65E-01	Intron (NM_001012188/362339, intron 2 of 11)
Slco6d1	367321	chr9	105081267	105081567	-211033	1.78	2.63E-02	2.65E-01	Distal Intergenic
Baiap2	117542	chr10	109118110	109118410	10721	-1.24	2.63E-02	2.65E-01	Intron (NM_057196/117542, intron 1 of 13)
Ube2g1	64631	chr10	59173583	59173883	315	0.99	2.63E-02	2.65E-01	Promoter (<=1kb)
Tardbp	298648	chr5	165667336	165667636	-225277	1.74	2.63E-02	2.65E-01	Distal Intergenic
Sesn3	315427	chr8	12823287	12823587	132	0.86	2.63E-02	2.65E-01	Promoter (<=1kb)
Pxmp4	282634	chr3	150203805	150204105	-94912	-1.34	2.63E-02	2.65E-01	Distal Intergenic
Magt1	116967	chrX	77061176	77061476	128	0.63	2.64E-02	2.65E-01	Promoter (<=1kb)
Zfp281	305083	chr13	53507000	53507300	-23780	1.29	2.64E-02	2.65E-01	Distal Intergenic
Emc8	361425	chr19	54210315	54210615	35195	1.24	2.64E-02	2.65E-01	Distal Intergenic
Nox1	114243	chrX	104925406	104925706	6802	1.56	2.64E-02	2.65E-01	Intron (NM_053683/114243, intron 2 of 12)
Gjb6	84403	chr15	37431743	37432043	-22075	1.07	2.64E-02	2.65E-01	Distal Intergenic
Axin2	29134	chr10	97269867	97270167	57384	-1.55	2.64E-02	2.65E-01	Distal Intergenic
Agap1	316611	chr9	96610593	96610893	-135547	-1.49	2.64E-02	2.65E-01	Distal Intergenic
Lrch1	502020	chr15	56898637	56898937	71428	-1.41	2.64E-02	2.65E-01	Intron (NM_001134727/502020, intron 1 of 18)
Dcn	29139	chr7	38400014	38400314	-341936	1.8	2.64E-02	2.66E-01	Distal Intergenic
Zfp36l2	298765	chr6	7494000	7494300	-72544	1.7	2.64E-02	2.66E-01	Intron (NM_001191769/313865, intron 29 of 36)
Agap2	65218	chr7	70364106	70364406	-407	0.82	2.64E-02	2.66E-01	Promoter (<=1kb)
Nr2f2	113984	chr1	131455124	131455424	-435	1.17	2.65E-02	2.66E-01	Promoter (<=1kb)
Mcur1	291034	chr17	24102859	24103159	79266	0.7	2.65E-02	2.66E-01	Distal Intergenic
Eif4enif1	305468	chr14	83425487	83425787	12519	-1.39	2.65E-02	2.66E-01	Intron (NM_001107230/305468, intron 2 of 18)
Ms4a15	365408	chr1	227057331	227057631	-10041	-1.43	2.65E-02	2.66E-01	Distal Intergenic
Ehd3	192249	chr6	25139530	25139830	63383	-1.28	2.65E-02	2.66E-01	Distal Intergenic
Blmh	287552	chr10	63255048	63255348	-14601	1.96	2.65E-02	2.66E-01	Distal Intergenic
Mpp6	362359	chr4	79846731	79847031	210	1.22	2.65E-02	2.66E-01	Promoter (<=1kb)
Mib1	307594	chr18	1971340	1971640	426	1.23	2.65E-02	2.66E-01	Promoter (<=1kb)
Ppp2r2d	246255	chr1	211206030	211206330	127	1	2.65E-02	2.66E-01	Promoter (<=1kb)
Bzw1	363232	chr9	65279382	65279682	0	0.64	2.65E-02	2.66E-01	Promoter (<=1kb)
Dnal4	300078	chr7	120989752	120990052	10220	-1.07	2.65E-02	2.66E-01	Intron (NM_001009666/300078, intron 2 of 3)
Npepps	50558	chr10	85200940	85201240	21621	-1.34	2.65E-02	2.66E-01	Intron (NM_080395/50558, intron 2 of 22)
Gata3	85471	chr17	72490208	72490508	59391	1.67	2.65E-02	2.66E-01	Distal Intergenic
Mir148b	100313977	chr7	144919261	144919561	7223	1.19	2.65E-02	2.66E-01	Intron (NM_001108117/315345, intron 4 of 8)
Ldhd	307858	chr19	43806790	43807090	41847	-1.56	2.66E-02	2.66E-01	Distal Intergenic
Dhx30	367172	chr8	118121252	118121552	70857	-1.56	2.66E-02	2.66E-01	Intron (NM_001024278/367171, intron 8 of 16)
Guca2b	64055	chr5	138851191	138851491	-153550	-1.14	2.66E-02	2.66E-01	Distal Intergenic
LOC100363294	100363294	chr18	27582684	27582984	32089	-1.07	2.66E-02	2.66E-01	Distal Intergenic
Strn3	114520	chr6	72474516	72474816	-11840	1.18	2.66E-02	2.66E-01	Intron (NM_001130999/366618, intron 1 of 5)
Mir297	100314063	chr10	101611468	101611768	84570	-1.51	2.66E-02	2.67E-01	Distal Intergenic
Hist2h2aa3	365877	chr2	198398005	198398305	9239	-1.42	2.66E-02	2.67E-01	Distal Intergenic
Elf5	366142	chr3	93337700	93338000	-7392	0.88	2.66E-02	2.67E-01	Distal Intergenic
Ppard	25682	chr20	7818319	7818619	30	1.09	2.67E-02	2.67E-01	Promoter (<=1kb)
Adamts10	314655	chr7	18409270	18409570	123	0.62	2.67E-02	2.67E-01	Promoter (<=1kb)
Sgms2	310849	chr2	236540169	236540469	-59667	-1.51	2.67E-02	2.67E-01	Distal Intergenic
Nanos2	365213	chr1	79870417	79870717	-23733	1.95	2.67E-02	2.67E-01	Distal Intergenic
Mbd3	362834	chr7	12179284	12179584	238	1.23	2.67E-02	2.67E-01	Promoter (<=1kb)
Nicn1	619581	chr8	117028902	117029202	-33787	-1.36	2.67E-02	2.67E-01	Distal Intergenic
Tas2r135	502757	chr4	71809967	71810267	14199	1.7	2.68E-02	2.67E-01	Distal Intergenic
Uap1	498272	chr13	88458848	88459148	38753	-1.02	2.68E-02	2.67E-01	Distal Intergenic
Nr2f6	245980	chr16	19777296	19777596	0	0.98	2.68E-02	2.67E-01	Promoter (<=1kb)
Stom	296655	chr3	14532803	14533103	5096	-1.25	2.68E-02	2.67E-01	Intron (NM_001011965/296655, intron 1 of 6)
Aif1l	362107	chr3	9262241	9262541	87	0.72	2.68E-02	2.67E-01	Promoter (<=1kb)
Tmem220	287405	chr10	53562574	53562874	-8106	-1.34	2.68E-02	2.67E-01	Distal Intergenic
Lin28c	298542	chr4	97112858	97113158	-116235	1.1	2.68E-02	2.67E-01	Distal Intergenic
Rsl24d1	363099	chr8	79852311	79852611	59724	-1.58	2.68E-02	2.67E-01	Distal Intergenic
Rab35	288700	chr12	46708468	46708768	9587	-0.82	2.68E-02	2.67E-01	Intron (NM_001013046/288700, intron 3 of 5)
Usp49	316211	chr9	15374387	15374687	678	0.74	2.68E-02	2.67E-01	Promoter (<=1kb)
Ttc7b	362768	chr6	124527643	124527943	-93579	1.01	2.68E-02	2.67E-01	Distal Intergenic
Lhx2	296706	chr3	22640370	22640670	0	1.11	2.68E-02	2.67E-01	Promoter (<=1kb)
Aida	682999	chr13	101698216	101698516	120	0.59	2.68E-02	2.67E-01	Promoter (<=1kb)
Map4k4	301363	chr9	46688907	46689207	30985	-1.33	2.68E-02	2.67E-01	Intron (NM_001106904/301363, intron 1 of 28)
Jun	24516	chr5	114060202	114060502	-45925	-1.35	2.68E-02	2.67E-01	Distal Intergenic
Tada2b	289717	chr14	79451284	79451584	5227	-1.42	2.69E-02	2.67E-01	Intron (NM_001170455/289717, intron 1 of 1)
Atg4c	313391	chr5	117864959	117865259	2213	-1.42	2.69E-02	2.67E-01	Promoter (2-3kb)
Kbtbd2	312372	chr4	87100140	87100440	10585	-1.34	2.69E-02	2.67E-01	5' UTR
Ltbp1	59107	chr6	21493362	21493662	106780	-1.04	2.69E-02	2.67E-01	Intron (NM_021587/59107, intron 3 of 33)
Nupl2	499974	chr4	7451127	7451427	77679	-1.17	2.69E-02	2.67E-01	Distal Intergenic
Schip1	295105	chr2	172923414	172923714	163522	-1.38	2.69E-02	2.67E-01	Intron (NM_001100666/295105, intron 4 of 10)
Fstl1	79210	chr11	65811452	65811752	33623	-1.47	2.69E-02	2.67E-01	Intron (NM_024369/79210, intron 2 of 10)
Mkln1	83536	chr4	58589850	58590150	-103234	-1.21	2.69E-02	2.67E-01	Distal Intergenic
Serinc5	170907	chr2	22195595	22195895	88	0.63	2.69E-02	2.67E-01	Promoter (<=1kb)
Camk2d	24246	chr2	230946095	230946395	44969	-1.5	2.69E-02	2.67E-01	Intron (NM_012519/24246, intron 3 of 20)
Snx7	310815	chr2	221099465	221099765	0	0.91	2.69E-02	2.67E-01	Promoter (<=1kb)
Kcnh2	117018	chr4	7372112	7372412	16511	1.94	2.69E-02	2.68E-01	Intron (NM_053949/117018, intron 2 of 14)
Miip	298643	chr5	164664551	164664851	14599	0.6	2.69E-02	2.68E-01	Distal Intergenic
Tmbim6	24822	chr7	141072043	141072343	18167	1.18	2.69E-02	2.68E-01	Distal Intergenic
Ccl5	81780	chr10	70749900	70750200	-5597	-1.81	2.69E-02	2.68E-01	Distal Intergenic
Pdk1	116551	chr3	58734353	58734653	203483	1.07	2.69E-02	2.68E-01	Distal Intergenic
Rab31	246324	chr9	113494637	113494937	9669	-1.36	2.69E-02	2.68E-01	Intron (NM_145094/246324, intron 1 of 6)
Aldh16a1	361571	chr1	101144676	101144976	6344	1.61	2.69E-02	2.68E-01	Exon (NM_001033706/361571, exon 3 of 17)
Cbr4	359725	chr16	31913675	31913975	58532	-1.27	2.70E-02	2.68E-01	Distal Intergenic
Tas2r126	246219	chr4	71823869	71824169	-12508	1.27	2.70E-02	2.68E-01	Distal Intergenic
Dlat	81654	chr8	55081729	55082029	5803	-1.64	2.70E-02	2.68E-01	Intron (NM_031025/81654, intron 5 of 13)

Sh2d4a	306376	chr16	23104985	23105285	51540	1.69	2.70E-02	2.68E-01	Exon (NM_001012048/306376, exon 5 of 9)
Tgif1	316742	chr9	119100453	119100753	89945	-1.45	2.70E-02	2.68E-01	Intron (NM_022946/65040, intron 7 of 12)
Htr5b	79247	chr13	37453832	37454132	38548	1.9	2.70E-02	2.68E-01	Intron (NM_001024997/304743, intron 17 of 23)
Slc25a14	85263	chrX	135441541	135441841	-29322	1.35	2.70E-02	2.68E-01	Distal Intergenic
Zmiz1	361103	chr16	1662553	1662853	-86338	1.67	2.70E-02	2.68E-01	Distal Intergenic
Hat1	296501	chr3	57986659	57986959	-35436	-1.39	2.70E-02	2.68E-01	Distal Intergenic
Scx	680712	chr7	117519316	117519616	241	0.97	2.70E-02	2.68E-01	Promoter (<=1kb)
Emc2	362905	chr7	82549880	82550180	211241	1.09	2.70E-02	2.68E-01	Distal Intergenic
Igsf3	295325	chr2	203764105	203764405	-4324	1.87	2.70E-02	2.68E-01	Intron (NM_012830/497761, intron 1 of 6)
Tyrp1	298182	chr5	98286445	98286745	-100546	-1.35	2.70E-02	2.68E-01	Distal Intergenic
Sp4	25162	chr6	146086890	146087190	108629	-1.14	2.70E-02	2.68E-01	Distal Intergenic
Ap1s2	302671	chrX	32355690	32355990	-383	1.25	2.70E-02	2.68E-01	Promoter (<=1kb)
Tax1bp1	246244	chr4	83110324	83110624	333578	1.76	2.70E-02	2.68E-01	Distal Intergenic
Yeats4	299810	chr7	60294552	60294852	0	0.67	2.70E-02	2.68E-01	Promoter (<=1kb)
Cdc42ep5	361505	chr1	73721129	73721429	2124	0.63	2.71E-02	2.68E-01	Promoter (2-3kb)
Hmga2	84017	chr7	65432329	65432629	-156921	1.04	2.71E-02	2.68E-01	Distal Intergenic
Rnf34	282845	chr12	39130777	39131077	54091	1.2	2.71E-02	2.68E-01	Intron (NM_001100679/304495, intron 18 of 28)
Brd1	315210	chr7	129723232	129723532	771	1.05	2.71E-02	2.68E-01	Promoter (<=1kb)
Stip1	192277	chr1	222292743	222293043	93	0.89	2.71E-02	2.68E-01	Promoter (<=1kb)
Ampd3	25095	chr1	175573662	175573962	-12135	-1.55	2.71E-02	2.68E-01	Distal Intergenic
Agap1	316611	chr9	96721925	96722225	-24215	1.23	2.71E-02	2.68E-01	Distal Intergenic
Slco6d1	367321	chr9	105127334	105127634	-257100	-1.59	2.71E-02	2.68E-01	Distal Intergenic
Cyp51	25427	chr4	27068062	27068362	-107202	-1.49	2.71E-02	2.68E-01	Distal Intergenic
Kras	24525	chr4	179515783	179516083	-3312	1.27	2.71E-02	2.68E-01	Distal Intergenic
Txn2	79462	chr7	119118283	119118583	39590	1.78	2.71E-02	2.68E-01	Distal Intergenic
LOC500350	500350	chr4	170404181	170404481	56771	2	2.71E-02	2.68E-01	Distal Intergenic
Spp1	25353	chr14	6727763	6728063	-47842	-0.88	2.71E-02	2.68E-01	Distal Intergenic
Cd2ap	316258	chr9	20807376	20807676	12696	-1.12	2.71E-02	2.68E-01	Intron (NM_181475/316258, intron 2 of 16)
Nomo1	361578	chr1	102017245	102017545	0	0.73	2.71E-02	2.68E-01	Promoter (<=1kb)
Nsd2	680537	chr14	82192880	82193180	-21400	-1.28	2.71E-02	2.68E-01	Distal Intergenic
LOC682259	682259	chr1	129376631	129376931	237472	1.66	2.71E-02	2.68E-01	Distal Intergenic
Nap1l1	89825	chr7	54213549	54213849	230	1.16	2.71E-02	2.68E-01	Promoter (<=1kb)
Rab21	299799	chr7	58286074	58286374	396	0.67	2.71E-02	2.68E-01	Promoter (<=1kb)
Scaf11	312030	chr7	137680688	137680988	175349	0.84	2.71E-02	2.68E-01	Distal Intergenic
Rab15	299156	chr6	99865129	99865429	4891	-1.2	2.71E-02	2.68E-01	Intron (NM_198749/299156, intron 1 of 6)
Gnrh1	25194	chr15	44602897	44603197	161041	-1.49	2.72E-02	2.68E-01	Distal Intergenic
Tram1	312903	chr5	5355579	5355879	140383	-1.21	2.72E-02	2.68E-01	Distal Intergenic
Jmjd1c	171120	chr20	22882628	22882928	0	1.43	2.72E-02	2.68E-01	Promoter (<=1kb)
Arhgap22	306279	chr16	9471786	9472086	-91132	-1.26	2.72E-02	2.68E-01	Distal Intergenic
Igtp	303163	chr10	43645191	43645491	14086	0.71	2.72E-02	2.68E-01	Distal Intergenic
Elk1	314436	chrX	1288929	1289229	0	0.68	2.72E-02	2.68E-01	Promoter (<=1kb)
Rpl21	79449	chr12	10018295	10018595	-18297	-1.14	2.72E-02	2.68E-01	Distal Intergenic
Ing3	312154	chr4	49017389	49017689	78	0.89	2.72E-02	2.68E-01	Promoter (<=1kb)
Kmt5a	689820	chr12	37576140	37576440	-1390	0.58	2.72E-02	2.68E-01	Promoter (1-2kb)
Afap1	140935	chr14	80006327	80006627	-18412	-1.44	2.72E-02	2.68E-01	Distal Intergenic
Slc41a3	641603	chr4	123423021	123423321	71421	1.25	2.72E-02	2.68E-01	Distal Intergenic
Bin1	117028	chr18	25128278	25128578	-35070	1.87	2.72E-02	2.68E-01	Distal Intergenic
Myof	309499	chr1	256648149	256648449	86278	-1.21	2.72E-02	2.68E-01	Exon (NM_001354115/309499, exon 21 of 53)
Sdccag8	305002	chr13	94887069	94887369	-805	1.07	2.72E-02	2.68E-01	Promoter (<=1kb)
Pdgfrb	24629	chr18	56365213	56365513	536	0.6	2.73E-02	2.68E-01	Promoter (<=1kb)
RGD1562024	498699	chr17	8694833	8695133	-75096	1.27	2.73E-02	2.68E-01	Distal Intergenic
Dcn	29139	chr7	38743638	38743938	1388	1.5	2.73E-02	2.68E-01	Promoter (1-2kb)
Spred1	296072	chr3	108804699	108804999	9362	-1.36	2.73E-02	2.68E-01	Intron (NM_001047089/296072, intron 1 of 6)
Galnt1	79214	chr18	16211083	16211383	64636	-0.69	2.73E-02	2.68E-01	Distal Intergenic
Borcs5	362452	chr4	168453859	168454159	57084	-1.56	2.73E-02	2.68E-01	Intron (NM_001108650/362452, intron 3 of 3)
Rgs3	54293	chr5	78637405	78637705	153512	-1.39	2.73E-02	2.68E-01	Distal Intergenic
Rap1b	171337	chr7	61001151	61001451	-140161	-0.95	2.73E-02	2.68E-01	Distal Intergenic
Sparc	24791	chr10	40777244	40777544	-13103	1.71	2.73E-02	2.68E-01	Distal Intergenic
Sptbn1	305614	chr14	114586501	114586801	105963	-1.35	2.73E-02	2.68E-01	Intron (NM_001013130/305614, intron 2 of 36)
Rspo2	500863	chr7	81937038	81937338	122321	-1.39	2.73E-02	2.68E-01	Distal Intergenic
Cyb561	303601	chr10	94049002	94049302	98254	-1.6	2.73E-02	2.68E-01	Intron (NM_001191653/303599, intron 11 of 25)
Dpy19l1	315496	chr8	25669071	25669371	23	1.41	2.73E-02	2.69E-01	Promoter (<=1kb)
Med9	497914	chr10	46314625	46314925	0	0.66	2.74E-02	2.69E-01	Promoter (<=1kb)
Snx18	310097	chr2	45422837	45423137	57661	1.73	2.74E-02	2.69E-01	Distal Intergenic
Ddah2	294239	chr20	5045418	5045718	-3809	-1.44	2.74E-02	2.69E-01	Exon (NM_001002807/406864, exon 5 of 6)
Ppfibp1	312855	chr4	181329324	181329624	13880	-1.21	2.74E-02	2.69E-01	Intron (NM_001107896/312855, intron 1 of 25)
Fam220a	498145	chr12	13035849	13036149	30894	-0.64	2.74E-02	2.69E-01	Distal Intergenic
Fndc1	308099	chr1	47629202	47629502	3103	1.27	2.74E-02	2.69E-01	Intron (NM_001038615/308099, intron 1 of 19)
Foxj2	502886	chr4	155644833	155645133	-8585	0.59	2.74E-02	2.69E-01	Distal Intergenic
Magi1	500261	chr4	126241089	126241389	58021	0.6	2.74E-02	2.69E-01	Intron (NM_001030045/500261, intron 1 of 22)
C1rl	408246	chr4	157114260	157114560	6070	-1.51	2.74E-02	2.69E-01	Intron (NM_001002804/408246, intron 1 of 4)
Slc14a2	54302	chr18	75376387	75376687	-434998	1.9	2.74E-02	2.69E-01	Distal Intergenic
Tent4b	307745	chr19	19802071	19802371	30088	-1.48	2.74E-02	2.69E-01	Intron (NM_001107416/307745, intron 1 of 9)
Hcn1	84390	chr2	50158312	50158612	58736	1.46	2.75E-02	2.69E-01	Intron (NM_053375/84390, intron 2 of 7)
Arhgap21	307178	chr17	87856304	87856604	-6575	-1.53	2.75E-02	2.69E-01	Distal Intergenic
Ankrd1	27064	chr1	254730887	254731187	4358	1.46	2.75E-02	2.69E-01	Exon (NM_013220/27064, exon 6 of 9)
Rasgrp1	29434	chr3	109412684	109412984	-368264	2.05	2.75E-02	2.69E-01	Distal Intergenic
Rcor3	684192	chr13	110795509	110795809	68660	-1.48	2.75E-02	2.69E-01	Distal Intergenic
Lcmt2	296098	chr3	113091016	113091316	454	0.58	2.75E-02	2.69E-01	Promoter (<=1kb)
Kifbp	606294	chr20	32207500	32207800	3495	2.06	2.75E-02	2.69E-01	Exon (NM_001031627/606294, exon 2 of 7)
Usp53	295425	chr2	227160029	227160329	50	0.69	2.75E-02	2.69E-01	Promoter (<=1kb)
Pitpna	29525	chr10	63731781	63732081	14	1.05	2.75E-02	2.69E-01	Promoter (<=1kb)
Anxa4	79124	chr4	118595116	118595416	164	0.64	2.75E-02	2.69E-01	Promoter (<=1kb)
Ptpn1	24697	chr3	164569322	164569622	-95910	-1.19	2.75E-02	2.69E-01	Distal Intergenic
Atg7	312647	chr4	146665919	146666219	67503	-1.46	2.76E-02	2.69E-01	Intron (NM_001012097/312647, intron 16 of 17)
Ppef1	317498	chrX	35966102	35966402	143415	1.1	2.76E-02	2.69E-01	Distal Intergenic
Mir708	100314266	chr1	161134515	161134815	-86431	1.96	2.76E-02	2.69E-01	Distal Intergenic
Pex14	64460	chr5	165695526	165695826	222619	1.38	2.76E-02	2.69E-01	Distal Intergenic
Celsr2	83465	chr2	211204079	211204379	3079	1.66	2.76E-02	2.69E-01	Exon (NM_001191110/83465, exon 1 of 34)
Bank1	365948	chr2	241390287	241390587	154663	-1.45	2.76E-02	2.69E-01	Intron (NM_001047918/365948, intron 7 of 16)
Agap1	316611	chr9	96604971	96605271	-141169	-1.4	2.76E-02	2.69E-01	Distal Intergenic
Sema4g	361764	chr1	264738823	264739123	-2788	0.93	2.76E-02	2.69E-01	Promoter (2-3kb)
Slc2a1	24778	chr5	138164552	138164852	9872	-1.3	2.76E-02	2.69E-01	Intron (NM_138827/24778, intron 1 of 9)
LOC100911360	100911360	chr8	73388099	73388399	146596	1.82	2.76E-02	2.69E-01	Distal Intergenic

Cdh1	83502	chr19	38727618	38727918	-40549	-1.6	2.76E-02	2.69E-01	Distal Intergenic
Spag9	360600	chr10	81698250	81698550	4480	-1.33	2.76E-02	2.69E-01	Intron (NM_001108290/360600, intron 1 of 29)
Dym	291433	chr18	70996190	70996490	50	0.94	2.76E-02	2.69E-01	Promoter (<=1kb)
RGD1566386	304336	chr12	18137546	18137846	1574	1.49	2.76E-02	2.69E-01	Promoter (1-2kb)
Ppp4r3a	314388	chr6	125070082	125070382	12610	-1.42	2.76E-02	2.69E-01	Intron (NM_001108050/314388, intron 1 of 19)
Bmf	246142	chr3	110304590	110304890	19194	1.66	2.76E-02	2.69E-01	Downstream (1-2kb)
Maml3	310405	chr2	141146102	141146402	130484	-1.3	2.76E-02	2.69E-01	Intron (NM_001107675/310405, intron 1 of 5)
Efhc1	301295	chr9	27152346	27152646	83841	-1.07	2.76E-02	2.69E-01	Distal Intergenic
Ociad1	289590	chr14	37550039	37550339	-74621	-1.41	2.76E-02	2.69E-01	Distal Intergenic
Utrn	25600	chr1	6931056	6931356	38684	1.57	2.76E-02	2.69E-01	Intron (NM_013070/25600, intron 2 of 74)
Cyp26a1	154985	chr1	256378004	256378304	-4557	0.68	2.77E-02	2.70E-01	Distal Intergenic
Coq10a	362810	chr7	2786851	2787151	0	1.18	2.77E-02	2.70E-01	Promoter (<=1kb)
Igf2r	25151	chr1	48222528	48222828	46433	1.45	2.77E-02	2.70E-01	Intron (NM_012756/25151, intron 10 of 47)
Rab2a	65158	chr5	21642286	21642586	9377	-1.48	2.77E-02	2.70E-01	Intron (NM_031718/65158, intron 1 of 7)
Ntan1	360462	chr10	3217958	3218258	-217	1.2	2.77E-02	2.70E-01	Promoter (<=1kb)
Cdv3	315970	chr8	111886610	111886910	-36217	1.47	2.77E-02	2.70E-01	Distal Intergenic
Sema5a	310207	chr2	85631349	85631649	254031	-1.44	2.77E-02	2.70E-01	Intron (NM_001107659/310207, intron 5 of 22)
Spast	362700	chr6	22281542	22281842	44	0.93	2.77E-02	2.70E-01	Promoter (<=1kb)
Mef2d	81518	chr2	187504030	187504330	-7834	1.83	2.77E-02	2.70E-01	Distal Intergenic
Msc	312897	chr5	4446638	4446938	491411	-1.38	2.77E-02	2.70E-01	Distal Intergenic
Bmp7	85272	chr3	170758605	170758905	196915	2	2.78E-02	2.70E-01	Distal Intergenic
Casp2	64314	chr4	71652430	71652730	0	0.6	2.78E-02	2.70E-01	Promoter (<=1kb)
Comt	24267	chr11	86680137	86680437	-35544	-1.23	2.78E-02	2.70E-01	Intron (NM_022584/50551, intron 11 of 17)
Commd10	361323	chr18	41139742	41140042	117187	1.39	2.78E-02	2.70E-01	Intron (NM_001004276/361323, intron 5 of 6)
Fbxl17	316663	chr9	110936985	110937285	-354	1.32	2.78E-02	2.70E-01	Promoter (<=1kb)
Rab10	50993	chr6	27710390	27710690	10430	-1.45	2.78E-02	2.70E-01	Intron (NM_017359/50993, intron 1 of 5)
Atp6v1a	685232	chr11	61548432	61548732	16854	1.86	2.78E-02	2.70E-01	Intron (NM_001108318/685232, intron 1 of 14)
Zfp51	308232	chr1	61313786	61314086	0	0.68	2.78E-02	2.70E-01	Promoter (<=1kb)
Atp13a4	288026	chr11	74831408	74831708	-2549	-1.33	2.78E-02	2.70E-01	Promoter (2-3kb)
Adamtsl3	308787	chr1	144311007	144311307	72049	1.53	2.78E-02	2.70E-01	Intron (NM_001107533/308787, intron 3 of 29)
Rad18	362412	chr4	144685592	144685892	-65008	1.71	2.78E-02	2.70E-01	Intron (NM_001191975/500287, intron 9 of 21)
Hnrnpk	117282	chr17	6665901	6666201	-1363	0.86	2.78E-02	2.70E-01	Promoter (1-2kb)
Gas2l1	360973	chr14	85317963	85318263	0	0.91	2.78E-02	2.70E-01	Promoter (<=1kb)
Gps1	117039	chr10	109966709	109967009	0	0.89	2.78E-02	2.70E-01	Promoter (<=1kb)
Niban2	362115	chr3	11919449	11919749	-1966	1.1	2.78E-02	2.70E-01	Promoter (1-2kb)
Col3a1	84032	chr9	52102848	52103148	79553	-1.44	2.78E-02	2.70E-01	Exon (NM_053488/85250, exon 49 of 58)
Six2	366542	chr6	9146856	9147156	-190968	1.78	2.78E-02	2.70E-01	Distal Intergenic
Gabpb1l	364738	chr17	52785468	52785768	-153212	-1.46	2.78E-02	2.70E-01	Distal Intergenic
Agfg1	363266	chr9	88631296	88631596	23788	-1.46	2.78E-02	2.70E-01	Intron (NM_001135596/363266, intron 2 of 13)
Eloc	64525	chr5	2050449	2050749	6866	-1.65	2.78E-02	2.70E-01	Intron (NM_022593/64525, intron 1 of 3)
Pnpo	64533	chr10	84880893	84881193	0	0.63	2.79E-02	2.70E-01	Promoter (<=1kb)
Mcam	78967	chr8	48487706	48488006	14882	-1.28	2.79E-02	2.70E-01	Distal Intergenic
Aqp1	25240	chr4	85508918	85509218	-42285	1.65	2.79E-02	2.70E-01	Distal Intergenic
Mtdh	170910	chr7	72772725	72773025	285	0.61	2.79E-02	2.70E-01	Promoter (<=1kb)
Sapcd1	406170	chr20	5019811	5020111	0	1.81	2.79E-02	2.70E-01	Promoter (<=1kb)
Aak1	500244	chr4	118655948	118656248	220	0.7	2.79E-02	2.70E-01	Promoter (<=1kb)
Tagln2	304983	chr13	90813520	90813820	299	0.62	2.79E-02	2.70E-01	Promoter (<=1kb)
Cpq	58952	chr7	71734415	71734715	25076	2.1	2.79E-02	2.70E-01	Intron (NM_031640/58952, intron 1 of 8)
Itga11	315744	chr8	67472347	67472647	-94552	1.48	2.79E-02	2.70E-01	Distal Intergenic
Plpp7	296635	chr3	11172333	11172633	57782	-1.44	2.79E-02	2.70E-01	Distal Intergenic
Slc25a26	362403	chr4	126436107	126436407	-85928	-1.24	2.79E-02	2.70E-01	Distal Intergenic
Rassf8	312846	chr4	180080533	180080833	17734	-0.97	2.79E-02	2.70E-01	Intron (NM_001191753/312846, intron 1 of 4)
Prkd3	313834	chr6	1621825	1622125	-6974	1.46	2.79E-02	2.70E-01	Distal Intergenic
Akap6	64553	chr6	73731059	73731359	177849	-1.55	2.80E-02	2.70E-01	Intron (NM_022618/64553, intron 2 of 13)
Rdh8	690953	chr8	21855991	21856291	20888	-1.33	2.80E-02	2.70E-01	Distal Intergenic
Nrbp1	619579	chr6	26497018	26497318	10	1.29	2.80E-02	2.70E-01	Promoter (<=1kb)
Fgf1	25317	chr18	32287362	32287662	-48440	-1.5	2.80E-02	2.70E-01	Intron (NM_012576/24413, intron 1 of 7)
Atoh8	500200	chr4	100117813	100118113	-18296	1.54	2.80E-02	2.70E-01	Distal Intergenic
Aff1	305152	chr14	7342769	7343069	-17124	0.68	2.80E-02	2.70E-01	Distal Intergenic
Cd9	24936	chr4	157970488	157970788	39303	-1.45	2.80E-02	2.70E-01	Distal Intergenic
Arih1	300756	chr8	64262936	64263236	5319	-1.57	2.80E-02	2.70E-01	Intron (NM_001013108/300756, intron 1 of 13)
Shox2	25546	chr2	164122474	164122774	4009	1.83	2.80E-02	2.70E-01	Intron (NM_013028/25546, intron 2 of 5)
Dcakd	360639	chr10	91104218	91104518	13371	0.78	2.80E-02	2.70E-01	Intron (NM_001007724/360639, intron 1 of 4)
Oxnad1	306270	chr16	8173394	8173694	-33529	-1.18	2.80E-02	2.70E-01	Distal Intergenic
Skp1	287280	chr10	37594711	37595011	133	0.77	2.80E-02	2.70E-01	Promoter (<=1kb)
Nacc1	171454	chr19	25801121	25801421	105	1.03	2.80E-02	2.70E-01	Promoter (<=1kb)
Gtpbp10	312054	chr4	25630381	25630681	-5084	-1.33	2.80E-02	2.70E-01	Distal Intergenic
Cacna2d1	25399	chr4	16062130	16062430	68133	-1.49	2.80E-02	2.70E-01	Intron (NM_012919/25399, intron 1 of 38)
Olr1455	405046	chr11	55877359	55877659	380125	1.59	2.80E-02	2.70E-01	Distal Intergenic
Pik3ap1	294048	chr1	260737925	260738225	-99109	-1.35	2.80E-02	2.70E-01	Distal Intergenic
Pcmt1	25604	chr1	1754709	1755009	12750	-1.52	2.80E-02	2.70E-01	Intron (NM_013073/25604, intron 1 of 8)
Smyd4	287525	chr10	62198638	62198938	6969	1.23	2.81E-02	2.70E-01	Intron (NM_001105810/287525, intron 2 of 10)
Saraf	290796	chr16	61622167	61622467	130971	-1.29	2.81E-02	2.70E-01	Distal Intergenic
Diras2	291006	chr17	12991006	12991306	228254	-1.38	2.81E-02	2.70E-01	Distal Intergenic
Cdc42se2	691031	chr10	40122544	40122844	71	0.59	2.81E-02	2.70E-01	Promoter (<=1kb)
Ca14	791259	chr2	198016328	198016628	231	1.77	2.81E-02	2.70E-01	Promoter (<=1kb)
Osbpl5	361686	chr1	216914222	216914522	6103	-1.38	2.81E-02	2.70E-01	Intron (NM_001015024/361686, intron 1 of 21)
Actr3	81732	chr13	41551394	41551694	186928	1.54	2.81E-02	2.70E-01	Intron (NM_001177819/100362110, intron 2 of 8)
Galnt9	304571	chr12	52290037	52290337	-102873	1.01	2.81E-02	2.70E-01	Distal Intergenic
Rspo1	313589	chr5	143006026	143006326	19500	1.79	2.81E-02	2.70E-01	Exon (NM_001107980/313589, exon 5 of 6)
Scd2	83792	chr1	264050228	264050528	-8846	1.22	2.81E-02	2.70E-01	Distal Intergenic
Cdk14	362316	chr4	26047313	26047613	221746	1.39	2.81E-02	2.70E-01	Intron (NM_001108617/362316, intron 4 of 12)
C1galt1	65044	chr4	33948964	33949264	40665	0.78	2.81E-02	2.70E-01	Distal Intergenic
Cth	24962	chr2	264246770	264247070	45946	-1.23	2.81E-02	2.71E-01	Distal Intergenic
Nfia	25492	chr5	116548251	116548551	126353	0.65	2.81E-02	2.71E-01	Intron (NM_012988/25492, intron 1 of 9)
Atp13a3	678704	chr11	73936796	73937096	46	0.87	2.81E-02	2.71E-01	Promoter (<=1kb)
Psma3	29670	chr6	93428562	93428862	5533	-1.39	2.81E-02	2.71E-01	Intron (NM_001004094/408248, intron 2 of 10)
Tatdn2	500295	chr4	145660875	145661175	7273	-1.55	2.82E-02	2.71E-01	Intron (NM_001109252/500295, intron 4 of 7)
Copb1	114023	chr1	181770474	181770774	1958795	1.33	2.82E-02	2.71E-01	Distal Intergenic
Sst	24797	chr11	80350325	80350625	-7547	-0.96	2.82E-02	2.71E-01	Distal Intergenic
Bles03	266609	chr1	220806742	220807042	179	0.82	2.82E-02	2.71E-01	Promoter (<=1kb)
Foxn3	314374	chr6	123358085	123358385	219310	-1.45	2.82E-02	2.71E-01	Intron (NM_001108047/314374, intron 3 of 6)
Phtf2	296762	chr4	10995303	10995603	172045	1.11	2.82E-02	2.71E-01	3' UTR
Arntl	29657	chr1	178081146	178081446	42083	-1.3	2.82E-02	2.71E-01	Intron (NM_024362/29657, intron 3 of 20)

Cnksr3	308113	chr1	43842850	43843150	41117	-1.3	2.82E-02	2.71E-01	Intron (NM_001012061/308113, intron 1 of 12)
Palm2	103692368	chr5	74733804	74734104	84039	-1.35	2.82E-02	2.71E-01	Intron (NM_001305995/103692368, intron 3 of 5)
Slc20a1	81826	chr3	121732901	121733201	6825	-1.35	2.82E-02	2.71E-01	Intron (NM_031148/81826, intron 4 of 9)
Laptm4a	298875	chr6	34042267	34042567	135	0.58	2.82E-02	2.71E-01	Promoter (<=1kb)
Fgf5	60662	chr14	13073389	13073689	-77805	0.65	2.82E-02	2.71E-01	Distal Intergenic
Sp1	24790	chr7	144014075	144014375	0	0.81	2.82E-02	2.71E-01	Promoter (<=1kb)
Marchf3	364878	chr18	51967687	51967987	28997	-1.6	2.82E-02	2.71E-01	Intron (NM_001007759/364878, intron 1 of 4)
Taf2	170844	chr7	94755736	94756036	0	0.6	2.82E-02	2.71E-01	Promoter (<=1kb)
Ahsp	293522	chr1	199747062	199747362	27024	-1.1	2.82E-02	2.71E-01	Distal Intergenic
Setd2	316013	chr8	118802639	118802939	161	0.87	2.82E-02	2.71E-01	Promoter (<=1kb)
Ccdc174	297458	chr4	123754293	123754593	-6150	1.02	2.82E-02	2.71E-01	Distal Intergenic
B4galt1	24390	chr5	57153426	57153726	14884	-1.27	2.82E-02	2.71E-01	Intron (NM_053287/24390, intron 1 of 5)
Sp2	303499	chr10	84926276	84926576	-5390	0.69	2.83E-02	2.71E-01	Distal Intergenic
Ptgfrn	29602	chr2	203501317	203501617	-6926	0.84	2.83E-02	2.71E-01	Distal Intergenic
Naprt	315085	chr7	116915645	116915945	10491	0.71	2.83E-02	2.71E-01	Distal Intergenic
Dgcr8	287954	chr11	86852868	86853168	157	0.85	2.83E-02	2.71E-01	Promoter (<=1kb)
Ldhal6b	369018	chr1	46031819	46032119	277157	0.89	2.83E-02	2.71E-01	Distal Intergenic
Med23	309565	chr1	21586619	21586919	0	0.55	2.83E-02	2.71E-01	Promoter (<=1kb)
Cfap77	499765	chr3	7624881	7625181	7130	1.32	2.83E-02	2.71E-01	Intron (NM_001127563/499765, intron 1 of 5)
Nav2	171563	chr1	104649960	104650260	73371	1.21	2.83E-02	2.71E-01	Intron (NM_138529/171563, intron 1 of 37)
Tcf7l2	679869	chr1	276586463	276586763	-118082	1.42	2.83E-02	2.71E-01	Distal Intergenic
Mir129-2	100313984	chr3	83184962	83185262	-47569	-1.56	2.83E-02	2.71E-01	Distal Intergenic
Tnfaip6	84397	chr3	37599604	37599904	54361	1.03	2.83E-02	2.71E-01	Distal Intergenic
Eed	293104	chr1	154215910	154216210	130	0.84	2.83E-02	2.71E-01	Promoter (<=1kb)
Brpf3	309647	chr20	6049018	6049318	-4880	1.4	2.84E-02	2.71E-01	Distal Intergenic
Itprid2	311146	chr3	66609186	66609486	15411	-1.63	2.84E-02	2.71E-01	Exon (NM_001107738/311146, exon 9 of 18)
Mdm1	314859	chr7	61140137	61140437	-33932	0.84	2.84E-02	2.71E-01	Distal Intergenic
Zc4h2	367838	chrX	64908426	64908726	0	1.11	2.84E-02	2.71E-01	Promoter (<=1kb)
Fbln1	315191	chr7	126134068	126134368	37275	1.27	2.84E-02	2.71E-01	Exon (NM_001127547/315191, exon 14 of 17)
Il3ra	246144	chr12	18540048	18540348	0	0.73	2.84E-02	2.71E-01	Promoter (<=1kb)
Stk11ip	301535	chr9	82729419	82729719	10710	-1.29	2.84E-02	2.71E-01	Exon (NM_001106922/301535, exon 17 of 25)
Banp	292064	chr19	54802378	54802678	35783	1.92	2.84E-02	2.71E-01	Intron (NM_001106191/292064, intron 5 of 13)
Casp3	25402	chr16	48779756	48780056	83148	1.8	2.84E-02	2.71E-01	Distal Intergenic
Dio2	65162	chr6	114489000	114489300	143	-1.4	2.84E-02	2.71E-01	Promoter (<=1kb)
Ric8b	314681	chr7	25885216	25885516	-34351	2.12	2.84E-02	2.71E-01	Intron (NM_001271382/100910996, intron 2 of 5)
Ppp2r5c	691318	chr6	134845163	134845463	504	0.67	2.84E-02	2.71E-01	Promoter (<=1kb)
Klhl23	311114	chr3	56057818	56058118	775	1.36	2.84E-02	2.71E-01	Promoter (<=1kb)
Klhl9	313348	chr5	107333088	107333388	9830	-1.32	2.84E-02	2.71E-01	Distal Intergenic
Slc5a3	114507	chr11	32211171	32211471	-17895	1.16	2.84E-02	2.71E-01	Distal Intergenic
Lrrc8b	305135	chr14	5629624	5629924	-204134	-1.47	2.84E-02	2.71E-01	Intron (NM_001037205/360997, intron 10 of 15)
Dtwd2	361326	chr18	43536755	43537055	408218	-0.96	2.85E-02	2.71E-01	Distal Intergenic
Myl6l	362816	chr7	2876151	2876451	32671	1.2	2.85E-02	2.71E-01	Distal Intergenic
Rpl39	25347	chr14	33032796	33033096	-1522	1.36	2.85E-02	2.71E-01	Promoter (1-2kb)
Dipk2a	315891	chr8	101702417	101702717	457111	2	2.85E-02	2.72E-01	Distal Intergenic
Zfp36l1	29344	chr6	103376472	103376772	-63398	-1.16	2.85E-02	2.72E-01	3' UTR
Cyp19a1	25147	chr8	58769457	58769757	2651	-1.68	2.85E-02	2.72E-01	Promoter (2-3kb)
Sinhcaf	686611	chr4	183424005	183424305	2134	0.73	2.85E-02	2.72E-01	Promoter (2-3kb)
Spint1	311331	chr3	111066067	111066367	16752	-1.34	2.85E-02	2.72E-01	Distal Intergenic
Mir702	100526603	chr12	22704870	22705170	16782	0.93	2.85E-02	2.72E-01	Distal Intergenic
Prtg	315806	chr8	79489671	79489971	0	1.07	2.86E-02	2.72E-01	Promoter (<=1kb)
Zfp36l1	29344	chr6	103284222	103284522	28552	1.58	2.86E-02	2.72E-01	Distal Intergenic
LOC100909970	100909970	chr17	27842460	27842760	1779	0.66	2.86E-02	2.72E-01	Promoter (1-2kb)
Mios	362324	chr4	34238653	34238953	213	0.79	2.86E-02	2.72E-01	Promoter (<=1kb)
Thap12	308845	chr1	163643872	163644172	-19235	-1.56	2.86E-02	2.72E-01	Distal Intergenic
Zmynd11	291259	chr17	63900396	63900696	13148	-1.12	2.86E-02	2.72E-01	Intron (NM_203369/291259, intron 1 of 13)
Gch1	29244	chr15	24012296	24012596	-43325	1.95	2.86E-02	2.72E-01	Distal Intergenic
Wnt10a	316527	chr9	82044204	82044504	-9077	0.73	2.86E-02	2.72E-01	Exon (NM_001108226/316526, exon 3 of 4)
LOC304725	304725	chr13	13324159	13324459	2071158	0.69	2.86E-02	2.72E-01	Distal Intergenic
Ift43	299209	chr6	109956691	109956991	17346	0.92	2.86E-02	2.72E-01	Intron (NM_001134525/299209, intron 2 of 8)
Pbk	290326	chr15	42221320	42221620	-267754	-1.12	2.86E-02	2.72E-01	Distal Intergenic
Rnpc3	691538	chr2	216509428	216509728	0	0.65	2.86E-02	2.72E-01	Promoter (<=1kb)
Ddx54	360815	chr12	41484834	41485134	0	0.66	2.86E-02	2.72E-01	Promoter (<=1kb)
Pcmtd1	366300	chr5	12203864	12204164	-31855	0.65	2.87E-02	2.72E-01	Distal Intergenic
Zswim2	296455	chr3	71161741	71162041	133569	-1.76	2.87E-02	2.72E-01	Distal Intergenic
Htra4	306564	chr16	71782364	71782664	-5302	0.69	2.87E-02	2.72E-01	Distal Intergenic
Lats1	308265	chr1	1784035	1784335	0	1.27	2.87E-02	2.72E-01	Promoter (<=1kb)
Pmf1	681050	chr2	187763738	187764038	7800	1.8	2.87E-02	2.72E-01	Intron (NM_001191568/681050, intron 1 of 4)
Ubc	50522	chr12	36629777	36630077	-8380	-1.21	2.87E-02	2.72E-01	Distal Intergenic
Lrfn4	688721	chr1	219853310	219853610	0	1.17	2.87E-02	2.72E-01	Promoter (<=1kb)
Foxq1	64826	chr17	34156020	34156320	70621	0.91	2.87E-02	2.72E-01	Distal Intergenic
Nek7	360850	chr13	55570864	55571164	-9138	-1.25	2.87E-02	2.72E-01	Distal Intergenic
Olr1356	302954	chr10	11935117	11935417	7468	0.66	2.87E-02	2.73E-01	Distal Intergenic
Pard3	81918	chr19	60023993	60024293	6247	-1.62	2.87E-02	2.73E-01	Intron (NM_031235/81918, intron 1 of 24)
Hnrnpk	117282	chr17	6637281	6637581	-29983	-1.59	2.88E-02	2.73E-01	Distal Intergenic
Ccdc190	498270	chr13	88197211	88197511	-67820	1.72	2.88E-02	2.73E-01	Distal Intergenic
Prkaca	25636	chr19	25095041	25095341	0	1.12	2.88E-02	2.73E-01	Promoter (<=1kb)
Cul1	362356	chr4	77212520	77212820	706	0.84	2.88E-02	2.73E-01	Promoter (<=1kb)
Msantd3	362516	chr5	64282780	64283080	429	0.76	2.88E-02	2.73E-01	Promoter (<=1kb)
Htr2a	29595	chr15	56665724	56666024	-128	0.57	2.89E-02	2.73E-01	Promoter (<=1kb)
Tma16	290686	chr16	24981620	24981920	2805	1.86	2.89E-02	2.73E-01	Promoter (2-3kb)
Plet1	363060	chr8	54873518	54873818	-51848	-1.19	2.89E-02	2.73E-01	Distal Intergenic
Josd1	315134	chr7	120919546	120919846	-285	1.15	2.89E-02	2.73E-01	Promoter (<=1kb)
Pitpna	29525	chr10	63716006	63716306	-15461	-1.19	2.89E-02	2.73E-01	Intron (NM_001105812/287532, intron 13 of 13)
Prdm2	313678	chr5	161784238	161784538	94595	-1.34	2.89E-02	2.73E-01	Intron (NM_001077648/313678, intron 8 of 9)
Ankrd13d	361699	chr1	219535150	219535450	-2477	-1.52	2.89E-02	2.73E-01	Promoter (2-3kb)
Tprg1	360731	chr11	78824369	78824669	37076	-1.52	2.89E-02	2.73E-01	Intron (NM_001108320/360731, intron 2 of 5)
Kat6a	306571	chr16	74014753	74015053	5697	-1.52	2.89E-02	2.73E-01	Intron (NM_001100570/306571, intron 1 of 16)
Ern1	498013	chr10	94681373	94681673	-28715	1.08	2.89E-02	2.73E-01	Distal Intergenic
Stxbp1	25558	chr3	11864439	11864739	20678	-1.46	2.89E-02	2.73E-01	Intron (NM_013038/25558, intron 1 of 18)
Arnt2	25243	chr1	146461401	146461701	94442	1.53	2.89E-02	2.73E-01	Intron (NM_012781/25243, intron 6 of 18)
Srm	84596	chr5	165404748	165405048	-120	0.71	2.89E-02	2.73E-01	Promoter (<=1kb)
Zfp638	312491	chr4	115555362	115555662	-45999	0.6	2.89E-02	2.73E-01	Distal Intergenic
Mettl7a	315306	chr7	141996094	141996394	22541	1.13	2.89E-02	2.73E-01	Distal Intergenic
Numb	29419	chr6	107347986	107348286	-22641	1.66	2.89E-02	2.73E-01	Distal Intergenic

Larp4b	307070	chr17	63269907	63270207	33900	-1.3	2.89E-02	2.73E-01	Intron (NM_001107361/307070, intron 4 of 18)
Zfp830	497967	chr10	70118385	70118685	135	0.57	2.89E-02	2.73E-01	Promoter (<=1kb)
Twist2	59327	chr9	98997345	98997645	73211	-1.3	2.89E-02	2.73E-01	Distal Intergenic
Abhd6	305795	chr15	18677295	18677595	17538	-1.08	2.90E-02	2.73E-01	Intron (NM_001007680/305795, intron 1 of 8)
Chchd6	297436	chr4	121261990	121262290	302922	1.29	2.90E-02	2.73E-01	Distal Intergenic
Mep1b	25727	chr18	14992977	14993277	96712	-1.32	2.90E-02	2.73E-01	Intron (NM_001101000/679221, intron 3 of 6)
Rab5b	288779	chr7	3116830	3117130	6167	-1.35	2.90E-02	2.74E-01	Intron (NM_001079936/288779, intron 1 of 5)
Kif9	501059	chr8	118740138	118740438	-3795	1.92	2.90E-02	2.74E-01	Distal Intergenic
Hmga2	84017	chr7	65274625	65274925	483	0.94	2.90E-02	2.74E-01	Promoter (<=1kb)
Cdc25b	171103	chr3	123731710	123732010	171	0.58	2.90E-02	2.74E-01	Promoter (<=1kb)
Gan	307893	chr19	49695731	49696031	152	0.83	2.90E-02	2.74E-01	Promoter (<=1kb)
Fkbp5	361810	chr20	8037066	8037366	24358	-1.33	2.90E-02	2.74E-01	Intron (NM_001012174/361810, intron 1 of 10)
Mrpl35	297334	chr4	99707237	99707537	39023	-1.34	2.90E-02	2.74E-01	Intron (NM_001108633/362384, intron 5 of 6)
Runx3	156726	chr5	153419345	153419645	-87444	-1.18	2.91E-02	2.74E-01	Distal Intergenic
Xpa	298074	chr5	61792543	61792843	85	1.03	2.91E-02	2.74E-01	Promoter (<=1kb)
LOC499469	499469	chr20	46788326	46788626	80964	1.42	2.91E-02	2.74E-01	Distal Intergenic
Ifi35	287719	chr10	89361771	89362071	3395	-1.49	2.91E-02	2.74E-01	Intron (NM_001009625/287719, intron 1 of 6)
Slc1a4	305540	chr14	104600030	104600330	12087	-1.49	2.91E-02	2.74E-01	Intron (NM_198763/305540, intron 1 of 7)
Amd1	81640	chr20	45398823	45399123	571	1.09	2.91E-02	2.74E-01	Promoter (<=1kb)
Spred2	305539	chr14	104191078	104191378	-139	0.82	2.91E-02	2.74E-01	Promoter (<=1kb)
Pars2	313429	chr5	126246423	126246723	-7419	0.98	2.91E-02	2.74E-01	Distal Intergenic
Stk38l	691337	chr4	181040509	181040809	13297	-1.29	2.91E-02	2.74E-01	Intron (NM_001083336/691337, intron 1 of 14)
Mmadhc	362134	chr3	35919344	35919644	-117937	-1.36	2.91E-02	2.74E-01	Distal Intergenic
Cyp27b1	114700	chr7	70332038	70332338	-2723	-1.41	2.91E-02	2.74E-01	Promoter (2-3kb)
Gltp	288707	chr12	47667479	47667779	0	0.97	2.91E-02	2.74E-01	Promoter (<=1kb)
Ubr2	363188	chr9	16017682	16017982	14624	1.9	2.91E-02	2.74E-01	Intron (NM_001178071/363188, intron 3 of 45)
Sox4	364712	chr17	37491984	37492284	-122738	-1.29	2.91E-02	2.74E-01	Distal Intergenic
Rgs17	308118	chr1	42584425	42584725	2996	-1.37	2.91E-02	2.74E-01	Promoter (2-3kb)
Cnot8	363603	chr10	43446177	43446477	-91833	1.17	2.91E-02	2.74E-01	Distal Intergenic
Nagk	297393	chr4	115474088	115474388	56	0.67	2.91E-02	2.74E-01	Promoter (<=1kb)
Fnip2	310538	chr2	178284846	178285146	49754	-1.33	2.91E-02	2.74E-01	Intron (NM_001271167/310538, intron 1 of 16)
Adam19	303068	chr10	31146164	31146464	57	0.63	2.91E-02	2.74E-01	Promoter (<=1kb)
Cdc42ep3	313838	chr6	1930424	1930724	12248	0.64	2.91E-02	2.74E-01	Intron (NM_001048044/313838, intron 1 of 1)
Gpr180	306165	chr15	103344414	103344714	0	1.05	2.92E-02	2.74E-01	Promoter (<=1kb)
Slc12a2	83629	chr18	52917392	52917692	268	1.06	2.92E-02	2.74E-01	Promoter (<=1kb)
Hdgf	114499	chr2	187275451	187275751	331	0.81	2.92E-02	2.74E-01	Promoter (<=1kb)
Usp6nl	291309	chr17	75885983	75886283	240	0.99	2.92E-02	2.74E-01	Promoter (<=1kb)
Dtnbp1	641528	chr17	20115096	20115396	24960	-1.28	2.92E-02	2.74E-01	Intron (NM_001037664/641528, intron 5 of 8)
Phlpp1	59265	chr13	26164815	26165115	-7100	-1.25	2.92E-02	2.74E-01	Distal Intergenic
Zfp516	291406	chr18	79761043	79761343	-52416	1.13	2.92E-02	2.74E-01	Distal Intergenic
Angptl2	171100	chr3	12385850	12386150	123028	-1.04	2.93E-02	2.74E-01	Distal Intergenic
Kif1b	117548	chr5	166073271	166073571	59920	1.79	2.93E-02	2.74E-01	Intron (NM_057200/117548, intron 14 of 48)
Map3k8	116596	chr17	56109497	56109797	94	0.68	2.93E-02	2.74E-01	Promoter (<=1kb)
Clptm1	292696	chr1	80580331	80580631	34	0.69	2.93E-02	2.74E-01	Promoter (<=1kb)
Ube2q1	295252	chr2	189105948	189106248	0	1.46	2.93E-02	2.74E-01	Promoter (<=1kb)
Bmp7	85272	chr3	170770385	170770685	185135	1.15	2.93E-02	2.74E-01	Distal Intergenic
Arhgap21	307178	chr17	87658404	87658704	191025	1.5	2.93E-02	2.74E-01	Distal Intergenic
Tomm40l	304971	chr13	89593162	89593462	1144	-1.46	2.93E-02	2.74E-01	Promoter (1-2kb)
Padi6	298595	chr5	159233201	159233501	23198	1.44	2.93E-02	2.74E-01	Distal Intergenic
Trim2	361970	chr2	183089846	183090146	133673	-1.3	2.93E-02	2.74E-01	Distal Intergenic
Igsf10	310448	chr2	149612228	149612528	-48332	1.56	2.93E-02	2.75E-01	Distal Intergenic
Arl4a	29308	chr6	59930758	59931058	19528	0.73	2.93E-02	2.75E-01	Distal Intergenic
Setd5	297514	chr4	145028353	145028653	10745	-1.25	2.93E-02	2.75E-01	Intron (NM_001106614/297514, intron 1 of 23)
Mroh7	298301	chr5	126322370	126322670	1127	1.88	2.93E-02	2.75E-01	Promoter (1-2kb)
Zc3hav1	252832	chr4	65951841	65952141	109954	-1.1	2.93E-02	2.75E-01	Distal Intergenic
Bend6	363212	chr9	38147866	38148166	-149087	-1.5	2.93E-02	2.75E-01	Distal Intergenic
Plekha5	246237	chr4	174616959	174617259	11497	-1.54	2.93E-02	2.75E-01	Intron (NM_139340/246237, intron 3 of 31)
Fbxo31	498959	chr19	53625262	53625562	111	0.96	2.93E-02	2.75E-01	Promoter (<=1kb)
LOC100302372	100302372	chr13	77816322	77816622	31515	-1.41	2.93E-02	2.75E-01	Intron (NM_001162897/100302372, intron 2 of 2)
Pip5k1a	365865	chr2	196246337	196246637	24189	-1.28	2.94E-02	2.75E-01	Exon (NM_001042621/365865, exon 3 of 15)
Aig1	292486	chr1	8038550	8038850	0	0.97	2.94E-02	2.75E-01	Promoter (<=1kb)
Sgms2	310849	chr2	236517138	236517438	-36636	-1.49	2.94E-02	2.75E-01	Distal Intergenic
Me3	361602	chr1	153960605	153960905	99036	-1.22	2.94E-02	2.75E-01	Intron (NM_001108491/361602, intron 4 of 14)
Scyl3	360866	chr13	82231229	82231529	199	0.72	2.94E-02	2.75E-01	Promoter (<=1kb)
Tmem106c	315286	chr7	139444928	139445228	0	0.63	2.94E-02	2.75E-01	Promoter (<=1kb)
Lhfpl2	294643	chr2	23800745	23801045	30037	-1.26	2.94E-02	2.75E-01	Intron (NM_001106402/294643, intron 1 of 2)
Ccdc174	297458	chr4	123760877	123761177	134	0.63	2.94E-02	2.75E-01	Promoter (<=1kb)
Pdlim5	64353	chr2	247939854	247940154	48308	1.56	2.94E-02	2.75E-01	Intron (NM_053326/64353, intron 2 of 12)
Scaf11	312030	chr7	137856121	137856421	0	1.16	2.94E-02	2.75E-01	Promoter (<=1kb)
Foxp1	297480	chr4	131403893	131404193	194111	1.61	2.94E-02	2.75E-01	Intron (NM_001034131/297480, intron 7 of 15)
Micalcl	293180	chr1	177172195	177172495	-21240	1.44	2.94E-02	2.75E-01	Distal Intergenic
Pik3r1	25513	chr2	31801577	31801877	14508	-1.21	2.94E-02	2.75E-01	Intron (NM_013005/25513, intron 1 of 14)
Tax1bp1	246244	chr4	82768617	82768917	-7829	-1.46	2.94E-02	2.75E-01	Distal Intergenic
Fgf5	60662	chr14	13207359	13207659	-211775	-1.33	2.94E-02	2.75E-01	Distal Intergenic
Nrarp	499745	chr3	2394027	2394327	-1816	1.54	2.95E-02	2.75E-01	Promoter (1-2kb)
Cyp19a1	25147	chr8	58764309	58764609	7799	-1.04	2.95E-02	2.75E-01	Intron (NM_017085/25147, intron 3 of 9)
Nedd4l	291553	chr18	60543991	60544291	151615	-1.37	2.95E-02	2.75E-01	Intron (NM_001008300/291553, intron 2 of 31)
Abi1	79249	chr17	89986782	89987082	35030	-1.56	2.95E-02	2.75E-01	Intron (NM_024397/79249, intron 2 of 10)
Zdhhc13	365252	chr1	104106209	104106509	0	0.69	2.95E-02	2.75E-01	Promoter (<=1kb)
Slc29a1	63997	chr9	17768263	17768563	-15905	-1.01	2.95E-02	2.75E-01	Distal Intergenic
Prkce	29340	chr6	9482507	9482807	-601	1.21	2.95E-02	2.75E-01	Promoter (<=1kb)
Col4a1	290905	chr16	83461872	83462172	-59990	1.97	2.95E-02	2.75E-01	Distal Intergenic
Rlbp1	293049	chr1	141113045	141113345	-1670	1.84	2.95E-02	2.75E-01	Promoter (1-2kb)
Rras	361568	chr1	101012770	101013070	0	0.78	2.95E-02	2.75E-01	Promoter (<=1kb)
Srpk1	361811	chr20	5865205	5865505	349	0.87	2.95E-02	2.75E-01	Promoter (<=1kb)
Tbx5	304514	chr12	42004124	42004424	-95409	1.88	2.95E-02	2.75E-01	Distal Intergenic
Ssbp3	84354	chr5	126466443	126466743	-44607	1.82	2.95E-02	2.75E-01	Distal Intergenic
Zfp592	293038	chr1	142814852	142815152	-20066	0.69	2.95E-02	2.75E-01	Distal Intergenic
Lnpep	171105	chr1	59362918	59363218	21126	-1.34	2.95E-02	2.75E-01	Intron (NM_001113403/171105, intron 1 of 18)
Cox6c	54322	chr7	74721135	74721435	14215	1.6	2.96E-02	2.75E-01	3' UTR
Bod1	287173	chr10	16259879	16260179	148	1.06	2.96E-02	2.75E-01	Promoter (<=1kb)
Mapk1ip1l	361028	chr15	24108491	24108791	30211	-1.04	2.96E-02	2.75E-01	Distal Intergenic
Psma4	29671	chr8	59532634	59532934	178	0.56	2.96E-02	2.75E-01	Promoter (<=1kb)
Ino80c	291737	chr18	15985905	15986205	23434	0.81	2.96E-02	2.75E-01	Distal Intergenic

Smc3	29486	chr1	274282854	274283154	-26966	0.98	2.96E-02	2.75E-01	Distal Intergenic
Tagap	308097	chr1	47513777	47514077	-11757	0.82	2.96E-02	2.75E-01	Distal Intergenic
Pkn3	296619	chr3	8633048	8633348	5015	1.09	2.96E-02	2.75E-01	Intron (NM_001047861/296619, intron 4 of 25)
Gcnt1	64043	chr1	236754510	236754810	174476	-1.36	2.96E-02	2.75E-01	Distal Intergenic
LOC102550367	102550367	chr3	120930938	120931238	151998	-1.32	2.96E-02	2.75E-01	Distal Intergenic
Zfp653	300446	chr8	23081436	23081736	3143	1.82	2.96E-02	2.75E-01	Exon (NM_001106807/300446, exon 2 of 9)
Plin2	298199	chr5	105100293	105100593	-89494	0.59	2.96E-02	2.75E-01	Distal Intergenic
Tmem158	117582	chr8	132345158	132345458	250	1.07	2.96E-02	2.75E-01	Promoter (<=1kb)
Supt6h	303281	chr10	65523170	65523470	-15589	0.81	2.97E-02	2.75E-01	Distal Intergenic
LOC308990	308990	chr1	198529177	198529477	542	-0.99	2.97E-02	2.75E-01	Promoter (<=1kb)
Aass	296925	chr4	50208873	50209173	0	0.85	2.97E-02	2.75E-01	Promoter (<=1kb)
Ppp1r27	287881	chr10	109727161	109727461	1558	0.86	2.97E-02	2.75E-01	Promoter (1-2kb)
Cycs	25309	chr4	80207171	80207471	125855	-1.44	2.97E-02	2.75E-01	Distal Intergenic
Dpf3	299186	chr6	106970645	106970945	305	0.78	2.97E-02	2.76E-01	Promoter (<=1kb)
Large1	361368	chr19	12909918	12910218	32725	1.23	2.97E-02	2.76E-01	Intron (NM_001108439/361368, intron 1 of 12)
LOC100361083	100361083	chr5	141172728	141173028	-71765	-1.7	2.97E-02	2.76E-01	Exon (NM_001135758/362587, exon 29 of 98)
Marchf2	362849	chr7	18585045	18585345	0	0.83	2.97E-02	2.76E-01	Promoter (<=1kb)
Kpna5	294392	chr20	32514705	32515005	1032	-1.45	2.97E-02	2.76E-01	Promoter (1-2kb)
Rps3a	29288	chr2	185268320	185268620	176226	1.2	2.97E-02	2.76E-01	Distal Intergenic
Plekhd1	500685	chr6	104444706	104445006	104592	-1.38	2.97E-02	2.76E-01	Distal Intergenic
Col5a1	85490	chr3	6478486	6478786	47895	1.59	2.98E-02	2.76E-01	Intron (NM_134452/85490, intron 4 of 65)
Epn2	60443	chr10	47845599	47845899	11451	-1.8	2.98E-02	2.76E-01	Intron (NM_001033914/60443, intron 1 of 9)
Itga8	364786	chr17	79652330	79652630	23869	-1.14	2.98E-02	2.76E-01	Intron (NM_001173972/364786, intron 2 of 29)
Lpin3	362261	chr3	156858368	156858668	-27786	-1.18	2.98E-02	2.76E-01	Distal Intergenic
Thegl	498350	chr14	33420055	33420355	10601	-1.28	2.98E-02	2.76E-01	Intron (NM_001017498/498350, intron 1 of 8)
Fzd1	58868	chr4	26721163	26721463	250299	1.75	2.98E-02	2.76E-01	Distal Intergenic
Hexb	294673	chr2	27998404	27998704	4556	-1.22	2.98E-02	2.76E-01	Intron (NM_001003401/294674, intron 1 of 3)
Ptgs1	24693	chr3	15606036	15606336	45313	1.44	2.98E-02	2.76E-01	Distal Intergenic
Neurod6	500137	chr4	85891366	85891666	23441	-1.28	2.98E-02	2.76E-01	Distal Intergenic
Rnaset2	292306	chr1	53174906	53175206	27	0.68	2.98E-02	2.76E-01	Promoter (<=1kb)
Sgk2	171497	chr3	159374019	159374319	12706	1.26	2.98E-02	2.76E-01	Exon (NM_134463/171497, exon 10 of 15)
Fam135a	367235	chr9	30161188	30161488	172488	1.06	2.98E-02	2.76E-01	Distal Intergenic
Ankrd55	361898	chr2	44148963	44149263	22145	1.6	2.98E-02	2.76E-01	Intron (NM_001270039/361898, intron 4 of 9)
R3hdm1	304763	chr13	44812024	44812324	-243	0.69	2.98E-02	2.76E-01	Promoter (<=1kb)
Rsph6a	292684	chr1	79981428	79981728	21837	1.38	2.98E-02	2.76E-01	Distal Intergenic
Cebpb	24253	chr3	164425448	164425748	946	1.33	2.98E-02	2.76E-01	Promoter (<=1kb)
Atp2b1	29598	chr7	41114963	41115263	266	1.18	2.99E-02	2.76E-01	Promoter (<=1kb)
Mir210	100314053	chr1	214209241	214209541	-777	1.42	2.99E-02	2.76E-01	Promoter (<=1kb)
Ehbp1	305556	chr14	107083190	107083490	41892	2.12	2.99E-02	2.76E-01	Intron (NM_001305130/305556, intron 4 of 24)
Crhr1	58959	chr10	92206803	92207103	15330	0.73	2.99E-02	2.76E-01	Intron (NM_001301812/58959, intron 1 of 12)
RGD1562146	500612	chr6	9643010	9643310	116858	1.87	2.99E-02	2.76E-01	Intron (NM_017171/29340, intron 1 of 13)
Naa30	498489	chr15	26038975	26039275	5184	-1.37	2.99E-02	2.76E-01	Intron (NM_001109099/498489, intron 1 of 3)
Tek	89804	chr5	113725899	113726199	182	0.84	2.99E-02	2.76E-01	Promoter (<=1kb)
Cdk8	498140	chr12	10561053	10561353	0	0.79	2.99E-02	2.76E-01	Promoter (<=1kb)
Ptgs1	24693	chr3	15589239	15589539	28516	-1.66	2.99E-02	2.76E-01	Distal Intergenic
Dmac1	298147	chr5	93376887	93377187	-1337741	1.76	2.99E-02	2.76E-01	Distal Intergenic
Hhex	79237	chr1	256081910	256082210	-19784	1.54	2.99E-02	2.76E-01	Intron (NM_001169112/171304, intron 19 of 21)
Foxp1	297480	chr4	131762863	131763163	-164559	-1.19	3.00E-02	2.76E-01	Distal Intergenic
Slc25a4	85333	chr16	49173930	49174230	-92673	-1.25	3.00E-02	2.76E-01	Distal Intergenic
Ptpn2	117063	chr18	63475470	63475770	12257	-1.44	3.00E-02	2.76E-01	Intron (NM_053990/117063, intron 1 of 9)
Zfp622	294846	chr2	78228622	78228922	-33917	1.87	3.00E-02	2.77E-01	Intron (NM_001034912/619558, intron 3 of 8)
Dot1l	362831	chr7	11824220	11824520	222	1.33	3.00E-02	2.77E-01	Promoter (<=1kb)
Id3	25585	chr5	154524774	154525074	35171	-1.21	3.00E-02	2.77E-01	Distal Intergenic
Slc7a14	499587	chr2	115742321	115742621	63977	0.61	3.00E-02	2.77E-01	Intron (NM_001134615/499587, intron 2 of 7)
Kif13b	305967	chr15	48090136	48090436	59062	1.77	3.00E-02	2.77E-01	Intron (NM_213626/305967, intron 3 of 37)
Marchf8	312656	chr4	148290978	148291278	4124	-1.5	3.01E-02	2.77E-01	Intron (NM_001107882/312656, intron 2 of 7)
Cdk17	314743	chr7	34001747	34002047	241	1.06	3.01E-02	2.77E-01	Promoter (<=1kb)
Azin1	58961	chr7	77371690	77371990	408	1.2	3.01E-02	2.77E-01	Promoter (<=1kb)
Map3k10	308463	chr1	84523842	84524142	-32363	1.87	3.01E-02	2.77E-01	Distal Intergenic
Fas	246097	chr1	252581046	252581346	-8439	1.87	3.01E-02	2.77E-01	Distal Intergenic
Myo18a	360570	chr10	65080006	65080306	229	0.78	3.01E-02	2.77E-01	Promoter (<=1kb)
RGD1561161	294747	chr2	45279732	45280032	97341	-1.67	3.01E-02	2.77E-01	Distal Intergenic
Cxcl1	81503	chr14	18750394	18750694	-4937	-1.38	3.01E-02	2.77E-01	Distal Intergenic
Sertad2	498423	chr14	104857562	104857862	36347	-0.88	3.01E-02	2.77E-01	Intron (NM_001024903/498423, intron 1 of 1)
Bdkrb1	81509	chr6	129427690	129427990	-10168	-1.44	3.01E-02	2.77E-01	3' UTR
Klf6	58954	chr17	67939375	67939675	-34457	-1.44	3.01E-02	2.77E-01	Distal Intergenic
RGD1565033	498014	chr10	95318804	95319104	75818	-1.21	3.01E-02	2.77E-01	Distal Intergenic
Wnt11	140584	chr1	163797484	163797784	0	1.14	3.01E-02	2.77E-01	Promoter (<=1kb)
Pgm2	289632	chr14	45759031	45759331	104541	1.79	3.01E-02	2.77E-01	Distal Intergenic
Plcg1	25738	chr3	156727619	156727919	0	0.95	3.02E-02	2.77E-01	Promoter (<=1kb)
Lrrc15	246296	chr11	74041104	74041404	-8762	-1.34	3.02E-02	2.77E-01	Distal Intergenic
Hgf	24446	chr4	15344919	15345219	160064	1.61	3.02E-02	2.77E-01	Distal Intergenic
Gpr20	60667	chr7	114805358	114805658	139686	-1.22	3.02E-02	2.77E-01	Distal Intergenic
Me1	24552	chr8	94368396	94368696	138	0.8	3.02E-02	2.77E-01	Promoter (<=1kb)
Hsph1	288444	chr12	6512227	6512527	-170329	1.1	3.02E-02	2.77E-01	Distal Intergenic
Pla2g12a	362039	chr2	235314493	235314793	2774	-1.7	3.02E-02	2.77E-01	Promoter (2-3kb)
Rasa1	25676	chr2	13696251	13696551	0	0.97	3.02E-02	2.77E-01	Promoter (<=1kb)
Hnf1b	25640	chr10	71205836	71206136	45973	1.98	3.02E-02	2.77E-01	Intron (NM_001308148/25640, intron 7 of 9)
Scaf8	245926	chr1	44124851	44125151	105792	-1.64	3.02E-02	2.77E-01	Distal Intergenic
Hist1h2ail1	291159	chr17	43846001	43846301	-30818	-1.21	3.02E-02	2.77E-01	Distal Intergenic
Irs2	29376	chr16	83821624	83821924	-2591	1.71	3.02E-02	2.77E-01	Promoter (2-3kb)
Rac1	363875	chr12	13101629	13101929	11257	-1.44	3.02E-02	2.77E-01	Intron (NM_134366/363875, intron 2 of 5)
Coq7	25249	chr1	188192677	188192977	-1817	1.81	3.02E-02	2.77E-01	Promoter (1-2kb)
Wiz	314598	chr7	14402163	14402463	-153	0.83	3.02E-02	2.77E-01	Promoter (<=1kb)
Mir28	100314152	chr11	79134086	79134386	246222	-0.64	3.02E-02	2.77E-01	Distal Intergenic
Ndc80	301701	chr9	119831132	119831432	24715	-1.32	3.02E-02	2.77E-01	Distal Intergenic
Slc35e3	362883	chr7	60777811	60778111	25	0.92	3.02E-02	2.77E-01	Promoter (<=1kb)
Kcnj5	29713	chr8	33353408	33353708	109702	-1.22	3.02E-02	2.77E-01	Distal Intergenic
Ap5z1	641386	chr12	14197138	14197438	-21141	-1.28	3.02E-02	2.77E-01	Intron (NM_001037219/304298, intron 2 of 8)
Rnf115	362002	chr2	198905460	198905760	53047	-1.47	3.02E-02	2.77E-01	Intron (NM_001108560/362002, intron 3 of 8)
Fktn	362520	chr5	70441240	70441540	-80461	0.82	3.02E-02	2.77E-01	Distal Intergenic
Nicn1	619581	chr8	117038716	117039016	-23973	1.37	3.03E-02	2.77E-01	Distal Intergenic
Mir193	100314244	chr10	67059848	67060148	-5748	1.86	3.03E-02	2.77E-01	Distal Intergenic
Crtap	363158	chr8	122331428	122331728	70470	1.09	3.03E-02	2.77E-01	Distal Intergenic

Sox4	364712	chr17	37697002	37697302	81980	-1.31	3.03E-02	2.77E-01	Distal Intergenic
Lhfpl6	499615	chr2	142411229	142411529	148993	1.74	3.03E-02	2.77E-01	Intron (NM_001109183/499615, intron 2 of 3)
Hnrnpm	116655	chr7	18553858	18554158	90	0.62	3.03E-02	2.77E-01	Promoter (<=1kb)
Tmem18	362722	chr6	49529214	49529514	0	0.62	3.03E-02	2.77E-01	Promoter (<=1kb)
Coro6	245982	chr10	62721066	62721366	-22525	0.79	3.03E-02	2.77E-01	Exon (NM_001107024/303342, exon 12 of 14)
Mical2	365352	chr1	177103346	177103646	9959	-1.49	3.03E-02	2.77E-01	Intron (NM_001139508/365352, intron 1 of 17)
Taok3	304530	chr12	45199525	45199825	-16551	1.2	3.03E-02	2.77E-01	Distal Intergenic
Esr1	24890	chr1	41655664	41655964	332460	-1.54	3.03E-02	2.77E-01	Distal Intergenic
Dnajb6	362293	chr4	2842287	2842587	130902	1.18	3.03E-02	2.77E-01	Distal Intergenic
Prph	24688	chr7	140742759	140743059	341	1.09	3.03E-02	2.77E-01	Promoter (<=1kb)
Kalrn	84009	chr11	69464631	69464931	-19362	-1.46	3.03E-02	2.77E-01	Distal Intergenic
Prpf4b	291078	chr17	30714071	30714371	39	0.93	3.03E-02	2.77E-01	Promoter (<=1kb)
Cavin3	85332	chr1	170311605	170311905	6783	-1.62	3.03E-02	2.77E-01	Distal Intergenic
Gpr88	64443	chr2	219217573	219217873	45028	-1.46	3.04E-02	2.77E-01	Distal Intergenic
Zmym4	313598	chr5	144995895	144996195	236	1.28	3.04E-02	2.77E-01	Promoter (<=1kb)
Fa2h	307855	chr19	43713942	43714242	-117154	0.59	3.04E-02	2.77E-01	Distal Intergenic
Dtnbp1	641528	chr17	20348027	20348327	257891	1.81	3.04E-02	2.77E-01	Distal Intergenic
Rasa2	25597	chr8	104540100	104540400	1913	-1.37	3.04E-02	2.77E-01	Promoter (1-2kb)
Capn5	171495	chr1	163119666	163119966	9770	-1.3	3.04E-02	2.77E-01	Intron (NM_134461/171495, intron 1 of 12)
Abcf2	311959	chr4	7123007	7123307	91	0.62	3.04E-02	2.77E-01	Promoter (<=1kb)
Odc1	24609	chr6	42852791	42853091	132	0.64	3.04E-02	2.77E-01	Promoter (<=1kb)
Spen	690911	chr5	160051199	160051499	19416	-0.99	3.04E-02	2.77E-01	Intron (NM_001271495/690911, intron 2 of 14)
Cct6a	288620	chr12	30507411	30507711	-5598	1.53	3.04E-02	2.77E-01	Distal Intergenic
Plekha3	295674	chr3	63536339	63536639	173	1.08	3.04E-02	2.77E-01	Promoter (<=1kb)
Man2a2	308757	chr1	142164006	142164306	0	1	3.04E-02	2.77E-01	Promoter (<=1kb)
Runx3	156726	chr5	153447249	153447549	-59540	1.27	3.04E-02	2.77E-01	Distal Intergenic
Eea1	314764	chr7	37089607	37089907	-11507	-1.24	3.04E-02	2.77E-01	Distal Intergenic
LOC103695172	103695172	chr3	59687000	59687300	-130633	0.79	3.05E-02	2.77E-01	Distal Intergenic
Tle3	84424	chr8	66495945	66496245	199062	1.66	3.05E-02	2.77E-01	Distal Intergenic
Prnd	113910	chr3	124547781	124548081	2417	-1.38	3.05E-02	2.77E-01	Promoter (2-3kb)
Rnf144b	364681	chr17	17848616	17848916	98861	-1.46	3.05E-02	2.78E-01	Intron (NM_001108881/364681, intron 2 of 7)
Col5a2	85250	chr9	52222981	52223281	15454	-1.46	3.05E-02	2.78E-01	Intron (NM_053488/85250, intron 1 of 55)
Mir532	100314265	chrX	15986326	15986626	-123244	1.58	3.05E-02	2.78E-01	Distal Intergenic
Snx16	64088	chr2	93560037	93560337	41228	-1.32	3.05E-02	2.78E-01	Distal Intergenic
Cavin1	287710	chr10	88883899	88884199	-9404	1.99	3.05E-02	2.78E-01	Distal Intergenic
Rpl7a	296596	chr18	4597470	4597770	-225571	-1.49	3.05E-02	2.78E-01	Distal Intergenic
Echs1	140547	chr1	141767470	141767770	-54170	0.8	3.05E-02	2.78E-01	Distal Intergenic
Pard6b	362279	chr3	164848892	164849192	26781	1.64	3.05E-02	2.78E-01	Distal Intergenic
Tent5a	300870	chr8	93055729	93056029	-113504	1.37	3.06E-02	2.78E-01	Distal Intergenic
Nub1	296731	chr4	6981079	6981379	21	0.76	3.06E-02	2.78E-01	Promoter (<=1kb)
Zc3h10	685928	chr7	2970755	2971055	1466	-0.99	3.06E-02	2.78E-01	Promoter (1-2kb)
Mir3565	100526634	chr14	66650362	66650662	-272421	1.62	3.06E-02	2.78E-01	Intron (NM_181365/259243, intron 3 of 8)
Epgn	289515	chr14	18631438	18631738	3397	-1.28	3.06E-02	2.78E-01	Intron (NM_001305170/289515, intron 2 of 4)
Gpr107	311857	chr3	10689952	10690252	4402	-1.49	3.06E-02	2.78E-01	Intron (NM_001107828/311857, intron 1 of 17)
Ppm1h	314897	chr7	67116862	67117162	0	1.15	3.06E-02	2.78E-01	Promoter (<=1kb)
Cplx2	116657	chr17	10782796	10783096	-16543	-1.34	3.06E-02	2.78E-01	Distal Intergenic
Mir671	100314113	chr4	7098107	7098407	10420	0.61	3.06E-02	2.78E-01	Intron (NM_001011966/296732, intron 2 of 13)
Stx18	360953	chr14	77476859	77477159	15751	-1.4	3.06E-02	2.78E-01	Intron (NM_001012151/360953, intron 1 of 10)
Cryba1	25583	chr10	65199085	65199385	37933	1.14	3.06E-02	2.78E-01	Distal Intergenic
Tmcc2	305095	chr13	49130973	49131273	38498	1.05	3.06E-02	2.78E-01	Downstream (1-2kb)
Map10	307948	chr19	58534841	58535141	29030	1.7	3.06E-02	2.78E-01	Distal Intergenic
Ndufb9	299954	chr7	98879268	98879568	66206	0.76	3.06E-02	2.78E-01	Intron (NM_001130563/362918, intron 4 of 15)
Itgb1bp1	298914	chr6	43354925	43355225	8110	1.17	3.06E-02	2.78E-01	Intron (NM_001106719/298914, intron 3 of 7)
Nr3c1	24413	chr18	32672510	32672810	-1832	0.81	3.06E-02	2.78E-01	Promoter (1-2kb)
Ccdc39	310315	chr2	120407988	120408288	-91631	-1.45	3.06E-02	2.78E-01	Distal Intergenic
Atg10	688555	chr2	20418474	20418774	-265590	1.12	3.06E-02	2.78E-01	Distal Intergenic
Taco1	360645	chr10	94264071	94264371	3923	-1.53	3.07E-02	2.78E-01	Intron (NM_001108302/360645, intron 2 of 7)
Flnb	306204	chr15	18812585	18812885	70074	1.61	3.07E-02	2.78E-01	Intron (NM_001107288/306204, intron 8 of 44)
Slc26a5	83819	chr4	9838813	9839113	17272	-1.4	3.07E-02	2.78E-01	Intron (NM_030840/83819, intron 7 of 17)
Pkn3	296619	chr3	8625423	8625723	-2310	1.76	3.07E-02	2.78E-01	Promoter (2-3kb)
Cxcl12	24772	chr4	149449252	149449552	188208	-1.33	3.07E-02	2.78E-01	Distal Intergenic
Creb5	500131	chr4	83137465	83137765	-328730	1.08	3.07E-02	2.78E-01	Distal Intergenic
Armc12	294301	chr20	8096119	8096419	-13272	-1.28	3.07E-02	2.78E-01	Distal Intergenic
Lmbr1l	300215	chr7	140600383	140600683	60	0.5	3.07E-02	2.78E-01	Promoter (<=1kb)
Srpx2	317181	chrX	104734510	104734810	142	-0.79	3.07E-02	2.78E-01	Promoter (<=1kb)
Fam221b	298398	chr5	59165568	59165868	-470	0.85	3.07E-02	2.78E-01	Promoter (<=1kb)
Acot11	100363074	chr5	126444469	126444769	-48814	1.4	3.07E-02	2.78E-01	Distal Intergenic
Rac1	363875	chr12	13104829	13105129	14457	-1.18	3.08E-02	2.78E-01	Intron (NM_134366/363875, intron 3 of 5)
Kcmf1	684322	chr4	100817686	100817986	-34669	-1.21	3.08E-02	2.78E-01	Distal Intergenic
Ptprm	29616	chr9	115265197	115265497	289764	0.59	3.08E-02	2.78E-01	Intron (NM_001168632/29616, intron 4 of 32)
Wwp2	291999	chr19	39549215	39549515	5967	-0.87	3.08E-02	2.78E-01	Intron (NM_001106184/291999, intron 2 of 19)
Ntn4	299737	chr7	34554029	34554329	20486	1.53	3.08E-02	2.78E-01	Intron (NM_001106780/299737, intron 2 of 11)
Dgke	497978	chr10	76530655	76530955	-122666	-1.18	3.08E-02	2.78E-01	Distal Intergenic
Mkln1	83536	chr4	58697963	58698263	4579	-1.56	3.08E-02	2.78E-01	Intron (NM_031359/83536, intron 1 of 24)
Dyrk2	314862	chr7	61799197	61799497	-468	1.1	3.08E-02	2.78E-01	Promoter (<=1kb)
Cxcl1	81503	chr14	18753443	18753743	-7986	-0.87	3.08E-02	2.78E-01	Distal Intergenic
Chmp6	287873	chr10	109086795	109087095	13117	-1.38	3.08E-02	2.78E-01	Distal Intergenic
Fnbp1l	310839	chr2	226761611	226761911	17355	-1.42	3.08E-02	2.78E-01	Intron (NM_001039609/310839, intron 1 of 15)
Nckap1	58823	chr3	67804296	67804596	84	1.42	3.08E-02	2.78E-01	Promoter (<=1kb)
Smap2	298500	chr5	139930891	139931191	175	0.95	3.08E-02	2.78E-01	Promoter (<=1kb)
Srgap1	314903	chr7	64251249	64251549	-139	1.5	3.08E-02	2.78E-01	Promoter (<=1kb)
Naa50	288108	chr11	61519060	61519360	11470	-1.49	3.08E-02	2.78E-01	Intron (NM_001105881/288108, intron 1 of 4)
Stk38	361813	chr20	6257209	6257509	-26013	0.73	3.08E-02	2.78E-01	Distal Intergenic
Trmt5	362754	chr6	96151774	96152074	19555	-1.63	3.08E-02	2.78E-01	Intron (NM_153472/266713, intron 7 of 7)
Zfp800	500057	chr4	55526693	55526993	-618	1.06	3.08E-02	2.78E-01	Promoter (<=1kb)
Ociad1	289590	chr14	37537913	37538213	-62495	-0.85	3.08E-02	2.78E-01	Distal Intergenic
Maml3	310405	chr2	141081985	141082285	194601	-1.23	3.08E-02	2.78E-01	Intron (NM_001107675/310405, intron 1 of 5)
Gsk3a	50686	chr1	82107776	82108076	127	1.24	3.09E-02	2.78E-01	Promoter (<=1kb)
Tph1	24848	chr1	102634885	102635185	64257	-1.67	3.09E-02	2.78E-01	Distal Intergenic
Plpp4	309014	chr1	202966771	202967071	534405	1.78	3.09E-02	2.78E-01	Distal Intergenic
Tent5b	313019	chr5	151659067	151659367	-32701	-1.04	3.09E-02	2.78E-01	Distal Intergenic
Dpp8	315758	chr8	70591480	70591780	69383	0.85	3.09E-02	2.78E-01	Distal Intergenic
Tead1	361630	chr1	177511703	177512003	15921	-1.3	3.09E-02	2.78E-01	Intron (NM_001198589/361630, intron 1 of 12)
Txlna	682457	chr5	147842440	147842740	3355	-1.38	3.09E-02	2.78E-01	Intron (NM_001127633/682457, intron 3 of 10)

Myc	24577	chr7	102672548	102672848	86235	-1.43	3.09E-02	2.78E-01	Distal Intergenic
Zfp362	297879	chr5	147019822	147020122	-45890	1.62	3.09E-02	2.78E-01	Distal Intergenic
Thoc5	360972	chr14	85141192	85141492	-787	-1.37	3.09E-02	2.78E-01	Promoter (<=1kb)
Amz1	304317	chr12	16030796	16031096	-33857	0.68	3.09E-02	2.78E-01	Distal Intergenic
Cfdp1	292027	chr19	44057168	44057468	20852	-1.03	3.09E-02	2.78E-01	Intron (NM_199378/292027, intron 4 of 6)
Cd44	25406	chr3	92763518	92763818	19840	-1.39	3.09E-02	2.78E-01	Intron (NM_012924/25406, intron 1 of 19)
Ampd3	25095	chr1	175605465	175605765	19368	-1.46	3.09E-02	2.78E-01	Intron (NM_031544/25095, intron 2 of 14)
Nr5a2	60349	chr13	54036125	54036425	-166009	1.73	3.09E-02	2.78E-01	Distal Intergenic
Otud7b	310677	chr2	198256935	198257235	25582	1.63	3.09E-02	2.78E-01	Intron (NM_001107697/310677, intron 1 of 11)
Prex1	311647	chr3	163247097	163247397	230425	1.88	3.10E-02	2.78E-01	Distal Intergenic
Sh2b2	114203	chr12	23186571	23186871	-286399	-1.43	3.10E-02	2.79E-01	Distal Intergenic
Hsd11b1	25116	chr13	111958484	111958784	13819	-1.29	3.10E-02	2.79E-01	Intron (NM_017080/25116, intron 4 of 5)
Gch1	29244	chr15	23970339	23970639	-1368	1.8	3.10E-02	2.79E-01	Promoter (1-2kb)
Enthd1	685900	chr7	121703465	121703765	79670	-1.36	3.10E-02	2.79E-01	Intron (NM_001135913/685900, intron 5 of 6)
Npepo	290963	chr17	553726	554026	-4158	1.92	3.10E-02	2.79E-01	Distal Intergenic
LOC288913	288913	chr19	25358282	25358582	12	0.63	3.10E-02	2.79E-01	Promoter (<=1kb)
Amz1	304317	chr12	15957855	15958155	38784	-1.17	3.10E-02	2.79E-01	Intron (NM_031034/81663, intron 2 of 3)
Vom1r9	100312665	chr1	60105812	60106112	-142583	1.65	3.10E-02	2.79E-01	Distal Intergenic
Adam11	360638	chr10	90731029	90731329	-536	1.88	3.10E-02	2.79E-01	Promoter (<=1kb)
LOC102550367	102550367	chr3	120994573	120994873	88363	-0.82	3.10E-02	2.79E-01	Distal Intergenic
Sox4	364712	chr17	37615993	37616293	971	0.91	3.10E-02	2.79E-01	Promoter (<=1kb)
Sh2b2	114203	chr12	23229368	23229668	-243602	-1.38	3.10E-02	2.79E-01	Distal Intergenic
Maml3	310405	chr2	141007011	141007311	269575	-1.27	3.10E-02	2.79E-01	Intron (NM_001107675/310405, intron 2 of 5)
Il2	116562	chr2	123898444	123898744	-46590	-1.36	3.10E-02	2.79E-01	Distal Intergenic
Rit1	499652	chr2	188068556	188068856	-18630	1.89	3.10E-02	2.79E-01	Distal Intergenic
Tbc1d2	313234	chr5	62238237	62238537	21552	-1.66	3.10E-02	2.79E-01	Intron (NM_001107933/313234, intron 3 of 10)
Osbp	365410	chr1	228395649	228395949	91	0.9	3.10E-02	2.79E-01	Promoter (<=1kb)
Itga10	310683	chr2	198774043	198774343	1106	-1.4	3.10E-02	2.79E-01	Promoter (1-2kb)
Tenm3	306451	chr16	46622515	46622815	198646	-1.21	3.11E-02	2.79E-01	Intron (NM_001169133/306451, intron 2 of 28)
Sptbn1	305614	chr14	114694937	114695237	-2173	-1.42	3.11E-02	2.79E-01	Promoter (2-3kb)
Ptk7	301242	chr9	16662937	16663237	303	0.76	3.11E-02	2.79E-01	Promoter (<=1kb)
Hip1r	81917	chr12	38052683	38052983	102	1.42	3.11E-02	2.79E-01	Promoter (<=1kb)
M6pr	312689	chr4	155084598	155084898	0	0.74	3.11E-02	2.79E-01	Promoter (<=1kb)
Lrrc51	293156	chr1	167029388	167029688	-12196	-1.4	3.11E-02	2.79E-01	Distal Intergenic
Eqtn	500502	chr5	113938715	113939015	-57804	0.68	3.11E-02	2.79E-01	Distal Intergenic
Hic1	303310	chr10	61959150	61959450	51324	1.57	3.11E-02	2.79E-01	Intron (NM_001105808/287522, intron 13 of 18)
Wdr60	314523	chr6	144161712	144162012	-38116	-1.53	3.11E-02	2.79E-01	Distal Intergenic
Mkln1	83536	chr4	58742948	58743248	49564	-1.34	3.11E-02	2.79E-01	Intron (NM_031359/83536, intron 8 of 24)
Rps6ka1	81771	chr5	152122389	152122689	0	1.42	3.12E-02	2.79E-01	Promoter (<=1kb)
Lars2	363172	chr8	132461660	132461960	20346	1.65	3.12E-02	2.79E-01	Intron (NM_001108787/363172, intron 3 of 18)
Grip1	84016	chr7	64716510	64716810	43789	-1.19	3.12E-02	2.79E-01	Intron (NM_032069/84016, intron 4 of 22)
Mras	25482	chr8	107698959	107699259	-42108	-1.54	3.12E-02	2.79E-01	Distal Intergenic
Glud1	24399	chr16	10678646	10678946	16735	1.8	3.12E-02	2.79E-01	Intron (NM_012570/24399, intron 4 of 12)
Adam19	303068	chr10	31180401	31180701	34294	-1.12	3.12E-02	2.79E-01	Intron (NM_001160228/303068, intron 3 of 22)
Nop10	691534	chr3	103747761	103748061	107	0.58	3.12E-02	2.79E-01	Promoter (<=1kb)
Acap3	313772	chr5	173340346	173340646	286	0.78	3.12E-02	2.79E-01	Promoter (<=1kb)
Akap2	298024	chr5	74939731	74940031	-2060	-1.36	3.12E-02	2.79E-01	Promoter (2-3kb)
Cspg4	81651	chr8	61533213	61533513	748	0.82	3.12E-02	2.79E-01	Promoter (<=1kb)
Srcin1	56029	chr10	85479914	85480214	37450	0.73	3.12E-02	2.79E-01	Exon (NM_019378/56029, exon 4 of 18)
Fndc3b	294925	chr2	113389167	113389467	-43616	-1.45	3.12E-02	2.79E-01	Distal Intergenic
Spata25	499943	chr3	161286837	161287137	6988	1.91	3.13E-02	2.79E-01	Exon (NM_001107801/311630, exon 2 of 2)
Depdc1	295538	chr2	264707730	264708030	2979	1.64	3.13E-02	2.80E-01	Promoter (2-3kb)
Lhfpl2	294643	chr2	23705606	23705906	-64802	1.15	3.13E-02	2.80E-01	Distal Intergenic
Flt3	140635	chr12	9331136	9331436	-29003	1.45	3.13E-02	2.80E-01	Distal Intergenic
Jakmip2	307479	chr18	38033263	38033563	54793	-1.22	3.13E-02	2.80E-01	Intron (NM_001107391/307479, intron 1 of 25)
Cgrrf1	116679	chr15	23622512	23622812	3336	-1.3	3.13E-02	2.80E-01	Intron (NM_053899/116679, intron 1 of 5)
Tnfaip8	307428	chr18	44752222	44752522	15068	-1.16	3.13E-02	2.80E-01	Intron (NM_001107387/307428, intron 3 of 3)
Nceh1	294930	chr2	112897465	112897765	28631	-1.3	3.13E-02	2.80E-01	Intron (NM_001127524/294930, intron 1 of 4)
Cuedc1	303419	chr10	75354559	75354859	-6286	0.67	3.13E-02	2.80E-01	Distal Intergenic
Psme4	498433	chr14	115166254	115166554	89	1.21	3.14E-02	2.80E-01	Promoter (<=1kb)
Rnf111	300813	chr8	76939781	76940081	115	1.21	3.14E-02	2.80E-01	Promoter (<=1kb)
Cyth3	116693	chr12	12959091	12959391	-51319	-0.72	3.14E-02	2.80E-01	Distal Intergenic
Creb5	500131	chr4	83714006	83714306	247511	-1.45	3.14E-02	2.80E-01	Intron (NM_001134621/500131, intron 6 of 11)
Trim29	300656	chr8	47648338	47648638	-25683	-1.29	3.14E-02	2.80E-01	Distal Intergenic
Apol9a	503164	chr7	118933057	118933357	421	-0.77	3.14E-02	2.80E-01	Promoter (<=1kb)
Thap1	306547	chr16	70636318	70636618	29213	-1.71	3.14E-02	2.80E-01	Distal Intergenic
Tnfsf8	683163	chr5	79649124	79649424	41491	-1.71	3.14E-02	2.80E-01	Distal Intergenic
Sdcbp	83841	chr5	19467489	19467789	-3875	1.83	3.14E-02	2.80E-01	Distal Intergenic
Acpp	56780	chr8	112799828	112800128	83894	-1.31	3.14E-02	2.80E-01	Distal Intergenic
C1qtnf7	305423	chr14	72269593	72269893	-244456	-0.84	3.14E-02	2.80E-01	Distal Intergenic
Agap1	316611	chr9	96621782	96622082	-124358	-1.22	3.15E-02	2.80E-01	Distal Intergenic
Gpsm1	246254	chr3	3760398	3760698	-6696	1.87	3.15E-02	2.80E-01	Distal Intergenic
Irs3	84021	chr12	22117195	22117495	-5458	1.56	3.15E-02	2.80E-01	3' UTR
Spata31d1	498693	chr17	5373010	5373310	52023	1.25	3.15E-02	2.80E-01	Distal Intergenic
Tmem178a	362691	chr6	3614341	3614641	-42714	-1.15	3.15E-02	2.80E-01	Distal Intergenic
Samhd1	311580	chr3	153192636	153192936	57705	1.39	3.15E-02	2.80E-01	Distal Intergenic
Asap1	314961	chr7	104984884	104985184	-33794	0.98	3.15E-02	2.80E-01	Distal Intergenic
Nrp1	246331	chr19	61196636	61196936	-135415	1.47	3.15E-02	2.80E-01	Distal Intergenic
Arhgap22	306279	chr16	9493710	9494010	-69208	-1.35	3.15E-02	2.80E-01	Distal Intergenic
Gnb1	24400	chr5	172934808	172935108	0	-1.21	3.15E-02	2.80E-01	Promoter (<=1kb)
Tmie	501061	chr8	119163342	119163642	-6271	1.32	3.15E-02	2.80E-01	Distal Intergenic
Tnfrsf1b	156767	chr5	163143615	163143915	23384	-1.02	3.15E-02	2.80E-01	Intron (NM_130426/156767, intron 8 of 9)
Mettl14	295428	chr2	227521442	227521742	60852	1.77	3.15E-02	2.80E-01	Intron (NM_001309453/310843, intron 10 of 22)
Mvb12b	362118	chr3	12944103	12944403	139	1.09	3.15E-02	2.80E-01	Promoter (<=1kb)
Fgfr2	25022	chr1	200446838	200447138	249790	2.01	3.15E-02	2.80E-01	Distal Intergenic
Slc6a6	29464	chr4	123690749	123691049	22270	-1.22	3.15E-02	2.80E-01	Intron (NM_017206/29464, intron 2 of 14)
Bnip3l	140923	chr15	43678774	43679074	-11745	1.78	3.16E-02	2.80E-01	Intron (NM_053999/117104, intron 8 of 9)
Tead1	361630	chr1	177445240	177445540	-50242	-1.43	3.16E-02	2.80E-01	Distal Intergenic
Steap2	312052	chr4	25507213	25507513	0	0.79	3.16E-02	2.80E-01	Promoter (<=1kb)
Nfix	81524	chr19	25929469	25929769	-14773	0.67	3.16E-02	2.80E-01	Distal Intergenic
Trim8	688785	chr1	266256301	266256601	504	1.2	3.16E-02	2.80E-01	Promoter (<=1kb)
Larp7	686883	chr2	231479447	231479747	402255	0.9	3.16E-02	2.80E-01	Distal Intergenic
Insr	24954	chr12	1673598	1673898	142516	1.46	3.16E-02	2.80E-01	Distal Intergenic
Fam107b	498796	chr17	78544150	78544450	17163	-1.59	3.16E-02	2.80E-01	Intron (NM_001025034/498796, intron 1 of 3)

Tnrc6a	308971	chr1	192905654	192905954	-170476	-1.65	3.16E-02	2.80E-01	Distal Intergenic
Cmss1	288176	chr11	44982933	44983233	-48464	-1.66	3.16E-02	2.80E-01	Intron (NM_001107100/304021, intron 2 of 3)
Tdrd7	85425	chr5	61558042	61558342	-6522	0.63	3.16E-02	2.80E-01	Distal Intergenic
RGD1561161	294747	chr2	45171037	45171337	-11054	-1.19	3.16E-02	2.80E-01	Distal Intergenic
Aarsd1	619440	chr10	89330726	89331026	52	0.58	3.16E-02	2.80E-01	Promoter (<=1kb)
Suv39h1	302553	chrX	15171776	15172076	277	1.16	3.16E-02	2.81E-01	Promoter (<=1kb)
Gtf3c1	171063	chr1	197098274	197098574	102	0.52	3.16E-02	2.81E-01	Promoter (<=1kb)
Slc43a2	287532	chr10	63671720	63672020	-5376	-1.19	3.17E-02	2.81E-01	Intron (NM_001107022/303313, intron 8 of 9)
Slc23a2	50622	chr3	124776402	124776702	-52150	0.94	3.17E-02	2.81E-01	Distal Intergenic
Ccn2	64032	chr1	21842257	21842557	12216	-0.95	3.17E-02	2.81E-01	Distal Intergenic
Capzb	298584	chr5	157653230	157653530	10453	-1.25	3.17E-02	2.81E-01	Intron (NM_001005903/298584, intron 1 of 8)
Mras	25482	chr8	107680967	107681267	-24116	0.78	3.17E-02	2.81E-01	Distal Intergenic
Ptpn21	171070	chr6	122708338	122708638	12858	-1.63	3.17E-02	2.81E-01	Intron (NM_133545/171070, intron 2 of 18)
Tor4a	311795	chr3	2412821	2413121	-1277	1.84	3.17E-02	2.81E-01	Promoter (1-2kb)
Lats1	308265	chr1	1778007	1778307	-5771	1.88	3.17E-02	2.81E-01	Intron (NM_001128191/683983, intron 5 of 6)
Cxcl12	24772	chr4	149417912	149418212	156868	-1.22	3.17E-02	2.81E-01	Distal Intergenic
Rhbdd2	360793	chr12	24113834	24114134	-11753	1.56	3.17E-02	2.81E-01	Distal Intergenic
Slc11a2	25715	chr7	142061238	142061538	1332	0.76	3.17E-02	2.81E-01	Promoter (1-2kb)
Tnfrsf26	361685	chr1	216868867	216869167	-40286	-1.16	3.17E-02	2.81E-01	Intron (NM_001015024/361686, intron 21 of 21)
Atoh8	500200	chr4	100109197	100109497	-9680	0.87	3.17E-02	2.81E-01	Distal Intergenic
Serpinh1	29345	chr1	164359924	164360224	-51617	-1.15	3.17E-02	2.81E-01	Distal Intergenic
Umps	288051	chr11	70088594	70088894	54413	1.54	3.17E-02	2.81E-01	Intron (NM_147139/257645, intron 10 of 15)
Bcl2l1	24888	chr3	148296453	148296753	15532	-1.18	3.17E-02	2.81E-01	Intron (NM_001033671/24888, intron 1 of 1)
Pak2	29432	chr11	71959295	71959595	46	1.19	3.18E-02	2.81E-01	Promoter (<=1kb)
Zfp131	310375	chr2	52773194	52773494	-242109	-1.55	3.18E-02	2.81E-01	Distal Intergenic
Sdc3	116673	chr5	148899421	148899721	-23377	1.66	3.18E-02	2.81E-01	Intron (NM_001108684/362609, intron 18 of 21)
Plcb4	25031	chr3	128713549	128713849	112219	-1.04	3.18E-02	2.81E-01	Intron (NM_024353/25031, intron 2 of 38)
Map4k4	301363	chr9	46657264	46657564	-358	1.75	3.18E-02	2.81E-01	Promoter (<=1kb)
Slc29a1	63997	chr9	17771120	17771420	-13048	-1.44	3.18E-02	2.81E-01	Distal Intergenic
Mst1r	300999	chr8	116688158	116688458	1557	-1.68	3.18E-02	2.81E-01	Promoter (1-2kb)
Txk	305311	chr14	37981425	37981725	-48587	-1.07	3.18E-02	2.81E-01	Intron (NM_053432/84492, intron 3 of 18)
Tmem176b	171411	chr4	78445831	78446131	12048	-1.31	3.18E-02	2.81E-01	Distal Intergenic
Fam167a	498533	chr15	46647514	46647814	18284	-1.35	3.18E-02	2.81E-01	Distal Intergenic
Adra2b	24174	chr3	119873131	119873431	67190	1.17	3.18E-02	2.81E-01	Distal Intergenic
Snn	29140	chr10	4659173	4659473	-14603	-1.18	3.18E-02	2.81E-01	Distal Intergenic
Foxn3	314374	chr6	123408393	123408693	169002	1.41	3.18E-02	2.81E-01	Intron (NM_001108047/314374, intron 2 of 6)
Rhno1	297627	chr4	161599960	161600260	84734	-1.1	3.18E-02	2.81E-01	Distal Intergenic
Mef2a	309957	chr1	128255691	128255991	85272	-0.86	3.19E-02	2.81E-01	Intron (NM_001014035/309957, intron 4 of 10)
Fbp2	114508	chr17	397389	397689	-7471	-1.29	3.19E-02	2.81E-01	Intron (NM_012558/24362, intron 8 of 8)
Rasgrp2	361714	chr1	221773877	221774177	623	0.93	3.19E-02	2.81E-01	Promoter (<=1kb)
Marchf5	294079	chr1	255885530	255885830	42885	-1.31	3.19E-02	2.81E-01	Distal Intergenic
Zfand6	293067	chr1	146823286	146823586	176	1.34	3.19E-02	2.81E-01	Promoter (<=1kb)
Fchsd1	307482	chr18	31153714	31154014	-44699	0.61	3.19E-02	2.81E-01	Distal Intergenic
Rbms1	362138	chr3	47024224	47024524	604	0.77	3.19E-02	2.81E-01	Promoter (<=1kb)
Abhd16b	311720	chr3	176958568	176958868	-27032	1.65	3.19E-02	2.81E-01	Distal Intergenic
Ica1	81024	chr4	34543601	34543901	236206	1.58	3.19E-02	2.81E-01	Intron (NM_001177442/296884, intron 4 of 10)
Itgb5	257645	chr11	70128970	70129270	42894	-1.38	3.19E-02	2.81E-01	Intron (NM_147139/257645, intron 5 of 15)
Fzd1	58868	chr4	26308135	26308435	-162429	0.76	3.19E-02	2.81E-01	Intron (NM_001108617/362316, intron 10 of 12)
Prkar2b	24679	chr6	51339637	51339937	16446	-1.23	3.19E-02	2.81E-01	Intron (NM_001030020/24679, intron 1 of 10)
Tmed1	315461	chr8	22542608	22542908	89	0.61	3.19E-02	2.81E-01	Promoter (<=1kb)
Arhgef16	687105	chr5	171719989	171720289	-71488	1.33	3.19E-02	2.81E-01	Distal Intergenic
Csnk1e	58822	chr7	120675054	120675354	-2704	1.03	3.19E-02	2.81E-01	Promoter (2-3kb)
Ctsh	25425	chr8	97409233	97409533	-29536	-1.37	3.20E-02	2.81E-01	Distal Intergenic
Ubl7	300744	chr8	62699833	62700133	3113	-1.23	3.20E-02	2.81E-01	Intron (NM_001004247/300744, intron 3 of 10)
Hic2	287940	chr11	88016487	88016787	38	0.94	3.20E-02	2.81E-01	Promoter (<=1kb)
Letm2	361169	chr16	71241455	71241755	120	0.62	3.20E-02	2.81E-01	Promoter (<=1kb)
Trim32	313264	chr5	81431598	81431898	0	0.65	3.20E-02	2.81E-01	Promoter (<=1kb)
Col14a1	314981	chr7	94939010	94939310	-115567	1.85	3.20E-02	2.81E-01	Distal Intergenic
Ust	361450	chr1	2643633	2643933	-16158	-1.48	3.20E-02	2.81E-01	Distal Intergenic
Asz1	170578	chr4	45333613	45333913	80264	1.44	3.20E-02	2.81E-01	Distal Intergenic
Artn	362572	chr5	136762745	136763045	0	0.7	3.20E-02	2.82E-01	Promoter (<=1kb)
Lpar1	116744	chr5	75535039	75535339	141245	-1.15	3.20E-02	2.82E-01	Distal Intergenic
Ntng2	311836	chr3	7764675	7764975	30783	-1.29	3.21E-02	2.82E-01	Intron (NM_001107825/311836, intron 2 of 7)
Chsy1	292999	chr1	127032934	127033234	22320	-1.1	3.21E-02	2.82E-01	Intron (NM_001106268/292999, intron 2 of 2)
Dipk2a	315891	chr8	102159047	102159347	481	0.86	3.21E-02	2.82E-01	Promoter (<=1kb)
Foxe3	171302	chr5	133136328	133136628	589028	1.76	3.21E-02	2.82E-01	Distal Intergenic
Adcy5	64532	chr11	68985155	68985455	-142835	1.71	3.21E-02	2.82E-01	Distal Intergenic
Mir100	100314234	chr8	45731626	45731926	-15022	-1.21	3.21E-02	2.82E-01	Intron (NR_126581/104845260, intron 2 of 3)
Nek7	360850	chr13	55580039	55580339	-18313	-1.84	3.21E-02	2.82E-01	Distal Intergenic
Bri3	304284	chr12	12330539	12330839	90	1.33	3.21E-02	2.82E-01	Promoter (<=1kb)
Hectd2	309514	chr1	255186732	255187032	3	0.73	3.21E-02	2.82E-01	Promoter (<=1kb)
Kcnrg	305947	chr15	41981716	41982016	43836	0.98	3.21E-02	2.82E-01	Distal Intergenic
Tbc1d9	304645	chr19	24329325	24329625	302	0.74	3.21E-02	2.82E-01	Promoter (<=1kb)
Zfp217	311764	chr3	166885698	166885998	107942	0.71	3.21E-02	2.82E-01	Distal Intergenic
Otud4	307774	chr19	31937649	31937949	4231	-1.52	3.21E-02	2.82E-01	Intron (NM_001191700/307774, intron 2 of 20)
Zdhhc8	303796	chr11	86903248	86903548	126	0.79	3.22E-02	2.82E-01	Promoter (<=1kb)
Cep104	246295	chr5	171262007	171262307	0	0.73	3.22E-02	2.82E-01	Promoter (<=1kb)
Timm13	252928	chr7	11677348	11677648	8	0.53	3.22E-02	2.82E-01	Promoter (<=1kb)
Trim13	364398	chr15	41927135	41927435	0	0.76	3.22E-02	2.82E-01	Promoter (<=1kb)
RGD1307603	293656	chr1	219308188	219308488	3752	0.91	3.22E-02	2.82E-01	Downstream (1-2kb)
Hexa	300757	chr8	64325386	64325686	0	0.73	3.22E-02	2.82E-01	Promoter (<=1kb)
Paqr8	316275	chr9	27040107	27040407	6085	1.8	3.22E-02	2.82E-01	Intron (NM_001014099/316275, intron 1 of 2)
Hipk2	362342	chr4	66522026	66522326	102586	1.77	3.22E-02	2.82E-01	Intron (NM_001108622/362342, intron 2 of 14)
Laptm4b	315047	chr7	72918979	72919279	-5632	-1.38	3.22E-02	2.82E-01	Distal Intergenic
Hoxc12	300262	chr7	144550161	144550461	2222	1.79	3.22E-02	2.82E-01	Promoter (2-3kb)
Prpf4	298095	chr5	78267268	78267568	20	0.67	3.22E-02	2.82E-01	Promoter (<=1kb)
Prr15	312358	chr4	84506007	84506307	27168	1.3	3.22E-02	2.82E-01	Distal Intergenic
Slc25a37	306000	chr15	51140694	51140994	27390	-1.08	3.22E-02	2.82E-01	Intron (NM_001013996/306000, intron 1 of 3)
Nmnat3	363118	chr8	106317171	106317471	0	0.83	3.22E-02	2.82E-01	Promoter (<=1kb)
Phka2	678739	chrX	36923759	36924059	1853	-1.5	3.23E-02	2.82E-01	Promoter (1-2kb)
Srgap2	360840	chr13	48179095	48179395	107325	-1.66	3.23E-02	2.82E-01	Intron (NM_001134958/360840, intron 3 of 21)
Peak1	315686	chr8	61079157	61079457	69	1.2	3.23E-02	2.82E-01	Promoter (<=1kb)
Serp2	498546	chr15	58817335	58817635	-105463	-1.21	3.23E-02	2.82E-01	Distal Intergenic
RGD1566099	360851	chr13	56015477	56015777	-128923	1.1	3.23E-02	2.82E-01	Distal Intergenic

Rnf24	362218	chr3	123897976	123898276	-48188	1	3.23E-02	2.82E-01	Distal Intergenic
Specc1	303208	chr10	48281691	48281991	41361	-1.13	3.23E-02	2.82E-01	Intron (NM_001039017/303208, intron 1 of 7)
Enpp3	54410	chr1	21607539	21607839	-5309	1.48	3.23E-02	2.82E-01	Distal Intergenic
Ptch1	89830	chr17	1174412	1174712	-88527	1.59	3.23E-02	2.82E-01	Distal Intergenic
Nudt14	299346	chr6	137758440	137758740	-13297	-1.57	3.23E-02	2.82E-01	Distal Intergenic
Ehmt1	362078	chr3	2123227	2123527	279	0.81	3.23E-02	2.82E-01	Promoter (<=1kb)
Zc3hav1	252832	chr4	65959222	65959522	102573	-1.5	3.23E-02	2.82E-01	Distal Intergenic
Map4k3	170920	chr6	3439043	3439343	5176	-1.39	3.23E-02	2.82E-01	Intron (NM_133407/170920, intron 1 of 32)
Lamc2	192362	chr13	70503944	70504244	122008	1.72	3.23E-02	2.82E-01	Intron (NM_001048042/289095, intron 1 of 10)
Ptma	29222	chr9	93550100	93550400	4704	-1.38	3.23E-02	2.82E-01	Distal Intergenic
Chd2	308738	chr1	134816315	134816615	54553	1.59	3.23E-02	2.82E-01	Intron (NM_001107523/308738, intron 17 of 38)
Atf1	315305	chr7	141740098	141740398	-141863	-1.4	3.23E-02	2.82E-01	Distal Intergenic
Cyfip1	308666	chr1	114258906	114259206	113	0.64	3.24E-02	2.82E-01	Promoter (<=1kb)
Macroh2a1	29384	chr17	9283248	9283548	573	0.66	3.24E-02	2.82E-01	Promoter (<=1kb)
Flt3lg	103691134	chr1	101135374	101135674	-3961	1.86	3.24E-02	2.82E-01	Downstream (2-3kb)
Stx19	685180	chr7	709635	709935	-435067	-1.08	3.24E-02	2.82E-01	Distal Intergenic
Dnaja2	84026	chr19	22570002	22570302	3	1.23	3.24E-02	2.82E-01	Promoter (<=1kb)
G3bp1	171092	chr10	40812923	40813223	65	1.19	3.24E-02	2.82E-01	Promoter (<=1kb)
Lrrc1	367113	chr8	84834915	84835215	0	1.01	3.24E-02	2.82E-01	Promoter (<=1kb)
Zkscan3	306977	chr17	45250927	45251227	3133	-1.36	3.24E-02	2.82E-01	Intron (NM_001012053/306977, intron 1 of 5)
Stxbp5	81022	chr1	4011269	4011569	0	1.31	3.24E-02	2.82E-01	Promoter (<=1kb)
Afdn	26955	chr1	53958378	53958678	-117122	-1.59	3.24E-02	2.82E-01	Distal Intergenic
Fam241a	691931	chr2	232300941	232301241	-55622	-1.59	3.24E-02	2.82E-01	Distal Intergenic
Pax1	311505	chr3	141905315	141905615	327811	-1.59	3.24E-02	2.82E-01	Distal Intergenic
Cdkn2b	25164	chr5	108046674	108046974	-189289	-1.59	3.24E-02	2.82E-01	Distal Intergenic
Lemd3	680066	chr7	63008362	63008662	37066	-1.59	3.24E-02	2.82E-01	Intron (NM_001191000/680066, intron 4 of 12)
Lrrc49	300763	chr8	65575904	65576204	11223	-1.59	3.24E-02	2.82E-01	Intron (NM_001134469/300763, intron 5 of 15)
Ppp1r15b	304799	chr13	49903584	49903884	-29271	-1.13	3.24E-02	2.82E-01	Exon (NM_001105951/289021, exon 26 of 32)
Pid1	501174	chr9	91864872	91865172	-181404	1.42	3.24E-02	2.82E-01	Distal Intergenic
Cdkl2	305242	chr14	17536208	17536508	4810	-1.51	3.25E-02	2.82E-01	Intron (NM_001012035/305242, intron 2 of 9)
Dusp6	116663	chr7	41473377	41473677	-1486	0.61	3.25E-02	2.82E-01	Promoter (1-2kb)
Armcx2	367903	chrX	105621492	105621792	364	0.81	3.25E-02	2.82E-01	Promoter (<=1kb)
C2cd2	304055	chr11	38351193	38351493	68539	1.48	3.25E-02	2.82E-01	Distal Intergenic
Ccdc190	498270	chr13	88272306	88272606	6975	-1.01	3.25E-02	2.82E-01	3' UTR
Trim41	303088	chr10	34161593	34161893	4706	-1.4	3.25E-02	2.82E-01	Intron (NM_001134737/303088, intron 1 of 5)
Dmrt2	309430	chr1	243978108	243978408	315285	0.8	3.25E-02	2.82E-01	Distal Intergenic
Cfap45	304984	chr13	90901765	90902065	-7486	0.8	3.25E-02	2.82E-01	Distal Intergenic
Prpf40a	295607	chr3	38734971	38735271	5842	-1.55	3.25E-02	2.82E-01	Intron (NM_001106480/295607, intron 2 of 25)
Rras2	365355	chr1	178956751	178957051	53206	-1.31	3.25E-02	2.82E-01	Intron (NM_001013434/365355, intron 1 of 5)
Wdr63	292165	chr2	251973279	251973579	-2580	-1.53	3.25E-02	2.82E-01	Promoter (2-3kb)
Lmo7	361084	chr15	86381580	86381880	138432	-1.28	3.25E-02	2.82E-01	Intron (NM_001001515/361084, intron 4 of 30)
Lbx1	499362	chr1	264968823	264969123	6009	-1.29	3.25E-02	2.83E-01	Distal Intergenic
Tor1aip1	246314	chr13	73698026	73698326	6342	-1.72	3.26E-02	2.83E-01	Intron (NM_145092/246314, intron 3 of 9)
Rfx3	361746	chr1	246110610	246110910	-68	0.67	3.26E-02	2.83E-01	Promoter (<=1kb)
RGD1562136	501195	chr9	111327895	111328195	562	1.29	3.26E-02	2.83E-01	Promoter (<=1kb)
Mrpl27	287635	chr10	82380909	82381209	5337	1.43	3.26E-02	2.83E-01	3' UTR
Srgap3	500287	chr4	144838868	144839168	29957	1.68	3.26E-02	2.83E-01	Intron (NM_001191975/500287, intron 1 of 21)
Stk38l	691337	chr4	181038714	181039014	11502	-1.18	3.26E-02	2.83E-01	Intron (NM_001083336/691337, intron 1 of 14)
Utp18	303456	chr10	81587544	81587844	52	0.84	3.26E-02	2.83E-01	Promoter (<=1kb)
Efna2	84358	chr7	12393016	12393316	0	1.16	3.26E-02	2.83E-01	Promoter (<=1kb)
Xpc	312560	chr4	123155608	123155908	6077	-1.03	3.26E-02	2.83E-01	Intron (NM_001107874/312560, intron 2 of 15)
Nadk	100125370	chr5	172993898	172994198	7607	-1.42	3.27E-02	2.83E-01	Intron (NM_001109678/100125370, intron 1 of 11)
Dtx2	304591	chr12	23711916	23712216	15348	1.71	3.27E-02	2.83E-01	Intron (NM_001107157/304591, intron 3 of 11)
Stom	296655	chr3	14523701	14524001	14198	0.93	3.27E-02	2.83E-01	Intron (NM_001011965/296655, intron 4 of 6)
Pls3	81748	chrX	119087057	119087357	56638	-1.34	3.27E-02	2.83E-01	Intron (NM_031084/81748, intron 2 of 15)
Ltc4s	114097	chr10	35740684	35740984	-1065	1.33	3.27E-02	2.83E-01	Promoter (1-2kb)
Canx	29144	chr10	35824953	35825253	8608	1.43	3.27E-02	2.83E-01	Intron (NM_172008/29144, intron 1 of 14)
Iba57	363611	chr10	45514545	45514845	64	0.81	3.27E-02	2.83E-01	Promoter (<=1kb)
Styx	100912536	chr15	19690161	19690461	0	0.97	3.27E-02	2.83E-01	Promoter (<=1kb)
Kremen1	114107	chr14	85483288	85483588	20073	1.21	3.27E-02	2.83E-01	Intron (NM_053649/114107, intron 2 of 8)
Gpat3	305166	chr14	10394654	10394954	45	0.61	3.27E-02	2.83E-01	Promoter (<=1kb)
Lifr	81680	chr2	56344344	56344644	-92082	-1.23	3.27E-02	2.83E-01	Distal Intergenic
Eef1e1	291057	chr17	26792018	26792318	7001	-1.3	3.28E-02	2.84E-01	Intron (NM_001106106/291057, intron 3 of 3)
Xpnpep1	170751	chr1	273676749	273677049	81198	1.67	3.28E-02	2.84E-01	Distal Intergenic
Gdf7	252833	chr6	33596577	33596877	-89035	-1.39	3.28E-02	2.84E-01	Distal Intergenic
Synpo	60324	chr18	55940565	55940865	-48855	1.47	3.28E-02	2.84E-01	Distal Intergenic
Capn13	362701	chr6	24537647	24537947	138471	1.12	3.28E-02	2.84E-01	Distal Intergenic
Large1	361368	chr19	12943070	12943370	-127	1.19	3.28E-02	2.84E-01	Promoter (<=1kb)
Nfe2l2	83619	chr3	62581212	62581512	-56218	-1.34	3.29E-02	2.84E-01	Distal Intergenic
Vapa	58857	chr9	113330838	113331138	181	1.49	3.29E-02	2.84E-01	Promoter (<=1kb)
Stub1	287155	chr10	15200109	15200409	-74	0.64	3.29E-02	2.84E-01	Promoter (<=1kb)
Rn5-8s	100861534	chr6	30361279	30361579	-269322	0.59	3.29E-02	2.84E-01	Distal Intergenic
Sgms1	353229	chr1	250949884	250950184	1502	1.03	3.29E-02	2.84E-01	Promoter (1-2kb)
Oxnad1	306270	chr16	8114426	8114726	-92497	1.61	3.29E-02	2.84E-01	Distal Intergenic
Ccdc25	361059	chr15	42562526	42562826	40418	1.37	3.29E-02	2.84E-01	Distal Intergenic
Itpr1	25262	chr4	140425140	140425440	177805	1.79	3.29E-02	2.84E-01	Exon (NM_001007235/25262, exon 25 of 62)
Pabpn1	116697	chr15	33555676	33555976	36	1.09	3.29E-02	2.84E-01	Promoter (<=1kb)
Hus1	498411	chr14	89173920	89174220	34605	-1.39	3.29E-02	2.84E-01	Distal Intergenic
Ythdc1	170956	chr14	22996988	22997288	4723	-1.55	3.30E-02	2.84E-01	Intron (NM_133423/170956, intron 1 of 16)
Sgce	432360	chr4	29760185	29760485	9417	-1.55	3.30E-02	2.84E-01	Intron (NM_001002023/432360, intron 3 of 9)
Ube2r2	689226	chr5	57491886	57492186	19571	-1.55	3.30E-02	2.84E-01	Intron (NM_001127573/689226, intron 1 of 4)
Tmtc3	314785	chr7	39819919	39820219	396837	-1.55	3.30E-02	2.84E-01	Distal Intergenic
Pdzd11	302422	chrX	70490621	70490921	-28986	-1.55	3.30E-02	2.84E-01	Distal Intergenic
Atp6v0d1	291969	chr19	37514716	37515016	10746	1.79	3.30E-02	2.84E-01	Intron (NM_001011927/291969, intron 1 of 7)
Inpp1	316376	chr9	53544759	53545059	-17565	0.6	3.30E-02	2.84E-01	Distal Intergenic
Stag1	315958	chr8	108958722	108959022	623	0.72	3.30E-02	2.84E-01	Promoter (<=1kb)
Ahsp	293522	chr1	199725516	199725816	5478	-1.62	3.30E-02	2.84E-01	Distal Intergenic
Slc4a7	117955	chr15	12010061	12010361	-97145	-1.62	3.30E-02	2.84E-01	Distal Intergenic
Lrp10	305880	chr15	33111994	33112294	3323	-1.62	3.30E-02	2.84E-01	Exon (NM_001037777/305880, exon 4 of 7)
Fndc3b	294925	chr2	113268117	113268417	77134	-1.62	3.30E-02	2.84E-01	Intron (NM_001191704/294925, intron 2 of 24)
Itprid2	311146	chr3	66577071	66577371	-16404	-1.62	3.30E-02	2.84E-01	Distal Intergenic
Smad6	367100	chr8	68871558	68871858	94250	-1.62	3.30E-02	2.84E-01	Distal Intergenic
Lrp1	299858	chr7	70900385	70900685	26218	-1.26	3.30E-02	2.84E-01	Intron (NM_001130490/299858, intron 8 of 88)
Timm10	64464	chr3	72229789	72230089	3176	-1.5	3.30E-02	2.84E-01	3' UTR

Ptpn13	498331	chr14	7891924	7892224	-28260	-1.57	3.30E-02	2.84E-01	Distal Intergenic
Atic	81643	chr9	78865932	78866232	3919	-1.62	3.30E-02	2.84E-01	Intron (NM_031014/81643, intron 4 of 15)
Afap1	140935	chr14	80034638	80034938	-46723	0.63	3.30E-02	2.84E-01	Distal Intergenic
Arhgef28	361882	chr2	25844167	25844467	166962	0.75	3.30E-02	2.84E-01	Intron (NM_001108542/361882, intron 9 of 35)
Pinlyp	308429	chr1	81411022	81411322	755	1.47	3.30E-02	2.84E-01	Promoter (<=1kb)
Mbnl1	282635	chr2	150826789	150827089	70531	-1.54	3.30E-02	2.84E-01	Intron (NM_001191566/282635, intron 1 of 7)
Smim15	100359861	chr2	39314480	39314780	0	0.66	3.31E-02	2.84E-01	Promoter (<=1kb)
Cdv3	315970	chr8	111849540	111849840	553	1.38	3.31E-02	2.84E-01	Promoter (<=1kb)
Dkkl1	499150	chr1	101196396	101196696	13979	-1.54	3.31E-02	2.84E-01	Distal Intergenic
Tcf7l2	679869	chr1	276624511	276624811	-80034	1.74	3.31E-02	2.84E-01	Distal Intergenic
Fbxl21	306750	chr17	8523479	8523779	-3332	0.85	3.31E-02	2.84E-01	Distal Intergenic
Kcng1	296395	chr3	165031745	165032045	7662	-0.97	3.31E-02	2.85E-01	Intron (NM_001106545/296395, intron 1 of 2)
RGD1359290	360649	chr10	95618013	95618313	-16393	1.83	3.31E-02	2.85E-01	Distal Intergenic
Bcas1	246755	chr3	167596840	167597140	436317	0.62	3.31E-02	2.85E-01	Distal Intergenic
Zfp707	362936	chr7	116993768	116994068	-2778	-1.51	3.31E-02	2.85E-01	Promoter (2-3kb)
Pkd2	498328	chr14	6644847	6645147	110	1.05	3.31E-02	2.85E-01	Promoter (<=1kb)
Snx18	310097	chr2	45391331	45391631	89167	-1.21	3.31E-02	2.85E-01	Distal Intergenic
Runx1	50662	chr11	32816031	32816331	42494	-1.41	3.32E-02	2.85E-01	Intron (NM_017325/50662, intron 3 of 5)
Dnajb9	24908	chr6	64169639	64169939	183	0.67	3.32E-02	2.85E-01	Promoter (<=1kb)
Taar8b	319106	chr1	23626004	23626304	58394	1.59	3.32E-02	2.85E-01	Distal Intergenic
Mbnl1	282635	chr2	150802767	150803067	46509	-1.58	3.32E-02	2.85E-01	Intron (NM_001191566/282635, intron 1 of 7)
Cdh11	84407	chr19	2537241	2537541	144182	-1.08	3.32E-02	2.85E-01	Intron (NM_053392/84407, intron 11 of 12)
Mgst3	289197	chr13	85621254	85621554	760	-0.59	3.32E-02	2.85E-01	Promoter (<=1kb)
Tada1	360874	chr13	84588816	84589116	229	0.82	3.32E-02	2.85E-01	Promoter (<=1kb)
Dtnbp1	641528	chr17	20221744	20222044	131608	-1.39	3.32E-02	2.85E-01	Distal Intergenic
Ece1	94204	chr5	156206462	156206762	-8707	-1.48	3.32E-02	2.85E-01	Distal Intergenic
Fat1	83720	chr16	50686195	50686495	-184479	-1.52	3.32E-02	2.85E-01	Distal Intergenic
Pik3c2a	361632	chr1	185207599	185207899	-3023	1.6	3.32E-02	2.85E-01	Distal Intergenic
Epb42	362202	chr3	113032854	113033154	-47536	0.81	3.33E-02	2.85E-01	Distal Intergenic
C1qtnf7	305423	chr14	71979125	71979425	45712	0.61	3.33E-02	2.85E-01	Distal Intergenic
Mir2985	100526643	chr2	179523607	179523907	67514	0.91	3.33E-02	2.85E-01	Distal Intergenic
Rcc1	682908	chr5	150573618	150573918	-48364	-1.05	3.33E-02	2.85E-01	Distal Intergenic
Orai1	304496	chr12	38995132	38995432	138	0.81	3.33E-02	2.85E-01	Promoter (<=1kb)
RGD1302996	294231	chr20	3364889	3365189	75	1.13	3.33E-02	2.85E-01	Promoter (<=1kb)
Ppm1j	295341	chr2	207189607	207189907	-73680	-1.44	3.33E-02	2.85E-01	Distal Intergenic
Ankrd46	299982	chr7	75213532	75213832	86574	-1.27	3.33E-02	2.85E-01	Distal Intergenic
Dusp18	305477	chr14	84154808	84155108	3900	-1.58	3.33E-02	2.85E-01	3' UTR
Gne	114711	chr5	59564971	59565271	-21870	-1.53	3.33E-02	2.85E-01	Distal Intergenic
Gas7	85246	chr10	54084369	54084669	-2174	1.66	3.33E-02	2.85E-01	Promoter (2-3kb)
Sugct	361253	chr17	50603015	50603315	611701	2.06	3.33E-02	2.85E-01	Intron (NM_001014146/361253, intron 10 of 12)
Grin2d	24412	chr1	101856076	101856376	505	1.71	3.34E-02	2.85E-01	Promoter (<=1kb)
Icam4	298702	chr8	22045973	22046273	-1424	-1.34	3.34E-02	2.85E-01	Promoter (1-2kb)
Runx3	156726	chr5	153396829	153397129	-109960	-1.55	3.34E-02	2.85E-01	Distal Intergenic
Sh3glb1	292156	chr2	250675982	250676282	67914	1.38	3.34E-02	2.85E-01	Distal Intergenic
Zc3h15	362154	chr3	71147364	71147664	126830	1.91	3.34E-02	2.85E-01	Distal Intergenic
Csad	60356	chr7	143812946	143813246	-18976	-1.36	3.34E-02	2.85E-01	Distal Intergenic
Lsm3	297455	chr4	123300155	123300455	138069	1.64	3.34E-02	2.85E-01	Distal Intergenic
Dbh	25699	chr3	5721902	5722202	12666	1	3.34E-02	2.85E-01	Intron (NM_013158/25699, intron 9 of 11)
Smim38	246306	chr1	218253434	218253734	120885	-1.27	3.34E-02	2.85E-01	Distal Intergenic
Map2	25595	chr9	73332387	73332687	-45373	1.95	3.34E-02	2.85E-01	Distal Intergenic
Ap3b1	309969	chr2	24166122	24166422	141288	-1.29	3.34E-02	2.85E-01	Intron (NM_001107646/309969, intron 20 of 25)
Scrg1	64458	chr16	36200032	36200332	-38991	0.57	3.34E-02	2.85E-01	Distal Intergenic
Setd6	291844	chr19	9749409	9749709	15775	-1.37	3.34E-02	2.85E-01	Intron (NM_001134840/291841, intron 39 of 48)
RGD1310852	314992	chr7	97894692	97894992	7593	1.14	3.34E-02	2.85E-01	Intron (NM_001025007/314992, intron 4 of 5)
Hcn4	59266	chr8	63803061	63803361	203154	1.37	3.34E-02	2.85E-01	Distal Intergenic
Paqr9	315904	chr8	103295002	103295302	-42170	-1.29	3.34E-02	2.85E-01	Distal Intergenic
Olig1	60394	chr11	31439101	31439401	10724	-1.2	3.35E-02	2.85E-01	Distal Intergenic
Rfx3	361746	chr1	246407003	246407303	-296461	-1.39	3.35E-02	2.85E-01	Distal Intergenic
Ackr3	84348	chr9	97521729	97522029	165805	1.88	3.35E-02	2.85E-01	Distal Intergenic
Pglyrp1	84387	chr1	79798239	79798539	7360	1.22	3.35E-02	2.85E-01	Exon (NM_001106229/292680, exon 10 of 14)
Lman1	116666	chr18	61855242	61855542	-147925	1.62	3.35E-02	2.85E-01	Distal Intergenic
Otulin	100362554	chr2	80292802	80293102	84	1.06	3.35E-02	2.85E-01	Promoter (<=1kb)
Ptges	59103	chr3	9738385	9738685	67	-1.09	3.35E-02	2.85E-01	Promoter (<=1kb)
Accs	311218	chr3	82751650	82751950	5003	1.46	3.35E-02	2.85E-01	Intron (NM_001267534/311218, intron 3 of 15)
Tns1	301509	chr9	81344609	81344909	56078	0.7	3.35E-02	2.85E-01	Exon (NM_001191810/301509, exon 3 of 35)
Tbl1xr1	365755	chr2	107229803	107230103	-2951	-1.35	3.35E-02	2.85E-01	Promoter (2-3kb)
St6gal1	25197	chr11	80962863	80963163	18259	-1.23	3.35E-02	2.85E-01	Intron (NM_001113344/25197, intron 3 of 5)
Fn1	25661	chr9	79242492	79242792	-273479	-0.88	3.35E-02	2.85E-01	Distal Intergenic
Lrrc32	293135	chr1	163477226	163477526	31658	-1.5	3.35E-02	2.85E-01	Distal Intergenic
Fign	295649	chr3	50197356	50197656	-76964	-1.5	3.35E-02	2.85E-01	Distal Intergenic
Raf1	24703	chr4	147561887	147562187	30512	-1.21	3.35E-02	2.85E-01	Intron (NM_012639/24703, intron 1 of 16)
Musk	81725	chr5	75377186	75377486	-15304	1.33	3.35E-02	2.85E-01	Distal Intergenic
Hps3	310288	chr2	104830551	104830851	47	0.67	3.35E-02	2.85E-01	Promoter (<=1kb)
Cyp1b1	25426	chr6	2277106	2277406	39316	-1.22	3.35E-02	2.85E-01	Intron (NM_001037200/313840, intron 10 of 10)
Raf1	24703	chr4	147558914	147559214	33485	-1.05	3.35E-02	2.85E-01	Intron (NM_012639/24703, intron 1 of 16)
Cct8l1	499967	chr4	6084170	6084470	-21529	0.9	3.35E-02	2.85E-01	Distal Intergenic
Fzd6	282581	chr7	77908009	77908309	8687	1.94	3.35E-02	2.85E-01	Intron (NM_001130536/282581, intron 2 of 6)
Zfp36l1	29344	chr6	103088117	103088417	224657	-1.15	3.36E-02	2.85E-01	Distal Intergenic
Arhgap18	293947	chr1	19224080	19224380	151921	1.11	3.36E-02	2.85E-01	Downstream (2-3kb)
Letmd1	681352	chr7	142106262	142106562	0	0.58	3.36E-02	2.85E-01	Promoter (<=1kb)
Hnrnpul1	361522	chr1	82546391	82546691	246	0.66	3.36E-02	2.85E-01	Promoter (<=1kb)
Ppp6c	171121	chr3	23510527	23510827	132	0.6	3.36E-02	2.86E-01	Promoter (<=1kb)
Uchl1	29545	chr14	43301575	43301875	-157633	-0.83	3.36E-02	2.86E-01	Distal Intergenic
B3galt6	298690	chr5	173425264	173425564	47	0.58	3.36E-02	2.86E-01	Promoter (<=1kb)
Mast3	688540	chr16	20400588	20400888	11	0.64	3.36E-02	2.86E-01	Promoter (<=1kb)
Agpat3	294324	chr20	11118577	11118877	4413	1.73	3.36E-02	2.86E-01	Intron (NM_001106378/294324, intron 1 of 8)
Col8a1	304021	chr11	44918384	44918684	40466	-1.41	3.36E-02	2.86E-01	Intron (NM_001107100/304021, intron 1 of 3)
Akap6	64553	chr6	73694588	73694888	141378	-1.38	3.36E-02	2.86E-01	Intron (NM_022618/64553, intron 2 of 13)
Socs3	89829	chr10	107004290	107004590	-28250	-1.17	3.37E-02	2.86E-01	Distal Intergenic
Cfh	155012	chr13	57070251	57070551	9989	1.74	3.37E-02	2.86E-01	Intron (NM_130409/155012, intron 1 of 21)
Gpx3	64317	chr10	40233881	40234181	-13172	-1.4	3.37E-02	2.86E-01	Distal Intergenic
Phf20	311575	chr3	152273387	152273687	118	0.9	3.37E-02	2.86E-01	Promoter (<=1kb)
Srrm2	302969	chr10	13139866	13140166	11046	0.62	3.37E-02	2.86E-01	Intron (NM_001277154/302969, intron 2 of 16)
P2rx4	29659	chr12	39325433	39325733	0	0.62	3.37E-02	2.86E-01	Promoter (<=1kb)

Rab14	94197	chr3	14388577	14388877	-3048	-1.34	3.37E-02	2.86E-01	Distal Intergenic
Map3k12	25579	chr7	144120065	144120365	0	0.7	3.37E-02	2.86E-01	Promoter (<=1kb)
Bmp8a	680931	chr5	141062076	141062376	-28565	-0.95	3.37E-02	2.86E-01	Intron (NM_001135758/362587, intron 85 of 97)
Dusp1	114856	chr10	16896272	16896572	-74070	0.67	3.37E-02	2.86E-01	Distal Intergenic
Camta1	362665	chr5	169016742	169017042	253	0.91	3.37E-02	2.86E-01	Promoter (<=1kb)
Cebpb	24253	chr3	164433883	164434183	9381	-1.21	3.37E-02	2.86E-01	Distal Intergenic
Phf14	500030	chr4	38255323	38255623	141	0.85	3.38E-02	2.86E-01	Promoter (<=1kb)
Psip1	313323	chr5	102080003	102080303	-491905	-0.78	3.38E-02	2.86E-01	Distal Intergenic
Kcng3	171011	chr6	6906723	6907023	-63965	0.64	3.38E-02	2.86E-01	Distal Intergenic
Abr	287537	chr10	64664197	64664497	-7118	1.67	3.38E-02	2.86E-01	Distal Intergenic
Ssh3	365396	chr1	219519125	219519425	0	0.57	3.38E-02	2.86E-01	Promoter (<=1kb)
Hus1	498411	chr14	89055468	89055768	153057	-1.42	3.38E-02	2.86E-01	Distal Intergenic
Fam210b	296408	chr3	170355913	170356213	1772	-1.3	3.38E-02	2.86E-01	Promoter (1-2kb)
Plec	64204	chr7	117289398	117289698	263	0.8	3.38E-02	2.86E-01	Promoter (<=1kb)
Zbtb16	353227	chr8	53069781	53070081	76684	-1.37	3.38E-02	2.86E-01	Intron (NM_001013181/353227, intron 3 of 6)
Itm2c	301575	chr9	92955188	92955488	38719	-1.32	3.38E-02	2.86E-01	Distal Intergenic
Itpr1	25262	chr4	140445070	140445370	197735	-1.16	3.38E-02	2.86E-01	Intron (NM_001007235/25262, intron 36 of 61)
Stxbp6	362734	chr6	65313594	65313894	5633	-1.35	3.38E-02	2.86E-01	Intron (NM_001191872/362734, intron 1 of 5)
Prr7	498704	chr17	9700607	9700907	-5315	-0.95	3.39E-02	2.86E-01	Distal Intergenic
Fbxl19	308999	chr1	199195631	199195931	-1207	0.77	3.39E-02	2.86E-01	Promoter (1-2kb)
Ppa2	310856	chr2	238612256	238612556	83184	1.36	3.39E-02	2.86E-01	Distal Intergenic
Mprip	116504	chr10	45992101	45992401	-26258	1.72	3.39E-02	2.86E-01	Distal Intergenic
Myo9b	25486	chr16	19704473	19704773	35062	-0.98	3.39E-02	2.86E-01	Intron (NM_001271066/25486, intron 2 of 41)
Ccdc62	689909	chr12	38092672	38092972	78	0.55	3.39E-02	2.86E-01	Promoter (<=1kb)
Zfp512b	311721	chr3	177088727	177089027	172	0.86	3.39E-02	2.86E-01	Promoter (<=1kb)
Spen	690911	chr5	160086417	160086717	-15502	0.97	3.39E-02	2.86E-01	Distal Intergenic
Tns3	360980	chr14	88745785	88746085	100786	1.13	3.39E-02	2.86E-01	Intron (NM_001170459/360980, intron 14 of 28)
Mtss2	307845	chr19	40945241	40945541	-19581	1.43	3.39E-02	2.86E-01	Intron (NM_177930/307842, intron 14 of 18)
LOC499469	499469	chr20	46699022	46699322	-8040	1.11	3.39E-02	2.86E-01	Intron (NM_001106396/294518, intron 1 of 7)
Uhmk1	246332	chr13	88536427	88536727	1	1.31	3.39E-02	2.86E-01	Promoter (<=1kb)
Mcart1	313241	chr5	61050736	61051036	78990	-1.4	3.39E-02	2.86E-01	Distal Intergenic
Ccdc150	316399	chr9	60843271	60843571	-56642	1.15	3.39E-02	2.86E-01	Distal Intergenic
Igfbp6	25641	chr7	143749459	143749759	74	0.7	3.39E-02	2.86E-01	Promoter (<=1kb)
Naa50	288108	chr11	61499313	61499613	31217	1.37	3.39E-02	2.86E-01	Distal Intergenic
Cyp2c23	83790	chr1	263805502	263805802	-2352	1.73	3.39E-02	2.86E-01	Promoter (2-3kb)
C1d	289810	chr14	101121981	101122281	706336	-1.41	3.39E-02	2.86E-01	Distal Intergenic
Sgk1	29517	chr1	24258583	24258883	-65016	1.4	3.39E-02	2.86E-01	Distal Intergenic
Lsm1	364624	chr16	71057885	71058185	-2	0.81	3.39E-02	2.86E-01	Promoter (<=1kb)
Tatdn2	500295	chr4	145647396	145647696	-5906	-1.55	3.39E-02	2.86E-01	Exon (NM_001025422/362418, exon 12 of 13)
Arhgap21	307178	chr17	87068076	87068376	781353	-1.2	3.40E-02	2.86E-01	Distal Intergenic
Sag	25539	chr9	94936927	94937227	9861	1.3	3.40E-02	2.86E-01	Intron (NM_013023/25539, intron 4 of 15)
Gcsh	171133	chr19	49532500	49532800	11	0.63	3.40E-02	2.86E-01	Promoter (<=1kb)
Mansc1	690606	chr4	168361143	168361443	13873	-1.36	3.40E-02	2.86E-01	Downstream (1-2kb)
S100a10	81778	chr2	193904942	193905242	12353	1.34	3.40E-02	2.86E-01	Distal Intergenic
Paics	140946	chr14	33584143	33584443	-3577	-1.35	3.40E-02	2.86E-01	Intron (NM_057198/117544, intron 1 of 10)
Sh2b2	114203	chr12	23254314	23254614	-218656	-1.37	3.40E-02	2.86E-01	Distal Intergenic
Nol10	313981	chr6	42745727	42746027	89066	1.75	3.40E-02	2.86E-01	Distal Intergenic
Fam184b	289671	chr14	69765668	69765968	-204066	1.45	3.40E-02	2.86E-01	Distal Intergenic
Cfap126	498278	chr13	89440295	89440595	-39463	1.59	3.40E-02	2.87E-01	Distal Intergenic
Klf9	117560	chr1	240918018	240918318	9535	-1.38	3.40E-02	2.87E-01	Intron (NM_057211/117560, intron 1 of 1)
Nmd3	310512	chr2	166406933	166407233	4095	-1.51	3.40E-02	2.87E-01	Exon (NM_001107682/310512, exon 4 of 16)
Klf7	363243	chr9	70805370	70805670	-17457	-0.75	3.40E-02	2.87E-01	Distal Intergenic
Sap30bp	360662	chr10	104500339	104500639	17328	1.03	3.41E-02	2.87E-01	Intron (NM_001108305/360662, intron 3 of 10)
Gcfc2	312474	chr4	112662609	112662909	0	0.76	3.41E-02	2.87E-01	Promoter (<=1kb)
Zfp763	314586	chr7	15314821	15315121	124	0.7	3.41E-02	2.87E-01	Promoter (<=1kb)
Map4k5	503027	chr6	92229228	92229528	0	0.91	3.41E-02	2.87E-01	Promoter (<=1kb)
Fam160a1	365834	chr2	185120835	185121135	-127485	-1.21	3.41E-02	2.87E-01	Distal Intergenic
Nudt21	291877	chr19	11473672	11473972	22273	0.97	3.41E-02	2.87E-01	Distal Intergenic
Lnc215	104845260	chr8	45544129	45544429	-146767	-1.34	3.41E-02	2.87E-01	Distal Intergenic
Tlr12	362604	chr5	146818778	146819078	-23415	-1.71	3.41E-02	2.87E-01	Distal Intergenic
Plaur	50692	chr1	81336879	81337179	8706	-1.28	3.41E-02	2.87E-01	Intron (NM_017350/50692, intron 4 of 7)
Rpl36	58927	chr9	10442658	10442958	-677	1.73	3.41E-02	2.87E-01	Promoter (<=1kb)
Crim1	298744	chr6	788941	789241	393	1.16	3.41E-02	2.87E-01	Promoter (<=1kb)
Arfgef1	312915	chr5	8673153	8673453	6953	-1.31	3.41E-02	2.87E-01	Intron (NM_001277056/312915, intron 1 of 38)
Rlbp1	293049	chr1	141115209	141115509	-3834	0.84	3.41E-02	2.87E-01	Distal Intergenic
Map1lc3a	362245	chr3	150820990	150821290	19701	-1.53	3.41E-02	2.87E-01	Intron (NM_181637/353304, intron 8 of 11)
Runx1	50662	chr11	33177219	33177519	-318394	1.42	3.41E-02	2.87E-01	Distal Intergenic
Thap4	363291	chr9	100887769	100888069	38	0.79	3.42E-02	2.87E-01	Promoter (<=1kb)
Mmd	303439	chr10	77728444	77728744	-26844	-1.26	3.42E-02	2.87E-01	Distal Intergenic
Tob2	315159	chr7	123087935	123088235	5	0.57	3.42E-02	2.87E-01	Promoter (<=1kb)
Sirt4	304539	chr12	46862532	46862832	-5584	0.52	3.42E-02	2.87E-01	Distal Intergenic
Sox4	364712	chr17	37849413	37849713	234391	0.82	3.42E-02	2.87E-01	Distal Intergenic
Trappc9	315059	chr7	114308570	114308870	220	0.66	3.42E-02	2.87E-01	Promoter (<=1kb)
Dnaaf1	361419	chr19	52225187	52225487	-95	1.1	3.42E-02	2.87E-01	Promoter (<=1kb)
Gsdme	353316	chr4	80015328	80015628	-15650	1.3	3.42E-02	2.87E-01	Distal Intergenic
Prelp	84400	chr13	50760830	50761130	148	1.49	3.42E-02	2.87E-01	Promoter (<=1kb)
Cbln3	501998	chr15	34550336	34550636	214	1.36	3.43E-02	2.87E-01	Promoter (<=1kb)
Fzd1	58868	chr4	26306414	26306714	-164150	1.84	3.43E-02	2.87E-01	Intron (NM_001108617/362316, intron 10 of 12)
Klhl24	303803	chr11	84643370	84643670	4	0.69	3.43E-02	2.88E-01	Promoter (<=1kb)
Cnnm2	294014	chr1	266561575	266561875	31098	-1.35	3.43E-02	2.88E-01	Intron (NM_001011942/294014, intron 1 of 7)
Sema5a	310207	chr2	85381127	85381427	3809	-1.34	3.43E-02	2.88E-01	Intron (NM_001107659/310207, intron 1 of 22)
Ppwd1	294711	chr2	34556918	34557218	-243824	-1.2	3.43E-02	2.88E-01	Distal Intergenic
Zfp148	58820	chr11	70654020	70654320	-35617	-1.31	3.43E-02	2.88E-01	Distal Intergenic
Cspg4	81651	chr8	61547228	61547528	14763	-1.52	3.43E-02	2.88E-01	Intron (NM_031022/81651, intron 1 of 10)
Usp15	171329	chr7	66595961	66596261	219	0.88	3.43E-02	2.88E-01	Promoter (<=1kb)
Pex26	297570	chr4	153703108	153703408	-44935	1.5	3.43E-02	2.88E-01	Distal Intergenic
Zbtb10	80338	chr2	94731185	94731485	-209	1.08	3.43E-02	2.88E-01	Promoter (<=1kb)
Nifk	246042	chr13	34380750	34381050	113279	1.95	3.43E-02	2.88E-01	Distal Intergenic
Ccng1	25405	chr10	26536562	26536862	-626264	1.31	3.44E-02	2.88E-01	Distal Intergenic
Spink7	408237	chr18	57990964	57991264	-70834	-1.47	3.44E-02	2.88E-01	Intron (NM_001109539/689570, intron 1 of 4)
Cnih3	690252	chr13	99743243	99743543	80093	-1.09	3.44E-02	2.88E-01	Intron (NM_001166578/690252, intron 3 of 5)
Cald1	25687	chr4	62104285	62104585	-116426	0.6	3.44E-02	2.88E-01	Distal Intergenic
Zfp287	303212	chr10	49150601	49150901	-1122	0.84	3.44E-02	2.88E-01	Promoter (1-2kb)
Cdc42ep3	313838	chr6	1869427	1869727	73245	1.47	3.44E-02	2.88E-01	Distal Intergenic

Rhno1	297627	chr4	161541529	161541829	143165	1.83	3.44E-02	2.88E-01	Distal Intergenic
Thbs4	29220	chr2	22427576	22427876	-41721	0.57	3.44E-02	2.88E-01	Distal Intergenic
Tbc1d19	289657	chr14	59502154	59502454	14400	-1.53	3.44E-02	2.88E-01	Intron (NM_001106008/289657, intron 1 of 20)
Rcan1	266766	chr11	32577720	32578020	-27197	1.21	3.44E-02	2.88E-01	Distal Intergenic
Mn1	498194	chr12	51269508	51269808	-19278	0.9	3.44E-02	2.88E-01	Distal Intergenic
Jak1	84598	chr5	120080305	120080605	3299	-1.37	3.44E-02	2.88E-01	Intron (NM_053466/84598, intron 1 of 24)
Ermp1	373544	chr1	247829191	247829491	-7463	1.84	3.44E-02	2.88E-01	Distal Intergenic
Pik3ca	170911	chr2	118793178	118793478	-37872	-1.39	3.45E-02	2.88E-01	Distal Intergenic
Zfp395	305972	chr15	48763307	48763607	-25713	0.71	3.45E-02	2.88E-01	Distal Intergenic
Eif4g3	298573	chr5	156433137	156433437	36442	-1.12	3.45E-02	2.88E-01	Intron (NM_001106693/298573, intron 1 of 30)
Pid1	501174	chr9	91994517	91994817	-311049	-1.22	3.45E-02	2.88E-01	Distal Intergenic
Actl7b	313183	chr5	73244849	73245149	248881	1.68	3.45E-02	2.88E-01	Distal Intergenic
Tmem163	360839	chr13	44278508	44278808	66927	-1.46	3.45E-02	2.88E-01	Intron (NM_001110763/360839, intron 2 of 7)
Nobox	502759	chr4	72717351	72717651	39236	-1.31	3.45E-02	2.88E-01	Distal Intergenic
Vgf	29461	chr12	22675198	22675498	5132	1.17	3.45E-02	2.88E-01	3' UTR
Esf1	366203	chr3	133231973	133232273	46	0.69	3.45E-02	2.88E-01	Promoter (<=1kb)
Crtap	363158	chr8	122350113	122350413	51785	-1.27	3.45E-02	2.88E-01	Distal Intergenic
Chek2	114212	chr12	51839866	51840166	37458	0.85	3.45E-02	2.88E-01	Distal Intergenic
Eif4g3	298573	chr5	156343727	156344027	-52668	1.33	3.45E-02	2.88E-01	Distal Intergenic
Crlf3	54395	chr10	67400465	67400765	1073	1.31	3.45E-02	2.88E-01	Promoter (1-2kb)
Aldh1l2	299699	chr7	26360760	26361060	-14806	1.23	3.45E-02	2.88E-01	Distal Intergenic
Rgr	306307	chr16	14165938	14166238	134713	-1.32	3.46E-02	2.88E-01	Distal Intergenic
Lhfpl4	353230	chr4	145092674	145092974	54423	1.38	3.46E-02	2.88E-01	Exon (NM_001106614/297514, exon 24 of 24)
Sgce	432360	chr4	29794252	29794552	-24350	0.93	3.46E-02	2.88E-01	Distal Intergenic
Cep112	287776	chr10	96760065	96760365	96325	1.87	3.46E-02	2.88E-01	Intron (NM_001105849/287776, intron 8 of 29)
Syap1	302678	chrX	33528226	33528526	141	0.74	3.46E-02	2.88E-01	Promoter (<=1kb)
Cnot11	363221	chr9	46283931	46284231	13702	-1.37	3.46E-02	2.88E-01	Distal Intergenic
Slc1a5	292657	chr1	78717820	78718120	7134	-1.43	3.46E-02	2.88E-01	Intron (NM_175758/292657, intron 4 of 7)
Mir181a-1	100314195	chr13	54734525	54734825	-217917	-1.51	3.46E-02	2.88E-01	Distal Intergenic
Sec61g	689134	chr14	99223764	99224064	556900	-1.51	3.46E-02	2.88E-01	Distal Intergenic
Arhgef12	367072	chr8	47354038	47354338	38704	-1.51	3.46E-02	2.88E-01	Intron (NM_001013246/367072, intron 1 of 52)
Pcca	687008	chr15	108960534	108960834	4	0.63	3.46E-02	2.88E-01	Promoter (<=1kb)
Rap2b	170923	chr2	151689797	151690097	4546	-1.36	3.46E-02	2.88E-01	Distal Intergenic
Mfsd13a	309454	chr1	266101163	266101463	-3661	-1.08	3.46E-02	2.88E-01	Distal Intergenic
LOC690784	690784	chr4	181350288	181350588	-1489	-0.81	3.46E-02	2.88E-01	Promoter (1-2kb)
Psip1	313323	chr5	101639427	101639727	-51329	0.58	3.47E-02	2.88E-01	Distal Intergenic
Spire1	307348	chr18	63356863	63357163	135	1.43	3.47E-02	2.88E-01	Promoter (<=1kb)
Skap1	286975	chr10	84272258	84272558	-36872	-1.5	3.47E-02	2.88E-01	Distal Intergenic
Aox2	316421	chr9	65191427	65191727	13916	-1.02	3.47E-02	2.88E-01	Intron (NM_001008522/316421, intron 7 of 35)
Lars1	291624	chr18	36628418	36628718	-49055	-1.15	3.47E-02	2.89E-01	Distal Intergenic
Wbp4	114765	chr15	61772251	61772551	0	0.79	3.47E-02	2.89E-01	Promoter (<=1kb)
Pex14	64460	chr5	165693550	165693850	224595	1.37	3.47E-02	2.89E-01	Distal Intergenic
Pid1	501174	chr9	91837962	91838262	-154494	1.44	3.47E-02	2.89E-01	Distal Intergenic
Ipo13	116458	chr5	136744646	136744946	4011	-1.4	3.47E-02	2.89E-01	Intron (NM_053778/116458, intron 3 of 20)
Hip1r	81917	chr12	38034389	38034689	18396	0.85	3.47E-02	2.89E-01	Intron (NM_001134763/81917, intron 7 of 31)
Rnf14	619577	chr18	31443530	31443830	-642	0.6	3.47E-02	2.89E-01	Promoter (<=1kb)
Capn12	308476	chr1	87090754	87091054	24465	1.62	3.47E-02	2.89E-01	Intron (NM_031675/63836, intron 9 of 20)
Eng	497010	chr3	11679641	11679941	111	0.56	3.47E-02	2.89E-01	Promoter (<=1kb)
Pink1	298575	chr5	156688964	156689264	0	1.01	3.48E-02	2.89E-01	Promoter (<=1kb)
Tmem158	117582	chr8	132358986	132359286	-13278	1.74	3.48E-02	2.89E-01	Distal Intergenic
Cnih4	289324	chr13	99482311	99482611	9267	-1.48	3.48E-02	2.89E-01	Intron (NM_001105981/289324, intron 3 of 4)
Dhrs3	313689	chr5	162931720	162932020	122630	1.39	3.48E-02	2.89E-01	Intron (NM_001108006/313825, intron 66 of 69)
Gcc2	309798	chr20	27892793	27893093	59800	-1.59	3.48E-02	2.89E-01	Distal Intergenic
G3bp1	171092	chr10	40810037	40810337	-2521	1.61	3.48E-02	2.89E-01	Promoter (2-3kb)
Smo	25273	chr4	57019862	57020162	0	0.87	3.48E-02	2.89E-01	Promoter (<=1kb)
Mansc1	690606	chr4	168356290	168356590	18726	1.33	3.48E-02	2.89E-01	Distal Intergenic
Nrf1	312195	chr4	57204876	57205176	-131489	0.54	3.48E-02	2.89E-01	Distal Intergenic
LOC103695172	103695172	chr3	59709308	59709608	-108325	-1.34	3.48E-02	2.89E-01	Distal Intergenic
Ldlr	300438	chr8	22704827	22705127	-45298	1.48	3.48E-02	2.89E-01	Intron (NM_134368/171379, intron 19 of 33)
Sumf1	362409	chr4	140161477	140161777	58705	1.74	3.48E-02	2.89E-01	Intron (NM_001108639/362409, intron 7 of 8)
Lrp11	292462	chr1	1702874	1703174	178	1.24	3.48E-02	2.89E-01	Promoter (<=1kb)
Etv4	360635	chr10	89692191	89692491	7792	-1.78	3.48E-02	2.89E-01	Exon (NM_001108299/360635, exon 5 of 13)
Hcn2	114244	chr7	12871379	12871679	-1292	0.9	3.49E-02	2.89E-01	Promoter (1-2kb)
Itgb8	362800	chr6	147168259	147168559	4254	-1.65	3.49E-02	2.89E-01	Intron (NM_001108726/362800, intron 1 of 11)
Gls	24398	chr9	54229492	54229792	16725	-1.66	3.49E-02	2.89E-01	Intron (NM_001109968/24398, intron 3 of 14)
Bicc1	361832	chr20	18775133	18775433	-5172	1.8	3.49E-02	2.89E-01	Distal Intergenic
Rnf182	498726	chr17	24325392	24325692	82750	-1.46	3.49E-02	2.89E-01	Distal Intergenic
Cdon	50938	chr8	36625586	36625886	-30758	1.03	3.49E-02	2.89E-01	Distal Intergenic
Osbpl11	303888	chr11	70841145	70841445	-7416	0.85	3.49E-02	2.89E-01	Distal Intergenic
Fam172a	294606	chr2	4942888	4943188	86	0.62	3.49E-02	2.89E-01	Promoter (<=1kb)
Ap4e1	311404	chr3	119480716	119481016	-3698	1.64	3.49E-02	2.89E-01	Distal Intergenic
Ifngr1	116465	chr1	14962943	14963243	-99137	-1.11	3.49E-02	2.89E-01	Distal Intergenic
Dlg1	25252	chr11	72469978	72470278	-90995	1.01	3.49E-02	2.89E-01	Distal Intergenic
Nabp1	363227	chr9	55007348	55007648	-42546	-1.58	3.49E-02	2.89E-01	Distal Intergenic
Nus1	294400	chr20	33557112	33557412	60	0.76	3.49E-02	2.89E-01	Promoter (<=1kb)
Lcn2	170496	chr3	11408344	11408644	8890	-1.4	3.50E-02	2.89E-01	Intron (NM_001106568/296639, intron 17 of 17)
Mtmr12	310155	chr2	62267430	62267730	30840	1.55	3.50E-02	2.89E-01	Intron (NM_001012077/310155, intron 5 of 14)
Synpo	60324	chr18	55941663	55941963	-49953	1.68	3.50E-02	2.89E-01	Distal Intergenic
Cox7b	303393	chrX	77068676	77068976	3249	-1.49	3.50E-02	2.89E-01	Intron (NM_182819/303393, intron 1 of 2)
Nxnl2	689232	chr17	14229230	14229530	-170588	-1.39	3.50E-02	2.89E-01	Distal Intergenic
LOC307727	307727	chrX	10851822	10852122	40345	1.19	3.50E-02	2.89E-01	Distal Intergenic
Hspb3	78951	chr2	45590564	45590864	-72062	-1.62	3.50E-02	2.89E-01	Distal Intergenic
Sfi1	305467	chr14	83404817	83405117	0	0.51	3.50E-02	2.89E-01	Promoter (<=1kb)
Slc20a1	81826	chr3	121712551	121712851	-13225	-1.26	3.50E-02	2.89E-01	Distal Intergenic
Kif2a	84391	chr2	38430827	38431127	-222062	-1.45	3.50E-02	2.89E-01	Distal Intergenic
Plagl2	296281	chr3	148717701	148718001	4741	-1.53	3.50E-02	2.89E-01	Intron (NM_001106528/296281, intron 1 of 3)
Egr1	24330	chr18	27657618	27657918	0	0.81	3.50E-02	2.89E-01	Promoter (<=1kb)
Rabggta	58983	chr15	34400033	34400333	89	0.66	3.50E-02	2.89E-01	Promoter (<=1kb)
Tnrc6b	192178	chr7	121930754	121931054	116	0.86	3.50E-02	2.89E-01	Promoter (<=1kb)
Smad2	29357	chr18	72473318	72473618	-76601	0.9	3.50E-02	2.89E-01	Distal Intergenic
Kif13a	308173	chr17	18169900	18170200	17918	1.36	3.51E-02	2.89E-01	Intron (NM_001107462/308173, intron 1 of 36)
Atp11a	306600	chr16	82078960	82079260	20962	-1.12	3.51E-02	2.89E-01	Intron (NM_001107324/306600, intron 1 of 28)
Ldah	313949	chr6	33407214	33407514	0	0.54	3.51E-02	2.89E-01	Promoter (<=1kb)
Abca1	313210	chr5	69929454	69929754	22712	-1.35	3.51E-02	2.89E-01	Exon (NM_178095/313210, exon 4 of 49)

Topors	362501	chr5	56568869	56569169	-2366	1.83	3.51E-02	2.89E-01	Promoter (2-3kb)
Fam241b	294499	chr20	31688819	31689119	46996	-1.1	3.51E-02	2.89E-01	Distal Intergenic
Ngrn	499191	chr1	142046553	142046853	-3605	-1.43	3.51E-02	2.89E-01	Intron (NM_001134962/308762, intron 11 of 14)
Myh8	252942	chr10	53936362	53936662	117544	-1.28	3.51E-02	2.89E-01	Distal Intergenic
Snap25	25012	chr3	129662335	129662635	-34773	1.53	3.51E-02	2.89E-01	Distal Intergenic
Tmem14a	363206	chr9	27274989	27275289	-58667	0.7	3.51E-02	2.89E-01	Distal Intergenic
Sh3bp5	117186	chr16	7532942	7533242	4959	1.77	3.51E-02	2.89E-01	Intron (NM_054011/117186, intron 2 of 8)
Inafm1	100192314	chr1	78221880	78222180	-9530	-1.22	3.51E-02	2.89E-01	Distal Intergenic
Nsd3	290831	chr16	71236219	71236519	599	1.01	3.52E-02	2.89E-01	Promoter (<=1kb)
Pdlim3	114108	chr16	49574389	49574689	-68	1.82	3.52E-02	2.89E-01	Promoter (<=1kb)
Sh3bp1	300067	chr7	120118855	120119155	-6592	1.69	3.52E-02	2.89E-01	3' UTR
Znf474	498866	chr18	47632536	47632836	18682	1.78	3.52E-02	2.89E-01	Intron (NM_001109125/498866, intron 3 of 3)
B3galt2	686081	chr13	60304563	60304863	-131083	1.73	3.52E-02	2.89E-01	Distal Intergenic
Mir99a	100314019	chr11	15761307	15761607	-290546	0.81	3.52E-02	2.89E-01	Distal Intergenic
Ksr1	360573	chr10	66481816	66482116	34	1.05	3.52E-02	2.89E-01	Promoter (<=1kb)
Plagl2	296281	chr3	148722435	148722735	7	0.98	3.52E-02	2.89E-01	Promoter (<=1kb)
Lrrc8b	305135	chr14	5505267	5505567	-79777	-1.47	3.52E-02	2.90E-01	Distal Intergenic
Hip1	192154	chr12	24238275	24238575	57338	0.82	3.52E-02	2.90E-01	Intron (NM_001100475/192154, intron 1 of 30)
Arhgef6	363509	chrX	159808143	159808443	32361	-1.21	3.52E-02	2.90E-01	Intron (NM_001005565/363509, intron 2 of 21)
Tmem232	501199	chr9	113045445	113045745	192648	-1.24	3.52E-02	2.90E-01	Intron (NM_001270390/501199, intron 11 of 12)
Klf2	306330	chr16	19224085	19224385	652	1.18	3.52E-02	2.90E-01	Promoter (<=1kb)
Mospd1	317312	chrX	157748212	157748512	2178	-1.42	3.52E-02	2.90E-01	Promoter (2-3kb)
Rnf146	308051	chr1	30857018	30857318	-5899	1.25	3.53E-02	2.90E-01	Distal Intergenic
Bmp5	315824	chr8	82862240	82862540	192774	1.32	3.53E-02	2.90E-01	Intron (NM_001108168/315824, intron 1 of 6)
Ston1	360202	chr6	12354975	12355275	-7538	1.17	3.53E-02	2.90E-01	Distal Intergenic
Ubp1	301038	chr8	122197227	122197527	199	0.76	3.53E-02	2.90E-01	Promoter (<=1kb)
Foxe3	171302	chr5	133177448	133177748	547908	-1.63	3.53E-02	2.90E-01	Distal Intergenic
Chst13	500257	chr4	122282502	122282802	-44748	1.04	3.53E-02	2.90E-01	Distal Intergenic
Enah	360891	chr13	100413936	100414236	-8597	1.29	3.53E-02	2.90E-01	Distal Intergenic
LOC497899	497899	chr10	36716975	36717275	-374	0.73	3.53E-02	2.90E-01	Promoter (<=1kb)
Tanc1	311055	chr3	45749273	45749573	30692	-1.12	3.53E-02	2.90E-01	Intron (NM_001002854/311055, intron 1 of 25)
RGD1308706	291925	chr19	22699963	22700263	155	1.04	3.53E-02	2.90E-01	Promoter (<=1kb)
Hace1	361866	chr20	50652570	50652870	0	1.04	3.53E-02	2.90E-01	Promoter (<=1kb)
Sumo1	301442	chr9	66473673	66473973	9641	1.36	3.53E-02	2.90E-01	Intron (NM_001009672/301442, intron 1 of 4)
Mcam	78967	chr8	48471441	48471741	-1083	0.82	3.53E-02	2.90E-01	Promoter (1-2kb)
Cep76	291540	chr18	63394411	63394711	55	0.65	3.54E-02	2.90E-01	Promoter (<=1kb)
Mreg	501162	chr9	79440833	79441133	103891	1.39	3.54E-02	2.90E-01	Distal Intergenic
Irf8	292060	chr19	54433113	54433413	114650	-0.69	3.54E-02	2.90E-01	Distal Intergenic
RGD1359508	361941	chr2	128675378	128675678	-136	0.92	3.54E-02	2.90E-01	Promoter (<=1kb)
Stard13	498130	chr12	1116726	1117026	78553	-1.2	3.54E-02	2.90E-01	Intron (NM_001109060/498130, intron 1 of 13)
Foxa1	25098	chr6	78549297	78549597	72	0.67	3.54E-02	2.90E-01	Promoter (<=1kb)
Rtf1	366169	chr3	111476078	111476378	-2004	1.58	3.54E-02	2.90E-01	Promoter (2-3kb)
Bmpr2	140590	chr9	66555724	66556024	-12050	2.09	3.54E-02	2.90E-01	Distal Intergenic
Rdx	315655	chr8	56535921	56536221	-34507	-1.44	3.54E-02	2.90E-01	Distal Intergenic
Cnksr3	308113	chr1	43878553	43878853	5414	-1.07	3.54E-02	2.90E-01	Intron (NM_001012061/308113, intron 1 of 12)
Bhlhe40	79431	chr4	140750913	140751213	47294	0.79	3.54E-02	2.90E-01	Distal Intergenic
Efnb2	306636	chr16	86639206	86639506	8055	-1.45	3.54E-02	2.90E-01	Intron (NM_001107328/306636, intron 1 of 4)
Cep295nl	498028	chr10	107365005	107365305	11340	1.56	3.54E-02	2.90E-01	5' UTR
Ehd2	361512	chr1	77865531	77865831	39	0.7	3.54E-02	2.90E-01	Promoter (<=1kb)
Rbmx2	367930	chrX	135551101	135551401	170	0.8	3.54E-02	2.90E-01	Promoter (<=1kb)
Ttbk2	311349	chr3	112789114	112789414	0	0.77	3.54E-02	2.90E-01	Promoter (<=1kb)
Tmem184b	362959	chr7	120621704	120622004	14588	2.1	3.54E-02	2.90E-01	Intron (NM_001173370/362959, intron 1 of 8)
Mrps24	498406	chr14	85951581	85951881	10	0.89	3.54E-02	2.90E-01	Promoter (<=1kb)
Gatm	81660	chr3	114698430	114698730	29425	1.39	3.54E-02	2.90E-01	Distal Intergenic
Mbd6	362892	chr7	70577889	70578189	-742	0.56	3.55E-02	2.90E-01	Promoter (<=1kb)
Usf2	81817	chr1	89386323	89386623	-3318	-1.35	3.55E-02	2.90E-01	Intron (NM_032616/64355, intron 5 of 9)
Chpf	316533	chr9	82673476	82673776	122	0.97	3.55E-02	2.90E-01	Promoter (<=1kb)
Gdi2	29662	chr17	70325595	70325895	0	0.6	3.55E-02	2.90E-01	Promoter (<=1kb)
Msmo1	140910	chr16	26868608	26868908	9167	-1.35	3.55E-02	2.90E-01	Intron (NM_080886/140910, intron 2 of 5)
Megf9	313270	chr5	86667102	86667402	28986	-1.46	3.55E-02	2.90E-01	Intron (NM_001107940/313270, intron 1 of 5)
Dnajc24	362184	chr3	96068659	96068959	-2948	1.83	3.55E-02	2.90E-01	Promoter (2-3kb)
Fgfrl1	360903	chr14	2032303	2032603	-134	0.94	3.55E-02	2.90E-01	Promoter (<=1kb)
Tm2d1	362545	chr5	117065730	117066030	-26848	-1.39	3.55E-02	2.90E-01	Intron (NM_080398/140581, intron 7 of 43)
Grm1	24414	chr1	5123121	5123421	42435	-1.02	3.55E-02	2.90E-01	Intron (NM_001114330/24414, intron 2 of 9)
Zfp161	282825	chr9	117827750	117828050	88683	0.72	3.55E-02	2.90E-01	Distal Intergenic
Pip4k2a	116723	chr17	85700304	85700604	48239	-1.1	3.55E-02	2.90E-01	Intron (NM_053926/116723, intron 1 of 8)
Relt	361615	chr1	165816798	165817098	74529	-1.54	3.56E-02	2.90E-01	Intron (NM_001108494/361614, intron 1 of 8)
RGD1565059	499630	chr2	154508611	154508911	0	0.57	3.56E-02	2.90E-01	Promoter (<=1kb)
Prdx5	113898	chr1	222134507	222134807	32640	-0.81	3.56E-02	2.90E-01	Distal Intergenic
Wee1	308937	chr1	174808263	174808563	40303	-1.13	3.56E-02	2.90E-01	Distal Intergenic
Fbxo25	364637	chr16	80806341	80806641	20	0.72	3.56E-02	2.90E-01	Promoter (<=1kb)
Pdp1	54705	chr5	25584050	25584350	0	0.98	3.56E-02	2.90E-01	Promoter (<=1kb)
Zfp36l1	29344	chr6	103139235	103139535	173539	-0.84	3.56E-02	2.90E-01	Distal Intergenic
Antxr1	362393	chr4	119049354	119049654	81548	0.86	3.56E-02	2.90E-01	Intron (NM_001044249/362393, intron 10 of 17)
Col3a1	84032	chr9	52106723	52107023	83428	-1.37	3.56E-02	2.90E-01	Intron (NM_053488/85250, intron 41 of 55)
Dlx5	25431	chr4	32373682	32373982	18025	1.01	3.56E-02	2.90E-01	Distal Intergenic
Slc35g3	691976	chr10	56368249	56368549	0	1.92	3.56E-02	2.90E-01	Promoter (<=1kb)
Slc25a54	691448	chr2	211859525	211859825	-20437	-1.35	3.56E-02	2.90E-01	Distal Intergenic
Kremen1	114107	chr14	85484970	85485270	18391	1.18	3.56E-02	2.90E-01	Intron (NM_053649/114107, intron 1 of 8)
Nubpl	299008	chr6	72915067	72915367	23309	-1.23	3.56E-02	2.90E-01	Intron (NM_001185025/299008, intron 3 of 10)
Ppp1r12a	116670	chr7	51507264	51507564	7657	-1.31	3.57E-02	2.91E-01	Intron (NM_053890/116670, intron 1 of 23)
Wsb2	288692	chr12	44962499	44962799	0	0.92	3.57E-02	2.91E-01	Promoter (<=1kb)
Hoxc12	300262	chr7	144553648	144553948	5709	0.58	3.57E-02	2.91E-01	Distal Intergenic
Rgs19	59293	chr3	177201571	177201871	-46	0.66	3.57E-02	2.91E-01	Promoter (<=1kb)
Nedd4	25489	chr8	79339558	79339858	16150	-0.97	3.57E-02	2.91E-01	Intron (NM_012986/25489, intron 5 of 28)
Suclg2	362404	chr4	127824650	127824950	20	0.68	3.57E-02	2.91E-01	Promoter (<=1kb)
Man2a1	25478	chr9	112186273	112186573	-106815	1.45	3.57E-02	2.91E-01	Distal Intergenic
Serpine2	29366	chr9	85659531	85659831	-33437	-1.22	3.57E-02	2.91E-01	Distal Intergenic
Khsrp	171137	chr9	10013960	10014260	30	1.47	3.57E-02	2.91E-01	Promoter (<=1kb)
Hey2	155430	chr1	29240933	29241233	49763	-1.44	3.58E-02	2.91E-01	Distal Intergenic
Lrig2	310753	chr2	206858611	206858911	138608	-1.34	3.58E-02	2.91E-01	Distal Intergenic
Sdc3	116673	chr5	148860141	148860441	-62657	-1.34	3.58E-02	2.91E-01	Intron (NM_001108684/362609, intron 7 of 21)
Slit2	360272	chr14	67375908	67376208	-205547	-1.31	3.58E-02	2.91E-01	Distal Intergenic
Osbpl2	296461	chr3	175490453	175490753	-2945	1.13	3.58E-02	2.91E-01	Promoter (2-3kb)

Utrn	25600	chr1	6912843	6913143	56897	-1.51	3.58E-02	2.91E-01		Intron (NM_013070/25600, intron 2 of 74)
Uchl1	29545	chr14	43293337	43293637	-149395	1.93	3.58E-02	2.91E-01		Distal Intergenic
Cenpq	363198	chr9	23351827	23352127	-144	0.65	3.58E-02	2.91E-01		Promoter (<=1kb)
Srp14	296076	chr3	110249199	110249499	128	0.57	3.58E-02	2.91E-01		Promoter (<=1kb)
Zfp513	313913	chr6	26541526	26541826	3819	2.07	3.58E-02	2.91E-01		3' UTR
Efna5	116683	chr9	110328425	110328725	962	0.74	3.58E-02	2.91E-01		Promoter (<=1kb)
Setd6	291844	chr19	9765066	9765366	118	0.84	3.59E-02	2.91E-01		Promoter (<=1kb)
Cisd3	287661	chr10	85603109	85603409	-25202	0.83	3.59E-02	2.91E-01		Distal Intergenic
Otub1	293705	chr1	222459609	222459909	43	1.01	3.59E-02	2.92E-01		Promoter (<=1kb)
Rnf114	362277	chr3	164248785	164249085	49	1	3.59E-02	2.92E-01		Promoter (<=1kb)
Pla2g2e	298581	chr5	157316615	157316915	-10742	1.8	3.60E-02	2.92E-01		Distal Intergenic
Slc48a1	300191	chr7	139324962	139325262	53264	1.45	3.60E-02	2.92E-01		Distal Intergenic
Syncrip	363113	chr8	96117175	96117475	15263	-1.48	3.60E-02	2.92E-01		Intron (NM_001047916/363113, intron 4 of 8)
Mtch2	295922	chr3	79681695	79681995	3554	-1.35	3.60E-02	2.92E-01		Intron (NM_001106488/295922, intron 3 of 10)
Sgsm3	362963	chr7	122203551	122203851	19	0.65	3.60E-02	2.92E-01		Promoter (<=1kb)
Bid	64625	chr4	153457646	153457946	7301	-1.57	3.60E-02	2.92E-01		Intron (NM_022684/64625, intron 1 of 5)
Pik3cb	85243	chr8	107342602	107342902	4671	-1.43	3.60E-02	2.92E-01		Intron (NM_053481/85243, intron 2 of 21)
L3mbtl2	300320	chr7	122818092	122818392	-78550	0.91	3.60E-02	2.92E-01		Distal Intergenic
Tmem263	362857	chr7	24721361	24721661	-54060	1.66	3.60E-02	2.92E-01		Distal Intergenic
Rev1	316344	chr9	44925263	44925563	-164214	1.05	3.60E-02	2.92E-01		Intron (NM_001191887/363220, intron 9 of 21)
Zfp367	306695	chr17	1665761	1666061	5960	-1.22	3.61E-02	2.92E-01		Intron (NM_001012051/306695, intron 1 of 4)
Abcd2	84356	chr7	132200459	132200759	142410	1.01	3.61E-02	2.92E-01		Distal Intergenic
Mirlet7b	100313990	chr7	126592220	126592520	1593	1.69	3.61E-02	2.92E-01		Promoter (1-2kb)
Fcgr3a	304966	chr13	89410780	89411080	24761	0.58	3.61E-02	2.92E-01		Distal Intergenic
Fam219a	691024	chr5	57933681	57933981	13485	-1.36	3.61E-02	2.92E-01		Intron (NM_001109616/691024, intron 1 of 5)
Ubn1	302935	chr10	10725089	10725389	266	0.83	3.61E-02	2.93E-01		Promoter (<=1kb)
Phkb	361377	chr19	22272132	22272432	9346	1.56	3.61E-02	2.93E-01		Intron (NM_001014152/361377, intron 1 of 29)
Otulinl	310190	chr2	80688422	80688722	-279750	-1.28	3.61E-02	2.93E-01		Distal Intergenic
Bsn	29138	chr8	116965273	116965573	0	1.54	3.62E-02	2.93E-01		Promoter (<=1kb)
Zc3hav1	252832	chr4	66002017	66002317	59778	0.92	3.62E-02	2.93E-01		Distal Intergenic
LOC100294508	100294508	chr5	144800090	144800390	20409	1.24	3.62E-02	2.93E-01		Intron (NM_001159655/100294508, intron 2 of 20)
Slc25a33	691431	chr5	166726401	166726701	93	0.9	3.62E-02	2.93E-01		Promoter (<=1kb)
Arhgap22	306279	chr16	9560685	9560985	-2233	-1.11	3.62E-02	2.93E-01		Promoter (2-3kb)
Myo1d	25485	chr10	68063300	68063600	79264	-0.89	3.62E-02	2.93E-01		Intron (NM_012983/25485, intron 1 of 21)
Scoc	364981	chr19	24590393	24590693	23326	0.89	3.62E-02	2.93E-01		Intron (NM_001013235/364981, intron 1 of 3)
Foxp1	297480	chr4	131421108	131421408	176896	-1.31	3.62E-02	2.93E-01		Intron (NM_001034131/297480, intron 6 of 15)
Atg7	312647	chr4	146696276	146696576	97860	-1.2	3.62E-02	2.93E-01		Intron (NM_001012097/312647, intron 17 of 17)
Cluh	303300	chr10	61432892	61433192	20	0.93	3.62E-02	2.93E-01		Promoter (<=1kb)
Hipk3	83617	chr3	94334356	94334656	84392	1.7	3.62E-02	2.93E-01		Distal Intergenic
Rpl6	117042	chr12	40836403	40836703	44423	-1.13	3.62E-02	2.93E-01		Distal Intergenic
Cyth3	116693	chr12	12921448	12921748	-13676	-0.85	3.62E-02	2.93E-01		Distal Intergenic
Kcnh6	116745	chr10	94215805	94216105	8469	-1.34	3.62E-02	2.93E-01		Intron (NM_053937/116745, intron 4 of 12)
Akap13	293024	chr1	137014417	137014717	145	1.04	3.62E-02	2.93E-01		Promoter (<=1kb)
Ppp3ca	24674	chr2	242087842	242088142	178010	-1.52	3.62E-02	2.93E-01		Intron (NM_017041/24674, intron 2 of 13)
Sgcg	305941	chr15	41486941	41487241	108034	-0.94	3.63E-02	2.93E-01		Distal Intergenic
Tspan2	64521	chr2	205160339	205160639	0	0.76	3.63E-02	2.93E-01		Promoter (<=1kb)
Pacsin3	311187	chr3	80072404	80072704	0	0.71	3.63E-02	2.93E-01		Promoter (<=1kb)
Cerk	300129	chr7	127044031	127044331	13725	-1.4	3.63E-02	2.93E-01		Intron (NM_001134861/300129, intron 1 of 12)
Fpgt	310935	chr2	261399477	261399777	3124	-1.43	3.63E-02	2.93E-01		Intron (NM_199494/310935, intron 2 of 3)
Vmp1	192129	chr10	73969690	73969990	31905	-0.91	3.63E-02	2.93E-01		Intron (NM_138839/192129, intron 5 of 11)
Il12rb2	171334	chr4	98081427	98081727	37092	1.16	3.63E-02	2.93E-01		Intron (NM_001191750/171334, intron 7 of 14)
LOC499584	499584	chr2	109401368	109401668	-904695	-1.1	3.63E-02	2.93E-01		Distal Intergenic
Mybl2	296344	chr3	159421729	159422029	58	0.89	3.63E-02	2.93E-01		Promoter (<=1kb)
Adcy8	29241	chr7	105585167	105585467	7337	-1.3	3.64E-02	2.93E-01		Intron (NM_017142/29241, intron 1 of 17)
Hmces	500251	chr4	119889857	119890157	47840	1.15	3.64E-02	2.93E-01		Distal Intergenic
Ggta1l1	652927	chr3	14572387	14572687	-747	-1.34	3.64E-02	2.93E-01		Promoter (<=1kb)
Ube2i	25573	chr10	14642188	14642488	-7569	1.27	3.64E-02	2.93E-01		Distal Intergenic
Tmem243	499990	chr4	21920151	21920451	200	1.05	3.64E-02	2.93E-01		Promoter (<=1kb)
LOC367117	367117	chr8	86148385	86148685	138306	1.27	3.64E-02	2.93E-01		Distal Intergenic
Tex264	300988	chr8	115401112	115401412	-12936	1.59	3.64E-02	2.93E-01		Intron (NM_001134520/363135, intron 18 of 22)
Stim2	117087	chr14	59302421	59302721	21226	-1.56	3.64E-02	2.93E-01		Intron (NM_001105750/117087, intron 1 of 11)
Zbtb26	311910	chr3	21697788	21698088	0	0.6	3.64E-02	2.93E-01		Promoter (<=1kb)
Kat6a	306571	chr16	74023205	74023505	-2455	0.54	3.64E-02	2.93E-01		Promoter (2-3kb)
Prickle1	315259	chr7	134798938	134799238	-76723	1.14	3.65E-02	2.94E-01		Distal Intergenic
Mir3596a	100526642	chr8	45772197	45772497	-18830	-1.3	3.65E-02	2.94E-01		Intron (NR_126581/104845260, intron 3 of 3)
Thsd7a	500032	chr4	39102810	39103110	-3	0.67	3.65E-02	2.94E-01		Promoter (<=1kb)
Epyc	314772	chr7	38903557	38903857	6279	0.98	3.65E-02	2.94E-01		Intron (NM_001108088/314772, intron 3 of 7)
Pde6h	114248	chr4	171058773	171059073	100577	-1.59	3.65E-02	2.94E-01		Distal Intergenic
Myh9	100911597	chr7	119083420	119083720	-11698	1.72	3.65E-02	2.94E-01		Distal Intergenic
Crlf1	290655	chr16	20687711	20688011	-1394	1.73	3.65E-02	2.94E-01		Promoter (1-2kb)
Bcl3	680611	chr1	80733679	80733979	11014	-1.33	3.65E-02	2.94E-01		Exon (NM_001109422/680611, exon 4 of 9)
Map3k9	500690	chr6	105487289	105487589	31159	1.96	3.65E-02	2.94E-01		Intron (NM_001100872/500690, intron 2 of 10)
Akap6	64553	chr6	73738901	73739201	185691	2.04	3.65E-02	2.94E-01		Intron (NM_022618/64553, intron 3 of 13)
Chmp4bl1	679886	chr4	67001021	67001321	-58618	-1.44	3.65E-02	2.94E-01		Distal Intergenic
Taok2	64666	chr1	198315308	198315608	4467	-1.51	3.65E-02	2.94E-01		Exon (NM_022702/64666, exon 5 of 16)
Polr3f	311487	chr3	138684775	138685075	90	0.59	3.65E-02	2.94E-01		Promoter (<=1kb)
Ica1l	316432	chr9	66817021	66817321	8513	-1.28	3.65E-02	2.94E-01		Intron (NM_199400/316432, intron 2 of 10)
Spdya	192209	chr6	23542853	23543153	13	0.79	3.65E-02	2.94E-01		Promoter (<=1kb)
Pou6f2	681092	chr17	49056714	49057014	3035	1.05	3.66E-02	2.94E-01		Intron (NM_001101002/681092, intron 2 of 13)
Scara5	305974	chr15	49001433	49001733	-67447	-1.63	3.66E-02	2.94E-01		Distal Intergenic
Dab2	79128	chr2	55643738	55644038	-103318	1.47	3.66E-02	2.94E-01		Distal Intergenic
Gins1	499914	chr3	146693055	146693355	-2011	1.71	3.66E-02	2.94E-01		Promoter (2-3kb)
Aftph	305544	chr14	104900018	104900318	75277	0.6	3.66E-02	2.94E-01		Distal Intergenic
Mef2d	81518	chr2	187512471	187512771	307	0.84	3.66E-02	2.94E-01		Promoter (<=1kb)
Tusc2	501052	chr8	116319370	116319670	407	0.96	3.66E-02	2.94E-01		Promoter (<=1kb)
Plekhg5	310999	chr5	169259329	169259629	14551	0.78	3.66E-02	2.94E-01		Intron (NM_201272/310999, intron 1 of 21)
Chtf8	364996	chr19	39087703	39088003	0	1.02	3.66E-02	2.94E-01		Promoter (<=1kb)
Ptms	83801	chr4	157438975	157439275	232	1.53	3.66E-02	2.94E-01		Promoter (<=1kb)
Prim2	301323	chr9	38544245	38544545	8789	0.55	3.66E-02	2.94E-01		Intron (NM_001024762/301323, intron 3 of 14)
Tanc2	303599	chr10	93811533	93811833	164	0.73	3.66E-02	2.94E-01		Promoter (<=1kb)
Hdlbp	64474	chr9	100592007	100592307	32331	0.76	3.66E-02	2.94E-01		Intron (NM_172039/64474, intron 1 of 27)
Il34	498951	chr19	40453168	40453468	-437076	0.95	3.66E-02	2.94E-01		Distal Intergenic
Gask1b	310540	chr2	178696204	178696504	17163	1.7	3.66E-02	2.94E-01		Intron (NM_199105/310540, intron 2 of 4)
Wasf1	294568	chr20	48503117	48503417	351	1.27	3.67E-02	2.94E-01		Promoter (<=1kb)

Abhd17c	361601	chr1	146256668	146256968	32497	1.58	3.67E-02	2.94E-01	Intron (NM_001100736/361601, intron 1 of 2)
Cwf19l2	362804	chr6	19250004	19250304	279388	-1.37	3.67E-02	2.94E-01	Distal Intergenic
Chst11	314694	chr7	26859317	26859617	99	0.75	3.67E-02	2.94E-01	Promoter (<=1kb)
Rnf181	297337	chr4	100226655	100226955	1854	1.61	3.67E-02	2.94E-01	Promoter (1-2kb)
Stxbp6	362734	chr6	65286074	65286374	33153	1.27	3.67E-02	2.94E-01	Intron (NM_001191872/362734, intron 1 of 5)
RGD1562024	498699	chr17	8612970	8613270	6467	0.93	3.67E-02	2.94E-01	3' UTR
Nrp2	81527	chr9	69337679	69337979	-159455	1.34	3.67E-02	2.94E-01	Intron (NM_001191808/301455, intron 21 of 22)
Hsf2bp	499413	chr20	10790665	10790965	51405	-1.02	3.67E-02	2.94E-01	Intron (NM_001127683/499413, intron 7 of 8)
Nav2	171563	chr1	104773183	104773483	196594	1.21	3.67E-02	2.94E-01	Intron (NM_138529/171563, intron 6 of 37)
Rftn1	501095	chr9	12779314	12779614	38429	-1.33	3.67E-02	2.94E-01	Intron (NM_001135011/501095, intron 2 of 9)
Pde2a	81743	chr1	166564376	166564676	-19	1.22	3.67E-02	2.94E-01	Promoter (<=1kb)
Rad18	362412	chr4	144620305	144620605	0	0.67	3.67E-02	2.94E-01	Promoter (<=1kb)
Etaa1	498420	chr14	102438140	102438440	-144463	1.49	3.67E-02	2.94E-01	Distal Intergenic
Clptm1l	316916	chr1	32296650	32296950	240	0.69	3.68E-02	2.94E-01	Promoter (<=1kb)
Kcnj13	94341	chr9	94439055	94439355	55978	-1.6	3.68E-02	2.94E-01	Distal Intergenic
Csrnp1	363165	chr8	128700012	128700312	-34024	1	3.68E-02	2.94E-01	Distal Intergenic
Clstn1	313717	chr5	166533435	166533735	200	0.84	3.68E-02	2.94E-01	Promoter (<=1kb)
Prrc2a	294250	chr20	5144778	5145078	8321	1.45	3.68E-02	2.94E-01	Exon (NM_212462/294250, exon 17 of 30)
Ostf1	259275	chr1	234781941	234782241	32373	-1.38	3.68E-02	2.94E-01	Intron (NM_148892/259275, intron 2 of 10)
Hus1	498411	chr14	89057619	89057919	150906	-1.32	3.68E-02	2.94E-01	Distal Intergenic
Slc9a9	363115	chr8	102341127	102341427	37032	-1.42	3.68E-02	2.94E-01	Intron (NM_001271438/363115, intron 2 of 15)
Zfp879	497896	chr10	36336207	36336507	121	0.78	3.68E-02	2.94E-01	Promoter (<=1kb)
Itch	311567	chr3	150667595	150667895	-18864	-1.48	3.68E-02	2.94E-01	Distal Intergenic
Prkar2b	24679	chr6	51662223	51662523	-305840	1.29	3.68E-02	2.94E-01	Distal Intergenic
Hs6st1	316325	chr9	42670513	42670813	50507	-1.31	3.69E-02	2.94E-01	Distal Intergenic
Rnf13	681578	chr2	147858628	147858928	5205	-1.47	3.69E-02	2.94E-01	Intron (NM_001109444/681578, intron 1 of 9)
Vav2	296603	chr3	5970456	5970756	5488	-1.47	3.69E-02	2.94E-01	Intron (NM_001106563/296603, intron 1 of 27)
Mir378b	102465146	chr5	5466735	5467035	108664	1.31	3.69E-02	2.94E-01	Distal Intergenic
Mink1	303259	chr10	57210472	57210772	25125	-1.28	3.69E-02	2.94E-01	Intron (NM_001271136/303259, intron 1 of 32)
Paip1	365684	chr2	52301819	52302119	-51	1.3	3.69E-02	2.94E-01	Promoter (<=1kb)
Tmsb10	50665	chr4	100882735	100883035	240	0.66	3.69E-02	2.94E-01	Promoter (<=1kb)
Trmt44	305443	chr14	80381565	80381865	20043	0.85	3.69E-02	2.94E-01	Distal Intergenic
Prkd1	85421	chr6	71204357	71204657	144592	-1.5	3.69E-02	2.94E-01	Intron (NM_001276715/85421, intron 1 of 18)
Abhd13	306630	chr16	85330011	85330311	0	0.86	3.69E-02	2.94E-01	Promoter (<=1kb)
Tyw5	301419	chr9	64291708	64292008	-195443	0.81	3.69E-02	2.94E-01	Distal Intergenic
Zdhhc7	170906	chr19	52763327	52763627	-13020	1.23	3.69E-02	2.94E-01	Distal Intergenic
Me2	307270	chr18	69798759	69799059	-14660	0.85	3.69E-02	2.94E-01	Distal Intergenic
Hic1	303310	chr10	61942023	61942323	68451	-1.29	3.69E-02	2.94E-01	Intron (NM_001105808/287522, intron 13 of 18)
Gpc1	58920	chr9	99954027	99954327	-43948	1.5	3.69E-02	2.94E-01	Intron (NM_001009825/301618, intron 5 of 9)
Foxq1	64826	chr17	34228591	34228891	-1650	-1.5	3.69E-02	2.94E-01	Promoter (1-2kb)
Csrnp3	311093	chr3	51862058	51862358	-21201	-1.5	3.69E-02	2.94E-01	Distal Intergenic
Arrdc4	293019	chr1	129779696	129779996	-3420	0.76	3.69E-02	2.94E-01	Distal Intergenic
Socs6	307200	chr18	85927943	85928243	52590	-1.39	3.70E-02	2.94E-01	Distal Intergenic
Vta1	292640	chr1	8880682	8880982	-2546	1.16	3.70E-02	2.95E-01	Promoter (2-3kb)
Zfp472	314587	chr7	15273238	15273538	31089	1.54	3.70E-02	2.95E-01	Distal Intergenic
Irf1	24508	chr10	39139480	39139780	29950	-1.34	3.70E-02	2.95E-01	Distal Intergenic
Basp1	64160	chr2	76306101	76306401	1326225	-1.34	3.70E-02	2.95E-01	Distal Intergenic
Ube2q1	295252	chr2	189115997	189116297	9958	1.5	3.70E-02	2.95E-01	Distal Intergenic
Gsg1	312793	chr4	169035090	169035390	1560	0.66	3.71E-02	2.95E-01	Promoter (1-2kb)
Man2a1	25478	chr9	112212625	112212925	-80463	1.51	3.71E-02	2.95E-01	Distal Intergenic
Ssrp1	81785	chr3	72450648	72450948	2679	-1.26	3.71E-02	2.95E-01	Promoter (2-3kb)
Bhmt	81508	chr2	23139786	23140086	116072	-1.46	3.71E-02	2.95E-01	Distal Intergenic
Mlycd	85239	chr19	52033057	52033357	107	0.76	3.71E-02	2.95E-01	Promoter (<=1kb)
Mbd3	362834	chr7	12192239	12192539	13193	0.75	3.71E-02	2.95E-01	Distal Intergenic
Hmgcll1	367112	chr8	82893762	82894062	-176432	-1.36	3.71E-02	2.95E-01	Intron (NM_001108168/315824, intron 3 of 6)
Igfbp7	289560	chr14	32951666	32951966	-58334	0.79	3.71E-02	2.95E-01	Distal Intergenic
Tgfb2	81809	chr13	105140704	105141004	0	0.6	3.71E-02	2.95E-01	Promoter (<=1kb)
Ubl3	363869	chr12	7866212	7866512	274	0.92	3.71E-02	2.95E-01	Promoter (<=1kb)
Smad4	50554	chr18	69673398	69673698	-16025	-1.57	3.71E-02	2.95E-01	Distal Intergenic
Hopx	171160	chr14	33314504	33314804	-40003	1.14	3.71E-02	2.95E-01	Distal Intergenic
Aox3	493909	chr9	65000258	65000558	-13304	1.94	3.71E-02	2.95E-01	Intron (NM_019363/54349, intron 30 of 34)
Daam1	314212	chr6	94662905	94663205	26683	-1.43	3.71E-02	2.95E-01	Exon (NM_001108030/314212, exon 3 of 24)
Bcas3	363662	chr10	72769423	72769723	12875	-1.31	3.72E-02	2.95E-01	Intron (NM_001173430/363662, intron 2 of 17)
Abl1	311860	chr3	10111542	10111842	33234	-1.49	3.72E-02	2.95E-01	Intron (NM_001100850/311860, intron 1 of 10)
Lmo7	361084	chr15	86421673	86421973	178525	1.81	3.72E-02	2.95E-01	Exon (NM_001001515/361084, exon 14 of 31)
Prpf40a	295607	chr3	38741304	38741604	-191	0.54	3.72E-02	2.95E-01	Promoter (<=1kb)
Fry	304244	chr12	5689548	5689848	133026	1.66	3.72E-02	2.95E-01	Intron (NM_001170398/304244, intron 17 of 72)
Prag1	306506	chr16	59564998	59565298	155948	1.13	3.72E-02	2.95E-01	Distal Intergenic
Pex2	29534	chr2	97584804	97585104	-667821	1.42	3.72E-02	2.95E-01	Distal Intergenic
Eif4ebp3	100365872	chr18	29456843	29457143	-33838	1.89	3.72E-02	2.95E-01	Intron (NM_001204053/100529260, intron 15 of 33)
Fscn2	303741	chr10	109547923	109548223	14436	-1.46	3.72E-02	2.95E-01	Distal Intergenic
Cmas	312826	chr4	176994209	176994509	80	0.9	3.72E-02	2.95E-01	Promoter (<=1kb)
Ambra1	59319	chr3	80634578	80634878	108	0.77	3.72E-02	2.95E-01	Promoter (<=1kb)
Gtse1	300126	chr7	126743967	126744267	7235	-1.16	3.73E-02	2.96E-01	Exon (NM_001130500/300126, exon 6 of 11)
Nr3c2	25672	chr19	34758822	34759122	1881	0.55	3.73E-02	2.96E-01	Promoter (1-2kb)
Cox7c	100188937	chr2	14701302	14701602	301	0.6	3.73E-02	2.96E-01	Promoter (<=1kb)
Fbxo30	308283	chr1	5366274	5366574	0	1.06	3.73E-02	2.96E-01	Promoter (<=1kb)
Evpl	303687	chr10	105129463	105129763	-12661	1.18	3.73E-02	2.96E-01	Intron (NM_001108840/363707, intron 10 of 15)
Sox4	364712	chr17	37610102	37610402	-4620	0.61	3.73E-02	2.96E-01	Distal Intergenic
LOC100361087	100361087	chr13	88610382	88610682	31329	-1.51	3.73E-02	2.96E-01	Distal Intergenic
Frs2	314850	chr7	60181576	60181876	28606	-1.36	3.73E-02	2.96E-01	Intron (NM_001108097/314850, intron 1 of 9)
Fyb1	499537	chr2	56014068	56014368	122526	1.19	3.73E-02	2.96E-01	Distal Intergenic
Zfp367	306695	chr17	1671409	1671709	312	0.88	3.73E-02	2.96E-01	Promoter (<=1kb)
Ubash3b	315579	chr8	45495234	45495534	-120100	-1.16	3.73E-02	2.96E-01	Distal Intergenic
Naca	288770	chr7	2469072	2469372	9931	-1.33	3.73E-02	2.96E-01	Exon (NM_001198562/288770, exon 4 of 8)
Nup50	25497	chr7	125833853	125834153	296	0.94	3.73E-02	2.96E-01	Promoter (<=1kb)
Fundc2	361288	chr18	402401	402701	106	0.58	3.74E-02	2.96E-01	Promoter (<=1kb)
Ankrd2	309374	chr1	261268285	261268585	-13274	-1.32	3.74E-02	2.96E-01	Intron (NM_001013153/309373, intron 1 of 2)
Itgbl1	498564	chr15	110217801	110218101	103653	1.97	3.74E-02	2.96E-01	Intron (NM_001017505/498564, intron 2 of 10)
Arhgap35	306400	chr1	78540751	78541051	32323	-1.44	3.74E-02	2.96E-01	Intron (NM_001271132/306400, intron 1 of 6)
Kcne4	367302	chr9	84690123	84690423	973	-1.28	3.74E-02	2.96E-01	Promoter (<=1kb)
Egfl7	245963	chr3	4021785	4022085	-12860	1.19	3.74E-02	2.96E-01	Distal Intergenic
Mir30d	100314010	chr7	109287822	109288122	-1278	1.83	3.74E-02	2.96E-01	Promoter (1-2kb)
Mtfr2	100911069	chr1	15831933	15832233	-2546	1.66	3.74E-02	2.96E-01	Promoter (2-3kb)

Rin2	311494	chr3	139954092	139954392	59761	-0.94	3.74E-02	2.96E-01	Intron (NM_001107786/311494, intron 2 of 12)
Pltp	296371	chr3	161315665	161315965	6324	1.55	3.74E-02	2.96E-01	Intron (NM_001168543/296371, intron 8 of 15)
Med16	299607	chr7	12619709	12620009	0	0.66	3.74E-02	2.96E-01	Promoter (<=1kb)
Mrpl17	171061	chr1	170653561	170653861	121	0.61	3.74E-02	2.96E-01	Promoter (<=1kb)
Actg1	287876	chr10	109521497	109521797	-209	0.81	3.74E-02	2.96E-01	Promoter (<=1kb)
Tkt	64524	chr16	6593817	6594117	-15553	-1.38	3.75E-02	2.96E-01	Distal Intergenic
Hcn4	59266	chr8	63599518	63599818	-89	0.96	3.75E-02	2.96E-01	Promoter (<=1kb)
Rbm17	291295	chr17	70586458	70586758	64	1.06	3.75E-02	2.96E-01	Promoter (<=1kb)
RGD1561161	294747	chr2	45278753	45279053	96362	-1.48	3.75E-02	2.96E-01	Distal Intergenic
Rab14	94197	chr3	14409122	14409422	-23593	-1.48	3.75E-02	2.96E-01	Distal Intergenic
Cdkn2aip	306455	chr16	47608734	47609034	-56782	1.54	3.75E-02	2.96E-01	Distal Intergenic
Cyp1b1	25426	chr6	2350230	2350530	-33508	-0.85	3.75E-02	2.96E-01	Distal Intergenic
Lmnb1	116685	chr18	51829262	51829562	44138	-1.55	3.75E-02	2.96E-01	Distal Intergenic
Relt	361615	chr1	165811798	165812098	79529	-1.44	3.75E-02	2.96E-01	Intron (NM_001108494/361614, intron 1 of 8)
Serpini1	116459	chr2	174028260	174028560	14834	-1.44	3.75E-02	2.96E-01	Intron (NM_053779/116459, intron 1 of 8)
Zfp330	361387	chr19	23946333	23946633	267	0.59	3.75E-02	2.96E-01	Promoter (<=1kb)
Pdhb	289950	chr15	18563068	18563368	22155	1.9	3.75E-02	2.96E-01	Exon (NM_182821/306203, exon 15 of 18)
Grasp	192254	chr7	142869824	142870124	39	0.86	3.76E-02	2.96E-01	Promoter (<=1kb)
Fmo9	685365	chr13	85109246	85109546	-314165	1.25	3.76E-02	2.96E-01	Distal Intergenic
Pald1	294508	chr20	31001098	31001398	-25626	0.73	3.76E-02	2.96E-01	Distal Intergenic
Rad18	362412	chr4	144687742	144688042	-67158	1.44	3.76E-02	2.96E-01	Exon (NM_001191975/500287, exon 9 of 22)
Lrrc26	311803	chr3	2508584	2508884	2158	1.05	3.76E-02	2.96E-01	Promoter (2-3kb)
Plekhn1	298694	chr5	173653039	173653339	566	-1.35	3.76E-02	2.96E-01	Promoter (<=1kb)
Nsdhl	309262	chrX	152932718	152933018	-108	0.73	3.76E-02	2.96E-01	Promoter (<=1kb)
Klf9	117560	chr1	240908651	240908951	168	1	3.76E-02	2.96E-01	Promoter (<=1kb)
MGC108823	307414	chr18	55605863	55606163	29624	-1.43	3.77E-02	2.96E-01	Distal Intergenic
Smim15	100359861	chr2	39058054	39058354	-256360	1.7	3.77E-02	2.96E-01	Distal Intergenic
Rassf9	65053	chr7	44175925	44176225	29580	1.7	3.77E-02	2.96E-01	Intron (NM_022959/65053, intron 1 of 1)
RGD1308147	307008	chr17	53088797	53089097	-1523	-1.42	3.77E-02	2.96E-01	Promoter (1-2kb)
Rab12	25530	chr9	114619828	114620128	89485	1.15	3.77E-02	2.96E-01	Distal Intergenic
Clec2e	689853	chr4	162868888	162869188	-12457	-1.09	3.77E-02	2.96E-01	Distal Intergenic
Tmem37	245953	chr13	36100155	36100455	658	0.98	3.77E-02	2.96E-01	Promoter (<=1kb)
Trim9	155812	chr6	92745268	92745568	14450	-1.41	3.77E-02	2.96E-01	Intron (NM_130420/155812, intron 1 of 9)
Stk10	29398	chr10	17447473	17447773	26504	-1.17	3.77E-02	2.96E-01	Intron (NM_019206/29398, intron 2 of 20)
Ppp3cc	171378	chr15	51953978	51954278	13619	0.86	3.77E-02	2.96E-01	Intron (NM_134367/171378, intron 1 of 13)
Trpm7	679906	chr3	119346570	119346870	214	0.7	3.77E-02	2.96E-01	Promoter (<=1kb)
Zfp347	170902	chr7	9866896	9867196	123764	-1.1	3.77E-02	2.96E-01	Distal Intergenic
Mir761	100314211	chr5	128743752	128744052	91885	-1.29	3.77E-02	2.96E-01	Intron (NM_001044234/298369, intron 4 of 23)
Ftl1	29292	chr1	101439485	101439785	10249	-1.21	3.77E-02	2.96E-01	Exon (NM_001109615/690987, exon 12 of 16)
Cald1	25687	chr4	62255587	62255887	34576	1.18	3.77E-02	2.96E-01	Intron (NM_013146/25687, intron 2 of 11)
Ptges3	362809	chr7	2479531	2479831	220	0.62	3.77E-02	2.96E-01	Promoter (<=1kb)
Tprg1	360731	chr11	78771787	78772087	89658	-1.24	3.78E-02	2.96E-01	Intron (NM_001108320/360731, intron 4 of 5)
Btn1a1	306956	chr17	43914267	43914567	13582	0.61	3.78E-02	2.96E-01	Distal Intergenic
Dop1b	304077	chr11	33925499	33925799	-3343	1.17	3.78E-02	2.96E-01	Distal Intergenic
Slc20a1	81826	chr3	121738432	121738732	12356	-1.41	3.78E-02	2.96E-01	3' UTR
Bicdl1	304537	chr12	46614718	46615018	-631	2.07	3.78E-02	2.96E-01	Promoter (<=1kb)
Pccb	24624	chr8	109469334	109469634	-50463	-1.42	3.78E-02	2.96E-01	Distal Intergenic
Hadhb	171155	chr6	27583120	27583420	6119	-1.33	3.78E-02	2.96E-01	Intron (NM_133618/171155, intron 1 of 15)
Smad7	81516	chr18	71479414	71479714	83584	1.06	3.78E-02	2.96E-01	Intron (NM_001108891/364900, intron 3 of 4)
Mtmr1	317296	chr18	325085	325385	0	0.95	3.78E-02	2.96E-01	Promoter (<=1kb)
Sfpq	252855	chr5	145080264	145080564	461	0.82	3.78E-02	2.96E-01	Promoter (<=1kb)
Dctn2	299850	chr7	70556018	70556318	0	0.64	3.78E-02	2.96E-01	Promoter (<=1kb)
Ifi44	310969	chr2	256914890	256915190	373	-0.77	3.78E-02	2.96E-01	Promoter (<=1kb)
Dip2a	690211	chr20	13044101	13044401	45	0.82	3.78E-02	2.96E-01	Promoter (<=1kb)
N4bp2l1	498131	chr12	567827	568127	8617	-1.47	3.78E-02	2.96E-01	Intron (NM_001035222/498131, intron 4 of 7)
Cobl	305497	chr14	92524745	92525045	-28851	-1.47	3.78E-02	2.96E-01	Distal Intergenic
Wdyhv1	362914	chr7	97972782	97973082	4053	-1.47	3.78E-02	2.96E-01	Intron (NM_001025024/362914, intron 1 of 5)
Lrrfip2	301035	chr8	119431470	119431770	49459	-1.47	3.78E-02	2.96E-01	Intron (NM_001024761/301035, intron 5 of 15)
Dvl1	83721	chr5	173296175	173296475	0	0.92	3.78E-02	2.96E-01	Promoter (<=1kb)
Lrrc14	500900	chr7	117780107	117780407	6159	0.65	3.78E-02	2.96E-01	Exon (NM_001135896/362945, exon 7 of 7)
Pkdcc	313860	chr6	6426528	6426828	-829	0.82	3.78E-02	2.96E-01	Promoter (<=1kb)
Prkag2	373545	chr4	6767941	6768241	14513	1.51	3.78E-02	2.96E-01	Intron (NM_184051/373545, intron 1 of 11)
Ppp1r2	192361	chr11	72748804	72749104	15	0.64	3.78E-02	2.96E-01	Promoter (<=1kb)
Tapbp	25217	chr20	5471992	5472292	3715	-1.33	3.78E-02	2.97E-01	Intron (NM_033098/25217, intron 3 of 7)
Arntl	29657	chr1	177998324	177998624	-40439	-1.25	3.78E-02	2.97E-01	Distal Intergenic
Siva1	362791	chr6	137150975	137151275	-58846	-1.11	3.79E-02	2.97E-01	Distal Intergenic
Arrdc4	293019	chr1	130012602	130012902	-236326	-1.13	3.79E-02	2.97E-01	Distal Intergenic
Rab33b	365793	chr2	140546045	140546345	4426	-1.35	3.79E-02	2.97E-01	Intron (NM_001108944/365793, intron 1 of 1)
Pfdn1	361310	chr18	29250179	29250479	39968	1.65	3.79E-02	2.97E-01	Intron (NM_001108427/361310, intron 3 of 3)
Ripk2	362491	chr5	29916859	29917159	-46469	-1.18	3.79E-02	2.97E-01	Distal Intergenic
Bin2	366988	chr7	142295105	142295405	4977	-1.16	3.79E-02	2.97E-01	Intron (NM_001012223/366988, intron 1 of 10)
Kmt2e	311968	chr4	8255505	8255805	0	0.56	3.79E-02	2.97E-01	Promoter (<=1kb)
Map11	288545	chr12	19578713	19579013	3172	1.63	3.79E-02	2.97E-01	Exon (NM_001024969/288545, exon 10 of 11)
Gas6	58935	chr16	81243467	81243767	0	0.73	3.79E-02	2.97E-01	Promoter (<=1kb)
Ptf1a	117034	chr17	86435829	86436129	236206	0.75	3.79E-02	2.97E-01	Distal Intergenic
Galc	314360	chr6	122239057	122239357	54	0.61	3.79E-02	2.97E-01	Promoter (<=1kb)
Krcc1	312437	chr4	99133968	99134268	126	0.69	3.80E-02	2.97E-01	Promoter (<=1kb)
Rps23	124323	chr2	19823237	19823537	3	0.69	3.80E-02	2.97E-01	Promoter (<=1kb)
Sppl2b	362828	chr7	11724527	11724827	16	0.65	3.80E-02	2.97E-01	Promoter (<=1kb)
Kif13a	308173	chr17	18206178	18206478	54196	-1.33	3.80E-02	2.97E-01	Intron (NM_001107462/308173, intron 1 of 36)
Rps6ka1	81771	chr5	152052816	152053116	69568	-1.16	3.80E-02	2.97E-01	Distal Intergenic
Setd5	297514	chr4	145034587	145034887	16979	-1.44	3.80E-02	2.97E-01	Intron (NM_001106614/297514, intron 1 of 23)
Wdtc1	313020	chr5	151531426	151531726	4792	1.95	3.80E-02	2.97E-01	Intron (NM_001107908/313020, intron 1 of 16)
Xrcc4	309995	chr2	18931896	18932196	-4531	1.73	3.80E-02	2.97E-01	Distal Intergenic
Med8	362575	chr5	137189535	137189835	-53259	0.81	3.80E-02	2.97E-01	Distal Intergenic
Jak1	84598	chr5	120079556	120079856	4048	1.75	3.80E-02	2.97E-01	Intron (NM_053466/84598, intron 1 of 24)
Pex14	64460	chr5	165878301	165878601	39844	1.45	3.80E-02	2.97E-01	Intron (NM_172063/64460, intron 2 of 8)
Col5a2	85250	chr9	52220428	52220728	18007	-1.39	3.80E-02	2.97E-01	Intron (NM_053488/85250, intron 1 of 55)
Sema4f	29745	chr4	113731407	113731707	32825	1.41	3.81E-02	2.97E-01	Distal Intergenic
Pank2	296167	chr3	123808010	123808310	58	0.97	3.81E-02	2.97E-01	Promoter (<=1kb)
Maff	366960	chr7	120586664	120586964	5921	-1.48	3.81E-02	2.97E-01	Intron (NM_001130573/366960, intron 1 of 2)
Clic4	83718	chr5	153657290	153657590	-31621	-1.39	3.81E-02	2.97E-01	Distal Intergenic
Ptpn9	266611	chr8	61691643	61691943	32198	-1.26	3.81E-02	2.98E-01	Intron (NM_001013040/266611, intron 1 of 12)
Tmx1	362751	chr6	92874691	92874991	10568	1.26	3.82E-02	2.98E-01	Distal Intergenic

Sh3d21	362598	chr5	144185430	144185730	-8656	1.7	3.82E-02	2.98E-01	Intron (NM_001009693/313591, intron 8 of 11)
Lum	81682	chr7	38820371	38820671	313	1.23	3.82E-02	2.98E-01	Promoter (<=1kb)
Foxe3	171302	chr5	133734169	133734469	-8513	-1.28	3.82E-02	2.98E-01	Distal Intergenic
Srsf3	361814	chr20	6313327	6313627	25042	1.6	3.82E-02	2.98E-01	Distal Intergenic
Slc25a15	306574	chr16	74531574	74531874	-2196	0.84	3.82E-02	2.98E-01	Promoter (2-3kb)
Sptbn1	305614	chr14	114699501	114699801	-6737	1.12	3.82E-02	2.98E-01	Distal Intergenic
Dbf4	312046	chr4	22661914	22662214	0	0.62	3.82E-02	2.98E-01	Promoter (<=1kb)
Ctnnd1	311163	chr3	72023997	72024297	28693	-1.37	3.82E-02	2.98E-01	Exon (NM_001107740/311163, exon 3 of 18)
Adam19	303068	chr10	31175406	31175706	29299	-1.44	3.82E-02	2.98E-01	Intron (NM_001160228/303068, intron 3 of 22)
Crlf3	54395	chr10	67325307	67325607	76231	0.9	3.82E-02	2.98E-01	Distal Intergenic
Slc35f5	288993	chr13	41903876	41904176	20739	-1.4	3.82E-02	2.98E-01	Intron (NM_001105950/288993, intron 8 of 15)
Xpo4	290280	chr15	37926511	37926811	0	0.87	3.82E-02	2.98E-01	Promoter (<=1kb)
Ext1	299907	chr7	92968380	92968680	-86988	-1.3	3.82E-02	2.98E-01	Distal Intergenic
Rtn4	83765	chr14	114127377	114127677	411	1.36	3.83E-02	2.98E-01	Promoter (<=1kb)
Arl5a	117050	chr3	37894000	37894300	53134	-1.18	3.83E-02	2.98E-01	Distal Intergenic
Rhno1	297627	chr4	161670098	161670398	14596	-1.54	3.83E-02	2.98E-01	Distal Intergenic
Pabpc1	171350	chr7	75417519	75417819	4055	-1.35	3.83E-02	2.98E-01	Intron (NM_134353/171350, intron 3 of 14)
Arhgap22	306279	chr16	9529504	9529804	-33414	-1.37	3.83E-02	2.98E-01	Distal Intergenic
Cd55	64036	chr13	47153136	47153436	85	0.71	3.83E-02	2.98E-01	Promoter (<=1kb)
Nptn	56064	chr8	63407661	63407961	28574	-1.5	3.84E-02	2.98E-01	Intron (NM_019380/56064, intron 1 of 8)
RGD1307947	314788	chr7	40315119	40315419	0	0.6	3.84E-02	2.98E-01	Promoter (<=1kb)
Map1a	25152	chr3	113231410	113231710	-25978	0.57	3.84E-02	2.98E-01	Distal Intergenic
Top2b	361100	chr15	10319731	10320031	0	1.12	3.84E-02	2.99E-01	Promoter (<=1kb)
Pak1	29431	chr1	162768339	162768639	183	0.86	3.84E-02	2.99E-01	Promoter (<=1kb)
Fgd3	361223	chr17	15736036	15736336	-13627	-1.21	3.84E-02	2.99E-01	Distal Intergenic
Dhrs3	313689	chr5	162809495	162809795	405	0.72	3.84E-02	2.99E-01	Promoter (<=1kb)
LOC688765	688765	chr16	58838653	58838953	-106980	-1.16	3.84E-02	2.99E-01	Distal Intergenic
Psmb4	58854	chr2	195997286	195997586	48734	0.92	3.84E-02	2.99E-01	Distal Intergenic
Hace1	361866	chr20	50630459	50630759	-21986	-1.33	3.84E-02	2.99E-01	Distal Intergenic
Tfdp1	361178	chr16	81088746	81089046	-262	0.86	3.84E-02	2.99E-01	Promoter (<=1kb)
Sema5a	310207	chr2	85381967	85382267	4649	-1.45	3.85E-02	2.99E-01	Intron (NM_001107659/310207, intron 1 of 22)
Cmtm7	501065	chr8	122840681	122840981	498	0.67	3.85E-02	2.99E-01	Promoter (<=1kb)
Sdcbp	83841	chr5	19471637	19471937	0	0.69	3.85E-02	2.99E-01	Promoter (<=1kb)
Ttc1	287208	chr10	29364420	29364720	3145	-1.12	3.85E-02	2.99E-01	Intron (NM_001005529/287208, intron 2 of 7)
Thbs1	445442	chr3	109833660	109833960	-28160	1.77	3.85E-02	2.99E-01	Distal Intergenic
Fbxo6	192351	chr5	164961478	164961778	-1631	0.92	3.85E-02	2.99E-01	Promoter (1-2kb)
Rsl24d1	363099	chr8	79811639	79811939	19052	1.62	3.85E-02	2.99E-01	Distal Intergenic
Caprin1	362173	chr3	93733655	93733955	327	0.69	3.85E-02	2.99E-01	Promoter (<=1kb)
Zfp36l2	298765	chr6	7421676	7421976	-220	0.62	3.85E-02	2.99E-01	Promoter (<=1kb)
Pigb	315807	chr8	79715031	79715331	0	0.59	3.85E-02	2.99E-01	Promoter (<=1kb)
Smad2	29357	chr18	72408254	72408554	-141665	-1.36	3.85E-02	2.99E-01	Intron (NM_001127375/679155, intron 3 of 5)
Slc16a5	690212	chr10	104017508	104017808	-2080	-1.26	3.85E-02	2.99E-01	Promoter (2-3kb)
Creb3l2	362339	chr4	64975805	64976105	5414	-1.56	3.85E-02	2.99E-01	Intron (NM_001012188/362339, intron 1 of 11)
Ythdf1	296467	chr3	176431030	176431330	93	0.91	3.85E-02	2.99E-01	Promoter (<=1kb)
Luzp1	79428	chr5	154856934	154857234	25196	-1.41	3.85E-02	2.99E-01	Intron (NM_030830/79428, intron 1 of 4)
Tnr	25567	chr13	77642961	77643261	40712	-0.99	3.85E-02	2.99E-01	Intron (NM_013045/25567, intron 16 of 25)
Jag1	29146	chr3	130108684	130108984	5797	-1.34	3.85E-02	2.99E-01	Intron (NM_019147/29146, intron 2 of 27)
Dusp10	63995	chr13	104383869	104384169	99209	-1.11	3.85E-02	2.99E-01	Distal Intergenic
Gins2	292058	chr19	54192982	54193282	-29374	1.84	3.86E-02	2.99E-01	Distal Intergenic
Rps6ka4	361715	chr1	222110050	222110350	-11766	0.72	3.86E-02	2.99E-01	Distal Intergenic
Gjd4	266707	chr17	62290923	62291223	8432	-1.21	3.86E-02	2.99E-01	Distal Intergenic
Foxq1	64826	chr17	34218641	34218941	8000	-1.4	3.86E-02	2.99E-01	Distal Intergenic
Uvrag	308846	chr1	164003117	164003417	98151	1.56	3.86E-02	2.99E-01	Intron (NM_001107536/308846, intron 6 of 13)
Mafk	246760	chr12	16987257	16987557	-52551	-1.01	3.86E-02	2.99E-01	Distal Intergenic
Coro2a	313235	chr5	62210530	62210830	-22600	1.11	3.86E-02	2.99E-01	Downstream (2-3kb)
Txndc5	100362805	chr17	26925872	26926172	44	1.14	3.86E-02	2.99E-01	Promoter (<=1kb)
Osbpl5	361686	chr1	216908544	216908844	11781	-1.11	3.86E-02	2.99E-01	Intron (NM_001015024/361686, intron 1 of 21)
Nkiras2	287707	chr10	88567889	88568189	31183	1.2	3.86E-02	2.99E-01	Distal Intergenic
Usp34	360990	chr14	108060442	108060742	0	1.31	3.87E-02	2.99E-01	Promoter (<=1kb)
RGD1304694	362974	chr7	125892660	125892960	208	0.68	3.87E-02	2.99E-01	Promoter (<=1kb)
Vom2r80	502285	chr1	62267418	62267718	-144320	0.65	3.87E-02	2.99E-01	Distal Intergenic
Emb	114511	chr2	49448114	49448414	-234349	-1.4	3.87E-02	2.99E-01	Distal Intergenic
Gmps	295088	chr2	154555931	154556231	0	0.63	3.87E-02	2.99E-01	Promoter (<=1kb)
Cdpf1	362975	chr7	126691495	126691795	-263	0.54	3.87E-02	2.99E-01	Promoter (<=1kb)
Tmem86a	308602	chr1	103128371	103128671	-44361	0.65	3.87E-02	2.99E-01	Distal Intergenic
Fkbp4	260321	chr4	161757153	161757453	0	0.85	3.87E-02	2.99E-01	Promoter (<=1kb)
Slc4a3	24781	chr9	82742633	82742933	-95	0.62	3.87E-02	2.99E-01	Promoter (<=1kb)
Fcf1	299198	chr6	108798226	108798526	2044	-1.4	3.87E-02	2.99E-01	Promoter (2-3kb)
Uso1	56042	chr14	17427965	17428265	8632	-1.53	3.87E-02	2.99E-01	Intron (NM_019379/56042, intron 1 of 23)
Plekhf1	308543	chr1	94561508	94561808	49036	1.22	3.88E-02	2.99E-01	Distal Intergenic
Zfp24	360204	chr18	15923619	15923919	-1938	-1.23	3.88E-02	2.99E-01	Promoter (1-2kb)
Slc25a1	29743	chr11	87204355	87204655	107	0.91	3.88E-02	2.99E-01	Promoter (<=1kb)
Inhbb	25196	chr13	35443461	35443761	-1239	1.1	3.88E-02	2.99E-01	Promoter (1-2kb)
Gnaq	81666	chr1	233382863	233383163	85	1.14	3.88E-02	3.00E-01	Promoter (<=1kb)
Erc1	266806	chr4	152379791	152380091	0	0.68	3.88E-02	3.00E-01	Promoter (<=1kb)
Sgsm1	288743	chr12	49432028	49432328	102594	1.18	3.88E-02	3.00E-01	Distal Intergenic
Cwc27	361887	chr2	34668121	34668421	254300	-1.36	3.88E-02	3.00E-01	Distal Intergenic
Plec	64204	chr7	117248322	117248622	6143	0.68	3.88E-02	3.00E-01	Exon (NM_001164308/64204, exon 13 of 32)
Anp32a	25379	chr8	67291355	67291655	-4075	-1.32	3.88E-02	3.00E-01	Distal Intergenic
Hibadh	63938	chr4	82686966	82687266	15163	-1.15	3.88E-02	3.00E-01	Intron (NM_022243/63938, intron 2 of 7)
Hibadh	63938	chr4	82702146	82702446	0	0.61	3.89E-02	3.00E-01	Promoter (<=1kb)
Smad7	81516	chr18	71396997	71397297	1167	1.07	3.89E-02	3.00E-01	Promoter (1-2kb)
Abca8a	303638	chr10	98470647	98470947	-80263	0.88	3.89E-02	3.00E-01	Distal Intergenic
Tbrg4	360977	chr14	86881500	86881800	1837	-1.39	3.89E-02	3.00E-01	Promoter (1-2kb)
Smad5	59328	chr17	8336346	8336646	-13926	0.8	3.89E-02	3.00E-01	Distal Intergenic
Fbxl3	306129	chr15	93661718	93662018	5377	-1.49	3.89E-02	3.00E-01	Intron (NM_001100568/306129, intron 2 of 4)
Tmem154	361972	chr2	183811577	183811877	137055	0.77	3.89E-02	3.00E-01	Distal Intergenic
Cyp11a1	29680	chr8	62779904	62780204	-18113	-1.16	3.89E-02	3.00E-01	Distal Intergenic
Serinc2	313057	chr5	148488941	148489241	2991	-1.51	3.89E-02	3.00E-01	Promoter (2-3kb)
Atp6v0e1	94170	chr10	16818334	16818634	-25404	1.39	3.89E-02	3.00E-01	Distal Intergenic
Pif1	367645	chr8	71099183	71099483	-25931	1.07	3.90E-02	3.00E-01	Distal Intergenic
Dyrk1a	25255	chr11	34877814	34878114	12282	1.8	3.90E-02	3.00E-01	Intron (NM_012791/25255, intron 1 of 11)
Mettl9	100302453	chr1	190964788	190965088	0	0.97	3.90E-02	3.00E-01	Promoter (<=1kb)
Rnf20	313216	chr5	64893126	64893426	49	0.66	3.90E-02	3.00E-01	Promoter (<=1kb)

Ptpn9	266611	chr8	61646222	61646522	-12923	-1.37	3.90E-02	3.00E-01	Intron (NM_001004270/316108, intron 8 of 8)
Kifap3	289168	chr13	82072715	82073015	218	0.68	3.90E-02	3.00E-01	Promoter (<=1kb)
Bend6	363212	chr9	38158853	38159153	-138100	-1.11	3.90E-02	3.00E-01	Distal Intergenic
Gxylt1	300173	chr7	134495594	134495894	0	1.16	3.91E-02	3.01E-01	Promoter (<=1kb)
Zfp91	246282	chr1	229638987	229639287	0	1.07	3.91E-02	3.01E-01	Promoter (<=1kb)
Unc45b	303373	chr10	70265373	70265673	3012	-1.26	3.91E-02	3.01E-01	5' UTR
Ak2	24184	chr5	147212518	147212818	27044	-1.17	3.91E-02	3.01E-01	Distal Intergenic
Txndc11	302899	chr10	4578709	4579009	240	0.85	3.91E-02	3.01E-01	Promoter (<=1kb)
Habp4	361196	chr17	1718865	1719165	39248	1.78	3.91E-02	3.01E-01	Intron (NM_001108404/361195, intron 13 of 15)
Vmac	363327	chr9	10370425	10370725	31350	2.04	3.91E-02	3.01E-01	Distal Intergenic
Atp5pd	641434	chr10	103965018	103965318	7195	0.77	3.91E-02	3.01E-01	Downstream (2-3kb)
Galntl5	499968	chr4	6449566	6449866	83	-0.99	3.91E-02	3.01E-01	Promoter (<=1kb)
Hyal2	64468	chr8	116325392	116325692	106	0.62	3.91E-02	3.01E-01	Promoter (<=1kb)
Rbm4b	474154	chr1	220026974	220027274	17577	-1.48	3.91E-02	3.01E-01	Distal Intergenic
Zfp655	360764	chr12	11144344	11144644	110	0.64	3.91E-02	3.01E-01	Promoter (<=1kb)
Smim3	286910	chr18	55768103	55768403	3327	-1.28	3.91E-02	3.01E-01	Intron (NM_173126/286910, intron 1 of 1)
Ermp1	373544	chr1	247821428	247821728	0	1.04	3.91E-02	3.01E-01	Promoter (<=1kb)
RGD1310352	303122	chr10	37815011	37815311	57865	-1.5	3.91E-02	3.01E-01	Distal Intergenic
Shld2	364514	chr16	10631421	10631721	29807	-1.5	3.91E-02	3.01E-01	Intron (NM_001025028/364514, intron 2 of 10)
Mapre1	114764	chr3	149221410	149221710	33	0.78	3.92E-02	3.01E-01	Promoter (<=1kb)
Prpf6	366276	chr3	177097897	177098197	0	0.53	3.92E-02	3.01E-01	Promoter (<=1kb)
Bst2	378947	chr16	19942137	19942437	0	-0.67	3.92E-02	3.01E-01	Promoter (<=1kb)
Grsf1	305256	chr14	21040414	21040714	424	0.88	3.92E-02	3.01E-01	Promoter (<=1kb)
Map3k5	365057	chr1	15415872	15416172	3269	-1.31	3.92E-02	3.01E-01	Intron (NM_001277694/365057, intron 1 of 34)
Bmp6	25644	chr17	27134848	27135148	-22028	-1.54	3.92E-02	3.01E-01	Distal Intergenic
Chsy1	292999	chr1	127010683	127010983	69	1.05	3.92E-02	3.01E-01	Promoter (<=1kb)
Zfp949	100233177	chr8	96334227	96334527	-21561	1.7	3.92E-02	3.01E-01	Distal Intergenic
Ldhd	307858	chr19	43772809	43773109	75828	-1.16	3.92E-02	3.01E-01	Distal Intergenic
Plcb2	85240	chr3	110540906	110541206	-23645	1	3.92E-02	3.01E-01	Distal Intergenic
Gypc	364837	chr18	25259213	25259513	85101	-1.37	3.92E-02	3.01E-01	Distal Intergenic
Srsf7	362687	chr6	2885968	2886268	197	0.53	3.92E-02	3.01E-01	Promoter (<=1kb)
Ptp4a1	29463	chr9	37231370	37231670	-79	0.79	3.93E-02	3.01E-01	Promoter (<=1kb)
Ccdc8	494320	chr1	78943891	78944191	10519	1.37	3.93E-02	3.01E-01	3' UTR
Kcmf1	684322	chr4	100822271	100822571	-39254	0.67	3.93E-02	3.01E-01	Distal Intergenic
Dok1	312477	chr4	113866082	113866382	292	0.51	3.93E-02	3.01E-01	Promoter (<=1kb)
Cul4b	302502	chrX	124869947	124870247	82	-1.48	3.93E-02	3.01E-01	Promoter (<=1kb)
Dhx30	367172	chr8	118146927	118147227	45182	-1.16	3.93E-02	3.01E-01	Intron (NM_001024278/367171, intron 13 of 16)
Slc4a11	311423	chr3	123233393	123233693	2842	-1.41	3.93E-02	3.01E-01	Promoter (2-3kb)
Cald1	25687	chr4	62251249	62251549	30238	1.36	3.93E-02	3.01E-01	Intron (NM_013146/25687, intron 1 of 11)
Samd4a	305826	chr15	23708809	23709109	-68729	-1.29	3.93E-02	3.01E-01	Distal Intergenic
Rnf103	84508	chr4	99398979	99399279	0	1.02	3.93E-02	3.01E-01	Promoter (<=1kb)
Septin11	305227	chr14	16602587	16602887	-149697	-1.23	3.93E-02	3.01E-01	Distal Intergenic
Helq	360912	chr14	10447172	10447472	191	0.66	3.93E-02	3.01E-01	Promoter (<=1kb)
Syngr1	29205	chr7	121311038	121311338	23	0.91	3.93E-02	3.01E-01	Promoter (<=1kb)
Fgl1	246186	chr16	54220162	54220462	67076	-1.5	3.93E-02	3.01E-01	Distal Intergenic
Rsbn1	310749	chr2	206407753	206408053	15553	-1.5	3.93E-02	3.01E-01	Intron (NM_001191710/310749, intron 2 of 6)
Arfgef1	312915	chr5	8678912	8679212	12712	1.71	3.93E-02	3.01E-01	Intron (NM_001277056/312915, intron 1 of 38)
Ets1	24356	chr8	33825506	33825806	9120	-1.27	3.94E-02	3.01E-01	Intron (NM_012555/24356, intron 1 of 7)
Gprc5d	500349	chr4	168879832	168880132	4754	1.33	3.94E-02	3.01E-01	Intron (NM_001109254/500349, intron 2 of 3)
Rfx1	288906	chr19	25181557	25181857	0	0.72	3.94E-02	3.01E-01	Promoter (<=1kb)
Rab20	689377	chr16	83362184	83362484	4068	-1.4	3.94E-02	3.01E-01	Intron (NM_001109535/689377, intron 1 of 1)
RGD1559896	498967	chr19	57421523	57421823	270	0.98	3.94E-02	3.01E-01	Promoter (<=1kb)
Ppp3cc	171378	chr15	51985598	51985898	-17701	-1.34	3.94E-02	3.01E-01	3' UTR
Enah	360891	chr13	100414989	100415289	-9650	0.71	3.94E-02	3.01E-01	Distal Intergenic
Ppp1r12a	116670	chr7	51498326	51498626	16595	-1.49	3.94E-02	3.01E-01	Intron (NM_053890/116670, intron 1 of 23)
Igsf9	304982	chr13	90831888	90832188	0	0.65	3.94E-02	3.01E-01	Promoter (<=1kb)
Foxj3	313554	chr5	138596966	138597266	8998	-1.47	3.94E-02	3.01E-01	Intron (NM_001107971/313554, intron 1 of 12)
Enpp6	306460	chr16	48509794	48510094	-72552	1.53	3.94E-02	3.01E-01	Distal Intergenic
Arl13b	304037	chr7	1187739	1188039	170	0.58	3.94E-02	3.01E-01	Promoter (<=1kb)
Chst14	691394	chr3	110710737	110711037	-23068	0.85	3.94E-02	3.01E-01	Distal Intergenic
Wdr5b	303907	chr11	67644061	67644361	3457	1.73	3.94E-02	3.01E-01	Intron (NM_001029903/360721, intron 4 of 5)
Ctsm	306720	chr17	3662180	3662480	-158126	0.51	3.94E-02	3.01E-01	Distal Intergenic
Rnf216	304294	chr12	13508418	13508718	0	0.67	3.95E-02	3.01E-01	Promoter (<=1kb)
Stk24	361092	chr15	106670537	106670837	51202	-1.37	3.95E-02	3.01E-01	Intron (NM_001127494/361092, intron 2 of 10)
Nol12	362955	chr7	120161441	120161741	203	0.55	3.95E-02	3.01E-01	Promoter (<=1kb)
Prickle1	315259	chr7	134721495	134721795	420	0.63	3.95E-02	3.01E-01	Promoter (<=1kb)
Dnajb4	295549	chr2	257404745	257405045	20197	0.68	3.95E-02	3.01E-01	Intron (NM_001013076/295549, intron 4 of 5)
Lamtor2	295234	chr2	187912828	187913128	1777	-1.13	3.95E-02	3.01E-01	Promoter (1-2kb)
Klc1	171041	chr6	136330456	136330756	8	0.96	3.95E-02	3.01E-01	Promoter (<=1kb)
Ripk4	304053	chr11	38346140	38346440	-71906	-1.62	3.95E-02	3.01E-01	Distal Intergenic
Gfod1	306842	chr17	23888530	23888830	34859	-1.27	3.95E-02	3.01E-01	Intron (NM_001170334/306842, intron 1 of 1)
Adam9	290834	chr16	71842184	71842484	31807	1.41	3.95E-02	3.01E-01	Intron (NM_001014772/290834, intron 11 of 22)
Veph1	361954	chr2	158261666	158261966	-105516	-1.32	3.95E-02	3.01E-01	Distal Intergenic
Slco3a1	140915	chr1	135866981	135867281	206202	1.13	3.95E-02	3.01E-01	Intron (NM_177481/140915, intron 3 of 9)
Tbca	366995	chr2	24475488	24475788	25951	1.61	3.95E-02	3.01E-01	Intron (NM_001013245/366995, intron 1 of 3)
Czib	298384	chr5	127487778	127488078	-1340	1.76	3.95E-02	3.01E-01	Promoter (1-2kb)
Mir193	100314244	chr10	67056607	67056907	-8989	-1.08	3.96E-02	3.01E-01	Distal Intergenic
RGD1560854	499106	chr1	84003541	84003841	-4422	-1.25	3.96E-02	3.01E-01	Intron (NM_001008303/292729, intron 4 of 6)
Il17rd	498576	chr16	2684667	2684967	14049	1.79	3.96E-02	3.01E-01	Intron (NM_001191937/498576, intron 1 of 12)
Etnk1	312828	chr4	177410990	177411290	8086	-1.33	3.96E-02	3.01E-01	Intron (NM_001107894/312828, intron 1 of 8)
Borcs5	362452	chr4	168396649	168396949	0	0.64	3.96E-02	3.01E-01	Promoter (<=1kb)
Gem	297902	chr5	25309208	25309508	-29971	-1.27	3.96E-02	3.02E-01	Distal Intergenic
Asap1	314961	chr7	104964187	104964487	-13097	0.69	3.96E-02	3.02E-01	Distal Intergenic
Sos2	85384	chr6	91866426	91866726	141191	1.31	3.97E-02	3.02E-01	Distal Intergenic
Tob1	170842	chr10	81912157	81912457	-1232	0.68	3.97E-02	3.02E-01	Promoter (1-2kb)
Arglu1	290912	chr16	86601157	86601457	280	0.79	3.97E-02	3.02E-01	Promoter (<=1kb)
Mbnl1	282635	chr2	150815549	150815849	59291	0.78	3.97E-02	3.02E-01	Intron (NM_001191566/282635, intron 1 of 7)
Txk	305311	chr14	37976525	37976825	-53487	0.65	3.97E-02	3.02E-01	Intron (NM_053432/84492, intron 3 of 18)
Map3k8	116596	chr17	56123956	56124256	14553	-1.29	3.97E-02	3.02E-01	Intron (NM_053847/116596, intron 5 of 7)
Rest	83618	chr14	33151687	33151987	-1178	0.89	3.97E-02	3.02E-01	Promoter (1-2kb)
Fgf5	60662	chr14	13222080	13222380	-226496	-1.38	3.97E-02	3.02E-01	Distal Intergenic
Spdl1	303037	chr10	19551058	19551358	101515	1.41	3.97E-02	3.02E-01	Distal Intergenic
Olr1271	286959	chr2	35059765	35060065	-115199	1.23	3.97E-02	3.02E-01	Distal Intergenic
Usp10	307905	chr19	52566650	52566950	43	1.2	3.97E-02	3.02E-01	Promoter (<=1kb)

Etv3	295297	chr2	186872540	186872840	-1395	0.87	3.97E-02	3.02E-01	Promoter (1-2kb)
Trim36	291597	chr18	40123175	40123475	11029	0.71	3.98E-02	3.02E-01	Intron (NM_001106147/291597, intron 1 of 9)
Chd6	311607	chr3	157098832	157099132	174	1.48	3.98E-02	3.02E-01	Promoter (<=1kb)
Pde8b	309962	chr2	24790102	24790402	33967	0.85	3.98E-02	3.02E-01	Intron (NM_199268/309962, intron 5 of 20)
Xirp2	311098	chr3	53569158	53569458	5913	-1.29	3.98E-02	3.02E-01	Intron (NM_201989/311098, intron 1 of 8)
Hist1h4b	64627	chr17	44420396	44420696	-101448	1.44	3.98E-02	3.02E-01	Distal Intergenic
Galk2	296117	chr3	118221465	118221765	76869	-1.11	3.98E-02	3.02E-01	Exon (NM_001013919/296117, exon 7 of 10)
Mir146a	100314241	chr10	28937549	28937849	24721	-1.46	3.98E-02	3.02E-01	Distal Intergenic
Tax1bp1	246244	chr4	82773862	82774162	-2584	-0.92	3.98E-02	3.02E-01	Promoter (2-3kb)
Fmo4	246247	chr13	80703235	80703535	40	0.96	3.98E-02	3.02E-01	Promoter (<=1kb)
Gsdme	353316	chr4	80110795	80111095	-111117	-1.15	3.98E-02	3.02E-01	Distal Intergenic
Tbx19	304935	chr13	83429842	83430142	-4201	0.65	3.98E-02	3.02E-01	Distal Intergenic
Ldhd	307858	chr19	43760770	43761070	87867	-0.7	3.99E-02	3.02E-01	Distal Intergenic
Prss16	364719	chr17	44549760	44550060	-5979	1.38	3.99E-02	3.02E-01	Distal Intergenic
Zfp280d	315798	chr8	78858496	78858796	-13306	1.01	3.99E-02	3.02E-01	Distal Intergenic
Foxn3	314374	chr6	123577459	123577759	0	0.97	3.99E-02	3.02E-01	Promoter (<=1kb)
Psma8	364814	chr18	6206987	6207287	92230	0.86	3.99E-02	3.02E-01	Distal Intergenic
Haao	56823	chr6	7073914	7074214	-15600	0.66	3.99E-02	3.02E-01	Distal Intergenic
Foxo3	294515	chr20	46367568	46367868	-60210	1.27	3.99E-02	3.02E-01	Distal Intergenic
Fa2h	307855	chr19	43672771	43673071	-75983	-1.23	3.99E-02	3.02E-01	Distal Intergenic
Cbx7	362962	chr7	121153037	121153337	46	1.16	3.99E-02	3.02E-01	Promoter (<=1kb)
Gli3	140588	chr17	52496305	52496605	72431	1.12	3.99E-02	3.02E-01	Intron (NM_080405/140588, intron 2 of 15)
Swap70	293410	chr1	174980182	174980482	117457	1.61	3.99E-02	3.02E-01	Intron (NM_001134970/691042, intron 29 of 39)
Dpp9	301130	chr9	10846242	10846542	0	1.4	3.99E-02	3.02E-01	Promoter (<=1kb)
Pkig	266709	chr3	160061897	160062197	14601	-1.02	3.99E-02	3.02E-01	Intron (NM_153469/266709, intron 1 of 2)
Acadl	25287	chr9	73796892	73797192	74662	-1.44	3.99E-02	3.02E-01	Distal Intergenic
Adgrl4	64124	chr2	256493395	256493695	-116092	1.83	3.99E-02	3.02E-01	Distal Intergenic
Rad51c	497976	chr10	74778580	74778880	-54633	1.5	4.00E-02	3.02E-01	Distal Intergenic
Myo9b	25486	chr16	19697069	19697369	27658	1.61	4.00E-02	3.03E-01	Intron (NM_001271066/25486, intron 2 of 41)
Nfkbiz	304005	chr11	47260539	47260839	17197	0.7	4.00E-02	3.03E-01	5' UTR
Arl4d	303559	chr10	89581844	89582144	3632	1.65	4.00E-02	3.03E-01	Distal Intergenic
Cmahp	361245	chr17	42605699	42605999	34222	-1.74	4.00E-02	3.03E-01	Intron (NM_001024273/361245, intron 9 of 14)
Smoc1	314280	chr6	104854232	104854532	135555	1.07	4.00E-02	3.03E-01	Intron (NM_001002835/314280, intron 7 of 12)
Kif13a	308173	chr17	18243838	18244138	91856	0.66	4.00E-02	3.03E-01	Intron (NM_001107462/308173, intron 2 of 36)
Phtf2	296762	chr4	10823432	10823732	174	1.42	4.00E-02	3.03E-01	Promoter (<=1kb)
Strbp	84476	chr3	21957110	21957410	-52840	1.08	4.00E-02	3.03E-01	Distal Intergenic
Ntan1	360462	chr10	3203525	3203825	-14650	-1.28	4.00E-02	3.03E-01	Intron (NM_001135846/304714, intron 12 of 17)
Jun	24516	chr5	114058775	114059075	-44498	-1.27	4.00E-02	3.03E-01	Distal Intergenic
Lrp6	312781	chr4	168308798	168309098	-11425	-1.28	4.00E-02	3.03E-01	Distal Intergenic
Wdr53	498097	chr11	71803503	71803803	-7707	-1.31	4.00E-02	3.03E-01	Distal Intergenic
Cyb5d1	363629	chr10	55969071	55969371	27928	0.94	4.00E-02	3.03E-01	Distal Intergenic
Sirt5	306840	chr17	23986964	23987264	168	0.56	4.01E-02	3.03E-01	Promoter (<=1kb)
Bola3	297388	chr4	115125098	115125398	78405	-1.27	4.01E-02	3.03E-01	Distal Intergenic
Cb707485	100310783	chr20	3520263	3520563	14613	-1.17	4.01E-02	3.03E-01	Distal Intergenic
Cpsf6	299811	chr7	60416514	60416814	111	1.22	4.01E-02	3.03E-01	Promoter (<=1kb)
Prkar2a	29699	chr8	117486185	117486485	100	0.82	4.01E-02	3.03E-01	Promoter (<=1kb)
Flrt2	299236	chr6	119573301	119573601	53587	-1.33	4.02E-02	3.03E-01	Intron (NM_001106750/299236, intron 1 of 1)
Fyttd1	360726	chr11	71093404	71093704	42969	-1.4	4.02E-02	3.03E-01	Distal Intergenic
Pgf	94203	chr6	109021173	109021473	-16575	-1.38	4.02E-02	3.03E-01	Distal Intergenic
Cldn18	315953	chr8	107920110	107920410	32120	-1.12	4.02E-02	3.03E-01	Distal Intergenic
Prkca	24680	chr10	96584511	96584811	136	0.75	4.02E-02	3.03E-01	Promoter (<=1kb)
Sgms2	310849	chr2	236517998	236518298	-37496	-1.41	4.02E-02	3.03E-01	Distal Intergenic
Med26	306328	chr16	19001177	19001477	116	0.7	4.02E-02	3.03E-01	Promoter (<=1kb)
Clul1	367345	chr9	121980388	121980688	-8333	0.64	4.02E-02	3.03E-01	Distal Intergenic
Ier5l	499772	chr3	9037033	9037333	609	1.26	4.02E-02	3.03E-01	Promoter (<=1kb)
Crip1	691657	chr6	138023109	138023409	57871	1.2	4.02E-02	3.03E-01	Distal Intergenic
Mir350	100313985	chr13	43359259	43359559	-467933	0.58	4.02E-02	3.03E-01	Distal Intergenic
Sik3	684112	chr8	50335432	50335732	25169	-1.27	4.02E-02	3.03E-01	Intron (NM_001271216/684112, intron 1 of 24)
Atp8b1	291555	chr18	60207809	60208109	-112475	1.55	4.02E-02	3.03E-01	Distal Intergenic
Tcta	306587	chr8	117090892	117091192	-8730	-1.58	4.02E-02	3.03E-01	Distal Intergenic
Samd4a	305826	chr15	23862577	23862877	84739	1.46	4.03E-02	3.03E-01	Intron (NM_001107254/305826, intron 8 of 13)
Ntrk2	25054	chr17	5900994	5901294	343318	1.28	4.03E-02	3.03E-01	Distal Intergenic
Atg7	312647	chr4	146625884	146626184	27468	1.03	4.03E-02	3.03E-01	Exon (NM_001012097/312647, exon 10 of 18)
Cd276	315716	chr8	63343424	63343724	6588	-1.16	4.03E-02	3.03E-01	Intron (NM_182824/315716, intron 1 of 7)
Gpr137b	289287	chr17	90714289	90714589	23179	1.69	4.03E-02	3.03E-01	Exon (NM_001191893/364755, exon 15 of 16)
Zbtb7c	679155	chr18	72005147	72005447	-119510	1.01	4.03E-02	3.03E-01	Distal Intergenic
Acvr2a	29263	chr3	32947300	32947600	-373	0.9	4.03E-02	3.03E-01	Promoter (<=1kb)
Rab3gap2	289350	chr13	103157921	103158221	87	0.55	4.03E-02	3.03E-01	Promoter (<=1kb)
Mnt	287521	chr10	61692194	61692494	6953	0.63	4.03E-02	3.04E-01	Exon (NM_001105807/287521, exon 4 of 8)
Pkn1	29355	chr19	24831006	24831306	194	0.76	4.03E-02	3.04E-01	Promoter (<=1kb)
Nup155	117021	chr2	57298352	57298652	91687	-1.62	4.03E-02	3.04E-01	Distal Intergenic
Cwc27	361887	chr2	34666960	34667260	255461	1.68	4.04E-02	3.04E-01	Distal Intergenic
P3h2	288016	chr11	78028938	78029238	53	0.94	4.04E-02	3.04E-01	Promoter (<=1kb)
Gmps	295088	chr2	154552836	154553136	-2831	1.76	4.04E-02	3.04E-01	Promoter (2-3kb)
Osbpl9	298369	chr5	128834882	128835182	4253	1.76	4.04E-02	3.04E-01	Intron (NM_001044234/298369, intron 1 of 23)
Col5a2	85250	chr9	52183414	52183714	55021	1.76	4.04E-02	3.04E-01	Intron (NM_053488/85250, intron 1 of 55)
Acvr1	79558	chr3	44474640	44474940	26046	1.67	4.04E-02	3.04E-01	Intron (NM_024486/79558, intron 2 of 9)
Rps6kc1	289342	chr13	109431413	109431713	52155	-1.04	4.04E-02	3.04E-01	Intron (NM_001105984/289342, intron 6 of 13)
Dock6	367039	chr8	22878194	22878494	-3557	-1.19	4.04E-02	3.04E-01	Distal Intergenic
Atl2	298757	chr6	2569174	2569474	159	0.95	4.04E-02	3.04E-01	Promoter (<=1kb)
Foxp1	297480	chr4	131393105	131393405	204899	0.88	4.04E-02	3.04E-01	Intron (NM_001034131/297480, intron 8 of 15)
Slc6a3	24898	chr1	32375092	32375392	-11109	-1.27	4.04E-02	3.04E-01	Distal Intergenic
Pten	50557	chr1	251469558	251469858	47744	-1.32	4.04E-02	3.04E-01	Intron (NM_031606/50557, intron 5 of 8)
Cldn11	84588	chr2	115890561	115890861	-53715	0.98	4.04E-02	3.04E-01	Distal Intergenic
Srrm1	313620	chr5	153707720	153708020	94	0.55	4.05E-02	3.04E-01	Promoter (<=1kb)
Ankrd27	361555	chr1	91857237	91857537	167	0.76	4.05E-02	3.04E-01	Promoter (<=1kb)
Ndel1	170845	chr10	55437056	55437356	7364	-1.24	4.05E-02	3.04E-01	Intron (NM_133320/170845, intron 1 of 8)
Ing1	306626	chr16	83282890	83283190	-4770	1.22	4.05E-02	3.04E-01	Distal Intergenic
Col11a1	25654	chr2	216883276	216883576	19848	1.72	4.05E-02	3.04E-01	Intron (NM_013117/25654, intron 1 of 66)
Dysf	312492	chr4	115698260	115698560	-14444	1.72	4.05E-02	3.04E-01	Distal Intergenic
Atp23	299828	chr7	70241213	70241513	-42558	-1.43	4.05E-02	3.04E-01	Distal Intergenic
Slc30a1	58976	chr13	110652663	110652963	-24847	1.59	4.06E-02	3.04E-01	Distal Intergenic
Dysf	312492	chr4	115937550	115937850	224546	-1.08	4.06E-02	3.04E-01	Distal Intergenic
Cox17	89786	chr11	64971248	64971548	-2811	1.11	4.06E-02	3.04E-01	Promoter (2-3kb)

Tent5c	310721	chr2	202816510	202816810	0	0.7	4.06E-02	3.04E-01	Promoter (<=1kb)
Cxcr3	84475	chrX	71821965	71822265	-204968	1.52	4.06E-02	3.04E-01	Distal Intergenic
Hat1	296501	chr3	57999946	58000246	-22149	1.77	4.06E-02	3.04E-01	Distal Intergenic
Tcof1	291571	chr18	56122228	56122528	-6509	1.92	4.06E-02	3.04E-01	Distal Intergenic
Mrpl45	287656	chr10	85302051	85302351	44175	0.9	4.06E-02	3.04E-01	Distal Intergenic
Kcnip2	56817	chr1	262685596	262685896	-206711	-1.18	4.06E-02	3.04E-01	Intron (NM_001135762/368128, intron 3 of 15)
Isg15	298693	chr5	173622355	173622655	3534	0.68	4.06E-02	3.04E-01	Downstream (2-3kb)
Irak2	362418	chr4	145596637	145596937	2062	-1.39	4.06E-02	3.05E-01	Promoter (2-3kb)
N4bp2l2	288416	chr12	660190	660490	-9814	1.21	4.07E-02	3.05E-01	Distal Intergenic
Nectin1	192183	chr8	47959887	47960187	-134046	-1.21	4.07E-02	3.05E-01	Distal Intergenic
Rab12	25530	chr9	114585018	114585318	124295	-1.18	4.07E-02	3.05E-01	Distal Intergenic
Ppp3cc	171378	chr15	51990480	51990780	-22583	-1.54	4.07E-02	3.05E-01	Intron (NM_001107275/306009, intron 5 of 9)
Cnot11	363221	chr9	46437900	46438200	167671	0.88	4.07E-02	3.05E-01	Distal Intergenic
Rcor2	305811	chr1	222550793	222551093	31532	-1.24	4.07E-02	3.05E-01	Intron (NM_021699/60328, intron 1 of 16)
Acer2	313339	chr5	105263563	105263863	32983	1.75	4.08E-02	3.05E-01	Intron (NM_001107943/313339, intron 4 of 5)
Ush2a	289369	chr13	106903236	106903536	151611	-1.45	4.08E-02	3.05E-01	Intron (NM_001302219/289369, intron 13 of 70)
Emc7	296050	chr3	103980593	103980893	35239	1.55	4.08E-02	3.05E-01	Intron (NM_017362/53949, intron 1 of 1)
Rimbp2	266780	chr12	31368571	31368871	-24358	-1.35	4.08E-02	3.05E-01	Distal Intergenic
Lamc2	192362	chr13	70572494	70572794	53458	-1.16	4.08E-02	3.05E-01	Intron (NM_001100640/192362, intron 21 of 23)
Rpusd4	315550	chr8	36463805	36464105	-3601	-1.41	4.08E-02	3.05E-01	Distal Intergenic
Pax6	25509	chr3	95705080	95705380	-2006	1.68	4.08E-02	3.05E-01	Promoter (2-3kb)
Trabd	300142	chr7	130051724	130052024	-6228	0.62	4.08E-02	3.05E-01	3' UTR
Fbxo22	300724	chr8	59770119	59770419	87	0.69	4.08E-02	3.05E-01	Promoter (<=1kb)
Fbxo42	362646	chr5	159670772	159671072	110	0.76	4.08E-02	3.05E-01	Promoter (<=1kb)
Dmrt2	309430	chr1	244025451	244025751	362628	0.94	4.08E-02	3.05E-01	Distal Intergenic
Smco4	363020	chr8	14104895	14105195	44501	1.73	4.08E-02	3.05E-01	Intron (NM_001014087/315438, intron 12 of 13)
Mink1	303259	chr10	57199572	57199872	14225	-1.28	4.08E-02	3.05E-01	Intron (NM_001271136/303259, intron 1 of 32)
U2af1	687575	chr20	10407127	10407427	113	0.98	4.08E-02	3.05E-01	Promoter (<=1kb)
Cox6a2	25278	chr1	199635318	199635618	-10535	1.2	4.09E-02	3.05E-01	Distal Intergenic
Stard3nl	291182	chr17	47817887	47818187	155334	-0.7	4.09E-02	3.05E-01	Distal Intergenic
Wbp11	297695	chr4	170209317	170209617	-41609	-1.42	4.09E-02	3.05E-01	Distal Intergenic
Pdlim5	64353	chr2	247875665	247875965	112497	-1.27	4.09E-02	3.05E-01	Intron (NM_053326/64353, intron 7 of 12)
Taf8	316216	chr9	15492900	15493200	-19851	0.78	4.09E-02	3.05E-01	Distal Intergenic
Map3k10	308463	chr1	84504962	84505262	-13483	-1.23	4.09E-02	3.05E-01	Distal Intergenic
Rab12	25530	chr9	114612664	114612964	96649	-1.62	4.09E-02	3.05E-01	Distal Intergenic
Plcd4	140693	chr9	81830126	81830426	13254	-1.43	4.09E-02	3.05E-01	Intron (NM_080688/140693, intron 6 of 16)
Prrx2	113931	chr3	9682241	9682541	370	0.88	4.09E-02	3.05E-01	Promoter (<=1kb)
Mir207	100314253	chr5	57028549	57028849	-10	0.72	4.09E-02	3.05E-01	Promoter (<=1kb)
Vldlr	25696	chr1	245237420	245237720	-16	0.93	4.09E-02	3.05E-01	Promoter (<=1kb)
Fkbp3	299104	chr6	86823534	86823834	-1419	0.71	4.10E-02	3.05E-01	Promoter (1-2kb)
Rps6kc1	289342	chr13	109211533	109211833	272035	1.05	4.10E-02	3.05E-01	Distal Intergenic
Aox3	493909	chr9	65002384	65002684	-11178	1.74	4.10E-02	3.05E-01	Intron (NM_019363/54349, intron 31 of 34)
Ptprq	360417	chr7	50125657	50125957	-90725	-1.21	4.10E-02	3.05E-01	Intron (NM_001033680/25716, intron 9 of 10)
Il1rap	25466	chr11	77576162	77576462	16664	-1.13	4.10E-02	3.05E-01	Intron (NM_001167840/25466, intron 1 of 10)
Adgrl4	64124	chr2	256456876	256457176	-152611	-1.26	4.10E-02	3.05E-01	Distal Intergenic
Cflar	117279	chr9	65551144	65551444	16516	-1.34	4.10E-02	3.05E-01	Intron (NM_057138/117279, intron 2 of 5)
Smc3	29486	chr1	274310277	274310577	157	1.09	4.10E-02	3.05E-01	Promoter (<=1kb)
Smad3	25631	chr8	68642104	68642404	35945	-1.08	4.10E-02	3.05E-01	Intron (NM_013095/25631, intron 1 of 8)
Il6st	25205	chr2	44298055	44298355	7980	-1.06	4.10E-02	3.06E-01	Exon (NM_001008725/25205, exon 5 of 15)
Limd2	360646	chr10	94329011	94329311	23569	-1.22	4.10E-02	3.06E-01	Intron (NM_001107058/303604, intron 6 of 15)
Kif2a	84391	chr2	38313153	38313453	-104388	-1.35	4.10E-02	3.06E-01	Distal Intergenic
Rnf222	363627	chr10	55462454	55462754	-15544	-1.29	4.10E-02	3.06E-01	Distal Intergenic
Tigd5	300034	chr7	116943056	116943356	0	1.02	4.11E-02	3.06E-01	Promoter (<=1kb)
Map4k4	301363	chr9	46690797	46691097	32875	-1.25	4.11E-02	3.06E-01	Intron (NM_001106904/301363, intron 1 of 28)
Tent5b	313019	chr5	151659970	151660270	-31798	1.12	4.11E-02	3.06E-01	Distal Intergenic
Uggt1	171129	chr9	42809516	42809816	-4056	-1.32	4.11E-02	3.06E-01	Distal Intergenic
Cdkn2aip	306455	chr16	47657996	47658296	-7520	-0.92	4.11E-02	3.06E-01	Distal Intergenic
Rps6ka1	81771	chr5	152145282	152145582	-22598	0.7	4.11E-02	3.06E-01	Distal Intergenic
Asap1	314961	chr7	104912897	104913197	37893	1.11	4.11E-02	3.06E-01	Intron (NM_001044245/314961, intron 1 of 27)
Crip1	691657	chr6	137968089	137968389	2851	0.63	4.11E-02	3.06E-01	Promoter (2-3kb)
Zfp672	303165	chr10	43760626	43760926	4	0.67	4.11E-02	3.06E-01	Promoter (<=1kb)
Cyb5r1	304805	chr13	51230123	51230423	81	0.68	4.11E-02	3.06E-01	Promoter (<=1kb)
Cinp	299334	chr6	135304526	135304826	-104	0.69	4.11E-02	3.06E-01	Promoter (<=1kb)
Mef2a	309957	chr1	128305092	128305392	35871	-1.31	4.11E-02	3.06E-01	Intron (NM_001014035/309957, intron 2 of 10)
Adprhl1	290880	chr16	81628485	81628785	11881	-1.35	4.11E-02	3.06E-01	Intron (NM_001013054/290880, intron 6 of 6)
Ulk1	360827	chr12	51908310	51908610	0	1.07	4.11E-02	3.06E-01	Promoter (<=1kb)
Zfp706	500855	chr7	75809465	75809765	88	0.95	4.11E-02	3.06E-01	Promoter (<=1kb)
Itch	311567	chr3	150664092	150664392	-22367	0.94	4.12E-02	3.06E-01	Distal Intergenic
Ifng	25712	chr7	61546760	61547060	209377	1.05	4.12E-02	3.06E-01	Distal Intergenic
Cxcr5	29363	chr8	48825059	48825359	12863	1.72	4.12E-02	3.06E-01	Intron (NM_001106817/300673, intron 5 of 10)
Idnk	498695	chr17	6933940	6934240	-41482	0.59	4.12E-02	3.06E-01	Distal Intergenic
Tnn	304913	chr13	77892077	77892377	4364	1.46	4.12E-02	3.06E-01	Intron (NM_001107189/304913, intron 1 of 21)
Cfh	155012	chr13	57079710	57080010	530	-1.04	4.12E-02	3.06E-01	Promoter (<=1kb)
Epn2	60443	chr10	47876672	47876972	-19322	-1.28	4.12E-02	3.06E-01	Distal Intergenic
Pcnp	288165	chr11	47055909	47056209	-4276	0.65	4.12E-02	3.06E-01	Distal Intergenic
Cysltr2	170926	chr15	54902555	54902855	3348	1.47	4.12E-02	3.06E-01	Downstream (1-2kb)
F2rl1	116677	chr2	25381267	25381567	-145992	-1.28	4.12E-02	3.06E-01	Distal Intergenic
Fubp3	362106	chr3	10269421	10269721	0	1.02	4.12E-02	3.06E-01	Promoter (<=1kb)
Gatad2a	290669	chr16	21221135	21221435	43342	1.06	4.12E-02	3.06E-01	Intron (NM_001013881/290669, intron 1 of 12)
Zfp292	50552	chr5	50354477	50354777	7567	1.7	4.12E-02	3.06E-01	Intron (NM_001008879/50552, intron 1 of 7)
Far1	293173	chr1	178351703	178352003	-12151	1.21	4.12E-02	3.06E-01	Distal Intergenic
Nkx6-3	685102	chr16	73673667	73673967	-3185	1.38	4.12E-02	3.06E-01	Distal Intergenic
Klf7	363243	chr9	70927723	70928023	-139810	-0.98	4.12E-02	3.06E-01	Distal Intergenic
Chchd3	296966	chr4	60358192	60358492	70	0.53	4.12E-02	3.06E-01	Promoter (<=1kb)
Osbpl11	303888	chr11	70833517	70833817	0	0.7	4.12E-02	3.06E-01	Promoter (<=1kb)
Ptbp1	29497	chr7	12673401	12673701	22	0.67	4.13E-02	3.06E-01	Promoter (<=1kb)
Pum1	362609	chr5	148791954	148792254	10687	-1.31	4.13E-02	3.06E-01	Intron (NM_001108684/362609, intron 2 of 21)
Ccdc34	362187	chr3	101156331	101156631	119	0.53	4.13E-02	3.06E-01	Promoter (<=1kb)
Jam3	315509	chr8	28208015	28208315	151	0.6	4.13E-02	3.06E-01	Promoter (<=1kb)
Sptbn1	305614	chr14	114602128	114602428	90336	-1.22	4.13E-02	3.06E-01	Intron (NM_001013130/305614, intron 2 of 36)
Gdap2	362004	chr2	202470481	202470781	0	0.69	4.13E-02	3.06E-01	Promoter (<=1kb)
Poll	361767	chr1	265281233	265281533	17264	0.92	4.13E-02	3.06E-01	Distal Intergenic
Asb6	296627	chr3	9669405	9669705	-5087	1.69	4.13E-02	3.06E-01	Distal Intergenic
Rgs9	29481	chr10	97407387	97407687	174501	-1.37	4.13E-02	3.06E-01	Distal Intergenic

Wdtc1	313020	chr5	151536040	151536340	178	0.55	4.13E-02	3.06E-01	Promoter (<=1kb)
Smpd4	303790	chr11	87548997	87549297	26026	0.85	4.13E-02	3.06E-01	Distal Intergenic
Rexo4	311826	chr3	5519431	5519731	-8523	1.59	4.13E-02	3.06E-01	Distal Intergenic
Spp1	25353	chr14	6706478	6706778	-26557	1.71	4.13E-02	3.06E-01	Distal Intergenic
Marcksl1	81520	chr5	147714357	147714657	194	1.29	4.13E-02	3.06E-01	Promoter (<=1kb)
Pdgfrl	290771	chr16	54450044	54450344	82	0.65	4.14E-02	3.06E-01	Promoter (<=1kb)
Ccdc24	500534	chr5	136699752	136700052	6946	1.31	4.14E-02	3.06E-01	Intron (NM_053818/116509, intron 12 of 13)
Mib1	307594	chr18	2001512	2001812	30598	-1	4.14E-02	3.06E-01	Intron (NM_001107405/307594, intron 2 of 21)
Sulf2	311642	chr3	162829332	162829632	43199	-1.34	4.14E-02	3.06E-01	Intron (NM_001034927/311642, intron 3 of 20)
Zfp474	307310	chr18	47557049	47557349	-20217	1.5	4.14E-02	3.06E-01	Intron (NM_017061/24914, intron 2 of 8)
Wdsub1	362137	chr3	45955104	45955404	51	0.67	4.14E-02	3.06E-01	Promoter (<=1kb)
Lpgat1	679692	chr13	110396958	110397258	-1542	1.73	4.14E-02	3.06E-01	Promoter (1-2kb)
Ube2j2	298689	chr5	173372690	173372990	31	0.72	4.14E-02	3.06E-01	Promoter (<=1kb)
Acat2	308100	chr1	47972382	47972682	0	0.59	4.14E-02	3.06E-01	Promoter (<=1kb)
Rock1	81762	chr18	1390050	1390350	-12	0.58	4.14E-02	3.06E-01	Promoter (<=1kb)
Kdm2a	361700	chr1	219640244	219640544	0	0.82	4.14E-02	3.06E-01	Promoter (<=1kb)
Scaf8	245926	chr1	44025131	44025431	6072	-1.45	4.14E-02	3.06E-01	Intron (NM_139094/245926, intron 1 of 19)
Akap6	64553	chr6	73474491	73474791	-78419	1.66	4.15E-02	3.06E-01	Distal Intergenic
Slc25a17	300083	chr7	122583637	122583937	-1860	1.74	4.15E-02	3.06E-01	Promoter (1-2kb)
Rps23	124323	chr2	19938148	19938448	114914	-1.01	4.15E-02	3.06E-01	Intron (NM_001109505/688555, intron 5 of 7)
Tbc1d2	313234	chr5	62259893	62260193	0	0.65	4.15E-02	3.06E-01	Promoter (<=1kb)
Nfatc3	361400	chr19	38043688	38043988	3959	-1.41	4.15E-02	3.06E-01	Intron (NM_001108447/361400, intron 1 of 10)
Ppp2r5c	691318	chr6	134868412	134868712	23753	-1.17	4.15E-02	3.06E-01	Intron (NM_001191112/691318, intron 1 of 13)
Galnt10	170501	chr10	43140135	43140435	72819	1.43	4.15E-02	3.06E-01	Intron (NM_130742/170501, intron 1 of 11)
Snx24	361328	chr18	48201843	48202143	115	1.12	4.15E-02	3.06E-01	Promoter (<=1kb)
Mapkap1	296648	chr3	13634818	13635118	149838	1.87	4.16E-02	3.07E-01	Intron (NM_001011964/296648, intron 7 of 11)
Tmod3	300838	chr8	82452322	82452622	39621	-1.21	4.16E-02	3.07E-01	Intron (NM_001011997/300838, intron 3 of 9)
Crnkl1	100910202	chr3	140729490	140729790	-587811	1.62	4.16E-02	3.07E-01	Distal Intergenic
Tnnc1	290561	chr16	7284732	7285032	-7175	1.41	4.16E-02	3.07E-01	Distal Intergenic
Neu3	117185	chr1	164830974	164831274	-16323	1.83	4.16E-02	3.07E-01	3' UTR
Ppp1r3c	309513	chr1	255372615	255372915	3918	-1.34	4.16E-02	3.07E-01	3' UTR
Rev1	316344	chr9	44787160	44787460	-26111	0.68	4.16E-02	3.07E-01	Distal Intergenic
Rhobtb3	309922	chr2	2655239	2655539	27766	1.35	4.16E-02	3.07E-01	Intron (NM_001107645/309922, intron 6 of 11)
Nxf1	59087	chr1	224963883	224964183	6348	1.76	4.16E-02	3.07E-01	Exon (NM_021579/59087, exon 6 of 21)
Mb	59108	chr7	118044768	118045068	63796	0.71	4.16E-02	3.07E-01	Distal Intergenic
Tasp1	311468	chr3	133167696	133167996	-44941	-1.19	4.16E-02	3.07E-01	Distal Intergenic
Elf2	361944	chr2	140333669	140333969	943	0.78	4.16E-02	3.07E-01	Promoter (<=1kb)
Sox12	689988	chr3	147864608	147864908	485	0.99	4.16E-02	3.07E-01	Promoter (<=1kb)
Dusp6	116663	chr7	41462562	41462862	-12301	-0.84	4.16E-02	3.07E-01	Distal Intergenic
Tyms	29261	chr9	121908528	121908828	22736	1.67	4.16E-02	3.07E-01	Distal Intergenic
Crim1	298744	chr6	813672	813972	25124	1.19	4.16E-02	3.07E-01	Intron (NM_001169103/298744, intron 1 of 16)
Ctnnd1	311163	chr3	72035526	72035826	17164	-0.98	4.16E-02	3.07E-01	Intron (NM_001107740/311163, intron 1 of 17)
Habp4	361196	chr17	1679732	1680032	115	0.9	4.17E-02	3.07E-01	Promoter (<=1kb)
Maf	54267	chr19	48602403	48602703	-405655	-0.83	4.17E-02	3.07E-01	Distal Intergenic
Pparg	25664	chr4	147308149	147308449	-24599	-1.1	4.17E-02	3.07E-01	Intron (NM_001145367/25664, intron 2 of 7)
Nnt	310378	chr2	52215770	52216070	66478	-1.49	4.17E-02	3.07E-01	Intron (NM_001013157/310378, intron 17 of 21)
Lifr	81680	chr2	56426537	56426837	-9889	0.98	4.17E-02	3.07E-01	Distal Intergenic
Ncoa1	313929	chr6	28931570	28931870	0	0.71	4.17E-02	3.07E-01	Promoter (<=1kb)
Dele1	307480	chr18	31311890	31312190	-84797	0.77	4.17E-02	3.07E-01	Distal Intergenic
Rps10	81773	chr20	7214974	7215274	5067	-1.21	4.18E-02	3.07E-01	Downstream (<1kb)
RGD1311084	311852	chr3	9533241	9533541	102517	-1.16	4.18E-02	3.07E-01	Distal Intergenic
Cdc6	360621	chr10	86836947	86837247	17018	-1.11	4.18E-02	3.07E-01	Distal Intergenic
Zbed4	315211	chr7	129747045	129747345	-2369	0.92	4.18E-02	3.07E-01	Promoter (2-3kb)
Klf14	312203	chr4	58292809	58293109	-42011	-0.75	4.18E-02	3.07E-01	Distal Intergenic
Peli3	309157	chr1	220199560	220199860	4172	1.21	4.18E-02	3.07E-01	Exon (NM_001127542/309157, exon 3 of 7)
Oaf	315594	chr8	47529063	47529363	326	0.75	4.18E-02	3.07E-01	Promoter (<=1kb)
Arpc1b	54227	chr12	11258124	11258424	7425	1.47	4.18E-02	3.07E-01	Exon (NM_019289/54227, exon 3 of 10)
Cish	83681	chr8	116054551	116054851	4	0.52	4.18E-02	3.07E-01	Promoter (<=1kb)
Cyth4	500906	chr7	119810993	119811293	-9247	-1.38	4.18E-02	3.07E-01	Distal Intergenic
Prpsap2	117272	chr10	48043962	48044262	82	1.09	4.18E-02	3.07E-01	Promoter (<=1kb)
Pemt	25511	chr10	46411687	46411987	-7047	0.76	4.18E-02	3.07E-01	Distal Intergenic
Pfas	287420	chr10	55582124	55582424	7554	-1.36	4.18E-02	3.07E-01	Intron (NM_001105791/287420, intron 9 of 27)
Adamtsl5	314626	chr7	12229459	12229759	80	0.61	4.18E-02	3.07E-01	Promoter (<=1kb)
Zfp532	307362	chr18	61173889	61174189	-87229	-0.79	4.19E-02	3.07E-01	Distal Intergenic
Cers5	366984	chr7	141442221	141442521	-17878	1.24	4.19E-02	3.07E-01	Intron (NM_001191615/300228, intron 8 of 9)
Txn2	79462	chr7	119128672	119128972	29201	1.44	4.19E-02	3.07E-01	Distal Intergenic
Plekhm2	313667	chr5	160219607	160219907	0	0.88	4.19E-02	3.07E-01	Promoter (<=1kb)
Dnm2	25751	chr8	22488265	22488565	29325	-1.3	4.19E-02	3.07E-01	Intron (NM_013199/25751, intron 1 of 20)
Arhgap35	306400	chr1	78543378	78543678	29696	-1	4.19E-02	3.07E-01	Intron (NM_001271132/306400, intron 1 of 6)
Apobec4	498251	chr13	70019275	70019575	-137947	0.66	4.19E-02	3.07E-01	Intron (NM_001105957/289080, intron 2 of 19)
Slc2a1	24778	chr5	138154910	138155210	230	1.06	4.19E-02	3.07E-01	Promoter (<=1kb)
Meis2	311311	chr3	107350061	107350361	410228	1.58	4.19E-02	3.07E-01	Distal Intergenic
Slc16a7	29735	chr7	68292272	68292572	257191	-1.31	4.19E-02	3.07E-01	Distal Intergenic
Ptbp2	310820	chr2	223321880	223322180	362	0.86	4.19E-02	3.07E-01	Promoter (<=1kb)
Uchl1	29545	chr14	43284003	43284303	-140061	-1.39	4.19E-02	3.07E-01	Distal Intergenic
Bphl	361239	chr17	31534723	31535023	149	0.55	4.19E-02	3.07E-01	Promoter (<=1kb)
Ddit4	140942	chr20	29478447	29478747	32635	-1.27	4.19E-02	3.07E-01	Distal Intergenic
Kif2a	84391	chr2	38208271	38208571	194	0.78	4.19E-02	3.07E-01	Promoter (<=1kb)
Arhgap22	306279	chr16	9499216	9499516	-63702	-1.46	4.20E-02	3.07E-01	Distal Intergenic
Ube2z	303478	chr10	83888413	83888713	110	1.02	4.20E-02	3.07E-01	Promoter (<=1kb)
Pdgfa	25266	chr12	17731136	17731436	-2705	-1.57	4.20E-02	3.07E-01	Promoter (2-3kb)
Khdrbs1	117268	chr5	147952680	147952980	113	0.63	4.20E-02	3.07E-01	Promoter (<=1kb)
Mreg	501162	chr9	79563712	79564012	-18688	-1.24	4.20E-02	3.07E-01	Distal Intergenic
Tbc1d8	680133	chr9	46155049	46155349	51304	-1.54	4.20E-02	3.07E-01	Intron (NM_001191067/680133, intron 1 of 19)
Adamtsl3	308787	chr1	144333717	144334017	94759	-1.22	4.20E-02	3.07E-01	Intron (NM_001107533/308787, intron 3 of 29)
Txndc12	298370	chr5	128449647	128449947	-883	0.87	4.20E-02	3.07E-01	Promoter (<=1kb)
Sfxn5	261737	chr4	117127839	117128139	-16291	-1.02	4.20E-02	3.07E-01	Distal Intergenic
Crybb3	64349	chr12	49479990	49480290	-85702	0.56	4.20E-02	3.07E-01	Distal Intergenic
Sumo2	690244	chr10	104097088	104097388	595	0.71	4.20E-02	3.07E-01	Promoter (<=1kb)
Got1	24401	chr1	263269743	263270043	0	0.59	4.20E-02	3.07E-01	Promoter (<=1kb)
Fmr1	24948	chrX	154653156	154653456	68858	0.6	4.20E-02	3.07E-01	Distal Intergenic
Gins2	292058	chr19	54176123	54176423	-12515	-1.03	4.20E-02	3.07E-01	Distal Intergenic
Chd6	311607	chr3	157077327	157077627	21679	-1.41	4.20E-02	3.07E-01	Intron (NM_001107797/311607, intron 1 of 36)
Sacm1l	116482	chr8	132616174	132616474	9059	-1.49	4.20E-02	3.07E-01	Intron (NM_001357497/116482, intron 1 of 19)

Wdr60	314523	chr6	144120948	144121248	2348	-1.41	4.20E-02	3.07E-01	Promoter (2-3kb)
Spp2	94168	chr9	95561057	95561357	59545	-0.95	4.20E-02	3.07E-01	Distal Intergenic
RGD1310352	303122	chr10	37755162	37755462	-1684	1.73	4.20E-02	3.07E-01	Promoter (1-2kb)
Sav1	299116	chr6	92391599	92391899	6779	-1.19	4.20E-02	3.07E-01	Intron (NM_001097581/299116, intron 2 of 4)
Fam76b	367021	chr8	12498251	12498551	142484	0.83	4.20E-02	3.07E-01	Distal Intergenic
Rab30	308821	chr1	157592318	157592618	-53555	-1.41	4.20E-02	3.07E-01	Distal Intergenic
Ap1s1	360785	chr12	22662496	22662796	-2362	1.72	4.21E-02	3.07E-01	Promoter (2-3kb)
Ahcyl1	362013	chr2	210474771	210475071	-1010	-1.4	4.21E-02	3.07E-01	Promoter (1-2kb)
Cfap36	289859	chr14	113771207	113771507	-126428	1.05	4.21E-02	3.07E-01	Distal Intergenic
Fdx1	29189	chr8	56394350	56394650	-1151	1.73	4.21E-02	3.07E-01	Promoter (1-2kb)
Ints2	360589	chr10	73690453	73690753	134	0.59	4.21E-02	3.07E-01	Promoter (<=1kb)
Tcta	306587	chr8	117082606	117082906	-444	1.29	4.21E-02	3.07E-01	Promoter (<=1kb)
Actr2	289820	chr14	104317722	104318022	57626	0.57	4.21E-02	3.07E-01	Distal Intergenic
Otud5	363452	chrX	15503190	15503490	208	0.99	4.21E-02	3.07E-01	Promoter (<=1kb)
Nsmce2	299957	chr7	99686522	99686822	9187	-1.43	4.21E-02	3.07E-01	Intron (NM_001024876/299957, intron 2 of 6)
Specc1l	361828	chr20	14374277	14374577	19295	1.93	4.21E-02	3.07E-01	Intron (NM_001039455/361828, intron 2 of 15)
Ptpn11	25622	chr12	40895571	40895871	56	0.93	4.21E-02	3.07E-01	Promoter (<=1kb)
Chst3	84468	chr20	29765578	29765878	-27170	1.44	4.22E-02	3.07E-01	Distal Intergenic
Lrrfip2	301035	chr8	119387690	119387990	5679	-1.17	4.22E-02	3.07E-01	Intron (NM_001024761/301035, intron 2 of 15)
Srsf7	362687	chr6	2882006	2882306	4159	-1.43	4.22E-02	3.07E-01	Exon (NM_001039035/362687, exon 6 of 8)
Tead2	308582	chr1	101213956	101214256	-337	0.74	4.22E-02	3.07E-01	Promoter (<=1kb)
Sp5	296510	chr3	56608842	56609142	-157333	-1.22	4.22E-02	3.07E-01	Intron (NM_001191901/366069, intron 27 of 30)
Fosl2	25446	chr6	25783063	25783363	-166068	1.44	4.22E-02	3.07E-01	Intron (NM_199270/362704, intron 7 of 11)
Rhno1	297627	chr4	161605424	161605724	79270	-1.54	4.22E-02	3.08E-01	Distal Intergenic
Mir3075	102465157	chr16	1786265	1786565	-36240	-0.95	4.22E-02	3.08E-01	Intron (NM_001108393/361103, intron 1 of 23)
Rap1a	295347	chr2	208271599	208271899	23	1.58	4.22E-02	3.08E-01	Promoter (<=1kb)
R3hdm1	304763	chr13	44804383	44804683	-7884	1.41	4.22E-02	3.08E-01	Distal Intergenic
Trim8	688785	chr1	266230562	266230862	-24935	1	4.23E-02	3.08E-01	Intron (NM_001024899/361769, intron 10 of 11)
Nexn	246172	chr2	257484232	257484532	-9499	1	4.23E-02	3.08E-01	Distal Intergenic
Dpp8	315758	chr8	70521905	70522205	0	0.85	4.23E-02	3.08E-01	Promoter (<=1kb)
Mir3074	100526631	chr17	836105	836405	-12049	-1.29	4.23E-02	3.08E-01	Intron (NM_012557/24361, intron 14 of 14)
Hnrnph1	140931	chr10	35870883	35871183	-1436	0.69	4.23E-02	3.08E-01	Promoter (1-2kb)
Slc25a24	310791	chr2	212006344	212006644	75910	1.14	4.23E-02	3.08E-01	Distal Intergenic
Ttc3	360702	chr11	34598391	34598691	67	0.88	4.23E-02	3.08E-01	Promoter (<=1kb)
Ccnl1	114121	chr2	157755436	157755736	4102	-0.82	4.23E-02	3.08E-01	Intron (NM_053662/114121, intron 3 of 10)
Vps53	287535	chr10	64356629	64356929	-15565	1.75	4.23E-02	3.08E-01	Distal Intergenic
Enpp1	85496	chr1	21770001	21770301	21765	1.8	4.23E-02	3.08E-01	Intron (NM_053535/85496, intron 1 of 24)
Atg14	305831	chr15	24482833	24483133	-108057	1.71	4.23E-02	3.08E-01	Distal Intergenic
Rasl12	315762	chr8	70911555	70911855	-4098	1.69	4.23E-02	3.08E-01	Distal Intergenic
Lima1	300228	chr7	141630450	141630750	-135665	0.61	4.23E-02	3.08E-01	Distal Intergenic
Gli3	140588	chr17	52715560	52715860	-146524	1.44	4.23E-02	3.08E-01	Distal Intergenic
Dhrs7l1	299131	chr6	95371781	95372081	130764	-1.01	4.24E-02	3.08E-01	Distal Intergenic
St3gal1	362924	chr7	108264290	108264590	-358862	1.69	4.24E-02	3.08E-01	Distal Intergenic
Csnk1g3	64823	chr18	48847017	48847317	-5854	-1.28	4.24E-02	3.08E-01	Distal Intergenic
Fzd5	317674	chr9	71426837	71427137	18404	0.95	4.24E-02	3.08E-01	Distal Intergenic
Tspan18	311210	chr3	82185694	82185994	50670	1.68	4.24E-02	3.08E-01	Intron (NM_001107750/311210, intron 2 of 9)
Pgrmc2	361940	chr2	128115967	128116267	-113962	1.66	4.24E-02	3.08E-01	Distal Intergenic
Ndufa8	296658	chr3	15311251	15311551	65216	0.96	4.24E-02	3.08E-01	Distal Intergenic
Otud3	500572	chr5	157356511	157356811	11639	1.1	4.24E-02	3.08E-01	Intron (NM_001191983/500572, intron 3 of 7)
Rabepk	296649	chr3	13868304	13868604	-2461	0.57	4.24E-02	3.08E-01	Promoter (2-3kb)
Dele1	307480	chr18	31360668	31360968	-36019	-1.16	4.24E-02	3.08E-01	Distal Intergenic
Traf3ip2	361857	chr20	44678860	44679160	-1289	1.39	4.24E-02	3.08E-01	Promoter (1-2kb)
Mir190	100314160	chr8	73096863	73097163	-66732	1.2	4.24E-02	3.08E-01	Distal Intergenic
Tmem86a	308602	chr1	103121627	103121927	-51105	-1.02	4.24E-02	3.08E-01	Distal Intergenic
Pwwp3a	362838	chr7	12347844	12348144	43	1.1	4.24E-02	3.08E-01	Promoter (<=1kb)
Bclaf3	501559	chrX	38352976	38353276	185	0.64	4.24E-02	3.08E-01	Promoter (<=1kb)
Lrrc8d	305131	chr14	5035349	5035649	65528	0.8	4.24E-02	3.08E-01	Distal Intergenic
Dnase1	25633	chr10	11785722	11786022	-25102	0.54	4.24E-02	3.08E-01	Distal Intergenic
Ckap4	362859	chr7	24939458	24939758	0	0.69	4.24E-02	3.08E-01	Promoter (<=1kb)
Ehd4	192204	chr3	111928492	111928792	13049	-1.35	4.24E-02	3.08E-01	Intron (NM_139324/192204, intron 1 of 5)
Mill1	292671	chr1	79631495	79631795	-26	-1.07	4.24E-02	3.08E-01	Promoter (<=1kb)
Eif3h	299899	chr6	9969747	9970047	-181685	-1.07	4.24E-02	3.08E-01	Intron (NM_017171/29340, intron 13 of 13)
Mepe	79110	chr14	6752824	6753124	40437	-0.8	4.25E-02	3.08E-01	Distal Intergenic
Scyl2	314717	chr7	30344020	30344320	106	0.7	4.25E-02	3.08E-01	Promoter (<=1kb)
Pbk	290326	chr15	42229746	42230046	-259328	-1.24	4.25E-02	3.08E-01	Distal Intergenic
Sucla2	361071	chr15	55586716	55587016	125021	-1.3	4.25E-02	3.08E-01	Distal Intergenic
Arhgap26	307459	chr18	32205993	32206293	1508	-1.57	4.25E-02	3.08E-01	Promoter (1-2kb)
Rab7a	29448	chr4	119958663	119958963	210	1.01	4.25E-02	3.08E-01	Promoter (<=1kb)
Safb	64196	chr9	10465003	10465303	5800	1.47	4.25E-02	3.08E-01	Intron (NM_022394/64196, intron 2 of 20)
Rasgrp3	313874	chr6	21338951	21339251	-203071	1.41	4.25E-02	3.08E-01	Intron (NM_021587/59107, intron 9 of 33)
Cd180	294706	chr2	32799133	32799433	-20889	-1.25	4.25E-02	3.08E-01	Distal Intergenic
Csdc2	266600	chr7	123163026	123163326	-5485	-1.26	4.25E-02	3.08E-01	Distal Intergenic
Bcl3	680611	chr1	80735991	80736291	8702	-1.27	4.25E-02	3.08E-01	Intron (NM_001109422/680611, intron 2 of 8)
Ptgs2	29527	chr13	67364763	67365063	13533	0.78	4.25E-02	3.08E-01	Distal Intergenic
Zfp608	307296	chr18	50211196	50211496	-273674	-1.45	4.26E-02	3.08E-01	Distal Intergenic
Nr5a2	60349	chr13	53986361	53986661	-116245	1.17	4.26E-02	3.08E-01	Distal Intergenic
Pex11b	310682	chr2	198759036	198759336	-2802	-1.43	4.26E-02	3.08E-01	Promoter (2-3kb)
Kdm1b	306819	chr17	18029241	18029541	-433	0.59	4.26E-02	3.08E-01	Promoter (<=1kb)
Ap1m2	367038	chr8	22351224	22351524	-14397	0.65	4.26E-02	3.08E-01	Distal Intergenic
Btg1	29618	chr7	37906084	37906384	93253	-1.47	4.26E-02	3.08E-01	Distal Intergenic
Aebp1	305494	chr14	86101382	86101682	50	0.55	4.26E-02	3.08E-01	Promoter (<=1kb)
Myrip	360034	chr8	128940265	128940565	-63122	-1.09	4.26E-02	3.08E-01	Distal Intergenic
Man2c1	246136	chr8	61802804	61803104	-2595	-1.33	4.26E-02	3.08E-01	Promoter (2-3kb)
Taf5l	307927	chr19	56800426	56800726	72	0.85	4.26E-02	3.08E-01	Promoter (<=1kb)
Cpq	58952	chr7	71797904	71798204	88565	1.83	4.26E-02	3.08E-01	Intron (NM_031640/58952, intron 2 of 8)
Vps4a	246772	chr19	39230126	39230426	0	0.76	4.26E-02	3.08E-01	Promoter (<=1kb)
Afdn	26955	chr1	53840459	53840759	497	0.74	4.26E-02	3.08E-01	Promoter (<=1kb)
Golgb1	192243	chr11	66818765	66819065	14	0.72	4.26E-02	3.08E-01	Promoter (<=1kb)
Pi4kb	81747	chr2	196139903	196140203	148	0.76	4.26E-02	3.08E-01	Promoter (<=1kb)
Naa60	363545	chr10	11890204	11890504	11261	-0.92	4.26E-02	3.08E-01	Intron (NM_001014226/363545, intron 2 of 6)
Klf15	85497	chr4	122417593	122417893	52500	-1.4	4.27E-02	3.08E-01	Distal Intergenic
Lekr1	361953	chr2	157305751	157306051	-147377	-1.17	4.27E-02	3.08E-01	Distal Intergenic
Mesd	308796	chr1	146163177	146163477	125751	1.31	4.27E-02	3.08E-01	Distal Intergenic
Washc2c	297530	chr4	148114858	148115158	-24379	0.57	4.27E-02	3.08E-01	Distal Intergenic

Crtc1	684527	chr16	20740856	20741156	30	0.74	4.27E-02	3.08E-01	Promoter (<=1kb)
Ehf	295965	chr3	93198422	93198722	17694	1.34	4.27E-02	3.08E-01	Intron (NM_001106493/295965, intron 1 of 8)
Pank4	171053	chr5	172367777	172368077	184	0.55	4.27E-02	3.08E-01	Promoter (<=1kb)
Epha4	316539	chr9	83651181	83651481	-397723	-0.92	4.27E-02	3.08E-01	Distal Intergenic
Cd99	652929	chr20	56189165	56189465	7496	0.6	4.27E-02	3.08E-01	Downstream (2-3kb)
RGD1566099	360851	chr13	55975617	55975917	-89063	-1.34	4.27E-02	3.08E-01	Distal Intergenic
Cysltr2	170926	chr15	54883172	54883472	22731	1.45	4.27E-02	3.08E-01	Distal Intergenic
Hsf2	64441	chr20	38936124	38936424	304	1.09	4.27E-02	3.08E-01	Promoter (<=1kb)
Ip6k1	50560	chr8	116786420	116786720	-17731	1.56	4.27E-02	3.08E-01	Distal Intergenic
Mpp1	652956	chr1	148450103	148450403	0	0.75	4.28E-02	3.08E-01	Promoter (<=1kb)
Wnk1	116477	chr4	152570568	152570868	7535	-1.13	4.28E-02	3.08E-01	Intron (NM_001002823/116477, intron 1 of 27)
Dld	298942	chr6	50461640	50461940	156754	-1.44	4.28E-02	3.08E-01	Distal Intergenic
Tor1aip1	246314	chr13	73637654	73637954	66714	0.82	4.28E-02	3.08E-01	Distal Intergenic
Zfp37	115768	chr5	77944831	77945131	0	0.59	4.28E-02	3.08E-01	Promoter (<=1kb)
Dao	114027	chr12	48382014	48382314	-8367	0.68	4.28E-02	3.08E-01	Distal Intergenic
Tmem184c	291946	chr19	33993210	33993510	-47709	-1.3	4.28E-02	3.08E-01	Distal Intergenic
Hist1h4m	291152	chr17	44793290	44793590	22405	0.6	4.28E-02	3.08E-01	Distal Intergenic
Fbxo39	303287	chr10	58861269	58861569	-14262	-0.79	4.28E-02	3.08E-01	Distal Intergenic
Lsm5	306222	chr4	87008942	87009242	9977	-1.23	4.28E-02	3.08E-01	Distal Intergenic
Zfp39	303173	chr10	45243272	45243572	81	0.65	4.28E-02	3.08E-01	Promoter (<=1kb)
Sod3	25352	chr14	60956630	60956930	7394	-1.16	4.28E-02	3.08E-01	Downstream (1-2kb)
Prickle1	315259	chr7	135062828	135063128	-340613	0.86	4.28E-02	3.08E-01	Distal Intergenic
Gtpbp3	290633	chr16	19903793	19904093	6658	0.74	4.29E-02	3.08E-01	Downstream (2-3kb)
Xylb	316067	chr8	128044124	128044424	0	0.7	4.29E-02	3.08E-01	Promoter (<=1kb)
Sult5a1	292077	chr19	55967212	55967512	196	1.26	4.29E-02	3.08E-01	Promoter (<=1kb)
Pdlim5	64353	chr2	247864827	247865127	123335	-1.04	4.29E-02	3.08E-01	Intron (NM_053326/64353, intron 7 of 12)
Tbcc	316221	chr9	16432098	16432398	-25760	-1.38	4.29E-02	3.08E-01	Distal Intergenic
Dpp9	301130	chr9	10876794	10877094	30264	-1.26	4.29E-02	3.08E-01	Intron (NM_001305241/301130, intron 13 of 20)
Scel	361086	chr15	87540181	87540481	-163859	0.84	4.29E-02	3.08E-01	Distal Intergenic
Pgr	25154	chr8	7129495	7129795	839	0.67	4.29E-02	3.08E-01	Promoter (<=1kb)
Ido1	66029	chr16	72269122	72269422	52796	-1.05	4.29E-02	3.08E-01	Distal Intergenic
Ccsap	307926	chr19	56632640	56632940	185	0.76	4.29E-02	3.08E-01	Promoter (<=1kb)
Slc12a2	83629	chr18	52896774	52897074	-20050	-0.99	4.29E-02	3.08E-01	Distal Intergenic
Fmo3	84493	chr13	80852989	80853289	2925	1.83	4.29E-02	3.08E-01	Promoter (2-3kb)
Lhfpl2	294643	chr2	23608282	23608582	-162126	-1.25	4.29E-02	3.08E-01	Distal Intergenic
Lrrc49	300763	chr8	65482864	65483164	104263	1.87	4.29E-02	3.08E-01	Intron (NM_001134469/300763, intron 15 of 15)
Hnf1a	24817	chr12	47362295	47362595	-45216	0.57	4.29E-02	3.08E-01	Distal Intergenic
B4galt4	303923	chr11	64583714	64584014	0	1.22	4.29E-02	3.08E-01	Promoter (<=1kb)
Jag1	29146	chr3	130061084	130061384	53397	1.85	4.29E-02	3.08E-01	Intron (NM_001271320/499895, intron 6 of 7)
Adcyap1	24166	chr9	121539998	121540298	172826	-0.94	4.30E-02	3.08E-01	Distal Intergenic
Frmd4a	307128	chr17	77839241	77839541	78669	-1.21	4.30E-02	3.08E-01	Intron (NM_001191821/307128, intron 1 of 21)
Atxn1	25049	chr17	19364176	19364476	203182	1.37	4.30E-02	3.08E-01	Intron (NM_012726/25049, intron 3 of 6)
Tprg1l	687090	chr5	171459096	171459396	92	0.97	4.30E-02	3.08E-01	Promoter (<=1kb)
Rnf7	300948	chr8	104367070	104367370	-31341	0.88	4.30E-02	3.08E-01	Distal Intergenic
Leprotl1	361160	chr16	61758841	61759141	0	1.03	4.30E-02	3.08E-01	Promoter (<=1kb)
Atp6v1f	116664	chr4	56748374	56748674	3811	1.53	4.30E-02	3.08E-01	Distal Intergenic
Tcf7l2	679869	chr1	276632451	276632751	-72094	1.61	4.30E-02	3.09E-01	Distal Intergenic
Mir125b2	100314025	chr11	16091053	16091353	-5993	-1.39	4.30E-02	3.09E-01	Distal Intergenic
Tns1	301509	chr9	81372988	81373288	27699	1.36	4.30E-02	3.09E-01	Intron (NM_001191810/301509, intron 1 of 34)
Inha	24504	chr9	82691067	82691367	-9115	-1.36	4.30E-02	3.09E-01	Distal Intergenic
Gnpda2	289608	chr14	62645705	62646005	105	0.67	4.31E-02	3.09E-01	Promoter (<=1kb)
Rab3gap2	289350	chr13	103168923	103169223	11089	-0.76	4.31E-02	3.09E-01	Intron (NM_001040154/289350, intron 7 of 40)
Cdca2	305984	chr15	44318767	44319067	91937	1.43	4.31E-02	3.09E-01	Distal Intergenic
Adamts7	315879	chr8	97573346	97573646	37569	-1.47	4.31E-02	3.09E-01	Intron (NM_001047101/315879, intron 25 of 25)
Rhbdd1	316557	chr9	88212009	88212309	78125	-1.35	4.31E-02	3.09E-01	Intron (NM_001024891/316557, intron 6 of 6)
Hat1	296501	chr3	57963445	57963745	-58650	-1.61	4.31E-02	3.09E-01	Distal Intergenic
Rab21	299799	chr7	58219361	58219661	67109	0.59	4.32E-02	3.09E-01	Distal Intergenic
Gpd2	25062	chr3	43214214	43214514	-9413	-1.25	4.32E-02	3.09E-01	Distal Intergenic
Sec61b	298068	chr5	63222547	63222847	30225	-1.38	4.32E-02	3.09E-01	Distal Intergenic
Clic4	83718	chr5	153628590	153628890	-2921	-1.48	4.32E-02	3.09E-01	Promoter (2-3kb)
Zbtb17	313666	chr5	159993980	159994280	181	1.53	4.32E-02	3.09E-01	Promoter (<=1kb)
Htra3	360959	chr14	80252666	80252966	4526	1.08	4.32E-02	3.09E-01	Intron (NM_001271027/360959, intron 1 of 8)
LOC100911177	100911177	chr3	163829297	163829597	13977	-1.35	4.32E-02	3.09E-01	Exon (NM_001047860/296384, exon 4 of 17)
Slc39a14	306009	chr15	52009788	52010088	19728	-1.28	4.32E-02	3.09E-01	5' UTR
Il34	498951	chr19	40441946	40442246	-448298	0.84	4.32E-02	3.09E-01	Distal Intergenic
Ctcf	83726	chr19	37600824	37601124	447	0.75	4.32E-02	3.09E-01	Promoter (<=1kb)
Fez2	94269	chr6	1000370	1000670	3254	-1.04	4.32E-02	3.09E-01	Intron (NM_053600/94269, intron 1 of 8)
Pitpna	29525	chr10	63705674	63705974	-25793	-1.33	4.32E-02	3.09E-01	Intron (NM_001105812/287532, intron 6 of 13)
Rpl3	300079	chr7	121297967	121298267	4472	-1.22	4.33E-02	3.09E-01	Exon (NM_198753/300079, exon 7 of 10)
Tac3	29191	chr7	71026516	71026816	2540	1.58	4.33E-02	3.09E-01	Promoter (2-3kb)
Ythdc1	170956	chr14	23083705	23084005	-81694	1.49	4.33E-02	3.09E-01	Distal Intergenic
Faim	140930	chr8	107217516	107217816	-7396	1.35	4.33E-02	3.09E-01	Distal Intergenic
Gjc2	497913	chr10	45535207	45535507	-637	1.12	4.33E-02	3.09E-01	Promoter (<=1kb)
Jdp2	116674	chr6	109491945	109492245	17505	1.31	4.33E-02	3.09E-01	Intron (NM_053894/116674, intron 1 of 2)
Asap1	314961	chr7	104947283	104947583	3507	1.82	4.33E-02	3.09E-01	Intron (NM_001044245/314961, intron 1 of 27)
Abcc2	25303	chr1	263606840	263607140	52414	-1.09	4.33E-02	3.09E-01	Exon (NM_012833/25303, exon 29 of 32)
Osbpl1a	259221	chr18	4308608	4308908	-14472	0.58	4.33E-02	3.09E-01	Distal Intergenic
E2f3	291105	chr17	36333446	36333746	-390	0.67	4.33E-02	3.09E-01	Promoter (<=1kb)
Lama1	316758	chr9	115954555	115954855	37648	-1.18	4.33E-02	3.09E-01	Intron (NM_001108237/316758, intron 3 of 62)
Srpk1	361811	chr20	5871070	5871370	-5216	1.7	4.33E-02	3.09E-01	Distal Intergenic
Ndufaf7	298748	chr6	1560347	1560647	25753	-1.32	4.33E-02	3.09E-01	Intron (NM_001024263/313834, intron 11 of 18)
Tnfrsf26	361685	chr1	216835430	216835730	-6849	-1.37	4.33E-02	3.09E-01	Distal Intergenic
Dgkq	100361138	chr14	2069440	2069740	31	0.65	4.33E-02	3.09E-01	Promoter (<=1kb)
Csnk1d	64462	chr10	110202415	110202715	-20007	-1.21	4.33E-02	3.09E-01	Distal Intergenic
Adck2	312258	chr4	67359625	67359925	94	0.59	4.33E-02	3.09E-01	Promoter (<=1kb)
Gak	81659	chr14	2118725	2119025	18619	1.34	4.34E-02	3.09E-01	Intron (NM_031030/81659, intron 6 of 27)
Tbcd	100361417	chr10	110737764	110738064	94005	1.75	4.34E-02	3.09E-01	Intron (NM_001271364/100361417, intron 15 of 38)
Septin5	116728	chr11	86512826	86513126	-3251	0.72	4.34E-02	3.10E-01	Distal Intergenic
Ywhae	29753	chr10	63901190	63901490	16852	1.25	4.34E-02	3.10E-01	Intron (NM_031603/29753, intron 1 of 5)
Dlc1	58834	chr16	59092477	59092777	-104590	-1.65	4.34E-02	3.10E-01	Distal Intergenic
Rpe	501157	chr9	73623941	73624241	-63545	-1.65	4.34E-02	3.10E-01	Distal Intergenic
Sdf4	155173	chr5	173431101	173431401	5179	-1.02	4.34E-02	3.10E-01	Exon (NM_130412/155173, exon 2 of 7)
Hand2	64637	chr16	36373649	36373949	-100	0.82	4.34E-02	3.10E-01	Promoter (<=1kb)
Irf1	24508	chr10	39140192	39140492	30662	-1.38	4.34E-02	3.10E-01	Distal Intergenic

Sec22b	310710	chr2	200106752	200107052	30673	-1.38	4.34E-02	3.10E-01	Distal Intergenic
Helz	287773	chr10	95768885	95769185	-969	0.71	4.34E-02	3.10E-01	Promoter (<=1kb)
Phf12	303274	chr10	64813584	64813884	48404	0.92	4.34E-02	3.10E-01	Downstream (1-2kb)
Gtf2a1	83830	chr6	115386403	115386703	-33744	1.44	4.34E-02	3.10E-01	Distal Intergenic
Trip10	116717	chr9	9701579	9701879	474	0.53	4.34E-02	3.10E-01	Promoter (<=1kb)
Dpysl3	25418	chr18	37746326	37746626	29827	-1.25	4.34E-02	3.10E-01	Intron (NM_012934/25418, intron 2 of 13)
Fbxo42	362646	chr5	159686884	159687184	16222	-1.38	4.35E-02	3.10E-01	Intron (NM_001108691/362646, intron 1 of 9)
Hnrnpl	80846	chr1	86988017	86988317	-7776	1.27	4.35E-02	3.10E-01	Distal Intergenic
Radil	304299	chr12	14092615	14092915	74	0.85	4.35E-02	3.10E-01	Promoter (<=1kb)
N4bp1	291921	chr19	21218334	21218634	0	1.05	4.35E-02	3.10E-01	Promoter (<=1kb)
Lhfpl2	294643	chr2	23609449	23609749	-160959	-1.28	4.35E-02	3.10E-01	Distal Intergenic
Sec24d	310843	chr2	227455728	227456028	5	0.82	4.35E-02	3.10E-01	Promoter (<=1kb)
Lmcd1	494021	chr4	144174537	144174837	-18152	-1.42	4.35E-02	3.10E-01	Distal Intergenic
Zfp691	313548	chr5	138201403	138201703	20831	-0.78	4.36E-02	3.10E-01	Distal Intergenic
Ece1	94204	chr5	156233620	156233920	18151	-1.31	4.36E-02	3.10E-01	Intron (NM_053596/94204, intron 1 of 18)
Sec31a	93646	chr14	10854679	10854979	0	0.64	4.36E-02	3.10E-01	Promoter (<=1kb)
Trim63	140939	chr5	152533682	152533982	320	1.06	4.36E-02	3.10E-01	Promoter (<=1kb)
Eepd1	315500	chr8	26651323	26651623	-498	0.66	4.36E-02	3.10E-01	Promoter (<=1kb)
Crls1	366196	chr3	125506975	125507275	3337	1.63	4.36E-02	3.10E-01	Intron (NM_001014258/366196, intron 2 of 6)
Pfdn1	361310	chr18	29180114	29180414	110033	-0.7	4.36E-02	3.10E-01	Distal Intergenic
Jakmip2	307479	chr18	38032392	38032692	55664	-1.79	4.37E-02	3.10E-01	Intron (NM_001107391/307479, intron 1 of 25)
Tprn	499749	chr3	2480326	2480626	94	0.73	4.37E-02	3.10E-01	Promoter (<=1kb)
Ube2n	116725	chr7	36623506	36623806	13359	-1.32	4.37E-02	3.10E-01	Intron (NM_053928/116725, intron 1 of 3)
Mbd1	291439	chr18	70316126	70316426	67413	1.19	4.37E-02	3.10E-01	Distal Intergenic
Fam131b	500102	chr4	71712978	71713278	0	1.23	4.37E-02	3.10E-01	Promoter (<=1kb)
Usp25	304150	chr11	15442362	15442662	6093	-1.33	4.37E-02	3.10E-01	Intron (NM_001107114/304150, intron 1 of 23)
Grik4	24406	chr8	47237067	47237367	-129150	1.35	4.37E-02	3.10E-01	Distal Intergenic
Swt1	289088	chr13	68785396	68785696	75	0.69	4.37E-02	3.10E-01	Promoter (<=1kb)
Mir107	100314022	chr1	253122597	253122897	5439	-1.33	4.37E-02	3.10E-01	Intron (NM_001106373/294088, intron 5 of 6)
Dyrk2	314862	chr7	61980336	61980636	-181607	1.31	4.37E-02	3.10E-01	Distal Intergenic
B3galnt1	310508	chr2	166255476	166255776	-174	0.81	4.37E-02	3.10E-01	Promoter (<=1kb)
Tnfrsf1a	25625	chr4	157913653	157913953	48663	-1.33	4.37E-02	3.10E-01	Distal Intergenic
Dip2c	307067	chr17	63454780	63455080	27764	-1.24	4.37E-02	3.10E-01	Intron (NM_001107360/307067, intron 1 of 37)
Fgfbp1	64535	chr14	71668407	71668707	19133	1.52	4.37E-02	3.10E-01	Distal Intergenic
R3hdm1	304763	chr13	44770271	44770571	-41996	1.12	4.37E-02	3.10E-01	Distal Intergenic
Aanat	25120	chr10	105552529	105552829	-15262	0.76	4.37E-02	3.10E-01	Distal Intergenic
Aff1	305152	chr14	7379170	7379470	-53525	-1.6	4.38E-02	3.11E-01	Distal Intergenic
Foxa1	25098	chr6	78602177	78602477	-52508	0.98	4.38E-02	3.11E-01	Distal Intergenic
Paqr8	316275	chr9	26986819	26987119	-46903	-0.89	4.38E-02	3.11E-01	Distal Intergenic
Msn	81521	chrX	65191337	65191637	-35197	-0.94	4.38E-02	3.11E-01	Distal Intergenic
Orc5	362304	chr4	9160218	9160518	113	0.65	4.38E-02	3.11E-01	Promoter (<=1kb)
Mir140	100314276	chr19	39623997	39624297	15046	-0.92	4.38E-02	3.11E-01	Distal Intergenic
Bckdk	29603	chr1	199349304	199349604	-2024	-1.31	4.38E-02	3.11E-01	Promoter (2-3kb)
Dag1	114489	chr8	116997732	116998032	-4550	1.67	4.38E-02	3.11E-01	Distal Intergenic
Acap3	313772	chr5	173344790	173345090	4730	1.5	4.38E-02	3.11E-01	5' UTR
Sp7	300260	chr7	143983677	143983977	-16193	0.65	4.39E-02	3.11E-01	Distal Intergenic
Rn7sl1	102723236	chr6	91678954	91679254	1270	1.78	4.39E-02	3.11E-01	Promoter (1-2kb)
Nek9	299204	chr6	109162054	109162354	55	0.57	4.39E-02	3.11E-01	Promoter (<=1kb)
Uqcc3	690344	chr1	225072549	225072849	4230	1.5	4.39E-02	3.11E-01	Distal Intergenic
Fbxl5	305424	chr14	71852867	71853167	137	1.32	4.39E-02	3.11E-01	Promoter (<=1kb)
Tmem107	691750	chr10	55649965	55650265	-3681	0.53	4.39E-02	3.11E-01	Distal Intergenic
Eif4g2	361628	chr1	175957039	175957339	-61529	1.72	4.39E-02	3.11E-01	Distal Intergenic
Padi6	298595	chr5	159209403	159209703	46996	-1.15	4.39E-02	3.11E-01	Distal Intergenic
Btg1	29618	chr7	37812934	37813234	103	0.77	4.39E-02	3.11E-01	Promoter (<=1kb)
Ndrg1	299923	chr7	107775197	107775497	204	0.65	4.39E-02	3.11E-01	Promoter (<=1kb)
Cisd1	294362	chr20	18493562	18493862	24	0.53	4.39E-02	3.11E-01	Promoter (<=1kb)
Mapk8	116554	chr16	9708932	9709232	110	0.92	4.39E-02	3.11E-01	Promoter (<=1kb)
Dtnbp1	641528	chr17	20093189	20093489	3053	-1.38	4.39E-02	3.11E-01	Intron (NM_001037664/641528, intron 1 of 8)
RGD1308706	291925	chr19	22673441	22673741	-26067	0.65	4.39E-02	3.11E-01	Distal Intergenic
Slc9a8	311651	chr3	164174353	164174653	0	0.63	4.39E-02	3.11E-01	Promoter (<=1kb)
Crip3	501100	chr9	16978801	16979101	-44087	1.51	4.40E-02	3.11E-01	Distal Intergenic
Cd200	24560	chr11	60375312	60375612	3583	-1.31	4.40E-02	3.11E-01	Intron (NM_031518/24560, intron 1 of 5)
Mb	59108	chr7	118202562	118202862	-93698	-1.03	4.40E-02	3.11E-01	Intron (NM_001079895/362950, intron 2 of 12)
Fam185a	499979	chr4	10270341	10270641	-342	0.64	4.40E-02	3.11E-01	Promoter (<=1kb)
Baz1a	314126	chr6	75877248	75877548	-3394	1.31	4.40E-02	3.11E-01	Distal Intergenic
Sntb1	299940	chr7	95784811	95785111	-115036	-1.34	4.40E-02	3.11E-01	Distal Intergenic
Impad1	312952	chr5	17590684	17590984	72743	-1.42	4.40E-02	3.11E-01	Distal Intergenic
Tnn	304913	chr13	77891218	77891518	5223	1.44	4.40E-02	3.11E-01	Intron (NM_001107189/304913, intron 1 of 21)
Map3k10	308463	chr1	84491273	84491573	0	1.04	4.41E-02	3.11E-01	Promoter (<=1kb)
Alg5	295051	chr2	143951753	143952053	19014	0.8	4.41E-02	3.11E-01	Distal Intergenic
Gnb5	83579	chr8	82236375	82236675	-21174	-1.3	4.41E-02	3.12E-01	Distal Intergenic
Cwc15	300361	chr8	13109188	13109488	115033	0.57	4.42E-02	3.12E-01	Distal Intergenic
Atp6v0e1	94170	chr10	16814349	16814649	-21419	0.59	4.42E-02	3.12E-01	Distal Intergenic
Msl1	303514	chr10	86716614	86716914	5374	-1.45	4.42E-02	3.12E-01	Intron (NM_001107048/303514, intron 3 of 7)
LOC102554317	102554317	chr11	31587933	31588233	-10013	-1.45	4.42E-02	3.12E-01	Distal Intergenic
Cab39	301574	chr9	92788461	92788761	-45596	-1.45	4.42E-02	3.12E-01	Distal Intergenic
Gab2	84477	chr1	162082922	162083222	0	1.47	4.42E-02	3.12E-01	Promoter (<=1kb)
Itgb5	257645	chr11	70322506	70322806	-150342	1	4.42E-02	3.12E-01	Distal Intergenic
Amn1	302032	chr4	183764737	183765037	-67206	-1.73	4.42E-02	3.12E-01	Distal Intergenic
Ireb2	64831	chr8	59464492	59464792	7474	-1.73	4.42E-02	3.12E-01	Intron (NM_022863/64831, intron 2 of 21)
Cwc27	361887	chr2	34854705	34855005	67716	-1.28	4.42E-02	3.12E-01	Intron (NM_001013199/361887, intron 10 of 13)
Ociad1	289590	chr14	37515156	37515456	-39738	0.71	4.42E-02	3.12E-01	Distal Intergenic
Mier3	310086	chr2	43302631	43302931	31539	0.74	4.42E-02	3.12E-01	Distal Intergenic
LOC499469	499469	chr20	46789256	46789556	81894	-1.06	4.42E-02	3.12E-01	Distal Intergenic
Pgm1	24645	chr5	118772547	118772847	28915	-0.95	4.42E-02	3.12E-01	Intron (NM_017033/24645, intron 1 of 10)
Tm4sf4	116467	chr2	147591352	147591652	59320	-0.93	4.42E-02	3.12E-01	Intron (NM_001024869/295062, intron 4 of 6)
Chmp4bl1	679886	chr4	66899707	66900007	-159932	1.14	4.42E-02	3.12E-01	Distal Intergenic
Mtmr2	315422	chr8	12203730	12204030	-79645	-1.01	4.43E-02	3.12E-01	Distal Intergenic
Rdh10	353252	chr5	2632401	2632701	-496	0.87	4.43E-02	3.12E-01	Promoter (<=1kb)
Tfrc	64678	chr11	71418818	71419118	145	0.92	4.43E-02	3.12E-01	Promoter (<=1kb)
Mcart1	313241	chr5	61076951	61077251	52775	0.98	4.43E-02	3.12E-01	Distal Intergenic
Cycs	25309	chr4	80320240	80320540	12786	1.38	4.43E-02	3.12E-01	Distal Intergenic
LOC100911360	100911360	chr8	74178145	74178445	-643150	1.86	4.43E-02	3.12E-01	Distal Intergenic
Pdk2	81530	chr10	82887149	82887449	-34391	0.6	4.43E-02	3.12E-01	Distal Intergenic

Nfkb1	81736	chr2	240822983	240823283	66770	-1.18	4.43E-02	3.12E-01	Intron (NM_001276711/81736, intron 6 of 24)
Gbe1	288333	chr11	7210248	7210548	79	0.61	4.43E-02	3.12E-01	Promoter (<=1kb)
Pum2	298874	chr6	33789969	33790269	3342	-1.33	4.43E-02	3.12E-01	Intron (NM_001106715/298874, intron 1 of 20)
Phox2a	116648	chr1	166832660	166832960	-60774	-1.35	4.43E-02	3.12E-01	Intron (NM_022947/65041, intron 6 of 15)
Nxnl2	689232	chr17	14058516	14058816	0	0.8	4.43E-02	3.12E-01	Promoter (<=1kb)
Cfap77	499765	chr3	7599922	7600222	32089	-1.36	4.43E-02	3.12E-01	Intron (NM_001127563/499765, intron 1 of 5)
Ybx1	500538	chr5	138335693	138335993	488	0.99	4.44E-02	3.12E-01	Promoter (<=1kb)
Nrep	338475	chr18	26213463	26213763	-2018	1.08	4.44E-02	3.12E-01	Promoter (2-3kb)
Cul2	361258	chr17	56992274	56992574	0	0.61	4.44E-02	3.12E-01	Promoter (<=1kb)
Retreg1	619558	chr2	78103490	78103790	35	0.83	4.44E-02	3.12E-01	Promoter (<=1kb)
Myo1e	25484	chr8	76752932	76753232	108217	-1.27	4.44E-02	3.12E-01	Intron (NM_173101/25484, intron 4 of 26)
Atp1b1	25650	chr13	82721943	82722243	35438	-1.15	4.44E-02	3.12E-01	Intron (NM_138532/171566, intron 17 of 17)
Matr3	29150	chr18	28364613	28364913	3330	-0.85	4.44E-02	3.12E-01	Intron (NM_019149/29150, intron 1 of 14)
Smarcad1	312398	chr4	95884318	95884618	-125	1.1	4.44E-02	3.12E-01	Promoter (<=1kb)
Rnft2	304521	chr12	43959567	43959867	18638	1.74	4.44E-02	3.12E-01	Intron (NM_001107144/304521, intron 7 of 11)
Hcn4	59266	chr8	63743248	63743548	143341	-1.3	4.44E-02	3.12E-01	Distal Intergenic
Masp1	64023	chr11	80757294	80757594	20628	1.4	4.45E-02	3.13E-01	Intron (NM_022257/64023, intron 2 of 15)
Ptov1	292888	chr1	100865534	100865834	60	1.28	4.45E-02	3.13E-01	Promoter (<=1kb)
Pygo1	691857	chr8	79606622	79606922	-91	1.12	4.45E-02	3.13E-01	Promoter (<=1kb)
Specc1l	361828	chr20	14393419	14393719	153	0.85	4.45E-02	3.13E-01	Promoter (<=1kb)
Sox11	84046	chr6	46778969	46779269	-146981	0.92	4.45E-02	3.13E-01	Distal Intergenic
Zbtb38	315936	chr8	104593425	104593725	0	-1.25	4.45E-02	3.13E-01	Promoter (<=1kb)
Il1a	24493	chr3	121855361	121855661	-19239	-0.68	4.45E-02	3.13E-01	Distal Intergenic
Gsdme	353316	chr4	80128391	80128691	-128713	-1.11	4.45E-02	3.13E-01	Distal Intergenic
Kmt5a	689820	chr12	37574485	37574785	0	0.91	4.45E-02	3.13E-01	Promoter (<=1kb)
Csrp1	29276	chr13	52450627	52450927	-102916	-1.38	4.45E-02	3.13E-01	Distal Intergenic
Slc6a4	25553	chr10	63193898	63194198	-19078	-1.28	4.45E-02	3.13E-01	Distal Intergenic
Mepe	79110	chr14	6771474	6771774	21787	-1.37	4.46E-02	3.13E-01	Distal Intergenic
Lppos	106455137	chr11	79772777	79773077	-54651	1.14	4.46E-02	3.13E-01	Distal Intergenic
Cmss1	288176	chr11	44989517	44989817	-41880	-1.4	4.46E-02	3.13E-01	Intron (NM_001107100/304021, intron 2 of 3)
Luzp1	79428	chr5	154884330	154884630	52592	-1.57	4.46E-02	3.13E-01	Intron (NM_030830/79428, intron 2 of 4)
Ankrd13a	360823	chr12	47551862	47552162	36173	0.93	4.46E-02	3.13E-01	Distal Intergenic
Fnta	25318	chr16	70804296	70804596	-30361	-1.23	4.46E-02	3.13E-01	Intron (NM_001136098/306548, intron 16 of 27)
Tmod2	58814	chr8	82546918	82547218	-13239	1.05	4.46E-02	3.13E-01	Distal Intergenic
Mtss2	307845	chr19	40941578	40941878	-15918	1.72	4.46E-02	3.13E-01	Intron (NM_177930/307842, intron 14 of 18)
Ccdc50	288022	chr11	76804186	76804486	0	1.05	4.46E-02	3.13E-01	Promoter (<=1kb)
Cnot6l	360917	chr14	15058816	15059116	-271	1.45	4.46E-02	3.13E-01	Promoter (<=1kb)
Dmac1	298147	chr5	93368002	93368302	-1328856	1.65	4.46E-02	3.13E-01	Distal Intergenic
Pdlim5	64353	chr2	247943836	247944136	44326	1.66	4.46E-02	3.13E-01	Intron (NM_053326/64353, intron 2 of 12)
LOC499331	499331	chr1	234634674	234634974	35139	1.66	4.46E-02	3.13E-01	Distal Intergenic
Hint1	690660	chr10	40208441	40208741	205	0.81	4.46E-02	3.13E-01	Promoter (<=1kb)
Tnfrsf26	361685	chr1	216822940	216823240	5341	-1.05	4.46E-02	3.13E-01	Intron (NM_001108511/361685, intron 1 of 7)
Wwtr1	295062	chr2	147651204	147651504	41529	-1.33	4.46E-02	3.13E-01	Intron (NM_001024869/295062, intron 2 of 6)
Sdc1	25216	chr6	33870076	33870376	-15200	1.4	4.47E-02	3.13E-01	Distal Intergenic
Rnf10	288710	chr12	47103381	47103681	68	0.81	4.47E-02	3.13E-01	Promoter (<=1kb)
Uhrf1	316129	chr9	10756969	10757269	1134	0.54	4.47E-02	3.13E-01	Promoter (1-2kb)
Top2a	360243	chr10	86928463	86928763	2186	-1.01	4.47E-02	3.13E-01	Promoter (2-3kb)
Lrp6	312781	chr4	168279091	168279391	17982	-1.27	4.47E-02	3.13E-01	Intron (NM_001107892/312781, intron 1 of 19)
Mapk14	81649	chr20	5944144	5944444	10841	-1.27	4.47E-02	3.13E-01	Intron (NM_031020/81649, intron 1 of 11)
Sntb2	689421	chr19	39159352	39159652	32763	-1.08	4.47E-02	3.13E-01	Intron (NM_001168674/689421, intron 1 of 6)
Flnb	306204	chr15	18882592	18882892	67	0.56	4.47E-02	3.13E-01	Promoter (<=1kb)
Smad2	29357	chr18	72635585	72635885	85131	-1.08	4.47E-02	3.13E-01	Distal Intergenic
Armc5	361653	chr1	199650963	199651263	-4384	-1.62	4.47E-02	3.13E-01	Distal Intergenic
Pgm3	363109	chr8	94243163	94243463	0	0.63	4.47E-02	3.13E-01	Promoter (<=1kb)
Agpat4	170919	chr1	48824923	48825223	155	0.66	4.47E-02	3.13E-01	Promoter (<=1kb)
Mecp2	29386	chrX	156650644	156650944	255	0.62	4.47E-02	3.13E-01	Promoter (<=1kb)
Gucy1a2	66012	chr8	876718	877018	468717	1.8	4.47E-02	3.13E-01	Intron (NM_023956/66012, intron 7 of 7)
Tcp11l1	499846	chr3	94657094	94657394	73	0.94	4.48E-02	3.13E-01	Promoter (<=1kb)
Olr1387	287241	chr10	34542161	34542461	-10823	1.05	4.48E-02	3.13E-01	Distal Intergenic
Zmynd8	296374	chr3	162611320	162611620	-32119	-1.12	4.48E-02	3.13E-01	Distal Intergenic
Rad51c	497976	chr10	74735776	74736076	-11829	1.7	4.48E-02	3.13E-01	Distal Intergenic
Col11a1	25654	chr2	216817550	216817850	-45578	1.57	4.48E-02	3.13E-01	Distal Intergenic
Paqr3	305203	chr14	14107510	14107810	0	0.71	4.48E-02	3.13E-01	Promoter (<=1kb)
Cd101	310727	chr2	203431759	203432059	-18634	1.26	4.49E-02	3.13E-01	Intron (NM_019243/29602, intron 7 of 8)
Dhx33	287464	chr10	57637945	57638245	240	0.6	4.49E-02	3.13E-01	Promoter (<=1kb)
Sil1	291673	chr18	28327160	28327460	-25297	0.83	4.49E-02	3.14E-01	Distal Intergenic
Foxp1	297480	chr4	131522777	131523077	75227	0.99	4.49E-02	3.14E-01	Intron (NM_001034131/297480, intron 1 of 15)
Rev3l	309812	chr20	44798315	44798615	-5051	1.85	4.49E-02	3.14E-01	Distal Intergenic
Ca4	29242	chr10	72238924	72239224	-33062	-1.2	4.49E-02	3.14E-01	Distal Intergenic
Arrb1	25387	chr1	164500435	164500735	-1717	1.37	4.49E-02	3.14E-01	Promoter (1-2kb)
Vof16	259227	chr8	45811434	45811734	13078	-1.02	4.49E-02	3.14E-01	Distal Intergenic
Trir	288920	chr19	26143057	26143357	337	0.65	4.49E-02	3.14E-01	Promoter (<=1kb)
Cxcr3	84475	chrX	71742247	71742547	-125250	0.58	4.49E-02	3.14E-01	Distal Intergenic
Nfix	81524	chr19	25913828	25914128	568	0.62	4.50E-02	3.14E-01	Promoter (<=1kb)
Arl6ip5	66028	chr4	129857484	129857784	283121	1.59	4.50E-02	3.14E-01	Distal Intergenic
Pars2	313429	chr5	126253316	126253616	-526	1.38	4.50E-02	3.14E-01	Promoter (<=1kb)
Nxnl2	689232	chr17	14209512	14209812	-150870	0.75	4.50E-02	3.14E-01	Distal Intergenic
Tcaim	363169	chr8	131845835	131846135	139	0.54	4.50E-02	3.14E-01	Promoter (<=1kb)
Ilkap	64538	chr9	98536370	98536670	0	1.01	4.50E-02	3.14E-01	Promoter (<=1kb)
Dock9	259237	chr15	108210762	108211062	0	1.19	4.50E-02	3.14E-01	Promoter (<=1kb)
Twist2	59327	chr9	98937771	98938071	13637	-1.16	4.50E-02	3.14E-01	Intron (NM_021691/59327, intron 1 of 1)
Prpf18	171552	chr17	77601945	77602245	31	0.56	4.50E-02	3.14E-01	Promoter (<=1kb)
Exoc1	305287	chr14	34115242	34115542	65031	0.85	4.50E-02	3.14E-01	Distal Intergenic
Myo1e	25484	chr8	76715359	76715659	70644	-1.32	4.50E-02	3.14E-01	Intron (NM_173101/25484, intron 1 of 26)
Mapk14	81649	chr20	5937376	5937676	4073	-1.12	4.50E-02	3.14E-01	Intron (NM_031020/81649, intron 1 of 11)
Fign	295649	chr3	50260293	50260593	-139901	1.72	4.50E-02	3.14E-01	Distal Intergenic
Sertad2	498423	chr14	104764538	104764838	-56377	1.14	4.50E-02	3.14E-01	Distal Intergenic
Gatad2a	290669	chr16	21198555	21198855	20762	0.85	4.50E-02	3.14E-01	Intron (NM_001013881/290669, intron 1 of 12)
Itpr1	25262	chr4	140304449	140304749	57114	-1.43	4.50E-02	3.14E-01	Intron (NM_001007235/25262, intron 4 of 61)
Apc	24205	chr18	27011856	27012156	-35226	0.98	4.50E-02	3.14E-01	Distal Intergenic
Klrb1c	683758	chr4	162576139	162576439	34200	1.45	4.50E-02	3.14E-01	Distal Intergenic
Fzd5	317674	chr9	71544200	71544500	-98659	0.94	4.51E-02	3.14E-01	Distal Intergenic
Usp32	303394	chr10	72440188	72440488	-23118	-1.32	4.51E-02	3.14E-01	Distal Intergenic
Dusp6	116663	chr7	41678744	41679044	203581	-1.04	4.51E-02	3.14E-01	Distal Intergenic

Fgfr3	84489	chr14	82287050	82287350	0	0.68	4.51E-02	3.14E-01	Promoter (<=1kb)
Cdkn2aip	306455	chr16	47655882	47656182	-9634	1.03	4.51E-02	3.14E-01	Distal Intergenic
Gng2	80850	chr15	4658239	4658539	-89703	0.66	4.51E-02	3.14E-01	Distal Intergenic
Btg1	29618	chr7	37750169	37750469	-62362	-1.35	4.51E-02	3.14E-01	Distal Intergenic
Ogg1	81528	chr4	145278176	145278476	-4567	1.15	4.51E-02	3.14E-01	Intron (NM_001191572/679713, intron 11 of 12)
Dusp16	297682	chr4	168553121	168553421	-36142	-1.23	4.51E-02	3.14E-01	Distal Intergenic
Dnajc5	79130	chr3	177013310	177013610	0	0.7	4.52E-02	3.14E-01	Promoter (<=1kb)
Prkd1	85421	chr6	71343828	71344128	5121	-1.29	4.52E-02	3.14E-01	Intron (NM_001276715/85421, intron 1 of 18)
Eif6	305506	chr3	151384416	151384716	-21301	-1.14	4.52E-02	3.14E-01	Downstream (1-2kb)
Akr1b8	286921	chr4	61772295	61772595	231	-0.67	4.52E-02	3.14E-01	Promoter (<=1kb)
Ltbp1	59107	chr6	21445549	21445849	154593	-1.24	4.52E-02	3.14E-01	Intron (NM_021587/59107, intron 4 of 33)
Plcb4	25031	chr3	128399004	128399304	-202026	1.13	4.52E-02	3.14E-01	Intron (NM_001077641/24654, intron 32 of 32)
Eml1	362783	chr6	132416281	132416581	48939	-1.36	4.52E-02	3.14E-01	Intron (NM_001025741/362783, intron 4 of 24)
Atp5mf	690441	chr12	11192015	11192315	216	0.59	4.52E-02	3.14E-01	Promoter (<=1kb)
Dguok	297389	chr4	115156953	115157253	50808	1.09	4.52E-02	3.14E-01	Distal Intergenic
Slc28a3	140944	chr17	6430843	6431143	-27966	0.83	4.53E-02	3.14E-01	Distal Intergenic
LOC102548399	102548399	chr6	136469100	136469400	-24358	0.89	4.53E-02	3.14E-01	Distal Intergenic
Arl11	364396	chr15	41627292	41627592	-15949	-1.15	4.53E-02	3.14E-01	Distal Intergenic
Nipsnap2	498174	chr12	30559744	30560044	5971	-1.14	4.53E-02	3.14E-01	Intron (NM_001017486/498174, intron 1 of 10)
RT1-T24-1	361787	chr20	3269413	3269713	98	-1.14	4.53E-02	3.14E-01	Promoter (<=1kb)
Rbpj	679028	chr14	59736035	59736335	-585	0.52	4.53E-02	3.14E-01	Promoter (<=1kb)
Anks6	362515	chr5	62683907	62684207	180	1.18	4.53E-02	3.14E-01	Promoter (<=1kb)
Eloc	64525	chr5	2043280	2043580	-3	0.6	4.53E-02	3.14E-01	Promoter (<=1kb)
Oxr1	117520	chr7	80713409	80713709	36	1.05	4.53E-02	3.14E-01	Promoter (<=1kb)
Pmpcb	64198	chr4	9921327	9921627	0	0.65	4.53E-02	3.14E-01	Promoter (<=1kb)
Cpd	25306	chr10	63326032	63326332	268	0.93	4.54E-02	3.14E-01	Promoter (<=1kb)
Dok7	305448	chr14	80958351	80958651	0	1.1	4.54E-02	3.14E-01	Promoter (<=1kb)
Arhgap18	293947	chr1	19375821	19376121	180	0.58	4.54E-02	3.14E-01	Promoter (<=1kb)
Phc3	310258	chr2	116022638	116022938	-9517	-0.74	4.54E-02	3.14E-01	Distal Intergenic
Hpcal1	50871	chr6	43158975	43159275	157027	1.18	4.54E-02	3.15E-01	Distal Intergenic
Rassf8	312846	chr4	180059358	180059658	-3141	0.72	4.54E-02	3.15E-01	Distal Intergenic
Marchf8	312656	chr4	148286869	148287169	15	1.13	4.54E-02	3.15E-01	Promoter (<=1kb)
Ptpra	25167	chr3	122976245	122976545	141	0.89	4.54E-02	3.15E-01	Promoter (<=1kb)
Sox4	364712	chr17	37311426	37311726	-303296	1.2	4.54E-02	3.15E-01	Distal Intergenic
Mex3c	307271	chr18	69549990	69550290	53	0.76	4.54E-02	3.15E-01	Promoter (<=1kb)
Phospho2	295663	chr3	56030840	56031140	0	0.51	4.54E-02	3.15E-01	Promoter (<=1kb)
Myo10	310178	chr2	77803044	77803344	-65088	-0.97	4.54E-02	3.15E-01	Distal Intergenic
Irf3	292892	chr1	100992583	100992883	178	0.55	4.54E-02	3.15E-01	Promoter (<=1kb)
Rad23a	361381	chr19	25954958	25955258	113	0.73	4.55E-02	3.15E-01	Promoter (<=1kb)
Ryk	140585	chr8	111358450	111358750	32096	-1.36	4.55E-02	3.15E-01	Intron (NM_080402/140585, intron 2 of 14)
Ptges2	311865	chr3	11433673	11433973	9478	-1.31	4.55E-02	3.15E-01	Distal Intergenic
Cdh13	192248	chr19	50850903	50851203	2110	-1.12	4.55E-02	3.15E-01	Promoter (2-3kb)
Lefty1	498299	chr13	99212723	99213023	-1366	-0.94	4.55E-02	3.15E-01	Promoter (1-2kb)
Cfi	79126	chr2	235262572	235262872	-1426	0.7	4.55E-02	3.15E-01	Promoter (1-2kb)
Nod1	500133	chr4	85152818	85153118	21813	-1.16	4.55E-02	3.15E-01	Intron (NM_001109236/500133, intron 1 of 11)
Cnot8	363603	chr10	43455586	43455886	-82424	-1.72	4.55E-02	3.15E-01	Distal Intergenic
Osr1	298878	chr6	35067990	35068290	-250811	-1.42	4.55E-02	3.15E-01	Distal Intergenic
Gnl1	309593	chr20	3299302	3299602	0	1.03	4.55E-02	3.15E-01	Promoter (<=1kb)
Gpcpd1	362219	chr3	125213077	125213377	221	0.7	4.55E-02	3.15E-01	Promoter (<=1kb)
Cast	25403	chr2	1778666	1778966	-217202	2.01	4.55E-02	3.15E-01	Distal Intergenic
Fam53b	309060	chr1	204812821	204813121	-7083	-1.23	4.55E-02	3.15E-01	Distal Intergenic
Fbln5	29158	chr6	125723645	125723945	12	0.52	4.55E-02	3.15E-01	Promoter (<=1kb)
Foxj2	502886	chr4	155653563	155653863	0	0.84	4.56E-02	3.15E-01	Promoter (<=1kb)
Ctdsp1	363249	chr9	81672926	81673226	313	0.67	4.56E-02	3.15E-01	Promoter (<=1kb)
Actn1	81634	chr6	103501531	103501831	-31034	1.41	4.56E-02	3.15E-01	Distal Intergenic
LOC690276	690276	chr6	42316797	42317097	-3882	-0.8	4.56E-02	3.15E-01	Distal Intergenic
Eif3c	293484	chr1	197988911	197989211	-6310	1.07	4.56E-02	3.15E-01	Exon (NM_001006971/293485, exon 13 of 16)
Ptprq	360417	chr7	50078711	50079011	-43779	-1.33	4.56E-02	3.15E-01	Distal Intergenic
Chac1	362196	chr3	111164142	111164442	3937	1.29	4.56E-02	3.15E-01	Distal Intergenic
Mpp7	307035	chr17	59987075	59987375	-72638	-1.03	4.57E-02	3.15E-01	Distal Intergenic
Rbis	499567	chr2	88359365	88359665	14838	-1.53	4.57E-02	3.15E-01	Distal Intergenic
Klrb1b	25192	chr4	161996988	161997288	27802	0.79	4.57E-02	3.15E-01	Distal Intergenic
Nefl	83613	chr15	44627412	44627712	-171666	0.84	4.57E-02	3.15E-01	Distal Intergenic
Tmc1	361739	chr1	238662760	238663060	-136968	1.17	4.57E-02	3.15E-01	Distal Intergenic
Rp9	363032	chr8	23416507	23416807	66132	0.57	4.57E-02	3.15E-01	Distal Intergenic
Taf6	288533	chr12	19328257	19328557	80	0.58	4.57E-02	3.15E-01	Promoter (<=1kb)
Calm1	24242	chr6	124313473	124313773	96232	1.15	4.57E-02	3.15E-01	Downstream (2-3kb)
Fcsk	307848	chr19	43323782	43324082	-14089	0.8	4.57E-02	3.15E-01	Distal Intergenic
Rin2	311494	chr3	139850176	139850476	-43855	1.55	4.57E-02	3.15E-01	Distal Intergenic
Spg7	353231	chr19	55798429	55798729	-81832	-1.24	4.57E-02	3.15E-01	Distal Intergenic
Etaa1	498420	chr14	102437348	102437648	-143671	1.6	4.57E-02	3.15E-01	Distal Intergenic
R3hdm1	304763	chr13	44842057	44842357	29490	-1.29	4.57E-02	3.15E-01	Intron (NM_001134867/304763, intron 1 of 26)
Ccdc65	362994	chr7	140387258	140387558	3797	1.59	4.57E-02	3.15E-01	Intron (NM_001014203/362994, intron 2 of 7)
Steap1	297738	chr4	25435841	25436141	0	0.54	4.57E-02	3.15E-01	Promoter (<=1kb)
Ccdc82	300359	chr8	11948575	11948875	60055	-1.19	4.57E-02	3.15E-01	Distal Intergenic
Fndc3b	294925	chr2	113368891	113369191	-23340	-0.91	4.57E-02	3.15E-01	Distal Intergenic
Ripk1	306886	chr17	31569561	31569861	43	0.55	4.57E-02	3.15E-01	Promoter (<=1kb)
Tacc2	309025	chr1	201209495	201209795	68442	0.53	4.57E-02	3.15E-01	Intron (NM_001004415/309025, intron 4 of 21)
Ccdc148	311051	chr3	45214947	45215247	-4473	1.65	4.57E-02	3.15E-01	Distal Intergenic
Hs6st1	316325	chr9	42337241	42337541	-282465	0.86	4.57E-02	3.15E-01	Distal Intergenic
Cmip	292051	chr19	49852915	49853215	60642	-1.06	4.57E-02	3.15E-01	Intron (NM_001163273/292051, intron 1 of 20)
Utrn	25600	chr1	7015555	7015855	-45515	-1.34	4.57E-02	3.15E-01	Distal Intergenic
Cox4i2	84683	chr3	148269191	148269491	34645	-1.16	4.57E-02	3.15E-01	Intron (NM_001033671/24888, intron 1 of 1)
Spc25	295661	chr3	55456642	55456942	-4844	1.56	4.58E-02	3.15E-01	Distal Intergenic
B4galt5	362275	chr3	164081291	164081591	-25730	1.17	4.58E-02	3.15E-01	Distal Intergenic
Zc3h15	362154	chr3	71020616	71020916	82	0.76	4.58E-02	3.15E-01	Promoter (<=1kb)
Herc2	308669	chr1	114456656	114456956	3381	1.67	4.58E-02	3.15E-01	Intron (NM_001107520/308669, intron 2 of 91)
Afap1	140935	chr14	79967990	79968290	19625	1.67	4.58E-02	3.15E-01	Intron (NM_080900/140935, intron 6 of 16)
Pltp	296371	chr3	161316378	161316678	5611	1.64	4.58E-02	3.15E-01	Exon (NM_001168543/296371, exon 8 of 16)
Dhrs3	313689	chr5	162912661	162912961	103571	1.64	4.58E-02	3.15E-01	Intron (NM_001108006/313825, intron 66 of 69)
Ccdc8	494320	chr1	78933451	78933751	79	0.68	4.58E-02	3.15E-01	Promoter (<=1kb)
Zfp52	361487	chr1	61724470	61724770	325976	0.59	4.58E-02	3.15E-01	Distal Intergenic
Tango2	360738	chr11	86797505	86797805	0	0.53	4.58E-02	3.15E-01	Promoter (<=1kb)
Ypel2	360590	chr10	74298241	74298541	58	0.75	4.58E-02	3.15E-01	Promoter (<=1kb)

Ets2	304063	chr11	36115219	36115519	39510	-1.35	4.58E-02	3.15E-01	Distal Intergenic
Wls	362065	chr2	266315244	266315544	202	0.56	4.58E-02	3.15E-01	Promoter (<=1kb)
Neurl1	309459	chr1	266952818	266953118	-21	1.63	4.58E-02	3.15E-01	Promoter (<=1kb)
Sh3glb1	292156	chr2	250736013	250736313	7883	-1.29	4.58E-02	3.15E-01	Intron (NM_001011929/292156, intron 2 of 8)
Sestd1	295678	chr3	64176736	64177036	-63404	1.86	4.58E-02	3.15E-01	Distal Intergenic
Ube2r2	689226	chr5	57472019	57472319	0	1.15	4.59E-02	3.15E-01	Promoter (<=1kb)
Polg	85472	chr1	141188460	141188760	-627	0.78	4.59E-02	3.15E-01	Promoter (<=1kb)
Cars	293638	chr1	216810475	216810775	-8831	1.51	4.59E-02	3.15E-01	Intron (NM_001108511/361685, intron 7 of 7)
Tmbim7	362319	chr4	27647060	27647360	-8384	1.04	4.59E-02	3.15E-01	Distal Intergenic
Lingo3	690755	chr7	11737120	11737420	0	1.12	4.59E-02	3.15E-01	Promoter (<=1kb)
Tenm3	306451	chr16	46658592	46658892	234723	-1.16	4.59E-02	3.15E-01	Intron (NM_001169133/306451, intron 2 of 28)
Inpp5b	362590	chr5	142731946	142732246	179	0.5	4.59E-02	3.15E-01	Promoter (<=1kb)
Ap3d1	314633	chr7	11838610	11838910	0	0.85	4.59E-02	3.15E-01	Promoter (<=1kb)
Tspyl1	29544	chr20	41083363	41083663	46	0.65	4.59E-02	3.15E-01	Promoter (<=1kb)
Slc26a2	117267	chr18	56505462	56505762	28653	0.63	4.59E-02	3.15E-01	Distal Intergenic
Myom2	306616	chr16	79623740	79624040	47679	1.61	4.60E-02	3.15E-01	Intron (NM_001169141/306616, intron 13 of 36)
Cnot4	312227	chr4	62647919	62648219	14981	1.94	4.60E-02	3.15E-01	Intron (NM_001037782/312227, intron 1 of 11)
Psma5	29672	chr2	211050446	211050746	102	0.66	4.60E-02	3.15E-01	Promoter (<=1kb)
Hdgfl3	252941	chr1	143751525	143751825	0	1.01	4.60E-02	3.15E-01	Promoter (<=1kb)
Vom2r67	689485	chr14	1339612	1339912	21551	0.68	4.60E-02	3.15E-01	Distal Intergenic
Pex14	64460	chr5	165724246	165724546	193899	0.79	4.60E-02	3.15E-01	Distal Intergenic
Zcrb1	362990	chr7	134601927	134602227	12	1.08	4.60E-02	3.15E-01	Promoter (<=1kb)
Tdg	114521	chr7	27213353	27213653	23	0.98	4.60E-02	3.15E-01	Promoter (<=1kb)
Stk25	373542	chr9	100779295	100779595	120	0.69	4.60E-02	3.15E-01	Promoter (<=1kb)
Bmp2	29373	chr3	126460551	126460851	124366	1.75	4.60E-02	3.15E-01	Distal Intergenic
Tnc	116640	chr5	79797590	79797890	51975	1.56	4.60E-02	3.15E-01	Intron (NM_053861/116640, intron 19 of 24)
Noct	310395	chr2	140218171	140218471	-68190	-1.1	4.60E-02	3.15E-01	Distal Intergenic
Tagln3	63837	chr11	57536987	57537287	31303	1.64	4.60E-02	3.15E-01	Distal Intergenic
Slc2a6	296600	chr3	5578796	5579096	-3660	-1.16	4.61E-02	3.16E-01	Distal Intergenic
Lrp12	314941	chr7	78958860	78959160	-28028	-1.39	4.61E-02	3.16E-01	Distal Intergenic
Ak1	24183	chr3	11649180	11649480	-2663	0.67	4.61E-02	3.16E-01	Promoter (2-3kb)
Gmds	291095	chr17	33665508	33665808	256786	-1.32	4.61E-02	3.16E-01	Intron (NM_001039606/291095, intron 7 of 10)
Nfib	29227	chr5	100313379	100313679	333619	-1.37	4.61E-02	3.16E-01	Distal Intergenic
Uqcr11	690848	chr7	12171263	12171563	78	0.55	4.61E-02	3.16E-01	Promoter (<=1kb)
Nab1	64824	chr9	53906190	53906490	117	1.17	4.61E-02	3.16E-01	Promoter (<=1kb)
Cln6	315746	chr8	67733185	67733485	0	0.87	4.61E-02	3.16E-01	Promoter (<=1kb)
Neu3	117185	chr1	164827831	164828131	-13180	1.6	4.61E-02	3.16E-01	Downstream (1-2kb)
Ncam1	24586	chr8	53986017	53986317	148414	1.03	4.61E-02	3.16E-01	Intron (NM_031521/24586, intron 1 of 18)
Il15	25670	chr19	23608755	23609055	-54161	0.67	4.61E-02	3.16E-01	Distal Intergenic
Hacd4	362540	chr5	106497450	106497750	260282	1.79	4.61E-02	3.16E-01	Distal Intergenic
Tti1	499935	chr3	154351794	154352094	138761	1.37	4.61E-02	3.16E-01	Intron (NM_001024870/296320, intron 12 of 15)
Col6a2	361821	chr20	12783172	12783472	9652	1.8	4.62E-02	3.16E-01	Exon (NM_001100741/361821, exon 5 of 28)
Tsku	308843	chr1	163256235	163256535	72056	-1.19	4.62E-02	3.16E-01	Distal Intergenic
Trim47	690374	chr10	104658537	104658837	351	1.13	4.62E-02	3.16E-01	Promoter (<=1kb)
Lrp11	292462	chr1	1674221	1674521	-28175	-1.32	4.62E-02	3.16E-01	Distal Intergenic
Il7	25647	chr2	96434530	96434830	3762	2.08	4.62E-02	3.16E-01	Intron (NM_013110/25647, intron 1 of 4)
Gyg1	81675	chr2	104942350	104942650	13157	-1.33	4.62E-02	3.16E-01	Intron (NM_031043/81675, intron 3 of 5)
Dmrt2	309430	chr1	244069146	244069446	406323	1.83	4.62E-02	3.16E-01	Distal Intergenic
Mrpl45	287656	chr10	85355631	85355931	97755	0.57	4.62E-02	3.16E-01	Distal Intergenic
Notch2	29492	chr2	200195440	200195740	8256	-1.24	4.62E-02	3.16E-01	Intron (NM_024358/29492, intron 1 of 33)
Rps28	691531	chr7	18676308	18676608	6832	0.68	4.62E-02	3.16E-01	Exon (NM_001108989/366848, exon 3 of 11)
Cdh15	361432	chr19	55597816	55598116	-71545	-1.41	4.62E-02	3.16E-01	Distal Intergenic
Niban2	362115	chr3	11962885	11963185	41170	-0.92	4.62E-02	3.16E-01	Intron (NM_001109885/362115, intron 5 of 13)
Exoc6	50556	chr1	256254065	256254365	27881	1.04	4.63E-02	3.16E-01	Intron (NM_019277/50556, intron 1 of 21)
Sgsm2	303304	chr10	61745012	61745312	0	0.91	4.63E-02	3.16E-01	Promoter (<=1kb)
Ak2	24184	chr5	147185564	147185864	90	0.54	4.63E-02	3.16E-01	Promoter (<=1kb)
RGD1309106	360864	chr13	79895949	79896249	3230	0.67	4.63E-02	3.16E-01	Intron (NM_001014130/360864, intron 1 of 7)
Rnf38	171501	chr5	59646869	59647169	-8484	0.7	4.63E-02	3.16E-01	Distal Intergenic
Prepl	298771	chr6	8327156	8327456	18741	-1.32	4.63E-02	3.16E-01	Intron (NM_001010951/298771, intron 7 of 13)
Rnase12	364302	chr15	27988857	27989157	-7194	0.51	4.64E-02	3.16E-01	Distal Intergenic
Desi2	289277	chr13	96112122	96112422	322	0.67	4.64E-02	3.16E-01	Promoter (<=1kb)
Csf3	25610	chr10	86620024	86620324	3239	-1.38	4.64E-02	3.16E-01	3' UTR
Cebpg	25301	chr1	91324345	91324645	-27678	-0.74	4.64E-02	3.16E-01	Distal Intergenic
Bcl6	303836	chr11	80184566	80184866	-70924	1.28	4.64E-02	3.16E-01	Distal Intergenic
LOC681419	681419	chr5	147515974	147516274	-6391	-0.89	4.64E-02	3.16E-01	Intron (NM_001107912/313048, intron 2 of 11)
Ppm1j	295341	chr2	207239717	207240017	-23570	0.67	4.64E-02	3.16E-01	Distal Intergenic
RGD1359290	360649	chr10	95604172	95604472	-30234	-1.02	4.64E-02	3.16E-01	Distal Intergenic
Hmgcs1	29637	chr2	52439210	52439510	11631	-1.31	4.64E-02	3.16E-01	Exon (NM_017268/29637, exon 6 of 11)
Atg7	312647	chr4	146554967	146555267	-43149	1.59	4.64E-02	3.16E-01	Distal Intergenic
Ctsm	306720	chr17	3769779	3770079	-50527	0.57	4.65E-02	3.17E-01	Distal Intergenic
Cbarp	314622	chr7	12436482	12436782	3060	1.65	4.65E-02	3.17E-01	5' UTR
Trim3	83616	chr1	170463899	170464199	142	0.83	4.65E-02	3.17E-01	Promoter (<=1kb)
Pgd	100360180	chr5	165970838	165971138	11189	-1.3	4.65E-02	3.17E-01	Intron (NM_001305435/100360180, intron 8 of 12)
Epdr1	291180	chr17	47518816	47519116	121258	-1.24	4.65E-02	3.17E-01	Distal Intergenic
Spry2	306141	chr15	89854853	89855153	320649	-1.11	4.65E-02	3.17E-01	Distal Intergenic
Fam219a	691024	chr5	57947018	57947318	148	0.87	4.65E-02	3.17E-01	Promoter (<=1kb)
Atg5	365601	chr20	49301821	49302121	38	0.58	4.65E-02	3.17E-01	Promoter (<=1kb)
Acsl4	113976	chrX	113641106	113641406	18472	-1.23	4.65E-02	3.17E-01	Intron (NM_053623/113976, intron 1 of 15)
Tnfrsf26	361685	chr1	216816000	216816300	12281	1.57	4.66E-02	3.17E-01	Intron (NM_001108511/361685, intron 4 of 7)
Mpp7	307035	chr17	60126926	60127226	66913	1.54	4.66E-02	3.17E-01	Intron (NM_001100575/307035, intron 2 of 15)
Fam53b	309060	chr1	204728039	204728339	77399	-1.23	4.66E-02	3.17E-01	Intron (NM_001107556/309060, intron 4 of 4)
Kmt2e	311968	chr4	8235674	8235974	19604	-1.27	4.66E-02	3.17E-01	Intron (NM_001100851/311968, intron 2 of 26)
Mn1	498194	chr12	51058780	51059080	191150	1.39	4.66E-02	3.17E-01	Distal Intergenic
Mir29b1	100314008	chr4	58499369	58499669	-154979	-1.08	4.66E-02	3.17E-01	Distal Intergenic
Fos	314322	chr6	109300605	109300905	172	0.55	4.66E-02	3.17E-01	Promoter (<=1kb)
Kctd3	305055	chr13	107471410	107471710	133	0.79	4.66E-02	3.17E-01	Promoter (<=1kb)
Cd47	29364	chr11	53519740	53520040	55135	0.86	4.66E-02	3.17E-01	Intron (NM_019195/29364, intron 7 of 8)
Arhgap31	288093	chr11	64658708	64659008	57740	1.41	4.66E-02	3.17E-01	Intron (NM_001105879/288093, intron 1 of 11)
Amph	60668	chr17	48562620	48562920	0	0.68	4.66E-02	3.17E-01	Promoter (<=1kb)
Cntn6	27256	chr4	136560485	136560785	48284	1.17	4.66E-02	3.17E-01	Intron (NM_013225/27256, intron 1 of 23)
Cdc42se1	499672	chr2	196402821	196403121	0	0.72	4.66E-02	3.17E-01	Promoter (<=1kb)
Smurf1	690516	chr12	11380135	11380435	-53380	-1.16	4.66E-02	3.17E-01	Intron (NM_153312/266682, intron 8 of 15)
Ccdc134	500909	chr7	123362545	123362845	-1467	0.6	4.66E-02	3.17E-01	Promoter (1-2kb)
Abr	287537	chr10	64642428	64642728	14351	0.61	4.66E-02	3.17E-01	Intron (NM_001105814/287537, intron 1 of 21)

RSA-14-44	297173	chr4	96659096	96659396	-172484	0.67	4.66E-02	3.17E-01	Distal Intergenic
Eogt	494219	chr4	129514534	129514834	601	0.67	4.66E-02	3.17E-01	Promoter (<=1kb)
Mapk1ip1l	361028	chr15	24078382	24078682	102	0.72	4.66E-02	3.17E-01	Promoter (<=1kb)
Ndufaf8	690871	chr10	109372989	109373289	94277	0.53	4.66E-02	3.17E-01	Distal Intergenic
Slc4a7	117955	chr15	11955984	11956284	-43068	-1.21	4.67E-02	3.17E-01	Distal Intergenic
Piwil4	689972	chr8	13137025	13137325	114487	0.75	4.67E-02	3.17E-01	Distal Intergenic
Bgn	25181	chrX	157323636	157323936	7268	-0.93	4.67E-02	3.17E-01	5' UTR
Fam53b	309060	chr1	204777436	204777736	28002	1.36	4.67E-02	3.17E-01	Intron (NM_001107556/309060, intron 2 of 4)
Uba52	64156	chr16	20669048	20669348	77	0.85	4.67E-02	3.17E-01	Promoter (<=1kb)
Trnau1ap	65241	chr5	150506631	150506931	0	0.61	4.67E-02	3.17E-01	Promoter (<=1kb)
Qser1	311266	chr3	94767034	94767334	0	0.84	4.67E-02	3.17E-01	Promoter (<=1kb)
Zc3h12d	308266	chr1	1942140	1942440	3721	-1.1	4.67E-02	3.17E-01	5' UTR
Map3k7	313121	chr9	114247247	114247547	225212	-1.4	4.67E-02	3.17E-01	Distal Intergenic
Neil3	290729	chr16	41083121	41083421	3677	-1.56	4.68E-02	3.17E-01	Intron (NM_001170346/290729, intron 1 of 10)
Hs2st1	292155	chr2	250556744	250557044	43473	-1.56	4.68E-02	3.17E-01	Intron (NM_001100518/292155, intron 1 of 6)
Actr3	81732	chr13	41727720	41728020	10602	1.6	4.68E-02	3.17E-01	Intron (NM_001177819/100362110, intron 2 of 8)
Tanc1	311055	chr3	45833881	45834181	115300	1.52	4.68E-02	3.17E-01	Intron (NM_001002854/311055, intron 4 of 25)
Mitf	25094	chr4	130189985	130190285	17129	-0.99	4.68E-02	3.17E-01	Intron (NM_001191089/25094, intron 1 of 9)
Cmss1	288176	chr11	44982082	44982382	-49315	1.59	4.68E-02	3.17E-01	Intron (NM_001107100/304021, intron 2 of 3)
Slc30a5	294698	chr2	30845412	30845712	84	0.78	4.68E-02	3.17E-01	Promoter (<=1kb)
LOC100909416	100909416	chr8	79413361	79413661	-5742	-1.03	4.68E-02	3.17E-01	Distal Intergenic
Hs6st1	316325	chr9	42641113	42641413	21107	-0.86	4.68E-02	3.17E-01	Intron (NM_001108210/316325, intron 1 of 1)
Tcf4	84382	chr18	65240938	65241238	-44082	-1.14	4.68E-02	3.17E-01	Distal Intergenic
Rhbdf2	303690	chr10	105592710	105593010	7875	-0.88	4.69E-02	3.18E-01	Intron (NM_001107067/303690, intron 1 of 18)
Ctbs	81652	chr2	252329430	252329730	23556	-1.17	4.69E-02	3.18E-01	Intron (NM_001014177/362056, intron 10 of 14)
Rap1gap2	303298	chr10	61238800	61239100	-6469	-1.55	4.69E-02	3.18E-01	Distal Intergenic
Thbs2	292406	chr1	56534617	56534917	148654	-1.2	4.69E-02	3.18E-01	Distal Intergenic
Ttc30a1	311123	chr3	62802775	62803075	298	1.24	4.69E-02	3.18E-01	Promoter (<=1kb)
Rcbtb1	361050	chr15	39622545	39622845	0	0.91	4.69E-02	3.18E-01	Promoter (<=1kb)
Pdgfc	79429	chr2	179951593	179951893	-334	0.67	4.69E-02	3.18E-01	Promoter (<=1kb)
Mid1ip1	404280	chrX	13115585	13115885	858	0.71	4.69E-02	3.18E-01	Promoter (<=1kb)
Ephb3	287989	chr11	83500507	83500807	45867	1.22	4.69E-02	3.18E-01	Distal Intergenic
Spata4	306441	chr16	39970290	39970590	0	1.22	4.70E-02	3.18E-01	Promoter (<=1kb)
Eml4	313861	chr6	6559583	6559883	-59193	0.92	4.70E-02	3.18E-01	Distal Intergenic
Lrch1	502020	chr15	56950919	56951219	19146	1.58	4.70E-02	3.18E-01	Intron (NM_001134727/502020, intron 1 of 18)
Epha7	171287	chr5	43621553	43621853	18650	-1.31	4.70E-02	3.18E-01	Intron (NM_134331/171287, intron 3 of 16)
Pcbp4	363133	chr8	115155435	115155735	0	0.67	4.70E-02	3.18E-01	Promoter (<=1kb)
Dbnl	83527	chr14	86036760	86037060	7425	-1.05	4.70E-02	3.18E-01	Intron (NM_001277211/83527, intron 4 of 12)
Got1	24401	chr1	263258077	263258377	11385	-0.89	4.70E-02	3.18E-01	Intron (NM_012571/24401, intron 2 of 8)
Odf2l	685425	chr2	250998503	250998803	2986	-1.31	4.70E-02	3.18E-01	Promoter (2-3kb)
LOC361646	361646	chr1	197137619	197137919	9208	1.72	4.70E-02	3.18E-01	Intron (NM_001134574/361646, intron 1 of 27)
Uvrag	308846	chr1	164042718	164043018	58550	-1.04	4.70E-02	3.18E-01	Intron (NM_001107536/308846, intron 5 of 13)
Prkd1	85421	chr6	71002446	71002746	346503	-1.21	4.70E-02	3.18E-01	Distal Intergenic
Ctbp2	81717	chr1	205194055	205194355	-163490	1.2	4.70E-02	3.18E-01	Distal Intergenic
LOC102550367	102550367	chr3	121012559	121012859	70377	-1.28	4.70E-02	3.18E-01	Intron (NR_110721/102550367, intron 2 of 4)
Vom2r1	678740	chr10	112544264	112544564	-977590	1.82	4.70E-02	3.18E-01	Distal Intergenic
Papola	314417	chr6	129609236	129609536	162	0.78	4.70E-02	3.18E-01	Promoter (<=1kb)
Ank1	306570	chr16	73885618	73885918	-58130	-1.17	4.70E-02	3.18E-01	Distal Intergenic
Cdc42se2	691031	chr10	40114429	40114729	8186	-1.34	4.71E-02	3.18E-01	Intron (NM_001126089/691031, intron 1 of 5)
Daglb	304289	chr12	13159713	13160013	-2061	1.44	4.71E-02	3.18E-01	Promoter (2-3kb)
Ngf	310738	chr2	204881261	204881561	-4641	1.62	4.71E-02	3.18E-01	Distal Intergenic
Mtmr12	310155	chr2	62236671	62236971	81	0.86	4.71E-02	3.18E-01	Promoter (<=1kb)
Sbds	288615	chr12	29921443	29921743	0	0.56	4.71E-02	3.18E-01	Promoter (<=1kb)
Rnf166	365022	chr19	55300074	55300374	29	1.23	4.71E-02	3.18E-01	Promoter (<=1kb)
Tead1	361630	chr1	177496000	177496300	218	0.77	4.71E-02	3.18E-01	Promoter (<=1kb)
Nup93	291874	chr19	11263691	11263991	0	0.55	4.72E-02	3.18E-01	Promoter (<=1kb)
Mcmdc2	500392	chr5	9115036	9115336	-76442	0.73	4.72E-02	3.18E-01	Distal Intergenic
Slc35f2	300713	chr8	58347988	58348288	231	0.7	4.72E-02	3.18E-01	Promoter (<=1kb)
Bud13	300687	chr8	50573081	50573381	55	0.54	4.72E-02	3.18E-01	Promoter (<=1kb)
Dusp4	60587	chr16	61084476	61084776	6197	0.97	4.72E-02	3.18E-01	Intron (NM_022199/60587, intron 1 of 3)
Lrrn3	81514	chr6	61405008	61405308	0	-1.49	4.72E-02	3.18E-01	Promoter (<=1kb)
Slc25a37	306000	chr15	51167747	51168047	337	0.58	4.72E-02	3.18E-01	Promoter (<=1kb)
Alx3	365900	chr2	210364361	210364661	-11849	-1.26	4.72E-02	3.18E-01	Distal Intergenic
Dgkz	81821	chr3	80860269	80860569	13318	1.47	4.72E-02	3.18E-01	Intron (NM_031143/81821, intron 1 of 30)
Cc2d1b	313478	chr5	128219628	128219928	3917	-1.56	4.72E-02	3.18E-01	Exon (NM_001270984/313478, exon 3 of 25)
Col5a2	85250	chr9	52226164	52226464	12271	-1.29	4.72E-02	3.18E-01	Intron (NM_053488/85250, intron 1 of 55)
Ndrg2	171114	chr15	28314154	28314454	5	0.62	4.72E-02	3.18E-01	Promoter (<=1kb)
Sike1	362007	chr2	205503602	205503902	15420	1.23	4.72E-02	3.18E-01	Distal Intergenic
Nsf	60355	chr10	92007702	92008002	50	0.66	4.72E-02	3.18E-01	Promoter (<=1kb)
Tmem212	499586	chr2	113603965	113604265	12501	1.2	4.72E-02	3.18E-01	Intron (NM_001164439/499586, intron 3 of 3)
Tlr2	310553	chr2	183030785	183031085	-184724	0.8	4.72E-02	3.18E-01	Distal Intergenic
Tanc1	311055	chr3	45684101	45684401	-34180	0.74	4.73E-02	3.18E-01	Distal Intergenic
Ptf1a	117034	chr17	86378683	86378983	179060	-1.31	4.73E-02	3.18E-01	Distal Intergenic
Fgd6	500824	chr7	34952090	34952390	212	0.63	4.73E-02	3.18E-01	Promoter (<=1kb)
Taf9b	171152	chrX	77293068	77293368	13	0.65	4.73E-02	3.18E-01	Promoter (<=1kb)
Capza2	493810	chr4	44856421	44856721	-79569	1.31	4.73E-02	3.18E-01	Distal Intergenic
Aagab	171435	chr8	68526096	68526396	1	0.58	4.73E-02	3.18E-01	Promoter (<=1kb)
Dlc1	58834	chr16	59152913	59153213	-44154	-1.3	4.73E-02	3.18E-01	Distal Intergenic
Txndc15	307180	chr17	9463587	9463887	-22061	1.07	4.73E-02	3.18E-01	Distal Intergenic
Nlgn1	116647	chr2	111377470	111377770	415192	-1.13	4.73E-02	3.18E-01	Intron (NM_053868/116647, intron 4 of 6)
Clic4	83718	chr5	153614151	153614451	11218	-1.07	4.73E-02	3.18E-01	Intron (NM_031818/83718, intron 1 of 5)
Olr996	404924	chr7	15927788	15928088	82486	1.66	4.73E-02	3.18E-01	Distal Intergenic
Ankib1	368062	chr4	27473431	27473731	0	0.86	4.73E-02	3.18E-01	Promoter (<=1kb)
Tti1	499935	chr3	154353144	154353444	137411	1.63	4.73E-02	3.18E-01	Exon (NM_001024870/296320, exon 13 of 16)
Cops8	363283	chr9	97916489	97916789	144265	-1.25	4.73E-02	3.18E-01	Distal Intergenic
Pak4	292756	chr1	85183117	85183417	20655	0.83	4.74E-02	3.18E-01	Intron (NM_001106238/292756, intron 1 of 9)
Tpcn1	246215	chr12	41525808	41526108	18024	-1.43	4.74E-02	3.18E-01	Intron (NM_139332/246215, intron 2 of 29)
Ldlrad4	679578	chr18	63841146	63841446	-2553	-1.4	4.74E-02	3.18E-01	Promoter (2-3kb)
Hpf1	290706	chr16	32539987	32540287	0	0.7	4.74E-02	3.19E-01	Promoter (<=1kb)
Tra2a	500116	chr4	78842774	78843074	81107	-1.27	4.74E-02	3.19E-01	Distal Intergenic
Arid3a	314616	chr7	12601048	12601348	-2678	0.56	4.74E-02	3.19E-01	Promoter (2-3kb)
Fndc3b	294925	chr2	113319403	113319703	25848	-0.99	4.74E-02	3.19E-01	Intron (NM_001191704/294925, intron 2 of 24)
Ehbp1	305556	chr14	107016351	107016651	108731	1.02	4.74E-02	3.19E-01	Intron (NM_001305130/305556, intron 7 of 24)
Zcchc17	500555	chr5	148559279	148559579	17713	-1.08	4.74E-02	3.19E-01	Intron (NM_001109267/500555, intron 3 of 8)

Rasa2	25597	chr8	104541981	104542281	32	1.63	4.74E-02	3.19E-01	Promoter (<=1kb)
Hibadh	63938	chr4	82736784	82737084	-34355	-1.37	4.74E-02	3.19E-01	Distal Intergenic
Psap	25524	chr20	29814446	29814746	-16568	0.56	4.74E-02	3.19E-01	Distal Intergenic
Crbn	297498	chr4	138885259	138885559	0	0.66	4.74E-02	3.19E-01	Promoter (<=1kb)
Dtnbp1	641528	chr17	20285970	20286270	195834	-0.96	4.75E-02	3.19E-01	Distal Intergenic
Ephb3	287989	chr11	83546091	83546391	283	1.1	4.75E-02	3.19E-01	Promoter (<=1kb)
Med1	497991	chr10	86144745	86145045	0	0.59	4.75E-02	3.19E-01	Promoter (<=1kb)
Ikzf2	301476	chr9	76770386	76770686	-1580	0.83	4.75E-02	3.19E-01	Promoter (1-2kb)
Lrrc69	500415	chr5	28298097	28298397	19546	-1.33	4.75E-02	3.19E-01	Intron (NM_001134624/500415, intron 1 of 7)
Cox8c	360229	chr6	126747872	126748172	-18795	1.25	4.75E-02	3.19E-01	Distal Intergenic
Cahm	106182183	chr1	50598670	50598970	228425	-1.18	4.75E-02	3.19E-01	Distal Intergenic
Rp9	363032	chr8	23399771	23400071	82868	1.68	4.75E-02	3.19E-01	Distal Intergenic
Pafah1b1	83572	chr10	61577157	61577457	0	0.64	4.75E-02	3.19E-01	Promoter (<=1kb)
Cyth3	116693	chr12	12949724	12950024	-41952	-1.26	4.75E-02	3.19E-01	Distal Intergenic
Spsb1	313722	chr5	166971837	166972137	-45201	-1.04	4.75E-02	3.19E-01	Distal Intergenic
Rab30	308821	chr1	157612526	157612826	-33347	1.68	4.75E-02	3.19E-01	Distal Intergenic
Rnf19b	313806	chr5	147246178	147246478	-11200	0.78	4.75E-02	3.19E-01	Distal Intergenic
Mapk15	286997	chr7	117060209	117060509	4564	1.19	4.76E-02	3.19E-01	Exon (NM_173331/286997, exon 12 of 14)
RGD1309748	302913	chr10	6850207	6850507	19504	-1.08	4.76E-02	3.19E-01	Intron (NM_001106972/302913, intron 4 of 4)
Szrd1	500575	chr5	159662340	159662640	114	0.59	4.76E-02	3.19E-01	Promoter (<=1kb)
Ncl	25135	chr9	93389731	93390031	-12088	0.93	4.76E-02	3.19E-01	Distal Intergenic
Bcas2	295334	chr2	205783283	205783583	120650	1.4	4.76E-02	3.19E-01	Distal Intergenic
Sirt5	306840	chr17	23990253	23990553	3457	-1.37	4.76E-02	3.19E-01	Intron (NM_001004256/306840, intron 1 of 8)
Adora2b	29316	chr10	48578797	48579097	9231	-1.2	4.76E-02	3.19E-01	Intron (NM_017161/29316, intron 1 of 1)
Nemp2	503257	chr9	53794603	53794903	-61745	-1.12	4.76E-02	3.19E-01	Distal Intergenic
Clmp	286939	chr8	44861238	44861538	14081	-0.9	4.76E-02	3.19E-01	Intron (NM_173154/286939, intron 1 of 6)
Mcoln1	288371	chr12	2070173	2070473	15493	0.61	4.76E-02	3.19E-01	Distal Intergenic
Cflar	117279	chr9	65554278	65554578	19650	-1.18	4.76E-02	3.19E-01	Exon (NM_057138/117279, exon 3 of 6)
Btbd1	293060	chr1	143646894	143647194	0	1.02	4.76E-02	3.19E-01	Promoter (<=1kb)
Il1rap	25466	chr11	77288820	77289120	304006	1.23	4.76E-02	3.19E-01	Distal Intergenic
Nampt	297508	chr6	52056516	52056816	-65269	0.88	4.76E-02	3.19E-01	Distal Intergenic
Nfkb1	81736	chr2	240889487	240889787	266	0.82	4.77E-02	3.19E-01	Promoter (<=1kb)
Irs2	29376	chr16	83856920	83857220	32405	1.67	4.77E-02	3.19E-01	Distal Intergenic
Naxe	295229	chr2	187422182	187422482	3898	1.67	4.77E-02	3.19E-01	Exon (NM_001024979/295228, exon 6 of 8)
Ppp1r12a	116670	chr7	51504602	51504902	10319	-1.27	4.77E-02	3.19E-01	Intron (NM_053890/116670, intron 1 of 23)
Slc25a26	362403	chr4	126713451	126713751	191116	-1.24	4.77E-02	3.19E-01	Distal Intergenic
Cand1	117152	chr7	62376713	62377013	-214262	1.16	4.77E-02	3.19E-01	Distal Intergenic
Pdk4	89813	chr4	30514778	30515078	41696	0.89	4.77E-02	3.19E-01	Distal Intergenic
Sdc2	25615	chr7	71634537	71634837	61806	1.74	4.77E-02	3.19E-01	Intron (NM_013082/25615, intron 1 of 4)
Arf6	79121	chr6	91773200	91773500	76091	-1.3	4.77E-02	3.19E-01	Distal Intergenic
Clca2	362052	chr2	250786303	250786603	-8034	0.67	4.77E-02	3.19E-01	Intron (NM_001077356/499721, intron 13 of 13)
Trpv2	29465	chr10	48894601	48894901	-8639	-1.26	4.77E-02	3.19E-01	Distal Intergenic
Tacc1	306562	chr16	71677924	71678224	48399	1.75	4.77E-02	3.19E-01	3' UTR
Grk4	59077	chr14	81339310	81339610	0	0.67	4.77E-02	3.19E-01	Promoter (<=1kb)
Parn	360464	chr10	1460822	1461122	1	0.63	4.77E-02	3.19E-01	Promoter (<=1kb)
Grb14	58844	chr3	51251274	51251574	-196827	1.47	4.78E-02	3.19E-01	Distal Intergenic
Ppp3cc	171378	chr15	51980576	51980876	-12679	-1.11	4.78E-02	3.19E-01	Downstream (2-3kb)
Rpl22	81768	chr5	169492459	169492759	-13391	0.84	4.78E-02	3.19E-01	Distal Intergenic
LOC499584	499584	chr2	109409456	109409756	-896607	-0.98	4.78E-02	3.19E-01	Distal Intergenic
Raf1	24703	chr4	147582586	147582886	9813	-1.39	4.78E-02	3.19E-01	Intron (NM_012639/24703, intron 1 of 16)
Stt3b	363160	chr8	123370409	123370709	20	0.8	4.78E-02	3.19E-01	Promoter (<=1kb)
Flnc	362332	chr4	56711096	56711396	10	0.7	4.78E-02	3.19E-01	Promoter (<=1kb)
Foxn3	314374	chr6	123395177	123395477	182218	-1.27	4.78E-02	3.19E-01	Intron (NM_001108047/314374, intron 3 of 6)
Slc25a4	85333	chr16	49267266	49267566	363	0.74	4.78E-02	3.19E-01	Promoter (<=1kb)
Arhgap21	307178	chr17	87798200	87798500	51229	-1.31	4.78E-02	3.19E-01	Intron (NM_001191693/307178, intron 3 of 26)
Marf1	170946	chr10	1151394	1151694	242588	0.95	4.78E-02	3.19E-01	Distal Intergenic
Fem1b	315745	chr8	67708064	67708364	0	0.8	4.78E-02	3.19E-01	Promoter (<=1kb)
Ca14	791259	chr2	198027493	198027793	-10634	-1.36	4.78E-02	3.19E-01	Distal Intergenic
Cyren	500077	chr4	62395228	62395528	507	0.52	4.78E-02	3.19E-01	Promoter (<=1kb)
Gnptg	287134	chr10	14581259	14581559	16410	-1.24	4.78E-02	3.19E-01	Distal Intergenic
Mapk14	81649	chr20	5937997	5938297	4694	-1.12	4.78E-02	3.19E-01	Intron (NM_031020/81649, intron 1 of 11)
Adam10	29650	chr8	77107745	77108045	209	0.97	4.78E-02	3.19E-01	Promoter (<=1kb)
Phf20l1	314964	chr7	107409575	107409875	-162	1.38	4.79E-02	3.19E-01	Promoter (<=1kb)
Hmox2	79239	chr10	11035098	11035398	86	0.58	4.79E-02	3.19E-01	Promoter (<=1kb)
LOC499544	499544	chr2	61978149	61978449	-13293	1.23	4.79E-02	3.19E-01	Distal Intergenic
Ubqln1	114590	chr17	6840372	6840672	0	1.08	4.79E-02	3.19E-01	Promoter (<=1kb)
Bbof1	500693	chr6	108123418	108123718	-137	0.74	4.79E-02	3.19E-01	Promoter (<=1kb)
Rab15	299156	chr6	99867427	99867727	2593	-1.21	4.79E-02	3.19E-01	Promoter (2-3kb)
Habp4	361196	chr17	1726565	1726865	46948	-0.94	4.79E-02	3.19E-01	Intron (NM_001108404/361195, intron 10 of 15)
Dynlt3	363448	chrX	14641997	14642297	59	0.73	4.79E-02	3.19E-01	Promoter (<=1kb)
Nt5dc1	294456	chr20	41209039	41209339	425	0.57	4.79E-02	3.19E-01	Promoter (<=1kb)
Enpp1	85496	chr1	21780832	21781132	32596	-1.25	4.79E-02	3.19E-01	Exon (NM_053535/85496, exon 4 of 25)
Elavl1	363854	chr12	2461524	2461824	22	0.85	4.79E-02	3.19E-01	Promoter (<=1kb)
Pum3	499339	chr1	245459403	245459703	57323	1.37	4.79E-02	3.19E-01	Distal Intergenic
Yap1	363014	chr8	6203134	6203434	145	0.76	4.79E-02	3.19E-01	Promoter (<=1kb)
Cttnbp2nl	310760	chr2	207537862	207538162	3480	-1.35	4.80E-02	3.19E-01	Intron (NM_001107712/310760, intron 1 of 5)
Ptbp2	310820	chr2	223316273	223316573	5969	-1.36	4.80E-02	3.19E-01	Intron (NM_001005555/310820, intron 2 of 13)
Slc25a26	362403	chr4	126626061	126626361	103726	1.45	4.80E-02	3.19E-01	Distal Intergenic
Bag3	293524	chr1	199937562	199937862	-3396	1.61	4.80E-02	3.19E-01	Distal Intergenic
Sh3bp4	64634	chr9	96441763	96442063	231013	1.14	4.80E-02	3.19E-01	Distal Intergenic
Ints7	289382	chr13	110263237	110263537	5666	1.71	4.80E-02	3.19E-01	Intron (NM_001191675/289382, intron 1 of 19)
Ube2e3	295686	chr3	65831854	65832154	16778	1.71	4.80E-02	3.19E-01	Intron (NM_001047857/295686, intron 3 of 5)
LOC100911664	100911664	chr17	42268406	42268706	0	0.99	4.81E-02	3.19E-01	Promoter (<=1kb)
Lif	60584	chr14	84528847	84529147	32377	-0.81	4.81E-02	3.19E-01	Distal Intergenic
Rhpn1	300030	chr7	116739011	116739311	0	0.61	4.81E-02	3.19E-01	Promoter (<=1kb)
RGD1306941	316406	chr9	63919882	63920182	-146397	-1.26	4.81E-02	3.19E-01	Distal Intergenic
Arhgap35	306400	chr1	78557053	78557353	16021	-1.31	4.81E-02	3.19E-01	Intron (NM_001271132/306400, intron 1 of 6)
Cobl	305497	chr14	92641516	92641816	-145622	0.95	4.81E-02	3.19E-01	Distal Intergenic
Set	307947	chr19	58436965	58437265	16330	-1.49	4.81E-02	3.20E-01	Distal Intergenic
Myc	24577	chr7	102655564	102655864	69251	-0.88	4.81E-02	3.20E-01	Distal Intergenic
Cited4	114491	chr5	139611468	139611768	13737	1.53	4.81E-02	3.20E-01	Distal Intergenic
Mrps23	360594	chr10	75499400	75499700	-29309	0.67	4.81E-02	3.20E-01	Intron (NM_001013971/303419, intron 2 of 10)
Gucy1a2	66012	chr8	881900	882200	473899	1.08	4.81E-02	3.20E-01	Intron (NM_023956/66012, intron 7 of 7)
Zfp638	312491	chr4	115601881	115602181	220	0.66	4.82E-02	3.20E-01	Promoter (<=1kb)

Tnks	290794	chr16	60925245	60925545	152	1.06	4.82E-02	3.20E-01	Promoter (<=1kb)
Ncald	553106	chr7	76365874	76366174	-71211	-1.15	4.82E-02	3.20E-01	Distal Intergenic
Arnt	25242	chr2	196594300	196594600	0	0.57	4.82E-02	3.20E-01	Promoter (<=1kb)
Grm1	24414	chr1	5155289	5155589	10267	0.88	4.82E-02	3.20E-01	Intron (NM_001114330/24414, intron 2 of 9)
Wdr35	503018	chr6	34084390	34084690	-9616	1.64	4.82E-02	3.20E-01	Exon (NM_001108013/313954, exon 8 of 11)
Rasgrf1	192213	chr8	97243937	97244237	-33013	-1.49	4.82E-02	3.20E-01	Distal Intergenic
Piezo1	361430	chr19	55367006	55367306	47	1.02	4.82E-02	3.20E-01	Promoter (<=1kb)
Arih1	300756	chr8	64267932	64268232	323	0.98	4.82E-02	3.20E-01	Promoter (<=1kb)
Otulin	100362554	chr2	80238212	80238512	54674	1.22	4.82E-02	3.20E-01	Intron (NM_053714/114506, intron 9 of 12)
H2az1	58940	chr2	243105723	243106023	493	0.7	4.82E-02	3.20E-01	Promoter (<=1kb)
Sfmbt1	58967	chr16	6779216	6779516	0	0.78	4.82E-02	3.20E-01	Promoter (<=1kb)
Plbd2	246120	chr12	41600028	41600328	23	0.97	4.82E-02	3.20E-01	Promoter (<=1kb)
Serp2	498546	chr15	59121820	59122120	-409948	-1.35	4.83E-02	3.20E-01	Distal Intergenic
Snapin	295217	chr2	189888114	189888414	-7366	-1.27	4.83E-02	3.20E-01	3' UTR
Fbxo43	315034	chr7	74928962	74929262	9401	-1.43	4.83E-02	3.20E-01	Intron (NM_001012117/315034, intron 3 of 4)
Mtmr12	310155	chr2	62279305	62279605	42715	1.68	4.83E-02	3.20E-01	Intron (NM_001012077/310155, intron 7 of 14)
Hdac8	363481	chrX	72369442	72369742	309	0.66	4.83E-02	3.20E-01	Promoter (<=1kb)
Sh2b2	114203	chr12	23265082	23265382	-207888	-1.17	4.83E-02	3.20E-01	Distal Intergenic
Tmlhe	170898	chr20	237441	237741	0	0.67	4.83E-02	3.20E-01	Promoter (<=1kb)
Rassf9	65053	chr7	44264667	44264967	118322	-1.2	4.83E-02	3.20E-01	Distal Intergenic
Cerk	300129	chr7	127057481	127057781	275	0.8	4.83E-02	3.20E-01	Promoter (<=1kb)
Ythdf2	313053	chr5	150377056	150377356	12817	-1.52	4.83E-02	3.20E-01	Intron (NM_001047099/313053, intron 6 of 7)
Aamdc	361606	chr1	162600166	162600466	-50961	1.32	4.83E-02	3.20E-01	Distal Intergenic
Adamtsl3	308787	chr1	144271473	144271773	32515	1.63	4.83E-02	3.20E-01	Intron (NM_001107533/308787, intron 2 of 29)
Hs2st1	292155	chr2	250600172	250600472	45	1.11	4.83E-02	3.20E-01	Promoter (<=1kb)
Tcf20	366964	chr7	123754694	123754994	12803	0.91	4.83E-02	3.20E-01	Intron (NM_001130574/366964, intron 1 of 4)
Gja6	54256	chrX	35408745	35409045	-212760	0.96	4.83E-02	3.20E-01	Distal Intergenic
Smim20	501923	chr14	59964291	59964591	105279	-1.17	4.83E-02	3.20E-01	Distal Intergenic
Mtg2	296462	chr3	175448421	175448721	-22	0.52	4.83E-02	3.20E-01	Promoter (<=1kb)
Lamc2	192362	chr13	70542707	70543007	83245	1.16	4.83E-02	3.20E-01	Intron (NM_001048042/289095, intron 8 of 10)
Fosl2	25446	chr6	25808539	25808839	-191544	0.97	4.83E-02	3.20E-01	Intron (NM_199270/362704, intron 7 of 11)
Lrrc8d	305131	chr14	5194381	5194681	-93204	0.91	4.83E-02	3.20E-01	Distal Intergenic
Ubr4	313658	chr5	157848223	157848523	17	0.59	4.83E-02	3.20E-01	Promoter (<=1kb)
Klhl8	289457	chr14	7169672	7169972	153	1.04	4.84E-02	3.20E-01	Promoter (<=1kb)
Pdik1l	313609	chr5	152503710	152504010	2673	1.44	4.84E-02	3.20E-01	Promoter (2-3kb)
Cnih2	361705	chr1	220479700	220480000	132	1.17	4.84E-02	3.20E-01	Promoter (<=1kb)
Per2	63840	chr9	98597066	98597366	0	0.8	4.84E-02	3.20E-01	Promoter (<=1kb)
Rab31	246324	chr9	113504280	113504580	26	0.84	4.84E-02	3.20E-01	Promoter (<=1kb)
Pole4	362385	chr4	113497153	113497453	2	0.64	4.84E-02	3.20E-01	Promoter (<=1kb)
Prpf3	361995	chr2	197929450	197929750	41706	-1.3	4.84E-02	3.20E-01	Distal Intergenic
Vps13b	315036	chr7	74218186	74218486	99352	-1.32	4.84E-02	3.20E-01	Intron (NM_001134886/315036, intron 17 of 61)
Samd4b	308473	chr1	85342084	85342384	4279	1.35	4.84E-02	3.20E-01	Intron (NM_001107498/308473, intron 1 of 11)
Galt	298003	chr5	58145669	58145969	964	-1.13	4.85E-02	3.20E-01	Promoter (<=1kb)
Nlrp3	287362	chr10	45891839	45892139	-879	-0.91	4.85E-02	3.20E-01	Promoter (<=1kb)
Golim4	310526	chr2	174326785	174327085	86277	1.74	4.85E-02	3.20E-01	Intron (NM_001191567/310526, intron 15 of 16)
Ly86	291359	chr17	28077384	28077684	101198	0.6	4.85E-02	3.20E-01	Distal Intergenic
Thpo	81811	chr11	82832901	82833201	-14223	0.64	4.85E-02	3.20E-01	Intron (NM_001013978/303823, intron 2 of 14)
Zc3h12d	308266	chr1	1942819	1943119	4400	-1	4.85E-02	3.20E-01	Exon (NM_001107469/308266, exon 2 of 6)
Plpp4	309014	chr1	202921949	202922249	489583	-1.07	4.85E-02	3.20E-01	Distal Intergenic
Arntl	29657	chr1	178018644	178018944	-20119	0.72	4.86E-02	3.20E-01	Distal Intergenic
Hist1h2bl	64647	chr17	43796030	43796330	-12343	-0.94	4.86E-02	3.21E-01	Distal Intergenic
Ube2j1	297961	chr5	48274217	48274517	0	0.84	4.86E-02	3.21E-01	Promoter (<=1kb)
Tfap2a	306862	chr17	25643606	25643906	988704	-1.64	4.86E-02	3.21E-01	Distal Intergenic
Zeb2os	103691779	chr3	30297483	30297783	302730	1.26	4.86E-02	3.21E-01	Distal Intergenic
Sp2	303499	chr10	84914980	84915280	5606	0.62	4.86E-02	3.21E-01	Intron (NM_001107045/303499, intron 2 of 7)
Cdk2ap1	360804	chr12	37669169	37669469	800	0.88	4.86E-02	3.21E-01	Promoter (<=1kb)
Sh3d21	362598	chr5	144195688	144195988	-18914	-1.08	4.86E-02	3.21E-01	Intron (NM_001009693/313591, intron 3 of 11)
Snx29	689142	chr10	4083129	4083429	166286	1.74	4.86E-02	3.21E-01	Intron (NM_001109526/689142, intron 13 of 20)
Tmem67	313067	chr5	25753033	25753333	-31977	-1.34	4.86E-02	3.21E-01	Distal Intergenic
Chst11	314694	chr7	26721941	26722241	137475	-1.23	4.86E-02	3.21E-01	Intron (NM_001108079/314694, intron 2 of 2)
Agfg1	363266	chr9	88607828	88608128	320	0.93	4.86E-02	3.21E-01	Promoter (<=1kb)
Mcart1	313241	chr5	61048772	61049072	80954	-1.33	4.86E-02	3.21E-01	Distal Intergenic
Ccdc34	362187	chr3	101252506	101252806	96294	-1.37	4.87E-02	3.21E-01	Distal Intergenic
Usp48	362636	chr5	155942170	155942470	6616	-1.36	4.87E-02	3.21E-01	Intron (NM_198785/362636, intron 1 of 26)
Bdh1	117099	chr11	72577665	72577965	35959	-1.34	4.87E-02	3.21E-01	Downstream (<1kb)
Bri3bp	498176	chr12	36590267	36590567	-2428	1.42	4.87E-02	3.21E-01	Promoter (2-3kb)
Dhcr24	298298	chr5	126164788	126165088	80	0.66	4.87E-02	3.21E-01	Promoter (<=1kb)
Pfdn1	361310	chr18	29176855	29177155	113292	-1.31	4.87E-02	3.21E-01	Distal Intergenic
Stxbp3	114095	chr2	211658936	211659236	-20180	-1.46	4.87E-02	3.21E-01	Intron (NM_001107717/310787, intron 11 of 12)
Pkig	266709	chr3	160047246	160047546	0	0.8	4.87E-02	3.21E-01	Promoter (<=1kb)
Htra1	65164	chr1	201504873	201505173	5806	-1.12	4.87E-02	3.21E-01	Intron (NM_031721/65164, intron 1 of 8)
Col6a2	361821	chr20	12773535	12773835	15	0.77	4.87E-02	3.21E-01	Promoter (<=1kb)
Olr806	405331	chr4	72142714	72143014	-78536	0.56	4.88E-02	3.21E-01	Distal Intergenic
Slc6a9	116509	chr5	136670221	136670521	547	0.62	4.88E-02	3.21E-01	Promoter (<=1kb)
Irak1bp1	300862	chr8	90343326	90343626	19	0.6	4.88E-02	3.21E-01	Promoter (<=1kb)
Rnf19a	362900	chr7	75097948	75098248	83	0.81	4.88E-02	3.21E-01	Promoter (<=1kb)
Mir23a	100314228	chr19	25311269	25311569	-7013	0.58	4.88E-02	3.21E-01	Distal Intergenic
Mir3568	100526584	chr10	10860734	10861034	-5600	1.76	4.88E-02	3.21E-01	Exon (NM_001013964/302938, exon 2 of 17)
Sipa1l1	246212	chr6	106134797	106135097	82585	-1.06	4.88E-02	3.21E-01	Intron (NM_139330/246212, intron 14 of 20)
Ift81	373066	chr12	39459153	39459453	38992	-1.09	4.88E-02	3.21E-01	Intron (NM_199120/373066, intron 11 of 18)
Dele1	307480	chr18	31351837	31352137	-44850	0.98	4.88E-02	3.21E-01	Distal Intergenic
Dpy19l4	297824	chr5	24559671	24559971	106	1	4.88E-02	3.21E-01	Promoter (<=1kb)
Hecw1	291209	chr17	53230740	53231040	-303	0.82	4.88E-02	3.21E-01	Promoter (<=1kb)
Il6r	24499	chr2	189200265	189200565	53987	-1.1	4.88E-02	3.21E-01	Distal Intergenic
Zfp251	366954	chr7	117939577	117939877	-303	0.6	4.88E-02	3.21E-01	Promoter (<=1kb)
Pdlim2	290354	chr15	51849370	51849670	5655	1.57	4.88E-02	3.21E-01	Intron (NM_001007622/290354, intron 6 of 9)
Synrg	84479	chr10	71278704	71279004	6	1.17	4.88E-02	3.21E-01	Promoter (<=1kb)
Dennd5a	308942	chr1	174581545	174581845	6684	-1.31	4.88E-02	3.21E-01	Intron (NM_001107546/308942, intron 1 of 22)
Ncs1	65153	chr3	10597480	10597780	4892	1.71	4.89E-02	3.21E-01	Intron (NM_024366/65153, intron 1 of 7)
Brd1	315210	chr7	129719152	129719452	4851	-1.32	4.89E-02	3.21E-01	Exon (NM_001108103/315210, exon 2 of 13)
Add1	24170	chr14	81426264	81426564	0	1.03	4.89E-02	3.21E-01	Promoter (<=1kb)
Elovl5	171400	chr8	85249116	85249416	-10572	-0.92	4.89E-02	3.21E-01	Distal Intergenic
Fscn1	683788	chr12	13661141	13661441	7154	-1.24	4.89E-02	3.21E-01	Intron (NM_001100806/683788, intron 1 of 10)
Cbfa2t2	296293	chr3	149935751	149936051	20	0.73	4.89E-02	3.21E-01	Promoter (<=1kb)

Pccb	24624	chr8	109455554	109455854	-36683	0.94	4.89E-02	3.21E-01	Distal Intergenic
Cdc73	304832	chr13	60496205	60496505	6	0.98	4.89E-02	3.21E-01	Promoter (<=1kb)
LOC301165	301165	chr9	4512810	4513110	-119597	-1.46	4.89E-02	3.21E-01	Intron (NM_001013177/316153, intron 4 of 8)
Rabggta	58983	chr15	34403893	34404193	-3471	1.04	4.90E-02	3.21E-01	Distal Intergenic
Exd2	362759	chr6	104017937	104018237	48	0.5	4.90E-02	3.21E-01	Promoter (<=1kb)
Rab18	307039	chr17	59833618	59833918	-10863	-1.34	4.90E-02	3.21E-01	Distal Intergenic
Capn12	308476	chr1	87086695	87086995	20406	1.22	4.90E-02	3.21E-01	Exon (NM_031675/63836, exon 12 of 21)
Adam9	290834	chr16	71836642	71836942	26265	-1.1	4.90E-02	3.21E-01	Intron (NM_001014772/290834, intron 11 of 22)
Scara5	305974	chr15	49106287	49106587	37107	1.39	4.90E-02	3.21E-01	Intron (NM_001135855/305974, intron 3 of 8)
Taldo1	83688	chr1	214366922	214367222	-8333	1.38	4.90E-02	3.21E-01	Distal Intergenic
Smim1	500595	chr5	171307990	171308290	3736	1.57	4.90E-02	3.21E-01	Downstream (1-2kb)
Phtf2	296762	chr4	10923419	10923719	100161	-1.2	4.90E-02	3.21E-01	Intron (NM_001106577/296762, intron 13 of 17)
LOC100363521	100363521	chr4	40420424	40420724	0	0.52	4.90E-02	3.21E-01	Promoter (<=1kb)
Tmem17	360985	chr14	107369663	107369963	66855	1.13	4.90E-02	3.21E-01	Distal Intergenic
Syt2	24805	chr13	51630210	51630510	60962	0.77	4.90E-02	3.21E-01	Intron (NM_001107178/304813, intron 18 of 24)
Bmp1	83470	chr15	52204957	52205257	5489	-1.28	4.91E-02	3.21E-01	Intron (NM_031323/83470, intron 1 of 19)
Gatad2a	290669	chr16	21223092	21223392	45299	1.85	4.91E-02	3.21E-01	5' UTR
Mapt	29477	chr10	92425432	92425732	136430	-1.36	4.91E-02	3.21E-01	Distal Intergenic
Akap7	361458	chr1	21067225	21067525	-74619	1.21	4.91E-02	3.21E-01	Distal Intergenic
Smim13	690806	chr17	21353181	21353481	0	0.89	4.91E-02	3.21E-01	Promoter (<=1kb)
Dbn1	81653	chr17	9679913	9680213	301	0.69	4.91E-02	3.21E-01	Promoter (<=1kb)
Fam72a	681249	chr13	48299391	48299691	11320	-1.24	4.91E-02	3.21E-01	Distal Intergenic
Hivep1	117140	chr17	22421496	22421796	-18134	-1.15	4.91E-02	3.21E-01	Distal Intergenic
Cobl	305497	chr14	92341544	92341844	154050	1.62	4.91E-02	3.21E-01	Downstream (1-2kb)
Smurf2	303614	chr10	95124820	95125120	-6713	1.64	4.91E-02	3.21E-01	Distal Intergenic
Trps1	299897	chr7	90143781	90144081	174140	1.64	4.91E-02	3.21E-01	Intron (NM_001134837/299897, intron 5 of 6)
Ppp2r5a	312754	chr13	110077686	110077986	0	1.07	4.91E-02	3.21E-01	Promoter (<=1kb)
Ackr3	84348	chr9	97363461	97363761	7537	1.37	4.91E-02	3.21E-01	Intron (NM_053352/84348, intron 1 of 1)
RGD1565212	498534	chr15	47510893	47511193	-171962	-1.17	4.91E-02	3.21E-01	Intron (NM_053307/29447, intron 5 of 8)
Nt5m	287368	chr10	46216793	46217093	0	0.79	4.91E-02	3.21E-01	Promoter (<=1kb)
Neurod6	500137	chr4	85915508	85915808	-401	1.4	4.91E-02	3.21E-01	Promoter (<=1kb)
Zhx1	171159	chr7	97944160	97944460	31	0.69	4.92E-02	3.22E-01	Promoter (<=1kb)
Trpc7	282822	chr17	8148306	8148606	13055	1.9	4.92E-02	3.22E-01	Intron (NM_001191691/282822, intron 2 of 11)
Prkar2b	24679	chr6	51479822	51480122	-123439	1.57	4.92E-02	3.22E-01	Distal Intergenic
Plcb1	24654	chr3	127721652	127721952	408	0.8	4.92E-02	3.22E-01	Promoter (<=1kb)
Mal	25263	chr3	120205171	120205471	28184	1.91	4.92E-02	3.22E-01	Distal Intergenic
Ago4	298533	chr5	144556608	144556908	0	1.15	4.92E-02	3.22E-01	Promoter (<=1kb)
Sav1	299116	chr6	92398714	92399014	-36	1.02	4.92E-02	3.22E-01	Promoter (<=1kb)
Zfp958	100302405	chr12	5422441	5422741	69513	1.16	4.93E-02	3.22E-01	Distal Intergenic
Mgst2	295037	chr2	140701105	140701405	-6993	-1.22	4.93E-02	3.22E-01	Distal Intergenic
Mocs2	294753	chr2	47096580	47096880	115604	-0.83	4.93E-02	3.22E-01	Distal Intergenic
Lrrc28	361588	chr1	128563411	128563711	40459	-1.02	4.93E-02	3.22E-01	Intron (NM_001108486/361588, intron 5 of 9)
Coq2	498332	chr14	10581142	10581442	6	0.96	4.93E-02	3.22E-01	Promoter (<=1kb)
Ccdc130	304656	chr19	25378610	25378910	-3242	0.56	4.93E-02	3.22E-01	Distal Intergenic
Ccdc82	300359	chr8	11958067	11958367	69547	-1.27	4.93E-02	3.22E-01	Distal Intergenic
Anp32a	25379	chr8	67395927	67396227	100197	1.26	4.93E-02	3.22E-01	Distal Intergenic
Cyp26a1	154985	chr1	256460884	256461184	78023	-1.26	4.93E-02	3.22E-01	Distal Intergenic
Grk5	59075	chr1	282312543	282312843	47121	0.95	4.93E-02	3.22E-01	Intron (NM_030829/59075, intron 1 of 15)
Serinc1	294421	chr20	38984745	38985045	11	0.59	4.93E-02	3.22E-01	Promoter (<=1kb)
Pln	64672	chr20	34621510	34621810	-11347	1.59	4.93E-02	3.22E-01	Intron (NM_001177829/100365935, intron 3 of 12)
Btbd10	308890	chr1	178196168	178196468	0	0.59	4.93E-02	3.22E-01	Promoter (<=1kb)
Eif2s3	299027	chrX	63204163	63204463	86662	0.83	4.94E-02	3.22E-01	Distal Intergenic
Mef2a	309957	chr1	128417481	128417781	-76218	1.74	4.94E-02	3.22E-01	Distal Intergenic
Uckl1	499956	chr3	177078352	177078652	0	0.68	4.94E-02	3.22E-01	Promoter (<=1kb)
Piwil4	689972	chr8	13204870	13205170	46642	1.33	4.94E-02	3.22E-01	Distal Intergenic
Sdcbp2	311532	chr3	147066551	147066851	-6309	-1.29	4.94E-02	3.22E-01	Distal Intergenic
Pim3	64534	chr7	129851685	129851985	-4482	1.47	4.94E-02	3.22E-01	Distal Intergenic
Amotl2	65157	chr8	111250926	111251226	40115	-1.09	4.94E-02	3.22E-01	Distal Intergenic
Pdia4	116598	chr4	77465183	77465483	24052	1.51	4.94E-02	3.22E-01	Distal Intergenic
Slc30a1	58976	chr13	110653352	110653652	-24158	1.76	4.94E-02	3.22E-01	Distal Intergenic
Zfp438	307024	chr17	55008280	55008580	15	0.57	4.94E-02	3.22E-01	Promoter (<=1kb)
Lrrc1	367113	chr8	84837247	84837547	-2173	1.52	4.94E-02	3.22E-01	Promoter (2-3kb)
Agk	502749	chr4	68483643	68483943	5	0.64	4.94E-02	3.22E-01	Promoter (<=1kb)
Grem1	50566	chr3	105146340	105146640	68349	1.62	4.94E-02	3.22E-01	Distal Intergenic
Wdr19	305349	chr14	44791792	44792092	-24672	1.5	4.94E-02	3.22E-01	Distal Intergenic
Stx19	685180	chr7	711947	712247	-432755	0.5	4.95E-02	3.22E-01	Distal Intergenic
Fam83h	362937	chr7	117072333	117072633	-1397	1.52	4.95E-02	3.22E-01	Promoter (1-2kb)
Ppp6r3	309144	chr1	218805737	218806037	4081	-1.28	4.95E-02	3.22E-01	Intron (NM_001287134/309144, intron 1 of 22)
RGD1304770	309810	chr20	45004777	45005077	19238	0.54	4.95E-02	3.22E-01	Distal Intergenic
Siah1	140941	chr19	21383391	21383691	11228	1.62	4.95E-02	3.22E-01	Intron (NM_080905/140941, intron 1 of 1)
Kcmf1	684322	chr4	100790846	100791146	-7829	-1.23	4.95E-02	3.22E-01	Distal Intergenic
Arl6ip1	293551	chr1	187891611	187891911	-111936	-1.13	4.95E-02	3.22E-01	Distal Intergenic
Kremen1	114107	chr14	85503440	85503740	0	1.37	4.95E-02	3.22E-01	Promoter (<=1kb)
Unc119	29402	chr10	65606920	65607220	1	0.84	4.95E-02	3.22E-01	Promoter (<=1kb)
Ndst1	29633	chr18	56013549	56013849	-20666	1.02	4.95E-02	3.22E-01	Distal Intergenic
LOC690206	690206	chr5	155812268	155812568	18039	0.78	4.95E-02	3.22E-01	Distal Intergenic
Zbtb6	366029	chr3	21684031	21684331	0	0.61	4.95E-02	3.22E-01	Promoter (<=1kb)
Fam241a	691931	chr2	232244951	232245251	68	1.11	4.96E-02	3.22E-01	Promoter (<=1kb)
Mctp2	308742	chr1	132581319	132581619	921534	1.34	4.96E-02	3.22E-01	Distal Intergenic
Bhmt	81508	chr2	23099938	23100238	155920	0.84	4.96E-02	3.22E-01	Distal Intergenic
Twist1	85489	chr6	53401666	53401966	425	0.86	4.96E-02	3.22E-01	Promoter (<=1kb)
Tdrd3	306066	chr15	70732243	70732543	79	0.97	4.96E-02	3.22E-01	Promoter (<=1kb)
Sod1	24786	chr11	30363360	30363660	78	0.91	4.96E-02	3.22E-01	Promoter (<=1kb)
Calm2	50663	chr6	11093251	11093551	-13173	0.67	4.96E-02	3.22E-01	Distal Intergenic
St3gal4	363040	chr8	36323962	36324262	-9151	1.36	4.96E-02	3.22E-01	Exon (NM_153302/266605, exon 5 of 6)
Ndufa6	315167	chr7	123586553	123586853	66	0.63	4.96E-02	3.22E-01	Promoter (<=1kb)
Tmtc3	314785	chr7	40216653	40216953	103	0.6	4.96E-02	3.22E-01	Promoter (<=1kb)
Aco2	79250	chr7	123128353	123128653	25860	1.57	4.96E-02	3.22E-01	Intron (NM_024398/79250, intron 3 of 17)
Sgk1	29517	chr1	24392237	24392537	-198670	-1.24	4.96E-02	3.22E-01	Distal Intergenic
Zfp746	312303	chr4	77743303	77743603	3467	-1.23	4.97E-02	3.22E-01	Intron (NM_001100852/312303, intron 3 of 6)
Fat1	83720	chr16	50734956	50735256	-233240	-1.28	4.97E-02	3.22E-01	Distal Intergenic
Rhobtb1	309722	chr20	20670259	20670559	42569	0.66	4.97E-02	3.22E-01	Intron (NM_001107622/309722, intron 5 of 11)
Ranbp2	294429	chr20	28032327	28032627	5273	-1.4	4.97E-02	3.22E-01	Intron (NM_001191604/294429, intron 1 of 28)
Sh3glb1	292156	chr2	250737475	250737775	6421	-1.22	4.97E-02	3.22E-01	Intron (NM_001011929/292156, intron 1 of 8)

Eif3l	300069	chr7	120345212	120345512	24665	0.87	4.97E-02	3.22E-01	Distal Intergenic
Crebbp	54244	chr10	11594965	11595265	0	0.85	4.97E-02	3.22E-01	Promoter (<=1kb)
Cwc27	361887	chr2	34963116	34963416	-40395	1.53	4.97E-02	3.22E-01	Distal Intergenic
Atp6v1e1	297566	chr4	153373146	153373446	112	0.5	4.97E-02	3.22E-01	Promoter (<=1kb)
Cadm4	365216	chr1	81353914	81354214	-10986	1.48	4.97E-02	3.22E-01	Distal Intergenic
Ano4	299714	chr7	29995280	29995580	28956	1.65	4.97E-02	3.22E-01	Intron (NM_001106778/299714, intron 1 of 25)
Dnajb6	362293	chr4	2812665	2812965	101280	-1.3	4.97E-02	3.22E-01	Distal Intergenic
Yy1	24919	chr6	132716319	132716619	13738	-1.32	4.97E-02	3.22E-01	Intron (NM_173290/24919, intron 1 of 4)
Tmtc4	290501	chr15	109394507	109394807	98	0.91	4.97E-02	3.22E-01	Promoter (<=1kb)
Txnl1	140922	chr18	59096705	59097005	-10454	0.54	4.97E-02	3.22E-01	Distal Intergenic
Hspb7	50565	chr5	159966530	159966830	-1247	0.98	4.97E-02	3.22E-01	Promoter (1-2kb)
Polk	171525	chr2	27335882	27336182	28724	-0.97	4.98E-02	3.22E-01	Intron (NM_138516/171525, intron 3 of 14)
Acot2	192272	chr6	107460850	107461150	182	0.83	4.98E-02	3.22E-01	Promoter (<=1kb)
Ube2v1	296390	chr3	164357269	164357569	9115	-1.24	4.98E-02	3.22E-01	Intron (NM_001110345/296390, intron 1 of 3)
Rras	361568	chr1	101011734	101012034	-788	0.63	4.98E-02	3.23E-01	Promoter (<=1kb)
Gdf6	252834	chr5	22742100	22742400	-313945	1.53	4.98E-02	3.23E-01	Distal Intergenic
Comp	25304	chr16	20825084	20825384	-18014	0.91	4.98E-02	3.23E-01	Distal Intergenic
Cox7a2l	298762	chr6	6711464	6711764	-1681	1.34	4.98E-02	3.23E-01	Promoter (1-2kb)
Ptprm	29616	chr9	115555025	115555325	0	0.55	4.98E-02	3.23E-01	Promoter (<=1kb)
RGD1309748	302913	chr10	6846740	6847040	22971	-1.41	4.98E-02	3.23E-01	Intron (NM_001106972/302913, intron 4 of 4)
Vgll4	297523	chr4	146782563	146782863	56534	-1.08	4.98E-02	3.23E-01	Intron (NM_001015004/297523, intron 2 of 4)
Psmg4	689623	chr17	31411490	31411790	129	0.52	4.98E-02	3.23E-01	Promoter (<=1kb)
Ino80	296084	chr3	111283349	111283649	-16807	0.83	4.99E-02	3.23E-01	Distal Intergenic
Fnbp4	311183	chr3	79570754	79571054	129	0.52	4.99E-02	3.23E-01	Promoter (<=1kb)
Ddx4	310090	chr2	44510645	44510945	-6499	1.62	4.99E-02	3.23E-01	Distal Intergenic
Ttc7b	362768	chr6	124416455	124416755	17309	1.4	4.99E-02	3.23E-01	Exon (NM_001108719/362768, exon 4 of 19)
Gramd2b	307288	chr18	51470719	51471019	-21177	-0.98	4.99E-02	3.23E-01	Distal Intergenic
Mthfd2	680308	chr4	115015615	115015915	50	0.54	4.99E-02	3.23E-01	Promoter (<=1kb)
Tp53i11	311209	chr3	82122153	82122453	40256	1.07	4.99E-02	3.23E-01	Intron (NM_001107750/311210, intron 2 of 9)
Cnksr3	308113	chr1	43772367	43772667	111600	-1.19	4.99E-02	3.23E-01	Distal Intergenic
Plcb4	25031	chr3	128775242	128775542	173912	-1.26	4.99E-02	3.23E-01	Intron (NM_024353/25031, intron 3 of 38)
Gcnt3	286976	chr8	76458879	76459179	-6325	-1.21	4.99E-02	3.23E-01	Distal Intergenic
Sec24a	287275	chr10	37177088	37177388	38511	1.66	4.99E-02	3.23E-01	Intron (NM_001105780/287275, intron 10 of 23)
Gtf2i	353256	chr12	25408390	25408690	-2804	1.66	4.99E-02	3.23E-01	Promoter (2-3kb)
Gorab	304923	chr13	81596682	81596982	101845	1.66	4.99E-02	3.23E-01	Distal Intergenic
Cdk6	114483	chr4	28010841	28011141	-44443	1.09	4.99E-02	3.23E-01	Distal Intergenic
Hif1a	29560	chr6	96675203	96675503	-135682	-1.31	4.99E-02	3.23E-01	Intron (NM_031085/81749, intron 13 of 13)
Tprg1	360731	chr11	78764673	78764973	96772	-1.51	4.99E-02	3.23E-01	Intron (NM_001108320/360731, intron 4 of 5)
Gdpd1	303407	chr10	74384755	74385055	-8423	-1.08	5.00E-02	3.23E-01	Distal Intergenic
Dhrs7	299135	chr6	95541078	95541378	15	0.57	5.00E-02	3.23E-01	Promoter (<=1kb)
Pecam1	29583	chr10	94869977	94870277	42925	-1.12	5.00E-02	3.23E-01	Intron (NM_031591/29583, intron 8 of 8)
Ptbp1	29497	chr7	12683006	12683306	-9283	1.65	5.00E-02	3.23E-01	Intron (NM_001108067/314618, intron 22 of 22)
Nsmaf	353233	chr5	19518138	19518438	40806	1.65	5.00E-02	3.23E-01	Intron (NM_181389/353233, intron 21 of 31)

TABLE S2B Altered H3K4me3 peaks and associated genes after GRWD1 knockdown in KMM cells

Symbol	GeneID	Chromosome	Start	End	Distance To TSS	Fold	P-Value	FDR	Annotation
Mllt11	295264	chr2	196397766	196398066	4040	-2.34	1.09E-07	4.04E-03	Intron (NM_001024302/499671, intron 3 of 3)
RGD1562024	498699	chr17	8607268	8607568	12169	2.54	5.14E-07	4.75E-03	Distal Intergenic
Tax1bp1	246244	chr4	82850577	82850877	73831	3.03	5.54E-07	4.75E-03	Distal Intergenic
Ogfod2	288657	chr12	37913042	37913342	-2603	-2.38	5.84E-07	4.75E-03	Promoter (2-3kb)
Limk1	65172	chr12	25039082	25039382	2452	-2.25	6.44E-07	4.75E-03	Promoter (2-3kb)
Aqp1	25240	chr4	85551675	85551975	172	1.92	1.12E-06	6.89E-03	Promoter (<=1kb)
Golga4	501069	chr8	127271369	127271669	99733	-1.29	1.59E-06	8.39E-03	Intron (NM_001106865/301056, intron 1 of 7)
Larp6	315731	chr8	65611547	65611847	0	2.2	2.71E-06	1.18E-02	Promoter (<=1kb)
RGD1359127	299612	chr7	12253198	12253498	-2889	1.61	2.89E-06	1.18E-02	Promoter (2-3kb)
Aagab	171435	chr8	68599036	68599336	72941	3.04	3.78E-06	1.39E-02	Intron (NM_013095/25631, intron 1 of 8)
Mrpl55	287356	chr10	45555010	45555310	2215	-2.29	4.40E-06	1.41E-02	Promoter (2-3kb)
Pdk1	116551	chr3	58693590	58693890	162720	2.49	4.58E-06	1.41E-02	Distal Intergenic
St6galnac1	287920	chr10	105723222	105723522	178	2.48	5.16E-06	1.47E-02	Promoter (<=1kb)
Apol9a	503164	chr7	118922206	118922506	11272	3.07	5.58E-06	1.47E-02	Distal Intergenic
Trim71	301042	chr8	122594910	122595210	9577	2.12	6.28E-06	1.54E-02	Intron (NM_001191801/301042, intron 1 of 3)
Tnfrsf1a	25625	chr4	157932429	157932729	67439	2.71	6.69E-06	1.54E-02	Distal Intergenic
Hs3st3b1	303218	chr10	50401853	50402153	431	-2.16	1.09E-05	2.35E-02	Promoter (<=1kb)
Ercc6l	317252	chrX	72007555	72007855	26285	-1.73	1.16E-05	2.35E-02	Distal Intergenic
Hs2st1	292155	chr2	250440771	250441071	159446	2.52	1.21E-05	2.35E-02	Distal Intergenic
Slc44a2	363024	chr8	22368808	22369108	63	1.39	1.84E-05	3.33E-02	Promoter (<=1kb)
Scn5a	25665	chr8	128236257	128236557	30082	1.69	1.89E-05	3.33E-02	Intron (NM_001160162/25665, intron 4 of 26)
Bbs4	300754	chr8	64152663	64152963	1433	-2.33	2.61E-05	4.37E-02	Promoter (1-2kb)
Cdc42ep1	315121	chr7	120054627	120054927	-12625	1.92	2.73E-05	4.38E-02	Distal Intergenic
Cmtm8	301045	chr8	122904401	122904701	212	1.42	2.92E-05	4.49E-02	Promoter (<=1kb)
Pxylp1	315939	chr8	104792510	104792810	-1871	-1.87	3.22E-05	4.75E-02	Promoter (1-2kb)
Ifna2	298213	chr5	107538376	107538676	-121681	-2.06	3.88E-05	5.51E-02	Distal Intergenic
Gng7	58979	chr7	11603673	11603973	20689	1.81	5.74E-05	7.60E-02	Intron (NM_024138/58979, intron 1 of 2)
Timm21	307210	chr18	81804993	81805293	2334	-2.15	5.76E-05	7.60E-02	Promoter (2-3kb)
Scd2	83792	chr1	264058792	264059092	-282	1.45	6.43E-05	8.18E-02	Promoter (<=1kb)
Cerk	300129	chr7	126966585	126966885	91171	1.52	6.92E-05	8.33E-02	Distal Intergenic
Jrk	315073	chr7	115947696	115947996	-1225	-1.45	7.00E-05	8.33E-02	Promoter (1-2kb)
Cers2	310667	chr2	196486012	196486312	-1344	-2.28	7.23E-05	8.34E-02	Promoter (1-2kb)
Asz1	170578	chr4	45332481	45332781	81396	-1.66	8.13E-05	8.77E-02	Distal Intergenic
Taf10	293345	chr1	170583715	170584015	2413	-1.42	8.26E-05	8.77E-02	Promoter (2-3kb)
Pelo	294754	chr2	47264595	47264895	3631	2.13	8.32E-05	8.77E-02	Intron (NM_030994/25118, intron 1 of 28)
Ddost	313648	chr5	156665708	156666008	-2916	-1.5	8.92E-05	9.09E-02	Promoter (2-3kb)
Slc38a2	29642	chr7	138115309	138115609	-14486	-1.95	9.51E-05	9.09E-02	Distal Intergenic
Sestd1	295678	chr3	64123363	64123663	-10031	1.83	9.52E-05	9.09E-02	Distal Intergenic
Plet1	363060	chr8	54552451	54552751	-372915	1.98	9.74E-05	9.09E-02	Distal Intergenic
Myc	24577	chr7	102696074	102696374	109761	2.04	9.85E-05	9.09E-02	Distal Intergenic
Dram1	679937	chr7	28907971	28908271	24370	2.11	1.01E-04	9.09E-02	Intron (NM_001173427/679937, intron 4 of 6)
Rlbp1	293049	chr1	141065439	141065739	45636	-1.54	1.18E-04	1.01E-01	Intron (NM_001106275/293050, intron 5 of 10)
Dync1li2	81655	chr19	747067	747367	6840	1.3	1.22E-04	1.01E-01	Intron (NM_031026/81655, intron 4 of 11)
Mdm1	314859	chr7	61088881	61089181	-85188	2.27	1.23E-04	1.01E-01	Distal Intergenic
Ccdc190	498270	chr13	88197194	88197494	-67837	2.08	1.23E-04	1.01E-01	Distal Intergenic
Sirpa	25528	chr3	122117271	122117571	2517	1.54	1.44E-04	1.15E-01	Promoter (2-3kb)
Ptger4	84023	chr2	54964594	54964894	-1146	1.37	1.52E-04	1.19E-01	Promoter (1-2kb)
Atg101	300240	chr7	142962374	142962674	32957	1.21	1.61E-04	1.23E-01	Distal Intergenic
Bola3	297388	chr4	115039822	115040122	-6571	-2.07	1.63E-04	1.23E-01	3' UTR
Mybpc1	362867	chr7	29144879	29145179	26608	2	1.72E-04	1.25E-01	Intron (NM_001100758/362867, intron 3 of 34)
Marchf7	311059	chr3	46289328	46289628	-2286	-1.63	1.75E-04	1.25E-01	Promoter (2-3kb)
Meis1	686117	chr14	103310802	103311102	10027	-1.23	1.78E-04	1.25E-01	Intron (NM_001134702/686117, intron 6 of 12)
Fgf1	25317	chr18	32284692	32284992	-51110	-1.53	1.80E-04	1.25E-01	Intron (NM_012576/24413, intron 1 of 7)
Tmem125	313545	chr5	137374167	137374467	2342	1.07	1.85E-04	1.26E-01	Promoter (2-3kb)
Actr2	289820	chr14	104317631	104317931	57717	1.81	1.99E-04	1.33E-01	Distal Intergenic
Foxp2	500037	chr4	41779622	41779922	415181	1.53	2.29E-04	1.50E-01	Intron (NM_001271104/500037, intron 4 of 18)
LOC690352	690352	chr14	108297691	108297991	-12683	2.1	2.31E-04	1.50E-01	Distal Intergenic
Sh2d4a	306376	chr16	23285856	23286156	-129031	1.98	2.35E-04	1.50E-01	Distal Intergenic
Lsm1	364624	chr16	71059890	71060190	-2007	-1.56	2.58E-04	1.61E-01	Promoter (2-3kb)
Psmd1	83806	chr9	93074905	93075205	-5413	1.45	2.76E-04	1.70E-01	Distal Intergenic
Dclre1b	310745	chr2	206290894	206291194	2405	-1.82	2.82E-04	1.70E-01	Promoter (2-3kb)
Rtp4	360733	chr11	80642522	80642822	7980	1.22	2.86E-04	1.70E-01	Intron (NM_001108321/360733, intron 3 of 3)
Capn8	170808	chr13	100988252	100988552	7678	2.03	3.21E-04	1.87E-01	Intron (NM_133309/170808, intron 1 of 20)
Tmc1	361739	chr1	238662697	238662997	-136905	2.17	3.24E-04	1.87E-01	Distal Intergenic
Rdh8	690953	chr8	21859415	21859715	24312	2.03	3.38E-04	1.88E-01	Distal Intergenic
Sdccag8	305002	chr13	94880285	94880585	-7589	-1.46	3.43E-04	1.88E-01	Distal Intergenic
Dlg4	29495	chr10	56628497	56628797	950	2.13	3.44E-04	1.88E-01	Promoter (<=1kb)
Hesx1	498575	chr16	2605637	2605937	-28666	-1.39	3.46E-04	1.88E-01	Distal Intergenic
Gaa	367562	chr10	108345116	108345416	-50573	2.29	3.59E-04	1.91E-01	Intron (NM_001134688/287867, intron 5 of 26)
Ythdc1	170956	chr14	23005530	23005830	-3519	1.71	3.65E-04	1.91E-01	Distal Intergenic
Me2	307270	chr18	69798622	69798922	-14523	1.78	3.68E-04	1.91E-01	Distal Intergenic
Crb2	366031	chr3	22029073	22029373	-8984	1.4	3.85E-04	1.94E-01	Distal Intergenic
Afap1l2	292130	chr1	277826802	277827102	167	0.89	3.86E-04	1.94E-01	Promoter (<=1kb)
Hp1bp3	313647	chr5	156578717	156579017	-2715	-1.41	3.90E-04	1.94E-01	Promoter (2-3kb)
Tubgcp6	362980	chr7	130104220	130104520	-2362	-1.67	4.00E-04	1.96E-01	Promoter (2-3kb)
Fgf5	60662	chr14	13188244	13188544	-192660	1.84	4.03E-04	1.96E-01	Distal Intergenic
Eif1b	301068	chr8	129082865	129083165	-74947	1.17	4.11E-04	1.97E-01	Intron (NM_182844/360034, intron 2 of 15)
Epha2	366492	chr5	159821978	159822278	-23495	2.79	4.36E-04	2.01E-01	Distal Intergenic
Bard1	64557	chr9	78366419	78366719	2058	1.41	4.38E-04	2.01E-01	Promoter (2-3kb)
Extl1	313610	chr5	152574540	152574840	14116	-1.58	4.40E-04	2.01E-01	3' UTR
Magt1	116967	chrX	76976302	76976602	85002	-1.39	4.41E-04	2.01E-01	Distal Intergenic
Eef2k	25435	chr1	190810698	190810998	11993	-1.75	4.49E-04	2.01E-01	Intron (NM_012947/25435, intron 1 of 16)
Ndst4	362035	chr2	229171101	229171401	-25215	-1.16	4.51E-04	2.01E-01	Distal Intergenic
Nrp1	246331	chr19	61245045	61245345	-87006	1.47	4.80E-04	2.11E-01	Distal Intergenic
Mir3568	1.01E+08	chr10	10866764	10867064	-11630	-1.64	4.94E-04	2.12E-01	Intron (NM_001013964/302938, intron 1 of 16)
Clmp	286939	chr8	44845710	44846010	-1147	-1.23	5.01E-04	2.12E-01	Promoter (1-2kb)
Adamts19	361332	chr18	53915703	53916003	0	-1.9	5.04E-04	2.12E-01	Promoter (<=1kb)
Phf21b	300117	chr7	125653647	125653947	3959	-1.71	5.06E-04	2.12E-01	Intron (NM_001130680/300117, intron 2 of 12)
Bmi1	307151	chr17	85362994	85363294	-1189	-1.16	5.21E-04	2.16E-01	Promoter (1-2kb)
Thtpa	305889	chr15	33754040	33754340	-1138	-1.99	5.33E-04	2.16E-01	Promoter (1-2kb)
Pwwp2b	361671	chr1	211567247	211567547	-14530	1.53	5.33E-04	2.16E-01	Intron (NM_001143756/499281, intron 7 of 9)
Mir9-2	1E+08	chr2	11719299	11719599	-165072	-1.33	5.44E-04	2.16E-01	Distal Intergenic

Mob3b	366352	chr5	50656704	50657004	-18568	1.97	5.47E-04	2.16E-01	Distal Intergenic
Pi4ka	64161	chr11	87860332	87860632	1879	-1.33	5.52E-04	2.16E-01	Promoter (1-2kb)
Spdl1	303037	chr10	19654532	19654832	-1659	-1.34	5.55E-04	2.16E-01	Promoter (1-2kb)
RT1-T24-4	414784	chr20	3186045	3186345	-3112	-1.09	5.72E-04	2.20E-01	Distal Intergenic
Smim38	246306	chr1	218363573	218363873	10746	1.75	5.78E-04	2.20E-01	Distal Intergenic
Abcd2	84356	chr7	132462214	132462514	-119045	1.65	5.86E-04	2.21E-01	Intron (NM_133611/171147, intron 7 of 9)
Cers1	290658	chr16	20860450	20860750	39	0.95	5.94E-04	2.22E-01	Promoter (<=1kb)
Taf1a	360893	chr13	102120620	102120920	350342	1.6	6.05E-04	2.23E-01	Distal Intergenic
Tgfb2	81809	chr13	105013445	105013745	127035	1.36	6.13E-04	2.24E-01	Distal Intergenic
Irf5	296953	chr4	56806213	56806513	1081	1.87	6.36E-04	2.29E-01	Promoter (1-2kb)
Tfb1m	308140	chr1	44475968	44476268	-1294	-1.66	6.38E-04	2.29E-01	Promoter (1-2kb)
Nsf	60355	chr10	92008281	92008581	-229	-1.08	6.50E-04	2.31E-01	Promoter (<=1kb)
Hnf4a	25735	chr3	159898792	159899092	-3349	1.75	6.80E-04	2.35E-01	Distal Intergenic
Vps37b	288659	chr12	37971453	37971753	-13037	1.62	6.91E-04	2.35E-01	Distal Intergenic
Mir568	1E+08	chr11	62097849	62098149	-101727	1.3	6.96E-04	2.35E-01	Intron (NM_001105880/288105, intron 5 of 10)
Dnajc7	303536	chr10	88521512	88521812	11354	2.01	7.00E-04	2.35E-01	Intron (NM_213625/303536, intron 1 of 13)
Atf5	282840	chr1	100809601	100809901	621	-1.89	7.02E-04	2.35E-01	Promoter (<=1kb)
Ttyh3	304315	chr12	16091715	16092015	7449	1.61	7.11E-04	2.35E-01	Intron (NM_001107124/304315, intron 1 of 13)
Mylip	306825	chr17	19741455	19741755	-37774	1.95	7.12E-04	2.35E-01	Distal Intergenic
Supv3l1	294385	chr20	32043328	32043628	36542	1.89	7.15E-04	2.35E-01	Distal Intergenic
Tubgcp5	308663	chr1	114188644	114188944	1791	-1.37	7.28E-04	2.38E-01	Promoter (1-2kb)
Lrp5	293649	chr1	218914119	218914419	5675	1.3	7.42E-04	2.40E-01	Intron (NM_001106321/293649, intron 1 of 22)
Acsl4	113976	chrX	113761893	113762193	-102015	1.76	7.56E-04	2.42E-01	Distal Intergenic
Zfp111	170849	chr1	81057802	81058102	-10868	1.61	7.64E-04	2.42E-01	Distal Intergenic
Ctcf	83726	chr19	37603867	37604167	3490	-1.72	7.67E-04	2.42E-01	Intron (NM_031824/83726, intron 1 of 11)
Gls	24398	chr9	54130415	54130715	-82052	1.29	7.96E-04	2.47E-01	Distal Intergenic
Cyp2s1	308445	chr1	82610409	82610709	-59	1.47	7.98E-04	2.47E-01	Promoter (<=1kb)
Usp18	312688	chr4	153835670	153835970	29590	2.69	8.07E-04	2.47E-01	Distal Intergenic
LOC100910620	1.01E+08	chr5	74458594	74458894	-15922	1.72	8.16E-04	2.47E-01	Distal Intergenic
Disc1	307940	chr19	58092477	58092777	272217	-1.15	8.25E-04	2.47E-01	Distal Intergenic
Oat	64313	chr1	204429517	204429817	152253	1.56	8.35E-04	2.47E-01	Distal Intergenic
Clip1	65201	chr12	38354907	38355207	9451	-1.56	8.37E-04	2.47E-01	Intron (NM_031745/65201, intron 1 of 24)
Lrfn1	365222	chr1	85290820	85291120	13923	1.73	8.43E-04	2.47E-01	3' UTR
Glg1	29476	chr19	42956945	42957245	-26634	1.83	8.50E-04	2.47E-01	Distal Intergenic
Ptpru	116680	chr5	149994820	149995120	1214	1.34	8.51E-04	2.47E-01	Promoter (1-2kb)
Atf2	81647	chr3	60676612	60676912	119039	-0.89	8.76E-04	2.50E-01	Distal Intergenic
Adcy6	25289	chr7	140285755	140286055	4741	-2.23	8.77E-04	2.50E-01	Intron (NM_012821/25289, intron 1 of 21)
Cyp51	25427	chr4	27072204	27072504	-103060	-1.96	8.79E-04	2.50E-01	Distal Intergenic
Ubb	192255	chr10	48878192	48878492	-1739	-1.23	9.05E-04	2.51E-01	Promoter (1-2kb)
Hjv	310681	chr2	198562918	198563218	-92219	1.53	9.06E-04	2.51E-01	Distal Intergenic
Acd	307798	chr19	37664724	37665024	2963	-1.55	9.13E-04	2.51E-01	Promoter (2-3kb)
Serpine1	24617	chr12	22650206	22650506	9102	-0.89	9.24E-04	2.51E-01	3' UTR
Homer1	29546	chr2	22864012	22864312	-45825	1.59	9.24E-04	2.51E-01	Distal Intergenic
Cyp26b1	312495	chr4	116276806	116277106	1509	1.49	9.37E-04	2.51E-01	Promoter (1-2kb)
Oasl	304545	chr12	47455941	47456241	0	1.69	9.40E-04	2.51E-01	Promoter (<=1kb)
Trpv4	66026	chr12	47690010	47690310	-8637	-0.84	9.41E-04	2.51E-01	Distal Intergenic
Cd37	29185	chr1	101235261	101235561	72	1.6	9.52E-04	2.53E-01	Promoter (<=1kb)
Fstl3	114031	chr7	12810253	12810553	3	1.16	9.63E-04	2.54E-01	Promoter (<=1kb)
Ndufs1	301458	chr9	69950850	69951150	2032	1.48	9.86E-04	2.56E-01	Promoter (2-3kb)
Slc25a24	310791	chr2	211932258	211932558	1824	-1.25	9.87E-04	2.56E-01	Promoter (1-2kb)
Jpt1	287828	chr10	104071268	104071568	4208	-1.53	9.91E-04	2.56E-01	Intron (NM_001005876/287828, intron 1 of 4)
Atxn1	25049	chr17	19283239	19283539	122245	-1.56	1.03E-03	2.63E-01	Intron (NM_012726/25049, intron 2 of 6)
Srsf3	361814	chr20	6310280	6310580	21995	1.2	1.04E-03	2.65E-01	Distal Intergenic
Cpm	314855	chr7	60601904	60602204	-52146	-1.05	1.05E-03	2.65E-01	Distal Intergenic
Lrrc17	502715	chr4	10138178	10138478	174	1.54	1.07E-03	2.66E-01	Promoter (<=1kb)
Pias2	83422	chr18	73359659	73359959	-18735	1.53	1.07E-03	2.66E-01	Distal Intergenic
Metap1	295500	chr2	243778892	243779192	-1219	1.24	1.07E-03	2.66E-01	Promoter (1-2kb)
Ldhal6b	369018	chr1	46080335	46080635	228641	-1.28	1.09E-03	2.68E-01	Distal Intergenic
Psmd4	83499	chr2	196183211	196183511	24052	-1.45	1.10E-03	2.68E-01	Distal Intergenic
Cntnap5b	301650	chr13	20105148	20105448	-1573064	1.26	1.11E-03	2.69E-01	Distal Intergenic
C1galt1	65044	chr4	33948954	33949254	40655	1.3	1.13E-03	2.72E-01	Distal Intergenic
Eif2b1	64514	chr12	37447147	37447447	3011	-1.66	1.14E-03	2.72E-01	Intron (NM_172029/64514, intron 4 of 8)
Maf	54267	chr19	48259665	48259965	-62917	1.02	1.17E-03	2.77E-01	Distal Intergenic
Amz1	304317	chr12	16004544	16004844	-7605	1.95	1.18E-03	2.77E-01	Distal Intergenic
Zfp638	312491	chr4	115570059	115570359	-31302	1.13	1.18E-03	2.77E-01	Distal Intergenic
Nr2c2	50659	chr4	123948313	123948613	-56674	1.76	1.19E-03	2.78E-01	Distal Intergenic
Ints2	360589	chr10	73554304	73554604	136283	-1.57	1.21E-03	2.80E-01	Distal Intergenic
Csnk1a1	113927	chr18	56892122	56892422	4400	-1.55	1.22E-03	2.80E-01	Intron (NM_053615/113927, intron 2 of 8)
Msx1	81710	chr14	77688649	77688949	-23313	1.66	1.23E-03	2.80E-01	Distal Intergenic
Htatip2	292935	chr1	105097849	105098149	3007	1.54	1.24E-03	2.80E-01	Intron (NM_001106263/292935, intron 2 of 4)
Ptpn13	498331	chr14	7894889	7895189	-31225	1.69	1.24E-03	2.80E-01	Distal Intergenic
Ccdc77	312677	chr4	152886617	152886917	-3407	-0.79	1.25E-03	2.80E-01	Distal Intergenic
Plcb4	25031	chr3	128276272	128276572	-324758	-1.52	1.27E-03	2.85E-01	Intron (NM_001077641/24654, intron 15 of 32)
Acsl5	94340	chr1	276244783	276245083	4080	-1.45	1.31E-03	2.90E-01	Intron (NM_053607/94340, intron 1 of 24)
LOC499584	499584	chr2	109401291	109401591	-904772	2.18	1.34E-03	2.95E-01	Distal Intergenic
Slco3a1	140915	chr1	135967591	135967891	105592	1.5	1.36E-03	2.95E-01	Intron (NM_177481/140915, intron 2 of 9)
Ldha	24533	chr1	102892761	102893061	-7227	1.63	1.36E-03	2.95E-01	Distal Intergenic
Zdhhc7	170906	chr19	52725471	52725771	24536	1.34	1.36E-03	2.95E-01	Distal Intergenic
Lnx1	360926	chr14	35950063	35950363	-96781	1.34	1.37E-03	2.95E-01	Distal Intergenic
Trappc4	367073	chr8	48724890	48725190	1766	-1.32	1.38E-03	2.95E-01	Promoter (1-2kb)
Pdgfa	25266	chr12	17720902	17721202	-12939	1.13	1.38E-03	2.95E-01	Distal Intergenic
Nectin1	192183	chr8	47913944	47914244	-179989	1.13	1.39E-03	2.95E-01	Distal Intergenic
Nxf1	59087	chr1	224955570	224955870	-1665	-1.29	1.40E-03	2.95E-01	Promoter (1-2kb)
Pigu	353304	chr3	150850111	150850411	35064	1.06	1.41E-03	2.95E-01	Exon (NM_181637/353304, exon 6 of 13)
Magoh	298385	chr5	127403044	127403344	-77171	-1.58	1.42E-03	2.95E-01	Distal Intergenic
Tmem154	361972	chr2	183674419	183674719	0	1.19	1.42E-03	2.95E-01	Promoter (<=1kb)
C2cd2	304055	chr11	38353528	38353828	66204	-1.81	1.43E-03	2.95E-01	Distal Intergenic
Ist1	307833	chr19	41930700	41931000	-37705	-1.36	1.46E-03	2.99E-01	Distal Intergenic
Cplx2	116657	chr17	10835063	10835363	-68810	1.57	1.48E-03	3.02E-01	Distal Intergenic
Cidea	291541	chr18	63082774	63083074	0	1.59	1.51E-03	3.05E-01	Promoter (<=1kb)
Creb3l1	362165	chr3	81102108	81102408	-168825	-1.17	1.51E-03	3.05E-01	Distal Intergenic
Hcn4	59266	chr8	63802267	63802567	202360	-0.77	1.52E-03	3.05E-01	Distal Intergenic
Pdlim1	54133	chr1	259353555	259353855	3151	1.36	1.53E-03	3.05E-01	Intron (NM_017365/54133, intron 1 of 6)
Vom2r67	689485	chr14	1339673	1339973	21612	-0.82	1.56E-03	3.10E-01	Distal Intergenic

Mtbp	500870	chr7	95307596	95307896	-2032	-1.43	1.58E-03	3.12E-01	Promoter (2-3kb)
Abcb10	361439	chr19	56770710	56771010	1894	-1.45	1.60E-03	3.13E-01	Promoter (1-2kb)
Smad7	81516	chr18	71494700	71495000	98870	1.55	1.61E-03	3.14E-01	Intron (NM_001108891/364900, intron 3 of 4)
Ccnl1	114121	chr2	157773260	157773560	-13422	1.83	1.62E-03	3.14E-01	Distal Intergenic
Ago2	59117	chr7	114385390	114385690	-8113	-0.96	1.62E-03	3.14E-01	Distal Intergenic
Cnot6l	360917	chr14	15039757	15040057	-19330	-1.05	1.68E-03	3.24E-01	Distal Intergenic
Arhgef9	66013	chrX	64212488	64212788	215656	1.33	1.70E-03	3.24E-01	Distal Intergenic
Phpt1	296571	chr3	2805018	2805318	-6582	1.26	1.72E-03	3.27E-01	Downstream (1-2kb)
H3f3b	117056	chr10	104579791	104580091	-3904	-1.52	1.76E-03	3.31E-01	Distal Intergenic
Cdkn2aipnl	287278	chr10	37395100	37395400	-27247	1.64	1.77E-03	3.31E-01	Distal Intergenic
Cox6a2	25278	chr1	199634488	199634788	-9705	1.41	1.77E-03	3.31E-01	Distal Intergenic
Pc	25104	chr1	219762368	219762668	3185	-1.29	1.78E-03	3.31E-01	Intron (NM_012744/25104, intron 1 of 19)
Mfsd2a	298504	chr5	140657287	140657587	158	0.98	1.79E-03	3.31E-01	Promoter (<=1kb)
Tbcc	316221	chr9	16403303	16403603	2735	-1.22	1.80E-03	3.31E-01	Promoter (2-3kb)
Rps17	29286	chr1	143167029	143167329	2586	-1.38	1.81E-03	3.31E-01	Promoter (2-3kb)
Elmod1	315670	chr8	58542629	58542929	0	0.92	1.81E-03	3.31E-01	Promoter (<=1kb)
Crot	83842	chr4	22083756	22084056	2152	-1.9	1.84E-03	3.34E-01	Promoter (2-3kb)
Cpn2	303861	chr11	74074326	74074626	16931	1.5	1.86E-03	3.37E-01	Distal Intergenic
Fbxl5	305424	chr14	71857578	71857878	4848	-1.25	1.90E-03	3.40E-01	Intron (NM_001107222/305424, intron 1 of 9)
Cep120	307302	chr18	48716700	48717000	3472	-1.21	1.90E-03	3.40E-01	Intron (NM_001191697/307302, intron 2 of 20)
Cnep1r1	291914	chr19	19913923	19914223	-318	1.42	1.92E-03	3.41E-01	Promoter (<=1kb)
Tm7sf2	293688	chr1	221428323	221428623	1717	-1.15	1.92E-03	3.41E-01	Promoter (1-2kb)
Tanc1	311055	chr3	45743325	45743625	24744	1.48	1.94E-03	3.43E-01	Intron (NM_001002854/311055, intron 1 of 25)
Ugcg	83626	chr5	76558325	76558625	171487	1.39	1.95E-03	3.43E-01	Distal Intergenic
Phex	25512	chrX	41037475	41037775	577428	1.87	1.96E-03	3.43E-01	Distal Intergenic
Zfp709l1	690419	chr7	10364192	10364714	72003	1.5	1.98E-03	3.45E-01	Distal Intergenic
Tns1	301509	chr9	81241276	81241873	159114	-1.22	2.04E-03	3.52E-01	Intron (NM_001191810/301509, intron 18 of 34)
Cacna1g	29717	chr10	82197802	82198102	-282	0.8	2.06E-03	3.52E-01	Promoter (<=1kb)
Slc16a5	690212	chr10	104017877	104018177	-1711	1.03	2.06E-03	3.52E-01	Promoter (1-2kb)
Nxpe3	681096	chr11	47189758	47190058	1263	-1.33	2.06E-03	3.52E-01	Promoter (1-2kb)
LOC100910620	1.01E+08	chr5	74461394	74461694	-18722	-1.25	2.07E-03	3.52E-01	Distal Intergenic
Abhd3	291793	chr18	1946508	1946808	32	0.67	2.14E-03	3.62E-01	Promoter (<=1kb)
Larp4b	307070	chr17	63232981	63233281	-2726	-1.39	2.15E-03	3.62E-01	Promoter (2-3kb)
Sgms1	353229	chr1	250858380	250858680	93006	1.5	2.20E-03	3.69E-01	Intron (NM_181386/353229, intron 2 of 9)
Pax8	81819	chr3	1684417	1684717	-99492	1.3	2.23E-03	3.72E-01	Distal Intergenic
Avpi1	171386	chr1	261377776	261378076	-6268	1.47	2.24E-03	3.72E-01	Distal Intergenic
Ralgapb	362257	chr3	154979553	154979853	68927	-1.29	2.25E-03	3.72E-01	3' UTR
LOC100911360	1.01E+08	chr8	73419747	73420047	114948	1.31	2.26E-03	3.72E-01	Distal Intergenic
LOC102548847	1.03E+08	chr6	144294416	144294716	89899	-1.38	2.30E-03	3.77E-01	Distal Intergenic
Adprhl2	362600	chr5	144317619	144317919	23667	1.88	2.32E-03	3.79E-01	Distal Intergenic
Cyb561	303601	chr10	94147031	94147331	225	0.75	2.35E-03	3.82E-01	Promoter (<=1kb)
Trrap	288471	chr12	11624966	11625266	2297	-1.3	2.38E-03	3.83E-01	Promoter (2-3kb)
Ly6i	246138	chr7	116445084	116445384	-20514	1.46	2.39E-03	3.83E-01	Intron (NM_001113792/503162, intron 3 of 4)
Ccdc152	499536	chr2	53199238	53199538	-60965	-1.34	2.41E-03	3.83E-01	Intron (NM_017094/25235, intron 3 of 9)
Bcas1	246755	chr3	168011956	168012256	21201	1.67	2.41E-03	3.83E-01	Intron (NM_145670/246755, intron 3 of 10)
Cxxc5	291670	chr18	28692899	28693199	39246	1.13	2.42E-03	3.83E-01	Distal Intergenic
Ccdc181	360867	chr13	82574588	82574888	-92	-0.97	2.42E-03	3.83E-01	Promoter (<=1kb)
Uxt	299313	chrX	1273488	1273788	-1824	-1.51	2.50E-03	3.94E-01	Promoter (1-2kb)
Comt	24267	chr11	86695397	86695697	-20284	1.47	2.51E-03	3.94E-01	Intron (NM_022584/50551, intron 8 of 17)
Reck	313488	chr5	59355495	59355795	6855	-1.14	2.52E-03	3.94E-01	Intron (NM_001107954/313488, intron 1 of 19)
Abtb1	297432	chr4	120768220	120768520	16287	1.29	2.54E-03	3.94E-01	Exon (NM_138502/29254, exon 7 of 8)
Adarb1	25367	chr20	12012549	12012849	40168	1.32	2.54E-03	3.94E-01	Intron (NM_001111055/25367, intron 1 of 10)
Ppm1b	24667	chr6	8219526	8219826	141	-1.2	2.55E-03	3.94E-01	Promoter (<=1kb)
Tns3	360980	chr14	88984705	88985005	-137834	1.76	2.57E-03	3.96E-01	Distal Intergenic
Hrh2	25461	chr17	10852533	10852833	77228	1.27	2.59E-03	3.96E-01	Distal Intergenic
LOC100911827	1.01E+08	chr1	265043163	265043463	36547	0.83	2.60E-03	3.96E-01	Distal Intergenic
Rph3al	171123	chr10	64139642	64139942	-31088	1.1	2.62E-03	3.98E-01	Distal Intergenic
Gpcpd1	362219	chr3	125318814	125319114	-105216	-1.2	2.64E-03	3.98E-01	Distal Intergenic
Tanc1	311055	chr3	45742631	45742931	24050	1.24	2.64E-03	3.98E-01	Intron (NM_001002854/311055, intron 1 of 25)
Cog8	291990	chr19	39254890	39255190	2216	-1.38	2.68E-03	4.00E-01	Promoter (2-3kb)
Fndc3b	294925	chr2	113361239	113361539	-15688	1.49	2.68E-03	4.00E-01	Distal Intergenic
RGD1306441	290425	chr15	80058372	80058672	17530	1.3	2.69E-03	4.00E-01	Distal Intergenic
Tm9sf4	296279	chr3	148614933	148615233	-20589	1.22	2.70E-03	4.00E-01	Intron (NM_013185/25734, intron 10 of 12)
Bach1	304127	chr11	27742745	27743045	377550	-1.21	2.71E-03	4.00E-01	Distal Intergenic
Camk2d	24246	chr2	231382185	231382485	481059	-1.18	2.76E-03	4.03E-01	Distal Intergenic
Tnfaip8	307428	chr18	44749721	44750021	12567	-1.05	2.76E-03	4.03E-01	Intron (NM_001107387/307428, intron 3 of 3)
LOC689713	689713	chr16	84685438	84685738	219782	-1.12	2.77E-03	4.03E-01	Intron (NM_138893/192253, intron 26 of 33)
Eps8l3	295361	chr2	210609777	210610077	-58326	1.86	2.78E-03	4.03E-01	Distal Intergenic
Twist1	85489	chr6	53399432	53399732	-1509	-1.54	2.84E-03	4.08E-01	Promoter (1-2kb)
Vrk1	362779	chr6	129789733	129790033	-45886	-1.13	2.84E-03	4.08E-01	Distal Intergenic
Eif6	305506	chr3	151364073	151364373	-958	-1.29	2.85E-03	4.08E-01	Promoter (<=1kb)
Mon1a	315999	chr8	116656665	116656965	-434	-0.91	2.85E-03	4.08E-01	Promoter (<=1kb)
Zkscan7	363170	chr8	131935070	131935370	1683	-1.34	2.86E-03	4.08E-01	Promoter (1-2kb)
Larp4b	307070	chr17	63269546	63269846	33539	-1.17	2.88E-03	4.08E-01	Intron (NM_001107361/307070, intron 4 of 18)
Pltp	296371	chr3	161321249	161321549	740	1.05	2.90E-03	4.09E-01	Promoter (<=1kb)
Inpp5j	171088	chr14	83741602	83741902	67	0.91	2.91E-03	4.10E-01	Promoter (<=1kb)
Mtx1	295241	chr2	188523903	188524203	3924	-1.5	2.92E-03	4.10E-01	Exon (NM_001100667/295241, exon 4 of 7)
Hsp90b1	362862	chr7	27242662	27242962	-2129	-1.13	2.96E-03	4.12E-01	Promoter (2-3kb)
Cant1	246272	chr10	107435142	107435442	10080	1.57	2.96E-03	4.12E-01	Intron (NM_144754/246272, intron 2 of 3)
Grina	266668	chr7	117309974	117310274	5232	-1.23	2.97E-03	4.12E-01	Distal Intergenic
Pxylp1	315939	chr8	104748456	104748756	41883	-1.22	3.02E-03	4.16E-01	Intron (NM_001007710/315939, intron 2 of 5)
Cyb5a	64001	chr18	81694155	81694455	-363	-0.92	3.03E-03	4.16E-01	Promoter (<=1kb)
Sncg	64347	chr16	10725222	10725522	1123	1.73	3.08E-03	4.16E-01	Promoter (1-2kb)
Phlda1	29380	chr7	54364188	54364488	116728	0.94	3.08E-03	4.16E-01	Distal Intergenic
Vcpip1	286761	chr5	9173861	9174161	-57019	-1.88	3.09E-03	4.16E-01	Distal Intergenic
Six1	114634	chr6	95928520	95928820	5476	-1.35	3.09E-03	4.16E-01	Downstream (<1kb)
Foxp1	297480	chr4	131387208	131387508	210796	1.12	3.09E-03	4.16E-01	Intron (NM_001034131/297480, intron 9 of 15)
Nog	25495	chr10	76686382	76686682	126704	0.84	3.10E-03	4.16E-01	Distal Intergenic
Fam160a2	293343	chr1	170237547	170237847	1045	-1.17	3.12E-03	4.16E-01	Promoter (1-2kb)
Comp	25304	chr16	20808733	20809033	-1663	1.11	3.13E-03	4.16E-01	Promoter (1-2kb)
Mllt3	114510	chr5	106341767	106342067	-139206	-1.36	3.14E-03	4.16E-01	Distal Intergenic
Azin2	366473	chr5	147112593	147112893	35683	1.68	3.15E-03	4.16E-01	Distal Intergenic
Clec2g	362447	chr4	162930324	162930624	-3571	1	3.15E-03	4.16E-01	Distal Intergenic
Cyp26b1	312495	chr4	116275502	116275802	2813	1.76	3.18E-03	4.17E-01	Promoter (2-3kb)

Wnt5a	64566	chr16	4468264	4468564	-887	-1.42	3.18E-03	4.17E-01	Promoter (<=1kb)
Cox7c	1E+08	chr2	15348470	15348770	-646567	-1.04	3.19E-03	4.17E-01	Distal Intergenic
Rdm1	287726	chr10	89539744	89540044	-289	-1.15	3.21E-03	4.18E-01	Promoter (<=1kb)
Atxn1	25049	chr17	19274572	19274872	113578	1.43	3.22E-03	4.18E-01	Intron (NM_012726/25049, intron 2 of 6)
Slc9a3r1	59114	chr10	103710024	103710324	-2721	1.44	3.24E-03	4.19E-01	Promoter (2-3kb)
Bbc3	317673	chr1	78261816	78262116	311	1.51	3.25E-03	4.19E-01	Promoter (<=1kb)
Zbtb3	499313	chr1	224997489	224997789	-383	-0.77	3.26E-03	4.19E-01	Promoter (<=1kb)
Chd2	308738	chr1	134962601	134962901	-91433	0.75	3.28E-03	4.19E-01	Distal Intergenic
Ltbp2	59106	chr6	108602619	108602919	-6173	-1.17	3.29E-03	4.19E-01	Distal Intergenic
Ppp1r15b	304799	chr13	49905108	49905408	-27747	1.54	3.30E-03	4.19E-01	Intron (NM_001105951/289021, intron 28 of 31)
Camk2d	24246	chr2	230838679	230838979	-62147	-0.94	3.32E-03	4.20E-01	Distal Intergenic
Dpy30	286897	chr6	22303220	22303520	1151	-1.15	3.32E-03	4.20E-01	Promoter (1-2kb)
Tgfbr3	29610	chr14	3508665	3508965	2249	-0.89	3.34E-03	4.20E-01	Promoter (2-3kb)
Mybl1	297783	chr5	9285440	9285740	5575	1.74	3.34E-03	4.20E-01	Intron (NM_001106632/297783, intron 1 of 15)
Elac2	282826	chr10	51480488	51480788	2110	-1.34	3.39E-03	4.23E-01	Promoter (2-3kb)
Lyzl6	287751	chr10	91690394	91690694	8730	-0.93	3.40E-03	4.23E-01	Distal Intergenic
Plet1	363060	chr8	54600867	54601167	-324499	1.76	3.40E-03	4.23E-01	Distal Intergenic
Cacna1a	25398	chr19	25610709	25611009	83958	-1.02	3.46E-03	4.27E-01	Intron (NM_012918/25398, intron 3 of 46)
Etfbkmt	316976	chr4	183657789	183658089	1776	-1.37	3.48E-03	4.27E-01	Promoter (1-2kb)
Fam241b	294499	chr20	31688768	31689068	47047	0.89	3.48E-03	4.27E-01	Distal Intergenic
Ccl28	114492	chr2	52393059	52393359	13718	-1.08	3.49E-03	4.27E-01	Intron (NM_053700/114492, intron 1 of 2)
Ror2	306782	chr17	11954106	11954406	554	-1.63	3.49E-03	4.27E-01	Promoter (<=1kb)
Hmgcl	79238	chr5	154296074	154296374	1233	1.26	3.50E-03	4.27E-01	Promoter (1-2kb)
Zfp958	1E+08	chr12	5422402	5422702	69552	-1.18	3.54E-03	4.29E-01	Distal Intergenic
Wdr89	314243	chr6	98856231	98856531	-250122	1.44	3.55E-03	4.29E-01	Distal Intergenic
Tesk1	29460	chr5	58847364	58847664	-89951	1.19	3.57E-03	4.29E-01	Distal Intergenic
Cebpb	24253	chr3	164524613	164524913	100111	1.06	3.57E-03	4.29E-01	Distal Intergenic
Ago2	59117	chr7	114413639	114413939	-36362	1.96	3.59E-03	4.29E-01	Distal Intergenic
Ptbp2	310820	chr2	223323424	223323724	-882	-1.61	3.60E-03	4.29E-01	Promoter (<=1kb)
Mgarp	689931	chr2	140452996	140453296	0	1.32	3.69E-03	4.37E-01	Promoter (<=1kb)
Txnl4a	1.01E+08	chr18	76724210	76724510	-711	-1.61	3.69E-03	4.37E-01	Promoter (<=1kb)
Etv5	303828	chr11	82198690	82198990	4033	1.12	3.70E-03	4.37E-01	Intron (NM_001107082/303828, intron 5 of 12)
Pak1	29431	chr1	162768482	162768782	326	0.78	3.72E-03	4.37E-01	Promoter (<=1kb)
Fam98a	313873	chr6	21050638	21050938	-389	-1	3.72E-03	4.37E-01	Promoter (<=1kb)
Pbx3	311876	chr3	13447062	13447362	-11040	1.69	3.74E-03	4.38E-01	Distal Intergenic
Diaph1	307483	chr18	31013688	31013988	57383	-1.27	3.75E-03	4.38E-01	Intron (NM_001107393/307483, intron 19 of 29)
Sptb	314251	chr6	99671782	99672082	110987	1.29	3.78E-03	4.41E-01	Distal Intergenic
Hspb6	192245	chr1	89010842	89011142	2725	-1.09	3.81E-03	4.42E-01	Promoter (2-3kb)
Fbxo39	303287	chr10	58861064	58861364	-14467	0.72	3.83E-03	4.43E-01	Distal Intergenic
Angptl4	362850	chr7	18636121	18636421	-2078	-1.09	4.01E-03	4.62E-01	Promoter (2-3kb)
Gpn1	688393	chr6	26272918	26273218	-163	-1.09	4.04E-03	4.62E-01	Promoter (<=1kb)
Dnajb6	362293	chr4	2754717	2755017	43332	1.76	4.04E-03	4.62E-01	Intron (NM_001013209/362293, intron 8 of 9)
Rab11b	79434	chr7	18608481	18608781	3351	-1.46	4.05E-03	4.62E-01	Intron (NM_032617/79434, intron 1 of 4)
LOC100361087	1E+08	chr13	88608588	88608888	33123	-1.57	4.06E-03	4.62E-01	Distal Intergenic
Adrm1	65138	chr3	175547521	175547821	-360	1.34	4.11E-03	4.66E-01	Promoter (<=1kb)
Hexim1	498008	chr10	91196754	91197054	-3820	-0.76	4.18E-03	4.72E-01	Distal Intergenic
Zdhhc18	362613	chr5	151856669	151856969	-413	1.51	4.20E-03	4.72E-01	Promoter (<=1kb)
Abt1	306960	chr17	43924034	43924334	-21796	0.84	4.20E-03	4.72E-01	Distal Intergenic
Sdcbp	83841	chr5	19456786	19457086	-14578	1.14	4.22E-03	4.73E-01	Distal Intergenic
Bicra	292622	chr1	77915583	77915883	-22074	-1.16	4.24E-03	4.74E-01	Distal Intergenic
Abhd2	293050	chr1	141011383	141011683	13143	1.12	4.34E-03	4.83E-01	Intron (NM_001106275/293050, intron 1 of 10)
Pklr	24651	chr2	188439171	188439471	-10190	1.14	4.35E-03	4.83E-01	Distal Intergenic
Ppp1r14a	114004	chr1	87231685	87231985	3104	1.4	4.37E-03	4.83E-01	Intron (NM_130403/114004, intron 3 of 3)
Zfp112	308420	chr1	80944724	80945024	490	-1.29	4.39E-03	4.83E-01	Promoter (<=1kb)
Zfp112	308420	chr1	80944409	80944709	175	-1.09	4.39E-03	4.83E-01	Promoter (<=1kb)
Sdccag8	305002	chr13	94881208	94881508	-6666	-1.33	4.40E-03	4.83E-01	Distal Intergenic
Klhl40	316088	chr8	130352484	130352784	-63571	-1.28	4.49E-03	4.90E-01	Distal Intergenic
Sh3bp5	117186	chr16	7538877	7539177	-676	0.89	4.50E-03	4.90E-01	Promoter (<=1kb)
Dclk2	310698	chr2	186149102	186149402	96369	-1.19	4.53E-03	4.90E-01	Intron (NM_001009691/310698, intron 6 of 17)
G6pc	25634	chr10	89303956	89304256	17947	-1.41	4.54E-03	4.90E-01	Distal Intergenic
Zw10	363059	chr8	53365049	53365349	-532	-1.23	4.55E-03	4.90E-01	Promoter (<=1kb)
Pxn	360820	chr12	46832970	46833270	11809	-1.2	4.55E-03	4.90E-01	Intron (NM_001012147/360820, intron 1 of 9)
Pnn	368070	chr6	80218347	80218647	58983	-0.98	4.56E-03	4.90E-01	Distal Intergenic
Aldh16a1	361571	chr1	101146476	101146776	4544	1.28	4.57E-03	4.91E-01	Intron (NM_001033706/361571, intron 1 of 16)
Oas1a	192281	chr12	41200762	41201062	33	0.76	4.59E-03	4.91E-01	Promoter (<=1kb)
S100a4	24615	chr2	189996682	189996982	-296	0.82	4.64E-03	4.94E-01	Promoter (<=1kb)
Pelp1	360552	chr10	56995168	56995468	9643	1.68	4.65E-03	4.94E-01	Intron (NM_001024270/360552, intron 5 of 16)
Tcea3	298559	chr5	154600872	154601172	2107	0.99	4.66E-03	4.94E-01	Promoter (2-3kb)
Scaf11	312030	chr7	137676546	137676846	179491	-1.19	4.71E-03	4.98E-01	Distal Intergenic
Polk	171525	chr2	27294174	27294474	70432	1.2	4.76E-03	5.01E-01	Distal Intergenic
B4galnt3	500306	chr4	152834969	152835269	0	0.62	4.77E-03	5.01E-01	Promoter (<=1kb)
Pkm	25630	chr8	64519852	64520152	38678	1.04	4.82E-03	5.04E-01	Distal Intergenic
Ddx39a	89827	chr19	24851734	24852034	4796	1.08	4.83E-03	5.04E-01	Intron (NM_053563/89827, intron 3 of 10)
Unc119b	288702	chr12	47242708	47243008	3045	-1.38	4.84E-03	5.04E-01	Intron (NM_001105934/288702, intron 1 of 4)
Slc38a7	291840	chr19	9624048	9624348	1412	1.74	4.85E-03	5.04E-01	Promoter (1-2kb)
RGD1309106	360864	chr13	80000590	80000890	-101111	-1.05	4.87E-03	5.04E-01	Intron (NM_138538/171574, intron 16 of 20)
Id4	291023	chr17	16692785	16693085	2041	-1.23	4.90E-03	5.06E-01	Promoter (2-3kb)
Npy	24604	chr4	79699788	79700088	125778	1.08	4.91E-03	5.06E-01	Distal Intergenic
Lpar1	116744	chr5	75674978	75675278	1306	-1.28	4.94E-03	5.06E-01	Promoter (1-2kb)
Cd180	294706	chr2	33015480	33015780	195158	-1.41	4.94E-03	5.06E-01	Distal Intergenic
Mir107	1E+08	chr1	253121238	253121538	6798	-1.17	4.99E-03	5.07E-01	Intron (NM_001106373/294088, intron 6 of 6)
Pla2g4a	24653	chr13	66942226	66942526	264162	1.52	5.01E-03	5.07E-01	Distal Intergenic
Lasp1	29278	chr10	85753909	85754209	9247	1.6	5.02E-03	5.07E-01	Intron (NM_032613/29278, intron 2 of 7)
Clcn3	84360	chr16	32475326	32475626	26210	-1.21	5.04E-03	5.07E-01	Intron (NM_053363/84360, intron 2 of 12)
LOC686031	686031	chr13	100276447	100276937	62094	1.75	5.04E-03	5.07E-01	Distal Intergenic
Tmem181	502228	chr1	47162430	47162730	36	0.66	5.04E-03	5.07E-01	Promoter (<=1kb)
Map2	25595	chr9	73254910	73255210	-122850	-1.11	5.04E-03	5.07E-01	Distal Intergenic
Prkci	84006	chr2	116003068	116003368	-518	-0.79	5.12E-03	5.13E-01	Promoter (<=1kb)
Pdlim2	290354	chr15	51854931	51855231	94	0.7	5.23E-03	5.22E-01	Promoter (<=1kb)
Mir297	1E+08	chr10	101685016	101685316	11022	1.53	5.25E-03	5.22E-01	Distal Intergenic
Faap20	362678	chr5	172576954	172577254	-71696	1.58	5.25E-03	5.22E-01	Distal Intergenic
Scd2	83792	chr1	264071434	264071734	12060	1.15	5.26E-03	5.22E-01	3' UTR
LOC100911360	1.01E+08	chr8	73437869	73438169	96826	-1.15	5.29E-03	5.24E-01	Distal Intergenic
LOC100912266	1.01E+08	chr2	25005668	25005968	-1250	1.06	5.33E-03	5.25E-01	Promoter (1-2kb)

Parp1	25591	chr13	98856696	98856996	-259	-0.89	5.34E-03	5.25E-01	Promoter (<=1kb)
Tubb3	246118	chr19	56181280	56181580	-39179	1.43	5.39E-03	5.29E-01	Distal Intergenic
Slc16a1	25027	chr2	207179860	207180160	71308	-1.25	5.46E-03	5.34E-01	Distal Intergenic
Fem1b	315745	chr8	67713139	67713439	-4826	1.76	5.48E-03	5.34E-01	Distal Intergenic
Txk	305311	chr14	38072574	38072874	42262	1.41	5.48E-03	5.34E-01	Intron (NM_001024255/305311, intron 10 of 14)
Ranbp10	361396	chr19	37766362	37766662	28635	1.41	5.51E-03	5.35E-01	Intron (NM_001135875/361396, intron 3 of 13)
Exoc7	64632	chr10	105201240	105201540	-18352	-1.01	5.54E-03	5.36E-01	Distal Intergenic
Gng2	80850	chr15	4660474	4660774	-87468	-1.05	5.59E-03	5.37E-01	Distal Intergenic
Nmt1	259274	chr10	91146985	91147285	20296	1.15	5.59E-03	5.37E-01	Intron (NM_148891/259274, intron 3 of 11)
Thy1	24832	chr8	48291679	48291979	-90142	1.62	5.60E-03	5.37E-01	Distal Intergenic
Ca4	29242	chr10	72236059	72236359	-35927	-1.31	5.60E-03	5.37E-01	Distal Intergenic
Ppfia1	293645	chr1	217718410	217718710	1511	-1.46	5.64E-03	5.38E-01	Promoter (1-2kb)
Ube2ql1	679949	chr1	36245065	36245365	59149	0.74	5.65E-03	5.38E-01	Distal Intergenic
Slc1a1	25550	chr1	246675544	246675844	-279173	-1.09	5.68E-03	5.41E-01	Distal Intergenic
Elf5	366142	chr3	93337902	93338202	-7190	-1.26	5.73E-03	5.43E-01	Distal Intergenic
LOC680254	680254	chr13	75021311	75021611	-38578	1.65	5.76E-03	5.43E-01	Distal Intergenic
Fopnl	360461	chr10	723887	724187	-6060	1.6	5.77E-03	5.43E-01	Distal Intergenic
Gadd45g	291005	chr17	13389610	13389910	3309	-1.18	5.77E-03	5.43E-01	Downstream (1-2kb)
Scfd2	498353	chr14	36219067	36219367	3065	-1.26	5.78E-03	5.43E-01	Intron (NM_001017499/498353, intron 1 of 8)
Ndufb2	362344	chr4	67379911	67380211	1723	-0.92	5.82E-03	5.43E-01	Promoter (1-2kb)
Chmp6	287873	chr10	109017403	109017703	-55975	1.17	5.83E-03	5.43E-01	Exon (NM_001134499/287871, exon 18 of 34)
Afdn	26955	chr1	53905379	53905679	-64123	1.47	5.84E-03	5.43E-01	Distal Intergenic
Gmpr	117533	chr17	19578659	19578959	1970	-1.02	5.84E-03	5.43E-01	Promoter (1-2kb)
Armc7	287827	chr10	104033352	104033652	-2715	1.17	5.91E-03	5.46E-01	Promoter (2-3kb)
Adsl	315150	chr7	122122856	122123156	-34045	1.04	5.91E-03	5.46E-01	Exon (NM_138845/192178, exon 19 of 25)
Plxdc2	361282	chr17	83223112	83223412	1285	1.08	5.92E-03	5.46E-01	Promoter (1-2kb)
Srsf4	362612	chr5	150114188	150114488	81189	1.42	5.95E-03	5.47E-01	Distal Intergenic
Plpp7	296635	chr3	11129216	11129516	14665	1.23	5.99E-03	5.49E-01	Exon (NM_001012349/296635, exon 2 of 2)
Slc44a2	363024	chr8	22366101	22366401	-2344	-1.28	6.09E-03	5.56E-01	Promoter (2-3kb)
Taok3	304530	chr12	45200729	45201029	-17755	1.52	6.11E-03	5.56E-01	Distal Intergenic
Mir671	1E+08	chr4	7101808	7102108	6719	1.01	6.14E-03	5.56E-01	Exon (NM_001011966/296732, exon 3 of 14)
Dbnl	83527	chr14	86013209	86013509	-15826	1.06	6.14E-03	5.56E-01	Distal Intergenic
Mtss2	307845	chr19	40922928	40923228	2432	-1.25	6.15E-03	5.56E-01	Promoter (2-3kb)
Lmx1a	289201	chr13	86028198	86028498	109946	0.95	6.18E-03	5.56E-01	Intron (NM_001105967/289201, intron 3 of 7)
Pigk	295543	chr2	258110571	258110871	199445	-1	6.18E-03	5.56E-01	Intron (NM_001271361/365984, intron 2 of 4)
Rgma	308739	chr1	134699408	134699708	355	0.85	6.20E-03	5.56E-01	Promoter (<=1kb)
Clul1	367345	chr9	122079858	122080158	-107803	1.15	6.21E-03	5.56E-01	Distal Intergenic
Evc2	289711	chr14	78211594	78211894	37	0.83	6.22E-03	5.56E-01	Promoter (<=1kb)
Tjap1	316233	chr9	17092431	17092731	5956	1.68	6.22E-03	5.56E-01	Intron (NM_001108203/316233, intron 2 of 11)
Psmb1	94198	chr1	57486932	57487232	2473	-1.49	6.24E-03	5.56E-01	Promoter (2-3kb)
Mx2	286918	chr11	38035857	38036157	344	1.07	6.29E-03	5.59E-01	Promoter (<=1kb)
Krt4	315323	chr7	143537058	143537358	-13601	1.17	6.35E-03	5.60E-01	Distal Intergenic
Cenpc	305270	chr14	23610854	23611154	-755	-0.79	6.35E-03	5.60E-01	Promoter (<=1kb)
Tex26	498133	chr12	6589764	6590064	113640	0.76	6.38E-03	5.60E-01	Distal Intergenic
Hdac5	84580	chr10	90172410	90172710	-2560	-1.11	6.39E-03	5.60E-01	Promoter (2-3kb)
Setd6	291844	chr19	9750764	9751064	14420	1.06	6.39E-03	5.60E-01	Intron (NM_001134840/291841, intron 39 of 48)
Ndfip1	291609	chr18	31567113	31567413	-7408	-1.72	6.39E-03	5.60E-01	Distal Intergenic
Fkbp1b	58950	chr6	29974002	29974302	-3591	-1.16	6.42E-03	5.61E-01	Distal Intergenic
Bcl2l14	500348	chr4	168146414	168146714	6406	1.03	6.43E-03	5.61E-01	Intron (NM_001024338/500348, intron 2 of 6)
Oxr1	117520	chr7	80750782	80751082	173	-0.69	6.49E-03	5.65E-01	Promoter (<=1kb)
Thoc3	290519	chr17	10661467	10661767	-15176	1.4	6.53E-03	5.67E-01	Distal Intergenic
Rpp40	291071	chr17	29567747	29568047	-71032	0.76	6.54E-03	5.67E-01	Distal Intergenic
Myl12b	50685	chr9	119326696	119326996	-5196	1.33	6.59E-03	5.67E-01	Intron (NM_001135017/501203, intron 3 of 3)
Actn4	63836	chr1	87132740	87133040	14268	-0.98	6.60E-03	5.67E-01	Intron (NM_031675/63836, intron 1 of 20)
Nanos2	365213	chr1	79870053	79870353	-24097	1.2	6.60E-03	5.67E-01	Distal Intergenic
Ube3a	361585	chr1	117305975	117306275	718160	-0.99	6.61E-03	5.67E-01	Distal Intergenic
Mageb16	317274	chrX	46902917	46903217	120885	-1	6.67E-03	5.69E-01	Distal Intergenic
Dimt1	294718	chr2	38133731	38134031	13145	-1.03	6.68E-03	5.69E-01	Exon (NM_001106408/294718, exon 5 of 14)
Cndp2	291394	chr18	81579942	81580242	-40877	-0.75	6.68E-03	5.69E-01	Distal Intergenic
Macf1	362587	chr5	141367197	141367497	-3673	0.83	6.73E-03	5.69E-01	Distal Intergenic
Wdr25	314443	chr6	132812190	132812490	6396	-1.14	6.73E-03	5.69E-01	Intron (NM_001135894/314443, intron 2 of 6)
Sgpl1	286896	chr20	30748913	30749213	727	1.76	6.76E-03	5.69E-01	Promoter (<=1kb)
Cmtr2	292016	chr19	41379278	41379578	53789	-0.99	6.76E-03	5.69E-01	Distal Intergenic
Aif1l	362107	chr3	9262241	9262541	87	0.81	6.77E-03	5.69E-01	Promoter (<=1kb)
Mocs2	294753	chr2	47025440	47025740	44464	-1.29	6.78E-03	5.69E-01	Distal Intergenic
Stag3	114522	chr12	19644537	19644837	44577	-0.82	6.79E-03	5.69E-01	Distal Intergenic
Slc39a10	363229	chr9	59808547	59808847	-212687	-1	6.83E-03	5.71E-01	Distal Intergenic
Trim71	301042	chr8	122626761	122627061	-21974	1.19	6.86E-03	5.73E-01	Distal Intergenic
Mir17	1E+08	chr15	100179806	100180106	0	1.11	6.91E-03	5.75E-01	Promoter (<=1kb)
Tex44	501180	chr9	93492897	93493197	47895	1.21	6.93E-03	5.75E-01	Distal Intergenic
Arhgap31	288093	chr11	64660034	64660334	59066	1.31	6.93E-03	5.75E-01	Intron (NM_001105879/288093, intron 1 of 11)
Med17	300367	chr8	13837020	13837320	-1718	-1.01	7.01E-03	5.79E-01	Promoter (1-2kb)
Ttc1	287208	chr10	29384478	29384778	-16613	-1.25	7.03E-03	5.79E-01	Distal Intergenic
RGD1304884	307907	chr19	53768080	53768380	44258	1.42	7.03E-03	5.79E-01	Distal Intergenic
Ifitm1	293618	chr1	213765821	213766121	0	1.38	7.19E-03	5.89E-01	Promoter (<=1kb)
Efna4	310643	chr2	188637756	188638056	22123	0.99	7.20E-03	5.89E-01	Distal Intergenic
Dcps	266605	chr8	36374896	36375196	-231	-0.56	7.20E-03	5.89E-01	Promoter (<=1kb)
Mtmr3	305482	chr14	84817084	84817384	3031	-1.21	7.27E-03	5.94E-01	Intron (NM_001012038/305482, intron 1 of 18)
Fads1	84575	chr1	226233358	226233658	-455	1.28	7.30E-03	5.94E-01	Promoter (<=1kb)
Ptpro	50677	chr4	171150343	171150643	-100600	0.99	7.32E-03	5.94E-01	Distal Intergenic
Ndufb4	288088	chr11	65952868	65953168	-7109	-1.02	7.32E-03	5.94E-01	Distal Intergenic
Cd180	294706	chr2	33021672	33021972	201350	1.17	7.42E-03	6.00E-01	Distal Intergenic
Ptprf	360406	chr5	137137623	137137923	-32630	-1.16	7.46E-03	6.02E-01	Distal Intergenic
Mir145	1E+08	chr18	56961100	56961400	8594	1.19	7.51E-03	6.05E-01	Distal Intergenic
LOC689766	689766	chr13	95846279	95846579	-41129	1.35	7.57E-03	6.08E-01	Distal Intergenic
Hbp1	27080	chr6	51256269	51256569	1056	-0.59	7.60E-03	6.09E-01	Promoter (1-2kb)
S100a10	81778	chr2	193904098	193904398	11509	-1.16	7.62E-03	6.09E-01	Distal Intergenic
Mir30c1	1E+08	chr5	139661386	139661686	38279	1.33	7.62E-03	6.09E-01	Distal Intergenic
Slc39a11	287796	chr10	102080947	102081247	2233	-1.28	7.65E-03	6.10E-01	Promoter (2-3kb)
Unc5b	60630	chr20	30380356	30380656	25541	1.17	7.73E-03	6.15E-01	Intron (NM_022207/60630, intron 2 of 17)
Eif3el1	299872	chr13	100135226	100135526	5469	-1.26	7.78E-03	6.18E-01	Intron (NM_001011990/299872, intron 2 of 12)
Cc2d1a	288908	chr19	25263950	25264250	-2039	1.12	7.82E-03	6.19E-01	Promoter (2-3kb)
Sesn3	315427	chr8	12779138	12779438	-43717	1.48	7.88E-03	6.22E-01	Distal Intergenic
Foxp1	297480	chr4	131636567	131636867	-38263	0.88	7.89E-03	6.22E-01	Distal Intergenic

Rpn1	25596	chr4	120021120	120021420	23888	-1.01	7.91E-03	6.22E-01	Distal Intergenic
Twist1	85489	chr6	53504281	53504581	103040	-1.29	7.96E-03	6.23E-01	Intron (NM_001200045/687001, intron 25 of 25)
Ifi47	246208	chr10	34278398	34278698	405	1.11	7.98E-03	6.23E-01	Promoter (<=1kb)
Cox4i2	84683	chr3	148268429	148268729	33883	-1.02	7.99E-03	6.23E-01	Intron (NM_001033671/24888, intron 1 of 1)
Pds5a	305343	chr14	44113400	44113700	-71779	1.94	8.00E-03	6.23E-01	Distal Intergenic
Marchf10	303596	chr10	93719785	93720085	-39811	-0.98	8.00E-03	6.23E-01	Distal Intergenic
Cav1	25404	chr4	44678176	44678476	79619	1.99	8.05E-03	6.25E-01	Distal Intergenic
Pnma3	293840	chrX	153054893	153055193	-8835	-0.92	8.09E-03	6.27E-01	Distal Intergenic
Plec	64204	chr7	117248683	117248983	5782	1.43	8.14E-03	6.29E-01	Intron (NM_001164308/64204, intron 12 of 31)
LOC290876	290876	chr16	81299692	81299992	13984	1.34	8.16E-03	6.29E-01	Downstream (1-2kb)
Usp25	304150	chr11	15383780	15384080	-52189	-0.99	8.20E-03	6.29E-01	Distal Intergenic
Ide	25700	chr1	256011444	256011744	1751	-1.09	8.23E-03	6.29E-01	Promoter (1-2kb)
Ifrd2	300994	chr8	116350314	116350614	7218	-0.91	8.25E-03	6.29E-01	Distal Intergenic
LOC100911367	1.01E+08	chr18	31793492	31793792	989	0.84	8.29E-03	6.29E-01	Promoter (<=1kb)
LOC689840	689840	chr8	14746485	14746785	-64842	0.93	8.33E-03	6.29E-01	Intron (NM_138544/191571, intron 3 of 26)
Paqr7	313615	chr5	152727725	152728025	9571	1.25	8.34E-03	6.29E-01	Distal Intergenic
Kif2a	84391	chr2	38431116	38431416	-222351	-1.44	8.35E-03	6.29E-01	Distal Intergenic
Rcc1	682908	chr5	150573683	150573983	-48429	1.32	8.36E-03	6.29E-01	Distal Intergenic
Camk2d	24246	chr2	231331172	231331472	430046	1.65	8.36E-03	6.29E-01	Distal Intergenic
Bmyc	311807	chr3	2916883	2917183	221	0.59	8.37E-03	6.29E-01	Promoter (<=1kb)
Larp4b	307070	chr17	63207999	63208299	-27708	-1.07	8.38E-03	6.29E-01	Distal Intergenic
Chst11	314694	chr7	26813511	26813811	45905	-1.01	8.39E-03	6.29E-01	Intron (NM_001108079/314694, intron 1 of 2)
Zdhhc16	654495	chr1	261186264	261186564	6256	2.13	8.39E-03	6.29E-01	Exon (NM_001039346/654495, exon 6 of 11)
Muc19	497227	chr7	133195110	133195410	146383	-1.29	8.40E-03	6.29E-01	Distal Intergenic
Syngap1	192117	chr20	5537918	5538218	2484	-1.17	8.41E-03	6.29E-01	Promoter (2-3kb)
Gas8	361438	chr19	56355368	56355668	38924	0.78	8.45E-03	6.31E-01	Distal Intergenic
Gucy2g	245708	chr1	276205247	276205547	23027	0.91	8.50E-03	6.32E-01	Intron (NM_139042/245708, intron 12 of 20)
Kcnn4	65206	chr1	81240448	81240748	9601	-1.21	8.50E-03	6.32E-01	Intron (NM_001270701/65206, intron 4 of 8)
Klhl25	293023	chr1	137425793	137426093	-66721	-0.61	8.52E-03	6.33E-01	Distal Intergenic
Mir6215	1.02E+08	chr5	158061229	158061529	-169	1.46	8.60E-03	6.34E-01	Promoter (<=1kb)
Fbxo39	303287	chr10	58862736	58863036	-12795	1.44	8.60E-03	6.34E-01	Distal Intergenic
Hnrnpa2b1	362361	chr4	81239918	81240218	1063	1.42	8.62E-03	6.34E-01	Promoter (1-2kb)
RGD1563365	299700	chr7	26484990	26485290	-6801	-1.15	8.63E-03	6.34E-01	Distal Intergenic
Stim2	117087	chr14	59360538	59360838	-36591	1.49	8.65E-03	6.34E-01	Distal Intergenic
Dok1	312477	chr4	113842340	113842640	24034	-1.04	8.67E-03	6.34E-01	Distal Intergenic
Agtrap	298646	chr5	164872375	164872675	25712	-0.93	8.69E-03	6.34E-01	Distal Intergenic
Sucla2	361071	chr15	55586759	55587059	125064	-1.19	8.69E-03	6.34E-01	Distal Intergenic
Thumpd2	313851	chr6	3792670	3792970	1531	-1.56	8.71E-03	6.34E-01	Promoter (1-2kb)
Impa1	83523	chr2	93674638	93674938	-265	-0.98	8.72E-03	6.34E-01	Promoter (<=1kb)
Ino80c	291737	chr18	15985073	15985373	24266	1.4	8.73E-03	6.34E-01	Distal Intergenic
Epha7	171287	chr5	43621862	43622162	18959	-1.1	8.84E-03	6.40E-01	Intron (NM_134331/171287, intron 3 of 16)
Samd5	365038	chr1	3762664	3762964	428	0.64	8.85E-03	6.40E-01	Promoter (<=1kb)
Pear1	295293	chr2	187133902	187134202	0	1.21	8.89E-03	6.41E-01	Promoter (<=1kb)
Farsb	301544	chr9	84349669	84349969	33669	-0.9	8.90E-03	6.41E-01	Intron (NM_001004252/301544, intron 15 of 16)
Smarce1	303518	chr10	87108423	87108723	29236	0.81	8.91E-03	6.41E-01	Distal Intergenic
Ptk2b	50646	chr15	42888202	42888502	59154	1.26	8.95E-03	6.42E-01	Intron (NM_017318/50646, intron 1 of 30)
Aspm	289054	chr13	56548372	56548672	2351	-1.2	8.97E-03	6.42E-01	Promoter (2-3kb)
Camkmt	299521	chr6	8431151	8431451	84506	1.2	8.98E-03	6.42E-01	Intron (NM_001134463/299521, intron 3 of 10)
Neurl1	309459	chr1	267078174	267078474	125035	1.29	9.01E-03	6.43E-01	Intron (NM_001107606/309460, intron 7 of 13)
Fcgr1a	295279	chr2	198436435	198436735	2718	0.95	9.08E-03	6.45E-01	Promoter (2-3kb)
Washc3	299707	chr7	28762011	28762311	-106	1.41	9.10E-03	6.45E-01	Promoter (<=1kb)
Cpne2	498972	chr19	10934789	10935089	-50742	-0.93	9.11E-03	6.45E-01	Distal Intergenic
Hmgb3	305373	chr14	51462164	51462464	257	1.9	9.11E-03	6.45E-01	Promoter (<=1kb)
Dysf	312492	chr4	115957180	115957480	244176	1.04	9.17E-03	6.47E-01	Distal Intergenic
Dpysl2	25416	chr15	43519943	43520243	22650	0.84	9.17E-03	6.47E-01	Intron (NM_001105717/25416, intron 3 of 13)
Ilkap	64538	chr9	98505982	98506282	30304	-1.12	9.24E-03	6.50E-01	Distal Intergenic
Cdyl	361237	chr17	30085723	30086023	-190337	1.44	9.31E-03	6.54E-01	Distal Intergenic
Ccn5	29576	chr3	160197078	160197378	-10591	1.07	9.38E-03	6.58E-01	Distal Intergenic
Nostrin	311111	chr3	55119136	55119436	-249846	1.29	9.47E-03	6.61E-01	Distal Intergenic
Tmpo	25359	chr7	31978576	31978876	-106205	1.41	9.48E-03	6.61E-01	Distal Intergenic
Nr2f2	113984	chr1	131944106	131944406	-489417	-0.64	9.51E-03	6.61E-01	Distal Intergenic
RGD1309748	302913	chr10	6862362	6862662	7349	-0.95	9.52E-03	6.61E-01	Intron (NM_001106972/302913, intron 3 of 4)
Jmjd1c	171120	chr20	22822736	22823036	59636	1.06	9.53E-03	6.61E-01	Intron (NM_001191719/171120, intron 2 of 25)
Iqca1l	499971	chr4	7149262	7149562	4588	2.01	9.54E-03	6.61E-01	Exon (NM_001024317/499971, exon 6 of 19)
Snx27	260323	chr2	195818421	195818721	2887	-1.15	9.55E-03	6.61E-01	Promoter (2-3kb)
Ndfip2	361089	chr15	89769955	89770255	362529	-0.99	9.56E-03	6.61E-01	Distal Intergenic
Kbtbd8	500262	chr4	127164207	127164507	-386	-1.02	9.64E-03	6.65E-01	Promoter (<=1kb)
Cdk19	309804	chr20	45462804	45463104	4246	0.99	9.67E-03	6.65E-01	Intron (NM_001107634/309804, intron 1 of 12)
Vcl	305679	chr15	3518018	3518318	26367	-1.07	9.68E-03	6.65E-01	Intron (NM_001107248/305679, intron 2 of 20)
Snx6	362738	chr6	75668376	75668676	1459	-1.08	9.69E-03	6.65E-01	Promoter (1-2kb)
Pdp2	246311	chr19	586202	586502	14967	-1.1	9.84E-03	6.68E-01	Distal Intergenic
Mrpl49	309176	chr1	221417265	221417565	2542	1.39	9.85E-03	6.68E-01	Promoter (2-3kb)
Utp15	310019	chr2	28301646	28301946	68315	1.34	9.87E-03	6.68E-01	Distal Intergenic
Nedd4	25489	chr8	79330233	79330533	6825	-1.01	9.88E-03	6.68E-01	Exon (NM_012986/25489, exon 2 of 29)
Zfat	362925	chr7	109054782	109055082	150287	0.99	9.90E-03	6.68E-01	Exon (NM_001134957/362925, exon 15 of 16)
Zfp7	315101	chr7	118457240	118457540	-30958	-0.93	9.91E-03	6.68E-01	Distal Intergenic
RGD1309139	362020	chr2	211279953	211280253	-40167	1.32	9.91E-03	6.68E-01	Distal Intergenic
Fign	295649	chr3	50120020	50120320	72	-0.88	9.94E-03	6.68E-01	Promoter (<=1kb)
Bbc3	317673	chr1	78249672	78249972	-11533	1.55	9.94E-03	6.68E-01	Distal Intergenic
Adam34	685846	chr16	51280035	51280335	-470455	1.19	9.95E-03	6.68E-01	Distal Intergenic
Trpv2	29465	chr10	48897171	48897471	-6069	1.52	9.95E-03	6.68E-01	Distal Intergenic
Mfap5	362429	chr4	155301758	155302058	-11613	-1.18	9.95E-03	6.68E-01	Distal Intergenic
Trim3	83616	chr1	170452811	170453111	11230	1.18	9.99E-03	6.69E-01	Intron (NM_031786/83616, intron 2 of 12)
Acsl1	25288	chr16	49047726	49048026	-65746	1.1	1.01E-02	6.72E-01	Distal Intergenic
Hsd17b4	79244	chr18	44811775	44812075	1313	-1.21	1.01E-02	6.72E-01	Promoter (1-2kb)
Rpp38	291317	chr17	78917609	78917909	2005	-1.1	1.01E-02	6.72E-01	Promoter (2-3kb)
Sptbn1	305614	chr14	114619160	114619460	73304	-1.17	1.01E-02	6.72E-01	Intron (NM_001013130/305614, intron 2 of 36)
RGD1562885	502412	chr20	3704314	3704614	-13150	0.81	1.01E-02	6.72E-01	Distal Intergenic
Acvr2b	25366	chr8	128110020	128110320	22675	1.08	1.01E-02	6.72E-01	Intron (NM_031554/25366, intron 1 of 10)
Mir3596a	1.01E+08	chr8	45759095	45759395	-5728	-1.01	1.02E-02	6.72E-01	Intron (NR_126581/104845260, intron 3 of 3)
Ubap1	362502	chr5	57720381	57720681	-17640	0.94	1.02E-02	6.72E-01	Distal Intergenic
Pbdc1	363485	chrX	76034562	76034862	-7377	-0.95	1.02E-02	6.72E-01	Distal Intergenic
C2cd2	304055	chr11	38397054	38397354	22678	-1.01	1.03E-02	6.74E-01	Intron (NM_199391/304055, intron 2 of 13)
Zfyve28	305454	chr14	81724084	81724384	-1129	-1.57	1.03E-02	6.75E-01	Promoter (1-2kb)

Kin	689197	chr17	72159507	72159807	49425	1.67	1.03E-02	6.77E-01	Distal Intergenic
Usp14	291796	chr18	1217150	1217450	-771	-0.99	1.04E-02	6.77E-01	Promoter (<=1kb)
Snx7	310815	chr2	220913836	220914136	185602	-1.07	1.04E-02	6.77E-01	Distal Intergenic
Stat6	362896	chr7	70938051	70938351	-7877	-1.12	1.04E-02	6.77E-01	Distal Intergenic
Mybphl	310782	chr2	211166434	211166734	7151	1.39	1.04E-02	6.77E-01	Intron (NM_001014042/310782, intron 1 of 8)
Rtf1	366169	chr3	111477844	111478144	-238	-0.59	1.04E-02	6.77E-01	Promoter (<=1kb)
Armc8	315949	chr8	107807430	107807730	11320	-0.82	1.05E-02	6.77E-01	Intron (NM_001173354/315949, intron 1 of 21)
Niban1	63912	chr13	68965318	68965618	15653	1.2	1.05E-02	6.77E-01	Intron (NM_022242/63912, intron 1 of 13)
Twist1	85489	chr6	53404278	53404578	3037	-1.07	1.05E-02	6.78E-01	Distal Intergenic
Stard13	498130	chr12	1193537	1193837	1742	-0.67	1.05E-02	6.78E-01	Promoter (1-2kb)
Col3a1	84032	chr9	52022903	52023203	-92	-1.17	1.05E-02	6.78E-01	Promoter (<=1kb)
Dhrs4	266686	chr15	34155136	34155436	-68	-1.18	1.06E-02	6.79E-01	Promoter (<=1kb)
Bik	114496	chr7	124391469	124391769	37	0.56	1.06E-02	6.79E-01	Promoter (<=1kb)
F7	260320	chr16	81870764	81871064	-35841	0.87	1.06E-02	6.80E-01	Exon (NM_053951/117020, exon 5 of 31)
Mr1	25119	chr13	72788140	72788440	1298	-0.87	1.07E-02	6.82E-01	Promoter (1-2kb)
Mbd1	291439	chr18	70247813	70248113	-600	-0.6	1.07E-02	6.82E-01	Promoter (<=1kb)
Ccdc184	500925	chr7	139770093	139770393	7479	1.49	1.07E-02	6.84E-01	Distal Intergenic
Mir568	1E+08	chr11	62115355	62115655	-119233	1.51	1.08E-02	6.84E-01	Intron (NM_001105880/288105, intron 5 of 10)
Tns1	301509	chr9	81368357	81368657	32330	1.14	1.08E-02	6.84E-01	Intron (NM_001191810/301509, intron 1 of 34)
Adamts15	300474	chr8	32018172	32018472	-17794	0.75	1.08E-02	6.84E-01	Distal Intergenic
S100a10	81778	chr2	193933305	193933605	40716	1.14	1.08E-02	6.86E-01	Distal Intergenic
Scaf11	312030	chr7	137853544	137853844	2493	-1.07	1.09E-02	6.86E-01	Promoter (2-3kb)
Sspn	500364	chr4	180231253	180231553	-59836	-0.82	1.09E-02	6.86E-01	Distal Intergenic
Tmem212	499586	chr2	113580117	113580417	36349	0.97	1.09E-02	6.87E-01	Distal Intergenic
Mepe	79110	chr14	6779447	6779747	13814	-0.9	1.09E-02	6.87E-01	Downstream (2-3kb)
Smim3	286910	chr18	55752423	55752723	19007	-0.92	1.10E-02	6.87E-01	Intron (NM_173126/286910, intron 1 of 1)
Sh2b2	114203	chr12	23344482	23344782	-128488	1.37	1.10E-02	6.89E-01	Distal Intergenic
Ugcg	83626	chr5	76419997	76420297	33159	1.11	1.10E-02	6.89E-01	Distal Intergenic
Scarb1	25073	chr12	36766995	36767295	72038	0.98	1.10E-02	6.89E-01	Distal Intergenic
Trim41	303088	chr10	34171252	34171552	-4653	1.48	1.11E-02	6.89E-01	Distal Intergenic
Tmem179	314472	chr6	137143918	137144218	-59179	0.94	1.11E-02	6.92E-01	Distal Intergenic
Ptpn14	305064	chr13	108755058	108755358	86124	1.07	1.12E-02	6.94E-01	Intron (NM_001107200/305064, intron 3 of 17)
Gpr84	688730	chr7	144943744	144944044	-6941	1.58	1.12E-02	6.94E-01	Exon (NM_001135088/685474, exon 4 of 8)
Nsmaf	353233	chr5	19659461	19659761	-100217	-0.99	1.13E-02	6.99E-01	Distal Intergenic
Gadd45g	291005	chr17	13187747	13188047	205172	1.15	1.13E-02	6.99E-01	Distal Intergenic
Cntnap5b	301650	chr13	20404792	20405092	-1273420	1.04	1.13E-02	6.99E-01	Distal Intergenic
Tbc1d2b	315880	chr8	97635293	97635593	11479	0.78	1.14E-02	6.99E-01	Intron (NM_001108175/315880, intron 2 of 13)
Chrna1	79557	chr3	60387464	60387764	72960	0.94	1.14E-02	6.99E-01	Distal Intergenic
Arhgap22	306279	chr16	9454356	9454656	-108562	1.02	1.14E-02	6.99E-01	Distal Intergenic
Csf1	78965	chr2	210550068	210550368	178	0.53	1.15E-02	6.99E-01	Promoter (<=1kb)
Thumpd2	313851	chr6	3806980	3807280	-12479	0.89	1.15E-02	6.99E-01	Distal Intergenic
Gpr31	292310	chr1	53468921	53469221	51567	1.11	1.15E-02	6.99E-01	Intron (NM_057128/117269, intron 11 of 20)
Top2b	361100	chr15	10343555	10343855	-23814	1.09	1.15E-02	6.99E-01	Distal Intergenic
Arfgap3	503165	chr7	124191393	124191693	6570	1.77	1.15E-02	6.99E-01	Intron (NM_001044273/503165, intron 1 of 19)
Swt1	289088	chr13	68783659	68783959	1812	-1.16	1.15E-02	7.01E-01	Promoter (1-2kb)
Pax3	114502	chr9	83692465	83692765	408407	1.19	1.16E-02	7.01E-01	Distal Intergenic
Oat	64313	chr1	204578397	204578697	3373	-1.13	1.17E-02	7.06E-01	Intron (NM_022521/64313, intron 1 of 9)
Zbtb7b	295248	chr2	188712484	188712784	-5504	1.49	1.17E-02	7.06E-01	Distal Intergenic
Ednra	24326	chr19	33814023	33814323	-114033	0.95	1.17E-02	7.06E-01	Distal Intergenic
Fn1	25661	chr9	79238769	79239069	-269756	1.18	1.17E-02	7.06E-01	Distal Intergenic
Sgsm3	362963	chr7	122212734	122213034	9202	1.11	1.18E-02	7.11E-01	Intron (NM_198787/362963, intron 1 of 21)
Synpo	60324	chr18	55883522	55883822	7888	-1.06	1.18E-02	7.11E-01	Intron (NM_021695/60324, intron 1 of 1)
Nbl1	50594	chr5	157536757	157537057	-1056	-1.33	1.19E-02	7.12E-01	Promoter (1-2kb)
Elk3	362871	chr7	34087236	34087536	34158	1.49	1.19E-02	7.12E-01	3' UTR
Stx5	65134	chr1	224938881	224939181	-316	-0.69	1.20E-02	7.17E-01	Promoter (<=1kb)
Adam9	290834	chr16	71841376	71841676	30999	-1.17	1.20E-02	7.17E-01	Intron (NM_001014772/290834, intron 11 of 22)
Tgfb3	25717	chr6	109820723	109821023	114510	-0.9	1.20E-02	7.17E-01	Distal Intergenic
Mir29b1	1E+08	chr4	58394394	58394694	-50004	-0.54	1.21E-02	7.17E-01	Distal Intergenic
Nop10	691534	chr3	103747702	103748002	48	-0.52	1.21E-02	7.17E-01	Promoter (<=1kb)
Zfp524	365179	chr1	72371745	72372045	5389	-1.03	1.21E-02	7.18E-01	Distal Intergenic
Sos2	85384	chr6	91866947	91867247	140670	1.07	1.22E-02	7.21E-01	Distal Intergenic
Pde4b	24626	chr5	122105150	122105450	345914	-1.09	1.22E-02	7.21E-01	Intron (NM_017031/24626, intron 6 of 14)
Oxsm	289934	chr15	10460007	10460307	-2442	-1.22	1.23E-02	7.23E-01	Promoter (2-3kb)
Cntln	679640	chr5	103009962	103010262	-241724	-1.49	1.23E-02	7.23E-01	Distal Intergenic
Mid1ip1	404280	chrX	13119629	13119929	-2886	0.93	1.23E-02	7.23E-01	Promoter (2-3kb)
Aars	292023	chr19	43183790	43184090	31191	1.94	1.23E-02	7.23E-01	Distal Intergenic
Tcta	306587	chr8	117086904	117087204	-4742	1.5	1.24E-02	7.25E-01	Distal Intergenic
Tns1	301509	chr9	81374258	81374558	26429	1	1.24E-02	7.25E-01	Intron (NM_001191810/301509, intron 1 of 34)
Rgs3	54293	chr5	78539016	78539316	55123	0.53	1.25E-02	7.25E-01	Intron (NM_019340/54293, intron 10 of 15)
Mir344b-1	1.01E+08	chr1	119834183	119834483	1358752	-1.02	1.25E-02	7.25E-01	Distal Intergenic
Snrnp200	296126	chr3	119639770	119640070	-254	-0.58	1.25E-02	7.25E-01	Promoter (<=1kb)
Cmpk1	298410	chr5	133771998	133772298	14154	-1.18	1.25E-02	7.25E-01	Intron (NM_001025655/298410, intron 1 of 5)
Dctpp1	192252	chr1	198703955	198704255	2597	-1.09	1.25E-02	7.25E-01	Promoter (2-3kb)
Stard10	293150	chr1	166458068	166458368	24959	-1.01	1.25E-02	7.25E-01	Distal Intergenic
Ablim3	307395	chr18	57259765	57260065	-14066	0.9	1.26E-02	7.25E-01	Distal Intergenic
P2ry10	317219	chrX	78222560	78222860	26260	1.39	1.26E-02	7.25E-01	Distal Intergenic
Olr1666	291929	chr19	27623096	27623396	-87304	-0.86	1.26E-02	7.25E-01	Distal Intergenic
Elavl2	286973	chr5	109625998	109626298	-199	-0.61	1.26E-02	7.25E-01	Promoter (<=1kb)
Nfic	29228	chr7	11169616	11169916	17578	0.88	1.26E-02	7.25E-01	Intron (NM_031567/29228, intron 3 of 9)
Lamc1	117036	chr13	70753066	70753366	30149	-1.08	1.26E-02	7.25E-01	Intron (NM_053966/117036, intron 1 of 27)
Dennd6a	306229	chr16	2283904	2284204	5203	1.11	1.27E-02	7.25E-01	Intron (NM_001134467/306229, intron 1 of 19)
Tefm	287554	chr10	67480374	67480674	-1547	-1.43	1.27E-02	7.26E-01	Promoter (1-2kb)
Npas2	316351	chr9	45949531	45949831	47790	1.49	1.27E-02	7.26E-01	Intron (NM_001108214/316351, intron 1 of 20)
Edem3	289085	chr13	69279051	69279351	143522	-0.96	1.27E-02	7.26E-01	Intron (NM_001105959/289084, intron 5 of 5)
Pgk1	24644	chrX	77204943	77205243	-58156	-1.16	1.28E-02	7.29E-01	Distal Intergenic
Pacsin2	124461	chr7	124298796	124299096	-12318	0.92	1.28E-02	7.29E-01	Distal Intergenic
Dync1h1	29489	chr6	134964451	134964751	5597	-1.21	1.28E-02	7.29E-01	Intron (NM_019226/29489, intron 1 of 80)
Mms22l	313108	chr5	38848897	38849197	4351	-1.37	1.29E-02	7.29E-01	Intron (NM_001135780/313108, intron 3 of 23)
Farp1	306183	chr15	106378800	106379100	-55077	1.29	1.29E-02	7.29E-01	Distal Intergenic
Wnt11	140584	chr1	163797359	163797659	-1	1.09	1.29E-02	7.30E-01	Promoter (<=1kb)
Cp	24268	chr2	104744939	104745239	496	-0.96	1.30E-02	7.31E-01	Promoter (<=1kb)
Msln	60333	chr10	15124845	15125145	243	0.92	1.30E-02	7.32E-01	Promoter (<=1kb)
Nsl1	498310	chr13	109671562	109671862	1882	-0.96	1.30E-02	7.32E-01	Promoter (1-2kb)
Tmem43	362401	chr4	123128291	123128591	9820	1.43	1.31E-02	7.32E-01	Intron (NM_001007745/362401, intron 10 of 11)

Bora	306102	chr15	83272169	83272469	-169892	-1.06	1.31E-02	7.32E-01	Distal Intergenic
Ndst4	362035	chr2	229172603	229172903	-23713	-1.09	1.31E-02	7.32E-01	Distal Intergenic
Pdlim1	54133	chr1	259354059	259354359	2647	1.22	1.31E-02	7.33E-01	Promoter (2-3kb)
Ndst4	362035	chr2	229173289	229173589	-23027	-1.05	1.32E-02	7.33E-01	Distal Intergenic
Psma8	364814	chr18	6206243	6206543	91486	-0.75	1.32E-02	7.33E-01	Distal Intergenic
Mrm3	360569	chr10	64471090	64471390	72751	-0.97	1.32E-02	7.33E-01	Intron (NM_001108285/360577, intron 1 of 7)
Acot9	302640	chrX	43592538	43592838	-338	-0.9	1.32E-02	7.33E-01	Promoter (<=1kb)
Rap1gap	313644	chr5	156053275	156053575	26376	1.56	1.32E-02	7.33E-01	Intron (NM_001100713/313644, intron 3 of 25)
Nrn1	83834	chr17	28071657	28071957	-102231	1.09	1.33E-02	7.35E-01	Distal Intergenic
Manba	310864	chr2	240676326	240676626	8113	1.06	1.33E-02	7.35E-01	Intron (NM_001031655/310864, intron 1 of 16)
Mest	58827	chr4	58052757	58053057	0	0.67	1.33E-02	7.36E-01	Promoter (<=1kb)
Celf2	29428	chr17	75053647	75053947	-205322	-1.04	1.34E-02	7.38E-01	Distal Intergenic
Hax1	291202	chr2	189334729	189335029	-1424	-1.17	1.35E-02	7.41E-01	Promoter (1-2kb)
Capn12	308476	chr1	87105939	87106239	39650	1.68	1.35E-02	7.41E-01	Intron (NM_031675/63836, intron 1 of 20)
Cfap126	498278	chr13	89450039	89450339	-29719	0.51	1.35E-02	7.41E-01	Distal Intergenic
Metrn	287151	chr10	15165160	15165460	997	-0.98	1.35E-02	7.41E-01	Promoter (<=1kb)
Nqo1	24314	chr19	38419679	38419979	-2231	-1.07	1.36E-02	7.43E-01	Promoter (2-3kb)
Susd4	289335	chr13	101182101	101182401	95	-1.13	1.36E-02	7.43E-01	Promoter (<=1kb)
Slc1a4	305540	chr14	104633790	104634090	-21373	1.16	1.36E-02	7.43E-01	Distal Intergenic
Taf3	1E+08	chr17	72332777	72333077	91925	-1.2	1.36E-02	7.43E-01	Intron (NM_001271342/100360100, intron 2 of 7)
Angpt2	89805	chr16	75933987	75934287	-32193	-1.19	1.37E-02	7.43E-01	Distal Intergenic
Smim8	297971	chr5	50237852	50238152	1285	1.33	1.37E-02	7.43E-01	Promoter (1-2kb)
Snrk	170837	chr8	130749473	130749773	-12901	1.06	1.37E-02	7.44E-01	Distal Intergenic
Brf2	306542	chr16	69082467	69082767	-7188	1.02	1.37E-02	7.44E-01	Distal Intergenic
Tns3	360980	chr14	88920156	88920456	-73285	0.85	1.38E-02	7.46E-01	Distal Intergenic
Rfk	499328	chr1	236499603	236499903	1830	-0.97	1.39E-02	7.50E-01	Promoter (1-2kb)
Slc16a6	303772	chr10	97756948	97757248	-427	0.88	1.39E-02	7.50E-01	Promoter (<=1kb)
Tnc	116640	chr5	79938988	79939288	-89123	0.83	1.39E-02	7.50E-01	Distal Intergenic
Tbc1d9	304645	chr19	24225771	24226071	-102952	1.17	1.40E-02	7.50E-01	Intron (NM_001191093/364983, intron 6 of 6)
Tm2d3	292995	chr1	126616663	126616963	43189	0.73	1.40E-02	7.50E-01	Distal Intergenic
Dtx2	304591	chr12	23702909	23703209	24355	0.98	1.41E-02	7.50E-01	Intron (NM_001107157/304591, intron 5 of 11)
Utp20	314713	chr7	29378603	29378903	-683	-1.25	1.41E-02	7.50E-01	Promoter (<=1kb)
Psmb1	94198	chr1	57472817	57473117	16588	1.64	1.41E-02	7.50E-01	Intron (NM_053590/94198, intron 4 of 5)
Rps3a	29288	chr2	185286553	185286853	157993	-0.95	1.41E-02	7.50E-01	Distal Intergenic
Por	29441	chr12	24043199	24043499	3315	1.27	1.41E-02	7.50E-01	Intron (NM_031576/29441, intron 1 of 15)
Abhd16b	311720	chr3	176987054	176987354	1154	1.29	1.42E-02	7.50E-01	Promoter (1-2kb)
Rasl11a	304268	chr12	9987163	9987463	2821	-1.03	1.42E-02	7.50E-01	Promoter (2-3kb)
Rxfp3	294807	chr2	61007718	61008018	0	0.97	1.42E-02	7.50E-01	Promoter (<=1kb)
Hrk	117271	chr12	44001747	44002047	27164	1.5	1.42E-02	7.50E-01	Intron (NM_001107144/304521, intron 11 of 11)
Gadd45g	291005	chr17	13193045	13193345	199874	1.63	1.42E-02	7.50E-01	Distal Intergenic
Cbr3	304078	chr11	33909430	33909730	13	0.53	1.42E-02	7.50E-01	Promoter (<=1kb)
Atcay	362826	chr7	11356746	11357046	729	1.07	1.42E-02	7.50E-01	Promoter (<=1kb)
Creb3l1	362165	chr3	80915562	80915862	17421	-1.13	1.43E-02	7.50E-01	Intron (NM_001005562/362165, intron 1 of 11)
Cyp2d1	266684	chr7	123629730	123630030	15	1.02	1.43E-02	7.51E-01	Promoter (<=1kb)
Apobec3	315137	chr7	121094593	121094893	-14357	0.91	1.44E-02	7.53E-01	Distal Intergenic
Kif5b	117550	chr17	54184013	54184313	2166	-1.3	1.44E-02	7.53E-01	Promoter (2-3kb)
Lmod1	304816	chr13	52147468	52147768	0	1.36	1.44E-02	7.53E-01	Promoter (<=1kb)
Apod	25239	chr11	72718645	72718945	13441	0.88	1.44E-02	7.53E-01	Intron (NM_012777/25239, intron 3 of 4)
Osbpl11	303888	chr11	70841849	70842149	-8120	1.12	1.45E-02	7.54E-01	Distal Intergenic
Tmx1	362751	chr6	92866820	92867120	2697	-1.21	1.45E-02	7.54E-01	Promoter (2-3kb)
Insyn1	501007	chr8	63228102	63228402	-58441	-0.82	1.46E-02	7.54E-01	Distal Intergenic
Cdk1	54237	chr20	20575801	20576101	-276	1.31	1.46E-02	7.54E-01	Promoter (<=1kb)
Cdk5rap2	286919	chr5	86554430	86554730	-328	-0.69	1.46E-02	7.54E-01	Promoter (<=1kb)
Nup58	245922	chr15	40542689	40542989	2835	-1.21	1.46E-02	7.54E-01	Promoter (2-3kb)
Ppif	282819	chr16	1980788	1981088	1597	-1.08	1.46E-02	7.54E-01	Promoter (1-2kb)
Fgr	79113	chr5	151172725	151173025	519	0.94	1.47E-02	7.56E-01	Promoter (<=1kb)
Kank2	1E+08	chr8	22814681	22814981	6242	1.04	1.48E-02	7.56E-01	Intron (NM_001270413/100361376, intron 3 of 11)
Ywhag	56010	chr12	23794965	23795265	5327	-1.12	1.48E-02	7.56E-01	Intron (NM_019376/56010, intron 1 of 2)
Cyp26a1	154985	chr1	256513247	256513547	130386	0.83	1.48E-02	7.56E-01	Distal Intergenic
Lrrc75b	1E+08	chr20	14054222	14054522	3757	-0.9	1.48E-02	7.56E-01	Exon (NM_001101016/100365744, exon 3 of 4)
Rad51	499870	chr3	110920499	110920799	2256	1.11	1.48E-02	7.56E-01	Promoter (2-3kb)
Gadd45g	291005	chr17	13394558	13394858	-1339	1.18	1.48E-02	7.56E-01	Promoter (1-2kb)
Chchd3	296966	chr4	59882791	59883091	475471	1.02	1.48E-02	7.56E-01	Distal Intergenic
Fam110d	500563	chr5	152469271	152469571	4297	-0.87	1.49E-02	7.56E-01	Downstream (1-2kb)
Dipk1b	362090	chr3	4083872	4084172	75	1.11	1.49E-02	7.56E-01	Promoter (<=1kb)
Wdr26	498301	chr13	99529434	99529734	2225	-1.03	1.49E-02	7.56E-01	Promoter (2-3kb)
Ndufb8	293991	chr1	264303960	264304260	-248	-0.73	1.49E-02	7.56E-01	Promoter (<=1kb)
Scrn2	360612	chr10	84967957	84968257	905	-1.43	1.49E-02	7.56E-01	Promoter (<=1kb)
Idh2	361596	chr1	141890315	141890615	3059	-1.26	1.50E-02	7.57E-01	Intron (NM_001014161/361596, intron 1 of 10)
Trim71	301042	chr8	122604709	122605009	0	-1.14	1.50E-02	7.57E-01	Promoter (<=1kb)
Zfp516	291406	chr18	79724613	79724913	-88846	-0.72	1.50E-02	7.58E-01	Distal Intergenic
Mybbp1a	60571	chr10	59002786	59003086	2037	-0.98	1.51E-02	7.59E-01	Promoter (2-3kb)
Jun	24516	chr5	114114871	114115171	-100594	1.1	1.52E-02	7.59E-01	Distal Intergenic
Mir138-1	1E+08	chr8	131462972	131463272	-268454	-0.92	1.52E-02	7.59E-01	Distal Intergenic
Psmd9	161475	chr12	38747293	38747593	57525	1.28	1.52E-02	7.59E-01	Distal Intergenic
Actr2	289820	chr14	104349295	104349595	26053	-1.13	1.52E-02	7.59E-01	Intron (NM_001009268/289820, intron 6 of 8)
Bcl2	24224	chr13	26692433	26692733	76641	0.72	1.52E-02	7.59E-01	Intron (NM_016993/24224, intron 1 of 1)
Ppt1	29411	chr5	140500403	140500703	-37557	-1.12	1.52E-02	7.59E-01	Distal Intergenic
Sirt4	304539	chr12	46867761	46868061	-355	-0.5	1.52E-02	7.59E-01	Promoter (<=1kb)
Nr2f2	113984	chr1	130771376	130771676	683013	-1.02	1.53E-02	7.60E-01	Distal Intergenic
Kcnf1	298908	chr6	42469044	42469344	4394	0.87	1.53E-02	7.60E-01	Downstream (1-2kb)
Snx11	303493	chr10	84641293	84641593	-2188	-1.08	1.53E-02	7.60E-01	Promoter (2-3kb)
Exoc6b	500233	chr4	116784139	116784439	1985	1.4	1.54E-02	7.61E-01	Promoter (1-2kb)
Sohlh1	362085	chr3	3295698	3295998	-597	-0.88	1.54E-02	7.61E-01	Promoter (<=1kb)
Gsk3b	84027	chr11	65205101	65205401	3436	-1.31	1.54E-02	7.61E-01	Intron (NM_032080/84027, intron 1 of 10)
Gcat	366959	chr7	120263797	120264097	729	-1.05	1.54E-02	7.62E-01	Promoter (<=1kb)
Apod	25239	chr11	72676874	72677174	-28030	0.72	1.55E-02	7.63E-01	Distal Intergenic
Slc19a1	29723	chr20	12365376	12365676	-12999	0.89	1.55E-02	7.64E-01	Distal Intergenic
Apol9a	503164	chr7	118925533	118925833	7945	1.26	1.55E-02	7.64E-01	Downstream (1-2kb)
Nsun2	361191	chr1	36319529	36319829	67	0.53	1.56E-02	7.67E-01	Promoter (<=1kb)
Hip1r	81917	chr12	38034804	38035104	17981	0.83	1.56E-02	7.67E-01	Intron (NM_001134763/81917, intron 7 of 31)
Neurl1b	303019	chr10	17037055	17037355	37784	0.74	1.57E-02	7.67E-01	Distal Intergenic
Uchl1	29545	chr14	43210862	43211162	-66920	-0.92	1.57E-02	7.67E-01	Distal Intergenic
Ranbp6	309326	chr1	247980087	247980387	5110	-0.9	1.57E-02	7.67E-01	Downstream (<1kb)

Rab36	690407	chr20	14543727	14544268	29251	-0.78	1.57E-02	7.67E-01	Distal Intergenic
Mllt11	295264	chr2	196386052	196386352	15754	-1.5	1.58E-02	7.68E-01	Intron (NM_001024302/499671, intron 3 of 3)
Hand2	64637	chr16	36380463	36380763	-6914	-0.95	1.59E-02	7.70E-01	Distal Intergenic
Syt5	54309	chr1	72860839	72861139	621	0.84	1.59E-02	7.70E-01	Promoter (<=1kb)
Mpnd	681944	chr9	11059567	11059867	1744	1.03	1.59E-02	7.70E-01	Promoter (1-2kb)
Itpkc	308451	chr1	84033063	84033363	7070	0.95	1.60E-02	7.73E-01	Intron (NM_178094/308451, intron 1 of 6)
RGD1562136	501195	chr9	111331265	111331565	3932	-1.52	1.60E-02	7.73E-01	Intron (NM_001109086/501195, intron 1 of 2)
Fahd2a	296131	chr3	120047715	120048015	-36341	-1.71	1.60E-02	7.73E-01	Intron (NM_032462/65199, intron 2 of 8)
Efhd2	298609	chr5	160504480	160504780	-80669	0.75	1.61E-02	7.73E-01	Distal Intergenic
Slc25a25	246771	chr3	11492622	11492922	-40093	1.47	1.61E-02	7.73E-01	Distal Intergenic
Kif3c	85248	chr6	27792386	27792686	23443	1.17	1.61E-02	7.73E-01	Exon (NM_053486/85248, exon 3 of 8)
Rft1	290552	chr16	6695210	6695510	-16879	0.82	1.61E-02	7.73E-01	Distal Intergenic
Vom2r77	690227	chr9	7902338	7902638	-87194	-0.65	1.61E-02	7.73E-01	Distal Intergenic
Aars	292023	chr19	43185808	43186108	29173	-0.59	1.62E-02	7.74E-01	Distal Intergenic
Mrps27	361883	chr2	29640130	29640430	41786	-0.88	1.62E-02	7.74E-01	Intron (NM_001108543/361883, intron 4 of 4)
Nvl	289323	chr13	99450237	99450537	18241	-0.93	1.62E-02	7.74E-01	Exon (NM_001105980/289323, exon 10 of 23)
Zfp689	286996	chr1	198914963	198915263	-14599	-0.67	1.63E-02	7.75E-01	Distal Intergenic
Slc10a4	305309	chr14	37772910	37773210	-2851	1.4	1.63E-02	7.75E-01	Promoter (2-3kb)
Grpel1	79563	chr14	79474664	79474964	-9894	1.14	1.63E-02	7.75E-01	Distal Intergenic
Mecr	29470	chr5	150002477	150002777	1196	-1.03	1.64E-02	7.75E-01	Promoter (1-2kb)
Chic2	83835	chr14	35681251	35681551	-2106	1.16	1.64E-02	7.75E-01	Promoter (2-3kb)
Nup210	58958	chr4	122707813	122708113	32997	1.02	1.64E-02	7.75E-01	Exon (NM_053322/58958, exon 7 of 40)
Traf6	311245	chr3	91254847	91255147	2018	-1.03	1.64E-02	7.75E-01	Promoter (2-3kb)
Sox9	140586	chr10	101288784	101289084	256	0.59	1.65E-02	7.75E-01	Promoter (<=1kb)
Robo3	315564	chr8	39931595	39931895	-24117	0.86	1.65E-02	7.75E-01	Distal Intergenic
LOC102550367	1.03E+08	chr3	120937915	120938215	145021	0.99	1.65E-02	7.75E-01	Distal Intergenic
Ppib	64367	chr8	71722101	71722401	2420	-1.03	1.65E-02	7.75E-01	Promoter (2-3kb)
Ntrk3	29613	chr1	140350906	140351206	-88403	1.26	1.65E-02	7.75E-01	Distal Intergenic
Nradd	246143	chr8	118880992	118881292	12269	-1.11	1.66E-02	7.75E-01	Intron (NM_001108189/316013, intron 18 of 20)
Htt	29424	chr14	81251415	81251715	2922	-0.92	1.66E-02	7.75E-01	Promoter (2-3kb)
Cdh15	361432	chr19	55744196	55744496	74535	-1.09	1.66E-02	7.75E-01	Distal Intergenic
Zkscan1	498160	chr12	19220897	19221197	-9895	0.98	1.66E-02	7.75E-01	Distal Intergenic
Tead3	294299	chr20	7933953	7934253	-3004	-1.29	1.66E-02	7.75E-01	Intron (NM_001107642/309900, intron 11 of 12)
Mllt11	295264	chr2	196392496	196392796	9310	1.72	1.67E-02	7.75E-01	Intron (NM_001024302/499671, intron 3 of 3)
Klhl40	316088	chr8	130396356	130396656	-19699	-1.16	1.67E-02	7.75E-01	Distal Intergenic
Trmt10a	295496	chr2	243422384	243422684	-127	-0.8	1.67E-02	7.75E-01	Promoter (<=1kb)
Lmo7	361084	chr15	86391364	86391664	148216	0.76	1.67E-02	7.75E-01	Intron (NM_001001515/361084, intron 4 of 30)
Tnni1	29388	chr13	52619341	52619641	-5800	0.75	1.67E-02	7.76E-01	Distal Intergenic
Ca14	791259	chr2	198019682	198019982	-2823	-0.83	1.68E-02	7.77E-01	Promoter (2-3kb)
Lmntd1	500362	chr4	179719486	179719786	-19356	1.73	1.68E-02	7.77E-01	Distal Intergenic
Dram1	679937	chr7	28958573	28958873	-25932	-1.31	1.68E-02	7.77E-01	Distal Intergenic
Rasl11a	304268	chr12	9951198	9951498	38786	-1.16	1.69E-02	7.77E-01	Distal Intergenic
Nt5c3a	312373	chr4	87262333	87262633	18502	-1.83	1.69E-02	7.78E-01	Intron (NM_001107862/312373, intron 1 of 9)
LOC499136	499136	chr1	98001004	98001304	-215514	-0.81	1.69E-02	7.78E-01	Distal Intergenic
Atp10a	365266	chr1	115977577	115977877	4234	0.94	1.70E-02	7.80E-01	Intron (NM_001141935/365266, intron 2 of 21)
Runx2	367218	chr9	18819358	18819658	175788	-1.06	1.70E-02	7.81E-01	Distal Intergenic
Yeats4	299810	chr7	60282095	60282395	12223	-0.9	1.70E-02	7.81E-01	Distal Intergenic
Parn	360464	chr10	1245518	1245818	215305	0.93	1.71E-02	7.81E-01	Distal Intergenic
Slc2a13	171147	chr7	132691630	132691930	65628	0.95	1.71E-02	7.83E-01	Intron (NM_133611/171147, intron 2 of 9)
Arl6ip5	66028	chr4	129584543	129584843	10180	0.84	1.71E-02	7.83E-01	Intron (NM_023972/66028, intron 1 of 2)
Ncoa4	619385	chr16	8306145	8306445	-2794	1.43	1.72E-02	7.83E-01	Promoter (2-3kb)
Pgm2	289632	chr14	45914082	45914382	-50210	1.27	1.72E-02	7.83E-01	Distal Intergenic
Smpd3	94338	chr19	38338820	38339120	-17292	1.24	1.73E-02	7.85E-01	Distal Intergenic
P4ha3	361612	chr1	165296151	165296451	304	0.57	1.73E-02	7.88E-01	Promoter (<=1kb)
Ppcs	298490	chr5	138457116	138457416	12318	0.9	1.74E-02	7.88E-01	Distal Intergenic
Peli3	309157	chr1	220203701	220204001	31	0.7	1.74E-02	7.88E-01	Promoter (<=1kb)
Tiam1	304109	chr11	29893164	29893464	167709	0.85	1.75E-02	7.88E-01	Distal Intergenic
Krt8	25626	chr7	143605100	143605400	-1355	-0.82	1.75E-02	7.88E-01	Promoter (1-2kb)
Dhps	288923	chr19	26187375	26187675	2710	-1.08	1.75E-02	7.88E-01	Promoter (2-3kb)
Wfdc2	286888	chr3	161014388	161014688	-3814	0.95	1.75E-02	7.88E-01	Distal Intergenic
Optn	246294	chr17	77198677	77198977	21972	-0.8	1.75E-02	7.88E-01	Intron (NM_145081/246294, intron 10 of 15)
Ogdh	360975	chr14	86414525	86414825	-151	-0.78	1.76E-02	7.88E-01	Promoter (<=1kb)
Gmfb	81661	chr15	23612766	23613066	-1876	-0.85	1.76E-02	7.88E-01	Promoter (1-2kb)
B3galt4	171079	chr20	5446032	5446332	0	-0.62	1.76E-02	7.88E-01	Promoter (<=1kb)
Per3	78962	chr5	168123921	168124221	-891	0.7	1.76E-02	7.88E-01	Promoter (<=1kb)
Osbpl2	296461	chr3	175491664	175491964	-1734	-0.91	1.76E-02	7.88E-01	Promoter (1-2kb)
Tspyl1	29544	chr20	41085704	41086004	2387	-0.97	1.76E-02	7.88E-01	Promoter (2-3kb)
Rin3	314397	chr6	126171223	126171523	503	-0.99	1.76E-02	7.88E-01	Promoter (<=1kb)
Neurod6	500137	chr4	85880723	85881023	34084	0.9	1.77E-02	7.89E-01	Distal Intergenic
Mir181c	1E+08	chr19	25302847	25303147	-12531	0.72	1.77E-02	7.89E-01	Distal Intergenic
Nudt9	305149	chr14	7044233	7044533	10015	1.08	1.77E-02	7.89E-01	Exon (NM_001006991/305149, exon 5 of 8)
Thap1	306547	chr16	70616516	70616816	49015	0.73	1.78E-02	7.89E-01	Distal Intergenic
LOC307727	307727	chrX	10851836	10852136	40359	0.99	1.78E-02	7.89E-01	Distal Intergenic
Spcs2	293142	chr1	164850029	164850329	-280	-0.54	1.78E-02	7.90E-01	Promoter (<=1kb)
Crygn	296730	chr4	6909036	6909336	5838	1.19	1.79E-02	7.90E-01	3' UTR
Gins2	292058	chr19	53989964	53990264	173344	1.08	1.79E-02	7.90E-01	Distal Intergenic
Dusp16	297682	chr4	168488473	168488773	28206	0.86	1.79E-02	7.90E-01	Intron (NM_001106624/297682, intron 3 of 5)
Brca1	497672	chr10	89445133	89445433	9248	0.73	1.79E-02	7.91E-01	Intron (NM_012514/497672, intron 2 of 22)
Naa20	362228	chr3	140060916	140061216	-45510	-0.71	1.80E-02	7.91E-01	Exon (NM_001107786/311494, exon 9 of 13)
Ppp6r1	361502	chr1	72755748	72756048	-1833	-0.74	1.80E-02	7.91E-01	Promoter (1-2kb)
Wac	307029	chr17	61234894	61235194	-288264	1.53	1.80E-02	7.92E-01	Distal Intergenic
Sumo3	499417	chr20	11736368	11736668	382	0.85	1.81E-02	7.95E-01	Promoter (<=1kb)
Zfp362	297879	chr5	146992080	146992380	-18148	-0.61	1.82E-02	7.96E-01	Distal Intergenic
Myc	24577	chr7	102655399	102655699	69086	0.87	1.82E-02	7.96E-01	Distal Intergenic
Paics	140946	chr14	33582855	33583155	-2289	-0.87	1.82E-02	7.96E-01	Promoter (2-3kb)
Galnt7	29750	chr16	35995839	35996139	60780	-0.9	1.82E-02	7.96E-01	Intron (NM_022926/29750, intron 1 of 11)
Uchl1	29545	chr14	43239752	43240052	-95810	1.44	1.83E-02	7.98E-01	Distal Intergenic
Rhpn2	308516	chr1	91611343	91611643	14946	1.04	1.84E-02	7.98E-01	Intron (NM_001107505/308516, intron 1 of 15)
B3gnt2	305571	chr14	107604592	107604892	12269	-1.11	1.84E-02	7.98E-01	Intron (NM_001107240/305571, intron 2 of 2)
Lamb3	305078	chr13	112031647	112031947	0	1.11	1.84E-02	7.98E-01	Promoter (<=1kb)
Zfp219	305848	chr15	28419090	28419390	-13044	-0.7	1.84E-02	7.98E-01	Distal Intergenic
Trerf1	316219	chr9	15682161	15682461	17851	-1.13	1.85E-02	8.00E-01	Intron (NM_001108199/316219, intron 8 of 12)
Aqp7	29171	chr5	57351687	57351987	20252	0.83	1.85E-02	8.00E-01	Distal Intergenic
Nup35	295692	chr3	68455562	68455862	584994	1.37	1.86E-02	8.03E-01	Distal Intergenic

Rab1a	81754	chr14	104524123	104524423	49041	-1.12	1.88E-02	8.10E-01	Distal Intergenic
LOC310926	310926	chr14	46565270	46565570	-71360	-0.97	1.88E-02	8.10E-01	Distal Intergenic
Tagap	308097	chr1	47433832	47434132	67888	1.16	1.88E-02	8.10E-01	Distal Intergenic
Rsbn1	310749	chr2	206407598	206407898	15398	1.51	1.88E-02	8.10E-01	Intron (NM_001191710/310749, intron 2 of 6)
Elmod1	315670	chr8	58643556	58643856	-100712	1.63	1.89E-02	8.10E-01	Distal Intergenic
Lrrc28	361588	chr1	128608628	128608928	-4458	-0.78	1.89E-02	8.11E-01	Distal Intergenic
Aldh4a1	641316	chr5	158092352	158092652	2114	-1.11	1.89E-02	8.11E-01	Promoter (2-3kb)
Sema4c	301346	chr9	43140302	43140602	-432	1.16	1.90E-02	8.12E-01	Promoter (<=1kb)
Clip1	65201	chr12	38328052	38328352	-17104	-1.05	1.90E-02	8.13E-01	Distal Intergenic
Smad6	367100	chr8	68911491	68911791	54317	-1.19	1.90E-02	8.13E-01	Intron (NM_001109002/367100, intron 3 of 3)
Zmpste24	313564	chr5	140013199	140013499	2042	-1	1.91E-02	8.13E-01	Promoter (2-3kb)
Arhgef19	362648	chr5	159761068	159761368	5533	0.95	1.91E-02	8.14E-01	Exon (NM_001108692/362648, exon 3 of 18)
Rgs3	54293	chr5	78591136	78591436	107243	0.63	1.91E-02	8.14E-01	Distal Intergenic
Rin2	311494	chr3	139964770	139965070	70439	0.91	1.91E-02	8.14E-01	Intron (NM_001107786/311494, intron 2 of 12)
Emp3	81505	chr1	101898873	101899173	4737	1.33	1.92E-02	8.16E-01	Exon (NM_001107513/308593, exon 8 of 9)
Ehbp1	305556	chr14	107047854	107048154	77228	-0.92	1.93E-02	8.17E-01	Intron (NM_001305130/305556, intron 5 of 24)
Nucb2	59295	chr1	185141179	185141479	1728	-0.9	1.93E-02	8.17E-01	Promoter (1-2kb)
Csrp3	117505	chr1	104204660	104204960	-46805	-1.06	1.94E-02	8.17E-01	Distal Intergenic
Egr1	24330	chr18	27681394	27681694	23491	0.67	1.94E-02	8.17E-01	Distal Intergenic
Zdhhc7	170906	chr19	52728150	52728450	21857	0.95	1.94E-02	8.17E-01	Distal Intergenic
Rbms1	362138	chr3	46917767	46918067	107061	-0.96	1.94E-02	8.17E-01	Intron (NM_001012184/362138, intron 1 of 13)
Nfkb2	309452	chr1	266056177	266056477	3175	-0.95	1.95E-02	8.17E-01	Exon (NM_001008349/309452, exon 12 of 22)
Serp2	498546	chr15	58711717	58712017	0	0.57	1.95E-02	8.17E-01	Promoter (<=1kb)
Cpz	83575	chr14	80382480	80382780	-20202	1.23	1.95E-02	8.17E-01	Distal Intergenic
Sun1	360773	chr12	17531378	17531678	-8844	-1	1.95E-02	8.17E-01	Distal Intergenic
Smurf1	690516	chr12	11391881	11392181	-41634	0.88	1.95E-02	8.17E-01	Intron (NM_153312/266682, intron 8 of 15)
Zcchc24	361104	chr16	2027018	2027318	20974	1.02	1.96E-02	8.20E-01	Intron (NM_001108394/361104, intron 2 of 3)
Jmjd6	360665	chr10	105771663	105771963	15840	1.62	1.96E-02	8.20E-01	Distal Intergenic
Nln	117041	chr2	34039822	34040122	145560	1.14	1.97E-02	8.20E-01	Distal Intergenic
Map7	293016	chr1	15641738	15642038	-115	0.86	1.97E-02	8.20E-01	Promoter (<=1kb)
Yju2	501285	chr9	11103407	11103707	5034	-0.97	1.97E-02	8.20E-01	Exon (NM_001109673/501285, exon 5 of 8)
Rhbg	310625	chr2	187622195	187622495	0	0.9	1.97E-02	8.20E-01	Promoter (<=1kb)
Ssna1	311802	chr3	2485820	2486120	4361	-1.09	1.97E-02	8.20E-01	Intron (NM_001024309/499749, intron 1 of 3)
Hspb1	24471	chr12	23838492	23838792	2259	-0.89	1.97E-02	8.20E-01	Promoter (2-3kb)
Abcc10	316231	chr9	17042893	17043193	1542	-1	1.98E-02	8.20E-01	Promoter (1-2kb)
Slc66a3	298906	chr6	42299527	42299827	2427	-1.39	1.98E-02	8.20E-01	Promoter (2-3kb)
Slc22a5	29726	chr10	39194384	39194684	33406	-0.82	1.98E-02	8.22E-01	Distal Intergenic
Lepr	24536	chr5	120507671	120507971	4196	-0.89	1.99E-02	8.22E-01	Intron (NM_020099/56766, intron 3 of 3)
Vps33b	64060	chr1	142062954	142063254	1999	1.06	1.99E-02	8.22E-01	Promoter (1-2kb)
Larp4b	307070	chr17	63270120	63270420	34113	-0.69	2.00E-02	8.22E-01	Intron (NM_001107361/307070, intron 4 of 18)
Cstf2t	309338	chr1	249312087	249312387	-262567	0.93	2.00E-02	8.22E-01	Intron (NM_001105731/54286, intron 5 of 16)
Slfn4	114247	chr10	70412918	70413218	1180	-1.19	2.00E-02	8.22E-01	Promoter (1-2kb)
Ldb3	498587	chr16	10908819	10909119	32299	0.75	2.00E-02	8.22E-01	Exon (NM_001277165/498587, exon 8 of 14)
Rhot1	303351	chr10	67558920	67559220	-200	-0.99	2.00E-02	8.22E-01	Promoter (<=1kb)
Cadm3	360882	chr13	91923136	91923436	-50183	-0.96	2.01E-02	8.25E-01	Distal Intergenic
Padi6	298595	chr5	159118371	159118671	138028	1.13	2.02E-02	8.28E-01	Distal Intergenic
Klhl38	314996	chr7	98195916	98196216	871	-1.19	2.02E-02	8.28E-01	Promoter (<=1kb)
Tead1	361630	chr1	177678236	177678536	182454	-0.97	2.03E-02	8.28E-01	Intron (NM_001198589/361630, intron 9 of 12)
Gsdme	353316	chr4	80144051	80144351	-144373	-0.87	2.03E-02	8.28E-01	Distal Intergenic
Exo5	313563	chr5	139869608	139869908	-16361	-1.22	2.03E-02	8.28E-01	Distal Intergenic
Smim38	246306	chr1	218252228	218252528	122091	0.73	2.04E-02	8.28E-01	Distal Intergenic
Plpp1	64369	chr2	44663661	44663961	-489	-0.89	2.04E-02	8.28E-01	Promoter (<=1kb)
Tchp	304547	chr12	47630810	47631110	21597	-0.91	2.04E-02	8.28E-01	3' UTR
Nudt19	308518	chr1	91808219	91808519	23394	0.94	2.04E-02	8.30E-01	Distal Intergenic
Acot7	26759	chr5	169368029	169368329	3723	1.17	2.05E-02	8.31E-01	Intron (NM_013214/26759, intron 1 of 8)
Spred2	305539	chr14	104076522	104076822	-114695	1.25	2.05E-02	8.32E-01	Distal Intergenic
Oaz2	501454	chr8	71215462	71215762	-249	-0.71	2.06E-02	8.32E-01	Promoter (<=1kb)
Mir222	1E+08	chrX	3605762	3606062	-77857	1.1	2.06E-02	8.32E-01	Distal Intergenic
Tmem30c	288175	chr11	45261256	45261556	-75962	1.27	2.06E-02	8.32E-01	Intron (NM_001013866/288176, intron 1 of 9)
Tfrc	64678	chr11	71419351	71419651	-88	-0.61	2.07E-02	8.32E-01	Promoter (<=1kb)
Nudc	29648	chr5	151768586	151768886	-485	-0.99	2.07E-02	8.32E-01	Promoter (<=1kb)
Pias3	83614	chr2	198825940	198826240	2571	1.55	2.07E-02	8.32E-01	Promoter (2-3kb)
Notch1	25496	chr3	3951313	3951613	-298	-0.83	2.07E-02	8.32E-01	Promoter (<=1kb)
Rtl8a	679038	chrX	73581531	73581831	-34540	-0.66	2.07E-02	8.32E-01	Distal Intergenic
Eml1	362783	chr6	132339119	132339419	-27923	0.66	2.08E-02	8.34E-01	Distal Intergenic
Zbtb10	80338	chr2	94531793	94532093	198883	0.92	2.09E-02	8.36E-01	Distal Intergenic
Magee1	317232	chrX	76083762	76084062	209	1.08	2.09E-02	8.37E-01	Promoter (<=1kb)
Eif4g2	361628	chr1	176057845	176058145	-162335	0.82	2.10E-02	8.37E-01	Distal Intergenic
Rfc2	116468	chr12	25149116	25149416	-5636	-1.18	2.10E-02	8.37E-01	Distal Intergenic
Trps1	299897	chr7	90353044	90353344	-34823	-1.02	2.10E-02	8.37E-01	Distal Intergenic
Pkd2l2	291683	chr18	27187407	27187707	-3267	-0.83	2.11E-02	8.37E-01	Distal Intergenic
Vrk1	362779	chr6	129724092	129724392	-111527	1.39	2.11E-02	8.37E-01	Distal Intergenic
Abhd2	293050	chr1	141007361	141007661	9121	-0.97	2.11E-02	8.37E-01	Intron (NM_001106275/293050, intron 1 of 10)
Ston1	360202	chr6	12332106	12332406	-30407	-1.06	2.11E-02	8.37E-01	Distal Intergenic
Ech1	64526	chr1	87007516	87007816	-1982	-1.26	2.12E-02	8.37E-01	Promoter (1-2kb)
Kbtbd6	306073	chr15	61731147	61731447	-127	-1.11	2.12E-02	8.37E-01	Promoter (<=1kb)
Mrps17	288621	chr12	30573738	30574038	-175	-1.07	2.12E-02	8.37E-01	Promoter (<=1kb)
Rps6ka3	501560	chrX	37468848	37469148	-748	-0.82	2.12E-02	8.37E-01	Promoter (<=1kb)
Smc4	295107	chr2	165600853	165601153	0	-0.62	2.13E-02	8.41E-01	Promoter (<=1kb)
Ppid	361967	chr2	178354344	178354644	-186	-0.63	2.14E-02	8.42E-01	Promoter (<=1kb)
Ptprm	29616	chr9	115506209	115506509	48752	1.31	2.14E-02	8.42E-01	Intron (NM_001168632/29616, intron 1 of 32)
Rho	24717	chr4	147798987	147799287	-32849	0.9	2.14E-02	8.42E-01	Intron (NM_001009416/312651, intron 14 of 17)
Rrlt	679825	chr1	1180037	1180337	-63111	1.3	2.14E-02	8.42E-01	Intron (NM_001013063/292461, intron 5 of 6)
Mir148b	1E+08	chr7	144912612	144912912	574	-1.06	2.16E-02	8.45E-01	Promoter (<=1kb)
Glrx	64045	chr2	2459391	2459691	-146341	0.89	2.16E-02	8.45E-01	Distal Intergenic
Fmod	64507	chr13	50832870	50833170	-41716	0.85	2.16E-02	8.45E-01	Distal Intergenic
Capn2	29154	chr13	100938147	100938447	-9336	0.99	2.16E-02	8.45E-01	Distal Intergenic
Otulinl	310190	chr2	80752406	80752706	-343734	-0.93	2.17E-02	8.45E-01	Distal Intergenic
Ryk	140585	chr8	111379760	111380060	53406	0.97	2.17E-02	8.45E-01	Intron (NM_080402/140585, intron 8 of 14)
Myrip	360034	chr8	128941090	128941390	-62297	0.75	2.17E-02	8.45E-01	Distal Intergenic
Dhcr7	64191	chr1	217022735	217023035	3819	-1.02	2.17E-02	8.45E-01	Intron (NM_022389/64191, intron 2 of 8)
Srp9	690345	chr13	100573922	100574222	22615	0.79	2.18E-02	8.45E-01	Distal Intergenic
Mesd	308796	chr1	146162220	146162520	124794	0.69	2.18E-02	8.45E-01	Distal Intergenic
Igf1r	25718	chr1	129036371	129036671	111450	-1.01	2.18E-02	8.45E-01	Intron (NM_052807/25718, intron 2 of 20)

Tnik	294917	chr2	113984717	113985017	71	-1.61	2.18E-02	8.45E-01	Promoter (<=1kb)
Lsm3	297455	chr4	123320954	123321254	158868	1.01	2.19E-02	8.47E-01	Distal Intergenic
Ppp1r1b	360616	chr10	86303773	86304073	46	0.51	2.19E-02	8.47E-01	Promoter (<=1kb)
Arfgap3	503165	chr7	124186396	124186696	11567	1.04	2.19E-02	8.47E-01	Intron (NM_001044273/503165, intron 2 of 19)
RT1-T24-4	414784	chr20	3185472	3185772	-3685	0.62	2.19E-02	8.47E-01	Distal Intergenic
Smc2	362519	chr5	68714812	68715112	-2517	-1.15	2.20E-02	8.48E-01	Promoter (2-3kb)
Slc38a2	29642	chr7	138089962	138090262	10561	-1.13	2.21E-02	8.50E-01	3' UTR
Clcf1	365395	chr1	219465484	219465784	-3082	0.79	2.22E-02	8.50E-01	Distal Intergenic
Smurf2	303614	chr10	95090991	95091291	26816	1.19	2.22E-02	8.50E-01	Intron (NM_001107061/303614, intron 1 of 23)
Whrn	313255	chr5	79317589	79317889	-383	0.67	2.22E-02	8.50E-01	Promoter (<=1kb)
Rttn	291377	chr18	86055274	86055574	-16310	-0.93	2.22E-02	8.50E-01	Distal Intergenic
Tfcp2	315309	chr7	142181166	142181466	-372	0.94	2.23E-02	8.50E-01	Promoter (<=1kb)
Umps	288051	chr11	70058759	70059059	24578	0.99	2.23E-02	8.50E-01	Exon (NM_147139/257645, exon 15 of 16)
Riok3	361293	chr18	3450841	3451141	-114025	-0.62	2.23E-02	8.50E-01	Distal Intergenic
Twist2	59327	chr9	98922239	98922539	-1595	-1.01	2.24E-02	8.50E-01	Promoter (1-2kb)
Pmepa1	311676	chr3	171339964	171340264	-37466	0.97	2.24E-02	8.50E-01	Distal Intergenic
Foxe3	171302	chr5	133620715	133621015	104641	0.58	2.24E-02	8.50E-01	Distal Intergenic
LOC100910620	1.01E+08	chr5	74374103	74374403	68269	1.05	2.24E-02	8.50E-01	Distal Intergenic
Tdg	114521	chr7	27182301	27182601	31075	1.03	2.24E-02	8.50E-01	Distal Intergenic
Pex11g	288369	chr12	1970416	1970716	36800	-0.91	2.24E-02	8.50E-01	Intron (NM_001107115/304193, intron 10 of 27)
Ninj2	59115	chr4	152722062	152722362	90861	0.86	2.25E-02	8.50E-01	Intron (NM_021595/59115, intron 1 of 3)
Tnfaip8	307428	chr18	44737262	44737562	108	-0.9	2.25E-02	8.50E-01	Promoter (<=1kb)
Prex1	311647	chr3	163354085	163354385	123437	0.86	2.25E-02	8.50E-01	Exon (NM_001135718/311647, exon 22 of 44)
Olr806	405331	chr4	72141199	72141499	-80051	-1.19	2.25E-02	8.50E-01	Distal Intergenic
Serhl2	500911	chr7	123962996	123963296	-85	1.08	2.25E-02	8.50E-01	Promoter (<=1kb)
Ctsb	64529	chr15	46291753	46292053	-24688	1.3	2.26E-02	8.51E-01	Distal Intergenic
Mir761	1E+08	chr5	128684682	128684982	32815	1.36	2.26E-02	8.51E-01	Intron (NM_012993/25499, intron 20 of 30)
Tmem150a	245966	chr4	100215088	100215388	-3273	-0.72	2.26E-02	8.51E-01	Distal Intergenic
Gid8	296466	chr3	176211398	176211698	-5430	-1.09	2.27E-02	8.51E-01	Distal Intergenic
Srfbp1	291469	chr18	47455710	47456010	-396	-0.97	2.27E-02	8.51E-01	Promoter (<=1kb)
Vmac	363327	chr9	10381655	10381955	42580	-0.65	2.27E-02	8.51E-01	Distal Intergenic
Pitx2	54284	chr2	233323697	233323997	-278735	0.98	2.27E-02	8.51E-01	Distal Intergenic
Fa2h	307855	chr19	43596706	43597006	0	0.97	2.28E-02	8.51E-01	Promoter (<=1kb)
Dagla	309207	chr1	226329369	226329669	23942	1.1	2.28E-02	8.51E-01	Intron (NM_001005886/309207, intron 1 of 19)
Atp6v1b2	117596	chr16	22350326	22350626	-183	-0.88	2.28E-02	8.51E-01	Promoter (<=1kb)
Rab36	690407	chr20	14486613	14486913	86606	1.11	2.28E-02	8.51E-01	Distal Intergenic
Rap2a	114560	chr15	105848792	105849092	-2451	-0.92	2.29E-02	8.51E-01	Promoter (2-3kb)
Mir199a2	1E+08	chr13	80124699	80124999	-488	0.91	2.29E-02	8.51E-01	Promoter (<=1kb)
Pygo2	295251	chr2	188754421	188754721	-2894	-1.28	2.29E-02	8.51E-01	Promoter (2-3kb)
Pipox	303272	chr10	64952209	64952509	0	0.98	2.30E-02	8.51E-01	Promoter (<=1kb)
RGD1359158	361740	chr1	239240171	239240471	25526	-0.95	2.30E-02	8.51E-01	Intron (NM_001007737/361740, intron 1 of 3)
Atoh8	500200	chr4	100083720	100084020	15497	1.05	2.30E-02	8.51E-01	Intron (NM_001109241/500200, intron 2 of 2)
Rnls	361751	chr1	252100242	252100542	217	0.66	2.30E-02	8.51E-01	Promoter (<=1kb)
Zbtb5	298084	chr5	60557305	60557605	730	-0.99	2.30E-02	8.51E-01	Promoter (<=1kb)
Nkx2-3	309389	chr1	263371218	263371518	3952	0.57	2.31E-02	8.51E-01	Distal Intergenic
Septin11	305227	chr14	16594369	16594669	-141479	-0.96	2.31E-02	8.51E-01	Distal Intergenic
Dnajc18	291677	chr18	28496022	28496322	4631	-0.82	2.31E-02	8.52E-01	Intron (NM_001013887/291677, intron 1 of 9)
RT1-T24-1	361787	chr20	3270878	3271178	1563	1.13	2.32E-02	8.54E-01	Promoter (1-2kb)
Sgk2	171497	chr3	159360856	159361156	-157	-0.76	2.32E-02	8.54E-01	Promoter (<=1kb)
Dlx3	287638	chr10	82917320	82917620	-20351	-0.88	2.32E-02	8.54E-01	Distal Intergenic
Gpr107	311857	chr3	10720078	10720378	-25424	1	2.33E-02	8.54E-01	Distal Intergenic
Ushbp1	290629	chr16	19792145	19792445	-313	0.66	2.33E-02	8.54E-01	Promoter (<=1kb)
Slc27a5	79111	chr1	65613263	65613563	36664	-0.79	2.33E-02	8.54E-01	Distal Intergenic
Slc15a4	246280	chr12	32757929	32758229	4105	-1	2.34E-02	8.55E-01	Intron (NM_144758/246280, intron 1 of 7)
Zkscan3	306977	chr17	45239222	45239522	-8272	-1.22	2.34E-02	8.55E-01	Distal Intergenic
Cep120	307302	chr18	48715447	48715747	4725	-0.95	2.34E-02	8.55E-01	Intron (NM_001191697/307302, intron 3 of 20)
Inpp1	316376	chr9	53562851	53563151	227	-0.84	2.36E-02	8.60E-01	Promoter (<=1kb)
Rpp40	291071	chr17	29566882	29567182	-71897	1.24	2.36E-02	8.61E-01	Distal Intergenic
Krt8	25626	chr7	143608791	143609091	-5046	0.85	2.36E-02	8.61E-01	Distal Intergenic
Adss	289276	chr13	95940222	95940522	3239	-0.7	2.37E-02	8.61E-01	Intron (NM_001105975/289276, intron 1 of 12)
Pja2	192256	chr9	111981884	111982184	66663	-0.71	2.38E-02	8.64E-01	Distal Intergenic
Cda	362638	chr5	156800179	156800479	-18851	0.77	2.38E-02	8.64E-01	Distal Intergenic
Twsg1	363294	chr9	113698535	113698835	-2641	1.19	2.39E-02	8.66E-01	Promoter (2-3kb)
Pitpna	29525	chr10	63736586	63736886	4819	-0.96	2.39E-02	8.67E-01	Intron (NM_017231/29525, intron 2 of 11)
Pmp22	24660	chr10	49538752	49539052	164	-1.03	2.41E-02	8.70E-01	Promoter (<=1kb)
Pafah1b2	64189	chr8	50273498	50273798	3999	0.9	2.41E-02	8.70E-01	Intron (NM_022387/64189, intron 1 of 5)
Scp2	25541	chr5	127735321	127735621	72	-0.86	2.42E-02	8.72E-01	Promoter (<=1kb)
Pex26	297570	chr4	153758725	153759025	10382	0.59	2.42E-02	8.72E-01	3' UTR
Ptp4a2	85237	chr5	148023417	148023717	-27409	1.2	2.43E-02	8.72E-01	Distal Intergenic
LOC498265	498265	chr13	82296224	82296524	1880	-1.21	2.43E-02	8.73E-01	Promoter (1-2kb)
Tob1	170842	chr10	81860261	81860561	-53128	0.69	2.44E-02	8.74E-01	Distal Intergenic
Eci3	291076	chr17	30616825	30617125	-257	-0.51	2.44E-02	8.74E-01	Promoter (<=1kb)
Epha7	171287	chr5	43622761	43623061	19858	-1.02	2.44E-02	8.74E-01	Intron (NM_134331/171287, intron 3 of 16)
Mir100	1E+08	chr8	45729934	45730234	-16714	0.82	2.45E-02	8.76E-01	Intron (NR_126581/104845260, intron 2 of 3)
Eef2k	25435	chr1	190791462	190791762	-6943	0.93	2.45E-02	8.76E-01	Distal Intergenic
Chd2	308738	chr1	134951867	134952167	-80699	-1.15	2.46E-02	8.76E-01	Distal Intergenic
Pgap1	316400	chr9	61298962	61299262	-163999	1.16	2.46E-02	8.76E-01	Intron (NM_001191807/301415, intron 16 of 27)
Acaa2	170465	chr18	70766732	70767032	32860	1.33	2.46E-02	8.76E-01	Distal Intergenic
Itgb1	24511	chr19	61683735	61684035	6193	1.52	2.46E-02	8.76E-01	Intron (NM_017022/24511, intron 1 of 15)
Rexo2	300689	chr8	52815936	52816236	-1615	-0.91	2.46E-02	8.76E-01	Promoter (1-2kb)
Hey2	155430	chr1	29227766	29228066	36596	-0.93	2.47E-02	8.77E-01	Distal Intergenic
Ubash3b	315579	chr8	45405605	45405905	-30471	0.78	2.47E-02	8.77E-01	Distal Intergenic
Tapbpl	297602	chr4	157743895	157744195	-696	-1.48	2.48E-02	8.78E-01	Promoter (<=1kb)
Rps13	161477	chr1	185356363	185356663	27461	0.57	2.48E-02	8.78E-01	Distal Intergenic
Dclk1	83825	chr2	144993188	144993488	131743	0.88	2.49E-02	8.78E-01	Distal Intergenic
Capzb	298584	chr5	157684949	157685249	42172	0.72	2.49E-02	8.78E-01	Intron (NM_001005903/298584, intron 1 of 8)
Zfp347	170902	chr7	9883589	9883889	107071	-0.91	2.49E-02	8.78E-01	Distal Intergenic
Eps8l3	295361	chr2	210610596	210610896	-57507	1.29	2.49E-02	8.78E-01	Distal Intergenic
Mir761	1E+08	chr5	128735808	128736108	83941	-0.87	2.49E-02	8.78E-01	Intron (NM_001044234/298369, intron 4 of 23)
RGD1310852	314992	chr7	97888378	97888678	13907	1.44	2.50E-02	8.78E-01	Distal Intergenic
Zfpm1	691504	chr19	55117894	55118194	23309	-0.8	2.50E-02	8.79E-01	Intron (NM_001242627/691504, intron 2 of 10)
Cd1d1	25109	chr2	186333248	186333548	257	-0.74	2.50E-02	8.79E-01	Promoter (<=1kb)
Rad54b	313063	chr5	25141138	25141438	-5525	-0.97	2.51E-02	8.80E-01	Distal Intergenic
Mir551b	1E+08	chr2	117341200	117341500	473553	-0.72	2.51E-02	8.80E-01	Distal Intergenic

Arntl	29657	chr1	178000153	178000453	-38610	0.95	2.52E-02	8.80E-01	Distal Intergenic
Rpl10	81764	chrX	156441025	156441325	-564	-0.72	2.52E-02	8.80E-01	Promoter (<=1kb)
Hs6st1	316325	chr9	42641335	42641635	21329	0.97	2.52E-02	8.80E-01	Intron (NM_001108210/316325, intron 1 of 1)
Klhl40	316088	chr8	130355428	130355728	-60627	-1.09	2.53E-02	8.83E-01	Distal Intergenic
Pfkp	60416	chr17	68559080	68559380	15007	-0.77	2.54E-02	8.85E-01	Intron (NM_206847/60416, intron 2 of 23)
Pnkd	1E+08	chr9	81617229	81617529	1976	-0.88	2.54E-02	8.86E-01	Promoter (1-2kb)
Fbxo5	292263	chr1	42463404	42463704	3942	-0.89	2.55E-02	8.87E-01	Exon (NM_001106206/292263, exon 3 of 5)
Sohlh1	362085	chr3	3293843	3294143	958	-0.65	2.55E-02	8.87E-01	Promoter (<=1kb)
Syne3	299356	chr6	128693036	128693336	22725	-0.79	2.55E-02	8.87E-01	Intron (NM_001106762/299356, intron 1 of 23)
Pex11a	85249	chr1	141478842	141479142	2128	-1.07	2.55E-02	8.87E-01	Promoter (2-3kb)
Taf5l	307927	chr19	56795721	56796021	4777	-1.03	2.56E-02	8.87E-01	Intron (NM_001107442/307927, intron 1 of 4)
Foxj2	502886	chr4	155659395	155659695	5677	-0.51	2.56E-02	8.87E-01	Intron (NM_001109352/502886, intron 1 of 10)
Hyal2	64468	chr8	116328268	116328568	2982	-0.99	2.56E-02	8.87E-01	Promoter (2-3kb)
Adcy6	25289	chr7	140283422	140283722	7074	1.16	2.56E-02	8.87E-01	Intron (NM_012821/25289, intron 2 of 21)
Plxdc1	303505	chr10	85938002	85938302	0	0.61	2.57E-02	8.87E-01	Promoter (<=1kb)
Steap3	170824	chr13	36289815	36290115	-15669	-0.73	2.57E-02	8.87E-01	Distal Intergenic
Mat2a	171347	chr4	100315044	100315344	-11997	-1.03	2.58E-02	8.87E-01	Distal Intergenic
Ablim3	307395	chr18	57245601	57245901	0	0.79	2.58E-02	8.87E-01	Promoter (<=1kb)
Tmem181	502228	chr1	47161992	47162292	-102	1.01	2.58E-02	8.87E-01	Promoter (<=1kb)
Fer	301737	chr9	111562222	111562522	-87109	-0.91	2.59E-02	8.87E-01	Distal Intergenic
Panx1	315435	chr8	13577934	13578234	27806	-0.73	2.59E-02	8.87E-01	Intron (NM_001106801/300367, intron 11 of 11)
RT1-DOb	365542	chr20	4016141	4016441	-3944	0.59	2.59E-02	8.87E-01	Distal Intergenic
Socs6	307200	chr18	85987572	85987872	-6739	-1.28	2.59E-02	8.87E-01	Distal Intergenic
Slc4a1ap	298805	chr6	26156138	26156438	84899	-1.02	2.59E-02	8.87E-01	Distal Intergenic
Cmas	312826	chr4	176996268	176996568	2139	-0.86	2.60E-02	8.87E-01	Promoter (2-3kb)
Lepr	24536	chr5	120507073	120507373	3598	-0.68	2.60E-02	8.87E-01	Exon (NM_020099/56766, exon 3 of 4)
Slc38a4	170573	chr7	138469250	138469550	43402	-0.86	2.60E-02	8.87E-01	Intron (NM_130748/170573, intron 13 of 15)
Atf6	304962	chr13	89237895	89238195	4265	0.96	2.60E-02	8.87E-01	Intron (NM_001107196/304962, intron 1 of 15)
Dmbx1	313512	chr5	134814949	134815249	-38702	0.76	2.61E-02	8.90E-01	Distal Intergenic
Krt8	25626	chr7	143576530	143576830	26915	1.4	2.62E-02	8.91E-01	Distal Intergenic
Ugcg	83626	chr5	76393925	76394225	7087	-0.95	2.62E-02	8.91E-01	Intron (NM_031795/83626, intron 1 of 8)
Samd4a	305826	chr15	23776751	23777051	-787	0.85	2.62E-02	8.91E-01	Promoter (<=1kb)
Hspb7	50565	chr5	159971563	159971863	3486	0.94	2.63E-02	8.91E-01	3' UTR
Mapk14	81649	chr20	5940196	5940496	6893	-0.87	2.63E-02	8.91E-01	Intron (NM_031020/81649, intron 1 of 11)
Ptp4a2	85237	chr5	148038247	148038547	-12579	0.91	2.63E-02	8.91E-01	Distal Intergenic
Kbtbd11	306617	chr16	79734580	79734880	-33588	-0.82	2.64E-02	8.92E-01	Distal Intergenic
Nr3c2	25672	chr19	35074236	35074536	-313233	-0.78	2.64E-02	8.92E-01	Distal Intergenic
Bcl2l11	64547	chr3	120823601	120823901	96695	1.31	2.64E-02	8.92E-01	Distal Intergenic
Slc35d1	298280	chr5	122730000	122730300	16515	1.13	2.65E-02	8.92E-01	Intron (NM_001106668/298280, intron 8 of 11)
RGD1310352	303122	chr10	37759317	37759617	2171	-0.88	2.65E-02	8.92E-01	Promoter (2-3kb)
Specc1	303208	chr10	48389156	48389456	148826	1.17	2.65E-02	8.92E-01	Intron (NM_001039017/303208, intron 3 of 7)
Tnpo1	309126	chr2	29327905	29328205	-206801	1.12	2.65E-02	8.92E-01	Distal Intergenic
Bckdhb	29711	chr8	91418091	91418391	-45838	-1	2.66E-02	8.92E-01	Distal Intergenic
P4ha2	360526	chr10	39446933	39447233	11706	0.87	2.66E-02	8.92E-01	Intron (NM_001108275/360526, intron 5 of 15)
Mst1r	300999	chr8	116686518	116686818	0	0.7	2.66E-02	8.92E-01	Promoter (<=1kb)
Slc25a25	246771	chr3	11452823	11453123	-294	-0.68	2.66E-02	8.92E-01	Promoter (<=1kb)
Lrrc20	499430	chr20	31095614	31095914	-300	-0.7	2.66E-02	8.92E-01	Promoter (<=1kb)
Nfil3	114519	chr17	12129823	12130123	-130979	1.02	2.67E-02	8.92E-01	Intron (NM_001107339/306782, intron 7 of 8)
Lmx1a	289201	chr13	85889800	85890100	-28152	1.35	2.67E-02	8.92E-01	Distal Intergenic
Tmem158	117582	chr8	132328837	132329137	16571	1.4	2.67E-02	8.92E-01	Distal Intergenic
Ap2m1	116563	chr11	84045643	84045943	1599	-0.97	2.67E-02	8.92E-01	Promoter (1-2kb)
Dusp10	63995	chr13	104309280	104309580	24620	0.92	2.68E-02	8.93E-01	Intron (NM_001105734/63995, intron 2 of 3)
Acvr1	79558	chr3	44549428	44549728	-48442	1.41	2.68E-02	8.95E-01	Distal Intergenic
Tes	500040	chr4	44324185	44324485	2302	-1.07	2.69E-02	8.96E-01	Promoter (2-3kb)
Crnkl1	1.01E+08	chr3	140751567	140751867	-609888	-1.02	2.70E-02	8.97E-01	Distal Intergenic
Fam149b1	289900	chr15	4411028	4411328	-11439	-1.27	2.70E-02	8.97E-01	Distal Intergenic
Dhrs3	313689	chr5	162911801	162912101	102711	0.8	2.70E-02	8.97E-01	Intron (NM_001108006/313825, intron 66 of 69)
Atp2a2	29693	chr12	39544354	39544654	-9249	0.85	2.70E-02	8.97E-01	Distal Intergenic
Maea	298982	chr14	82690666	82690966	6707	0.98	2.72E-02	9.00E-01	Intron (NM_001008319/298982, intron 1 of 8)
Sesn2	502988	chr5	150700738	150701038	2808	-0.77	2.72E-02	9.01E-01	Promoter (2-3kb)
Casp6	83584	chr2	235392244	235392544	50870	0.96	2.73E-02	9.01E-01	Distal Intergenic
Ptpro	50677	chr4	171176373	171176673	-74570	0.72	2.73E-02	9.01E-01	Distal Intergenic
Il9r	24500	chr10	15715013	15715313	17786	0.88	2.73E-02	9.01E-01	Distal Intergenic
Uqcr10	685322	chr14	84909735	84910035	27706	1.6	2.74E-02	9.01E-01	Intron (NM_001109091/498402, intron 14 of 18)
Pdia4	116598	chr4	77486977	77487277	2258	1.28	2.74E-02	9.01E-01	Promoter (2-3kb)
Alcam	79559	chr11	50896526	50896826	115399	-0.9	2.74E-02	9.01E-01	Intron (NM_031753/79559, intron 1 of 15)
Ifit1	56824	chr1	252959556	252959856	15452	0.74	2.74E-02	9.01E-01	Distal Intergenic
Nat8f5	114020	chr4	117542882	117543182	-11402	-0.76	2.75E-02	9.01E-01	Distal Intergenic
Ncor2	360801	chr12	36944050	36944350	72051	0.73	2.75E-02	9.01E-01	Exon (NM_001108334/360801, exon 6 of 47)
Cbln1	498922	chr19	20501543	20501843	-105664	1.3	2.75E-02	9.01E-01	Distal Intergenic
Slc29a4	288499	chr12	13887241	13887541	36965	-0.89	2.76E-02	9.01E-01	Intron (NM_001107123/304302, intron 17 of 34)
Camk1g	171358	chr13	112115984	112116284	-16648	1.3	2.76E-02	9.01E-01	Distal Intergenic
Adcy8	29241	chr7	105594049	105594349	-1245	-0.77	2.76E-02	9.01E-01	Promoter (1-2kb)
Ctif	364900	chr18	71843356	71843656	-141933	0.85	2.76E-02	9.01E-01	Distal Intergenic
Tmem170b	361230	chr17	22960961	22961261	-96995	0.95	2.76E-02	9.01E-01	Distal Intergenic
Ddb1	64470	chr1	226660157	226660457	2596	-1.07	2.77E-02	9.01E-01	Promoter (2-3kb)
Comp	25304	chr16	20824242	20824542	-17172	-0.87	2.77E-02	9.01E-01	Distal Intergenic
Hist1h2bo	291157	chr17	44755872	44756172	1998	0.81	2.77E-02	9.01E-01	Promoter (1-2kb)
Mir16	1E+08	chr2	165608637	165608937	2565	-0.9	2.78E-02	9.01E-01	Promoter (2-3kb)
Oaf	315594	chr8	47520941	47521241	8448	-0.76	2.78E-02	9.01E-01	Intron (NM_001014090/315594, intron 1 of 3)
Sh3bp5	117186	chr16	7525051	7525351	12850	-0.92	2.78E-02	9.02E-01	Intron (NM_054011/117186, intron 2 of 8)
Ahsp	293522	chr1	199730086	199730386	10048	-0.84	2.79E-02	9.02E-01	Distal Intergenic
Dhx34	684903	chr1	78098440	78098740	13748	1.24	2.79E-02	9.04E-01	Intron (NM_001271452/684903, intron 7 of 15)
Lrrc20	499430	chr20	31094531	31094831	-1383	-0.9	2.80E-02	9.04E-01	Promoter (1-2kb)
Med18	682988	chr5	150605349	150605649	48459	1.2	2.80E-02	9.04E-01	Distal Intergenic
Rpl35al1	57809	chr12	7086701	7087001	99873	-1.12	2.80E-02	9.04E-01	Distal Intergenic
Ak2	24184	chr5	147213590	147213890	28116	-0.86	2.80E-02	9.04E-01	Distal Intergenic
Rpia	362383	chr4	98590635	98590935	2729	-0.79	2.81E-02	9.05E-01	Promoter (2-3kb)
Zfp202	500981	chr8	44049991	44050291	2399	-0.88	2.81E-02	9.05E-01	Promoter (2-3kb)
Zp3	114639	chr12	23757576	23757876	-15386	0.83	2.81E-02	9.05E-01	Distal Intergenic
Klf13	499171	chr1	124803065	124803365	0	0.69	2.82E-02	9.05E-01	Promoter (<=1kb)
Ptprm	29616	chr9	115265108	115265408	289853	-1.01	2.82E-02	9.06E-01	Intron (NM_001168632/29616, intron 4 of 32)
Rell1	289635	chr14	45985202	45985502	2891	-0.86	2.83E-02	9.06E-01	Promoter (2-3kb)
Qsox2	681023	chr3	3689688	3689988	1984	-0.78	2.83E-02	9.06E-01	Promoter (1-2kb)

Xylt2	64134	chr10	82396715	82397015	2470	1.14	2.83E-02	9.06E-01	Promoter (2-3kb)
Ece2	408243	chr11	83963957	83964257	0	0.68	2.83E-02	9.06E-01	Promoter (<=1kb)
Npas2	316351	chr9	45968735	45969035	66994	-0.87	2.84E-02	9.06E-01	Intron (NM_001108214/316351, intron 1 of 20)
Gmfb	81661	chr15	23612431	23612731	-1541	-0.92	2.84E-02	9.06E-01	Promoter (1-2kb)
Tob1	170842	chr10	81846176	81846476	-67213	0.85	2.85E-02	9.06E-01	Distal Intergenic
Prkg1	54286	chr1	250297136	250297436	3516	-0.93	2.85E-02	9.06E-01	Intron (NM_001105731/54286, intron 1 of 16)
Bmp2	29373	chr3	126333291	126333591	-2594	-0.74	2.85E-02	9.06E-01	Promoter (2-3kb)
Cd14	60350	chr18	29544473	29544773	17186	-1.05	2.85E-02	9.06E-01	Distal Intergenic
Rad54l2	363135	chr8	115491846	115492146	-1206	-0.78	2.85E-02	9.06E-01	Promoter (1-2kb)
Pag1	64019	chr2	94267466	94267766	1362	0.94	2.86E-02	9.06E-01	Promoter (1-2kb)
Dhx33	287464	chr10	57638673	57638973	-188	-0.76	2.86E-02	9.06E-01	Promoter (<=1kb)
Rreb1	306873	chr17	27711696	27711996	-46430	0.66	2.86E-02	9.06E-01	Distal Intergenic
Zfp467	500110	chr4	78024486	78024786	50695	-0.91	2.86E-02	9.06E-01	Distal Intergenic
Spast	362700	chr6	22282318	22282618	-432	-0.52	2.87E-02	9.06E-01	Promoter (<=1kb)
Rhpn2	308516	chr1	91599577	91599877	3180	-1	2.87E-02	9.06E-01	Intron (NM_001107505/308516, intron 1 of 15)
Scfd2	498353	chr14	36232449	36232749	16447	1.04	2.87E-02	9.06E-01	Intron (NM_001017499/498353, intron 2 of 8)
Cdkn1b	83571	chr4	168711233	168711533	22070	0.77	2.87E-02	9.06E-01	Distal Intergenic
LOC499469	499469	chr20	46736686	46736986	29324	0.74	2.87E-02	9.06E-01	Intron (NM_001106396/294518, intron 1 of 7)
Grik4	24406	chr8	47236152	47236452	-128235	0.77	2.88E-02	9.06E-01	Distal Intergenic
Rnf182	498726	chr17	24242613	24242913	0	0.68	2.88E-02	9.06E-01	Promoter (<=1kb)
Rps6ka2	117269	chr1	53359416	53359716	-441	0.66	2.88E-02	9.06E-01	Promoter (<=1kb)
LOC100910620	1.01E+08	chr5	74459306	74459606	-16634	0.89	2.88E-02	9.06E-01	Distal Intergenic
Col1a1	29393	chr10	82742783	82743083	-2718	1.02	2.89E-02	9.06E-01	Promoter (2-3kb)
Mrpl22	287302	chr10	43604123	43604423	2337	-1.09	2.89E-02	9.06E-01	Promoter (2-3kb)
Cds1	81925	chr14	9391033	9391333	65631	1.16	2.90E-02	9.06E-01	Distal Intergenic
Ppm1g	259229	chr6	26520429	26520729	2589	-1.03	2.90E-02	9.06E-01	Promoter (2-3kb)
Anks1b	314721	chr7	30709625	30709925	202490	-0.77	2.90E-02	9.06E-01	Intron (NM_001271371/314721, intron 8 of 30)
Tjap1	316233	chr9	17096301	17096601	9826	1.15	2.91E-02	9.06E-01	Intron (NM_001108203/316233, intron 2 of 11)
Oas1b	246268	chr12	41266016	41266316	114	0.62	2.91E-02	9.06E-01	Promoter (<=1kb)
Ceacam19	680640	chr1	80797537	80797837	11932	0.7	2.91E-02	9.06E-01	Distal Intergenic
Cd48	245962	chr13	90118953	90119253	2110	-0.84	2.92E-02	9.06E-01	Promoter (2-3kb)
Ppp4r3a	314388	chr6	125027289	125027589	55403	0.55	2.92E-02	9.06E-01	Distal Intergenic
Foxo3	294515	chr20	46368892	46369192	-58886	0.86	2.92E-02	9.06E-01	Distal Intergenic
Arhgef19	362648	chr5	159761473	159761773	5938	0.74	2.93E-02	9.06E-01	Intron (NM_001108692/362648, intron 3 of 17)
Tnfaip2	299339	chr6	135911603	135911903	20672	-1.21	2.93E-02	9.06E-01	Distal Intergenic
Gpr3	266769	chr5	151369264	151369564	26943	-0.9	2.93E-02	9.06E-01	Intron (NM_001013167/313024, intron 2 of 13)
Rhbdd3	289753	chr14	85353098	85353398	1740	0.95	2.93E-02	9.06E-01	Promoter (1-2kb)
Tor4a	311795	chr3	2411675	2411975	-131	-1.08	2.93E-02	9.06E-01	Promoter (<=1kb)
Zkscan1	498160	chr12	19220549	19220849	-10243	1.02	2.93E-02	9.06E-01	Distal Intergenic
Ptgfrn	29602	chr2	203519879	203520179	-25488	1.1	2.93E-02	9.06E-01	Distal Intergenic
Ipo13	116458	chr5	136750409	136750709	-1452	-1.08	2.94E-02	9.08E-01	Promoter (1-2kb)
Ccnk	500715	chr6	132128931	132129231	38298	-0.78	2.95E-02	9.08E-01	Distal Intergenic
Pacsin2	124461	chr7	124243924	124244224	42254	-0.99	2.95E-02	9.08E-01	Intron (NM_130740/124461, intron 2 of 10)
Mrps5	296134	chr3	120163511	120163811	-2142	-0.87	2.95E-02	9.08E-01	Promoter (2-3kb)
Tpmt	690050	chr17	18030025	18030325	-903	-0.92	2.96E-02	9.10E-01	Promoter (<=1kb)
Nek1	290705	chr16	32441152	32441452	-1731	0.96	2.96E-02	9.10E-01	Promoter (1-2kb)
Abhd5	316122	chr8	130933395	130933695	-39527	-1	2.97E-02	9.10E-01	Distal Intergenic
Phf10	292404	chr1	56969690	56969990	-1797	-1.18	2.97E-02	9.10E-01	Promoter (1-2kb)
Pir	363465	chrX	31965957	31966257	1895	-1.02	2.97E-02	9.10E-01	Promoter (1-2kb)
Notch3	56761	chr7	14185059	14185359	4329	0.82	2.97E-02	9.10E-01	Intron (NM_020087/56761, intron 2 of 34)
Pde4a	25638	chr8	22184589	22184889	-4644	-0.98	2.97E-02	9.11E-01	Distal Intergenic
Pknox2	680549	chr8	39401789	39402089	58755	0.96	2.98E-02	9.11E-01	Intron (NM_001271279/680549, intron 6 of 14)
Smad2	29357	chr18	72741494	72741794	191040	-0.64	2.99E-02	9.11E-01	Distal Intergenic
Klf4	114505	chr5	72885629	72885929	-597960	0.84	2.99E-02	9.11E-01	Distal Intergenic
LOC498933	498933	chr19	32382866	32383166	38788	1.12	2.99E-02	9.11E-01	Distal Intergenic
Ddn	25113	chr7	140479700	140480000	3693	0.91	2.99E-02	9.11E-01	3' UTR
Zbtb10	80338	chr2	94546520	94546820	184156	-1.19	3.00E-02	9.12E-01	Distal Intergenic
Tmem38a	306327	chr16	18957129	18957429	17516	1.14	3.00E-02	9.13E-01	Downstream (2-3kb)
Asah2	114104	chr1	250774268	250774568	-143539	-0.7	3.01E-02	9.13E-01	Intron (NM_181386/353229, intron 5 of 9)
Lrrc58	303919	chr11	65756125	65756425	3133	-0.68	3.01E-02	9.13E-01	Intron (NM_001195558/303919, intron 1 of 3)
Ppil2	360746	chr11	88149291	88149591	1914	-1	3.01E-02	9.13E-01	Promoter (1-2kb)
Zmynd11	291259	chr17	63911126	63911704	2140	-0.8	3.01E-02	9.13E-01	Promoter (2-3kb)
Gmnn	291137	chr17	42305580	42305880	3040	-0.91	3.02E-02	9.13E-01	Exon (NM_001106112/291137, exon 3 of 7)
Sectm1b	287884	chr10	110260431	110260731	-503	-0.64	3.02E-02	9.13E-01	Promoter (<=1kb)
Vgll4	297523	chr4	146730269	146730569	108828	1.08	3.02E-02	9.13E-01	Intron (NM_001012097/312647, intron 17 of 17)
Ankrd46	299982	chr7	75212849	75213149	87257	0.83	3.04E-02	9.17E-01	Distal Intergenic
Ankle2	360829	chr12	52504235	52504535	2591	-0.95	3.04E-02	9.18E-01	Promoter (2-3kb)
Hist1h2bo	291157	chr17	44706049	44706349	51821	0.68	3.05E-02	9.18E-01	Distal Intergenic
Rabggta	58983	chr15	34403356	34403656	-2934	1.28	3.05E-02	9.18E-01	Promoter (2-3kb)
Cluh	303300	chr10	61436025	61436325	3153	-0.94	3.05E-02	9.18E-01	Intron (NM_001305213/303300, intron 1 of 25)
Rbm8a	295284	chr2	198753500	198753800	-1462	-0.81	3.06E-02	9.20E-01	Promoter (1-2kb)
Aqp1	25240	chr4	85553058	85553358	1555	1.13	3.07E-02	9.22E-01	Promoter (1-2kb)
Dcaf5	314273	chr6	103792762	103793062	-187506	0.68	3.07E-02	9.22E-01	Distal Intergenic
B3gntl1	367384	chr10	110861457	110862010	1011	-0.79	3.08E-02	9.23E-01	Promoter (1-2kb)
Pgam1	24642	chr1	261081415	261081715	-76489	-0.93	3.08E-02	9.23E-01	Distal Intergenic
Sympk	292683	chr1	79930564	79930864	-292	0.76	3.08E-02	9.23E-01	Promoter (<=1kb)
Dsn1	499933	chr3	153167889	153168189	-53389	-0.79	3.09E-02	9.23E-01	Distal Intergenic
Nr2f1	81808	chr2	5582286	5582586	-2392	-0.57	3.09E-02	9.24E-01	Promoter (2-3kb)
Nfia	25492	chr5	116468950	116469250	47052	-0.9	3.09E-02	9.24E-01	Intron (NM_012988/25492, intron 1 of 9)
Vwa5b2	303812	chr11	84008117	84008417	529	0.51	3.10E-02	9.24E-01	Promoter (<=1kb)
Cdkn2aip	306455	chr16	47657852	47658152	-7664	1.07	3.10E-02	9.24E-01	Distal Intergenic
F7	260320	chr16	81867307	81867607	-32384	0.59	3.10E-02	9.24E-01	Exon (NM_053951/117020, exon 7 of 31)
Thnsl1	498805	chr17	88140475	88140775	44645	1.16	3.10E-02	9.24E-01	Distal Intergenic
Ncmap	689826	chr5	153752666	153752966	-15505	-0.87	3.11E-02	9.24E-01	Distal Intergenic
Septin11	305227	chr14	16407873	16408173	44717	-0.79	3.11E-02	9.24E-01	Intron (NM_001107208/305227, intron 1 of 10)
Nuf2	304951	chr13	87847562	87847862	-339	-0.5	3.11E-02	9.24E-01	Promoter (<=1kb)
Rnf144b	364681	chr17	17894724	17895024	52753	1.13	3.12E-02	9.25E-01	Intron (NM_001108881/364681, intron 1 of 7)
Tars1	294810	chr2	61414323	61414623	-285	-0.58	3.12E-02	9.25E-01	Promoter (<=1kb)
Nt5e	58813	chr8	95926897	95927197	-41805	1.31	3.12E-02	9.25E-01	Distal Intergenic
Ttc17	311224	chr3	83289850	83290150	16440	-0.85	3.13E-02	9.27E-01	Intron (NM_001107752/311224, intron 1 of 24)
Bbc3	317673	chr1	78264069	78264369	2564	0.55	3.13E-02	9.27E-01	Promoter (2-3kb)
Bend5	362564	chr5	131317661	131317961	60534	0.96	3.14E-02	9.27E-01	Intron (NM_001350232/100911395, intron 6 of 12)
Slc1a3	29483	chr2	57909594	57909894	25448	-0.85	3.14E-02	9.27E-01	Intron (NM_019225/29483, intron 3 of 9)
Rfc1	89809	chr14	44616792	44617092	-10499	1.03	3.14E-02	9.27E-01	Distal Intergenic

RGD1561161	294747	chr2	45171260	45171560	-10831	0.95	3.14E-02	9.27E-01	Distal Intergenic
Fv1	308568	chr1	100218705	100219005	-1840	0.87	3.15E-02	9.28E-01	Promoter (1-2kb)
LOC100361083	1E+08	chr5	141112944	141113244	-11981	0.83	3.16E-02	9.29E-01	Intron (NM_001135758/362587, intron 57 of 97)
Ergic3	296306	chr3	151553122	151553422	-170	-0.8	3.16E-02	9.29E-01	Promoter (<=1kb)
Trarg1	360576	chr10	63552309	63552609	-60596	0.67	3.17E-02	9.30E-01	Distal Intergenic
Casp2	64314	chr4	71651571	71651871	-561	0.61	3.17E-02	9.30E-01	Promoter (<=1kb)
Lca5	300866	chr8	90977279	90977579	6645	-0.93	3.17E-02	9.30E-01	Intron (NM_001013954/300866, intron 2 of 10)
Mast3	688540	chr16	20396217	20396517	-4060	1.17	3.18E-02	9.30E-01	Distal Intergenic
F13a1	60327	chr17	28855259	28855559	350609	-0.69	3.18E-02	9.30E-01	Distal Intergenic
Dcaf6	289181	chr13	83678078	83678378	1952	-1.26	3.19E-02	9.30E-01	Promoter (1-2kb)
Dmbx1	313512	chr5	134807168	134807468	-30921	0.9	3.19E-02	9.30E-01	Distal Intergenic
Dlg4	29495	chr10	56629772	56630072	2225	0.71	3.19E-02	9.30E-01	Promoter (2-3kb)
Tent5b	313019	chr5	151707902	151708202	15834	1.06	3.19E-02	9.30E-01	Distal Intergenic
Pten	50557	chr1	251425403	251425703	3589	-0.74	3.20E-02	9.30E-01	Intron (NM_031606/50557, intron 1 of 8)
Grin3a	191573	chr5	66404852	66405152	-1331840	1.23	3.20E-02	9.30E-01	Distal Intergenic
Npepps	50558	chr10	85239742	85240042	-16881	0.98	3.20E-02	9.30E-01	Distal Intergenic
Slc35f5	288993	chr13	41873910	41874210	-8927	0.8	3.21E-02	9.31E-01	Distal Intergenic
Zfp24	360204	chr18	15905474	15905774	15907	0.61	3.21E-02	9.31E-01	Distal Intergenic
Nppa	24602	chr5	164814107	164814407	5700	-1.19	3.22E-02	9.31E-01	3' UTR
Rtraf	302247	chr15	4871725	4872025	-11008	-0.91	3.22E-02	9.31E-01	Distal Intergenic
LOC304725	304725	chr13	13322100	13322400	2073217	1.44	3.22E-02	9.31E-01	Distal Intergenic
Irak3	314870	chr7	64983159	64983459	-935	0.98	3.22E-02	9.31E-01	Promoter (<=1kb)
Htr2a	29595	chr15	56668181	56668481	2029	0.76	3.23E-02	9.31E-01	Promoter (2-3kb)
Olr1666	291929	chr19	27667636	27667936	-131844	-0.88	3.23E-02	9.31E-01	Distal Intergenic
Ubn1	302935	chr10	10722185	10722485	3170	-0.75	3.23E-02	9.31E-01	Intron (NM_001106977/302935, intron 1 of 17)
Fzd2	64512	chr10	90499694	90499994	-50153	-0.96	3.23E-02	9.31E-01	Distal Intergenic
Snx33	315696	chr8	61574814	61575114	19918	0.79	3.24E-02	9.32E-01	Distal Intergenic
Uchl1	29545	chr14	43227046	43227346	-83104	1.2	3.24E-02	9.32E-01	Distal Intergenic
Prkci	84006	chr2	115970006	115970306	32244	0.7	3.24E-02	9.32E-01	Intron (NM_032059/84006, intron 2 of 17)
Prss8	192107	chr1	199381759	199382059	-4724	-0.57	3.25E-02	9.32E-01	Intron (NM_001011560/497040, intron 10 of 14)
Bckdhb	29711	chr8	92001525	92001825	537296	-1.02	3.25E-02	9.32E-01	Distal Intergenic
Kat6a	306571	chr16	74019848	74020148	602	-1.08	3.25E-02	9.32E-01	Promoter (<=1kb)
Mtnr1a	114211	chr16	50427970	50428270	-69161	-0.7	3.26E-02	9.32E-01	Intron (NM_031819/83720, intron 3 of 26)
Arpc2	301511	chr9	81516810	81517110	-1085	-1.06	3.26E-02	9.32E-01	Promoter (1-2kb)
Mical2	365352	chr1	177113424	177113724	20037	1.15	3.26E-02	9.32E-01	Intron (NM_001139508/365352, intron 1 of 17)
Mcmbp	309009	chr1	200163317	200163617	-211	0.88	3.26E-02	9.32E-01	Promoter (<=1kb)
Nanp	311530	chr3	146815213	146815513	-2225	1.32	3.27E-02	9.32E-01	Promoter (2-3kb)
Creb3l2	362339	chr4	64944970	64945270	36249	0.95	3.27E-02	9.32E-01	Intron (NM_001012188/362339, intron 1 of 11)
Abcc4	170924	chr15	103925294	103925594	1998	-0.94	3.27E-02	9.32E-01	Promoter (1-2kb)
Pak2	29432	chr11	71968108	71968408	8859	0.73	3.27E-02	9.32E-01	Intron (NM_053306/29432, intron 1 of 14)
Foxj2	502886	chr4	155645664	155645964	-7754	1	3.27E-02	9.32E-01	Distal Intergenic
Tprg1	360731	chr11	78730237	78730537	131208	-1.16	3.28E-02	9.34E-01	Downstream (2-3kb)
Eps8l3	295361	chr2	210620840	210621140	-47263	-0.96	3.29E-02	9.34E-01	Distal Intergenic
Sh3pxd2a	309460	chr1	267079661	267079961	124025	0.74	3.29E-02	9.35E-01	Intron (NM_001107606/309460, intron 7 of 13)
Bfsp1	25394	chr3	137944331	137944631	25027	1.26	3.30E-02	9.35E-01	Intron (NM_031555/25394, intron 5 of 7)
Prelp	84400	chr13	50801989	50802289	-40711	-0.77	3.30E-02	9.35E-01	Distal Intergenic
Dlg4	29495	chr10	56626571	56626871	-676	-1.23	3.30E-02	9.36E-01	Promoter (<=1kb)
Metrnl	316842	chr10	110939134	110939434	45873	0.82	3.31E-02	9.36E-01	Distal Intergenic
Gng11	64199	chr4	31098875	31099175	-288245	1.12	3.31E-02	9.36E-01	Intron (NM_019234/29564, intron 14 of 16)
Gpr141	291179	chr17	47219780	47220080	-20937	1.3	3.31E-02	9.36E-01	Distal Intergenic
Efhc2	302507	chrX	5888157	5888457	62376	-0.6	3.32E-02	9.38E-01	Intron (NM_001106952/302507, intron 2 of 14)
Npc1	266732	chr18	3648320	3648620	14036	-0.81	3.33E-02	9.38E-01	Intron (NM_153624/266732, intron 1 of 24)
Bpgm	296973	chr4	61914596	61914896	2386	1.12	3.33E-02	9.39E-01	Promoter (2-3kb)
Taf8	316216	chr9	15506686	15506986	-6065	0.92	3.34E-02	9.39E-01	Distal Intergenic
Polr3c	310685	chr2	198850391	198850691	1470	-0.92	3.34E-02	9.39E-01	Promoter (1-2kb)
Armcx1	501619	chrX	105835772	105836072	-75853	-0.86	3.34E-02	9.39E-01	Distal Intergenic
Snca	29219	chr4	90518290	90518590	363668	-0.55	3.35E-02	9.39E-01	Distal Intergenic
Timm22	79463	chr10	64558023	64558323	1959	-1.14	3.36E-02	9.41E-01	Promoter (1-2kb)
RGD1359158	361740	chr1	239263777	239264077	1920	-0.84	3.36E-02	9.42E-01	Promoter (1-2kb)
Eif4b	300253	chr7	143700892	143701192	21236	-0.95	3.37E-02	9.42E-01	3' UTR
Pla2g4a	24653	chr13	67186456	67186756	19932	0.74	3.37E-02	9.42E-01	Intron (NM_133551/24653, intron 1 of 17)
Peli1	305549	chr14	105900490	105900790	-107296	1.31	3.38E-02	9.43E-01	Distal Intergenic
Rprd1b	311591	chr3	154505005	154505305	-1730	-0.91	3.38E-02	9.43E-01	Promoter (1-2kb)
Sod1	24786	chr11	30322110	30322410	-40872	1.1	3.38E-02	9.43E-01	Distal Intergenic
Ptgs1	24693	chr3	15563844	15564144	3121	0.91	3.38E-02	9.43E-01	Intron (NM_017043/24693, intron 2 of 10)
Kmt5a	689820	chr12	37575663	37575963	-913	-1.46	3.39E-02	9.43E-01	Promoter (<=1kb)
Mical2	365352	chr1	177098435	177098735	5048	-1.23	3.39E-02	9.44E-01	Intron (NM_001139508/365352, intron 1 of 17)
Coro1c	501841	chr12	48489987	48490287	-19455	-1.09	3.40E-02	9.45E-01	Distal Intergenic
Grina	266668	chr7	117312572	117312872	7830	0.68	3.40E-02	9.45E-01	Distal Intergenic
Syndig1	362235	chr3	145553962	145554262	521785	0.83	3.41E-02	9.45E-01	Distal Intergenic
Parp3	300985	chr8	115176298	115176598	2593	1.21	3.41E-02	9.46E-01	Promoter (2-3kb)
Lyrm7	686506	chr10	40202330	40202630	-338	-0.59	3.41E-02	9.46E-01	Promoter (<=1kb)
Ccnd1	58919	chr1	218130088	218130388	-29816	1.32	3.42E-02	9.48E-01	Distal Intergenic
Ccnl1	114121	chr2	157856576	157856876	-96738	1.26	3.43E-02	9.49E-01	Distal Intergenic
Ankh	114506	chr2	79845187	79845487	-286079	-0.78	3.44E-02	9.50E-01	Distal Intergenic
Otos	246044	chr9	99878452	99878752	-56498	1	3.44E-02	9.50E-01	Intron (NM_001009825/301618, intron 5 of 9)
Ppm1d	287585	chr10	72985467	72985767	75917	0.85	3.45E-02	9.50E-01	Intron (NM_001173430/363662, intron 13 of 17)
Map3k10	308463	chr1	84639398	84639698	-147919	1.52	3.45E-02	9.50E-01	Distal Intergenic
S100a3	114216	chr2	189977177	189977477	-15785	0.94	3.46E-02	9.50E-01	Distal Intergenic
Mkln1	83536	chr4	58576903	58577203	-116181	-0.78	3.46E-02	9.50E-01	Distal Intergenic
Batf3	60462	chr13	109740661	109740961	27172	-0.68	3.46E-02	9.50E-01	Distal Intergenic
LOC500846	500846	chr7	69217646	69217946	-112696	-1.01	3.46E-02	9.50E-01	Distal Intergenic
Poll	361767	chr1	265281098	265281398	17399	0.79	3.46E-02	9.50E-01	Distal Intergenic
Ttc17	311224	chr3	83288473	83288773	17817	0.97	3.46E-02	9.50E-01	Intron (NM_001107752/311224, intron 1 of 24)
Ptpro	50677	chr4	171151190	171151490	-99753	1	3.46E-02	9.50E-01	Distal Intergenic
RT1-A1	24973	chr20	5414516	5414816	21	0.84	3.47E-02	9.50E-01	Promoter (<=1kb)
Oxct2a	366463	chr5	140809744	140810044	-3913	0.84	3.48E-02	9.51E-01	Distal Intergenic
Osgepl1	314548	chr9	53097798	53098098	0	-0.64	3.49E-02	9.53E-01	Promoter (<=1kb)
Fgfrl1	360903	chr14	2034640	2034940	-2471	-0.52	3.49E-02	9.53E-01	Promoter (2-3kb)
Nedd4	25489	chr8	79329717	79330017	6309	-0.77	3.50E-02	9.55E-01	Intron (NM_012986/25489, intron 1 of 28)
Sf3b2	293671	chr1	220665847	220666147	-236	-0.54	3.52E-02	9.58E-01	Promoter (<=1kb)
Cdt1	292071	chr19	55384911	55385211	3346	-0.9	3.52E-02	9.58E-01	Exon (NM_001106192/292071, exon 8 of 10)
Mgat3	29582	chr7	121403184	121403484	-5345	1.05	3.53E-02	9.59E-01	Distal Intergenic
Hspbap1	171460	chr11	68195883	68196183	1600	-0.83	3.53E-02	9.59E-01	Promoter (1-2kb)

Gpatch2	289362	chr13	105683848	105684148	-339	-0.88	3.54E-02	9.59E-01	Promoter (<=1kb)
Kcnq5	259273	chr9	27913741	27914041	227073	0.96	3.54E-02	9.59E-01	Intron (NM_001134643/259273, intron 1 of 14)
Trim16	303214	chr10	49233022	49233322	1061	0.74	3.54E-02	9.59E-01	Promoter (1-2kb)
Taok1	286993	chr10	62533898	62534198	-32444	-0.57	3.55E-02	9.59E-01	Distal Intergenic
LOC100360244	1E+08	chr15	61877074	61877374	-3668	-0.77	3.55E-02	9.59E-01	Intron (NM_001013051/290408, intron 2 of 12)
Vav3	295378	chr2	212493805	212494105	246354	-1.04	3.56E-02	9.59E-01	Intron (NM_001191714/295378, intron 19 of 26)
Cyp4v3	266761	chr16	50078049	50078349	-33454	1.04	3.56E-02	9.59E-01	Distal Intergenic
Exoc1	305287	chr14	34180659	34180959	-86	-0.92	3.56E-02	9.59E-01	Promoter (<=1kb)
Katna1	292464	chr1	1825667	1825967	-203	-0.53	3.57E-02	9.59E-01	Promoter (<=1kb)
Pdk1	116551	chr3	58814688	58814988	283818	-0.61	3.57E-02	9.59E-01	Distal Intergenic
Dapk3	64391	chr7	11402169	11402469	-1364	-0.82	3.57E-02	9.59E-01	Promoter (1-2kb)
Septin11	305227	chr14	16602153	16602453	-149263	0.96	3.58E-02	9.59E-01	Distal Intergenic
Efhc2	302507	chrX	5889640	5889940	63859	-0.93	3.58E-02	9.59E-01	Intron (NM_001106952/302507, intron 2 of 14)
Exoc6	50556	chr1	256294489	256294789	68305	1.89	3.58E-02	9.59E-01	Intron (NM_019277/50556, intron 17 of 21)
Kcnn4	65206	chr1	81226408	81226708	-4139	1	3.58E-02	9.59E-01	Distal Intergenic
Pofut2	309686	chr20	12134685	12134985	-5938	0.91	3.59E-02	9.59E-01	Distal Intergenic
Fam149b1	289900	chr15	4412380	4412680	-12791	-0.61	3.59E-02	9.59E-01	Distal Intergenic
LOC499544	499544	chr2	61978167	61978467	-13275	0.88	3.59E-02	9.59E-01	Distal Intergenic
Tlk1	311118	chr3	57183347	57183647	-79317	-0.56	3.59E-02	9.59E-01	Distal Intergenic
Mapre2	679221	chr18	14756685	14756985	-57165	-0.61	3.59E-02	9.59E-01	Distal Intergenic
Csrp1	29276	chr13	52435636	52435936	-117907	-1.16	3.60E-02	9.59E-01	Distal Intergenic
Patj	140581	chr5	116839574	116839874	-198756	0.93	3.60E-02	9.59E-01	Distal Intergenic
Eif4g2	361628	chr1	175977306	175977606	-81796	0.81	3.61E-02	9.59E-01	Distal Intergenic
Sde2	289315	chr13	99120514	99120814	-16107	0.52	3.61E-02	9.59E-01	Distal Intergenic
Med16	299607	chr7	12626466	12626766	6724	-0.82	3.61E-02	9.59E-01	Intron (NM_001106768/299607, intron 6 of 14)
Arpc2	301511	chr9	81528166	81528466	9971	-0.75	3.61E-02	9.59E-01	Intron (NM_001106919/301511, intron 3 of 10)
Hspb1	24471	chr12	23841315	23841615	-264	-0.65	3.61E-02	9.59E-01	Promoter (<=1kb)
Pacsin2	124461	chr7	124308554	124308854	-22076	0.85	3.61E-02	9.59E-01	Distal Intergenic
Nptx1	266777	chr10	108653052	108653352	38240	0.82	3.62E-02	9.60E-01	Distal Intergenic
Mxi1	25701	chr1	274064478	274064778	14802	-1.05	3.62E-02	9.60E-01	Intron (NM_013160/25701, intron 2 of 5)
Bnip3	84480	chr1	211262647	211262947	2335	1.2	3.62E-02	9.60E-01	Promoter (2-3kb)
Phf3	363210	chr9	36988804	36989104	154911	1.22	3.62E-02	9.60E-01	Distal Intergenic
Purb	498407	chr14	86732835	86733135	6200	-0.87	3.63E-02	9.60E-01	Distal Intergenic
Crmp1	25415	chr14	78308330	78308630	303	-1.12	3.63E-02	9.60E-01	Promoter (<=1kb)
P3h3	297595	chr4	157375353	157375653	-2492	-0.84	3.63E-02	9.60E-01	Promoter (2-3kb)
Sptssa	500651	chr6	75433203	75433503	118042	1.16	3.64E-02	9.60E-01	Distal Intergenic
Snf8	287645	chr10	83858440	83858740	2109	-0.86	3.64E-02	9.60E-01	Promoter (2-3kb)
Atp1a1	24211	chr2	204032240	204032540	-217	0.59	3.64E-02	9.60E-01	Promoter (<=1kb)
Nnat	94270	chr3	154050088	154050388	6215	0.95	3.64E-02	9.60E-01	Distal Intergenic
Klf3	114845	chr14	45191743	45192043	-26536	0.76	3.65E-02	9.61E-01	Distal Intergenic
Cmip	292051	chr19	49834168	49834468	41895	0.65	3.67E-02	9.62E-01	Intron (NM_001163273/292051, intron 1 of 20)
RGD1565212	498534	chr15	47511054	47511354	-172123	-0.77	3.67E-02	9.62E-01	Intron (NM_053307/29447, intron 5 of 8)
Kif3b	296284	chr3	148827658	148827958	54399	-1.04	3.67E-02	9.62E-01	Distal Intergenic
Dmtn	361069	chr15	52308713	52309013	11372	0.57	3.68E-02	9.63E-01	5' UTR
Robo3	315564	chr8	39931904	39932204	-24426	1.04	3.69E-02	9.63E-01	Distal Intergenic
Pgr	25154	chr8	7443286	7443586	314630	-1	3.69E-02	9.63E-01	Distal Intergenic
Tfpi	29436	chr3	71902168	71902468	-5749	0.99	3.70E-02	9.63E-01	Distal Intergenic
Kcnc1	25327	chr1	102411531	102411831	-2794	0.9	3.70E-02	9.63E-01	Promoter (2-3kb)
Gpx4	29328	chr7	12503509	12503809	14875	0.77	3.70E-02	9.63E-01	Intron (NM_001108068/314619, intron 6 of 32)
Vom2r80	502285	chr1	62135287	62135587	-276451	-0.56	3.71E-02	9.63E-01	Distal Intergenic
Atp6v1a	685232	chr11	61547244	61547544	15666	-0.79	3.71E-02	9.63E-01	Intron (NM_001108318/685232, intron 1 of 14)
Fam135a	367235	chr9	30208022	30208322	125654	1.63	3.71E-02	9.63E-01	Distal Intergenic
Eea1	314764	chr7	37069493	37069793	-31621	-0.6	3.71E-02	9.63E-01	Distal Intergenic
Cops8	363283	chr9	97820508	97820808	48284	1.05	3.71E-02	9.63E-01	Distal Intergenic
Spata2	114210	chr3	164236589	164236889	2361	-0.92	3.71E-02	9.63E-01	Promoter (2-3kb)
Epc1	1E+08	chr17	57388971	57389271	5704	0.87	3.71E-02	9.63E-01	Intron (NM_001309462/100362678, intron 1 of 13)
Adcy7	84420	chr19	19762346	19762646	-35265	0.58	3.72E-02	9.63E-01	Distal Intergenic
Nptn	56064	chr8	63377429	63377729	-1358	0.83	3.72E-02	9.63E-01	Promoter (1-2kb)
Dact2	308212	chr1	56147474	56147774	20577	-0.63	3.72E-02	9.63E-01	Distal Intergenic
Ppp4r4	500711	chr6	127436142	127436442	35557	0.74	3.72E-02	9.63E-01	Intron (NM_001134632/500711, intron 3 of 26)
Smad6	367100	chr8	68920370	68920670	45438	1.15	3.73E-02	9.63E-01	Intron (NM_001109002/367100, intron 3 of 3)
Tssk5	315095	chr7	117438079	117438379	1963	-1.66	3.73E-02	9.63E-01	Promoter (1-2kb)
Tent4a	306672	chr1	36391790	36392090	-8353	1	3.74E-02	9.63E-01	Distal Intergenic
Gne	114711	chr5	59536017	59536317	6784	1.14	3.74E-02	9.63E-01	Intron (NM_053765/114711, intron 2 of 11)
Mt2A	689415	chr19	11310033	11310333	-1293	0.81	3.75E-02	9.63E-01	Promoter (1-2kb)
Tmbim6	24822	chr7	141053200	141053500	-376	-0.71	3.75E-02	9.63E-01	Promoter (<=1kb)
Pgm3	363109	chr8	94241047	94241347	1868	-1	3.75E-02	9.63E-01	Promoter (1-2kb)
Arrb2	25388	chr10	57039225	57039525	-726	-0.87	3.75E-02	9.63E-01	Promoter (<=1kb)
Golga4	501069	chr8	127272698	127272998	101062	-0.91	3.75E-02	9.63E-01	Intron (NM_001106865/301056, intron 1 of 7)
Lgalsl	360983	chr14	105126705	105127005	-71284	-0.76	3.75E-02	9.63E-01	Distal Intergenic
Slc40a1	170840	chr9	52912186	52912486	0	-0.54	3.76E-02	9.63E-01	Promoter (<=1kb)
Foxp1	297480	chr4	131393373	131393673	204631	0.78	3.76E-02	9.63E-01	Intron (NM_001034131/297480, intron 8 of 15)
Cep131	360672	chr10	109267799	109268099	-194	0.52	3.76E-02	9.63E-01	Promoter (<=1kb)
Cwc27	361887	chr2	34925423	34925723	-2702	-1	3.76E-02	9.63E-01	Promoter (2-3kb)
Rasgrf1	192213	chr8	97247751	97248051	-29199	0.83	3.76E-02	9.63E-01	Distal Intergenic
Mtnr1a	114211	chr16	50423803	50424103	-64994	-1.18	3.76E-02	9.63E-01	Intron (NM_031819/83720, intron 3 of 26)
Zfp523	361809	chr20	7754993	7755293	-7034	-0.85	3.77E-02	9.63E-01	Distal Intergenic
Necap2	298598	chr5	159602422	159602722	-181	0.52	3.77E-02	9.63E-01	Promoter (<=1kb)
Tfe3	317376	chrX	15598657	15598957	-10959	0.58	3.77E-02	9.63E-01	Distal Intergenic
Efnb2	306636	chr16	87031581	87031881	400430	0.85	3.78E-02	9.63E-01	Distal Intergenic
Mir344b-1	1.01E+08	chr1	119696967	119697267	1495968	-1.03	3.78E-02	9.63E-01	Distal Intergenic
Arhgef7	114559	chr16	83065315	83065615	-6471	-0.85	3.78E-02	9.63E-01	Intron (NM_001113521/114559, intron 1 of 20)
Amhr2	29530	chr7	144050357	144050657	-1545	0.74	3.78E-02	9.63E-01	Promoter (1-2kb)
Mrpl37	56281	chr5	126668440	126668740	-1610	1	3.79E-02	9.63E-01	Promoter (1-2kb)
Itpkb	54260	chr13	98649889	98650189	34602	-0.81	3.79E-02	9.63E-01	Intron (NM_019312/54260, intron 2 of 7)
Arf6	79121	chr6	91769953	91770253	72844	0.64	3.79E-02	9.63E-01	Distal Intergenic
Rexo1	314630	chr7	12044662	12044962	22394	0.64	3.79E-02	9.63E-01	Distal Intergenic
C1qtnf7	305423	chr14	72042790	72043090	-17653	1.09	3.79E-02	9.63E-01	Distal Intergenic
Plppr1	298062	chr5	67097139	67097439	887869	-0.94	3.80E-02	9.63E-01	Distal Intergenic
Aldh16a1	361571	chr1	101147144	101147444	3876	0.73	3.82E-02	9.65E-01	Intron (NM_001033706/361571, intron 1 of 16)
Fcgr1a	295279	chr2	198528642	198528942	-89189	0.67	3.82E-02	9.65E-01	Distal Intergenic
Cmklr1	60669	chr12	48779298	48779598	-9663	0.66	3.82E-02	9.65E-01	Distal Intergenic
Rybp	312603	chr4	132876801	132877101	-135978	1.3	3.82E-02	9.65E-01	Distal Intergenic
Hip1	192154	chr12	24191975	24192275	11038	-0.82	3.83E-02	9.67E-01	Intron (NM_001100475/192154, intron 1 of 30)

Rgs17	308118	chr1	42703246	42703546	-115525	-0.87	3.83E-02	9.67E-01	Distal Intergenic
Gsap	311984	chr4	10557296	10557596	-39464	0.92	3.84E-02	9.67E-01	Distal Intergenic
Usp46	289584	chr14	36689313	36689613	2179	-0.83	3.84E-02	9.67E-01	Promoter (2-3kb)
Trak1	316085	chr8	129946086	129946386	-63187	-0.58	3.84E-02	9.67E-01	Distal Intergenic
Rnasel	359726	chr13	71188043	71188343	-5400	-1.02	3.84E-02	9.67E-01	Distal Intergenic
Cbfa2t3	361431	chr19	55533121	55533421	-22661	1	3.85E-02	9.67E-01	Distal Intergenic
Map3k4	308106	chr1	48627878	48628178	2135	0.88	3.85E-02	9.67E-01	Promoter (2-3kb)
Tead1	361630	chr1	177634143	177634443	138361	-0.9	3.86E-02	9.67E-01	Intron (NM_001198589/361630, intron 2 of 12)
Zfp598	287119	chr10	14032698	14032998	-2151	-1.01	3.86E-02	9.67E-01	Promoter (2-3kb)
Tanc1	311055	chr3	45671510	45671810	-46771	0.92	3.87E-02	9.67E-01	Distal Intergenic
Uqcc2	361805	chr20	5727985	5728285	-4083	-1.16	3.87E-02	9.67E-01	Distal Intergenic
Gmds	291095	chr17	33469514	33469814	60792	0.9	3.87E-02	9.67E-01	Intron (NM_001039606/291095, intron 1 of 10)
RGD1563482	498179	chr12	37697657	37697957	1659	-1.06	3.88E-02	9.67E-01	Promoter (1-2kb)
Dusp15	362238	chr3	148428403	148428703	0	1.09	3.88E-02	9.67E-01	Promoter (<=1kb)
Stk26	317589	chrX	137718094	137718394	-328145	-1.8	3.88E-02	9.67E-01	Distal Intergenic
Csnk1e	58822	chr7	120675669	120675969	-3319	0.54	3.88E-02	9.67E-01	Distal Intergenic
Egln2	308457	chr1	83973240	83973540	3696	-0.98	3.88E-02	9.67E-01	Intron (NM_001004083/308457, intron 2 of 5)
Ripk2	362491	chr5	29866409	29866709	3681	-0.93	3.89E-02	9.67E-01	Intron (NM_001191865/362491, intron 1 of 10)
Plekhg1	679812	chr1	40288497	40288797	-145193	0.88	3.90E-02	9.67E-01	Distal Intergenic
Tent4a	306672	chr1	36390101	36390401	-10042	-0.84	3.90E-02	9.67E-01	Distal Intergenic
Tlr3	364594	chr16	49989392	49989692	-27165	0.69	3.91E-02	9.67E-01	Distal Intergenic
Dab2ip	192126	chr3	15025361	15025661	136098	1.01	3.91E-02	9.67E-01	Intron (NM_138710/192126, intron 3 of 15)
Slc38a1	170567	chr7	138012941	138013241	26389	0.73	3.91E-02	9.67E-01	Intron (NM_138832/170567, intron 2 of 15)
Sinhcaf	686611	chr4	183426187	183426487	0	-0.68	3.91E-02	9.67E-01	Promoter (<=1kb)
Dnajb6	362293	chr4	2713677	2713977	2292	-1.18	3.92E-02	9.67E-01	Promoter (2-3kb)
Bmp4	25296	chr15	20670602	20670902	108900	1.15	3.92E-02	9.67E-01	Distal Intergenic
Laptm4a	298875	chr6	34018307	34018607	-23525	1.14	3.92E-02	9.67E-01	Distal Intergenic
Ccnb2	363088	chr1	21331657	21331957	-184811	-1.14	3.92E-02	9.67E-01	Distal Intergenic
Mir21	1E+08	chr10	73917602	73917902	-15301	0.97	3.92E-02	9.67E-01	Intron (NM_138839/192129, intron 10 of 11)
Mrm3	360569	chr10	64399904	64400204	1565	-0.91	3.93E-02	9.67E-01	Promoter (1-2kb)
Gtpbp3	290633	chr16	19895559	19895859	-1276	0.79	3.93E-02	9.67E-01	Promoter (1-2kb)
Mbip	362740	chr6	77300378	77300678	-56701	0.96	3.94E-02	9.67E-01	Distal Intergenic
Rpl37	81770	chr10	64303580	64303880	-32309	0.74	3.94E-02	9.67E-01	Intron (NM_001105813/287535, intron 6 of 21)
Aspa	79251	chr10	59889789	59890089	-1591	-1.43	3.95E-02	9.67E-01	Promoter (1-2kb)
Mras	25482	chr8	107583489	107583789	73062	0.79	3.95E-02	9.67E-01	Distal Intergenic
Prkra	311130	chr3	63506015	63506315	1603	-1.28	3.95E-02	9.67E-01	Promoter (1-2kb)
Nfe2	366998	chr7	144883615	144883915	-3523	0.82	3.96E-02	9.67E-01	Distal Intergenic
Stoml1	300748	chr8	63042143	63042443	4987	-0.53	3.96E-02	9.67E-01	Intron (NM_001305238/300748, intron 4 of 6)
Actg1	287876	chr10	109441065	109441365	79923	-0.93	3.96E-02	9.67E-01	Distal Intergenic
Trps1	299897	chr7	90422520	90422820	-104299	-0.69	3.97E-02	9.67E-01	Distal Intergenic
Ttc39b	298186	chr5	101295086	101295386	110270	1.07	3.97E-02	9.67E-01	Distal Intergenic
Elk4	304786	chr13	48793613	48793913	3104	-0.84	3.97E-02	9.67E-01	Intron (NM_001107173/304786, intron 1 of 5)
Synj1	85238	chr11	31164537	31164837	16732	-0.75	3.97E-02	9.67E-01	Intron (NM_053476/85238, intron 2 of 31)
Tectb	292124	chr1	276000798	276001098	43100	0.83	3.98E-02	9.67E-01	Distal Intergenic
Ccsap	307926	chr19	56622194	56622494	10631	0.69	3.98E-02	9.67E-01	Intron (NM_001107441/307926, intron 1 of 2)
Olig3	293012	chr1	14581984	14582284	-215482	1.32	3.98E-02	9.67E-01	Distal Intergenic
Krcc1	312437	chr4	99133180	99133480	-362	-0.54	3.99E-02	9.67E-01	Promoter (<=1kb)
Mir29b1	1E+08	chr4	58380797	58381097	-36407	-0.82	3.99E-02	9.67E-01	Distal Intergenic
Vps37c	308178	chr1	226742576	226742876	0	1.49	3.99E-02	9.67E-01	Promoter (<=1kb)
Ubc	50522	chr12	36619779	36620079	-18378	0.89	3.99E-02	9.67E-01	Distal Intergenic
Gatc	360821	chr12	47031953	47032253	367	-1.19	3.99E-02	9.67E-01	Promoter (<=1kb)
Col26a1	685612	chr12	23044371	23044671	209505	0.73	3.99E-02	9.67E-01	Distal Intergenic
Dpysl3	25418	chr18	37813646	37813946	-37193	1	3.99E-02	9.67E-01	Distal Intergenic
Rho	24717	chr4	147798085	147798385	-33751	0.66	4.00E-02	9.67E-01	Intron (NM_001009416/312651, intron 14 of 17)
Dyrk1a	25255	chr11	34896110	34896410	30578	-1.03	4.00E-02	9.67E-01	Intron (NM_012791/25255, intron 2 of 11)
C17h6orf52	498729	chr17	21597806	21598106	-2326	1.03	4.00E-02	9.67E-01	Promoter (2-3kb)
Fem1a	316131	chr9	10783046	10783346	17794	-0.86	4.01E-02	9.67E-01	Distal Intergenic
Anapc5	288671	chr12	39211349	39211649	2189	1.05	4.01E-02	9.67E-01	Promoter (2-3kb)
Ppp1r2	192361	chr11	72745578	72745878	-2911	-0.78	4.02E-02	9.67E-01	Promoter (2-3kb)
Stac2	363674	chr10	86025166	86025466	-20957	0.9	4.02E-02	9.67E-01	Distal Intergenic
Zbtb38	315936	chr8	104686563	104686863	-93064	1.05	4.02E-02	9.67E-01	Distal Intergenic
Gfra2	25136	chr15	52557718	52558018	111	0.61	4.02E-02	9.67E-01	Promoter (<=1kb)
Atf6	304962	chr13	89240035	89240335	2125	-0.82	4.03E-02	9.67E-01	Promoter (2-3kb)
Nfatc1	1E+08	chr18	77264325	77264625	58065	0.9	4.03E-02	9.67E-01	Intron (NM_001244933/100361818, intron 6 of 10)
Lin54	305171	chr14	10773306	10773606	-12793	-1.64	4.03E-02	9.67E-01	Distal Intergenic
Stk38	361813	chr20	6204686	6204986	26210	-0.63	4.03E-02	9.67E-01	Distal Intergenic
Jun	24516	chr5	114062351	114062651	-48074	0.99	4.04E-02	9.67E-01	Distal Intergenic
Ndufb9	299954	chr7	98880345	98880645	67283	-0.81	4.05E-02	9.67E-01	Intron (NM_001130563/362918, intron 4 of 15)
Tgoln2	192152	chr4	100487139	100487439	-3971	1.13	4.05E-02	9.67E-01	Distal Intergenic
Actr1b	316333	chr9	43274245	43274545	2172	-0.77	4.07E-02	9.67E-01	Promoter (2-3kb)
RGD1304770	309810	chr20	45006448	45006748	17567	-1.18	4.07E-02	9.67E-01	Distal Intergenic
Nfia	25492	chr5	116453192	116453492	31294	-0.63	4.07E-02	9.67E-01	Intron (NM_012988/25492, intron 1 of 9)
Crem	25620	chr17	57023423	57023723	1719	-1.09	4.08E-02	9.67E-01	Promoter (1-2kb)
Cwc27	361887	chr2	34975588	34975888	-52867	-0.86	4.08E-02	9.67E-01	Distal Intergenic
Dnaaf1	361419	chr19	52223030	52223330	-2252	-1.18	4.08E-02	9.67E-01	Promoter (2-3kb)
Slc45a1	246258	chr5	167619344	167619644	76687	-0.66	4.09E-02	9.67E-01	Intron (NM_053885/116665, intron 12 of 22)
Ppp4c	171366	chr1	198228509	198228809	-2929	1.19	4.09E-02	9.67E-01	Promoter (2-3kb)
Rad54b	313063	chr5	25141777	25142077	-4886	-0.57	4.09E-02	9.67E-01	Distal Intergenic
Ftx	302873	chrX	74483549	74483849	315	-0.53	4.09E-02	9.67E-01	Promoter (<=1kb)
Aagab	171435	chr8	68589867	68590167	63772	-1.06	4.09E-02	9.67E-01	Exon (NM_013095/25631, exon 2 of 9)
Mical2	365352	chr1	177051755	177052055	-41332	-0.63	4.10E-02	9.67E-01	Distal Intergenic
Mtor	56718	chr5	165328571	165328871	64753	0.79	4.10E-02	9.67E-01	Intron (NM_019906/56718, intron 29 of 57)
Atoh8	500200	chr4	100081384	100081684	17833	0.68	4.10E-02	9.67E-01	Intron (NM_001109241/500200, intron 2 of 2)
Clec2d2	362445	chr4	162482399	162482699	45300	1.27	4.10E-02	9.67E-01	Distal Intergenic
Triobp	362956	chr7	120172853	120173153	-3377	-1.07	4.11E-02	9.67E-01	Distal Intergenic
Rreb1	306873	chr17	27645216	27645516	19750	1.06	4.11E-02	9.67E-01	Intron (NM_001107348/306873, intron 2 of 12)
LOC688553	688553	chr14	89249978	89250278	-2824	-0.76	4.11E-02	9.67E-01	Promoter (2-3kb)
Ppef2	305246	chr14	17320676	17320976	12612	0.92	4.11E-02	9.67E-01	Intron (NM_001107210/305246, intron 9 of 15)
Lamc1	117036	chr13	70759024	70759324	24191	-1.02	4.11E-02	9.67E-01	Intron (NM_053966/117036, intron 1 of 27)
Rplp0	64205	chr12	46813255	46813555	-18458	1.16	4.11E-02	9.67E-01	Intron (NM_001012147/360820, intron 1 of 9)
Arpc3	288669	chr12	39665234	39665534	2283	-0.88	4.11E-02	9.67E-01	Promoter (2-3kb)
B4galt5	362275	chr3	164059558	164059858	-3997	-0.6	4.12E-02	9.67E-01	Distal Intergenic
Chst12	304322	chr12	16214808	16215108	-13180	1.01	4.12E-02	9.67E-01	Distal Intergenic
Rpn1	25596	chr4	119999816	120000116	2584	-0.85	4.13E-02	9.67E-01	Promoter (2-3kb)

Fndc3b	294925	chr2	113477727	113478027	-132176	1.02	4.13E-02	9.67E-01	Distal Intergenic
Gmds	291095	chr17	33522396	33522696	113674	1	4.13E-02	9.67E-01	Intron (NM_001039606/291095, intron 4 of 10)
Ch25h	309527	chr1	252767352	252767652	40728	-0.96	4.14E-02	9.67E-01	Distal Intergenic
Stoml1	300748	chr8	63034384	63034684	-2472	-0.6	4.14E-02	9.67E-01	Promoter (2-3kb)
Pdlim4	24915	chr10	39396200	39396500	8811	0.55	4.14E-02	9.67E-01	Exon (NM_017062/24915, exon 3 of 7)
Rps4y2	690845	chr4	182837154	182837454	91706	-0.96	4.16E-02	9.67E-01	Distal Intergenic
Casp1	25166	chr8	2616400	2616700	10657	-0.65	4.16E-02	9.67E-01	Distal Intergenic
Pou1f1	25517	chr11	2725722	2726022	79857	1.14	4.16E-02	9.67E-01	Distal Intergenic
Tgif1	316742	chr9	119093186	119093486	97212	1.13	4.16E-02	9.67E-01	Intron (NM_022946/65040, intron 7 of 12)
Mcu	294560	chr20	29162349	29162649	36575	-0.57	4.16E-02	9.67E-01	Intron (NM_001106398/294560, intron 1 of 7)
Pik3ip1	305472	chr14	83565397	83565697	4856	-0.9	4.17E-02	9.67E-01	Intron (NM_001017453/305472, intron 6 of 6)
Slc24a2	84550	chr5	105865456	105865756	-285497	0.98	4.17E-02	9.67E-01	Distal Intergenic
Gmpr	117533	chr17	19558553	19558853	22076	1.04	4.17E-02	9.67E-01	Intron (NM_057188/117533, intron 5 of 8)
Ackr3	84348	chr9	97358019	97358319	2095	-0.73	4.17E-02	9.67E-01	Promoter (2-3kb)
Arfgap3	503165	chr7	124190848	124191148	7115	-0.85	4.17E-02	9.67E-01	Intron (NM_001044273/503165, intron 1 of 19)
Ivd	24513	chr3	110668710	110669010	-345	-0.59	4.18E-02	9.67E-01	Promoter (<=1kb)
Flnb	306204	chr15	18810774	18811074	71885	1	4.18E-02	9.67E-01	Exon (NM_001107288/306204, exon 10 of 45)
Zfp639	683504	chr2	118903820	118904120	-4588	-0.88	4.18E-02	9.67E-01	Distal Intergenic
Herpud2	300463	chr8	26312316	26312616	-966	0.68	4.18E-02	9.67E-01	Promoter (<=1kb)
Esyt1	29579	chr7	2955025	2955325	-13812	-0.77	4.19E-02	9.67E-01	Distal Intergenic
Mir466d	1.01E+08	chr17	71846294	71846594	-49503	-0.69	4.19E-02	9.67E-01	Intron (NM_001107364/307106, intron 4 of 18)
Klf4	114505	chr5	72407216	72407516	-119547	-0.59	4.19E-02	9.67E-01	Distal Intergenic
Mir328b	1.01E+08	chr14	45589778	45590078	258632	-1.13	4.20E-02	9.67E-01	Distal Intergenic
Cldn20	680178	chr1	44310045	44310345	-136420	-0.84	4.20E-02	9.67E-01	Distal Intergenic
Pxylp1	315939	chr8	104692323	104692623	98016	0.87	4.21E-02	9.67E-01	Distal Intergenic
Pik3ip1	305472	chr14	83562367	83562667	1826	-0.63	4.21E-02	9.67E-01	Promoter (1-2kb)
Glul	24957	chr13	71600459	71600759	269407	-0.94	4.21E-02	9.67E-01	Distal Intergenic
Nav3	314814	chr7	52164614	52164914	343231	-0.8	4.21E-02	9.67E-01	3' UTR
Plrg1	60376	chr2	182076304	182076604	35966	-0.56	4.22E-02	9.67E-01	Distal Intergenic
Aph1b	300802	chr8	72645614	72645914	-16349	0.79	4.22E-02	9.67E-01	Intron (NM_153317/266688, intron 5 of 7)
Fbxo30	308283	chr1	5332475	5332775	-33604	0.96	4.22E-02	9.67E-01	Distal Intergenic
Cbln1	498922	chr19	20502200	20502500	-105007	0.97	4.23E-02	9.67E-01	Distal Intergenic
Zfand2a	360772	chr12	17255311	17255611	3218	-0.91	4.23E-02	9.67E-01	Intron (NM_001008363/360772, intron 3 of 6)
Mettl23	287918	chr10	105790954	105791254	3016	-1.07	4.23E-02	9.67E-01	Exon (NM_001008283/287918, exon 2 of 5)
Cldn7	65132	chr10	56572138	56572438	-4004	0.78	4.23E-02	9.67E-01	Distal Intergenic
Angpt1	89807	chr7	81530969	81531269	61030	1.15	4.23E-02	9.67E-01	Intron (NM_053546/89807, intron 1 of 8)
Smarcc1	301020	chr8	118206208	118206508	-195	-0.51	4.23E-02	9.67E-01	Promoter (<=1kb)
Hdac2	84577	chr20	43079658	43079958	28240	0.65	4.24E-02	9.67E-01	Distal Intergenic
Zdhhc6	361771	chr1	276307672	276307972	1661	-0.79	4.24E-02	9.67E-01	Promoter (1-2kb)
Ldhd	307858	chr19	43839879	43840179	8758	1.03	4.25E-02	9.67E-01	Exon (NM_001008893/307858, exon 6 of 11)
Ccdc120	317377	chrX	15599415	15599715	-10515	0.92	4.25E-02	9.67E-01	Distal Intergenic
Slc44a1	85254	chr5	70200180	70200480	-45363	-0.66	4.25E-02	9.67E-01	Distal Intergenic
Olr1461	287349	chr10	45195350	45195650	36008	-0.94	4.26E-02	9.67E-01	Distal Intergenic
Mfap3l	306424	chr16	32778051	32778351	-24773	-0.92	4.26E-02	9.67E-01	Distal Intergenic
Arhgap35	306400	chr1	78555708	78556008	17366	-0.73	4.27E-02	9.67E-01	Intron (NM_001271132/306400, intron 1 of 6)
Cnot10	316034	chr8	122696838	122697138	-372	-0.68	4.27E-02	9.67E-01	Promoter (<=1kb)
Rap2c	302495	chrX	138210975	138211275	25882	0.76	4.27E-02	9.67E-01	Distal Intergenic
Septin9	83788	chr10	106471904	106472204	164871	0.91	4.27E-02	9.67E-01	Distal Intergenic
Mras	25482	chr8	107698390	107698690	-41539	1.34	4.27E-02	9.67E-01	Distal Intergenic
Prrx1	266813	chr13	81184058	81184358	30464	-0.94	4.27E-02	9.67E-01	Intron (NM_153821/266813, intron 1 of 3)
Pdcd4	64031	chr1	274571564	274571864	-57155	0.86	4.27E-02	9.67E-01	Exon (NM_001107611/309544, exon 10 of 14)
Mir6324	1.02E+08	chr11	36418055	36418355	-34406	0.93	4.28E-02	9.67E-01	Distal Intergenic
Cd200	24560	chr11	60421505	60421805	49776	-0.86	4.28E-02	9.67E-01	Distal Intergenic
Ier3	294235	chr20	3448689	3448989	-8687	0.89	4.28E-02	9.67E-01	Distal Intergenic
Rnf145	287212	chr10	30162998	30163298	-5959	-0.95	4.28E-02	9.67E-01	Distal Intergenic
Gmeb2	83635	chr3	176790103	176790403	1557	-0.8	4.29E-02	9.67E-01	Promoter (1-2kb)
Sfmbt2	307106	chr17	71868693	71868993	28979	-1.04	4.29E-02	9.67E-01	Intron (NM_001107364/307106, intron 4 of 18)
Ndfip1	291609	chr18	31576589	31576889	1768	-0.72	4.30E-02	9.67E-01	Promoter (1-2kb)
Dguok	297389	chr4	115204108	115204408	3653	-1.02	4.30E-02	9.67E-01	Intron (NM_001106602/297389, intron 1 of 6)
Mbd2	680172	chr18	65813430	65813730	-296	-1.03	4.30E-02	9.67E-01	Promoter (<=1kb)
Riok3	361293	chr18	3553665	3553965	-11201	-0.68	4.30E-02	9.67E-01	Distal Intergenic
Nfu1	297416	chr4	118799979	118800279	-14005	0.91	4.30E-02	9.67E-01	Distal Intergenic
Slit1	65047	chr1	261073421	261073721	-81055	-0.6	4.30E-02	9.67E-01	Distal Intergenic
Ssc5d	308341	chr1	72444964	72445264	16283	0.9	4.31E-02	9.68E-01	Exon (NM_001134545/308341, exon 14 of 14)
Fam76a	362618	chr5	151137633	151137933	-20491	0.79	4.31E-02	9.68E-01	Distal Intergenic
Sertad2	498423	chr14	104847539	104847839	26324	-0.82	4.32E-02	9.68E-01	Intron (NM_001024903/498423, intron 1 of 1)
Slc29a4	288499	chr12	13942162	13942462	-17656	1.01	4.33E-02	9.68E-01	Distal Intergenic
Hoxc11	1.01E+08	chr7	144570998	144571298	5606	1.23	4.34E-02	9.68E-01	Distal Intergenic
Smad3	25631	chr8	68666566	68666866	11483	1.1	4.34E-02	9.68E-01	Intron (NM_013095/25631, intron 1 of 8)
Psgb1	59313	chr1	79157758	79158058	-23797	-0.69	4.35E-02	9.68E-01	Distal Intergenic
Asb7	365277	chr1	127596357	127596657	2600	-0.85	4.35E-02	9.68E-01	Promoter (2-3kb)
Vamp2	24803	chr10	55677877	55678177	2340	0.94	4.36E-02	9.68E-01	Promoter (2-3kb)
Ddx46	245957	chr17	9519980	9520280	-466	-0.68	4.36E-02	9.68E-01	Promoter (<=1kb)
Josd2	292876	chr1	100472970	100473270	-373	-0.83	4.37E-02	9.68E-01	Promoter (<=1kb)
LOC100909970	1.01E+08	chr17	27780325	27780625	-60056	1.29	4.37E-02	9.68E-01	Distal Intergenic
Id3	25585	chr5	154504011	154504311	14408	0.96	4.37E-02	9.68E-01	Distal Intergenic
Ppp1r27	287881	chr10	109725755	109726055	2964	-0.57	4.37E-02	9.68E-01	Promoter (2-3kb)
Tbx19	304935	chr13	83381637	83381937	43704	0.71	4.37E-02	9.68E-01	Distal Intergenic
Qars1	290868	chr8	117295716	117296016	-1654	-1.11	4.37E-02	9.68E-01	Promoter (1-2kb)
Ubiad1	313706	chr5	165201221	165201521	57707	0.62	4.37E-02	9.68E-01	Distal Intergenic
Tmie	501061	chr8	119155330	119155630	1441	1.14	4.37E-02	9.68E-01	Promoter (1-2kb)
Zkscan3	306977	chr17	45249394	45249694	1600	-1.03	4.38E-02	9.68E-01	Promoter (1-2kb)
Pcdh11x	317204	chrX	93684228	93684528	1087867	1.55	4.38E-02	9.68E-01	Distal Intergenic
Tipin	363076	chr8	69747529	69747829	-5534	-0.63	4.38E-02	9.68E-01	Distal Intergenic
Ankrd39	367251	chr9	43126496	43126796	1120	-0.68	4.38E-02	9.68E-01	Promoter (1-2kb)
Traf2	311786	chr3	2773039	2773339	-2419	0.71	4.38E-02	9.68E-01	Promoter (2-3kb)
Foxq1	64826	chr17	34208644	34208944	17997	0.74	4.39E-02	9.68E-01	Distal Intergenic
Bcat1	29592	chr4	179195284	179195584	111511	-0.64	4.39E-02	9.68E-01	Distal Intergenic
Zfp362	297879	chr5	146993460	146993760	-19528	-0.93	4.39E-02	9.68E-01	Distal Intergenic
Acad9	294973	chr2	122781397	122781697	-434	-0.51	4.40E-02	9.68E-01	Promoter (<=1kb)
Timp2	29543	chr10	107396753	107397053	-10681	0.62	4.41E-02	9.68E-01	Distal Intergenic
Mcart1	313241	chr5	61062244	61062544	67482	-0.59	4.41E-02	9.68E-01	Distal Intergenic
Psap	25524	chr20	29814278	29814578	-16736	0.58	4.41E-02	9.68E-01	Distal Intergenic
Chchd6	297436	chr4	121310172	121310472	254740	-0.85	4.41E-02	9.68E-01	Distal Intergenic

Vmp1	192129	chr10	73999859	74000159	1736	-1.05	4.42E-02	9.68E-01	Promoter (1-2kb)
Zbed5	288622	chr12	30314658	30314958	-138	-0.58	4.42E-02	9.68E-01	Promoter (<=1kb)
Gcnt3	286976	chr8	76472547	76472847	-19993	-0.68	4.43E-02	9.68E-01	Distal Intergenic
Cul2	361258	chr17	57006662	57006962	-14173	-0.89	4.43E-02	9.68E-01	Distal Intergenic
Sh3bgrl3	298544	chr5	152360048	152360348	-1405	-0.83	4.43E-02	9.68E-01	Promoter (1-2kb)
Miip	298643	chr5	164431822	164432122	123521	-0.87	4.44E-02	9.68E-01	Distal Intergenic
Tas1r1	29407	chr5	169215467	169215767	-3354	-0.97	4.45E-02	9.68E-01	Distal Intergenic
Optn	246294	chr17	77169393	77169693	-7012	-0.84	4.46E-02	9.68E-01	Distal Intergenic
Ampd3	25095	chr1	175525588	175525888	-60209	1.09	4.46E-02	9.68E-01	Distal Intergenic
Septin11	305227	chr14	16445602	16445902	6988	-0.66	4.46E-02	9.68E-01	Intron (NM_001107208/305227, intron 1 of 10)
Abca15	293442	chr1	190071112	190071412	-1008	0.92	4.46E-02	9.68E-01	Promoter (1-2kb)
Cdk17	314743	chr7	34028168	34028468	26662	1.14	4.47E-02	9.68E-01	Intron (NM_001108082/314743, intron 1 of 16)
Dnase2	171575	chr19	26022388	26022688	-167	-0.52	4.47E-02	9.68E-01	Promoter (<=1kb)
Npepps	50558	chr10	85214211	85214511	8350	-0.78	4.47E-02	9.68E-01	Intron (NM_080395/50558, intron 1 of 22)
Mrgpre	404660	chr1	216983644	216983944	-12461	0.83	4.47E-02	9.68E-01	Downstream (2-3kb)
Tlr6	305353	chr14	45011993	45012293	-12058	0.56	4.47E-02	9.68E-01	Distal Intergenic
Acat1	25014	chr8	58183309	58183609	12275	1.27	4.47E-02	9.68E-01	Exon (NM_017075/25014, exon 3 of 12)
Efna5	116683	chr9	110478814	110479114	-149127	-0.83	4.49E-02	9.68E-01	Distal Intergenic
Ndufaf6	297821	chr5	24335570	24335870	-14766	0.89	4.49E-02	9.68E-01	Distal Intergenic
Angel1	362765	chr6	110670233	110670533	-323	-0.73	4.49E-02	9.68E-01	Promoter (<=1kb)
Znrf4	301127	chr9	10536792	10537351	1452	0.58	4.49E-02	9.68E-01	Promoter (1-2kb)
Sertad2	498423	chr14	104803360	104803660	-17555	-0.51	4.50E-02	9.68E-01	Distal Intergenic
Epha2	366492	chr5	159828330	159828630	-17143	0.97	4.50E-02	9.68E-01	Distal Intergenic
Shisa3	498356	chr14	42005545	42005845	215380	0.85	4.51E-02	9.68E-01	Distal Intergenic
Fras1	289486	chr14	14852473	14852773	243	0.62	4.51E-02	9.68E-01	Promoter (<=1kb)
Dusp21	302867	chrX	4942814	4943114	14441	-0.85	4.51E-02	9.68E-01	Distal Intergenic
Msl3l2	309790	chr20	37588208	37588508	2495	0.52	4.51E-02	9.68E-01	Promoter (2-3kb)
Sema3f	315996	chr8	116469526	116469826	89	0.67	4.51E-02	9.68E-01	Promoter (<=1kb)
Tssc4	361682	chr1	216258065	216258365	3154	-0.88	4.51E-02	9.68E-01	Exon (NM_001191896/365391, exon 23 of 24)
Arhgap35	306400	chr1	78567874	78568174	5200	-0.79	4.52E-02	9.68E-01	Intron (NM_001271132/306400, intron 1 of 6)
Cbx8	303731	chr10	108165606	108165906	-15095	0.62	4.52E-02	9.68E-01	Intron (NM_001134688/287867, intron 1 of 26)
Idh1	24479	chr9	71904218	71904518	-495	-0.56	4.52E-02	9.68E-01	Promoter (<=1kb)
Fmr1	24948	chrX	154654049	154654349	67965	-0.84	4.52E-02	9.68E-01	Distal Intergenic
Utp20	314713	chr7	29376500	29376800	1120	-0.83	4.52E-02	9.68E-01	Promoter (1-2kb)
Lrrc26	311803	chr3	2508536	2508836	2110	0.54	4.52E-02	9.68E-01	Promoter (2-3kb)
Mir325	1E+08	chrX	76485627	76485927	-151259	-0.72	4.53E-02	9.68E-01	Distal Intergenic
Oip5-as1	1.01E+08	chr3	111396481	111396781	-270	0.62	4.53E-02	9.68E-01	Promoter (<=1kb)
Hacl1	85255	chr16	7756381	7756681	1438	-1.21	4.53E-02	9.68E-01	Promoter (1-2kb)
Strbp	84476	chr3	21956011	21956311	-51741	-0.93	4.53E-02	9.68E-01	Distal Intergenic
Ttc1	287208	chr10	29365512	29365812	2053	1.01	4.54E-02	9.68E-01	Promoter (2-3kb)
Tead1	361630	chr1	177610611	177610911	114829	0.72	4.54E-02	9.68E-01	Intron (NM_001198589/361630, intron 2 of 12)
Rnf10	288710	chr12	47108775	47109075	5462	1.01	4.54E-02	9.68E-01	Intron (NM_001011904/288710, intron 1 of 16)
Klhl31	315833	chr8	84937978	84938278	-7166	-0.74	4.54E-02	9.68E-01	Distal Intergenic
Stxbp5	81022	chr1	4012696	4012996	-1185	-1.03	4.54E-02	9.68E-01	Promoter (1-2kb)
Marcks	25603	chr20	42965632	42965932	-208	-0.53	4.54E-02	9.68E-01	Promoter (<=1kb)
Col16a1	366474	chr5	148260434	148260734	2841	1.03	4.54E-02	9.68E-01	Promoter (2-3kb)
Ca12	363085	chr8	72494849	72495149	88926	-0.66	4.55E-02	9.68E-01	Distal Intergenic
Tmem174	499516	chr2	28636654	28636954	113283	-0.79	4.55E-02	9.68E-01	Distal Intergenic
Bcs1l	301514	chr9	81869275	81869575	1010	-0.74	4.55E-02	9.68E-01	Promoter (1-2kb)
Tmem43	362401	chr4	123133855	123134155	15384	0.92	4.55E-02	9.68E-01	Downstream (<1kb)
Pxn	360820	chr12	46823460	46823760	21319	1.01	4.56E-02	9.68E-01	Intron (NM_001012147/360820, intron 1 of 9)
Bod1	287173	chr10	16299332	16299632	39601	-0.82	4.56E-02	9.68E-01	Distal Intergenic
Mbnl1	282635	chr2	150853510	150853810	97252	-0.79	4.56E-02	9.68E-01	Intron (NM_001191566/282635, intron 3 of 7)
Arrdc4	293019	chr1	130551870	130552170	-775594	-1.04	4.56E-02	9.68E-01	Distal Intergenic
LOC362901	362901	chr7	75536314	75536614	33164	0.77	4.56E-02	9.68E-01	Distal Intergenic
Tti2	290811	chr16	64742756	64743056	2151	-1.01	4.56E-02	9.68E-01	Promoter (2-3kb)
Kctd15	499129	chr1	90521315	90521615	-1303	0.65	4.57E-02	9.68E-01	Promoter (1-2kb)
Kif18b	303575	chr10	91028841	91029141	-1376	-0.88	4.57E-02	9.68E-01	Promoter (1-2kb)
Phtf2	296762	chr4	11209469	11209769	386211	-0.88	4.57E-02	9.68E-01	Intron (NM_053621/113970, intron 10 of 23)
Capn12	308476	chr1	87088277	87088577	21988	0.93	4.58E-02	9.71E-01	Intron (NM_031675/63836, intron 11 of 20)
Gtpbp10	312054	chr4	25635762	25636062	0	-0.53	4.59E-02	9.71E-01	Promoter (<=1kb)
Cstf2t	309338	chr1	249574331	249574631	-323	-0.65	4.59E-02	9.71E-01	Promoter (<=1kb)
Stoml1	300748	chr8	63036618	63036918	-238	0.63	4.60E-02	9.71E-01	Promoter (<=1kb)
Tll2	365460	chr1	260514779	260515079	-53988	1.02	4.60E-02	9.71E-01	Distal Intergenic
Dtnbp1	641528	chr17	20086291	20086591	-3545	1.15	4.60E-02	9.71E-01	Distal Intergenic
Ttc39b	298186	chr5	101289970	101290270	115386	0.59	4.60E-02	9.71E-01	Distal Intergenic
Mknk2	299618	chr7	11917761	11918061	9654	-0.94	4.61E-02	9.72E-01	3' UTR
Tas2r134	295589	chr3	37243461	37243761	96506	0.86	4.61E-02	9.72E-01	Distal Intergenic
P4ha2	360526	chr10	39469272	39469572	34045	1.01	4.62E-02	9.72E-01	Distal Intergenic
Tcf4	84382	chr18	65171821	65172121	-113199	-0.71	4.62E-02	9.72E-01	Distal Intergenic
Tmpo	25359	chr7	31883427	31883727	-11056	-0.7	4.63E-02	9.72E-01	Distal Intergenic
LOC689840	689840	chr8	14713937	14714237	-97390	-0.68	4.63E-02	9.72E-01	Intron (NM_138544/191571, intron 3 of 26)
Tmem248	288616	chr12	29945174	29945474	12576	-0.73	4.63E-02	9.72E-01	Intron (NM_001004204/288616, intron 4 of 9)
Itgb1bp1	298914	chr6	43202606	43202906	160429	-0.64	4.64E-02	9.72E-01	Distal Intergenic
Top1	64550	chr3	156617720	156618020	-17668	-0.56	4.65E-02	9.72E-01	Distal Intergenic
Olr434	296694	chr3	21383634	21383934	20349	-1.07	4.65E-02	9.72E-01	Distal Intergenic
Reep3	294375	chr20	23077934	23078234	164285	1.12	4.65E-02	9.72E-01	Distal Intergenic
Zfp385b	311137	chr3	64882856	64883156	-339756	-0.52	4.65E-02	9.72E-01	Distal Intergenic
Usp12	360763	chr12	10061515	10061815	23829	0.79	4.65E-02	9.72E-01	Intron (NM_001166576/360763, intron 1 of 8)
Melk	362510	chr5	59711267	59711567	-72323	-0.98	4.66E-02	9.72E-01	Distal Intergenic
Camk2d	24246	chr2	231226104	231226404	324978	-1.09	4.66E-02	9.72E-01	Distal Intergenic
Faap20	362678	chr5	172604976	172605276	-43674	1.33	4.67E-02	9.72E-01	Distal Intergenic
Bmf	246142	chr3	110313011	110313311	10773	-0.81	4.67E-02	9.72E-01	Intron (NM_139258/246142, intron 4 of 4)
Hexim2	303580	chr10	91228436	91228736	11357	-1.23	4.67E-02	9.72E-01	Distal Intergenic
Fads2	83512	chr1	226148836	226149136	3432	-1.27	4.68E-02	9.72E-01	Intron (NM_031344/83512, intron 1 of 11)
Otulinl	310190	chr2	80751088	80751388	-342416	-0.93	4.68E-02	9.72E-01	Distal Intergenic
Mtnr1a	114211	chr16	50422823	50423386	-64014	-0.67	4.68E-02	9.72E-01	Intron (NM_031819/83720, intron 3 of 26)
Eif2ak1	27137	chr12	12780077	12780377	1701	-0.95	4.68E-02	9.72E-01	Promoter (1-2kb)
Pkd2l2	291683	chr18	27185622	27185922	-5052	-1.13	4.68E-02	9.72E-01	Distal Intergenic
Adgrl4	64124	chr2	256489659	256489959	-119828	-0.85	4.68E-02	9.72E-01	Distal Intergenic
Selenop	29360	chr2	53106443	53106743	355	-1	4.68E-02	9.72E-01	Promoter (<=1kb)
Tmem9	289046	chr13	52844674	52844974	-9057	0.94	4.69E-02	9.72E-01	Distal Intergenic
Vcl	305679	chr15	3540814	3541114	3571	-0.74	4.69E-02	9.72E-01	Intron (NM_001107248/305679, intron 1 of 20)
Adamts3	305253	chr14	19717267	19717567	-148841	1.06	4.69E-02	9.72E-01	Distal Intergenic

Ubl3	363869	chr12	7808950	7809250	-56688	0.73	4.70E-02	9.72E-01	Distal Intergenic
Etfa	300726	chr8	60098074	60098374	-11722	1.48	4.70E-02	9.72E-01	Distal Intergenic
Fgf7	29348	chr3	118312941	118313241	-4520	1.09	4.70E-02	9.73E-01	Intron (NM_001013920/296118, intron 11 of 16)
Ivd	24513	chr3	110692267	110692567	22912	0.65	4.70E-02	9.73E-01	Distal Intergenic
Thap7	287944	chr11	87403755	87404055	-1224	-0.83	4.71E-02	9.73E-01	Promoter (1-2kb)
Mex3c	307271	chr18	69520982	69521282	-28655	-0.88	4.72E-02	9.73E-01	Distal Intergenic
Ccn2	64032	chr1	21855675	21855975	-902	-0.95	4.72E-02	9.73E-01	Promoter (<=1kb)
Cox4i2	84683	chr3	148264749	148265049	30203	0.96	4.72E-02	9.73E-01	Intron (NM_001033671/24888, intron 1 of 1)
Prdx5	113898	chr1	222164422	222164722	2725	-0.66	4.72E-02	9.73E-01	Promoter (2-3kb)
Sem1	680532	chr4	32087754	32088054	-154	-0.82	4.73E-02	9.73E-01	Promoter (<=1kb)
Mtus1	306487	chr16	54358437	54358737	25728	0.63	4.74E-02	9.76E-01	Intron (NM_178093/306487, intron 2 of 9)
Laptm4a	298875	chr6	34019173	34019473	-22659	0.94	4.75E-02	9.76E-01	Distal Intergenic
Eif6	305506	chr3	151360827	151361127	1988	-0.99	4.75E-02	9.76E-01	Promoter (1-2kb)
Pcdha3	116780	chr18	29966819	29967119	574	1.12	4.75E-02	9.76E-01	Promoter (<=1kb)
Pde4b	24626	chr5	121781183	121781483	21947	-0.71	4.76E-02	9.77E-01	Intron (NM_017031/24626, intron 1 of 14)
Tlr2	310553	chr2	182868600	182868900	-22539	0.81	4.76E-02	9.78E-01	Distal Intergenic
Btbd3	311462	chr3	131099457	131099757	-251830	0.8	4.78E-02	9.81E-01	Distal Intergenic
Vti1a	65277	chr1	276311292	276311592	1220	-0.68	4.78E-02	9.81E-01	Promoter (1-2kb)
Sae1	308384	chr1	78300193	78300493	32788	-0.92	4.79E-02	9.82E-01	Intron (NM_001012063/308384, intron 6 of 8)
Mllt1	301119	chr9	10116345	10116645	5956	0.89	4.79E-02	9.82E-01	Intron (NM_001106876/301119, intron 1 of 11)
Foxn1	287469	chr10	65626786	65627086	7580	0.59	4.80E-02	9.82E-01	Intron (NM_001100648/287469, intron 5 of 7)
Dnaja4	300721	chr8	59281790	59282090	3528	-0.89	4.80E-02	9.82E-01	Intron (NM_001025411/300721, intron 2 of 7)
Ocel1	290628	chr16	19767775	19768075	511	0.81	4.81E-02	9.82E-01	Promoter (<=1kb)
Sh3bp4	64634	chr9	96214529	96214829	3779	1.23	4.81E-02	9.82E-01	Intron (NM_022693/64634, intron 1 of 5)
Rtp4	360733	chr11	80643499	80643799	7003	0.52	4.81E-02	9.82E-01	Exon (NM_001108321/360733, exon 3 of 4)
Scn5a	25665	chr8	128237195	128237495	29144	0.76	4.82E-02	9.82E-01	Intron (NM_001160162/25665, intron 3 of 26)
Atp1b2	24214	chr10	56211129	56211429	428	0.64	4.82E-02	9.82E-01	Promoter (<=1kb)
Cmip	292051	chr19	49915411	49915711	123138	-0.58	4.82E-02	9.82E-01	Intron (NM_001163273/292051, intron 1 of 20)
Fendrr	1.05E+08	chr19	52897131	52897431	113078	1.21	4.83E-02	9.82E-01	Distal Intergenic
Crtac1	171438	chr1	261752914	261753214	-83391	-0.68	4.83E-02	9.82E-01	Distal Intergenic
Prrc2c	360865	chr13	80615118	80615418	-193	-0.58	4.83E-02	9.82E-01	Promoter (<=1kb)
Tuba1a	64158	chr7	140675581	140675881	-34628	-1.09	4.83E-02	9.82E-01	Distal Intergenic
Smad6	367100	chr8	68912098	68912398	53710	0.71	4.83E-02	9.82E-01	Intron (NM_001109002/367100, intron 3 of 3)
Ccdc65	362994	chr7	140371959	140372259	-11202	1.32	4.83E-02	9.82E-01	Distal Intergenic
Npepo	290963	chr17	680454	680754	122270	0.78	4.84E-02	9.82E-01	Intron (NM_001012346/290963, intron 4 of 14)
Chsy1	292999	chr1	127078104	127078404	67490	1.02	4.84E-02	9.82E-01	Distal Intergenic
Asb7	365277	chr1	127478938	127479238	120019	0.88	4.85E-02	9.83E-01	Distal Intergenic
Smad6	367100	chr8	68908383	68908683	57425	-0.96	4.85E-02	9.83E-01	Intron (NM_001109002/367100, intron 3 of 3)
Dmrt1	114498	chr1	243378592	243378892	-98601	-0.73	4.86E-02	9.83E-01	Intron (NM_001037197/309429, intron 4 of 12)
Lysmd2	300839	chr8	82559808	82560108	-653	-0.92	4.86E-02	9.83E-01	Promoter (<=1kb)
Pigt	296360	chr3	160965206	160965506	19627	-0.88	4.86E-02	9.83E-01	Distal Intergenic
Aspa	79251	chr10	59887770	59888070	128	0.77	4.87E-02	9.84E-01	Promoter (<=1kb)
Sned1	316638	chr9	100380678	100380978	163	0.76	4.88E-02	9.85E-01	Promoter (<=1kb)
Fam117a	497983	chr10	83154179	83154479	-321	-0.71	4.89E-02	9.86E-01	Promoter (<=1kb)
Gpa33	360873	chr13	84342357	84342657	7759	0.6	4.89E-02	9.86E-01	Intron (NM_001191829/360873, intron 1 of 6)
Cbfb	361391	chr19	37135006	37135306	7498	-0.67	4.89E-02	9.86E-01	Exon (NM_001013191/361391, exon 3 of 6)
Nsdhl	309262	chrX	152936323	152936623	3197	-0.9	4.90E-02	9.86E-01	Intron (NM_001009399/309262, intron 1 of 7)
Foxn3	314374	chr6	123421299	123421599	156096	-0.58	4.90E-02	9.86E-01	Intron (NM_001108047/314374, intron 2 of 6)
Map2	25595	chr9	73335825	73336125	-41935	-0.77	4.90E-02	9.86E-01	Distal Intergenic
Aplp2	64312	chr8	32352393	32352693	-23572	0.96	4.91E-02	9.86E-01	Distal Intergenic
Acot13	291135	chr17	42242326	42242626	1185	-0.87	4.91E-02	9.86E-01	Promoter (1-2kb)
Mir3556b	1.01E+08	chr1	38597341	38597641	-358339	-0.67	4.92E-02	9.86E-01	Distal Intergenic
Ramac	293058	chr1	143585083	143585383	1408	-0.78	4.92E-02	9.86E-01	Promoter (1-2kb)
Rpl36al	81769	chr18	1505194	1505494	1016	1.15	4.92E-02	9.86E-01	Promoter (1-2kb)
Gab2	84477	chr1	162056228	162056528	-26514	-0.78	4.92E-02	9.86E-01	Distal Intergenic
Prkca	24680	chr10	96607384	96607684	-22437	0.62	4.93E-02	9.86E-01	Distal Intergenic
Amotl2	65157	chr8	111222316	111222616	11505	0.76	4.93E-02	9.86E-01	Exon (NM_031717/65157, exon 7 of 10)
Kat6a	306571	chr16	73995954	73996254	24496	-0.64	4.93E-02	9.86E-01	Intron (NM_001100570/306571, intron 1 of 16)
Bbs2	113948	chr19	11385532	11385832	-192	-0.61	4.94E-02	9.86E-01	Promoter (<=1kb)
Igflr1	499126	chr1	89018897	89019197	-1144	-1.16	4.94E-02	9.86E-01	Promoter (1-2kb)
Med29	292751	chr1	85371477	85371777	3189	-0.87	4.94E-02	9.86E-01	Intron (NM_001106237/292751, intron 3 of 3)
RGD1563159	361312	chr18	30800468	30800768	1474	-0.89	4.95E-02	9.86E-01	Promoter (1-2kb)
Cep120	307302	chr18	48717948	48718248	2224	-0.89	4.95E-02	9.86E-01	Promoter (2-3kb)
Gosr2	64154	chr10	91753904	91754204	1877	-0.73	4.96E-02	9.86E-01	Promoter (1-2kb)
Ccnd2	64033	chr4	160013851	160014151	-316644	0.83	4.96E-02	9.86E-01	Distal Intergenic
Dtx2	304591	chr12	23702190	23702490	25074	0.66	4.96E-02	9.86E-01	Intron (NM_001107157/304591, intron 5 of 11)
Mdfic	362325	chr4	41903066	41903366	-299472	-0.53	4.96E-02	9.86E-01	Exon (NM_001271104/500037, exon 12 of 19)
Rit1	499652	chr2	188083107	188083407	-4079	1.18	4.96E-02	9.86E-01	Distal Intergenic
Clcn6	295586	chr5	164842090	164842390	2031	-0.82	4.96E-02	9.86E-01	Promoter (2-3kb)
Tpp1	83534	chr1	170591817	170592117	2042	-0.81	4.96E-02	9.86E-01	Promoter (2-3kb)
Rpl28	64638	chr1	72624072	72624372	-2229	-0.56	4.96E-02	9.86E-01	Promoter (2-3kb)
Prickle3	317380	chrX	15694212	15694512	-739	-1.01	4.97E-02	9.86E-01	Promoter (<=1kb)
Mllt3	114510	chr5	106229780	106230080	-27219	0.91	4.97E-02	9.86E-01	Distal Intergenic
Atp6v0e1	94170	chr10	16778166	16778466	14464	-1.05	4.98E-02	9.88E-01	Intron (NM_053578/94170, intron 2 of 3)
Ldhal6b	369018	chr1	45888238	45888538	420738	1.54	5.00E-02	9.89E-01	Distal Intergenic
