# Supplementary material for: GRWD1-WDR5-MLL2 Epigenetic Complex Mediates H3K4me3 Mark and Is Essential for Kaposi’s Sarcoma-Associated Herpesvirus-Induced Cellular Transformation
Source: mBio. 2021 Dec 21;12(6):e03431-21. doi: 10.1128/mbio.03431-21 (PMC8689518; doi:10.1128/mbio.03431-21)
Supplement: TABLE S3 [file mbio.03431-21-st003.pdf]

TABLE S3A Common and distinct genes altered in MM cells following knockdown of GRWD1, WDR5 and MLL2

GRWD1	WDR5	MLL2	GRWD1&WDR5	GRWD1&MLL2	WDR5&MLL2	GRWD1&WDR5&MLL2
AABR07000658.1	AABR07000222.1	AABR07000222.1	AABR07000658.1	AABR07000658.1	AABR07000658.1	AABR07000658.1
AABR07001512.1	AABR07000658.1	AABR07000658.1	AABR07001512.1	AABR07001512.1	AABR07001512.1	AABR07001512.1
AABR07002564.1	AABR07001512.1	AABR07001389.1	AABR07002564.1	AABR07002564.1	AABR07002564.1	AABR07002564.1
AABR07005838.1	AABR07002564.1	AABR07001512.1	AABR07005838.1	AABR07005838.1	AABR07002711.1	AABR07005838.1
AABR07007134.1	AABR07002711.1	AABR07002564.1	AABR07010705.1	AABR07015180.1	AABR07002774.4	AABR07015180.1
AABR07010705.1	AABR07002774.4	AABR07002711.1	AABR07013288.4	AABR07019399.1	AABR07004269.4	AABR07026311.1
AABR07011697.1	AABR07003537.1	AABR07002774.4	AABR07015180.1	AABR07026311.1	AABR07005838.1	AABR07028352.1
AABR07012129.1	AABR07004269.4	AABR07004269.4	AABR07025328.1	AABR07028352.1	AABR07007690.1	AABR07044366.1
AABR07013288.4	AABR07005004.1	AABR07004881.1	AABR07026311.1	AABR07036855.1	AABR07012575.1	AABR07049223.1
AABR07015006.1	AABR07005838.1	AABR07005838.1	AABR07026797.1	AABR07044273.1	AABR07015180.1	AABR07053516.1
AABR07015180.1	AABR07007690.1	AABR07006860.2	AABR07027753.3	AABR07044366.1	AABR07018244.2	AABR07055919.1
AABR07019399.1	AABR07007758.2	AABR07007690.1	AABR07028352.1	AABR07049223.1	AABR07024500.1	AABR07062599.1
AABR07021384.1	AABR07010041.1	AABR07008097.1	AABR07038926.1	AABR07049695.3	AABR07025301.1	AABR07068852.1
AABR07021596.4	AABR07010705.1	AABR07009357.2	AABR07038948.2	AABR07053516.1	AABR07025787.1	AABR07072853.1
AABR07025328.1	AABR07012100.2	AABR07011697.1	AABR07044366.1	AABR07055919.1	AABR07026311.1	AC115273.1
AABR07026302.1	AABR07012575.1	AABR07011996.1	AABR07044959.1	AABR07062599.1	AABR07026424.1	AC129365.1
AABR07026311.1	AABR07013288.4	AABR07012054.1	AABR07049223.1	AABR07068852.1	AABR07027811.2	Adrb2
AABR07026797.1	AABR07013701.1	AABR07012575.1	AABR07053516.1	AABR07072853.1	AABR07028352.1	Ahcy
AABR07027744.1	AABR07014550.1	AABR07012795.1	AABR07055919.1	AC115273.1	AABR07030647.1	Alyref
AABR07027753.3	AABR07014756.1	AABR07013255.1	AABR07055919.2	AC127920.1	AABR07032480.1	Anapc15
AABR07028352.1	AABR07014836.1	AABR07014804.1	AABR07062599.1	AC129365.1	AABR07033023.1	Ankrd49
AABR07029613.1	AABR07014996.1	AABR07015180.1	AABR07066944.1	AY172581.21	AABR07033047.1	Anp32e
AABR07030162.1	AABR07015180.1	AABR07018058.1	AABR07068127.1	Adamtsl4	AABR07038269.1	Arhgap11a
AABR07032523.1	AABR07017999.1	AABR07018244.2	AABR07068316.1	Adrb2	AABR07043654.1	Asf1b
AABR07033745.1	AABR07018244.2	AABR07019399.1	AABR07068852.1	Ahcy	AABR07044366.1	Aspm
AABR07034263.1	AABR07019341.1	AABR07021734.1	AABR07072853.1	Aida	AABR07044420.2	Atad2
AABR07034362.1	AABR07024500.1	AABR07024500.1	AC112350.1	Alyref	AABR07044940.1	Atad5
AABR07034767.1	AABR07024593.2	AABR07024972.1	AC115273.1	Anapc15	AABR07046628.1	Aurka
AABR07035541.3	AABR07025301.1	AABR07025301.1	AC119015.4	Ankrd49	AABR07049223.1	Aurkb
AABR07036855.1	AABR07025328.1	AABR07025787.1	AC129365.1	Anln	AABR07053516.1	B2m
AABR07037203.1	AABR07025787.1	AABR07026311.1	AC130391.5	Anp32b	AABR07053707.1	Bard1
AABR07038926.1	AABR07026311.1	AABR07026424.1	AC134224.3	Anp32e	AABR07055834.1	Birc5
AABR07038948.2	AABR07026424.1	AABR07027407.1	Abhd4	Arhgap11a	AABR07055919.1	Blcap
AABR07043951.1	AABR07026797.1	AABR07027810.4	Adamts1	Arl2bp	AABR07058464.1	Bora
AABR07044273.1	AABR07027009.1	AABR07027811.2	Adrb2	Asf1b	AABR07059171.1	Borcs6
AABR07044366.1	AABR07027575.1	AABR07028027.1	Aen	Aspm	AABR07060291.1	Brca1
AABR07044574.1	AABR07027753.3	AABR07028352.1	Ahcy	Atad2	AABR07060593.1	Bub1
AABR07044711.1	AABR07027811.2	AABR07029195.1	Alg8	Atad5	AABR07062599.1	Bub1b
AABR07044959.1	AABR07028349.1	AABR07029198.1	Alyref	Aurka	AABR07063829.2	Cbx3
AABR07049038.1	AABR07028352.1	AABR07029958.2	Anapc15	Aurkb	AABR07066516.1	Ccn4
AABR07049223.1	AABR07028779.1	AABR07029970.1	Ankrd1	B2m	AABR07066529.1	Ccna2
AABR07049353.1	AABR07029198.1	AABR07030647.1	Ankrd49	Bard1	AABR07067526.1	Ccnb1
AABR07049578.1	AABR07029958.1	AABR07031972.1	Anp32e	Birc5	AABR07067600.1	Ccne1
AABR07049695.2	AABR07030019.1	AABR07032480.1	Ap5b1	Blcap	AABR07068417.2	Ccne2
AABR07049695.3	AABR07030156.2	AABR07032856.1	Apol9a	Bora	AABR07068852.1	Ccnf
AABR07053516.1	AABR07030494.1	AABR07033023.1	Apold1	Borcs6	AABR07069282.1	Cdc20
AABR07055919.1	AABR07030603.1	AABR07033047.1	Arhgap11a	Brca1	AABR07072133.1	Cdc25b
AABR07055919.2	AABR07030603.2	AABR07035383.1	Arpin	Bub1	AABR07072853.1	Cdc45
AABR07057302.1	AABR07030647.1	AABR07035486.1	Asf1b	Bub1b	AC094126.2	Cdc6
AABR07058514.3	AABR07030861.1	AABR07036855.1	Aspm	Card19	AC094643.2	Cdc7
AABR07058534.1	AABR07030911.2	AABR07037356.1	Atad2	Cbx3	AC095390.1	Cdca2
AABR07060293.1	AABR07031089.1	AABR07038019.1	Atad5	Ccdc17	AC098125.3	Cdca3
AABR07061902.1	AABR07031666.1	AABR07038269.1	Atxn7l3b	Ccn4	AC105515.1	Cdca4
AABR07062477.2	AABR07031675.1	AABR07038895.2	Aurka	Ccna2	AC115273.1	Cdca7
AABR07062599.1	AABR07032480.1	AABR07038939.1	Aurkb	Ccnb1	AC119762.5	Cdca8
AABR07064634.1	AABR07032751.1	AABR07039307.1	B2m	Ccne1	AC123425.1	Cdk1
AABR07065113.1	AABR07033023.1	AABR07041078.1	Bard1	Ccne2	AC129365.1	Cdk2
AABR07066944.1	AABR07033047.1	AABR07042301.1	Becn1	Ccnf	AC133265.1	Cdkn1a
AABR07068127.1	AABR07034315.1	AABR07043654.1	Bhlhe41	Cdc20	AC136867.1	Cdkn2c
AABR07068316.1	AABR07034573.1	AABR07044273.1	Birc5	Cdc25b	AY172581.8	Cdt1
AABR07068852.1	AABR07035539.1	AABR07044366.1	Blcap	Cdc45	Aaas	Cenpa
AABR07069433.1	AABR07035541.2	AABR07044420.2	Bora	Cdc6	Actr1b	Cenpe
AABR07072853.1	AABR07035780.4	AABR07044635.1	Borcs6	Cdc7	Adrb2	Cenpf
AABR07072916.1	AABR07035796.1	AABR07044940.1	Brca1	Cdca2	Ahcy	Cenph
AC097153.2	AABR07036247.1	AABR07046628.1	Brca2	Cdca3	Akt1	Cenpk
AC106191.1	AABR07036498.1	AABR07049223.1	Btn2a2	Cdca4	Aktip	Cenpo
AC111734.1	AABR07038269.1	AABR07049695.3	Bub1	Cdca7	Alyref	Cenpu
AC112350.1	AABR07038873.1	AABR07049769.2	Bub1b	Cdca8	Amdhd2	Cenpw
AC115273.1	AABR07038926.1	AABR07050545.1	Casp8ap2	Cdk1	Anapc15	Cep295
AC118439.1	AABR07038948.2	AABR07051190.1	Cbx3	Cdk2	Ankle1	Cep55
AC119015.4	AABR07039037.1	AABR07053169.1	Ccdc47	Cdk20	Ankrd49	Chaf1a

AC123213.1	AABR07039210.1	AABR07053516.1	Ccn4	Cdkn1a	Anp32e	Chaf1b
AC124839.1	AABR07039210.2	AABR07053667.1	Ccna2	Cdkn2c	Anxa2	Chtf18
AC125248.1	AABR07040840.1	AABR07053669.1	Ccnb1	Cdt1	Api5	Cip2a
AC127920.1	AABR07040892.1	AABR07053707.1	Ccne1	Cenpa	Arhgap11a	Ckap2
AC129365.1	AABR07041627.1	AABR07054266.1	Ccne2	Cenpe	Arl6ip1	Ckap2l
AC130391.5	AABR07041778.1	AABR07054368.1	Ccnf	Cenpf	Asf1b	Ckap5
AC130555.1	AABR07042866.1	AABR07055280.1	Cdc20	Cenph	Aspm	Cks1b
AC132752.2	AABR07043654.1	AABR07055834.1	Cdc25b	Cenpk	Atad2	Cks2
AC134224.3	AABR07044366.1	AABR07055919.1	Cdc45	Cenpo	Atad5	Clspn
AC139392.1	AABR07044375.1	AABR07056686.1	Cdc6	Cenpu	Atg12	Clu
AY172581.2	AABR07044375.2	AABR07058464.1	Cdc7	Cenpw	Atp6v1d	Cryba4
AY172581.21	AABR07044420.2	AABR07059171.1	Cdca2	Cep295	Aunip	Ctdsp2
Abhd4	AABR07044421.1	AABR07060291.1	Cdca3	Cep55	Aurka	Cycs
Adamts1	AABR07044940.1	AABR07060593.1	Cdca4	Chaf1a	Aurkb	Dctpp1
Adamtsl4	AABR07044959.1	AABR07061964.1	Cdca7	Chaf1b	B2m	Ddx11
Ado	AABR07045322.1	AABR07062599.1	Cdca8	Chtf18	Bard1	Dek
Adrb2	AABR07046628.1	AABR07063829.2	Cdk1	Cip2a	Bax	Dlgap5
Adrb3	AABR07047089.1	AABR07064998.2	Cdk2	Ckap2	Bdh2	Dnajc9
Aen	AABR07047219.1	AABR07066516.1	Cdkn1a	Ckap2l	Birc5	Dnmt1
Ahctf1	AABR07049223.1	AABR07066529.1	Cdkn2c	Ckap5	Blcap	Dscc1
Ahcy	AABR07049405.1	AABR07067526.1	Cdt1	Cks1b	Bloc1s4	Dsn1
Aida	AABR07049755.1	AABR07067583.1	Cebpd	Cks2	Bora	Dtl
Akap12	AABR07052430.1	AABR07067600.1	Cenpa	Clec2d2	Borcs6	Dut
Akirin1	AABR07053136.1	AABR07068154.1	Cenpe	Clspn	Brca1	E2f2
Akr1c14	AABR07053152.1	AABR07068253.1	Cenpf	Clu	Btg2	E2f8
Aldh2	AABR07053500.1	AABR07068285.2	Cenph	Cryba4	Bub1	Ect2
Alg8	AABR07053516.1	AABR07068417.2	Cenpk	Ctdnep1	Bub1b	Eme1
Alyref	AABR07053707.1	AABR07068852.1	Cenpl	Ctdsp2	Cbx3	Ercc6l
Anapc15	AABR07055834.1	AABR07069282.1	Cenpm	Ctdspl	Ccdc43	Espl1
Anapc2	AABR07055917.1	AABR07070270.1	Cenpo	Cycs	Ccn4	Exo1
Angpt4	AABR07055919.1	AABR07070275.1	Cenpu	Dctpp1	Ccna2	Exosc2
Angptl4	AABR07055919.2	AABR07071000.1	Cenpw	Ddx11	Ccnb1	Exosc8
Ankrd1	AABR07057190.1	AABR07072133.1	Cep250	Dek	Ccne1	Ezh2
Ankrd49	AABR07057250.1	AABR07072853.1	Cep295	Dlgap5	Ccne2	Fadd
Anln	AABR07057423.1	AC094126.2	Cep55	Dnajb1	Ccnf	Fam111a
Anp32b	AABR07057683.1	AC094643.2	Cep76	Dnajc9	Cdc20	Fam83d
Anp32e	AABR07058464.1	AC095390.1	Chaf1a	Dnmt1	Cdc25b	Fancb
Ap5b1	AABR07058519.1	AC098125.3	Chaf1b	Dnpep	Cdc45	Fancd2
Aplp2	AABR07059171.1	AC098459.1	Chpf2	Dpp9	Cdc6	Fanci
Apol9a	AABR07059925.1	AC105515.1	Chtf18	Dscc1	Cdc7	Fbl
Apold1	AABR07060291.1	AC108572.4	Cip2a	Dsn1	Cdca2	Fbxo5
Arf5	AABR07060593.1	AC115273.1	Cit	Dtl	Cdca3	Fen1
Arhgap11a	AABR07061614.1	AC119111.1	Ckap2	Dut	Cdca4	Fignl1
Arl2bp	AABR07062154.1	AC119762.5	Ckap2l	Dynlt3	Cdca7	Foxm1
Arl4a	AABR07062290.1	AC123425.1	Ckap5	E2f2	Cdca8	G2e3
Arl5a	AABR07062599.1	AC126897.1	Cks1b	E2f8	Cdhr1	Gaa
Arl6ip4	AABR07063279.1	AC127920.1	Cks2	Ect2	Cdk1	Gadd45a
Arpin	AABR07063829.2	AC128212.1	Clspn	Eme1	Cdk2	Gas2l3
Asf1b	AABR07063855.1	AC129365.1	Clu	Entpd4	Cdkn1a	Gdf15
Aspm	AABR07064349.1	AC132627.2	Cryba4	Ercc6l	Cdkn2c	Gemin6
Atad2	AABR07065438.1	AC133265.1	Ctdsp2	Espl1	Cdt1	Gen1
Atad5	AABR07065625.2	AC136867.1	Ctsa	Etfrf1	Cenpa	Gins1
Atxn7l3b	AABR07066516.1	AC141220.3	Cycs	Exo1	Cenpe	Gins4
Aurka	AABR07066529.1	AC142180.1	Dbn1	Exosc2	Cenpf	Gmnn
Aurkb	AABR07066944.1	AC142458.1	Dctpp1	Exosc8	Cenph	Gpsm2
B2m	AABR07067526.1	AC242615.2	Ddr1	Ezh2	Cenpk	Grina
B3gnt7	AABR07067600.1	AY172581.21	Ddx11	Fadd	Cenpo	Gtse1
Bag2	AABR07068127.1	AY172581.8	Dek	Fam107b	Cenpt	H2afz
Bard1	AABR07068316.1	Aaas	Diaph1	Fam111a	Cenpu	Hapln3
Baz1b	AABR07068417.2	Aamp	Dlgap5	Fam136a	Cenpw	Hat1
Becn1	AABR07068852.1	Abcc5	Dnajc9	Fam83d	Cep295	Haus4
Bend6	AABR07069219.1	Abtb1	Dnmt1	Fancb	Cep55	Hirip3
Bhlhe41	AABR07069282.1	Actr1b	Dscc1	Fancd2	Chaf1a	Hjurp
Bid	AABR07071779.2	Adamtsl4	Dsn1	Fanci	Chaf1b	Hmmr
Birc5	AABR07071814.1	Add1	Dtl	Fbl	Chtf18	Hnrnpul1
Blcap	AABR07072133.1	Adgra2	Dtymk	Fbxo5	Cilp	Hspa14
Bloc1s2	AABR07072236.1	Adrb2	Dusp1	Fen1	Cip2a	Hyls1
Bloc1s3	AABR07072809.1	Agpat1	Dut	Fignl1	Ckap2	Idh2
Bok	AABR07072853.1	Ahcy	E2f2	Fkbp1a	Ckap2l	Incenp
Bora	AABR07073181.1	Ahsa1	E2f8	Fkbp4	Ckap5	Ing1
Borcs6	AC094126.2	Ahsp	Ect2	Foxm1	Cks1b	Kif11
Brca1	AC094217.1	Aida	Emc8	G2e3	Cks2	Kif18b
Brca2	AC094643.2	Akr1c12	Eme1	Gaa	Cldnd1	Kif20a

Bst2	AC095390.1	Akt1	Ercc6l	Gadd45a	Clic1	Kif20b
Btn2a2	AC098125.3	Akt2	Eri1	Gas2l3	Clspn	Kif22
Bub1	AC099089.1	Aktip	Esco2	Gdf15	Clu	Kif23
Bub1b	AC103090.1	Alas1	Espl1	Gemin6	Cnot9	Kif2c
Bub3	AC103335.1	Aldh5a1	Exo1	Gen1	Cntf	Kif4a
C1qtnf5	AC105515.1	Alg10	Exosc2	Gins1	Cox6a2	Kifc1
Cactin	AC106663.2	Alyref	Exosc8	Gins4	Crip2	Knstrn
Cadm4	AC108572.3	Amdhd2	Ezh2	Gmnn	Cryba4	Kntc1
Cald1	AC109048.1	Amfr	Fadd	Gpsm2	Cse1l	LOC100359539
Capn1	AC109542.3	Anapc1	Fam111a	Grina	Cst3	LOC100359600
Card19	AC110351.1	Anapc15	Fam214b	Gtse1	Ctdsp2	LOC100909474
Carhsp1	AC112018.1	Ankle1	Fam83d	H2afz	Ctsb	LOC102546716
Casp2	AC112350.1	Ankrd13a	Fanca	Hapln3	Cycs	LOC361346
Casp8ap2	AC115159.2	Ankrd49	Fancb	Hat1	Cyp1b1	Lig4
Cbx3	AC115273.1	Ankrd52	Fancd2	Haus4	Dclre1b	Lin54
Cbx6	AC116220.3	Anln	Fancg	Haus8	Dctpp1	Lmnb1
Ccdc17	AC119015.4	Anp32b	Fanci	Hirip3	Ddit4	Lmnb2
Ccdc34	AC119762.5	Anp32e	Fbl	Hjurp	Ddx11	Lsm3
Ccdc47	AC119762.6	Anxa1	Fbxo5	Hmmr	Ddx39a	Lsm5
Cchcr1	AC119762.7	Anxa2	Fen1	Hnrnpd	Dek	Lsm8
Ccn4	AC123425.1	Aox1	Fignl1	Hnrnpul1	Dlgap5	Mad2l1
Ccna2	AC125873.1	Apbb1	Filip1l	Hnrnpul2	Dnajb9	Mcm10
Ccnb1	AC128960.1	Api5	Fmnl3	Hspa14	Dnajc9	Mcm2
Ccne1	AC129365.1	Aqp1	Foxm1	Hyls1	Dnmt1	Mcm3
Ccne2	AC130391.5	Areg	G2e3	Idh2	Dpp7	Mcm4
Ccnf	AC133265.1	Arfip2	G3bp2	Ifi27	Dscc1	Mcm5
Ccsap	AC134224.3	Arhgap11a	Gaa	Incenp	Dsel	Mcm6
Cd14	AC136867.1	Arl2bp	Gadd45a	Ing1	Dsn1	Mcm7
Cd200	AC141169.2	Arl4a	Gas2l3	Jpt2	Dstn	Mdc1
Cd44	AC141959.1	Arl6ip1	Gdf15	Kif11	Dtl	Mdm2
Cd48	AC242953.1	Arpc5l	Gemin6	Kif18b	Dut	Mis18a
Cd63	AY172581.1	Arrdc3	Gen1	Kif20a	Dvl3	Mki67
Cdc20	AY172581.8	Asf1b	Ghitm	Kif20b	E2f2	Msh6
Cdc25b	Aaas	Asl	Ginm1	Kif22	E2f8	Mybl2
Cdc26	Aarsd1	Aspm	Gins1	Kif23	Ect2	Nasp
Cdc34	Abca3	Atad2	Gins3	Kif2c	Eef2	Ncapd2
Cdc45	Abcb8	Atad5	Gins4	Kif4a	Ehd4	Ncapd3
Cdc6	Abhd4	Atcay	Glb1l	Kifc1	Eif4ebp2	Ncapg
Cdc7	Abo3	Atg12	Gmnn	Klhl24	Elk3	Ncapg2
Cdca2	Acpp	Atp6v1a	Gpsm2	Knstrn	Eme1	Ncaph
Cdca3	Actg1	Atp6v1b2	Grina	Kntc1	Eme2	Ncaph2
Cdca4	Actr1b	Atp6v1d	Grwd1	LOC100359539	Emg1	Ndc80
Cdca7	Adam19	Atp6v1f	Gtse1	LOC100359600	Ercc6l	Necab3
Cdca8	Adamts1	Atp6v1g1	H2afz	LOC100909474	Espl1	Nrm
Cdk1	Adcy7	Aunip	Hapln3	LOC102546716	Esrra	Nsmce4a
Cdk2	Adipor1	Aurka	Hat1	LOC361346	Etv5	Nuf2
Cdk20	Adm	Aurkb	Haus3	Lamb1	Exo1	Nup107
Cdkn1a	Adrb2	Avpr1a	Haus4	Lgals3bp	Exosc2	Nup188
Cdkn2c	Aen	B2m	Haus5	Lig4	Exosc8	Nup85
Cdt1	Aga	Bard1	Hirip3	Lin54	Ezh2	Nusap1
Cebpd	Ahcy	Bax	Hist1h1d	Lmf2	F2rl1	Nxt1
Cebpg	Aif1l	Bbs10	Hist1h2bk	Lmnb1	Fadd	Orc1
Cebpzos	Ajuba	Bcs1l	Hjurp	Lmnb2	Fam111a	Orc6
Cenpa	Ak2	Bdh2	Hmgb2	Lsm3	Fam83d	P2rx4
Cenpb	Akap2	Birc5	Hmgn2	Lsm5	Fam84a	Pa2g4
Cenpc	Akap5	Blcap	Hmmr	Lsm8	Fancb	Pask
Cenpe	Akip1	Bloc1s4	Hnrnpul1	Mad2l1	Fancd2	Paxip1
Cenpf	Akt1	Bloc1s6	Hspa14	Mapk6	Fanci	Pbk
Cenph	Aktip	Bmf	Hyls1	Mcm10	Fbl	Pclaf
Cenpi	Alg8	Bnip3l	Icam1	Mcm2	Fbxo5	Pcna
Cenpk	Alkbh7	Bora	Idh2	Mcm3	Fen1	Pfas
Cenpl	Alyref	Borcs6	Ier5	Mcm4	Fignl1	Phf5a
Cenpm	Amdhd2	Bpgm	Il17rc	Mcm5	Fktn	Pif1
Cenpo	Amigo1	Brca1	Ilf2	Mcm6	Foxm1	Pimreg
Cenpu	Amotl2	Btbd19	Incenp	Mcm7	Fuca1	Plekhb2
Cenpw	Anapc15	Btg2	Ing1	Mdc1	Fut4	Plk1
Cep250	Anapc16	Bub1	Irf7	Mdm2	G2e3	Plk2
Cep295	Angptl2	Bub1b	Itga7	Mir27a	G3bp1	Plk4
Cep55	Ankh	C1qtnf6	Kif11	Mirlet7i	Gaa	Pmf1
Cep57	Ankle1	C1r	Kif14	Mis18a	Gadd45a	Pold2
Cep76	Ankrd1	C1rl	Kif15	Mki67	Gas2l3	Pole
Cerk	Ankrd12	Cad	Kif18b	Msh6	Gas6	Pole2
Cggbp1	Ankrd34a	Car11	Kif20a	Mybl2	Gdf15	Ppm1g

Chaf1a	Ankrd49	Card19	Kif20b	Nasp	Gemin6	Ppp2r5d
Chaf1b	Ano8	Carm1	Kif22	Ncapd2	Gen1	Prc1
Chchd1	Anp32e	Cavin1	Kif23	Ncapd3	Gfpt1	Prim1
Chpf2	Anxa2	Cbfb	Kif2c	Ncapg	Gigyf1	Ptma
Chst12	Anxa3	Cbx3	Kif4a	Ncapg2	Gins1	Ptpa
Chtf18	Ap5b1	Ccdc115	Kifc1	Ncaph	Gins2	Pttg1
Chtf8	Aph1a	Ccdc17	Knl1	Ncaph2	Gins4	RGD1560010
Cip2a	Api5	Ccdc43	Knstrn	Ndc80	Gltp	RT1-CE10
Cit	Apol9a	Ccdc96	Kntc1	Necab3	Gmnn	RT1-N3
Cited2	Apold1	Ccn4	LOC100294508	Nit1	Gnptg	Racgap1
Ckap2	Aqp1	Ccna2	LOC100359539	Npm3	Gpr108	Rad21
Ckap2l	Arg1	Ccnb1	LOC100359600	Nrm	Gpsm2	Rad51ap1
Ckap5	Arhgap11a	Ccnd3	LOC100365363	Nsmce4a	Grina	Rad54l
Cks1b	Arhgap19	Ccne1	LOC100909474	Nuf2	Gtse1	Rasl11b
Cks2	Arhgap21	Ccne2	LOC100911361	Nup107	H2afz	Rbbp7
Clec2d2	Arhgap29	Ccnf	LOC102546716	Nup188	Hapln3	Reck
Clspn	Arhgap33	Ccng1	LOC361346	Nup85	Haspin	Rfc2
Clu	Arhgap5	Ccnt2	LOC691807	Nusap1	Hat1	Rfc3
Cnot6	Arhgdia	Ccpg1	Lig4	Nxt1	Haus4	Rfc5
Cnpy4	Arl6ip1	Cdc20	Lin54	Orc1	Hbp1	Rpa2
Cryba4	Arl8a	Cdc25b	Lmnb1	Orc6	Hcfc1	Rpa3
Ctdnep1	Armcx2	Cdc42ep1	Lmnb2	Ormdl2	Hilpda	Rrm1
Ctdsp2	Armcx3	Cdc42ep4	Lpar6	P2rx4	Hirip3	Rrm2
Ctdspl	Arpc1a	Cdc42ep5	Lrrcc1	Pa2g4	Hist1h1c	Samd9
Ctsa	Arpin	Cdc45	Lsm3	Parp2	Hjurp	Sapcd2
Cxcl12	Arrdc4	Cdc6	Lsm5	Pask	Hmmr	Sf3b3
Cxcl16	Arse	Cdc7	Lsm8	Paxip1	Hmox1	Sgo1
Cycs	Asah1	Cdca2	Mad2l1	Pbk	Hnrnpul1	Sgo2
Cyp51	Asb6	Cdca3	Mastl	Pclaf	Hsdl1	Shcbp1
Cyren	Asf1b	Cdca4	Mcm10	Pcna	Hsp90ab1	Siva1
Dad1	Asmtl	Cdca7	Mcm2	Pfas	Hsp90b1	Ska1
Dbn1	Aspm	Cdca8	Mcm3	Phf5a	Hspa14	Ska3
Dcakd	Atad2	Cdh3	Mcm4	Pif1	Hspa5	Slbp
Dck	Atad5	Cdhr1	Mcm5	Pimreg	Hyls1	Slc25a10
Dctpp1	Atcay	Cdk1	Mcm6	Plekhb2	Hyou1	Slc38a7
Ddias	Atf5	Cdk10	Mcm7	Plk1	Idh2	Slc46a1
Ddr1	Atg12	Cdk2	Mdc1	Plk2	Ifrd2	Slfn13
Ddx11	Atp13a2	Cdk20	Mdm2	Plk4	Incenp	Smc2
Dek	Atp1a1	Cdk5	Melk	Pmf1	Ing1	Smc4
Depdc1	Atp2a3	Cdkn1a	Mgme1	Pnpo	Iqgap3	Snrpa
Depp1	Atp5f1a	Cdkn2c	Mir125b1	Pold2	Itm2b	Spag5
Diaph1	Atp5f1e	Cdr2	Mis18a	Pole	Itm2c	Spc25
Dlgap5	Atp6v1d	Cdt1	Mis18bp1	Pole2	Kif11	Srsf1
Dlx1	Atp8b2	Celf1	Mki67	Ppm1g	Kif18b	Srsf2
Dnajb1	Atrn	Cenpa	Mms22l	Ppp2r5d	Kif20a	Srsf7
Dnajc9	Atxn7l3b	Cenpe	Mrpl14	Prc1	Kif20b	Stil
Dnmt1	Aunip	Cenpf	Mrpl18	Prim1	Kif22	Stip1
Dnpep	Aurka	Cenph	Msh6	Prss23	Kif23	Stmn1
Donson	Aurkb	Cenpk	Msln	Ptges3	Kif2c	Suv39h1l1
Dpp9	Avpi1	Cenpo	Mybl2	Ptma	Kif4a	Tacc3
Dscc1	B2m	Cenpt	Myh10	Ptpa	Kifc1	Tceanc
Dsn1	B3gat3	Cenpu	Nabp1	Ptprv	Klhl23	Tcf19
Dtl	B9d2	Cenpw	Nans	Pttg1	Knstrn	Tedc1
Dtx3l	Bahd1	Cep295	Nasp	RGD1560010	Kntc1	Tedc2
Dtymk	Bak1	Cep55	Ncapd2	RGD1562378	Kpna2	Tex30
Dusp1	Bard1	Cep68	Ncapd3	RT1-CE10	LOC100359539	Ticrr
Dut	Bax	Cfap298	Ncapg	RT1-N3	LOC100359583	Timeless
Dynlt3	Bcar1	Cfh	Ncapg2	Rac1	LOC100359600	Tipinl1
E2f2	Bcat1	Cfp	Ncaph	Racgap1	LOC100365839	Tk1
E2f7	Bccip	Ch25h	Ncaph2	Rad21	LOC100909474	Tmem97
E2f8	Bdh2	Chaf1a	Ndc80	Rad51ap1	LOC102546716	Tmpo
Ect2	Becn1	Chaf1b	Necab3	Rad54l	LOC102549726	Tnfaip8l1
Edem1	Bgn	Chrm3	Necap1	Ranbp1	LOC102553785	Tonsl
Ell3	Bhlhe41	Chst12	Nemp1	Rasl11b	LOC102555453	Top2a
Emc8	Bicra	Chst2	Nono	Rbbp7	LOC103691238	Topbp1
Eme1	Bin1	Chtf18	Nrm	Reck	LOC108351584	Tp53inp1
Entpd4	Birc5	Cilp	Nsl1	Rfc2	LOC108351936	Tpx2
Ercc6l	Blcap	Cip2a	Nsmce4a	Rfc3	LOC361346	Traip
Eri1	Bloc1s4	Ckap2	Nucks1	Rfc5	Lamp2	Trim47
Esco2	Bmp1	Ckap2l	Nuf2	Rpa2	Lgmn	Trim59
Espl1	Bora	Ckap5	Nup107	Rpa3	Lig4	Tspan17
Etfrf1	Borcs6	Cks1b	Nup188	Rpp21	Lin54	Ttk
Exo1	Brca1	Cks2	Nup35	Rrm1	Lmnb1	Tuba1b

Exoc8	Brca2	Clba1	Nup85	Rrm2	Lmnb2	Tubb5
Exosc2	Brpf1	Cldn12	Nusap1	Samd9	Lonrf2	Tubgcp2
Exosc3	Bsg	Cldnd1	Nxt1	Sapcd2	Lrrc73	Ube2c
Exosc8	Btg2	Clec2d2	Oip5	Set	Lsm2	Ube2s
Ezh2	Btn2a2	Clic1	Orc1	Sf3b3	Lsm3	Ube2t
F2r	Bub1	Clspn	Orc6	Sgo1	Lsm5	Uhrf1
Fadd	Bub1b	Clu	P2rx4	Sgo2	Lsm8	Ung
Fam107b	C1galt1	Cmpk1	Pa2g4	Shcbp1	Lta4h	Usp1
Fam111a	C1s	Cmtm6	Pagr1	Siva1	Lypla1	Wdhd1
Fam136a	C2cd2	Cmtr2	Pask	Ska1	Mad2l1	Xrcc2
Fam168b	Calr	Cnih1	Paxip1	Ska3	Maf	Yod1
Fam214b	Casp12	Cnnm4	Pbk	Slbp	Mapre3	Zfp513
Fam83d	Casp8ap2	Cnot9	Pclaf	Slc16a1	Mcm10	Zw10
Fam91a1	Cavin4	Cnpy2	Pcna	Slc25a10	Mcm2	Zwilch
Fanca	Cbfb	Cntf	Pfas	Slc38a7	Mcm3	
Fancb	Cbx3	Col12a1	Phf19	Slc46a1	Mcm4	
Fancd2	Cc2d1a	Commd4	Phf5a	Slfn13	Mcm5	
Fancg	Cc2d1b	Commd8	Pif1	Smc2	Mcm6	
Fanci	Ccdc126	Coro1c	Pimreg	Smc4	Mcm7	
Fbl	Ccdc163	Coro6	Plekhb2	Snrpa	Mdc1	
Fbxo5	Ccdc43	Cotl1	Plk1	Spag5	Mdm2	
Fen1	Ccdc47	Cox6a2	Plk2	Spc25	Med21	
Fez2	Ccdc74a	Cp	Plk3	Spns1	Mir23a	
Fhl1	Ccdc80	Cpd	Plk4	Srsf1	Mir3064	
Fignl1	Ccdc9b	Cpt1c	Pmf1	Srsf2	Mir3564	
Filip1l	Ccl20	Crat	Pold2	Srsf3	Mir3568	
Fitm2	Ccn4	Crebzf	Pole	Srsf7	Mir374b	
Fkbp1a	Ccna2	Crip2	Pole2	Stil	Mir6334	
Fkbp4	Ccnb1	Cry2	Pole3	Stip1	Mis18a	
Fkbp5	Ccne1	Cryba4	Polr2c	Stmn1	Mk1	
Fmnl3	Ccne2	Cse1l	Ppm1g	Suv39h1l1	Mki67	
Fmo3	Ccnf	Csprs	Ppp1ca	Tacc3	Mkrn3	
Fmod	Ccp110	Csrp1	Ppp2r5d	Tceanc	Msh2	
Fn3krp	Cct4	Cst3	Pradc1	Tcf19	Msh6	
Foxm1	Cct7	Cstf2	Prc1	Tedc1	Mthfr	
Fst	Ccz1b	Ctbp1	Prim1	Tedc2	Mtss1l	
Fstl1	Cd55	Ctdnep1	Prpf4	Terf2ip	Mustn1	
Fus	Cdc20	Ctdsp2	Ptma	Tex30	Mybl2	
G2e3	Cdc25b	Ctdspl	Ptpa	Tgfb2	Myl12b	
G3bp2	Cdc25c	Ctps1	Pttg1	Ticrr	Myo10	
Gaa	Cdc42ep2	Ctrb1	Qdpr	Timeless	Naa40	
Gadd45a	Cdc45	Ctsb	RGD1560010	Tipinl1	Nasp	
Galnt1	Cdc6	Ctsf	RT1-CE10	Tk1	Ncapd2	
Gas2l3	Cdc7	Cxcr3	RT1-N3	Tmem205	Ncapd3	
Gclm	Cdca2	Cyb5d1	Racgap1	Tmem97	Ncapg	
Gdf15	Cdca3	Cycs	Rad21	Tmpo	Ncapg2	
Gemin6	Cdca4	Cyp1b1	Rad51ap1	Tnfaip8l1	Ncaph	
Gen1	Cdca7	Cyp26b1	Rad51c	Tonsl	Ncaph2	
Gfra4	Cdca8	Dag1	Rad54l	Top2a	Ndc1	
Ghitm	Cdhr1	Dbr1	Ran	Topbp1	Ndc80	
Ginm1	Cdipt	Dclre1b	Rasl11b	Tp53inp1	Necab3	
Gins1	Cdk1	Dctpp1	Rbbp7	Tpp1	Nicn1	
Gins3	Cdk2	Dcxr	Rdm1	Tpx2	Nnt	
Gins4	Cdk2ap2	Ddit4	Reck	Traip	Nol3	
Glb1l	Cdkn1a	Ddx11	Rell2	Trim47	Nop10	
Glipr1	Cdkn2c	Ddx20	Rfc2	Trim59	Nr4a1	
Gm23880	Cdt1	Ddx39a	Rfc3	Tspan17	Nrm	
Gm2a	Cebpb	Dek	Rfc4	Ttk	Nsmce4a	
Gmip	Cebpd	Depp1	Rfc5	Tuba1b	Nuf2	
Gmnn	Cenpa	Des	Rfwd3	Tubb5	Nup107	
Gnat2	Cenpe	Dgkz	Rnaseh2a	Tubgcp2	Nup188	
Gng10	Cenpf	Dhrs9	Rnd1	U2af1	Nup205	
Gng2	Cenph	Dhx57	Rpa2	Ube2c	Nup43	
Golga7	Cenpk	Dlgap5	Rpa3	Ube2s	Nup85	
Gpd1	Cenpl	Dlx5	Rrm1	Ube2t	Nusap1	
Gprin3	Cenpm	Dmpk	Rrm2	Ugdh	Nxt1	
Gpsm2	Cenpo	Dnajb1	Samd9	Uhrf1	Orc1	
Grina	Cenpt	Dnajb5	Sapcd2	Ung	Orc6	
Grk6	Cenpu	Dnajb9	Serpine1	Usp1	P2rx4	
Grwd1	Cenpw	Dnajc9	Sf3a3	Wdhd1	Pa2g4	
Gtse1	Cep170b	Dnase2	Sf3b3	Xrcc2	Palb2	
Guk1	Cep250	Dnmt1	Sft2d3	Yod1	Pask	
H2afx	Cep295	Dnpep	Sgo1	Zfp422	Paxip1	

H2afz	Cep41	Dok1	Sgo2	Zfp513	Pbk
H6pd	Cep44	Dpp7	Shcbp1	Zw10	Pclaf
Hacd1	Cep55	Dpp9	Siglec10	Zwilch	Pcna
Hapln3	Cep72	Dscc1	Siva1		Pdcd4
Hat1	Cep76	Dsel	Ska1		Pfas
Haus3	Cetn2	Dsn1	Ska3		Pgrmc1
Haus4	Cfap157	Dstn	Slbp		Phf1
Haus5	Cfl2	Dtd2	Slc16a4		Phf5a
Haus8	Chac1	Dtl	Slc20a2		Pif1
Hbegf	Chaf1a	Dtx3	Slc25a10		Pimreg
Hif1an	Chaf1b	Dut	Slc38a7		Pla2g15
Hirip3	Chd3	Dvl2	Slc46a1		Plagl1
Hist1h1d	Chek2	Dvl3	Slfn13		Plekhb2
Hist1h2bk	Chfr	Dync2li1	Smc2		Plk1
Hjurp	Chpf2	Dynlt3	Smc4		Plk2
Hmgb2	Chrac1	Dyrk1b	Snapin		Plk4
Hmgn2	Chtf18	E130309D02Rik	Snrpa		Pln
Hmgn3	Cilp	E2f2	Spag5		Pmf1
Hmmr	Cip2a	E2f8	Spc24		Pnpla6
Hnrnpd	Cisd1	Echdc1	Spc25		Pnrc1
Hnrnpm	Cit	Ecm1	Spdl1		Pold1
Hnrnpul1	Ckap2	Ect2	Srsf1		Pold2
Hnrnpul2	Ckap2l	Eef1a1	Srsf2		Pole
Hspa14	Ckap4	Eef2	Srsf4		Pole2
Hspe1	Ckap5	Efhd2	Srsf7		Pop4
Htra1	Cks1b	Ehd4	Ssrp1		Ppat
Hyls1	Cks2	Eid1	Stil		Ppidl1
Icam1	Clcn3	Eif4ebp1	Stip1		Ppm1f
Id2	Clcn7	Eif4ebp2	Stmn1		Ppm1g
Id3	Cldnd1	Elk3	Suv39h1l1		Ppp2r5d
Idh2	Clec2dl1	Elk4	Syngr2		Prc1
Ier3	Clic1	Emd	Tacc3		Prim1
Ier5	Clspn	Eme1	Tbc1d2		Prob1
Ifi27	Cltb	Eme2	Tceanc		Prpf31
Ifi30	Clu	Emg1	Tcf19		Psap
Igfbp6	Cmtm3	Emp1	Tedc1		Psmc3ip
Il17rc	Cnn3	Enpep	Tedc2		Ptgs2
Il1rl1	Cnot9	Enpp4	Tex30		Ptma
Ilf2	Cnppd1	Entpd4	Tfdp1		Ptpa
Ilvbl	Cntf	Eps8l2	Tfpi		Pttg1
Incenp	Col11a1	Ercc6l	Thoc7		Pura
Ing1	Col4a1	Ereg	Ticrr		RF00085
Inhbb	Col4a2	Ergic3	Timeless		RF00594
Ints7	Col4a5	Erlec1	Tinagl1		RGD1305350
Irf7	Col5a1	Erlin1	Tiparp		RGD1308117
Irgc	Col5a2	Ermard	Tipinl1		RGD1560010
Irgm	Col8a1	Espl1	Tk1		RGD1562690
Isg15	Cops8	Esrra	Tmbim1		RT1-CE10
Itga7	Cox6a2	Etfrf1	Tmem138		RT1-N3
Ivns1abp	Cpe	Etv5	Tmem150a		Rab15
Jpt2	Cplane2	Exo1	Tmem230		Racgap1
Kcnj2	Cpsf2	Exosc2	Tmem80		Rad1
Kdelr2	Cpsf3	Exosc8	Tmem97		Rad21
Kdm5b	Creb3	Ezh2	Tmpo		Rad51ap1
Kif11	Creb3l1	F2r	Tnfaip8l1		Rad54l
Kif14	Crip2	F2rl1	Tob1		Rad9a
Kif15	Crtapl1	Fabp5	Toe1		Rangap1
Kif18b	Cryab	Fadd	Tonsl		Rasl11b
Kif20a	Cryba4	Fam107b	Top2a		Rassf1
Kif20b	Cse1l	Fam111a	Topbp1		Rbbp7
Kif22	Csnk1e	Fam136a	Tp53inp1		Rcc1
Kif23	Cspg4	Fam32a	Tpm1		Reck
Kif2c	Csrnp1	Fam83d	Tpx2		Recql4
Kif4a	Cst3	Fam84a	Traip		Reep3
Kifc1	Cstf1	Fam98c	Trappc6a		Reep4
Klhdc4	Ctdsp2	Fancb	Trim25		Rfc2
Klhl24	Ctsa	Fancd2	Trim47		Rfc3
Knl1	Ctsb	Fanci	Trim59		Rfc5
Knstrn	Ctsd	Fau	Trip13		Rnasek
Kntc1	Cxcl1	Fbl	Tsc22d1		Rnf10
Kprp	Cxxc5	Fbxo5	Tshz1		Rnf166
LOC100294508	Cyb561d1	Fbxw9	Tspan17		Rnf26
LOC100359539	Cyb5b	Fcgrt	Ttk		Rogdi

LOC100359600	Cycs	Fen1	Tuba1b		Rpa2	
LOC100362999	Cyp1b1	Fignl1	Tubb5		Rpa3	
LOC100363502	Dapk3	Fkbp1a	Tube1		Rpl22l1	
LOC100365363	Dazap2	Fkbp4	Tubgcp2		Rps3a	
LOC100909474	Dbi	Fkbp7	Ube2c		Rrm1	
LOC100911252	Dbn1	Fktn	Ube2s		Rrm2	
LOC100911361	Dbndd2	Flot2	Ube2t		Samd9	
LOC102546716	Dbnl	Fos	Ubr7		Sapcd2	
LOC102547056	Dcaf15	Foxa2	Uhrf1		Selenoi	
LOC102553386	Dcbld2	Foxm1	Ung		Sephs1	
LOC102556092	Dclre1b	Foxo4	Usp1		Serinc4	
LOC301444	Dctpp1	Fpgs	Vegfa		Sesn2	
LOC361346	Ddit3	Fsd1	Vrk1		Sesn3	
LOC499331	Ddit4	Fubp1	Vwa1		Setsip	
LOC684762	Ddr1	Fuca1	Wdhd1		Sf3b3	
LOC691807	Ddx11	Fuca2	Xrcc2		Sgo1	
Lamb1	Ddx39a	Fut11	Yod1		Sgo2	
Lgals3	Ddx39b	Fut4	Zbtb4		Shcbp1	
Lgals3bp	Def8	Fzr1	Zcchc3		Sirt6	
Lig4	Dek	G2e3	Zfp513		Siva1	
Lin54	Dgat2	G3bp1	Zswim4		Ska1	
Lix1l	Dhfr	Gaa	Zw10		Ska3	
Lmf2	Dhrs4	Gabarapl1	Zwilch		Slbp	
Lmna	Diaph1	Gadd45a	Zwint		Slc19a2	
Lmnb1	Dlgap5	Gas2l3			Slc25a10	
Lmnb2	Dmrt2	Gas6			Slc25a5	
Lpar6	Dnaaf3	Gatad2a			Slc30a1	
Lrrc14	Dnajb9	Gba			Slc38a7	
Lrrc75a	Dnajc10	Gdf15			Slc46a1	
Lrrcc1	Dnajc9	Gdi1			Slc52a3	
Lrrn4cl	Dnase2	Gemin6			Slc9a3r1	
Lsm3	Dnlz	Gen1			Slfn13	
Lsm5	Dnmt1	Get4			Smc2	
Lsm8	Dpf2	Gfpt1			Smc4	
Lyrm2	Dpp7	Ggt1			Smim14	
Mad2l1	Dpysl2	Ghdc			Snrpa	
Mad2l1bp	Dscc1	Gigyf1			Snrpa1	
Maff	Dsel	Gins1			Sod3	
Magt1	Dsn1	Gins2			Sowahc	
Mapk6	Dstn	Gins4			Spag5	
Mastl	Dtl	Giot1			Sparc	
Mbtps1	Dtymk	Gja1			Spc25	
Mcm10	Dusp1	Gltp			Sqstm1	
Mcm2	Dusp3	Glyctk			Srsf1	
Mcm3	Dusp8	Gmnn			Srsf2	
Mcm4	Dut	Gna11			Srsf7	
Mcm5	Dvl3	Gnai2			Stil	
Mcm6	Dync1li2	Gnb1			Stip1	
Mcm7	Dyrk2	Gnptg			Stmn1	
Mdc1	E2f2	Gpat4			Suv39h1l1	
Mdm1	E2f4	Gpd1l			Synj1	
Mdm2	E2f5	Gpr108			Tacc3	
Melk	E2f8	Gpr150			Tagln2	
Metrn	Eci1	Gpsm2			Tap1	
Mettl14	Ect2	Gpx1			Tceanc	
Mgme1	Edf1	Gpx8			Tcf19	
Micall2	Edn1	Grina			Tedc1	
Mid1ip1	Eef1aknmt	Grk2			Tedc2	
Mir125b1	Eef1g	Gtse1			Tent4a	
Mir145	Eef2	H2afz			Tent5a	
Mir21	Egln2	Haghl			Tex30	
Mir22	Egr1	Hapln3			Tfrc	
Mir27a	Ehbp1l1	Hapln4			Thoc3	
Mir339	Ehd3	Haspin			Thyn1	
Mirlet7i	Ehd4	Hat1			Ticrr	
Mis18a	Eif2a	Haus4			Timeless	
Mis18bp1	Eif3d	Haus8			Tipinl1	
Misp3	Eif4a2	Hbp1			Tk1	
Mki67	Eif4ebp1	Hcfc1			Tmem109	
Mme	Eif4ebp2	Hdgf			Tmem160	
Mms22l	Elk3	Hes6			Tmem167b	
Mrpl14	Eloc	Hilpda			Tmem185b	
Mrpl18	Elovl1	Hint2			Tmem202	

Mrpl51	Emc8	Hirip3			Tmem97	
Mrps25	Emd	Hist1h1c			Tmpo	
Mrps26	Eme1	Hjurp			Tnfaip8l1	
Msh6	Eme2	Hk2			Tonsl	
Msln	Emg1	Hmmr			Top2a	
Mtmr4	Emilin1	Hmox1			Topbp1	
Mvb12a	Emp3	Hnrnpa1			Tp53inp1	
Mybl2	Eno3	Hnrnpd			Tpx2	
Myh10	Enpp1	Hnrnpf			Traip	
NEWGENE_619861	Ercc6l	Hnrnpul1			Trim47	
Nabp1	Eri1	Hnrnpul2			Trim59	
Nans	Eri2	Hoxb5			Troap	
Nap1l1	Esco2	Hoxb7			Tspan17	
Nasp	Espl1	Hoxd9			Ttk	
Nbl1	Esrra	Hpdl			Tuba1b	
Ncapd2	Etv3	Hs1bp3			Tubb4b	
Ncapd3	Etv5	Hsdl1			Tubb5	
Ncapg	Evi2a	Hsf1			Tubb6	
Ncapg2	Exd2	Hsp90ab1			Tubgcp2	
Ncaph	Exo1	Hsp90b1			Tvp23b	
Ncaph2	Exosc2	Hspa14			Ube2c	
Ncl	Exosc8	Hspa1l			Ube2q1	
Ndc80	Ezh2	Hspa5			Ube2s	
Nde1	F2rl1	Hyls1			Ube2t	
Ndrg1	F3	Hyou1			Uhrf1	
Necab3	Faap24	Iah1			Ung	
Necap1	Fadd	Icam5			Usp1	
Nemp1	Fam102a	Idh2			Vamp2	
Neurod6	Fam111a	Idh3a			Wdhd1	
Nfe2l2	Fam131b	Ids			Wdr5	
Nfya	Fam214b	Idua			Wee1	
Nhlrc3	Fam3a	Ifi27			Xpo1	
Nit1	Fam71f1	Ifitm6			Xrcc2	
Nlrp3	Fam72a	Ifrd2			Ydjc	
Nmral1	Fam83d	Il18bp			Yod1	
Nono	Fam83h	Impdh2			Ypel5	
Npm3	Fam84a	Incenp			Zbtb12	
Nrm	Fam8a1	Ing1			Zfp219	
Nsl1	Fanca	Ing4			Zfp513	
Nsmce4a	Fancb	Ip6k2			Zfp688	
Nt5c3b	Fancd2	Iqgap3			Zfr2	
Nucks1	Fancg	Irak1			Zw10	
Nudt18	Fanci	Isy1			Zwilch	
Nuf2	Fbl	Itga5				
Nup107	Fbln5	Itgb1bp1				
Nup188	Fbxo2	Itm2b				
Nup35	Fbxo5	Itm2c				
Nup85	Fen1	Josd1				
Nusap1	Fermt2	Jpt2				
Nxt1	Fgfbp3	Kcnj2				
Oasl2	Fh	Kctd11				
Oip5	Fhod1	Kif11				
Orc1	Fignl1	Kif18b				
Orc6	Filip1l	Kif20a				
Ormdl2	Fkbp2	Kif20b				
Ostc	Fkbp3	Kif22				
P2rx4	Fkbp9	Kif23				
PVR	Fktn	Kif2c				
Pa2g4	Flrt3	Kif4a				
Pagr1	Fmnl3	Kifc1				
Paip2b	Fosl2	Kifc2				
Pank3	Foxa1	Klc4				
Parp1	Foxm1	Klhl23				
Parp2	Ftl1	Klhl24				
Parp9	Fuca1	Klhl31				
Pask	Fundc2	Kmt2b				
Paxip1	Fut4	Knstrn				
Pbk	Fv1	Kntc1				
Pcdh1	G2e3	Kpna2				
Pcdhga7	G3bp1	Krt79				
Pcif1	G3bp2	LOC100174910				
Pclaf	Gaa	LOC100359539				
Pcna	Gabbr1	LOC100359583				

Pdp2	Gadd45a	LOC100359600				
Pfas	Gadd45b	LOC100361025				
Phf19	Gadd45g	LOC100361265				
Phf5a	Gas2l3	LOC100364062				
Pif1	Gas6	LOC100365839				
Pigc	Gask1b	LOC100909474				
Pigm	Gata3	LOC100912483				
Pim1	Gchfr	LOC102546716				
Pimreg	Gdf11	LOC102549726				
Pitpnb	Gdf15	LOC102553785				
Pkm	Gemin6	LOC102555453				
Plekha1	Gen1	LOC103691238				
Plekhb2	Gfpt1	LOC108351584				
Plk1	Ghitm	LOC108351936				
Plk2	Gigyf1	LOC308990				
Plk3	Ginm1	LOC314140				
Plk4	Gins1	LOC361346				
Pmf1	Gins2	LOC680491				
Pmvk	Gins3	LOC690276				
Pnma1	Gins4	Lamb1				
Pnn	Gipc1	Lamp2				
Pnpo	Gjb4	Lancl1				
Pold2	Glb1l	Lasp1				
Pold3	Gltp	Lats1				
Pole	Gmnn	Lbh				
Pole2	Gnb2	Ldha				
Pole3	Gnptg	Ldlr				
Pole4	Golim4	Lgals3bp				
Polr2c	Golt1b	Lgmn				
Ppm1g	Got1	Lig3				
Ppp1ca	Gpank1	Lig4				
Ppp1r3c	Gpi	Limd2				
Ppp2r5d	Gpn1	Lin54				
Ppt2	Gpr108	Lin7c				
Pradc1	Gprasp2	Lmf2				
Prc1	Gpsm2	Lmnb1				
Prdm8	Grina	Lmnb2				
Prim1	Grwd1	Lonrf2				
Prpf4	Gstk1	Lox				
Prpf8	Gtf3c4	Loxl2				
Prr11	Gtse1	Loxl3				
Prss23	Gucd1	Lpcat2				
Psma1	Gypc	Lpin3				
Psmd3	Gys1	Lrpap1				
Ptges3	H2afz	Lrrc59				
Ptma	H3f3b	Lrrc73				
Ptpa	Hand2	Lrrc75b				
Ptprn	Hapln3	Lsm2				
Ptprv	Haspin	Lsm3				
Pttg1	Hat1	Lsm5				
Pycr2	Haus3	Lsm8				
Pygo2	Haus4	Lta4h				
Qdpr	Haus5	Ltbp2				
RF00088	Haus7	Lypla1				
RF00282	Hbp1	Lypla2				
RF00302	Hcfc1	Lysmd1				
RF00342	Hdac1	Lztfl1				
RF00402	Hdac1l	Mad2l1				
RF00409	Hddc3	Mad2l2				
RF00575	Herc3	Maf				
RF00586	Hilpda	Manea				
RGD1308134	Hipk3	Map3k6				
RGD1560010	Hirip3	Mapk6				
RGD1562378	Hist1h1c	Mapre3				
RT1-A2	Hist1h1d	Mblac1				
RT1-CE10	Hist1h2bk	Mcm10				
RT1-N3	Hjurp	Mcm2				
Rabggtb	Hmgb2	Mcm3				
Rac1	Hmgn2	Mcm4				
Racgap1	Hmmr	Mcm5				
Rad21	Hmox1	Mcm6				
Rad51ap1	Hnrnpul1	Mcm7				
Rad51c	Homer3	Mdc1				

Rad54l	Hoxa1	Mdm2					
Ran	Hoxb6	Me2					
Ranbp1	Hoxc6	Med1					
Rap1a	Hsdl1	Med21					
Rarg	Hsp90ab1	Med25					
Rasl11b	Hsp90b1	Med6					
Rasl2-9	Hspa13	Mettl23					
Rbbp7	Hspa14	Mex3c					
Rbl1	Hspa2	Mfsd1					
Rbm15b	Hspa5	Mfsd11					
Rbm3	Htr2b	Mief1					
Rdm1	Hyls1	Mief2					
Reck	Hyou1	Mir132					
Rell2	Iars	Mir1956					
Retreg2	Icam1	Mir23a					
Rfc2	Idh2	Mir27a					
Rfc3	Ier5	Mir3064					
Rfc4	Ifi27l2b	Mir34a					
Rfc5	Ifitm1	Mir3564					
Rfwd3	Ifitm2	Mir3568					
Rif1	Ifitm3	Mir374b					
Rnaseh1	Ifngr2	Mir6318					
Rnaseh2a	Ifrd1	Mir6334					
Rnaseh2c	Ifrd2	Mirlet7i					
Rnd1	Igsf3	Mis18a					
Rnf181	Il17rc	Mk1					
Rpa1	Il17re	Mki67					
Rpa2	Il6st	Mknk2					
Rpa3	Ilf2	Mkrn3					
Rpia	Impact	Mlycd					
Rpp21	Inafm1	Mme					
Rps4y2	Incenp	Mmgt1					
Rps6kb1	Ing1	Mmp13					
Rras	Ipo4	Mob3a					
Rrm1	Iqgap3	Morn2					
Rrm2	Irf7	Mpg					
Rtl8a	Irf9	Mprip					
Rwdd1	Itga11	Mpv17l2					
Samd9	Itga3	Mpzl3					
Sapcd2	Itga7	Mrpl45					
Sc5d	Itgb5	Mrpl57					
Scd	Itm2b	Msh2					
Scd2	Itm2c	Msh6					
Sdc3	Itpripl1	Msn					
Sdc4	Jak2	Mtch1					
Sema3f	Jtb	Mtcp1					
Sema4b	Katnb1	Mthfr					
Serpinb9	Kazald1	Mtss1l					
Serpine1	Kctd21	Mustn1					
Set	Kdm6b	Mxd1					
Sf1	Kif11	Mybl2					
Sf3a2	Kif14	Myl12b					
Sf3a3	Kif15	Myo10					
Sf3b2	Kif18a	Myo1e					
Sf3b3	Kif18b	Myo5a					
Sft2d3	Kif1c	Naa10					
Sfxn1	Kif20a	Naa40					
Sgms2	Kif20b	Nacc1					
Sgo1	Kif22	Nap1l4					
Sgo2	Kif23	Nasp					
Shcbp1	Kif2c	Ncapd2					
Shox2	Kif4a	Ncapd3					
Shroom1	Kif5b	Ncapg					
Siglec10	Kifc1	Ncapg2					
Siva1	Klc2	Ncaph					
Ska1	Klf2	Ncaph2					
Ska3	Klf4	Ndc1					
Skil	Klhdc8a	Ndc80					
Slbp	Klhl23	Ndrg4					
Slc16a1	Kmt5a	Ndufc1					
Slc16a4	Knl1	Necab3					
Slc20a2	Knstrn	Net1					
Slc25a10	Kntc1	Neu1					

Slc25a16	Kpna2	Nfat5
Slc35f5	Kpna6	Nicn1
Slc38a7	Krt79	Nit1
Slc46a1	Krt8	Nlrp3
Slfn13	Ktn1	Nme3
Sltm	LOC100294508	Nnt
Slurp1	LOC100359539	Nol3
Smad4	LOC100359583	Nolc1
Smc1a	LOC100359600	Nop10
Smc2	LOC100360117	Nop56
Smc3	LOC100362333	Npm3
Smc4	LOC100362400	Nr4a1
Smc6	LOC100362830	Nrbp1
Snapin	LOC100363469	Nrbp2
Snrnp25	LOC100365363	Nrm
Snrpa	LOC100365839	Nrp1
Snrpd1	LOC100909474	Nsd2
Snrpe	LOC100910554	Nsmce4a
Sox9	LOC100910717	Nuf2
Spag5	LOC100911361	Nup107
Spc24	LOC102546716	Nup153
Spc25	LOC102549726	Nup188
Spdl1	LOC102551095	Nup205
Speg	LOC102553785	Nup43
Spns1	LOC102555453	Nup62
Srsf1	LOC103690354	Nup85
Srsf2	LOC103691238	Nusap1
Srsf3	LOC108351584	Nxt1
Srsf4	LOC108351936	Nxt2
Srsf7	LOC290595	Odf2
Ssr1	LOC303566	Ogg1
Ssrp1	LOC308990	Orc1
Stard7	LOC361346	Orc6
Stil	LOC497940	Ormdl2
Stip1	LOC689039	P2rx4
Stmn1	LOC691807	P2ry4
Stom	Lacc1	P3h3
Stub1	Lama5	P4ha3
Suv39h1l1	Lamb2	P4hb
Syne3	Lamp2	Pa2g4
Syngr2	Lamtor5	Pak1ip1
Tacc3	Las1l	Pak4
Tapbpl	Lats2	Palb2
Tbc1d2	Lgals1	Parp10
Tceanc	Lgmn	Parp2
Tcf19	Lig4	Pask
Tedc1	Lin54	Paxip1
Tedc2	Lipa	Pbk
Terf2ip	Lman1	Pcbp2
Tesk1	Lmnb1	Pcdh18
Tex30	Lmnb2	Pcdhb14
Tfdp1	Lonrf2	Pclaf
Tfpi	Lpar6	Pcmtd2
Tgfb2	Lrfn4	Pcna
Thoc7	Lrig1	Pdcd4
Ticrr	Lrr1	Pdk4
Timeless	Lrrc40	Pdlim1
Tinagl1	Lrrc45	Pdlim2
Tiparp	Lrrc73	Pfas
Tipinl1	Lrrcc1	Pgk1
Tk1	Lsm2	Pgpep1
Tmbim1	Lsm3	Pgrmc1
Tmed5	Lsm5	Phf1
Tmem101	Lsm7	Phf5a
Tmem138	Lsm8	Phgdh
Tmem150a	Lta4h	Pias4
Tmem175	Lyar	Pif1
Tmem177	Lypla1	Pigq
Tmem205	Mad2l1	Pik3ip1
Tmem230	Maf	Pimreg
Tmem80	Mafg	Pink1
Tmem81	Mafk	Pkp1
Tmem97	Magoh	Pla2g15

Tmpo	Manf	Plagl1				
Tmtc3	Map1a	Plat				
Tnfaip8l1	Map1b	Pld3				
Tnfsf12	Map2k2	Plekhb2				
Tob1	Mapk1	Plk1				
Toe1	Mapre3	Plk2				
Tonsl	Marcks	Plk4				
Top2a	Mastl	Pln				
Topbp1	Mbd4	Plod2				
Tp53inp1	Mcm10	Plp2				
Tpm1	Mcm2	Pmf1				
Tpp1	Mcm3	Pmm2				
Tpx2	Mcm4	Pmp22				
Tra2b	Mcm5	Pnpla6				
Traip	Mcm6	Pnpo				
Trappc6a	Mcm7	Pnrc1				
Trim25	Mcrip1	Podnl1				
Trim34	Mdc1	Pold1				
Trim47	Mdm2	Pold2				
Trim59	Me1	Pold4				
Trip13	Med18	Pole				
Tsc22d1	Med21	Pole2				
Tshz1	Melk	Polg				
Tspan17	Mfrp	Pomgnt2				
Ttk	Mfsd10	Pop4				
Tuba1b	Mgat1	Ppat				
Tubb5	Mgme1	Ppidl1				
Tube1	Mib2	Ppm1f				
Tubgcp2	Mical1	Ppm1g				
Tusc2	Midn	Ppp1r9b				
Txlna	Mir125b1	Ppp2r5d				
Tyms	Mir23a	Prc1				
U2af1	Mir3064	Prdx5				
Uba7	Mir3564	Prim1				
Ubac1	Mir3568	Prkar2a				
Ubald2	Mir374b	Prob1				
Ube2c	Mir615	Prpf31				
Ube2s	Mir6334	Prss23				
Ube2t	Mis12	Psap				
Ubr7	Mis18a	Psenen				
Ubtf	Mis18bp1	Psmb9				
Ufd1	Mk1	Psmc3ip				
Ugdh	Mki67	Psmd5				
Uhrf1	Mkrn3	Ptdss1				
Ung	Mllt11	Ptges3				
Usp1	Mmd	Ptges3l1				
Vamp5	Mms22l	Ptgs2				
Vdac2	Mob1a	Ptma				
Vdac3	Mre11a	Ptpa				
Vegfa	Mrfap1	Ptprv				
Vps4a	Mrpl14	Pttg1				
Vrk1	Mrpl18	Pura				
Vwa1	Mrpl28	Pwp2				
Wdhd1	Mrpl35	Pxdn				
Wnk1	Mrps5	RF00085				
Xkr5	Msh2	RF00187				
Xrcc2	Msh6	RF00282				
Ykt6	Msln	RF00404				
Yod1	Mt1m	RF00594				
Ywhah	Mthfr	RF01182				
Zbtb4	Mtss1l	RGD1305350				
Zcchc24	Mustn1	RGD1305938				
Zcchc3	Mutyh	RGD1306502				
Zfand2b	Mxd4	RGD1308117				
Zfp207	Mybl2	RGD1311899				
Zfp367	Myh10	RGD1311946				
Zfp395	Myl12b	RGD1560010				
Zfp422	Myo10	RGD1561149				
Zfp513	Mzt2b	RGD1562136				
Zswim4	N4bp1	RGD1562378				
Zw10	Naa25	RGD1562690				
Zwilch	Naa40	RGD1563620				
Zwint	Nabp1	RGD1564613				

	Nacc2	RGD1564804				
	Nagk	RGD1566099				
	Nans	RT1-CE10				
	Nasp	RT1-M3-1				
	Nbas	RT1-N2				
	Ncam1	RT1-N3				
	Ncapd2	Rab15				
	Ncapd3	Rab29				
	Ncapg	Rac1				
	Ncapg2	Racgap1				
	Ncaph	Rad1				
	Ncaph2	Rad21				
	Nckap5l	Rad51ap1				
	Ndc1	Rad54l				
	Ndc80	Rad9a				
	Ndel1	Raly				
	Ndufb9	Ranbp1				
	Necab3	Ranbp3				
	Necap1	Rangap1				
	Nedd1	Rars				
	Nelfcd	Rasl11b				
	Nemp1	Rassf1				
	Nexmif	Rbbp7				
	Nfe2l1	Rbm17				
	Nfu1	Rcc1				
	Ngrn	Reck				
	Nicn1	Recql4				
	Nisch	Reep3				
	Nkd2	Reep4				
	Nkiras2	Renbp				
	Nnt	Retreg3				
	Nol3	Rexo1				
	Nono	Rfc2				
	Nop10	Rfc3				
	Npepps	Rfc5				
	Nptxr	Rgl2				
	Nr1i3	Rhpn1				
	Nr4a1	Rilpl2				
	Nr4a2	Ripk3				
	Nras	Rnasek				
	Nrm	Rnd2				
	Nrn1	Rnf10				
	Nrp2	Rnf166				
	Nsl1	Rnf26				
	Nsmce4a	Rnft1				
	Nucb1	Rock2				
	Nucks1	Rogdi				
	Nudt1	Rpa2				
	Nuf2	Rpa3				
	Numbl	Rpl22l1				
	Nup107	Rplp0				
	Nup133	Rpp21				
	Nup155	Rps3a				
	Nup160	Rpusd3				
	Nup188	Rrm1				
	Nup205	Rrm2				
	Nup35	Rrp15				
	Nup37	S100a10				
	Nup43	S100a16				
	Nup85	Samd1				
	Nup93	Samd9				
	Nusap1	Sapcd2				
	Nxt1	Saraf				
	Oard1	Scarf2				
	Oaz1	Scoc				
	Ogfrl1	Sdc1				
	Oip5	Sec13				
	Olfml3	Selenoi				
	Orc1	Selenos				
	Orc6	Sema7a				
	Ormdl3	Sephs1				
	Osbpl5	11-Sep				
	Osmr	Serinc4				

	Osr1	Serpina3n					
	Otx1	Serpinh1					
	Oxct1	Sesn2					
	Oxnad1	Sesn3					
	P2rx4	Set					
	P4ha2	Setd6					
	Pa2g4	Setsip					
	Pagr1	Sf3b3					
	Palb2	Sfpq					
	Pars2	Sfrp4					
	Pask	Sfxn3					
	Paxip1	Sgo1					
	Pbk	Sgo2					
	Pcdhgb8	Shcbp1					
	Pcdhgc3	Sirt6					
	Pcgf2	Siva1					
	Pclaf	Ska1					
	Pcna	Ska3					
	Pcyox1	Slbp					
	Pdcd2	Slc10a5					
	Pdcd4	Slc16a1					
	Pdia4	Slc19a2					
	Pelp1	Slc1a4					
	Pfas	Slc22a17					
	Pfdn4	Slc25a10					
	Pgrmc1	Slc25a2					
	Pgrmc2	Slc25a39					
	Phf1	Slc25a5					
	Phf19	Slc27a1					
	Phf5a	Slc30a1					
	Phlda3	Slc30a4					
	Phldb3	Slc38a7					
	Phyh	Slc39a1					
	Piezo1	Slc39a5					
	Pif1	Slc39a9					
	Pih1d1	Slc43a1					
	Pik3cd	Slc46a1					
	Pimreg	Slc52a3					
	Pkmyt1	Slc8b1					
	Pla2g15	Slc9a3r1					
	Plagl1	Slfn13					
	Plat	Slfnl1					
	Plcd4	Smarcd1					
	Plekha3	Smc2					
	Plekhb2	Smc4					
	Plekhg2	Smim14					
	Plekho1	Snn					
	Plin3	Snrpa					
	Plk1	Snrpa1					
	Plk2	Snrpf					
	Plk3	Socs2					
	Plk4	Sod2					
	Pln	Sod3					
	Plpp6	Sowahc					
	Plscr3	Sp110					
	Pmf1	Spaca6					
	Pnpla6	Spag5					
	Pnrc1	Sparc					
	Pola2	Spc25					
	Pold1	Spns1					
	Pold2	Spon2					
	Pole	Spp1					
	Pole2	Spry4					
	Pole3	Sqstm1					
	Polm	Srsf1					
	Polr2c	Srsf2					
	Polr2e	Srsf3					
	Polr2k	Srsf7					
	Pop4	Ssx2ip					
	Ppat	St3gal4					
	Ppidl1	St6gal1					
	Ppie	Steap2					
	Ppif	Stil					

	Ppih	Stip1				
	Ppm1f	Stmn1				
	Ppm1g	Sumo4				
	Ppp1ca	Sun1				
	Ppp1r13l	Supt4h1				
	Ppp1r15a	Suv39h1l1				
	Ppp2r5d	Sv2a				
	Ppp5c	Synj1				
	Pqlc2	Syt11				
	Pradc1	Tacc3				
	Prc1	Tagln2				
	Prdx3	Tap1				
	Prdx6	Tbc1d17				
	Prim1	Tceanc				
	Prkab2	Tcf19				
	Prob1	Tcn2				
	Prpf19	Tcof1				
	Prpf31	Tcp11l2				
	Prpf4	Tcta				
	Prps2	Tedc1				
	Prr15	Tedc2				
	Psap	Tent4a				
	Psip1	Tent5a				
	Psmc3ip	Terf2ip				
	Psmd13	Tes				
	Ptger1	Tex30				
	Ptgfrn	Tfrc				
	Ptgs1	Tgfb1i1				
	Ptgs2	Tgfb2				
	Ptma	Tgfb3				
	Ptpa	Tgoln2				
	Ptpn23	Thap12				
	Ptprf	Them6				
	Ptrh1	Thnsl1				
	Pttg1	Thoc3				
	Pura	Thtpa				
	Purg	Thyn1				
	Pus3	Ticrr				
	Pusl1	Timeless				
	Pycard	Timm8a1				
	Qdpr	Timp1				
	RF00085	Tipinl1				
	RF00264	Tk1				
	RF00402	Tle2				
	RF00416	Tmed3				
	RF00594	Tmem109				
	RF01161	Tmem160				
	RGD1305350	Tmem167b				
	RGD1306227	Tmem179b				
	RGD1306941	Tmem185b				
	RGD1308117	Tmem202				
	RGD1309748	Tmem203				
	RGD1310553	Tmem205				
	RGD1559896	Tmem208				
	RGD1560010	Tmem214				
	RGD1562136	Tmem30a				
	RGD1562690	Tmem43				
	RGD1563861	Tmem53				
	RGD1564855	Tmem86a				
	RT1-CE10	Tmem97				
	RT1-M3-1	Tmpo				
	RT1-N3	Tnfaip8l1				
	Rab11fip1	Tnfrsf26				
	Rab11fip5	Tns2				
	Rab15	Tomm6				
	Rab31	Tonsl				
	Rab32	Top2a				
	Rab3d	Topbp1				
	Rab8a	Tp53inp1				
	Rabl2	Tpp1				
	Racgap1	Tpx2				
	Rack1	Traip				
	Rad1	Trappc2				

	Rad21	Trim47				
	Rad51	Trim59				
	Rad51ap1	Troap				
	Rad51ap2	Tsn				
	Rad51c	Tspan13				
	Rad54l	Tspan17				
	Rad9a	Tspan3				
	Ramac	Ttc9c				
	Ran	Ttk				
	Rangap1	Ttpal				
	Rap2b	Tuba1b				
	Rasl11b	Tuba4a				
	Rassf1	Tubb4b				
	Rassf7	Tubb5				
	Rassf8	Tubb6				
	Rbbp7	Tubgcp2				
	Rbm18	Tufm				
	Rcc1	Tulp3				
	Rdm1	Tvp23b				
	Reck	Txnrd1				
	Recql4	U2af1				
	Reep3	Ube2c				
	Reep4	Ube2g2				
	Rell2	Ube2q1				
	Rexo4	Ube2s				
	Rfc2	Ube2t				
	Rfc3	Ubiad1				
	Rfc4	Ubqln4				
	Rfc5	Ucp2				
	Rfwd3	Ugdh				
	Riok3	Uhrf1				
	Rnase1l2	Ung				
	Rnaseh2a	Usp1				
	Rnasek	Vamp2				
	Rnd1	Vars				
	Rnd3	Vgll4				
	Rnf10	Vmp1				
	Rnf14	Wdhd1				
	Rnf166	Wdr1				
	Rnf167	Wdr45				
	Rnf185	Wdr5				
	Rnf26	Wdtc1				
	Rnf44	Wee1				
	Rnps1	Wfdc18				
	Rogdi	Wipf1				
	Rpa2	Wnt4				
	Rpa3	Wnt5b				
	Rpe	Wsb2				
	Rpl11	Xpo1				
	Rpl15	Xrcc2				
	Rpl18	Ydjc				
	Rpl19	Yipf2				
	Rpl22l1	Yod1				
	Rpl23	Ypel3				
	Rpl24	Ypel5				
	Rpl26	Ywhaz				
	Rpl27	Zbtb12				
	Rpl29	Zbtb18				
	Rpl3	Zbtb2				
	Rpl31	Zdhhc16				
	Rpl34	Zdhhc7				
	Rpl35	Zfp219				
	Rpl37a	Zfp275				
	Rpl39	Zfp36l1				
	Rpl41	Zfp422				
	Rpl5	Zfp496				
	Rpl6	Zfp513				
	Rpl7	Zfp688				
	Rpl7l1	Zfr2				
	Rps15	Zw10				
	Rps17	Zwilch				
	Rps18l1					
	Rps27a					

	Rps28<br>Rps3<br>Rps3a<br>Rps4x<br>Rps7<br>Rps8<br>Rrm1<br>Rrm2<br>Rtn4<br>Rusc2<br>Ruvbl1<br>Rwdd4<br>S100a1<br>Sae1<br>Samd9<br>Sapcd2<br>Sart3<br>Selenoi<br>Sema3b<br>Sema3c<br>Sema5a<br>Sema7a<br>Sephs1<br>Sephs2<br>Serinc3<br>Serinc4<br>Serpinb6a<br>Serpine1<br>Sesn2<br>Sesn3<br>Setd7<br>Setsip<br>Setx<br>Sf3a3<br>Sf3b3<br>Sft2d3<br>Sgo1<br>Sgo2<br>Sgpp1<br>Sh3gl1<br>Shc1<br>Shcbp1<br>Shkbp1<br>Siglec10<br>Sirt6<br>Siva1<br>Ska1<br>Ska3<br>Slbp<br>Slc12a4<br>Slc16a4<br>Slc19a2<br>Slc20a2<br>Slc25a10<br>Slc25a11<br>Slc25a5<br>Slc29a1<br>Slc30a1<br>Slc35a4<br>Slc35b2<br>Slc35d2<br>Slc38a7<br>Slc39a3<br>Slc46a1<br>Slc4a2<br>Slc52a3<br>Slc5a6<br>Slc9a3r1<br>Slfn13<br>Slfn2<br>Smarca5<br>Smc2<br>Smc4					

	Smim14					
	Snapin					
	Snrpa					
	Snrpa1					
	Snrpb					
	Snx5					
	Sod3					
	Sorbs3					
	Sowahc					
	Spag5					
	Sparc					
	Spata2					
	Spc24					
	Spc25					
	Spcs2					
	Spdl1					
	Sphk1					
	Spp1					
	Sptan1					
	Sqor					
	Sqstm1					
	Srebf2					
	Srpra					
	Srrm1					
	Srsf1					
	Srsf2					
	Srsf4					
	Srsf7					
	Ssr3					
	Ssrp1					
	St6galnac1					
	St6galnac6					
	Stbd1					
	Steap3					
	Stil					
	Stip1					
	Stk40					
	Stmn1					
	Sumf1					
	Suox					
	Suv39h1l1					
	Suv39h2					
	Svbp					
	Syde1					
	Syngr2					
	Synj1					
	Synm					
	Sypl1					
	Tacc3					
	Taf13					
	Taf15					
	Tagln2					
	Tap1					
	Tbc1d2					
	Tceanc					
	Tcerg1					
	Tcf19					
	Tcp1					
	Tdg					
	Tead3					
	Tedc1					
	Tedc2					
	Tent4a					
	Tent5a					
	Tent5b					
	Tex261					
	Tex264					
	Tex30					
	Tfdp1					
	Tfpi					
	Tfrc					
	Tgfbr2					
	Tgoln2					

	Thbs2					
	Thoc3					
	Thoc7					
	Thy1					
	Thyn1					
	Ticrr					
	Tigar					
	Timeless					
	Timm21					
	Tinagl1					
	Tiparp					
	Tipinl1					
	Tk1					
	Tkt					
	Tlr3					
	Tm4sf1					
	Tmbim1					
	Tmed1					
	Tmed2					
	Tmem106c					
	Tmem107					
	Tmem109					
	Tmem119					
	Tmem127					
	Tmem129					
	Tmem138					
	Tmem139					
	Tmem14c					
	Tmem150a					
	Tmem160					
	Tmem167b					
	Tmem185b					
	Tmem202					
	Tmem230					
	Tmem267					
	Tmem63b					
	Tmem80					
	Tmem87b					
	Tmem8a					
	Tmem9					
	Tmem97					
	Tmpo					
	Tnfaip2					
	Tnfaip8l1					
	Tnfrsf11b					
	Tnfsf18					
	Tnip3					
	Tnrc18					
	Tob1					
	Toe1					
	Tomm40l					
	Tonsl					
	Top2a					
	Topbp1					
	Tor1b					
	Tor4a					
	Tp53inp1					
	Tp53inp2					
	Tpm1					
	Tpx2					
	Trabd					
	Traf4					
	Trafd1					
	Traip					
	Trappc6a					
	Trib2					
	Trim25					
	Trim41					
	Trim47					
	Trim59					
	Trim7					
	Trip10					
	Trip13					

	Trip6					
	Trmt10a					
	Trmt12					
	Troap					
	Tsc22d1					
	Tshz1					
	Tsn					
	Tspan17					
	Tspan7					
	Ttc30b					
	Ttk					
	Tuba1b					
	Tubb4b					
	Tubb5					
	Tubb6					
	Tube1					
	Tubg1					
	Tubgcp2					
	Tubgcp6					
	Tvp23b					
	Twsg1					
	Txnip					
	Tymp					
	Tyro3					
	Ube2c					
	Ube2q1					
	Ube2s					
	Ube2t					
	Ubqln2					
	Ubr7					
	Ubxn4					
	Uckl1					
	Uhrf1					
	Uhrf1bp1l					
	Unc119					
	Unc5b					
	Ung					
	Use1					
	Usp1					
	Vamp2					
	Vamp8					
	Vasp					
	Vat1					
	Vegfa					
	Vgll3					
	Vps11					
	Vps26b					
	Vps9d1					
	Vrk1					
	Vwa1					
	Wdfy1					
	Wdhd1					
	Wdr5					
	Wdr90					
	Wee1					
	Wls					
	Xpo1					
	Xrcc2					
	Ydjc					
	Yod1					
	Ypel5					
	Zbtb12					
	Zbtb4					
	Zbtb8os					
	Zc2hc1a					
	Zcchc3					
	Zdhhc6					
	Zfp219					
	Zfp322a					
	Zfp513					
	Zfp612					
	Zfp688					
	Zfp846					

	Zfr2<br>Znrd1<br>Zscan22<br>Zswim4<br>Zw10<br>Zwilch<br>Zwint<br>mrpl11					

TABLE S3B Common and distinct genes altered in KMM cells following knockdown of GRWD1, WDR5 and MLL2

GRWD1	WDR5	MLL2	GRWD1&WDR5	GRWD1&MLL2	WDR5&MLL2	GRWD1&WDR5&MLL2
AABR07000159.2	AABR07000222.1	AABR07000658.1	AABR07000658.1	AABR07000658.1	AABR07000658.1	AABR07000658.1
AABR07000658.1	AABR07000658.1	AABR07000747.1	AABR07011698.1	AABR07005838.1	AABR07001512.1	AABR07011698.1
AABR07005838.1	AABR07001433.1	AABR07001512.1	AABR07013288.4	AABR07011698.1	AABR07003344.1	AABR07013288.4
AABR07011697.1	AABR07001512.1	AABR07002564.1	AABR07026311.1	AABR07013288.4	AABR07006081.1	AABR07026311.1
AABR07011698.1	AABR07002711.1	AABR07003344.1	AABR07035539.1	AABR07026311.1	AABR07006111.1	AABR07044421.1
AABR07013288.4	AABR07003344.1	AABR07004269.4	AABR07044273.1	AABR07028352.1	AABR07010705.1	AC095390.1
AABR07013798.1	AABR07005031.1	AABR07004881.1	AABR07044421.1	AABR07044421.1	AABR07011698.1	AC112350.1
AABR07015941.2	AABR07006081.1	AABR07005838.1	AABR07053516.1	AC095390.1	AABR07012329.1	AY172581.24
AABR07018792.1	AABR07006111.1	AABR07006081.1	AC095390.1	AC112350.1	AABR07013288.4	Anp32e
AABR07026311.1	AABR07006269.1	AABR07006111.1	AC112350.1	AC116236.2	AABR07014756.1	Arhgap11a
AABR07026893.1	AABR07007000.1	AABR07006258.2	AC118419.1	AY172581.24	AABR07015180.1	Asf1b
AABR07028352.1	AABR07010705.1	AABR07008097.1	AY172581.24	AY172581.9	AABR07018244.2	Aspm
AABR07029863.1	AABR07011698.1	AABR07008420.1	Aen	Anln	AABR07024500.1	Atad2
AABR07029955.1	AABR07012100.2	AABR07009357.2	Anp32e	Anp32e	AABR07025301.1	Atp6v1d
AABR07030183.1	AABR07012329.1	AABR07010705.1	Arhgap11a	Arhgap11a	AABR07025787.1	Aurka
AABR07030494.1	AABR07013288.4	AABR07011697.1	Asf1b	Arl2bp	AABR07026311.1	Aurkb
AABR07030866.1	AABR07014550.1	AABR07011698.1	Aspm	Asf1b	AABR07027575.1	B2m
AABR07035539.1	AABR07014756.1	AABR07011977.1	Atad2	Aspm	AABR07027811.2	Birc5
AABR07038926.1	AABR07015180.1	AABR07011996.1	Atp6v1d	Atad2	AABR07027811.3	Bub1
AABR07039153.2	AABR07018244.2	AABR07012329.1	Aurka	Atp6v1d	AABR07029605.1	Bub1b
AABR07039210.2	AABR07018321.3	AABR07012583.2	Aurkb	Aurka	AABR07029613.1	Ccn2
AABR07039338.1	AABR07018331.1	AABR07013147.1	B2m	Aurkb	AABR07030200.1	Ccn4
AABR07041724.1	AABR07021759.1	AABR07013255.1	B4galt5	B2m	AABR07030861.1	Ccna2
AABR07042326.2	AABR07021988.1	AABR07013288.4	Birc5	Bhlhe41	AABR07030911.2	Ccnb1
AABR07043101.1	AABR07024500.1	AABR07014756.1	Bok	Birc5	AABR07031734.15	Ccnf
AABR07043167.1	AABR07024972.1	AABR07015040.1	Bub1	Bub1	AABR07032751.1	Cdc20
AABR07044273.1	AABR07025301.1	AABR07015180.1	Bub1b	Bub1b	AABR07033047.1	Cdca3
AABR07044421.1	AABR07025328.1	AABR07016572.1	Ccn2	Ccdc96	AABR07035541.2	Cdca8
AABR07044574.1	AABR07025787.1	AABR07017999.1	Ccn4	Ccn2	AABR07039210.1	Cdk1
AABR07045487.1	AABR07026311.1	AABR07018244.2	Ccna2	Ccn4	AABR07042875.1	Cdkn1a
AABR07049578.1	AABR07026805.2	AABR07019399.1	Ccnb1	Ccna2	AABR07043557.1	Cenpe
AABR07053516.1	AABR07027010.1	AABR07021022.1	Ccne2	Ccnb1	AABR07044366.1	Cenpf
AABR07060293.1	AABR07027575.1	AABR07024500.1	Ccnf	Ccnf	AABR07044375.1	Cenph
AABR07062136.1	AABR07027811.2	AABR07024972.1	Cdc20	Cdc20	AABR07044375.2	Ckap2l
AABR07062915.2	AABR07027811.3	AABR07025301.1	Cdca3	Cdc25b	AABR07044421.1	Cks1b
AABR07072761.1	AABR07028970.1	AABR07025787.1	Cdca8	Cdca3	AABR07044635.1	Dek
AC095390.1	AABR07029198.1	AABR07026002.1	Cdk1	Cdca8	AABR07049223.1	Dlgap5
AC109877.1	AABR07029605.1	AABR07026311.1	Cdkn1a	Cdk1	AABR07050283.2	Dnmt1
AC110690.1	AABR07029613.1	AABR07026361.2	Cenpe	Cdkn1a	AABR07053136.1	Dut
AC112350.1	AABR07029955.1	AABR07027575.1	Cenpf	Cenpe	AABR07053716.1	E2f8
AC114363.1	AABR07030019.1	AABR07027753.3	Cenph	Cenpf	AABR07055919.1	Ect2
AC116236.2	AABR07030156.2	AABR07027811.2	Cenpt	Cenph	AABR07058464.1	Eme1
AC118419.1	AABR07030200.1	AABR07027811.3	Cfl2	Ckap2l	AABR07059891.1	Fancd2
AC120066.1	AABR07030366.1	AABR07027910.1	Ckap2l	Cks1b	AABR07060291.1	Fanci
AC123095.1	AABR07030603.1	AABR07028352.1	Cks1b	Dek	AABR07060593.1	Fbxo5
AC127106.1	AABR07030603.2	AABR07029605.1	Cnpy4	Dlgap5	AABR07061614.1	Foxm1
AC128792.2	AABR07030861.1	AABR07029613.1	Cryab	Dnmt1	AABR07067526.1	Gaa
AC130741.1	AABR07030911.2	AABR07030200.1	Cxcl16	Dut	AABR07068127.1	Gadd45a
AC133400.1	AABR07031089.1	AABR07030265.1	Dek	E2f8	AABR07069282.1	H2afz
AC135310.2	AABR07031734.15	AABR07030462.1	Dlgap5	Ect2	AABR07069433.1	Haspin
AC141526.2	AABR07032751.1	AABR07030630.1	Dnmt1	Eme1	AABR07070810.1	Haus4
AY172581.10	AABR07032821.1	AABR07030861.1	Dtx3l	Espl1	AABR07071000.1	Hist1h1t
AY172581.18	AABR07033047.1	AABR07030911.2	Dtymk	Fancd2	AABR07072236.1	Hjurp
AY172581.2	AABR07033745.1	AABR07030914.1	Dut	Fanci	AABR07072559.2	Hmmr
AY172581.22	AABR07034315.1	AABR07031734.13	E2f8	Fbxo5	AC094126.2	Hspb2
AY172581.24	AABR07035074.1	AABR07031734.15	Ect2	Foxm1	AC095390.1	Hyls1
AY172581.9	AABR07035539.1	AABR07032751.1	Eme1	Gaa	AC099089.1	Idh2
Abhd5	AABR07035541.2	AABR07033020.1	Fam214b	Gadd45a	AC103090.1	Incenp
Actg2	AABR07035819.1	AABR07033047.1	Fancd2	H2afz	AC106663.1	Iqgap3
Actn1	AABR07036087.1	AABR07033162.1	Fanci	Haspin	AC112018.1	Kif11
Aen	AABR07036247.1	AABR07035317.1	Fbxo5	Haus4	AC112350.1	Kif20a
Aida	AABR07038873.1	AABR07035317.2	Foxm1	Hjurp	AC119015.4	Kif22
Ankrd1	AABR07038948.2	AABR07035339.1	G3bp2	Hmmr	AC119762.7	Kif2c
Anln	AABR07039037.1	AABR07035541.2	Gaa	Hoxb7	AC121413.2	Kifc1
Anp32e	AABR07039133.2	AABR07036016.1	Gadd45a	Hspb2	AC123425.1	Knstrn
Ap5b1	AABR07039210.1	AABR07036498.1	Gins3	Hyls1	AC129365.1	Kntc1
Arf5	AABR07040624.1	AABR07037356.1	Gpx8	Idh2	AC130232.1	Kpna2
Arhgap11a	AABR07040892.1	AABR07038895.2	H2afz	Ids	AC130391.1	LOC100359539
Arl2bp	AABR07042875.1	AABR07038939.1	Haspin	Ifi44	AC130391.5	LOC100359600
Arl6ip4	AABR07043557.1	AABR07039210.1	Haus4	Incenp	AC134224.1	Lamp1
Asf1b	AABR07043829.1	AABR07039210.2	Hjurp	Iqgap3	AC134224.2	Lmnb1

Aspm	AABR07044273.1	AABR07039303.4	Hmgb2	Kcnf1	AC134224.3	Lmnb2
Atad2	AABR07044366.1	AABR07039304.1	Hmmr	Kif11	AC142458.1	Lsm2
Atp6v1d	AABR07044375.1	AABR07039356.1	Hspb2	Kif20a	AY172581.20	Mad2l1
Aurka	AABR07044375.2	AABR07039483.1	Hyls1	Kif22	AY172581.24	Mcm10
Aurkb	AABR07044388.2	AABR07041778.1	Idh2	Kif23	Aaas	Mcm2
B2m	AABR07044421.1	AABR07042454.2	Incenp	Kif2c	Adamtsl5	Mcm3
B4galt5	AABR07044635.1	AABR07042611.1	Iqgap3	Kifc1	Adrb2	Mcm4
Bag2	AABR07044959.1	AABR07042875.1	Irf7	Knstrn	Aga	Mcm6
Bhlhe41	AABR07046628.1	AABR07043169.1	Itga7	Kntc1	Ahcy	Mdm2
Birc5	AABR07047011.1	AABR07043557.1	Kif11	Kpna2	Akt1	Mki67
Bloc1s3	AABR07047089.1	AABR07043844.1	Kif20a	LOC100359539	Aktip	Mybl2
Bok	AABR07047219.1	AABR07044001.1	Kif22	LOC100359600	Alyref	Nasp
Bub1	AABR07049223.1	AABR07044366.1	Kif2c	LOC100364062	Anapc15	Ncapd2
Bub1b	AABR07049353.1	AABR07044375.1	Kifc1	LOC102547056	Ankrd49	Ncapg
Cadm4	AABR07049695.2	AABR07044375.2	Klf2	Lamp1	Anp32e	Ncapg2
Car5b	AABR07049755.1	AABR07044420.1	Knstrn	Lmnb1	Apol9a	Ncaph
Carhsp1	AABR07050283.2	AABR07044421.1	Kntc1	Lmnb2	Arhgap11a	Ndc80
Casq2	AABR07051240.1	AABR07044593.1	Kpna2	Lsm2	Arhgdia	Nrm
Ccdc17	AABR07052430.1	AABR07044635.1	LOC100359539	Mad2l1	Arl4a	Nuf2
Ccdc47	AABR07053136.1	AABR07044959.1	LOC100359600	Mcm10	Asf1b	Nup85
Ccdc96	AABR07053152.1	AABR07049223.1	LOC290595	Mcm2	Aspm	Nusap1
Cchcr1	AABR07053166.1	AABR07049792.1	Lamp1	Mcm3	Atad2	Orc6
Ccn2	AABR07053516.1	AABR07050283.2	Lmnb1	Mcm4	Atad5	P2rx4
Ccn4	AABR07053716.1	AABR07051399.1	Lmnb2	Mcm6	Atg12	Pbk
Ccna2	AABR07055539.2	AABR07051450.1	Lsm2	Mdm2	Atp6v1d	Pclaf
Ccnb1	AABR07055919.1	AABR07053136.1	Mad2l1	Mki67	Aunip	Phlda3
Ccne2	AABR07055943.1	AABR07053669.1	Mcm10	Mybl2	Aurka	Pla2g15
Ccnf	AABR07057190.1	AABR07053707.1	Mcm2	Nasp	Aurkb	Plk1
Cd164	AABR07057237.1	AABR07053716.1	Mcm3	Ncapd2	B2m	Plk2
Cdc20	AABR07057250.1	AABR07053734.1	Mcm4	Ncapg	Bak1	Plk4
Cdc25b	AABR07057683.1	AABR07054266.1	Mcm6	Ncapg2	Bdh2	Pole
Cdc34	AABR07058464.1	AABR07054368.1	Mdm2	Ncaph	Birc5	Prc1
Cdca3	AABR07059891.1	AABR07055919.1	Mki67	Ndc80	Blcap	Prim1
Cdca8	AABR07060291.1	AABR07058287.1	Mybl2	Neurl2	Bora	Psat1
Cdk1	AABR07060593.1	AABR07058464.1	Nasp	Nrm	Borcs6	Ptma
Cdkn1a	AABR07061614.1	AABR07059198.1	Ncapd2	Nuf2	Brca1	Pttg1
Cdkn2c	AABR07062477.2	AABR07059891.1	Ncapg	Nup85	Btbd19	Racgap1
Cenpe	AABR07062599.1	AABR07060291.1	Ncapg2	Nusap1	Btg2	Rad21
Cenpf	AABR07063581.2	AABR07060593.1	Ncaph	Orc6	Bub1	Rfc5
Cenph	AABR07063829.2	AABR07061614.1	Ncaph2	P2rx4	Bub1b	Rhob
Cenpt	AABR07065438.1	AABR07062138.1	Ndc80	Pbk	Cavin2	Rogdi
Cfl2	AABR07067042.2	AABR07062138.2	Necab3	Pclaf	Cbx3	Rpa2
Cggbp1	AABR07067526.1	AABR07063082.1	Nrm	Phlda2	Ccn2	Rpa3
Chfr	AABR07068127.1	AABR07065265.1	Nuf2	Phlda3	Ccn4	Rrm1
Chst12	AABR07068150.1	AABR07065970.1	Nup85	Pla2g15	Ccna2	Sgo1
Chsy1	AABR07068285.2	AABR07067506.1	Nusap1	Plk1	Ccnb1	Shcbp1
Ckap2l	AABR07069282.1	AABR07067526.1	Orc6	Plk2	Ccne1	Ska1
Cks1b	AABR07069433.1	AABR07067583.1	P2rx4	Plk4	Ccnf	Ska3
Cnpy4	AABR07069490.1	AABR07068127.1	Pbk	Pole	Cct4	Slbp
Col18a1	AABR07070810.1	AABR07068253.1	Pclaf	Prc1	Cdc20	Smc2
Cryab	AABR07071000.1	AABR07068316.1	Phlda3	Prim1	Cdc42ep2	Smc4
Cxcl16	AABR07071779.2	AABR07069282.1	Pimreg	Psat1	Cdc45	Spag5
Dek	AABR07072236.1	AABR07069433.1	Pla2g15	Ptma	Cdc6	Spc25
Des	AABR07072559.2	AABR07069462.1	Plk1	Pttg1	Cdc7	Srsf7
Dlgap5	AC094126.2	AABR07070161.3	Plk2	Racgap1	Cdca2	Stil
Dnajb4	AC094217.1	AABR07070810.1	Plk4	Rad21	Cdca3	Stmn1
Dnmt1	AC094647.1	AABR07071000.1	Pole	Rasl2-9	Cdca4	Tacc3
Dtx3l	AC095390.1	AABR07072236.1	Prc1	Rfc5	Cdca7	Tcf19
Dtymk	AC095678.1	AABR07072559.2	Prim1	Rhob	Cdca8	Timeless
Dusp4	AC095947.1	AABR07072761.1	Psat1	Rogdi	Cdk1	Tk1
Dut	AC096239.2	AABR07073038.1	Ptma	Rpa2	Cdk2	Top2a
E2f8	AC098125.3	Aamp	Pttg1	Rpa3	Cdkn1a	Tpx2
Ect2	AC099089.1	Abtb1	Pycard	Rps6ka4	Cdkn1b	Troap
Edem1	AC099453.2	AC094126.2	RGD1560010	Rrm1	Cdt1	Ttk
Edf1	AC103024.1	AC094643.1	Racgap1	S100a10	Cenpa	Ube2c
Eef1g	AC103090.1	AC094643.2	Rad21	Sgo1	Cenpe	Ube2s
Eme1	AC106663.1	AC095390.1	Rfc3	Shcbp1	Cenpf	Uhrf1
Espl1	AC112018.1	AC099089.1	Rfc5	Ska1	Cenph	Usp1
Fam168b	AC112350.1	AC099137.1	Rhob	Ska3	Cenpo	Wdhd1
Fam214b	AC118419.1	AC103090.1	Rogdi	Slbp	Cenpu	Zwint
Fancd2	AC118772.2	AC106663.1	Rpa2	Slc38a7	Cenpw	
Fanci	AC118772.3	AC107096.2	Rpa3	Smc2	Cep295	
Fbxo5	AC119015.4	AC110351.2	Rrm1	Smc4	Cep55	

Fgf22	AC119496.1	AC112018.1	Sgo1	Spag5	Chaf1a
Foxa2	AC119762.3	AC112350.1	Shcbp1	Spc25	Chaf1b
Foxm1	AC119762.7	AC114460.1	Siglec10	Srsf7	Chmp1a
G3bp2	AC120262.2	AC115420.2	Siva1	Stil	Chpf2
Gaa	AC120486.3	AC116236.2	Ska1	Stmn1	Cip2a
Gadd45a	AC120486.9	AC119015.4	Ska3	Tacc3	Ckap2l
Galnt1	AC121413.2	AC119762.7	Slbp	Tcf19	Cks1b
Gins3	AC123425.1	AC120096.3	Slc20a2	Timeless	Cks2
Glt8d1	AC126899.1	AC120246.2	Slc25a10	Tk1	Clba1
Gm23880	AC127784.2	AC121413.2	Smc2	Tlr5	Clu
Gpx8	AC128207.2	AC122625.1	Smc4	Tmem101	Cnnm4
Grwd1	AC128960.1	AC122630.1	Spag5	Tmem150a	Cnot9
H2afx	AC129365.1	AC123425.1	Spc25	Tnfaip8l1	Col3a1
H2afz	AC130232.1	AC124205.1	Srsf7	Top2a	Cpt1c
H3f3c	AC130232.2	AC126572.7	Stil	Tpm3	Cry2
Hacd1	AC130391.1	AC126897.1	Stmn1	Tpx2	Cst3
Haspin	AC130391.5	AC127920.1	Tacc3	Troap	Ctsa
Haus4	AC134224.1	AC128212.1	Tcf19	Ttk	Ctsb
Hist1h1t	AC134224.2	AC129365.1	Timeless	Tuba1b	Cyp1a1
Hjurp	AC134224.3	AC130232.1	Tk1	Ube2c	Dbn1
Hmgb2	AC141028.1	AC130391.1	Tob1	Ube2s	Dctpp1
Hmmr	AC141152.1	AC130391.5	Top2a	Uhrf1	Ddit3
Hoxb7	AC142138.1	AC131360.1	Tpx2	Usp1	Ddit4
Hspb2	AC142180.1	AC134224.1	Trim25	Wdhd1	Ddx11
Htra1	AC142458.1	AC134224.2	Troap	Zwint	Ddx39a
Hyls1	AY172581.20	AC134224.3	Tspo		Dek
Icam1	AY172581.24	AC139392.1	Ttk		Depp1
Idh2	Aaas	AC141220.2	Tusc2		Dhfr
Ids	Abcc5	AC141377.3	Ubald2		Dlgap5
Ifi30	Abcd1	AC141966.1	Ube2c		Dnajb9
Ifi44	Abcd4	AC142458.1	Ube2s		Dnajc9
Igfbp6	Abhd14b	Aaas	Uhrf1		Dnmt1
Incenp	Abhd4	Acad11	Usp1		Dpp7
Ins2	Adam19	Acadm	Wdhd1		Dscc1
Ip6k1	Adam8	Acads	Zwint		Dsn1
Iqgap3	Adamts4	Acot7			Dstn
Irf7	Adamts7	Actb			Dtl
Itga7	Adamtsl5	Actl7b			Dtx3
Itpr3	Adipor1	Actr1b			Dut
Jpt1	Adm	Actrt3			Dvl2
Jsrp1	Adrb2	Acy3			Dyrk1b
Kcnf1	Aen	Adam15			E2f4
Kif11	Aga	Adamtsl5			E2f8
Kif20a	Ahcy	Adgra2			Eci1
Kif22	Ahr	Ado			Ect2
Kif23	Ahsp	Adrb2			Eef2
Kif2c	Ajuba	Adss			Ehmt2
Kifc1	Akr1b1	Aga			Eme1
Klf2	Akt1	Agpat1			Emg1
Klhl9	Aktip	Agpat2			Epha7
Knstrn	Aldh2	Ahcy			Ephx1
Kntc1	Aldh7a1	Ahsa1			Ercc6l
Kpna2	Alg8	Akr1c14			Ergic3
LOC100359539	Alyref	Akt1			Etv5
LOC100359600	Amotl2	Akt1s1			Exo1
LOC100362333	Anapc15	Aktip			Exosc8
LOC100364062	Ankh	Alas1			F11r
LOC100910678	Ankrd49	Aldh5a1			F2rl1
LOC102547056	Ankzf1	Alg10			Fadd
LOC290595	Anp32e	Alkbh7			Fam111a
LOC317456	Anxa1	Alyref			Fam3a
LOC499331	Anxa3	Amdhd2			Fam83d
Lamp1	Apol9a	Anapc1			Fancd2
Lfng	Apold1	Anapc15			Fanci
Lgals3	App	Angptl4			Fbl
Lgals3bp	Arhgap11a	Ankrd13a			Fbxo5
Lix1l	Arhgap19	Ankrd49			Fen1
Lmf2	Arhgap23	Ankrd52			Fignl1
Lmnb1	Arhgap29	Ankrd53			Fkbp4
Lmnb2	Arhgdia	Anln			Flcn
Lsm2	Arl4a	Anp32b			Foxm1
Mad2l1	Arl6ip1	Anp32e			Ftl1
Mapk6	Arpc4	Anxa1			Fzd2

Mcm10	Asap1	Aox1			G2e3	
Mcm2	Asf1b	Ap2a2			G3bp1	
Mcm3	Aspm	Api5			Gaa	
Mcm4	Atad2	Apol9a			Gabarapl1	
Mcm6	Atad5	Aqp1			Gadd45a	
Mdm2	Atg12	Aqp8			Gadd45g	
Mid1ip1	Atp1a1	Areg			Gale	
Mir1956	Atp2b1	Arfip2			Gas2l3	
Mir33	Atp2b4	Arhgap11a			Gbp2	
Mir3564	Atp6v1d	Arhgap4			Gfpt1	
Mir615	Atraid	Arhgdia			Gga2	
Mki67	Atrn	Arl2bp			Ggt1	
Mmp11	Atxn7l3b	Arl4a			Gigyf1	
Mob4	Aunip	Armc7			Gins1	
Mras	Aurka	Arpc5			Gins4	
Mt1m	Aurkb	Arrdc3			Giot1	
Mybl2	Avpi1	Asf1b			Gja1	
Myod1	Axl	Asl			Gltp	
Nagpa	B2m	Asmtl			Gnptg	
Nanos1	B3galt4	Aspm			Gpd1l	
Nans	B4galt5	Atad2			Gpnmb	
Nasp	B9d2	Atad5			Gpr146	
Ncapd2	Babam1	Atf5			Gpr88	
Ncapg	Bak1	Atg101			Gprasp2	
Ncapg2	Bard1	Atg12			Gprin3	
Ncaph	Bax	Atp1b1			Gpsm2	
Ncaph2	Baz2a	Atp2b4			Grina	
Ndc80	Bcat1	Atp5f1e			Gtpbp2	
Ndrg1	Bccip	Atp6v0d1			H2afz	
Necab3	Bcl2l1	Atp6v1a			Haspin	
Neurl2	Bcl2l12	Atp6v1b2			Hat1	
Nhlrc3	Bdh2	Atp6v1d			Haus4	
Nono	Becn1	Atp6v1f			Hbp1	
Nrm	Birc5	Atp6v1g1			Hirip3	
Nsmce4a	Blcap	Aunip			Hist1h1c	
Nuf2	Bmp1	Aurka			Hist1h1d	
Nup85	Bmp2	Aurkb			Hist1h1t	
Nusap1	Bod1	Avpr2			Hist1h2bd	
Olr1684	Bok	AY172581.20			Hjurp	
Orc6	Bora	AY172581.24			Hmmr	
P2rx4	Borcs6	AY172581.9			Hmox1	
Parp14	Brca1	B2m			Hnrnpa1	
Parp9	Brca2	B3gnt9			Homer3	
Pbk	Bsg	Bak1			Hoxc8	
Pclaf	Bst2	Bbip1			Hsdl1	
Phlda2	Btbd19	Bbs10			Hsp90b1	
Phlda3	Btg2	Bcdin3d			Hspa5	
Pimreg	Btn2a2	Bckdha			Hspb2	
Pla2g15	Bub1	Bcl2l11			Hyls1	
Plin2	Bub1b	Bdh2			Hyou1	
Plk1	Bzw2	Bhlhe41			Idh2	
Plk2	C1qtnf5	Birc5			Ier5	
Plk4	C1rl	Blcap			Il6r	
Pmepa1	C1s	Bles03			Incenp	
Pole	Calr	Bmf			Ip6k2	
Prc1	Car1	Bnip3l			Iqgap3	
Prdx2	Car9	Bora			Isg15	
Prim1	Casp8ap2	Borcs6			Itgb1bp1	
Prss23	Cast	Brca1			Itm2c	
Psat1	Cavin2	Bri3			Jag2	
Ptma	Cbx3	Btbd19			Kcnn4	
Pttg1	Cc2d1a	Btg2			Kif11	
Pycard	Ccdc163	Bub1			Kif20a	
Pycr2	Ccdc43	Bub1b			Kif20b	
Pygo2	Ccdc80	C1qtnf6			Kif22	
RF00265	Ccdc9b	C1r			Kif2c	
RF00288	Ccn1	C1s			Kif4a	
RF00438	Ccn2	Cacybp			Kifc1	
RF00594	Ccn4	Cad			Klhdc3	
RF00614	Ccna2	Calml3			Klhl24	
RF01294	Ccnb1	Calml4			Klhl31	
RGD1560010	Ccne1	Camk1			Klhl41	
Racgap1	Ccne2	Car5b			Knstrn	

Rad21	Ccnf	Carm1			Kntc1	
Rasl2-9	Ccng1	Cavin1			Kpna2	
Rbmx	Cct4	Cavin2			Krt26	
Rfc3	Cct7	Cbx3			LOC100359539	
Rfc5	Ccz1b	Cc2d1b			LOC100359583	
Rfwd3	Cd63	Ccdc115			LOC100359600	
Rgs2	Cd80	Ccdc189			LOC102546716	
Rhob	Cdc20	Ccdc96			LOC102547645	
Rnase17	Cdc42ep1	Ccl21			LOC102553785	
Rnf181	Cdc42ep2	Ccn2			LOC102555453	
Rogdi	Cdc45	Ccn4			LOC498368	
Rpa2	Cdc6	Ccna2			LOC684773	
Rpa3	Cdc7	Ccnb1			LOC686013	
Rpp38	Cdca2	Ccnd3			LOC688459	
Rps6ka4	Cdca3	Ccndbp1			LOC689412	
Rps6kb1	Cdca4	Ccne1			Lamb2	
Rrm1	Cdca7	Ccnf			Lamp1	
S100a10	Cdca8	Ccpg1			Lamp2	
Sat1	Cdipt	Ccpg1os			Laptm4a	
Sdf2l1	Cdk1	Cct4			Las1l	
Selenoh	Cdk2	Cct6a			Ldb1	
Sfrp2	Cdk2ap2	Cd1d1			Lgmn	
Sgo1	Cdkn1a	Cd320			Lig4	
Shcbp1	Cdkn1b	Cd37			Limd2	
Siglec10	Cdt1	Cd3eap			Lmnb1	
Siva1	Cebpa	Cdc20			Lmnb2	
Ska1	Cebpg	Cdc25b			Loxl4	
Ska3	Cenpa	Cdc42ep2			Lrrcc1	
Skil	Cenpe	Cdc45			Lsm2	
Slbp	Cenpf	Cdc6			Lsm3	
Slc20a2	Cenph	Cdc7			Lsm4	
Slc25a10	Cenpm	Cdca2			Lsm8	
Slc35c1	Cenpo	Cdca3			Lyar	
Slc38a7	Cenpt	Cdca4			Mad2l1	
Smarcd2	Cenpu	Cdca7			Maf	
Smc1a	Cenpw	Cdca8			Mapre3	
Smc2	Cep170b	Cdh3			Mbd3	
Smc4	Cep250	Cdhr1			Mcm10	
Socs1	Cep295	Cdk1			Mcm2	
Sohlh1	Cep55	Cdk2			Mcm3	
Spag5	Cerk	Cdk2ap2			Mcm4	
Spc25	Cetn2	Cdk5r1			Mcm5	
Srsf7	Cetn4	Cdkn1a			Mcm6	
Stbd1	Cfl2	Cdkn1b			Mcm7	
Stil	Chac1	Cdr2			Mdm2	
Stmn1	Chaf1a	Cdt1			Mepe	
Syngr2	Chaf1b	Cebpa			Mfsd1	
Syt8	Chchd1	Cenpa			Mir146b	
Tacc3	Chchd7	Cenpb			Mir196c	
Tcf19	Chmp1a	Cenpe			Mirlet7d	
Tedc1	Chmp4c	Cenpf			Mis18a	
Tgfb2	Chpf2	Cenph			Mk1	
Tgfb3	Chrac1	Cenpo			Mki67	
Timeless	Chtf18	Cenpu			Mme	
Tk1	Cip2a	Cenpw			Msln	
Tlr5	Cisd1	Cep295			Mtss1l	
Tm4sf1	Ckap2	Cep55			Mxd1	
Tmem101	Ckap2l	Cfap298			Mxd4	
Tmem150a	Cks1b	Cfh			Mybl2	
Tmem199	Cks2	Chaf1a			Nap1l3	
Tmem38b	Clba1	Chaf1b			Nasp	
Tnfaip8l1	Clcf1	Chmp1a			Ncapd2	
Tob1	Cldn12	Chmp5			Ncapd3	
Top2a	Cldnd1	Chpf2			Ncapg	
Tpm3	Clec2dl1	Chst12			Ncapg2	
Tpx2	Clip2	Chst14			Ncaph	
Trim25	Clspn	Churc1			Ndc80	
Troap	Cltb	Cip2a			Nfat5	
Tspan13	Clu	Ckap2l			Nnt	
Tspo	Cmtm3	Ckap4			Nrm	
Ttk	Cnnm4	Ckap5			Nucks1	
Tuba1b	Cnot9	Cks1b			Nuf2	
Tusc2	Cnppd1	Cks2			Numbl	

Tymp	Cnpy4	Clba1			Nup107	
Ubald2	Col1a1	Clic1			Nup35	
Ube2c	Col3a1	Clu			Nup43	
Ube2s	Col4a5	Cmklr1			Nup85	
Ucp2	Col5a1	Cmpk1			Nusap1	
Uhrf1	Col5a2	Cmtm6			Nxph3	
Usp1	Col6a1	Cmtr2			Nxt1	
Wdhd1	Col6a2	Cnih1			Orc1	
Wnt4	Cpsf3	Cnnm4			Orc6	
Ykt6	Cpt1c	Cnot9			Osgin1	
Zfp395	Creb3	Cnp			P2rx4	
Zfp513	Creld2	Cnpy2			Pa2g4	
Zwint	Cry2	Cog4			Palb2	
	Cryab	Col3a1			Paqr4	
	Cspg4	Commd4			Pbk	
	Cst3	Commd8			Pclaf	
	Cstf2	Comtd1			Pcna	
	Ctdsp1	Cox6a2			Pcolce	
	Ctsa	Cpe			Pdia4	
	Ctsb	Cpt1c			Pelp1	
	Ctsd	Crat			Pgp	
	Cttnbp2nl	Crebrf			Pgpep1	
	Cuedc1	Creld1			Pgrmc1	
	Cuedc2	Crot			Phf19	
	Cxcl16	Cry2			Phgdh	
	Cyp1a1	Csf1			Phlda3	
	Cyp1b1	Csrp1			Phldb3	
	Dazap2	Cst3			Pik3ip1	
	Dbi	Ctbp1			Pinlyp	
	Dbn1	Ctdsp2			Pla2g15	
	Dbnl	Ctns			Plag1	
	Dcbld2	Ctps1			Plcd4	
	Dctpp1	Ctsa			Plcg1	
	Ddit3	Ctsb			Pld3	
	Ddit4	Ctxn1			Plekhb2	
	Ddr1	Cyb561a3			Plk1	
	Ddx11	Cyp1a1			Plk2	
	Ddx28	Cyp3a9			Plk4	
	Ddx39a	Dact3			Pmf1	
	Dek	Dbn1			Pnrc1	
	Depp1	Dbp			Pold1	
	Dhfr	Dcakd			Pold2	
	Dhrs13	Dclre1b			Pole	
	Diaph1	Dctpp1			Ppat	
	Dipk1a	Ddit3			Ppm1g	
	Dlgap5	Ddit4			Pqlc2	
	Dlx1	Ddx11			Prc1	
	Dlx2	Ddx21			Prim1	
	Dmac2	Ddx39a			Psap	
	Dnajb9	Decr1			Psat1	
	Dnajc9	Dek			Psca	
	Dnmt1	Depdc1			Psmc3ip	
	Dpp7	Depp1			Ptgs1	
	Dpysl2	Dgat2			Ptma	
	Dscc1	Dhfr			Pttg1	
	Dsn1	Dhrs1			RF00405	
	Dstn	Dhrs9			RF00421	
	Dtd2	Dlgap5			RF00492	
	Dtl	Dlx5			RF00582	
	Dtx3	Dmpk			RF00586	
	Dtx3l	Dnaja4			RGD1308117	
	Dtymk	Dnajb1			RGD1309748	
	Dusp18	Dnajb5			RGD1562378	
	Dusp6	Dnajb9			RT1-S3	
	Dusp8	Dnajc9			Racgap1	
	Dut	Dnm1			Rad21	
	Dvl2	Dnmt1			Rad51	
	Dvl3	Dnpep			Rad51ap1	
	Dync1li2	Dpep3			Ranbp1	
	Dyrk1b	Dpp7			Rangap1	
	E2f4	Dpp9			Rapsn	
	E2f8	Dscc1			Rasl11b	
	Eci1	Dsn1			Rbmx2	

	Ect2	Dstn			Rbmxl1b	
	Eddm3b	Dtl			Rcc2	
	Eef1aknmt	Dtx3			Reep3	
	Eef1g	Dusp1			Retreg3	
	Eef2	Dusp7			Rfc2	
	Efna4	Dusp8			Rfc5	
	Egfl7	Dut			Rhob	
	Egln2	Dvl2			Rhod	
	Egr1	Dynlt1			Rnd3	
	Ehmt2	Dynlt3			Rnf166	
	Eif2a	Dyrk1b			Rnps1	
	Eif3d	Dyrk3			Rogdi	
	Elovl1	E2f4			Rpa2	
	Emc10	E2f8			Rpa3	
	Emc8	Ech1			Rrm1	
	Eme1	Eci1			Rrm2	
	Emg1	Ect2			Samd9	
	Epgn	Eef1a1			Sapcd2	
	Epha2	Eef2			Scarf2	
	Epha7	Efhd2			Sec22b	
	Ephx1	Ehd4			Sema3b	
	Ercc6l	Ehmt2			Serpinb7	
	Ergic3	Eid1			Serpinb8	
	Esyt1	Eid2b			Sesn2	
	Etv3	Eif4a1			Set	
	Etv5	Eif4e			Sf3b3	
	Eva1b	Eif4ebp1			Sft2d3	
	Exd2	Eif4ebp2			Sgo1	
	Exo1	Eif5a			Sh3gl1	
	Exosc2	Elk3			Shc1	
	Exosc8	Elk4			Shcbp1	
	F11r	Emd			Ska1	
	F2rl1	Eme1			Ska3	
	F3	Eme2			Slbp	
	Fabp4	Emg1			Slc10a5	
	Fadd	Emp1			Slc19a2	
	Fam111a	Emp3			Slc1a5	
	Fam214b	Eno3			Slc25a44	
	Fam3a	Enpep			Slc25a5	
	Fam83d	Entpd4			Slc35d2	
	Fam89b	Epha7			Slc39a9	
	Fancd2	Ephx1			Slc47a1	
	Fancg	Epn2			Slc52a3	
	Fanci	Eps8l2			Slc6a8	
	Farsa	Ercc1			Slc7a5	
	Fat1	Ercc6l			Smagp	
	Fbl	Ereg			Smc2	
	Fbxo5	Ergic3			Smc4	
	Fcho2	Erlec1			Snn	
	Fen1	Erlin1			Snrpa	
	Fermt2	Ermard			Snrpa1	
	Fhl1	Espl1			Snrpb	
	Fhl2	Etfrf1			Snrpf	
	Fhod1	Etv5			Snx33	
	Fignl1	Evi2a			Sod3	
	Filip1l	Exo1			Sowahc	
	Fkbp3	Exoc3l1			Spag5	
	Fkbp4	Exosc8			Sparc	
	Fkbp9	Extl3			Spc24	
	Flcn	Ezh1			Spc25	
	Flnc	Ezh2			Sqstm1	
	Flrt3	F11r			Srsf7	
	Fnip2	F2r			Ssr3	
	Fosl2	F2rl1			Ssrp1	
	Foxm1	F3			St3gal1	
	Fst	Fabp3			Stil	
	Fstl1	Fadd			Stip1	
	Ftl1	Fam107b			Stmn1	
	Fxyd5	Fam110b			Suv39h1l1	
	Fzd1	Fam111a			Svbp	
	Fzd2	Fam136a			Syde1	
	Fzd5	Fam168b			Tacc3	
	G2e3	Fam229a			Tap1	

	G3bp1	Fam25a			Tbc1d17	
	G3bp2	Fam3a			Tcf19	
	Gaa	Fam83d			Tcn2	
	Gab1	Fam98c			Tent5a	
	Gabarapl1	Fancd2			Terf2ip	
	Gadd45a	Fanci			Tfdp1	
	Gadd45g	Far1			Ticrr	
	Gale	Fau			Timeless	
	Gas2l3	Fbl			Timp2	
	Gata2	Fbxl7			Tipinl1	
	Gata3	Fbxo5			Tk1	
	Gbp2	Fbxw9			Tmem127	
	Gdf15	Fcgrt			Tmem132a	
	Gemin6	Fem1b			Tmem138	
	Gfpt1	Fen1			Tmem185b	
	Gga2	Fgfbp3			Tmem59	
	Ggh	Fhl3			Tmem97	
	Ggt1	Fignl1			Tmpo	
	Gigyf1	Fis1			Tnnc1	
	Gins1	Fkbp10			Tonsl	
	Gins3	Fkbp1a			Top2a	
	Gins4	Fkbp4			Topbp1	
	Giot1	Fkbp7			Tp53inp1	
	Gja1	Fkbpl			Tpp1	
	Gja4	Flcn			Tpx2	
	Gjb4	Flot2			Traf4	
	Gjb5	Fmod			Traip	
	Glb1l	Fosl1			Trim34	
	Glb1l2	Foxc1			Trip13	
	Glrx3	Foxm1			Troap	
	Gltp	Foxo4			Tsc22d3	
	Gmfg	Ftl1			Ttk	
	Gmip	Fubp1			Tubb5	
	Gnptg	Fuca1			Tvp23b	
	Golt1b	Fzd2			U2af1	
	Got1	G2e3			Ube2c	
	Gpank1	G3bp1			Ube2s	
	Gpd1l	Gaa			Ube2t	
	Gper1	Gabarapl1			Ugdh	
	Gpi	Gadd45a			Ugt1a1	
	Gpn1	Gadd45g			Uhrf1	
	Gpnmb	Gale			Ung	
	Gpr146	Gar1			Usp1	
	Gpr88	Gas2l3			Wbp1l	
	Gprasp2	Gatad2a			Wdhd1	
	Gprc5a	Gba			Wdr5	
	Gprin3	Gbp2			Yod1	
	Gpsm2	Gcdh			Ypel5	
	Gpx8	Gdi1			Zfp219	
	Grina	Gfpt1			Zscan25	
	Gtpbp2	Gga2			Zwint	
	H1fx	Ggt1				
	H2afz	Ggt7				
	H3f3b	Ghdc				
	Hadha	Gigyf1				
	Hadhb	Gins1				
	Hapln3	Gins4				
	Haspin	Giot1				
	Hat1	Gja1				
	Haus3	Glrx5				
	Haus4	Gltp				
	Haus5	Glyctk				
	Hbp1	Gna11				
	Hdac1	Gng8				
	Hdac1l	Gnpda1				
	Hexb	Gnptg				
	Hid1	Gpd1l				
	Hirip3	Gpnmb				
	Hist1h1c	Gpr146				
	Hist1h1d	Gpr165				
	Hist1h1t	Gpr88				
	Hist1h2bd	Gprasp2				
	Hist2h2be	Gprin3				

	Hjurp	Gpsm2				
	Hlx	Gpt				
	Hmgb2	Grina				
	Hmgn2	Grk2				
	Hmmr	Grk6				
	Hmox1	Grn				
	Hnrnpa1	Gtpbp2				
	Homer3	Gtse1				
	Hoxa1	H2afz				
	Hoxc8	Haghl				
	Hoxd8	Hapln4				
	Hr	Haspin				
	Hsdl1	Hat1				
	Hsp90b1	Haus4				
	Hspa13	Hbp1				
	Hspa5	Hdgf				
	Hspa9	Hexa				
	Hspb2	Hexim2				
	Htr2a	Hfe				
	Hyal2	Hgh1				
	Hyls1	Hif1an				
	Hyou1	Higd2a				
	Id4	Hint2				
	Idh2	Hirip3				
	Ier2	Hist1h1c				
	Ier3	Hist1h1d				
	Ier5	Hist1h1t				
	Ifi27l2b	Hist1h2ah				
	Ifitm2	Hist1h2ail1				
	Ifngr2	Hist1h2ao				
	Ifrd1	Hist1h2bd				
	Ikbip	Hist1h2bk				
	Il1rl1	Hist1h2bl				
	Il6r	Hist1h4b				
	Il6st	Hist2h2be				
	Impact	Hjurp				
	Incenp	Hjv				
	Ing1	Hmga1				
	Inpp5d	Hmgcl				
	Ip6k2	Hmmr				
	Iqgap3	Hmox1				
	Irf7	Hnrnpa1				
	Irf9	Hnrnpul2				
	Isg15	Homer3				
	Itga3	Hoxb5				
	Itga5	Hoxb7				
	Itga7	Hoxc10				
	Itgax	Hoxc6				
	Itgb1bp1	Hoxc8				
	Itm2c	Hpx				
	Itpripl1	Hrct1				
	Jag2	Hs1bp3				
	Jtb	Hsd17b1				
	Jund	Hsd17b8				
	Kat2a	Hsd3b7				
	Kazald1	Hsdl1				
	Kcnh6	Hsf1				
	Kcnn4	Hsp90ab1				
	Kctd21	Hsp90b1				
	Kdelr3	Hspa14				
	Kdm5b	Hspa5				
	Kdm6b	Hspb2				
	Kif11	Hspd1				
	Kif15	Hyal1				
	Kif1c	Hyls1				
	Kif20a	Hyou1				
	Kif20b	Iah1				
	Kif22	Iba57				
	Kif2c	Icoslg				
	Kif4a	Idh2				
	Kifc1	Idh3a				
	Kifc3	Idnk				
	Klc2	Ids				

	Klf2	Idua				
	Klhdc3	Ier5				
	Klhdc8a	Ifi27				
	Klhl24	Ifi35				
	Klhl31	Ifi44				
	Klhl41	Ifitm1				
	Kmt5a	Ifitm3				
	Knstrn	Ifitm6				
	Kntc1	Ifrd2				
	Kpna2	Igbp1				
	Kpna3	Igfbp5				
	Kpna6	Igfbp7				
	Kprp	Igip				
	Krt26	Il18bp				
	Krt79	Il6r				
	Krt8	Ilvbl				
	Krtcap2	Incenp				
	Ktn1	Insyn1				
	LOC100125364	Ip6k2				
	LOC100294508	Iqcd				
	LOC100359539	Iqgap3				
	LOC100359583	Irak1				
	LOC100359600	Iscu				
	LOC100360117	Isg15				
	LOC100362400	Islr				
	LOC100362830	Isy1				
	LOC100363452	Itga10				
	LOC100363469	Itga3				
	LOC100365363	Itgb1bp1				
	LOC100365839	Itm2b				
	LOC100911361	Itm2c				
	LOC100912365	Jag2				
	LOC102546716	Jmjd6				
	LOC102547645	Jpt2				
	LOC102553785	Kcnf1				
	LOC102555453	Kcnh2				
	LOC102556092	Kcnn4				
	LOC102557137	Kctd11				
	LOC103694328	Khk				
	LOC252890	Khnyn				
	LOC290595	Kif11				
	LOC292543	Kif18b				
	LOC301444	Kif20a				
	LOC303566	Kif20b				
	LOC361346	Kif22				
	LOC498368	Kif23				
	LOC684773	Kif2c				
	LOC686013	Kif4a				
	LOC688459	Kifc1				
	LOC689039	Kifc2				
	LOC689065	Klc4				
	LOC689412	Klf15				
	LOC691170	Klhdc3				
	LOC691807	Klhdc8b				
	Lama5	Klhl24				
	Lamb2	Klhl31				
	Lamp1	Klhl41				
	Lamp2	Kmt2b				
	Lamtor5	Knstrn				
	Laptm4a	Kntc1				
	Las1l	Kpna2				
	Lasp1	Krt26				
	Lats2	Lamb2				
	Ldb1	Lamc2				
	Lgmn	Lamp1				
	Lig4	Lamp2				
	Limd2	Laptm4a				
	Limk2	Laptm4b				
	Lman1	Las1l				
	Lmbr1l	Lcn12				
	Lmnb1	Ldb1				
	Lmnb2	Ldha				
	Lox	Ldlr				

	Loxl4	Lgmn				
	Lpar6	Lig4				
	Lpin3	Limd2				
	Lrfn4	Lin37				
	Lrig1	Lin7c				
	Lrr1	Lmnb1				
	Lrrc32	Lmnb2				
	Lrrc40	LOC100174910				
	Lrrcc1	LOC100359539				
	Lsm2	LOC100359583				
	Lsm3	LOC100359600				
	Lsm4	LOC100361265				
	Lsm5	LOC100363502				
	Lsm7	LOC100364062				
	Lsm8	LOC100365043				
	Lyar	LOC100909474				
	Lypla1	LOC100909912				
	Lzts3	LOC100912427				
	MGC116202	LOC102546716				
	Mad2l1	LOC102547056				
	Maf	LOC102547645				
	Mafg	LOC102551095				
	Magoh	LOC102551606				
	Maml1	LOC102553785				
	Manf	LOC102554034				
	Map1a	LOC102555453				
	Mapk3	LOC103690190				
	Mapre3	LOC103691238				
	Marcks	LOC108348201				
	Mbd3	LOC108348302				
	Mbtps1	LOC108348771				
	Mcm10	LOC108351584				
	Mcm2	LOC361985				
	Mcm3	LOC363337				
	Mcm4	LOC498368				
	Mcm5	LOC499235				
	Mcm6	LOC679894				
	Mcm7	LOC680491				
	Mcrip1	LOC684762				
	Mdc1	LOC684773				
	Mdh1	LOC686013				
	Mdm2	LOC687707				
	Me1	LOC688459				
	Med18	LOC688672				
	Melk	LOC689412				
	Mepe	LOC691418				
	Metrn	Loxl1				
	Metrnl	Loxl2				
	Mfge8	Loxl4				
	Mfsd1	Lrpap1				
	Mfsd10	Lrrc59				
	Mgat1	Lrrc73				
	Mgme1	Lrrc75b				
	Midn	Lrrcc1				
	Mir146b	Lrrfip1				
	Mir193a	Lrrn4cl				
	Mir196c	Lsm2				
	Mir22	Lsm3				
	Mir221	Lsm4				
	Mir222	Lsm8				
	Mir3064	Lsmem2				
	Mir365-1	Ltbp4				
	Mir6326	Lyar				
	Mirlet7c2	Lyrm2				
	Mirlet7d	Lztfl1				
	Mis18a	Mad2l1				
	Mk1	Maf				
	Mki67	Maf1				
	Mllt11	Manea				
	Mmab	Map3k6				
	Mme	Mapre3				
	Mmp19	March3				
	Mms22l	Mbd3				

	Mob1a	Mcm10				
	Mprip	Mcm2				
	Mre11a	Mcm3				
	Mrfap1	Mcm4				
	Mrpl14	Mcm5				
	Mrpl28	Mcm6				
	Mrpl35	Mcm7				
	Mrps12	Mdm2				
	Mrps18b	Me2				
	Msln	Med1				
	Msmp	Med25				
	Mt1	Med31				
	Mt2A	Med6				
	Mtss1l	Med9				
	Mxd1	Mepe				
	Mxd4	Mex3c				
	Mxra8	Mfsd1				
	Mybl1	Mfsd11				
	Mybl2	Mief2				
	Myo10	Mir145				
	Myof	Mir146b				
	Myrf	Mir196c				
	N4bp1	Mir206				
	Naca	Mir24-2				
	Nacc2	Mir2964				
	Nagk	Mir3074				
	Nap1l3	Mir874				
	Nasp	Mirlet7d				
	Nbas	Mis18a				
	Ncam1	Mk1				
	Ncapd2	Mki67				
	Ncapd3	Mkrn3				
	Ncapg	Mlkl				
	Ncapg2	Mlycd				
	Ncaph	Mmaa				
	Ncaph2	Mmd				
	Nckap5l	Mme				
	Ndc80	Mmgt1				
	Ndel1	Mmp13				
	Ndfip1	Mocs3				
	Ndufaf3	Mpg				
	Ndufc1	Mpst				
	Necab3	Mpv17l2				
	Necap1	Mrc2				
	Nfat5	Mrpl2				
	Nfe2l2	Mrpl45				
	Nicn1	Mrpl52				
	Nisch	Mrpl57				
	Nkiras2	Mrps6				
	Nlgn2	Mrto4				
	Nnt	Msln				
	Npepps	Msn				
	Npnt	Mtch1				
	Nptxr	Mtcp1				
	Nr1i3	Mtmr11				
	Nras	Mtss1l				
	Nrm	Mustn1				
	Nuak2	Mx1				
	Nucb1	Mxd1				
	Nucks1	Mxd4				
	Nudt1	Myadm				
	Nuf2	Mybl2				
	Numbl	Myc				
	Nup107	Mynn				
	Nup133	Myo1e				
	Nup155	Myo5a				
	Nup35	Naa10				
	Nup43	Nacc1				
	Nup62	Nadk2				
	Nup85	Nap1l3				
	Nusap1	Nasp				
	Nxph3	Nat9				
	Nxt1	Ncapd2				

	Oip5	Ncapd3				
	Orai3	Ncapg				
	Orc1	Ncapg2				
	Orc6	Ncaph				
	Osbpl5	Ncl				
	Osgin1	Ncstn				
	Otulinl	Ndc1				
	P2rx4	Ndc80				
	P4ha2	Ndor1				
	Pa2g4	Ndufb2				
	Pacsin3	Nemp1				
	Pagr1	Net1				
	Palb2	Neurl2				
	Paqr4	Nfat5				
	Pars2	Ngp				
	Pawr	Ngrn				
	Pbk	Nhlrc3				
	Pcdh20	Nit1				
	Pcgf2	Nkx3-2				
	Pclaf	Nme2				
	Pcna	Nme3				
	Pcolce	Nnt				
	Pcyox1	Nolc1				
	Pdcd2	Nop53				
	Pdia4	Nop56				
	Pdlim1	Nop58				
	Pdlim7	Npc2				
	Pdrg1	Npm3				
	Pea15	Nqo1				
	Pelo	Nradd				
	Pelp1	Nrbp1				
	Pfas	Nrbp2				
	Pfdn4	Nrm				
	Pgghg	Ns5atp4				
	Pgp	Nsun2				
	Pgpep1	Nt5c				
	Pgrmc1	Nucks1				
	Pgrmc2	Nuf2				
	Phf1	Numbl				
	Phf19	Nup107				
	Phgdh	Nup205				
	Phlda3	Nup35				
	Phldb3	Nup43				
	Phospho1	Nup85				
	Pias3	Nusap1				
	Pid1	Nxf7				
	Pif1	Nxph3				
	Pih1d1	Nxt1				
	Pik3c2a	Orc1				
	Pik3ip1	Orc6				
	Pimreg	Ormdl2				
	Pin4	Osgin1				
	Pinlyp	P2rx4				
	Pkmyt1	P2ry4				
	Pkp1	P4ha3				
	Pla2g15	P4hb				
	Plag1	Pa2g4				
	Plat	Pak1ip1				
	Plcd3	Pak4				
	Plcd4	Palb2				
	Plcg1	Paqr4				
	Pld3	Pbk				
	Plekha3	Pcbp2				
	Plekhb2	Pcdh20				
	Plekhg2	Pcdhb14				
	Plekho1	Pcdhb19				
	Plk1	Pcdhb20				
	Plk2	Pcdhb21				
	Plk3	Pcdhb22				
	Plk4	Pcdhga1				
	Plpp2	Pcdhga2				
	Pls1	Pcdhga4				
	Plscr3	Pcdhga7				

	Pmf1	Pclaf			
	Pmm1	Pcmtd2			
	Pnrc1	Pcna			
	Podnl1	Pcolce			
	Pold1	Pdcd4			
	Pold2	Pdf			
	Pole	Pdgfrb			
	Pole2	Pdia4			
	Pole4	Pdk2			
	Polr1b	Pdp2			
	Polr2c	Pelp1			
	Pomgnt2	Per1			
	Pomt2	Pex11a			
	Postn	Pfkm			
	Ppat	Pgam2			
	Ppic	Pgk1			
	Ppif	Pgp			
	Ppm1f	Pgpep1			
	Ppm1g	Pgrmc1			
	Ppp1ca	Phb2			
	Ppp1r13b	Phf19			
	Ppp1r13l	Phf7			
	Ppp1r15a	Phgdh			
	Ppp2r3b	Phlda2			
	Ppp5c	Phlda3			
	Pqlc2	Phldb3			
	Pradc1	Pigq			
	Prc1	Pigy			
	Prdx5	Pik3ip1			
	Prim1	Pink1			
	Prpf19	Pinlyp			
	Prr15	Pitpnb			
	Prrg4	Pitpnm1			
	Psap	Pla2g15			
	Psat1	Plag1			
	Psca	Plaur			
	Psmc3ip	Plbd2			
	Psmc5	Plcd4			
	Psmd7	Plcg1			
	Psph	Pld3			
	Ptgs1	Plec			
	Ptgs2	Plekhb2			
	Ptma	Plekhg5			
	Ptp4a2	Plekhj1			
	Ptpn23	Plk1			
	Ptprf	Plk2			
	Pttg1	Plk4			
	Pus3	Plod2			
	Pwwp3a	Plp2			
	Pycard	Pmf1			
	RF00024	Pnpo			
	RF00072	Pnrc1			
	RF00158	Pold1			
	RF00264	Pold2			
	RF00322	Pole			
	RF00324	Polg			
	RF00377	Pomgnt1			
	RF00405	Pomk			
	RF00409	Ppat			
	RF00421	Ppcs			
	RF00492	Ppl			
	RF00553	Ppm1g			
	RF00582	Ppp1r3c			
	RF00586	Ppp1r9b			
	RGD1308117	Ppt1			
	RGD1309350	Pqlc2			
	RGD1309748	Praf2			
	RGD1359290	Prc1			
	RGD1560010	Prim1			
	RGD1560108	Prkar2a			
	RGD1561149	Prorsd1			
	RGD1561671	Prr11			
	RGD1562114	Prr3			

	RGD1562378	Prss53				
	RGD1564855	Prune1				
	RGD1565498	Psap				
	RGD1565616	Psat1				
	RT1-A2	Psca				
	RT1-S3	Psenen				
	Rab11fip1	Psma4				
	Rab11fip5	Psmc3ip				
	Rab23	Psmd5				
	Rab3d	Psmg3				
	Racgap1	Ptdss1				
	Rack1	Ptges3				
	Rad1	Ptges3l1				
	Rad21	Ptgs1				
	Rad51	Ptma				
	Rad51ap1	Ptrh1				
	Rad51c	Pts				
	Rad54l	Pttg1				
	Ranbp1	Pura				
	Rangap1	PVR				
	Rap2a	Pxdn				
	Rap2b	Qsox2				
	Rapsn	Rab29				
	Rasd1	Rab3a				
	Rasl11b	Rab42				
	Rassf7	Rac1				
	Rbmx2	Racgap1				
	Rbmxl1b	Rad21				
	Rcc2	Rad51				
	Rdm1	Rad51ap1				
	Reck	Ran				
	Reep3	Ranbp1				
	Reep4	Rangap1				
	Resf1	Rapsn				
	Retreg3	Rasd1				
	Rexo4	Rasl11b				
	Rfc2	Rasl2-9				
	Rfc3	Rbm17				
	Rfc4	Rbm5				
	Rfc5	Rbmx2				
	Rflnb	Rbmxl1b				
	Rgl2	Rcc1				
	Rhob	Rcc2				
	Rhod	Recql4				
	Riok1	Reep3				
	Riok3	Relt				
	Ripor1	Renbp				
	Rnd1	Retreg3				
	Rnd3	RF00087				
	Rnf145	RF00186				
	Rnf166	RF00218				
	Rnf185	RF00263				
	Rnf19a	RF00405				
	Rnf24	RF00421				
	Rnf26	RF00431				
	Rnf44	RF00492				
	Rnps1	RF00581				
	Rogdi	RF00582				
	Rpa2	RF00586				
	Rpa3	Rfc2				
	Rpe	Rfc5				
	Rpl11	RGD1305938				
	Rpl13a	RGD1306441				
	Rpl15	RGD1306502				
	Rpl17	RGD1307929				
	Rpl18	RGD1308117				
	Rpl18a	RGD1309748				
	Rpl22l1	RGD1311946				
	Rpl23	RGD1561102				
	Rpl24	RGD1562136				
	Rpl26	RGD1562378				
	Rpl27	RGD1562690				
	Rpl29	RGD1563941				

	Rpl3	RGD1564171				
	Rpl31	RGD1564804				
	Rpl34	RGD1564836				
	Rpl35	RGD1565784				
	Rpl37a	RGD1566099				
	Rpl39	Rhob				
	Rpl41	Rhod				
	Rpl5	Rif1				
	Rpl6	Rilpl2				
	Rpl7	Ripk3				
	Rpl7l1	Rnaset2				
	Rpl9	Rnd1				
	Rps10l1	Rnd2				
	Rps15	Rnd3				
	Rps17	Rnf10				
	Rps18l1	Rnf166				
	Rps24	Rnf183				
	Rps3	Rnps1				
	Rps3a	Rogdi				
	Rps4x	Rpa2				
	Rps7	Rpa3				
	Rps8	Rpl10l				
	Rras2	Rpp21				
	Rrm1	Rps6ka4				
	Rrm2	Rrm1				
	Rtl3	Rrm2				
	Rtn4	Rrp15				
	Rusc2	RT1-M3-1				
	Rwdd4	RT1-N2				
	Samd9	RT1-S3				
	Sapcd2	Rwdd2a				
	Scarf2	Rxfp3				
	Scpep1	Rxrb				
	Scrn1	Rybp				
	Sdcbp	S100a1				
	Sdf2l1	S100a10				
	Sec22b	S100a11				
	Selenoi	S100a13				
	Sema3b	S100a16				
	Sema3c	Sacs				
	Sema4b	Samd1				
	Sephs2	Samd9				
	Serpinb6a	Sap30				
	Serpinb7	Sapcd2				
	Serpinb8	Scarf2				
	Serpine1	Scrn2				
	Serpine2	Sdc1				
	Sesn2	Sec13				
	Set	Sec14l2				
	Setd6	Sec22b				
	Setd7	Selenop				
	Sf3b3	Selenos				
	Sft2d1	Sema3b				
	Sft2d3	Sephs1				
	Sgms2	Sept11				
	Sgo1	Serf1				
	Sgo2	Serinc2				
	Sgpp1	Serinc4				
	Sh2d4a	Serpinb7				
	Sh3bp2	Serpinb8				
	Sh3bp5l	Serpine1				
	Sh3gl1	Serpini1				
	Sh3glb1	Sesn2				
	Shc1	Sesn3				
	Shcbp1	Set				
	Siglec10	Sf3b3				
	Siva1	Sfpq				
	Ska1	Sft2d3				
	Ska3	Sfxn3				
	Slbp	Sgk1				
	Slc10a5	Sgo1				
	Slc12a4	Sh3gl1				
	Slc16a14	Sh3glb2				

	Slc19a2	Shc1				
	Slc1a5	Shcbp1				
	Slc20a2	Shmt2				
	Slc25a10	Shroom1				
	Slc25a11	Sirt4				
	Slc25a44	Ska1				
	Slc25a5	Ska3				
	Slc27a4	Slbp				
	Slc29a1	Slc10a5				
	Slc29a4	Slc19a2				
	Slc2a5	Slc1a4				
	Slc31a1	Slc1a5				
	Slc35a4	Slc20a1				
	Slc35b1	Slc22a17				
	Slc35d1	Slc22a18				
	Slc35d2	Slc25a2				
	Slc35f5	Slc25a39				
	Slc39a13	Slc25a44				
	Slc39a7	Slc25a5				
	Slc39a9	Slc27a1				
	Slc47a1	Slc30a4				
	Slc4a2	Slc35d2				
	Slc52a3	Slc38a7				
	Slc5a6	Slc39a10				
	Slc6a8	Slc39a9				
	Slc7a5	Slc43a2				
	Slc9a3r2	Slc47a1				
	Slfn2	Slc48a1				
	Smagp	Slc49a3				
	Smc2	Slc52a3				
	Smc4	Slc6a8				
	Snapin	Slc7a5				
	Snf8	Slitrk6				
	Snn	Smagp				
	Snrpa	Smc2				
	Snrpa1	Smc4				
	Snrpb	Smim14				
	Snrpb2	Smim22				
	Snrpd1	Smug1				
	Snrpf	Snapc2				
	Snx18	Snn				
	Snx33	Snrpa				
	Sod3	Snrpa1				
	Sorbs3	Snrpb				
	Sowahc	Snrpf				
	Sp110	Snx32				
	Spag5	Snx33				
	Sparc	Sod3				
	Spc24	Sort1				
	Spc25	Sowahb				
	Spcs2	Sowahc				
	Specc1	Spaca6				
	Sphk1	Spag5				
	Spn	Spag8				
	Spp1	Sparc				
	Spry4	Spata31d1				
	Sptlc2	Spc24				
	Sqstm1	Spc25				
	Src	Spint2				
	Srebf2	Spns1				
	Srsf4	Sprr1a				
	Srsf7	Sqstm1				
	Ssr3	Srpk1				
	Ssrp1	Srsf1				
	St3gal1	Srsf2				
	St6gal1	Srsf3				
	St6galnac1	Srsf7				
	Stard3	Ssr3				
	Stil	Ssrp1				
	Stip1	Ssx2ip				
	Stk40	St3gal1				
	Stmn1	Stc1				
	Stn1	Stil				

Sun1	Stip1
Suv39h1l1	Stmn1
Svbp	Sumo2
Syde1	Sumo4
Synj1	Supt4h1
Synm	Suv39h1l1
Synpo	Sv2a
Sypl1	Svbp
Tacc3	Syde1
Taf12	Syngap1
Taf13	Syt11
Taf7	Sytl1
Tagln	Taar7b
Taldo1	Tacc3
Tap1	Taf1d
Tbc1d17	Tagln2
Tceanc	Tap1
Tcf19	Tbc1d13
Tcn2	Tbc1d17
Tdg	Tcf19
Tead3	Tcirg1
Tead4	Tcn2
Tedc2	Tcp1
Tef	Tcp11l2
Tent4a	Tcta
Tent5a	Tctex1d2
Tent5b	Tent5a
Terf2ip	Terf2ip
Tfdp1	Tex264
Tgfb1	Tex30
Thbs2	Tfdp1
Thoc3	Tfrc
Ticrr	Tgif1
Timeless	Tgoln2
Timp1	Thap12
Timp2	Thap3
Tinagl1	Them6
Tiparp	Thra
Tipinl1	Thyn1
Tjp1	Ticrr
Tk1	Tigd2
Tmbim1	Timeless
Tmem106a	Timp2
Tmem119	Tipinl1
Tmem127	Tk1
Tmem129	Tle2
Tmem132a	Tlr5
Tmem138	Tm4sf1
Tmem185b	Tmed3
Tmem263	Tmem101
Tmem41a	Tmem106c
Tmem59	Tmem109
Tmem63a	Tmem127
Tmem63b	Tmem132a
Tmem69	Tmem138
Tmem8a	Tmem140
Tmem97	Tmem150a
Tmpo	Tmem160
Tnc	Tmem185b
Tnfaip2	Tmem203
Tnfaip8l3	Tmem205
Tnfrsf12a	Tmem208
Tnfrsf1a	Tmem256
Tnip3	Tmem43
Tnnc1	Tmem59
Tnpo1	Tmem80
Tnrc18	Tmem97
Tob1	Tmpo
Tomm40l	Tnfaip8l1
Tonsl	Tnfrsf26
Top2a	Tnip1
Topbp1	Tnnc1
Tor1aip2	Tonsl

	Tp53inp1	Top2a				
	Tp53inp2	Topbp1				
	Tpcn1	Tp53inp1				
	Tpm1	Tpm3				
	Tpp1	Tpp1				
	Tpx2	Tpra1				
	Traf4	Tprn				
	Trafd1	Tpx2				
	Traip	Traf4				
	Trim25	Traip				
	Trim34	Trib2				
	Trim47	Trim34				
	Trip13	Trip13				
	Trip6	Troap				
	Trmt10a	Tsc22d3				
	Trmt112	Tspan3				
	Troap	Tssk3				
	Tsc22d1	Ttc30b				
	Tsc22d2	Ttk				
	Tsc22d3	Ttll13				
	Tsn	Tuba1b				
	Tspan5	Tubb4b				
	Tspo	Tubb5				
	Tssk6	Tubb6				
	Ttf2	Tufm				
	Ttk	Tulp3				
	Tubb5	Tvp23b				
	Tube1	Txnrd1				
	Tubg1	U2af1				
	Tubgcp2	Uap1				
	Tusc2	Uap1l1				
	Tvp23b	Ube2c				
	Txnip	Ube2g2				
	Tyro3	Ube2s				
	U2af1	Ube2t				
	Uba5	Ubiad1				
	Ubald2	Ubqln4				
	Ube2c	Uchl1				
	Ube2s	Uckl1				
	Ube2t	Ufsp1				
	Ubqln2	Ugdh				
	Ubtd1	Ugt1a1				
	Ubxn4	Uhrf1				
	Ugcg	Ung				
	Ugdh	Usp1				
	Ugt1a1	Vamp1				
	Uhrf1	Vamp2				
	Unc119	Vamp3				
	Ung	Vars				
	Upk3b	Vgll4				
	Usp1	Vhl				
	Vat1	Vim				
	Vegfd	Vkorc1				
	Vgll3	Vps11				
	Vps26b	Washc2c				
	Wbp1l	Washc3				
	Wdfy1	Washc5				
	Wdhd1	Wbp1l				
	Wdr5	Wdhd1				
	Wipi1	Wdr1				
	Wls	Wdr45				
	Xdh	Wdr5				
	Xkr5	Wdr81				
	Xrcc2	Wee1				
	Yod1	Wrb				
	Ypel5	Wsb2				
	Zbtb4	Xylb				
	Zbtb8os	Xylt2				
	Zc2hc1a	Yipf2				
	Zcchc3	Yipf3				
	Zdhhc9	Yod1				
	Zfand5	Ypel3				
	Zfp219	Ypel5				

	Zfp266	Ywhaz				
	Zfp292	Zadh2				
	Zfp385a	Zbtb5				
	Zfp512b	Zbtb9				
	Zfp703	Zer1				
	Zfp706	Zfp219				
	Zfp846	Zfp358				
	Zfp964	Zfp467				
	Zfr	Zfp523				
	Zmat2	Zfp579				
	Zmat3	Zfp580				
	Zscan25	Zfp597				
	Zswim4	Zfp688				
	Zwint	Zfp691				
	Zyx	Zmynd10				
	mrpl11	Znf740				
		Zscan25				
		Zwilch				
		Zwint				
		Zyx				
