# Supplementary material for: GRWD1-WDR5-MLL2 Epigenetic Complex Mediates H3K4me3 Mark and Is Essential for Kaposi’s Sarcoma-Associated Herpesvirus-Induced Cellular Transformation
Source: mBio. 2021 Dec 21;12(6):e03431-21. doi: 10.1128/mbio.03431-21 (PMC8689518; doi:10.1128/mbio.03431-21)
Supplement: TABLE S4 [file mbio.03431-21-st004.pdf]

TABLE S4A Top altered genes in MM cells following GRWD1 knockdown

Gene	P-Value	Fold Change (shRNA/WT)
Rasl11b	0.01	1.65
AABR07021384.1	0.02	1.63
Gprin3	0.02	1.36
Tfpi	0.01	1.45
AC125248.1	0.04	1.33
Yod1	0.02	1.39
Slurp1	0.04	1.40
Vamp5	0.03	1.55
Msln	0.01	1.56
Kdm5b	0.00	1.40
Snapin	0.01	1.45
Reck	0.01	1.57
Pigc	0.01	1.31
Ccdc17	0.00	1.40
Tmem175	0.01	1.31
Mir125b1	0.00	1.86
Neurod6	0.05	1.39
Ctsa	0.03	1.43
AABR07035541.3	0.04	1.34
Ddr1	0.01	1.36
AABR07044574.1	0.01	1.70
Pigm	0.00	1.53
C1qtnf5	0.04	1.89
Lrrn4cl	0.04	1.75
B2m	0.01	1.62
Terf2ip	0.04	1.58
Siglec10	0.03	1.72
Zwint	0.04	2.87
Lamb1	0.01	1.43
Cited2	0.03	1.35
Tceanc	0.00	1.36
AC139392.1	0.00	1.72
Gaa	0.02	1.53
Ccn4	0.03	1.81
AY172581.2	0.03	2.01
Nlrp3	0.00	1.55
Glipr1	0.01	1.47
Ifi27	0.02	1.69
Tpp1	0.04	1.31
Micall2	0.04	1.31
Rell2	0.02	1.33
AABR07058534.1	0.01	1.33
Kprp	0.05	1.44
Mapk6	0.01	1.49
Tmem177	0.00	1.33
Slc46a1	0.05	1.36
Dtx3l	0.03	1.39
Ptprv	0.03	1.34
Gnat2	0.03	1.42
Tp53inp1	0.04	2.13
Tob1	0.03	1.67
Cebpd	0.05	1.76

Sema3f	0.03	1.50
Dcakd	0.00	1.32
AABR07053516.1	0.00	1.37
AABR07057302.1	0.04	1.84
Serpine1	0.00	2.91
AC134224.3	0.02	1.80
Ptprn	0.03	1.35
Nudt18	0.04	1.37
Cxcl12	0.04	1.88
Abhd4	0.05	1.35
Cxcl16	0.01	1.85
Tmem80	0.02	1.44
AABR07021596.4	0.04	1.55
AC111734.1	0.01	1.62
Depp1	0.04	1.56
Tbc1d2	0.02	1.32
Arl6ip4	0.01	1.30
Uba7	0.04	1.35
Cd14	0.04	1.35
AABR07044959.1	0.04	1.34
Speg	0.01	1.32
Ginm1	0.04	1.40
RT1-A2	0.02	1.55
Borcs6	0.04	1.37
Ghitm	0.01	1.37
Ormdl2	0.02	1.40
Filip1l	0.01	1.31
Syngr2	0.02	1.53
AABR07034767.1	0.02	1.42
Lig4	0.00	1.58
Lyrm2	0.01	1.31
Fhl1	0.03	1.42
Tmbim1	0.01	1.39
Golga7	0.00	1.31
Akap12	0.04	1.51
Atxn7l3b	0.03	1.60
Grina	0.01	1.51
Angpt4	0.04	1.75
Skil	0.05	1.32
Tmed5	0.00	1.35
AC097153.2	0.01	1.31
Akirin1	0.02	1.32
Nfe2l2	0.02	1.42
Cdkn1a	0.00	2.49
G3bp2	0.02	1.46
Oasl2	0.02	1.40
AABR07032523.1	0.00	3.70
Plekhb2	0.04	1.38
Gpd1	0.02	1.34
AC130391.5	0.00	1.42
Irf7	0.03	3.61
Samd9	0.04	1.57
LOC100294508	0.02	1.56
AY172581.21	0.02	1.89

Parp9	0.04	1.33
Zbtb4	0.05	1.48
Bhlhe41	0.00	1.59
Ppt2	0.00	1.38
Gm2a	0.03	1.35
Dynlt3	0.04	1.60
Itga7	0.01	1.58
Trappc6a	0.02	1.33
Inhbb	0.01	1.68
Maff	0.03	1.53
Hif1an	0.04	1.53
Hist1h1d	0.01	1.67
Tmem101	0.00	1.36
Cald1	0.04	1.58
Bid	0.02	1.43
Zswim4	0.02	1.34
Slc35f5	0.03	1.31
Entpd4	0.00	1.33
Adamts1	0.03	1.59
Tapbpl	0.04	1.37
Mir27a	0.02	2.79
Ifi30	0.03	1.55
Mirlet7i	0.02	2.43
Serpinb9	0.02	1.51
Gfra4	0.02	1.33
Tmtc3	0.01	1.41
Glb1l	0.04	1.38
Clu	0.02	1.57
Mdm2	0.00	1.35
Lpar6	0.01	1.71
P2rx4	0.03	1.66
Apol9a	0.04	1.53
Cryba4	0.05	1.38
Tgfb2	0.01	1.91
PVR	0.04	1.96
Bloc1s3	0.03	1.33
Il1rl1	0.01	1.56
AABR07072853.1	0.01	2.25
Fez2	0.01	1.30
Vegfa	0.01	1.47
Mir145	0.03	3.10
Ier3	0.00	1.82
Tmem150a	0.01	1.69
Hbegf	0.01	1.52
AC118439.1	0.05	1.48
Sgms2	0.01	1.33
AABR07030162.1	0.05	1.41
Gmip	0.04	1.39
Cd200	0.00	1.50
Mir339	0.03	1.32
Gdf15	0.02	1.91
Nabp1	0.00	1.65
Pcdhga7	0.05	1.36
Wnk1	0.02	1.35

Bag2	0.02	1.30
AABR07062477.2	0.04	1.40
Angptl4	0.04	1.41
AC130555.1	0.02	1.96
Tusc2	0.00	1.79
Sft2d3	0.00	1.61
RGD1562378	0.04	1.72
H6pd	0.05	1.32
Slc16a4	0.00	1.67
Hist1h2bk	0.03	2.03
Tmem205	0.02	1.38
Prss23	0.02	1.56
Mir22	0.01	2.00
Mir21	0.02	3.28
Fstl1	0.01	1.44
AABR07072916.1	0.03	1.41
AABR07038926.1	0.01	1.50
Aen	0.00	1.38
Klhl24	0.03	1.51
Cd63	0.02	1.34
Ankrd1	0.01	1.78
Trim25	0.01	1.48
AABR07027753.3	0.01	2.51
AABR07069433.1	0.03	1.32
Cnpy4	0.00	1.34
Clec2d2	0.01	1.48
Magt1	0.04	1.40
Gadd45a	0.04	1.62
LOC691807	0.00	1.49
AC119015.4	0.01	1.68
Slc20a2	0.01	1.60
AABR07026302.1	0.04	1.61
B3gnt7	0.05	1.38
Ap5b1	0.02	1.47
Pcdh1	0.03	1.39
Zfp513	0.03	1.41
Ankrd49	0.03	1.37
Nit1	0.00	1.30
AABR07010705.1	0.01	1.33
Pnpo	0.01	1.34
Icam1	0.05	1.92
Blcap	0.03	1.64
Hacd1	0.00	1.52
Dbn1	0.04	1.41
Cadm4	0.04	1.56
Dusp1	0.04	1.42
Tmem230	0.00	1.37
Spns1	0.03	1.53
Slc38a7	0.01	1.78
Adamtsl4	0.05	1.56
Tsc22d1	0.01	1.34
Sema4b	0.02	1.33
Chpf2	0.03	1.40
Necap1	0.03	1.46

Cdc34	0.04	1.48
Tpm1	0.02	2.10
Trim34	0.01	1.50
AABR07044273.1	0.03	1.31
Rnd1	0.01	2.24
Shroom1	0.03	1.38
Irgc	0.00	1.34
Ilvbl	0.01	1.30
Btn2a2	0.04	1.36
Irgm	0.04	1.31
RT1-CE10	0.00	1.45
Dnpep	0.02	1.33
Fam91a1	0.00	1.33
Plk3	0.01	1.74
Plk2	0.02	2.25
LOC100909474	0.02	1.49
AC106191.1	0.04	1.46
Lgals3bp	0.00	2.34
Zcchc3	0.02	1.94
Adrb2	0.03	1.42
Adrb3	0.01	1.64
Fst	0.00	1.80
Fmod	0.02	1.61
Isg15	0.04	2.54
Ier5	0.00	1.48
AABR07068852.1	0.00	1.84
Cd48	0.00	1.42
F2r	0.02	1.49
Ccdc47	0.03	1.47
Cd44	0.04	1.32
Fam214b	0.00	1.73
Tinagl1	0.01	1.56
Rnf181	0.03	1.30
Rras	0.01	1.53
Sox9	0.02	1.34
Tiparp	0.00	1.38
Hapln3	0.01	1.59
Bst2	0.03	1.99
LOC100365363	0.05	1.34
Etfrf1	0.01	1.71
Rnaseh1	0.00	0.76
Rbm3	0.00	0.68
Gas2l3	0.00	0.49
Cep57	0.00	0.75
Cep55	0.00	0.51
Pmvk	0.00	0.69
Ccnf	0.02	0.58
AABR07068316.1	0.03	0.73
Asf1b	0.00	0.38
Cyren	0.03	0.75
Siva1	0.01	0.49
Ccdc34	0.00	0.72
Ska1	0.00	0.53
Ska3	0.00	0.51

AABR07064634.1	0.01	0.76
Cactin	0.04	0.77
Ilf2	0.01	0.76
AABR07062599.1	0.01	0.71
Nap1l1	0.03	0.67
Tacc3	0.00	0.45
Cks1b	0.04	0.58
Ncapd2	0.03	0.47
Ncapd3	0.00	0.68
Dctpp1	0.01	0.73
Rrm2	0.00	0.43
Rrm1	0.00	0.43
Cdk20	0.01	0.71
Cebpg	0.01	0.72
AABR07026311.1	0.00	0.47
AC112350.1	0.00	0.55
Slfn13	0.00	0.56
LOC102546716	0.01	0.62
Sf3a3	0.01	0.77
Ell3	0.01	0.70
AABR07044366.1	0.01	0.57
Pitpnb	0.04	0.72
Ccnb1	0.00	0.39
Pim1	0.03	0.71
Srsf4	0.00	0.76
Sltm	0.01	0.76
Zcchc24	0.00	0.69
AABR07029613.1	0.01	0.72
Nde1	0.01	0.69
AABR07058514.3	0.01	0.60
Brca1	0.00	0.64
LOC301444	0.04	0.77
Mad2l1	0.00	0.48
Snrpd1	0.01	0.67
Ubr7	0.01	0.66
Dnajb1	0.01	0.69
Rad54l	0.00	0.64
Pif1	0.05	0.60
Baz1b	0.00	0.76
Mme	0.01	0.74
Ndrg1	0.03	0.74
Xrcc2	0.00	0.63
Rbm15b	0.00	0.62
Spc25	0.00	0.49
Spc24	0.00	0.52
Lrrc14	0.01	0.75
Cep250	0.00	0.64
Jpt2	0.00	0.76
Tmpo	0.01	0.55
Shcbp1	0.00	0.57
Pole4	0.02	0.65
Pole3	0.01	0.65
Pole2	0.00	0.68
Tubgcp2	0.00	0.64

RF00282	0.05	0.64
Apold1	0.03	0.38
AABR07019399.1	0.00	0.66
Ints7	0.03	0.76
Mbtps1	0.03	0.74
Tshz1	0.00	0.65
Gng10	0.02	0.68
Parp1	0.01	0.75
Parp2	0.00	0.73
Ttk	0.00	0.51
Arhgap11a	0.00	0.46
Tedc2	0.00	0.59
Bora	0.02	0.67
Akr1c14	0.00	0.69
Chst12	0.04	0.70
Clspn	0.03	0.72
Stub1	0.01	0.69
AC127920.1	0.02	0.70
RGD1308134	0.04	0.71
Tyms	0.00	0.65
AABR07044711.1	0.00	0.59
Mrpl14	0.02	0.66
Bloc1s2	0.03	0.71
Mrpl18	0.01	0.68
Psmd3	0.00	0.77
AABR07028352.1	0.00	0.50
Pnn	0.01	0.74
AABR07033745.1	0.00	0.66
Lmna	0.01	0.75
AABR07065113.1	0.04	0.76
Zfand2b	0.03	0.74
E2f7	0.00	0.73
Gins3	0.01	0.58
Nasp	0.00	0.54
Gins1	0.00	0.67
E2f2	0.00	0.74
Gins4	0.01	0.64
Dtl	0.01	0.66
Sf3b3	0.02	0.70
E2f8	0.00	0.51
Fancd2	0.00	0.57
Pttg1	0.00	0.52
Rdm1	0.02	0.73
Anln	0.01	0.74
AABR07027744.1	0.04	0.74
Fbxo5	0.04	0.44
Rfc5	0.00	0.52
Rfc4	0.01	0.58
Rfc3	0.00	0.61
Rfc2	0.00	0.56
Phf5a	0.02	0.70
Lrrc75a	0.01	0.74
RF00575	0.04	0.73
Ssr1	0.00	0.68

Hirip3	0.00	0.50
Gm23880	0.01	0.59
Tex30	0.00	0.72
Pimreg	0.05	0.48
Klhdc4	0.03	0.71
Gpsm2	0.00	0.65
Topbp1	0.00	0.66
Pygo2	0.02	0.72
AABR07049353.1	0.00	0.77
Birc5	0.00	0.40
Alyref	0.00	0.51
Sf1	0.02	0.73
Rabggtb	0.01	0.67
Vps4a	0.01	0.69
Trim47	0.01	0.57
Sapcd2	0.00	0.45
AABR07060293.1	0.02	0.38
Rif1	0.01	0.75
Oip5	0.04	0.72
Nup107	0.00	0.62
Usp1	0.01	0.47
Cit	0.01	0.72
Msh6	0.01	0.69
AABR07013288.4	0.00	0.65
Melk	0.00	0.66
Fn3krp	0.03	0.77
Nans	0.00	0.61
Top2a	0.00	0.37
Bend6	0.02	0.74
Ppp1r3c	0.02	0.73
Hat1	0.00	0.63
Nuf2	0.00	0.52
Hyls1	0.00	0.60
Rad51ap1	0.00	0.52
Nfya	0.00	0.77
Stard7	0.05	0.72
Exosc8	0.00	0.64
Zfp367	0.01	0.66
Polr2c	0.00	0.76
Exosc2	0.03	0.73
Lrrcc1	0.00	0.76
Prc1	0.00	0.43
Fignl1	0.00	0.63
Ing1	0.02	0.69
RF00302	0.02	0.67
Hmmr	0.00	0.52
Toe1	0.00	0.75
AC124839.1	0.04	0.74
Pole	0.00	0.51
Lin54	0.00	0.71
Aspm	0.00	0.49
Mdc1	0.00	0.55
Retreg2	0.01	0.76
Plekha1	0.00	0.71

Vrk1	0.00	0.72
Kif20b	0.00	0.55
Pa2g4	0.00	0.68
Cdc6	0.00	0.50
Cdc7	0.00	0.66
Cip2a	0.00	0.59
Becn1	0.04	0.64
Ccsap	0.00	0.77
Ercc6l	0.00	0.55
Arf5	0.00	0.65
Eri1	0.02	0.64
Prpf8	0.02	0.75
Prr11	0.00	0.65
Aurka	0.01	0.42
Aurkb	0.00	0.43
Fkbp1a	0.04	0.70
Slc25a16	0.01	0.77
Syne3	0.02	0.76
Tcf19	0.00	0.39
Prpf4	0.04	0.76
Donson	0.01	0.71
Sf3a2	0.04	0.73
Nono	0.01	0.51
Ung	0.00	0.59
Rfwd3	0.00	0.56
Fitm2	0.01	0.74
Ssrp1	0.01	0.73
Pask	0.00	0.66
Depdc1	0.00	0.68
Dtymk	0.00	0.60
Rtl8a	0.00	0.71
Smc2	0.00	0.49
Gmnn	0.00	0.47
Chtf8	0.02	0.69
Tesk1	0.02	0.59
LOC100363502	0.04	0.71
Pkm	0.01	0.68
Mki67	0.00	0.32
Stil	0.00	0.58
Mcm10	0.00	0.44
Haus8	0.03	0.75
Scd2	0.01	0.54
Haus4	0.01	0.56
Haus5	0.01	0.75
Haus3	0.04	0.75
Gen1	0.00	0.61
AABR07012129.1	0.04	0.72
Pold2	0.00	0.66
Pold3	0.01	0.75
Dut	0.00	0.56
Slbp	0.00	0.58
Ctdnep1	0.03	0.72
Nrm	0.01	0.63
Bard1	0.00	0.67

Pradc1	0.00	0.60	
AABR07049038.1	0.02	0.75	
Emc8	0.03	0.76	
Sfxn1	0.01	0.76	
Aplp2	0.00	0.72	
Wdhd1	0.00	0.55	
Mettl14	0.00	0.71	
Ddias	0.00	0.70	
Lmf2	0.01	0.62	
RF00342	0.02	0.67	
Fus	0.03	0.73	
Rarg	0.02	0.70	
Cerk	0.03	0.73	
Kif4a	0.00	0.63	
NEWGENE_619861	0.00	0.73	
Pdp2	0.04	0.74	
RF00586	0.04	0.68	
AC129365.1	0.01	0.38	
Set	0.01	0.67	
LOC361346	0.00	0.67	
Sc5d	0.03	0.69	
U2af1	0.00	0.72	
Cdc26	0.03	0.72	
Lix1l	0.01	0.62	
Cdc20	0.02	0.38	
Zwilch	0.00	0.65	
Paip2b	0.01	0.74	
AABR07066944.1	0.03	0.66	
Fmo3	0.00	0.72	
Slc16a1	0.04	0.76	
H2afx	0.04	0.64	
AABR07007134.1	0.04	0.73	
H2afz	0.01	0.47	
Mcm7	0.00	0.42	
Mcm6	0.00	0.47	
Mcm5	0.00	0.42	
Mcm4	0.00	0.44	
Mcm3	0.00	0.45	
Mcm2	0.00	0.40	
Capn1	0.00	0.75	
Fadd	0.00	0.62	
AABR07049578.1	0.01	0.50	
Fam168b	0.00	0.62	
Ugdh	0.00	0.66	
RGD1560010	0.00	0.44	
AABR07061902.1	0.03	0.63	
Nucks1	0.00	0.73	
AABR07015180.1	0.03	0.64	
Rad51c	0.00	0.73	
Plk1	0.02	0.42	
LOC100362999	0.04	0.71	
Nt5c3b	0.00	0.75	
Brca2	0.00	0.67	
RF00088	0.01	0.52	

Tspan17	0.03	0.55
Rasl2-9	0.00	0.67
Mgme1	0.00	0.72
Rap1a	0.03	0.58
AABR07002564.1	0.01	0.75
Dlgap5	0.00	0.55
Ppp2r5d	0.00	0.67
Stip1	0.00	0.76
AABR07011697.1	0.00	0.66
Ube2s	0.04	0.65
Alg8	0.00	0.72
Ticrr	0.00	0.67
Spdl1	0.02	0.69
Cdt1	0.00	0.44
Cyp51	0.03	0.66
Paxip1	0.00	0.67
Prim1	0.00	0.49
LOC100359600	0.00	0.48
Anapc2	0.02	0.68
Prdm8	0.00	0.62
Tfdp1	0.00	0.66
Card19	0.03	0.72
Pclaf	0.00	0.43
Nmral1	0.00	0.72
Nup35	0.00	0.65
Ostc	0.01	0.70
Tmem97	0.02	0.69
Trim59	0.00	0.69
Mybl2	0.00	0.46
Ckap2l	0.00	0.54
AABR07034362.1	0.01	0.64
Pank3	0.00	0.66
Bub1	0.00	0.48
Bub3	0.00	0.70
Dsn1	0.00	0.58
Ubac1	0.00	0.73
Kif14	0.01	0.75
Kif15	0.00	0.71
Psma1	0.00	0.76
Kif11	0.00	0.50
AABR07026797.1	0.00	0.68
Nusap1	0.00	0.52
Pfas	0.04	0.66
Pcna	0.00	0.41
AC132752.2	0.01	0.62
Hjurp	0.00	0.42
Cdc25b	0.02	0.42
Tubb5	0.02	0.67
Nup188	0.01	0.73
Rps6kb1	0.00	0.68
Nbl1	0.01	0.73
Mid1ip1	0.00	0.62
Mrpl51	0.01	0.66
Kcnj2	0.01	0.56

Uhrf1	0.00	0.38
Lsm8	0.04	0.70
AABR07005838.1	0.00	0.54
Rac1	0.04	0.76
Lsm5	0.01	0.72
Lsm3	0.00	0.54
LOC100911361	0.00	0.65
Guk1	0.02	0.67
Lgals3	0.01	0.63
Cbx6	0.00	0.69
Zfp395	0.00	0.74
Cbx3	0.00	0.67
Tpx2	0.00	0.48
Dck	0.00	0.77
Pmf1	0.00	0.63
Bub1b	0.00	0.54
Qdpr	0.00	0.67
Sdc3	0.00	0.75
Gemin6	0.00	0.70
Gng2	0.00	0.68
Pycr2	0.00	0.59
AABR07049223.1	0.01	0.71
AC123213.1	0.01	0.51
Lmnb2	0.01	0.56
Lmnb1	0.00	0.53
AABR07034263.1	0.01	0.74
G2e3	0.00	0.60
Timeless	0.03	0.68
Rbl1	0.00	0.68
Kntc1	0.00	0.50
Nup85	0.01	0.56
Phf19	0.05	0.72
Hnrnpul2	0.00	0.60
AABR07068127.1	0.01	0.55
Hmgn3	0.00	0.67
Hmgn2	0.00	0.53
Arpin	0.00	0.71
Fkbp4	0.04	0.76
Cdk1	0.00	0.45
Cdk2	0.00	0.49
Rpia	0.01	0.75
Trip13	0.00	0.76
Kifc1	0.00	0.43
Misp3	0.00	0.74
Rpp21	0.00	0.63
Ezh2	0.00	0.64
Mdm1	0.00	0.69
Mtmr4	0.02	0.75
Tra2b	0.00	0.74
Smad4	0.03	0.71
Traip	0.01	0.65
Kif18b	0.01	0.55
Snrnp25	0.00	0.69
Foxm1	0.04	0.42

Hnrnpul1	0.01	0.75
Vdac3	0.02	0.59
Vdac2	0.01	0.74
Ctdsp2	0.04	0.69
Fam83d	0.04	0.55
Cchcr1	0.01	0.64
AC115273.1	0.00	0.53
Tk1	0.00	0.41
LOC684762	0.01	0.68
Mad2l1bp	0.03	0.75
Ctdspl	0.00	0.73
Ncaph2	0.00	0.52
Cebpzos	0.00	0.72
Ptpa	0.02	0.70
Grk6	0.01	0.71
Cggbp1	0.03	0.71
LOC102553386	0.00	0.68
Chaf1a	0.00	0.54
Arl5a	0.01	0.74
Htra1	0.00	0.70
Exo1	0.01	0.72
Casp2	0.00	0.76
Orc6	0.00	0.60
Cks2	0.00	0.46
Ube2c	0.01	0.39
Orc1	0.01	0.71
Cdca2	0.00	0.63
Cdca3	0.00	0.36
Igfbp6	0.00	0.76
Cdca7	0.00	0.59
Cdca4	0.02	0.72
Cenpk	0.05	0.76
Cdca8	0.00	0.54
Suv39h1l1	0.02	0.60
Il17rc	0.00	0.68
Ube2t	0.00	0.54
Npm3	0.04	0.60
Rnaseh2a	0.01	0.71
Rnaseh2c	0.01	0.71
Fkbp5	0.00	0.76
Tmem81	0.00	0.73
Tuba1b	0.01	0.61
Cep76	0.00	0.76
Kif23	0.00	0.55
Kif22	0.02	0.43
Stom	0.00	0.76
Anp32e	0.01	0.53
Anp32b	0.00	0.59
Knl1	0.00	0.74
Thoc7	0.04	0.75
Slc25a10	0.00	0.52
Carhsp1	0.02	0.61
Nsmce4a	0.00	0.65
AABR07038948.2	0.00	0.69

LOC100911252	0.00	0.70	
Fen1	0.00	0.51	
Fam136a	0.00	0.70	
AABR07043951.1	0.02	0.75	
Myh10	0.04	0.64	
Hmgb2	0.00	0.54	
Cep295	0.00	0.57	
Aida	0.03	0.68	
Rbbp7	0.00	0.73	
Nxt1	0.01	0.58	
Tedc1	0.01	0.51	
Cdc45	0.00	0.55	
Dlx1	0.00	0.75	
Kif2c	0.00	0.49	
AABR07037203.1	0.00	0.74	
Ncapg2	0.00	0.55	
Ahctf1	0.00	0.76	
LOC499331	0.00	0.50	
Cenpw	0.00	0.47	
Hnrnpm	0.02	0.72	
Hspe1	0.00	0.70	
Exosc3	0.01	0.68	
Zfp207	0.00	0.75	
Hnrnpd	0.00	0.71	
Cnot6	0.01	0.76	
Ubald2	0.00	0.59	
Mms22l	0.00	0.76	
Pnma1	0.00	0.75	
Sdc4	0.00	0.58	
Tmem138	0.00	0.66	
Cenpo	0.00	0.60	
Smc1a	0.01	0.57	
Cenpm	0.00	0.65	
Cenpl	0.00	0.69	
Ccna2	0.00	0.44	
Cenpi	0.00	0.73	
Cenph	0.00	0.51	
Atad5	0.00	0.71	
Cenpf	0.00	0.46	
Cenpe	0.00	0.49	
Cenpc	0.00	0.76	
Cenpb	0.00	0.60	
Cenpa	0.00	0.72	
Atad2	0.00	0.52	
Ckap2	0.00	0.61	
Cenpu	0.02	0.71	
Pbk	0.00	0.39	
Ckap5	0.00	0.64	
Nhlrc3	0.00	0.70	
Dnajc9	0.00	0.49	
Ptges3	0.01	0.75	
Casp8ap2	0.00	0.67	
Tnfsf12	0.04	0.74	
Ran	0.00	0.69	

Shox2	0.00	0.73
Ppm1g	0.00	0.51
Ranbp1	0.00	0.69
Ptma	0.00	0.65
RF00402	0.04	0.63
AABR07015006.1	0.01	0.66
Idh2	0.01	0.65
Txlna	0.00	0.64
Snrpa	0.00	0.70
Ndc80	0.00	0.52
Snrpe	0.02	0.71
Xkr5	0.00	0.70
Rad21	0.00	0.49
Rpa1	0.00	0.72
Rpa3	0.00	0.54
Rpa2	0.00	0.50
Pcif1	0.02	0.70
Edem1	0.00	0.61
Kdelr2	0.04	0.64
Eme1	0.00	0.57
Ubtf	0.02	0.77
Mrps26	0.04	0.72
Ywhah	0.02	0.65
Mrps25	0.00	0.71
Plk4	0.00	0.48
Rwdd1	0.00	0.66
Bok	0.02	0.56
Zfp422	0.05	0.75
Pagr1	0.01	0.72
Incenp	0.00	0.48
Tipinl1	0.00	0.55
Scd	0.01	0.45
Spag5	0.00	0.50
Ppp1ca	0.04	0.75
Fam107b	0.05	0.71
Ncaph	0.00	0.47
Ncapg	0.01	0.46
Rps4y2	0.00	0.71
Tnfaip8l1	0.00	0.49
Hspa14	0.01	0.70
Esco2	0.00	0.73
Gtse1	0.01	0.65
Ado	0.04	0.71
Arl2bp	0.00	0.28
Dad1	0.00	0.77
Chchd1	0.04	0.72
Ect2	0.01	0.61
Sgo2	0.00	0.59
Sgo1	0.00	0.54
Cycs	0.04	0.74
Racgap1	0.00	0.64
Ncl	0.00	0.70
Ivns1abp	0.01	0.71
Arl4a	0.00	0.67

Knstrn	0.00	0.50
Gclm	0.00	0.76
Diaph1	0.00	0.75
Ccne2	0.01	0.61
Exoc8	0.05	0.68
Ccne1	0.00	0.59
Aldh2	0.03	0.74
AABR07001512.1	0.00	0.39
Nsl1	0.00	0.72
AABR07055919.1	0.00	0.60
AABR07055919.2	0.00	0.56
Dek	0.00	0.50
Ykt6	0.00	0.52
Ahcy	0.00	0.69
Fam111a	0.01	0.52
LOC102556092	0.01	0.58
Ddx11	0.01	0.61
AABR07000658.1	0.00	0.46
Cdkn2c	0.01	0.52
Srsf7	0.00	0.57
Srsf1	0.00	0.69
Srsf3	0.03	0.72
Srsf2	0.05	0.69
Mis18bp1	0.00	0.72
Tonsl	0.01	0.60
Id2	0.00	0.67
Id3	0.04	0.76
Grwd1	0.00	0.46
Espl1	0.02	0.54
Chaf1b	0.00	0.58
Galnt1	0.04	0.66
Tube1	0.00	0.75
Mis18a	0.00	0.55
Smc3	0.00	0.75
AABR07036855.1	0.05	0.75
Mastl	0.00	0.70
Smc4	0.00	0.51
Smc6	0.03	0.68
AABR07025328.1	0.01	0.56
Chtf18	0.02	0.56
Kif20a	0.00	0.47
Necab3	0.04	0.56
Metrn	0.01	0.48
Fmnl3	0.05	0.70
RF00409	0.01	0.66
Dnmt1	0.00	0.56
AABR07049695.2	0.03	0.66
AABR07049695.3	0.00	0.66
Vwa1	0.01	0.74
RT1-N3	0.00	0.73
Stmn1	0.00	0.46
Sf3b2	0.01	0.74
Zw10	0.00	0.63
Dscc1	0.00	0.59

Nemp1	0.04	0.70
Anapc15	0.05	0.69
LOC100359539	0.01	0.44
LOC102547056	0.01	0.59
Fancg	0.01	0.76
Dpp9	0.04	0.71
Fancb	0.00	0.70
Fanca	0.00	0.74
Ufd1	0.00	0.73
Mvb12a	0.04	0.74
Fanci	0.00	0.58
Fbl	0.02	0.69

TABLE S4B Top altered genes in MM cells following WDR5 knockdown

Gene	P-Value	Fold Change (shRNA/WT)
Plekhg2	0.01	1.33
Cyp1b1	0.00	1.65
Rasl11b	0.00	2.37
Nr4a2	0.00	1.53
RF00416	0.00	1.58
Atp13a2	0.01	1.30
Zfp846	0.00	1.38
Foxa1	0.01	1.56
Msln	0.00	1.83
Tob1	0.00	1.36
Rnf185	0.04	1.40
Armcx3	0.01	1.33
Armcx2	0.03	1.39
Hbp1	0.01	1.35
B2m	0.00	1.46
Klf4	0.02	2.08
Klf2	0.01	2.69
Zwint	0.00	2.73
Homer3	0.01	1.43
AABR07005004.1	0.01	1.65
Cnppd1	0.02	1.30
Ccn4	0.00	1.65
AABR07073181.1	0.04	1.35
Asah1	0.00	1.42
Snx5	0.00	1.38
AABR07063829.2	0.01	1.92
Tigar	0.00	1.31
Tinagl1	0.03	1.93
Gys1	0.00	1.52
Zfp219	0.01	1.46
AABR07044421.1	0.00	1.35
Cebpb	0.01	2.29
Cebpd	0.02	2.04
AABR07057250.1	0.00	1.60
Zfp322a	0.00	1.38
Vps11	0.04	1.33
Ptprf	0.01	1.32
Eci1	0.00	1.44
Ipo4	0.00	1.53
Lrrc73	0.01	1.32
Tyro3	0.00	1.50
Fundc2	0.01	1.36
AABR07014836.1	0.01	1.39
Lacc1	0.00	1.43
Syde1	0.00	1.94
Serpinb6a	0.00	1.50
AABR07044959.1	0.01	2.10
Synj1	0.00	1.31
AABR07027753.3	0.05	1.89
AABR07055834.1	0.01	1.42
Dnajb9	0.01	1.43
Trafd1	0.00	1.35

Btg2	0.01	1.76
Fam3a	0.00	1.32
Tmem185b	0.00	1.70
Hoxb6	0.01	1.53
Zswim4	0.00	1.34
RGD1562136	0.04	1.52
Ajuba	0.00	1.80
Nisch	0.00	1.31
Tap1	0.00	1.65
Prkab2	0.00	1.42
AABR07025787.1	0.05	1.61
Tnrc18	0.04	1.57
Ccdc80	0.00	2.06
Cpe	0.00	1.71
Aga	0.00	1.34
Osmr	0.01	1.53
Abca3	0.00	1.33
Zfp612	0.01	1.35
Sorbs3	0.00	1.60
Mxd4	0.00	1.60
Rps27a	0.01	1.37
Pnrc1	0.00	1.54
Lama5	0.00	1.45
Zbtb4	0.00	1.37
AABR07027575.1	0.00	2.39
Fktn	0.01	1.36
B3gat3	0.00	1.46
N4bp1	0.00	1.37
Cxcl1	0.02	1.63
Pus3	0.00	1.47
Mtss1l	0.00	1.54
Mir3064	0.01	2.36
Tent5a	0.00	2.01
Csrnp1	0.02	1.44
Golim4	0.03	1.59
Tex264	0.00	1.33
P2rx4	0.00	2.00
Cryba4	0.01	1.50
Rap2b	0.02	1.41
Etv3	0.00	1.37
Bloc1s4	0.01	1.32
Actg1	0.03	1.32
Ier5	0.01	1.62
Rab11fip5	0.00	1.43
Fut4	0.01	1.57
H3f3b	0.00	1.35
Tead3	0.01	1.40
Chfr	0.00	1.43
Rnf14	0.02	1.35
Cep170b	0.01	1.42
Tnfsf18	0.00	1.52
AC119762.6	0.00	1.40
AC119762.7	0.04	1.43
AABR07065625.2	0.01	2.58

Arhgap21	0.00	1.65
Cfl2	0.00	1.74
Wdfy1	0.01	1.43
Arhgap29	0.00	1.82
Phldb3	0.00	1.50
Inafm1	0.05	1.79
Serpine1	0.03	3.52
F3	0.01	1.50
AABR07030647.1	0.00	1.89
Sh3gl1	0.00	1.30
Creb3	0.00	1.50
AABR07053500.1	0.00	2.48
Gadd45a	0.05	2.63
Spata2	0.02	1.32
AABR07003537.1	0.03	1.46
AABR07024593.2	0.02	1.39
Ccdc126	0.05	1.86
Cavin4	0.00	1.46
Cdhr1	0.02	1.33
AABR07030603.2	0.01	1.61
AABR07030603.1	0.00	1.58
AABR07032751.1	0.00	1.53
Icam1	0.04	1.59
Slfn2	0.00	1.48
Dbn1	0.00	1.89
Kctd21	0.00	1.56
Rnasek	0.05	1.33
Stk40	0.00	1.36
Vat1	0.00	1.53
Sesn2	0.01	3.31
Cd55	0.01	2.73
Tsc22d1	0.00	2.10
Hilpda	0.05	1.48
Sparc	0.00	1.46
Ifi27l2b	0.03	4.12
Bsg	0.02	1.36
Ncam1	0.00	1.44
Prob1	0.00	1.36
AC136867.1	0.01	1.44
Slc12a4	0.00	1.36
Adrb2	0.03	1.71
Uhrf1bp1l	0.03	1.57
Kdm6b	0.02	1.44
Osbpl5	0.00	1.37
Rab32	0.03	1.48
Cfap157	0.03	1.56
Serinc4	0.02	1.93
Sesn3	0.02	2.06
Serinc3	0.00	1.31
Itga11	0.02	1.32
Mzt2b	0.00	1.36
Tmem80	0.00	1.32
Gypc	0.00	1.38
Hapln3	0.00	1.36

Trim41	0.02	1.44
Pcgf2	0.00	1.49
Ano8	0.00	1.66
Ktn1	0.00	1.42
Tpm1	0.03	2.48
LOC100910554	0.04	1.31
Avpi1	0.00	1.46
Snapin	0.00	1.56
AABR07004269.4	0.03	1.36
Anxa3	0.00	2.03
Irf7	0.02	1.44
Irf9	0.00	1.67
Flrt3	0.00	1.54
AC103335.1	0.04	1.47
Atf5	0.02	2.39
AABR07014996.1	0.02	1.40
Lamb2	0.00	1.61
Pusl1	0.00	1.47
Akip1	0.02	1.65
LOC290595	0.04	1.48
Riok3	0.01	1.86
Pgrmc2	0.00	1.38
Pgrmc1	0.00	1.48
Mthfr	0.04	1.40
Nr4a1	0.02	1.40
Bax	0.01	1.35
Arhgap5	0.03	1.37
Cmtm3	0.03	1.89
AABR07027811.2	0.01	2.27
Fuca1	0.02	1.30
LOC108351584	0.04	1.49
AABR07033047.1	0.01	1.62
Sema3c	0.00	1.78
Ddit3	0.05	2.13
Ddit4	0.01	2.49
Pqlc2	0.01	1.39
AABR07053516.1	0.00	1.30
Tp53inp1	0.00	3.47
Arg1	0.01	1.33
Cst3	0.00	1.60
AABR07049405.1	0.02	1.51
Plagl1	0.00	1.75
Abhd4	0.00	1.47
Atp6v1d	0.00	1.58
AC133265.1	0.04	1.47
Rtn4	0.01	1.32
Otx1	0.00	1.32
Htr2b	0.02	1.56
Marcks	0.01	1.78
RGD1309748	0.00	1.34
Tmem87b	0.01	1.33
Jtb	0.02	1.45
Asmtl	0.04	1.31
Dmrt2	0.00	1.31

Ndufb9	0.00	1.32
Bdh2	0.01	1.52
Syngr2	0.04	1.31
Yod1	0.02	1.58
Bak1	0.00	1.48
Mir125b1	0.00	1.72
AC103090.1	0.01	1.43
Phlda3	0.00	1.93
Vasp	0.01	1.61
Hsdl1	0.00	1.47
Phf1	0.01	1.95
Pla2g15	0.04	1.36
Tp53inp2	0.00	1.56
Zfp688	0.01	1.36
Rassf8	0.00	1.39
Hoxa1	0.00	1.46
Pdcd4	0.05	1.40
Pdcd2	0.00	1.58
AC094126.2	0.03	1.93
Tgfbr2	0.00	1.38
AABR07045322.1	0.04	1.59
Sirt6	0.00	1.56
Plpp6	0.01	1.35
Mical1	0.00	1.39
Amdhd2	0.03	1.94
Lamp2	0.01	1.38
Prdx6	0.00	1.49
AABR07062290.1	0.04	1.65
Mafg	0.03	1.31
Impact	0.01	1.89
Piezo1	0.01	1.34
Bicra	0.00	1.36
Ptrh1	0.01	1.55
Pura	0.01	1.80
Purg	0.01	1.49
Glb1l	0.00	1.57
Clu	0.00	1.63
Bin1	0.01	1.33
Apol9a	0.00	1.71
Iars	0.03	1.38
Traf4	0.00	1.36
AABR07012575.1	0.00	1.75
Atp2a3	0.03	1.37
Arpc1a	0.00	1.49
Col4a5	0.00	1.39
Slc9a3r1	0.03	1.42
Mir23a	0.01	3.42
AABR07031675.1	0.04	1.45
Ndel1	0.00	1.40
AABR07064349.1	0.05	1.60
Stbd1	0.01	1.93
Arse	0.04	1.40
Vamp2	0.00	1.54
Sft2d3	0.00	1.59

Vamp8	0.00	1.48
Sphk1	0.03	1.39
Mir615	0.03	1.56
Itm2c	0.00	1.35
Itm2b	0.03	1.31
RGD1563861	0.01	1.30
Taf15	0.00	1.31
Nacc2	0.00	1.42
Svbp	0.00	1.43
LOC108351936	0.01	1.39
LOC102553785	0.00	1.62
Clcn3	0.01	1.32
Clcn7	0.02	1.32
Bahd1	0.01	1.40
AABR07060291.1	0.00	1.65
Slc4a2	0.00	1.36
AABR07044940.1	0.05	1.44
AABR07030911.2	0.01	1.32
Mapre3	0.00	1.40
AC119015.4	0.00	1.63
Slc20a2	0.00	1.64
Aif1l	0.00	1.45
Uckl1	0.00	1.50
Hmox1	0.00	2.08
Slc16a4	0.02	1.48
Cilp	0.02	1.64
Atg12	0.04	1.56
Dusp8	0.00	2.02
Blcap	0.00	2.19
Cyb561d1	0.04	1.30
Dapk3	0.02	1.51
Tmem230	0.00	1.38
Dusp3	0.00	1.40
Dcbld2	0.00	1.58
Rnf166	0.00	1.31
Cnn3	0.01	1.54
Mustn1	0.00	1.38
Mllt11	0.02	1.84
Adam19	0.00	1.60
AABR07002711.1	0.05	1.88
RT1-CE10	0.01	1.46
Slc5a6	0.01	1.40
Cdc42ep2	0.00	1.45
Plk3	0.00	2.13
Plk2	0.00	2.58
AABR07007690.1	0.03	1.59
Fam8a1	0.00	1.30
Ydjc	0.01	1.56
AABR07067600.1	0.00	1.38
Chac1	0.00	2.96
Shkbp1	0.00	1.30
Eif4a2	0.01	1.34
Dnlz	0.03	1.36
Gjb4	0.00	1.44

AABR07058464.1	0.00	1.86
Cc2d1a	0.01	1.40
Pnpla6	0.01	1.33
Cc2d1b	0.00	1.40
Tmem167b	0.03	1.46
AABR07026424.1	0.03	1.33
Kif1c	0.00	1.48
Tdg	0.00	1.89
AC141959.1	0.01	1.32
Reck	0.01	1.33
Lgmn	0.01	1.33
Atp5f1e	0.01	1.31
Ddr1	0.00	1.78
Ptger1	0.00	1.78
Creb3l1	0.01	1.31
Tceanc	0.03	1.47
Slc30a1	0.00	1.36
Midn	0.01	1.34
AC115159.2	0.04	1.85
Gnptg	0.00	1.47
Rell2	0.02	1.49
Fv1	0.01	1.30
AABR07044375.1	0.00	1.98
AC125873.1	0.03	1.72
AABR07030494.1	0.03	1.30
Gask1b	0.03	1.81
Hipk3	0.00	1.36
Tmem160	0.02	1.45
Mafk	0.01	1.38
AABR07013701.1	0.01	1.70
Ppp1r13l	0.02	1.54
AC106663.2	0.02	1.33
Gas6	0.00	1.82
Dbndd2	0.00	1.38
Lonrf2	0.02	1.53
Tbc1d2	0.00	1.37
Dgat2	0.01	1.34
RGD1308117	0.01	1.56
Sqor	0.01	1.61
Fam71f1	0.03	1.31
Fosl2	0.00	1.38
Ghitm	0.04	1.59
AABR07071814.1	0.05	1.58
Mcrip1	0.00	1.47
Lig4	0.00	1.48
AABR07044375.2	0.00	1.51
Pln	0.03	1.97
Atxn7l3b	0.00	1.96
Gata3	0.05	1.31
AABR07035780.4	0.01	1.37
Ube2q1	0.00	1.34
Wls	0.00	1.76
Vps26b	0.00	1.43
Grina	0.02	1.35

Plekhb2	0.00	1.68
AC130391.5	0.00	1.69
Gfpt1	0.02	1.40
Ankrd12	0.00	1.33
AABR07044420.2	0.01	1.64
Zfr2	0.05	1.66
G3bp2	0.00	1.38
Itga7	0.00	1.80
Trappc6a	0.00	1.38
Ctsa	0.02	1.49
Trip10	0.00	1.32
Numbl	0.00	1.65
Fkbp9	0.00	1.33
Nckap5l	0.02	1.33
Nkd2	0.00	1.82
Adamts1	0.03	1.99
Gigyf1	0.05	1.41
AABR07029958.1	0.05	1.53
Kif5b	0.03	1.67
Cryab	0.00	3.74
LOC689039	0.01	1.74
Aktip	0.00	1.42
Ngrn	0.00	1.33
Slc35d2	0.00	1.42
Mdm2	0.00	1.55
Hist1h2bk	0.03	1.67
Sqstm1	0.03	2.66
Pik3cd	0.00	1.57
Rad51ap2	0.00	1.41
Casp12	0.00	1.39
Fam83h	0.00	1.37
Gdf15	0.00	1.94
Edf1	0.04	1.32
Emd	0.05	1.51
Esrra	0.01	1.35
AABR07053152.1	0.01	1.39
Ankrd49	0.00	1.43
Mir6334	0.01	1.88
Ypel5	0.01	1.33
Mk1	0.00	1.41
AABR07038926.1	0.00	1.54
Aen	0.00	1.72
Tmem127	0.00	1.41
AABR07035796.1	0.00	1.53
Mfrp	0.04	1.39
AABR07041778.1	0.00	1.32
Bcat1	0.01	1.48
Ftl1	0.01	1.87
Fgfbp3	0.01	1.33
LOC102549726	0.01	2.05
Reep3	0.00	1.54
LOC691807	0.02	1.40
AABR07032480.1	0.01	1.40
Itga3	0.00	1.32

AABR07010705.1	0.01	1.47
Arrdc4	0.00	1.52
AABR07046628.1	0.00	1.86
Zfp513	0.00	1.33
Nfe2l1	0.00	1.30
Ppif	0.00	1.31
Il17re	0.00	1.76
Map1a	0.04	1.40
Map1b	0.00	1.41
Dusp1	0.00	2.00
AABR07053136.1	0.03	1.34
Dstn	0.05	1.64
Lrfn4	0.03	1.38
Btn2a2	0.00	1.57
Plin3	0.00	1.54
Akap5	0.03	1.33
Akap2	0.03	1.45
Adipor1	0.00	1.32
AABR07040840.1	0.05	1.53
Sptan1	0.00	1.49
Fkbp3	0.00	1.40
Nras	0.00	1.39
Rassf7	0.00	2.15
Fermt2	0.00	1.32
Zcchc3	0.04	1.56
Nabp1	0.00	1.49
Chd3	0.00	1.42
Tmem63b	0.00	1.36
Col11a1	0.00	1.34
Smim14	0.00	1.34
Ccdc47	0.01	1.45
Fam214b	0.00	2.14
Ccdc43	0.00	1.35
Dvl3	0.00	1.52
Tiparp	0.00	1.44
Dync1li2	0.01	1.36
AABR07041627.1	0.00	1.47
LOC102551095	0.00	1.65
Cox6a2	0.02	2.46
Ccdc74a	0.04	1.43
Psap	0.01	1.42
Rnf44	0.01	1.33
RF01161	0.04	1.95
Trip6	0.00	1.59
Slc19a2	0.01	1.40
Tmbim1	0.01	1.75
Rnd1	0.00	1.89
Ubqln2	0.01	1.48
AABR07057423.1	0.00	1.52
Ctsb	0.00	1.57
Arl8a	0.00	1.37
Ctsd	0.04	1.38
Tent5b	0.01	2.74
Dyrk2	0.01	1.34

Tvp23b	0.01	1.35
Siglec10	0.00	2.34
AC141169.2	0.03	1.33
Gipc1	0.02	1.39
Slc46a1	0.01	1.32
Gaa	0.03	1.92
Ptgs2	0.03	1.45
AABR07039210.1	0.00	1.31
Zc2hc1a	0.01	1.37
Mapk1	0.00	1.34
AC105515.1	0.01	1.90
Npepps	0.01	1.38
Cdipt	0.00	1.58
Plscr3	0.00	1.78
Cldnd1	0.00	1.53
AC108572.3	0.00	1.58
Hoxc6	0.01	1.50
Mgat1	0.04	1.54
Hand2	0.01	1.49
Cltb	0.00	1.82
Nagk	0.01	1.34
Fbln5	0.00	1.49
Necap1	0.00	1.39
Dhrs4	0.00	1.35
Rogdi	0.00	1.50
Osr1	0.00	1.49
Ubxn4	0.00	1.36
AC099089.1	0.00	2.46
Ginm1	0.05	1.43
Trim7	0.00	1.43
Borcs6	0.01	1.48
LOC100909474	0.00	1.95
Filip1l	0.00	1.35
Mib2	0.04	1.36
Col5a2	0.01	1.31
Col5a1	0.02	1.49
LOC103691238	0.01	1.31
AABR07063855.1	0.00	1.30
Nexmif	0.02	1.33
Eme2	0.02	1.32
C2cd2	0.02	1.39
Rab11fip1	0.01	1.50
Amotl2	0.01	1.67
AABR07068417.2	0.01	1.79
Lrig1	0.01	1.54
Cdkn1a	0.00	2.85
AC134224.3	0.03	1.92
AC110351.1	0.01	1.49
Thyn1	0.02	1.46
Txnip	0.00	2.52
Gabbr1	0.00	1.73
Nbas	0.01	1.75
Samd9	0.00	1.58
Ccdc9b	0.00	1.97

AABR07030861.1	0.00	1.36
Plekho1	0.00	1.38
Bhlhe41	0.05	1.41
Gadd45b	0.01	1.80
Gadd45g	0.01	2.62
Me1	0.00	1.35
Hist1h1d	0.01	2.30
Alkbh7	0.01	1.49
Klc2	0.00	1.37
Nucb1	0.03	1.30
Hist1h1c	0.00	1.95
Ehbp1l1	0.02	1.40
Nfu1	0.00	1.41
AABR07030019.1	0.02	1.36
AABR07017999.1	0.00	1.79
Vegfa	0.00	2.18
Adm	0.01	1.42
Lpar6	0.00	1.34
Nol3	0.00	1.33
Tmem150a	0.02	2.01
AABR07072853.1	0.04	1.42
Prdx3	0.00	1.34
Mkrn3	0.02	1.35
Atrn	0.00	1.43
Lats2	0.01	1.38
AC123425.1	0.00	1.81
Col4a2	0.00	1.76
Col4a1	0.00	1.55
AABR07068852.1	0.05	1.30
Ifrd1	0.02	2.40
Dpf2	0.01	1.36
Ptpn23	0.00	1.41
P4ha2	0.00	1.44
Nkiras2	0.01	1.44
Sowahc	0.00	1.68
Eif4ebp1	0.02	1.56
Rbm18	0.03	1.31
Ifngr2	0.00	1.55
Tfpi	0.01	1.34
AABR07014756.1	0.05	1.33
LOC100294508	0.00	1.33
Ankrd1	0.03	1.55
Trim25	0.00	1.52
AABR07019341.1	0.01	2.00
Sypl1	0.01	1.43
Sema3b	0.03	1.49
Use1	0.00	1.34
Gpr108	0.00	1.32
Ccl20	0.03	2.01
Egln2	0.00	1.46
AABR07033023.1	0.02	1.32
Unc119	0.01	1.94
Ap5b1	0.01	1.39
Lypla1	0.00	1.30

Rps28	0.03	1.30
Rusc2	0.00	2.01
Mir3568	0.01	1.57
RGD1559896	0.00	1.31
Abo3	0.00	1.32
Slc39a3	0.05	1.31
Mir374b	0.02	1.94
Vgll3	0.02	1.84
Mir3564	0.00	1.43
Dsel	0.00	1.43
Slc38a7	0.00	1.57
Chpf2	0.01	1.32
Kmt5a	0.01	1.41
Rpl19	0.05	1.31
Map2k2	0.00	1.38
Rnd3	0.00	1.58
AABR07062154.1	0.00	1.43
Maf	0.00	1.87
Edn1	0.00	1.74
Myo10	0.04	1.38
AABR07072236.1	0.01	1.34
RF00402	0.05	1.34
Vps9d1	0.00	1.31
AABR07071779.2	0.00	1.36
AABR07043654.1	0.00	1.43
AC119762.5	0.03	1.52
AABR07066529.1	0.01	1.31
Bmp1	0.00	1.38
Anapc16	0.02	1.31
Tmem202	0.05	1.30
Il6st	0.00	1.54
Ppp1r15a	0.01	2.25
Jak2	0.05	1.41
Bcar1	0.01	1.40
LOC100365363	0.04	2.06
Setd7	0.00	1.38
Dpp7	0.03	1.71
Tsn	0.00	0.72
Wdr90	0.00	0.59
AABR07030156.2	0.01	0.56
Rad1	0.00	0.72
Gas2l3	0.00	0.47
Cep55	0.00	0.45
Rbbp7	0.00	0.74
Gpank1	0.00	0.75
AC242953.1	0.03	0.67
Kpna6	0.00	0.66
Ccnf	0.00	0.50
Kpna2	0.00	0.67
Pttg1	0.00	0.45
AABR07068316.1	0.04	0.73
Psip1	0.01	0.74
Asf1b	0.00	0.36
Ticrr	0.00	0.63

AABR07066516.1	0.00	0.59
Siva1	0.01	0.56
Ska1	0.00	0.49
Ska3	0.00	0.54
Gtf3c4	0.00	0.74
AABR07057683.1	0.00	0.71
Ilf2	0.01	0.73
Olfml3	0.00	0.54
AABR07062599.1	0.01	0.69
Ppp5c	0.00	0.72
Tacc3	0.00	0.45
Cstf1	0.00	0.75
AABR07028779.1	0.00	0.64
Ncapd2	0.00	0.45
Ncapd3	0.00	0.69
AABR07034573.1	0.05	0.56
Akt1	0.01	0.73
Aunip	0.00	0.63
Dctpp1	0.00	0.58
Rrm2	0.00	0.46
Rrm1	0.00	0.41
Cit	0.00	0.77
Cops8	0.00	0.76
AABR07026311.1	0.00	0.47
Unc5b	0.04	0.75
AC112350.1	0.02	0.52
Ptgfrn	0.01	0.76
Tnip3	0.01	0.75
AABR07040892.1	0.02	0.70
LOC102546716	0.00	0.66
Sf3a3	0.00	0.70
AABR07044366.1	0.00	0.51
Tspan7	0.00	0.74
Oxnad1	0.00	0.77
Ccnb1	0.00	0.37
Dpysl2	0.00	0.61
Pradc1	0.00	0.70
Palb2	0.00	0.67
Tmem139	0.01	0.64
Arl6ip1	0.00	0.52
AABR07047089.1	0.00	0.55
Slc35b2	0.00	0.76
Sema7a	0.04	0.76
Tmed2	0.00	0.71
Brca1	0.00	0.59
Cntf	0.00	0.67
Brca2	0.00	0.64
Tmed1	0.00	0.68
AABR07047219.1	0.00	0.66
Nrn1	0.01	0.73
Rad54l	0.00	0.59
Pif1	0.01	0.59
Pcdhgc3	0.03	0.74
Exd2	0.00	0.69

Mmd	0.04	0.74
Xrcc2	0.00	0.55
Spc25	0.00	0.48
Spc24	0.03	0.52
Pop4	0.00	0.69
Trmt12	0.02	0.76
Adcy7	0.00	0.73
Srrm1	0.00	0.72
Cep250	0.00	0.69
Shcbp1	0.00	0.57
Pole3	0.00	0.59
Pole2	0.00	0.68
Tubgcp2	0.00	0.59
Apold1	0.01	0.49
Tubgcp6	0.00	0.76
Lipa	0.00	0.72
Srpra	0.00	0.75
LOC100362400	0.02	0.74
Haspin	0.00	0.49
Hsp90b1	0.00	0.59
AABR07061614.1	0.00	0.69
Tmem109	0.02	0.69
Arhgap11a	0.00	0.49
Pelp1	0.00	0.63
Tmem107	0.00	0.74
Tedc2	0.00	0.56
Bora	0.00	0.66
AABR07053707.1	0.00	0.70
Tnfrsf11b	0.04	0.74
Setsip	0.05	0.70
Clspn	0.00	0.69
AABR07007758.2	0.01	0.63
Ung	0.00	0.59
Ramac	0.00	0.71
RF00594	0.03	0.62
Bccip	0.01	0.73
Mre11a	0.00	0.68
Twsg1	0.00	0.70
Ccp110	0.00	0.73
Gchfr	0.02	0.72
Rfc4	0.00	0.60
AABR07039037.1	0.03	0.74
Etv5	0.00	0.75
Mrpl18	0.00	0.76
Smc2	0.00	0.49
Orc6	0.00	0.58
AABR07028352.1	0.00	0.58
Ifrd2	0.02	0.76
Nptxr	0.02	0.68
Fh	0.00	0.76
Phyh	0.00	0.74
Gins2	0.00	0.66
Gins3	0.00	0.62
E2f5	0.00	0.77

Gins1	0.00	0.65
Rnf10	0.02	0.69
E2f2	0.00	0.70
Gins4	0.00	0.63
Dtl	0.00	0.58
Prpf19	0.01	0.62
Eif3d	0.00	0.61
Sf3b3	0.00	0.68
E2f8	0.00	0.52
Atp8b2	0.00	0.76
Fancd2	0.00	0.54
Lamtor5	0.01	0.65
Orc1	0.00	0.61
S100a1	0.00	0.71
Timm21	0.00	0.64
Crip2	0.04	0.73
AABR07063279.1	0.00	0.68
Itpripl1	0.00	0.72
Rdm1	0.00	0.74
Ogfrl1	0.00	0.71
Dhfr	0.00	0.70
Mt1m	0.00	0.72
Fbxo2	0.03	0.74
Fbxo5	0.00	0.40
Aph1a	0.00	0.75
Spag5	0.00	0.47
Tomm40l	0.00	0.66
Recql4	0.00	0.56
Rfc5	0.00	0.51
Ak2	0.00	0.75
Rfc3	0.03	0.76
Rfc2	0.00	0.51
Sumf1	0.00	0.72
Phf5a	0.00	0.77
Ppie	0.00	0.73
Pcyox1	0.00	0.58
Mrps5	0.00	0.77
Selenoi	0.00	0.74
Elk3	0.01	0.69
Herc3	0.04	0.65
Rab3d	0.01	0.73
Emp3	0.00	0.73
Dclre1b	0.00	0.70
Hirip3	0.00	0.46
Rab8a	0.00	0.68
Dbnl	0.00	0.77
Rps15	0.01	0.64
Tex30	0.00	0.72
Pimreg	0.00	0.42
Cks1b	0.00	0.54
Ccz1b	0.00	0.75
Zbtb8os	0.00	0.63
Gpsm2	0.00	0.66
RGD1564855	0.00	0.69

Rpl37a	0.00	0.72
Slfn13	0.00	0.49
Rab31	0.00	0.72
Nup107	0.00	0.64
Birc5	0.00	0.44
Tlr3	0.00	0.76
AABR07018244.2	0.01	0.59
Alyref	0.00	0.64
Nup43	0.00	0.73
Slc29a1	0.03	0.66
Trim47	0.00	0.68
F2rl1	0.00	0.70
Sapcd2	0.02	0.51
Znrd1	0.00	0.75
RGD1305350	0.02	0.71
Oip5	0.00	0.68
Mki67	0.00	0.41
Usp1	0.00	0.51
Msh2	0.00	0.70
Stil	0.00	0.58
Msh6	0.01	0.73
AABR07013288.4	0.00	0.65
Melk	0.00	0.72
Mis12	0.02	0.63
Nans	0.00	0.70
Ppat	0.00	0.69
Top2a	0.00	0.43
Anxa2	0.03	0.66
Tnfaip8l1	0.00	0.48
AABR07034315.1	0.04	0.55
Pars2	0.01	0.75
Hat1	0.00	0.68
Rpl34	0.00	0.69
Nuf2	0.00	0.50
Rangap1	0.00	0.61
Hyls1	0.00	0.47
Rpl31	0.04	0.45
Rad51ap1	0.00	0.48
Ssr3	0.00	0.70
Spp1	0.00	0.49
Pfas	0.00	0.68
Emg1	0.00	0.64
Smarca5	0.00	0.73
Zbtb12	0.00	0.73
Knl1	0.00	0.76
Rexo4	0.00	0.64
Thbs2	0.00	0.44
Polr2e	0.00	0.70
Exosc8	0.00	0.59
Polr2c	0.00	0.72
Exosc2	0.00	0.53
Lrrcc1	0.00	0.73
Prc1	0.00	0.45
Fignl1	0.00	0.54

Setx	0.00	0.75
Ing1	0.00	0.60
Polr2k	0.00	0.77
Hmmr	0.00	0.51
Toe1	0.02	0.76
Col8a1	0.03	0.77
Zdhhc6	0.00	0.70
Ran	0.00	0.75
Enpp1	0.00	0.74
Tubb6	0.01	0.62
Pole	0.00	0.51
Polm	0.01	0.76
Lin54	0.00	0.75
AABR07059925.1	0.00	0.76
Cpsf2	0.00	0.69
Wdr5	0.00	0.36
Ccdc163	0.00	0.75
Aspm	0.00	0.51
Mdc1	0.00	0.53
Nup93	0.00	0.76
Eno3	0.01	0.68
Rpl7l1	0.00	0.69
Plekha3	0.00	0.65
Rps17	0.00	0.52
Oaz1	0.02	0.75
Kif20b	0.00	0.53
Pa2g4	0.00	0.76
Cdc6	0.00	0.49
Cdc7	0.00	0.67
Ankle1	0.00	0.55
Cip2a	0.00	0.61
Becn1	0.01	0.75
Naa25	0.00	0.77
RGD1562690	0.01	0.76
LOC497940	0.02	0.72
Ercc6l	0.00	0.46
Eri2	0.00	0.70
Ndc1	0.00	0.71
Eri1	0.00	0.74
Mbd4	0.04	0.75
AABR07052430.1	0.00	0.56
Aurka	0.00	0.49
Aurkb	0.00	0.43
Prr15	0.01	0.71
AABR07072809.1	0.02	0.66
Tcf19	0.00	0.47
Prpf4	0.00	0.69
Slc25a11	0.02	0.75
Slc25a10	0.00	0.44
Kazald1	0.02	0.38
Shc1	0.00	0.73
Cspg4	0.05	0.72
Nup155	0.00	0.71
Nono	0.00	0.66

Tfrc	0.00	0.61
AC094643.2	0.00	0.66
Kif18a	0.00	0.76
Rps18l1	0.00	0.52
Tor4a	0.01	0.75
Reep4	0.00	0.68
AABR07036498.1	0.04	0.66
Dbi	0.03	0.74
Synm	0.01	0.72
Ppp1ca	0.00	0.53
Ssrp1	0.00	0.45
AABR07038269.1	0.00	0.58
Clic1	0.00	0.73
Cct7	0.00	0.62
Cct4	0.00	0.72
Pask	0.00	0.68
C1galt1	0.01	0.73
Dtymk	0.00	0.68
Eloc	0.00	0.76
Sgo2	0.00	0.67
Gmnn	0.00	0.54
Rps3	0.00	0.55
Sgo1	0.00	0.54
Fam72a	0.00	0.71
Ifitm3	0.04	0.63
Ifitm2	0.00	0.64
Ifitm1	0.02	0.50
Mcm10	0.00	0.46
Acpp	0.04	0.66
AC116220.3	0.01	0.74
Haus7	0.01	0.74
Haus4	0.00	0.57
Haus5	0.00	0.59
Haus3	0.00	0.64
Gen1	0.00	0.61
Tubg1	0.00	0.72
Pold1	0.00	0.52
Pold2	0.00	0.66
Ankrd34a	0.03	0.63
Oxct1	0.00	0.76
Abcb8	0.02	0.75
Dut	0.00	0.60
AC094217.1	0.04	0.68
Hdac1	0.00	0.68
Dazap2	0.02	0.76
Slbp	0.02	0.66
Ppidl1	0.01	0.74
Rfwd3	0.00	0.72
Nrm	0.00	0.59
Bard1	0.00	0.65
Ankh	0.00	0.63
Gltp	0.03	0.68
Pdia4	0.00	0.61
Cplane2	0.00	0.72

Tmem119	0.01	0.62
Emc8	0.01	0.70
Lsm5	0.00	0.67
St6galnac6	0.00	0.77
Wdhd1	0.00	0.63
St6galnac1	0.00	0.73
Myh10	0.01	0.73
Lsm7	0.00	0.75
Ttc30b	0.00	0.70
Kif4a	0.00	0.63
Hddc3	0.00	0.76
Mutyh	0.04	0.76
AC129365.1	0.00	0.43
Klhl23	0.00	0.71
Tmem97	0.00	0.49
Cdc20	0.00	0.35
Srebf2	0.01	0.71
Tshz1	0.01	0.74
Zscan22	0.01	0.73
Mob1a	0.01	0.72
AABR07066944.1	0.00	0.68
Ppm1g	0.00	0.70
Myl12b	0.00	0.64
Eef1aknmt	0.01	0.73
Calr	0.01	0.61
Spcs2	0.00	0.74
AC115273.1	0.00	0.49
H2afz	0.00	0.47
Mcm7	0.00	0.33
AABR07031089.1	0.00	0.62
Mcm5	0.00	0.39
Cbfb	0.04	0.73
Mcm3	0.00	0.38
Mcm2	0.00	0.44
Fadd	0.00	0.56
Krt8	0.00	0.73
Diaph1	0.00	0.74
Asb6	0.01	0.68
Faap24	0.00	0.75
Cenpk	0.00	0.72
Rpl6	0.00	0.69
LOC103690354	0.00	0.32
RGD1306941	0.00	0.75
Nucks1	0.00	0.76
AABR07015180.1	0.00	0.52
Rad51c	0.00	0.73
AABR07042866.1	0.01	0.67
mrpl11	0.00	0.77
RGD1560010	0.00	0.53
Ddx39b	0.04	0.76
Oard1	0.00	0.71
Ddx39a	0.00	0.65
Plk1	0.00	0.41
Rpl5	0.00	0.69

Plk4	0.00	0.50
Tmem8a	0.00	0.74
Mad2l1	0.00	0.47
Timeless	0.03	0.74
RF00085	0.02	0.72
Tspan17	0.01	0.75
Ormdl3	0.00	0.74
Taf13	0.01	0.71
Rassf1	0.04	0.77
Vrk1	0.00	0.67
AABR07055919.2	0.02	0.67
Crtapl1	0.00	0.69
Rpe	0.00	0.73
Dlgap5	0.00	0.43
Ppp2r5d	0.01	0.77
Stip1	0.01	0.69
Gprasp2	0.02	0.68
Ube2s	0.01	0.59
Alg8	0.00	0.65
Gpi	0.00	0.64
Spdl1	0.00	0.73
Gemin6	0.00	0.59
LOC100910717	0.00	0.75
Paxip1	0.00	0.74
Golt1b	0.00	0.65
Prim1	0.00	0.55
LOC100359600	0.00	0.52
Chek2	0.00	0.66
Suox	0.00	0.51
Tfdp1	0.00	0.59
Iqgap3	0.00	0.46
Tmpo	0.00	0.59
Katnb1	0.00	0.76
Nup35	0.00	0.58
Atp5f1a	0.00	0.75
Nup37	0.00	0.76
Tcp1	0.00	0.70
Trim59	0.00	0.56
Dnajc10	0.00	0.76
Tkt	0.02	0.76
Steap3	0.00	0.68
Mybl2	0.00	0.50
Rabl2	0.00	0.76
Ckap2l	0.00	0.56
Eef2	0.00	0.55
AY172581.1	0.04	0.32
AABR07000222.1	0.01	0.67
Bub1	0.00	0.51
AY172581.8	0.00	0.39
Dsn1	0.00	0.59
Kif14	0.00	0.74
Kif15	0.00	0.71
Brpf1	0.00	0.76
RF00264	0.02	0.73

Kif11	0.00	0.52
AABR07026797.1	0.02	0.75
Tnfaip2	0.00	0.59
Cdk2ap2	0.00	0.66
Pcna	0.00	0.38
Hjurp	0.00	0.43
LOC361346	0.00	0.67
Cdc25c	0.00	0.74
Cdc25b	0.00	0.57
Tubb5	0.01	0.59
Slc25a5	0.00	0.76
AC095390.1	0.00	0.39
Nup188	0.00	0.67
Lrrc40	0.00	0.75
Lrrc45	0.01	0.74
B9d2	0.04	0.76
Sephs2	0.00	0.71
Uhrf1	0.00	0.36
Lsm8	0.00	0.63
AABR07005838.1	0.00	0.63
Eef1g	0.00	0.66
LOC100362830	0.00	0.53
Tcerg1	0.01	0.73
Lsm2	0.00	0.57
Lsm3	0.00	0.51
LOC100911361	0.00	0.72
Lyar	0.01	0.61
Angptl2	0.00	0.56
Cse1l	0.00	0.67
Lgals1	0.01	0.76
Cbx3	0.00	0.57
Mrfap1	0.00	0.62
Lta4h	0.00	0.71
Ptpa	0.00	0.76
Tpx2	0.00	0.48
Mrpl35	0.00	0.66
Atcay	0.00	0.72
Pmf1	0.00	0.62
Bub1b	0.00	0.51
Qdpr	0.04	0.76
Cdt1	0.01	0.46
Sae1	0.00	0.73
LOC100359583	0.00	0.62
Nedd1	0.00	0.73
AABR07049223.1	0.01	0.53
AABR07000658.1	0.00	0.38
Lmnb2	0.03	0.65
AABR07069282.1	0.00	0.54
Lmnb1	0.01	0.57
Hdac1l	0.00	0.66
G2e3	0.00	0.55
Kntc1	0.00	0.45
AC109542.3	0.04	0.60
Nup85	0.00	0.57

Bgn	0.04	0.76
Phf19	0.01	0.73
Sart3	0.00	0.75
LOC100365839	0.00	0.61
Zwilch	0.00	0.72
Dnaaf3	0.00	0.75
AABR07068127.1	0.03	0.59
Api5	0.04	0.76
Aaas	0.00	0.57
Cisd1	0.05	0.74
Hmgn2	0.00	0.70
Tmem267	0.00	0.73
Exo1	0.00	0.60
AC109048.1	0.00	0.76
Nr1i3	0.00	0.74
Cdk1	0.00	0.46
Cep76	0.00	0.70
Cep72	0.00	0.68
Med18	0.00	0.69
LOC100363469	0.00	0.52
Trip13	0.00	0.75
Kifc1	0.00	0.45
Rpl35	0.00	0.53
Tmem106c	0.00	0.69
Srsf4	0.00	0.72
AABR07036247.1	0.01	0.64
AABR07067526.1	0.00	0.39
Ezh2	0.00	0.74
Rps3a	0.00	0.58
Tk1	0.00	0.40
Hspa2	0.02	0.67
Hspa5	0.00	0.55
Tymp	0.03	0.75
Srsf2	0.00	0.62
AABR07028349.1	0.00	0.67
Traip	0.00	0.62
Ubr7	0.00	0.77
Kif18b	0.00	0.59
Dnase2	0.00	0.74
Foxm1	0.00	0.47
Hnrnpul1	0.01	0.76
Rps4x	0.00	0.54
Rpl22l1	0.00	0.60
Gdf11	0.00	0.64
Fam83d	0.00	0.59
RT1-M3-1	0.00	0.71
AABR07038948.2	0.01	0.67
AABR07060593.1	0.00	0.54
Cetn2	0.02	0.69
Pkmyt1	0.00	0.76
LOC100360117	0.01	0.72
Mgme1	0.00	0.67
Rpl41	0.00	0.62
Gstk1	0.00	0.77

Ncaph2	0.00	0.65
Rnps1	0.01	0.61
Mcm6	0.00	0.45
Tmem129	0.01	0.74
Mcm4	0.00	0.41
Elovl1	0.04	0.75
Chaf1a	0.00	0.48
Got1	0.01	0.74
Rab15	0.03	0.69
Troap	0.00	0.43
AABR07058519.1	0.00	0.65
LOC100362333	0.00	0.64
Arpin	0.01	0.76
Chrac1	0.00	0.71
Cks2	0.00	0.43
Ube2c	0.01	0.43
Gnb2	0.04	0.74
Cdca2	0.00	0.63
Cdca3	0.00	0.35
AABR07057190.1	0.00	0.70
Ehd4	0.00	0.72
Cdca7	0.00	0.52
Cdca4	0.00	0.54
Ppih	0.00	0.76
Ehd3	0.01	0.55
Cdca8	0.00	0.51
Suv39h1l1	0.00	0.45
Il17rc	0.00	0.73
Ube2t	0.00	0.47
Ttk	0.00	0.57
Slc52a3	0.01	0.66
Rnaseh2a	0.00	0.72
Topbp1	0.00	0.54
Mfsd10	0.01	0.64
Tuba1b	0.02	0.64
Cdk2	0.00	0.54
Kif23	0.01	0.60
Kif22	0.00	0.39
Anp32e	0.00	0.69
Tube1	0.01	0.72
Dcaf15	0.00	0.76
AABR07002774.4	0.02	0.75
Thoc3	0.00	0.53
Arhgap19	0.00	0.74
Thoc7	0.01	0.76
Cenpe	0.00	0.49
Nsmce4a	0.00	0.73
Fkbp2	0.01	0.75
Aqp1	0.00	0.52
Med21	0.00	0.74
Cep44	0.03	0.76
Prpf31	0.00	0.66
Cep41	0.00	0.68
Rps7	0.00	0.65

Nop10	0.03	0.69
Fen1	0.00	0.36
Cyb5b	0.03	0.65
C1s	0.04	0.73
Rnf167	0.00	0.69
AC128960.1	0.00	0.59
Rps8	0.05	0.70
RGD1310553	0.00	0.68
Sema5a	0.00	0.75
Hmgb2	0.00	0.62
Cep295	0.00	0.61
LOC303566	0.00	0.67
Fam84a	0.04	0.73
Nxt1	0.01	0.74
Tedc1	0.00	0.62
Cdc45	0.00	0.62
AABR07002564.1	0.01	0.70
Kif2c	0.00	0.39
Ncapg2	0.00	0.48
Cenpw	0.00	0.44
Cnot9	0.00	0.65
Cxxc5	0.00	0.74
Rad51	0.00	0.69
AABR07065438.1	0.00	0.58
AABR07072133.1	0.02	0.75
Itgb5	0.00	0.76
AABR07038873.1	0.01	0.63
AABR07035541.2	0.04	0.67
Mms22l	0.00	0.77
AABR07014550.1	0.02	0.51
Hsp90ab1	0.00	0.76
Gucd1	0.02	0.69
Pbk	0.00	0.41
Evi2a	0.00	0.68
Trmt10a	0.00	0.65
Cenpo	0.00	0.53
Cenpm	0.05	0.75
Cenpl	0.00	0.76
Ccna2	0.00	0.46
Cenph	0.00	0.48
Atad5	0.00	0.70
Cenpf	0.00	0.47
AABR07039210.2	0.01	0.69
Ptgs1	0.00	0.65
Cenpa	0.02	0.76
Atad2	0.00	0.55
Slc35a4	0.00	0.60
Lman1	0.00	0.70
Manf	0.03	0.63
Ckap2	0.00	0.57
Cenpu	0.00	0.67
Cenpt	0.00	0.39
Ckap4	0.00	0.67
Ckap5	0.00	0.73

Rcc1	0.00	0.69
Dnajc9	0.00	0.50
Nudt1	0.00	0.66
Casp8ap2	0.00	0.70
Aarsd1	0.00	0.76
Tex261	0.00	0.76
Psmc3ip	0.00	0.60
Igsf3	0.00	0.71
Trabd	0.00	0.74
Csnk1e	0.00	0.65
AABR07010041.1	0.03	0.72
Gpn1	0.00	0.73
AABR07055917.1	0.04	0.66
Nnt	0.00	0.66
Klhdc8a	0.02	0.51
Ptma	0.00	0.66
Racgap1	0.01	0.68
Idh2	0.00	0.70
AC112018.1	0.04	0.55
Krt79	0.00	0.66
Eif2a	0.01	0.77
Rpl15	0.00	0.60
Rpl11	0.00	0.63
Atp1a1	0.00	0.68
Snrpb	0.01	0.54
Snrpa	0.00	0.66
Ndc80	0.00	0.53
RGD1306227	0.00	0.68
AC098125.3	0.00	0.61
Lrr1	0.01	0.74
Rad21	0.00	0.57
Rpa3	0.00	0.40
Rpa2	0.00	0.47
Tent4a	0.00	0.68
AABR07025301.1	0.00	0.49
Trib2	0.03	0.71
G3bp1	0.00	0.71
Thy1	0.00	0.59
Eme1	0.00	0.52
AABR07049755.1	0.00	0.69
Emilin1	0.00	0.63
Hyou1	0.00	0.62
Rpl39	0.04	0.62
Pfdn4	0.00	0.62
Mrpl14	0.00	0.72
Pagr1	0.04	0.65
LOC102555453	0.01	0.57
Incenp	0.00	0.50
Pcdhgb8	0.01	0.75
Las1l	0.00	0.73
Tipinl1	0.00	0.74
Cpsf3	0.00	0.72
Rad9a	0.00	0.66
Egr1	0.00	0.56

Mrpl28	0.00	0.73
Plcd4	0.00	0.73
Pih1d1	0.00	0.66
Ncaph	0.00	0.43
Ncapg	0.00	0.53
Magoh	0.00	0.75
Clec2dl1	0.00	0.72
Rack1	0.00	0.57
Hspa13	0.00	0.65
Rwdd4	0.00	0.64
Suv39h2	0.00	0.68
Nicn1	0.00	0.61
Sod3	0.00	0.74
Hcfc1	0.00	0.70
Hspa14	0.00	0.72
Esco2	0.00	0.72
Gtse1	0.01	0.68
Arhgap33	0.03	0.67
Tor1b	0.00	0.69
Pycard	0.00	0.65
Plat	0.00	0.77
Sgpp1	0.00	0.67
AABR07035539.1	0.01	0.66
Ect2	0.00	0.51
Naa40	0.00	0.69
AABR07027009.1	0.03	0.63
Cycs	0.01	0.64
Tm4sf1	0.01	0.70
Tgoln2	0.00	0.68
Pclaf	0.00	0.46
Tagln2	0.00	0.75
Necab3	0.00	0.65
Nup160	0.00	0.76
Knstrn	0.00	0.53
Ppm1f	0.00	0.58
AABR07069219.1	0.00	0.65
Ccne2	0.00	0.58
Rpl7	0.01	0.61
Ccne1	0.00	0.52
Rpl3	0.02	0.76
AABR07001512.1	0.00	0.44
Nsl1	0.00	0.74
AABR07055919.1	0.00	0.69
AABR07031666.1	0.00	0.58
Dek	0.00	0.47
Eif4ebp2	0.00	0.76
LOC308990	0.01	0.52
Rpl29	0.00	0.46
Ahcy	0.00	0.63
Fam111a	0.00	0.48
Fam102a	0.01	0.74
Nusap1	0.00	0.56
Ddx11	0.00	0.66
Tubb4b	0.00	0.61

Def8	0.00	0.76
Snrpa1	0.02	0.75
Sephs1	0.00	0.70
Tmem9	0.00	0.69
Ctdsp2	0.00	0.74
Amigo1	0.00	0.76
Wee1	0.00	0.71
Rnase1l2	0.01	0.35
Cdkn2c	0.00	0.47
Srsf7	0.00	0.51
Srsf1	0.00	0.65
Nelfcd	0.00	0.74
Ruvbl1	0.00	0.76
Mis18bp1	0.00	0.71
Tonsl	0.00	0.63
Nasp	0.00	0.54
Psmd13	0.00	0.75
Grwd1	0.01	0.75
E2f4	0.00	0.69
Espl1	0.00	0.54
AABR07012100.2	0.04	0.75
Chaf1b	0.00	0.59
Nrp2	0.00	0.73
Rnf26	0.00	0.45
Tmem14c	0.02	0.77
Mis18a	0.00	0.58
Rpl18	0.00	0.64
Tmem138	0.00	0.61
AABR07029198.1	0.01	0.66
Mastl	0.00	0.68
Smc4	0.00	0.66
Nup205	0.00	0.73
AABR07025328.1	0.03	0.62
Chtf18	0.00	0.55
Arhgdia	0.00	0.73
Kif20a	0.00	0.41
Actr1b	0.00	0.75
Fhod1	0.00	0.67
Prps2	0.00	0.74
Nup133	0.00	0.65
AABR07024500.1	0.01	0.68
Fmnl3	0.00	0.68
Dnmt1	0.00	0.63
Vwa1	0.00	0.59
RT1-N3	0.00	0.57
Stmn1	0.00	0.53
AABR07059171.1	0.02	0.71
Zw10	0.00	0.74
Dscc1	0.00	0.63
Xpo1	0.00	0.74
Pola2	0.00	0.73
Nemp1	0.00	0.76
Anapc15	0.00	0.62
Rpl24	0.01	0.71

Rpl27	0.01	0.55
Rpl26	0.00	0.66
LOC100359539	0.00	0.43
Rpl23	0.00	0.57
Fancg	0.00	0.72
Fancb	0.00	0.71
Fanca	0.00	0.73
Fam131b	0.00	0.76
Fanci	0.00	0.49
Fbl	0.02	0.76

TABLE S4C Top altered genes in MM cells following MLL2 knockdown

Gene	P-Value	Fold Change (shRNA/WT)
Tsn	0.00	1.30
Cyp1b1	0.04	1.31
Rasl11b	0.01	1.44
AABR07068285.2	0.02	1.53
Hs1bp3	0.00	1.31
Cyb5d1	0.04	1.31
Tspan3	0.00	1.36
Foxa2	0.01	1.62
Mblac1	0.04	1.40
RGD1311899	0.00	1.31
Mir3064	0.03	1.87
Hbp1	0.00	1.43
Gpx1	0.03	1.46
Slc39a9	0.01	1.41
B2m	0.00	1.45
Ctrb1	0.05	1.32
Gpx8	0.04	1.30
AC141220.3	0.02	1.39
Hes6	0.00	1.35
Ccn4	0.01	1.62
Them6	0.00	1.37
Kctd11	0.00	1.45
AABR07063829.2	0.03	1.57
Ccdc115	0.02	1.33
Zfp219	0.01	1.43
Med6	0.00	1.31
Ptprv	0.00	1.53
Timp1	0.00	1.41
Bpgm	0.01	1.62
AABR07064998.2	0.04	1.57
Tmem179b	0.00	1.34
Eme2	0.00	1.63
Itgb1bp1	0.02	1.33
RT1-M3-1	0.00	1.36
Haghl	0.01	1.45
Synj1	0.00	1.40
Flot2	0.00	1.31
AABR07041078.1	0.01	1.43
Sema7a	0.02	1.49
AABR07055834.1	0.01	1.44
Dnajb9	0.02	1.46
Btg2	0.02	1.59
Hoxb7	0.00	1.45
Hoxb5	0.02	1.46
Bbs10	0.00	1.68
Mme	0.01	1.39
AC142458.1	0.03	1.62
Ndrg4	0.00	1.42
Echdc1	0.00	1.39
Tap1	0.00	1.77
AABR07025787.1	0.00	2.16
Spaca6	0.02	1.37

Rnft1	0.00	1.63	
Mxd1	0.00	1.32	
Slc27a1	0.00	1.32	
Coro6	0.03	1.33	
Mpv17l2	0.00	1.30	
Fktn	0.00	1.31	
Renbp	0.01	1.68	
Chst12	0.05	1.37	
Akr1c12	0.01	1.61	
Mief2	0.00	1.32	
AABR07053169.1	0.01	1.40	
Mtss1l	0.01	1.37	
Tent5a	0.00	1.61	
AC108572.4	0.02	1.56	
Tmem185b	0.00	1.56	
Pdlim2	0.01	1.40	
P2rx4	0.00	1.69	
Cryba4	0.00	1.82	
Fsd1	0.02	1.39	
Bloc1s6	0.00	1.43	
Bloc1s4	0.00	1.30	
Klhl31	0.04	1.30	
AABR07068253.1	0.05	1.41	
Ccnt2	0.03	1.34	
Idua	0.02	1.46	
Terf2ip	0.00	1.31	
Ypel5	0.00	1.50	
RF00187	0.05	1.99	
Ypel3	0.00	1.63	
AC119762.5	0.03	1.35	
Tmem202	0.03	1.33	
Ggt1	0.01	1.99	
Tmem205	0.00	1.30	
Prss23	0.03	1.31	
Tmem208	0.00	1.35	
RF01182	0.03	1.50	
RGD1566099	0.02	1.38	
AABR07030647.1	0.00	1.65	
Slfnl1	0.03	1.30	
Mir1956	0.04	1.71	
Rilpl2	0.04	1.45	
Mir132	0.01	1.39	
Cdhr1	0.01	1.54	
Zfp513	0.00	1.30	
Nit1	0.00	1.70	
Icam5	0.04	1.52	
Pnpo	0.00	1.64	
Rnasek	0.03	1.34	
Sesn2	0.03	1.61	
Iah1	0.01	1.34	
Hilpda	0.03	1.72	
Tcn2	0.00	1.62	
Sparc	0.01	1.33	
Foxo4	0.00	1.40	

Dyrk1b	0.00	1.32	
Prob1	0.00	1.30	
Mir6318	0.01	1.50	
AC136867.1	0.03	1.37	
Mmp13	0.02	3.25	
Lgals3bp	0.00	1.71	
Adrb2	0.00	1.52	
AABR07068154.1	0.00	1.35	
LOC108351936	0.01	1.33	
Rhpn1	0.00	1.33	
AABR07012054.1	0.03	1.46	
Serinc4	0.04	1.33	
Sesn3	0.01	1.52	
Cry2	0.01	1.34	
Hapln4	0.01	1.31	
Neu1	0.00	1.73	
Hapln3	0.02	2.98	
Nfat5	0.03	1.36	
Slc8b1	0.00	1.47	
Lgmn	0.00	1.50	
AABR07004881.1	0.04	1.73	
Lrpap1	0.00	1.45	
Slc22a17	0.00	1.54	
Mrpl57	0.02	1.49	
Ip6k2	0.00	1.35	
AABR07004269.4	0.02	1.37	
Nme3	0.00	1.86	
Mir27a	0.00	1.82	
Spp1	0.02	1.52	
Lamb1	0.00	1.34	
Car11	0.00	1.33	
Abtb1	0.00	1.52	
Pgrmc1	0.00	1.39	
Mthfr	0.01	1.34	
Tpp1	0.01	1.45	
Nr4a1	0.04	1.34	
Bax	0.01	1.32	
P3h3	0.00	1.34	
Ing4	0.04	1.30	
AABR07070275.1	0.03	1.39	
AABR07027811.2	0.04	2.31	
Gdi1	0.00	1.53	
Cmtm6	0.00	1.47	
Fuca1	0.00	1.54	
Fuca2	0.01	1.33	
Slc46a1	0.01	1.35	
Fam98c	0.00	1.30	
AABR07033047.1	0.04	1.36	
Ddit4	0.00	2.69	
Cfh	0.00	1.47	
Enpp4	0.01	1.37	
Zfp36l1	0.04	1.33	
AABR07053516.1	0.00	1.30	
Slc39a1	0.00	1.39	

Tp53inp1	0.00	4.03	
Cst3	0.00	1.46	
Plagl1	0.03	1.47	
Cfp	0.05	1.45	
Atp6v1d	0.00	1.80	
Atp6v1f	0.00	1.40	
Atp6v1a	0.00	1.35	
AABR07071000.1	0.00	1.37	
Retreg3	0.00	1.35	
AABR07031972.1	0.02	1.31	
Pias4	0.00	1.33	
Ube2q1	0.00	1.31	
Tcta	0.00	1.62	
Gpr150	0.01	1.36	
Yod1	0.01	1.41	
Hsdl1	0.00	1.53	
Phf1	0.03	1.34	
AABR07027407.1	0.01	1.34	
Pla2g15	0.01	1.80	
Tmed3	0.00	1.33	
Ifi27	0.00	1.39	
Zfp688	0.00	1.65	
Dmpk	0.00	1.64	
Pdcd4	0.00	1.57	
AC094126.2	0.02	1.88	
Commd8	0.00	1.38	
Sirt6	0.02	1.32	
Cfap298	0.00	1.43	
Dhx57	0.00	1.32	
Commd4	0.00	1.47	
Amdhd2	0.00	1.83	
Lamp2	0.01	1.33	
Atp6v1g1	0.00	1.39	
Crebzf	0.02	1.53	
Ifitm6	0.00	1.40	
Ergic3	0.00	1.40	
Csprs	0.03	1.40	
Mirlet7i	0.01	1.90	
Pura	0.04	1.61	
Pold4	0.00	1.35	
Clu	0.00	1.86	
Bdh2	0.03	1.32	
Lpin3	0.03	1.34	
Spon2	0.01	1.86	
Ahsp	0.00	2.34	
Bmf	0.00	1.46	
AABR07012575.1	0.02	1.66	
RGD1564804	0.00	1.42	
Mir23a	0.01	3.07	
Btbd19	0.03	1.36	
Pink1	0.00	1.44	
Vamp2	0.00	1.34	
St6gal1	0.00	1.35	
Itm2c	0.00	1.72	

Itm2b	0.00	1.50	
Wnt5b	0.00	1.53	
Giot1	0.04	1.56	
P2ry4	0.01	1.51	
LOC102553785	0.01	1.62	
Klhl24	0.01	1.42	
AABR07061964.1	0.01	1.30	
Reep3	0.00	1.36	
Pgpep1	0.00	1.34	
AABR07060291.1	0.04	1.57	
Clec2d2	0.04	1.75	
AABR07044940.1	0.04	1.44	
Mapre3	0.00	1.40	
Ndufc1	0.00	1.36	
AABR07006860.2	0.02	1.47	
Morn2	0.01	1.39	
Trappc2	0.00	1.37	
Hmox1	0.01	1.66	
Cilp	0.01	1.38	
Atg12	0.02	1.34	
Cbfb	0.04	1.31	
LOC690276	0.00	1.58	
Blcap	0.00	1.45	
AC132627.2	0.05	1.31	
Dvl2	0.01	1.37	
Rnf166	0.00	1.46	
Adamtsl4	0.00	1.33	
Mir34a	0.02	1.31	
Mustn1	0.01	1.47	
Supt4h1	0.00	1.38	
Spry4	0.00	1.51	
Tmem30a	0.02	1.41	
AABR07002711.1	0.01	2.49	
Asl	0.00	1.64	
Dnpep	0.00	1.72	
Tspan13	0.03	2.60	
Plk2	0.01	1.84	
AABR07007690.1	0.00	1.61	
Ydjc	0.01	1.59	
AABR07067600.1	0.00	1.63	
AABR07068852.1	0.00	1.68	
Cyp26b1	0.01	1.38	
AABR07054266.1	0.00	1.50	
Pnpla6	0.00	1.37	
Tmem167b	0.02	1.40	
AABR07011697.1	0.00	1.60	
Pcmtd2	0.00	1.39	
AABR07026424.1	0.01	1.41	
Reck	0.00	1.36	
Cpt1c	0.00	1.43	
Pnrc1	0.00	1.52	
Rab29	0.00	1.39	
C1qtnf6	0.00	1.53	
Ccng1	0.01	1.39	

Fut4	0.05	1.31	
Ghdc	0.02	1.37	
AABR07038019.1	0.00	1.32	
Tceanc	0.03	1.31	
Slc30a1	0.00	1.38	
Net1	0.03	1.32	
AABR07000222.1	0.01	1.35	
Gnptg	0.00	1.38	
Mtcp1	0.00	1.38	
Tmem53	0.00	1.35	
C1rl	0.02	1.82	
AABR07053667.1	0.04	1.43	
Slc25a2	0.00	1.44	
Mettl23	0.01	1.31	
Tmem160	0.03	1.36	
Pdk4	0.00	1.69	
Pik3ip1	0.02	1.47	
Gas6	0.00	1.60	
Kcnj2	0.03	1.41	
Lonrf2	0.00	1.43	
Fos	0.00	1.53	
Parp10	0.00	1.35	
RGD1308117	0.04	1.54	
Ccdc17	0.02	1.33	
Ormdl2	0.00	1.31	
Atcay	0.03	1.44	
Lig4	0.01	1.65	
Tnfrsf26	0.01	1.73	
Eid1	0.00	1.34	
Pln	0.00	2.13	
Tcp11l2	0.00	1.39	
Slc43a1	0.01	1.37	
Cldn12	0.00	1.50	
Slc10a5	0.01	1.58	
Grina	0.00	1.57	
Sqstm1	0.00	1.37	
Plekhb2	0.00	1.90	
Gfpt1	0.00	1.59	
AABR07044420.2	0.02	1.54	
Setd6	0.02	1.40	
Zfr2	0.00	1.37	
AC242615.2	0.02	1.39	
Pld3	0.00	1.43	
Mfsd11	0.04	1.40	
Fkbp7	0.00	1.41	
Cdk5	0.04	1.31	
Aldh5a1	0.03	1.34	
Kifc2	0.00	1.48	
Ctsb	0.00	1.31	
Ch25h	0.00	1.75	
Vmp1	0.01	1.32	
AABR07029958.2	0.01	1.54	
Gigyf1	0.02	1.68	
AC105515.1	0.03	1.60	

Aktip	0.00	1.41	
Limd2	0.01	1.47	
Mdm2	0.02	1.37	
Gja1	0.00	1.44	
Ltbp2	0.00	1.31	
Mfsd1	0.01	1.40	
Dcxr	0.00	1.34	
Plat	0.00	1.33	
Dnase2	0.00	1.31	
Mkrn3	0.01	1.38	
AABR07049769.2	0.03	1.50	
Tle2	0.01	1.37	
Gdf15	0.04	1.48	
Il18bp	0.04	1.34	
Tvp23b	0.00	1.49	
Esrra	0.01	1.52	
Sfrp4	0.01	1.35	
Gba	0.00	1.57	
Ankrd49	0.01	1.32	
Mir6334	0.00	1.85	
LOC100174910	0.00	1.34	
Spns1	0.00	1.76	
Mk1	0.00	1.47	
Abcc5	0.03	1.33	
Tmem86a	0.02	1.47	
Scarf2	0.01	1.81	
Gaa	0.00	1.75	
Tgfb1i1	0.00	1.40	
Cp	0.02	1.40	
Hint2	0.00	1.43	
AABR07032480.1	0.05	1.43	
AABR07001389.1	0.01	1.47	
Ube2g2	0.00	1.46	
Arrdc3	0.00	1.43	
Pcdh18	0.00	1.33	
Lin7c	0.00	1.81	
Nrp1	0.00	1.33	
Dstn	0.03	1.49	
Vgll4	0.00	1.32	
AC133265.1	0.01	1.59	
Lysmd1	0.00	1.30	
Zbtb18	0.02	1.32	
Aqp1	0.05	1.63	
Med25	0.02	1.43	
Alas1	0.03	1.34	
Aamp	0.00	1.46	
Alg10	0.00	1.53	
C1r	0.01	1.33	
Ermard	0.04	1.31	
Smim14	0.00	1.43	
Ccdc43	0.00	1.32	
Nxt2	0.00	1.30	
Dvl3	0.00	1.36	
Syt11	0.01	1.39	

Dlx5	0.01	1.37	
Cox6a2	0.00	2.20	
Gabarapl1	0.01	1.84	
Wfdc18	0.02	1.80	
Rpusd3	0.00	1.33	
Psap	0.00	1.32	
Saraf	0.00	1.35	
Sp110	0.00	1.32	
Bnip3l	0.00	1.33	
Slc19a2	0.01	1.48	
Nrbp2	0.00	1.77	
Mpg	0.00	1.33	
Cdk10	0.00	1.34	
Ctsf	0.00	1.66	
Fau	0.00	1.34	
Lancl1	0.00	1.36	
AABR07044635.1	0.00	2.03	
AABR07056686.1	0.00	1.30	
LOC102549726	0.00	1.39	
Mlycd	0.00	1.33	
Mpzl3	0.01	1.43	
Ptgs2	0.02	1.38	
RGD1306502	0.00	1.41	
Lztfl1	0.01	1.32	
Cxcr3	0.00	1.65	
Mapk6	0.00	1.42	
AABR07046628.1	0.01	1.60	
AC119111.1	0.00	1.56	
RGD1305938	0.05	1.30	
AABR07035486.1	0.00	1.43	
AABR07009357.2	0.00	1.60	
AABR07014804.1	0.00	1.42	
LOC100361265	0.02	1.46	
Cldnd1	0.01	1.36	
RT1-CE10	0.03	1.40	
Arfip2	0.04	1.31	
Socs2	0.00	1.32	
Ccdc96	0.00	1.60	
Krt79	0.05	1.43	
Cnpy2	0.00	1.46	
Psmb9	0.00	1.56	
Borcs6	0.03	1.36	
LOC100909474	0.03	1.89	
RF00404	0.05	1.84	
LOC103691238	0.00	1.62	
Lpcat2	0.00	1.35	
AABR07011996.1	0.00	1.58	
Fcgrt	0.00	1.49	
Lrrc73	0.00	1.47	
AABR07068417.2	0.03	1.67	
Enpep	0.03	1.33	
Plod2	0.00	1.63	
Cdkn1a	0.00	2.11	
RF00282	0.03	1.87	

Dtd2	0.00	1.32	
Lbh	0.00	1.35	
AABR07027810.4	0.04	1.41	
Yipf2	0.00	1.38	
Samd9	0.03	1.45	
AY172581.21	0.03	1.96	
Selenos	0.01	1.43	
Gadd45a	0.00	2.45	
Dynlt3	0.00	1.68	
Wnt4	0.00	1.57	
Sv2a	0.02	1.36	
Klc4	0.00	1.38	
Hist1h1c	0.01	1.75	
Tomm6	0.03	1.34	
Aox1	0.00	1.31	
Entpd4	0.00	1.40	
Sod2	0.00	1.36	
Erlec1	0.00	1.38	
Glyctk	0.01	1.30	
Ecm1	0.02	1.33	
Fbxw9	0.00	1.47	
Dync2li1	0.00	1.34	
S100a16	0.01	1.63	
Nol3	0.03	1.30	
Tgfb2	0.00	1.32	
AABR07072853.1	0.01	2.20	
Prdx5	0.02	1.39	
AABR07029195.1	0.01	1.54	
Thyn1	0.01	1.30	
Tgoln2	0.00	1.66	
AC123425.1	0.00	1.89	
AABR07070270.1	0.02	1.45	
LOC100912483	0.03	1.65	
Arl4a	0.01	1.69	
St3gal4	0.04	2.02	
Rogdi	0.00	1.52	
Pomgnt2	0.01	1.44	
Des	0.02	1.38	
Serpina3n	0.02	1.73	
Sowahc	0.00	1.63	
LOC308990	0.00	1.92	
Tbc1d17	0.00	1.35	
Hspa1l	0.00	1.37	
LOC108351584	0.03	1.40	
Wdr45	0.00	1.74	
Pcdhb14	0.04	1.32	
Gpr108	0.00	1.33	
AABR07033023.1	0.03	1.30	
Lypla1	0.00	1.35	
Lypla2	0.02	1.31	
Cotl1	0.01	1.36	
Mir3568	0.04	1.53	
Mir374b	0.05	1.49	
Mir3564	0.00	1.60	

Thnsl1	0.00	1.50	
Dsel	0.02	1.58	
Slc38a7	0.00	1.71	
Clba1	0.00	1.36	
Snn	0.00	1.62	
Slc9a3r1	0.01	1.52	
AABR07029198.1	0.01	1.32	
AABR07044273.1	0.01	1.56	
Rnd2	0.00	1.52	
AABR07058464.1	0.01	1.45	
Maf	0.00	1.96	
Psenen	0.03	1.30	
Atp6v1b2	0.00	1.35	
Myo10	0.00	1.36	
RGD1562378	0.05	1.57	
Dtx3	0.01	1.39	
Ttpal	0.04	1.31	
Ccpg1	0.00	1.40	
AABR07043654.1	0.00	1.32	
RT1-N2	0.01	1.33	
Podnl1	0.01	1.39	
AABR07066529.1	0.00	1.34	
Myo1e	0.00	1.56	
Tmem203	0.03	1.34	
Chst2	0.00	1.37	
Etfrf1	0.00	2.12	
Dpp7	0.00	1.81	
Cnih1	0.00	0.70	
Rad1	0.00	0.75	
Gas2l3	0.00	0.51	
Cmpk1	0.01	0.68	
Cep55	0.00	0.61	
Rbbp7	0.00	0.74	
Ccnf	0.01	0.59	
AABR07021734.1	0.00	0.69	
Kpna2	0.01	0.76	
Ctbp1	0.00	0.76	
Asf1b	0.00	0.55	
Ticrr	0.00	0.70	
AABR07066516.1	0.00	0.77	
Hnrnpa1	0.01	0.68	
Siva1	0.03	0.70	
Ska1	0.00	0.62	
Ska3	0.00	0.69	
Slc1a4	0.00	0.64	
Slc39a5	0.00	0.71	
Ptdss1	0.00	0.76	
Avpr1a	0.01	0.60	
LOC314140	0.00	0.73	
AABR07062599.1	0.02	0.71	
Tacc3	0.00	0.63	
Nap1l4	0.00	0.75	
Cks1b	0.00	0.70	
Ncapd2	0.00	0.58	

Cstf2	0.00	0.76	
Akt1	0.00	0.70	
Akt2	0.02	0.73	
Med1	0.00	0.65	
Dctpp1	0.01	0.71	
Dag1	0.01	0.73	
Rrm1	0.00	0.51	
Cdk20	0.00	0.73	
Get4	0.00	0.68	
AABR07026311.1	0.00	0.55	
Naa10	0.02	0.75	
Fubp1	0.01	0.76	
Slfn13	0.00	0.69	
LOC102546716	0.00	0.70	
Prim1	0.00	0.53	
AABR07044366.1	0.00	0.63	
Ccnb1	0.00	0.53	
Slc25a39	0.00	0.60	
Palb2	0.01	0.77	
AABR07024972.1	0.04	0.54	
Arl6ip1	0.03	0.69	
Wipf1	0.00	0.77	
Brca1	0.00	0.73	
Cntf	0.00	0.74	
Mad2l1	0.00	0.58	
Tmem109	0.02	0.74	
Dnajb1	0.00	0.59	
Dnajb5	0.00	0.70	
Pif1	0.02	0.70	
Fpgs	0.05	0.75	
RGD1562136	0.01	0.63	
Pclaf	0.00	0.59	
Serpinh1	0.01	0.64	
Ppp1r9b	0.00	0.66	
Spc25	0.00	0.53	
Jpt2	0.00	0.68	
Tmpo	0.00	0.68	
Shcbp1	0.00	0.70	
Pole2	0.00	0.76	
Pmm2	0.00	0.70	
Fam32a	0.01	0.77	
11-Sep	0.00	0.69	
Ldha	0.00	0.69	
AABR07019399.1	0.00	0.64	
Zbtb2	0.00	0.75	
Ubiad1	0.00	0.70	
Haspin	0.01	0.58	
AABR07042301.1	0.03	0.63	
Hsp90b1	0.00	0.69	
Ttk	0.00	0.66	
Amfr	0.03	0.75	
Arhgap11a	0.00	0.57	
Mmgt1	0.00	0.55	
Pak4	0.04	0.70	

Tedc2	0.00	0.69	
Bora	0.00	0.74	
Dgkz	0.00	0.76	
AABR07053707.1	0.00	0.65	
Pkp1	0.02	0.74	
Setsip	0.04	0.64	
Clspn	0.00	0.74	
AC127920.1	0.00	0.75	
RF00594	0.03	0.63	
Thtpa	0.00	0.74	
Tufm	0.01	0.74	
Pmp22	0.00	0.75	
Etv5	0.00	0.66	
Mknk2	0.00	0.66	
Cdr2	0.00	0.71	
Smc2	0.00	0.62	
AABR07028352.1	0.00	0.51	
Agpat1	0.01	0.70	
Pop4	0.00	0.72	
Lta4h	0.00	0.77	
Tns2	0.00	0.68	
Gins2	0.00	0.76	
AABR07008097.1	0.00	0.74	
Nasp	0.00	0.56	
Gins1	0.00	0.71	
Rnf10	0.01	0.58	
E2f2	0.00	0.77	
Gins4	0.00	0.74	
Dtl	0.00	0.70	
Sf3b3	0.00	0.66	
E2f8	0.00	0.65	
Fancd2	0.00	0.68	
Manea	0.00	0.70	
Pttg1	0.00	0.68	
Crip2	0.04	0.74	
Kmt2b	0.00	0.60	
Steap2	0.00	0.71	
Erlin1	0.04	0.77	
Ankrd13a	0.00	0.54	
Anln	0.00	0.70	
Tipinl1	0.00	0.59	
AABR07012795.1	0.02	0.77	
Fbxo5	0.00	0.57	
Cavin1	0.00	0.55	
Rfc5	0.00	0.60	
Rfc3	0.01	0.70	
Rfc2	0.00	0.62	
Phf5a	0.00	0.74	
Lrrc75b	0.00	0.59	
Ube2s	0.02	0.69	
Efhd2	0.02	0.73	
Ddx20	0.00	0.72	
Tcof1	0.00	0.65	
Elk3	0.00	0.73	

Ldlr	0.05	0.45	
Elk4	0.00	0.70	
Emp1	0.00	0.51	
Thap12	0.00	0.65	
Dclre1b	0.00	0.57	
Hirip3	0.00	0.56	
Tulp3	0.00	0.63	
Ncaph	0.00	0.63	
Zfp496	0.01	0.69	
Pimreg	0.01	0.62	
Cmtr2	0.00	0.70	
Ncapd3	0.00	0.71	
Gpsm2	0.00	0.69	
Birc5	0.00	0.67	
AABR07018244.2	0.00	0.74	
Aunip	0.01	0.71	
Nup43	0.00	0.74	
Dok1	0.00	0.68	
Trim47	0.00	0.77	
F2rl1	0.00	0.64	
Sapcd2	0.04	0.56	
Rrm2	0.00	0.63	
RGD1305350	0.04	0.76	
Nup107	0.00	0.72	
Usp1	0.00	0.59	
Msh2	0.01	0.75	
Msh6	0.01	0.72	
Gpd1l	0.00	0.61	
AC126897.1	0.05	0.67	
Top2a	0.00	0.60	
Anxa1	0.00	0.55	
Anxa2	0.00	0.77	
Raly	0.02	0.73	
Hat1	0.00	0.73	
Arpc5l	0.00	0.76	
Rangap1	0.00	0.71	
Hyls1	0.00	0.60	
Rad51ap1	0.00	0.68	
Sfpq	0.00	0.76	
Emg1	0.00	0.66	
Lrrc59	0.00	0.67	
Zbtb12	0.00	0.72	
Cep68	0.00	0.72	
Trim59	0.00	0.73	
Exosc8	0.00	0.68	
Pcbp2	0.00	0.66	
Phgdh	0.04	0.76	
Exosc2	0.00	0.76	
Prc1	0.00	0.53	
Gen1	0.00	0.68	
Ing1	0.02	0.68	
Cad	0.00	0.70	
Hmmr	0.00	0.63	
Zdhhc7	0.00	0.68	

Msn	0.00	0.76
Carm1	0.00	0.68
Polg	0.00	0.58
Tubb6	0.01	0.69
Pole	0.00	0.62
Lin54	0.00	0.72
Wdr5	0.00	0.70
Wdr1	0.00	0.65
Depp1	0.04	0.70
Aspm	0.00	0.64
Mdc1	0.00	0.61
Slc25a5	0.00	0.73
Pwp2	0.00	0.77
Rnf26	0.04	0.75
Kif20b	0.00	0.67
Pa2g4	0.00	0.64
Cdc6	0.00	0.58
Cdc7	0.00	0.71
Ankle1	0.03	0.73
Cip2a	0.00	0.69
Stmn1	0.00	0.63
AC142180.1	0.02	0.76
RGD1562690	0.00	0.61
Ercc6l	0.00	0.62
Ndc1	0.00	0.75
Rassf1	0.02	0.70
Aurka	0.00	0.55
Aurkb	0.00	0.66
Fkbp1a	0.00	0.64
Tcf19	0.00	0.52
Mex3c	0.00	0.68
Slc25a10	0.00	0.62
Tex30	0.00	0.72
Cnnm4	0.00	0.69
Rad54l	0.00	0.71
Tfrc	0.00	0.63
Mief1	0.00	0.71
AC094643.2	0.00	0.58
Ung	0.00	0.55
Reep4	0.00	0.75
Zfp275	0.01	0.75
AABR07038269.1	0.01	0.74
Bard1	0.00	0.71
Pask	0.00	0.72
Wsb2	0.00	0.64
Sgo2	0.00	0.75
Gmnn	0.00	0.62
Uhrf1	0.01	0.47
Txnrd1	0.00	0.69
Mki67	0.00	0.48
Stil	0.00	0.63
Mcm10	0.00	0.62
Haus8	0.00	0.76
Haus4	0.00	0.63

Fignl1	0.00	0.73	
Pold1	0.02	0.61	
Pold2	0.00	0.65	
Dut	0.02	0.73	
Slbp	0.01	0.64	
Ppidl1	0.02	0.73	
Ctdnep1	0.00	0.75	
Nrm	0.00	0.58	
Clic1	0.00	0.60	
Gltp	0.01	0.65	
Sfxn3	0.00	0.67	
Wdhd1	0.00	0.69	
Rrp15	0.00	0.77	
Rpl22l1	0.00	0.69	
Lmf2	0.01	0.74	
LOC100361025	0.00	0.74	
Add1	0.00	0.74	
Kif4a	0.00	0.67	
Rars	0.01	0.77	
Nacc1	0.00	0.72	
Col12a1	0.02	0.77	
AC129365.1	0.00	0.54	
Set	0.00	0.75	
LOC361346	0.03	0.76	
U2af1	0.00	0.76	
Ranbp1	0.04	0.64	
Tmem97	0.02	0.71	
Cdc20	0.00	0.57	
Zwilch	0.00	0.71	
AC128212.1	0.00	0.58	
AABR07072133.1	0.00	0.71	
Crat	0.00	0.66	
Ppm1g	0.00	0.75	
Myl12b	0.00	0.76	
Slc16a1	0.00	0.75	
AC115273.1	0.01	0.71	
H2afz	0.00	0.55	
Alyref	0.00	0.66	
Mcm7	0.00	0.57	
Mcm6	0.00	0.52	
Mcm5	0.01	0.56	
Mcm4	0.00	0.53	
Mcm3	0.00	0.46	
Mcm2	0.00	0.53	
AABR07029970.1	0.03	0.67	
Ugdh	0.00	0.72	
Rplp0	0.02	0.73	
RGD1560010	0.00	0.67	
Cdh3	0.00	0.61	
AABR07015180.1	0.00	0.57	
LOC100364062	0.00	0.75	
Mad2l2	0.00	0.76	
Ddx39a	0.00	0.64	
Plk1	0.00	0.53	

Cdc42ep1	0.00	0.75	
Plk4	0.00	0.54	
Cdc42ep5	0.01	0.68	
Cdc42ep4	0.00	0.75	
Fam107b	0.00	0.64	
Timeless	0.00	0.58	
AABR07051190.1	0.00	0.76	
RF00085	0.03	0.72	
Tspan17	0.03	0.75	
Ogg1	0.00	0.76	
P4ha3	0.00	0.69	
Ripk3	0.00	0.67	
Adgra2	0.02	0.73	
Dlgap5	0.00	0.65	
Lasp1	0.00	0.75	
Stip1	0.01	0.69	
AABR07028027.1	0.00	0.72	
RGD1311946	0.00	0.76	
Slc52a3	0.00	0.65	
Pdlim1	0.00	0.72	
Cdt1	0.03	0.56	
Apbb1	0.00	0.75	
Paxip1	0.00	0.74	
Pak1ip1	0.01	0.77	
LOC100359600	0.00	0.65	
Anapc1	0.00	0.76	
Iqgap3	0.00	0.56	
Card19	0.00	0.75	
Hoxd9	0.01	0.68	
Eef1a1	0.00	0.75	
Rad21	0.00	0.70	
Sumo4	0.01	0.68	
Map3k6	0.01	0.77	
AABR07050545.1	0.02	0.72	
Slc30a4	0.00	0.70	
Mybl2	0.01	0.47	
Gnai2	0.01	0.74	
Ckap2l	0.00	0.67	
Eef2	0.00	0.67	
Recql4	0.00	0.59	
Bub1	0.00	0.57	
AY172581.8	0.00	0.62	
Dsn1	0.00	0.60	
F2r	0.00	0.76	
Kif11	0.00	0.59	
Mrpl45	0.00	0.71	
Pfas	0.00	0.76	
Pcna	0.00	0.50	
Ttc9c	0.00	0.72	
Xrcc2	0.00	0.75	
Hjurp	0.00	0.59	
Klhl23	0.00	0.71	
AABR07035383.1	0.02	0.72	
Cdc25b	0.00	0.61	

Nolc1	0.00	0.77
Tubb5	0.02	0.55
Impdh2	0.00	0.72
AC095390.1	0.02	0.71
Nup188	0.00	0.73
RGD1563620	0.00	0.76
Areg	0.00	0.58
Zdhhc16	0.03	0.77
Lsm8	0.00	0.73
AABR07005838.1	0.00	0.65
Rac1	0.00	0.72
Lsm5	0.00	0.73
Lsm2	0.02	0.76
Lsm3	0.00	0.55
Tuba4a	0.05	0.74
Cse1l	0.00	0.72
Ucp2	0.00	0.69
Irak1	0.00	0.56
Cbx3	0.00	0.59
Sephs1	0.00	0.68
Tpx2	0.00	0.62
Pmf1	0.00	0.65
Bub1b	0.00	0.65
Sdc1	0.03	0.53
Gemin6	0.00	0.68
Rbm17	0.00	0.67
Nrbp1	0.00	0.62
LOC100359583	0.00	0.59
Lig3	0.00	0.76
AABR07049223.1	0.03	0.70
Lmnb2	0.02	0.57
AABR07069282.1	0.01	0.65
Lmnb1	0.01	0.57
AABR07018058.1	0.03	0.70
G2e3	0.00	0.71
Cpd	0.05	0.72
Kntc1	0.00	0.60
Nup85	0.00	0.66
LOC100365839	0.02	0.71
Csrp1	0.00	0.65
Hdgf	0.00	0.65
Hnrnpul2	0.00	0.73
Hnrnpul1	0.00	0.70
Ywhaz	0.00	0.61
Api5	0.00	0.65
Aaas	0.00	0.71
Exo1	0.00	0.68
Itga5	0.00	0.76
Celf1	0.00	0.77
Fkbp4	0.00	0.64
Cdk1	0.00	0.66
Cdk2	0.00	0.52
Kifc1	0.00	0.60
Rgl2	0.00	0.77

AABR07032856.1	0.04	0.72
Odf2	0.00	0.77
Rpp21	0.01	0.76
Ccnd3	0.02	0.54
AABR07067526.1	0.00	0.47
Ezh2	0.00	0.70
Rps3a	0.01	0.72
Lox	0.00	0.52
Tubgcp2	0.00	0.67
Hsp90ab1	0.00	0.68
Fut11	0.01	0.65
Hspa5	0.00	0.74
Srsf2	0.00	0.74
Traip	0.00	0.73
AABR07038895.2	0.01	0.69
Kif18b	0.00	0.67
Foxm1	0.00	0.52
Ctdsp2	0.00	0.47
Fam83d	0.03	0.68
AABR07053669.1	0.03	0.75
Bcs1l	0.00	0.75
AABR07060593.1	0.00	0.73
Tk1	0.00	0.46
Ahsa1	0.00	0.77
Ctdspl	0.01	0.73
Ncaph2	0.00	0.75
Nup62	0.00	0.76
Vars	0.03	0.75
Ptpa	0.00	0.76
Grk2	0.00	0.66
Ppat	0.00	0.67
Chaf1a	0.00	0.62
Eps8l2	0.00	0.76
Rab15	0.00	0.65
Troap	0.00	0.62
Pgk1	0.00	0.76
Hsf1	0.00	0.74
Cenpo	0.00	0.65
Nop56	0.03	0.74
Ctps1	0.00	0.67
Orc6	0.00	0.66
Cks2	0.00	0.61
Ube2c	0.03	0.55
Fadd	0.00	0.53
Gnb1	0.00	0.71
Orc1	0.00	0.66
Cdca2	0.00	0.74
Cdca3	0.00	0.43
Cdca7	0.01	0.53
Cdca4	0.00	0.67
Cenpk	0.00	0.76
Cdca8	0.00	0.60
Suv39h1l1	0.00	0.58
Ube2t	0.00	0.68

Npm3	0.02	0.76	
Topbp1	0.00	0.62	
Tuba1b	0.03	0.60	
Fzr1	0.03	0.72	
RGD1564613	0.01	0.71	
Kif23	0.00	0.68	
Kif22	0.00	0.56	
Nup153	0.00	0.76	
Anp32e	0.00	0.63	
Anp32b	0.00	0.63	
Isy1	0.00	0.70	
Cenpf	0.00	0.67	
Thoc3	0.00	0.75	
Ankrd52	0.00	0.61	
AC098459.1	0.02	0.57	
Nsmce4a	0.00	0.75	
Wdtc1	0.00	0.72	
Med21	0.00	0.76	
Prpf31	0.00	0.75	
Ssx2ip	0.00	0.69	
AABR07038939.1	0.03	0.70	
Nop10	0.02	0.68	
Fen1	0.00	0.68	
Fam136a	0.00	0.77	
Cep295	0.02	0.74	
Aida	0.00	0.75	
Fam84a	0.05	0.76	
Nxt1	0.00	0.66	
Tedc1	0.00	0.67	
Cdc45	0.00	0.74	
AABR07002564.1	0.01	0.63	
Kif2c	0.00	0.57	
Ncapg2	0.00	0.69	
Mtch1	0.00	0.64	
P4hb	0.01	0.69	
Cenpw	0.00	0.51	
Cnot9	0.01	0.71	
Myo5a	0.00	0.75	
Hnrnpd	0.00	0.76	
Hnrnpf	0.00	0.69	
Ubqln4	0.00	0.74	
AABR07037356.1	0.01	0.73	
Pigq	0.00	0.42	
Pbk	0.00	0.57	
AABR07039307.1	0.00	0.75	
Ehd4	0.00	0.76	
Ccna2	0.00	0.57	
Nlrp3	0.01	0.74	
Cenph	0.00	0.75	
Atad5	0.00	0.75	
AABR07002774.4	0.02	0.76	
Cenpe	0.00	0.60	
Cenpa	0.03	0.76	
Atad2	0.00	0.62	

Nuf2	0.00	0.74
AABR07067583.1	0.00	0.69
Rexo1	0.01	0.77
Ckap2	0.00	0.73
Cenpu	0.00	0.73
Cenpt	0.00	0.60
AABR07013255.1	0.02	0.67
Ckap5	0.00	0.68
Rcc1	0.00	0.68
Nsd2	0.00	0.72
Dnajc9	0.00	0.62
Ptges3	0.01	0.71
Psmc3ip	0.00	0.58
G3bp1	0.00	0.74
Timm8a1	0.01	0.74
E130309D02Rik	0.01	0.74
Nnt	0.00	0.68
Scoc	0.00	0.74
Ptma	0.00	0.63
Dhrs9	0.00	0.66
Racgap1	0.03	0.71
Idh2	0.00	0.54
LOC680491	0.02	0.74
Snrpa	0.00	0.75
Ndc80	0.00	0.67
Pxdn	0.00	0.58
Sun1	0.00	0.74
AC098125.3	0.00	0.77
Rpa3	0.01	0.69
Rpa2	0.00	0.67
Tent4a	0.00	0.75
AABR07025301.1	0.00	0.46
AABR07054368.1	0.00	0.73
Gna11	0.00	0.61
S100a10	0.00	0.62
Eme1	0.00	0.69
Idh3a	0.00	0.75
Hyou1	0.00	0.70
AABR07049695.3	0.00	0.67
Samd1	0.00	0.69
Zfp422	0.00	0.77
Plp2	0.00	0.66
Dbr1	0.00	0.75
LOC102555453	0.05	0.74
Incenp	0.00	0.57
Hk2	0.00	0.66
Rad9a	0.00	0.73
Spag5	0.00	0.62
Psmd5	0.00	0.57
Selenoi	0.00	0.77
Mob3a	0.00	0.70
Smarcd1	0.00	0.70
Me2	0.00	0.75
Ncapg	0.00	0.64

Nicn1	0.01	0.76
Sod3	0.00	0.49
Hcfc1	0.00	0.75
Hspa14	0.00	0.74
Sec13	0.00	0.70
Gtse1	0.01	0.70
Rock2	0.00	0.76
Coro1c	0.01	0.75
Ereg	0.01	0.66
Tmem214	0.00	0.70
Arl2bp	0.00	0.40
Tgfb3	0.00	0.74
Ect2	0.00	0.59
Naa40	0.00	0.71
Sgo1	0.00	0.71
Gpat4	0.00	0.74
Cycs	0.00	0.66
Prkar2a	0.02	0.76
Lats1	0.00	0.77
Tagln2	0.00	0.68
Necab3	0.00	0.65
Ranbp3	0.00	0.74
Knstrn	0.00	0.63
Ppm1f	0.00	0.71
Loxl2	0.02	0.73
Loxl3	0.05	0.73
Ifrd2	0.00	0.65
Ccne2	0.01	0.66
Ccne1	0.01	0.69
AABR07001512.1	0.00	0.61
AABR07055919.1	0.00	0.76
AABR07055280.1	0.00	0.75
Dek	0.00	0.55
Eif4ebp1	0.00	0.76
Eif4ebp2	0.00	0.53
Ahcy	0.00	0.67
Fam111a	0.00	0.52
Nusap1	0.00	0.71
Ddx11	0.00	0.71
Tubb4b	0.01	0.69
Snrpa1	0.01	0.70
Parp2	0.00	0.77
Fabp5	0.02	0.63
AABR07000658.1	0.00	0.63
Wee1	0.00	0.67
Tes	0.00	0.67
Cdkn2c	0.00	0.58
Srsf7	0.03	0.73
Srsf1	0.02	0.76
Srsf3	0.01	0.72
Emd	0.00	0.60
Tonsl	0.00	0.71
Espl1	0.00	0.64
Chaf1b	0.00	0.63

Gatad2a	0.00	0.67
Mis18a	0.01	0.69
Snrpf	0.03	0.75
AABR07036855.1	0.00	0.71
Smc4	0.00	0.64
Nup205	0.00	0.73
RGD1561149	0.01	0.74
Tmem43	0.00	0.62
Chtf18	0.01	0.70
Josd1	0.00	0.73
Kif20a	0.00	0.55
Hpdl	0.04	0.73
Actr1b	0.00	0.69
Ptges3l1	0.00	0.71
AABR07024500.1	0.01	0.69
Dnmt1	0.00	0.63
Ids	0.02	0.76
Ppp2r5d	0.00	0.70
RT1-N3	0.01	0.68
AABR07059171.1	0.03	0.71
Zw10	0.00	0.77
Dscc1	0.00	0.68
Xpo1	0.00	0.72
Mprip	0.00	0.76
Anapc15	0.00	0.52
Chrm3	0.00	0.70
LOC100359539	0.00	0.60
Dpp9	0.00	0.66
Tnfaip8l1	0.01	0.53
Fancb	0.00	0.75
Fanci	0.00	0.71
Fbl	0.00	0.63

TABLE S4D Top altered genes in KMM cells following GRWD1 knockdown

Gene	P-Value	Fold Change (shRNA/WT)
Zwint	0.04	1.95
Foxa2	0.04	1.51
Syt8	0.03	1.36
Irf7	0.04	2.54
Rnf181	0.00	1.34
B2m	0.01	1.36
Klf2	0.04	1.57
Rgs2	0.01	1.75
Gpx8	0.00	1.30
Gaa	0.00	1.47
LOC290595	0.02	1.34
Ccn4	0.04	1.63
Sat1	0.01	1.39
AY172581.2	0.04	1.56
Ccn2	0.03	1.55
AY172581.9	0.04	1.59
Mapk6	0.01	1.34
Sdf2l1	0.03	1.33
AABR07044421.1	0.01	1.41
Dtx3l	0.02	1.38
Olr1684	0.05	1.37
AABR07030494.1	0.01	1.35
AABR07053516.1	0.00	1.31
Skil	0.00	1.34
G3bp2	0.04	1.34
Jsrp1	0.00	1.67
Cxcl16	0.01	1.58
Atp6v1d	0.01	1.31
Actn1	0.02	1.34
Eef1g	0.01	1.32
Ccdc96	0.03	1.44
Arl6ip4	0.00	1.35
Rogdi	0.04	1.32
Cd164	0.03	1.50
Ccdc17	0.04	1.34
AABR07043101.1	0.04	1.42
Syngr2	0.01	1.35
Hoxb7	0.02	1.35
Dnajb4	0.01	1.37
AABR07042326.2	0.03	1.76
Phlda3	0.03	1.39
Ifi44	0.03	1.63
Pla2g15	0.04	1.39
AC116236.2	0.03	1.34
Cdkn1a	0.00	2.13
Tgfb2	0.01	1.55
AABR07015941.2	0.02	1.39
Tob1	0.03	1.53
RF00614	0.04	1.56
Slc35c1	0.02	1.32
Hspb2	0.02	1.49
Parp9	0.03	1.38

AY172581.24	0.02	2.17
AABR07013798.1	0.02	1.53
Gadd45a	0.02	1.67
Itga7	0.00	1.67
Lamp1	0.03	1.35
Tmem101	0.01	1.36
Abhd5	0.05	1.32
Bag2	0.00	1.34
Cryab	0.02	1.48
Ifi30	0.02	1.34
Tmem199	0.00	1.36
Mdm2	0.00	1.37
P2rx4	0.01	1.37
Mob4	0.02	1.32
Phlda2	0.01	1.40
AABR07029863.1	0.04	2.09
Tmem150a	0.01	1.95
AABR07062136.1	0.04	1.41
Bloc1s3	0.02	1.32
Actg2	0.05	1.48
Chsy1	0.01	1.35
AABR07000159.2	0.03	1.44
Stbd1	0.04	1.48
Chfr	0.04	1.34
Parp14	0.03	1.39
Edf1	0.01	1.30
Rpp38	0.03	1.34
Siglec10	0.01	1.37
Casq2	0.05	1.43
Des	0.02	1.47
Cfl2	0.03	1.31
Prss23	0.02	1.41
RF01294	0.00	1.31
AABR07038926.1	0.01	1.35
Aen	0.00	1.46
RF00265	0.01	1.89
Ankrd1	0.03	1.64
Trim25	0.01	1.50
Mir615	0.02	2.11
Ccdc47	0.04	1.45
Ins2	0.04	2.17
Cnpy4	0.05	1.31
AABR07029955.1	0.01	1.35
Bhlhe41	0.02	1.57
AABR07026893.1	0.05	1.35
B4galt5	0.04	1.45
AC128792.2	0.02	1.82
Slc20a2	0.01	1.32
Hacd1	0.00	1.33
Myod1	0.03	1.35
Ap5b1	0.00	1.32
AY172581.18	0.00	1.73
Rhob	0.02	1.57
Icam1	0.01	1.35

Zfp513	0.04	1.31
Dusp4	0.02	1.41
AY172581.10	0.03	2.26
Wnt4	0.03	1.69
Cadm4	0.01	1.64
Slc38a7	0.01	1.50
Tmem38b	0.02	1.41
Tusc2	0.00	1.44
Cdc34	0.02	1.40
AABR07044273.1	0.01	1.41
AC114363.1	0.02	1.36
Plin2	0.03	1.49
Klhl9	0.04	1.42
Nagpa	0.03	1.33
Tspan13	0.02	1.40
Gm23880	0.04	1.66
Plk2	0.01	1.56
Pmepa1	0.01	1.47
Lgals3bp	0.01	2.32
AC118419.1	0.05	1.50
AC133400.1	0.04	1.31
Fam214b	0.04	1.51
Kcnf1	0.01	1.31
Neurl2	0.02	1.32
AABR07060293.1	0.01	0.60
Rbmx	0.00	0.71
Ncapg2	0.02	0.75
Rad21	0.01	0.73
Ip6k1	0.01	0.76
Stil	0.01	0.75
AABR07013288.4	0.01	0.66
Ccnf	0.02	0.75
Mcm10	0.02	0.71
Prim1	0.03	0.71
Kpna2	0.04	0.75
LOC100359600	0.01	0.69
Nans	0.00	0.71
Asf1b	0.01	0.66
Iqgap3	0.04	0.69
Lfng	0.00	0.62
Mir3564	0.01	0.76
AABR07039338.1	0.02	0.73
AABR07044574.1	0.01	0.67
Ncaph2	0.02	0.71
Hyls1	0.02	0.73
Ska1	0.04	0.72
Ska3	0.00	0.67
AC141526.2	0.02	0.62
Usp1	0.00	0.66
Mybl2	0.04	0.69
Ckap2l	0.02	0.71
Selenoh	0.01	0.72
Nhlrc3	0.00	0.75
Ccna2	0.03	0.67

Cenph	0.04	0.64
Cenpf	0.00	0.68
AABR07039210.2	0.00	0.69
LOC317456	0.00	0.73
Cks1b	0.02	0.72
Ncapd2	0.00	0.62
Atad2	0.04	0.76
Kif11	0.01	0.67
Rrm1	0.03	0.69
Pclaf	0.01	0.62
Prc1	0.00	0.61
Cenpt	0.03	0.59
Pbk	0.02	0.67
Shcbp1	0.04	0.76
Hmmr	0.02	0.71
Hjurp	0.02	0.69
AABR07026311.1	0.01	0.60
Cdc25b	0.01	0.66
Plk4	0.01	0.65
AC095390.1	0.01	0.60
Pole	0.02	0.75
AC112350.1	0.03	0.63
Slc25a10	0.02	0.72
Mid1ip1	0.02	0.74
Tcf19	0.00	0.59
Ccnb1	0.00	0.53
Socs1	0.00	0.74
AABR07005838.1	0.04	0.68
Racgap1	0.01	0.75
Aspm	0.01	0.65
AABR07072761.1	0.03	0.77
Lsm2	0.01	0.63
Mras	0.00	0.76
Lgals3	0.00	0.64
Ucp2	0.01	0.69
Zfp395	0.02	0.76
Ndc80	0.02	0.71
Kif20a	0.01	0.67
Tpx2	0.01	0.68
Mad2l1	0.03	0.68
Rps6ka4	0.00	0.75
Tgfb3	0.00	0.75
Bub1b	0.02	0.75
Arf5	0.00	0.68
Rpa2	0.01	0.70
Aurka	0.00	0.63
Aurkb	0.00	0.60
Edem1	0.02	0.72
Ndrg1	0.01	0.64
Ubald2	0.03	0.71
Top2a	0.01	0.58
Eme1	0.00	0.64
Spc25	0.01	0.69
AABR07000658.1	0.01	0.65

Lmnb2	0.03	0.74
Lmnb1	0.02	0.67
RF00288	0.01	0.57
Nono	0.02	0.72
Timeless	0.04	0.75
S100a10	0.00	0.61
Kntc1	0.02	0.74
Nup85	0.03	0.71
Jpt1	0.01	0.74
Tedc1	0.02	0.67
Bok	0.01	0.75
AC127106.1	0.02	0.75
Rfwd3	0.02	0.74
AY172581.22	0.04	0.56
Rnase17	0.04	0.74
Nrm	0.02	0.74
Hist1h1t	0.00	0.77
AABR07062915.2	0.05	0.75
Haspin	0.02	0.74
Rps6kb1	0.00	0.74
Pycr2	0.04	0.69
Ttk	0.01	0.70
Cdk1	0.01	0.61
AC130741.1	0.01	0.64
Dtymk	0.00	0.66
Arhgap11a	0.01	0.67
Ncaph	0.01	0.68
Ncapg	0.00	0.67
AC109877.1	0.01	0.67
Uhrf1	0.02	0.63
LOC100910678	0.04	0.73
Kifc1	0.02	0.67
Mki67	0.01	0.63
LOC499331	0.01	0.59
Chst12	0.01	0.71
Tnfaip8l1	0.01	0.70
Nuf2	0.02	0.75
Smc4	0.01	0.73
Haus4	0.01	0.74
RF00438	0.01	0.64
Siva1	0.02	0.72
Psat1	0.00	0.66
RF00594	0.01	0.63
Tymp	0.01	0.71
Pycard	0.00	0.60
Arl2bp	0.02	0.40
Dut	0.01	0.70
Mcm6	0.04	0.72
AABR07035539.1	0.05	0.77
Slbp	0.01	0.70
Ptma	0.04	0.74
Ect2	0.03	0.74
Sgo1	0.03	0.76
Mir33	0.03	0.58

Tlr5	0.00	0.72
Prdx2	0.04	0.73
Rfc5	0.01	0.73
AABR07028352.1	0.04	0.72
Idh2	0.01	0.70
Foxm1	0.00	0.61
Wdhd1	0.01	0.69
Necab3	0.02	0.74
Knstrn	0.01	0.67
Gins3	0.02	0.72
Nasp	0.01	0.71
Cchcr1	0.04	0.75
AABR07039153.2	0.01	0.69
Incenp	0.02	0.72
Ccne2	0.02	0.74
Troap	0.04	0.64
Tm4sf1	0.02	0.73
AC120066.1	0.01	0.75
Tk1	0.01	0.59
AABR07011698.1	0.04	0.60
Rasl2-9	0.04	0.76
Dek	0.01	0.68
Rfc3	0.01	0.73
Fancd2	0.02	0.76
Itpr3	0.04	0.70
Pttg1	0.02	0.64
AABR07045487.1	0.00	0.60
Tspo	0.01	0.71
AABR07041724.1	0.00	0.54
Nusap1	0.00	0.66
Car5b	0.02	0.76
H3f3c	0.03	0.71
Cggbp1	0.00	0.76
Anln	0.02	0.77
Lix1l	0.01	0.71
Cdc20	0.01	0.57
Sfrp2	0.04	0.57
Mt1m	0.02	0.66
AABR07018792.1	0.00	0.61
Fbxo5	0.04	0.71
Nanos1	0.05	0.62
Mir1956	0.02	0.60
Igfbp6	0.00	0.66
Mcm3	0.04	0.69
AC135310.2	0.05	0.61
Fgf22	0.04	0.72
Htra1	0.03	0.73
LOC100362333	0.01	0.73
Cdkn2c	0.00	0.76
Srsf7	0.01	0.74
Spag5	0.01	0.66
Lmf2	0.03	0.74
Orc6	0.02	0.73
Ube2c	0.00	0.59

H2afx	0.03	0.63
H2afz	0.03	0.61
Sohlh1	0.00	0.50
Cdca3	0.01	0.58
Grwd1	0.00	0.46
Mcm4	0.05	0.69
Espl1	0.03	0.72
Mcm2	0.01	0.66
Ube2s	0.05	0.71
Bub1	0.00	0.63
Cdca8	0.02	0.74
Galnt1	0.01	0.69
AABR07049578.1	0.02	0.70
Fam168b	0.00	0.66
AC123095.1	0.02	0.76
Tuba1b	0.03	0.69
Smc2	0.02	0.69
Tpm3	0.00	0.75
Rpa3	0.02	0.68
Kif23	0.05	0.77
Kif22	0.01	0.64
RGD1560010	0.03	0.76
Anp32e	0.01	0.65
LOC100364062	0.02	0.69
Cenpe	0.01	0.71
Carhsp1	0.01	0.63
Tacc3	0.01	0.65
Nsmce4a	0.01	0.75
Mmp11	0.03	0.69
Plk1	0.01	0.63
Dnmt1	0.04	0.72
Ids	0.01	0.76
Pimreg	0.01	0.63
Ykt6	0.00	0.76
AABR07030183.1	0.03	0.69
Glt8d1	0.03	0.76
Stmn1	0.00	0.51
E2f8	0.01	0.67
AABR07043167.1	0.03	0.69
AC110690.1	0.00	0.31
Pygo2	0.04	0.73
Col18a1	0.00	0.75
Hmgb2	0.04	0.70
Birc5	0.03	0.59
Aida	0.01	0.74
LOC100359539	0.01	0.62
LOC102547056	0.01	0.69
Smc1a	0.01	0.69
AABR07030866.1	0.02	0.62
Kif2c	0.01	0.69
Dlgap5	0.03	0.75
Smarcd2	0.03	0.71
AABR07011697.1	0.01	0.73
Fanci	0.02	0.76

TABLE S4E Top altered genes in KMM cells following WDR5 knockdown

Gene	P-Value	Fold Change (shRNA/WT)
Plekhg2	0.03	1.44
Cyp1b1	0.01	2.82
Rasl11b	0.00	1.50
AABR07068285.2	0.01	1.54
Rnf44	0.01	1.44
Zfp846	0.00	1.33
Pelo	0.00	1.68
Tspan5	0.01	1.34
Msln	0.00	2.49
Kdm5b	0.01	1.38
Tob1	0.00	1.55
Rnf185	0.00	1.32
RF00492	0.05	1.44
Mir3064	0.03	1.56
Hbp1	0.00	1.69
Slc39a9	0.00	1.30
B2m	0.00	1.44
Klf2	0.03	2.25
Zwint	0.01	3.49
Gpx8	0.01	1.51
Homer3	0.01	1.62
Cnppd1	0.00	1.33
Slc39a7	0.03	1.31
Ccn4	0.00	2.54
AABR07028970.1	0.03	1.74
Ccn1	0.02	2.41
Ccn2	0.01	2.06
AABR07063829.2	0.04	1.52
Nap1l3	0.00	1.73
Cstf2	0.01	1.35
Zfp219	0.04	1.30
Col1a1	0.04	1.53
AABR07044421.1	0.00	1.40
Mir365-1	0.01	1.56
Gpr146	0.01	1.32
AABR07057250.1	0.00	1.69
Fcho2	0.00	1.31
Ptprf	0.00	1.59
Eci1	0.02	1.30
RGD1560108	0.01	1.60
Itgb1bp1	0.00	1.35
Ugt1a1	0.05	1.32
Tyro3	0.00	1.52
Impact	0.00	1.76
Wipi1	0.00	1.32
RGD1309748	0.00	1.55
Hexb	0.00	1.33
AABR07024972.1	0.04	1.36
Syde1	0.00	2.11
AABR07044959.1	0.00	1.59
Synj1	0.00	1.37
RF00322	0.03	1.75

AABR07069433.1	0.01	1.57
Gprc5a	0.02	1.57
Bcl2l1	0.00	1.31
Dnajb9	0.00	1.62
Trafd1	0.00	1.41
Btg2	0.02	2.43
Zfp266	0.00	1.32
Fam3a	0.02	1.42
Zswim4	0.00	1.62
Scpep1	0.04	1.40
AC120486.3	0.01	1.33
Cdkn1b	0.00	1.31
Mme	0.00	1.36
LOC102556092	0.00	1.41
AC142458.1	0.00	1.62
Ajuba	0.00	1.84
Nisch	0.00	1.50
AC120486.9	0.00	1.40
Tap1	0.00	1.72
AABR07025787.1	0.03	1.37
Tnrc18	0.00	1.45
Ccdc80	0.03	2.06
Aga	0.02	1.41
Gga2	0.01	1.47
Sorbs3	0.00	1.34
Mxd4	0.01	1.81
Mxd1	0.01	1.37
Pnrc1	0.00	1.78
Lama5	0.00	1.42
Zbtb4	0.00	1.53
AABR07029605.1	0.03	1.34
Rras2	0.00	1.37
Ahr	0.01	1.51
AABR07027575.1	0.01	2.71
LOC100125364	0.00	1.45
Atp2b1	0.00	1.33
N4bp1	0.01	1.34
Pus3	0.01	1.46
Pkp1	0.03	1.40
Mtss1l	0.01	1.40
Tent5a	0.00	1.56
Zfp964	0.01	1.47
Clip2	0.00	1.31
Zfp292	0.00	1.33
Tmem185b	0.00	1.69
P2rx4	0.01	1.70
Mir222	0.02	2.96
Etv3	0.00	1.32
Mir221	0.03	1.90
Klhl31	0.00	1.73
AABR07063581.2	0.00	2.35
Ier5	0.00	1.73
Nuak2	0.01	1.83
Jund	0.03	1.61

Ier3	0.02	1.81	
Rab11fip5	0.00	1.35	
Plcg1	0.00	1.48	
H3f3b	0.01	1.31	
Sh3bp2	0.00	1.37	
Tead3	0.00	1.34	
AC121413.2	0.04	1.47	
Tead4	0.00	1.32	
Prrg4	0.03	1.36	
Cep170b	0.00	1.49	
AC119762.3	0.01	1.37	
AC119762.7	0.01	2.07	
Arhgap23	0.01	1.34	
Ggt1	0.03	2.27	
Cfl2	0.00	1.73	
Wdfy1	0.00	1.34	
LOC691170	0.00	1.35	
Mir196c	0.02	1.60	
Arhgap29	0.00	1.32	
Phldb3	0.00	1.40	
Serpine1	0.05	3.30	
F3	0.04	1.59	
Timp2	0.00	1.84	
Timp1	0.00	1.32	
Sema3b	0.02	2.06	
Sh3gl1	0.00	1.43	
Eva1b	0.04	1.44	
Efna4	0.01	1.34	
Creb3	0.01	1.46	
Serpinb6a	0.01	1.73	
Clcf1	0.01	1.53	
Ripor1	0.00	1.31	
Slc27a4	0.00	1.85	
B4galt5	0.02	1.57	
Dyrk1b	0.04	1.68	
Slc35f5	0.01	1.56	
Cavin2	0.05	1.60	
Glb1l2	0.01	1.37	
AABR07030603.2	0.01	1.67	
AABR07030603.1	0.01	1.51	
RF00024	0.01	1.55	
RF00072	0.03	1.42	
AABR07032751.1	0.01	1.74	
Slfn2	0.00	1.49	
Dbn1	0.00	1.72	
Kctd21	0.00	1.70	
AABR07059891.1	0.00	1.46	
Lzts3	0.00	1.48	
Stk40	0.00	1.38	
Vat1	0.00	1.42	
Sesn2	0.00	2.23	
Limk2	0.00	1.33	
Ppic	0.03	1.47	
Tsc22d3	0.04	1.93	

Tsc22d2	0.01	1.47
Tpm1	0.04	2.07
Tcn2	0.03	2.24
Sparc	0.00	2.62
Ptgs2	0.04	2.00
Mxra8	0.01	1.36
Eddm3b	0.05	1.30
Ncam1	0.00	1.38
Mmp19	0.02	1.94
Slc12a4	0.01	1.41
Adrb2	0.04	1.94
Kdm6b	0.03	1.74
AC118419.1	0.00	1.88
Klhdc3	0.01	1.42
Osbpl5	0.00	1.59
Ugcg	0.03	1.35
Tusc2	0.01	1.60
Osgin1	0.00	1.36
Cry2	0.00	1.47
AABR07047011.1	0.00	1.36
Tsc22d1	0.00	1.97
Wbp1l	0.00	1.40
Snx33	0.01	1.37
AABR07068150.1	0.04	1.46
Hapln3	0.00	1.43
Nfat5	0.02	1.50
Pcgf2	0.01	1.30
Ktn1	0.00	1.38
Chac1	0.00	1.85
Sdcbp	0.01	1.77
Tdg	0.00	1.97
Avpi1	0.00	1.33
Upk3b	0.00	1.94
Chmp1a	0.00	1.32
Trim34	0.00	1.59
Asap1	0.00	1.48
Snapin	0.00	1.46
Anxa1	0.00	1.55
Anxa3	0.00	2.23
Irf7	0.05	1.51
Pld3	0.04	1.55
Irf9	0.00	1.45
Dhrs13	0.00	1.38
Flrt3	0.00	1.38
Sh2d4a	0.00	1.43
Stard3	0.00	1.32
Lamb2	0.01	1.34
LOC290595	0.02	1.36
Cyp1a1	0.02	3.00
Pgrmc2	0.00	1.31
Pgrmc1	0.00	1.49
LOC686013	0.01	1.33
Tpp1	0.04	1.74
LOC102557137	0.01	1.43

Bax	0.00	1.31
Uba5	0.00	1.35
Cmtm3	0.01	1.89
AABR07027811.2	0.02	1.49
AABR07027811.3	0.00	1.47
Cttnbp2nl	0.00	1.32
AABR07033047.1	0.01	1.46
Sema3c	0.00	1.49
Bsg	0.01	1.32
Inpp5d	0.00	1.33
Vegfd	0.03	1.43
Bod1	0.00	1.41
Gpnmb	0.05	1.52
Pqlc2	0.01	1.41
AABR07053516.1	0.00	1.35
Cst3	0.01	1.81
Plekho1	0.01	1.35
Abhd4	0.00	1.76
Atp6v1d	0.00	1.62
AABR07035819.1	0.01	1.45
AABR07071000.1	0.00	1.59
Tp53inp2	0.00	1.61
Retreg3	0.00	1.55
Pias3	0.00	1.39
Marcks	0.00	1.80
Pcolce	0.00	1.54
Rnf24	0.00	1.51
Flnc	0.01	1.87
AABR07018321.3	0.02	1.50
Yod1	0.01	1.53
Bak1	0.00	1.67
AABR07043829.1	0.04	1.30
AC103090.1	0.00	1.60
Serpinb7	0.00	1.65
Hsdl1	0.00	1.35
Phf1	0.01	1.73
Pla2g15	0.02	1.64
Zfp703	0.00	1.31
Tnpo1	0.00	1.43
Hoxa1	0.02	1.31
Reep3	0.00	1.77
Pdcd2	0.00	1.36
Chchd7	0.00	1.30
Hspb2	0.01	1.50
Zfp385a	0.02	1.39
App	0.00	1.58
AC141152.1	0.04	1.43
Gpr88	0.03	1.65
Lamp2	0.01	1.42
Lamp1	0.01	1.53
Myof	0.00	1.36
Mirlet7d	0.02	1.70
Synpo	0.02	1.34
Msmp	0.01	1.35

Gtpbp2	0.03	1.32
Ergic3	0.01	1.32
Tmem69	0.00	1.39
Il6r	0.00	1.32
LOC684773	0.03	1.46
Ldb1	0.00	1.38
Tp53inp1	0.00	3.47
Glb1l	0.01	1.51
Clu	0.01	1.55
Bdh2	0.04	1.37
Apol9a	0.00	1.49
Ephx1	0.01	1.30
Traf4	0.00	1.69
LOC102547645	0.03	1.30
AC123425.1	0.03	1.53
Btbd19	0.00	1.75
Dusp18	0.03	1.31
Ndel1	0.00	1.42
Zscan25	0.00	1.33
Sft2d3	0.00	1.36
Sft2d1	0.00	1.36
Itm2c	0.01	1.51
RF00586	0.04	1.57
Nacc2	0.00	1.34
Mir22	0.04	2.06
Sema4b	0.01	1.51
Giot1	0.03	1.40
LOC102553785	0.03	1.58
Klhl24	0.04	1.59
Pgpep1	0.01	1.31
AABR07060291.1	0.01	1.51
AABR07067042.2	0.03	1.48
Kprp	0.03	1.86
AABR07030911.2	0.02	1.48
Mapre3	0.01	1.54
AC119015.4	0.00	1.87
Slc20a2	0.00	1.60
Hmox1	0.00	2.24
Fstl1	0.00	1.43
Atg12	0.01	1.62
Dusp8	0.00	1.55
Blcap	0.00	1.84
Dusp6	0.01	1.45
Tnfrsf12a	0.00	1.50
Dcbld2	0.02	1.44
Rnf166	0.04	1.34
Mfge8	0.00	1.51
Adamtsl5	0.01	2.04
Metrnl	0.00	1.52
Mllt11	0.00	1.56
Adam19	0.00	1.52
Akr1b1	0.01	1.37
AABR07002711.1	0.00	1.62
Slc5a6	0.00	1.32

Maml1	0.00	1.30
Ikbip	0.00	1.40
Hyal2	0.00	1.40
Cdc42ep1	0.04	1.49
Plk2	0.00	4.25
Pdrg1	0.02	1.33
Col6a1	0.00	1.52
Col6a2	0.02	1.38
RF00582	0.02	2.14
Gjb5	0.04	1.56
Gjb4	0.01	1.57
Glrx3	0.00	1.35
AABR07058464.1	0.00	1.43
Cc2d1a	0.04	1.32
Lasp1	0.03	1.36
Pawr	0.00	1.33
AABR07072559.2	0.00	1.42
Slc39a13	0.02	1.36
Pdlim7	0.00	1.39
Pdlim1	0.00	1.36
Kif1c	0.00	1.37
Gprin3	0.00	1.72
Svbp	0.00	1.45
Reck	0.01	1.32
Cpt1c	0.01	1.42
Rab23	0.01	1.31
Mir146b	0.01	1.31
Lgmn	0.01	1.52
Ddr1	0.00	1.82
C1qtnf5	0.05	1.40
Ccng1	0.02	1.56
Ndfip1	0.01	1.33
Sptlc2	0.01	1.31
Terf2ip	0.01	1.36
Mybl1	0.00	1.80
Rhob	0.04	1.94
AABR07003344.1	0.02	1.48
Rhod	0.02	1.54
Midn	0.00	1.87
AABR07027010.1	0.03	1.46
Gnptg	0.03	1.35
Slc2a5	0.01	1.49
AABR07005031.1	0.01	1.71
Tmem59	0.00	1.48
Cd63	0.00	1.34
Atxn7l3b	0.00	2.13
AC095678.1	0.00	2.07
Tvp23b	0.00	1.52
AABR07026805.2	0.01	1.38
Ppp1r13b	0.00	1.32
Pik3ip1	0.03	1.38
Ppp1r13l	0.00	1.56
Mafg	0.01	1.33
Fnip2	0.00	1.31

Lpin3	0.01	1.39
Src	0.01	1.47
Rtn4	0.00	1.59
Trip6	0.00	1.48
RGD1308117	0.00	1.58
Ubtd1	0.00	1.31
Fosl2	0.01	1.41
AC120262.2	0.01	1.32
RF00553	0.01	1.73
Itgax	0.03	1.43
Mcrip1	0.00	1.52
Zmat3	0.00	1.36
Ip6k2	0.00	1.34
Lig4	0.00	1.68
Zfr	0.00	1.38
AABR07044375.2	0.00	1.79
LOC103694328	0.00	1.51
AABR07044375.1	0.00	2.66
Gata3	0.00	1.38
Cldn12	0.00	1.33
Wls	0.00	1.71
Slc10a5	0.01	1.55
Vps26b	0.00	1.32
Nfe2l2	0.00	1.39
Grina	0.00	1.32
Slc4a2	0.00	1.50
Plekhb2	0.00	1.75
Tmem263	0.04	1.32
AC130391.5	0.00	1.50
Gfpt1	0.03	1.50
AC130391.1	0.04	1.47
Axl	0.01	2.23
G3bp2	0.00	1.44
Itga5	0.01	1.47
Itga7	0.00	1.49
Ubqln2	0.00	1.93
Fkbp3	0.00	1.31
Numbl	0.00	1.70
Fkbp9	0.01	1.37
Nckap5l	0.01	1.56
Adamts7	0.03	1.49
Rgl2	0.00	1.31
Tmem132a	0.02	1.42
Gigyf1	0.01	1.58
Tmem106a	0.05	1.38
Cryab	0.00	3.49
Adam8	0.01	1.44
Emc10	0.03	1.30
Hist1h2bd	0.03	1.51
AC099453.2	0.01	1.31
Aktip	0.00	1.47
Limd2	0.00	1.56
Slc35d2	0.00	1.33
Lox	0.00	1.78

Dipk1a	0.00	1.30	
AABR07006269.1	0.02	1.31	
Gja1	0.03	1.36	
Pmm1	0.00	1.47	
Gja4	0.04	2.21	
Mfsd1	0.02	1.36	
Pea15	0.00	1.66	
Tjp1	0.00	1.48	
Sgms2	0.01	1.48	
Pid1	0.00	1.33	
Gdf15	0.04	2.48	
Tnnc1	0.03	2.03	
Sypl1	0.00	1.55	
AABR07062477.2	0.00	1.62	
AABR07053152.1	0.00	1.42	
Ankrd49	0.00	1.56	
Lrrc32	0.00	1.36	
RGD1562378	0.00	2.17	
Sh3glb1	0.00	1.33	
Ypel5	0.00	1.40	
Mprip	0.00	1.40	
Mk1	0.05	1.40	
Abcc5	0.00	1.39	
Myrf	0.01	1.47	
Aen	0.00	1.79	
Orai3	0.00	1.33	
Tmem127	0.00	1.41	
Car9	0.04	1.58	
Scarf2	0.01	1.37	
Car1	0.04	2.74	
Bcat1	0.00	1.50	
Ftl1	0.00	2.00	
LOC691807	0.03	1.39	
Scrn1	0.00	1.52	
Itga3	0.00	1.39	
Cuedc1	0.00	1.43	
Riok3	0.00	1.33	
Rflnb	0.02	2.13	
Ptp4a2	0.00	1.39	
AABR07031734.15	0.04	1.65	
AABR07010705.1	0.00	1.55	
Tnc	0.02	1.68	
Ppif	0.00	1.30	
Plag1	0.00	1.59	
Map1a	0.02	1.80	
Mir193a	0.01	1.39	
AABR07053136.1	0.00	1.67	
Dstn	0.00	1.49	
Lrfn4	0.00	1.35	
Btn2a2	0.00	1.66	
Rnf19a	0.00	1.45	
AABR07030366.1	0.02	1.32	
Nkiras2	0.01	1.48	
Adipor1	0.00	1.36	

AC141028.1	0.00	1.55	
Nras	0.01	1.36	
Rassf7	0.00	1.62	
Fermt2	0.00	1.34	
Zcchc3	0.03	1.46	
Fst	0.01	2.45	
Sphk1	0.00	2.12	
Tmem63b	0.00	1.45	
Fam214b	0.01	2.03	
Ccdc43	0.00	1.32	
Kifc3	0.01	1.78	
Dvl2	0.00	1.41	
Dvl3	0.00	1.37	
B3galt4	0.01	1.33	
Dync1li2	0.00	1.39	
Sec22b	0.01	1.35	
Gabarapl1	0.01	1.38	
Psap	0.01	1.59	
Gab1	0.00	1.36	
Sp110	0.00	1.32	
RGD1565616	0.00	1.38	
Slc19a2	0.00	1.87	
RF00158	0.05	1.47	
Tmbim1	0.00	1.73	
Flcn	0.03	1.44	
LOC498368	0.05	1.71	
Tmem41a	0.02	1.46	
Ctsa	0.02	1.73	
Ctsb	0.00	2.69	
Ctsd	0.04	1.47	
Tent5b	0.00	2.27	
Tceanc	0.02	1.32	
AABR07044635.1	0.04	2.43	
Siglec10	0.00	2.56	
Col3a1	0.02	2.42	
Gaa	0.04	2.27	
AC130232.1	0.01	1.44	
AABR07021988.1	0.00	1.40	
AC130232.2	0.00	1.32	
Nbas	0.00	1.42	
LOC689412	0.01	1.44	
AABR07039210.1	0.00	1.64	
Maf	0.03	2.31	
Zc2hc1a	0.00	1.37	
LOC689039	0.03	1.57	
Ddit3	0.01	1.30	
AABR07046628.1	0.00	1.81	
Ddit4	0.00	1.47	
Specc1	0.00	1.35	
Mirlet7c2	0.04	2.19	
Pacsin3	0.01	1.31	
Cdipt	0.00	1.43	
Dtx3l	0.00	1.39	
Hoxc8	0.00	1.40	

Fzd2	0.01	1.79	
Plscr3	0.00	1.59	
Pinlyp	0.00	1.57	
Slc6a8	0.05	1.68	
Cldnd1	0.00	1.66	
Snx18	0.01	1.85	
Mgat1	0.02	1.74	
AC134224.1	0.00	2.70	
AC134224.2	0.03	2.13	
AC134224.3	0.00	2.73	
Nagk	0.02	1.34	
Cxcl16	0.05	1.35	
Tor1aip2	0.00	1.32	
Necap1	0.00	1.55	
Rogdi	0.01	1.80	
Unc119	0.00	1.89	
Npnt	0.02	1.42	
Zfand5	0.00	1.33	
Cnpy4	0.00	1.79	
Ubxn4	0.00	1.31	
AC099089.1	0.00	2.09	
Mdm2	0.00	2.45	
RT1-A2	0.00	1.36	
Borcs6	0.01	1.44	
Filip1l	0.00	1.32	
Tnfrsf1a	0.01	1.36	
Col5a2	0.00	1.73	
Col5a1	0.00	1.73	
Cdc42ep2	0.00	1.63	
Kdelr3	0.00	1.37	
Plk3	0.00	2.67	
Rap2b	0.00	1.35	
Rab11fip1	0.00	1.52	
Amotl2	0.00	2.03	
Lmbr1l	0.01	1.42	
Lrig1	0.03	1.53	
Sqstm1	0.02	1.72	
Cd80	0.00	1.40	
Cdkn1a	0.00	2.71	
Cltb	0.00	1.41	
Tiparp	0.00	1.64	
Egfl7	0.04	1.42	
Rap2a	0.00	1.33	
AABR07044388.2	0.01	1.31	
Txnip	0.02	2.05	
Laptm4a	0.00	1.34	
AABR07043557.1	0.00	1.34	
Samd9	0.00	1.57	
Ccdc9b	0.00	2.33	
Tagln	0.01	3.07	
AABR07030861.1	0.00	1.45	
AY172581.20	0.02	2.03	
Otulinl	0.00	1.34	
AY172581.24	0.02	1.43	

Hist1h1t	0.02	1.44	
Gadd45a	0.01	3.78	
Gadd45g	0.00	2.08	
AABR07001433.1	0.01	1.36	
Me1	0.01	1.40	
Plcd3	0.00	1.31	
Hist1h1d	0.00	2.38	
Klc2	0.00	1.43	
Nucb1	0.00	1.47	
F11r	0.00	1.31	
Hist1h1c	0.00	2.49	
Slc25a44	0.00	1.43	
Gbp2	0.00	1.46	
Sh3bp5l	0.02	1.33	
AABR07030019.1	0.01	1.45	
Pik3c2a	0.00	1.33	
Adm	0.03	1.37	
Lpar6	0.00	1.76	
Phlda3	0.00	2.15	
Ankzf1	0.02	1.30	
Il1rl1	0.02	1.63	
Epha7	0.00	1.30	
Epha2	0.01	1.57	
Atrn	0.00	1.78	
Lats2	0.01	1.42	
Col4a5	0.00	1.49	
RT1-S3	0.00	1.35	
Gmip	0.02	1.36	
Rnf145	0.00	1.40	
Arl4a	0.01	1.42	
Ptpn23	0.00	1.51	
Loxl4	0.00	1.40	
Ifrd1	0.02	1.76	
Pomgnt2	0.03	1.44	
Chmp4c	0.00	1.32	
LOC292543	0.00	1.41	
P4ha2	0.00	1.45	
Sowahc	0.00	2.34	
AABR07055539.2	0.01	1.45	
AABR07030200.1	0.01	2.16	
Ifngr2	0.00	1.42	
Resf1	0.00	1.81	
Tbc1d17	0.04	1.38	
AABR07014756.1	0.00	1.84	
LOC100294508	0.01	1.33	
Epgn	0.04	1.64	
Trim25	0.00	1.56	
Tef	0.02	1.38	
Fhl2	0.01	1.37	
Fhl1	0.01	2.30	
Isg15	0.03	1.48	
Egln2	0.00	1.32	
Zyx	0.03	1.41	
AABR07006111.1	0.00	1.45	

Lypla1	0.01	1.33
Mir6326	0.01	1.69
Rusc2	0.00	1.77
Vgll3	0.00	1.52
Abcd4	0.04	1.43
Zdhhc9	0.00	1.31
Taf7	0.00	1.31
Zfp512b	0.01	1.43
Psca	0.01	2.02
Nlgn2	0.02	1.33
Clba1	0.02	1.32
Snn	0.00	1.64
Slc9a3r2	0.03	1.33
Chpf2	0.00	1.32
Kmt5a	0.00	1.32
Npepps	0.00	1.52
Rapsn	0.04	1.43
AABR07044273.1	0.01	1.37
Rnd1	0.01	1.78
Rnd3	0.00	2.58
RGD1561149	0.00	1.47
Phospho1	0.05	1.39
LOC252890	0.01	2.08
Jtb	0.00	1.34
Myo10	0.01	1.31
AABR07072236.1	0.00	1.40
RF00405	0.04	1.51
Dtx3	0.03	1.76
LOC100912365	0.03	1.31
AABR07071779.2	0.00	1.36
Pomt2	0.00	1.41
Podnl1	0.04	1.32
AABR07006081.1	0.00	1.69
Bmp1	0.00	1.52
Tinagl1	0.00	1.95
Il6st	0.00	1.65
Ppp1r15a	0.00	1.79
Fat1	0.03	1.36
Baz2a	0.00	1.32
LOC100365363	0.00	1.46
Setd7	0.00	1.45
Dpp7	0.01	2.11
Tsn	0.00	0.75
Kcnh6	0.00	0.52
AABR07030156.2	0.00	0.65
Rad1	0.02	0.72
Gas2l3	0.00	0.58
Cep55	0.00	0.56
Gpank1	0.00	0.74
Kpna6	0.00	0.75
Ccnf	0.02	0.66
Kpna3	0.00	0.76
Kpna2	0.01	0.68
Pttg1	0.00	0.50

Asf1b	0.00	0.41	
Ticrr	0.00	0.67	
Hnrnpa1	0.01	0.72	
Siva1	0.00	0.60	
Stn1	0.00	0.77	
Ska1	0.00	0.53	
AABR07042875.1	0.00	0.72	
AABR07057683.1	0.00	0.59	
Mbd3	0.01	0.67	
AABR07062599.1	0.03	0.64	
Ppp5c	0.00	0.69	
Tacc3	0.00	0.51	
Cks1b	0.00	0.54	
Ncapd2	0.00	0.51	
Ncapd3	0.00	0.66	
Akt1	0.02	0.72	
Aunip	0.00	0.68	
Dctpp1	0.00	0.64	
Rrm2	0.02	0.59	
Rrm1	0.00	0.47	
Cebpa	0.00	0.49	
Cebpg	0.00	0.67	
AABR07026311.1	0.00	0.50	
AC112350.1	0.00	0.69	
Tnip3	0.03	0.75	
AABR07040892.1	0.00	0.77	
LOC102546716	0.04	0.73	
AABR07044366.1	0.02	0.57	
Ccnb1	0.00	0.46	
Dpysl2	0.00	0.65	
Pradc1	0.00	0.76	
Palb2	0.00	0.72	
RF00324	0.02	0.60	
Arl6ip1	0.00	0.57	
AABR07029613.1	0.01	0.70	
AABR07047089.1	0.00	0.52	
Shc1	0.00	0.56	
AABR07057237.1	0.04	0.63	
LOC301444	0.04	0.59	
Brca2	0.00	0.69	
Snrpd1	0.00	0.75	
AABR07047219.1	0.00	0.58	
Taldo1	0.00	0.73	
LOC689065	0.04	0.63	
Pif1	0.04	0.77	
Exd2	0.01	0.61	
Pclaf	0.00	0.45	
Xrcc2	0.00	0.63	
Spc25	0.00	0.55	
Spc24	0.01	0.41	
Cep250	0.00	0.71	
Shcbp1	0.01	0.66	
Krtcap2	0.01	0.76	
Klhl41	0.01	0.75	

Pole4	0.00	0.70	
Pole2	0.00	0.73	
Tubgcp2	0.00	0.63	
Apold1	0.00	0.54	
Mbtps1	0.01	0.75	
LOC100362400	0.00	0.66	
Haspin	0.00	0.57	
Ttk	0.02	0.70	
Hsp90b1	0.00	0.74	
AABR07061614.1	0.00	0.75	
Xdh	0.02	0.58	
Arhgap11a	0.00	0.54	
AC142138.1	0.03	0.75	
Atp2b4	0.01	0.76	
Bora	0.01	0.69	
Clspn	0.00	0.74	
Bccip	0.00	0.71	
Klhdc8a	0.03	0.63	
AABR07039037.1	0.00	0.66	
Etv5	0.00	0.69	
Rbmxl1b	0.00	0.76	
Rad21	0.01	0.68	
Psmd7	0.02	0.73	
Ska3	0.00	0.62	
Ier2	0.05	0.69	
Orc6	0.00	0.64	
AABR07033745.1	0.03	0.75	
Ube2c	0.00	0.47	
Gins3	0.00	0.67	
Nasp	0.00	0.65	
E2f4	0.00	0.66	
Gins4	0.00	0.65	
Dtl	0.00	0.61	
Ppp2r3b	0.01	0.77	
Eif3d	0.00	0.63	
Sf3b3	0.00	0.67	
E2f8	0.00	0.57	
Plpp2	0.02	0.74	
Fancd2	0.00	0.59	
Lamtor5	0.00	0.61	
Serpine2	0.01	0.59	
Pfdn4	0.00	0.72	
Tssk6	0.00	0.64	
Rdm1	0.03	0.72	
Hlx	0.03	0.71	
Dhfr	0.00	0.69	
Fbxo5	0.00	0.41	
AABR07029955.1	0.04	0.72	
Snf8	0.03	0.74	
Ehmt2	0.01	0.73	
Tomm40l	0.00	0.66	
Rfc5	0.01	0.65	
Rfc4	0.01	0.71	
Rfc3	0.03	0.77	

Rfc2	0.00	0.59	
Rbmx2	0.00	0.73	
Ube2s	0.01	0.55	
Pcyox1	0.00	0.59	
Ddx28	0.00	0.71	
Selenoi	0.00	0.75	
Rab3d	0.01	0.71	
Ifi27l2b	0.00	0.71	
Ggh	0.01	0.71	
Hirip3	0.00	0.55	
AABR07032821.1	0.00	0.73	
Dbnl	0.01	0.73	
Rps15	0.00	0.65	
RF00421	0.04	0.72	
Krt26	0.00	0.76	
Pimreg	0.01	0.42	
Ccz1b	0.00	0.74	
Slc47a1	0.00	0.52	
Zbtb8os	0.00	0.67	
Gpsm2	0.00	0.71	
RGD1564855	0.03	0.73	
Rpl37a	0.00	0.59	
Rpl13a	0.03	0.71	
AABR07049353.1	0.01	0.74	
Birc5	0.00	0.43	
AABR07018244.2	0.02	0.66	
Alyref	0.00	0.65	
Nup43	0.00	0.73	
AC126899.1	0.01	0.57	
Slc29a4	0.00	0.60	
Pgp	0.00	0.74	
Trim47	0.00	0.70	
F2rl1	0.00	0.57	
Sapcd2	0.00	0.54	
Tspo	0.00	0.60	
Snrpf	0.01	0.74	
Oip5	0.00	0.72	
Mki67	0.00	0.47	
Usp1	0.00	0.54	
AABR07013288.4	0.00	0.57	
Melk	0.00	0.72	
Gpd1l	0.01	0.72	
Top2a	0.00	0.45	
Trmt112	0.00	0.65	
LOC100365839	0.01	0.59	
AABR07034315.1	0.00	0.62	
Pars2	0.02	0.77	
Hat1	0.03	0.76	
Rpl34	0.00	0.53	
Rpl35	0.00	0.45	
Rangap1	0.00	0.72	
Hyls1	0.00	0.53	
Rpl31	0.00	0.50	
Rad51ap1	0.00	0.57	

Ssr3	0.00	0.74	
Spp1	0.05	0.70	
Pfas	0.01	0.74	
Emg1	0.00	0.69	
Riok1	0.00	0.77	
Rad51	0.01	0.70	
Creld2	0.03	0.63	
Thbs2	0.00	0.64	
Mrps18b	0.00	0.71	
Hid1	0.00	0.74	
Exosc8	0.00	0.66	
Phgdh	0.00	0.67	
Exosc2	0.00	0.56	
Lrrcc1	0.00	0.69	
Prc1	0.00	0.51	
Ing1	0.03	0.77	
Sgpp1	0.01	0.74	
Hmmr	0.00	0.60	
Ptgs1	0.01	0.49	
Serpinb8	0.00	0.65	
Farsa	0.00	0.76	
AABR07050283.2	0.01	0.76	
Cpsf3	0.00	0.73	
Wdr5	0.00	0.40	
Depp1	0.00	0.63	
Ccdc163	0.00	0.73	
AC094647.1	0.01	0.69	
Mdc1	0.01	0.61	
Rpl7l1	0.00	0.71	
Plekha3	0.00	0.62	
Rps17	0.00	0.44	
Pelp1	0.00	0.54	
Kif20b	0.00	0.62	
Pa2g4	0.00	0.70	
Cdc6	0.03	0.60	
Kif20a	0.00	0.44	
Cip2a	0.00	0.70	
Becn1	0.01	0.75	
AC142180.1	0.01	0.76	
Ercc6l	0.00	0.53	
AABR07052430.1	0.00	0.55	
Aurka	0.01	0.61	
Aurkb	0.00	0.45	
Prr15	0.00	0.56	
Tcf19	0.00	0.46	
Slc25a11	0.00	0.67	
Slc25a10	0.01	0.50	
Kazald1	0.01	0.43	
Cnnm4	0.00	0.72	
Cspg4	0.00	0.77	
Spag5	0.00	0.54	
Rad54l	0.00	0.68	
Dazap2	0.02	0.74	
AABR07018331.1	0.03	0.76	

Ung	0.00	0.68
Reep4	0.00	0.72
AC094126.2	0.00	0.48
Dbi	0.02	0.69
Fxyd5	0.00	0.72
Rasd1	0.01	0.51
Ppp1ca	0.00	0.57
Ssrp1	0.00	0.45
Cct7	0.00	0.63
Cct4	0.00	0.72
Xkr5	0.00	0.67
Dtymk	0.01	0.71
Smc2	0.00	0.58
RGD1359290	0.00	0.73
Rpe	0.00	0.75
Ifitm2	0.00	0.72
Stil	0.00	0.64
Mcm10	0.00	0.52
Haus4	0.00	0.64
Haus5	0.00	0.70
Haus3	0.00	0.75
Fignl1	0.00	0.61
Tubg1	0.00	0.71
Pold1	0.00	0.56
Pold2	0.00	0.66
AC096239.2	0.00	0.70
Htr2a	0.00	0.75
Dut	0.00	0.56
AC094217.1	0.03	0.64
Hdac1	0.00	0.67
Ahsp	0.01	0.74
Slbp	0.00	0.69
Rps18l1	0.00	0.58
Nrm	0.00	0.60
Bard1	0.00	0.74
Ankh	0.00	0.73
Gltp	0.00	0.74
Pdia4	0.01	0.64
Arpc4	0.00	0.75
Tmem119	0.01	0.63
Emc8	0.00	0.75
Lsm5	0.01	0.74
Wdhd1	0.00	0.68
St6galnac1	0.03	0.56
Rpl22l1	0.01	0.67
AC127784.2	0.04	0.68
Lsm7	0.00	0.70
St6gal1	0.00	0.68
AABR07021759.1	0.00	0.63
Cerk	0.01	0.74
Kif4a	0.00	0.67
Taf13	0.01	0.71
Taf12	0.01	0.77
Rpl9	0.01	0.76

RGD1565498	0.02	0.76
AC129365.1	0.00	0.54
Slc35b1	0.00	0.73
Set	0.01	0.75
LOC361346	0.02	0.76
U2af1	0.00	0.75
Ranbp1	0.02	0.71
H1fx	0.04	0.52
Paqr4	0.01	0.59
Cdc20	0.00	0.41
Srebf2	0.01	0.66
Psmc5	0.01	0.77
Zmat2	0.00	0.77
Mob1a	0.01	0.73
Ndufc1	0.02	0.76
AC103024.1	0.01	0.58
Eef1aknmt	0.00	0.69
Calr	0.02	0.76
Spcs2	0.00	0.70
Rps10l1	0.00	0.66
H2afz	0.00	0.53
Mcm7	0.00	0.39
AABR07031089.1	0.00	0.65
Mcm5	0.00	0.45
Mcm4	0.00	0.51
Mcm3	0.00	0.47
Mcm2	0.00	0.48
Fadd	0.00	0.60
Tnfaip2	0.01	0.60
Krt8	0.00	0.74
Spn	0.00	0.62
Arhgap19	0.00	0.59
Hadhb	0.00	0.66
Hadha	0.00	0.69
RGD1560010	0.00	0.59
Rpl6	0.00	0.62
Nxt1	0.00	0.69
AABR07040624.1	0.00	0.60
Nucks1	0.00	0.74
AABR07015180.1	0.00	0.60
Rad51c	0.00	0.68
Brca1	0.00	0.59
Rpl5	0.00	0.58
Slc16a14	0.00	0.67
Slc29a1	0.05	0.49
Ddx39a	0.00	0.73
Plk1	0.00	0.47
Rps3	0.00	0.54
Plk4	0.02	0.65
Tmem8a	0.00	0.74
Mad2l1	0.01	0.56
Timeless	0.04	0.74
Slc1a5	0.00	0.59
AABR07001512.1	0.00	0.39

RGD1561671	0.00	0.75	
AABR07012329.1	0.04	0.71	
AABR07039133.2	0.04	0.61	
Sgo1	0.01	0.66	
Gper1	0.01	0.58	
Babam1	0.00	0.68	
Dlx2	0.02	0.65	
Dlgap5	0.00	0.50	
Tnfaip8l3	0.00	0.60	
Stip1	0.00	0.69	
Gprasp2	0.00	0.65	
Alg8	0.00	0.60	
Gpi	0.00	0.68	
Cdt1	0.01	0.56	
Golt1b	0.00	0.67	
Polr1b	0.00	0.76	
Ncapg2	0.00	0.55	
Prim1	0.00	0.52	
LOC100359600	0.00	0.58	
Tfdp1	0.00	0.63	
Iqgap3	0.00	0.45	
Tmpo	0.03	0.75	
Hoxd8	0.01	0.75	
Nup107	0.00	0.72	
Nup35	0.00	0.63	
Tmem97	0.00	0.58	
Mybl2	0.00	0.55	
Ckap2l	0.00	0.59	
Eef2	0.00	0.57	
Bzw2	0.01	0.76	
AC119496.1	0.03	0.62	
AABR07000222.1	0.00	0.74	
Bub1	0.00	0.58	
Dsn1	0.01	0.71	
Kif15	0.00	0.75	
Kif11	0.00	0.55	
Sdf2l1	0.05	0.75	
Pcdh20	0.00	0.57	
Cdk2ap2	0.00	0.68	
Pcna	0.00	0.48	
Hjurp	0.00	0.57	
C1rl	0.00	0.77	
Gata2	0.00	0.73	
Tubb5	0.00	0.70	
Rpl18a	0.00	0.76	
Slc25a5	0.03	0.72	
AC095390.1	0.00	0.43	
Lrrc40	0.00	0.73	
LOC688459	0.04	0.74	
B9d2	0.00	0.75	
AC106663.1	0.02	0.71	
RGD1309350	0.00	0.76	
Uhrf1	0.00	0.39	
Lsm8	0.03	0.68	

Psph	0.01	0.73	
Lsm4	0.02	0.70	
Eef1g	0.00	0.61	
LOC100362830	0.00	0.56	
Lsm2	0.00	0.59	
Lsm3	0.00	0.64	
LOC100911361	0.02	0.72	
Lyar	0.00	0.69	
AABR07070810.1	0.00	0.56	
Cbx3	0.00	0.66	
Mrfap1	0.00	0.72	
Tpx2	0.00	0.56	
Mrpl35	0.00	0.72	
Pmf1	0.00	0.69	
Bub1b	0.00	0.55	
Gemin6	0.00	0.73	
Slc31a1	0.01	0.77	
LOC100359583	0.00	0.61	
AABR07049223.1	0.00	0.55	
Nptxr	0.00	0.71	
Lmnb2	0.00	0.63	
AABR07069282.1	0.00	0.55	
Lmnb1	0.01	0.64	
Hdac1l	0.01	0.59	
G2e3	0.00	0.65	
Gmfg	0.00	0.76	
Kntc1	0.00	0.54	
Nup85	0.01	0.66	
Phf19	0.00	0.60	
AABR07036087.1	0.00	0.71	
AABR07068127.1	0.00	0.63	
Smagp	0.00	0.76	
Aaas	0.00	0.67	
Cisd1	0.01	0.71	
Hmgn2	0.00	0.66	
Exo1	0.00	0.68	
Fkbp4	0.01	0.76	
Cdk1	0.00	0.45	
Cdk2	0.00	0.67	
AABR07035074.1	0.01	0.68	
Med18	0.00	0.72	
LOC100363469	0.00	0.58	
Trip13	0.01	0.74	
Kifc1	0.00	0.50	
Adamts4	0.01	0.75	
Sun1	0.00	0.71	
AABR07053166.1	0.00	0.60	
AABR07036247.1	0.00	0.59	
Mt1	0.01	0.54	
Srsf7	0.00	0.53	
AABR07067526.1	0.00	0.43	
Psat1	0.00	0.52	
Rps3a	0.01	0.60	
Metrn	0.00	0.56	

Slc35d1	0.00	0.76	
Tgfb1	0.00	0.76	
Tk1	0.00	0.43	
Snrpb2	0.01	0.76	
Hspa5	0.00	0.65	
Hspa9	0.02	0.72	
Atraid	0.01	0.75	
Plat	0.00	0.68	
Foxm1	0.00	0.54	
Rps4x	0.01	0.55	
Ctdsp1	0.00	0.72	
Fam83d	0.00	0.65	
AABR07060593.1	0.00	0.57	
Cetn2	0.00	0.64	
Pkmyt1	0.01	0.76	
Postn	0.00	0.53	
LOC100360117	0.00	0.56	
Rpl41	0.00	0.61	
Ncaph2	0.00	0.73	
Naca	0.00	0.74	
Nup62	0.00	0.70	
Rnps1	0.00	0.60	
Mcm6	0.00	0.54	
Tmem129	0.03	0.76	
Zfp706	0.00	0.74	
Ppat	0.00	0.65	
Elovl1	0.02	0.73	
Got1	0.01	0.76	
Troap	0.00	0.47	
Pls1	0.00	0.71	
Mre11a	0.00	0.71	
Cuedc2	0.02	0.76	
Chrac1	0.01	0.74	
Cks2	0.00	0.52	
RF00377	0.00	0.69	
Orc1	0.01	0.67	
Cdca2	0.01	0.69	
Cdca3	0.00	0.39	
AABR07057190.1	0.00	0.62	
Cdca7	0.00	0.48	
Cdca4	0.00	0.62	
Cdca8	0.00	0.57	
Suv39h1l1	0.00	0.57	
Ube2t	0.00	0.50	
Nr1i3	0.02	0.71	
Slc52a3	0.01	0.68	
Topbp1	0.00	0.60	
Mfsd10	0.01	0.69	
Kif22	0.00	0.49	
AABR07053716.1	0.01	0.63	
Anp32e	0.00	0.63	
Nup155	0.00	0.74	
Thoc3	0.00	0.63	
Hist2h2be	0.00	0.73	

Mepe	0.00	0.40
AABR07038948.2	0.01	0.71
Sephs2	0.00	0.71
Rps7	0.01	0.56
Jag2	0.01	0.71
Fen1	0.00	0.43
C1s	0.01	0.74
AC128960.1	0.00	0.64
Rps8	0.00	0.65
Tmem63a	0.00	0.70
Hmgb2	0.00	0.62
Cep295	0.02	0.69
LOC303566	0.00	0.66
Tedc2	0.00	0.65
Ugdh	0.00	0.72
Kcnn4	0.02	0.75
Mrps12	0.00	0.72
Bst2	0.04	0.77
Cdc45	0.00	0.65
Dlx1	0.00	0.77
Kif2c	0.00	0.46
AABR07029198.1	0.01	0.74
Mdh1	0.00	0.77
Pgghg	0.01	0.74
Cenpw	0.00	0.56
Cnot9	0.00	0.68
AABR07065438.1	0.00	0.67
Ubald2	0.01	0.63
Tpcn1	0.00	0.69
AABR07038873.1	0.00	0.66
AABR07035541.2	0.00	0.58
Mms22l	0.00	0.76
AABR07014550.1	0.03	0.47
Rexo4	0.01	0.70
Synm	0.00	0.64
Trmt10a	0.00	0.67
Pin4	0.00	0.75
AABR07055943.1	0.01	0.71
Cenpo	0.00	0.60
Cenpm	0.02	0.73
Ccna2	0.01	0.55
Ndufaf3	0.00	0.72
Cenph	0.00	0.51
Atad5	0.00	0.71
Cenpf	0.00	0.51
Cenpe	0.00	0.56
Spry4	0.00	0.65
Ttf2	0.00	0.76
Cenpa	0.00	0.63
Atad2	0.00	0.60
Slc35a4	0.01	0.73
Mapk3	0.02	0.76
Abcd1	0.00	0.73
Manf	0.02	0.69

Ckap2	0.04	0.71	
Cenpu	0.00	0.74	
Cenpt	0.01	0.45	
Pbk	0.00	0.51	
Fzd5	0.02	0.73	
Fzd1	0.01	0.66	
Rcc2	0.01	0.69	
Dnajc9	0.00	0.53	
MGC116202	0.00	0.70	
Nudt1	0.00	0.69	
Casp8ap2	0.00	0.75	
Kat2a	0.00	0.77	
G3bp1	0.00	0.72	
Gpn1	0.00	0.76	
Nnt	0.01	0.74	
Ppm1g	0.00	0.72	
Ptma	0.00	0.70	
mrpl11	0.01	0.70	
AABR07051240.1	0.00	0.63	
Idh2	0.01	0.76	
AC112018.1	0.00	0.63	
Krt79	0.00	0.71	
Eif2a	0.00	0.77	
Rpl15	0.00	0.57	
Rpl17	0.00	0.70	
Psmc3ip	0.00	0.72	
Atp1a1	0.00	0.56	
Slc7a5	0.00	0.42	
Nxph3	0.00	0.50	
Snrpa	0.00	0.63	
Ndc80	0.00	0.60	
AC118772.2	0.01	0.68	
AC095947.1	0.05	0.73	
AC098125.3	0.03	0.64	
Lrr1	0.01	0.77	
Hr	0.00	0.69	
AABR07024500.1	0.00	0.59	
Pole	0.00	0.61	
Rpa3	0.00	0.46	
Rpa2	0.00	0.62	
Tent4a	0.00	0.74	
AABR07025301.1	0.01	0.59	
Lman1	0.00	0.70	
Eme1	0.00	0.54	
AABR07049755.1	0.00	0.72	
Hyou1	0.00	0.66	
Rpl39	0.01	0.63	
Dtd2	0.00	0.71	
Bok	0.00	0.55	
Mrpl14	0.00	0.77	
Fam89b	0.04	0.71	
Pagr1	0.02	0.67	
LOC102555453	0.00	0.61	
Incenp	0.00	0.58	

AABR07069490.1	0.05	0.70
Las1l	0.00	0.75
Tipinl1	0.01	0.69
Egr1	0.00	0.55
Mrpl28	0.00	0.76
Gale	0.03	0.69
Plcd4	0.01	0.67
Pih1d1	0.00	0.68
Ncaph	0.00	0.47
Ncapg	0.00	0.61
Magoh	0.00	0.75
Clec2dl1	0.02	0.73
Rack1	0.00	0.58
Hspa13	0.02	0.72
Rwdd4	0.00	0.67
Nicn1	0.00	0.69
Sod3	0.00	0.77
Nuf2	0.00	0.53
Rtl3	0.01	0.55
Traip	0.00	0.62
AABR07007000.1	0.00	0.75
Pycard	0.00	0.56
RGD1562114	0.00	0.72
AABR07035539.1	0.00	0.53
Chchd1	0.03	0.75
Ect2	0.02	0.67
Sgo2	0.00	0.74
Prdx5	0.04	0.69
LOC100363452	0.02	0.71
Racgap1	0.01	0.73
Aspm	0.00	0.59
Necab3	0.00	0.65
Knstrn	0.00	0.55
Ppm1f	0.02	0.75
St3gal1	0.00	0.72
Diaph1	0.00	0.76
Bcl2l12	0.00	0.67
Ccne2	0.02	0.70
Rpl7	0.00	0.55
Ccne1	0.00	0.51
Rpl3	0.00	0.71
Aldh2	0.00	0.68
AABR07011698.1	0.00	0.50
AABR07055919.1	0.01	0.67
Mgme1	0.00	0.75
Dek	0.00	0.60
Ahcy	0.00	0.66
Fam111a	0.01	0.61
RF00264	0.00	0.71
Nusap1	0.00	0.60
Ddx11	0.00	0.69
Polr2c	0.01	0.73
Snrpa1	0.02	0.74
Fabp4	0.04	0.75

AABR07000658.1	0.00	0.42
Rpl11	0.00	0.54
Srsf4	0.00	0.75
Rps24	0.00	0.54
Id4	0.05	0.75
Tonsl	0.01	0.70
Abhd14b	0.00	0.73
Snrpb	0.00	0.54
Gins1	0.00	0.68
Chaf1a	0.00	0.56
AABR07012100.2	0.01	0.69
Chaf1b	0.00	0.71
Dmac2	0.01	0.75
Mmab	0.05	0.77
Tube1	0.00	0.76
Rnf26	0.00	0.57
Mt2A	0.00	0.61
Mis18a	0.00	0.62
Rpl18	0.00	0.66
Tmem138	0.00	0.70
Itpripl1	0.00	0.69
Smc4	0.04	0.76
Prpf19	0.03	0.68
AABR07025328.1	0.00	0.68
Chtf18	0.00	0.62
Arhgdia	0.00	0.70
Pwwp3a	0.01	0.76
Cdc7	0.01	0.73
Fhod1	0.04	0.73
Cetn4	0.00	0.74
Nup133	0.00	0.72
AC118772.3	0.02	0.70
RF00409	0.03	0.68
Dnmt1	0.00	0.67
AABR07049695.2	0.00	0.77
Esyt1	0.01	0.75
Cast	0.01	0.75
Aldh7a1	0.00	0.72
Stmn1	0.00	0.55
Dscc1	0.00	0.66
Rpl29	0.00	0.40
Bmp2	0.00	0.61
Anapc15	0.01	0.66
Rpl24	0.03	0.63
Rpl27	0.01	0.49
Rpl26	0.00	0.66
LOC100359539	0.03	0.60
Rpl23	0.00	0.60
Fancg	0.03	0.73
AC128207.2	0.04	0.55
Setd6	0.00	0.64
Fanci	0.00	0.57
Fbl	0.00	0.71

TABLE S4F Top altered genes in KMM cells following MLL2 knockdown

Gene	P-Value	Fold Change (shRNA/WT)
Smug1	0.02	1.34
Rbm5	0.00	1.31
Wrb	0.00	1.41
Mk1	0.01	1.60
AABR07012583.2	0.01	1.45
Hs1bp3	0.00	1.44
Zer1	0.00	1.33
Islr	0.04	2.12
Med31	0.00	1.44
AABR07044001.1	0.03	1.73
Tspan3	0.00	1.31
Hsd3b7	0.05	1.36
AABR07068316.1	0.00	1.33
Ppcs	0.00	1.35
Rasl11b	0.00	1.47
Iscu	0.01	1.30
AABR07021022.1	0.01	1.34
RF00492	0.04	1.50
Kcnh2	0.00	1.39
Plod2	0.00	1.55
Rnf183	0.04	1.94
AABR07039483.1	0.01	1.39
Slc39a9	0.00	1.46
B2m	0.00	1.33
Mrpl2	0.00	1.42
Mlkl	0.00	1.52
Zwint	0.04	1.41
Rab42	0.02	1.33
AC141220.2	0.03	1.33
Sh3gl1	0.00	1.32
Ccn4	0.01	1.37
Them6	0.01	1.40
Kctd11	0.00	1.33
Ccn2	0.01	1.51
Hmgcl	0.01	1.39
LOC102554034	0.00	1.40
Ccdc115	0.00	1.37
Vhl	0.00	1.33
Zfp219	0.01	1.38
RGD1566099	0.01	1.40
Med6	0.00	1.36
Wdr81	0.01	1.53
AABR07044421.1	0.00	1.34
S100a13	0.00	1.42
Timp2	0.00	1.51
Cebpa	0.00	1.99
AABR07049792.1	0.00	1.35
Higd2a	0.01	1.44
Vps11	0.00	1.47
Mx1	0.01	1.50
Ccdc189	0.00	1.38
Eci1	0.00	1.36

Tnip1	0.00	1.34	
Dpep3	0.00	1.49	
Itgb1bp1	0.00	1.31	
Ugt1a1	0.00	1.33	
Ptrh1	0.01	1.33	
Hexa	0.02	1.33	
Haghl	0.02	1.36	
Cog4	0.00	1.40	
Bcdin3d	0.00	1.31	
Flot2	0.00	1.32	
2-Mar	0.00	1.34	
Grn	0.01	1.43	
Ftl1	0.02	1.80	
Tnfrsf26	0.00	1.78	
Dnajb9	0.02	1.52	
Btg2	0.01	1.48	
Fam3a	0.00	1.33	
Washc5	0.00	1.30	
Hoxb7	0.00	1.34	
Hoxb5	0.03	1.35	
Bbs10	0.00	1.68	
Mme	0.00	1.41	
AC142458.1	0.00	2.00	
AABR07013147.1	0.01	1.39	
Tap1	0.00	1.34	
AABR07025787.1	0.00	2.27	
Cpe	0.00	1.41	
Aga	0.00	1.56	
AABR07026361.2	0.03	1.40	
Gga2	0.01	1.45	
Traf4	0.01	1.42	
Plekhg5	0.00	1.35	
Mxd4	0.01	1.46	
Mxd1	0.00	1.44	
Zbtb5	0.00	1.31	
AC120246.2	0.03	1.36	
AABR07029605.1	0.03	1.35	
AABR07027575.1	0.00	1.73	
Hint2	0.00	1.50	
Mpv17l2	0.00	1.38	
Hif1an	0.00	1.31	
Tmem101	0.00	1.33	
AABR07051399.1	0.03	1.94	
Atp2b4	0.00	1.40	
Renbp	0.00	1.78	
Akr1c14	0.03	1.37	
Chst12	0.01	1.40	
Bnip3l	0.00	1.34	
Mief2	0.00	1.40	
Mtss1l	0.00	2.36	
Rxfp3	0.01	1.74	
Tent5a	0.00	1.57	
AABR07070161.3	0.00	1.51	
Thap3	0.00	1.46	

Klhdc8b	0.01	1.37	
Tex264	0.00	1.46	
Tcp11l2	0.00	1.59	
Hbp1	0.00	1.71	
P2rx4	0.00	1.66	
Spint2	0.00	1.47	
Klf15	0.01	1.30	
Klhl31	0.00	1.58	
Ier5	0.00	1.60	
Mir145	0.04	1.54	
Tmem150a	0.02	1.55	
Plcg1	0.00	1.38	
Iba57	0.00	1.35	
AABR07068253.1	0.00	1.43	
AC121413.2	0.01	1.84	
Idua	0.00	1.70	
Terf2ip	0.00	1.38	
Ypel5	0.00	1.53	
Slitrk6	0.00	1.35	
Mynn	0.00	1.32	
Ypel3	0.01	2.03	
AC119762.7	0.01	1.53	
AABR07015040.1	0.00	1.35	
Ggt1	0.02	1.66	
Ggt7	0.00	1.32	
Rhob	0.02	1.37	
Tmem208	0.00	1.46	
Mir196c	0.05	1.56	
Msln	0.01	1.53	
Phldb3	0.00	1.32	
S100a1	0.04	1.48	
Homer3	0.00	1.44	
LOC679894	0.04	1.62	
Fkbp10	0.00	1.36	
Car5b	0.00	1.34	
Selenos	0.00	1.48	
AC131360.1	0.01	1.38	
Net1	0.01	1.33	
RGD1564171	0.01	1.56	
Bhlhe41	0.03	1.35	
Rilpl2	0.02	1.46	
Slc27a1	0.00	1.31	
Dact3	0.00	1.37	
Cavin2	0.00	1.45	
AC122625.1	0.04	1.63	
Tmem256	0.05	1.36	
AC141377.3	0.01	1.97	
Cdhr1	0.00	1.48	
Igfbp5	0.00	1.67	
AABR07032751.1	0.02	1.35	
Pnpo	0.00	1.64	
Rpl10l	0.02	1.31	
Dbn1	0.01	1.47	
Fam25a	0.03	1.54	

Syde1	0.04	1.31
AABR07059891.1	0.00	1.55
Lsmem2	0.03	1.32
AABR07006258.2	0.02	1.34
Tssk3	0.01	1.52
Sesn2	0.02	1.49
Iah1	0.01	1.48
Mrps6	0.00	1.33
Tsc22d3	0.02	1.53
Tcn2	0.00	2.79
Sparc	0.00	1.98
Rab3a	0.00	1.37
Foxo4	0.00	1.75
Ilvbl	0.00	1.37
Dyrk1b	0.00	1.81
Pqlc2	0.01	1.37
Nap1l3	0.00	1.53
Lamp1	0.00	1.53
Mmp13	0.01	1.49
Atg101	0.00	1.34
Zfp580	0.04	1.79
AABR07039304.1	0.04	1.34
Sv2a	0.03	1.39
Adrb2	0.00	1.67
Gtpbp2	0.00	1.36
Sort1	0.00	1.35
Fem1b	0.00	1.40
Klhdc3	0.01	1.44
AABR07008420.1	0.01	1.69
Spaca6	0.02	1.49
Pts	0.00	1.52
Osgin1	0.00	1.36
Cry2	0.00	1.73
Serinc2	0.00	1.40
Itga10	0.00	1.71
Ccpg1os	0.00	1.44
Prune1	0.00	1.42
Hapln4	0.02	1.39
Snx33	0.00	1.44
Snx32	0.03	1.42
Mir206	0.05	1.31
Nfat5	0.01	1.36
Lgmn	0.00	1.62
AABR07043169.1	0.05	1.34
Ccndbp1	0.00	1.33
Slc22a17	0.04	1.41
Tmem59	0.00	1.40
Mirlet7d	0.03	1.51
Mrpl57	0.00	1.38
Ngp	0.00	1.50
Mrpl52	0.00	1.35
Chmp1a	0.00	1.53
Trim34	0.00	1.64
Ip6k2	0.00	1.43

AABR07004269.4	0.00	1.41	
Igbp1	0.00	1.35	
Med9	0.00	1.33	
Ppp1r3c	0.01	1.58	
Nme3	0.00	2.15	
Taar7b	0.00	1.55	
Iqcd	0.00	1.82	
Atf5	0.01	1.39	
Atp6v0d1	0.00	1.46	
Icoslg	0.01	1.33	
AC139392.1	0.02	1.86	
Erlec1	0.00	1.36	
Cd320	0.00	1.52	
Abtb1	0.00	1.59	
Washc2c	0.00	1.38	
Pgrmc1	0.00	1.41	
LOC686013	0.00	1.57	
Tpp1	0.00	1.56	
Creld1	0.00	1.33	
Gpr146	0.00	1.53	
Fbxl7	0.02	1.34	
Ncstn	0.00	1.30	
Hsd17b8	0.02	1.30	
Cdk5r1	0.00	1.77	
Idnk	0.00	1.42	
Hsd17b1	0.05	1.55	
AABR07027811.2	0.01	2.72	
AABR07027811.3	0.00	1.82	
Cmtm6	0.00	1.31	
Fuca1	0.00	1.71	
AABR07044635.1	0.02	1.59	
Fam98c	0.00	1.50	
AABR07033047.1	0.01	1.42	
Prorsd1	0.00	1.38	
Ddit3	0.03	1.62	
Ddit4	0.00	2.66	
Cfh	0.03	1.32	
AABR07065970.1	0.01	1.40	
Gpnmb	0.01	1.62	
Uap1l1	0.02	1.78	
Eid2b	0.05	1.47	
Cst3	0.00	1.84	
Atp6v1d	0.00	1.76	
Atp6v1f	0.00	1.49	
Atp6v1a	0.00	1.45	
AABR07071000.1	0.00	1.38	
Retreg3	0.00	1.45	
Pcolce	0.00	1.34	
Psca	0.03	1.64	
Tcta	0.00	1.76	
Asmtl	0.00	1.33	
Tmem160	0.00	1.52	
Yod1	0.00	1.55	
Bak1	0.00	1.33	

Pdk2	0.01	1.41
AC103090.1	0.04	1.34
Serpinb7	0.03	1.40
Phf7	0.00	1.33
Hsdl1	0.00	1.62
Pla2g15	0.00	1.93
Tmed3	0.00	1.42
AC116236.2	0.01	1.45
RGD1306441	0.00	1.83
Ifi27	0.00	1.35
Nqo1	0.02	1.45
Zfp688	0.00	1.45
Per1	0.00	1.44
Pitpnm1	0.00	1.32
AC094643.1	0.03	1.60
Dmpk	0.00	2.00
Hexim2	0.00	1.34
Pdf	0.02	1.32
Pdcd4	0.00	1.55
Aox1	0.00	1.61
Commd8	0.00	1.37
Hspb2	0.00	1.60
Cfap298	0.00	1.46
Sirt4	0.00	1.33
Pfkm	0.00	1.33
Commd4	0.01	1.64
Gpr88	0.00	1.54
Bri3	0.00	1.37
Amdhd2	0.00	1.59
Lamp2	0.00	1.54
Atp6v1g1	0.00	1.37
Dbp	0.00	1.60
Ifitm3	0.01	1.51
Ifitm1	0.00	1.48
Ifitm6	0.00	1.34
Tmem205	0.00	1.47
Ergic3	0.00	1.52
Il6r	0.01	1.33
LOC684773	0.03	1.72
Alg10	0.00	1.32
Pura	0.03	1.51
Ldb1	0.00	1.44
Tp53inp1	0.00	3.62
Clu	0.00	1.65
Bdh2	0.00	1.68
Apol9a	0.00	1.32
Ephx1	0.00	1.58
Bmf	0.00	1.61
RGD1564804	0.00	1.52
LOC363337	0.01	1.40
Btbd19	0.00	1.92
Nkx3-2	0.00	1.41
Pink1	0.00	1.67
Zscan25	0.04	1.31

LOC108348302	0.03	1.32	
Ufsp1	0.02	1.38	
Vamp1	0.03	1.66	
Vamp3	0.01	1.37	
S100a16	0.04	1.44	
Ttc30b	0.00	1.49	
Sft2d3	0.03	1.31	
Sesn3	0.00	1.77	
Itm2c	0.00	1.78	
Itm2b	0.00	2.13	
RF00586	0.03	1.57	
Slc49a3	0.02	1.37	
Svbp	0.00	1.39	
Giot1	0.02	1.49	
Ghdc	0.00	1.37	
LOC102553785	0.01	1.63	
Klhl24	0.00	1.66	
Igip	0.02	1.45	
Znf740	0.01	1.32	
Reep3	0.00	1.51	
Sec14l2	0.00	1.38	
Comtd1	0.01	1.46	
Zfp467	0.01	1.34	
Pgpep1	0.00	1.52	
AABR07060291.1	0.03	1.50	
Fam110b	0.00	1.31	
Serinc4	0.00	1.44	
AABR07030911.2	0.01	1.52	
Mapre3	0.00	1.67	
AC119015.4	0.03	1.35	
Uckl1	0.00	1.32	
Gnpda1	0.01	1.38	
Ankrd49	0.00	1.33	
AABR07011977.1	0.04	1.33	
Atg12	0.03	1.41	
Vamp2	0.00	1.63	
Ttll13	0.00	1.43	
Blcap	0.00	1.50	
Loxl4	0.04	1.40	
Dusp1	0.03	1.87	
AABR07027910.1	0.00	1.34	
Spns1	0.00	1.93	
Zfp358	0.01	1.33	
Fam168b	0.00	1.40	
Rnf166	0.00	1.68	
Adamtsl5	0.01	1.43	
Mustn1	0.00	1.46	
Supt4h1	0.00	1.36	
Mir874	0.00	1.65	
Adam15	0.00	1.56	
AABR07009357.2	0.00	1.34	
Asl	0.00	1.56	
Dnpep	0.00	1.34	
Cdc42ep2	0.00	1.52	

Hyal1	0.00	1.32	
Plk2	0.01	1.79	
Akt1s1	0.00	1.35	
Fmod	0.00	1.72	
Nadk2	0.02	1.34	
RF00582	0.01	2.01	
LOC100365043	0.01	1.81	
Crot	0.01	1.31	
Neurl2	0.00	1.30	
AABR07054266.1	0.00	1.51	
Cc2d1b	0.01	1.31	
AABR07011697.1	0.00	1.54	
AABR07072559.2	0.01	2.06	
Gpr165	0.05	2.44	
Pcmtd2	0.00	1.33	
Gprin3	0.00	1.62	
Fam229a	0.02	1.39	
Gpt	0.04	1.49	
LOC361985	0.00	1.51	
Tgif1	0.00	1.32	
Cpt1c	0.00	1.56	
AC120096.3	0.05	1.37	
Mir146b	0.01	1.43	
Pnrc1	0.00	1.66	
Mtmr11	0.03	1.71	
Hoxc10	0.00	1.40	
Atp5f1e	0.04	1.37	
C1qtnf6	0.00	1.80	
Lrrn4cl	0.00	2.33	
Lrpap1	0.00	1.55	
AABR07000747.1	0.00	2.71	
P2ry4	0.00	2.39	
Spata31d1	0.01	1.32	
Armc7	0.02	1.31	
AABR07003344.1	0.03	1.41	
Chmp5	0.00	1.30	
Rhod	0.03	1.43	
Nit1	0.01	1.32	
LOC687707	0.01	1.33	
Gnptg	0.00	1.62	
Serf1	0.00	1.42	
AY172581.9	0.02	1.34	
RGD1563941	0.01	1.54	
Mtcp1	0.03	1.41	
Mpst	0.00	1.46	
Wbp1l	0.00	1.40	
Bckdha	0.00	1.47	
Pcdh20	0.05	1.37	
Cdk2ap2	0.01	1.36	
Ifi44	0.00	1.65	
Slc25a2	0.00	1.56	
Prr3	0.01	1.32	
Ns5atp4	0.00	1.41	
Slc43a2	0.00	1.32	

Npc2	0.01	1.59	
Slc22a18	0.00	1.41	
AABR07069433.1	0.01	1.44	
AABR07033020.1	0.02	1.37	
Pik3ip1	0.00	2.50	
Zfp523	0.00	1.33	
AABR07016572.1	0.00	1.58	
Lin37	0.00	1.33	
Dgat2	0.00	1.31	
AABR07072761.1	0.00	1.40	
RGD1308117	0.02	1.50	
Angptl4	0.00	1.43	
Zmynd10	0.04	1.53	
Mir24-2	0.02	1.62	
Foxc1	0.00	1.30	
Mrc2	0.00	1.32	
Ormdl2	0.00	1.32	
Fkbpl	0.00	1.41	
AABR07043844.1	0.01	1.44	
Mir2964	0.01	1.71	
Lig4	0.01	1.57	
AC115420.2	0.04	1.51	
Fis1	0.00	1.32	
Rab29	0.00	1.41	
Eid1	0.01	1.30	
AABR07044375.2	0.04	1.43	
AABR07044375.1	0.02	1.89	
Ctxn1	0.01	1.83	
Gng8	0.01	1.39	
Slc10a5	0.01	1.60	
Mpg	0.00	1.40	
Grina	0.00	1.44	
Sqstm1	0.00	1.50	
LOC102547645	0.00	1.66	
Snapc2	0.00	1.51	
Plekhb2	0.00	1.72	
Hist2h2be	0.02	1.42	
AC130391.5	0.00	1.65	
Gfpt1	0.00	1.43	
RGD1565784	0.00	1.32	
AC130391.1	0.00	1.84	
Tpra1	0.00	1.42	
AABR07044420.1	0.01	1.34	
Hjv	0.01	1.45	
Sap30	0.00	1.52	
Plbd2	0.00	1.47	
AABR07051450.1	0.00	1.30	
Nt5c	0.02	1.61	
Pld3	0.00	1.86	
Nop53	0.00	1.35	
Mfsd11	0.01	1.53	
Fkbp7	0.00	1.31	
Bles03	0.00	1.33	
Aldh5a1	0.00	1.69	

Numbl	0.00	1.36	
Kifc2	0.01	1.44	
Tctex1d2	0.00	1.39	
Acads	0.00	1.41	
Tmem132a	0.00	1.39	
Gigyf1	0.00	1.74	
Ifi35	0.00	1.32	
Ezh1	0.00	1.46	
Aktip	0.00	1.35	
Ngrn	0.02	1.40	
Limd2	0.04	1.44	
Crebrf	0.00	1.45	
Slc35d2	0.00	1.31	
Mdm2	0.00	1.31	
Fau	0.01	1.34	
Acadm	0.00	1.33	
Hist1h2bk	0.01	1.53	
Gja1	0.01	1.48	
Xylb	0.02	1.40	
Laptm4a	0.00	1.34	
AABR07042454.2	0.01	1.42	
Laptm4b	0.00	1.44	
Mfsd1	0.00	1.37	
Churc1	0.01	1.33	
Mkrn3	0.02	1.37	
Pcdhga4	0.00	1.33	
Pcdhga2	0.00	1.40	
Pcdhga1	0.00	1.46	
Il18bp	0.00	1.32	
Pcdhga7	0.01	1.33	
AABR07042611.1	0.01	1.56	
Tvp23b	0.00	1.43	
LOC108348201	0.01	1.37	
Nxf7	0.00	1.43	
RT1-M3-1	0.00	1.73	
Gba	0.00	1.66	
Hmox1	0.03	2.18	
RGD1562378	0.00	3.36	
LOC100174910	0.00	1.38	
Sh3glb2	0.00	1.32	
LOC684762	0.01	1.75	
Pigy	0.00	1.47	
AC122630.1	0.04	1.90	
Pdgfrb	0.00	1.72	
Tmem127	0.00	1.34	
Lamb2	0.00	1.40	
Sytl1	0.01	1.38	
Scarf2	0.02	1.47	
AABR07062138.1	0.00	1.38	
AABR07041778.1	0.01	1.36	
AABR07027753.3	0.03	2.36	
AABR07062138.2	0.02	1.59	
AABR07035339.1	0.01	1.58	
Fgfbp3	0.01	1.34	

LOC103690190	0.01	1.51	
Scrn2	0.00	1.35	
Cmklr1	0.02	1.69	
Zbtb9	0.00	1.31	
Gcdh	0.00	1.33	
Hpx	0.00	1.32	
Ube2g2	0.00	1.48	
Cyp1a1	0.00	1.67	
AABR07031734.13	0.00	1.42	
Ppt1	0.02	1.33	
AABR07031734.15	0.02	1.78	
AABR07010705.1	0.00	1.46	
AC114460.1	0.00	1.34	
Igfbp7	0.00	1.42	
Arrdc3	0.00	1.35	
Plag1	0.01	1.34	
Pex11a	0.00	1.35	
Lin7c	0.00	1.45	
Tmem80	0.00	1.43	
AABR07053136.1	0.00	1.60	
LOC689412	0.02	1.41	
Zfp691	0.00	1.33	
Dstn	0.02	1.42	
Arhgap4	0.00	1.30	
Ctns	0.00	1.43	
Ankrd53	0.00	1.32	
AABR07069462.1	0.00	1.39	
Aqp1	0.04	2.52	
Hist1h2ail1	0.03	1.56	
Med25	0.00	1.47	
Ndufb2	0.02	1.34	
Aqp8	0.04	2.50	
Alas1	0.01	1.42	
Dnm1	0.00	1.36	
Aamp	0.01	1.45	
Stc1	0.00	1.42	
C1s	0.00	1.32	
C1r	0.00	1.54	
Ermard	0.00	1.39	
LOC100909912	0.00	1.37	
Acad11	0.00	1.42	
AABR07030630.1	0.00	1.66	
Cd1d1	0.04	1.59	
Smim14	0.00	1.36	
Rxrb	0.00	1.37	
Vkorc1	0.00	1.54	
Mir3074	0.05	1.84	
Dvl2	0.00	1.58	
Snn	0.00	1.96	
Syt11	0.01	1.53	
Dlx5	0.00	1.49	
Uchl1	0.02	1.32	
LOC102551095	0.00	1.53	
AABR07030265.1	0.03	2.05	

Cox6a2	0.00	1.87	
Sec22b	0.00	1.37	
Gabarapl1	0.00	1.92	
Psap	0.00	1.55	
Rapsn	0.00	1.49	
Slc19a2	0.00	1.49	
Nrbp2	0.01	1.37	
Tmem106c	0.00	1.31	
Flcn	0.00	1.54	
Gdi1	0.00	1.35	
Prss53	0.00	1.40	
LOC498368	0.00	1.92	
Ctsa	0.01	1.46	
Ctsb	0.00	1.77	
AC107096.2	0.00	2.38	
Cd37	0.02	1.55	
Tcirg1	0.01	1.40	
Ech1	0.00	1.86	
Dyrk3	0.00	1.33	
Rnd3	0.00	1.51	
Tnnc1	0.00	1.48	
Pcdhb22	0.00	1.49	
Col3a1	0.00	2.54	
Pcdhb20	0.01	1.37	
Pcdhb21	0.00	1.74	
Gaa	0.00	2.37	
AC130232.1	0.01	1.34	
Mlycd	0.00	1.39	
RGD1561102	0.04	1.34	
Hoxc8	0.00	1.38	
AABR07039210.1	0.01	1.39	
AABR07039210.2	0.00	1.35	
Praf2	0.02	1.42	
Lztfl1	0.00	1.43	
Sema3b	0.00	1.71	
Psenen	0.00	1.36	
RGD1305938	0.01	1.39	
Khnyn	0.04	1.38	
Hrct1	0.00	1.68	
Nhlrc3	0.03	1.31	
Hist1h2bd	0.00	1.63	
Fzd2	0.01	1.46	
Pinlyp	0.00	1.82	
AABR07059198.1	0.04	1.39	
LOC100361265	0.02	1.42	
Slc6a8	0.03	1.41	
AABR07035317.2	0.02	1.59	
AABR07035317.1	0.02	1.40	
Khk	0.00	1.31	
AC134224.1	0.01	2.19	
AC134224.2	0.01	2.32	
AC134224.3	0.00	2.49	
Serpini1	0.00	1.42	
Arfip2	0.01	1.37	

Dcakd	0.00	1.45	
Ccdc96	0.00	1.92	
Rogdi	0.00	1.67	
Dhrs1	0.04	1.31	
Hist1h2bl	0.04	1.45	
Cnpy2	0.00	1.54	
AABR07030914.1	0.01	1.35	
AC099089.1	0.01	2.97	
Hoxc6	0.00	1.31	
Borcs6	0.01	1.48	
LOC100909474	0.03	1.66	
Xylt2	0.00	1.31	
Lyrm2	0.01	1.35	
Pgam2	0.03	1.70	
Trib2	0.00	1.42	
LOC103691238	0.00	1.53	
AABR07011996.1	0.00	1.73	
Fcgrt	0.00	1.86	
Eme2	0.04	1.36	
Washc3	0.00	1.30	
Enpep	0.00	1.42	
Plekhj1	0.01	1.31	
Cdkn1b	0.00	1.48	
Cdkn1a	0.00	1.66	
AC110351.2	0.00	1.36	
Hist1h2ao	0.01	1.85	
Cyb561a3	0.00	1.36	
Hist1h2ah	0.00	1.34	
Thyn1	0.00	1.32	
Tmem185b	0.01	1.38	
Yipf3	0.00	1.33	
Yipf2	0.00	1.52	
AABR07043557.1	0.00	1.61	
Samd9	0.00	1.55	
AABR07030861.1	0.00	1.39	
AY172581.20	0.00	2.18	
Spag8	0.02	1.55	
AY172581.24	0.02	1.41	
Hist1h1t	0.00	1.44	
Gadd45a	0.00	2.75	
Selenop	0.00	1.33	
Dynlt3	0.00	1.66	
Gadd45g	0.04	1.46	
Syngap1	0.00	1.40	
Hist1h1d	0.00	3.01	
Alkbh7	0.00	1.43	
Klc4	0.00	1.44	
F11r	0.00	1.38	
Hist1h1c	0.00	2.87	
AABR07004881.1	0.02	1.70	
Entpd4	0.00	1.36	
Slc25a44	0.00	1.30	
Exoc3l1	0.01	1.48	
Gbp2	0.00	1.50	

Avpr2	0.01	1.40	
Glyctk	0.00	1.39	
Fbxw9	0.00	1.55	
AABR07017999.1	0.04	1.71	
Lrrc73	0.00	1.37	
Phlda3	0.00	1.40	
Phlda2	0.05	1.31	
Rwdd2a	0.00	1.36	
Rasd1	0.00	1.88	
Epha7	0.00	1.30	
Actrt3	0.02	1.34	
Tgoln2	0.00	1.56	
AC123425.1	0.03	2.16	
Tigd2	0.02	1.37	
RT1-S3	0.02	1.34	
Smim22	0.01	1.33	
Nradd	0.00	1.32	
Arl4a	0.00	1.80	
Bcl2l11	0.00	1.39	
Zfp579	0.03	1.45	
Tm4sf1	0.01	1.37	
Sowahc	0.00	2.12	
AABR07030200.1	0.01	2.16	
Ndor1	0.00	1.34	
Tbc1d13	0.02	1.32	
Thra	0.01	1.41	
RGD1306502	0.00	1.35	
Tbc1d17	0.00	1.60	
AABR07014756.1	0.02	1.85	
LOC108351584	0.01	1.94	
Wdr45	0.00	1.35	
Tprn	0.02	1.41	
Isg15	0.01	1.45	
Fabp3	0.05	1.32	
Pcdhb14	0.00	1.46	
Ccl21	0.00	3.14	
Pcdhb19	0.00	1.62	
Rnaset2	0.00	1.44	
AABR07053734.1	0.01	1.41	
AABR07006111.1	0.00	1.54	
Cnp	0.00	1.31	
Tle2	0.01	1.32	
Zfp597	0.00	1.32	
Kcnf1	0.04	1.33	
Vgll4	0.00	1.38	
Hist1h4b	0.00	1.54	
Slc38a7	0.01	1.40	
RGD1309748	0.00	1.41	
Clba1	0.00	1.44	
Calml3	0.00	1.48	
Chpf2	0.01	1.42	
Calml4	0.00	2.11	
Mocs3	0.00	1.47	
Actl7b	0.01	1.41	

Rnd2	0.00	1.53
AABR07058464.1	0.00	1.36
Maf1	0.01	1.31
Maf	0.00	1.48
LOC108348771	0.00	1.37
Atp6v1b2	0.00	1.34
Zadh2	0.00	1.36
Pomk	0.00	1.33
AABR07072236.1	0.00	1.60
Nat9	0.00	1.35
RF00405	0.02	1.56
Dtx3	0.00	1.91
Camk1	0.00	1.48
Lcn12	0.00	1.32
Slc48a1	0.00	1.52
Ccpg1	0.00	1.36
RT1-N2	0.00	1.51
Psmg3	0.00	1.35
Decr1	0.01	1.36
Hfe	0.01	1.37
AC126572.7	0.01	1.35
AABR07006081.1	0.00	1.60
Myo1e	0.00	1.37
AABR07026002.1	0.01	1.48
Atp1b1	0.00	1.47
Tmem203	0.00	1.62
Tmem140	0.00	1.38
Acy3	0.00	1.36
Etfrf1	0.00	1.86
Dpp7	0.00	2.75
Cnih1	0.01	0.72
LOC102551606	0.00	0.76
Gas2l3	0.00	0.57
Cmpk1	0.00	0.70
Cep55	0.00	0.66
Slc39a10	0.00	0.73
Ccnf	0.00	0.64
Kpna2	0.00	0.71
Ctbp1	0.00	0.76
Asf1b	0.00	0.61
Hnrnpa1	0.00	0.62
Ska1	0.00	0.64
AABR07042875.1	0.00	0.74
Slc1a4	0.00	0.76
Cacybp	0.00	0.70
Ptdss1	0.00	0.75
Mbd3	0.02	0.76
Tacc3	0.00	0.60
Cks1b	0.00	0.63
Ncapd2	0.00	0.60
Ncapd3	0.00	0.73
Akt1	0.01	0.73
Aunip	0.00	0.72
Med1	0.00	0.69

Dctpp1	0.01	0.75	
Rrm2	0.00	0.57	
Rrm1	0.00	0.53	
Myadm	0.00	0.77	
AABR07026311.1	0.00	0.53	
Naa10	0.00	0.74	
AC112350.1	0.00	0.72	
Fubp1	0.00	0.74	
Srpk1	0.00	0.76	
LOC102546716	0.01	0.66	
Prim1	0.00	0.54	
AABR07044366.1	0.04	0.71	
Shmt2	0.00	0.55	
Pitpnb	0.00	0.76	
Ccnb1	0.00	0.48	
Palb2	0.00	0.76	
AABR07024972.1	0.00	0.66	
AABR07029613.1	0.00	0.60	
AABR07044959.1	0.00	0.71	
Rybp	0.00	0.77	
Shc1	0.00	0.64	
Brca1	0.00	0.72	
Mad2l1	0.00	0.59	
Ercc1	0.00	0.74	
Dnajb1	0.00	0.63	
Dnajb5	0.00	0.73	
RGD1562136	0.00	0.62	
Mmd	0.01	0.61	
Ppp1r9b	0.01	0.73	
Spc25	0.00	0.59	
Spc24	0.05	0.70	
Jpt2	0.00	0.65	
Shcbp1	0.00	0.67	
Klhl41	0.00	0.60	
Sprr1a	0.01	0.38	
11-Sep	0.00	0.69	
Ldha	0.00	0.66	
AABR07019399.1	0.00	0.75	
Qsox2	0.01	0.75	
Haspin	0.02	0.68	
Hsp90b1	0.00	0.65	
AABR07061614.1	0.00	0.71	
Tmem109	0.00	0.71	
Tfrc	0.04	0.52	
Arhgap11a	0.00	0.56	
Mmgt1	0.00	0.55	
Jag2	0.00	0.72	
Ptges3l1	0.00	0.71	
Pgk1	0.00	0.65	
Bora	0.02	0.74	
AC124205.1	0.01	0.75	
AABR07053707.1	0.00	0.71	
Chst14	0.01	0.71	
AC127920.1	0.00	0.72	

Tufm	0.03	0.76
Etv5	0.00	0.70
Rbmxl1b	0.00	0.72
Psmd5	0.00	0.70
Cdr2	0.00	0.72
Ska3	0.00	0.67
AABR07028352.1	0.00	0.52
AABR07036498.1	0.04	0.58
Agpat1	0.01	0.75
Plaur	0.02	0.73
Eif4e	0.00	0.76
RF00186	0.02	0.75
AABR07008097.1	0.00	0.60
Nasp	0.00	0.62
Gins1	0.00	0.71
Rnf10	0.01	0.72
Gins4	0.00	0.74
Dtl	0.00	0.73
Phb2	0.02	0.73
Sf3b3	0.00	0.61
E2f8	0.00	0.70
Fancd2	0.00	0.72
Manea	0.00	0.74
Pttg1	0.00	0.67
Serpine1	0.01	0.49
Kmt2b	0.00	0.65
F3	0.00	0.74
Erlin1	0.00	0.74
Ankrd13a	0.00	0.61
Dhfr	0.00	0.77
Anln	0.02	0.74
Ssrp1	0.00	0.72
Fbxo5	0.00	0.53
Cavin1	0.01	0.66
Ehmt2	0.00	0.69
Rfc5	0.01	0.71
B3gnt9	0.00	0.67
Rfc2	0.00	0.72
Rbmx2	0.00	0.76
Lrrc75b	0.00	0.69
Ube2s	0.02	0.66
Efhd2	0.00	0.72
Ddx21	0.00	0.75
Cd3eap	0.04	0.75
Tpm3	0.00	0.76
Csf1	0.04	0.74
Elk3	0.00	0.74
Ldlr	0.03	0.36
Ssr3	0.00	0.76
Emp3	0.00	0.72
Elk4	0.01	0.76
Emp1	0.03	0.66
Thap12	0.00	0.64
Dclre1b	0.00	0.71

Hirip3	0.00	0.64	
Jmjd6	0.00	0.72	
RF00421	0.02	0.64	
Ncaph	0.00	0.68	
Krt26	0.00	0.70	
Ap2a2	0.00	0.73	
Cmtr2	0.00	0.77	
Slc47a1	0.00	0.74	
Gpsm2	0.00	0.76	
Dnaja4	0.00	0.76	
Hspd1	0.00	0.75	
Birc5	0.00	0.60	
AABR07018244.2	0.00	0.69	
Alyref	0.00	0.69	
Nup43	0.00	0.73	
Pgp	0.00	0.74	
F2rl1	0.00	0.50	
Sapcd2	0.00	0.72	
Rif1	0.00	0.74	
Psma4	0.01	0.77	
Nup107	0.00	0.74	
Usp1	0.00	0.58	
AABR07013288.4	0.00	0.75	
Gpd1l	0.00	0.59	
AC126897.1	0.00	0.70	
Top2a	0.00	0.60	
Anxa1	0.00	0.70	
Tulp3	0.00	0.76	
Nme2	0.01	0.73	
Hat1	0.00	0.75	
Nuf2	0.00	0.68	
Rangap1	0.00	0.70	
Hyls1	0.02	0.61	
Rad51ap1	0.00	0.74	
Sfpq	0.00	0.73	
Emg1	0.00	0.67	
Lrrc59	0.00	0.69	
Gar1	0.01	0.74	
Rad51	0.01	0.76	
Bbip1	0.02	0.71	
Exosc8	0.00	0.72	
Pcbp2	0.00	0.75	
Phgdh	0.00	0.70	
Lrrcc1	0.00	0.72	
Prc1	0.00	0.52	
Mrto4	0.00	0.76	
Cad	0.00	0.71	
Hmmr	0.00	0.64	
Ptgs1	0.04	0.75	
Msn	0.00	0.76	
Ran	0.00	0.73	
Carm1	0.00	0.71	
Polg	0.00	0.58	
Pole	0.00	0.70	

AABR07050283.2	0.01	0.73	
Wdr5	0.00	0.70	
Tubb5	0.00	0.61	
Wdr1	0.00	0.63	
Depp1	0.00	0.77	
Aspm	0.00	0.68	
Eno3	0.02	0.74	
Pelp1	0.00	0.65	
Kif20b	0.00	0.68	
Pa2g4	0.00	0.53	
Cdc6	0.00	0.63	
Cdc7	0.00	0.74	
Cip2a	0.00	0.71	
Stmn1	0.00	0.66	
RGD1562690	0.00	0.63	
Ercc6l	0.00	0.58	
Ndc1	0.00	0.74	
Prr11	0.00	0.71	
Aurka	0.00	0.58	
Aurkb	0.00	0.64	
Fkbp1a	0.00	0.63	
Tcf19	0.00	0.67	
Mex3c	0.00	0.72	
Troap	0.02	0.62	
Tex30	0.02	0.74	
Cnnm4	0.00	0.68	
Slbp	0.00	0.65	
AC094643.2	0.01	0.67	
Ung	0.00	0.66	
Cct6a	0.00	0.72	
AC094126.2	0.00	0.55	
Adss	0.00	0.72	
AC099137.1	0.00	0.74	
Cct4	0.00	0.75	
Vim	0.02	0.74	
Depdc1	0.02	0.76	
Wsb2	0.00	0.69	
Uhrf1	0.00	0.46	
LOC100363502	0.00	0.72	
Txnrd1	0.00	0.72	
Mki67	0.01	0.53	
Stil	0.00	0.66	
Mcm10	0.00	0.65	
Taf1d	0.02	0.76	
Haus4	0.00	0.72	
Relt	0.00	0.76	
Fignl1	0.00	0.72	
Pold1	0.00	0.63	
Pold2	0.00	0.71	
Dut	0.01	0.70	
PVR	0.00	0.77	
Nrm	0.01	0.68	
Clic1	0.00	0.59	
Gltp	0.02	0.76	

Pdia4	0.03	0.77	
Arpc5	0.00	0.74	
Sfxn3	0.04	0.76	
Wdhd1	0.00	0.73	
Rrp15	0.00	0.72	
LOC100912427	0.00	0.68	
Kif4a	0.00	0.70	
Pdp2	0.02	0.66	
RF00581	0.00	0.72	
Nacc1	0.00	0.75	
AC129365.1	0.00	0.52	
Set	0.00	0.70	
U2af1	0.00	0.73	
Slc25a39	0.01	0.70	
Paqr4	0.02	0.73	
Cdc20	0.00	0.55	
Far1	0.01	0.76	
Lyar	0.00	0.71	
AC128212.1	0.00	0.63	
Crat	0.00	0.75	
Ppm1g	0.00	0.77	
Slc20a1	0.02	0.70	
AABR07039303.4	0.01	0.69	
St3gal1	0.00	0.76	
H2afz	0.00	0.52	
Mcm7	0.00	0.62	
Mcm6	0.00	0.53	
Mcm5	0.00	0.57	
Mcm4	0.00	0.51	
Mcm3	0.00	0.48	
Dusp8	0.00	0.70	
Fadd	0.00	0.58	
Dusp7	0.01	0.76	
Sumo2	0.01	0.76	
Ugdh	0.00	0.67	
Hmga1	0.01	0.46	
Nxt1	0.00	0.66	
Nop56	0.00	0.67	
Cdh3	0.03	0.75	
AABR07015180.1	0.00	0.65	
LOC100364062	0.00	0.76	
Ddx39a	0.00	0.68	
Plk1	0.00	0.51	
Hgh1	0.03	0.67	
Plk4	0.00	0.63	
Fam107b	0.00	0.76	
Timeless	0.00	0.61	
RF00087	0.00	0.52	
Slc1a5	0.00	0.61	
AABR07011698.1	0.00	0.64	
Eif4a1	0.00	0.76	
AABR07012329.1	0.03	0.66	
P4ha3	0.01	0.74	
AABR07058287.1	0.00	0.74	

Glrx5	0.01	0.69	
Sowahb	0.05	0.60	
Ripk3	0.02	0.60	
Adgra2	0.00	0.65	
Dlgap5	0.00	0.66	
Stip1	0.00	0.62	
Gprasp2	0.01	0.76	
RGD1311946	0.00	0.77	
Eif5a	0.00	0.71	
AABR07039356.1	0.00	0.75	
Ticrr	0.00	0.74	
Nsun2	0.00	0.72	
Pak1ip1	0.00	0.64	
LOC100359600	0.00	0.63	
Anapc1	0.00	0.71	
Actb	0.01	0.74	
Tfdp1	0.00	0.75	
Iqgap3	0.00	0.62	
Tmpo	0.00	0.75	
Eef1a1	0.00	0.71	
Rad21	0.00	0.75	
Nup35	0.00	0.75	
Sumo4	0.04	0.65	
Tcp1	0.00	0.67	
Plec	0.04	0.73	
Slc30a4	0.00	0.51	
AABR07073038.1	0.01	0.67	
Insyn1	0.01	0.65	
Ckap2l	0.00	0.64	
Eef2	0.00	0.71	
Recql4	0.00	0.69	
Bub1	0.00	0.62	
Dsn1	0.00	0.70	
F2r	0.00	0.64	
Kif11	0.00	0.58	
Mrpl45	0.01	0.75	
Pclaf	0.00	0.58	
RF00263	0.00	0.72	
Pcna	0.00	0.56	
Hjurp	0.00	0.71	
Tubb6	0.00	0.74	
Cdc25b	0.01	0.75	
Nolc1	0.00	0.71	
Extl3	0.00	0.76	
RGD1564836	0.03	0.75	
AABR07063082.1	0.00	0.66	
Slc25a5	0.00	0.65	
AC095390.1	0.00	0.64	
Lamc2	0.03	0.76	
Acot7	0.00	0.75	
AC106663.1	0.03	0.70	
Areg	0.00	0.72	
Lsm8	0.05	0.77	
AABR07005838.1	0.02	0.70	

Rac1	0.00	0.71	
Lsm4	0.01	0.72	
Lsm2	0.01	0.70	
Lsm3	0.00	0.65	
Fosl1	0.00	0.72	
AABR07070810.1	0.00	0.68	
Irak1	0.00	0.66	
Cbx3	0.01	0.68	
Tpx2	0.00	0.64	
Pmf1	0.00	0.69	
Bub1b	0.00	0.65	
Sdc1	0.00	0.62	
Cdt1	0.00	0.64	
Rbm17	0.00	0.76	
LOC100359583	0.00	0.61	
AABR07049223.1	0.01	0.57	
Lmnb2	0.00	0.60	
AABR07069282.1	0.00	0.64	
Lmnb1	0.00	0.60	
G2e3	0.00	0.71	
Map3k6	0.00	0.67	
Kntc1	0.00	0.67	
Nup85	0.00	0.71	
Phf19	0.00	0.70	
Zwilch	0.00	0.70	
Csrp1	0.00	0.64	
Hdgf	0.00	0.74	
Hnrnpul2	0.00	0.70	
AABR07068127.1	0.00	0.66	
Ywhaz	0.01	0.64	
Api5	0.00	0.61	
Aaas	0.00	0.77	
Nop58	0.00	0.75	
Exo1	0.00	0.71	
Fkbp4	0.00	0.68	
Cdk1	0.00	0.61	
Cdk2	0.00	0.62	
Tlr5	0.00	0.75	
Trip13	0.00	0.75	
Rasl2-9	0.01	0.72	
Kifc1	0.00	0.64	
Rpp21	0.01	0.67	
Ccnd3	0.02	0.64	
AABR07067526.1	0.00	0.52	
Psat1	0.00	0.68	
Ezh2	0.00	0.75	
Hsp90ab1	0.00	0.67	
Ltbp4	0.02	0.73	
Hspa5	0.00	0.67	
Srsf2	0.02	0.74	
Traip	0.00	0.74	
AABR07038895.2	0.00	0.70	
Kif18b	0.00	0.74	
Foxm1	0.00	0.59	

Serpinb8	0.00	0.77	
Ctdsp2	0.00	0.56	
Fam83d	0.00	0.71	
AABR07053669.1	0.00	0.67	
Uap1	0.00	0.70	
LOC688459	0.00	0.52	
Tk1	0.00	0.47	
Ahsa1	0.01	0.71	
AABR07067506.1	0.03	0.68	
Rnps1	0.00	0.75	
Vars	0.00	0.62	
Tmem97	0.00	0.55	
Grk6	0.04	0.76	
Grk2	0.00	0.76	
Ubiad1	0.00	0.74	
Ppat	0.00	0.63	
Chaf1a	0.00	0.68	
Eps8l2	0.02	0.77	
AABR07065265.1	0.01	0.75	
Mcm2	0.00	0.58	
Hsf1	0.00	0.77	
Itga3	0.00	0.66	
LOC688672	0.00	0.76	
Ctps1	0.00	0.68	
Orc6	0.00	0.77	
Cks2	0.00	0.57	
Ube2c	0.00	0.53	
Shroom1	0.00	0.75	
Orc1	0.00	0.63	
Cdca2	0.00	0.74	
Cdca3	0.00	0.46	
Cdca7	0.00	0.50	
Cdca4	0.00	0.73	
Cdca8	0.00	0.61	
Suv39h1l1	0.00	0.66	
LOC691418	0.01	0.68	
Ube2t	0.00	0.69	
Npm3	0.00	0.61	
Ttk	0.00	0.66	
Slc52a3	0.01	0.68	
Topbp1	0.00	0.62	
Tuba1b	0.01	0.64	
Kif23	0.00	0.72	
Kif22	0.00	0.57	
AABR07053716.1	0.01	0.71	
Anp32e	0.00	0.65	
Anp32b	0.00	0.66	
Isy1	0.00	0.76	
Cyp3a9	0.02	0.71	
Ankrd52	0.00	0.72	
Mepe	0.01	0.53	
Lrrfip1	0.00	0.77	
Sephs1	0.00	0.73	
Ssx2ip	0.00	0.76	

AABR07038939.1	0.04	0.69	
Pak4	0.00	0.77	
Fen1	0.00	0.68	
Fam136a	0.00	0.71	
Sacs	0.00	0.73	
Cep295	0.05	0.74	
Ppl	0.01	0.75	
Kcnn4	0.00	0.66	
Cdc45	0.00	0.75	
AABR07002564.1	0.03	0.70	
Kif2c	0.00	0.59	
Ncapg2	0.00	0.69	
Mtch1	0.00	0.75	
P4hb	0.01	0.75	
Cnot9	0.00	0.74	
Myo5a	0.00	0.75	
AABR07033162.1	0.00	0.74	
Nrbp1	0.00	0.73	
Smagp	0.00	0.76	
Sgk1	0.00	0.73	
AABR07035541.2	0.00	0.66	
Ubqln4	0.00	0.75	
AABR07037356.1	0.00	0.68	
Pigq	0.00	0.54	
Pbk	0.00	0.68	
Evi2a	0.00	0.66	
Cenpo	0.00	0.74	
Ehd4	0.00	0.71	
Ccna2	0.00	0.54	
Cenph	0.00	0.70	
Atad5	0.00	0.76	
Cenpf	0.00	0.69	
Cenpe	0.00	0.60	
Nucks1	0.00	0.74	
Cenpb	0.03	0.75	
Cenpa	0.00	0.60	
Atad2	0.00	0.69	
AABR07067583.1	0.02	0.65	
Cenpw	0.00	0.66	
Cenpu	0.00	0.75	
Ckap4	0.00	0.76	
Ckap5	0.00	0.76	
Rcc1	0.00	0.75	
Rcc2	0.00	0.73	
Dnajc9	0.00	0.62	
Ptges3	0.00	0.71	
Psmc3ip	0.00	0.64	
G3bp1	0.00	0.72	
RGD1307929	0.00	0.69	
Myc	0.00	0.74	
Nnt	0.00	0.75	
Ptma	0.00	0.67	
Dhrs9	0.00	0.48	
Racgap1	0.02	0.74	

Idh2	0.00	0.68	
AC112018.1	0.00	0.65	
RF00431	0.00	0.56	
LOC680491	0.02	0.65	
Mybl2	0.00	0.54	
Slc7a5	0.00	0.72	
Nxph3	0.02	0.71	
Snrpa	0.00	0.68	
Ndc80	0.00	0.70	
Pxdn	0.00	0.67	
Rps6ka4	0.00	0.71	
Rpa3	0.01	0.66	
Rpa2	0.00	0.76	
AABR07025301.1	0.00	0.57	
AABR07054368.1	0.00	0.76	
Gna11	0.00	0.68	
S100a11	0.03	0.73	
S100a10	0.05	0.75	
Eme1	0.00	0.66	
Idh3a	0.00	0.72	
Hyou1	0.00	0.75	
Samd1	0.05	0.76	
Plp2	0.00	0.74	
Zyx	0.00	0.75	
LOC102555453	0.00	0.76	
Incenp	0.00	0.63	
Las1l	0.00	0.70	
Tipinl1	0.00	0.52	
Spag5	0.00	0.61	
Dynlt1	0.01	0.74	
Gale	0.01	0.62	
Plcd4	0.00	0.61	
Me2	0.00	0.76	
Ncapg	0.00	0.70	
Sod3	0.00	0.66	
Tnfaip8l1	0.00	0.59	
Hspa14	0.00	0.76	
Sec13	0.00	0.75	
Gtse1	0.00	0.67	
Epn2	0.00	0.73	
AABR07044593.1	0.00	0.58	
Ereg	0.00	0.57	
LOC499235	0.04	0.75	
Ado	0.05	0.69	
RF00218	0.02	0.63	
Arl2bp	0.00	0.49	
AABR07030462.1	0.00	0.75	
Ect2	0.00	0.64	
Sgo1	0.00	0.76	
Prkar2a	0.00	0.73	
Ncl	0.00	0.74	
Tagln2	0.00	0.70	
Ranbp1	0.03	0.67	
Agpat2	0.00	0.75	

Knstrn	0.00	0.70	
Loxl1	0.01	0.72	
Loxl2	0.00	0.55	
Ifrd2	0.00	0.75	
Pomgnt1	0.02	0.76	
Ccne1	0.01	0.63	
AABR07001512.1	0.01	0.57	
AABR07055919.1	0.03	0.76	
AABR07013255.1	0.00	0.56	
Dek	0.00	0.63	
Eif4ebp1	0.00	0.74	
Eif4ebp2	0.00	0.72	
Ahcy	0.00	0.64	
Fam111a	0.00	0.64	
AC141966.1	0.03	0.73	
Nusap1	0.00	0.72	
Ddx11	0.00	0.69	
Tubb4b	0.00	0.72	
AABR07036016.1	0.02	0.72	
Snrpa1	0.00	0.66	
Fhl3	0.02	0.76	
AABR07000658.1	0.00	0.55	
Wee1	0.00	0.71	
Srsf7	0.00	0.67	
Srsf1	0.01	0.77	
Srsf3	0.01	0.76	
Emd	0.00	0.61	
Tonsl	0.03	0.76	
Snrpb	0.00	0.75	
E2f4	0.00	0.74	
Espl1	0.00	0.71	
Chaf1b	0.00	0.73	
Mmaa	0.00	0.73	
Gatad2a	0.00	0.69	
Mis18a	0.00	0.67	
Snrpf	0.01	0.72	
Tmem138	0.00	0.76	
Smc2	0.00	0.72	
Smc4	0.00	0.66	
Rnd1	0.00	0.70	
Nup205	0.00	0.76	
Tmem43	0.00	0.71	
Arhgdia	0.00	0.69	
Kif20a	0.00	0.52	
AABR07060593.1	0.02	0.70	
AABR07024500.1	0.00	0.64	
Dnmt1	0.00	0.70	
Ids	0.02	0.75	
Dscc1	0.01	0.70	
Nemp1	0.00	0.73	
Anapc15	0.00	0.55	
LOC100359539	0.00	0.59	
LOC102547056	0.03	0.75	
Dpp9	0.01	0.74	

Actr1b	0.00	0.75
Fanci	0.00	0.74
Fbl	0.00	0.60
