# Supplementary material for: GRWD1-WDR5-MLL2 Epigenetic Complex Mediates H3K4me3 Mark and Is Essential for Kaposi’s Sarcoma-Associated Herpesvirus-Induced Cellular Transformation
Source: mBio. 2021 Dec 21;12(6):e03431-21. doi: 10.1128/mbio.03431-21 (PMC8689518; doi:10.1128/mbio.03431-21)
Supplement: TABLE S5 [file mbio.03431-21-st005.pdf]

TABLE S5A Top enriched pathways of common genes after GRWD1, WDR5, or MLL2 knockdown in MM cells

Ingenuity Canonical Pathways	-log(p-value)	Ratio	Molecules
Kinetochore Metaphase Signaling Pathway	39.9	0.324	AURKB,BIRC5,BUB1,BUB1B,CCNB1,CDC20,CDCA8,CDK1,CENPA,CENPE,CENPH,CENPK,CENPO,CENPU,CENPW,DSN1,ESPL1, INCENP,KIF2C,KNTC1,MAD2L1,NDC80,NUF2,PLK1,PMF1/PMF1-BGLAP,PPP2R5D,PTTG1,RAD21,SKA1,SKA3,SPC25,TTK,ZW10,ZWILCH
Cell Cycle Control of Chromosomal Replication	22.9	0.339	CDC45,CDC6,CDC7,CDK1,CDK2,CDT1,MCM2,MCM3,MCM4,MCM5,MCM6,MCM7,ORC1,ORC6,PCNA,POLE,RPA2,RPA3,TOP2A
Mitotic Roles of Polo-Like Kinase	18.2	0.258	CCNB1,CDC20,CDC25B,CDC7,CDK1,ESPL1,FBXO5,KIF11,KIF23,PLK1,PLK2,PLK4,PPP2R5D,PRC1,PTPA,PTTG1,RAD21
Role of CHK Proteins in Cell Cycle Checkpoint Control	16.3	0.263	BRCA1,CDK1,CDK2,CDKN1A,CLSPN,E2F2,E2F8,MDC1,PCNA,PLK1,PPP2R5D,PTPA,RFC2,RFC3,RFC5
Cell Cycle: G2/M DNA Damage Checkpoint Regulation	14.1	0.26	AURKA,BORA,BRCA1,CCNB1,CDC25B,CDK1,CDKN1A,CKS1B,CKS2,GADD45A,MDM2,PLK1,TOP2A
Role of BRCA1 in DNA Damage Response	13.9	0.188	BARD1,BRCA1,CDKN1A,E2F2,E2F8,FANCB,FANCD2,GADD45A,MDC1,MSH6,PLK1,RFC2,RFC3,RFC5,TOPBP1
ATM Signaling	12.6	0.155	BRCA1,CBX3,CCNB1,CDK1,CDK2,CDKN1A,FANCD2,GADD45A,MDC1,MDM2,PPP2R5D,PTPA,SMC2,SUV39H1,TOPBP1
GADD45 Signaling	12.4	0.45	BRCA1,CCNB1,CCNE1,CCNE2,CDK1,CDK2,CDKN1A,GADD45A,PCNA
Cyclins and Cell Cycle Regulation	12.3	0.167	CCNA2,CCNB1,CCNE1,CCNE2,CDK1,CDK2,CDKN1A,CDKN2C,E2F2,E2F8,PA2G4,PPP2R5D,PTPA,SUV39H1
NER (Nucleotide Excision Repair, Enhanced Pathway)	9.64	0.121	CHAF1A,CHAF1B,LIG4,PCNA,POLD2,POLE,POLE2,RFC2,RFC3,RFC5,RPA2,RPA3,TOP2A
Estrogen-mediated S-phase Entry	9.54	0.308	CCNA2,CCNE1,CCNE2,CDK1,CDK2,CDKN1A,E2F2,E2F8
Mismatch Repair in Eukaryotes	9.24	0.389	EXO1,FEN1,MSH6,PCNA,RFC2,RFC3,RFC5
Cell Cycle: G1/S Checkpoint Regulation	8.37	0.147	CCNE1,CCNE2,CDK2,CDKN1A,CDKN2C,E2F2,E2F8,MDM2,PA2G4,SUV39H1
DNA damage-induced 14-3-3σ Signaling	7.35	0.316	BRCA1,CCNB1,CCNE1,CCNE2,CDK1,CDK2
Hereditary Breast Cancer Signaling	7.08	0.0833	BARD1,BRCA1,CCNB1,CDK1,CDKN1A,FANCB,FANCD2,GADD45A,MSH6,RFC2,RFC3,RFC5
Cell Cycle Regulation by BTG Family Proteins	6.81	0.189	CCNE1,CCNE2,CDK2,E2F2,E2F8,PPP2R5D,PTPA
p53 Signaling	5.8	0.0918	BIRC5,BRCA1,CDK2,CDKN1A,GADD45A,MDM2,PCNA,TOPBP1,TP53INP1
Senescence Pathway	5.75	0.0497	CCNB1,CCNE1,CCNE2,CDC25B,CDK1,CDK2,CDKN1A,E2F2,E2F8,EZH2,GADD45A,ING1,MDM2,PPP2R5D,PTPA
Pancreatic Adenocarcinoma Signaling	5.74	0.0781	BIRC5,CCNE1,CCNE2,CDK2,CDKN1A,E2F2,E2F8,MDM2,PA2G4,SUV39H1
Molecular Mechanisms of Cancer	5.4	0.0397	AURKA,BRCA1,CCNE1,CCNE2,CDC25B,CDK1,CDK2,CDKN1A,CDKN2C,CYCS,E2F2,E2F8,FADD,FANCD2,HAT1,MDM2,PA2G4,SUV39H1
Small Cell Lung Cancer Signaling	4.8	0.0808	CCNE1,CCNE2,CDK2,CKS1B,CYCS,E2F2,PA2G4,SUV39H1
Prostate Cancer Signaling	4.31	0.069	CCNE1,CCNE2,CDK2,CDKN1A,E2F2,MDM2,PA2G4,SUV39H1
Glioma Signaling	3.91	0.0606	CDKN1A,CDKN2C,E2F2,E2F8,IDH2,MDM2,PA2G4,SUV39H1
Protein Ubiquitination Pathway	3.49	0.0394	B2M,BRCA1,CDC20,DNAJC9,HLA-A,HSPA14,MDM2,UBE2C,UBE2S,UBE2T,USP1
BER pathway	3.44	0.231	FEN1,PCNA,POLE
Granzyme B Signaling	3.16	0.188	CYCS,LMNB1,LMNB2
Chronic Myeloid Leukemia Signaling	2.89	0.0561	CDKN1A,E2F2,E2F8,MDM2,PA2G4,SUV39H1
Myc Mediated Apoptosis Signaling	2.6	0.0784	ADRB2,CYCS,FADD,MDM2
Aryl Hydrocarbon Receptor Signaling	2.58	0.0424	CCNA2,CCNE1,CCNE2,CDK2,CDKN1A,MCM7,MDM2
Regulation of Cellular Mechanics by Calpain Protease	2.47	0.0556	CCNA2,CCNE1,CCNE2,CDK1,CDK2
Glioblastoma Multiforme Signaling	2.43	0.0398	CCNE1,CCNE2,CDK2,CDKN1A,E2F2,E2F8,MDM2
Sumoylation Pathway	2.12	0.0459	MDM2,PCNA,RFC2,RFC3,RFC5
Coronavirus Pathogenesis Pathway	2.09	0.0345	CCNE1,CCNE2,CDK2,E2F2,E2F8,PA2G4,SUV39H1
Hypoxia Signaling in the Cardiovascular System	2.02	0.0541	MDM2,UBE2C,UBE2S,UBE2T
Bladder Cancer Signaling	2.01	0.0431	CDKN1A,E2F2,MDM2,PA2G4,SUV39H1
Pyrimidine Deoxyribonucleotides De Novo Biosynthesis I	2	0.075	DUT,RRM1,RRM2
DNA Double-Strand Break Repair by Homologous Recombination	1.98	0.143	BRCA1,GEN1
Pyridoxal 5'-phosphate Salvage Pathway	1.98	0.0526	CDK1,CDK2,PLK1,TTK
Breast Cancer Regulation by Stathmin1	1.77	0.0218	ADRB2,AURKA,AURKB,CDK1,CDK2,E2F2,E2F8,FOXM1,PPP2R5D,PTPA,SIVA1,STMN1,TUBB
Tumoricidal Function of Hepatic Natural Killer Cells	1.53	0.0833	CYCS,FADD
PD-1, PD-L1 cancer immunotherapy pathway	1.49	0.0374	B2M,CDK2,CIP2A,HLA-A
Ovarian Cancer Signaling	1.47	0.0312	BRCA1,E2F2,MSH6,PA2G4,SUV39H1
Induction of Apoptosis by HIV1	1.44	0.0462	CYCS,FADD,SLC25A10
HOTAIR Regulatory Pathway	1.42	0.0303	CDKN1A,EZH2,FOXM1,MDM2,RBBP7
Salvage Pathways of Pyrimidine Ribonucleotides	1.39	0.0348	CDK1,CDK2,PLK1,TTK
Sonic Hedgehog Signaling	1.36	0.0667	CCNB1,CDK1

TABLE S5B Top enriched pathways of common genes after GRWD1, WDR5, or MLL2 knockdown in KMM cells

Ingenuity Canonical Pathways	-log(p-value)	Ratio	Molecules
Kinetochore Metaphase Signaling Pathway	32.6	0.229	AURKB,BIRC5,BUB1,BUB1B,CCNB1,CDC20,CDCA8,CDK1,CENPE,CENPH,INCENP,<br>KIF2C,KNTC1,MAD2L1,NDC80,NUF2,PLK1,PTTG1,RAD21,SKA1,SKA3,SPC25,TTK,ZWINT
Mitotic Roles of Polo-Like Kinase	13.5	0.167	CCNB1,CDC20,CDK1,FBXO5,KIF11,PLK1,PLK2,PLK4,PRC1,PTTG1,RAD21
Cell Cycle Control of Chromosomal Replication	12.6	0.179	CDK1,MCM2,MCM3,MCM4,MCM6,ORC6,POLE,RPA2,RPA3,TOP2A
Cell Cycle: G2/M DNA Damage Checkpoint Regulation	11.4	0.18	AURKA,CCNB1,CDK1,CDKN1A,CKS1B,GADD45A,MDM2,PLK1,TOP2A
ATM Signaling	6.16	0.0722	CCNB1,CDK1,CDKN1A,FANCD2,GADD45A,MDM2,SMC2
GADD45 Signaling	5.52	0.2	CCNB1,CDK1,CDKN1A,GADD45A
Role of BRCA1 in DNA Damage Response	5.45	0.075	CDKN1A,E2F8,FANCD2,GADD45A,PLK1,RFC5
Estrogen-mediated S-phase Entry	5.04	0.154	CCNA2,CDK1,CDKN1A,E2F8
Role of CHK Proteins in Cell Cycle Checkpoint Control	4.95	0.0877	CDK1,CDKN1A,E2F8,PLK1,RFC5
Cyclins and Cell Cycle Regulation	4.14	0.0595	CCNA2,CCNB1,CDK1,CDKN1A,E2F8
Hereditary Breast Cancer Signaling	4	0.0417	CCNB1,CDK1,CDKN1A,FANCD2,GADD45A,RFC5
NER (Nucleotide Excision Repair, Enhanced Pathway)	3.64	0.0467	POLE,RFC5,RPA2,RPA3,TOP2A
Protein Ubiquitination Pathway	3.22	0.0251	B2M,CDC20,HSPB2,MDM2,UBE2C,UBE2S,USP1
Pyrimidine Deoxyribonucleotides De Novo Biosynthesis I	2.95	0.075	DUT,RRM1,RRM2
p53 Signaling	2.79	0.0408	BIRC5,CDKN1A,GADD45A,MDM2
Granzyme B Signaling	2.52	0.125	LMNB1,LMNB2
DNA damage-induced 14-3-3σ Signaling	2.38	0.105	CCNB1,CDK1
Pancreatic Adenocarcinoma Signaling	2.37	0.0312	BIRC5,CDKN1A,E2F8,MDM2
Senescence Pathway	2.33	0.0199	CCNB1,CDK1,CDKN1A,E2F8,GADD45A,MDM2
Glioma Signaling	2.32	0.0303	CDKN1A,E2F8,IDH2,MDM2
Cell Cycle: G1/S Checkpoint Regulation	2.29	0.0441	CDKN1A,E2F8,MDM2
Hypoxia Signaling in the Cardiovascular System	2.18	0.0405	MDM2,UBE2C,UBE2S
Pyridoxal 5'-phosphate Salvage Pathway	2.15	0.0395	CDK1,PLK1,TTK
Molecular Mechanisms of Cancer	2.05	0.0155	AURKA,CDK1,CDKN1A,E2F8,FANCD2,MDM2,RHOB
Sonic Hedgehog Signaling	1.99	0.0667	CCNB1,CDK1
Aryl Hydrocarbon Receptor Signaling	1.98	0.0242	CCNA2,CDKN1A,HSPB2,MDM2
Glioblastoma Multiforme Signaling	1.89	0.0227	CDKN1A,E2F8,MDM2,RHOB
Nucleotide Excision Repair Pathway	1.86	0.0571	RPA2,RPA3
Chronic Myeloid Leukemia Signaling	1.75	0.028	CDKN1A,E2F8,MDM2
Sumoylation Pathway	1.73	0.0275	MDM2,RFC5,RHOB
Mechanisms of Viral Exit from Host Cells	1.73	0.0488	LMNB1,LMNB2
Salvage Pathways of Pyrimidine Ribonucleotides	1.67	0.0261	CDK1,PLK1,TTK
Melanoma Signaling	1.53	0.0385	CDKN1A,MDM2
